# Simulating the NaK Eutectic Alloy with Monte Carlo and Machine Learning

**DOI:** 10.1038/s41598-018-36574-y

**Published:** 2019-01-24

**Authors:** Douglas M. Reitz, Estela Blaisten-Barojas

**Affiliations:** Center for Simulation and Modeling (formerly, Computational Materials Science Center) and Department of Computational and Data Sciences, George Mason University, Fairfax, Virginia 22030 USA

## Abstract

Combining atomistic simulations and machine learning techniques can expedite significantly the materials discovery process. We present an application of such methodological combination for the prediction of the melting transition and amorphous-solid behavior of the NaK alloy at the eutectic concentration. We show that efficient prediction of these properties is possible via machine learning methods trained on the topological local structural properties. The configurations resulting from Monte Carlo annealing of the NaK eutectic alloy are analyzed with topological attributes based on the Voronoi tessellation and using expectation-maximization clustering and Random Forest classification. We show that the Voronoi topological fingerprints make an accurate and fast prediction of the alloy thermal behavior by cataloguing the atomic configurations into three distinct phases: liquid, amorphous solid, and crystalline solid. Melting is found at 230 K by the sharp split of configurations classified as crystalline solid and as liquid. With the proposed metrics, an arrest-motion temperature is identified at 130–140 K through a top down clustering of the atomic configurations catalogued as amorphous solid. This statistical learning paradigm is not restricted to eutectic alloys or thermodynamics, extends the utility of topological attributes in a significant way, and harnesses the discovery of new material properties.

## Introduction

The characterization of metallic amorphous solids is more complex than the identification of crystalline matter^1^. Currently, bulk amorphous metals have become useful engineering materials in several applications despite the fact that their microscopic properties at the medium and local range are not as well understood^2^. For example, bulk amorphous alloys exhibit high strength, peculiar elastic properties, and other unusual engineering characteristics^3^. Concurrently, several machine learning techniques have been introduced in the field of condensed matter for enhancing the understanding of phenomena in materials design^4–7^. In this article we demonstrate that a topological inspection of the structure of the eutectic sodium-potassium (NaK) alloy using machine learning analyses predicts excellently the solidification fate of the liquid eutectic alloy leading to crystalline and amorphous solids. Indeed, our findings are in full agreement with our Metropolis Monte Carlo (MMC) simulations using the second moment approximation (SMA) potential^8^ described in upcoming sections.

When looking for compositions of binary metal alloys with high glass forming ability, the eutectic and slightly off-eutectic compositions is a good place to start^9,10^. Eutectic NaK is a binary alloy formed by 22% Na and 78% K in atomic weight^11,12^. This interesting alloy is liquid at room temperature, solidifies at temperatures below 260 K^12^, and is used as a coolant in nuclear reactors among other applications^13^. Experimental measurements of the mass density have been performed for the NaK liquid phase from room to higher temperatures at the eutectic concentration^14^. Numerical simulations of the NaK nanoalloy at various relative concentrations^15^ showed that the *magical* nanostructure size with minimum excess energy corresponded to the eutectic composition. However, there are no theoretical/computational studies of the extended condensed phases of eutectic NaK.

Our preliminary MMC simulations of the eutectic NaK alloy gave the thermal behavior of the enthalpy and the mass density along a computational procedure that annealed the system spanning high-to-low temperatures^16^. Now we analayze the thermodynamic parameters (structure, enthalpy, system volume) exhaustively from the MMC atomistic simulations with the goal of neatly identifying the liquid, crystalline, and amorphous phases that develop along the annealing process. An independent machine learning analysis using exclusively the atomic Cartesian coordinates obtained in the simulation runs is undertaken with the goal of verifying the identification of the three condensed phases predicted by the simulation. Our machine learning strategy is based on topological parameters determined by Voronoi-tessellating^17^ the 3D space occupied by the atoms inside the computational box. The Voronoi tessellation has been utilized for identifying topological characteristics of liquids^18–20^, glasses^2,21,22^, zeolites^23^, clusters^24^, among several others.

This article is organized as follows. In the *Model and methods* section the model potential for the NaK alloy is provided along with a description of the Monte Carlo methodology used in the atomistic simulations. The definition of the topological attributes used in the machine learning analyses is also included in this section. The section entitled *Energetics and structure of eutectic NaK* describes the thermodynamic properties obtained along the thermal annealing process of the NaK alloy obtained with atomistic simulations and the structural analysis performed to characterize the three different phases detected. The atomic positions in each configuration obtained in this section constitute the dataset that we used for further study. Description of the topological attributes for the machine learning approaches of the dataset is given in the *Data analyses of the topological attributes*, along with the data-based analyses of principal component, unsupervised learning data clustering, and data classification approaches. Our results highlight the ability of machine learning in analyzing intrinsic thermodynamic behavior, and at the same time providing valuable guidance for inspection of other metal alloys in condensed phases. This work is concluded in *Conclusion* with a discussion of the results.

## Model and Methods

### Model potential

The Second Moment Approximation (SMA) model potential is a many-body potential that approximates the local environment of every atom mimicking the distribution of electronic states in a d-band by a bonding term *U*_*el*_, supplemented with the *U*_*rep*_ Born-Mayer term for the short-range repulsion^8^. The SMA is a classical version of the tight-binding approach. As such, the SMA differs significantly from pair-additive classical models and has a characteristic very soft spheres repulsive wall. The SMA analytical expression is a sum, *U*_*coh*_ = *U*_*rep*_ + *U*_*el*_, with

1 $${U}_{rep}=\sum _{i=1}^{N}\,\sum _{j=1,i\ne j}^{N}\,{\varepsilon }_{ij}{e}^{-{p}_{ij}(\tfrac{{r}_{ij}}{{r}_{{0}_{ij}}}-1)}$$

2 $${U}_{el}=-\,\sum _{i=1}^{N}\,{\{\sum _{j=1,i\ne j}^{N}{{\zeta }_{ij}}^{2}{e}^{-2{q}_{ij}(\tfrac{{r}_{ij}}{{r}_{{0}_{ij}}}-1)}\}}^{1/2}$$

where *r*_*ij*_ are the interatomic distances and *N* is the total number of atoms. The parameters for pure Na and K were developed in previous work^8^. Combination rules were employed for the Na-K pairs, with the geometric mean of the K and Na parameters for *ζ*_0_, *ε*_0_, *p*, *q* and a weighted arithmetic mean for ${r}_{{0}_{NaK}}=\frac{{N}_{Na}}{N}{r}_{{0}_{Na}}+\frac{{N}_{K}}{N}{r}_{{0}_{K}}$  (*N*_*K*_, *N*_*Na*_ being the number of K and Na atoms). Table 1 lists all SMA parameters used in this work.

**Table 1:** The SMA Parameters for atomic pairs K-K^8^, Na-Na^8^, and Na-K^16^.

	*ζ*_0_ (eV)	*ε*_0_ (eV)	*p*	*q*	*r*_0_ (nm)
Na	0.29113	0.015955	10.13	1.30	0.3698949
K	0.26259	0.020545	10.58	1.34	0.4367299
NaK	0.27649	0.018105	10.35	1.32	0.4150866

Although this classical modeling of the atomic interactions is not unique, we believe that the parametrization of the SMA is very appropriate for describing soft metals as the NaK alloy.

### Metropolis Monte Carlo atomistic simulations

Currently, the science community employing atomistic simulations for researching condensed phases of materials in thermal equilibrium recur primarily to Molecular Dynamics (MD) and Metropolis Monte Carlo (MMC)^25^, two extensively used methods. Across time, atomistic simulations have become popular because of the research investments to produce software packages that automate the multitude of algorithms needed in these simulations. The increase in popularity of MD over MMC has been driven by the ease to computer parallelize the algorithms for solving the MD underlying ordinary differential equations. On the contrary, implementing Markov Chain Monte Carlo methods have faced the bottleneck of the intrinsically serial Markov chain process^26^. Over time, novel computational techniques have been developed commensurate with the advances of computer hardware resulting in several MMC packages^27–29^.

In this work, we employ our in-house MMC implementation^27^. The MMC algorithm allows calculation of system properties averages at a temperature T by performing an importance sampling of the system states with energy *E*_*i*_ and probability *P*_*i*_ = *exp*(−*E*_*i*_/*k*_*B*_*T*)/*Q*. Here, *k*_*B*_ is the Boltzmann constant and *Q* is the partition function of the system. Each sampled state is a configuration of the system given by the coordinates of all atoms composing the system. The generated sequence of samples are linked through a Markov chain that requires ratios of probabilities between two consecutive samples for transitioning between them. Thus, the algorithm eliminates the need of calculating the partition function *Q*. The acceptance or rejection for transitioning from state *i* to state *j* is given by min(1, *P*_*j*_/*P*_*i*_). We used the isobaric-isothermal (NPT) version of the MMC^25^.

MMC NPT simulations were run for a system of 2000 atoms with periodic boundary conditions and a cutoff radius of 2.381 nm at a constant pressure of 101.325 kPa. At the alloy eutectic concentration, the computational box had 648 sodium atoms and 1352 potassium atoms. The SMA model potential was used to compute the potential energy of the atomic configurations. The initial configuration had the sodium atoms randomly distributed in the sites of a perfect bcc lattice at the pure potassium experimental density. The remaining sites were populated with potassium atoms. A new system configuration is generated once each of the 2000 atoms was attempted a move of a fixed length step in random direction. The magnitude of the atomic movements was dynamically adjusted throughout the simulation to maintain approximately a 50% rejection rate of attempted atomic moves. The volume change of the computational box was attempted once every passage over the 2000 attempted atomic moves at constant volume. Typically, in order to obtain the average values reported in the next section, 2 million passages through the full 2000 atoms were attempted after the system was sought to be in equilibrium.

For assessing cooling rates, we determine a rough estimate of the time equivalent to one MMC passage over all atoms (referred to as lattice-step). A system of N = 2000 potassium atoms was first NPT-equilibrated at 337 K and 101.325 kPa. Next, six NVT runs at the same temperature were run from different initial configurations to collect the atomic mean square displacement (MSD) as a function of lattice-steps. From the ${\rm{M}}{\rm{S}}{\rm{D}}\,({\rm{t}})={\sum }_{m=1}^{6}\,{\sum }_{i=1}^{N}$ ${[{{\bf{r}}}_{{\bf{i}}}(t)-{{\bf{r}}}_{{\bf{i}}}{({t}_{o})}_{m}]}^{2}/(6N)=6{\rm{t}}{{\rm{D}}}_{self}$ and the potassium empirical value^30^ D_*self*_ = 3.59 × 10^−9^ m^2^/s, we very roughly estimate 33 × 10^6^ lattice-steps ≈1 *μ*s in the liquid phase. This estimate will be used in the next section when referring to cooling rates.

## Energetics and Structure of Eutectic NaK

**Figure 1:**
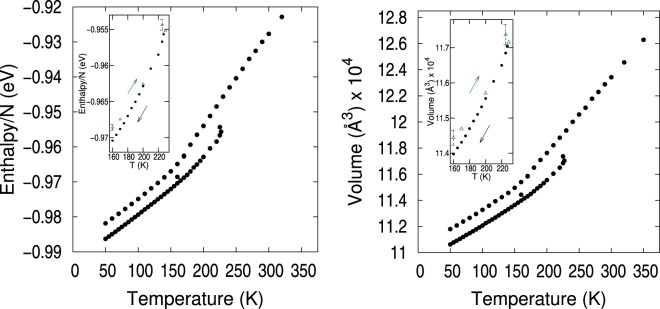
Average enthalpy and volume of the NaK alloy as a function of temperature showing two branches. The top branch corresponds to liquid-amorphous, while the lower branch is crystalline. Insets show in green the warming-up process leading to the crystalline branch.

The NaK alloy system was NPT equilibrated at 101.325 kPa and a high temperature of 700 K. Next, the system was annealed at constant pressure resulting in two branches of the the enthalpy and system volume below 230 K, as shown in Fig. 1. These two extensive properties of soft materials display such behavior^31–33^, which is predictable for a eutectic alloy. The lower enthalpy/volume branch was associated to crystalline packing. The higher enthalpy/volume branch corresponds to liquid states that were supercooled below 230 K and became an amorphous solid below approximately 140 K^31–33^.

Averages in Fig. 1 were calculated over 2 million lattice-steps after the system was equilibrated at each temperature. Using the estimate of 1 MMC lattice-step ≈0.03 ps, the following cooling/warming rates were applied along the annealing process. A first cooling process gave rise to the liquid-amorphous branch of higher enthalpy and volume in Fig. 1 by: (i) cooling from 700 to 150 K at a rate of 287 K/*μ*s, (ii) re-cooling from 170 to 150 K at a slower rate of 100 K/*μ*s enabled the system to reach a state of crystal character at 160 K (lowest temperature the system reached a crystal state from the upper branch), (iii) cooling from 150 to 50 K at 380 K/*μ*s. Secondly, starting from the state with crystal character at 160 K, an annealing process gave rise to the crystalline branch of lower enthalpy and volume in Fig. 1 by: (i) warming-up from 160 to 229 K at 180 K/*μ*s until at 230 K the system reverted to the liquid-amorphous branch (229 K was the highest temperature at which the system remained crystalline), (ii) slow cooling from 229 to 50 K at a rate of 73 K/*μ*s.

In Fig. 1, the amorphous-liquid branch displays a smooth behavior resulting from cooling at various rates. Meanwhile, the crystalline branch shows the two states at 160 K and 229 K between which the warming-up process was performed plus the states resulting from a slow cooling-down from 229 K to 50 K. The insets of Fig. 1 depict the warm-up states with green triangles and the cool-down states in black dots along the crystalline branch evidencing a hysteresis as observed in other alloys^34^. The annealing hysteresis is narrow in eutectic alloys because both metals melt simultaneously. The temperature above which the system did not remain crystalline while warming-up was 230 K. This temperature is lower than 260 K, the experimental melting temperature^12^. On the other hand, the passage from liquid to supercooled liquid occurred gradually in the 240 K to 140 K range. Around 130–140 K the upper branch had an inflection point consistent with an arrest in volume changes in configuration space and the system became a long-lived metastable amorphous solid^31,33^. Properties of the liquid were in good agreement with experimental values, indicating that the SMA potential gives a realistic representation of the liquid alloy. The liquid heat capacity *C*_*p*_ = 1019 J(kgK)^−1^ was calculated from a fit to the enthalpy slope between 290–310 K, agreeing well with the experimental value of 977 J(kgK)^−1^ at 298 K^14^. Additionally, the calculated equilibrium density of the liquid at 350 K was 0.89 g/cm^3^, comparing well with the experimental value^14^ of 0.86 g/cm^3^. No crystal structure measurements were found in the literature.

**Figure 2:**
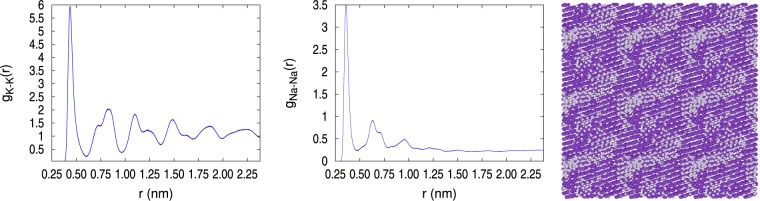
Pair correlation functions of the crystalline solid at 110 K and *ρ* = 1002.5 kg/m^3^. A system snapshot showing the formation of crystal planes and a segregated Na cluster is also depicted, violet is K and grey is Na.

**Figure 3:**
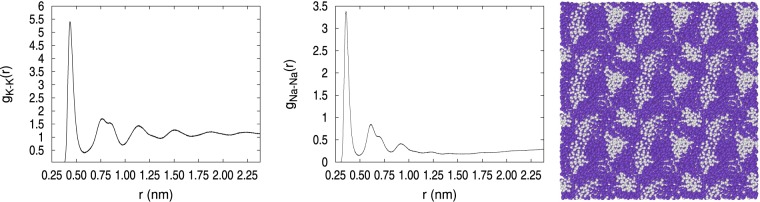
Pair correlation functions of the amorphous solid at 110 K and *ρ* = 992.57 kg/m^3^. A system snapshot showing the lack of atomic order and several Na atoms and clusters is also depicted, violet is K and grey is Na.

Analysis of the pair correlation function reveals clearly the different structural characteristics of the two types of solids, amorphous and crystalline, as illustrated in Figs 2 and 3, respectively. The *g*_*K*−*K*_(*r*) and *g*_*Na*−*Na*_(*r*) were calculated with NVT MMC at 110 K and 1002.5 kg/m^3^ for the crystalline solid and 992.57 kg/m^3^ for the amorphous solid. A clear difference between these two solids is seen in the *g*_*K*−*K*_(*r*) 2nd through 4rd peaks, where the peaks in the crystalline solid match well with the bcc lattice, while the amorphous solid displays the characteristic structural loss with a double bumped-second peak. The crystal peaks are broadened because the potassium atoms occupy only 70% of the sample volume and the rest is occupied by sodium atoms. The *g*_*Na*−*Na*_(*r*) shows differences between crystal and amorphous, although not as clear as for the K-K pairs. In Figs 2 and 3, the system snapshots of the two solid systems at 110 K show visually the difference between them. To help the visualization, the snapshots in these figures depict the computational box replicated three times in each Cartesian direction. The crystalline structure in Fig. 2 shows the periodic array of the atoms and the segregated Na cluster (grey) immersed in the potassium matrix (violet). This Na cluster was shaped as a raft with 3–4 atomic layers of thickness, as desiring to form a lamellar structure with large surface area. By contrast, the solid system depicted in Fig. 3 was amorphous throughout the occupied volume, had several segregated smaller Na clusters, and a significantly larger number of isolated Na atoms. Snapshots in Figs. 2, 3 were drawn with Ovito^35^.

Further analysis of the two solids structure was done at 140 K with the Adaptive Common Neighbor Analysis (a-CNA) algorithm^36–38^ that yielded a fingerprint of the local environment of each atom. In the crystalline solid, 55% of atoms had a bcc entourage and about 7% were fcc or hcp. The remaining atoms had no crystal symmetries in their local surroundings due to boundary atoms between the K matrix and the Na cluster. In contrast, in the amorphous solid none of the atoms had a bcc crystal entourage, only 10 atoms had fcc-, and 2 had ico- surroundings. Altogether, an insignificant number of atoms in the amorphous solid was characterized by a local surrounding with definite crystal signature.

In summary, the structural analyses provided additional support that two types of solids were obtained during the annealing process as previously differentiated by their thermodynamics. There is no experimental evidence that the eutectic NaK solidifies only as a crystal. Here, we have predicted that a metastable amorphous solid state is also reachable below 140 K along an annealing process. The lower enthalpy branch ranging from 50–230 K pertained to a crystalline matrix of potassium atoms spanning the computational box and encasing an extended cluster of sodium atoms. Meanwhile, the inflection point in the supercooled liquid-amorphous branch around 140 K suggested that below that temperature the system had transitioned to a solid, amorphous state. The transition resembled a glass transition. However, the amorphous solid did not display a homogeneous distribution of Na atoms in the K amorphous matrix as expected in a glass. Instead, an amorphous matrix of K atoms encased several small clusters of Na atoms that were themselves also amorphous. Therefore, we associated the inflection point with a temperature at which the supercooled liquid was viscous enough to transform definitely into a solid with amorphous structure but not necessarily a glass. Such temperature depends on the cooling rate^31–33^. We refer to it as T_*a*_ in following sections.

## Data Analysis Based on a Machine Learning Protocol

The current advent of publicly available trajectory data from numerous atomistic simulations drives interest to the implementation of smart tools that could extract information beyond the calculation that generated them. A goal of our data analyses was to find out if a machine learning protocol based exclusively on the knowledge of atomic coordinates collected along the MMC trajectories would provide a plausible physical description without knowing any of the thermodynamics findings described in the previous section. Machine learning analyses require a choice of attributes, also known as descriptors or features. We decided on the use of the Voronoi cells properties as sole attributes for the machine learning strategy described in this section because a Voronoi tessellation only requires knowledge of the coordinates of a set of points.

### Topological attributes based on Voronoi tessellation

A Voronoi tessellation is a segmentation of the available space into cells that fill such space densely^17^. This tessellation generates groupings of planes into convex polyhedra (Voronoi cells) such that all points on the cell are closer to the central site than to any other site. The resulting intersection of these planes is a Voronoi cell. We defined the machine learning attributes that described the atomic packing topology based on the Voronoi tessellation of the computational box volume, such that the position of each atom in the computational box was at the center of a Voronoi cell. Thus, a characteristic polyhedron enveloped each atom. Voronoi cells are characterized by the total number of faces CN (coordination number) and the number of 3-, 4-, 5-, and 6-edged faces comprising the cell. For example, CN*n* = 〈*n*_3_, *n*_4_, *n*_5_, *n*_6_〉 specifies a Voronoi cell with *n* faces, out of which there are *n*_3_ 3-edged faces, *n*_4_ 4-edged faces, etc. Certain Voronoi cells^2,22,39^ have proven useful in identifying glasses because they give rise to Z and Ze atomic arrangements where the nearest-neighbor atoms to the central atom are connected with fivefold bonds. We have tracked the actual Voronoi cells instead of their associated polytetrahedral atomic arrangements and labeled them as cells of type Z or Ze according to their classification^2^. All other Voronoi cells were identified by their CN type. Voro++^40^ with periodic boundary conditions was used to generate the tessellation of the atomic configurations saved from the MMC simulation runs. Configurations corresponding to the green points in Fig. 1 insets were not included.

Not only all encountered Voronoi cells were identified, but also several properties derived from them were defined as attributes. For example, attribute *f*5 was defined as the number of 5-edged cell faces. Along our study, 48 different attributes were found in the structures gathered from the MMC simulation runs. Table 2 provides a list of them. The Voronoi tessellation of the computational box containing 2000 atoms was performed for 18672 saved MMC configurations and the 48 topological attributes were calculated. Configurations from the green points in Fig. 1 inset were not included. Each of the 48 attributes was found a certain number of times in a given configuration, which defined its frequency of occurrence. These occurrence frequencies were entered in a data table of 18672 rows by 48 columns. Therefore, the dataset spanned a 48-dimensional space.

**Table 2:** Type of the topological attributes used in this study.

Attribute	Description
CN*x*	Number of Voronoi cells with *x* faces; where *x* = 8 to 22
Z*x*	Number of Voronoi cells with characteristics (intrinsic) for corresponding CN*x* = 8–17
Ze*x*	Number of Voronoi cells with the characteristics (extrinsic) for the corresponding CN*x* = 9–17
Z	Total number of cells with intrinsic Z characteristics
Ze	Total number of cells with extrinsic Z characteristics
f3 through f10	The combined number of f-edged faces in all Voronoi cells
bcc	Voronoi cell indicative of a body centered cubic CN14〈0, 6, 0, 8〉
faces	The combined number of faces of all Voronoi cells
V_*K*_, V_*Na*_	The average volume of potassium atom and sodium atom Voronoi cells

### Attribute selection

Reducing the number of attributes is a basic selection process in machine learning. There are various methods for selecting the most significant attributes based on different types of ranking processes. We used the Laplacian score^41^ for ranking the 48 topological attributes described in Table 2. This method acts as a filter for selecting attributes based on their ability of locality preservation. The 26 top Laplacian-ranked attributes and their scores are listed in Table 3. Our machine learning study was based on these 26 attributes such that the data table was reduced to have 18672 rows by 26 columns. The Appendix provides a figure depicting the ten top attributes and the data table is given in the Supplementary Information.

**Table 3:** The 26 highly ranked topological attributes based on their Laplacian score.

Rank	Attribute	Score
1	f5	0.99998
2	f6	0.99996
3	f4	0.99996
4	bcc	0.99962
5	f7	0.99940
6	f3	0.99938
7	faces	0.9986
8	CN14	0.9984
9	Z	0.9972
10	Ze	0.9947
11	Z12	0.9939
12	f8	0.9923
13	CN12	0.9915
14	Z13	0.9884
15	CN16	0.9876
16	V_*Na*_	0.9823
17	Ze15	0.9818
18	V_*K*_	0.9814
19	CN15	0.9813
20	Ze14	0.9793
21	CN13	0.9717
22	CN17	0.9707
23	Ze12	0.9421
24	Z14	0.9174
25	f9	0.9163
26	CN18	0.9128

### Principal component analysis

**Figure 4:**
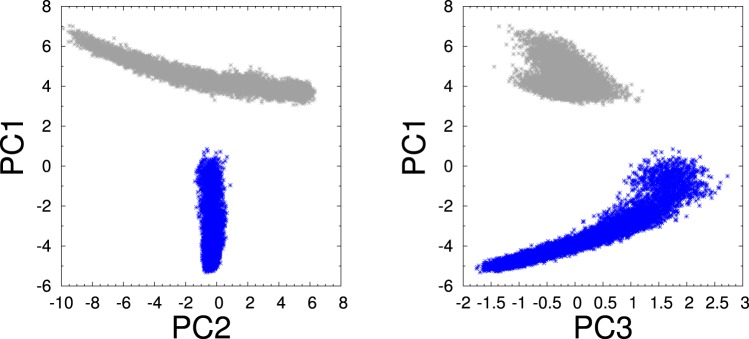
Correlation plots between the projections of the original dataset onto the 3 principal components. Posterior analysis identified the crystalline data of Fig. 1 as being the blue cluster and the combined amorphous and liquid regions corresponding to the gray-colored cluster.

In order to eliminate correlations among the top 26 attributes listed in Table 3, a principal component analysis (PCA)^42^ was performed. As a result, a set of 26 linearly uncorrelated attributes, called principal components (PC), were obtained. The principal components are linear combinations of the 26 original attributes that maximize their variance. This criterion is equivalent to minimizing the error function defined as the sum of squares in a regression analysis^43^. The first three PCs, PC1, PC2, PC3, yielded variances of 97%, with contributions of 63%, 32%, and 2%, respectively. A projection of the original 18672 data set onto the planes of the leading PCs allows for a type of data clustering, as visually shown in Fig. 4. An inspection of the data points in each of the two clusters based on what we know from the thermodynamics study, indicates that in the PC1-PC2 plane, the negative values correspond to crystalline structures (depicted blue) and the positive values correspond to the amorphous solid plus liquid data with no clear split between them. This same type of two-cluster split is visual in the PC1–PC3 plot of Fig. 4. Although it is common practice to use this approach for clustering data, clearly such clustering analysis is unable of discerning between the amorphous solid data and the liquid data. The PCA is also used for dimensionality reduction. In our case, there is a clear possibility of reducing the data space dimensions from 26 to 3. Next section describes an unsupervised learning algorithm for clustering the data making use of the reduced dimensionality of the dataset.

### Clustering of data

There are several machine learning clustering algorithms for unsupervised learning. We selected the expectation maximization (EM)^44,45^, a two phase iterative method to find an estimate of the maximum likelihood of model parameters. The EM algorithm attempts first to find an expected estimate of parameters for defining the log probability of the observed data, followed by a maximization of the log probability with respect to the parameters. The EM is appropriate for our data set because of its ability to create clusters sustaining a disjoint partition of the data when the data can be modeled by a mixture of Gaussian functions. The EM algorithm as implemented in Weka^46^ was adopted. The input attributes for the EM clustering were defined to be the projection of the original dataset onto the three predominant PCs, yielding a data table of 18672 rows by 3 columns. These attributes were linearly uncorrelated. The EM number of clusters to split the data was set to three, maximum number possible with three attributes. The resulting clusters were named *cluster*-*blue*, *cluster*-*red*, and *cluster*-*black*.

**Figure 5:**
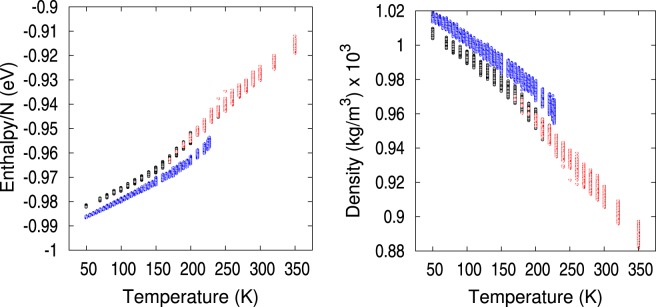
Enthalpy and mass density of the NaK eutectic alloy as a function of temperature colored by the EM cluster each point belongs to. Blue points pertain to structures in cluster-blue, which shows to contain all the crystalline structures. Black points correspond to structures in cluster-black, which are amorphous solid. Red points depict structures in cluster-red which are in the liquid/supercooled liquid region.

For visualizing the clustered data we recurred to identify the temperature at which each of the 18672 configurations was produced and its corresponding enthalpy and system density. Each configuration was given a color depending upon which of the three EM clusters it belonged to, blue for cluster-blue, red for cluster-red and black for cluster-black. Figure 5 shows how the data clustered. We remark that none of these thermodynamic values (temperature, enthalpy, density, volume) entered as attributes in the EM clustering or the PCA. Clearly, we see that cluster-blue corresponds to crystalline structures from our MMC simulations, cluster-black corresponds to amorphous solid structures, and cluster-red contains the liquid and supercooled liquid. Cluster-blue data are sharply separated from the rest, while there is a small region of temperature overlap between cluster-red and cluster-black in the 170–240 K temperature range. Indeed, cluster-red (liquid) has no configurations below 160 K. Likewise cluster-black (amorphous solid) has no configurations above 240 K.

In summary, this unsupervised machine learning approach is very successful. By only inspecting the topological characteristics of the simulated structures, the approach is able to assign the structures to the correct thermodynamics behavior obtained in the traditional simulations. To verify how stable is the data split determined by the clustering method, in the next section we create a data classification model defining three *classes* as the three clusters obtained in this section.

### Random Forests classification

To detect the quality of the clustering data split, we recurred to defining three classes based on the EM clustering and create a model classifier with them. We proceeded to sample a smaller dataset with 1934 points picked randomly from the 18672 data points. A table was constructed with the 3 PC attributes of the 1934-points dataset. This reduced size dataset was used for training a classification model with three classes: liquid, crystal, amorphous, depending upon which EM cluster the samples belonged to. The Random Forest classifier^47^, as implemented in Weka^46^, was used to build our model classifier with 100 random trees, trained with 10-fold validation. The evaluation on the training set had a mean absolute error of 0.002 with no confusion.

Once the 3-class classification model was created with the 1934-dataset, the remaining 16738 points were individually classified using the newly established data model. As a result, the crystalline structures classified with 100% accuracy into the crystal class. Meanwhile there was a minor confusion between the amorphous and the liquid classes, with the liquid class having 99.8% accuracy and the amorphous class a 98.9% accuracy. In total only 55 structures were classified within a class different than the EM cluster to which they belonged. This is shown in the confusion matrix given in Table 4.

**Table 4:** Confusion matrix for the Random Forest classification model.

Amorphous	Liquid	Crystal
4578	20	0
35	2897	0
0	0	9208

There are 55 samples out of the confusion matrix diagonal. The 20 samples wrongly classified as liquid were distributed between 180 K and 240 K. Likewise the 35 structures wrongly classified as amorphous solid were spread from 170 K to 250 K. Summarizing, the Random Forest model built with 10% of the available MMC configurations was appropriate for classifying the remaining 90% of the configurations into the three classes that originated from the unsupervised clustering analysis: crystal, amorphous solid, and liquid. Our results demonstrate explicitly the power of machine learning in estimating thermodynamic behavior and simultaneously providing valuable guidance to machine learning of metal alloys condensed phases.

**Figure 6:**
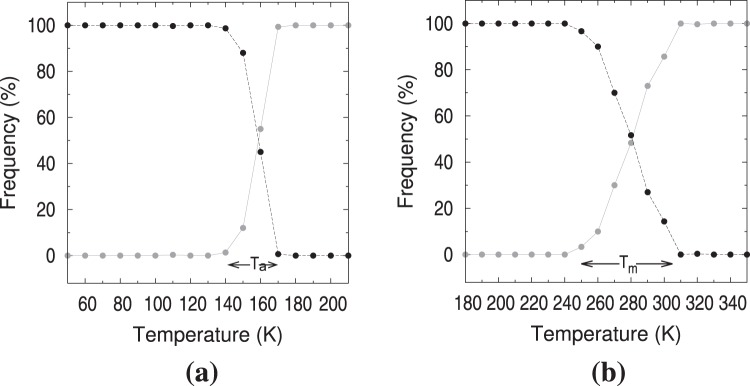
Number of samples in each of the two EM clusters obtained from the dataset belonging to (**a**) the amorphous class and (**b**) the liquid class. The temperature region 130–170 K in (**a**) was identified as estimate of the supercooled liquid transition to an amorphous solid where the volume has arrested its decrease. The temperature region 220–300 K in (**b**) was identified as an estimate for the melting transition.

As illustrated in Fig. 5, configurations belonging to the amorphous and liquid classes displayed thermodynamic a smooth temperature dependence in Fig. 1. Therefore, a final top down analysis of these two types of configurations was performed with the EM algorithm to yield a first layer of a hierarchical clustering^48^. The amorphous class displayed a sharp split of samples into two sub-clusters. By identifying each sample with its temperature during the simulation, Fig. 6a illustrates visually the results. The crossing region between130–170 K was identified as the amorphous system transitioning to a volume-arrested amorphous solid. The liquid class displayed a softer split of samples into two clusters, as shown in Fig. 6b. The crossing region between 220–300 K was identified as temperatures where the system was pre-melting and melting. Since the SMA potential of the pure metals predict melting temperature higher than experiment^8^, it is expected that the alloy melting region spreads beyond the experimental 260 K. The system behavior in these transition regions was embedded in the attributes used, which we chose not to inspect before applying the latest clustering. The Appendix includes the temperature behavior of ten top attributes.

As a validation of conclusions in Fig. 6, a moving interface NPT simulation^49^ was implemented by creating an initial hybrid system with half of the computational box containing the crystalline solid configuration at 150 K and the other half with the liquid configuration at several temperatures between 200–250 K. It was clearly seen that for all temperatures below 220 K the crystalline solid prevailed by solidifying the liquid half of the box. Meanwhile for temperatures 220–250 K, the liquid configuration overcame. As the temperature neared 220 K from above, a steep increase in MMC lattice steps were required.

In summary, this machine learning process has revealed the mechanism that the material underwent along annealing only based on the topological attributes generated from the available configurations of the system. This is a remarkable success of domain-based data analytics that opens up the possibility for analyzing the ever increasing number of simulation configurations that are becoming available to the condensed matter community.

## Conclusions

To conclude, we have shown that inspection of important thermodynamic properties of materials in the condensed phase is achievable by fusing the notions of topological attributes of the system and machine learning methods. Using a dataset consisting of 18672 configurations obtained from NPT MMC simulations of the NaK eutectic alloy, we have presented a machine learning protocol that allows us to reveal a mapping between independently accessible attributes of a system and its various thermodynamic properties. Firstly, we have shown that three phases of the eutectic NaK alloy can be identified, liquid, crystalline solid, and amorphous solid along the annealing procedure with NPT MMC simulations. From the current simulations, 18672 configurations registering the Cartesian coordinates of 2000 atoms were saved for further analysis. Secondly, based on the Voronoi tessellation, a set of 48 topological attributes were calculated for each configuration. Thirdly, with machine learning techniques these topological attributes were reduced to 26 based on ranking, and further to 3 through principal component analysis. The latter were used to cluster the configurations into three data clusters. These data clusters reproduced almost perfectly the liquid, amorphous, and crystalline condensed phases determined with the simulations. As a fourth step, a verification of the validity of the splitting into data clusters was carried out with the Random Forest classification. Analysis of these classes and the connection to the temperatures at which the configurations were obtained allowed to validate the clustering process and provided a robust estimate of the temperature range at which the system melts, 220–310 K, and at which the system transitions into a amorphous-like solid at 130–170 K.

The methodology presented here is relevant for identifying (or screening) unknown materials with a targeted combination of topological properties in an efficient manner with high fidelity. Our results highlight the ability of machine learning analyses for unraveling the embedded topological aspects of configuration space when inspecting condensed matter systems.

### Appendix

**Figure 7:**
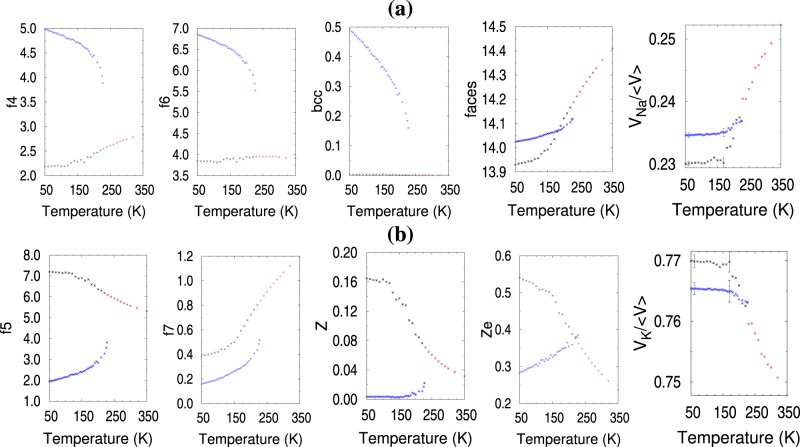
Topological attributes as a function of temperature; points are averages per atom. Colors are consistent with the three EM clusters: red is liquid, black is supercooled liquid-amorphous solid, blue is crystalline solid. (**a**) Attributes have higher values for crystalline solid than amorphous solid below 200 K; (**b**) attributes have higher values for the amorphous solid than crystalline solid below 200 K.

Ten of the top 48 topological attributes are depicted in Fig. 7(a,b). Average values per atom of the frequency of occurrence of each attribute at each simulation temperature are given for all attributes except V_*K*_ and V_*Na*_. For the latter two, a sum of all cell volumes of type K or Na is divided by the average volume of the full system. Colored points depict configurations belonging to the three EM clusters: liquid (red), crystalline solid (blue), amorphous solid (grey). Attributes in Fig. 7(a) displayed higher values for the crystalline structures below 200 K, while attributes in Fig. 7(b) favored the amorphous solid below 200 K. Note that f5, f7, Z and Ze in Fig. 7(b) have their highest and almost constant value for temperatures below 140 K and decrease at higher temperatures. This observation, plus the visible inflection point of the enthalpy and volume at that temperature, suggested that the supercooled liquid had transitioned to a solid amorphous state below approximately 140 K. The V_*K*_ and V_*Na*_ illustrate the volume split assigned by the tessellation to the K and Na atoms, respectively. Clearly shown is a Na volume contraction in the amorphous solid with respect to the crystalline solid, while the opposite effect is visible in the K volume.

## Electronic supplementary material

Dataset 1

## Data Availability

The Supplementary Information provides the dataset used in this work, with attributes as columns and data points as rows.
