# Supplementary material for: Simulating the NaK Eutectic Alloy with Monte Carlo and Machine Learning
Source: Sci Rep. 2019 Jan 24;9:704. doi: 10.1038/s41598-018-36574-y (PMC6346044; doi:10.1038/s41598-018-36574-y)
Supplement: Supplementary file 1 — Dataset 1 [file 41598_2018_36574_MOESM1_ESM.pdf]

SUPPLEMENTARY INFORMATION: Simulating the NaK Eutectic Alloy with Monte Carlo and Machine Learning, D. Reitz and E. Blaisten-Barojas, George Mason University, Fairfax, VA 22030, USA

f5	f6	f4	bcc	f7	f3	faces	CN14	Z	Ze	Z12	f8	CN12	Z13	CN16	v_m	Z15e	v_M	CN15	Z14e	CN13	CN17	Z12e	Z14	f9	CN18
5122	12896	9256	675	522	304	28112	1098	6	688	0	12	63	5	95	281.82	106	437.79	372	334	348	16	36	0	0	0
13860	7746	4558	7	1008	726	27970	626	278	970	144	70	248	107	179	276.61	240	440.85	487	373	426	24	90	11	2	1
3920	13630	9932	970	376	200	28062	1304	7	558	1	4	70	5	75	270.72	86	423.1	279	266	256	7	42	0	0	5
4778	13068	9444	722	510	294	28112	1155	0	669	0	16	68	0	68	278.7	107	434.34	353	338	325	26	45	0	2	3
4132	13570	9818	921	330	194	28054	1275	7	586	1	10	71	4	68	273.52	87	427.91	282	286	278	20	47	0	0	2
7200	11300	7944	343	1026	702	28254	929	35	756	13	78	90	17	144	287.51	125	446.16	418	399	373	35	40	0	4	3
14388	7506	4314	8	934	694	27914	646	333	975	169	70	269	126	166	272.39	234	435.89	475	406	416	23	85	16	8	1
6064	12240	8722	544	670	408	28134	1041	25	738	6	28	62	14	94	282.57	111	437.28	415	377	359	16	36	2	2	1
4328	13414	9702	872	396	224	28068	1260	11	620	3	4	55	6	73	273.13	81	426.75	292	311	298	15	32	0	0	2
4302	13450	9750	885	350	192	28058	1243	9	613	1	14	65	6	71	273.2	102	427.13	312	296	293	9	50	0	0	3
4620	13256	9558	814	396	228	28070	1228	8	665	2	12	60	5	84	275.91	96	431.18	306	343	302	13	41	0	0	0
14198	7614	4422	5	930	684	27926	629	331	997	151	70	241	139	175	273.63	229	437.37	467	411	458	22	80	22	8	1
12862	8088	4946	6	1176	910	28130	620	195	938	66	138	184	94	198	282.14	206	445.1	456	358	469	51	93	13	8	4
12232	7720	5110	0	1772	1300	28408	551	142	717	38	246	181	72	270	296.36	142	452.89	468	241	421	72	101	8	24	9
14286	7760	4406	6	790	594	27880	655	309	1076	152	42	251	125	169	269.39	262	431.97	465	446	447	11	92	16	2	0
4800	12918	9436	723	638	330	28134	1153	5	646	0	12	56	4	89	279.44	101	434.42	353	315	326	16	36	0	0	2
5100	12846	9244	685	566	342	28122	1134	9	676	1	24	55	5	85	278.79	112	436.05	362	333	335	19	37	0	0	0
5072	12964	9304	677	470	274	28100	1140	6	715	0	16	62	4	88	279.2	119	433.93	341	372	346	18	39	0	0	2
6004	12164	8700	557	768	492	28184	1099	19	696	8	56	69	10	124	285.7	106	443.14	348	371	332	20	42	0	0	2
4866	13084	9428	708	444	252	28090	1138	8	691	0	14	62	8	106	276.84	105	434.13	328	350	350	11	35	0	2	1
5250	12746	9154	615	606	364	28142	1072	11	711	0	22	62	11	98	278.88	116	434.97	394	355	350	14	44	0	0	2
14366	7684	4404	6	790	568	27856	635	334	1078	161	44	251	138	142	267.85	293	429.45	499	430	446	17	85	20	0	0
13586	7804	4602	4	1102	836	28028	606	269	943	132	98	231	106	184	274.52	219	440.9	493	365	447	31	85	14	0	1
4582	13200	9558	813	464	270	28096	1232	7	640	1	22	58	3	82	275.16	91	430.47	301	331	301	21	39	0	0	0
4074	13590	9872	935	324	178	28052	1310	6	606	1	14	64	3	70	271.95	78	425.64	271	316	263	14	47	0	0	2
14404	7676	4384	5	788	570	27866	653	313	1098	160	38	256	119	151	269.33	274	432.15	481	439	439	11	91	14	6	4
4066	13622	9886	947	294	160	28040	1267	7	614	1	12	57	2	52	270.42	107	423.93	306	270	292	18	37	0	0	1
14314	7678	4392	7	814	620	27890	642	312	1074	160	72	260	118	156	270.78	267	433.64	481	432	433	20	90	18	0	2
11224	7938	5354	2	2076	1652	28628	542	82	608	14	340	171	47	262	312.96	114	457.52	515	205	366	94	87	4	44	17
14322	7708	4438	5	776	564	27860	650	320	1098	163	44	266	117	148	270	284	432.2	489	449	420	17	97	18	6	1
13472	7786	4676	1	1172	858	28074	618	229	928	85	104	209	110	162	284.76	226	446.24	499	341	440	47	101	13	6	5
4578	13216	9554	789	474	268	28098	1192	9	621	0	8	56	7	77	275.49	99	429.13	343	312	309	13	40	0	0	2
3804	13788	10016	1002	264	152	28038	1329	6	578	1	14	68	3	66	270.12	81	422.59	253	290	262	16	38	0	0	3
10780	7626	5658	2	2440	1812	28820	499	71	440	13	428	166	39	292	330.99	78	472.18	465	128	353	141	81	2	68	32
11496	7890	5316	1	2032	1512	28548	557	102	612	21	278	167	53	271	305.5	117	461	474	193	385	91	90	4	20	20

SUPPLEMENTARY INFORMATION:Monte Carlo Atomistic Simulation and Machine Learning Analysis of Na-K Eutectic Alloy in Condensed Phases, D. Reitz and E. Blaisten-Barojas, George Mason University, Fairfax, VA 22030

10484	7876	5834	1	2400	1750	28820	478	52	483	5	432	148	21	305	334.38	90	471.01	485	142	371	125	82	2	40	35
4876	12906	9372	689	604	348	28136	1159	9	677	0	30	72	7	90	278.89	97	433.42	357	353	300	16	53	0	0	2
11912	7942	5180	3	1780	1362	28444	586	120	679	33	228	176	58	247	301.99	120	453.84	472	245	401	84	93	10	38	11
14448	7580	4316	3	810	640	27886	652	330	1049	153	88	266	131	153	272.04	259	436.22	475	431	420	22	98	21	2	4
12144	7838	5174	3	1698	1266	28394	516	147	684	34	238	193	76	267	302.56	144	453.18	472	216	411	83	103	11	28	14
13328	7628	4648	3	1360	1024	28148	595	235	803	88	144	212	120	207	283.1	173	446.44	485	301	436	46	92	12	16	4
5474	12502	9004	591	742	436	28182	1099	14	676	1	24	52	12	101	281.64	94	439.18	388	366	339	14	34	0	0	2
13908	7746	4564	2	980	692	27954	620	281	995	126	60	234	119	176	276.47	245	439.96	474	385	455	27	98	15	4	2
12396	7620	4946	0	1794	1368	28402	559	164	674	38	238	170	75	250	298.45	122	453.06	473	231	426	79	88	19	38	12
4436	13214	9606	820	512	312	28116	1203	8	581	1	36	68	6	97	277.58	101	433.51	331	289	290	6	42	0	0	2
7444	10990	7842	329	1138	748	28278	888	32	722	6	114	93	13	152	288.46	124	447.55	447	349	361	35	56	0	2	6
5012	13000	9360	701	458	250	28092	1173	7	708	0	12	63	4	79	277.56	106	432.95	333	372	327	18	47	0	0	3
4302	13374	9720	890	432	238	28078	1256	7	601	0	12	59	5	73	272.2	99	425.82	311	284	285	11	44	0	0	1
13658	7812	4612	1	1052	784	28006	612	280	934	115	82	220	125	183	276.08	223	439.87	487	367	459	27	83	18	6	1
12994	7774	4754	4	1378	1100	28206	561	192	829	68	194	194	90	227	283.34	185	447.27	485	286	449	48	98	9	12	10
6864	11638	8276	376	864	510	28192	964	29	819	8	38	74	16	123	287.73	135	442.31	423	418	383	22	40	1	2	3
4248	13468	9744	872	370	224	28072	1247	6	608	1	16	63	4	68	271.55	90	425	312	311	287	13	41	0	2	4
4038	13620	9880	935	326	180	28050	1253	7	574	1	6	58	4	73	272.97	87	425.83	289	270	304	15	40	0	0	2
4244	13508	9746	905	340	210	28060	1270	10	602	1	10	55	5	73	273	92	426.51	287	297	295	13	32	0	2	2
5386	12554	9094	608	702	394	28156	1119	7	706	1	24	71	3	108	279.55	101	436.95	343	379	325	23	45	0	2	4
4078	13488	9834	917	432	234	28074	1290	9	575	2	8	56	4	75	271.41	82	424.12	284	289	274	9	36	0	0	5
11712	8174	5200	4	1690	1394	28456	543	101	693	21	258	170	57	254	300.69	137	454.08	518	235	400	72	96	4	24	12
4006	13632	9896	962	328	182	28052	1297	6	576	2	8	60	3	69	271.71	85	425.91	268	281	287	16	40	0	0	2
14566	7670	4320	5	690	536	27840	650	337	1100	157	50	268	131	138	270.92	272	433.15	494	449	429	14	95	27	8	2
13910	7564	4434	3	1160	864	28034	609	262	908	99	96	220	127	189	278.7	199	446.74	466	362	461	38	102	16	6	4
5200	12804	9216	633	572	320	28122	1092	4	721	0	10	61	4	91	277.76	102	433.2	371	352	362	18	49	0	0	2
12256	7928	5002	4	1618	1302	28374	566	143	715	36	256	187	80	229	292.18	150	451.77	497	244	409	72	112	13	12	14
12888	8106	4888	11	1174	926	28114	642	227	909	98	128	184	98	190	276.58	204	441.97	486	346	445	29	70	12	4	7
4278	13372	9718	839	444	258	28092	1196	9	584	1	22	57	6	72	273.41	91	427.82	345	280	316	9	36	0	0	3
4450	13280	9650	790	454	240	28082	1200	10	628	1	8	63	7	72	272.96	88	427.98	326	328	310	19	41	0	0	3
11290	7844	5384	0	2106	1620	28628	501	92	572	19	342	172	43	281	310.08	107	458.92	502	203	380	101	82	5	40	23
3874	13660	9976	988	356	182	28056	1306	5	550	0	8	53	1	63	271.41	74	423.37	275	263	279	14	39	0	0	4
6424	12036	8516	463	696	442	28156	998	15	809	5	40	74	7	90	282.43	147	440.03	426	402	374	29	46	0	2	2
14364	7554	4330	11	894	682	27910	625	318	1019	162	82	260	114	160	272.4	243	434.71	497	404	424	21	92	22	4	2
12762	7794	4866	1	1504	1140	28266	583	172	810	61	190	190	83	212	290.82	200	449.41	516	288	417	54	100	10	10	7
13848	7652	4390	5	1124	924	28066	624	261	852	125	114	238	100	191	277.85	224	445.61	479	326	421	37	89	20	8	3

4578	13244	9586	778	424	234	28080	1158	2	661	0	14	57	1	82	276.99	104	433.11	333	305	349	17	42	0	0	1
10606	7806	5772	2	2402	1752	28814	500	61	457	6	424	155	30	303	330.77	83	471.29	473	140	353	129	75	2	52	30
13718	7744	4608	4	1044	776	27996	626	289	989	138	100	237	109	185	276.31	224	443.29	471	397	431	34	83	24	6	1
13450	7952	4778	7	1006	726	27996	653	263	982	110	76	210	110	175	278.41	226	443.16	484	411	436	23	82	15	6	1
12266	7864	5100	5	1634	1228	28340	574	143	719	42	232	193	74	232	293.01	137	452.17	484	260	414	71	105	5	12	11
4014	13526	9868	940	420	236	28082	1294	8	565	3	16	54	2	73	272.46	84	424.95	283	282	275	13	33	0	2	3
13070	7726	4770	5	1424	1050	28204	567	207	806	48	156	195	110	215	292.2	165	449.6	479	293	440	65	110	17	8	6
12280	8100	5108	4	1476	1132	28292	641	137	823	41	178	180	72	214	288.45	167	451.41	480	338	401	58	96	5	12	7
4546	13134	9528	792	560	338	28134	1204	4	616	0	28	53	3	85	278.03	96	432.47	330	311	304	17	37	0	0	2
4850	13078	9444	727	444	252	28092	1160	7	692	0	22	67	6	105	276.25	110	432.91	334	342	318	7	47	0	2	1
5352	12622	9108	608	654	386	28158	1095	6	704	0	36	66	5	89	280.27	128	436.81	397	352	329	15	46	0	0	4
12892	7774	4816	0	1428	1122	28252	600	192	803	56	200	185	95	213	287.56	178	449.83	492	298	420	60	96	11	18	4
13342	7974	4728	6	1064	836	28056	610	231	965	99	94	222	105	192	277.43	234	443.55	497	364	435	30	98	12	18	1
11122	7906	5524	0	2122	1586	28644	514	76	594	19	348	170	31	278	323.27	114	470.13	473	194	356	109	82	4	34	33
4786	12948	9408	719	626	356	28146	1189	7	612	0	22	56	5	91	278.25	74	435.89	336	346	301	18	43	0	0	2
4180	13444	9784	880	432	232	28078	1259	7	580	1	6	60	4	76	273.55	80	427.45	287	295	293	20	37	0	0	1
14126	7692	4456	7	896	672	27918	624	314	1026	153	70	254	125	165	274.19	263	436.8	492	403	430	22	88	12	6	1
5882	12406	8836	586	610	366	28120	1083	26	734	8	18	84	13	99	282.31	110	439.18	382	373	330	15	55	1	2	2
13652	7806	4596	3	1090	800	28018	660	245	967	110	68	224	107	177	274.98	224	439.71	473	384	429	29	97	14	6	1
4726	13128	9524	795	464	234	28082	1188	7	647	3	6	68	3	85	276.53	103	431.31	331	311	314	11	49	0	0	1
4014	13598	9884	935	350	200	28062	1283	5	595	2	16	55	2	65	271.12	88	424.03	292	289	286	12	36	0	0	3
13852	7858	4542	5	904	712	27952	640	273	1027	133	84	245	109	177	273.65	249	436.75	468	409	433	25	97	10	0	2
5244	12846	9210	655	492	288	28098	1099	10	743	0	18	88	7	107	278.19	117	435.72	358	355	326	16	62	0	0	0
7242	11280	7994	375	954	644	28212	900	49	726	14	86	96	23	146	286.95	111	443.72	414	356	382	41	49	3	12	1
4646	13138	9530	728	498	276	28106	1170	1	648	0	18	58	0	74	276.53	92	432.37	351	349	322	19	41	0	0	1
4178	13568	9802	898	318	180	28048	1247	7	617	0	2	61	5	63	273.36	107	427.34	302	299	303	14	47	0	0	5
14028	7592	4414	4	1034	804	27986	633	289	944	139	108	247	108	169	273.96	231	436.72	500	383	415	25	92	17	6	1
4370	13312	9664	849	470	266	28094	1229	8	589	1	10	56	5	76	275.88	78	431.73	305	295	311	18	40	1	2	3
4442	13140	9586	787	598	346	28138	1171	2	605	0	24	68	1	73	276.65	108	431.6	366	286	290	20	46	0	2	4
3856	13730	9974	997	302	174	28048	1299	7	565	1	12	56	3	68	271.49	97	424.01	283	256	272	12	34	0	0	1
4588	13220	9560	793	444	262	28098	1159	4	638	0	22	64	4	85	276.84	117	431.78	334	291	336	17	42	0	2	2
3926	13636	9926	977	366	200	28060	1319	8	552	1	6	59	5	71	269.34	78	422.33	265	273	268	12	41	0	0	3
4390	13434	9688	882	336	198	28058	1242	11	637	1	12	46	7	59	272.37	112	426.32	315	306	313	14	32	0	0	3
13112	7846	4758	4	1304	998	28170	608	203	847	74	138	203	98	178	283.23	178	447.07	513	321	426	55	103	14	14	3
4298	13410	9730	892	396	222	28070	1260	10	605	0	14	59	6	73	273.08	86	427.45	291	299	293	17	44	0	0	2
4654	13164	9556	757	458	244	28096	1166	4	639	1	20	75	3	91	276.03	92	433.23	338	315	310	16	45	0	0	0

SUPPLEMENTARY INFORMATION:Monte Carlo Atomistic Simulation and Machine Learning Analysis of Na-K Eutectic Alloy in Condensed Phases, D. Reitz and E. Blaisten-Barojas, George Mason University, Fairfax, VA 22030

5350	12738	9140	645	548	316	28106	1105	14	732	4	12	77	6	93	279.86	132	437.59	363	345	335	19	47	0	2	2
14314	7806	4352	6	724	614	27876	640	320	1087	155	62	248	129	137	270.79	292	434.13	498	442	449	21	78	16	4	1
4682	13242	9522	807	388	226	28068	1234	15	669	3	8	57	8	72	275.85	101	430.73	310	348	302	14	41	1	0	3
4874	13002	9412	731	502	284	28098	1144	11	649	0	24	66	7	84	277.81	94	433.24	348	323	328	21	49	0	0	1
4514	13268	9620	806	442	232	28080	1207	7	641	1	4	58	5	80	274.93	102	429.64	318	321	319	14	37	0	0	1
4468	13248	9602	810	490	282	28104	1182	10	593	0	14	60	5	90	276.39	92	431.42	329	289	313	18	44	0	0	0
13518	7642	4626	3	1240	920	28080	607	256	865	110	114	233	105	198	278.39	174	445.42	460	330	437	41	94	17	18	11
5016	12906	9336	701	536	296	28110	1145	9	683	2	20	59	4	85	279.27	106	435.94	345	351	336	22	36	0	0	1
3916	13726	9944	970	286	166	28046	1291	6	588	2	8	53	4	76	270.2	87	421.83	271	267	297	8	38	0	0	2
4822	13034	9404	759	528	312	28118	1206	3	667	1	16	57	1	90	277.05	99	432.22	316	345	310	17	35	0	2	1
13730	7844	4590	5	976	752	27984	611	277	995	122	86	240	115	176	277.2	247	441.5	488	384	443	31	96	18	6	1
4422	13308	9684	829	424	218	28066	1247	11	622	1	10	64	8	74	273.69	77	428.42	294	346	291	20	44	0	0	2
12230	7894	5074	3	1660	1266	28364	566	136	731	33	204	182	70	236	295.82	164	451.2	515	245	390	66	108	5	36	9
14278	7674	4374	8	842	656	27898	617	333	1019	160	70	253	131	142	271.85	265	435.43	510	407	446	23	82	22	4	2
13568	7768	4656	5	1122	814	28026	607	263	911	121	94	239	104	174	276.94	207	444.16	496	331	437	35	95	17	4	4
4184	13582	9832	917	282	150	28038	1271	6	623	0	8	69	4	66	272.68	95	426.44	279	308	291	18	48	1	0	3
13494	7626	4610	4	1272	962	28124	599	228	857	86	152	208	114	205	283.26	198	446.69	480	321	440	47	96	10	8	2
14278	7626	4412	5	886	634	27894	622	325	1029	152	56	248	128	161	272.2	258	436.69	485	403	457	18	85	23	2	2
14486	7674	4332	5	746	564	27850	649	336	1085	160	48	252	134	160	270.1	275	433.03	461	439	452	15	87	20	0	2
12592	7922	5050	5	1414	1056	28246	564	183	820	43	190	173	102	241	291.71	167	449.35	472	299	456	52	94	12	22	10
12186	7832	5122	3	1760	1264	28388	571	139	711	33	212	174	72	252	298.45	123	456.6	453	263	422	84	91	8	10	12
14056	7664	4450	7	954	730	27942	623	300	1000	141	72	247	119	164	271.81	250	437.34	487	395	443	24	96	22	16	4
4596	13150	9560	763	510	274	28104	1194	4	633	1	14	69	1	74	277.29	97	432.77	346	323	295	17	53	0	0	1
12132	7996	5188	1	1642	1186	28344	584	121	756	24	180	163	66	246	294.13	152	451.92	497	270	411	57	99	7	16	7
4008	13634	9888	936	334	186	28054	1285	8	579	2	4	62	4	61	274.52	90	427.84	290	289	279	19	40	0	0	0
6422	11770	8490	457	940	540	28202	989	35	685	8	38	92	16	124	285.47	101	443.16	423	358	332	22	53	2	2	5
12060	7746	5056	0	1860	1440	28462	595	136	644	33	274	173	64	243	302.85	125	458.6	472	248	376	89	91	8	20	15
4398	13284	9650	854	474	270	28092	1251	5	614	1	14	60	3	77	275.45	86	430.66	300	307	289	17	40	0	2	2
4288	13392	9706	870	436	252	28084	1242	4	607	2	10	57	2	67	273.81	91	427.81	313	296	299	17	43	0	0	2
13840	7610	4370	1	1134	958	28068	570	257	902	107	148	231	104	194	278.87	196	445.56	476	319	463	51	101	20	8	3
14158	7762	4450	5	830	634	27898	635	283	1059	152	62	260	103	150	272.07	268	435.6	492	418	434	21	91	10	2	3
14338	7666	4444	5	804	560	27862	659	344	1053	163	48	263	137	153	270.3	245	433.21	478	456	421	16	84	19	2	1
4492	13264	9604	824	444	264	28090	1196	12	613	2	18	68	8	82	273.19	98	427.21	332	299	299	14	43	0	4	2
11534	7906	5342	1	1944	1478	28538	559	123	615	32	296	167	62	292	306.22	128	457.79	455	216	389	83	83	5	36	16
14330	7618	4352	8	874	660	27900	644	322	1028	151	58	242	128	152	270.04	261	433.9	501	423	442	13	74	25	8	0
4104	13564	9852	910	334	186	28056	1251	7	598	2	16	68	5	69	273.05	102	426.13	303	289	284	16	48	0	0	2

SUPPLEMENTARY INFORMATION:Monte Carlo Atomistic Simulation and Machine Learning Analysis of Na-K Eutectic Alloy in Condensed Phases, D. Reitz and E. Blaisten-Barojas, George Mason University, Fairfax, VA 22030

13216	7748	4696	3	1328	1018	28168	615	211	805	83	154	217	93	214	282.03	160	448.24	458	316	428	49	101	20	6	7
4384	13286	9650	832	474	282	28102	1255	7	606	1	22	55	5	80	274.99	80	428.77	295	335	286	18	34	0	4	2
13320	7690	4646	2	1302	1014	28144	591	225	844	98	164	228	92	202	281.77	191	448.8	492	306	423	41	100	15	8	7
4514	13320	9618	818	386	220	28070	1207	9	644	0	12	61	6	76	273.73	96	428.36	325	319	309	14	41	0	0	1
4652	13164	9504	785	480	284	28100	1213	6	645	1	16	56	3	76	275.45	91	430.9	329	339	305	13	39	0	0	3
14034	7812	4546	8	824	616	27904	642	300	1051	141	66	243	113	150	275.02	252	438.17	466	432	451	33	89	20	6	3
12990	7732	4816	1	1442	1062	28218	575	192	798	59	162	198	92	213	290.83	186	449.65	503	286	422	52	96	14	14	7
4190	13560	9804	927	310	176	28048	1280	8	614	1	8	58	5	66	273.87	95	427.02	285	297	286	17	47	0	0	1
7400	11112	7834	342	1080	736	28258	889	50	726	10	92	98	28	148	290.04	131	446.5	447	340	366	32	59	1	4	6
3872	13666	9942	994	372	208	28064	1340	7	553	2	4	57	3	74	271.12	78	423.46	256	276	257	12	37	0	0	1
12848	7890	4888	6	1370	1030	28194	612	206	835	68	160	203	95	203	288.04	179	447.97	500	312	409	46	104	11	8	7
4108	13518	9856	959	378	190	28056	1291	8	571	0	6	58	5	72	273.97	92	426.71	283	270	277	11	43	0	0	2
13068	7694	4736	0	1438	1098	28220	578	203	817	64	162	191	102	200	284.3	178	449.85	508	292	438	54	96	10	24	8
12352	7956	4978	7	1612	1242	28340	574	169	752	49	180	168	87	224	290.16	179	449.73	527	275	421	55	81	10	18	6
4378	13306	9662	847	452	266	28088	1229	7	603	0	24	63	3	84	275.11	81	431.15	301	304	295	16	45	0	0	4
4326	13370	9706	872	424	238	28078	1261	8	616	0	14	63	5	79	276.1	103	429.61	298	317	276	11	49	1	0	4
13806	7726	4576	7	1014	758	27988	623	300	954	113	106	219	129	186	275.35	229	442.59	470	364	445	30	88	22	2	3
13588	7686	4560	3	1168	924	28076	596	247	882	87	140	205	125	185	278.3	197	444.55	494	338	465	35	94	14	8	5
12392	7954	5006	2	1572	1192	28312	599	171	765	48	180	187	91	252	291.97	141	451.46	478	289	404	49	100	8	16	8
5238	12670	9142	609	674	406	28162	1090	7	685	0	30	62	5	107	280.79	100	438.09	369	357	345	18	44	0	2	2
12346	8000	5062	4	1512	1160	28296	575	139	809	35	210	172	76	217	289.21	170	451.21	491	295	449	64	92	6	6	9
5170	12812	9226	635	562	330	28124	1095	6	698	2	22	72	4	101	278.37	105	435.93	363	359	344	18	48	0	2	2
4346	13452	9728	893	332	182	28048	1254	8	631	1	8	71	5	73	274.21	97	428.08	298	301	285	10	53	0	0	4
4880	13006	9402	689	498	292	28108	1152	10	699	0	28	66	7	87	279.04	93	434.28	332	377	327	27	48	0	2	1
14002	7674	4420	6	986	786	27970	599	291	989	137	92	254	114	168	273.25	265	438.28	511	374	432	25	94	17	10	3
5340	12694	9132	624	608	344	28132	1103	8	686	1	14	75	5	89	281.58	115	435.34	385	359	317	17	50	0	0	4
3900	13688	9958	970	314	174	28046	1287	7	560	2	12	63	5	67	269.9	91	422.64	284	268	277	15	39	0	0	1
13460	7950	4700	11	1006	796	28032	684	261	980	130	118	225	101	163	277.45	223	441.32	476	426	405	32	84	14	2	5
5156	12794	9230	637	600	336	28128	1089	11	703	0	12	62	7	90	278.28	110	436.67	386	347	347	15	47	0	0	3
13968	7740	4490	10	928	724	27940	618	293	1014	151	78	253	107	166	271.83	253	435.6	495	399	430	26	90	17	12	1
13538	7774	4622	3	1118	880	28082	631	225	925	91	146	208	101	176	281.89	205	446.4	472	374	436	50	97	8	4	6
13772	7782	4510	1	978	826	28010	589	277	931	116	134	244	127	185	277.53	211	445.48	476	363	451	40	103	13	8	4
4032	13576	9872	942	364	204	28060	1288	13	582	1	12	53	6	65	270.89	90	423.8	288	290	278	16	39	0	0	2
11846	7916	5228	2	1758	1394	28486	547	116	681	33	308	163	58	260	302.82	125	455.23	491	233	422	73	79	10	34	15
14356	7566	4336	4	902	676	27908	631	336	988	169	62	275	118	161	271.72	243	434.51	499	400	406	19	94	30	10	2
4136	13518	9814	903	376	212	28068	1269	5	593	1	12	57	3	74	273.23	71	427.36	287	298	287	14	43	0	0	4

SUPPLEMENTARY INFORMATION:Monte Carlo Atomistic Simulation and Machine Learning Analysis of Na-K Eutectic Alloy in Condensed Phases, D. Reitz and E. Blaisten-Barojas, George Mason University, Fairfax, VA 22030

14380	7702	4352	7	778	600	27864	618	348	1047	167	50	255	143	151	270.81	282	433.29	501	420	453	15	82	13	2	0
14242	7650	4376	5	874	688	27918	651	323	1019	157	82	247	124	152	270.56	252	433.31	491	432	428	22	76	23	4	1
12498	7686	4930	4	1692	1288	28340	597	184	702	61	210	196	81	234	293.22	135	448.79	497	265	384	55	95	17	34	13
6608	11680	8298	405	958	632	28246	988	17	736	3	68	78	11	123	287.65	126	446.51	422	392	352	28	49	1	2	3
5404	12644	9076	613	634	370	28144	1107	9	737	2	16	65	5	98	279.94	123	437.95	372	375	340	12	42	0	0	4
13364	7836	4636	8	1200	938	28096	618	225	910	98	116	220	99	188	277.11	205	445.06	498	368	430	36	93	14	6	2
3864	13694	9962	1002	328	190	28052	1329	5	567	1	12	60	3	75	270.53	75	422.06	247	283	268	13	42	0	2	4
13418	7702	4604	2	1258	984	28120	620	245	841	105	144	208	99	219	277.2	183	445.31	457	328	441	38	81	22	8	3
5716	12412	8892	537	688	418	28158	1052	15	725	1	30	73	10	106	283.24	117	439.83	389	384	342	23	46	0	2	2
3778	13766	10036	1008	304	154	28042	1324	6	551	1	4	59	4	64	269.63	90	422.09	271	268	264	12	37	0	0	1
14306	7646	4374	7	852	648	27898	633	329	1017	159	70	271	121	143	273.12	260	436.97	512	401	407	22	101	21	2	3
13328	7824	4638	2	1232	968	28122	590	247	861	88	122	211	123	196	279.86	187	446.56	489	327	457	45	92	17	8	3
7336	11272	7944	379	956	622	28196	946	49	729	13	64	97	21	147	287.83	101	444.39	376	357	373	40	50	6	2	4
10990	7928	5562	0	2176	1632	28684	514	85	562	17	358	159	47	279	316.28	94	463.95	493	198	392	106	90	2	34	27
4144	13564	9834	899	322	178	28054	1244	7	627	0	12	67	6	80	274.37	91	429.44	298	295	289	13	50	0	0	1
14302	7606	4422	7	860	622	27886	630	335	1011	171	70	267	122	153	272.81	255	438.33	472	398	436	34	81	16	4	0
4152	13486	9802	901	394	222	28070	1265	7	585	1	14	63	5	72	274.6	97	428.53	286	291	289	17	44	0	0	4
4004	13564	9894	945	382	206	28064	1277	8	564	1	14	65	6	61	272.34	80	425.87	302	280	275	14	41	0	0	3
4056	13562	9840	939	372	224	28072	1264	8	577	1	18	55	5	68	271.34	82	423.93	297	267	296	14	37	0	0	3
4950	12968	9342	703	522	312	28118	1157	5	677	0	24	51	3	80	281.53	118	437.5	362	348	331	12	36	0	0	2
3856	13668	9964	993	362	200	28060	1337	8	553	1	8	66	4	67	270.74	80	423.66	271	274	242	11	44	0	2	2
4154	13550	9806	918	334	200	28060	1273	8	595	1	16	58	4	71	273.59	82	427.24	285	296	290	17	46	0	0	1
4464	13230	9598	789	508	292	28106	1191	7	588	1	14	73	5	84	275.96	81	430.49	320	316	305	18	43	0	0	6
12232	7894	4990	1	1680	1328	28372	585	159	703	42	230	171	80	255	298.03	136	453.93	465	267	402	79	84	5	16	6
4518	13240	9598	781	468	258	28092	1215	5	650	0	10	55	3	74	275.47	90	430.68	314	346	313	21	39	0	0	2
4460	13310	9640	828	420	236	28080	1232	4	629	0	14	58	2	81	275.02	105	429.56	304	309	304	15	38	0	0	1
11790	7708	5188	1	2032	1500	28526	556	134	571	26	290	161	75	267	303.97	111	457.11	486	197	404	84	83	11	18	12
14662	7526	4258	10	760	572	27844	634	360	1062	176	64	263	139	153	270.14	260	433.25	473	441	448	19	73	21	2	2
13442	7730	4632	4	1232	938	28110	575	241	885	81	120	207	116	218	282.04	215	446.6	488	319	453	33	104	11	16	5
11720	7958	5292	1	1840	1376	28472	546	110	706	27	266	168	57	279	301.16	142	454.71	484	243	415	68	101	7	20	13
5716	12478	8916	567	600	382	28136	1059	14	738	2	40	79	11	93	281.08	113	438.64	401	372	341	18	56	0	2	3
4466	13342	9612	834	412	242	28076	1224	6	626	1	2	61	4	80	273.77	101	427.83	321	308	293	11	36	0	0	1
5284	12670	9150	647	644	370	28142	1138	6	686	1	22	63	2	106	279.06	107	436.4	350	361	319	14	46	0	2	2
10668	7816	5650	0	2412	1804	28806	520	76	487	13	402	154	41	311	321.98	68	466.67	473	167	364	115	80	6	52	26
4920	13096	9398	690	422	244	28092	1156	5	713	0	10	55	4	81	277.98	95	433.11	341	382	343	19	38	0	2	0
4478	13164	9606	795	552	298	28114	1204	4	610	0	16	64	3	79	274.99	82	429.56	324	327	300	21	36	0	0	3

SUPPLEMENTARY INFORMATION:Monte Carlo Atomistic Simulation and Machine Learning Analysis of Na-K Eutectic Alloy in Condensed Phases, D. Reitz and E. Blaisten-Barojas, George Mason University, Fairfax, VA 22030

4086	13526	9834	912	398	220	28072	1278	4	586	1	8	57	2	76	270.48	86	423.68	278	300	289	15	36	0	0	3
14192	7788	4462	7	780	592	27868	652	329	1062	169	50	260	116	155	267.42	270	429.67	481	437	424	13	82	18	4	4
13758	7736	4576	5	1062	780	28006	636	278	957	128	94	242	117	178	275.23	227	440.2	482	382	415	30	91	13	0	4
5144	12802	9234	673	596	338	28128	1132	9	668	0	12	72	9	92	278.68	103	436.23	357	354	320	16	50	0	2	4
4066	13584	9884	937	334	172	28048	1273	7	597	1	8	70	3	61	271.57	105	424.26	298	280	274	19	49	0	0	0
4864	12964	9386	708	576	320	28120	1136	6	655	2	10	68	2	85	280.5	98	436.48	359	327	324	19	44	0	0	3
5164	12878	9240	647	516	298	28106	1129	9	704	3	8	70	4	80	278.58	94	435.74	364	382	333	19	40	0	2	2
4418	13294	9658	845	440	250	28082	1215	7	607	0	22	63	4	80	275.95	97	431.47	312	292	299	19	45	0	0	2
13714	7672	4482	1	1152	916	28066	613	245	894	94	116	216	117	197	277.63	208	444.74	461	342	451	41	99	14	14	5
11224	8170	5526	1	1898	1412	28528	531	84	675	14	282	166	50	267	313.17	126	461.43	472	218	413	93	95	4	14	20
3914	13624	9912	956	390	224	28074	1309	5	552	0	10	51	2	60	272.02	81	425.55	282	270	270	19	38	0	0	2
13542	7982	4738	12	950	690	27970	602	250	1023	119	66	241	107	170	276.16	231	438.52	505	397	448	25	98	7	2	1
5336	12650	9116	585	646	374	28144	1068	8	704	1	22	66	4	95	283.71	100	438.37	386	384	359	23	37	0	0	0
4484	13180	9564	811	546	326	28122	1231	6	590	0	22	49	4	88	277.58	89	434.62	307	314	298	17	32	0	0	2
13330	7918	4710	6	1104	870	28056	629	245	933	105	116	222	102	179	277.18	196	443.56	479	378	438	41	97	15	8	2
4418	13360	9626	859	406	256	28082	1228	4	602	1	16	65	2	83	276.24	91	431.05	309	285	295	13	42	0	0	2
13812	7584	4506	1	1164	864	28048	647	268	906	108	102	230	111	198	278.82	185	447.15	447	347	419	40	94	23	16	3
4166	13536	9830	904	336	174	28048	1263	6	607	1	6	70	3	70	272.57	93	425.38	289	296	284	18	47	0	0	1
4686	13150	9504	759	470	272	28102	1178	3	661	0	20	53	1	87	276.76	91	433.17	336	325	326	14	37	0	0	0
11682	7898	5258	1	1910	1454	28518	574	104	627	20	298	149	66	281	304.41	134	453.91	484	229	397	67	69	5	18	16
6522	11828	8426	442	838	532	28210	943	26	722	5	60	73	15	126	285.61	127	443.64	435	350	383	25	47	0	4	2
11258	7952	5560	0	2014	1452	28576	512	75	620	9	306	188	32	274	313.59	103	462.7	479	213	376	108	104	6	28	19
4616	13176	9528	770	486	284	28108	1203	3	664	0	16	57	3	84	277.4	105	433.4	314	331	318	17	39	0	2	4
6814	11666	8244	392	860	562	28210	943	22	766	4	64	76	13	136	287.18	122	445.87	420	377	387	25	51	1	0	3
14478	7686	4312	6	762	576	27848	645	339	1068	158	34	257	137	146	270.35	299	433.44	492	417	433	13	91	20	0	2
12560	7778	4924	3	1618	1228	28332	560	171	734	48	202	189	85	228	295.42	154	451.96	490	246	426	76	106	9	18	7
12746	7788	4906	0	1520	1110	28248	615	160	819	57	166	201	74	216	286.09	182	449.27	505	295	396	47	107	11	12	4
11846	7882	5238	2	1822	1374	28456	578	135	658	27	262	179	73	236	308.24	146	456.84	473	242	383	91	93	8	30	22
7034	11418	8104	379	954	636	28238	929	26	752	10	84	91	15	148	289.13	121	443.05	425	360	370	23	57	0	6	6
13978	7698	4474	5	998	748	27978	646	281	982	124	74	214	124	153	276.58	237	440.03	485	403	454	31	76	15	8	4
5680	12298	8888	567	800	476	28184	1071	15	663	4	42	70	8	112	283.26	98	440.9	391	353	322	21	47	0	0	1
13862	7674	4520	8	1042	794	28004	645	283	981	125	96	238	116	184	275.91	224	443.92	476	394	418	24	97	23	14	3
14298	7562	4332	6	912	722	27932	582	326	1014	157	102	275	132	156	272.2	283	436.78	536	366	418	24	107	17	4	1
4498	13270	9592	798	450	268	28096	1221	7	626	0	18	54	5	77	275.6	95	431.63	315	329	306	18	36	0	0	2
11158	7824	5582	0	2182	1522	28606	520	94	560	17	310	181	47	312	321.4	102	462.71	454	178	370	95	96	9	26	20
4692	13130	9480	782	488	292	28102	1185	7	636	0	20	55	4	79	274.56	94	428.91	340	319	313	15	41	0	0	3

14182	7708	4434	7	876	642	27892	628	310	1037	169	50	263	109	161	269.41	253	432.41	479	404	442	21	82	19	0	2
11626	7772	5270	1	2008	1516	28534	513	116	631	21	320	171	63	278	309.6	132	458.57	499	206	391	85	95	7	22	19
4064	13536	9840	934	404	224	28074	1313	9	581	1	6	61	7	70	272.1	89	425.18	278	299	257	14	41	0	0	2
4164	13448	9772	908	432	252	28084	1261	10	577	1	16	58	7	81	272.1	88	426.16	293	279	287	13	42	0	0	2
13672	7698	4568	4	1128	860	28052	631	257	920	113	118	226	110	181	278	211	446.23	469	369	429	47	94	14	8	2
13200	7828	4676	3	1268	1020	28152	588	215	873	69	138	212	115	198	282.22	213	446.84	488	306	449	43	106	13	20	10
14232	7622	4426	7	906	652	27900	601	325	1021	163	56	271	123	167	273.74	258	438.6	485	388	438	28	96	16	4	1
4742	13058	9444	736	534	316	28116	1153	4	639	0	22	66	3	96	279.43	92	435.23	343	307	324	13	47	0	0	2
11846	7864	5236	1	1906	1364	28452	581	122	652	26	206	158	65	260	301.74	129	455.08	495	237	395	63	90	9	28	12
14386	7662	4364	3	796	600	27866	624	330	1053	172	56	280	120	173	270.38	240	432.05	467	424	430	19	101	19	2	1
14372	7628	4370	8	822	618	27880	644	318	1059	159	66	265	129	148	271.76	269	435.51	478	434	433	23	95	18	4	4
4436	13392	9680	851	356	188	28054	1236	5	644	1	2	75	2	79	273.09	97	427.26	298	316	291	17	51	0	0	0
4576	13312	9578	816	366	222	28070	1222	7	676	2	16	59	2	83	274.17	101	429.56	293	343	315	19	43	0	0	2
10770	7826	5588	2	2280	1818	28806	511	61	488	10	460	159	36	280	333.48	86	468.53	506	160	341	128	71	5	56	32
4590	13216	9566	812	448	254	28092	1216	5	658	1	18	56	1	81	277.81	102	432.11	318	323	307	15	40	0	0	1
4204	13514	9806	901	342	180	28052	1245	6	608	1	4	62	3	62	274.01	103	427.45	321	287	291	12	43	0	2	1
14032	7770	4506	6	876	658	27912	626	316	1012	155	68	247	122	161	270.5	256	433.73	488	398	450	17	76	17	2	4
11738	7948	5238	0	1842	1426	28498	539	135	621	14	274	153	76	260	307.1	109	459.38	490	212	411	81	97	10	28	20
4494	13284	9622	791	420	242	28084	1206	7	617	0	22	59	3	72	275.8	85	434.01	326	331	305	19	39	0	0	3
4786	12956	9424	708	604	340	28134	1171	6	642	0	22	62	3	91	279.53	99	435.16	340	347	313	15	39	0	2	3
12050	7994	5204	2	1666	1230	28374	580	132	739	31	208	177	72	239	293.3	145	451.33	480	265	405	77	103	10	22	9
3966	13682	9924	962	286	168	28042	1293	5	589	1	16	67	3	67	270.93	91	423.77	278	277	267	18	48	0	0	1
14440	7792	4416	9	638	486	27816	639	325	1147	170	40	262	120	135	267.71	296	429.55	481	463	459	19	83	18	4	1
5170	12684	9206	666	684	386	28158	1118	9	650	2	28	66	5	95	281.15	104	438.09	384	331	312	16	45	0	0	1
12894	7754	4868	4	1456	1070	28230	606	176	801	52	180	183	95	217	286.66	165	448.25	474	300	436	57	100	11	8	4
4388	13418	9668	840	366	222	28074	1235	5	643	0	12	48	4	78	273.23	106	426.64	304	316	318	12	32	0	0	1
4774	13208	9480	764	374	220	28066	1192	10	706	0	10	65	4	79	274.65	119	429.48	331	361	311	7	43	0	0	6
4114	13560	9832	900	346	196	28058	1256	6	599	1	10	61	3	84	272.43	85	425.39	288	298	292	11	42	0	0	1
12846	8072	4936	5	1198	932	28138	643	206	922	79	146	190	94	192	282.36	198	446.67	492	367	431	36	89	14	8	2
13470	7664	4600	2	1260	968	28108	627	248	858	111	126	238	104	194	277.89	212	446.78	480	345	399	42	100	17	18	6
14450	7588	4334	0	818	616	27880	610	335	1054	170	74	270	131	154	270.01	266	434.66	514	426	435	14	86	16	0	0
5580	12554	9004	586	612	356	28128	1101	12	749	3	22	64	6	102	282.77	110	437.2	374	393	334	15	43	0	0	0
13756	7860	4550	6	974	764	27988	648	272	983	124	78	224	116	167	274.79	231	439.06	469	408	439	35	83	15	6	3
13784	7862	4586	6	938	714	27960	627	273	1019	130	68	245	103	188	275.8	237	440.68	472	398	428	24	92	19	8	1
7388	11096	7860	341	1110	728	28268	900	42	731	9	76	102	23	162	288.47	119	444.42	434	355	353	30	71	3	10	5
10538	7856	5718	0	2434	1820	28836	503	50	465	13	414	169	24	313	334.92	86	471.84	470	166	343	126	74	2	52	33

14286	7700	4432	6	814	588	27870	646	319	1083	163	50	265	119	171	270.29	258	431.85	462	443	434	16	92	15	0	0
12216	7876	5122	2	1642	1240	28356	568	128	767	44	242	206	59	236	291.45	161	451.9	505	257	384	67	120	7	18	11
13378	7658	4624	2	1312	1016	28162	627	192	881	69	160	202	92	192	282.07	183	448.18	484	320	429	46	109	18	14	7
4240	13358	9720	865	474	282	28100	1220	7	562	1	26	64	4	81	273.63	81	427.24	315	269	294	17	39	0	0	3
4526	13232	9594	798	476	258	28092	1189	4	635	0	6	61	2	74	275.22	99	432.14	333	305	317	19	44	0	0	2
10896	8030	5594	1	2150	1616	28660	491	74	550	11	342	173	42	291	323.27	102	466.14	467	152	383	110	91	4	30	36
4924	12704	9270	664	806	466	28198	1119	6	593	0	28	53	2	96	282.24	87	439.07	378	312	322	23	30	0	0	2
4500	13298	9642	833	404	216	28074	1220	11	622	2	14	55	5	78	276.92	99	432.48	318	314	303	14	35	0	0	1
13246	7704	4696	4	1358	1008	28154	582	220	837	80	126	206	102	212	285.02	177	448.36	475	300	448	54	100	16	12	2
11102	7862	5472	0	2166	1660	28666	498	94	511	24	354	176	46	265	317.16	112	460.69	498	160	376	121	87	4	48	25
4100	13658	9860	953	258	150	28032	1282	6	622	1	6	53	4	64	270.18	107	422.3	284	288	295	11	39	0	0	3
4020	13604	9888	972	346	190	28058	1306	6	578	1	10	58	4	63	269.67	82	422.39	279	288	276	13	38	0	0	3
10924	7804	5510	1	2294	1766	28746	518	70	530	13	404	153	35	303	324.01	94	467.62	482	174	354	108	81	2	36	30
14420	7602	4330	3	818	634	27882	641	334	1046	172	72	264	124	149	267.7	265	429.28	495	424	421	19	85	19	4	2
5318	12772	9168	620	534	304	28112	1111	7	745	1	16	60	2	97	280.12	103	435.97	347	389	351	23	40	0	0	1
13050	7754	4726	1	1402	1096	28214	610	199	806	61	172	203	105	205	286	170	450.05	491	299	411	57	110	12	12	4
6508	11880	8422	436	816	520	28192	987	13	761	5	42	73	5	138	283.34	114	441.68	393	378	374	21	47	0	4	2
13152	7970	4868	7	1182	828	28092	625	222	940	72	86	207	122	191	283.41	217	443.79	489	371	443	32	103	13	6	3
4722	13212	9492	814	398	238	28072	1228	11	674	2	8	62	6	80	275.9	117	430.73	302	323	304	14	47	0	2	4
12250	7800	5160	0	1716	1214	28374	554	153	695	31	210	162	89	237	301.2	140	455.2	478	243	447	73	91	13	20	15
4054	13546	9830	911	406	236	28080	1266	5	579	1	8	62	2	71	270.67	96	423.76	302	270	279	13	41	0	0	3
12558	7862	4954	7	1506	1170	28274	552	178	766	64	204	191	84	235	291.12	150	450.02	470	270	441	69	101	9	18	9
4082	13434	9806	901	492	274	28100	1255	5	546	0	12	71	3	73	274.01	87	428.93	313	270	268	16	46	0	0	1
4972	12972	9340	702	504	298	28106	1168	6	694	0	18	59	5	78	277.55	101	433.76	353	368	317	12	39	0	2	5
14150	7674	4444	2	922	674	27932	599	296	1000	136	66	245	120	181	276.47	240	441.8	478	374	456	27	88	13	2	0
4466	13294	9646	821	430	232	28080	1244	8	649	1	12	60	4	89	276.85	101	432.24	291	340	290	12	39	0	0	4
13584	7602	4544	2	1288	954	28092	572	249	841	86	114	217	122	211	279.99	173	446.84	454	298	476	49	103	17	6	6
5138	12812	9242	635	570	338	28132	1109	3	718	0	30	73	3	91	276.8	118	433.68	371	366	327	21	58	0	2	2
14378	7670	4360	6	802	608	27876	663	327	1070	154	54	263	133	150	272.19	264	434.36	477	442	420	21	99	23	4	0
4150	13410	9762	878	472	284	28104	1252	7	555	1	24	60	5	79	273.92	69	427.84	299	291	287	17	35	0	2	3
12286	7870	5062	0	1644	1252	28364	586	156	716	45	232	179	73	233	291.84	144	452.49	512	264	391	55	93	17	14	8
3986	13666	9912	957	306	168	28042	1302	10	588	1	4	55	5	58	271.03	88	423.59	285	290	276	13	36	0	0	3
12470	7902	5000	2	1524	1184	28314	586	164	794	52	206	193	83	236	294.02	154	452.98	465	288	416	68	108	10	26	11
12478	7938	5016	2	1472	1152	28292	552	151	791	44	214	194	73	242	294.27	175	449.75	490	255	432	60	114	10	22	7
4708	13138	9476	775	484	284	28104	1200	6	673	1	14	63	3	86	275.99	103	430.08	327	346	306	14	43	0	0	1
13674	7762	4676	2	1084	738	28010	633	256	970	110	72	213	111	170	276.78	231	441.74	483	382	453	34	79	15	4	1

SUPPLEMENTARY INFORMATION:Monte Carlo Atomistic Simulation and Machine Learning Analysis of Na-K Eutectic Alloy in Condensed Phases, D. Reitz and E. Blaisten-Barojas, George Mason University, Fairfax, VA 22030

4018	13568	9840	935	390	244	28080	1281	7	575	2	20	67	3	80	272.57	89	425.92	285	275	263	15	44	0	0	2
4548	13192	9570	793	498	278	28098	1200	4	626	0	12	64	3	88	275.19	93	429.92	320	320	308	14	45	0	0	2
11358	7974	5444	3	1954	1484	28558	500	97	621	17	292	182	47	270	312.03	119	456.67	486	191	397	102	101	11	48	20
5764	12244	8808	518	814	520	28210	1025	12	702	2	54	74	10	105	285.38	98	440.73	406	360	351	28	40	0	6	5
14512	7658	4360	3	754	526	27838	629	340	1087	157	28	255	144	139	269.06	275	432.43	497	438	452	17	91	15	0	1
5112	12794	9210	669	622	382	28148	1113	7	660	0	28	70	5	106	278.25	95	438.27	358	330	328	19	42	0	0	1
12364	7896	4980	2	1616	1262	28354	579	150	741	44	216	182	72	249	299.14	162	453.53	468	251	420	64	108	14	18	10
13554	7740	4666	3	1164	836	28070	633	231	918	80	100	201	117	176	282.93	217	447.05	480	357	457	36	92	18	10	6
12314	7918	5062	2	1606	1212	28334	597	163	743	42	200	181	87	238	292.73	153	452.43	470	277	410	66	90	8	20	11
4496	13220	9590	808	494	282	28096	1204	12	597	1	14	60	5	89	276.3	95	431.45	320	305	304	14	39	0	0	1
11050	7818	5450	1	2274	1718	28704	505	84	547	18	354	178	33	277	324.65	111	466.66	505	170	342	112	85	4	38	28
12224	7856	5140	3	1690	1220	28362	573	149	717	36	222	154	81	234	300.39	152	456.29	487	241	442	66	82	17	10	11
4010	13598	9872	943	368	210	28066	1270	8	573	2	8	74	6	74	269.86	94	422.58	293	261	271	13	49	0	0	3
4920	13010	9372	714	506	290	28112	1125	6	678	0	14	55	3	85	277.79	108	433.76	364	333	346	17	37	0	0	1
11794	7858	5188	1	1850	1470	28504	518	120	664	33	304	161	60	264	305.81	139	455.82	488	203	421	90	96	6	38	16
11398	7946	5546	3	1942	1372	28522	529	99	619	16	294	177	48	260	315.2	118	465.18	485	204	382	97	101	8	20	22
14584	7626	4328	3	712	530	27838	640	345	1090	171	56	269	130	148	269.88	295	432.3	493	442	435	10	89	18	2	1
11872	7874	5102	1	1830	1486	28496	553	124	623	28	294	158	69	276	298.74	121	456.98	471	205	410	75	83	6	36	20
4576	13222	9572	811	444	256	28092	1212	6	625	0	22	61	6	92	276.13	89	431.79	299	315	314	15	36	0	0	3
4994	12842	9304	627	622	356	28142	1109	5	696	0	24	63	2	105	278.95	103	433.5	368	366	331	12	38	0	0	3
5636	12512	8924	592	622	412	28152	1064	12	710	4	40	76	6	110	283.65	126	439.67	402	349	332	7	50	0	6	3
4946	12896	9322	689	594	352	28136	1160	8	650	1	26	68	7	94	278.74	90	436.91	342	338	316	15	45	0	0	4
4790	12892	9380	715	668	392	28150	1148	3	604	0	26	61	2	100	279.89	74	437.38	343	316	323	18	37	0	2	3
4420	13284	9658	857	452	252	28086	1230	7	598	0	20	53	3	74	274.75	98	429.02	306	290	308	20	37	0	0	2
4640	13052	9482	739	602	344	28136	1159	6	603	1	14	66	4	89	276.73	82	433.58	352	323	314	13	37	0	2	5
5314	12720	9108	635	590	374	28136	1138	7	691	1	28	61	2	110	279.27	96	436.38	334	372	330	17	37	0	2	2
4494	13256	9628	822	448	242	28082	1223	12	632	2	14	54	5	86	273.81	87	426.58	311	323	302	9	32	0	0	4
12734	7724	4794	1	1596	1248	28314	591	157	749	48	186	193	72	224	291.11	174	452.38	476	267	407	75	101	12	30	9
13698	7796	4538	4	1048	832	28020	630	261	947	122	102	241	103	177	276.28	227	440.59	490	372	425	30	103	16	6	2
5310	12698	9090	610	622	400	28154	1099	9	692	3	34	67	5	98	280.71	108	438.09	377	358	331	17	43	0	0	4
14228	7664	4394	3	886	672	27916	668	309	1036	160	70	254	114	148	270.17	254	431.6	500	431	405	17	82	15	2	1
11392	7886	5400	0	2036	1526	28584	559	104	606	14	300	164	52	278	312.83	123	460.94	469	197	366	97	91	12	42	19
7288	11154	7894	296	1118	746	28298	881	25	752	3	98	80	20	154	289.52	132	445.78	452	370	389	33	48	1	0	3
3986	13620	9896	955	350	196	28056	1279	10	557	2	8	60	6	71	270.85	94	424.51	290	258	279	12	39	0	0	2
6704	11520	8326	375	1032	600	28242	953	17	722	1	56	75	15	130	288.16	120	443.69	430	386	370	27	43	0	4	4
4796	13096	9432	706	488	286	28112	1158	6	659	0	12	56	3	81	277.44	83	433.66	351	353	329	18	36	0	2	1

SUPPLEMENTARY INFORMATION:Monte Carlo Atomistic Simulation and Machine Learning Analysis of Na-K Eutectic Alloy in Condensed Phases, D. Reitz and E. Blaisten-Barojas, George Mason University, Fairfax, VA 22030

12094	8020	5258	4	1590	1140	28314	587	137	801	31	196	187	75	238	296.31	151	452.68	475	291	404	60	112	10	16	10
14418	7628	4372	4	832	580	27864	623	329	1049	158	32	269	130	157	273.65	260	437.87	471	404	450	25	90	18	2	1
10954	7844	5566	0	2248	1688	28718	492	70	533	13	362	168	36	296	325.63	99	468.04	473	164	375	121	79	6	46	26
4448	13358	9652	853	388	216	28068	1235	6	634	1	6	61	4	71	272.73	97	426.67	303	310	306	17	39	0	0	3
4814	13132	9462	742	412	238	28078	1178	15	683	0	20	64	8	81	275.95	123	431.67	335	352	314	17	47	1	0	1
4068	13644	9872	928	292	164	28046	1252	4	585	0	6	58	3	63	271.47	102	423.65	311	272	297	12	39	0	0	1
11892	7926	5128	2	1824	1420	28460	548	129	681	27	228	169	72	273	302.99	129	456.49	460	242	419	86	92	6	40	13
7404	11196	7936	351	976	618	28204	920	46	783	10	68	92	23	126	286.9	115	442.76	436	389	368	36	53	2	6	4
11758	7828	5164	1	1942	1512	28526	549	126	604	31	286	181	68	243	303.7	133	459.97	494	200	376	100	85	6	36	15
12466	7808	4918	4	1638	1276	28344	547	169	711	36	222	172	99	241	289.92	156	450.36	515	245	446	48	97	14	16	10
4458	13384	9664	824	350	192	28054	1212	7	643	1	6	68	4	79	273.03	99	428.15	307	326	314	13	46	0	0	3
4324	13480	9738	881	326	178	28050	1240	7	647	1	4	58	5	64	273.57	103	427.12	306	301	309	16	48	0	0	2
12344	7884	5032	3	1584	1234	28328	562	182	729	55	224	188	83	235	293.26	160	454.2	503	263	401	63	83	7	26	13
10842	7984	5620	2	2156	1660	28700	516	79	557	12	410	162	49	300	319.1	107	464.87	462	169	386	114	82	4	26	20
12994	7812	4762	3	1378	1068	28176	583	197	818	73	142	214	81	211	281.29	180	446.06	497	290	420	54	114	18	20	1
13396	7978	4746	4	1030	774	28012	658	240	1013	111	84	221	103	173	274.96	213	438.61	474	438	437	27	98	10	4	3
13628	7766	4564	3	1116	868	28060	623	251	928	104	110	219	107	180	278.71	226	444.89	493	353	428	35	95	16	8	5
14048	7728	4430	6	938	730	27952	626	306	972	150	74	246	117	167	272.66	225	436.49	490	382	439	21	81	15	4	3
10916	7790	5532	1	2356	1754	28760	517	74	484	10	354	150	41	295	327.15	79	467.83	520	166	342	98	71	5	54	27
14526	7520	4362	3	814	564	27848	609	350	1027	178	58	278	121	162	271.87	249	436.26	480	389	439	18	88	27	4	4
4534	13302	9614	821	390	216	28066	1207	7	659	1	10	58	3	64	273.36	100	427.48	323	328	320	19	42	0	0	3
14478	7602	4320	4	774	612	27874	590	325	1073	165	84	274	126	144	272.12	297	437.09	533	411	434	16	97	15	4	2
3722	13744	10054	1001	348	178	28054	1312	7	532	1	8	59	3	68	270.57	87	424.05	268	250	268	15	36	0	0	3
4860	13050	9350	719	514	330	28122	1186	8	651	0	18	52	5	91	276.52	100	432.53	332	353	316	14	31	0	0	2
4414	13348	9678	845	396	218	28068	1214	8	639	2	14	66	5	80	275.79	102	430.22	304	302	311	18	47	0	0	2
12944	7802	4840	8	1418	1038	28192	610	212	778	78	130	209	101	201	281.64	171	447.38	493	279	421	50	93	16	20	5
6504	11834	8452	399	820	518	28196	952	14	798	3	62	73	6	129	286.94	117	444.71	418	409	384	28	45	1	6	1
5134	12776	9222	654	608	368	28144	1115	5	681	1	34	68	2	90	281.39	99	437.18	387	350	315	15	50	0	2	2
12536	7838	4900	1	1572	1240	28316	604	171	755	48	210	176	79	249	290.89	143	451.47	473	276	402	54	94	11	16	9
14232	7614	4420	7	904	666	27916	637	327	971	160	74	259	135	177	272.91	239	436.89	472	401	429	18	87	9	6	1
13732	7754	4608	6	1060	760	27998	644	269	976	114	84	228	108	171	277.9	232	443.96	487	385	425	25	92	19	0	4
4340	13436	9706	849	354	212	28064	1214	6	630	1	14	65	2	73	275.49	103	430.14	326	306	302	13	48	0	2	1
4000	13578	9854	921	382	242	28082	1267	5	574	1	26	65	3	67	272.23	105	426.65	299	267	276	21	41	0	0	1
11536	7884	5270	0	2000	1540	28558	518	95	591	22	290	180	44	268	316.18	128	461.8	476	186	384	102	94	9	36	28
5812	12192	8816	551	826	496	28194	1056	17	675	2	50	59	13	123	286.47	88	441.14	372	346	364	18	38	0	2	4
12230	7928	5086	0	1658	1240	28362	581	139	741	31	196	157	73	248	300.08	144	453.26	471	271	427	67	82	10	24	11

5800	12276	8810	536	774	482	28186	1007	10	690	4	40	70	3	101	283.72	124	439.96	438	331	347	23	48	0	4	1
14492	7568	4334	5	826	590	27860	645	357	1044	174	46	263	136	156	271.6	258	434.49	461	428	440	24	78	24	4	2
5766	12410	8856	601	656	420	28146	1097	25	692	6	34	79	11	106	283.43	101	440.38	374	357	317	18	53	3	2	0
4678	13228	9556	773	380	208	28066	1186	9	677	1	14	70	5	74	276.72	101	432.87	337	347	313	12	48	0	2	2
4690	13080	9482	772	536	306	28112	1176	5	633	1	18	57	2	88	274.15	100	429.9	329	312	325	18	42	0	0	2
12330	7828	5036	0	1694	1248	28356	602	134	712	44	210	191	58	228	298.46	140	455.42	487	267	378	69	92	10	8	15
11978	7832	5136	3	1856	1386	28448	560	122	671	32	230	179	61	248	301.86	157	454.98	505	226	384	74	91	10	28	16
11012	7854	5514	1	2242	1668	28666	537	80	572	9	330	163	39	294	312.85	100	465.04	471	199	351	115	90	5	42	19
4594	13234	9574	804	434	236	28080	1218	10	649	3	8	55	5	77	273.81	107	428.04	322	332	309	11	40	0	0	2
4508	13238	9612	830	464	252	28084	1241	7	624	0	8	66	4	86	275.63	93	429.5	303	330	280	12	43	0	0	2
4278	13480	9752	893	354	192	28058	1252	10	611	1	2	54	6	64	274.4	98	426.85	316	300	295	9	31	0	0	3
5858	12370	8810	538	646	422	28154	1057	11	747	3	46	80	7	92	282.08	115	439.16	408	395	336	22	56	0	2	1
14234	7666	4374	4	872	682	27902	625	303	1012	153	66	260	108	169	274.79	235	438.81	461	401	439	31	96	16	8	2
4244	13418	9746	878	422	238	28080	1252	4	605	1	8	65	3	66	272.39	101	425.15	308	297	287	17	45	0	4	3
5188	12790	9186	670	588	360	28138	1136	12	682	4	26	68	4	87	283.05	107	439.03	369	345	315	19	42	0	0	1
4098	13524	9816	931	396	230	28076	1281	5	569	1	12	67	4	74	272.67	86	426.49	295	284	268	11	36	0	0	2
4744	13082	9478	761	498	282	28108	1211	3	671	0	24	63	2	74	276.93	108	431.07	325	347	303	18	45	0	0	4
10600	7712	5672	1	2478	1854	28810	530	60	497	9	456	165	31	300	329.28	77	471.31	445	163	333	126	83	2	36	42
12246	7692	5040	0	1808	1360	28424	577	135	673	27	236	185	83	257	299.53	136	456.42	485	243	382	71	113	9	42	10
4898	12978	9386	743	536	298	28110	1189	10	681	1	14	62	8	90	281.8	104	436.12	333	346	305	15	46	0	0	0
14378	7664	4394	3	796	584	27876	630	338	1044	148	58	235	144	150	273.69	248	437.22	488	441	464	19	73	18	2	1
4494	13396	9654	864	320	178	28050	1228	10	651	1	8	62	8	63	273.57	117	428.87	319	305	306	17	42	0	0	0
4078	13498	9848	914	408	226	28080	1257	4	575	0	22	48	2	72	271.49	102	424.46	306	272	293	14	34	0	0	1
14224	7730	4418	7	838	634	27906	656	317	1034	147	62	246	133	153	272.18	256	435.47	469	426	441	27	81	17	0	1
14168	7582	4370	4	988	754	27952	612	323	934	153	88	254	137	170	274.51	213	439.01	488	381	434	32	90	16	2	0
12004	7852	5188	0	1784	1324	28422	555	147	683	37	248	196	85	247	303.39	149	453.67	503	244	389	73	111	9	16	13
13506	7966	4696	5	984	762	28012	673	251	976	105	94	216	106	170	277.5	245	440.24	477	388	427	24	81	22	4	3
4078	13522	9856	918	396	208	28068	1276	7	576	1	6	53	4	61	272.71	84	425.93	292	287	293	19	35	0	2	2
4354	13254	9640	845	524	314	28112	1239	4	575	0	26	58	2	87	277.31	72	433.24	297	305	288	16	39	0	0	6
11168	7930	5514	2	2068	1560	28620	493	96	579	11	336	182	50	272	317.16	100	462.96	478	175	371	130	92	10	40	17
14404	7568	4362	4	858	622	27882	639	323	1038	162	68	261	128	153	271.82	249	435.62	481	429	438	22	86	17	0	1
12030	7760	5128	2	1822	1396	28446	543	137	668	26	280	168	79	248	302.16	130	456.54	477	229	416	94	91	8	28	13
5994	12128	8716	485	822	500	28210	1026	9	736	0	46	52	5	115	288.27	113	444.94	410	386	362	20	37	0	4	3
3974	13658	9912	980	316	178	28046	1318	9	571	1	8	67	6	68	270.35	93	423.43	266	281	258	14	40	0	0	3
11692	7762	5196	1	2020	1540	28542	555	127	569	34	316	172	71	280	308.39	99	457.11	444	181	402	100	86	6	12	15
11346	8056	5428	0	1946	1474	28574	552	89	630	17	296	155	41	263	310.25	115	459.39	504	221	385	87	87	9	26	17

SUPPLEMENTARY INFORMATION:Monte Carlo Atomistic Simulation and Machine Learning Analysis of Na-K Eutectic Alloy in Condensed Phases, D. Reitz and E. Blaisten-Barojas, George Mason University, Fairfax, VA 22030

10726	7770	5644	0	2352	1804	28806	491	73	466	10	456	174	35	289	333.1	92	470.51	487	139	338	121	88	7	54	42
5296	12700	9146	625	606	362	28144	1108	5	715	2	34	71	3	101	279.7	116	437.57	382	366	324	10	46	0	0	0
4468	13354	9640	829	382	218	28072	1209	11	635	1	10	54	7	83	274.29	101	427.43	320	301	317	8	34	0	0	2
5128	12922	9290	689	476	264	28092	1156	10	717	1	12	67	4	89	279.42	102	433.85	343	370	321	14	52	0	0	2
10650	7860	5666	4	2318	1794	28786	537	55	500	10	454	151	26	305	318.92	83	467.47	472	169	339	116	77	4	38	27
3898	13746	9956	969	270	160	28040	1306	10	574	1	10	62	7	54	270.69	85	423.49	277	289	277	19	47	0	0	2
4748	13062	9458	727	536	300	28118	1158	5	639	0	14	72	4	83	278.67	101	434.18	357	340	306	16	47	0	0	3
6182	11918	8594	509	920	546	28204	1035	19	679	5	44	82	9	144	286.3	104	441.05	368	357	333	23	41	0	0	2
13886	7728	4568	3	970	718	27966	605	281	991	127	84	235	110	158	275.77	229	441.3	487	399	458	34	91	20	10	6
14586	7638	4310	2	718	536	27836	641	337	1113	168	48	270	125	152	269.41	287	431.21	481	451	434	14	93	18	0	1
4142	13562	9812	903	338	196	28058	1250	7	583	1	8	63	3	69	273.67	95	426.27	311	288	288	11	39	0	0	2
11652	7852	5322	2	1974	1408	28472	547	111	641	20	248	161	56	255	303.33	140	456.75	504	210	391	77	92	5	14	18
4726	13048	9446	749	568	326	28128	1175	8	639	1	14	54	5	86	278.38	98	434.31	344	317	320	16	37	0	0	1
4408	13300	9632	853	452	276	28092	1252	7	630	1	24	61	3	67	274.45	98	427.52	316	321	281	12	48	0	0	6
4360	13276	9646	814	520	298	28112	1222	5	602	1	10	62	4	66	277	113	430.75	336	312	289	16	44	0	2	4
13984	7692	4508	4	988	710	27950	597	309	973	145	62	241	122	164	275.44	252	441.08	514	355	436	24	81	16	6	4
13332	7756	4752	1	1240	892	28104	634	228	891	68	124	190	119	182	279.96	193	444.5	478	328	448	47	96	14	8	2
4292	13504	9750	882	320	180	28052	1240	7	623	1	6	52	5	80	273.37	90	427.44	296	303	314	9	35	0	0	2
14380	7604	4330	1	856	652	27892	632	320	1030	163	70	254	122	159	269.29	267	431.13	485	420	437	21	82	15	0	1
11756	7982	5276	1	1794	1368	28462	547	124	669	28	244	172	63	252	305.78	138	458.28	481	232	404	87	90	6	38	19
12198	7994	5080	2	1574	1248	28348	595	157	722	44	234	164	79	241	300.7	140	452.39	459	279	429	61	80	4	20	15
11310	7746	5418	1	2126	1626	28652	519	95	532	21	384	170	44	294	317.21	99	465.25	453	180	382	117	82	7	38	21
11764	7910	5306	1	1866	1348	28458	584	116	666	31	240	177	47	255	306.06	137	458.02	472	244	373	89	87	7	22	13
3896	13656	9940	968	350	202	28060	1274	7	558	1	16	65	3	69	272.25	97	424.12	284	251	282	19	40	0	0	2
11706	7722	5178	0	2030	1572	28560	547	109	601	23	316	176	55	271	303.56	122	458.93	499	200	366	90	98	8	28	17
13250	7698	4720	4	1342	990	28156	616	211	846	72	150	185	105	230	286.77	156	449.88	437	340	455	46	81	10	6	6
3972	13642	9900	962	336	198	28060	1301	4	563	1	8	55	1	61	270.21	88	422.5	287	281	276	15	35	0	2	1
4226	13526	9784	911	332	182	28054	1261	5	624	1	4	61	3	71	271.33	101	424.35	297	295	291	13	44	0	0	1
3920	13584	9924	975	408	224	28074	1315	4	558	0	10	61	2	73	271.27	83	423.92	274	275	258	14	50	0	4	1
6168	12150	8664	490	704	428	28152	1007	11	753	2	38	71	7	120	285.64	110	440.2	378	377	389	24	46	0	0	4
13712	7818	4576	8	1016	784	28004	618	264	971	128	94	245	112	180	276.3	233	441.18	496	377	423	27	100	10	4	2
3918	13662	9968	967	330	164	28050	1289	4	582	0	8	70	1	59	274.21	105	428.78	300	272	257	15	42	0	0	2
11126	7736	5534	0	2232	1642	28686	538	85	520	14	368	171	46	279	321.36	89	463.44	464	167	370	105	89	6	42	33
4788	13110	9458	770	456	262	28090	1186	9	651	0	14	51	5	92	277.67	103	432.57	320	328	331	12	33	0	2	1
4582	13180	9556	776	482	280	28106	1198	6	634	0	26	52	4	90	277.61	86	432.82	322	328	321	10	34	0	0	3
11938	7818	5176	1	1812	1404	28482	588	128	652	32	312	153	58	243	301.27	138	454.25	472	223	402	97	76	12	22	10

4796	13012	9370	718	580	362	28140	1148	3	624	0	18	64	3	86	277.22	106	433.69	356	318	322	20	37	0	2	2
11778	7966	5290	0	1794	1356	28482	536	105	684	21	264	176	60	232	303.14	147	456.32	522	228	406	84	99	7	32	17
12006	7800	5176	0	1850	1342	28426	592	129	701	33	224	168	63	242	296.33	149	453.23	473	254	402	81	83	18	26	12
14556	7670	4376	4	682	486	27810	650	358	1097	169	36	262	140	137	270.13	259	432.96	468	458	457	19	81	21	4	2
11928	7794	5244	1	1846	1352	28454	554	127	693	38	246	193	59	269	310.04	128	458.96	454	249	378	93	103	6	42	18
10498	7694	5756	0	2546	1870	28880	486	54	445	9	476	140	27	329	333.93	68	471.82	458	140	381	114	61	4	38	44
14534	7634	4290	3	762	590	27868	647	319	1065	158	52	270	121	139	272.11	276	434.31	492	433	421	23	97	17	6	2
12062	7928	5172	4	1692	1274	28386	567	149	706	35	240	183	82	253	298.17	156	453.95	490	244	399	61	104	5	16	14
14308	7590	4342	5	914	694	27924	658	320	974	157	68	241	120	172	271.82	222	436.9	454	403	438	24	75	16	8	2
5724	12478	8890	582	630	392	28138	1099	14	731	5	22	81	6	92	280.37	125	438.1	379	391	317	24	50	0	2	1
6754	11594	8242	402	934	624	28236	956	25	756	4	88	83	18	138	289.83	114	444.92	405	401	374	31	54	1	0	6
4946	13004	9364	694	486	284	28104	1164	6	676	0	20	72	6	70	278.02	109	434.13	356	361	312	18	51	0	0	5
11032	7894	5532	2	2202	1638	28680	525	73	568	23	340	200	33	253	321.56	114	465.62	495	189	336	126	86	3	40	29
11600	7898	5154	1	1952	1606	28576	585	110	604	30	324	155	57	248	304.58	141	457.05	499	205	377	82	74	6	42	22
4022	13588	9894	952	358	188	28058	1318	7	583	1	8	62	4	73	273.03	82	425.81	268	301	259	11	39	0	0	3
6966	11466	8162	415	956	598	28212	969	40	757	11	64	91	22	126	288.24	116	443.63	406	379	361	35	53	0	0	4
5594	12472	9008	601	654	372	28136	1064	17	713	0	36	67	13	107	281.2	119	436.73	386	356	351	15	46	0	0	1
3896	13604	9914	969	414	238	28078	1317	8	551	1	12	55	4	74	270.82	82	424.45	266	280	267	15	37	0	0	2
12514	7840	4914	2	1570	1244	28326	584	163	755	44	228	208	86	241	291.15	148	451.21	468	266	399	62	114	13	16	14
6460	11790	8404	416	936	588	28224	957	23	724	7	44	71	12	128	285.84	137	443.49	446	345	369	18	43	0	0	2
14416	7638	4360	4	808	594	27868	649	333	1056	154	48	248	139	145	268.98	261	431.1	473	444	459	17	78	21	2	4
12562	7732	4918	3	1622	1252	28346	552	174	704	53	248	221	84	260	299.92	140	453.04	461	226	394	73	127	10	12	13
12834	7854	4856	1	1422	1084	28234	600	185	820	69	176	172	85	206	283.6	168	446.67	505	310	458	45	76	13	8	4
4448	13330	9662	819	396	220	28074	1227	7	651	0	18	49	3	72	276	91	430.18	302	343	321	19	30	0	0	3
5222	12852	9192	687	500	314	28104	1127	17	670	2	24	62	9	105	277.89	98	435.59	327	331	344	21	41	0	0	2
11190	7814	5420	0	2128	1672	28660	544	91	559	17	394	158	39	278	315.33	100	460.33	467	190	369	109	80	6	38	30
13930	7704	4542	4	992	714	27958	621	303	980	125	66	225	131	179	276.88	229	439.55	470	381	463	27	88	20	8	1
11722	7830	5256	1	1862	1474	28518	504	113	639	21	334	176	56	264	310.56	133	457.27	475	208	400	106	91	2	36	20
11836	7914	5210	2	1778	1404	28472	523	115	647	31	300	175	56	263	302.23	131	453.22	515	213	411	75	90	6	30	10
13652	7520	4458	1	1316	1018	28114	602	259	841	101	144	237	108	217	282.41	188	446.2	451	323	417	51	100	21	6	4
4894	12976	9354	715	548	330	28124	1137	1	653	0	22	65	1	90	278.43	98	433.26	345	320	339	21	45	0	0	2
14478	7638	4354	6	774	562	27854	670	320	1082	165	46	257	112	152	271.3	253	434.28	459	472	438	17	87	25	2	2
4602	13244	9568	797	418	236	28082	1190	7	647	2	14	60	4	69	275.54	106	430.91	338	317	322	15	36	0	0	3
12964	7896	4848	7	1320	984	28152	615	198	890	67	128	192	102	205	281.8	177	444.98	470	350	451	48	94	8	12	3
12424	8002	4954	0	1522	1206	28312	620	154	801	48	184	176	83	229	285.08	165	447.73	488	310	414	56	97	8	20	1
8472	10728	7332	243	932	656	28200	846	68	838	21	70	96	35	146	288.75	158	444.77	444	404	418	35	50	2	8	1

SUPPLEMENTARY INFORMATION:Monte Carlo Atomistic Simulation and Machine Learning Analysis of Na-K Eutectic Alloy in Condensed Phases, D. Reitz and E. Blaisten-Barojas, George Mason University, Fairfax, VA 22030

13822	7666	4494	5	1066	840	28014	625	271	963	125	114	238	110	193	275.19	224	440.17	464	368	430	32	93	15	12	4
7544	11054	7822	307	1064	678	28238	882	38	806	7	72	99	25	147	289.82	135	446.66	444	381	381	33	65	0	2	2
4020	13658	9906	965	290	158	28040	1284	7	591	1	8	64	4	70	272.16	95	426.38	285	277	282	7	46	0	0	4
13878	7676	4484	5	1026	814	28006	609	290	927	113	122	214	134	181	276.1	224	443.45	476	356	469	34	84	13	6	1
4210	13480	9750	887	392	234	28076	1240	4	597	0	10	56	3	80	273.12	96	427.25	306	279	301	10	45	0	0	2
14490	7670	4288	4	744	606	27866	641	332	1090	167	64	253	135	143	267.15	282	430.1	503	441	439	12	80	12	4	2
11178	7940	5514	2	2064	1558	28642	497	79	579	17	352	169	37	281	320.47	97	466.39	494	173	378	92	83	4	32	40
12766	8092	5018	5	1240	902	28148	607	172	918	57	122	205	81	180	285.93	216	448.87	511	334	407	52	111	10	8	8
11974	7932	5148	0	1734	1356	28434	566	120	681	24	266	179	54	239	304.71	128	454.38	479	237	391	84	106	9	24	22
5566	12524	8954	605	634	422	28152	1124	14	699	5	50	65	6	69	281.25	109	438.17	387	393	312	33	42	0	2	0
13798	7854	4574	8	948	718	27966	637	290	993	132	70	242	122	150	276.63	255	442.34	502	389	423	35	93	16	4	0
4440	13286	9628	816	452	266	28092	1186	8	604	0	18	50	3	67	274.78	104	429	338	288	326	17	36	0	2	5
13838	7896	4602	6	880	658	27940	661	282	1031	122	62	235	117	188	275.56	236	439.29	441	425	434	24	97	16	4	1
12494	7864	5048	2	1536	1134	28288	556	172	785	46	180	192	93	245	295.67	151	452.46	473	276	443	57	102	9	32	12
5174	12752	9186	615	632	382	28154	1108	4	719	0	24	58	2	105	277.52	104	434.85	370	376	334	13	44	0	4	3
5092	12778	9252	674	616	364	28138	1135	8	675	2	36	54	5	109	278.66	97	435.07	337	355	342	16	37	0	0	1
5114	12790	9250	681	614	348	28138	1120	10	672	2	22	55	7	89	277.09	102	434.95	367	339	347	17	37	0	0	2
4148	13572	9812	916	322	190	28054	1284	9	601	1	10	72	7	64	272.29	87	427.42	286	305	269	19	48	0	0	2
4276	13340	9706	809	488	280	28110	1195	2	570	0	20	55	1	91	278.51	87	432.67	330	288	314	10	31	0	0	1
3920	13710	9954	955	294	158	28040	1268	8	564	1	4	67	4	69	271.4	99	424.08	291	256	281	14	41	0	0	2
6402	11908	8520	465	814	484	28168	998	26	745	6	40	72	10	119	285.96	113	442.81	403	376	366	23	41	0	0	4
11366	7974	5532	5	1958	1392	28534	558	96	619	15	290	172	51	255	315.68	124	461.77	477	210	372	99	100	7	20	21
4318	13430	9744	887	362	192	28056	1254	10	638	1	10	67	7	68	273.66	105	428.63	297	301	288	17	53	0	0	3
5178	12754	9146	633	612	418	28162	1098	9	680	1	52	69	5	102	283.3	110	437.15	378	369	325	20	48	0	0	1
11590	8038	5348	1	1806	1400	28502	537	102	662	24	280	152	53	268	305.14	139	456.5	489	223	430	81	81	6	40	13
12428	7930	5052	5	1546	1146	28302	586	139	806	39	176	183	68	228	294.16	165	452.03	502	291	416	52	104	7	24	10
4180	13514	9818	914	340	184	28050	1262	9	610	2	14	50	3	70	273.41	83	427.56	282	291	310	17	37	0	0	2
5716	12338	8854	529	758	480	28198	1058	9	721	1	50	67	7	110	284.44	108	440.03	407	381	331	16	46	0	2	3
12404	7970	4992	1	1530	1196	28306	576	165	767	48	200	191	80	239	293.21	161	450.25	495	283	401	57	100	10	10	10
3814	13678	9974	981	382	212	28068	1301	5	530	1	8	62	3	67	270.6	92	423.33	295	244	257	11	37	0	0	0
13182	7682	4730	1	1364	1034	28174	555	223	847	67	168	199	114	220	283.94	201	445.85	502	285	461	39	108	20	12	7
5720	12462	8936	537	630	366	28144	1065	9	759	1	30	62	3	105	282.86	109	438.11	392	417	352	13	34	0	0	2
4756	13018	9420	710	580	340	28132	1151	3	635	0	18	57	2	96	278.44	83	433.93	337	341	335	17	32	0	0	2
4036	13576	9870	927	362	206	28066	1275	6	580	0	16	62	4	86	272.26	86	425.29	262	283	293	18	41	0	0	1
4254	13448	9742	884	380	228	28072	1265	5	618	2	18	64	3	69	272.35	96	425.7	293	313	281	20	44	0	2	2
13898	7764	4484	5	964	770	27984	627	287	955	144	100	232	112	181	273.28	238	439.6	476	384	452	24	73	11	4	2

SUPPLEMENTARY INFORMATION:Monte Carlo Atomistic Simulation and Machine Learning Analysis of Na-K Eutectic Alloy in Condensed Phases, D. Reitz and E. Blaisten-Barojas, George Mason University, Fairfax, VA 22030

4436	13270	9614	794	482	286	28108	1212	11	593	0	20	60	10	78	276.75	93	431.92	323	313	303	17	39	0	0	2
13916	7754	4518	3	954	730	27962	639	283	1018	134	82	238	115	167	274.69	232	439.1	468	417	445	34	87	11	8	1
14196	7700	4430	7	880	646	27908	664	327	1015	162	54	253	121	144	270.97	253	434.21	494	435	417	21	75	20	2	1
14444	7636	4374	6	784	568	27860	631	325	1094	154	50	259	125	167	272.73	270	438.72	473	428	447	12	94	28	4	1
4598	13026	9496	774	642	364	28144	1177	4	590	0	16	58	4	78	277.88	98	432.75	359	297	306	16	33	0	0	3
5768	12322	8878	549	722	426	28154	1066	16	706	4	34	82	9	97	283.1	102	439.61	399	363	325	19	53	0	4	3
4990	12984	9370	703	482	256	28090	1146	9	698	1	8	67	6	92	277.25	103	430.93	329	338	340	18	51	0	0	3
14318	7704	4414	10	800	584	27868	645	326	1074	162	46	268	127	159	270.68	256	433.03	479	430	432	14	98	20	2	1
10914	7966	5530	0	2196	1692	28700	480	65	551	14	354	173	26	291	324.76	106	467.18	500	157	366	114	83	3	46	22
5648	12472	8906	628	676	424	28154	1128	18	685	2	28	69	13	90	281.86	111	436.67	385	362	306	13	49	0	0	2
14232	7612	4358	5	952	716	27938	609	320	963	160	60	259	120	153	273.38	255	437.75	509	364	432	30	77	19	8	1
5180	12880	9242	628	488	292	28108	1122	8	710	0	26	70	6	82	279.98	99	438.48	373	395	327	17	45	0	0	2
4666	13152	9490	757	474	300	28118	1161	5	641	0	36	55	3	87	276.2	87	432.44	347	314	329	13	40	0	0	3
13682	7538	4450	2	1262	1012	28114	617	259	833	114	154	221	108	206	279.89	185	446.37	467	319	432	42	87	14	16	4
5280	12748	9170	648	572	330	28118	1124	5	736	0	16	70	3	89	280.69	128	436.42	353	375	331	25	49	0	2	1
5218	12724	9178	633	626	368	28140	1089	19	664	3	26	63	10	89	279.51	115	436.39	394	346	335	19	38	0	0	1
5116	12866	9282	686	532	296	28110	1117	8	698	0	18	63	5	89	279.55	100	434.93	352	341	351	20	45	0	0	3
6674	11738	8380	423	834	504	28184	999	22	784	6	50	76	12	125	286.35	97	443.54	390	412	369	29	48	0	4	2
4596	13120	9544	770	526	300	28114	1167	7	642	0	26	62	4	93	275.62	101	431.68	338	317	316	15	42	0	2	2
11834	7998	5310	3	1764	1262	28394	572	138	710	38	202	165	73	239	300.52	143	453.81	477	251	434	66	87	9	24	19
3684	13820	10064	1027	298	170	28048	1320	5	545	1	12	59	3	62	270.2	95	423.3	271	246	268	13	36	0	0	3
10868	7906	5512	2	2206	1770	28740	510	68	523	15	440	159	35	275	317.87	108	462.89	529	151	349	103	86	6	36	30
4764	12936	9384	726	652	392	28156	1154	6	623	0	28	69	4	94	279.22	108	435.28	355	309	304	20	41	0	0	1
11302	8052	5566	1	1954	1380	28550	536	97	641	15	270	156	48	280	313.71	126	462.49	492	207	385	84	92	7	24	19
4618	13164	9556	812	474	260	28090	1219	10	637	1	18	58	4	74	275.07	87	430.25	330	328	295	16	42	0	0	0
13788	7758	4520	6	1018	810	28010	631	276	939	108	108	222	131	174	276.45	223	442.37	480	373	448	29	91	15	8	5
4462	13254	9624	802	470	268	28100	1188	3	601	0	22	54	2	85	277.09	88	431.3	327	299	329	14	36	0	0	1
7070	11492	8116	391	888	574	28204	968	41	785	7	56	102	26	119	288.18	129	445.18	436	411	335	28	70	2	8	2
14352	7742	4398	6	764	568	27866	633	323	1089	163	38	259	114	142	269.21	287	431.95	505	431	436	13	88	24	4	4
4554	13270	9594	794	406	234	28076	1157	5	652	1	16	61	3	66	273.12	119	426.77	358	292	335	14	36	0	2	4
13328	7678	4632	1	1320	1026	28150	576	216	881	79	148	214	101	209	282.25	208	446.38	500	317	433	44	105	16	18	5
13136	7874	4756	3	1266	970	28142	599	219	860	66	128	183	114	209	282.34	192	445.74	499	333	451	33	90	9	12	3
4742	13214	9488	779	380	232	28072	1209	9	681	2	14	68	6	76	275.29	92	429.25	323	360	306	13	50	0	2	1
13940	7726	4564	5	962	684	27954	629	291	1022	129	76	243	125	164	275.81	233	439.41	498	409	427	22	100	13	2	4
4450	13306	9634	837	416	250	28082	1219	11	622	1	26	64	7	77	272.19	97	427.17	306	314	308	18	46	0	0	4
12440	7810	4992	1	1632	1228	28330	551	155	736	48	210	177	85	259	292.31	156	452.22	504	249	436	46	88	9	16	5

6510	11742	8414	442	916	570	28216	1012	23	732	7	60	92	15	143	287.81	89	443.96	368	394	347	31	55	0	4	3
5016	12906	9306	711	528	324	28116	1176	11	694	2	36	61	3	70	280.64	110	436.92	354	366	310	19	34	1	0	4
13534	7784	4642	9	1122	842	28034	667	253	959	128	108	240	92	179	276.14	210	440.65	486	409	389	27	98	18	2	2
14358	7694	4452	6	754	536	27852	657	328	1099	159	58	258	128	138	269.77	278	431.66	480	447	441	16	91	21	0	5
4444	13298	9608	829	456	280	28108	1253	4	644	1	22	58	2	78	276.73	100	431.52	312	332	280	12	41	0	0	3
4870	12954	9366	709	584	338	28128	1172	9	663	0	16	65	5	80	277.99	104	433.88	359	341	295	18	47	0	0	2
11608	7844	5330	1	1996	1456	28534	551	110	594	27	264	176	53	262	304.68	115	461.47	480	194	389	96	90	9	32	18
12836	7768	4798	1	1474	1172	28276	573	194	764	68	210	209	85	216	292.96	169	450.32	512	265	412	59	106	16	18	6
4974	12912	9346	710	558	310	28118	1159	7	671	0	16	64	3	88	278.18	96	435.07	341	342	322	17	48	0	2	4
4050	13436	9828	874	504	274	28104	1261	3	558	0	12	67	2	73	273.89	78	428.78	305	298	272	15	42	0	0	5
14212	7732	4438	6	824	620	27888	661	304	1050	157	62	259	113	136	271.04	265	434.14	500	433	413	21	86	16	0	2
4414	13230	9622	828	504	304	28106	1224	6	614	0	30	58	2	88	274.7	89	430.29	311	308	296	11	41	0	2	5
5968	12292	8784	596	626	398	28120	1080	34	713	10	52	75	13	102	281.27	122	438.21	381	359	332	16	43	1	0	2
5094	12852	9276	643	562	320	28126	1089	3	703	0	22	54	2	98	278.19	109	435.66	367	353	369	17	36	0	0	1
4954	12964	9342	690	536	310	28122	1143	6	696	0	16	65	5	90	278.36	105	434.24	347	346	328	20	50	0	0	2
4088	13598	9858	937	318	178	28050	1288	4	608	0	10	62	2	74	271.82	88	424.53	278	295	278	12	44	0	0	2
13924	7672	4522	7	1044	742	27974	651	277	964	143	60	240	104	187	275.23	209	438.69	441	399	446	27	79	24	10	4
11364	7868	5420	0	2042	1544	28614	542	99	586	16	336	161	56	270	317.99	119	465.2	465	184	393	102	88	7	38	25
4648	13150	9520	777	492	276	28098	1195	7	628	0	12	79	5	84	277	98	431.38	334	320	289	11	57	0	0	5
10894	7808	5532	1	2284	1768	28748	495	77	498	10	412	169	35	298	322.33	78	468.43	482	159	357	120	84	6	46	26
5260	12822	9214	654	510	282	28100	1121	14	719	3	12	67	10	95	278.76	108	436.77	355	373	344	11	39	0	0	3
3966	13606	9914	949	362	200	28064	1301	8	559	1	16	63	5	71	272.87	82	426.65	288	287	262	9	43	0	0	2
5582	12562	8988	602	608	374	28148	1092	5	733	1	30	64	4	89	282.65	112	438.93	395	371	341	17	45	0	4	0
4440	13326	9656	838	412	228	28074	1218	5	639	0	12	66	4	70	272.85	106	428.02	329	308	295	13	45	0	0	3
13828	7714	4530	4	1044	784	27990	593	277	979	120	74	239	125	192	275.96	234	441.11	479	363	460	27	98	13	16	2
13006	8006	4846	4	1204	926	28122	602	209	916	73	130	199	101	193	283.26	211	446.58	489	343	449	45	97	13	4	2
14294	7636	4376	3	862	654	27894	630	328	1025	165	70	264	125	167	270.91	248	434.52	467	426	431	27	87	18	2	2
4360	13346	9646	855	428	282	28094	1215	10	594	0	26	59	4	86	274.94	98	430.22	313	282	304	12	39	0	6	4
4800	13036	9440	729	526	294	28114	1159	10	650	0	18	54	6	76	279.35	109	435.62	353	337	326	22	28	1	0	1
13742	7844	4656	8	944	690	27964	620	276	1023	124	84	227	121	166	275.98	247	441.16	486	412	458	29	82	8	4	2
4584	13204	9552	796	456	272	28092	1180	5	641	1	24	65	4	82	273.26	96	427.47	333	305	321	16	43	0	0	1
11228	7938	5492	1	2054	1538	28620	553	74	611	14	346	152	40	274	314.48	121	464.72	472	214	387	94	86	3	18	23
4664	13098	9488	755	532	314	28120	1182	12	625	0	22	62	10	90	276.93	88	433.89	334	331	310	13	37	0	2	4
4778	13020	9424	718	564	326	28132	1164	7	629	0	20	51	4	93	276.85	98	433.34	340	327	327	16	34	0	0	2
12536	7884	4856	2	1514	1274	28332	579	175	712	52	248	195	83	233	298.58	166	451.1	480	245	393	71	90	14	20	15
4038	13684	9898	944	246	150	28034	1286	8	609	1	16	56	2	62	273.18	96	426.03	289	293	286	9	40	0	2	4

SUPPLEMENTARY INFORMATION:Monte Carlo Atomistic Simulation and Machine Learning Analysis of Na-K Eutectic Alloy in Condensed Phases, D. Reitz and E. Blaisten-Barojas, George Mason University, Fairfax, VA 22030

14672	7422	4190	4	886	654	27886	647	340	1012	167	58	262	132	152	271.71	260	436.12	478	413	427	24	86	23	4	2
13112	7838	4758	2	1296	992	28146	580	211	873	82	142	199	95	202	282.3	199	445.07	519	316	441	34	92	12	8	4
5814	12332	8824	603	704	438	28146	1079	26	699	6	30	79	15	107	281.75	106	439.79	371	349	325	27	49	1	4	0
11684	7842	5176	0	1992	1542	28554	556	116	600	33	288	169	62	273	302.01	111	458.99	471	207	394	90	90	7	26	19
4320	13428	9722	868	368	212	28066	1245	11	620	0	16	62	9	68	273.5	96	427.77	310	313	292	14	39	0	0	3
5684	12396	8854	536	750	474	28190	1066	9	702	0	32	62	7	105	285.11	121	439.95	394	368	345	22	38	0	0	1
12112	7822	5044	1	1796	1382	28422	515	152	670	43	248	176	78	261	297.87	163	451.86	502	213	429	77	87	9	18	10
4008	13580	9854	939	386	234	28078	1291	4	579	1	16	61	1	75	272.83	82	426.43	281	280	273	13	43	0	0	3
5114	12750	9248	663	644	358	28134	1122	10	675	2	20	58	5	95	279.9	100	436.92	363	344	337	17	36	0	0	1
6110	12040	8684	511	820	478	28178	1029	21	727	6	46	84	12	121	286.97	114	443.36	390	367	346	25	56	0	0	0
14382	7588	4298	3	870	690	27912	639	320	1018	159	74	271	120	147	272.35	250	436.74	486	423	418	29	100	26	10	4
5364	12644	9120	624	622	360	28140	1090	9	713	2	28	65	4	90	280.7	121	437.27	406	358	327	12	48	0	2	1
3826	13734	9982	991	328	182	28056	1298	7	552	0	4	63	5	54	270.29	98	422.93	289	250	270	21	41	0	0	1
4098	13548	9838	922	366	204	28064	1282	4	598	0	10	63	3	72	275.42	83	429.72	287	289	275	14	48	0	0	2
14368	7790	4370	4	722	564	27852	670	322	1083	167	36	259	119	132	269.82	291	432.61	495	443	423	15	82	15	2	1
13992	7632	4486	5	1008	746	27958	637	300	972	143	88	252	111	158	274.69	235	439.18	483	385	424	34	87	19	6	3
13634	7938	4716	6	954	666	27962	602	263	1023	125	52	240	98	168	277.06	241	444.27	485	384	454	36	104	17	2	2
13870	7702	4532	5	1024	770	28004	617	275	957	120	98	217	110	173	277.58	203	444.13	491	383	457	31	82	18	8	1
13128	7848	4734	1	1270	1012	28160	627	185	907	68	142	195	92	211	282.38	191	445.64	458	340	443	46	109	10	26	5
5060	12840	9258	684	596	352	28124	1140	9	678	1	18	65	4	104	279.64	105	436.02	357	343	315	8	44	0	0	2
5060	12922	9310	691	518	286	28106	1157	10	692	1	10	59	6	80	277.99	113	434.54	355	364	321	19	41	0	0	0
4102	13480	9814	915	438	240	28080	1277	6	574	1	6	62	4	65	271.02	96	424.07	312	280	262	15	39	0	0	0
14332	7694	4350	6	810	636	27886	650	311	1058	161	62	253	116	138	267.95	294	429.04	502	427	432	18	77	14	2	2
4030	13496	9796	899	460	300	28112	1277	2	548	0	28	49	2	85	274.38	83	428.37	287	288	284	14	32	0	2	1
4374	13384	9680	861	394	228	28072	1254	11	632	1	12	58	7	69	272.81	91	428.48	304	331	291	17	39	0	0	1
14404	7566	4364	3	852	624	27884	634	332	1047	145	70	262	150	147	272.38	247	436.05	471	434	442	35	103	17	4	1
12514	7876	5000	2	1518	1160	28300	585	164	781	40	220	185	91	236	295.07	150	452.25	452	288	440	75	104	11	12	6
5192	12726	9176	637	642	386	28152	1126	4	685	0	30	60	3	88	284.16	100	437.92	383	377	320	17	39	0	0	0
11092	8004	5430	0	2124	1650	28656	504	94	584	19	298	157	55	277	313.36	111	458.86	512	185	397	89	74	7	50	28
12526	7746	4918	4	1648	1270	28358	567	157	715	48	226	173	75	224	294.58	159	454.03	491	252	434	67	87	15	24	17
4374	13362	9690	842	394	230	28074	1230	6	616	0	22	62	4	68	273.97	88	428.83	324	309	295	16	47	0	2	0
4568	13220	9558	807	468	268	28092	1217	9	629	0	10	63	5	90	276.34	87	431.44	307	320	301	14	48	0	0	2
11678	7876	5268	2	1944	1448	28510	598	106	660	20	276	172	58	272	305.6	122	456.98	444	238	386	77	89	8	18	23
4364	13418	9678	843	376	228	28078	1216	6	614	1	14	64	4	75	274.94	95	429.73	312	300	312	14	43	0	0	5
4494	13320	9590	802	424	256	28092	1208	2	635	0	6	59	2	71	276.82	92	430.52	325	335	311	22	38	0	2	0
4354	13196	9612	814	596	354	28134	1224	5	583	0	22	60	3	87	274.74	81	429.12	317	293	288	17	40	0	0	3

SUPPLEMENTARY INFORMATION:Monte Carlo Atomistic Simulation and Machine Learning Analysis of Na-K Eutectic Alloy in Condensed Phases, D. Reitz and E. Blaisten-Barojas, George Mason University, Fairfax, VA 22030

4764	13124	9452	733	468	280	28108	1182	6	672	0	20	50	6	83	278.18	95	433.53	332	363	327	18	35	0	0	1
13460	7580	4520	4	1344	1080	28164	585	247	814	83	146	210	121	210	281.4	174	445.33	481	300	447	51	99	23	32	4
4716	13150	9496	751	434	264	28092	1175	4	676	0	30	60	4	72	276.68	96	431.24	341	348	322	21	43	0	2	2
14448	7644	4366	10	756	572	27852	648	337	1076	165	58	261	136	157	267.8	264	429.28	465	446	448	13	81	20	8	4
4180	13606	9832	924	260	144	28030	1308	8	638	2	8	62	3	56	271.46	98	424.05	282	347	272	13	42	0	0	2
4680	13202	9506	756	430	256	28088	1161	8	654	1	12	56	4	72	274.33	105	429.43	351	325	331	16	40	1	2	4
12056	7842	5184	1	1786	1298	28424	539	131	676	27	242	176	71	254	301.21	148	455.42	503	207	409	79	108	10	16	6
12740	7838	4882	4	1466	1126	28248	628	175	788	49	178	178	95	210	286.51	149	450.08	449	302	440	66	89	10	14	8
14098	7624	4470	4	972	694	27930	637	305	996	156	68	251	114	168	273.22	244	434.92	490	398	424	20	85	14	4	0
3944	13600	9890	951	398	232	28074	1298	6	556	1	8	50	3	70	270.88	87	423.61	276	272	283	16	32	0	2	2
4234	13442	9726	884	412	254	28086	1251	3	582	0	18	57	2	67	274.06	83	428.77	310	293	292	16	36	0	0	3
3920	13672	9932	973	336	188	28054	1312	9	586	0	6	56	5	69	271.01	89	424.64	272	282	268	12	41	0	0	3
7342	11232	7924	337	1004	662	28248	913	29	759	8	80	89	11	144	291.42	124	446.01	420	379	374	35	51	1	4	9
11890	7938	5150	0	1780	1410	28468	571	117	655	25	260	170	59	258	299.94	123	458.53	463	225	416	85	97	7	40	10
5156	12816	9236	658	566	326	28120	1129	9	694	1	20	63	7	99	279.47	97	436.58	345	370	340	18	42	0	0	1
12418	7972	5078	2	1524	1114	28294	561	144	805	34	172	181	82	232	292.33	174	452.06	504	293	434	49	104	12	16	11
4026	13650	9894	937	294	168	28044	1289	7	612	1	12	55	3	60	271.49	115	424.35	294	297	277	13	35	0	0	2
11510	7804	5372	2	2104	1484	28558	525	104	571	22	250	178	57	289	317.08	107	460.27	463	179	391	93	84	9	34	20
5144	12796	9210	654	596	358	28124	1120	9	689	2	18	78	5	103	279.03	82	437.53	345	363	327	18	54	0	2	3
4474	13222	9586	804	512	298	28106	1236	7	617	1	14	59	5	77	275.65	77	430.86	316	335	286	14	37	0	0	5
4046	13634	9904	948	296	150	28034	1292	7	618	2	4	68	4	63	270.3	107	422.55	286	298	270	14	47	0	0	1
4270	13452	9746	860	372	216	28074	1245	5	628	0	16	68	4	78	273.03	100	429.16	303	308	286	15	48	0	2	1
4762	13008	9382	759	588	374	28144	1180	3	616	1	30	63	1	88	278.96	97	436.47	343	309	302	17	43	0	0	1
7624	11274	7856	341	828	528	28156	923	42	849	14	46	101	19	125	288.64	142	443.83	420	413	386	30	65	0	0	3
5174	12792	9238	652	554	322	28110	1115	13	677	4	28	71	7	91	279.99	102	435.78	360	374	329	19	39	0	2	4
14158	7754	4464	8	842	620	27888	653	313	1054	154	46	263	123	158	269.1	271	432.25	481	422	422	18	100	17	4	0
4392	13290	9636	819	492	284	28106	1232	5	602	0	12	67	4	84	277.6	77	432.62	316	323	281	15	45	0	0	1
4970	13076	9404	717	406	212	28072	1169	8	716	0	4	56	3	78	277.56	104	432.92	336	379	338	13	37	0	0	3
14202	7670	4338	9	918	732	27936	654	320	996	150	72	248	128	161	271.91	245	436.65	477	412	429	26	87	22	4	0
5022	12786	9288	679	668	366	28144	1156	13	624	3	14	66	7	97	280.09	89	435.68	350	336	308	17	46	0	0	1
4498	13174	9574	799	546	312	28122	1228	4	589	0	18	60	2	86	276.28	71	431.5	311	314	293	15	43	0	0	4
14362	7616	4350	5	844	646	27894	599	336	999	157	72	255	143	161	273.01	269	437	481	381	469	28	85	18	4	1
11082	7896	5588	0	2140	1562	28650	540	76	568	19	342	170	33	292	317.85	100	464.45	475	187	363	100	96	7	40	23
3968	13612	9890	947	364	216	28066	1280	6	577	1	16	58	4	70	272.1	93	424.79	291	268	281	13	39	0	0	2
13826	7858	4558	4	924	718	27966	648	281	1006	110	74	218	134	159	274.9	230	438.7	482	414	454	29	92	16	8	0
5838	12264	8752	521	780	516	28202	1037	13	699	1	48	71	8	120	283.87	111	441.23	403	359	340	19	43	0	2	2

SUPPLEMENTARY INFORMATION:Monte Carlo Atomistic Simulation and Machine Learning Analysis of Na-K Eutectic Alloy in Condensed Phases, D. Reitz and E. Blaisten-Barojas, George Mason University, Fairfax, VA 22030

4630	13220	9528	767	450	256	28086	1171	11	638	0	2	46	6	82	274.77	96	429.37	335	326	342	12	30	0	0	3
4236	13458	9766	887	384	216	28076	1241	9	593	1	16	63	7	66	273.09	94	428.58	315	285	295	16	48	0	0	2
11390	7838	5310	1	2106	1618	28620	541	88	585	27	320	173	44	277	312.77	119	457.33	494	197	375	87	96	6	34	24
12500	7822	4956	2	1590	1222	28322	546	150	761	47	210	198	73	234	297.29	152	452.23	484	269	415	72	102	7	20	14
12320	7942	5062	4	1614	1194	28334	602	144	745	38	194	181	78	227	292.52	155	451.75	476	271	417	72	106	12	8	6
4396	13272	9640	823	496	284	28104	1206	7	602	0	16	63	4	87	277.65	108	431.5	317	292	303	19	32	0	0	0
3866	13656	9948	967	376	210	28064	1302	8	553	1	8	67	6	81	269.95	82	423.36	271	272	261	11	42	0	0	2
5164	12724	9186	640	674	394	28164	1109	2	678	0	22	62	2	100	279.48	96	435.35	385	334	328	12	46	0	0	1
5370	12694	9112	609	584	350	28140	1113	8	731	0	30	58	6	90	281.5	111	437.7	380	402	335	16	38	0	0	1
4752	12964	9382	700	628	398	28164	1151	3	623	0	38	57	2	83	278.45	104	434.26	366	322	314	21	42	0	2	3
11970	7786	5078	0	1920	1452	28480	562	138	651	38	262	180	64	244	305.37	138	454.98	507	235	377	74	91	10	10	24
4480	13346	9646	838	366	212	28070	1250	8	660	1	18	56	4	75	274.65	96	429.62	297	348	301	13	37	0	2	3
6506	11830	8420	458	868	534	28198	975	20	736	7	40	74	9	116	286.41	125	444.59	426	362	369	26	51	0	0	3
4576	13236	9580	806	436	240	28078	1213	7	661	1	10	70	3	65	275.83	104	430.17	326	329	297	22	52	0	0	2
7882	10862	7628	285	1080	702	28234	859	55	791	11	76	102	31	170	287.94	148	445.59	437	364	378	28	63	2	4	5
14482	7664	4360	6	738	548	27844	625	331	1088	163	48	276	118	147	269.78	276	432.37	503	435	428	15	101	24	4	0
4818	13090	9458	742	456	250	28086	1172	6	673	0	14	60	3	90	277.83	93	433.62	336	344	322	10	47	0	0	2
4062	13508	9842	936	430	232	28080	1278	6	579	2	6	54	4	65	270.37	88	422.88	303	272	284	11	40	0	0	3
4672	13074	9456	745	544	346	28130	1173	8	610	0	36	49	5	99	279.74	82	436.26	322	325	332	17	28	0	2	2
11256	7894	5384	1	2050	1646	28658	496	92	588	15	376	179	54	272	314.69	126	464.85	506	169	379	113	101	5	48	19
5722	12392	8874	545	698	436	28160	1044	13	717	3	34	82	7	122	283.61	111	440.61	380	373	342	20	47	0	4	2
14334	7628	4392	10	858	618	27886	653	332	1035	168	54	258	135	145	269.93	277	433.75	499	423	422	15	77	18	2	2
11558	8052	5284	2	1850	1458	28512	564	111	653	22	284	161	64	256	307	132	460.22	499	230	385	77	87	3	22	20
4986	12944	9296	675	536	336	28122	1138	5	668	0	22	61	4	91	277.93	76	433.45	343	353	337	22	38	0	2	2
12544	7744	4936	2	1642	1240	28334	568	161	747	50	196	205	77	239	293.06	156	451.81	499	268	383	61	119	10	26	15
4344	13304	9682	849	470	270	28094	1231	6	591	0	24	74	5	74	274.31	86	429.07	315	298	278	19	51	0	0	4
3904	13708	9948	1009	306	174	28048	1334	7	584	1	8	65	4	63	271.01	92	423.25	263	272	254	13	46	0	0	4
13612	7730	4622	9	1128	828	28024	630	288	895	125	100	224	118	204	277.63	191	444.02	438	356	447	35	77	23	4	4
4272	13562	9776	894	270	154	28042	1247	5	641	1	8	67	3	77	272.4	100	424.22	297	308	299	7	40	0	0	3
4718	13114	9506	742	492	258	28094	1147	7	649	1	6	63	2	75	277.72	111	433.09	368	316	321	17	36	0	0	0
11260	8042	5450	2	1978	1508	28574	537	98	580	17	306	179	50	288	312.36	125	459.7	457	173	379	100	107	9	26	21
11086	7876	5548	2	2180	1600	28662	509	75	537	18	318	167	29	268	315.86	106	461.79	500	157	380	101	82	7	50	31
4110	13480	9796	898	440	254	28092	1258	7	577	1	12	53	4	72	272.23	91	426.31	302	281	291	18	32	0	0	1
4364	13292	9660	852	494	278	28100	1248	9	601	1	12	57	5	83	275.28	93	429.05	301	309	288	14	37	0	0	3
4082	13544	9846	939	364	210	28066	1288	5	580	1	20	59	3	72	272.78	84	425.18	270	291	285	21	41	0	0	1
4462	13216	9616	825	504	284	28102	1213	4	591	0	18	66	2	75	275.65	103	431.75	330	292	291	20	45	0	2	0

4036	13664	9874	969	288	176	28050	1299	6	608	1	12	55	4	78	271.93	91	424.75	274	280	284	6	44	0	0	0
11254	7930	5546	1	1992	1474	28562	506	94	611	20	328	172	54	249	313.52	126	459.96	510	193	408	96	93	1	32	26
4140	13478	9770	876	428	262	28096	1245	5	569	0	16	56	0	72	275.99	100	429.5	314	287	289	15	31	0	2	3
4798	13022	9424	728	552	310	28118	1156	7	657	0	12	66	5	91	276.68	108	434.04	338	321	321	19	49	0	0	3
13242	7848	4726	2	1220	942	28122	610	220	889	78	132	215	108	197	283.38	198	446.36	481	335	428	45	103	11	12	6
8544	10306	7174	222	1260	882	28300	829	67	760	19	126	101	36	178	289.81	126	447.63	425	362	399	47	51	3	6	5
10848	7916	5558	0	2218	1748	28752	504	71	509	13	402	157	35	304	327.03	86	466.77	474	155	356	128	86	4	62	26
13578	7808	4668	9	1066	786	28004	620	245	994	109	88	226	107	160	275.55	240	439.98	488	381	452	38	94	13	10	4
4102	13510	9828	935	402	220	28070	1270	5	587	2	8	59	1	68	271.81	85	425.57	296	280	286	14	42	0	0	3
14500	7632	4312	6	770	586	27854	643	354	1061	167	50	255	141	143	267.61	266	429.43	487	431	442	17	72	20	4	3
5000	12812	9304	682	624	364	28142	1136	12	640	0	38	65	9	90	281.65	99	438.96	359	341	322	22	43	0	0	1
5246	12820	9186	640	530	316	28116	1113	13	719	2	18	71	9	95	280.08	111	437.11	346	376	344	23	51	0	0	3
6978	11614	8176	405	836	534	28184	954	23	755	4	42	94	14	137	287.95	117	445.38	404	362	371	25	57	1	4	5
4634	13322	9576	834	324	186	28052	1215	6	665	1	10	60	4	78	275.23	113	430.54	310	326	322	11	43	0	0	1
13728	7780	4560	5	1018	798	27992	612	276	978	129	100	241	110	169	276.18	240	440.68	497	373	437	29	99	17	8	5
14362	7686	4376	3	792	594	27862	625	318	1094	162	52	282	119	147	270.04	280	432.08	494	432	428	23	107	17	0	0
14184	7592	4440	4	970	680	27930	642	313	1006	157	58	253	121	160	271.42	256	435.74	509	407	412	14	87	13	6	1
12038	7894	5104	2	1754	1364	28434	570	130	693	25	256	165	77	235	300.35	151	454.88	519	243	394	73	92	7	24	12
3948	13706	9956	988	276	140	28028	1306	6	582	1	2	73	3	70	269.88	83	422.96	263	277	269	15	49	0	0	1
12386	7978	5066	3	1488	1146	28294	573	159	796	56	222	173	74	222	287.3	178	449.3	530	280	421	53	83	9	8	2
5212	12740	9160	633	604	388	28150	1106	8	693	1	46	55	6	88	279.61	107	436.56	377	361	345	21	38	0	0	2
14364	7590	4380	3	884	622	27892	630	323	1023	160	50	268	137	167	269.29	237	431.86	484	434	430	16	93	12	2	1
4656	13106	9512	730	530	294	28114	1149	1	634	0	16	75	0	97	278.97	98	435.41	339	326	315	19	46	0	0	1
4460	13328	9626	852	416	240	28078	1237	8	634	1	8	55	4	72	276.98	111	429.87	324	305	292	12	35	0	0	0
12246	7822	4986	3	1690	1366	28408	550	139	686	29	272	185	78	240	302.31	165	456.13	472	222	411	92	92	8	24	13
4610	13202	9568	788	460	242	28088	1199	6	637	0	6	51	3	72	274.66	103	429.24	335	319	321	14	39	0	0	2
5378	12732	9116	629	560	328	28130	1105	7	728	1	14	65	5	90	279.98	111	437	377	368	343	13	43	0	2	4
4322	13458	9724	852	340	202	28064	1246	5	622	0	18	62	4	69	273.87	101	427.75	309	324	293	15	34	0	0	1
4736	13082	9492	749	518	270	28104	1189	5	671	0	6	55	5	93	276.93	97	431.69	326	349	320	11	38	0	0	1
11824	8054	5270	3	1702	1302	28428	560	101	731	26	260	176	55	272	300.72	144	452.29	492	251	396	69	99	6	16	6
4076	13590	9844	945	346	198	28060	1302	8	585	1	6	69	5	83	272.67	78	424.54	270	294	261	9	47	0	0	2
4116	13518	9808	892	392	232	28082	1244	3	571	0	12	63	1	78	274	100	428.74	307	288	289	14	42	0	4	1
12260	7800	5126	4	1722	1226	28356	604	148	738	47	198	179	74	247	295.98	133	451.84	472	274	410	59	99	11	24	10
14240	7700	4432	3	828	626	27900	636	323	1034	152	68	243	134	142	271.39	262	434.5	504	432	451	19	79	22	6	1
12294	7910	5022	0	1626	1258	28344	553	151	741	40	216	182	89	251	291.6	155	452.11	494	261	434	58	101	9	18	7
4242	13476	9764	905	366	204	28060	1266	9	612	2	8	71	4	72	272.82	98	426.81	302	288	266	14	45	0	0	1

SUPPLEMENTARY INFORMATION:Monte Carlo Atomistic Simulation and Machine Learning Analysis of Na-K Eutectic Alloy in Condensed Phases, D. Reitz and E. Blaisten-Barojas, George Mason University, Fairfax, VA 22030

4548	13272	9600	795	414	232	28080	1184	8	634	1	12	70	4	82	277.39	93	433.76	318	310	319	23	50	0	2	0
12656	7830	4982	4	1484	1100	28266	561	181	771	48	206	204	106	226	292.38	162	453.02	499	276	427	61	112	13	8	5
6430	11880	8496	420	832	512	28212	985	19	758	1	60	71	9	101	286.84	116	443	443	405	355	30	47	1	2	3
14376	7728	4336	8	760	612	27872	641	326	1090	154	56	246	133	156	269.93	286	431.62	489	441	450	10	81	21	4	1
11390	7896	5360	3	2038	1548	28576	534	110	616	26	320	169	58	276	313.32	117	462.64	503	208	389	80	92	8	20	17
5216	12710	9138	617	652	412	28164	1094	9	669	0	32	61	7	97	279.95	105	437.82	373	347	340	25	40	0	4	2
14190	7706	4442	7	854	642	27904	641	320	1052	162	64	257	119	154	270.05	262	434.97	487	435	430	22	83	20	6	2
5578	12552	9004	580	606	360	28130	1088	18	718	2	30	60	11	101	283.45	114	438.83	371	389	345	20	35	0	0	1
13052	7818	4690	2	1346	1100	28190	612	203	836	68	168	197	98	203	282.36	192	448.04	502	298	433	40	103	21	14	4
4342	13428	9678	848	368	238	28076	1231	6	616	1	22	64	5	74	273.49	86	426.54	306	305	301	17	46	0	0	3
14324	7602	4338	7	884	680	27904	657	337	1000	158	72	258	132	146	271.57	257	436.1	481	418	418	31	83	25	4	0
11988	8052	5198	1	1676	1250	28384	583	128	731	28	204	169	69	232	294.89	135	449.95	504	267	419	66	103	14	14	8
13768	7572	4410	2	1232	974	28092	574	292	836	110	130	216	131	201	281.7	183	446.48	490	320	457	40	86	17	6	5
5416	12564	9048	592	680	416	28162	1082	8	687	1	34	65	2	112	285.57	92	442.25	359	362	346	25	48	0	4	2
5470	12602	9050	623	614	372	28142	1083	11	701	2	30	69	5	115	280.95	111	437.48	368	348	343	14	48	0	4	0
3968	13644	9916	953	340	180	28048	1273	7	583	1	0	62	4	64	270.02	106	422.36	296	265	285	12	40	0	0	3
14294	7652	4376	3	880	648	27904	638	302	1033	152	52	248	111	142	273.2	255	438.9	490	414	445	28	86	15	2	2
4062	13576	9854	924	356	204	28064	1278	5	578	0	10	62	3	68	272.03	92	426.05	295	284	278	13	41	0	2	2
5098	12848	9278	657	574	312	28118	1102	14	681	1	8	71	7	87	279.24	113	435.72	382	332	334	15	46	0	0	3
3608	13816	10072	1025	358	206	28066	1330	4	520	1	6	63	3	76	270.4	73	422.35	251	241	259	15	41	0	0	3
4602	13282	9580	824	378	212	28064	1237	11	683	1	10	51	5	71	275.14	100	429.94	308	345	309	15	40	0	0	1
13570	7764	4600	4	1144	874	28072	613	227	946	87	112	210	94	176	282.69	237	445.21	507	351	436	37	95	16	8	3
11698	7834	5274	0	1920	1456	28508	561	118	624	25	286	172	65	248	305.1	124	456.82	486	199	383	100	95	11	38	11
4156	13532	9808	914	346	204	28064	1251	10	586	2	16	55	5	66	273.5	91	427.21	305	273	302	17	33	0	2	0
11494	7896	5348	0	1946	1518	28582	516	105	585	24	344	184	57	276	314.83	98	459.96	473	190	400	94	103	7	34	25
4206	13450	9752	854	406	248	28086	1234	6	603	2	24	53	2	79	274.44	84	428.15	312	304	299	14	31	0	0	1
3842	13728	9984	977	308	174	28048	1298	6	557	1	12	59	3	68	270.77	85	423.51	270	264	282	15	37	0	0	3
5684	12386	8894	580	710	442	28158	1094	25	672	4	38	69	12	93	285.81	105	442.33	396	367	313	21	37	0	4	1
4824	12962	9420	698	588	320	28132	1126	5	663	0	18	54	2	81	276.85	120	434.2	384	320	334	14	37	0	0	1
5144	12742	9192	657	634	398	28152	1099	11	656	0	38	66	7	92	277.42	105	435.33	382	334	337	19	36	0	4	2
4062	13604	9856	932	330	192	28054	1291	6	616	1	10	58	5	72	273.1	93	425.11	279	296	281	12	43	0	0	2
12330	7778	5006	0	1672	1320	28408	599	168	693	42	278	177	92	264	299.51	131	453.98	443	267	410	68	91	16	22	14
6918	11484	8144	396	986	636	28230	942	34	721	8	58	95	21	139	287.91	110	443.77	440	360	349	25	69	1	4	1
4138	13542	9800	920	358	214	28062	1291	8	593	1	8	54	3	77	271.17	84	423.91	273	312	280	14	34	0	2	2
12318	8002	5058	4	1516	1184	28304	604	147	821	41	208	178	75	235	290.68	169	452.03	493	296	401	53	112	7	18	7
11090	7694	5388	0	2298	1816	28766	500	72	499	17	428	151	39	290	318.55	95	462.99	502	167	374	113	68	4	48	32

SUPPLEMENTARY INFORMATION:Monte Carlo Atomistic Simulation and Machine Learning Analysis of Na-K Eutectic Alloy in Condensed Phases, D. Reitz and E. Blaisten-Barojas, George Mason University, Fairfax, VA 22030

14098	7580	4382	4	1024	798	27992	610	298	927	129	100	241	119	188	276.04	220	443.35	466	352	452	32	99	28	8	3
5308	12666	9108	640	634	394	28144	1121	13	686	2	30	67	8	107	283.28	103	438.67	341	350	335	22	44	0	4	2
4450	13312	9640	852	424	242	28082	1254	5	629	1	10	59	4	81	277.26	94	431.04	289	335	295	16	39	0	4	2
4244	13398	9740	898	442	246	28080	1273	10	597	2	10	56	5	80	274.89	90	428.83	289	293	279	13	34	0	0	2
3930	13570	9900	958	430	242	28084	1294	6	542	2	10	64	2	76	273.05	88	426.21	280	254	266	13	45	0	2	4
4732	13064	9486	721	526	286	28112	1174	5	667	2	18	63	3	82	276.84	102	432.92	329	337	324	21	44	0	0	4
4122	13536	9806	923	370	222	28070	1266	9	576	1	12	55	6	61	272	95	425.03	300	276	292	19	33	0	2	2
4752	13006	9466	728	576	304	28114	1162	6	665	0	10	59	1	75	278.69	114	435.59	376	337	299	13	43	0	0	2
5426	12542	9058	592	690	410	28166	1083	3	702	2	38	72	0	87	282.37	107	438.11	402	375	325	23	50	0	2	2
5142	12790	9182	660	618	390	28150	1126	4	687	1	26	71	1	102	280.6	113	436.42	356	340	322	17	49	0	2	3
14188	7644	4362	4	914	736	27942	636	321	989	155	90	257	119	163	274.41	227	439.29	476	412	427	33	87	17	6	1
4004	13568	9866	946	386	230	28074	1298	8	570	0	20	47	4	66	273.57	86	427.24	281	279	278	17	33	0	0	3
12046	8016	5194	0	1624	1244	28380	575	125	766	20	230	170	71	265	298.83	147	453.11	459	262	429	70	111	12	22	7
4244	13444	9754	908	392	224	28074	1290	7	596	1	16	53	4	84	273.28	64	426.16	274	312	281	11	35	0	0	1
5482	12526	9022	552	704	406	28160	1054	12	686	0	20	61	7	101	280.6	116	437.96	392	369	356	22	32	0	0	3
5496	12508	9034	610	678	394	28142	1087	20	695	4	28	69	10	104	281.41	111	438.02	368	351	343	19	50	1	4	2
4748	13112	9486	776	462	258	28082	1179	9	647	2	16	72	4	80	275.61	104	430.58	344	323	305	11	47	0	0	3
13848	7782	4540	10	968	744	27974	653	280	990	132	84	232	113	183	276.11	232	439.85	455	415	439	26	85	13	8	2
4214	13526	9798	905	326	176	28046	1265	9	625	1	6	60	7	79	270.88	92	423.68	273	306	301	17	36	0	0	0
14158	7784	4510	6	796	578	27880	636	320	1041	159	46	252	124	153	274.89	266	438.62	477	409	451	22	76	16	8	2
14154	7590	4420	3	976	716	27936	576	317	994	158	74	254	129	164	272.81	269	438.36	500	368	461	33	86	13	6	2
4778	13128	9452	763	448	270	28098	1178	6	678	0	22	75	6	76	276.4	113	431.72	353	340	300	15	49	0	0	1
10842	8064	5666	1	2090	1596	28676	497	80	569	16	382	167	38	290	323.78	108	467.71	478	180	374	110	82	3	36	30
5620	12488	8930	564	660	420	28158	1072	7	736	0	40	72	6	104	281.16	112	438.15	389	396	334	21	47	0	0	0
5106	12864	9232	641	554	348	28134	1150	9	696	2	30	60	4	94	280.33	91	438.32	332	389	331	25	37	0	0	2
3922	13678	9954	984	314	168	28046	1323	7	568	1	10	54	5	66	271.62	80	424.53	270	283	269	9	35	0	0	3
4646	13156	9518	787	486	276	28094	1200	9	633	2	12	57	2	74	275.61	91	432.48	334	319	307	15	44	0	0	4
6284	11940	8540	498	856	524	28184	992	25	715	6	40	85	12	108	285.01	131	440.74	417	342	349	33	59	0	0	3
3994	13648	9904	962	306	180	28050	1295	6	568	1	18	59	3	72	272.06	80	424.09	275	277	276	11	42	0	0	4
4220	13450	9760	900	402	230	28076	1251	8	582	2	14	51	5	69	274.62	88	428.84	308	285	305	13	35	0	0	1
14558	7618	4376	5	732	502	27826	668	346	1102	167	36	256	137	145	267.84	277	430.73	471	455	437	14	80	21	4	0
11530	7938	5400	1	1898	1442	28560	553	90	669	16	302	165	41	263	316.57	148	463.34	500	227	367	86	90	9	46	18
6530	11758	8398	396	920	566	28224	956	16	754	5	52	78	9	122	287.28	108	444.77	442	367	369	27	47	0	0	0
14268	7664	4416	4	842	636	27906	619	319	1053	153	74	258	122	163	272.15	269	437.95	484	413	439	27	94	14	6	0
3952	13614	9904	937	364	216	28072	1289	5	561	0	22	59	4	75	272.21	77	427.22	275	281	280	14	40	0	0	4
4214	13494	9758	878	382	220	28072	1261	4	622	0	4	56	1	70	273.72	94	428.91	292	308	297	19	33	0	0	1

SUPPLEMENTARY INFORMATION:Monte Carlo Atomistic Simulation and Machine Learning Analysis of Na-K Eutectic Alloy in Condensed Phases, D. Reitz and E. Blaisten-Barojas, George Mason University, Fairfax, VA 22030

3808	13692	9968	989	366	218	28068	1308	6	542	1	16	59	3	80	270.16	82	423.54	262	255	269	13	38	0	0	3
11364	7966	5404	0	1954	1520	28580	558	93	599	23	338	159	47	273	311.99	118	460.76	477	203	374	90	71	9	34	25
14440	7696	4410	6	726	518	27832	618	329	1122	169	38	269	126	144	267.64	288	429.84	483	439	458	20	90	16	4	3
13812	7808	4564	5	934	738	27966	646	294	1001	141	110	242	118	148	275.26	245	438.51	497	418	423	32	85	16	0	3
5762	12440	8898	581	624	378	28134	1094	12	753	3	28	74	5	106	282.05	109	438.59	358	400	342	15	42	1	4	6
13792	7746	4568	6	1000	774	27998	624	284	984	134	104	245	114	179	277.27	232	441.77	484	390	424	28	101	18	14	5
4086	13534	9822	901	386	230	28074	1276	2	600	0	14	58	1	80	271.43	87	425.02	281	305	285	11	41	0	2	4
4902	13106	9440	741	402	212	28070	1157	6	714	0	8	58	3	89	277.7	105	434.63	325	345	349	14	44	0	0	2
5482	12516	8988	546	708	446	28182	1094	5	712	0	38	77	5	105	281.89	110	437.45	375	400	323	16	51	0	4	8
13360	7840	4778	5	1138	824	28062	610	244	926	91	116	203	115	183	283.3	208	447.28	491	360	456	38	97	13	6	2
11028	7842	5510	0	2226	1674	28676	488	70	568	8	344	150	31	299	318.65	99	461.46	502	180	391	83	86	4	52	31
4794	13032	9442	724	514	302	28118	1126	6	658	0	32	63	6	89	276.19	113	432.52	366	316	335	14	45	0	2	2
4464	13322	9634	852	408	232	28070	1251	5	624	2	10	68	1	82	274.47	87	430.18	302	315	279	10	49	0	0	2
4852	12996	9400	758	532	308	28110	1186	9	656	0	22	60	4	95	278.33	104	434.52	325	324	310	15	47	0	0	1
14120	7692	4480	7	892	652	27908	653	316	1017	163	72	262	105	155	273.26	232	435.56	484	433	409	24	84	24	0	2
4474	13328	9628	825	410	232	28080	1242	4	643	0	8	53	2	79	274.7	91	429.63	291	335	304	20	37	0	0	2
13490	7640	4596	3	1230	980	28124	631	223	878	76	172	198	113	191	282.24	173	445.06	443	337	454	55	98	13	16	10
5542	12562	9028	592	610	360	28136	1075	10	742	0	34	57	4	97	279.72	121	437.27	392	379	355	14	37	0	0	1
12666	7838	4926	2	1482	1140	28272	592	164	807	63	204	200	67	217	292.24	167	452.62	493	290	401	63	116	12	16	8
4480	13318	9648	831	400	212	28064	1223	11	633	1	6	65	5	80	274.44	107	428.65	309	306	300	11	45	0	0	4
11334	7840	5412	1	2032	1584	28614	527	78	596	21	364	177	41	286	313.41	109	457.78	479	205	388	97	91	5	42	15
7210	11464	8080	362	848	528	28182	958	28	851	9	50	96	16	114	289.4	149	446	449	434	347	22	68	0	2	3
4260	13470	9762	876	358	198	28058	1271	8	626	1	10	63	5	70	271.97	101	425.4	302	323	278	9	42	0	0	2
6976	11632	8208	364	810	508	28186	946	29	811	10	50	75	16	133	286.39	128	445.52	429	414	392	17	40	0	2	1
4926	12868	9328	673	636	366	28146	1129	6	651	0	22	66	4	95	278.96	110	435.55	365	330	316	20	45	0	0	1
5278	12786	9176	649	534	318	28116	1105	7	715	2	22	59	1	101	280	125	437.56	352	342	357	17	40	0	2	2
4116	13576	9828	932	340	192	28056	1284	4	593	0	4	68	2	79	271.88	83	425.69	282	282	270	11	50	0	0	1
13920	7736	4476	4	978	770	27978	654	280	978	126	90	246	120	180	275.84	222	442.17	454	388	427	31	107	15	8	2
14482	7498	4338	2	892	616	27880	614	329	1029	177	48	286	109	168	273.17	248	436.7	493	406	416	18	95	21	6	0
5270	12560	9152	612	738	414	28172	1087	5	675	1	36	75	2	107	284.46	108	440.34	384	341	313	20	49	0	2	3
5234	12724	9180	615	618	358	28138	1101	7	706	1	22	59	5	93	280.45	115	438.11	371	371	347	20	38	0	2	2
5540	12430	8978	609	744	438	28162	1066	18	648	5	30	70	11	113	283.35	104	440.02	392	332	335	12	42	0	2	3
4636	13228	9546	814	400	242	28078	1220	6	672	0	24	60	5	69	277.47	96	432.71	312	340	314	17	40	0	2	5
7418	11194	7896	369	970	656	28232	933	37	765	15	86	93	17	144	288.61	119	444.77	424	378	364	27	53	0	12	4
5434	12594	9106	596	622	346	28132	1048	14	724	1	28	58	7	95	279.8	118	437.95	385	353	383	19	40	0	2	5
4590	13206	9540	799	462	278	28096	1194	13	614	2	18	60	9	87	275.55	102	430.37	335	309	310	8	35	0	2	2

14256	7672	4352	3	892	690	27930	615	312	1012	151	66	249	124	151	271.99	273	436.22	510	395	447	21	78	21	2	3
4448	13316	9648	854	410	234	28074	1237	4	603	0	18	64	3	73	275.06	94	431.24	305	309	301	14	37	0	0	4
14320	7742	4414	6	774	570	27860	656	327	1084	165	36	253	116	138	270.14	287	432.98	495	440	433	16	79	25	4	1
4014	13618	9878	944	338	196	28054	1284	7	588	1	10	70	4	65	270.68	90	423.25	292	285	267	14	39	0	0	3
4384	13386	9688	845	392	216	28074	1226	7	648	0	8	64	4	73	274.98	107	428.36	327	301	293	11	47	0	0	1
4026	13554	9854	904	410	232	28086	1260	5	566	0	10	56	5	78	274.51	93	428.56	296	281	290	16	33	0	0	0
11924	7786	5170	1	1880	1412	28472	564	123	639	29	282	166	62	258	304.94	127	455.22	480	237	404	84	88	9	16	10
5524	12536	9008	619	652	392	28144	1104	12	699	1	32	59	8	99	282.42	89	437.41	366	361	346	19	39	0	0	0
4444	13254	9616	815	494	284	28108	1207	4	609	0	14	56	2	89	277.04	89	432.86	315	302	311	14	27	0	2	3
6372	11760	8458	397	978	612	28252	948	10	721	3	70	93	7	147	286.76	114	445.53	425	367	355	26	59	0	2	2
5108	12744	9246	675	662	362	28134	1116	14	660	3	12	54	7	93	280.14	107	437.21	370	326	341	14	36	0	0	3
11838	7892	5286	0	1794	1340	28450	580	123	684	24	266	172	67	249	303.39	136	455.1	462	252	405	91	101	9	32	12
4686	13126	9496	771	500	284	28106	1209	3	647	0	12	61	2	77	276.58	100	431.53	327	353	302	19	40	0	2	1
5242	12778	9194	617	556	326	28122	1089	7	712	0	26	68	1	103	280.83	101	436.7	365	363	345	18	43	0	0	2
5808	12220	8854	506	796	462	28192	1060	13	732	0	50	71	8	104	283.67	111	439.56	404	404	329	21	50	0	2	3
3790	13772	10014	989	298	164	28044	1313	7	562	2	6	58	2	63	270.25	91	422.79	276	273	270	10	38	0	0	4
4326	13478	9736	877	318	180	28048	1241	10	603	3	10	65	3	72	274.61	71	429.3	289	314	307	17	38	0	0	4
3992	13614	9894	952	336	200	28058	1269	8	568	2	22	65	4	76	269.83	90	422.7	300	267	272	8	42	0	0	2
4042	13672	9902	946	266	144	28032	1278	7	607	1	6	68	4	55	272.26	104	425.51	306	285	276	12	43	0	0	1
4066	13572	9836	934	360	218	28068	1299	7	577	1	16	62	4	69	271.47	87	425.12	273	300	275	18	37	0	0	2
14176	7674	4388	10	916	702	27924	654	309	1005	167	64	271	102	162	272.27	261	436.71	492	406	387	21	91	16	2	2
12822	7890	4828	2	1410	1098	28222	619	175	834	67	162	205	72	225	285.54	172	448.73	469	317	407	47	108	19	10	8
4688	13194	9518	770	438	242	28086	1178	5	679	0	6	75	3	67	276.53	122	433.23	356	325	301	17	45	0	0	2
13782	7734	4492	3	1082	830	28004	603	268	951	126	78	232	114	174	276.17	238	441.82	512	365	440	24	82	9	6	3
7524	11044	7806	358	1078	704	28240	928	45	716	13	82	91	27	142	289.58	114	445.36	424	353	365	36	56	1	2	2
4116	13566	9826	901	346	198	28060	1253	7	598	1	8	53	3	72	273.36	108	426.08	299	284	303	12	29	0	0	2
13478	7796	4642	2	1112	892	28074	662	252	941	91	140	216	109	193	276.81	200	443.98	437	365	429	48	98	25	12	2
13048	7744	4794	3	1398	1038	28190	618	231	824	71	152	195	116	202	283.09	188	449.42	461	323	446	53	94	21	16	11
5122	12824	9254	650	564	332	28126	1121	12	697	1	28	70	7	88	279.93	108	435.97	372	370	321	18	48	0	2	1
11456	8002	5430	2	1920	1416	28534	506	98	634	20	288	176	55	291	312.2	125	457.27	496	200	387	86	96	4	20	13
4572	13204	9550	809	464	280	28094	1229	6	635	0	24	57	5	64	276.26	102	430.54	328	327	293	21	41	0	0	1
3850	13638	9974	995	388	202	28060	1297	11	532	1	8	62	8	78	272.02	86	425.09	267	254	278	12	42	0	0	3
4110	13606	9830	935	312	186	28052	1306	5	612	2	8	56	2	65	271.42	84	425.64	281	311	275	12	37	0	0	1
13560	7734	4576	6	1174	902	28064	646	269	887	131	110	234	108	203	275.84	219	442.36	475	365	402	29	79	18	8	0
4608	13174	9574	776	484	248	28096	1195	5	658	0	8	65	4	82	277.91	101	432.99	326	341	308	17	38	0	0	2
10594	7774	5674	0	2476	1850	28844	460	54	436	9	418	173	25	315	336.43	83	475.56	487	119	349	134	76	2	54	32

6086	11942	8628	484	962	570	28230	1018	21	652	3	38	79	14	122	289.99	92	443.83	411	356	329	25	54	0	2	5
5002	12830	9304	734	618	352	28130	1185	10	657	2	24	65	3	87	280.27	100	436.31	342	339	295	16	39	0	0	3
11662	7880	5140	0	1942	1588	28576	539	107	632	24	328	158	60	266	305.7	121	460.21	483	203	399	99	88	4	36	18
4134	13510	9820	914	384	210	28068	1260	8	566	1	10	55	4	71	272.72	93	426.28	303	266	292	9	35	0	0	4
4654	13184	9524	772	448	262	28092	1215	8	663	1	20	53	3	83	275.34	86	428.99	318	364	309	15	41	0	0	0
5648	12468	8966	575	644	380	28144	1053	15	720	2	38	77	9	96	283.18	121	439.31	395	348	340	25	52	1	0	3
3932	13704	9962	981	286	146	28034	1318	5	595	1	4	63	2	60	270.68	83	423.42	273	292	264	15	44	0	0	1
4900	12950	9404	711	552	292	28114	1172	10	685	0	16	59	8	93	277.82	90	433.34	337	365	317	13	38	0	0	2
13994	7794	4520	6	868	670	27932	619	291	1056	146	82	258	107	167	275.1	240	440.4	484	419	438	26	99	16	4	2
13578	7708	4512	3	1192	964	28098	603	249	849	91	130	216	123	204	282.71	186	444.62	474	340	439	43	104	15	14	2
10796	7822	5742	0	2296	1646	28740	484	68	512	12	398	176	35	319	332.42	83	473.53	481	159	338	113	87	4	34	26
5038	12906	9316	684	530	300	28110	1130	4	723	0	20	76	2	83	277.03	129	432.95	358	363	322	22	46	0	0	3
12594	7982	4996	3	1406	1072	28240	586	166	844	49	174	185	83	216	291.73	185	450.74	478	295	440	62	104	10	16	7
14296	7628	4376	5	842	660	27894	615	335	1043	161	90	265	130	148	270.07	277	432.23	508	421	435	20	94	17	2	2
4780	12952	9392	729	620	372	28146	1146	5	628	1	30	60	3	104	280.96	98	437.97	351	307	320	12	40	0	0	2
7402	11186	7858	335	1022	694	28248	883	40	758	11	82	99	20	149	288.67	133	445.59	452	353	372	33	56	2	2	1
11574	7774	5270	0	2032	1572	28598	541	105	586	22	332	158	58	276	313.07	109	461.74	493	207	385	94	82	5	42	17
7138	11322	8050	370	1020	640	28236	949	33	741	7	60	94	22	135	287.63	132	444.02	434	379	347	27	52	0	6	4
4126	13514	9820	926	376	214	28066	1270	9	600	1	16	53	5	60	272.42	98	425.56	295	273	291	20	31	0	0	3
14424	7650	4400	7	764	554	27852	670	356	1067	167	58	257	135	140	271.89	270	435.4	470	435	425	26	84	24	2	0
14506	7688	4330	6	726	550	27842	652	343	1083	159	36	259	150	158	268.39	271	430.01	466	453	450	11	87	15	6	1
13020	7852	4830	3	1302	990	28162	599	194	865	67	156	200	93	217	283.45	191	446.17	485	312	440	38	104	15	12	5
14618	7642	4318	6	696	508	27822	662	347	1113	171	38	272	132	134	268.2	288	430.1	487	459	422	17	88	24	2	0
3944	13702	9956	974	280	144	28030	1294	9	567	2	4	61	4	67	271.53	81	424.55	272	272	286	13	39	0	0	2
13690	7798	4626	4	1042	758	27996	594	274	945	120	74	232	119	175	276.07	240	439.73	506	362	452	24	93	14	6	5
5316	12760	9170	608	536	312	28122	1085	12	727	0	28	67	6	80	282.89	116	438.97	405	372	336	18	51	0	0	0
4602	13220	9524	765	438	286	28104	1184	9	638	1	32	63	7	73	276.01	101	430.79	340	330	312	20	46	0	2	3
12360	7854	5060	3	1622	1196	28308	590	149	720	57	202	215	67	251	295.29	140	453.13	468	266	382	69	111	7	14	3
13996	7770	4500	8	916	682	27926	628	303	999	153	60	254	115	161	271	267	433.61	505	406	427	16	86	17	2	2
4644	13234	9556	788	396	224	28070	1197	13	669	2	16	70	8	82	273.78	88	429.98	315	347	311	17	43	0	0	1
11198	7934	5450	2	2078	1584	28602	532	86	592	18	302	180	51	265	313.75	106	459.6	471	193	384	114	101	4	56	22
4264	13472	9738	895	352	218	28064	1275	5	616	0	18	55	2	75	273.78	74	426.14	280	313	290	17	40	0	2	1
12772	7846	4932	3	1440	1060	28228	582	176	818	67	158	204	73	219	293.07	176	450.43	491	282	414	56	103	17	20	7
4102	13590	9848	937	312	182	28050	1302	8	627	1	16	59	5	65	271.04	106	423.56	280	311	271	14	39	0	0	2
14244	7606	4342	3	924	724	27928	621	315	976	158	88	268	113	180	272.54	228	438.5	466	399	431	25	93	21	0	3
13194	7924	4788	5	1184	890	28104	599	208	950	95	116	226	83	197	281.71	212	447.61	503	342	424	36	105	16	8	3

SUPPLEMENTARY INFORMATION:Monte Carlo Atomistic Simulation and Machine Learning Analysis of Na-K Eutectic Alloy in Condensed Phases, D. Reitz and E. Blaisten-Barojas, George Mason University, Fairfax, VA 22030

13084	8022	4856	8	1150	866	28094	640	203	961	76	104	182	95	185	280.43	222	443.7	473	377	466	37	87	13	12	2
6544	11668	8362	444	980	618	28240	966	21	676	5	62	84	13	133	287.63	110	442.99	432	327	350	24	54	1	6	2
3914	13656	9942	959	348	188	28054	1287	8	549	1	4	62	4	68	272.35	87	425.84	288	270	270	13	37	0	2	3
5240	12770	9172	639	576	348	28132	1100	10	696	1	26	69	7	88	279.55	116	437.46	376	344	332	21	51	0	0	3
12478	7782	4976	3	1614	1230	28320	579	155	753	35	214	197	79	256	289.37	154	450.23	485	252	390	47	115	11	26	13
4210	13492	9770	880	372	214	28068	1236	8	589	0	10	71	6	80	274.72	91	428.58	301	288	290	14	49	0	0	3
14230	7724	4436	5	802	618	27884	630	305	1085	162	68	259	113	164	269.3	272	432.05	484	425	442	16	86	14	6	0
14364	7754	4408	6	724	546	27842	638	317	1107	161	44	261	126	137	267.55	284	429.58	485	450	448	22	81	12	2	2
12230	8014	5048	1	1560	1242	28332	551	125	775	35	222	200	52	248	301.98	159	455.75	474	251	411	72	117	14	16	11
7688	11098	7838	322	914	576	28200	897	48	822	10	80	84	29	132	288.45	156	443.26	441	392	399	28	51	0	6	5
6786	11666	8288	398	884	536	28202	997	27	811	6	40	79	13	123	285.22	141	443.3	425	416	347	17	45	3	2	3
4386	13232	9656	810	526	292	28116	1203	6	587	1	24	56	4	95	277.65	86	432.52	309	297	315	17	36	0	0	0
5260	12684	9154	635	654	378	28154	1097	8	678	1	24	69	7	94	280.7	111	439.42	384	347	333	18	45	0	0	2
13734	7610	4488	5	1170	916	28056	623	275	896	114	128	237	119	200	278.01	193	444.47	441	355	437	45	99	16	8	6
5866	12216	8818	554	758	454	28164	1075	25	723	10	50	72	9	104	282.97	101	441.05	381	382	329	24	46	0	2	2
3982	13662	9932	968	296	158	28042	1288	9	594	2	12	65	4	75	271.08	92	423.13	274	271	281	11	40	0	0	2
7318	11042	7902	309	1158	758	28296	898	29	735	8	116	96	17	139	287.81	113	446.54	458	374	356	36	54	1	2	5
14188	7590	4462	10	950	658	27912	630	315	995	158	56	259	112	160	270.67	233	435.17	479	406	436	28	87	21	8	1
4506	13206	9572	803	510	300	28114	1224	7	623	2	20	58	3	67	276.41	101	432.66	327	321	294	22	43	0	0	2
11978	7914	5150	2	1786	1340	28414	566	145	703	36	230	167	72	252	301.48	160	451.58	480	236	410	80	73	12	12	9
5718	12412	8932	574	670	382	28142	1094	12	729	2	26	73	9	88	284.93	127	439.31	385	372	333	20	45	0	2	3
5114	12844	9226	664	564	352	28128	1101	13	687	1	26	61	7	87	280.23	112	437	380	342	341	19	40	1	2	2
5466	12530	9044	574	664	402	28152	1064	12	706	0	42	74	9	95	284.17	116	440.68	395	361	337	24	46	0	4	2
11038	7910	5634	0	2144	1546	28648	528	75	574	11	330	159	37	264	319.28	113	461.92	487	187	381	119	89	7	44	19
12368	7936	5106	2	1566	1130	28298	553	163	784	45	174	169	82	238	299.79	156	452.36	474	261	467	65	98	12	16	9
13550	7696	4544	2	1194	956	28088	624	277	846	111	132	229	118	211	277.94	182	443.91	453	329	419	40	97	16	16	5
6404	11816	8442	437	902	592	28236	995	20	722	6	78	89	10	112	289.67	115	445.49	439	391	329	29	52	1	2	2
14328	7738	4446	13	750	538	27842	648	341	1079	166	42	271	135	137	269.97	283	431.96	484	436	437	22	93	21	0	0
13122	7826	4806	0	1308	952	28152	599	216	838	80	132	222	91	196	283.19	184	450.47	491	314	415	53	109	15	6	5
14068	7728	4426	6	922	718	27936	574	304	1015	152	70	268	119	170	272.79	267	437.74	519	377	440	21	98	16	4	2
5146	12776	9204	676	620	370	28138	1160	15	673	2	22	56	7	97	279.78	94	435.22	339	353	320	19	33	1	0	1
5958	12212	8726	525	766	476	28174	1013	8	716	1	36	84	5	108	286.23	115	443.06	408	350	349	29	51	0	0	1
14128	7692	4472	7	894	654	27908	643	296	1042	148	64	256	106	163	272.56	240	438.25	485	432	418	20	93	13	4	2
11116	7986	5522	0	2028	1574	28634	523	72	611	19	374	171	35	278	313.78	133	463.39	489	181	362	105	91	5	30	26
4244	13492	9770	879	344	196	28060	1260	10	636	2	14	57	6	70	271.94	97	425.21	297	314	297	14	42	0	0	1
14348	7670	4372	4	812	620	27890	647	325	1064	161	64	250	130	154	270.9	265	433.62	490	440	436	15	82	19	4	1

SUPPLEMENTARY INFORMATION:Monte Carlo Atomistic Simulation and Machine Learning Analysis of Na-K Eutectic Alloy in Condensed Phases, D. Reitz and E. Blaisten-Barojas, George Mason University, Fairfax, VA 22030

5300	12724	9150	642	586	344	28126	1140	8	691	0	20	71	8	108	282.26	89	439.81	330	382	324	19	47	0	2	2
11756	7766	5220	0	1960	1484	28518	563	109	634	18	314	165	64	269	309.13	129	455.11	484	233	381	80	89	6	12	18
12114	7850	5148	1	1724	1278	28360	588	151	722	43	222	182	76	231	294.73	142	450.4	492	267	389	73	93	16	20	10
5278	12758	9180	642	558	322	28118	1112	11	699	1	20	64	8	85	279.17	100	436.27	370	373	340	20	42	0	2	1
11982	7854	5172	1	1790	1354	28434	553	127	702	28	248	173	71	268	304.32	144	456.76	471	243	411	77	80	9	34	13
13106	7918	4792	3	1212	960	28164	587	204	876	76	170	195	92	206	286.07	189	447.06	491	327	446	48	97	12	6	5
4444	13224	9606	805	512	304	28118	1222	3	604	0	28	64	2	83	275.89	82	430.96	316	319	291	20	43	0	0	1
6154	12200	8658	465	692	428	28162	992	10	788	3	28	86	6	123	282.99	118	441.11	392	394	375	22	53	0	2	5
13074	7604	4694	5	1482	1162	28240	572	216	754	79	206	203	94	248	292.25	145	450.42	464	292	424	56	85	13	18	5
4158	13510	9802	903	378	212	28070	1287	8	583	2	10	55	4	75	272.79	80	427.14	279	310	283	15	35	0	0	1
13684	7726	4594	3	1100	816	28026	620	249	931	101	98	238	106	204	277.23	213	445.31	461	359	432	28	113	23	8	5
11398	7892	5346	2	2024	1566	28588	520	111	557	30	322	173	54	283	314.12	117	462.48	475	165	388	99	83	4	40	25
3898	13686	9966	981	318	168	28044	1314	6	577	1	8	64	3	66	271.24	77	423.24	267	280	268	15	45	0	0	2
5518	12564	9058	592	604	344	28118	1071	8	716	0	28	72	5	107	280.68	95	438.43	355	371	363	23	59	0	2	2
14346	7636	4382	8	852	616	27884	645	297	1077	162	50	256	110	139	270.19	280	433.23	500	444	433	22	80	12	2	0
5666	12420	8874	548	710	462	28174	1048	15	696	5	36	62	7	103	282.27	110	440.82	394	370	360	25	36	1	6	1
12378	7924	5084	0	1592	1136	28290	606	137	821	46	166	200	66	208	293.15	183	450.67	495	295	399	70	112	9	10	5
14532	7636	4330	5	740	556	27852	611	353	1054	173	54	271	138	158	272.45	267	437.07	498	409	443	11	84	19	4	2
4086	13658	9876	930	266	144	28032	1276	5	616	1	2	70	4	79	271.71	79	424.91	270	304	289	10	45	0	0	3
11972	7908	5210	2	1750	1318	28438	553	126	699	33	246	170	69	232	302.92	149	453.45	523	248	399	82	98	7	32	10
13720	7588	4504	3	1186	922	28068	630	265	906	103	136	228	124	172	277.27	202	445.87	475	364	429	53	99	19	12	2
12186	7976	5074	3	1622	1266	28364	582	149	750	41	212	170	81	235	293.48	153	452.11	474	271	435	68	87	13	26	11
13838	7702	4524	5	1058	780	27984	644	274	973	131	78	238	111	181	275.13	221	441.87	460	392	434	30	89	16	4	3
10850	7904	5680	1	2256	1628	28718	499	68	539	14	364	170	33	306	321.98	101	472.3	483	160	364	104	93	5	34	30
14368	7568	4320	5	908	678	27908	646	326	1013	157	64	257	131	171	270.4	230	434.81	459	422	443	19	86	19	2	2
11054	8276	5584	4	1882	1452	28576	525	75	649	11	292	168	35	303	317.66	114	462.6	471	213	387	84	96	7	32	19
4002	13562	9890	943	386	212	28068	1306	2	587	0	16	68	2	66	271.21	80	423.82	281	301	256	17	49	0	0	2
4814	13030	9438	717	536	286	28110	1152	4	634	1	6	73	2	93	277.87	94	435.89	349	332	312	16	47	0	0	0
3850	13662	9934	975	386	232	28078	1310	5	551	1	14	62	4	71	271.02	88	423.54	274	271	259	16	40	0	0	3
13836	7720	4540	4	1050	760	27978	621	317	946	137	70	220	142	185	275.12	210	438.86	461	381	472	28	72	11	2	2
12712	8008	4900	2	1366	1068	28220	624	147	874	48	150	174	77	202	286.08	178	448.23	467	342	450	53	95	6	14	8
4016	13652	9888	946	304	176	28044	1263	6	583	1	8	72	4	74	270.35	97	423.7	292	272	276	12	45	0	0	3
11466	7894	5382	2	2006	1484	28554	542	90	604	18	294	172	47	272	311.57	109	458.49	485	209	372	95	95	5	24	20
14300	7728	4410	8	802	588	27868	653	329	1064	167	38	262	125	149	267.62	265	429.03	487	430	426	16	83	15	2	1
4652	13170	9534	764	470	258	28096	1175	3	642	0	12	60	1	79	275.51	100	432.88	339	327	324	14	37	0	0	4
4598	13168	9572	791	476	258	28092	1201	8	638	0	20	57	6	71	279.55	92	435.27	330	322	312	20	41	0	0	2

SUPPLEMENTARY INFORMATION:Monte Carlo Atomistic Simulation and Machine Learning Analysis of Na-K Eutectic Alloy in Condensed Phases, D. Reitz and E. Blaisten-Barojas, George Mason University, Fairfax, VA 22030

3910	13734	9968	1004	268	146	28034	1317	7	585	1	8	58	5	68	269.82	86	422.03	262	270	276	13	45	0	0	1
11414	7902	5318	1	2042	1576	28600	530	92	619	21	310	154	48	274	307.52	125	455.6	525	203	388	82	85	5	36	17
4806	12984	9410	724	580	332	28132	1178	7	640	0	18	52	3	88	280.32	82	434.72	348	345	313	10	36	0	2	4
5432	12504	9030	614	730	436	28164	1085	13	680	3	26	72	7	108	285.68	120	441.88	379	330	319	23	49	0	6	0
13378	7704	4650	2	1258	970	28120	612	231	862	95	150	213	101	206	284.43	190	447.39	462	326	445	40	96	15	10	9
11946	7944	5248	0	1714	1288	28426	574	118	723	32	266	173	53	236	304.47	141	456.5	481	247	397	87	107	7	20	15
12926	7970	4928	6	1256	916	28130	614	192	937	63	128	201	88	193	281.64	193	444.69	495	359	423	47	110	13	6	2
7352	11202	7942	319	1018	646	28234	879	35	763	10	66	102	19	130	288.25	134	446.53	456	375	379	38	58	1	8	5
4930	12870	9324	655	618	368	28142	1123	4	648	1	32	61	2	84	280.53	104	438.27	370	343	329	21	40	0	0	4
4232	13376	9726	875	476	272	28094	1266	7	583	3	12	62	3	82	274.2	85	428.37	294	303	279	15	38	0	0	0
5146	12794	9244	677	590	334	28130	1141	6	682	0	20	55	6	88	278.82	97	436.08	356	354	342	15	37	0	2	2
4058	13608	9884	949	322	168	28044	1286	7	593	1	4	62	4	73	270.87	84	423.59	273	290	282	16	47	0	0	1
3954	13614	9876	960	376	236	28074	1288	8	554	1	16	52	6	71	270.71	85	423.2	286	271	286	10	37	0	2	4
11280	7962	5428	1	2028	1558	28628	535	85	568	26	336	165	39	278	315.91	123	462.61	494	174	366	98	84	3	34	19
14360	7672	4384	3	796	600	27874	641	313	1068	154	56	264	122	157	271.23	263	435.57	464	425	435	26	98	21	4	3
11392	7902	5280	0	2074	1616	28608	527	99	560	24	310	164	49	311	312.36	114	461.54	470	161	387	85	77	8	30	19
4734	13016	9428	741	600	346	28138	1185	2	634	0	14	61	1	90	279.24	99	433.64	347	330	293	14	38	0	0	2
6020	12238	8762	517	698	406	28156	1070	12	781	2	32	60	5	106	284.05	137	440.44	380	406	355	21	45	0	0	0
5712	12334	8814	569	766	510	28186	1065	27	664	8	46	69	14	102	282.13	115	437.3	401	327	324	22	33	0	4	4
13950	7806	4550	2	900	662	27934	646	285	1024	150	62	251	100	170	273.75	237	436.61	473	418	426	24	89	16	4	1
14228	7718	4402	9	850	648	27906	653	321	1026	160	54	250	126	160	271.7	259	433.18	474	444	443	18	72	21	6	1
4338	13280	9684	837	492	278	28096	1210	6	602	0	24	69	3	82	277.66	94	434.91	312	285	295	25	46	1	0	0
5794	12416	8834	558	650	422	28154	1092	16	732	1	36	79	8	116	283.75	116	439.87	373	395	310	18	42	0	2	0
13316	7634	4618	7	1346	1066	28166	580	258	802	99	154	191	124	211	282.31	174	446.03	493	314	452	46	70	9	32	4
14476	7646	4328	6	784	580	27856	652	343	1071	173	38	262	134	143	268.6	278	429.35	486	459	432	18	76	18	4	1
5310	12604	9114	592	692	406	28158	1066	7	692	1	32	69	5	93	280.19	108	436.97	400	354	345	18	51	0	0	4
12720	8124	5052	5	1232	894	28152	608	182	911	60	118	184	87	192	281.18	193	445.9	482	330	462	52	98	15	10	3
13542	7762	4578	5	1160	904	28064	642	259	910	126	106	236	100	178	276.95	221	440.55	492	352	403	35	89	17	10	4
11726	7844	5188	1	1974	1490	28504	566	117	618	24	250	154	62	276	300.07	114	453.73	457	214	404	93	80	6	30	9
4730	13088	9458	774	496	304	28106	1199	9	651	1	28	57	3	84	274.39	98	429.58	320	328	313	20	35	0	2	1
12824	7834	4924	1	1380	1048	28220	633	176	856	51	194	186	100	210	286.48	162	449.49	458	346	437	54	103	12	16	8
3842	13688	9960	992	342	208	28062	1322	8	551	2	22	53	5	78	270.92	79	422.83	256	260	270	14	42	0	0	1
4084	13608	9854	937	312	180	28048	1268	13	581	2	10	52	10	64	272.68	91	427.09	292	277	297	16	38	0	0	2
5968	12282	8800	520	696	388	28152	1054	11	770	1	18	63	6	93	284.24	125	440.8	412	398	347	19	47	0	0	1
13644	7630	4606	3	1182	868	28062	576	267	888	86	120	191	151	175	276.55	217	444.72	505	329	495	36	82	12	10	5
4398	13284	9634	806	496	286	28112	1210	6	604	0	14	45	5	92	277.42	89	431.77	314	307	323	9	31	0	0	3

SUPPLEMENTARY INFORMATION:Monte Carlo Atomistic Simulation and Machine Learning Analysis of Na-K Eutectic Alloy in Condensed Phases, D. Reitz and E. Blaisten-Barojas, George Mason University, Fairfax, VA 22030

14616	7624	4308	1	722	524	27834	690	339	1089	167	38	268	126	138	271.8	257	435.94	472	478	407	15	89	22	2	2
14302	7606	4376	9	880	662	27904	622	309	1027	163	74	264	107	156	272.83	268	437.85	494	395	431	26	91	25	4	0
14404	7678	4378	8	786	572	27862	659	334	1086	167	42	262	129	139	270.46	278	434.59	489	446	420	22	88	15	2	0
13160	7856	4724	7	1258	992	28146	647	201	912	71	142	196	93	188	281.91	194	447.34	456	363	438	56	97	17	14	5
4496	13260	9588	817	466	274	28098	1240	7	637	1	14	59	3	80	276.99	96	432.32	306	331	290	18	43	0	0	1
14278	7644	4378	4	862	662	27898	645	327	1057	169	64	271	124	155	270.19	263	433.12	485	439	416	23	91	17	10	1
4126	13550	9838	935	346	188	28058	1298	9	594	2	10	51	4	80	271.54	88	424.58	272	296	284	6	31	0	0	3
5970	12208	8704	525	762	496	28190	1058	18	690	1	46	74	13	120	286.15	81	442.83	365	390	345	27	48	0	4	2
5182	12756	9192	640	616	370	28146	1097	5	692	1	30	72	4	97	280.46	111	438.15	369	342	338	21	49	0	0	3
4126	13504	9822	893	386	216	28070	1255	8	583	0	16	64	5	63	275.58	100	429.15	302	282	286	22	40	0	0	2
13822	7592	4452	1	1182	892	28044	578	259	903	96	92	236	118	184	276.29	217	444.76	500	320	441	37	120	15	12	6
11552	7912	5360	1	1954	1434	28506	535	114	659	28	264	179	64	262	304.83	149	456.83	512	212	398	71	96	7	28	16
12110	8080	5074	3	1576	1292	28394	573	147	751	35	230	180	76	258	300.16	160	454.79	486	269	394	66	99	12	30	10
12558	7854	4962	2	1558	1154	28272	586	161	779	61	172	196	74	223	286.52	167	450.71	489	272	422	60	90	13	14	4
4126	13368	9736	852	534	332	28126	1236	7	566	0	30	55	4	88	273.51	86	430.32	299	284	291	22	35	0	0	2
14166	7798	4532	7	764	550	27868	632	300	1099	142	58	256	122	155	273.87	247	436.87	479	452	450	21	100	12	0	0
12200	7904	5150	1	1680	1222	28384	563	130	755	27	198	173	73	230	297.35	158	455.83	493	259	422	80	111	8	30	10
4792	13084	9438	730	482	286	28104	1135	5	660	1	22	51	4	80	277	102	433.84	347	324	360	21	35	0	0	2
14500	7668	4318	5	728	574	27852	636	332	1097	162	62	258	130	141	267.97	285	430.27	498	441	440	17	88	18	2	1
4382	13352	9668	861	426	244	28084	1245	4	623	0	12	56	1	69	274.25	106	429.86	315	296	293	15	35	0	0	2
4574	13116	9542	818	544	314	28116	1230	11	611	0	22	55	5	72	276.36	99	430.96	330	304	287	16	41	0	4	3
3996	13582	9890	947	376	208	28064	1302	7	580	1	12	52	5	70	271.29	88	423.84	268	290	284	17	37	0	0	2
11644	7888	5364	0	1906	1392	28500	538	107	634	15	280	170	68	264	316.13	121	459.05	479	197	402	84	103	6	22	21
6284	11930	8568	453	844	518	28208	1005	14	748	7	62	86	7	118	287.07	109	444.08	421	408	340	21	51	0	2	4
4544	13246	9596	810	428	246	28082	1215	9	639	1	20	66	5	77	276.39	99	432.19	321	326	298	13	48	0	2	4
5094	12788	9242	639	630	364	28138	1120	15	660	2	20	60	9	99	278.35	93	436.14	358	364	338	17	35	0	0	2
4286	13418	9708	887	410	244	28078	1273	11	616	2	12	60	5	70	272.11	96	426.22	302	304	269	16	39	0	0	1
7194	11484	8054	364	838	556	28192	940	33	836	10	66	85	18	130	286.53	143	443.41	423	413	389	22	52	1	0	5
4380	13296	9642	837	486	286	28108	1232	6	598	0	18	60	6	67	276.54	110	431.15	332	292	288	14	41	0	0	3
5430	12552	9030	594	686	426	28164	1072	10	685	2	38	71	6	88	282.69	109	437.46	411	358	329	19	46	0	2	3
5116	12846	9232	650	588	344	28138	1111	6	675	0	12	75	5	95	278.02	105	434.25	382	349	323	10	50	0	0	3
4122	13570	9838	926	328	182	28048	1298	8	606	2	8	66	5	70	272.1	86	425.64	273	318	274	14	38	0	0	2
4226	13484	9760	876	362	218	28068	1265	7	607	0	16	57	5	69	274.36	89	428.18	285	320	294	18	35	0	2	5
14328	7676	4414	3	796	590	27866	671	329	1087	163	56	258	128	155	267.47	256	429.71	472	466	423	12	88	17	6	2
11280	7966	5360	0	2068	1604	28624	517	89	581	16	298	166	48	277	312.81	128	457.67	519	183	367	100	90	6	44	16
3962	13624	9898	966	356	210	28066	1319	8	560	1	16	53	5	79	270.86	70	422.35	261	289	272	11	33	0	0	1

SUPPLEMENTARY INFORMATION:Monte Carlo Atomistic Simulation and Machine Learning Analysis of Na-K Eutectic Alloy in Condensed Phases, D. Reitz and E. Blaisten-Barojas, George Mason University, Fairfax, VA 22030

14250	7728	4426	4	804	612	27884	616	310	1058	155	60	266	121	155	272.53	274	437.15	502	410	429	18	103	15	2	3
11470	7900	5402	3	1974	1464	28542	548	106	652	22	304	169	46	264	311.7	125	458.64	481	219	370	93	106	6	28	24
4094	13544	9812	913	386	228	28072	1282	6	592	0	8	63	3	70	274.35	85	429.23	289	300	270	16	43	0	0	3
4310	13500	9748	879	304	174	28048	1220	5	649	1	12	66	2	72	273.62	111	427.91	318	294	303	12	47	0	0	2
14444	7748	4418	7	668	496	27822	654	326	1154	158	44	266	131	151	267.28	273	429.67	465	486	444	14	98	16	4	1
14448	7626	4342	5	788	600	27872	633	352	1051	165	62	254	149	158	267.45	264	429.54	476	419	452	19	79	17	4	1
6124	11964	8654	487	884	524	28208	1024	22	721	1	54	74	15	107	285.29	128	441.19	428	370	332	23	52	0	4	2
4890	12932	9366	693	596	334	28132	1149	1	665	0	14	61	1	92	277.92	107	435.01	361	341	318	14	35	0	0	0
13836	7808	4600	4	960	684	27950	647	292	991	132	58	228	123	152	274.47	232	438.62	482	400	447	32	87	16	4	1
11660	7860	5196	2	1984	1524	28534	533	122	590	33	290	181	69	268	309.34	118	459.54	496	192	389	83	90	6	20	20
7566	11034	7820	349	1036	684	28250	956	41	749	7	108	94	26	145	288.36	127	446.62	425	380	337	29	58	2	2	1
12182	7764	5068	4	1788	1346	28418	557	147	666	33	244	193	75	249	301.99	118	456.13	475	219	394	81	107	12	24	17
11760	7788	5270	1	1958	1440	28532	525	120	628	22	288	174	60	260	311.08	141	458.98	516	206	380	91	89	8	26	14
4280	13344	9726	869	476	252	28084	1242	8	593	0	6	52	5	72	273.9	94	427.01	301	280	301	21	39	0	0	2
4614	13214	9556	787	434	248	28084	1193	6	645	0	18	53	3	65	276	93	430.17	344	324	315	18	41	0	0	2
4480	13206	9608	787	510	286	28112	1209	6	614	0	22	57	1	79	276.9	98	433.85	335	327	294	14	38	0	0	3
13702	7942	4642	3	910	692	27960	622	252	1042	121	64	240	103	169	275.11	243	440.87	482	414	442	32	96	10	8	1
13616	7896	4744	3	952	668	27950	623	282	1031	134	66	242	112	167	276.72	248	439.73	471	404	449	36	89	18	8	1
4096	13532	9800	916	400	242	28080	1261	6	583	1	8	61	4	69	271.47	105	423.82	302	269	286	15	37	0	2	3
13452	7938	4690	4	1012	810	28024	612	252	969	112	116	228	103	184	276.61	230	443.31	496	368	440	27	94	17	6	1
4164	13536	9820	925	342	186	28058	1279	6	612	1	10	62	5	66	273.02	96	426.67	291	299	283	13	43	0	0	3
5130	12766	9236	691	624	356	28134	1149	13	667	4	20	62	6	91	278.98	112	436.06	343	336	321	24	41	0	2	2
11862	7968	5182	0	1842	1364	28444	546	120	679	33	202	179	60	264	303.63	122	455.29	486	237	400	81	97	6	24	11
4382	13374	9668	856	404	236	28074	1217	8	622	1	8	60	4	71	273.33	107	427.74	316	285	310	15	35	0	2	5
12828	7894	4864	2	1374	1072	28224	626	175	848	48	180	174	95	201	285.56	186	449.7	481	332	446	54	85	14	10	4
14236	7668	4410	3	876	650	27904	631	315	1037	147	62	260	118	160	273.06	267	436.59	489	411	427	20	97	26	2	3
4252	13344	9678	833	510	314	28118	1227	4	604	0	20	47	2	73	273.84	107	427.57	328	300	296	19	32	0	0	1
4778	13054	9442	728	528	296	28108	1154	5	649	0	8	67	3	90	275.46	86	431.95	346	327	323	13	46	0	2	3
12774	7920	4926	4	1354	1034	28188	632	185	863	62	168	192	98	197	283.59	176	448.57	470	336	447	47	95	14	10	5
4742	13102	9468	745	504	282	28108	1197	5	658	1	10	51	3	87	278.43	95	434	334	338	315	11	35	0	0	0
4552	13196	9588	815	470	264	28094	1221	4	641	0	22	60	2	79	274.82	96	430.35	310	320	298	21	44	0	2	2
4032	13590	9874	944	346	198	28054	1274	9	582	1	14	59	2	65	270.42	91	423.71	294	275	284	14	45	0	0	3
14462	7684	4358	9	724	558	27848	633	323	1109	163	58	266	129	147	270.84	273	433.67	480	440	447	20	94	18	4	2
5316	12510	9114	625	752	434	28170	1079	13	615	2	38	72	9	106	284.81	97	439.39	370	311	326	24	43	0	6	8
6416	11790	8438	434	938	588	28226	992	25	697	4	52	77	13	127	286.24	93	442.33	409	368	347	27	45	1	2	7
11534	7768	5304	0	2086	1548	28572	533	116	574	35	298	161	43	280	311.41	105	460.29	462	194	409	94	78	9	32	24

SUPPLEMENTARY INFORMATION:Monte Carlo Atomistic Simulation and Machine Learning Analysis of Na-K Eutectic Alloy in Condensed Phases, D. Reitz and E. Blaisten-Barojas, George Mason University, Fairfax, VA 22030

4248	13378	9738	866	456	254	28088	1242	7	583	2	12	51	3	70	275.68	86	429.36	316	287	297	15	34	0	2	2
13556	7634	4566	1	1274	954	28114	601	237	862	90	116	196	118	191	277.24	167	444.08	486	346	473	38	89	19	12	6
3780	13780	10004	998	304	176	28050	1340	6	549	2	6	56	2	71	270.27	73	423.11	253	281	263	10	36	0	0	3
4148	13622	9830	916	264	164	28046	1265	4	613	0	18	59	2	60	271.03	92	423.91	307	301	290	13	39	0	0	1
4138	13552	9802	933	348	210	28062	1290	11	582	1	12	56	9	72	272.51	82	426.83	273	297	286	14	40	0	0	4
10980	7646	5556	1	2350	1760	28774	503	88	468	16	426	150	47	269	331.99	90	470.47	448	151	386	139	61	3	54	45
7400	11076	7814	339	1116	766	28274	890	49	722	14	96	96	26	161	287.94	116	445.87	442	353	364	30	55	1	6	4
4970	12916	9352	703	552	306	28116	1140	6	681	0	20	62	4	93	277.4	97	434.94	352	344	332	15	43	0	0	1
4460	13306	9642	846	412	236	28076	1258	8	633	0	20	62	4	69	274.13	108	428.81	318	325	275	11	48	0	0	1
13928	7668	4440	2	1074	798	27970	612	305	948	147	58	249	115	169	272.78	235	436.09	502	366	437	24	85	21	4	2
4424	13378	9664	853	370	218	28072	1223	8	631	0	18	63	4	58	274.07	108	428.46	345	298	289	13	47	0	0	2
13744	7836	4586	4	966	752	27982	652	254	1047	123	90	225	107	168	275.52	258	439.4	457	412	448	35	83	13	6	4
14312	7654	4358	6	864	652	27898	604	320	1001	147	54	249	140	169	271.96	252	436.33	480	379	470	21	89	16	4	1
12830	7936	4888	4	1354	1022	28184	593	181	854	58	146	181	93	198	280.94	194	445.7	486	310	459	58	90	8	6	4
4680	13174	9512	790	458	258	28092	1211	5	658	0	10	54	3	76	276.3	95	432.07	314	333	323	17	42	0	0	3
14240	7746	4450	6	794	590	27876	645	320	1084	155	52	258	126	157	270.59	261	433.42	475	432	436	20	91	18	4	1
11994	7804	5144	3	1860	1392	28474	562	133	678	31	244	170	77	251	303.97	133	455.03	493	246	410	75	94	6	34	14
14164	7646	4470	7	916	650	27914	626	323	984	158	66	260	121	158	271.22	241	436.48	499	392	420	25	88	18	2	0
14326	7642	4334	9	874	668	27902	629	300	1032	167	50	268	91	155	270.21	266	434.84	512	395	414	16	86	22	8	0
11884	7950	5244	0	1788	1324	28454	555	123	659	27	240	172	71	236	303.13	129	459.11	505	224	408	74	95	6	20	23
14406	7632	4368	6	806	598	27874	644	324	1072	173	62	268	116	135	269.8	286	431.22	513	432	416	18	85	20	2	1
13704	7876	4572	5	968	772	27988	624	261	1007	130	92	239	91	184	276.08	231	438.5	489	397	428	23	90	19	4	1
6138	12068	8646	489	790	486	28174	998	22	753	5	40	77	15	114	286.69	118	442.44	395	380	376	30	43	1	6	2
4742	13136	9480	772	460	258	28084	1192	6	680	0	8	54	5	78	275.05	106	429.89	325	343	327	16	45	0	0	2
14044	7810	4488	6	842	654	27906	644	285	1040	149	64	250	106	153	271.27	272	433.54	489	415	432	22	86	17	4	1
13724	7696	4518	2	1146	860	28040	614	254	945	123	94	250	108	181	275.35	235	441.72	512	365	411	23	104	13	2	5
14200	7716	4430	4	856	640	27898	634	316	1030	155	46	262	124	155	271.16	268	434.46	494	423	422	21	91	17	8	2
5810	12304	8850	571	738	424	28148	1085	20	711	2	22	67	11	107	282.16	108	440.14	372	374	331	24	48	0	0	0
14368	7558	4352	3	884	660	27902	607	346	993	158	74	260	153	185	274.6	244	437.13	468	398	453	15	81	15	4	4
4492	13312	9660	813	384	200	28062	1193	10	641	2	14	62	7	75	273.83	93	429.06	322	323	324	16	43	0	0	2
4050	13564	9868	948	372	202	28066	1292	6	582	0	10	50	5	66	271.91	80	424.86	280	285	293	15	35	0	0	2
14224	7800	4448	5	760	584	27872	618	305	1075	163	56	259	101	155	270.63	263	433.41	485	414	458	19	85	20	0	2
4040	13588	9844	917	360	220	28066	1276	8	593	1	14	59	4	63	271.52	89	425.47	290	298	284	18	42	0	0	4
4638	13086	9506	766	548	314	28114	1192	6	639	1	22	59	4	79	275.13	95	430.05	337	320	310	17	39	0	0	2
4304	13294	9680	834	524	296	28112	1231	4	570	0	14	54	3	94	275.7	81	432.4	290	301	307	19	33	0	0	1
14368	7656	4348	7	820	628	27882	651	341	1044	166	58	251	134	153	269.29	258	432.4	484	448	437	16	71	20	4	1

SUPPLEMENTARY INFORMATION:Monte Carlo Atomistic Simulation and Machine Learning Analysis of Na-K Eutectic Alloy in Condensed Phases, D. Reitz and E. Blaisten-Barojas, George Mason University, Fairfax, VA 22030

14144	7684	4426	5	910	686	27920	636	294	1058	152	66	259	116	155	268.84	266	430.69	505	418	419	19	97	12	4	1
5042	12774	9276	658	646	368	28132	1135	11	644	1	26	68	6	88	280.7	82	438.11	345	355	320	31	50	0	0	2
4010	13624	9854	941	346	220	28070	1282	8	572	1	16	62	4	67	271.09	93	423.82	296	273	272	13	40	0	0	3
6170	12108	8664	531	712	438	28140	1057	33	738	5	44	72	20	116	287.13	99	444.26	372	402	349	18	50	0	4	2
4032	13598	9862	934	348	208	28064	1297	7	586	1	16	49	4	72	270.89	90	423.75	277	288	282	13	34	0	0	2
12400	7870	4924	0	1622	1296	28350	583	154	751	42	204	164	79	223	293.02	153	450.14	523	277	425	53	92	10	30	9
5202	12868	9188	626	514	328	28122	1058	8	706	1	18	70	4	103	279.66	114	437.05	382	340	365	14	45	0	4	3
4518	13214	9598	798	492	266	28098	1174	6	598	1	10	66	2	93	276.71	77	433.95	319	296	318	20	50	0	0	2
4652	13146	9538	789	478	260	28088	1211	9	648	1	12	61	7	83	276.42	100	429.41	318	334	303	11	45	0	2	5
12494	7900	5030	2	1528	1134	28290	601	170	772	42	184	171	95	241	290.88	156	451.8	473	297	426	50	97	11	20	10
4368	13404	9704	881	368	204	28058	1256	8	638	2	10	67	5	68	272.12	106	427.04	297	306	289	16	49	0	0	3
5028	12968	9328	688	484	278	28102	1111	4	696	1	14	74	2	95	278.56	122	434.18	364	328	337	15	54	0	2	0
14454	7648	4380	4	764	552	27848	640	336	1058	170	44	270	124	141	268.81	272	433.17	488	441	428	23	90	19	6	1
11872	8048	5158	1	1748	1356	28422	570	136	683	32	216	157	72	274	300.63	136	454.93	461	228	410	72	81	5	20	10
5028	12886	9330	698	534	304	28114	1162	6	690	0	30	62	3	75	279.51	110	435.03	358	354	316	20	46	0	2	1
5382	12668	9106	612	594	356	28138	1084	13	711	2	28	62	5	102	280.53	104	436.89	382	371	350	12	38	1	4	2
6654	11612	8308	386	966	620	28240	944	24	734	2	76	73	18	149	287.48	129	444.1	418	375	371	26	49	0	4	3
13620	7708	4552	1	1180	906	28086	654	246	894	114	100	220	94	177	278.24	199	444.78	491	382	401	39	86	17	18	3
4578	13264	9546	800	420	262	28088	1186	7	622	1	18	55	3	81	274.14	94	430.08	332	299	325	13	34	0	0	2
4658	13076	9488	773	560	324	28128	1219	5	615	0	20	65	2	90	279.11	94	434.33	324	334	279	16	43	1	2	1
14296	7624	4408	8	852	632	27886	666	323	1044	174	66	266	118	152	269.67	255	432.21	478	450	412	21	79	11	8	0
5402	12626	9066	617	626	390	28148	1104	12	674	1	36	63	8	92	281.79	105	437.43	378	343	333	19	42	0	2	3
11148	7950	5494	1	2110	1566	28612	498	102	599	20	308	164	60	283	313.61	121	459.72	514	198	391	90	95	3	36	21
4646	13212	9528	793	428	254	28086	1165	11	638	1	18	65	7	90	274.84	103	430.73	340	296	318	10	43	0	0	3
13512	7880	4736	7	1020	752	28004	618	251	989	106	92	234	116	162	276.54	230	443.71	489	392	446	36	105	13	12	5
3980	13692	9934	964	270	146	28030	1316	7	608	1	8	63	4	72	270.1	90	424.09	258	303	270	11	42	0	0	3
4080	13634	9882	963	282	150	28034	1301	8	598	1	6	59	4	73	270.63	82	423.12	272	287	278	8	37	1	0	1
6486	11792	8420	416	892	570	28226	981	17	758	2	54	81	10	138	288.57	125	446.13	419	390	351	20	52	0	12	2
6460	11792	8430	464	870	576	28214	1000	27	714	5	80	85	13	119	289.12	121	446.51	422	374	331	25	49	3	6	3
3972	13670	9932	974	300	160	28042	1315	7	578	1	8	61	4	76	271.17	89	424.07	263	292	269	11	40	0	0	0
4018	13528	9854	929	418	242	28076	1260	10	552	1	14	61	6	82	270.49	93	423.84	293	269	280	12	37	0	2	3
6582	11756	8332	410	892	596	28226	966	29	735	1	62	87	22	134	286.14	116	443.87	417	357	361	29	62	2	6	1
4670	12920	9444	716	694	404	28168	1169	5	591	0	32	57	3	104	280.16	74	437.01	343	313	310	13	44	0	4	2
6060	12108	8688	471	806	482	28182	1013	15	747	3	38	73	9	118	285.4	114	441.92	409	394	353	21	50	0	0	2
4848	13054	9420	727	486	276	28098	1169	9	674	0	12	65	6	78	278.38	106	435.27	347	354	316	17	45	0	2	2
10594	7676	5702	1	2494	1868	28848	516	66	436	10	446	167	35	289	327.7	83	472.59	474	127	328	120	80	2	56	50

SUPPLEMENTARY INFORMATION:Monte Carlo Atomistic Simulation and Machine Learning Analysis of Na-K Eutectic Alloy in Condensed Phases, D. Reitz and E. Blaisten-Barojas, George Mason University, Fairfax, VA 22030

13860	7782	4554	3	942	732	27978	593	263	1021	111	102	229	126	194	277.73	245	441.23	461	367	481	28	99	11	6	4
4546	13306	9622	811	378	200	28058	1212	10	676	1	6	66	6	79	274.61	108	428.17	315	340	307	11	43	0	0	3
4218	13464	9780	879	390	208	28066	1239	6	597	1	6	62	3	69	273.55	83	427.71	319	289	293	11	41	0	0	2
14220	7644	4404	5	900	674	27914	637	312	1048	155	68	256	123	163	272.92	264	436.55	481	425	438	22	85	18	2	0
4824	12902	9372	696	654	378	28154	1136	1	631	0	24	49	1	73	279.32	101	434.51	372	318	335	28	38	0	0	1
4960	12878	9304	674	618	362	28140	1150	10	661	4	18	56	4	87	278.74	98	435.6	360	357	323	14	33	0	0	3
3994	13632	9898	935	334	188	28056	1267	7	560	1	10	58	3	73	270.67	84	423.23	290	256	290	13	43	0	0	2
14316	7660	4412	11	820	604	27876	640	318	1072	153	60	267	124	155	270.89	265	435.8	480	446	430	20	103	23	4	2
4396	13360	9668	853	410	230	28070	1215	11	630	1	6	54	5	77	273.27	105	428.15	308	290	319	17	36	0	0	2
4582	13232	9588	813	422	232	28072	1215	10	652	1	16	68	4	71	275.14	110	427.93	322	323	296	17	48	0	0	2
14158	7738	4426	9	840	666	27910	641	295	1045	139	78	250	124	169	270.76	265	433.33	487	415	433	13	98	17	4	0
5818	12392	8834	562	656	416	28154	1048	14	747	4	36	68	7	97	282.57	131	439.69	411	377	352	16	45	0	2	2
4828	13104	9412	731	446	284	28102	1157	6	682	0	26	52	6	89	276.38	102	432.35	332	346	346	18	32	0	2	1
4824	13134	9446	723	430	246	28092	1174	8	686	0	12	49	6	81	277.92	99	432.81	344	375	334	11	34	0	0	1
4654	13138	9488	755	510	308	28120	1181	4	654	1	22	54	3	73	279.43	99	435.13	349	336	316	17	32	0	0	4
12850	8004	4948	5	1270	940	28150	636	177	923	56	118	185	88	192	283.57	210	446.35	485	337	441	40	98	8	20	5
12236	7872	5142	3	1654	1206	28336	573	130	784	40	196	193	58	274	298.12	137	452.33	462	274	404	54	113	9	28	11
4188	13586	9802	909	288	172	28046	1270	7	620	2	10	63	4	69	272.78	98	425.48	289	306	290	13	41	0	0	2
14216	7766	4412	6	812	630	27894	626	324	1023	155	56	244	127	156	269.54	255	432.25	502	413	449	12	77	18	2	2
11992	7784	5120	5	1878	1410	28462	584	127	633	43	254	176	49	255	303.06	128	453.03	480	234	374	82	94	6	20	12
14440	7670	4342	3	744	590	27858	649	328	1098	155	66	250	134	156	269.46	268	431.91	465	448	456	17	88	15	6	1
6590	11788	8420	453	832	502	28184	1000	30	760	7	52	75	16	97	287.85	140	444.19	436	380	355	26	44	1	0	2
14504	7516	4298	3	862	636	27886	636	318	1035	162	64	264	119	151	271.62	258	437.01	497	419	425	18	89	13	6	2
13856	7684	4544	3	1058	764	27992	611	281	941	127	80	240	122	194	277.3	229	443.85	464	371	438	34	93	9	6	1
4048	13560	9856	925	380	216	28074	1273	7	579	1	14	52	5	66	272.42	88	425.85	310	281	282	8	31	0	0	3
4634	13188	9530	804	460	262	28084	1201	11	629	1	10	61	5	88	276.19	88	430.78	307	314	320	14	41	0	0	4
4550	13272	9564	805	408	264	28086	1214	8	649	1	22	68	4	72	274.12	97	428.85	321	328	296	21	49	0	6	2
5260	12670	9148	607	656	388	28154	1101	10	667	0	32	52	6	87	281.74	109	437.54	397	360	335	14	33	1	0	2
4232	13464	9776	906	362	206	28060	1255	10	597	1	20	59	6	67	272.9	90	426.26	304	285	293	13	44	0	0	3
14424	7648	4340	6	788	604	27868	673	329	1065	163	64	264	126	146	272.32	270	436.39	485	442	412	14	88	19	0	1
12234	7900	5108	1	1674	1220	28344	530	155	731	31	190	179	86	257	297.76	137	454.44	486	244	444	66	93	11	16	8
14248	7690	4400	4	868	644	27906	642	313	1023	166	56	251	119	144	267.67	261	429.58	505	423	434	19	77	13	0	1
13914	7738	4498	8	992	748	27970	631	274	983	121	78	246	121	175	277.11	237	442.78	470	378	428	32	95	15	2	4
4160	13444	9778	893	440	248	28080	1257	4	568	0	10	55	2	71	273.63	81	428.25	299	286	293	15	35	0	0	4
14298	7622	4398	9	874	634	27884	618	331	1032	155	52	261	131	148	271.53	265	437.31	493	385	446	24	94	23	6	3
10756	7836	5652	0	2330	1758	28796	539	68	507	7	394	156	36	306	328.04	94	468.59	466	164	333	110	80	3	64	24

13682	7838	4650	0	988	742	28008	622	247	995	93	104	210	115	172	276.44	225	444.87	485	399	464	27	102	20	4	6
11310	7766	5366	1	2142	1658	28652	537	94	544	17	364	171	47	267	310.9	98	460.22	479	165	369	112	93	10	40	26
13942	7734	4518	6	942	722	27956	664	298	983	131	92	239	131	176	276.81	227	440.07	444	408	432	33	83	15	6	1
11494	7964	5244	0	1962	1554	28542	550	99	636	19	290	166	56	265	306.58	131	457.38	485	222	396	92	90	7	34	17
14312	7666	4358	3	830	652	27894	649	313	1056	158	74	258	113	157	270.81	261	433.47	481	426	429	18	92	24	2	2
14334	7732	4452	8	748	534	27846	633	335	1085	162	46	267	132	155	270.62	269	434.32	473	423	449	18	96	21	0	1
10780	7820	5672	1	2306	1706	28728	485	94	487	14	398	157	47	310	327.18	95	465.71	483	147	365	116	84	8	44	24
4758	13076	9454	733	510	292	28106	1168	6	661	1	16	57	3	85	276.36	110	431.52	346	336	313	15	34	0	0	3
6800	11594	8276	414	904	566	28210	967	33	730	7	64	96	20	148	287.69	102	446.53	411	390	352	15	67	2	6	4
14492	7618	4382	2	762	538	27844	611	329	1067	159	48	261	133	142	271.21	275	434.77	494	414	456	23	89	16	4	2
14282	7662	4366	5	874	662	27906	650	304	1045	156	54	260	115	151	271.97	264	435.3	487	424	421	24	90	17	6	1
4330	13440	9718	894	356	202	28054	1278	8	622	1	8	62	4	82	273.06	88	425.44	262	311	290	19	43	0	0	1
12086	7828	5178	2	1818	1278	28402	606	136	710	30	186	155	67	210	298.94	137	457.56	498	264	404	78	79	10	26	15
14132	7642	4436	2	954	704	27946	657	302	989	150	74	251	111	162	273.59	228	436.86	493	423	399	23	87	15	2	1
13792	7822	4598	5	952	712	27962	615	284	1031	141	86	254	109	177	274.71	237	438.89	477	416	433	27	100	9	0	6
14268	7676	4364	6	860	672	27914	629	311	1020	155	70	244	119	166	270.92	259	432.91	489	405	451	16	81	21	4	0
3880	13684	9986	997	326	162	28044	1331	5	569	0	6	72	5	61	271.64	89	424.36	263	276	250	18	51	0	0	2
5674	12286	8876	603	804	496	28190	1100	25	623	6	52	78	14	101	285.48	86	441.13	370	337	311	28	48	1	2	4
11326	8092	5418	3	1898	1476	28544	536	98	633	23	312	173	50	291	307.3	134	455.99	499	201	376	68	95	6	20	19
14260	7696	4414	8	836	626	27894	655	332	1040	157	56	255	135	159	270	268	435.03	471	438	428	22	82	23	6	1
14476	7634	4272	4	798	634	27876	661	323	1037	166	62	268	115	165	271.51	250	434.99	462	418	419	18	91	25	0	1
4102	13568	9834	935	348	198	28058	1291	8	589	1	8	62	5	71	271.9	97	424.9	279	301	274	15	42	0	0	2
4082	13550	9822	906	370	228	28072	1259	4	587	0	20	59	2	75	274.57	93	429.06	294	287	291	10	42	0	0	5
3844	13638	9962	974	394	218	28070	1310	10	533	1	14	56	5	67	271.1	77	423.57	276	269	261	17	41	0	0	3
5972	12120	8696	567	804	534	28202	1057	22	635	1	68	59	15	102	286.26	95	441.92	404	334	340	24	40	0	6	2
3930	13656	9914	960	346	204	28062	1288	7	551	1	12	56	5	77	270.05	72	423.59	268	269	290	14	27	0	0	3
4312	13418	9746	889	380	196	28058	1245	6	609	1	6	62	3	71	273.68	96	427.28	309	287	293	14	40	0	0	0
7496	11108	7822	317	1044	698	28258	894	42	773	11	82	85	24	166	288.02	118	444.55	421	398	390	27	49	2	6	5
13562	7930	4726	6	1000	708	27992	635	251	997	112	60	237	108	169	276.5	240	442.93	484	386	437	29	103	21	6	3
5192	12846	9226	630	534	306	28118	1119	6	730	1	14	60	3	98	277.35	110	434.11	346	380	350	19	36	0	0	2
11598	8056	5234	0	1852	1470	28510	552	103	652	28	276	192	54	278	311.71	133	461.18	455	213	390	89	95	7	24	18
12072	7898	5126	1	1726	1320	28408	573	134	717	37	242	178	58	217	301.09	175	455.06	530	248	380	77	92	8	22	12
4932	13024	9382	717	458	270	28090	1133	10	695	2	24	64	3	93	276.97	119	431.91	351	336	331	14	43	0	0	1
6838	11508	8144	394	998	676	28240	958	33	717	10	72	97	18	127	286.08	112	446.57	426	371	341	32	50	1	4	7
4486	13212	9580	798	506	306	28118	1225	5	602	0	26	60	3	76	275.96	79	432.93	319	325	289	19	37	0	2	5
7280	11158	7966	364	1030	690	28246	915	49	713	19	114	86	23	144	289.69	114	444.92	422	369	388	27	34	3	8	10

SUPPLEMENTARY INFORMATION:Monte Carlo Atomistic Simulation and Machine Learning Analysis of Na-K Eutectic Alloy in Condensed Phases, D. Reitz and E. Blaisten-Barojas, George Mason University, Fairfax, VA 22030

4078	13616	9872	928	310	164	28042	1266	7	602	0	2	61	4	74	271.44	87	425.05	284	288	293	14	38	0	0	1
5730	12372	8904	563	702	414	28160	1059	17	716	4	36	63	11	110	282.7	115	438.75	378	361	359	21	35	0	2	2
6756	11706	8324	395	840	514	28190	961	18	808	2	46	74	12	124	289.2	137	444.57	437	400	378	17	48	0	2	1
5220	12708	9178	632	642	374	28150	1115	6	693	0	28	64	3	108	280.63	96	437.07	357	351	334	14	40	1	0	3
12582	7814	4828	3	1582	1280	28316	610	169	743	51	194	188	76	231	290.56	155	451.58	500	276	374	52	91	15	36	11
14392	7686	4350	3	780	604	27872	638	336	1064	158	56	250	137	141	270.67	271	435.89	497	446	435	23	81	20	4	1
13086	7968	4880	6	1128	872	28098	606	232	928	81	158	201	116	217	283.2	190	446.9	439	349	473	41	96	13	6	7
6832	11652	8272	360	852	532	28200	919	31	792	8	60	81	19	122	286.05	142	443.41	443	400	391	26	46	2	0	6
10904	7900	5688	1	2168	1590	28666	519	86	538	22	372	165	36	281	316.4	103	462.51	483	162	366	105	85	3	38	28
4642	13182	9492	760	488	296	28112	1164	1	652	0	10	62	0	85	278.48	106	433.61	345	313	324	14	37	0	2	3
3980	13744	9936	987	232	128	28022	1321	6	615	1	2	57	5	66	270.5	88	422.88	256	287	279	14	42	0	0	1
10726	7654	5580	0	2482	1892	28848	494	67	438	9	466	154	37	329	328.39	62	472.65	441	149	363	124	76	1	44	44
3846	13694	9956	986	360	204	28064	1320	5	545	1	2	65	3	64	270.78	81	422.72	280	271	253	12	39	0	2	3
5528	12456	8942	607	726	478	28188	1132	12	676	1	54	65	11	101	286.68	105	443.61	360	369	313	26	45	0	4	0
3976	13648	9912	960	322	182	28050	1313	7	587	1	8	53	4	68	272.53	80	424.23	278	294	275	7	32	0	0	2
3970	13668	9928	976	306	164	28042	1296	5	582	1	6	68	4	65	270.08	104	422.03	275	262	276	17	48	0	0	1
6038	12124	8682	518	792	498	28184	1032	19	728	4	46	70	13	115	287.51	97	442.56	399	367	342	25	45	0	4	2
13182	7698	4692	2	1402	1052	28182	601	204	833	78	148	207	97	220	284.01	177	446.75	473	309	430	51	95	9	8	2
4276	13432	9768	887	376	198	28064	1256	9	621	2	12	67	5	80	273.05	101	426.17	305	301	278	9	53	0	2	0
13166	7750	4740	2	1328	1008	28156	611	215	819	65	150	202	118	198	282.93	178	447.19	481	318	444	44	104	18	14	8
4590	13180	9538	770	486	292	28114	1199	3	651	0	28	59	2	84	278.21	99	433.66	330	331	304	17	43	0	0	0
4664	13096	9486	757	534	318	28126	1172	6	622	0	28	50	4	82	278.4	99	433.97	339	303	331	22	34	0	0	0
4628	13184	9502	766	486	294	28108	1184	4	652	1	14	60	0	83	276.9	109	433.07	335	328	313	18	35	0	0	1
12000	8146	5284	3	1566	1140	28338	582	121	785	33	184	173	58	236	297.35	177	451.14	508	279	403	51	103	8	18	13
11276	7854	5458	0	2132	1552	28614	509	86	577	14	310	163	44	278	316.1	122	460.54	500	169	396	95	100	8	32	21
5474	12498	8982	589	742	456	28184	1092	7	672	0	32	50	5	89	281.37	102	437.52	408	349	335	14	32	0	0	4
4422	13306	9650	842	436	250	28082	1250	11	627	1	18	61	7	81	274.55	93	427.99	299	329	284	16	43	0	0	1
5614	12494	8942	568	678	412	28166	1104	13	752	0	22	54	6	100	281.56	116	436.81	386	413	329	14	32	0	4	2
4844	13100	9464	725	422	230	28080	1146	3	697	0	20	78	2	81	278.6	101	435.73	350	349	319	19	55	0	0	1
7280	11318	7988	342	964	612	28226	934	32	799	6	64	80	21	135	287.86	123	446.5	434	408	380	23	42	0	0	4
5328	12696	9142	650	600	344	28132	1112	13	685	2	22	74	9	94	279.66	94	436.66	361	349	328	22	51	0	0	3
11488	7996	5436	2	1888	1402	28536	542	87	638	15	304	161	49	270	312.31	140	459.55	525	212	381	72	100	5	18	14
4146	13492	9826	903	394	200	28060	1265	10	597	1	2	50	6	68	272.11	90	425.1	284	294	303	21	35	0	0	1
13774	7748	4560	1	1026	782	27990	622	289	955	127	90	235	121	183	275.31	228	441.9	437	379	465	40	88	24	6	8
14254	7644	4420	5	860	646	27908	640	317	1025	157	80	247	123	163	271.53	255	435.35	475	422	446	20	75	18	4	2
4154	13524	9834	933	344	182	28050	1297	10	606	1	12	56	6	70	271.02	89	423.52	272	303	284	15	43	0	0	1

4542	13174	9582	764	514	282	28112	1182	2	622	0	16	64	2	82	277.11	91	434.08	336	328	313	18	40	0	2	2
4456	13314	9628	818	426	246	28082	1184	8	608	1	12	62	4	78	275.74	88	431.04	337	307	319	13	47	0	0	2
12386	7688	5052	1	1752	1246	28356	555	153	732	44	220	198	75	217	300.04	166	452.52	520	245	390	76	110	8	12	10
4782	13128	9462	770	444	258	28092	1163	6	660	1	18	64	4	82	276.32	104	433.67	339	315	326	19	44	0	0	1
13818	7720	4556	6	996	776	27994	616	283	967	127	122	248	110	190	276.77	230	440.33	479	368	428	28	103	26	6	0
4544	13320	9608	808	374	210	28064	1206	7	658	1	8	71	4	80	273.56	115	428.85	325	325	304	10	47	0	0	1
13076	7934	4778	2	1268	962	28138	605	210	890	72	110	191	98	195	283.84	203	449.78	489	339	448	45	101	14	10	5
4926	12950	9366	713	550	308	28116	1145	2	689	0	16	69	0	98	278.93	109	435.3	347	349	316	17	46	0	0	0
12882	7740	4836	1	1520	1106	28250	582	185	775	51	148	183	102	221	289.41	166	451.22	504	275	431	51	101	9	16	5
4878	12916	9356	692	604	358	28142	1134	3	652	0	30	62	3	110	279.03	98	435.07	339	335	331	16	34	0	0	2
5032	12866	9308	704	570	322	28120	1172	14	676	5	22	74	6	85	279.75	96	436.64	339	356	296	25	46	0	0	1
7136	11338	8060	298	1010	638	28260	933	18	828	3	74	72	11	132	288.25	135	445.58	448	441	373	26	41	0	4	5
4892	12922	9366	703	594	338	28132	1153	6	660	2	16	67	2	92	278.97	93	433.79	346	347	314	19	46	0	4	3
13698	7872	4616	3	964	740	27978	625	275	991	123	78	240	120	173	274.91	225	440.43	480	399	444	28	99	13	10	2
5280	12718	9162	631	612	348	28138	1096	5	696	0	18	61	4	101	278.19	104	433.17	385	356	345	8	40	0	0	1
4000	13636	9910	973	324	172	28048	1300	8	572	1	6	54	4	69	270.97	89	423.58	268	274	283	15	38	0	0	3
13084	7696	4668	3	1424	1140	28210	599	205	791	74	180	198	94	206	284.38	183	446.6	496	280	416	53	99	9	16	8
4388	13336	9672	843	440	240	28082	1228	9	599	1	6	60	5	80	274.32	89	429.49	316	300	297	11	43	0	0	2
5458	12548	9018	596	694	418	28162	1073	9	672	1	24	72	6	90	281.64	114	438.01	398	338	340	19	45	0	2	5
5908	12206	8760	518	782	488	28194	1014	16	723	1	40	72	12	107	281.84	119	441.03	424	360	356	17	46	2	10	5
4014	13574	9850	918	396	234	28078	1277	4	557	1	10	61	3	69	272.53	78	425.57	285	284	283	19	38	0	0	3
5162	12780	9220	669	600	348	28132	1118	12	675	1	20	60	9	92	280.98	108	437.18	373	335	334	14	36	0	2	2
7864	10920	7634	281	1052	690	28234	848	56	771	16	72	105	28	159	288.01	134	444.68	448	360	390	33	60	2	2	3
13350	7692	4660	2	1306	978	28128	601	236	830	86	128	207	105	210	283.62	173	446.27	484	321	432	40	95	17	14	3
12916	7894	4828	4	1332	1038	28174	589	219	838	72	146	181	113	185	281.99	193	445.41	500	295	470	51	87	15	18	8
13930	7714	4472	1	1006	768	27974	644	273	975	119	80	235	114	156	275.05	227	439.49	482	380	431	36	103	23	4	3
4668	13086	9456	726	546	346	28138	1170	3	643	0	36	58	2	83	277.96	98	433.67	355	339	309	16	31	0	0	3
13272	7724	4592	2	1346	1074	28170	604	227	804	76	154	198	113	231	281.43	167	445.54	464	299	448	40	89	20	8	2
4480	13298	9640	817	428	228	28082	1214	3	638	0	8	61	2	77	277.44	99	432.76	310	321	313	19	35	0	0	2
11182	7898	5446	1	2144	1608	28638	520	78	549	14	330	166	41	273	317.12	106	462	488	183	371	111	83	5	26	21
14380	7630	4338	5	848	636	27888	610	329	1031	166	52	272	121	169	271.26	265	435.75	495	395	432	15	98	19	4	1
4334	13456	9714	898	340	202	28060	1261	7	629	2	14	58	4	58	273.37	105	425.55	310	303	292	14	37	0	0	3
4646	13152	9548	747	496	254	28100	1173	7	636	1	4	58	3	85	277.37	98	433.69	340	327	321	16	41	0	0	0
3908	13676	9926	972	336	200	28058	1292	9	549	1	12	53	4	65	270.57	88	422.93	298	257	269	10	36	0	0	2
4442	13260	9602	819	484	294	28104	1212	11	605	1	22	55	8	68	276.52	107	430.01	338	298	302	16	35	0	0	3
14774	7480	4252	4	744	520	27816	626	371	1075	176	44	289	148	148	271.35	275	434.23	492	430	424	12	99	23	2	3

4430	13310	9608	826	446	280	28094	1221	8	609	2	20	57	4	82	273.71	92	427.59	312	310	304	17	35	0	0	1
14438	7576	4350	3	854	606	27874	654	340	1036	168	48	254	132	146	270.44	261	433.47	479	438	439	20	77	21	2	2
12468	7778	5010	1	1652	1204	28324	577	169	731	44	194	192	83	217	294.39	149	454.11	482	260	417	81	98	17	14	10
12968	7916	4786	4	1288	1038	28170	586	203	858	84	156	206	91	202	283.25	202	444.6	511	312	432	46	90	13	18	2
4718	13224	9506	780	388	228	28076	1216	7	666	0	10	47	5	79	276.17	91	430.83	315	353	320	11	28	1	2	3
14496	7628	4302	7	782	598	27862	636	342	1061	177	56	255	122	148	267.84	271	430.12	493	435	446	16	70	16	0	0
13684	7720	4590	9	1100	824	28030	633	266	937	129	100	231	93	197	277.35	208	444.23	477	355	427	22	88	20	12	2
3938	13648	9936	981	340	182	28052	1293	7	567	1	8	65	4	67	270.26	97	423.45	295	258	266	9	43	0	0	1
14284	7704	4418	6	814	606	27886	650	314	1060	156	56	266	128	147	269.27	261	431.46	499	442	415	17	98	16	4	1
13386	7862	4694	5	1144	858	28040	618	253	950	116	86	223	106	171	276.27	225	440.15	500	389	442	35	88	10	8	2
3782	13828	10024	1025	246	144	28032	1332	9	567	1	8	56	6	62	270.46	84	422.24	261	274	267	13	40	0	0	2
14484	7568	4280	3	844	644	27886	648	327	1043	159	66	251	119	155	269.78	267	430.3	480	431	441	19	82	22	0	0
13494	7870	4656	4	1060	838	28046	633	261	941	128	124	228	101	187	277.08	212	442.53	483	386	425	28	79	12	4	5
14460	7618	4296	3	822	630	27884	628	332	1043	160	54	254	124	148	271.86	276	436.39	504	409	441	17	75	28	4	1
3864	13722	9954	978	314	190	28056	1316	8	576	1	8	61	4	69	271.58	86	424.04	263	276	266	15	40	0	4	4
12420	7876	5056	3	1598	1164	28314	601	146	770	33	178	173	74	230	299.73	160	453.29	485	295	402	63	90	12	18	10
6302	11906	8550	457	870	524	28202	997	17	751	3	46	76	11	125	285.86	136	442.97	402	382	358	24	42	0	4	5
5678	12458	8938	537	660	390	28154	1029	7	700	1	26	69	4	92	279.9	111	438.47	413	371	365	20	39	0	4	4
14286	7670	4376	8	824	654	27898	627	319	1025	153	88	256	131	156	271.77	250	435.02	489	413	444	23	93	21	0	0
11730	7896	5194	2	1946	1476	28528	585	116	630	23	260	158	69	257	309.76	137	458.24	492	211	385	83	81	10	24	14
4418	13336	9676	856	402	224	28076	1226	11	612	1	20	50	5	65	273.82	104	427.73	328	299	308	11	37	0	0	3
6330	11914	8484	476	872	560	28210	1035	25	719	4	44	80	20	112	285.98	111	441.73	412	381	328	21	52	0	6	5
3924	13668	9914	969	340	204	28062	1305	6	554	1	12	61	4	65	271.91	88	424.03	286	278	260	15	38	0	0	1
4458	13352	9608	821	404	252	28086	1198	6	641	1	10	74	5	91	273.78	114	428.42	323	304	295	10	50	0	2	3
4890	13150	9458	731	370	192	28066	1163	7	713	1	6	73	5	81	277.44	117	431.98	340	366	322	15	48	0	0	1
5546	12420	8944	583	758	470	28180	1054	17	675	3	40	71	10	106	285.97	125	442.21	403	338	342	18	45	0	2	2
4876	13092	9418	739	438	250	28086	1193	9	693	1	12	63	6	83	275.36	123	432.21	343	370	297	6	38	0	0	4
3840	13612	9954	969	436	232	28078	1295	6	529	1	4	58	4	77	270.65	81	422.76	281	260	273	11	40	0	0	2
3968	13650	9920	971	328	176	28046	1284	10	556	1	4	61	8	70	273.37	87	426.34	269	264	294	14	40	0	0	5
3984	13510	9860	936	462	262	28092	1267	5	539	0	14	54	4	68	272.29	94	424.82	306	242	279	18	41	0	0	1
3806	13748	9984	1003	320	186	28050	1323	5	552	1	6	58	3	61	270.55	85	422.81	269	268	265	14	39	0	0	4
11900	7842	5230	2	1840	1362	28460	528	131	650	21	252	168	77	277	301.74	132	454.98	468	219	434	74	92	11	34	18
3760	13788	10028	1014	298	166	28048	1330	2	552	1	8	57	1	67	270.96	86	423.34	259	262	269	13	40	0	0	2
3968	13686	9934	966	292	156	28042	1290	9	575	2	6	59	5	68	271.95	86	424.81	279	272	282	12	45	0	0	3
5210	12828	9212	663	526	316	28120	1108	9	706	1	28	65	7	78	279.31	125	435.44	372	349	349	20	50	0	0	5
3868	13744	9976	992	288	160	28042	1318	8	563	1	6	54	5	68	270.87	84	422.94	265	278	279	8	35	0	0	4

SUPPLEMENTARY INFORMATION:Monte Carlo Atomistic Simulation and Machine Learning Analysis of Na-K Eutectic Alloy in Condensed Phases, D. Reitz and E. Blaisten-Barojas, George Mason University, Fairfax, VA 22030

12154	7856	5102	0	1710	1304	28388	550	138	717	36	230	171	64	251	297.81	154	451.87	490	234	415	70	97	12	28	15
13256	7748	4742	2	1268	950	28118	591	233	877	82	140	199	112	218	282.27	188	446	466	321	463	42	94	16	14	2
4526	13258	9596	802	442	252	28090	1220	7	631	1	16	70	4	79	275.6	101	431.12	322	338	287	16	42	0	0	1
12654	7854	4874	1	1506	1180	28274	564	164	765	47	182	195	86	232	291.15	166	449.43	494	271	433	58	105	13	24	6
14094	7712	4510	7	868	644	27912	611	327	1005	154	74	254	134	162	273.85	245	438.63	480	406	455	26	81	13	10	3
4564	13196	9540	796	488	296	28106	1211	7	620	1	22	53	6	78	276.26	94	429.88	334	324	304	12	32	0	0	2
3862	13674	9932	978	372	222	28072	1305	8	536	1	10	55	6	62	269.75	85	422.97	286	253	268	14	40	0	0	4
11870	7754	5198	1	1874	1454	28506	512	130	614	33	316	201	61	285	313.74	115	459.77	465	191	377	92	112	7	38	21
4298	13396	9722	862	406	236	28080	1230	6	618	2	22	65	0	81	274.36	95	428.48	309	302	293	13	42	0	0	3
4070	13594	9868	950	326	180	28048	1292	7	598	2	10	50	3	68	271.77	93	425.37	275	284	289	15	35	0	0	2
4750	13182	9438	753	436	280	28100	1195	7	660	0	12	61	5	81	277.58	103	432.76	321	348	317	16	39	0	2	5
12730	7856	4874	0	1416	1142	28252	579	181	799	60	214	194	94	225	293.73	167	448.29	483	286	434	59	100	12	20	5
5442	12532	9050	619	696	406	28158	1124	12	688	1	32	59	10	94	281.25	97	438.04	368	374	326	19	37	0	0	3
13106	7768	4718	5	1332	1066	28184	595	206	843	66	174	191	107	221	283.45	169	447.35	488	314	436	40	95	10	20	6
4384	13292	9672	825	470	258	28092	1211	5	592	1	16	58	4	76	277.23	90	431.75	310	293	316	19	37	0	0	4
6796	11614	8222	406	908	614	28238	967	30	724	6	80	86	18	133	289.38	105	444.45	422	366	349	29	49	1	2	4
12488	7808	5004	1	1628	1190	28324	590	156	725	47	182	173	83	236	290.7	149	454.16	480	272	426	59	97	7	22	11
5122	12910	9282	661	492	278	28098	1116	6	708	1	14	76	3	91	279.57	99	434.77	357	359	332	19	54	0	0	2
11328	7948	5460	0	2016	1490	28578	507	92	634	23	308	182	47	274	317.96	123	461.18	485	196	386	95	103	1	26	29
3924	13688	9928	980	324	184	28052	1305	9	567	1	4	72	6	82	270.56	92	422.24	266	268	258	9	41	0	0	3
14286	7622	4376	6	856	670	27906	604	337	999	151	96	254	145	160	271.82	260	437.38	499	381	449	22	85	22	0	2
10560	8016	5810	2	2258	1678	28772	526	61	525	9	402	146	33	303	330.52	87	471.15	462	165	360	127	77	6	40	24
5684	12408	8858	554	714	472	28184	1068	12	698	2	42	62	6	98	284.84	98	440.36	411	387	332	20	40	0	6	0
11702	7878	5192	0	1924	1496	28504	544	107	609	24	288	175	54	276	304.86	111	455.24	480	204	394	76	92	4	22	20
13570	7704	4540	3	1210	942	28094	611	247	896	99	120	229	108	199	277.24	205	444.3	484	348	420	40	98	16	6	3
4182	13588	9822	936	280	158	28040	1279	8	631	1	10	60	5	70	271.21	98	424.53	281	286	288	12	44	0	0	3
4450	13196	9590	776	536	322	28124	1200	6	601	0	30	52	4	84	277.17	92	434.79	327	310	307	17	34	0	0	4
3904	13702	9942	979	312	180	28048	1303	9	560	1	8	63	5	71	271.33	86	423.84	269	266	273	13	41	0	0	3
4058	13498	9822	912	434	256	28086	1245	5	547	1	18	64	4	80	272.22	86	426.29	300	256	289	17	40	0	0	1
12004	7930	5188	1	1754	1292	28406	529	125	715	39	214	191	62	243	301.22	126	454.35	503	255	419	76	105	7	22	13
11652	7802	5270	0	1982	1498	28544	552	108	606	20	316	171	63	262	311.52	123	459.17	462	192	399	104	85	7	24	17
13278	7850	4740	1	1204	912	28124	612	210	907	72	132	194	115	215	282.2	182	446.12	470	365	463	32	102	13	6	3
4016	13640	9886	943	322	182	28052	1295	2	602	0	6	64	1	76	272.12	89	425.25	270	289	273	15	44	0	0	1
10418	7830	5798	0	2496	1836	28860	507	48	427	6	424	158	26	290	331.07	62	472.35	462	128	349	159	68	1	54	30
14272	7664	4366	6	874	672	27918	635	308	1020	153	64	246	119	170	273.85	235	436.59	481	422	445	16	83	16	6	1
12090	7976	5124	3	1670	1286	28400	555	105	737	30	236	194	53	247	304.15	147	454.22	492	251	403	67	112	5	18	12

13760	7786	4602	0	1004	746	27994	644	285	985	127	90	217	121	166	274.13	242	438.94	491	396	444	25	78	17	6	2
5732	12352	8842	489	744	472	28186	1026	13	729	4	44	81	5	108	285.24	122	439.12	418	396	338	22	44	0	0	1
11410	7882	5356	1	2024	1548	28568	532	101	638	18	306	170	55	274	307.75	128	456.75	498	223	388	86	94	6	38	20
12014	7778	5154	1	1842	1384	28468	535	119	688	28	262	179	66	254	305.28	159	454.42	481	217	412	89	94	5	28	16
6640	11740	8350	431	864	550	28206	1015	24	764	7	60	89	15	119	286.96	126	444.43	404	409	331	32	50	0	2	1
5068	12900	9286	678	528	310	28112	1153	14	689	0	18	73	9	87	277.79	111	434.84	352	375	305	18	44	0	2	3
4560	13232	9564	808	450	266	28090	1202	5	620	2	18	59	1	81	274.36	89	430.59	318	313	318	15	37	0	0	2
3916	13686	9942	971	310	182	28054	1333	7	588	1	18	66	5	63	271.63	95	423.72	271	301	249	13	37	0	0	2
12586	7920	5004	3	1462	1092	28258	604	170	809	49	176	197	85	207	290.2	162	452.07	476	290	409	63	115	14	16	13
4806	13092	9428	773	472	282	28098	1220	11	664	1	18	57	6	86	276.2	99	430.09	317	358	299	13	38	0	0	1
4124	13478	9794	903	424	248	28084	1256	6	568	0	16	53	2	86	273.78	77	427.05	281	278	300	17	37	0	0	1
14468	7688	4338	3	726	570	27852	632	324	1121	162	58	258	128	143	269.41	291	432.14	486	446	450	22	86	16	4	1
7652	10946	7668	276	1124	784	28280	901	40	750	10	102	94	23	172	288.37	109	445.47	416	386	367	33	55	1	4	4
4118	13520	9794	918	392	238	28076	1271	6	572	2	14	56	2	74	272.06	84	426.92	295	284	288	10	40	0	0	3
13674	7644	4570	11	1154	870	28038	617	283	893	128	110	220	120	189	276.49	213	440.84	484	352	446	29	79	12	14	3
13420	7572	4620	2	1372	1010	28146	619	244	806	95	140	202	100	199	280.95	173	446.86	496	310	417	38	77	20	10	7
6290	12040	8582	478	734	464	28164	980	27	731	6	50	86	16	113	287.09	120	444.09	408	363	379	26	51	1	4	4
3980	13682	9908	960	290	174	28048	1306	8	588	2	14	60	4	65	269.99	99	423.65	275	288	273	13	36	0	0	3
11328	7974	5404	0	2036	1512	28556	557	94	615	24	270	179	37	274	307.86	131	461.88	490	207	362	85	88	12	32	15
3878	13738	9972	990	278	160	28038	1322	8	565	1	12	65	5	71	270.51	83	423.16	264	272	258	10	42	0	0	3
14466	7668	4400	2	732	522	27836	613	332	1121	162	44	273	124	134	269.18	279	431.13	498	436	449	25	100	25	4	2
11980	7924	5200	1	1772	1292	28402	544	141	701	36	218	178	66	271	300.36	146	452.11	489	243	408	61	102	4	16	12
4346	13336	9672	814	458	268	28098	1200	5	599	0	18	60	2	84	276.75	83	433.2	324	309	303	17	41	0	0	2
13554	7632	4576	1	1250	944	28098	607	256	847	93	138	212	116	208	281.34	158	445.72	450	335	457	45	99	24	4	6
11910	7962	5204	0	1780	1332	28448	566	111	689	26	248	178	60	238	306.54	123	457.67	462	226	417	96	104	12	10	17
4188	13472	9800	902	392	204	28060	1256	7	584	0	4	65	4	63	274.81	98	429.48	296	288	287	21	42	0	0	4
10960	7804	5524	0	2330	1708	28708	506	78	502	13	344	163	46	295	319.24	91	464.91	494	151	359	120	93	6	38	19
14428	7644	4364	7	802	578	27860	687	337	1060	168	44	265	124	144	270.28	254	433.02	477	455	407	13	89	23	0	2
5862	12332	8790	559	702	444	28164	1065	18	709	3	30	60	11	89	283.98	113	439.07	405	369	346	21	34	1	4	4
4934	12940	9334	737	538	340	28128	1165	7	643	0	42	56	5	79	279.9	104	435.69	344	314	326	21	48	1	0	4
13738	7826	4572	7	980	772	27990	609	293	962	137	88	235	114	177	275.52	236	440.32	487	370	453	28	79	16	14	3
6710	11578	8310	442	962	596	28230	994	31	708	8	66	76	19	122	287.93	124	445.5	417	374	348	26	46	0	8	6
11478	7850	5398	1	2052	1480	28572	544	88	576	22	280	169	41	279	313	121	463.56	497	193	360	81	89	8	34	26
13650	7786	4626	4	1052	792	28016	621	251	992	116	106	230	109	185	275.1	234	439.17	490	386	431	27	99	15	2	2
14266	7688	4430	2	844	614	27902	652	310	1045	143	56	250	129	174	274.5	250	437.86	459	433	441	18	94	15	4	0
4222	13426	9750	882	428	246	28086	1253	6	600	1	10	54	5	74	271.03	101	424.49	295	294	298	20	32	0	4	1

4866	13014	9372	721	518	320	28116	1152	6	662	0	26	59	4	85	277.58	116	434.12	346	324	330	21	42	0	0	1
4158	13542	9808	918	342	196	28056	1257	11	599	1	10	68	8	72	271.16	101	424.13	297	281	288	12	52	0	0	3
4638	13166	9520	785	490	276	28098	1184	5	641	2	8	63	3	87	277.74	84	432.25	319	316	320	22	50	0	0	0
14620	7610	4302	5	712	534	27834	631	342	1118	171	54	268	135	155	267.81	265	429.22	483	476	444	13	89	11	2	0
12194	8024	5140	0	1556	1192	28340	558	133	758	21	210	184	79	237	297.71	152	451.72	491	271	426	67	107	11	22	10
7300	11032	7862	307	1178	814	28328	897	23	738	3	138	81	14	159	291.94	108	445.95	446	380	365	35	54	0	4	3
4702	13122	9516	796	468	260	28090	1223	11	648	1	20	61	6	76	274.82	86	430.89	320	331	296	17	45	0	2	1
13866	7688	4620	3	1006	692	27954	595	284	1005	139	70	254	112	173	275.2	231	440.15	476	400	451	36	94	14	12	4
4074	13564	9856	935	358	196	28056	1290	8	598	1	8	56	5	76	271.79	81	426.61	278	297	276	12	37	0	0	2
12020	7834	5114	1	1780	1384	28432	598	134	670	42	284	170	65	228	298.41	148	454.66	476	227	400	83	86	6	12	15
14274	7706	4382	6	786	646	27888	618	321	1058	156	92	252	129	156	270.91	270	434.6	484	417	456	21	84	19	2	4
4852	13012	9412	729	520	294	28108	1193	6	680	0	16	64	4	105	279.13	96	435.59	301	356	316	15	40	0	2	2
4712	13102	9510	726	504	266	28106	1157	6	656	0	10	57	5	76	277.01	98	432.64	366	344	328	13	35	0	2	0
4830	12998	9406	731	546	316	28118	1173	6	660	0	22	57	3	100	275.76	96	430.91	326	348	321	16	36	0	0	0
12836	7760	4850	1	1466	1126	28248	588	188	787	50	190	210	88	203	298.34	167	450.68	485	294	405	66	109	19	18	13
4324	13318	9676	869	488	280	28100	1255	6	568	1	14	63	3	81	277.2	77	431.63	310	292	268	12	40	0	0	2
13308	7678	4560	3	1354	1102	28182	582	238	789	84	164	204	115	201	283.99	178	447.35	482	298	446	55	89	14	12	10
5178	12830	9216	682	542	328	28120	1136	10	688	1	26	67	6	85	280.97	104	437.37	379	349	307	14	45	0	0	1
5094	12960	9312	682	458	256	28094	1119	9	713	0	14	57	5	84	278.58	112	434.17	359	357	354	17	46	0	0	2
4334	13336	9678	835	466	268	28096	1222	4	602	0	14	55	4	68	277.31	97	432.4	322	298	305	22	34	0	0	1
13792	7718	4500	4	1070	828	28006	626	306	928	140	88	236	129	177	273.46	227	437.97	481	358	439	33	83	17	10	1
4374	13274	9642	825	512	294	28110	1237	4	600	0	14	67	3	84	277.28	96	432.85	309	311	277	18	43	0	0	2
4706	13122	9480	750	504	286	28108	1185	2	666	0	10	52	1	79	276.67	108	432.16	339	335	319	14	32	0	0	4
4324	13376	9700	854	414	244	28078	1225	9	601	1	18	63	4	81	272.93	85	427.06	306	302	300	15	41	0	2	3
5934	12304	8746	538	676	452	28164	1040	20	726	3	50	71	11	107	281.24	111	438.63	400	365	349	21	42	0	2	2
6514	11842	8338	383	882	604	28238	973	18	758	1	54	73	9	129	287.49	124	444.02	420	394	357	32	35	2	4	1
13280	7972	4776	4	1104	828	28062	664	215	970	70	94	211	112	182	275.94	201	443.99	463	391	427	37	119	14	8	3
11408	7956	5434	3	1990	1454	28548	518	99	623	19	270	168	42	274	314.06	140	459.36	521	180	378	80	95	8	36	19
14252	7754	4438	3	770	594	27878	665	310	1079	160	70	257	111	154	268.08	267	429.29	477	447	424	12	86	16	0	4
11880	7884	5210	1	1792	1388	28468	557	127	652	28	274	183	65	245	303.8	131	456.08	503	211	383	79	107	17	36	19
13312	7720	4598	0	1294	1056	28164	601	223	841	108	174	213	84	197	276.18	213	443.67	533	310	418	30	90	16	6	1
4954	12946	9320	717	552	330	28118	1144	6	647	1	16	56	1	89	279.47	105	433.54	360	328	325	16	35	0	0	0
4680	13186	9558	766	424	216	28074	1180	6	693	0	10	61	5	97	276	83	433.42	313	348	336	7	40	0	0	2
12498	8034	5078	7	1408	1050	28258	646	161	838	36	174	173	93	205	288.97	164	448.64	480	325	405	54	101	11	16	10
11186	7956	5528	2	2058	1526	28614	524	96	575	8	318	164	59	291	313.68	120	462.17	488	182	374	95	89	4	42	18
14340	7676	4378	3	814	612	27878	680	318	1081	159	56	261	123	151	267.48	273	430.15	475	458	412	14	88	17	2	2

13944	7664	4472	10	1012	780	27976	649	293	935	146	96	239	110	170	275.13	202	438.82	458	391	432	37	79	15	8	2
13840	7742	4588	5	958	726	27968	618	287	990	120	106	240	118	178	277.84	218	441.88	470	398	446	32	97	23	8	3
4998	12796	9306	651	660	364	28144	1118	4	671	0	18	81	1	87	278.28	113	435.2	380	342	306	20	52	0	2	2
4188	13502	9784	895	366	210	28060	1253	8	595	3	10	65	4	67	274.34	94	426.88	306	289	287	16	44	0	0	1
4422	13320	9628	821	448	266	28098	1214	4	629	0	14	61	3	83	276.19	97	431.54	318	316	307	11	39	0	0	4
3924	13606	9924	965	384	216	28072	1290	6	544	1	18	55	5	86	270.32	75	423	267	260	287	9	39	0	0	3
12336	7764	5124	1	1674	1196	28332	562	166	737	50	216	183	82	259	299.95	155	453.8	466	253	415	65	95	11	22	10
4420	13356	9652	823	408	232	28074	1218	6	631	1	6	66	3	82	273.44	88	427.48	314	319	304	11	49	0	0	2
5018	12974	9322	670	474	290	28106	1122	9	710	0	26	59	4	78	278.29	116	433.34	380	361	334	15	38	0	2	2
4770	12970	9416	731	588	354	28136	1179	7	621	0	38	67	4	84	278.87	90	434.34	344	340	298	21	41	0	0	2
7082	11544	8100	366	860	566	28210	941	27	795	7	54	81	15	141	287.74	129	444.36	414	403	384	26	49	0	4	3
5418	12558	9048	603	704	410	28158	1082	13	679	2	20	60	6	96	281.82	108	438.84	377	342	353	23	39	0	0	3
4658	13280	9578	790	332	186	28054	1198	6	681	1	20	65	2	65	275.91	107	430.87	338	345	307	15	42	0	0	2
4140	13522	9822	916	372	200	28062	1269	6	613	1	6	51	3	62	271.53	106	425.7	298	282	298	13	41	0	0	4
13718	7826	4666	9	986	696	27966	656	280	989	128	70	235	118	168	275.91	234	442.84	486	399	417	23	94	14	4	1
4228	13426	9746	882	418	246	28084	1252	10	593	1	20	64	6	66	273.5	94	426.42	313	297	283	17	41	0	0	2
4008	13550	9842	930	422	254	28090	1277	6	545	1	10	45	4	77	272.89	64	425.23	295	266	288	11	31	0	4	1
14204	7720	4440	5	850	624	27890	641	316	1054	157	50	253	122	138	270.8	261	433.6	497	443	439	24	86	20	2	2
14250	7660	4436	5	866	620	27888	642	317	1039	155	52	244	120	146	271.09	260	433.29	498	426	449	14	72	22	4	2
14360	7554	4370	4	902	644	27894	633	324	1043	158	62	264	122	144	271.35	267	435.14	507	403	426	22	90	22	2	0
4970	12932	9314	679	546	336	28128	1138	3	686	0	30	72	2	95	277.64	112	434.35	365	354	313	10	51	0	0	3
5008	12862	9272	667	616	368	28146	1137	10	665	0	20	62	9	95	277.59	113	434.12	363	347	322	12	39	0	0	2
10830	7940	5578	1	2214	1720	28724	500	78	524	13	396	173	42	320	321.04	102	466.43	475	165	360	99	86	5	44	29
5488	12492	8978	583	736	454	28182	1069	13	671	1	32	67	10	101	286.31	111	440.88	409	344	333	17	45	0	2	0
12628	7904	5014	2	1454	1058	28240	560	180	760	52	170	173	92	230	287.01	162	449.76	481	254	465	50	92	15	12	12
3830	13768	10002	1001	282	152	28038	1291	7	551	1	4	62	4	63	269.83	96	422.85	281	240	282	12	40	0	0	4
14324	7712	4412	9	780	584	27872	673	348	1034	159	60	252	140	154	270.81	253	432.94	466	458	429	17	79	28	0	1
4294	13404	9708	877	416	246	28084	1249	6	608	1	16	52	3	73	275.21	92	431.58	308	298	298	13	32	0	0	2
5450	12630	9056	621	612	366	28138	1085	6	713	1	24	78	3	82	280.84	120	436.78	405	335	324	20	53	0	0	1
6576	11814	8420	457	820	504	28184	993	29	748	10	44	83	16	110	287.81	112	443.54	422	380	357	26	51	0	4	2
4518	13274	9586	813	444	258	28090	1219	7	606	0	10	51	5	72	273.96	80	428.74	312	312	317	22	36	0	0	0
4426	13378	9656	844	380	222	28074	1228	6	655	0	12	62	3	84	274.02	109	428.32	312	311	296	9	44	0	0	2
13550	7878	4696	4	1020	768	28020	647	261	965	113	98	224	117	179	277.06	218	442.75	481	400	430	27	93	11	10	2
10976	7968	5604	1	2142	1590	28664	513	74	559	6	344	153	45	304	317.61	104	461.09	497	177	371	82	92	4	36	30
11288	7906	5356	1	2084	1610	28598	493	101	563	19	316	190	50	260	318.07	111	459.4	489	159	378	125	108	4	38	21
12386	7814	4978	1	1684	1278	28376	595	151	707	45	216	172	69	252	299.55	157	452.74	485	257	395	64	75	12	20	7

SUPPLEMENTARY INFORMATION:Monte Carlo Atomistic Simulation and Machine Learning Analysis of Na-K Eutectic Alloy in Condensed Phases, D. Reitz and E. Blaisten-Barojas, George Mason University, Fairfax, VA 22030

4488	13244	9594	808	484	278	28100	1222	10	624	1	10	55	6	86	274.57	90	429.2	312	311	301	13	39	0	2	3
4128	13590	9840	906	310	174	28052	1253	4	596	0	10	57	2	70	274.4	95	427.26	300	292	306	10	36	0	0	2
11084	7920	5504	0	2144	1622	28658	559	81	576	13	340	149	47	258	318.58	104	462.22	496	204	368	109	81	2	42	19
14378	7620	4360	8	832	622	27878	656	341	1044	171	66	265	127	154	269.45	263	432.93	487	440	418	14	81	22	0	1
13800	7750	4586	4	1014	744	27986	641	285	973	129	86	228	123	179	276.84	228	442.6	467	388	443	30	86	16	6	1
4390	13298	9634	841	482	284	28102	1227	3	593	0	14	57	2	86	274.48	81	431.04	301	299	302	21	38	0	0	0
14272	7746	4402	4	780	614	27880	663	307	1068	159	60	265	111	145	270.76	264	434.43	490	445	410	18	92	24	6	2
11628	7776	5254	0	1990	1546	28570	526	114	607	30	348	182	52	276	309.6	108	459.87	479	193	379	99	112	4	24	21
4116	13544	9832	928	350	204	28066	1276	4	603	2	18	59	2	69	274.84	96	429.16	289	295	284	15	36	0	2	3
11446	7760	5310	2	2078	1606	28584	502	112	580	31	348	197	51	288	311.76	134	461.7	480	175	366	99	93	7	34	24
14526	7634	4338	8	746	544	27832	663	348	1063	172	44	274	139	160	269.05	269	432.09	459	442	427	11	88	16	0	2
10722	7704	5628	0	2448	1828	28810	452	78	439	12	428	164	36	331	329.15	97	471.91	490	109	363	112	69	3	50	37
12146	7996	5080	3	1666	1262	28350	626	135	749	44	178	171	69	211	284.91	160	450.03	517	288	389	61	87	12	18	4
14474	7548	4326	4	848	616	27874	613	347	1036	163	60	266	142	150	271.26	268	434.58	487	407	448	27	93	22	2	2
14098	7864	4556	3	744	556	27884	602	291	1118	138	64	250	120	157	274.56	270	438.98	489	428	468	24	100	11	2	1
12904	7754	4816	2	1432	1118	28244	602	186	823	61	202	185	94	213	285.25	164	449.92	472	308	444	58	100	13	18	8
4984	13012	9356	712	472	264	28096	1158	9	693	1	8	61	7	95	278.69	101	434.85	326	352	342	14	39	0	0	2
13778	7976	4630	5	840	646	27936	653	274	1047	126	62	233	111	160	275.78	237	438.99	472	440	445	23	89	14	4	4
4026	13630	9864	948	320	202	28060	1284	6	574	1	16	58	5	69	269.94	83	422.72	284	281	286	15	44	0	0	1
11914	7984	5238	3	1708	1318	28456	545	137	704	33	256	177	76	265	303.83	151	454.5	488	237	402	75	110	8	30	14
5300	12724	9140	619	586	358	28142	1101	4	714	0	34	73	3	93	280.19	116	436.06	381	375	328	18	51	0	0	2
12588	7936	4998	4	1466	1080	28240	615	154	819	46	158	187	76	202	287.42	174	447.19	496	297	421	54	102	16	14	6
13588	7568	4458	0	1310	1050	28140	602	231	844	88	154	210	111	204	281.14	187	445.72	468	314	454	47	95	19	12	6
10956	8010	5592	2	2130	1602	28674	515	77	548	13	338	152	42	295	320.86	112	465.12	472	184	383	119	77	2	44	16
4988	12808	9306	654	648	372	28156	1130	3	684	1	34	66	1	96	279.49	105	435.98	369	358	320	15	44	0	0	2
4632	13116	9524	774	512	296	28108	1186	8	634	0	28	65	6	77	277.32	85	433.69	343	326	301	19	49	0	0	2
4494	13276	9612	812	434	248	28078	1226	10	633	2	14	66	2	72	275.73	101	429.77	328	331	282	14	45	0	0	2
4858	12940	9388	714	584	334	28130	1173	9	650	0	26	60	7	84	278.25	99	431.87	348	343	306	20	40	0	0	1
3856	13748	9982	994	282	162	28042	1314	7	563	1	12	51	5	73	270.69	82	423.41	257	265	288	11	32	0	0	2
4702	13206	9510	768	404	242	28086	1216	5	680	1	22	67	4	80	276.79	93	433.15	318	365	298	16	45	0	0	1
6796	11536	8266	430	950	590	28210	930	41	689	13	68	88	19	141	288.34	97	445.36	405	333	383	31	48	2	4	8
13064	7720	4724	3	1418	1094	28202	580	220	792	72	170	197	106	236	294.72	164	450.24	459	277	450	50	97	16	12	7
5002	12856	9294	683	614	356	28142	1159	4	659	0	16	63	1	87	280.2	103	437.4	364	349	300	15	37	0	4	3
12872	7686	4904	1	1526	1070	28228	595	186	787	58	150	209	84	205	291.35	157	449.63	481	289	411	68	102	21	18	8
7680	11012	7732	317	1050	684	28230	908	51	765	19	70	104	26	136	287.81	121	445.12	427	377	363	42	51	2	2	4
4166	13568	9832	901	306	164	28044	1267	7	613	0	8	61	5	77	273.09	84	427.3	280	309	298	10	43	0	0	3

SUPPLEMENTARY INFORMATION:Monte Carlo Atomistic Simulation and Machine Learning Analysis of Na-K Eutectic Alloy in Condensed Phases, D. Reitz and E. Blaisten-Barojas, George Mason University, Fairfax, VA 22030

3950	13528	9874	912	458	266	28094	1265	6	538	1	16	44	2	65	271.87	78	425.08	302	276	294	19	27	0	2	3
4036	13600	9880	949	344	188	28056	1270	8	584	1	8	64	5	68	273.2	107	425.83	306	259	275	9	42	0	0	2
4892	12918	9372	691	594	334	28132	1131	10	663	2	22	62	6	86	277.5	107	434.87	370	334	324	19	39	0	0	1
14334	7638	4364	4	860	638	27890	646	298	1065	158	52	273	108	150	272.12	285	435.52	496	420	414	16	97	17	4	3
4742	13126	9460	753	466	284	28102	1175	7	654	0	24	47	4	85	274.97	105	429.39	330	333	338	17	32	0	0	1
4644	13108	9514	804	524	296	28104	1219	10	640	0	18	64	5	77	275.89	100	430.96	319	315	288	21	44	0	0	3
4632	13194	9524	787	452	272	28096	1205	3	659	0	22	65	3	70	276.18	114	430.97	335	328	302	17	49	0	0	3
5830	12272	8760	547	760	506	28180	1053	25	687	6	50	73	13	123	283.18	107	438.09	382	352	338	18	45	0	0	3
4148	13466	9780	904	434	248	28084	1266	6	581	2	6	60	3	74	272.19	86	427.6	290	282	292	15	36	0	2	2
3970	13664	9920	969	308	174	28048	1293	4	572	0	12	54	1	76	271.89	86	425.41	271	264	289	11	32	0	0	1
11250	7954	5482	1	2054	1522	28606	520	82	584	12	308	159	48	276	319.24	112	464.8	491	192	379	96	82	3	34	23
5052	12916	9302	693	510	302	28108	1111	11	684	0	26	73	9	81	278.89	113	435.11	374	327	334	19	56	0	0	3
10998	7780	5462	1	2270	1782	28758	524	93	519	16	404	156	44	272	319.26	119	464.41	504	155	353	120	79	10	58	28
14466	7628	4326	4	780	596	27858	663	328	1084	158	60	270	116	155	268.8	268	432.1	475	455	410	15	102	29	2	2
11258	7984	5414	0	2018	1578	28634	543	94	585	14	344	161	54	277	313.77	104	461.83	493	206	359	101	83	4	38	19
12132	7868	5060	1	1752	1344	28412	583	132	696	39	238	174	65	248	298.29	159	455.69	478	233	402	73	87	8	16	14
5136	12898	9274	656	504	280	28102	1141	6	724	1	10	65	3	105	280.44	102	436.8	331	379	340	13	47	0	0	1
10320	7858	5716	0	2454	1988	28928	512	52	444	11	524	143	26	306	335.52	89	473.68	479	129	348	145	72	3	58	31
4902	12938	9348	687	586	340	28130	1153	10	649	0	16	61	6	88	279.73	99	436.9	351	343	317	17	45	0	0	2
14052	7772	4458	6	846	700	27936	635	285	1031	148	102	261	102	175	274.66	251	438.48	469	418	431	25	95	10	6	1
5912	12130	8780	518	814	490	28188	1039	14	702	3	58	72	8	121	285.03	95	441.32	386	376	340	26	48	0	4	2
5242	12790	9160	621	556	350	28126	1060	2	700	0	28	77	1	97	280.32	114	438.13	393	335	349	11	46	0	0	7
6250	11902	8544	437	924	566	28240	995	17	733	1	52	80	11	112	287.38	132	444.89	446	366	329	23	57	0	0	5
4136	13592	9832	953	304	174	28046	1305	4	620	1	6	62	3	72	271.07	96	423.47	276	299	266	11	43	0	2	1
4780	13022	9418	722	558	328	28126	1182	4	646	0	18	61	3	91	279.64	88	433.42	332	341	316	14	45	0	2	3
7500	10970	7818	327	1148	740	28276	878	39	706	8	92	102	22	166	289.53	112	447.33	408	339	385	43	59	1	8	5
13086	7926	4782	1	1220	968	28146	596	183	922	67	158	203	91	207	283.87	207	447.29	489	333	444	44	103	11	6	2
4004	13534	9876	948	422	230	28078	1293	11	553	2	12	54	6	68	272.96	76	425.53	287	272	276	14	32	0	0	3
10728	7944	5614	2	2296	1744	28750	523	61	545	9	384	171	35	292	323.8	114	465.61	500	189	334	111	75	1	36	26
13928	7680	4480	3	1026	778	27988	654	273	966	130	92	226	100	186	273.11	211	437.33	460	400	440	24	86	22	4	1
14000	7820	4570	3	820	620	27916	649	291	1071	124	78	226	123	151	275.52	228	438.36	457	440	471	34	84	20	8	2
4844	12940	9402	698	608	328	28134	1161	6	647	0	12	64	4	92	279.13	97	435.43	360	359	307	11	35	0	0	1
4824	12986	9382	701	570	346	28136	1157	5	655	0	28	59	3	74	278.22	106	434.65	356	347	323	22	35	0	0	5
12210	8010	5156	1	1578	1170	28336	594	131	806	26	184	179	72	237	292.95	152	452.22	467	284	415	68	118	9	26	12
4508	13314	9640	838	378	206	28060	1223	12	644	1	14	63	7	69	274.14	110	428.07	329	316	297	11	42	0	0	1
4364	13452	9724	866	324	176	28048	1239	8	639	1	8	63	6	71	272.5	97	427.49	303	318	304	12	43	0	0	3

11640	8000	5250	1	1842	1450	28482	566	107	693	25	258	151	58	280	298.74	130	452.72	449	244	430	80	89	7	36	14
6684	11656	8346	397	930	552	28218	942	16	773	3	48	87	12	118	287.23	150	443.09	457	368	361	24	61	0	2	3
7030	11396	8122	374	978	628	28240	917	33	725	6	80	95	19	151	288.53	120	444.62	421	357	365	33	58	2	4	3
3962	13640	9916	961	338	188	28054	1290	11	563	1	10	55	8	72	270.52	84	422.18	281	274	282	10	39	0	0	3
5270	12606	9144	615	706	404	28162	1120	15	665	0	32	71	6	90	281.18	109	439.32	375	368	308	23	43	0	0	4
11798	7904	5250	0	1842	1392	28486	555	115	650	22	268	164	56	251	308.55	123	459.37	482	225	409	87	95	9	32	18
4716	12978	9450	728	630	348	28136	1157	5	619	1	12	62	3	85	276.84	95	431.78	356	308	316	18	38	0	2	2
5842	12464	8936	534	540	292	28098	1034	15	808	1	22	70	11	98	281.7	124	439.17	385	404	378	20	47	1	2	3
5204	12764	9214	675	582	330	28114	1130	17	674	5	20	67	8	90	281.24	98	438.39	352	345	329	20	46	0	0	3
12970	7960	4892	2	1248	936	28156	627	181	912	63	136	207	85	195	285.05	194	449.17	480	343	423	51	112	14	14	3
10552	7810	5720	1	2378	1842	28836	529	59	473	10	466	157	29	303	330.16	88	469.69	442	151	341	135	75	6	64	33
14278	7632	4392	7	880	650	27896	652	314	1046	171	60	266	100	142	270.6	252	434.44	504	453	413	18	88	18	4	2
4258	13424	9728	867	404	244	28078	1259	7	619	1	18	55	5	86	271.82	86	425.33	287	314	292	13	35	0	2	1
14484	7636	4334	5	772	578	27858	656	342	1065	156	48	252	133	160	271.75	268	434.52	455	431	446	20	78	30	6	1
14302	7622	4380	2	854	656	27902	643	327	1027	152	88	259	127	157	271.74	249	436.18	486	428	424	23	100	28	0	0
4838	13078	9444	727	458	256	28092	1127	4	668	0	18	67	3	82	278.51	98	434.7	359	334	336	21	40	0	0	0
4204	13484	9770	894	370	222	28070	1256	9	608	1	20	59	6	73	274.22	94	428.67	296	293	295	15	45	0	0	2
3734	13786	10030	1003	316	178	28050	1320	8	542	0	6	58	6	58	270.76	86	423.6	268	264	273	16	34	1	0	4
14226	7712	4464	6	824	598	27880	617	325	1044	161	44	261	119	166	272.87	264	437.3	480	411	446	20	83	19	12	1
4078	13560	9850	946	366	200	28060	1283	8	574	1	6	60	5	66	272.29	83	424.85	295	273	275	14	44	0	0	1
4476	13284	9646	839	426	230	28078	1232	7	619	0	16	56	4	76	275.24	96	429.55	310	309	301	14	38	0	0	3
4734	13016	9436	754	590	340	28132	1184	10	614	0	12	57	5	96	279.82	84	437.04	327	320	310	18	44	0	4	1
4538	13208	9604	766	486	252	28096	1187	6	630	0	8	64	5	90	277.32	97	431.29	333	330	305	12	43	0	0	1
4854	13148	9420	747	392	244	28074	1176	10	680	0	14	57	6	66	274.66	113	430.32	357	351	314	16	40	0	2	1
12462	7728	4950	1	1752	1274	28370	578	152	721	37	182	169	82	256	294.93	150	451.1	498	253	405	55	101	8	20	7
4280	13330	9714	863	492	268	28094	1250	12	595	3	10	58	6	68	276.55	103	429.82	326	293	276	14	38	0	0	1
14136	7614	4448	6	926	700	27924	612	319	1030	159	94	262	117	157	271.92	262	436.23	496	401	429	32	86	21	6	1
4848	12972	9408	728	560	312	28120	1162	8	647	0	20	61	7	101	279.81	80	434.46	339	333	320	12	47	0	0	0
14438	7640	4344	5	784	600	27876	643	333	1057	160	68	253	132	138	271.23	284	434.59	514	439	429	13	85	19	2	2
5194	12810	9202	644	576	334	28128	1119	6	705	2	12	79	2	88	278.73	115	435.76	380	367	313	14	50	0	0	2
11360	7860	5446	2	2060	1520	28602	516	95	573	16	320	151	48	276	317.59	103	464.14	500	200	400	89	77	4	32	23
14222	7764	4456	6	772	590	27868	641	314	1094	161	60	264	115	151	269.98	280	432.16	493	444	425	15	91	14	4	2
14364	7492	4268	4	984	752	27942	611	332	949	152	74	255	134	189	273	221	438.34	463	378	446	24	88	18	8	3
5684	12398	8874	608	726	452	28164	1074	18	707	7	28	66	9	104	282.45	118	440.63	386	331	341	17	34	0	2	4
7226	11298	7960	324	1012	676	28252	915	31	767	11	78	87	16	145	287.3	134	444.5	438	384	368	29	44	1	2	5
13952	7596	4518	4	1050	758	27976	585	298	952	136	94	253	110	191	276.88	215	446.13	474	345	443	35	103	21	8	3

12844	7862	4834	7	1404	1084	28198	623	206	815	69	154	185	103	198	281.96	173	447.48	476	301	430	56	92	11	12	8
5472	12578	9030	619	648	388	28138	1120	11	684	1	18	73	6	99	281.28	97	437.79	359	377	318	22	43	0	4	1
5280	12652	9164	615	658	368	28148	1100	3	728	0	24	69	1	88	279.19	105	435.15	375	373	335	27	47	0	2	1
4142	13506	9826	924	376	198	28054	1298	7	604	1	6	56	3	74	274.27	88	428.02	264	313	279	19	39	0	0	1
4290	13482	9740	893	340	196	28056	1246	9	607	1	6	55	7	75	274.2	88	427.62	300	290	304	11	36	0	2	2
4054	13584	9864	930	354	196	28060	1277	5	596	2	8	74	3	74	271.03	94	424.46	290	283	266	12	48	0	0	3
4180	13478	9786	876	406	218	28068	1232	8	597	2	0	63	4	73	273.12	107	427.18	323	290	286	11	44	0	0	2
11942	8126	5240	3	1616	1226	28384	567	118	756	23	220	170	64	240	298.43	141	455.57	488	280	418	75	102	7	12	10
6694	11654	8262	392	932	620	28232	938	24	752	2	64	74	17	136	288.48	138	443.72	446	364	380	18	55	1	6	2
11086	8034	5366	2	2054	1726	28710	535	73	553	9	392	151	42	300	316.8	113	463.1	471	184	375	96	80	5	50	32
5658	12306	8876	532	796	506	28204	1062	7	677	1	56	67	3	111	283.27	104	441.48	393	377	327	25	37	0	4	3
14002	7580	4430	6	1062	806	27984	623	299	950	145	94	256	119	180	273.36	238	436.77	493	383	418	24	94	18	10	1
5378	12646	9116	628	618	354	28138	1100	10	706	2	24	56	6	101	280.62	99	438.35	362	358	357	18	35	0	2	1
4748	13028	9454	739	550	318	28126	1157	6	636	0	24	58	4	92	277.21	97	434.28	342	326	327	18	35	0	4	1
4692	13164	9518	762	426	256	28090	1176	12	660	1	32	60	7	82	274.37	107	430.79	341	328	322	13	45	1	2	1
12818	7716	4870	3	1560	1124	28260	566	185	760	66	150	204	87	234	294.46	164	451.09	476	249	428	56	108	14	18	12
11836	7906	5212	1	1824	1386	28452	570	114	675	25	270	161	64	242	299.29	134	452.72	494	233	414	74	96	5	18	17
4214	13390	9756	888	450	256	28088	1277	7	582	1	22	64	2	78	273.91	80	428.57	289	304	268	15	43	0	0	3
4766	13076	9474	736	490	272	28098	1175	3	658	0	20	64	3	80	276.97	98	433.07	340	344	316	16	48	0	0	3
4610	13224	9538	807	430	262	28084	1201	10	643	1	18	54	7	81	275.61	95	428.98	312	317	327	19	36	0	2	1
5004	12850	9312	701	588	342	28128	1136	11	655	2	30	76	8	95	279.78	100	436.33	354	327	313	20	48	0	2	1
4106	13518	9826	911	394	220	28076	1273	7	581	0	12	59	4	75	274.86	95	428.57	290	293	278	17	36	0	0	1
5508	12520	9004	648	668	408	28142	1140	27	661	4	30	72	19	102	281.64	93	439.27	347	341	307	23	51	0	4	0
11952	7962	5240	1	1722	1276	28412	548	130	724	24	242	175	73	243	302.35	155	455.74	489	218	401	82	115	6	18	18
4046	13638	9906	945	290	150	28038	1294	7	597	1	6	64	3	61	270.39	82	423.87	282	304	277	14	44	0	2	3
11210	7762	5348	0	2198	1744	28700	494	91	528	17	380	162	50	280	316.67	111	462.61	522	163	369	110	81	9	54	21
11472	8008	5448	4	1910	1388	28516	572	112	641	30	252	179	65	257	309.13	116	460.27	468	231	388	90	87	5	34	19
7378	11176	7850	362	1052	718	28262	959	36	727	8	84	99	21	137	289.06	133	444.75	430	380	335	26	59	1	4	8
4504	13280	9596	832	440	254	28082	1212	5	624	0	6	52	3	75	276.5	102	430.81	318	307	319	17	32	0	2	1
4596	13182	9538	789	498	282	28106	1209	5	629	0	10	58	3	87	278.5	109	434.18	333	321	293	8	39	0	0	3
4262	13444	9748	913	380	218	28068	1263	7	596	0	16	59	5	65	274.11	102	427.76	306	291	284	16	40	0	0	1
4698	13176	9546	769	434	216	28072	1205	6	674	1	2	61	3	86	276.99	101	431.48	319	353	309	12	46	0	0	0
14370	7464	4354	6	982	680	27914	638	333	973	157	56	260	126	160	272.06	252	435.45	489	386	420	23	87	27	8	1
12602	7806	4842	1	1582	1260	28318	569	159	742	46	200	190	81	220	292.39	167	451.58	512	265	416	61	104	18	22	11
12430	7868	4988	2	1592	1210	28302	580	161	788	46	200	178	85	210	285.37	178	451.38	502	273	433	63	96	6	12	11
3982	13672	9916	975	296	166	28040	1305	10	579	1	8	58	7	59	270.27	99	422.79	285	274	269	11	38	0	0	4

SUPPLEMENTARY INFORMATION:Monte Carlo Atomistic Simulation and Machine Learning Analysis of Na-K Eutectic Alloy in Condensed Phases, D. Reitz and E. Blaisten-Barojas, George Mason University, Fairfax, VA 22030

5622	12560	8984	569	574	352	28126	1097	13	755	0	30	66	10	95	282.65	101	439.19	366	409	344	19	44	0	4	4
12640	7910	4998	2	1386	1078	28244	628	165	835	49	214	185	87	208	287.92	162	451.24	474	326	424	56	108	12	16	7
4232	13526	9792	898	314	174	28050	1249	8	598	0	12	64	6	71	273.03	100	427.27	303	289	297	12	40	0	0	1
4570	13202	9550	811	486	280	28100	1226	7	622	1	12	63	3	81	275.82	90	431.99	321	315	285	12	42	0	0	4
3994	13566	9880	947	388	224	28070	1300	10	559	1	16	57	5	79	273.63	68	427.22	265	285	277	15	39	0	2	2
4728	13162	9482	756	448	260	28092	1182	9	668	0	12	69	7	88	276.28	115	430.21	340	334	303	12	52	0	0	0
11008	7898	5462	0	2248	1712	28712	537	78	516	19	338	165	34	304	317.29	111	464.68	491	170	351	88	71	1	42	25
4470	13318	9624	818	408	242	28080	1200	2	634	0	18	64	1	74	274.95	95	430.26	311	316	323	25	44	0	0	1
4098	13572	9848	944	342	188	28056	1302	8	603	1	8	54	5	54	270.24	91	422.3	292	304	277	15	35	0	0	2
5156	12806	9226	659	574	336	28118	1125	10	673	2	18	58	4	71	278.59	109	434.85	375	356	333	25	33	0	2	2
14174	7792	4416	10	808	644	27898	656	310	1035	158	62	248	112	159	271.84	245	434.39	468	431	444	20	80	21	2	1
4472	13402	9670	849	322	174	28046	1240	10	649	2	6	71	5	77	273.08	95	426.77	298	323	298	9	50	0	0	4
3858	13802	9998	1001	232	130	28026	1319	10	591	1	6	50	5	62	270.48	89	422.49	269	278	280	11	34	0	0	1
4658	13002	9494	734	616	342	28140	1159	3	638	0	28	57	2	89	278.82	81	433.9	339	328	321	26	42	0	0	1
4074	13610	9834	915	322	204	28060	1274	7	595	2	16	62	4	78	271.32	87	425.72	271	305	292	15	42	0	0	4
5232	12726	9170	639	618	370	28146	1110	6	699	0	26	66	6	86	277.38	102	434.66	359	366	342	31	48	0	4	2
4762	13186	9482	777	388	234	28072	1197	9	681	1	20	56	5	79	275.4	111	429.72	327	346	318	12	39	0	0	2
4676	13108	9510	768	516	280	28100	1192	11	642	1	10	68	7	77	276.82	104	430.77	353	323	291	12	46	1	0	1
12372	7918	4948	1	1564	1284	28346	612	139	779	42	224	182	71	242	287.63	158	447.51	476	297	415	50	97	11	36	12
5390	12558	9046	640	706	428	28158	1115	17	634	2	30	78	9	113	281.35	87	440.23	354	326	310	20	55	0	0	2
13708	7788	4576	8	1044	796	28010	648	270	969	138	92	240	91	179	274.35	215	439.26	480	401	406	31	87	17	4	2
12810	7936	4826	1	1380	1098	28228	619	174	819	46	160	192	100	220	286.64	168	449.8	483	318	421	46	105	6	16	3
4386	13308	9650	807	468	270	28096	1205	7	613	1	14	63	2	84	275.29	91	429.45	324	303	300	16	43	0	0	1
13476	7828	4670	3	1118	846	28052	636	251	948	101	106	212	123	165	278.68	217	441.37	494	390	445	38	83	8	8	2
11470	7988	5402	2	1912	1448	28548	558	94	648	18	282	186	52	290	310.74	107	457.94	458	231	373	80	110	10	42	23
11352	7880	5328	1	2064	1624	28632	527	90	580	14	340	175	40	288	317.26	108	465.52	448	196	364	119	92	13	40	23
13194	7754	4740	1	1320	990	28152	609	212	877	75	140	211	101	213	281.87	201	444.58	490	324	416	36	108	13	12	6
14450	7672	4366	3	764	562	27864	675	336	1069	166	46	249	128	140	269.95	265	432.13	464	452	438	24	74	20	4	2
5122	12794	9224	661	606	364	28138	1133	11	699	1	28	63	8	99	279.56	129	435.24	352	341	326	19	38	0	0	1
4560	13260	9612	849	412	216	28070	1258	12	654	1	10	68	6	78	276.13	96	428.56	299	328	273	15	49	0	0	1
4236	13454	9756	921	390	222	28070	1279	7	610	1	12	47	5	80	274.81	95	428.17	278	291	300	10	32	0	0	1
11940	7852	5170	0	1808	1382	28446	593	116	684	26	264	167	64	274	303.29	127	457.67	468	253	385	69	97	10	30	10
4988	12928	9332	706	536	308	28112	1167	8	652	0	20	70	6	96	276.99	98	434.07	344	343	300	13	47	0	0	1
3850	13726	9970	997	312	182	28052	1353	3	577	0	12	69	3	62	271.14	74	424.55	258	305	236	16	45	0	0	2
4018	13576	9860	928	382	224	28074	1269	5	579	1	14	50	2	67	272.5	86	426.77	298	272	288	19	30	0	0	0
14354	7644	4356	9	840	630	27880	635	318	1039	161	52	256	123	146	270.35	277	432.81	507	421	434	12	83	13	4	3

SUPPLEMENTARY INFORMATION:Monte Carlo Atomistic Simulation and Machine Learning Analysis of Na-K Eutectic Alloy in Condensed Phases, D. Reitz and E. Blaisten-Barojas, George Mason University, Fairfax, VA 22030

12774	8102	4988	13	1210	916	28126	626	185	939	64	120	207	93	207	282.37	203	447.15	477	357	435	31	114	12	16	6
13296	7710	4638	0	1338	1024	28152	615	205	870	70	132	202	110	225	281.06	183	446.56	438	305	457	50	110	14	14	2
11280	7950	5400	0	2062	1574	28612	493	102	575	15	306	172	55	297	317.73	110	461.34	486	190	384	102	83	6	32	20
14484	7456	4310	6	920	654	27894	633	319	1030	163	64	264	121	162	272.71	260	436.77	471	406	436	24	87	18	6	3
4470	13268	9588	797	480	286	28104	1221	3	629	0	12	53	1	80	274.23	95	428.21	308	324	311	20	39	0	0	2
5004	12910	9306	689	550	328	28122	1147	8	674	1	22	62	4	90	278.74	104	435.44	353	351	320	16	45	0	2	3
6332	11814	8470	472	922	604	28222	981	24	672	6	78	80	15	119	284.12	100	442.72	415	344	360	34	47	1	2	3
14330	7776	4392	12	738	576	27862	632	324	1096	152	46	253	137	145	269	296	431.47	500	441	449	15	89	16	4	0
14246	7678	4368	5	850	682	27910	656	323	1038	162	80	254	118	162	272.04	243	434.01	468	440	428	24	79	21	6	1
13058	7928	4794	6	1262	960	28128	591	206	882	70	114	191	108	200	282.29	189	446.29	473	321	480	44	92	10	12	7
5518	12602	9024	546	618	360	28136	1058	12	751	0	14	69	5	92	281.6	111	436.56	403	400	345	15	48	0	0	6
14178	7778	4474	10	804	596	27880	651	312	1075	162	40	261	117	149	269.72	270	431.94	489	441	428	17	88	14	10	1
13764	7748	4564	4	1054	790	28018	644	260	979	119	88	238	111	198	277.97	236	441.88	469	400	412	24	100	16	8	2
13310	7602	4612	1	1388	1088	28196	583	212	795	69	180	187	99	203	284.29	156	449.83	493	293	451	50	89	20	14	9
5014	12984	9336	681	468	278	28106	1135	5	706	0	26	67	4	81	278.46	107	434.49	372	368	325	14	45	0	0	1
13544	7852	4668	3	1056	792	28006	629	256	982	125	82	238	103	184	276.09	222	439.74	466	376	434	37	94	14	12	1
11648	7884	5368	1	1878	1398	28506	553	112	666	18	296	170	62	274	310.74	121	459.91	480	244	389	77	95	9	28	20
11256	7926	5456	2	2016	1554	28598	531	90	592	11	356	172	43	276	313.57	111	454.75	467	186	384	95	100	10	34	32
4840	12898	9374	687	642	372	28156	1137	5	641	0	28	49	4	84	279.57	102	436.71	369	319	338	17	31	0	2	2
5706	12304	8900	543	778	452	28182	1068	15	712	0	42	76	11	112	283.84	103	440.39	388	378	324	18	48	1	0	5
11692	7882	5302	2	1878	1426	28510	515	126	588	27	298	178	60	276	312.27	100	460.97	483	199	392	99	97	11	28	12
14598	7694	4290	5	684	526	27826	637	353	1095	173	30	272	129	144	268.57	279	430.39	490	436	433	15	91	23	4	1
11688	7934	5302	5	1900	1392	28486	564	121	618	21	246	167	75	283	306.47	104	457.68	451	223	397	86	91	5	20	12
5616	12546	8962	541	622	376	28144	1055	10	714	1	20	58	6	92	282.44	120	439.07	413	388	359	14	41	0	2	1
8404	10532	7316	240	1110	772	28250	812	64	783	16	106	114	37	159	289.64	145	447.51	464	352	391	37	65	2	10	7
3970	13630	9882	942	358	216	28068	1299	8	574	1	12	50	4	77	270.22	86	423.47	265	293	283	16	31	0	0	2
13506	7904	4758	11	1000	728	27992	633	263	989	127	84	228	102	182	277.39	229	443.37	472	396	436	28	83	13	10	4
11402	7788	5354	1	2080	1584	28578	489	92	614	25	328	182	47	278	314.16	107	458.28	479	183	399	107	95	5	42	23
3964	13510	9848	909	476	286	28104	1251	6	520	1	18	58	3	87	273.14	70	426.15	286	265	295	16	34	0	2	4
11606	8020	5342	0	1822	1404	28510	525	100	675	24	278	173	52	266	312.66	158	460.13	513	211	384	79	100	6	28	18
5530	12396	8972	621	762	458	28160	1071	24	628	10	40	73	10	112	281.43	90	439.04	375	327	338	22	39	1	2	1
4088	13624	9844	924	302	182	28050	1289	7	601	0	10	66	5	71	272.46	89	426.35	272	304	282	16	48	0	0	2
4092	13596	9848	940	322	184	28050	1283	5	609	1	6	67	3	65	271.78	98	424.94	297	298	268	13	48	0	2	1
14450	7672	4370	5	742	562	27860	631	325	1099	148	58	255	136	141	270.37	292	434.51	514	435	436	11	104	21	6	2
11746	7978	5364	5	1770	1290	28426	569	123	711	28	260	154	64	257	300.73	138	455.22	478	262	415	74	84	7	18	14
11950	7868	5214	0	1790	1334	28440	533	123	690	30	262	189	55	257	309.26	140	458.57	491	215	376	87	104	9	22	12

SUPPLEMENTARY INFORMATION:Monte Carlo Atomistic Simulation and Machine Learning Analysis of Na-K Eutectic Alloy in Condensed Phases, D. Reitz and E. Blaisten-Barojas, George Mason University, Fairfax, VA 22030

14274	7586	4370	6	928	682	27902	632	338	1010	169	56	272	121	166	271.22	250	434.82	474	397	424	22	93	25	4	4
7410	11152	7860	353	1046	692	28238	884	49	733	10	74	98	29	145	288.71	136	446.76	456	350	370	30	58	0	4	3
14486	7638	4354	3	756	556	27842	643	326	1089	166	50	260	119	150	268.74	268	431.43	481	443	438	16	89	21	2	1
14380	7626	4344	5	842	636	27892	645	333	1024	149	58	246	139	141	270.82	254	434.85	490	428	441	25	86	25	6	2
13258	7796	4674	5	1286	986	28132	576	215	856	65	120	195	108	180	279.48	196	446.16	513	316	467	47	96	19	10	6
4268	13462	9728	893	368	224	28064	1276	5	628	1	14	60	3	66	271.69	96	425.35	298	309	278	14	38	0	0	2
3952	13614	9880	949	374	236	28078	1303	7	582	1	20	49	4	65	271.34	95	424.39	283	286	278	13	31	0	2	3
10988	7772	5546	0	2280	1712	28736	503	76	495	13	386	153	46	280	324.29	89	467.17	482	142	402	118	79	7	46	25
12014	8038	5164	0	1670	1274	28396	566	128	728	29	214	164	72	234	297.86	155	453.71	507	250	420	67	95	9	22	14
5648	12520	8970	581	616	356	28130	1062	10	725	4	20	69	3	94	282.86	110	436.61	397	370	351	18	44	0	0	1
4830	13124	9444	749	428	246	28086	1173	7	684	1	14	62	4	84	277.75	120	432.96	334	348	326	15	40	0	0	1
5746	12402	8936	561	664	364	28134	1047	22	714	9	20	76	12	92	281.9	104	437.79	410	374	349	20	47	1	2	0
13572	7806	4632	1	1092	826	28038	630	251	949	120	108	232	99	185	276.84	217	443.47	476	376	431	34	100	11	2	3
4046	13558	9872	943	374	202	28062	1286	7	574	2	8	56	4	76	271.43	87	423.91	280	266	281	11	40	0	2	3
14340	7720	4402	9	760	580	27864	658	328	1077	166	62	262	120	162	270.39	267	434.64	462	444	431	18	85	18	0	0
4096	13564	9814	927	346	226	28074	1276	3	596	0	28	55	3	77	273.58	105	427.85	289	298	288	10	35	0	0	2
4678	13156	9496	768	474	282	28106	1197	5	659	0	18	65	5	89	277.39	96	432.46	320	336	311	16	39	0	2	1
12548	7868	4960	2	1550	1172	28304	580	150	763	36	186	182	78	224	300.3	179	452.39	504	276	413	61	96	8	18	8
11678	7856	5316	1	1926	1416	28490	559	114	645	26	264	168	58	254	309.31	125	458.9	492	229	381	89	101	10	32	15
3994	13688	9908	943	284	162	28042	1277	7	591	1	6	65	4	74	272.16	84	424.61	281	284	286	11	46	0	0	2
5348	12600	9100	662	666	394	28138	1121	22	611	6	26	65	11	108	281.28	85	439.42	345	332	334	18	38	0	4	1
13462	7862	4676	8	1100	836	28044	623	259	933	116	102	225	108	186	277.56	232	442.49	498	356	423	28	90	14	6	2
5094	12852	9276	721	552	318	28116	1180	13	668	4	24	64	6	82	278.97	93	435.68	339	359	309	19	41	0	0	2
3784	13744	10020	1009	308	174	28048	1309	7	549	1	16	57	5	77	271.05	86	423.88	252	249	283	14	34	0	2	3
11882	7888	5186	1	1814	1392	28456	565	130	661	34	266	178	65	252	297	135	453.26	518	245	374	65	96	4	28	17
11536	8008	5348	0	1900	1420	28494	550	101	642	19	256	170	48	275	305.26	135	456.14	483	214	384	86	86	9	24	10
3914	13632	9910	970	374	224	28070	1287	5	550	2	16	69	2	82	270.5	85	423.43	273	254	273	13	43	0	0	2
4676	13122	9492	765	512	292	28106	1196	6	646	0	12	69	1	77	276.75	89	431.1	338	337	295	18	44	0	0	2
10922	8080	5590	2	2104	1586	28642	558	84	564	13	326	152	50	292	314.65	99	460.82	449	199	369	113	74	4	32	16
13454	7654	4558	0	1310	1020	28148	593	225	838	75	138	206	107	205	282.39	180	444.4	492	310	439	44	105	20	12	4
5064	12868	9264	660	576	340	28130	1156	9	689	1	18	61	7	89	277.15	97	434.35	347	382	319	17	36	0	0	4
4598	13212	9560	768	448	254	28088	1225	6	665	1	16	68	2	67	276.98	103	432.23	329	370	285	17	42	0	0	2
4266	13382	9730	842	450	248	28086	1211	4	602	0	10	63	3	74	274.76	99	429.56	328	286	301	12	36	0	0	5
13254	7710	4666	4	1336	1032	28166	613	229	818	75	158	204	119	194	282.85	196	447.75	493	302	427	49	94	17	10	5
11378	7804	5310	2	2044	1646	28608	553	101	589	15	392	174	65	269	313.87	115	460.19	477	210	363	103	93	7	28	24
12744	7874	4934	3	1424	1072	28244	588	175	814	53	184	180	91	207	292.16	162	452.08	475	305	451	68	95	8	12	8

SUPPLEMENTARY INFORMATION:Monte Carlo Atomistic Simulation and Machine Learning Analysis of Na-K Eutectic Alloy in Condensed Phases, D. Reitz and E. Blaisten-Barojas, George Mason University, Fairfax, VA 22030

12718	7774	4888	3	1540	1150	28266	592	177	752	54	184	195	85	237	293.02	149	449.99	478	280	407	56	102	9	12	7
11242	7874	5408	3	2106	1632	28664	510	85	554	16	362	160	39	284	317.93	119	463.11	507	182	382	94	80	6	36	23
5686	12504	8932	592	614	368	28124	1079	24	708	7	20	72	13	105	281.7	112	438.65	381	350	335	15	41	0	0	1
10772	8022	5610	2	2200	1704	28734	503	61	524	8	376	168	37	302	327.64	109	470.96	483	156	356	112	91	3	48	27
10668	7908	5718	0	2282	1724	28774	483	59	499	6	428	182	32	315	333.6	74	469.56	447	165	361	121	93	5	46	39
6750	11638	8316	397	892	546	28206	987	23	798	4	64	79	15	127	287.78	122	443.92	419	426	349	25	47	0	0	1
4234	13472	9758	891	372	218	28070	1259	8	579	1	16	57	4	79	273.79	81	425.81	298	297	290	8	40	0	0	3
11170	7924	5580	0	2106	1494	28614	503	78	565	11	314	176	44	289	316.83	114	467.71	493	175	378	100	93	4	26	18
4762	13102	9472	740	488	270	28108	1169	5	661	0	14	54	5	93	277.93	112	432.85	328	332	337	16	34	0	0	0
7048	11474	8146	355	936	562	28210	905	27	784	3	42	71	19	116	287.07	134	447.63	463	394	399	27	44	0	2	5
4884	13018	9400	734	500	286	28108	1191	9	682	1	18	58	6	79	278.86	101	435.8	326	359	320	23	36	1	2	0
4966	12964	9324	684	526	320	28124	1130	5	672	1	24	65	2	102	279.34	98	435.36	356	344	325	13	43	0	0	1
14304	7788	4416	11	726	568	27858	671	328	1091	161	54	255	125	134	269.98	294	432.87	492	453	419	18	81	17	2	1
12706	7774	4870	3	1566	1172	28276	559	179	734	61	168	204	89	235	292.51	158	449.81	504	247	405	56	91	11	20	9
11326	7982	5408	4	1966	1528	28574	531	97	610	17	334	156	49	294	315.07	122	458.72	486	184	387	92	89	4	28	12
12388	7952	5026	2	1536	1186	28306	590	156	772	42	192	188	82	227	291	159	450.8	482	298	407	73	93	12	26	4
12860	7834	4938	3	1390	1000	28186	586	187	867	62	146	201	98	211	284.03	186	447.53	484	311	439	54	103	7	18	4
4070	13594	9848	943	332	198	28056	1286	9	573	0	14	61	5	70	272.06	91	425.36	281	278	277	17	43	0	0	1
4562	13252	9584	795	418	244	28082	1185	6	641	1	22	57	3	72	276.46	104	431.7	333	316	325	19	41	0	0	2
5206	12686	9158	633	654	410	28162	1107	9	671	0	46	58	7	99	282.06	100	437.8	362	363	342	26	33	0	2	0
11240	7982	5438	3	2026	1556	28600	511	89	600	18	318	161	47	281	313.25	112	458.25	503	195	391	93	87	3	38	22
13418	7814	4698	7	1130	862	28050	621	254	927	117	114	225	105	176	275.95	221	443.05	494	350	434	35	92	12	14	3
5078	12858	9258	653	552	348	28136	1103	6	681	0	40	64	4	102	280	107	437.59	366	346	339	15	50	0	2	3
14382	7572	4332	8	894	662	27908	660	320	1010	160	60	256	113	156	271.21	237	434.7	481	430	415	23	85	28	6	0
14204	7770	4394	6	808	658	27910	663	328	1048	162	70	252	125	140	270.66	277	433.57	499	438	422	18	76	20	6	3
13132	7812	4760	5	1322	986	28154	603	203	853	72	138	190	107	201	283.73	193	447.19	491	307	459	40	92	13	4	4
14342	7668	4388	7	818	604	27872	627	343	1048	173	44	262	135	144	270.65	289	431.85	496	417	439	21	77	18	8	3
13416	7712	4678	3	1242	924	28114	571	235	872	85	126	222	118	190	284.71	196	449.16	497	325	438	54	109	9	14	6
4044	13582	9854	928	360	212	28066	1266	9	588	2	14	54	4	69	271.55	102	425.72	297	269	293	11	37	0	0	3
4484	13308	9606	830	414	256	28092	1231	6	615	0	22	65	2	73	275.46	101	431.92	321	325	286	19	37	0	2	0
13606	7872	4658	7	1010	766	28010	646	266	964	122	86	222	112	175	276.75	224	442.48	492	408	425	23	84	17	10	2
4404	13412	9672	834	362	210	28068	1231	9	641	1	8	58	4	65	274.76	98	428.6	315	331	302	19	37	0	0	2
7734	10956	7660	287	1078	742	28266	853	42	782	8	86	90	28	155	287.85	149	445.46	472	364	380	29	57	1	10	3
3880	13700	9956	976	324	184	28054	1301	6	566	1	10	57	4	58	271.17	87	423.32	284	274	281	13	33	0	0	4
11228	7828	5462	1	2130	1596	28632	503	81	580	16	354	175	38	296	318.81	120	459.2	488	185	370	99	98	1	34	22
12322	7828	5032	1	1652	1286	28402	552	152	721	39	256	175	77	235	301.16	151	454.49	509	257	419	73	84	9	24	10

SUPPLEMENTARY INFORMATION:Monte Carlo Atomistic Simulation and Machine Learning Analysis of Na-K Eutectic Alloy in Condensed Phases, D. Reitz and E. Blaisten-Barojas, George Mason University, Fairfax, VA 22030

4348	13370	9686	869	416	244	28082	1230	10	612	3	18	68	2	71	274.62	119	430.29	327	284	279	16	48	0	0	1
3986	13630	9896	937	340	194	28056	1257	7	568	1	10	69	5	75	273.97	83	427.43	303	268	280	8	46	0	0	3
4490	13298	9622	812	420	238	28082	1196	4	651	1	14	70	2	75	276.09	101	430.17	332	322	303	17	44	0	0	2
4178	13446	9774	895	428	242	28080	1255	5	579	1	12	67	2	74	274.1	89	428.86	307	282	276	14	44	0	0	2
13374	7824	4672	2	1182	914	28102	599	228	891	75	130	200	116	211	280.79	193	446.22	469	323	465	36	95	16	6	4
11080	7668	5436	1	2322	1774	28736	517	76	481	16	416	161	34	291	323.47	82	467.4	470	155	357	109	73	8	40	37
4214	13636	9806	905	224	138	28026	1246	10	636	1	8	61	6	63	272.08	114	424.97	296	294	311	13	37	0	0	4
4944	13032	9382	714	462	262	28098	1136	3	713	0	16	53	1	76	275.81	113	432.86	354	340	356	17	42	0	0	4
4808	12936	9416	707	602	342	28134	1151	12	642	0	26	53	10	97	280.52	86	434.48	336	331	334	16	32	0	4	5
4358	13390	9710	867	396	210	28070	1280	6	649	1	6	63	4	58	273.27	95	427.01	302	342	274	19	40	0	0	1
5212	12750	9152	636	626	386	28150	1121	4	682	0	24	62	4	98	279.86	118	438.55	370	350	328	13	41	0	0	3
10846	7808	5656	0	2302	1692	28736	520	62	494	12	374	179	31	276	330.55	86	471.76	475	151	345	115	93	4	50	40
11026	7694	5484	0	2302	1776	28758	474	79	470	13	416	179	40	311	330.58	87	474.86	479	144	336	123	79	4	52	35
4770	13072	9448	711	514	288	28100	1157	8	670	0	8	59	4	73	274.83	101	429.27	357	353	328	19	37	0	0	1
11788	7998	5140	3	1824	1454	28488	544	130	622	25	250	147	75	235	302.59	134	454.54	515	213	433	85	64	6	30	13
12208	8012	5062	3	1614	1244	28352	581	147	767	42	188	178	76	238	293.48	167	451.77	501	292	414	59	95	15	22	8
12910	7908	4906	0	1330	976	28186	610	193	859	52	148	182	112	205	287.83	197	448.19	479	311	459	43	102	12	8	7
4170	13562	9802	901	324	186	28048	1259	8	603	1	4	68	3	78	272.28	96	426.5	280	295	292	17	38	0	0	1
6176	11966	8614	450	882	524	28208	975	16	730	2	44	86	6	110	285.08	119	442.74	439	358	350	25	55	1	2	6
13922	7642	4480	4	1054	790	27986	678	298	963	149	96	233	111	182	275.48	199	439.98	451	420	415	27	72	20	2	2
12406	7898	5050	5	1580	1172	28316	574	156	762	37	188	172	86	247	298.82	141	451.93	460	270	447	57	102	9	22	15
3802	13678	9952	1004	396	238	28078	1340	8	520	1	12	43	4	69	270.94	66	423.19	249	263	275	16	29	0	0	4
4576	13074	9506	740	600	356	28140	1179	5	611	0	28	62	3	81	278.25	84	434.33	346	318	301	24	44	0	0	1
13154	7918	4816	2	1194	898	28116	647	207	924	74	122	189	109	197	280.62	188	444.73	467	375	447	33	94	9	14	6
12026	8008	5172	2	1686	1266	28388	563	144	715	33	208	159	84	237	300.23	152	453.97	498	225	424	79	91	4	22	4
3922	13692	9948	946	310	168	28046	1294	6	581	0	6	63	4	63	270.88	89	423.66	289	287	272	11	40	0	0	3
12164	7786	5086	2	1782	1346	28446	605	145	662	32	256	162	74	251	305.03	127	457.01	448	241	411	79	88	13	24	16
5188	12780	9204	647	594	348	28136	1114	7	710	0	20	55	3	85	281.43	122	437.93	372	343	346	20	36	0	2	2
4166	13516	9760	892	386	240	28078	1271	7	582	1	10	58	6	79	272.21	84	425.9	286	305	288	13	33	0	0	2
4552	13210	9576	826	462	270	28096	1236	6	643	0	26	52	2	74	275.02	103	430.92	325	331	286	11	38	0	0	4
4694	13082	9484	745	536	302	28114	1169	6	637	0	16	55	6	89	277.85	94	433.75	332	330	331	16	41	0	0	3
13352	7672	4632	0	1318	1014	28154	614	225	876	75	158	196	109	208	282.26	197	446.11	483	333	438	40	92	15	8	4
13502	7574	4626	2	1318	960	28132	564	244	836	90	140	223	119	218	281.55	175	447.22	492	318	447	36	103	16	12	7
13922	7644	4504	3	1052	772	27990	612	291	949	132	94	234	120	172	276.45	233	442.39	486	366	457	29	85	17	2	4
5306	12744	9146	623	568	338	28122	1110	8	709	2	18	69	3	98	279.54	109	435.8	366	379	330	16	45	0	0	2
6310	12006	8546	457	780	494	28186	1006	22	770	5	46	82	9	116	286.2	129	441.3	418	389	343	20	59	1	4	4

5086	12788	9220	680	638	390	28150	1151	11	640	2	26	63	2	105	281.48	98	437.58	335	336	313	19	42	0	2	3
13426	7828	4690	5	1146	856	28056	659	244	939	105	102	214	106	188	277.34	205	442.93	455	383	426	40	93	13	8	2
5056	12794	9286	666	622	350	28136	1136	6	674	1	28	62	4	89	278.77	87	435.59	370	355	321	13	35	0	0	3
11458	7884	5452	1	2026	1428	28544	540	97	629	18	268	156	47	262	319.28	125	462.36	454	214	407	111	92	6	28	20
13724	7822	4622	12	974	730	27960	632	282	997	130	80	236	110	166	274.4	243	438.22	489	397	435	24	96	15	8	4
5008	13016	9370	679	436	242	28090	1125	2	740	0	16	57	1	95	278.14	109	435	342	375	359	15	42	0	2	1
7226	11318	8056	404	938	582	28192	916	44	743	11	70	87	26	155	287.44	113	447.73	391	348	389	38	48	0	2	2
13374	7682	4720	5	1290	908	28096	599	218	889	73	118	207	109	204	281.09	179	446.29	474	334	456	42	113	13	4	2
6440	11990	8528	472	726	432	28148	994	29	766	4	30	88	20	125	288.42	114	443.35	395	383	363	21	55	1	2	3
14062	7684	4456	6	928	716	27940	635	321	980	151	90	255	127	185	274.96	227	439.37	452	406	432	28	85	20	2	1
6840	11704	8338	419	768	454	28158	976	42	794	7	52	84	28	101	287.94	130	444.28	413	397	379	35	58	3	2	2
5730	12354	8848	525	742	468	28184	1036	15	711	3	38	76	10	114	283.7	120	438.58	412	374	346	15	42	1	4	0
3900	13634	9930	981	382	214	28070	1318	5	554	1	10	64	3	74	271.74	80	425.55	267	269	257	12	46	0	0	4
3912	13638	9938	963	364	200	28064	1281	7	561	1	12	52	3	73	270.98	82	424.14	280	266	293	14	37	0	0	2
12414	7788	5010	1	1650	1240	28346	549	154	718	48	228	185	69	245	297.06	156	452.27	493	253	427	63	87	12	16	10
4578	13176	9528	782	522	300	28110	1171	5	638	0	6	72	4	79	275.11	116	431.98	354	305	303	17	47	0	0	1
12658	7672	4948	1	1626	1172	28290	589	183	765	67	198	209	80	219	288.49	158	448.95	484	276	395	79	105	14	16	3
6970	11392	8118	407	1004	664	28240	933	28	709	7	82	101	18	151	288.08	111	444.3	411	333	364	26	71	0	10	7
4874	12980	9414	742	548	288	28112	1193	12	639	0	6	51	10	97	278.52	83	434.38	314	329	322	15	41	0	2	1
11098	7832	5624	2	2144	1552	28660	513	94	542	20	376	162	47	278	321.44	127	466.51	491	161	374	101	79	2	32	28
11008	7936	5506	3	2110	1686	28698	509	75	536	9	394	168	40	323	319.15	101	461.72	493	192	349	96	85	4	52	19
12694	7882	4942	0	1456	1096	28262	567	171	784	49	168	185	97	251	292.38	174	450.76	456	281	451	51	97	8	22	12
5634	12576	8956	578	590	356	28124	1069	13	734	3	12	68	6	93	282.24	138	439.13	402	368	346	13	41	0	0	1
14530	7626	4306	6	752	582	27862	679	325	1093	154	62	268	125	144	271.54	278	434.19	470	457	415	20	100	23	4	1
6302	11834	8504	456	938	586	28226	988	25	676	8	56	80	12	129	283.53	86	441.87	416	351	351	25	45	2	6	3
7738	11010	7720	274	1028	668	28238	855	32	820	9	68	94	11	128	287.43	147	444.08	495	371	381	26	60	3	6	5
10548	7798	5708	0	2496	1828	28830	532	56	471	12	406	162	29	296	333.2	84	471.37	486	157	329	130	73	1	44	27
4164	13502	9782	900	372	232	28076	1279	5	596	1	22	58	3	75	271.47	85	425.63	279	314	285	20	34	0	0	0
11368	7910	5404	2	2068	1520	28590	506	100	573	22	292	177	52	272	316.59	134	460.93	536	168	364	86	86	6	28	18
4726	13038	9454	724	560	326	28130	1158	4	643	1	26	60	2	85	277.77	92	433.09	349	334	323	18	40	0	0	3
10714	7824	5704	1	2282	1750	28790	507	64	501	6	458	160	32	286	333.28	89	469.55	471	163	356	130	81	4	58	36
4116	13564	9838	916	344	188	28056	1259	6	610	0	6	64	5	68	271.87	100	424.96	296	287	291	17	45	0	0	1
4448	13210	9620	780	518	294	28116	1182	6	617	0	26	51	4	83	277.27	88	432.83	341	309	324	12	37	0	0	3
14088	7748	4492	4	882	638	27898	652	324	1017	154	46	250	129	161	270.54	244	434.41	471	412	438	20	86	25	4	1
11318	7878	5340	0	2064	1622	28608	539	97	589	16	358	167	52	276	312.45	110	461.21	494	207	352	101	90	6	28	22
13124	7744	4666	6	1368	1106	28200	565	204	807	66	170	212	102	241	284.28	167	448.53	474	288	438	47	115	13	22	5

11144	8010	5540	2	2000	1528	28604	516	78	590	17	340	179	38	278	315.46	114	460.9	484	173	388	102	102	7	40	17
4334	13404	9710	837	394	220	28072	1211	5	617	0	10	57	3	81	274.52	90	427.8	315	311	315	12	38	0	0	1
5758	12518	8932	604	542	324	28100	1097	27	761	5	26	66	17	114	282.6	105	438.99	330	395	363	19	43	0	0	2
5838	12254	8812	552	772	458	28166	1070	20	676	4	32	73	12	114	285.63	93	442.1	376	362	333	18	45	0	0	5
11538	7880	5250	0	1984	1570	28590	560	103	596	21	340	178	53	274	312.15	121	459.45	466	205	357	100	92	6	28	23
12466	7790	4996	2	1612	1222	28328	586	149	764	37	216	158	83	208	290.78	177	451.32	499	277	431	73	85	9	24	11
3970	13634	9928	993	332	174	28046	1318	8	574	2	8	57	3	71	269.88	85	422.43	258	279	275	14	42	0	0	2
12540	7976	5016	1	1460	1094	28276	597	161	817	38	178	173	93	235	285.28	177	448.05	486	276	437	49	106	16	10	4
4954	12854	9320	699	624	362	28142	1166	6	652	1	28	51	4	84	281.02	110	436.29	359	338	309	15	32	0	0	3
14378	7686	4398	4	798	566	27862	628	330	1073	163	34	269	136	150	268.12	279	429.11	498	415	435	15	94	13	2	1
5104	12778	9268	661	626	344	28142	1124	6	675	0	20	57	5	81	279.29	108	436.09	390	353	331	13	45	0	2	0
5132	12830	9276	665	542	306	28116	1125	3	689	0	30	70	1	91	280.6	108	438.24	354	352	327	22	53	0	0	2
14698	7570	4278	4	722	514	27818	654	338	1105	182	34	291	120	152	268.17	286	429.33	481	442	406	8	101	18	2	3
11900	7770	5114	0	1898	1498	28520	561	136	591	36	300	158	68	244	303.65	124	456.02	505	214	404	83	78	6	36	17
13502	7878	4746	8	1056	740	27996	646	264	980	130	64	239	101	184	276.52	230	444.55	477	392	414	25	86	15	10	2
14322	7570	4372	4	900	664	27904	623	355	985	154	70	245	158	152	273.06	252	438.16	482	400	463	27	76	20	6	2
4714	13110	9486	761	490	284	28108	1171	7	658	0	24	44	3	83	276.83	112	432.67	332	324	343	18	31	0	0	2
3934	13766	9956	995	234	132	28026	1320	9	586	1	4	56	5	56	271.4	100	423.9	271	279	275	15	42	0	0	1
6198	12234	8706	492	602	352	28124	1009	15	817	2	32	75	11	108	283.94	135	440.62	401	404	380	15	52	0	0	4
4696	13150	9528	810	462	242	28086	1215	9	634	1	8	65	7	77	277.75	105	433.54	329	319	298	12	42	0	0	1
14242	7626	4422	4	894	654	27914	651	313	1027	148	74	240	130	160	271.34	266	436.65	470	418	453	19	77	20	2	3
10610	7876	5692	1	2294	1804	28796	512	68	500	8	452	166	35	304	328.63	93	470.83	476	161	331	117	74	7	64	31
12084	8008	5194	1	1632	1214	28360	604	130	771	31	206	155	74	234	294.29	142	451.83	470	286	437	64	84	7	18	11
10948	7916	5640	1	2136	1608	28684	520	77	538	10	392	161	47	278	325.67	101	466.31	473	157	380	115	81	6	44	28
13246	7742	4602	1	1312	1088	28184	627	229	835	82	180	201	112	228	282.2	175	446.09	468	340	419	36	86	13	14	4
3898	13726	9958	984	286	164	28042	1322	9	577	1	10	59	5	69	270.72	88	423.81	261	289	271	12	40	0	0	2
4558	13282	9606	801	392	218	28070	1245	6	682	0	12	75	4	65	276.02	107	431.76	319	364	274	15	51	0	0	3
4870	12812	9320	694	710	424	28170	1171	6	642	0	32	63	5	107	279.66	86	437.57	325	349	309	20	39	0	0	3
11666	7872	5258	1	1926	1478	28528	521	117	624	35	298	174	52	242	305.96	126	457.99	527	194	400	91	95	12	26	16
4512	13208	9582	787	506	288	28112	1197	8	617	0	14	56	6	86	279.4	104	432.89	329	310	314	14	36	0	2	1
4560	13304	9596	806	382	218	28072	1212	5	660	1	12	64	4	82	275.75	92	430.11	302	333	320	19	44	0	0	0
4952	12868	9308	707	616	372	28150	1126	9	654	1	34	60	5	87	280.66	108	437.81	371	315	326	20	39	0	0	3
14330	7676	4356	10	814	640	27888	632	334	1031	159	68	259	126	165	270.31	269	434.54	475	402	441	21	91	26	4	0
4644	13122	9520	780	516	292	28114	1180	10	615	0	16	48	7	80	277.8	97	432.16	346	310	323	16	31	1	4	0
4854	12982	9382	713	566	326	28124	1158	8	642	1	12	59	4	74	279.08	106	435.21	358	328	322	23	35	0	2	1
12732	7832	4854	4	1510	1152	28266	572	170	771	62	172	191	78	224	291.18	182	448.79	501	259	420	53	79	8	14	10

7370	11204	7912	341	1006	662	28238	867	36	763	5	78	95	20	120	288.95	155	443.56	491	346	378	28	64	1	6	8
4952	13014	9352	715	496	284	28104	1155	7	707	0	6	63	4	93	277.88	118	434.4	338	345	324	16	41	0	0	1
14056	7710	4490	3	914	678	27924	625	289	1028	156	72	259	96	177	272.99	242	436.94	467	390	431	28	88	19	4	0
3802	13684	9978	974	380	214	28068	1308	7	541	1	10	60	5	71	270.33	80	423.42	273	263	265	12	39	0	0	5
4466	13368	9634	797	374	220	28072	1187	5	655	1	10	66	3	78	273.61	100	427.56	336	321	313	13	37	0	0	1
11148	7978	5544	0	2058	1528	28610	526	88	563	18	312	167	46	274	318.87	120	462.64	501	172	372	87	80	3	30	30
7280	11344	8012	335	926	586	28214	929	40	805	9	62	94	24	120	289.4	136	447.03	433	419	372	37	57	2	4	5
4016	13638	9904	952	308	168	28044	1312	10	589	2	10	57	5	69	271.54	89	424.33	261	299	277	16	38	0	0	2
11266	7928	5568	1	2036	1444	28572	548	100	584	22	300	148	47	287	314.77	101	460.78	462	204	396	86	79	4	22	24
14458	7432	4276	5	968	704	27914	623	344	954	166	74	260	144	151	271.98	247	436.2	494	383	434	31	82	17	2	0
4818	12928	9402	734	614	348	28136	1191	9	642	0	26	61	7	89	277.86	87	434.28	333	355	298	18	46	0	0	3
4092	13494	9846	931	410	218	28074	1291	10	564	1	14	63	7	74	273.25	83	427.13	275	286	273	18	46	0	0	2
4808	13020	9394	711	568	328	28124	1135	8	621	0	6	61	4	75	278.58	97	434.13	369	319	328	23	36	1	0	2
14308	7628	4420	7	842	614	27884	624	342	1038	168	68	258	130	167	272.59	246	439.22	467	413	455	20	85	17	4	3
3926	13626	9916	948	378	212	28066	1283	4	556	0	8	56	3	68	271.6	86	423.31	283	273	292	14	34	0	0	3
5096	12854	9236	649	570	354	28140	1121	12	680	0	30	58	10	83	280.1	119	437.96	381	354	334	14	39	0	0	4
14416	7516	4356	2	908	638	27900	641	334	991	149	64	259	135	172	273.26	227	436.92	470	396	428	18	95	27	2	2
4186	13590	9806	926	286	168	28044	1275	8	638	2	8	72	6	64	271.94	110	426.94	293	300	276	16	48	0	0	1
11046	8038	5524	0	2036	1604	28654	524	82	594	17	352	166	49	296	315.48	119	461.1	464	176	386	101	96	7	50	27
14286	7662	4372	7	848	658	27902	620	319	1035	162	72	260	128	151	270.47	270	434.92	504	424	436	22	93	14	4	1
12868	8014	4914	6	1258	958	28160	584	190	866	50	132	180	102	207	282.76	173	448.05	478	308	479	47	108	12	16	5
12050	7906	5166	2	1742	1296	28412	586	142	693	43	230	163	69	234	300	160	454.26	526	245	394	60	78	10	20	10
12400	7790	5006	2	1678	1250	28356	531	172	664	44	212	187	85	250	297.84	166	452.34	512	213	418	58	98	19	20	13
10972	7928	5634	1	2180	1580	28668	496	71	545	17	326	186	24	311	324.08	99	463.83	446	165	368	119	89	5	44	23
12054	8056	5252	2	1596	1162	28336	561	126	811	30	200	167	70	222	293.85	180	452.19	487	257	448	79	98	10	16	7
14408	7786	4400	9	654	524	27832	657	334	1130	166	56	260	130	150	267.28	284	430.15	456	467	448	22	87	14	2	0
5112	12838	9274	645	552	320	28130	1134	8	695	1	34	61	4	85	280.8	105	437.91	364	376	329	19	42	0	0	2
4124	13386	9790	900	510	278	28102	1254	7	555	3	14	62	3	71	271.72	87	425.19	313	268	277	16	41	0	0	3
13712	7936	4630	5	902	704	27974	679	262	1048	122	88	232	103	171	278	231	442.19	461	447	416	28	93	14	2	1
3850	13720	9976	983	312	178	28048	1304	7	570	2	12	64	4	52	271.02	101	424.49	292	268	266	14	39	0	0	4
13234	7690	4654	2	1376	1062	28182	590	196	826	66	146	188	96	208	283.02	181	447.44	483	304	464	45	88	19	20	8
4718	13166	9510	737	424	246	28090	1180	4	673	0	26	63	2	87	276.62	97	431.56	323	360	324	14	40	0	0	4
3892	13696	9954	983	316	180	28050	1314	10	558	2	12	55	6	66	271.15	97	424.24	272	269	265	14	34	0	0	3
7392	11262	7932	332	942	612	28210	918	42	806	11	60	90	24	134	288.74	137	444.11	434	395	373	31	54	0	10	3
12320	7900	5000	0	1618	1272	28360	558	148	741	32	238	164	82	225	301.36	160	451.64	520	256	424	58	99	7	10	13
11450	7922	5386	2	1984	1480	28540	531	91	615	22	284	181	44	279	310.11	127	460.41	491	222	369	79	94	3	28	23

SUPPLEMENTARY INFORMATION:Monte Carlo Atomistic Simulation and Machine Learning Analysis of Na-K Eutectic Alloy in Condensed Phases, D. Reitz and E. Blaisten-Barojas, George Mason University, Fairfax, VA 22030

12718	7858	4942	2	1468	1080	28244	599	185	791	60	160	183	103	207	285.88	156	448.16	472	297	446	61	96	9	18	12
5534	12514	9020	567	660	386	28150	1076	15	723	6	36	64	9	90	281.2	104	438.63	393	388	348	21	41	0	0	1
12112	7826	5092	1	1740	1362	28438	565	135	708	28	278	174	69	255	300.26	133	456.17	494	248	386	62	101	12	26	24
14066	7674	4538	5	932	650	27938	602	271	1053	132	74	249	107	194	274.91	251	441.6	469	402	455	18	92	18	4	3
5442	12528	9022	603	708	434	28174	1082	12	672	2	40	65	8	103	282.65	117	439.72	384	354	338	19	39	0	0	2
4368	13366	9686	853	416	232	28078	1240	8	608	1	10	52	2	72	274.62	87	429.29	307	311	304	16	32	0	0	2
6758	11604	8212	363	968	644	28258	934	23	754	7	70	73	13	130	284.95	137	445.18	458	372	366	27	43	0	2	1
13524	8000	4718	5	924	722	27986	619	249	1054	114	92	220	99	183	275.76	247	440.14	486	414	453	24	100	14	6	0
14162	7790	4428	6	800	650	27910	653	303	1061	142	72	235	126	135	271.38	272	434.49	507	443	430	22	80	13	8	3
4032	13562	9868	960	388	210	28064	1293	8	574	2	2	54	4	66	270.86	92	423.88	285	264	279	15	40	0	2	2
4216	13496	9784	898	360	196	28058	1246	10	617	2	6	59	6	77	272.63	112	425.59	302	285	303	8	41	0	0	2
3824	13716	9988	998	332	182	28048	1311	6	550	1	4	49	4	69	270.33	78	423.26	268	261	282	13	33	0	2	1
10806	7530	5542	2	2492	1934	28872	506	69	420	12	508	160	34	315	333.56	80	471.29	464	130	342	132	76	6	58	35
4452	13328	9624	847	422	250	28090	1216	8	602	0	14	54	4	83	277.9	101	434.53	322	296	309	10	32	0	0	1
5226	12790	9190	631	558	338	28132	1108	5	716	0	28	70	3	89	279.18	111	438.11	380	368	326	18	50	0	2	2
4560	13150	9562	776	530	294	28114	1208	6	643	1	18	52	3	82	276.17	89	431.59	329	347	309	11	35	0	0	4
4548	13242	9560	794	436	280	28100	1192	6	630	1	30	60	5	71	275.45	97	429.66	338	318	318	19	43	0	4	1
5486	12640	9110	623	546	300	28112	1110	4	785	1	30	68	2	103	280.5	141	437.03	360	387	340	12	50	0	0	1
14378	7582	4340	3	860	656	27898	637	310	1047	152	80	265	125	164	273.54	271	438.47	484	421	423	19	96	17	2	1
4890	12966	9388	693	548	308	28120	1169	6	687	1	18	50	2	92	278.4	93	434.83	321	364	336	21	34	0	2	3
4562	13172	9546	793	520	302	28122	1196	4	624	0	20	57	3	84	276.91	93	433.07	330	288	313	15	42	0	0	3
4132	13448	9782	879	458	262	28094	1252	6	552	1	12	58	4	77	273.07	81	426.21	303	287	286	15	36	0	0	3
4334	13272	9676	864	510	288	28100	1268	6	576	1	20	59	2	70	275.38	80	429.58	298	307	274	20	38	0	0	4
10772	7778	5624	1	2296	1806	28808	484	74	470	11	464	156	41	285	330.61	93	469.36	484	148	370	136	70	2	58	34
5104	12906	9258	686	522	310	28114	1143	9	709	1	14	70	6	99	278.77	104	435.1	340	360	325	16	48	0	0	2
5496	12606	9066	585	600	340	28132	1083	10	732	0	24	61	6	91	281.61	117	438.9	388	368	349	16	44	0	0	3
13668	7904	4636	7	958	734	27990	659	266	990	110	86	213	124	177	278.26	229	441.17	456	415	458	27	92	17	4	2
14206	7704	4424	6	840	652	27908	658	295	1077	141	76	248	109	151	274.17	252	437.83	482	451	429	23	98	19	6	1
5170	12774	9196	656	610	368	28146	1135	11	683	1	28	64	6	98	279.2	97	436.23	357	363	318	17	46	0	0	2
4148	13490	9790	926	398	234	28076	1261	6	582	1	16	61	3	75	272.77	103	424.75	304	264	281	11	39	0	0	2
4366	13356	9670	849	438	248	28084	1248	5	613	1	6	65	1	74	274.65	89	428.7	315	311	277	13	47	0	0	2
14124	7680	4416	6	932	708	27932	617	315	992	158	68	261	120	159	268.82	279	432.06	507	378	428	21	87	18	4	1
5044	12898	9316	714	528	302	28114	1149	6	678	0	24	52	2	84	280.05	105	434.64	345	323	337	20	40	1	2	3
4742	13040	9422	729	550	344	28132	1169	5	628	0	34	59	4	70	278.33	95	434.75	351	330	318	27	41	0	0	2
13844	7624	4496	3	1096	844	28034	633	273	932	105	120	210	125	167	277.16	219	444.95	486	375	451	35	87	21	8	5
7268	11258	7946	344	1000	680	28248	900	32	745	8	88	88	13	155	285.89	131	445.41	422	352	383	33	60	2	8	5

SUPPLEMENTARY INFORMATION:Monte Carlo Atomistic Simulation and Machine Learning Analysis of Na-K Eutectic Alloy in Condensed Phases, D. Reitz and E. Blaisten-Barojas, George Mason University, Fairfax, VA 22030

4518	13250	9594	769	458	262	28098	1175	4	640	0	14	56	3	85	276.96	93	431.09	326	325	330	18	34	0	2	3
11636	7778	5232	3	1964	1566	28556	508	112	621	23	322	173	63	275	309.81	112	459.24	467	213	423	102	95	7	44	17
13696	7774	4566	3	1056	822	28028	621	260	953	111	106	216	111	200	277.38	201	443.87	459	368	464	30	86	17	6	1
4010	13584	9856	935	390	228	28076	1290	8	569	2	8	64	4	72	271.2	92	424.03	287	286	268	14	43	0	0	2
6012	12072	8654	526	844	560	28214	1030	18	665	1	64	77	15	105	286.89	103	441.3	408	334	333	34	46	0	6	3
7896	10792	7604	284	1128	742	28260	844	56	745	10	92	104	35	140	287.73	149	445.13	473	340	384	36	59	4	6	6
4992	12884	9296	672	580	352	28134	1141	3	685	1	30	55	0	90	274.97	102	430.87	358	349	332	15	42	0	0	3
5484	12626	9070	608	582	338	28128	1095	12	759	1	28	65	7	78	282.35	129	438.2	389	382	343	22	45	0	0	2
6108	12120	8652	475	766	488	28188	1028	18	756	5	52	71	7	124	283.74	113	440.84	387	408	352	21	51	1	2	5
11786	7828	5244	3	1898	1438	28528	546	133	634	26	314	150	73	257	307.63	134	456.12	513	228	410	75	86	11	18	18
13402	7714	4738	7	1242	876	28100	593	205	910	96	120	223	81	203	282.72	205	446.38	481	335	429	44	97	10	8	6
4618	13276	9540	810	380	244	28082	1219	12	639	2	22	66	8	88	275.36	96	429.72	298	328	306	18	41	0	2	1
14224	7680	4434	10	856	634	27894	647	317	1049	154	60	262	129	150	270.35	268	433.93	490	430	423	19	95	19	6	3
13732	7398	4428	2	1412	1028	28122	603	253	788	106	112	231	107	213	282.65	185	447.31	490	296	412	32	96	20	12	6
11442	7890	5374	0	2012	1518	28582	544	98	611	18	312	158	51	273	315.33	126	458.26	496	204	380	90	81	6	28	20
14208	7650	4432	3	880	662	27916	645	309	1038	155	74	268	114	178	271.63	246	435.47	464	407	420	20	97	22	10	1
13166	7788	4732	3	1334	1000	28162	569	194	852	60	130	208	96	204	284.63	199	446.49	504	306	455	42	104	12	12	7
3698	13818	10040	1006	314	180	28052	1317	8	532	1	2	59	6	61	270.93	85	424.04	273	244	266	13	34	0	0	5
3996	13564	9852	946	420	244	28082	1287	4	553	1	6	63	2	74	273.35	80	426.44	291	275	263	14	41	0	0	2
4440	13354	9678	854	378	198	28054	1217	11	664	1	6	65	6	66	273.91	123	427.81	324	300	301	15	49	0	0	3
4346	13336	9698	866	444	242	28078	1248	10	596	1	12	53	6	84	274.2	92	429.6	297	299	299	9	38	0	0	3
13778	7824	4586	4	950	736	27976	639	273	1012	119	98	233	122	169	274.55	235	438.9	469	404	450	33	99	17	4	1
4734	12988	9430	721	596	358	28140	1157	4	628	0	32	53	3	90	278.31	104	434.94	345	315	324	17	32	0	0	4
12112	7994	5104	2	1640	1284	28390	532	149	694	32	236	171	81	269	302.04	158	457.27	496	231	423	58	91	11	20	11
5226	12776	9168	641	590	356	28136	1073	7	680	0	20	67	5	89	281.07	126	437.86	377	332	362	23	45	0	0	5
14336	7682	4384	6	802	608	27874	607	333	1038	161	60	256	123	153	271.27	260	436.08	493	402	460	20	85	27	2	3
11158	7814	5402	0	2250	1688	28680	459	78	525	13	328	188	45	319	320.91	88	464.3	477	150	384	105	107	5	40	28
4592	13210	9578	830	446	240	28076	1228	9	656	1	8	60	3	79	276.14	94	430.17	316	325	298	8	45	0	2	3
7608	11130	7838	303	968	606	28214	905	34	872	6	58	84	15	137	289.68	145	447.53	435	426	390	27	52	4	6	7
4154	13508	9816	908	382	200	28062	1277	7	587	1	2	52	4	68	270.88	80	423.08	284	304	294	18	33	0	0	1
13158	7720	4746	2	1382	1024	28190	629	205	834	70	142	190	95	218	282.04	176	447.28	461	334	428	41	82	14	16	10
11268	7822	5464	1	2102	1598	28672	487	88	569	11	370	152	56	273	326.07	107	466.87	501	164	411	103	78	5	44	26
4528	13336	9616	818	352	212	28066	1220	9	639	1	22	52	4	72	275.9	92	430.22	311	339	318	19	32	0	0	0
14206	7836	4432	0	744	602	27884	640	310	1059	150	60	245	126	169	269.32	274	432.08	469	436	460	13	86	15	4	0
3780	13750	10008	995	322	182	28052	1305	9	552	1	10	53	4	69	270.76	98	422.37	274	247	276	14	31	0	0	1
13302	7800	4778	5	1234	866	28082	606	204	924	81	86	214	92	201	282.52	196	446.14	481	337	442	28	112	11	14	9

4584	13270	9594	768	396	216	28072	1173	4	689	0	12	55	4	85	275.81	96	431.83	329	341	335	10	42	0	0	4
14278	7708	4422	6	808	604	27882	623	301	1083	149	60	259	126	156	270.99	284	434.97	499	432	441	15	98	11	2	1
11738	7828	5236	2	1922	1466	28516	557	106	644	26	294	177	55	255	306.22	130	456.53	495	229	372	95	91	4	30	12
3918	13676	9914	963	342	204	28062	1319	9	563	1	8	58	5	66	270.49	90	422.5	280	288	256	13	34	0	0	1
14168	7738	4468	5	854	622	27908	633	288	1058	139	58	265	112	177	275.79	238	440.68	469	437	427	18	106	22	0	2
4440	13222	9634	830	506	280	28102	1245	9	599	0	18	54	5	73	274.92	101	430.46	310	307	287	20	40	0	2	2
12998	7762	4728	3	1438	1112	28208	618	201	810	72	158	185	90	220	284.62	164	448.94	453	312	442	52	85	11	12	9
5312	12832	9162	622	476	298	28106	1098	9	740	2	24	73	5	92	277.19	109	434.91	363	392	350	20	44	0	2	1
7622	10990	7734	334	1058	730	28240	918	58	751	15	96	111	27	137	289.48	120	445.49	434	371	342	36	72	6	10	7
4146	13408	9768	907	476	280	28100	1282	8	547	1	20	59	4	88	272.55	79	424.97	284	288	263	12	43	0	2	3
4746	13018	9450	711	574	320	28124	1144	5	635	1	16	53	3	74	275.82	91	433.44	357	323	338	24	30	0	0	3
12836	7642	4794	0	1606	1206	28294	558	186	720	50	194	186	101	226	291.92	174	452.52	498	262	436	63	96	13	16	9
5324	12676	9138	656	608	352	28122	1146	10	716	2	24	73	4	78	280.89	113	437.4	369	374	306	20	57	0	0	2
4120	13540	9802	930	368	226	28072	1278	7	583	1	14	53	4	74	271.29	88	425.56	285	285	289	15	39	0	2	1
5602	12376	8958	544	774	438	28176	1075	8	728	0	26	62	2	98	280.64	116	438.48	407	375	334	16	40	0	2	1
11528	7860	5380	0	1994	1470	28568	519	88	615	22	306	177	51	282	310.79	121	460.39	460	192	413	96	97	2	28	25
3908	13690	9930	978	322	194	28058	1320	6	572	1	14	61	4	78	271.48	78	423.69	248	280	271	13	40	0	0	5
12852	8066	4938	10	1242	922	28144	656	181	914	61	120	187	97	196	281.14	181	445.75	445	362	451	51	106	11	4	3
4014	13614	9900	940	334	180	28052	1272	4	580	0	10	57	3	63	272.15	100	424.29	293	282	292	17	40	0	0	1
4270	13470	9754	858	358	200	28062	1225	4	625	1	10	64	1	66	273.45	103	425.8	316	311	303	17	45	0	0	3
12000	7940	5172	1	1732	1312	28416	556	125	683	26	232	191	60	254	302.44	131	454.7	460	234	414	77	101	12	28	17
14442	7710	4344	3	754	566	27852	648	326	1093	172	34	257	114	158	267.6	292	429.54	484	440	436	9	78	14	2	0
6436	11848	8480	405	868	528	28214	956	16	774	1	50	67	10	107	289.46	130	443.25	453	405	379	27	39	1	4	2
12512	7898	4996	2	1522	1152	28290	589	161	770	44	192	196	83	231	293.57	150	450.59	473	290	418	59	105	16	18	12
13028	7754	4788	5	1396	1052	28192	610	199	805	72	160	206	96	205	283.21	163	447.19	479	306	427	52	100	10	12	7
4252	13328	9686	836	526	312	28120	1201	4	571	0	14	66	4	72	276.48	84	432	343	281	293	21	35	0	2	1
5634	12346	8894	559	794	484	28192	1067	13	680	0	40	48	10	118	282.98	96	438.77	389	366	357	14	30	0	0	0
13170	7768	4722	4	1342	1010	28154	607	216	847	81	126	195	100	217	282.03	192	446.03	492	324	438	28	90	15	14	6
5094	12838	9238	665	572	358	28136	1123	3	676	2	36	60	0	74	280.54	109	437.43	383	355	335	20	36	0	0	1
5714	12438	8892	578	664	406	28140	1075	20	740	7	26	74	11	95	280.96	121	439.56	394	373	340	16	50	1	0	2
12848	7858	4874	6	1372	1060	28208	614	184	846	65	184	198	79	218	281.91	201	445.59	508	316	400	40	102	10	12	1
5962	12276	8770	542	688	422	28158	1036	15	728	1	40	70	9	108	285.11	123	440.09	403	362	353	18	50	0	0	1
4130	13544	9822	921	350	200	28058	1246	11	592	1	12	56	6	79	271.11	89	424.08	298	277	302	11	37	0	0	1
5412	12554	9048	616	700	418	28162	1082	9	692	1	28	53	6	97	281.6	107	437.57	393	343	352	17	42	0	2	0
14188	7732	4450	4	838	622	27886	641	324	1045	163	52	271	125	168	269.97	262	432	465	420	429	20	94	19	4	2
4510	13350	9646	829	356	186	28050	1223	12	641	1	2	66	6	77	274.28	98	429.01	314	329	300	11	45	0	0	0

SUPPLEMENTARY INFORMATION:Monte Carlo Atomistic Simulation and Machine Learning Analysis of Na-K Eutectic Alloy in Condensed Phases, D. Reitz and E. Blaisten-Barojas, George Mason University, Fairfax, VA 22030

5406	12626	9086	650	632	366	28136	1108	14	687	0	16	62	9	84	281.82	114	439.16	386	348	334	15	43	1	4	3
4550	13166	9530	780	536	322	28124	1198	9	619	1	20	62	6	83	276.5	98	431.41	333	322	301	14	42	0	0	5
4142	13496	9826	927	372	208	28066	1274	4	599	2	20	68	2	64	271.62	96	424.01	292	289	278	21	51	0	2	1
4382	13468	9738	880	294	148	28034	1250	11	653	0	4	56	9	69	274.27	98	430.05	291	319	311	13	39	0	0	3
4432	13318	9630	844	436	260	28094	1230	5	615	0	18	62	4	67	275.79	112	430.29	329	301	290	15	41	0	0	3
13356	7800	4628	1	1212	972	28122	581	228	866	77	146	214	108	223	281.48	210	446.24	475	295	450	38	105	20	8	2
14302	7704	4390	5	810	618	27884	619	320	1052	164	54	263	120	150	268.92	291	431.03	514	419	427	15	87	15	6	2
4578	13234	9548	833	452	266	28088	1244	13	645	0	10	53	5	74	274.87	105	432.26	314	318	286	14	41	0	0	3
4756	13138	9486	729	434	254	28096	1130	3	671	0	28	53	2	87	277.64	101	433.17	350	329	360	14	32	0	0	2
3804	13736	9980	984	324	200	28062	1305	5	556	1	14	60	3	70	271.05	80	423.13	264	268	276	18	37	0	4	3
14354	7746	4404	7	750	558	27854	653	316	1110	160	40	253	121	136	270.4	293	433.73	502	470	432	15	81	16	2	0
6694	11712	8322	382	870	554	28216	932	19	785	4	62	74	10	132	284.18	135	442.02	446	384	376	24	45	1	2	1
11738	7848	5200	0	1900	1488	28502	534	114	636	34	286	185	50	253	304.39	134	458.05	504	202	377	94	102	5	38	16
11750	8040	5214	1	1788	1394	28460	585	128	664	19	250	135	74	246	300.02	149	452.64	493	238	427	73	74	10	24	10
11084	7802	5442	0	2192	1732	28714	520	89	538	18	418	181	37	302	318.22	99	461.26	484	172	333	113	96	6	40	21
4872	13002	9410	743	526	282	28098	1172	7	664	1	6	75	5	84	278.64	94	437.3	332	333	306	21	47	0	0	3
5580	12458	8984	578	692	406	28156	1070	9	708	1	34	76	6	94	283.83	126	439.88	403	351	329	17	49	0	2	4
14264	7566	4376	8	908	702	27922	631	317	1005	159	104	254	125	132	272.54	253	435.53	509	411	433	32	75	14	2	3
4714	13150	9498	750	456	262	28100	1187	4	647	0	20	65	4	77	278.65	93	433.6	337	343	315	15	45	0	0	3
12274	7826	5032	1	1678	1294	28364	582	137	733	42	242	198	68	235	294.27	160	454.25	469	254	405	72	114	10	14	16
13290	7788	4708	3	1248	946	28126	619	227	867	81	142	198	101	193	282.52	197	446.55	475	315	441	47	95	23	4	6
7184	11248	7950	338	1082	718	28258	897	26	738	5	72	89	16	140	287.93	130	445.63	436	350	381	36	53	0	4	8
3722	13766	10030	998	324	194	28056	1326	6	542	1	20	61	4	78	270.22	81	422.22	258	269	258	12	37	0	0	1
4426	13318	9678	848	414	220	28070	1233	9	614	2	14	52	6	54	274.47	80	427.51	309	310	318	26	33	0	0	3
4400	13364	9670	864	410	226	28074	1250	3	643	1	4	59	0	84	274.89	104	430.35	293	302	288	14	41	0	0	2
4710	13126	9500	740	480	270	28106	1185	5	671	0	20	57	4	74	277.84	103	432.99	336	346	323	19	46	0	0	3
4746	13206	9464	738	408	250	28082	1165	8	663	1	8	67	2	81	274.99	107	433.54	341	347	315	19	38	0	0	1
5208	12774	9208	617	574	336	28128	1116	6	743	1	26	59	4	97	277.68	106	435.55	362	392	345	14	38	0	2	2
3938	13682	9938	985	312	170	28046	1316	10	577	1	6	54	4	70	270.4	95	424.21	269	280	269	11	38	0	0	2
14542	7668	4304	4	714	560	27844	661	331	1100	161	52	256	125	142	267.03	275	428.27	484	465	434	14	80	20	4	1
3960	13678	9932	964	296	166	28044	1311	5	595	2	10	63	2	62	270.34	93	423.01	281	297	261	13	40	0	2	2
13580	7638	4528	2	1256	970	28114	642	235	848	83	136	197	107	188	281.99	183	448.87	459	346	444	50	91	23	6	3
6638	11720	8328	469	886	572	28202	1004	34	728	13	50	86	14	117	286.35	127	442.64	412	357	337	27	47	0	8	5
11932	7824	5194	2	1842	1380	28458	545	109	675	20	242	186	58	252	305.2	136	456.29	467	229	401	92	100	4	42	17
14080	7778	4514	6	850	622	27902	648	290	1065	140	50	254	107	152	274.84	252	439.3	476	418	426	29	99	20	8	2
4920	12958	9360	691	546	318	28126	1161	5	682	0	24	56	3	90	278.79	100	435.42	351	354	318	15	37	0	0	1

14144	7744	4506	1	850	604	27904	622	282	1084	147	50	261	103	161	274.03	263	436.41	499	425	433	16	103	13	6	2
13374	7534	4624	1	1404	1052	28168	577	249	792	92	166	223	117	218	284.53	165	448.05	486	300	427	49	92	12	14	5
4814	12946	9368	683	606	378	28148	1130	5	649	0	30	68	5	89	279.28	99	434.93	353	342	324	25	42	0	6	5
11436	7818	5368	2	2062	1552	28592	540	108	585	26	306	181	55	287	320.13	107	460.81	456	191	365	94	90	1	46	27
4658	13150	9496	773	476	294	28102	1203	7	649	0	28	61	4	86	275.81	98	431.14	312	339	311	21	37	0	0	1
13876	7610	4416	3	1116	890	28034	595	277	890	113	116	244	111	211	277.75	226	444.97	463	319	431	37	111	27	10	2
11488	7908	5352	0	1990	1502	28574	534	90	618	19	298	177	46	268	312.45	131	459.26	509	190	365	94	106	8	32	15
14180	7738	4414	3	880	658	27920	643	309	999	140	48	240	130	174	274.25	252	436.83	462	411	450	21	82	15	2	1
4038	13594	9876	944	336	196	28060	1299	8	579	1	18	56	6	70	270.19	89	423.09	273	292	283	13	40	0	2	3
4444	13260	9614	811	500	280	28104	1198	6	598	0	6	51	4	83	275.14	93	429.12	333	294	317	13	33	0	0	0
4280	13440	9714	865	388	238	28076	1252	5	609	1	14	67	2	72	274.06	83	429.46	302	312	283	15	50	0	2	3
14266	7656	4390	7	892	652	27908	653	309	1041	157	46	252	118	161	269.34	250	431.97	478	445	430	19	81	11	6	1
4160	13526	9812	923	356	196	28058	1285	10	595	1	8	56	6	76	273.15	83	427.02	285	309	277	11	39	0	0	1
5568	12378	8892	543	790	520	28206	1063	9	678	1	52	73	3	107	283.81	97	439.82	389	361	331	26	42	0	6	5
3888	13688	9970	980	330	170	28050	1320	5	558	1	4	63	4	70	271.16	83	424	265	281	259	13	38	0	0	3
4176	13502	9794	907	370	210	28064	1258	7	592	1	12	63	5	72	272.07	101	426.81	305	281	282	8	41	0	0	5
4158	13630	9856	942	246	128	28022	1292	13	618	1	4	56	9	65	271.08	101	423.42	277	306	288	10	34	0	0	3
5402	12672	9088	617	590	356	28136	1083	8	716	0	28	69	5	96	280.34	125	437.59	395	334	337	14	47	0	0	0
4646	13172	9504	771	476	290	28112	1188	8	643	2	24	56	4	81	277.22	114	432.28	339	321	314	16	36	0	0	1
13922	7746	4524	5	956	724	27958	627	289	1001	141	72	242	111	162	273.02	245	436.88	496	399	436	26	91	16	14	2
14152	7622	4428	5	942	698	27920	652	309	1012	155	74	253	113	162	271.65	243	436.63	460	418	433	29	83	20	4	3
13316	7846	4768	2	1180	854	28078	638	219	929	64	108	198	113	193	282.72	192	448.67	463	358	444	39	114	15	6	4
4684	13184	9510	748	450	256	28094	1190	5	665	0	8	65	3	82	278.77	100	434.12	337	353	305	14	41	0	2	1
4652	13094	9514	759	538	298	28114	1196	5	626	0	18	55	3	77	277.39	93	433.56	339	325	305	19	37	0	0	1
4652	13088	9474	734	552	336	28130	1161	2	621	0	28	72	2	93	279.38	84	435.5	348	322	304	16	39	0	0	2
5068	12838	9280	672	596	332	28128	1140	6	695	0	14	60	4	86	279.5	99	435.36	360	360	327	16	43	0	0	4
12692	7824	4924	3	1532	1120	28262	588	186	783	53	146	185	95	241	293.08	175	451.07	464	281	427	58	88	11	20	5
12900	7610	4782	3	1570	1186	28268	601	212	735	75	208	204	89	210	294.18	163	452.27	478	284	411	68	94	21	12	10
13714	7952	4724	6	880	612	27938	655	281	1057	116	50	230	134	172	275.68	244	439.78	464	438	443	20	93	14	6	3
4406	13354	9674	842	398	224	28070	1218	8	622	1	14	56	5	75	272.94	96	426.37	316	307	311	13	40	0	0	3
14272	7686	4396	4	832	636	27890	642	320	1050	165	64	259	117	151	269.29	272	431.12	503	421	427	14	80	20	4	0
3930	13728	9952	973	258	152	28036	1305	9	597	2	16	63	4	67	270.95	96	423.37	269	282	272	16	43	0	0	1
11992	7876	5054	2	1826	1436	28452	595	121	660	35	224	164	59	259	299.21	125	454.52	468	246	397	86	85	13	42	4
11120	7828	5490	2	2154	1650	28672	501	82	557	16	394	159	47	280	316.36	115	464.37	491	171	392	109	83	4	36	20
3932	13682	9922	976	320	188	28054	1337	6	581	1	10	58	3	65	270.99	79	422.28	260	306	255	16	38	0	0	2
3986	13622	9934	962	330	164	28044	1291	13	557	1	8	60	8	55	271.52	84	424.38	292	274	276	17	39	0	0	2

4512	13272	9614	842	424	240	28080	1235	5	641	1	18	67	2	68	274.21	105	427.88	321	316	285	19	49	0	0	0
7942	10892	7584	289	1030	702	28240	904	39	806	4	84	93	27	137	288.36	137	445.87	451	394	360	31	63	0	6	6
3944	13728	9914	973	272	176	28048	1301	9	579	2	14	57	3	68	270.95	98	423.89	277	281	277	12	38	0	0	2
14150	7606	4406	4	966	728	27944	643	308	998	155	84	255	114	162	274.29	236	438.98	477	425	420	29	82	10	4	4
5482	12500	9000	640	722	428	28154	1119	15	669	1	22	65	7	108	283	109	438.66	361	340	318	15	50	0	0	2
14450	7716	4380	5	714	534	27842	647	342	1092	165	46	249	131	141	269.8	264	432.17	479	455	460	19	74	24	2	0
11370	7882	5400	1	2070	1548	28616	542	94	597	20	294	165	49	281	314.6	129	463.91	482	209	380	91	78	6	52	22
11880	7868	5144	2	1826	1446	28484	572	117	649	25	280	167	57	247	304.52	139	456.23	470	225	404	93	91	13	40	15
14376	7710	4354	6	786	600	27872	664	318	1076	161	40	257	127	148	267.76	275	430.18	486	459	426	11	86	17	6	3
4098	13518	9804	907	398	244	28080	1280	6	581	0	14	53	4	69	272.67	95	425.92	288	284	280	16	30	0	4	5
4460	13254	9618	829	474	272	28096	1215	5	610	0	16	68	5	90	276.66	83	431.27	316	302	291	11	49	0	2	3
5022	12964	9342	671	478	272	28100	1126	6	702	0	22	66	4	88	277.94	104	433.56	355	375	337	18	42	0	0	2
11442	8030	5392	1	1872	1454	28534	530	97	653	18	316	182	51	275	313.23	127	461.31	467	199	387	94	104	7	28	22
5118	12916	9304	684	470	260	28086	1146	11	723	0	18	64	6	88	280.11	108	435.09	345	376	324	17	42	0	0	2
6224	12028	8558	482	824	526	28206	1024	18	731	7	44	74	5	123	284.11	106	441.57	414	382	340	16	43	0	2	2
5768	12288	8862	544	756	452	28170	1047	16	725	2	42	82	9	107	285.96	106	441.21	410	375	324	17	63	0	0	2
4670	13118	9526	751	488	274	28104	1188	3	668	0	28	68	2	97	276.67	91	432.63	308	346	313	18	45	0	0	3
4010	13630	9878	950	334	196	28058	1311	2	601	1	10	67	1	56	271.25	106	423.95	293	294	254	14	44	0	0	2
3792	13766	10008	997	288	172	28044	1319	8	547	2	18	61	4	64	269.76	79	422.52	263	266	268	15	41	0	0	4
4542	13268	9608	817	414	228	28074	1205	8	636	2	14	66	5	73	275.47	91	430.26	331	315	308	10	46	0	0	4
4894	12964	9362	726	554	328	28126	1180	15	619	0	24	51	11	89	280.57	90	435.82	338	341	314	16	29	0	0	2
4734	13252	9502	755	354	212	28064	1172	6	685	0	10	63	2	77	276.58	98	430.93	340	352	327	12	43	0	0	2
6004	12244	8776	524	690	398	28142	1042	19	740	4	30	84	12	105	282.41	117	441.29	392	380	342	20	53	0	0	5
7210	11408	8096	380	878	540	28198	911	34	783	10	56	94	21	134	287.2	122	444.81	428	368	391	31	63	0	8	3
11934	7908	5224	1	1806	1306	28414	588	124	705	33	216	180	58	258	301.44	143	453.84	483	272	386	61	89	10	18	14
6632	11730	8368	433	858	542	28196	975	19	762	5	62	84	11	121	287.26	113	444.01	423	391	355	28	56	0	4	2
5286	12742	9162	629	580	338	28130	1104	11	701	3	22	64	4	86	280.26	129	436.49	379	346	341	20	41	1	0	1
4062	13528	9838	911	398	232	28076	1260	8	573	0	18	63	5	76	275.36	86	429.48	291	278	286	16	45	0	0	3
12058	7896	5110	2	1714	1350	28420	554	137	698	26	268	170	77	251	298.3	148	452.59	496	231	408	68	97	10	18	16
5308	12800	9168	619	518	302	28112	1118	6	763	0	16	59	3	91	281.54	126	438.54	362	401	338	19	44	0	0	1
5428	12752	9130	604	504	280	28106	1084	7	753	0	12	72	6	101	280.37	119	436.32	358	385	356	21	47	0	0	1
3996	13604	9890	959	354	202	28060	1285	8	550	1	14	58	6	67	270.86	79	423.7	282	262	289	13	39	0	0	4
14126	7618	4440	2	978	704	27936	636	305	972	159	70	248	113	177	273.65	235	439.98	473	397	443	16	73	22	0	3
13642	7938	4666	4	950	712	27988	627	249	996	107	80	218	115	170	275.85	233	442.27	492	388	454	26	99	11	0	1
5620	12514	8954	589	636	388	28140	1090	11	702	1	28	65	7	109	284.75	91	439.29	360	371	346	18	46	0	0	3
4176	13582	9800	897	312	180	28052	1242	4	624	1	2	60	3	69	272.5	97	426.74	300	292	307	13	42	0	0	3

11832	7956	5304	1	1726	1312	28448	533	125	671	33	304	184	70	258	308.37	133	456.64	488	211	404	86	106	3	14	14
4786	13062	9460	718	502	280	28112	1150	4	656	0	22	53	2	68	277.12	113	431.38	373	338	328	19	34	0	0	1
14550	7692	4338	6	696	512	27820	637	349	1108	175	30	263	129	152	268.15	278	429.61	484	447	442	11	81	17	2	0
6362	11794	8492	437	938	572	28218	984	16	723	5	56	73	8	125	290.68	110	443.1	408	374	371	29	42	0	4	3
4548	13312	9608	831	378	210	28064	1259	8	660	2	8	63	3	71	276.04	95	430.74	304	348	278	15	44	0	0	1
4408	13446	9684	875	320	184	28046	1238	10	646	2	4	61	5	76	272.44	99	426.63	298	305	307	12	44	0	0	2
14238	7776	4444	10	772	586	27870	630	296	1101	151	50	249	120	147	271.57	269	435.52	493	438	455	18	89	11	4	1
4156	13522	9814	898	356	200	28062	1250	9	594	2	14	57	4	80	271.22	94	425.02	296	285	296	11	42	0	0	2
4624	13226	9514	806	444	272	28092	1243	6	658	1	12	65	3	89	275.86	104	433.7	291	360	289	15	35	1	0	3
6684	11622	8318	379	962	590	28238	948	20	784	3	58	72	15	120	284.19	137	441.85	465	393	373	16	48	1	4	3
4994	12900	9316	689	562	328	28124	1141	11	654	2	24	68	6	98	280.51	100	434.75	343	342	322	20	41	0	0	1
4082	13468	9834	913	442	242	28084	1262	4	574	0	14	57	3	69	274.05	92	428.1	302	277	285	15	42	0	2	4
6924	11570	8172	395	916	582	28210	932	35	766	7	40	85	19	144	286.11	134	443.54	425	376	374	21	49	0	6	5
3918	13708	9930	973	300	182	28050	1294	11	564	2	12	55	6	70	270.21	91	422.38	280	265	278	11	32	0	0	3
4050	13540	9856	933	388	218	28066	1287	13	555	1	14	58	8	82	272.86	71	426.41	271	283	277	13	41	0	0	3
13984	7654	4570	7	988	672	27942	605	289	1013	132	70	235	125	154	277.02	252	441.59	484	399	472	38	82	15	4	2
4998	12910	9322	678	546	318	28118	1107	7	643	0	22	67	5	89	281.13	98	436.41	368	326	345	17	39	0	2	3
5402	12518	9052	569	726	438	28182	1067	10	696	0	44	66	5	108	283.87	108	440.83	398	361	329	19	43	0	2	2
14278	7582	4314	6	920	738	27928	641	300	1038	161	90	269	105	177	269.57	259	434.06	480	418	405	19	93	17	4	1
5104	12874	9248	686	542	330	28122	1133	13	682	2	24	62	10	95	280.76	112	437.87	345	332	337	21	43	0	0	1
12540	7730	4928	1	1624	1252	28324	587	173	749	35	224	173	93	233	293.92	160	453.04	485	292	423	58	93	20	24	11
4360	13302	9676	849	484	264	28096	1263	7	613	0	10	54	3	76	277.13	79	433.16	298	331	286	17	40	0	0	1
4624	13232	9550	798	412	240	28074	1187	10	639	0	16	70	6	79	274.27	95	430.71	316	312	317	24	46	0	0	1
12030	7770	5280	0	1806	1252	28400	552	126	706	19	246	167	71	229	301.71	137	455.94	486	240	430	84	99	8	16	13
12162	7962	5124	4	1630	1244	28366	588	148	692	36	222	172	76	252	298.78	136	453	453	265	429	70	88	17	20	12
13700	7784	4536	6	1080	830	28014	622	271	955	120	70	224	124	185	276.31	216	441.61	471	374	449	35	88	10	14	1
4876	13018	9416	744	494	274	28096	1178	12	678	1	18	70	7	83	277.8	101	434.49	341	347	301	15	53	0	0	3
12480	7872	4982	5	1522	1194	28294	584	175	760	52	228	188	89	236	293.67	163	451.01	478	281	418	56	97	13	16	13
13526	7730	4634	2	1208	880	28082	593	234	866	78	88	203	108	188	283.85	199	446.23	496	314	452	42	97	15	14	3
5956	12402	8792	513	586	368	28132	1061	13	792	1	26	73	7	107	283.11	127	441.08	390	433	345	13	47	1	2	2
14288	7730	4388	8	798	620	27884	655	315	1061	155	54	259	121	149	270.95	266	433.52	480	440	430	19	93	21	6	3
4270	13478	9770	918	338	184	28052	1271	9	605	1	12	57	6	67	273.17	87	426.57	287	290	295	17	45	0	0	1
14540	7646	4340	7	722	538	27840	637	335	1118	157	50	255	140	139	268.92	287	432.47	484	437	455	21	91	22	4	1
5220	12644	9138	622	708	430	28178	1111	7	643	0	36	58	6	109	281.53	77	437.59	359	360	336	19	33	0	2	3
4192	13458	9794	900	410	210	28064	1271	7	600	1	0	68	4	82	272.29	87	425.82	279	303	282	12	41	0	0	3
12730	7884	4902	1	1450	1090	28228	585	183	826	61	164	195	94	214	285.22	181	448.97	498	307	429	53	99	7	6	4

SUPPLEMENTARY INFORMATION:Monte Carlo Atomistic Simulation and Machine Learning Analysis of Na-K Eutectic Alloy in Condensed Phases, D. Reitz and E. Blaisten-Barojas, George Mason University, Fairfax, VA 22030

13474	7728	4574	6	1226	966	28108	634	255	818	103	126	201	107	193	279.52	187	445.47	478	326	442	36	77	21	14	4
4850	12934	9380	710	592	348	28134	1158	9	653	0	28	70	7	86	277.3	84	434.29	344	342	311	20	54	1	2	5
4080	13588	9852	925	338	190	28056	1276	5	592	1	8	63	2	68	271.86	86	425.04	291	300	282	15	39	0	0	1
4754	13158	9484	782	418	244	28076	1195	6	670	0	18	63	4	85	275.07	97	429.78	326	336	308	14	43	0	0	0
6670	11892	8360	431	730	472	28162	980	34	782	10	38	86	18	113	288.38	135	444.55	427	393	360	18	49	0	0	5
13732	7914	4612	4	902	710	27960	625	271	1034	138	86	246	96	150	274.6	252	439.2	502	403	434	31	92	16	4	4
5072	12696	9222	679	714	422	28160	1135	11	621	0	34	61	8	92	282.91	93	439.15	362	323	315	19	42	0	0	5
4936	13054	9384	713	454	256	28092	1169	0	728	0	6	68	0	94	278.12	115	433.7	323	368	326	16	43	0	2	1
4100	13576	9844	913	312	196	28060	1263	8	592	1	32	60	6	71	273.4	90	426.15	290	294	288	20	45	0	0	0
14048	7746	4518	8	892	644	27910	623	316	1020	162	62	268	110	168	274.22	236	437.79	492	409	421	18	95	14	0	1
5326	12820	9176	624	498	276	28100	1103	6	758	0	4	65	5	80	280.68	126	436.45	371	384	357	19	46	0	0	2
14190	7630	4430	2	944	672	27926	634	303	1007	144	58	245	119	149	272.28	248	436.17	494	410	442	28	89	19	2	1
4250	13444	9714	868	406	254	28084	1244	6	595	1	14	58	4	75	273.24	78	427.87	302	293	295	19	35	0	2	1
13436	7606	4556	0	1362	1042	28152	576	238	826	88	140	217	105	219	280.82	181	446.17	497	313	435	33	104	13	8	8
11956	7818	5192	4	1850	1364	28452	567	131	656	31	234	187	61	258	305.61	132	455.77	476	211	375	86	104	6	38	15
4736	13134	9468	728	468	280	28108	1151	3	655	0	22	63	2	88	277.49	103	432.63	347	342	328	18	44	0	0	0
5468	12556	9046	602	648	388	28142	1119	16	710	6	32	66	7	104	281.32	101	438.39	363	386	322	15	41	0	4	2
10504	7866	5710	0	2378	1844	28820	496	67	457	6	480	157	41	300	332.2	77	472.68	456	157	362	129	72	1	34	39
5198	12722	9186	641	622	382	28152	1120	10	681	0	34	64	7	108	282.04	97	438.85	356	361	327	17	46	0	6	0
4326	13324	9690	867	482	264	28094	1216	15	570	2	8	46	6	83	274.48	108	430.02	315	265	314	15	28	0	0	1
4494	13274	9620	820	440	242	28080	1230	5	641	0	10	64	2	81	275.12	97	429.61	307	328	295	16	43	0	0	1
8068	10882	7524	255	1000	680	28224	867	47	839	13	56	98	23	155	288.62	130	445.99	427	392	401	33	56	3	12	5
4108	13462	9804	908	454	252	28088	1260	3	575	0	6	47	3	57	273.14	94	426.22	326	276	295	9	28	0	2	4
12024	7758	5122	1	1872	1402	28464	560	134	649	31	260	167	73	253	301.9	131	453.92	479	238	414	83	84	12	22	14
4542	13234	9580	744	462	264	28098	1159	1	658	0	16	66	1	87	276.47	99	434.46	338	332	327	19	39	0	0	0
4768	13114	9454	747	480	276	28104	1180	7	673	0	12	57	3	82	277.3	115	433.74	347	339	309	14	40	0	0	1
14314	7648	4376	5	842	644	27898	628	295	1076	151	66	265	104	160	272.33	273	435.38	475	411	439	25	100	23	8	3
14208	7658	4440	6	904	648	27920	660	302	1015	154	58	261	113	160	273.96	251	437.75	481	418	410	19	91	17	4	2
4892	12960	9380	712	560	316	28128	1162	9	662	0	20	52	7	76	279.19	114	434.92	363	338	324	16	34	0	0	2
11142	8052	5528	2	1976	1528	28610	556	91	599	14	348	169	51	282	316.66	113	463.79	482	223	361	85	88	5	34	26
11560	7766	5222	1	2066	1612	28582	566	104	595	23	298	171	55	269	306.07	121	457.35	481	197	363	90	91	6	54	20
4444	13248	9608	792	498	296	28118	1203	7	606	0	22	57	6	87	276.27	80	430.86	328	313	307	14	37	0	2	1
5468	12698	9062	586	550	330	28124	1074	5	747	1	16	71	4	91	282.15	125	438.22	387	391	353	17	54	0	0	3
5116	12798	9224	667	594	368	28136	1116	9	674	1	34	76	6	97	282.83	113	438.7	368	330	315	15	53	0	2	5
14342	7736	4410	10	758	564	27858	645	330	1082	157	48	261	132	139	271.32	261	434.8	499	453	431	17	93	21	0	1
12700	7864	4886	5	1504	1136	28268	600	180	765	57	160	193	85	216	292.03	157	452.03	467	277	423	66	87	13	16	11

SUPPLEMENTARY INFORMATION:Monte Carlo Atomistic Simulation and Machine Learning Analysis of Na-K Eutectic Alloy in Condensed Phases, D. Reitz and E. Blaisten-Barojas, George Mason University, Fairfax, VA 22030

13830	7838	4640	7	920	648	27942	638	292	1015	130	64	234	125	151	275.88	230	438.71	495	419	443	28	88	15	2	1
14514	7618	4342	3	762	558	27848	627	356	1043	167	52	263	143	160	269.12	259	432.8	467	420	458	19	89	22	2	1
4930	12948	9346	686	550	324	28120	1154	7	697	0	22	65	3	87	277.81	106	434.43	349	368	319	16	39	0	0	4
4024	13608	9894	960	334	180	28048	1270	6	584	1	6	63	4	76	271.43	99	424.02	284	256	287	12	43	0	2	2
4960	12968	9362	696	516	290	28114	1138	6	678	0	16	59	4	96	278.15	95	433.08	349	352	342	10	35	0	2	3
12634	7942	4986	2	1460	1052	28220	585	174	808	54	136	183	90	216	283.91	184	448.16	511	279	436	43	86	11	10	4
6378	12020	8534	441	744	464	28184	979	14	795	1	38	78	8	88	286.58	141	443.29	454	399	362	27	57	0	6	4
4540	13226	9560	799	470	284	28100	1216	3	639	0	18	62	0	75	276.76	97	432.55	320	324	298	21	44	0	2	2
4322	13382	9708	862	422	236	28082	1268	7	632	1	12	56	3	68	273.57	99	428.93	298	333	279	20	36	0	0	2
5858	12132	8788	551	868	506	28194	1041	26	643	2	42	65	17	125	286.76	80	442.78	374	330	353	30	40	1	0	0
5122	12790	9250	653	606	340	28126	1099	10	677	2	18	78	4	97	282.36	109	438.06	362	330	330	24	54	1	0	2
13714	7552	4488	4	1262	948	28094	597	265	846	91	118	232	118	198	283.06	182	447.74	480	330	427	43	106	29	12	7
5516	12438	8980	559	750	452	28176	1053	12	702	1	38	67	9	120	282.93	108	439.67	387	363	353	16	45	0	2	0
14292	7790	4432	9	726	556	27844	629	319	1104	173	46	287	109	143	269.17	294	432.64	493	418	419	23	102	21	2	1
4330	13396	9706	870	404	230	28080	1245	8	612	0	14	63	5	81	277.21	102	432.85	309	303	284	10	42	0	0	2
13874	7770	4556	6	942	720	27962	611	278	1021	125	96	229	122	183	274.54	225	441.91	447	409	483	34	91	10	4	4
10998	8084	5472	1	2026	1650	28644	481	83	578	17	380	161	41	303	315.35	105	459.32	490	174	396	105	85	3	30	19
13830	7682	4530	5	1056	796	28000	624	281	972	134	98	238	117	175	275.71	210	438.63	470	398	446	37	87	12	6	3
5678	12570	8954	611	542	340	28116	1118	19	727	7	32	73	12	95	282.45	112	438.31	365	368	329	16	52	0	0	0
4588	13234	9556	793	442	254	28084	1192	8	631	2	10	45	4	70	273.6	92	427.49	327	327	337	16	32	0	0	5
7044	11546	8140	340	870	554	28210	911	26	815	5	52	94	17	109	286.2	145	444.43	484	412	369	25	52	0	4	2
4680	13210	9544	758	410	222	28078	1161	5	662	0	12	64	2	85	279.68	104	432.97	335	341	333	12	41	0	0	4
7422	11066	7858	322	1114	724	28278	906	50	738	11	86	88	31	147	290.38	145	444.52	473	361	340	28	44	0	8	0
3920	13664	9930	970	350	190	28054	1289	4	583	1	0	62	2	64	271.77	98	424.89	287	264	274	17	46	0	0	1
5046	12960	9332	677	480	266	28098	1135	5	726	0	14	68	3	79	279.06	105	435.23	365	370	334	17	41	1	0	0
5566	12582	8988	579	600	372	28136	1072	11	724	1	26	62	8	100	281.5	102	439.43	365	374	367	25	41	0	2	2
14318	7768	4464	7	718	526	27844	658	335	1103	164	46	258	133	146	271.2	269	433.59	484	471	431	13	83	17	2	1
14230	7656	4430	11	880	642	27906	633	314	1033	162	68	255	115	148	272.47	264	437.03	506	417	429	17	82	19	0	4
5352	12582	9100	630	694	402	28160	1123	8	715	1	26	56	6	96	281.17	103	437.35	362	370	337	21	36	0	4	1
12226	7804	5078	2	1712	1300	28394	591	148	700	33	252	162	81	240	298.16	150	450.9	480	265	407	69	87	8	22	15
10942	7774	5444	1	2280	1828	28758	545	91	474	18	440	169	50	280	323.52	80	467.09	445	160	353	131	79	2	46	33
14276	7654	4390	3	848	650	27894	644	315	1045	157	70	263	119	150	271.56	269	434.02	489	434	423	21	96	19	6	3
4856	12984	9402	704	556	308	28120	1143	5	671	0	14	56	4	94	278.01	97	433.91	337	347	344	18	42	0	0	3
14516	7638	4348	6	746	542	27836	638	345	1082	177	46	265	136	157	268.15	273	429.63	482	454	442	9	77	12	0	1
4112	13516	9802	906	402	238	28082	1257	6	584	0	8	53	6	70	272.6	104	425.17	308	277	290	15	32	0	4	1
12730	8114	4954	5	1288	968	28174	652	179	917	59	108	171	91	207	281.32	198	447.7	476	380	442	34	85	9	10	3

13794	7866	4580	5	924	714	27962	641	285	1003	140	80	242	119	169	274.04	245	438.56	476	391	436	28	87	11	4	0
4434	13190	9580	794	568	338	28130	1205	2	586	0	20	60	0	86	276	82	431.76	322	299	301	19	42	0	0	3
11238	7886	5482	0	2062	1556	28606	529	87	576	14	338	185	48	292	312.49	103	458.8	471	196	368	95	97	2	44	23
10478	7866	5780	2	2428	1806	28838	497	51	465	5	416	160	27	305	333.98	109	473.48	460	132	353	136	69	5	60	37
11812	7884	5168	0	1834	1464	28498	551	137	628	35	304	173	68	262	306.37	143	455.04	504	214	379	72	84	7	30	19
13878	7512	4462	1	1196	880	28046	591	243	930	102	108	232	106	192	278.13	201	446.23	488	361	444	38	110	17	10	2
13944	7786	4566	6	898	656	27922	610	305	1013	134	70	238	132	159	273.25	249	439.43	491	392	454	29	88	12	2	2
14586	7600	4334	5	736	528	27834	669	341	1072	165	48	261	134	148	271.92	257	434.36	459	451	434	18	81	19	2	2
13878	7718	4570	8	990	718	27964	628	291	987	133	88	247	131	180	275.03	235	439.11	470	402	438	26	93	12	2	2
4696	13160	9502	757	458	262	28092	1162	9	644	0	14	60	4	86	279.12	115	435.15	350	311	316	13	43	0	0	1
3984	13632	9904	977	336	188	28054	1317	8	573	2	10	58	6	70	272.57	83	424.81	261	283	271	16	42	0	0	2
12066	8198	5212	6	1518	1132	28306	614	148	794	34	172	149	84	213	289.66	166	448.22	482	308	454	62	74	8	8	5
14186	7814	4502	6	752	558	27868	669	311	1120	157	56	258	121	142	269.31	283	431.25	484	466	427	15	91	16	0	2
13176	7702	4700	1	1416	1050	28192	637	198	809	69	136	192	95	212	281.71	170	448.21	477	322	415	43	96	13	10	5
5304	12574	9094	652	708	442	28174	1132	12	647	2	46	54	8	102	281.48	97	438.64	361	347	327	17	37	0	6	2
4206	13458	9756	879	406	240	28082	1223	5	582	1	16	57	3	72	274.48	98	429.4	313	280	309	21	40	0	0	0
11030	7880	5518	2	2174	1666	28682	520	90	559	17	358	170	51	288	317.56	94	462.07	486	192	363	104	88	5	52	24
4944	13060	9404	698	438	234	28088	1153	4	709	0	8	63	3	66	279.06	123	433.91	363	369	325	20	35	0	0	2
6238	12032	8662	495	758	426	28156	1008	29	730	7	38	82	13	117	286.21	99	444.37	386	371	357	30	48	2	2	4
4800	13060	9460	736	500	268	28100	1144	5	665	0	12	63	4	88	276.98	110	433.42	351	320	338	7	43	0	0	5
13718	7808	4558	4	1032	794	27998	641	280	959	113	82	220	122	175	274.38	237	441.68	476	372	446	31	89	22	4	0
13284	7826	4712	2	1232	934	28122	613	227	870	77	124	171	108	198	282.81	198	446.27	468	328	475	43	72	12	10	6
6296	12034	8534	470	752	508	28190	1018	24	739	7	62	77	15	105	286.39	113	442.79	412	394	351	27	49	0	4	2
4426	13392	9658	859	368	214	28066	1259	11	628	1	8	61	3	68	274.08	84	427	302	326	283	18	40	0	0	1
11004	7950	5562	4	2160	1612	28668	508	68	562	9	336	153	40	278	322.7	102	467.84	489	171	381	110	86	4	44	24
13212	7770	4776	1	1298	940	28134	569	215	848	63	130	183	113	182	284.47	200	446.66	522	303	468	45	94	13	6	6
5500	12568	9042	600	616	370	28134	1065	16	699	2	36	82	9	108	283.97	104	442.44	373	351	344	18	54	0	2	3
14422	7614	4352	8	814	606	27870	669	328	1044	163	58	271	122	157	271.75	238	435.86	461	453	415	20	95	22	2	2
5862	12396	8828	564	618	390	28128	1043	25	717	7	32	77	14	104	282.13	121	437.51	385	352	358	21	47	1	2	3
14502	7606	4338	5	774	576	27862	668	332	1087	159	66	256	132	157	269.87	262	432.5	463	454	440	13	87	23	0	1
12150	7924	5122	1	1658	1268	28378	581	134	715	31	230	171	76	226	298.55	140	451.76	494	260	417	67	94	5	24	18
7836	10940	7718	296	1016	632	28216	904	42	835	12	72	96	23	141	289.69	140	448.1	427	411	375	34	60	0	0	6
12432	7826	5018	4	1590	1218	28338	553	145	755	40	230	190	81	238	296.37	161	453.02	502	242	415	63	115	5	24	11
4356	13420	9694	885	378	214	28066	1251	5	604	0	4	64	5	74	271.39	95	426.12	292	296	300	15	48	0	0	3
4512	13332	9638	839	372	200	28062	1242	6	654	2	8	48	3	70	274.37	93	427.07	301	340	316	17	32	0	0	0
13616	7896	4624	3	1002	782	28020	659	231	1001	98	92	205	92	166	278.31	236	445.07	484	412	429	29	85	21	8	6

SUPPLEMENTARY INFORMATION:Monte Carlo Atomistic Simulation and Machine Learning Analysis of Na-K Eutectic Alloy in Condensed Phases, D. Reitz and E. Blaisten-Barojas, George Mason University, Fairfax, VA 22030

4240	13490	9794	884	348	176	28052	1237	7	610	0	4	64	4	67	272.29	92	426.52	306	292	303	14	38	0	0	4
4008	13608	9888	936	350	194	28056	1295	6	592	1	8	51	2	68	271.46	84	424.38	267	307	291	17	33	0	0	4
4738	13182	9456	723	442	272	28102	1135	5	658	0	12	65	4	90	276.95	104	433.37	351	326	337	15	42	0	0	2
14524	7590	4374	6	770	526	27824	626	333	1103	175	40	285	123	152	269.25	277	430.85	479	431	429	20	101	20	0	1
11954	8000	5292	2	1666	1218	28384	555	136	733	22	236	157	72	249	301.64	149	455.75	488	248	427	66	90	9	18	16
14154	7728	4456	8	870	638	27900	654	298	1050	153	52	257	112	148	271.01	258	434.7	490	428	425	22	93	15	2	0
14338	7660	4398	3	822	600	27872	660	314	1067	161	50	272	119	169	268.31	256	431.92	465	435	413	15	97	12	4	0
5228	12834	9216	668	520	296	28108	1109	10	691	0	14	52	7	86	276.81	118	433.48	374	346	356	14	31	0	0	1
11852	7846	5238	1	1854	1382	28462	558	105	687	28	250	176	50	273	304.65	127	458.14	459	241	402	74	101	11	40	16
5004	12954	9322	694	514	298	28106	1120	11	675	0	14	53	6	75	278.91	108	435.5	373	340	351	16	31	0	0	3
11420	7934	5466	1	1974	1430	28544	522	93	626	14	296	173	48	265	311.65	133	459.88	499	205	391	101	106	6	20	14
4978	12910	9316	687	574	332	28126	1150	11	653	1	16	58	10	89	278.16	96	434.39	347	352	334	16	37	0	0	3
7198	11366	8026	365	928	622	28224	935	40	767	13	66	84	19	132	287.99	128	446.08	442	383	368	25	53	1	16	3
7276	11510	8036	347	778	514	28168	913	36	830	4	52	83	22	132	288.27	147	444.72	431	406	399	23	53	0	0	4
14254	7688	4426	7	832	620	27886	628	319	1039	164	64	267	122	163	270.01	274	434	486	410	431	18	94	13	2	1
13596	7882	4642	7	1016	778	28010	662	253	979	118	90	228	103	158	276.6	231	441.98	483	407	416	38	87	13	6	1
13666	7766	4546	4	1078	854	28030	591	274	923	111	118	241	122	185	276.68	227	442.46	490	341	441	38	102	19	2	2
4704	12976	9464	734	616	348	28138	1164	8	600	0	30	49	6	88	278.38	84	435.7	352	315	319	15	28	0	0	3
5350	12644	9144	624	630	344	28132	1090	4	716	0	20	74	2	89	281.94	111	438.35	384	342	339	18	54	0	0	3
12636	7846	4910	3	1536	1154	28264	537	169	777	44	164	194	89	247	289.16	154	450.08	485	261	443	57	110	13	18	7
4360	13344	9692	860	438	238	28082	1262	4	627	0	10	63	2	74	275.47	88	431.18	293	317	281	18	41	0	0	3
6838	11496	8164	395	996	670	28252	940	25	724	6	84	86	14	126	288.62	115	445.75	455	349	348	28	54	0	4	2
5562	12590	9016	584	590	348	28130	1066	15	763	1	24	66	12	84	280.24	136	438.19	405	381	355	17	46	0	0	2
4566	13162	9562	780	508	290	28114	1198	3	639	0	26	72	2	95	277.19	95	432.81	326	326	291	11	50	0	0	3
4164	13554	9822	891	322	178	28050	1228	10	611	1	10	65	6	80	273.52	104	427.15	309	278	296	10	42	0	0	2
5406	12546	9088	598	696	390	28154	1089	14	693	1	28	61	6	90	285.6	110	439.97	396	370	334	17	37	0	0	2
14334	7616	4346	4	868	664	27900	648	330	1031	157	66	251	130	156	272.02	245	434.2	476	421	439	24	84	25	6	0
12876	7840	4804	8	1418	1094	28202	653	214	826	75	162	187	100	213	282.37	184	446.18	479	351	410	36	82	18	8	6
14356	7628	4356	4	850	640	27898	648	319	1043	163	66	253	118	153	271.17	255	433.43	485	437	432	22	78	14	2	0
12012	7894	5238	2	1748	1262	28410	577	126	707	31	244	184	59	243	304.44	152	456.8	517	234	373	58	110	11	10	16
4516	13322	9616	804	378	222	28072	1202	7	646	0	18	52	4	89	274.58	92	429.95	301	323	336	12	38	0	0	3
10968	7808	5430	0	2316	1790	28724	502	79	530	23	362	178	32	276	316.93	113	463.28	506	158	355	117	89	4	42	26
5346	12790	9142	636	522	304	28114	1138	7	753	0	10	66	5	85	279.21	123	435.18	367	392	322	15	50	0	0	1
6192	12092	8604	497	758	482	28172	1009	26	717	5	44	67	15	125	285.57	111	440.7	395	369	375	17	41	2	0	3
4546	13182	9570	779	518	284	28110	1207	6	632	0	10	61	5	86	278.43	97	434.65	315	326	305	19	44	0	0	2
5152	12860	9258	663	512	300	28110	1137	9	714	0	28	59	7	91	278.33	108	434.14	344	369	343	17	45	0	0	3

SUPPLEMENTARY INFORMATION:Monte Carlo Atomistic Simulation and Machine Learning Analysis of Na-K Eutectic Alloy in Condensed Phases, D. Reitz and E. Blaisten-Barojas, George Mason University, Fairfax, VA 22030

4308	13366	9700	858	446	256	28090	1243	8	599	0	12	64	4	70	273.49	108	427.51	322	286	283	12	45	0	2	3
11464	7920	5392	3	1972	1474	28542	551	84	663	14	268	169	47	258	309.07	132	458.75	501	226	388	84	107	3	46	18
14146	7686	4396	3	906	718	27942	668	297	1030	151	84	256	101	161	273.78	253	438.46	466	414	407	32	98	23	6	1
14370	7652	4360	5	798	620	27876	614	336	1048	171	76	267	129	150	270.99	259	433.03	492	406	451	21	84	19	0	3
4714	13134	9472	793	480	282	28094	1240	10	655	1	12	59	5	75	274.55	102	429.53	306	344	290	17	37	0	0	5
4412	13282	9652	824	474	262	28096	1217	4	615	0	14	69	2	84	279.18	85	435.19	315	301	288	19	50	0	0	1
4446	13328	9652	837	402	230	28076	1228	5	641	0	16	55	2	83	275.75	103	431.43	302	320	306	17	34	0	2	0
6318	11990	8562	442	756	490	28190	996	12	787	3	66	72	7	125	284.28	127	441.11	394	400	379	26	43	0	8	1
4160	13506	9814	917	376	200	28062	1263	6	591	1	6	62	4	73	271.69	82	425.71	290	292	291	14	43	0	0	3
4296	13476	9756	842	340	182	28056	1199	5	617	1	4	58	2	80	274.32	88	429.28	305	307	338	14	38	0	2	3
5122	12770	9232	687	620	364	28136	1134	9	672	2	26	55	5	85	280.79	107	434.9	366	345	334	18	35	0	2	2
4168	13474	9762	879	416	252	28088	1260	5	575	0	16	56	3	82	274.11	78	428.21	295	306	290	9	37	0	0	4
5136	12880	9246	674	522	310	28112	1143	4	722	0	18	71	4	103	277.66	113	435.47	338	363	327	14	46	0	0	1
14198	7644	4434	4	898	660	27908	646	316	1044	151	74	263	120	188	273.1	231	437.2	446	434	434	18	98	24	0	1
11050	7984	5496	1	2112	1632	28668	483	76	527	15	356	151	40	260	317.21	117	461.69	507	148	415	109	79	8	38	33
5636	12440	8966	567	694	390	28148	1061	13	703	8	22	71	2	104	281.11	103	437.77	404	361	336	12	45	0	0	2
5852	12216	8828	526	780	458	28184	1055	17	711	3	48	74	10	107	284.58	117	441.83	394	378	332	26	48	1	2	1
4804	13044	9422	737	530	302	28114	1179	3	669	0	12	74	3	98	277.63	103	433.64	325	347	308	15	48	0	0	1
13264	7704	4700	2	1366	986	28142	609	232	826	84	112	220	111	221	283.38	201	445.19	476	298	408	38	107	15	8	6
13426	7600	4698	7	1300	930	28104	596	272	847	93	140	205	137	196	277.42	207	443.94	499	314	447	38	93	12	8	2
13060	7864	4734	5	1284	1042	28162	582	204	883	72	164	211	97	206	282.28	201	447.21	496	330	428	54	96	8	14	2
14364	7652	4344	6	822	640	27896	670	317	1049	162	72	256	121	143	269.95	268	431.88	485	446	418	22	84	17	2	1
11224	8004	5382	1	2028	1620	28648	573	97	582	20	350	145	56	266	314.3	113	462.42	489	215	377	91	71	6	40	24
11794	7972	5342	0	1812	1276	28428	545	142	664	25	208	168	81	268	304.69	136	453.7	472	229	419	76	82	11	20	13
13216	7790	4656	3	1302	1036	28156	603	210	856	73	134	209	100	185	281.44	194	446.57	491	325	438	54	108	15	22	7
3836	13680	10000	993	352	176	28050	1329	9	561	1	6	62	6	63	270.75	84	423.71	272	265	253	13	45	0	0	2
3766	13758	10026	997	318	172	28048	1301	5	543	1	8	57	3	66	272.04	86	424.04	277	251	272	13	38	0	0	4
11414	7966	5322	0	1974	1544	28558	525	99	640	18	300	177	58	269	311.34	124	457.35	468	208	407	104	89	5	36	16
4904	13060	9394	733	448	264	28088	1159	10	680	1	18	61	5	88	275.89	109	431.87	329	343	339	16	39	0	0	3
13862	7772	4594	4	978	684	27950	640	306	1001	133	56	217	136	168	275.58	241	439.5	476	407	468	21	74	12	4	1
12008	8040	5194	0	1612	1262	28392	562	139	746	31	258	171	77	250	300.36	142	453.66	471	269	417	86	103	12	18	6
13830	7644	4480	4	1104	852	28026	632	260	914	118	108	251	101	188	276.87	210	446.51	455	360	414	45	105	16	6	3
3948	13740	9944	976	250	146	28040	1305	3	578	1	12	60	1	71	271.35	83	424.11	262	283	284	11	42	0	0	4
12766	7810	4918	3	1508	1082	28240	569	175	785	45	130	203	89	232	294.61	160	453.26	456	274	429	71	115	7	24	9
8470	10544	7254	253	1112	774	28244	860	60	772	18	82	112	33	167	286.87	138	446.49	438	380	377	29	57	2	8	3
12252	7942	5056	0	1594	1254	28354	563	136	756	35	236	173	73	242	297.82	158	453.35	477	259	429	77	98	9	20	8

SUPPLEMENTARY INFORMATION:Monte Carlo Atomistic Simulation and Machine Learning Analysis of Na-K Eutectic Alloy in Condensed Phases, D. Reitz and E. Blaisten-Barojas, George Mason University, Fairfax, VA 22030

6468	11760	8432	466	928	574	28224	1010	21	720	6	60	78	11	113	289.08	127	444.49	418	376	337	32	43	0	2	2
11972	7918	5162	2	1768	1340	28422	525	123	685	35	238	196	61	246	303.18	136	456.07	485	200	415	94	122	8	24	11
4480	13310	9638	833	402	226	28072	1228	12	635	1	16	64	9	76	273.59	106	427.72	313	320	297	13	47	0	0	3
4526	13218	9534	769	502	320	28126	1174	5	627	0	26	59	5	82	277.57	97	433.16	349	314	314	15	39	0	0	3
5326	12690	9114	651	596	374	28140	1098	17	635	4	40	68	11	85	283.7	92	439.96	372	328	343	24	44	0	0	5
4784	13176	9494	753	382	214	28066	1186	6	698	0	16	59	4	81	276.06	104	432.74	320	361	326	19	47	0	0	0
4072	13576	9836	936	352	212	28062	1266	9	565	2	14	51	5	69	271.84	91	424.83	283	266	304	18	33	0	0	3
14498	7664	4332	4	748	560	27848	669	328	1056	163	44	267	118	144	271.23	244	434.17	463	454	417	25	92	20	2	3
5442	12484	9024	568	740	446	28180	1071	13	677	1	44	60	10	109	286.29	116	441.12	380	363	345	22	34	0	0	3
11704	7856	5270	0	1916	1442	28498	567	109	649	18	278	146	60	274	302.19	121	456.2	479	229	408	76	71	9	30	10
13788	7852	4576	8	960	726	27978	631	265	980	133	70	241	105	189	275.56	225	441.66	469	386	439	23	89	9	6	1
4826	12880	9374	729	664	380	28148	1164	9	616	0	22	59	7	92	278.2	94	434.07	350	324	309	14	41	0	2	5
13148	7706	4696	1	1390	1074	28194	598	206	796	74	168	203	96	226	284.94	166	446.29	478	309	417	49	97	12	12	4
11618	7958	5340	0	1906	1396	28498	548	104	656	30	260	168	54	261	308.32	147	457.15	507	219	385	76	79	4	20	19
4348	13450	9726	844	340	184	28054	1236	11	610	1	6	61	6	73	274.6	81	427.88	299	328	304	17	44	0	0	2
14354	7572	4376	3	870	640	27888	605	335	1029	158	72	257	129	156	271.99	257	434.04	496	398	453	22	89	22	4	2
5958	12326	8746	536	658	430	28158	1048	15	750	2	38	63	7	102	283.8	127	440.05	406	382	356	14	39	1	2	3
10684	7890	5688	1	2240	1762	28788	498	60	505	11	468	155	27	299	333.22	88	470.12	482	146	347	118	87	2	54	35
13578	7882	4608	6	1056	812	28022	624	261	951	134	84	241	90	182	277.12	228	442.51	492	368	415	30	93	22	2	3
5370	12732	9136	616	546	316	28122	1100	6	751	0	22	58	3	102	282.58	107	437.12	369	385	353	12	39	0	0	0
4840	13126	9438	738	416	244	28080	1159	6	683	1	16	56	2	73	275.45	102	430.73	354	354	337	13	36	0	0	2
4446	13236	9602	815	508	298	28108	1224	11	602	2	18	50	7	83	276.18	92	431.78	318	304	304	13	29	0	0	2
4694	13082	9508	752	510	286	28108	1163	10	623	0	26	56	7	75	279.56	85	435.21	348	325	331	20	43	0	2	2
12690	7906	4874	7	1446	1138	28252	630	202	777	62	188	175	97	217	285.51	165	450.6	465	302	437	52	86	17	10	8
10552	7730	5712	1	2478	1858	28842	472	60	466	11	468	153	28	306	332.37	87	470.22	492	138	356	126	84	3	40	35
3752	13770	10018	1014	322	182	28050	1315	8	536	1	6	55	5	60	269.95	84	422.05	274	253	272	16	39	0	0	2
5586	12376	8894	581	790	500	28188	1073	24	650	4	42	64	16	106	281.84	95	439.54	374	344	349	29	41	0	0	1
4792	13168	9468	781	406	230	28070	1207	6	679	0	6	64	4	76	277.5	97	432.11	315	345	312	18	46	0	0	2
10286	7826	5836	0	2466	1918	28914	495	51	472	5	508	152	33	300	329.69	83	471.78	494	156	346	118	67	2	68	54
4830	13048	9444	741	496	266	28094	1175	5	660	1	10	71	2	87	275.7	93	431.98	339	337	311	13	51	0	0	1
13526	7948	4684	9	966	772	28012	630	251	1003	117	104	205	111	173	276.91	244	442.69	478	402	470	31	68	9	12	2
5194	12736	9194	661	618	368	28144	1135	13	652	0	34	58	7	111	283.1	89	439.92	334	347	332	20	35	0	0	1
13402	7880	4708	5	1132	836	28054	656	251	913	113	94	224	92	162	276.87	222	441.75	494	364	405	41	86	17	2	3
4990	12836	9284	680	628	380	28152	1131	5	650	0	32	61	3	100	276.46	99	432.55	370	323	319	11	47	0	2	1
14154	7822	4480	7	780	592	27878	635	317	1074	163	42	262	117	159	269.84	273	432.48	493	438	432	11	83	21	6	2
4420	13280	9634	800	482	274	28104	1206	3	617	1	14	55	2	78	275.68	97	430.51	331	321	306	14	33	0	0	3

13374	7858	4696	2	1146	890	28104	644	221	920	76	132	209	102	199	281.53	194	445.27	460	360	425	42	102	19	6	4
10688	7862	5566	0	2344	1854	28800	494	75	483	13	428	149	39	300	322.77	96	466.03	480	139	373	132	76	5	50	22
14156	7766	4392	7	834	684	27908	657	307	1048	163	72	259	113	168	267.59	264	429.88	471	443	423	18	81	12	2	0
5506	12518	8998	594	688	420	28166	1073	7	724	0	34	71	5	100	282.72	120	441.09	382	362	343	27	46	0	2	0
5928	12034	8722	499	920	554	28214	1008	20	664	6	54	77	9	141	287.05	86	443.03	384	347	354	25	46	1	2	1
6532	12014	8468	433	660	430	28152	988	19	822	1	46	76	12	111	285.07	119	441.13	408	423	387	24	54	1	2	1
4780	13062	9442	707	524	296	28118	1129	6	655	0	14	67	5	98	278.26	99	435.23	350	322	332	16	36	0	0	2
14380	7586	4306	2	890	676	27900	656	325	1036	168	56	263	125	147	268.35	268	430.28	501	424	404	17	89	13	6	3
4802	13090	9476	743	462	244	28086	1162	6	684	0	12	60	5	80	276.37	108	433.1	337	335	335	18	46	0	0	2
4396	13336	9670	884	422	238	28076	1265	10	619	1	14	48	7	83	273.09	94	428.22	281	304	304	12	30	0	0	2
11572	7940	5386	1	1936	1388	28494	574	102	686	31	248	161	38	269	301.67	141	455.77	493	238	377	77	79	5	20	9
7584	11142	7802	354	922	658	28222	923	51	797	14	102	101	22	138	289.68	131	447.8	435	391	354	31	64	5	12	4
13880	7772	4582	7	956	682	27936	581	283	1002	148	58	270	102	187	274.64	225	439.62	493	376	440	18	107	12	6	4
4112	13584	9836	902	312	190	28054	1245	7	585	1	18	62	3	73	273.06	79	426.38	288	273	303	20	41	0	2	2
4132	13472	9804	918	418	238	28082	1262	6	557	1	18	60	3	64	273.16	89	427.35	298	265	287	25	50	0	0	0
4422	13264	9630	811	492	282	28106	1206	4	600	0	12	64	3	84	278.8	95	435.65	326	309	297	14	46	0	4	2
4338	13348	9710	853	430	236	28080	1237	7	612	1	18	56	4	75	275.19	78	430.54	295	318	312	19	36	0	0	3
12604	7782	4944	0	1532	1190	28306	568	184	754	48	232	189	99	237	291.91	163	451.94	484	279	422	62	103	11	22	8
4174	13528	9798	915	348	202	28064	1270	8	593	1	14	56	5	69	271.83	74	425.44	301	290	288	11	38	0	0	1
12028	7786	5072	0	1862	1420	28434	546	142	667	36	228	187	68	266	301.5	137	455.56	489	226	392	76	93	9	38	11
12984	7904	4862	3	1274	966	28150	640	208	889	82	150	215	92	209	282.71	195	446.24	482	337	396	37	110	13	8	4
13352	7618	4576	2	1396	1074	28174	623	238	791	88	144	199	114	219	283.49	177	447.51	464	302	431	42	82	16	14	6
4618	13116	9504	776	526	324	28124	1167	13	607	0	36	54	10	75	277.56	98	434.54	359	296	317	16	38	0	0	4
3894	13708	9952	1014	300	180	28052	1336	9	584	1	16	59	8	73	269.93	98	422.5	259	258	258	9	40	0	2	2
5160	12888	9236	643	506	306	28118	1096	7	706	1	22	61	3	92	277.98	116	433.39	377	355	352	15	41	0	0	1
14186	7792	4448	5	804	608	27888	653	307	1074	162	48	245	110	132	271.81	283	434.31	511	428	433	19	72	17	2	0
10870	7950	5608	0	2204	1674	28740	525	56	530	5	400	158	37	308	322.4	96	465.41	487	166	363	102	91	4	32	23
6638	11726	8358	415	874	550	28210	962	26	764	5	62	85	19	126	287.65	139	443.04	430	372	363	26	55	0	2	1
11342	7828	5310	0	2136	1660	28650	517	81	546	15	324	162	44	282	316.58	104	458.97	469	181	393	103	88	8	42	32
11966	7962	5204	2	1710	1298	28410	575	126	709	32	252	170	60	254	302.99	133	456.79	478	255	395	77	86	7	18	9
10946	7904	5782	1	2182	1486	28676	501	80	568	17	344	172	40	301	336.3	100	473.53	466	182	358	117	87	5	30	25
11306	7968	5456	1	1994	1508	28588	519	94	601	18	322	151	44	287	314.41	107	461.18	479	208	413	89	81	5	32	18
4364	13314	9658	839	476	272	28094	1219	7	607	0	10	54	5	72	274.95	107	429.87	329	298	297	19	31	0	0	0
3798	13702	9976	1004	360	214	28066	1311	6	538	1	16	52	3	68	270.05	96	422.7	280	245	269	13	33	0	0	1
4836	13108	9444	743	444	244	28082	1191	9	691	0	6	65	7	73	276.07	113	431.64	343	359	304	13	44	0	0	3
7202	11172	7922	318	1132	766	28298	924	27	756	6	100	88	16	149	287.13	135	445.98	437	378	358	31	54	2	4	5

SUPPLEMENTARY INFORMATION:Monte Carlo Atomistic Simulation and Machine Learning Analysis of Na-K Eutectic Alloy in Condensed Phases, D. Reitz and E. Blaisten-Barojas, George Mason University, Fairfax, VA 22030

14104	7634	4462	2	962	702	27946	586	303	984	134	74	253	129	203	277.02	220	442.39	438	358	470	36	106	18	8	2
13722	7836	4600	6	968	754	27986	617	278	983	130	102	241	112	179	275.77	226	440.27	488	383	427	31	92	17	4	1
5044	12786	9264	669	642	380	28150	1163	11	658	2	32	61	7	86	281.84	97	437.51	358	371	307	16	29	0	2	4
4506	13270	9612	833	434	244	28080	1253	9	639	0	14	53	4	84	275.1	94	428.91	287	328	298	14	32	0	0	3
10532	7856	5752	1	2418	1782	28802	476	44	484	5	422	179	26	327	334.08	77	471.5	467	130	350	106	85	2	40	42
12814	7896	4876	4	1346	1074	28216	619	182	860	53	190	189	98	210	285.41	181	449.2	457	324	442	59	107	10	18	8
3948	13568	9904	947	402	230	28074	1290	4	548	1	22	61	2	61	270.34	77	423.05	288	276	272	19	42	0	0	4
5012	12876	9316	696	564	326	28122	1123	14	653	3	26	60	8	90	279.81	102	436.71	356	320	340	22	32	0	2	1
4714	13142	9490	728	464	272	28104	1168	6	649	0	20	60	4	91	277.37	84	433.8	326	349	332	16	42	0	2	3
4638	13154	9534	793	476	272	28096	1217	6	667	0	20	72	2	80	276.42	92	431.3	323	335	284	18	56	1	2	1
14236	7602	4386	2	936	696	27934	607	320	1009	153	74	261	125	172	272.38	261	437.45	504	400	427	18	88	17	4	2
4026	13616	9882	940	328	186	28048	1289	11	589	1	10	65	6	74	270.5	82	423.84	268	296	279	17	43	0	0	2
4510	13196	9612	815	490	264	28090	1189	7	626	0	18	59	4	84	275.19	83	430.46	320	301	326	13	40	1	0	5
3868	13696	9968	992	316	184	28052	1310	8	569	2	20	45	3	67	270.74	82	423.37	268	268	289	13	33	0	0	2
6006	12162	8736	513	774	460	28178	1034	21	697	2	36	77	18	128	287.76	105	444.02	382	369	348	19	46	0	4	3
14326	7786	4388	6	722	584	27870	646	326	1091	152	62	249	135	146	271.63	267	434.23	482	452	452	19	83	20	0	1
14338	7676	4374	8	818	616	27876	646	321	1072	174	46	270	112	162	268.12	281	429.23	476	434	420	19	88	12	8	0
4002	13636	9886	943	334	192	28058	1255	5	568	1	8	57	2	64	273.23	106	428.05	307	251	300	10	36	0	0	4
4190	13438	9766	891	426	244	28078	1252	9	573	0	14	67	4	68	274.71	88	428.53	302	282	281	17	42	0	0	5
12022	7930	5216	1	1726	1246	28362	585	118	734	30	202	190	63	244	300.86	137	451.22	466	261	404	73	116	6	18	10
4546	13330	9584	821	370	230	28074	1200	6	665	2	8	66	2	80	273.42	106	429.1	319	305	310	14	47	0	6	4
5274	12604	9136	588	716	410	28168	1070	6	686	1	24	68	3	105	284.18	112	439.97	397	354	337	16	45	0	4	1
13312	7840	4734	7	1168	896	28096	646	237	890	102	138	215	107	195	279.03	175	443.56	468	385	426	36	91	14	8	4
11716	7834	5138	0	1978	1558	28550	559	128	607	29	290	163	75	275	304.53	121	453.21	484	207	398	83	90	7	36	12
11240	7868	5456	0	2092	1582	28624	516	83	554	17	352	169	38	269	315.44	108	461.53	511	201	362	105	79	3	34	18
13076	7770	4764	3	1354	1044	28190	627	221	803	70	164	199	113	205	284.6	177	449.9	464	313	434	44	98	14	18	13
5154	12860	9260	680	514	294	28102	1142	10	689	1	18	75	7	89	280.7	101	435.54	334	357	325	26	51	0	2	2
14130	7694	4492	4	884	636	27904	637	308	1025	161	66	257	109	173	273.16	232	439.94	456	399	442	26	81	15	2	1
11094	8096	5596	2	1980	1480	28598	535	86	621	12	312	161	56	284	317.01	116	461.07	503	217	391	78	93	2	38	20
5404	12500	9004	610	738	480	28180	1093	15	646	4	48	70	7	114	281.92	99	439.91	372	347	322	17	43	0	6	4
14538	7608	4302	6	772	576	27842	612	355	1057	174	40	262	141	145	268.79	285	431.4	500	412	455	16	76	18	6	2
11238	7952	5420	0	2032	1600	28644	531	88	585	16	360	166	50	270	314.09	97	462.61	477	200	381	114	90	3	42	24
3982	13578	9880	946	388	226	28070	1304	6	565	1	16	69	4	67	273.97	78	428.72	274	290	258	19	43	0	0	4
4628	13056	9476	751	602	358	28144	1175	4	604	0	22	62	3	85	280.92	86	435.95	344	308	304	21	41	0	0	2
4710	13110	9472	758	494	298	28110	1162	4	635	1	24	62	1	97	276.18	107	432.46	331	320	325	16	41	0	2	1
3942	13776	9976	1003	208	106	28010	1316	13	590	1	2	63	9	77	270.73	85	422.3	246	275	278	10	44	0	0	2

SUPPLEMENTARY INFORMATION:Monte Carlo Atomistic Simulation and Machine Learning Analysis of Na-K Eutectic Alloy in Condensed Phases, D. Reitz and E. Blaisten-Barojas, George Mason University, Fairfax, VA 22030

5218	12850	9228	644	502	288	28104	1110	6	728	0	18	65	6	93	280.55	108	436.49	352	380	357	18	44	0	0	2
14446	7626	4332	5	804	606	27876	675	326	1062	165	62	263	121	148	267.36	273	430.5	485	451	409	15	90	19	0	0
5696	12466	8932	606	642	378	28140	1117	15	700	1	26	67	10	111	283.09	101	438.72	354	375	327	14	43	0	0	2
13688	7634	4524	2	1178	912	28078	575	252	875	92	132	244	111	202	281.56	193	445.93	472	316	432	53	118	24	10	3
7382	11184	7878	306	1048	690	28258	882	35	734	5	68	88	26	138	287.89	125	445.76	465	382	385	27	53	0	8	6
5328	12730	9128	626	568	350	28136	1107	8	719	0	30	61	6	79	283.46	99	437.67	377	369	346	23	48	0	2	3
12278	7870	4932	2	1696	1366	28410	552	157	680	49	262	188	72	258	294.33	160	453.12	507	221	394	65	94	16	4	9
7920	10898	7648	294	1000	656	28212	866	46	802	10	86	109	27	150	286.6	152	446.43	441	367	384	34	66	1	4	3
14440	7608	4394	6	796	560	27854	648	333	1059	167	56	269	129	152	270.16	273	432.98	471	441	434	18	93	18	0	3
3934	13628	9934	971	362	192	28058	1312	9	572	1	8	58	3	69	271.38	76	424.73	273	283	265	15	38	0	0	1
10566	7812	5740	1	2386	1808	28818	503	69	469	7	448	156	27	321	331.59	87	473.19	449	140	349	123	69	8	44	30
5914	12362	8828	490	628	374	28138	997	12	781	1	32	84	5	96	285.16	134	441.39	441	394	355	16	55	0	0	2
3630	13806	10034	1008	366	228	28072	1330	6	510	1	8	55	4	65	270.2	81	422.62	265	250	259	16	32	0	0	4
4562	13280	9596	801	408	226	28084	1217	8	658	0	12	54	6	75	277.77	108	430.98	319	331	312	15	33	0	0	2
4088	13634	9890	915	266	142	28034	1250	8	619	1	14	63	4	73	274.27	94	427.03	287	298	302	15	39	0	0	2
4352	13428	9726	869	354	186	28050	1243	9	636	0	4	64	6	66	273.08	108	427.64	300	303	300	20	51	0	0	1
5334	12742	9128	607	556	340	28126	1078	5	736	0	24	76	3	105	280.23	123	436.42	374	360	345	14	52	1	2	3
4840	13022	9424	738	506	290	28108	1168	8	672	1	26	57	3	74	277.26	114	433.89	359	335	312	16	40	0	0	3
14184	7638	4420	8	920	684	27922	676	309	1031	159	72	251	117	165	270.83	249	434.27	471	433	416	16	81	16	4	1
4418	13306	9630	819	444	270	28090	1233	14	613	1	22	54	8	70	274.95	98	430.52	307	321	304	23	32	1	0	2
11188	7798	5378	1	2200	1708	28682	559	92	532	24	362	167	48	280	316.71	88	461.47	472	178	350	114	74	4	48	21
4544	13216	9578	792	480	268	28098	1203	5	621	0	12	66	2	90	277.33	98	433.04	320	320	300	12	38	0	0	3
4642	13224	9568	789	392	226	28084	1212	6	689	1	32	59	3	77	277.42	115	433.46	316	341	315	16	41	0	0	2
4152	13540	9800	897	342	210	28064	1272	4	611	0	20	56	3	65	273.09	94	426.56	294	311	288	18	37	0	0	1
11170	7826	5528	0	2180	1568	28636	533	77	568	15	342	159	47	269	317.06	137	458.68	502	177	381	100	88	4	22	21
4460	13282	9620	776	454	264	28098	1184	3	636	0	16	68	2	96	277.47	94	433.18	309	329	320	18	46	0	2	2
13908	7758	4554	7	960	700	27954	663	276	997	138	70	232	105	170	274.1	215	439.12	463	406	441	19	82	14	2	5
4524	13282	9630	829	404	216	28070	1239	11	639	1	14	58	7	76	274.91	91	429.15	298	326	303	16	43	0	0	2
4950	13048	9400	705	440	234	28078	1113	8	704	1	6	67	5	88	277.95	111	434.12	345	324	359	19	49	0	0	3
11064	7852	5654	1	2188	1540	28674	520	55	557	9	326	164	32	298	323.85	98	466.93	469	180	374	102	102	1	48	26
13406	7760	4688	1	1202	904	28102	618	244	915	77	134	197	134	197	282.17	191	444.25	466	361	466	38	98	13	8	6
4272	13522	9774	896	296	168	28046	1249	6	632	1	14	60	2	81	273.36	91	428.31	284	300	309	11	44	0	0	2
14454	7522	4312	2	896	650	27896	646	320	1030	166	54	273	105	146	272.16	268	436.29	505	411	407	19	96	25	8	1
13742	7612	4468	4	1166	932	28066	610	273	893	96	132	209	133	191	277.79	201	443.34	463	329	459	48	96	17	12	2
11280	7932	5422	0	2020	1570	28608	486	90	605	17	346	185	50	290	309.9	128	461.11	497	171	384	93	103	5	30	26
3958	13642	9900	964	340	206	28064	1295	8	548	2	18	50	5	77	271.38	76	424.42	265	269	293	14	30	0	0	2

7748	10968	7688	256	1072	712	28274	913	36	827	7	70	80	22	141	287.72	140	449.1	448	436	367	33	45	1	16	4
11722	7990	5266	2	1770	1400	28478	553	106	664	26	306	169	51	244	305.28	130	459.77	495	228	398	86	90	7	24	19
4560	13224	9554	814	472	272	28088	1221	8	638	0	6	65	4	81	277.43	109	432.07	320	314	288	13	45	0	0	3
11786	8068	5356	3	1754	1234	28412	569	109	733	18	196	164	68	234	301.81	148	455.85	505	258	425	58	107	5	18	22
10682	7818	5658	2	2396	1794	28814	501	65	463	11	414	148	34	276	332.71	100	470.03	514	137	346	122	76	2	52	37
4078	13508	9810	904	412	254	28086	1255	5	577	1	24	58	2	84	273.18	94	427.61	276	277	299	20	36	0	0	3
4376	13228	9648	835	542	302	28114	1229	8	591	2	18	53	4	74	275.2	91	429.97	324	297	294	20	37	0	0	0
4208	13550	9802	911	310	170	28046	1284	8	638	1	6	66	4	61	273.86	106	429.04	300	316	268	11	48	0	0	3
4356	13464	9718	881	322	182	28050	1259	9	651	1	8	63	5	81	272.5	89	425.89	278	322	303	11	40	1	0	3
4630	13200	9514	765	452	282	28104	1184	7	656	0	26	54	4	84	276.54	104	430.91	337	341	322	12	37	0	0	2
3986	13578	9874	939	400	230	28080	1273	8	528	1	12	54	4	80	272.61	76	425.34	295	262	283	9	34	0	0	1
11622	7898	5260	1	1914	1496	28532	524	111	627	23	312	175	60	309	310.72	126	458.62	453	204	397	80	99	6	28	20
11326	7816	5462	0	2056	1560	28636	515	83	586	18	378	182	37	273	324.5	108	465.03	487	195	368	104	87	7	34	26
5588	12616	9034	576	542	310	28112	1077	12	758	1	22	64	9	89	281.14	116	437.13	370	399	370	22	41	0	0	3
4672	13158	9494	750	466	286	28102	1172	6	631	1	26	68	2	73	275.89	93	430.76	357	344	310	13	40	0	0	4
7160	11244	8036	413	1036	660	28220	960	39	692	12	76	96	18	116	289.13	108	445.17	423	354	346	42	56	2	8	3
4266	13442	9740	888	394	220	28068	1272	12	617	2	6	52	8	75	272.27	96	425.25	287	303	292	12	38	0	0	3
4312	13206	9684	855	576	316	28120	1278	2	601	1	26	56	1	92	275.88	73	430.36	276	322	272	17	39	0	0	3
4994	12896	9308	682	552	338	28124	1149	8	676	0	36	67	5	89	282.9	97	440.03	358	361	310	19	48	0	0	0
14262	7698	4436	6	804	604	27872	635	317	1071	169	62	263	112	147	269.57	279	431.94	493	434	437	17	84	17	6	3
13616	7812	4634	5	1020	800	28012	647	277	956	129	126	235	112	183	275.84	226	440.05	460	398	429	35	86	19	4	2
3936	13636	9918	965	358	202	28060	1314	6	579	2	10	52	2	72	270.38	80	423.78	272	284	272	9	34	0	0	3
3938	13684	9934	955	308	174	28048	1295	9	584	2	10	60	4	74	269.55	81	422.85	272	283	281	8	36	0	0	5
11780	7892	5286	1	1896	1354	28456	575	107	679	28	226	165	50	238	299.8	137	456.8	519	249	387	67	98	6	22	18
4336	13416	9712	874	380	212	28064	1233	6	621	1	8	61	3	71	273.17	98	427.89	302	297	312	16	37	0	0	3
5280	12624	9100	614	700	428	28164	1080	10	674	0	32	57	7	104	281.21	111	437.18	388	334	344	18	40	0	0	0
4094	13572	9842	916	338	196	28056	1257	7	580	2	14	59	3	66	270.9	82	423.26	298	284	297	16	39	0	0	2
11544	7846	5308	0	2026	1516	28566	556	97	589	18	300	149	52	283	311.62	129	459.42	471	192	392	89	75	6	22	16
4028	13596	9878	931	350	194	28054	1284	9	586	1	8	66	4	76	271.45	78	425.1	271	295	279	18	39	0	0	1
4412	13470	9712	874	274	154	28034	1236	2	649	0	12	79	1	66	273.69	105	427.69	308	311	288	16	55	0	0	2
11200	7692	5378	0	2244	1748	28716	489	89	525	17	406	164	46	291	323.09	111	466.11	488	156	370	115	82	8	48	30
5150	12770	9224	662	606	358	28140	1153	7	685	2	30	66	4	94	280.48	99	437.85	341	369	315	24	47	0	2	1
13712	7618	4494	2	1194	920	28066	608	271	875	118	118	227	115	214	276.26	187	445.35	459	345	442	34	88	17	10	3
11042	7780	5530	2	2290	1664	28690	510	85	510	14	350	166	44	282	323.65	104	467.19	501	166	360	118	74	6	32	19
13870	7888	4550	6	886	690	27958	653	269	1006	123	70	223	112	156	274.78	230	439.05	470	416	456	33	86	12	2	1
12562	7954	4952	2	1502	1130	28268	602	164	795	50	152	170	97	192	284.69	169	451	504	300	450	60	85	8	14	8

SUPPLEMENTARY INFORMATION:Monte Carlo Atomistic Simulation and Machine Learning Analysis of Na-K Eutectic Alloy in Condensed Phases, D. Reitz and E. Blaisten-Barojas, George Mason University, Fairfax, VA 22030

3934	13730	9934	978	272	162	28040	1302	7	570	1	8	73	3	66	270.4	89	423.81	283	278	257	12	44	0	0	2
4850	13080	9420	743	454	268	28092	1180	10	690	1	20	62	6	84	277.29	108	432.38	334	341	316	17	43	0	0	0
11192	7936	5368	0	2074	1670	28644	540	82	567	18	348	168	36	272	315.61	110	464.99	482	190	355	114	94	5	56	23
12568	7932	5012	1	1458	1092	28260	596	162	828	51	186	178	88	214	285.16	162	450.75	492	313	440	57	95	7	10	4
4426	13440	9676	854	316	182	28044	1245	9	639	0	4	61	5	58	272.65	94	428.78	316	335	288	15	36	0	0	4
13524	7854	4616	5	1094	850	28044	609	251	938	122	106	221	102	185	276.17	231	442.87	476	356	460	39	84	11	0	2
14604	7632	4352	1	696	490	27814	639	341	1123	177	38	275	128	144	268.7	277	429.99	479	475	443	13	91	17	2	2
4192	13604	9786	883	278	180	28052	1258	7	621	2	10	53	2	67	274.34	95	427.88	304	310	300	10	29	0	2	2
4200	13326	9724	834	536	312	28122	1218	3	555	0	24	65	1	80	275.14	91	431.96	335	276	276	15	38	0	0	3
4056	13604	9872	930	330	182	28052	1269	7	583	1	8	64	5	68	273.4	95	425.63	292	284	276	19	44	0	0	1
14378	7530	4348	4	928	658	27900	617	332	994	166	54	258	125	152	272.92	238	438.2	496	422	442	26	81	18	4	1
4192	13474	9788	868	398	214	28072	1247	6	593	1	6	47	3	70	274.6	85	429.2	298	308	312	14	29	0	0	5
6506	11720	8396	458	922	600	28230	978	29	708	9	82	75	15	127	287.72	131	446.22	416	332	359	27	47	1	4	6
4006	13626	9902	952	328	178	28048	1295	9	578	1	8	58	4	57	270.83	99	423.31	288	277	279	16	39	0	0	2
12256	7900	4942	1	1626	1374	28402	573	162	701	51	280	169	84	246	293.54	138	452.23	487	254	419	68	74	5	20	11
4548	13320	9596	819	366	220	28066	1173	14	644	1	14	60	7	80	273.23	117	428.13	331	293	327	17	45	0	2	1
5118	12724	9240	650	666	372	28144	1133	11	659	1	24	65	6	85	280.04	88	436.82	375	347	312	22	43	0	0	0
14208	7670	4374	10	888	702	27926	632	299	1031	162	78	270	111	150	270.44	265	433.81	524	414	405	15	94	14	6	2
10966	7938	5582	1	2160	1624	28678	483	73	567	10	384	183	36	314	320.64	96	466.7	485	185	363	105	98	7	22	24
5354	12782	9178	626	504	274	28106	1087	7	723	0	14	68	3	85	279.95	129	436.43	396	356	342	14	47	0	0	0
5010	12888	9310	677	556	332	28130	1146	3	682	0	32	56	2	84	277.54	88	433.65	364	357	325	16	43	0	2	2
4186	13542	9792	912	330	194	28054	1275	10	598	2	8	61	5	72	271.9	93	425.79	285	299	286	15	34	0	2	1
14150	7598	4388	7	966	748	27946	637	314	966	159	86	263	125	158	271.68	239	435.81	491	400	415	29	92	18	8	2
4646	13216	9520	764	430	262	28094	1179	4	650	0	18	68	4	99	277.51	99	433.98	323	324	317	10	47	0	2	0
4164	13518	9816	912	354	194	28058	1269	9	602	1	12	56	5	66	272.32	96	425.58	298	298	293	10	40	0	0	3
12994	7786	4832	0	1364	1044	28228	594	174	803	48	200	169	91	240	292.69	153	449.95	459	298	456	49	85	14	6	4
14150	7648	4394	4	926	726	27936	636	322	1017	173	88	263	111	157	272	250	437.35	485	414	422	29	79	18	2	2
4984	12984	9342	715	496	284	28102	1168	7	683	1	12	73	5	88	279.55	100	435.15	342	348	309	13	46	0	0	3
3956	13712	9924	962	268	166	28042	1300	6	594	1	14	72	5	61	270.48	90	423.36	291	295	257	11	50	0	2	3
12122	7820	5146	1	1750	1294	28400	575	151	663	39	260	178	72	264	299.78	153	453.88	506	227	385	50	90	15	6	12
5260	12706	9134	638	620	392	28154	1117	12	684	1	42	59	8	98	280.84	114	437.51	364	362	332	20	36	0	0	2
13722	7794	4562	9	1032	796	28004	612	280	954	121	94	220	125	175	276.22	231	442.53	490	368	462	28	84	16	2	3
4756	13094	9492	759	476	260	28100	1187	4	651	1	22	69	3	81	277.27	107	433.64	334	336	309	14	43	0	0	4
4096	13560	9840	937	364	198	28060	1284	6	586	1	0	65	4	78	271.32	91	424.3	295	289	268	5	45	0	2	1
4940	12934	9328	689	578	338	28134	1143	4	672	0	16	65	3	88	277.35	120	433.39	371	334	315	12	43	0	0	2
10862	7768	5626	0	2328	1736	28782	508	76	461	12	396	154	36	289	334.18	77	470.38	475	148	371	117	76	5	62	35

4668	13098	9504	740	516	300	28116	1155	9	624	0	30	57	6	82	277.48	82	432.89	350	329	328	19	38	0	0	1
4854	13012	9382	717	530	322	28126	1154	5	670	0	24	62	4	89	277.17	101	431.56	354	343	319	16	38	0	2	1
12790	7860	4930	1	1438	1046	28228	594	185	800	76	142	200	80	197	287.23	173	451.35	513	291	410	55	89	12	22	9
4694	13090	9480	730	534	304	28118	1166	6	639	0	16	48	4	84	279.75	107	434.93	345	331	330	14	34	0	0	4
4110	13552	9816	900	362	216	28070	1266	5	594	1	14	55	3	59	271.26	82	423.92	299	305	296	20	37	0	0	2
5524	12590	9028	604	604	360	28134	1086	7	749	0	28	67	4	94	280.89	126	436.23	373	377	351	22	42	0	0	2
13570	7696	4526	1	1190	962	28098	627	246	887	88	144	200	109	179	278.68	196	445.55	468	349	443	55	85	20	10	5
4906	13010	9384	712	498	290	28110	1174	5	687	0	20	53	4	78	276.51	99	433.31	341	360	331	14	40	0	2	5
11168	7902	5486	1	2112	1602	28666	518	83	547	24	358	177	35	281	319.68	116	465.77	486	154	353	115	93	7	34	23
12738	7866	4900	1	1450	1092	28224	622	205	773	57	170	167	100	180	286.59	177	449.4	497	311	444	56	77	14	8	9
4824	13150	9456	781	398	230	28072	1199	10	680	1	14	64	6	76	276.66	105	431.54	333	343	307	12	45	0	0	2
4156	13508	9792	923	376	224	28074	1279	11	583	2	18	64	8	78	271.02	85	423.55	282	284	279	15	45	0	0	1
3882	13758	9986	985	254	140	28030	1278	5	576	1	10	70	2	69	270.89	95	425.25	281	259	282	12	47	0	0	3
13562	7892	4714	8	1014	730	27994	625	273	956	112	80	207	127	161	276.13	222	440.67	483	380	473	37	79	16	2	1
4316	13400	9720	838	402	222	28070	1205	7	603	1	10	59	4	72	272.7	88	427.17	328	292	308	17	39	0	0	1
14086	7684	4446	5	914	710	27932	628	314	1013	153	84	254	125	147	271.38	272	435.69	507	413	425	28	85	20	6	2
13656	7802	4600	8	1052	814	28042	639	292	900	117	108	224	130	175	276.6	214	444.14	497	372	421	27	89	19	10	5
12240	7824	5106	1	1716	1260	28388	585	149	704	38	212	188	74	241	304.18	139	454.75	495	257	383	68	105	13	28	13
6370	11880	8482	448	870	550	28206	996	21	739	4	48	83	11	124	285.94	136	442.41	409	362	353	28	49	1	6	1
11326	7904	5378	1	2042	1582	28602	569	93	586	13	326	158	50	271	315.03	108	459.48	457	225	381	98	82	7	42	24
4426	13332	9648	814	422	242	28082	1205	8	618	0	10	52	4	78	276.35	88	430.46	325	313	317	13	34	0	2	2
14448	7752	4368	7	672	538	27840	653	320	1124	165	60	257	116	142	269.96	306	432.5	488	444	439	14	86	21	2	0
4100	13554	9836	941	368	200	28060	1284	5	585	1	2	62	3	76	270.36	84	424.83	276	274	282	14	46	0	0	2
13358	7466	4550	0	1468	1156	28218	565	243	738	85	202	199	113	229	281.96	162	450.18	506	262	434	41	88	20	18	5
12124	7872	5082	0	1712	1334	28404	554	144	704	40	262	170	77	247	301.18	145	453.5	468	231	439	76	83	6	18	18
14442	7618	4406	7	772	546	27844	658	356	1059	168	60	262	139	136	268.94	270	432.19	490	458	424	19	81	20	0	1
5650	12536	8960	570	600	362	28136	1074	13	756	3	28	68	7	92	280.29	122	435.99	388	380	345	23	45	0	0	1
4236	13246	9712	846	590	322	28124	1236	5	571	0	18	66	2	90	277.66	83	431.98	307	290	272	18	49	0	0	3
3716	13850	10038	1023	260	166	28044	1319	7	550	2	12	60	4	58	270.81	91	422.4	273	255	267	15	36	0	2	3
13192	7758	4740	2	1292	994	28148	635	227	846	79	156	204	99	211	282.24	180	446.19	454	336	423	40	89	21	14	11
14174	7590	4392	2	950	736	27946	635	298	991	161	96	266	97	175	273.26	245	436.91	486	402	410	18	88	22	8	4
13034	7832	4712	3	1358	1088	28194	609	207	809	74	146	197	99	226	283	179	446.18	467	294	429	43	89	11	24	7
13502	7768	4618	6	1156	902	28086	623	252	891	81	132	186	127	194	281.61	209	444.28	471	332	461	43	85	18	8	0
3808	13706	9976	987	354	206	28062	1311	5	552	2	12	56	3	59	270.3	93	424.56	280	262	274	15	36	0	0	3
4762	13116	9462	766	470	272	28096	1180	9	670	0	10	59	6	73	276.37	119	430.89	340	328	319	20	42	0	4	2
11968	7942	5134	1	1724	1366	28424	542	145	661	39	266	177	74	268	298.38	129	455.44	475	221	423	75	89	10	20	10

SUPPLEMENTARY INFORMATION:Monte Carlo Atomistic Simulation and Machine Learning Analysis of Na-K Eutectic Alloy in Condensed Phases, D. Reitz and E. Blaisten-Barojas, George Mason University, Fairfax, VA 22030

4486	13160	9568	788	566	328	28128	1195	6	616	1	20	57	3	87	276.96	92	433.35	321	310	313	20	37	0	0	3
13026	7770	4858	4	1396	976	28158	588	222	858	81	122	190	92	210	282.18	188	449.28	480	315	460	49	88	17	10	3
4074	13592	9854	955	342	188	28052	1320	9	581	2	2	60	5	71	270.82	90	424.02	266	295	258	15	41	0	0	1
13726	7594	4522	3	1220	890	28058	584	261	848	107	98	218	118	187	277.77	209	445.53	497	291	464	36	91	15	8	3
4096	13526	9832	921	378	218	28068	1279	6	579	1	18	57	3	82	271.18	95	425.82	270	299	290	15	34	0	0	2
11476	7936	5360	2	1990	1480	28552	514	96	611	16	288	175	49	282	310.35	132	461.42	479	176	393	103	115	7	18	12
13348	7780	4666	6	1212	954	28118	596	232	891	80	144	218	107	222	284.21	197	447.41	465	337	432	41	106	22	14	5
14456	7682	4366	8	748	554	27854	627	331	1081	163	42	257	132	150	268.75	275	432.5	486	430	457	18	80	16	6	0
4670	13252	9554	802	370	204	28060	1184	8	671	1	10	58	5	72	275.71	109	428.79	329	323	332	16	46	0	0	2
11512	8026	5354	0	1924	1436	28536	547	111	602	23	252	167	68	254	311.81	126	457.33	489	205	403	101	83	6	28	11
4838	13030	9424	730	510	290	28114	1162	7	647	1	20	63	4	83	278.57	97	433.16	359	331	310	11	38	0	2	4
4822	12994	9424	732	558	304	28114	1163	8	657	0	12	56	6	96	278.9	108	434.94	328	332	328	19	38	0	0	1
10598	7954	5750	1	2262	1738	28792	513	59	498	10	426	162	37	278	331.12	90	469.97	467	140	365	127	81	1	58	35
6520	11756	8408	448	912	562	28212	983	27	726	7	52	68	16	102	287.3	118	442.31	435	373	368	30	38	0	2	3
4200	13492	9764	877	384	224	28072	1244	6	605	1	8	58	3	65	273.34	92	426.57	318	301	294	15	36	0	0	1
4810	13026	9428	716	538	300	28116	1190	2	667	0	14	61	2	80	278.73	92	434.15	337	358	305	18	37	0	0	3
12076	7788	5146	0	1800	1340	28428	552	131	663	33	246	170	70	239	301.94	143	454.96	489	234	409	84	85	6	32	18
4310	13378	9710	867	430	240	28078	1255	12	601	1	10	60	8	77	274.42	86	428.57	297	311	288	15	44	0	0	2
4016	13584	9870	939	374	214	28070	1281	4	567	1	12	67	2	69	272.78	74	426.52	293	277	273	15	42	0	0	1
5232	12750	9218	624	588	316	28118	1088	10	715	0	14	69	8	88	280.88	117	437.18	380	370	345	18	45	0	0	4
5008	12906	9344	684	556	292	28114	1154	6	722	0	8	73	4	76	276.59	124	433.44	376	375	299	15	55	0	0	1
14346	7748	4394	5	732	578	27862	628	330	1084	162	56	261	133	151	267.73	291	430.25	502	443	435	14	90	13	8	0
5080	12936	9290	664	484	294	28112	1116	4	698	0	26	54	3	76	277.72	114	433.61	374	360	358	15	41	0	2	4
12090	7752	5120	2	1836	1366	28438	542	151	662	44	238	178	73	244	298.69	138	455.52	486	210	416	83	89	10	28	20
13652	7820	4588	6	1046	804	28004	636	274	963	134	90	246	113	172	275.51	222	441.03	464	395	427	42	82	20	2	4
13594	7876	4644	1	1034	780	28020	642	241	990	107	88	231	98	158	276.84	235	444.11	506	381	410	37	109	18	4	1
4844	13048	9432	743	480	272	28098	1180	5	674	1	22	72	4	81	277.61	102	434.25	321	351	319	23	49	0	0	3
5560	12566	9008	572	606	368	28144	1060	5	769	0	34	77	4	101	280.39	142	438.22	395	367	338	21	59	0	2	0
11324	7884	5278	0	2042	1696	28654	539	93	575	20	368	169	49	275	317.74	112	465.05	469	193	365	112	93	2	58	23
13960	7756	4496	3	952	712	27944	628	289	1007	138	68	242	113	175	273.87	233	437.89	470	393	449	24	90	13	0	3
3960	13700	9926	961	288	164	28046	1297	7	586	1	8	58	5	66	271.28	86	424.49	276	288	283	15	39	0	0	1
13056	7818	4768	0	1350	1040	28208	585	176	868	56	166	198	90	193	284.84	222	448.39	530	278	430	41	110	16	10	8
14206	7632	4410	6	914	686	27932	616	317	1021	134	82	247	132	173	275.11	247	439.78	485	405	440	23	102	19	2	2
4112	13640	9878	955	254	134	28026	1285	12	614	2	8	63	7	70	270.69	101	422.82	285	288	282	8	43	0	0	1
6420	11926	8476	439	810	512	28192	988	23	757	1	46	77	14	119	285.77	119	443.52	419	391	359	26	50	1	2	1
14208	7640	4354	7	924	724	27930	617	323	979	147	76	263	140	186	273.15	218	437.74	460	379	441	25	100	22	4	2

SUPPLEMENTARY INFORMATION:Monte Carlo Atomistic Simulation and Machine Learning Analysis of Na-K Eutectic Alloy in Condensed Phases, D. Reitz and E. Blaisten-Barojas, George Mason University, Fairfax, VA 22030

5424	12650	9050	640	602	378	28132	1130	15	699	2	28	75	9	98	279.84	118	436.74	358	362	309	19	51	0	0	2
11624	7974	5268	2	1886	1466	28532	561	112	632	21	274	176	59	256	308.63	124	457.05	482	214	386	93	88	16	36	16
14070	7700	4482	3	910	688	27942	620	290	1025	129	88	241	122	167	276.32	243	441.56	485	404	444	30	101	16	4	0
4300	13416	9724	870	400	224	28074	1245	6	597	1	10	56	2	67	273.93	93	428.23	315	310	295	14	30	0	0	2
4812	13144	9442	773	394	254	28078	1182	9	665	1	32	69	7	75	275.93	107	430.29	336	340	314	17	42	0	0	2
4262	13478	9758	881	362	196	28058	1231	11	600	2	2	67	4	63	272.38	105	426.37	326	285	290	13	40	0	0	3
14456	7758	4392	5	664	508	27826	670	327	1133	162	44	257	132	142	267.77	279	430.62	464	484	445	16	87	15	4	1
5528	12712	9018	590	492	328	28106	1088	9	780	3	28	75	4	87	280.65	112	438.03	359	405	354	29	50	0	0	2
5360	12786	9174	625	492	270	28094	1098	13	726	1	10	68	9	97	281.04	106	437.84	366	367	349	13	47	0	2	1
6918	11678	8262	376	796	474	28162	938	33	799	12	34	91	18	124	287.09	151	444.23	438	401	382	19	52	1	0	1
12382	7880	5138	3	1584	1106	28276	597	151	797	42	166	172	78	233	286.77	163	450.62	489	291	429	51	93	8	20	3
4736	13052	9470	748	534	302	28118	1175	4	664	0	24	62	2	91	278.37	96	433.11	323	328	318	20	42	0	0	4
6466	11922	8526	475	756	442	28156	1004	35	742	11	44	100	20	120	287.27	114	443.09	394	380	343	27	59	0	0	3
4304	13370	9670	845	456	284	28104	1233	5	609	0	20	43	2	67	273.96	108	428.01	339	302	302	9	30	0	0	2
13682	7930	4624	7	942	718	27964	633	251	1022	115	62	229	100	167	274.73	249	439.26	487	384	451	24	97	19	6	1
4364	13298	9678	834	480	260	28090	1213	6	595	1	10	66	4	82	273.75	94	428.97	320	307	299	15	40	0	0	1
13468	7940	4738	6	1020	754	28010	684	254	987	121	84	221	99	173	276.46	201	442.88	456	438	415	32	84	15	6	4
12956	7854	4900	3	1358	962	28166	623	192	868	75	124	205	91	210	281.5	194	446.99	476	340	426	47	108	12	12	1
13560	7580	4510	3	1304	1010	28112	605	231	847	89	138	209	108	187	278.96	199	446.54	499	328	443	43	98	14	10	2
5434	12654	9104	619	574	326	28114	1091	16	713	1	20	80	11	105	280.68	111	437.95	356	361	342	19	50	0	2	1
12054	7980	5134	1	1692	1284	28378	553	159	709	41	220	167	92	242	294.54	141	450.92	490	238	446	70	85	8	14	9
4444	13264	9620	811	468	278	28098	1205	6	603	1	22	57	2	84	274.97	87	430.73	324	305	309	13	36	0	2	2
4080	13522	9832	925	388	228	28070	1289	10	580	1	20	58	6	68	271.3	78	425.6	279	300	281	20	38	0	0	1
4268	13480	9746	871	348	208	28068	1246	8	634	0	18	49	6	76	274.04	89	428.14	291	315	319	13	33	0	0	3
5144	12822	9222	659	572	344	28128	1089	8	657	1	24	61	4	95	280.29	104	437.13	368	316	351	23	34	0	0	2
4498	13294	9628	811	418	228	28076	1225	6	638	0	10	61	5	57	274.39	102	430.22	339	324	294	15	50	0	0	3
14356	7610	4352	3	862	648	27896	637	324	1032	159	62	269	125	160	270.7	255	434.18	490	423	418	13	92	15	6	6
12888	7970	4888	3	1296	980	28170	580	165	889	48	138	180	90	194	282.18	184	447.83	500	308	481	44	101	13	8	8
13318	7856	4700	7	1196	910	28098	619	215	921	79	102	197	100	186	280.78	215	445.88	480	350	454	43	92	12	16	4
14236	7650	4430	8	856	644	27900	637	329	1009	149	80	254	132	152	271.29	233	434.68	485	414	439	25	91	26	4	1
12300	8036	5112	3	1496	1140	28300	585	162	764	47	200	199	77	243	291.14	152	454.39	475	286	406	55	94	8	16	13
13456	7774	4618	2	1184	926	28090	626	250	916	104	112	212	111	200	277.25	209	445.57	480	354	433	30	89	14	18	5
4368	13280	9640	844	504	296	28104	1234	5	590	1	16	67	4	73	274.71	96	428.47	318	301	282	20	39	0	0	2
4566	13322	9574	821	370	228	28074	1226	9	648	1	14	62	5	69	275.51	98	429.42	315	332	303	18	43	0	0	1
4786	13024	9422	706	552	318	28118	1150	9	653	0	16	48	7	88	276.09	97	432.16	345	343	341	15	35	0	0	4
5772	12358	8890	590	674	404	28142	1073	22	724	4	42	68	10	111	284.94	117	441.2	360	361	345	25	47	1	2	2

SUPPLEMENTARY INFORMATION:Monte Carlo Atomistic Simulation and Machine Learning Analysis of Na-K Eutectic Alloy in Condensed Phases, D. Reitz and E. Blaisten-Barojas, George Mason University, Fairfax, VA 22030

13942	7812	4542	7	906	664	27922	664	293	1030	143	56	246	113	171	274.09	241	438.73	459	431	423	21	89	14	0	2
4282	13374	9726	841	446	246	28086	1220	4	604	0	10	59	4	70	275.28	84	429.33	312	312	312	17	36	0	2	5
4978	12864	9344	675	584	328	28130	1119	3	667	0	30	57	2	103	278.87	92	435.52	348	345	349	15	40	0	2	3
12388	7850	4956	1	1666	1272	28352	599	148	709	32	210	182	79	219	297.85	150	453.61	489	255	393	74	104	15	10	12
4472	13344	9652	857	384	206	28062	1241	5	657	2	2	67	2	85	273.68	93	428.83	297	310	298	6	52	0	2	4
3816	13678	10012	998	358	178	28050	1322	8	536	1	8	65	4	84	271.33	72	423.71	246	262	260	15	45	0	0	1
14160	7712	4422	3	884	672	27918	650	295	1047	150	68	254	115	144	271.05	286	432.57	505	423	416	19	90	14	0	4
11494	8102	5382	0	1830	1402	28506	566	86	674	15	256	174	46	268	309.89	123	456.49	471	240	384	89	99	6	38	16
5526	12630	9034	598	572	338	28118	1088	13	716	1	18	76	10	98	281.63	99	437.05	362	377	346	20	47	0	0	4
10880	7910	5566	1	2232	1712	28732	486	82	533	14	382	156	44	330	325.03	87	466.68	486	170	375	97	70	4	48	29
12000	7998	5182	1	1656	1294	28406	564	125	726	30	242	176	70	219	298.83	148	456.77	513	259	404	71	105	8	30	18
7102	11306	8054	376	1040	662	28240	941	39	732	8	72	85	23	145	288.85	126	444.68	423	383	360	29	53	1	4	3
4398	13322	9668	851	434	244	28082	1246	11	633	2	16	65	6	86	273.85	95	427.32	301	328	284	8	40	0	0	3
12048	7902	5210	0	1688	1272	28400	533	130	709	25	246	172	68	254	306.16	138	457.83	484	247	412	78	90	9	32	19
5226	12722	9184	636	606	362	28136	1114	10	676	4	34	75	6	107	284.16	79	438.2	353	368	331	16	44	0	2	2
6008	12320	8810	524	624	340	28114	1068	20	761	5	12	77	13	92	282.74	110	438.44	390	429	347	20	44	1	0	0
13966	7638	4480	3	1032	770	27984	647	289	964	128	90	245	124	184	276.03	202	440.36	446	393	428	35	102	17	8	4
12088	7846	5188	2	1746	1272	28400	574	135	718	41	246	184	66	242	298.88	154	452.43	516	245	390	53	102	10	14	14
14158	7726	4424	10	884	664	27912	638	295	1035	152	54	259	109	165	269.28	270	433.84	487	403	422	18	91	21	2	2
4242	13426	9750	870	424	230	28076	1237	9	591	1	4	55	4	77	271.05	87	425.29	295	294	311	17	37	0	0	3
4270	13236	9692	829	594	324	28132	1245	5	557	0	16	69	4	85	277.86	86	432.52	321	289	256	18	45	0	0	0
11306	7650	5408	0	2188	1682	28696	501	89	508	19	406	176	42	270	320.94	98	465.62	490	162	373	120	86	6	56	34
14240	7654	4370	4	908	684	27916	633	316	1003	166	56	264	119	143	270.76	274	433.38	519	407	418	21	76	19	4	0
4720	13056	9482	766	536	300	28118	1163	6	643	1	24	58	4	98	277.58	107	433.18	334	301	327	14	33	0	0	1
13254	7788	4666	3	1276	1010	28156	603	221	810	63	146	183	120	203	282.74	174	447.09	481	311	468	39	88	16	16	8
12878	8034	4900	2	1242	950	28142	615	187	919	66	128	185	82	181	282.22	198	447.19	484	339	455	58	95	16	10	2
5016	12908	9314	693	550	314	28120	1129	6	662	0	18	67	2	89	279.36	102	435.45	363	336	327	16	45	0	0	3
4458	13344	9634	817	404	232	28080	1199	7	651	1	6	57	5	82	274.43	99	427.81	320	312	326	14	40	0	2	0
14264	7660	4392	2	882	650	27906	632	315	1043	160	58	265	117	157	269	264	431.92	513	420	412	14	100	19	0	0
14274	7610	4342	3	920	706	27928	652	319	997	149	72	238	129	175	271.46	237	435.52	456	408	448	22	75	19	4	1
4588	13120	9534	783	550	310	28120	1167	6	595	0	18	55	5	87	278.52	87	434.52	332	297	327	21	35	0	0	3
5200	12828	9178	653	540	348	28124	1140	14	710	0	30	56	8	95	277.67	94	434.42	348	382	334	12	37	0	0	6
3882	13760	9972	975	254	154	28038	1288	5	595	1	14	61	3	56	271.33	104	424.15	283	270	291	15	35	0	2	3
12880	7848	4834	3	1394	1082	28226	580	177	828	45	166	186	101	223	288.02	168	449.4	471	305	454	61	106	12	20	4
11458	7896	5298	2	2010	1564	28574	549	112	581	31	310	165	54	290	308.84	109	458.29	464	205	379	98	77	2	36	15
4060	13626	9852	940	310	190	28050	1284	11	575	2	12	53	6	75	272.21	82	425.72	272	290	289	15	35	0	0	2

SUPPLEMENTARY INFORMATION:Monte Carlo Atomistic Simulation and Machine Learning Analysis of Na-K Eutectic Alloy in Condensed Phases, D. Reitz and E. Blaisten-Barojas, George Mason University, Fairfax, VA 22030

11330	7946	5436	0	1998	1520	28590	556	94	589	21	320	141	47	257	310.93	123	458.43	515	198	383	82	74	5	36	24
12718	7876	4896	2	1432	1114	28234	632	187	835	68	178	174	89	215	282.8	171	448.37	480	341	429	39	81	10	16	10
5930	12146	8762	511	784	496	28192	1006	19	675	5	64	75	12	123	286.27	97	441.55	384	346	368	31	44	1	10	4
12028	7818	5166	1	1748	1352	28430	516	115	687	28	286	199	70	266	303.61	141	455.78	496	212	407	68	113	5	32	18
4692	13138	9454	737	494	314	28116	1176	5	643	0	24	61	5	88	277.68	92	434.24	330	337	319	18	39	0	0	3
11634	7742	5316	0	1994	1498	28548	518	128	584	36	328	178	61	268	301.86	118	456.83	500	197	394	86	100	13	34	19
7344	11190	7896	349	1022	702	28258	958	36	780	5	92	80	23	141	290.44	129	447.32	429	398	345	30	54	1	12	2
4404	13382	9690	813	374	206	28070	1197	3	657	0	14	58	3	71	277.37	95	432.82	314	328	333	24	37	0	0	0
4552	13184	9554	762	516	296	28118	1171	4	626	0	16	58	2	82	276.77	94	432.3	343	313	322	19	40	0	0	1
6812	11602	8226	354	934	600	28238	928	26	774	4	62	80	20	122	289.04	128	445.1	466	405	369	25	47	0	2	2
7220	11262	7996	371	1012	660	28238	913	26	745	4	84	95	19	138	286.94	122	446.27	432	336	374	28	67	0	4	10
10988	7946	5492	0	2202	1670	28666	542	83	554	5	328	138	47	289	315.91	103	463.53	462	180	392	104	76	2	36	25
13640	7804	4574	6	1062	846	28050	626	244	961	97	108	219	115	196	280.52	212	444.7	453	380	453	39	99	12	16	3
3972	13604	9894	937	370	212	28064	1270	5	563	1	10	64	4	67	271.11	78	424.39	291	273	286	14	44	0	2	5
10170	7840	5864	0	2558	1930	28908	495	40	464	4	512	153	18	324	332.3	70	472.45	433	146	371	139	74	4	30	40
13804	7598	4402	2	1186	952	28078	581	262	873	101	130	222	114	182	277.82	220	445.19	512	308	448	41	100	24	6	2
14222	7734	4396	5	854	646	27900	650	313	1020	160	46	249	118	131	270.59	272	433.83	523	422	426	17	73	15	2	0
13968	7730	4512	5	974	700	27942	644	291	1008	150	56	259	107	176	274.24	239	438.67	455	415	423	32	97	14	2	2
12412	7714	5018	2	1724	1250	28344	578	170	725	46	206	173	90	242	292.46	135	452.04	495	272	421	47	87	11	18	12
7462	11082	7850	317	1082	692	28246	914	43	751	10	78	96	20	150	289.9	123	445.82	433	378	359	30	52	3	0	3
11350	7996	5386	1	1986	1526	28578	561	96	606	25	292	180	54	280	309.97	118	460.67	502	215	343	82	102	5	38	16
4062	13594	9872	934	328	182	28050	1275	8	588	1	12	54	6	52	270.34	92	422.49	299	286	293	18	34	0	0	3
5066	12698	9210	673	728	438	28174	1100	15	618	2	32	69	11	98	281.99	102	439.01	394	306	312	16	44	0	0	3
4776	13104	9470	744	480	260	28098	1158	9	679	0	8	58	5	76	276.93	116	433.49	363	325	324	12	45	0	0	2
4012	13692	9932	955	244	132	28026	1276	10	606	1	14	70	8	70	271.41	99	425.38	281	287	284	12	44	0	0	2
7656	10796	7676	255	1270	824	28322	820	40	724	7	92	80	23	165	288.31	129	444.65	488	336	398	29	52	2	6	4
14326	7670	4364	5	828	636	27890	642	319	1020	154	64	265	128	157	273.56	261	437.18	484	409	425	19	93	19	2	2
3984	13704	9928	985	266	146	28034	1311	7	598	1	6	63	4	64	271.26	92	423.12	274	281	271	11	45	0	0	2
13652	7912	4630	6	966	746	27996	631	255	974	127	86	229	101	189	277.85	210	442.32	468	374	438	28	85	13	4	2
11998	7834	5184	0	1784	1346	28444	580	110	698	26	276	165	53	277	296.54	113	456.73	463	229	399	64	97	9	20	14
4506	13184	9578	786	524	302	28116	1189	3	610	0	22	61	2	100	277.63	89	431.99	319	313	313	12	40	0	0	2
13754	7726	4562	1	1080	796	28010	636	255	970	121	90	239	106	175	276.18	229	441.31	491	396	417	27	96	14	2	4
4586	13190	9548	785	466	282	28102	1228	6	638	0	28	55	3	78	275.83	95	430.5	315	342	296	18	41	0	2	2
7632	11038	7750	281	1034	704	28258	882	40	807	12	92	89	25	155	288.62	138	444.04	442	414	393	28	47	0	6	4
3788	13814	10010	1018	260	158	28040	1313	7	556	1	8	64	4	59	270.22	94	422.2	273	255	267	17	42	0	2	2
13968	7774	4520	3	914	690	27944	658	267	1065	133	70	247	105	174	274.43	250	438.43	460	440	425	26	103	11	8	1

SUPPLEMENTARY INFORMATION:Monte Carlo Atomistic Simulation and Machine Learning Analysis of Na-K Eutectic Alloy in Condensed Phases, D. Reitz and E. Blaisten-Barojas, George Mason University, Fairfax, VA 22030

11920	7886	5140	1	1810	1406	28452	556	123	692	29	264	179	61	239	299.39	144	452.8	529	224	381	74	102	10	24	13
5448	12424	8994	540	792	490	28202	1048	11	678	1	48	68	6	117	283.6	113	439.33	402	351	332	20	42	0	6	2
4022	13634	9892	940	316	176	28048	1263	2	599	1	6	61	1	68	271.06	96	424.03	297	273	295	11	43	0	2	2
12118	7898	5190	0	1694	1230	28370	592	128	754	31	228	177	73	217	297.63	169	451.59	504	276	402	69	97	9	10	13
11882	7894	5116	1	1814	1454	28470	545	132	660	35	268	178	78	265	294.2	145	452.59	503	230	395	76	76	8	42	11
11412	8010	5384	0	1950	1474	28542	524	99	639	19	284	172	50	294	312.95	106	458.59	463	207	392	95	102	5	28	18
4512	13164	9570	789	558	306	28116	1205	5	620	0	6	55	2	91	275.73	88	431.65	318	321	311	15	40	0	0	1
5278	12676	9118	608	642	406	28162	1072	8	667	1	36	61	5	105	279.46	97	437.58	386	334	349	16	37	0	6	2
12226	7990	5058	1	1610	1240	28342	548	142	770	27	202	156	85	229	295.35	178	451.04	490	255	460	76	93	11	16	9
12420	7806	4968	3	1670	1246	28316	602	147	724	46	196	188	71	225	292.44	148	447.89	488	258	404	65	103	12	8	7
3974	13670	9928	940	306	164	28050	1276	8	579	1	8	59	5	76	271.17	93	423.79	289	284	283	9	36	0	0	1
4100	13506	9818	906	402	234	28076	1262	9	579	2	12	65	5	79	271.27	86	424	293	291	283	13	46	0	4	2
11662	7910	5278	2	1850	1458	28520	557	113	623	31	344	167	50	276	306.18	122	457.46	492	224	382	81	81	7	18	10
4472	13178	9580	782	554	322	28128	1187	7	593	0	22	58	7	87	277.47	63	431.55	320	317	320	21	36	0	0	4
6650	11706	8334	358	910	572	28230	981	17	791	3	52	76	10	116	286.85	126	444.09	436	422	358	25	50	1	4	3
7250	11322	8062	388	914	564	28188	949	43	748	10	76	99	24	137	288.62	123	444.93	400	372	360	36	61	1	0	3
4194	13496	9768	902	378	226	28076	1250	6	594	1	12	68	3	69	275.71	97	429.74	318	291	280	10	38	0	2	3
7184	11342	8046	337	942	624	28236	918	28	794	10	88	90	12	141	289.47	131	446.39	425	397	381	32	52	1	10	5
5854	12322	8808	543	710	434	28158	1056	23	700	7	28	76	12	94	282.4	115	439.34	398	360	346	21	44	1	2	5
4302	13456	9746	896	344	194	28058	1262	11	630	1	16	58	5	78	274.15	98	428.57	288	301	293	12	40	0	0	2
11270	7820	5334	1	2146	1684	28658	521	88	549	17	368	172	46	293	317.26	95	465.06	456	178	385	107	77	4	36	24
5076	12834	9278	662	588	332	28128	1128	3	692	0	20	71	3	83	279.39	107	435.05	382	350	317	16	50	0	0	0
4330	13354	9708	849	446	242	28092	1243	3	597	1	10	55	1	73	278.04	86	432.62	310	315	297	17	31	0	2	1
4226	13542	9782	900	316	178	28048	1277	9	613	3	4	59	3	62	271.72	98	425.59	300	319	281	12	38	0	0	2
4738	13206	9492	781	390	234	28076	1202	11	672	0	14	65	5	87	274.95	108	430.96	317	351	306	15	46	0	2	0
12628	7826	4966	3	1504	1122	28252	603	178	832	55	196	177	91	208	286.19	176	451.3	479	307	448	55	95	16	10	11
4230	13524	9770	902	326	192	28052	1267	8	616	0	10	60	5	64	274.35	96	428.32	288	306	293	14	41	0	0	7
4226	13502	9762	899	362	208	28066	1261	3	602	1	6	56	2	62	271.58	86	426.77	313	294	293	12	42	0	0	1
3898	13584	9914	954	428	246	28086	1282	4	544	1	14	61	2	65	271.62	89	423.5	292	258	275	18	42	0	0	4
13914	7576	4502	1	1130	800	28010	579	281	925	123	76	240	113	197	277.65	219	444.51	481	326	445	36	101	17	12	3
4280	13420	9732	877	392	228	28070	1279	13	606	2	18	55	7	77	274.14	70	427.64	276	328	287	18	34	0	0	1
4650	13138	9504	803	496	296	28108	1214	9	628	1	22	56	5	80	276.1	92	430.59	330	326	291	16	41	0	2	2
3902	13686	9954	991	326	176	28050	1317	8	581	1	6	60	4	64	271.08	102	423.37	284	271	258	10	38	0	0	1
4774	13092	9482	743	474	256	28094	1182	9	666	0	16	62	6	87	276.9	93	433.43	324	346	324	12	41	0	0	5
11428	7764	5342	1	2152	1580	28594	532	115	576	23	294	173	56	280	313.02	121	460.65	485	183	367	95	84	14	26	22
4600	13240	9548	825	422	256	28086	1244	9	654	3	18	50	3	69	273.23	103	428.85	309	336	303	16	32	0	2	3

7584	11050	7778	307	1070	696	28256	876	48	768	12	76	96	25	155	288.83	140	444.43	455	355	374	26	59	2	2	5
14208	7700	4402	4	872	662	27904	641	305	1036	159	56	256	116	150	270.33	261	433.15	503	445	421	18	81	13	2	2
11854	7808	5188	2	1906	1430	28480	500	129	640	31	268	184	64	274	304.02	119	457.26	479	211	410	94	99	4	26	17
4852	13026	9408	757	506	294	28108	1190	4	662	0	22	55	3	82	275.76	94	431.78	328	339	326	16	43	0	0	2
4906	12882	9314	711	638	386	28152	1127	10	610	2	24	59	6	98	280.31	101	436.37	370	300	323	16	38	0	2	0
12698	7984	4924	0	1370	1064	28216	586	183	801	57	160	197	90	215	284.25	176	448.41	496	282	437	50	95	19	16	4
14322	7630	4398	8	858	620	27888	661	328	1050	162	60	263	130	161	270.26	261	433.29	472	437	420	17	92	20	0	1
14292	7618	4406	3	860	640	27898	616	319	1031	165	82	265	121	177	274.93	246	437.3	461	419	449	24	87	9	0	2
11106	7848	5482	0	2180	1652	28678	509	81	540	18	384	173	42	294	321.67	93	465.54	479	172	358	108	83	4	26	23
4736	13174	9502	773	416	236	28080	1181	6	674	1	16	65	2	84	276.94	98	432.33	320	335	326	14	46	0	0	4
5480	12610	9010	574	634	394	28152	1054	3	708	0	24	66	2	117	279.97	98	438.3	365	366	372	19	42	0	0	3
13476	7784	4696	5	1140	840	28056	625	258	931	121	112	225	108	188	278.66	220	443.66	471	366	433	42	88	19	8	2
5322	12628	9100	605	680	402	28156	1107	10	684	0	24	68	7	104	281.86	103	437.4	371	359	324	18	49	0	0	1
14318	7658	4358	3	854	648	27896	648	317	1036	157	56	261	121	162	273.22	237	436.2	473	424	427	18	94	20	4	4
11572	7962	5332	1	1908	1442	28526	534	98	652	26	280	188	52	284	310.2	123	460.44	491	229	382	78	101	5	26	15
11636	7750	5326	0	2010	1486	28552	523	124	600	28	304	172	62	281	309.51	119	460.8	482	184	399	84	92	7	34	22
3958	13674	9932	978	304	166	28042	1297	11	564	1	8	58	8	60	270.38	76	423.34	279	268	280	16	43	0	0	3
4624	13142	9528	784	508	284	28098	1224	12	647	0	12	64	7	81	275.6	91	432.08	315	345	291	18	43	0	0	1
14304	7662	4374	6	826	648	27894	632	329	1040	165	70	254	122	158	269.84	268	430.77	486	413	439	22	81	19	10	0
3936	13662	9924	961	340	188	28054	1305	9	573	1	4	66	6	67	271.44	81	424.35	277	286	263	15	46	0	0	2
4616	13174	9538	789	478	274	28098	1198	4	643	0	18	52	2	78	275.08	95	430.87	336	331	311	15	41	0	0	1
12058	8010	5136	1	1678	1274	28384	618	129	727	30	210	165	77	240	292.36	137	450.98	471	274	412	55	92	7	18	15
4594	13272	9578	796	400	222	28074	1193	6	656	0	8	63	5	73	275.88	100	431.77	338	336	309	14	41	0	0	2
4322	13388	9720	886	408	222	28070	1261	8	589	1	10	68	6	72	272.5	82	426.15	297	303	280	15	50	0	0	3
5090	12782	9240	634	640	374	28150	1114	6	679	1	20	58	2	101	279.58	99	435.44	368	366	334	13	43	0	4	4
4492	13216	9586	774	500	296	28116	1185	5	600	0	26	59	2	94	278.59	92	433.2	331	307	312	13	43	0	0	1
4278	13346	9710	848	466	272	28094	1239	10	592	1	18	58	7	79	272.74	87	428.99	305	306	297	16	38	0	4	2
4466	13378	9646	859	360	206	28064	1260	10	627	1	8	55	5	83	275.3	74	428.92	286	338	296	13	39	0	0	0
14340	7614	4332	4	876	674	27908	655	310	1037	159	66	256	116	153	271.67	256	435.13	487	419	421	22	87	16	6	0
5568	12548	8962	613	632	404	28150	1106	26	696	7	36	65	14	98	283	114	438.64	379	377	329	16	38	1	0	1
4830	13102	9460	753	428	238	28076	1162	15	663	0	18	66	10	88	277.66	100	434.87	334	327	326	16	47	0	0	0
5130	12894	9250	670	512	306	28110	1116	9	678	1	18	61	7	88	279.38	107	436.68	355	347	356	18	34	0	0	3
6620	11718	8342	363	948	574	28240	971	14	808	1	36	68	13	129	287.07	123	444.14	431	439	372	22	46	0	2	2
4948	12938	9344	691	548	322	28126	1137	7	655	1	24	66	4	98	282.14	101	439.74	348	347	324	20	33	0	2	0
13302	7778	4652	3	1290	980	28128	612	219	879	92	122	216	96	220	281.47	200	445.65	483	334	416	31	105	8	4	4
4290	13370	9724	839	458	240	28082	1213	7	590	1	0	59	5	74	273.95	87	428.83	321	298	313	15	38	0	0	2

SUPPLEMENTARY INFORMATION:Monte Carlo Atomistic Simulation and Machine Learning Analysis of Na-K Eutectic Alloy in Condensed Phases, D. Reitz and E. Blaisten-Barojas, George Mason University, Fairfax, VA 22030

14154	7782	4550	5	798	550	27886	629	330	1052	150	50	239	136	157	271.06	265	434.29	494	430	457	13	77	21	2	1
11616	7962	5402	2	1864	1348	28478	590	95	676	22	262	191	48	230	305.67	132	455.76	489	257	363	92	106	9	22	16
14126	7732	4502	2	864	622	27910	638	305	1046	139	60	242	122	156	273	254	437.1	483	409	452	23	93	22	4	0
3940	13640	9902	970	358	214	28068	1294	4	562	0	14	51	3	78	270.94	81	422.7	275	275	284	7	36	0	0	5
11054	7938	5608	1	2144	1534	28626	543	86	577	13	320	162	56	314	321	90	462.42	414	196	403	106	94	2	24	21
4456	13276	9628	829	458	258	28090	1219	13	620	0	14	56	8	63	276.39	93	431.76	330	303	302	23	47	0	0	0
5414	12586	9076	591	644	388	28150	1086	12	682	2	40	58	5	121	283.52	83	439.72	356	370	351	16	37	1	2	1
5258	12658	9170	622	670	372	28150	1091	6	688	1	22	74	4	83	281.29	110	437.79	393	360	329	24	52	0	0	2
4650	13054	9496	747	566	332	28132	1184	7	624	0	32	62	2	94	277.18	84	433.21	330	339	303	17	45	0	0	3
3906	13688	9940	975	312	190	28058	1301	7	567	1	22	63	4	73	273.59	87	425.9	265	263	278	15	46	0	0	3
6624	11706	8352	394	906	570	28222	962	20	773	4	60	68	11	115	286.17	130	444.72	440	396	381	25	49	1	4	3
4534	13284	9612	826	406	224	28072	1237	11	639	0	12	43	4	71	276.6	106	430.45	316	327	305	13	26	0	0	2
4420	13258	9626	818	496	288	28108	1231	4	601	0	20	60	2	78	275.74	87	429.91	317	310	287	17	32	0	0	3
4428	13252	9606	810	492	304	28112	1217	5	621	0	30	60	3	79	276.44	94	429.92	330	318	294	14	38	0	0	2
12266	7984	5076	4	1596	1196	28316	580	158	726	38	184	165	84	225	294.06	159	453.1	488	259	437	63	87	12	14	11
10728	7662	5652	0	2428	1852	28864	468	74	463	12	468	155	33	338	335.57	84	474.89	490	130	341	124	81	5	72	22
13238	7640	4604	5	1422	1116	28200	611	238	762	73	168	180	111	220	281.36	148	449.89	461	296	457	44	82	23	12	6
5590	12606	9024	609	556	320	28116	1086	15	746	2	18	74	9	86	279.67	124	438.48	400	376	328	14	48	1	2	2
11870	7914	5130	0	1864	1434	28480	535	128	633	29	236	182	75	258	302.03	144	455.64	513	188	398	72	95	6	32	17
14260	7702	4394	9	824	640	27890	618	308	1068	154	66	261	117	147	269.37	296	431.18	519	408	433	14	92	15	4	2
10834	7936	5548	1	2250	1750	28756	528	76	485	15	386	153	41	299	324.12	105	465.07	498	168	361	93	62	4	50	26
13568	7650	4566	4	1218	940	28084	577	259	867	96	124	197	121	192	283.23	185	446.88	479	312	489	47	75	12	18	2
12142	7726	5120	0	1848	1340	28432	557	133	679	46	218	199	62	264	302.3	141	458.4	473	222	395	77	107	8	34	13
14058	7754	4524	7	880	638	27922	631	286	1052	145	64	258	116	175	273.62	243	438.35	479	423	439	17	90	17	4	0
13074	7730	4768	0	1420	1064	28236	559	195	801	54	160	189	94	242	294.71	171	453.46	483	274	440	50	109	13	18	5
4012	13598	9898	953	350	186	28052	1277	7	571	1	8	69	4	82	272.64	90	425.46	274	269	277	13	44	0	0	2
5022	12824	9292	668	614	358	28142	1129	5	674	0	30	71	3	85	280.54	103	437.86	367	350	316	23	45	0	2	3
4510	13290	9604	825	422	244	28084	1194	3	634	1	14	65	1	75	276.07	113	431.49	333	288	310	15	39	0	0	3
5490	12514	9042	578	682	394	28158	1067	16	718	4	34	69	8	92	279.58	122	438.44	401	368	339	22	45	0	2	2
5738	12308	8880	561	750	448	28168	1055	12	701	0	42	74	3	112	283.74	120	441.27	387	352	326	20	49	1	0	6
13872	7566	4516	2	1138	826	28038	622	279	916	107	116	219	126	185	280.08	203	447.08	483	377	442	33	93	17	4	2
12640	7822	4986	0	1514	1110	28272	586	174	791	46	174	181	88	236	294.8	140	451.29	456	306	444	56	99	16	24	12
4350	13284	9632	807	522	316	28124	1223	7	580	0	20	55	4	75	280.29	77	435.12	319	313	297	20	33	0	0	5
13940	7748	4514	9	958	716	27954	628	293	977	146	76	244	115	150	271.68	254	437.43	514	369	431	24	86	15	2	2
12646	7912	4930	1	1434	1124	28258	567	177	815	43	194	177	99	219	291.41	173	451.19	482	288	459	62	97	12	16	10
11810	7828	5322	1	1862	1346	28474	516	125	643	18	290	162	69	279	306	126	458.33	494	202	411	78	98	12	14	14

6210	12040	8616	505	806	476	28180	1022	25	711	5	32	68	18	114	285.12	96	441.53	394	369	373	21	43	2	0	4
4198	13474	9788	893	386	214	28074	1258	5	605	1	12	67	2	69	274.86	89	430.62	305	295	279	16	45	0	2	2
13722	7760	4628	4	1040	748	27988	631	266	985	117	88	222	112	146	276.29	240	439.99	497	385	453	38	88	17	2	3
14346	7586	4336	5	880	676	27904	661	330	1024	159	74	262	132	169	271.18	247	434.85	460	430	423	19	87	24	6	2
13498	7672	4562	4	1270	978	28114	603	254	836	106	116	202	110	186	276.8	221	445.71	502	302	448	39	73	17	18	5
4174	13488	9782	885	390	228	28078	1272	3	593	0	16	57	2	68	273.09	94	427.3	302	304	283	13	43	0	0	2
13998	7778	4486	4	906	700	27946	615	309	1004	144	70	229	131	169	274.24	242	436.29	478	389	479	24	74	15	8	2
14172	7554	4378	4	994	752	27942	598	332	949	159	86	261	129	174	271.93	242	435.43	498	390	429	28	86	17	6	0
11548	7746	5236	2	2078	1610	28584	551	115	565	31	326	176	58	267	310.43	114	459.64	492	193	364	86	88	10	40	28
4852	12962	9368	732	590	346	28136	1150	8	630	0	18	57	5	93	278.62	93	434.49	340	323	330	22	38	0	0	2
4884	13062	9390	704	474	278	28100	1173	5	690	1	12	67	2	81	276.41	96	433.1	339	378	313	20	41	1	0	1
11698	8018	5258	1	1832	1394	28464	529	121	665	23	230	172	73	268	305.62	130	457.34	482	202	418	86	98	4	32	12
11766	7862	5240	0	1898	1436	28518	541	109	617	23	294	164	63	273	312.45	131	460.55	489	195	402	81	85	5	20	14
14424	7668	4410	4	774	534	27846	666	341	1081	168	36	266	130	154	268.8	268	432.16	469	446	420	15	85	20	0	1
4546	13288	9594	825	402	232	28076	1230	8	654	2	14	63	4	60	273.02	101	428.63	330	322	294	16	44	0	0	3
4088	13534	9836	919	386	214	28066	1292	9	593	1	8	60	5	76	272.87	85	426.26	275	303	278	11	43	0	0	4
13704	7832	4600	4	1024	768	28018	639	259	982	101	86	207	115	177	276.93	221	443.36	470	377	459	33	86	21	4	2
13892	7664	4522	4	1034	766	27976	589	309	937	140	94	233	126	194	276.7	225	442.29	463	332	472	33	78	22	4	2
4458	13350	9642	834	384	224	28072	1210	5	631	1	12	53	3	72	275.58	100	430.05	320	298	323	16	33	0	2	1
12872	7784	4772	2	1424	1170	28258	604	193	815	70	208	186	87	221	286.33	191	451.25	496	304	417	46	87	16	26	9
10640	7854	5678	2	2408	1776	28786	525	65	469	11	378	152	32	286	325.64	98	466.31	515	149	335	105	69	3	48	32
4322	13220	9672	851	570	314	28116	1210	5	544	1	16	68	3	83	275.3	87	430.42	329	265	287	13	43	0	2	5
13078	7684	4756	0	1444	1074	28210	536	219	789	65	158	202	117	235	292.33	163	448.8	472	291	462	63	105	13	12	5
4090	13616	9876	951	286	158	28040	1281	9	605	1	12	74	4	68	270.52	104	422.55	292	282	267	10	50	0	2	3
4328	13358	9704	865	430	242	28078	1243	3	602	1	16	59	2	70	276.01	90	431.32	303	310	298	17	38	0	0	2
4076	13682	9902	945	228	124	28024	1287	7	624	1	12	65	3	72	270.62	87	422.9	267	290	289	13	50	0	0	2
13654	7712	4562	3	1130	864	28038	580	266	911	119	108	239	105	196	276.73	214	443.19	493	330	445	35	100	22	8	1
7670	11080	7754	302	988	662	28236	881	44	793	7	72	84	27	158	289.52	136	446.35	416	406	403	36	46	2	8	5
4054	13522	9850	939	418	224	28072	1302	8	568	1	4	60	4	69	273.42	87	427.57	286	290	260	15	42	0	0	1
13596	7888	4676	4	1024	742	27996	637	277	961	117	62	220	124	173	277.45	215	443.5	468	396	450	37	84	15	6	1
10986	7908	5546	1	2206	1650	28680	541	71	566	15	334	169	30	304	314.94	130	464.32	475	172	335	103	88	4	46	25
4838	12992	9404	730	552	314	28118	1167	11	660	0	18	61	6	84	278.47	97	435.47	351	337	311	16	47	0	0	2
3736	13722	10008	991	386	214	28070	1314	5	515	1	4	59	3	78	269.89	63	421.69	265	264	264	14	34	0	0	1
4170	13452	9796	894	414	228	28076	1242	10	591	1	16	47	4	70	273.92	98	427.31	303	275	315	15	34	0	0	3
13578	7942	4702	11	962	716	27978	639	267	995	133	68	241	100	154	274.6	249	438.39	500	384	428	30	87	15	8	2
4174	13490	9784	898	386	224	28072	1261	6	584	1	14	60	2	74	273.61	87	428.46	294	289	287	16	36	0	0	2

SUPPLEMENTARY INFORMATION:Monte Carlo Atomistic Simulation and Machine Learning Analysis of Na-K Eutectic Alloy in Condensed Phases, D. Reitz and E. Blaisten-Barojas, George Mason University, Fairfax, VA 22030

11202	7882	5510	0	2080	1556	28624	532	96	589	21	374	173	49	273	315.8	106	461.82	471	200	378	105	84	5	18	30
4698	13076	9480	745	542	304	28114	1166	9	615	2	14	65	3	99	277.7	99	434.21	326	320	319	19	42	0	0	0
4060	13674	9900	953	254	134	28026	1287	4	616	1	4	72	3	62	271.05	97	422.93	279	283	275	16	49	0	0	3
4454	13246	9626	801	482	272	28100	1222	5	603	0	20	65	4	85	275.34	84	431.43	313	330	291	14	42	0	0	4
14382	7680	4372	4	794	592	27872	607	324	1059	155	48	265	133	163	273.2	268	438.7	482	412	455	21	95	15	4	1
11416	7862	5338	1	2048	1574	28602	516	106	589	19	330	167	51	259	319.38	137	462.78	510	168	375	110	87	11	30	17
3972	13692	9904	950	290	178	28048	1267	7	584	1	12	60	5	65	273.72	93	427.83	298	273	292	12	41	0	0	2
3928	13656	9942	978	334	182	28054	1308	10	578	1	12	66	6	65	270.5	92	422.64	279	279	260	16	45	0	0	1
12922	7776	4792	3	1442	1110	28228	591	196	807	61	164	185	99	192	286.27	180	451.23	507	310	441	62	98	12	22	4
5162	12882	9260	649	488	286	28104	1106	8	709	3	26	69	2	105	280.21	101	437.01	353	364	339	15	45	0	0	2
4452	13200	9590	816	528	320	28122	1213	10	571	2	32	57	7	86	276.65	80	432.42	308	293	304	19	38	0	0	6
13738	7678	4594	1	1118	798	28024	616	250	961	98	94	216	121	180	277.37	221	445.4	479	342	460	34	101	18	2	3
4420	13366	9656	855	382	230	28074	1253	8	635	0	20	51	5	65	274.48	106	428.03	318	318	293	10	37	0	0	3
5680	12486	8958	571	624	356	28124	1058	7	762	1	20	75	5	105	281.62	133	438.35	391	372	345	15	51	0	0	1
6396	11838	8510	432	892	522	28202	982	18	752	3	42	84	13	109	284.08	113	441.95	436	396	356	25	55	0	2	3
11940	7776	5092	1	1930	1464	28484	540	129	621	22	260	167	69	289	304.66	116	456.72	470	205	398	75	97	6	22	16
11272	8040	5534	1	2010	1410	28536	536	91	643	21	250	167	44	282	311.11	123	458.68	475	209	397	86	92	7	20	19
3926	13570	9902	939	432	242	28084	1272	8	522	1	12	52	5	71	272.14	78	426.36	291	255	287	17	34	0	0	3
11438	7760	5194	1	2140	1708	28626	547	104	569	26	350	176	45	301	307.91	120	459.56	458	185	371	95	87	11	34	21
14476	7580	4308	6	822	622	27874	625	350	1066	170	62	269	140	165	267.96	285	429.5	495	415	429	9	89	21	4	2
5092	12850	9236	665	572	358	28140	1135	6	677	1	30	73	2	106	280.36	98	435.04	344	353	320	17	48	1	2	2
13886	7734	4510	5	1008	758	27978	627	291	983	144	74	254	115	164	274.5	226	439.68	486	404	429	31	92	10	8	5
12476	7866	4902	3	1596	1272	28352	578	150	747	38	222	178	77	255	299.65	178	453.12	482	250	401	61	98	7	16	9
4656	13166	9536	778	464	252	28084	1192	9	656	1	10	55	5	86	277.51	89	432.17	325	328	316	14	35	0	0	1
4626	13172	9534	760	470	276	28104	1170	8	648	0	26	67	5	78	276.87	103	432.33	355	328	307	16	41	0	0	1
10768	7794	5552	0	2382	1840	28812	523	62	473	8	420	153	23	311	331.15	87	470.58	444	156	335	154	79	6	54	19
11046	7966	5496	3	2102	1644	28666	502	76	531	11	368	164	36	293	319.34	109	461.24	493	149	376	105	96	9	44	21
4504	13182	9572	809	532	306	28112	1192	7	594	1	14	67	3	84	277.62	88	434.5	332	276	302	17	46	0	2	1
10548	7788	5652	0	2474	1884	28830	478	61	475	14	428	186	28	313	330.4	94	469.67	479	133	336	117	89	2	50	40
5132	12870	9262	657	532	302	28112	1136	5	711	0	12	60	3	77	278.41	124	434.84	365	375	337	16	39	0	2	3
14308	7566	4380	4	924	666	27914	656	327	1007	153	68	253	129	162	272.29	238	436.01	474	433	423	20	89	27	2	3
4780	13110	9450	731	458	278	28104	1180	5	673	0	28	61	4	78	276.07	94	432.66	350	358	315	14	41	0	0	0
13598	7600	4534	2	1266	966	28110	577	253	818	97	140	228	116	196	282.98	198	448.76	505	300	423	41	97	14	6	9
4502	13298	9614	844	410	240	28084	1244	8	620	0	20	54	5	89	277.85	87	432.42	290	320	301	14	35	0	0	1
14446	7616	4336	5	790	608	27870	646	354	1016	160	70	260	156	163	271.89	257	437.83	457	407	443	23	89	21	4	1
5796	12408	8872	564	636	392	28142	1072	15	724	2	34	80	9	111	285.72	108	438.85	381	378	331	14	54	0	4	2

SUPPLEMENTARY INFORMATION:Monte Carlo Atomistic Simulation and Machine Learning Analysis of Na-K Eutectic Alloy in Condensed Phases, D. Reitz and E. Blaisten-Barojas, George Mason University, Fairfax, VA 22030

12864	7998	4860	4	1272	1012	28174	616	191	872	64	156	189	89	215	281.08	190	444.79	482	335	436	40	97	16	12	2
4078	13588	9862	927	332	184	28054	1284	8	579	1	8	58	4	55	271.02	82	423.06	301	292	278	16	45	0	2	2
3898	13674	9954	972	336	182	28052	1287	5	569	1	8	61	3	66	270.09	93	422.66	271	263	287	19	42	0	0	4
14236	7564	4426	9	936	670	27912	612	323	1020	167	78	269	114	170	273.29	252	437.06	496	407	428	16	89	18	2	3
7108	11398	8068	384	982	614	28222	960	38	778	10	52	79	21	136	287.7	124	443.74	413	388	364	28	48	0	0	5
14346	7634	4372	3	850	624	27882	640	312	1055	166	56	260	116	162	267.67	274	429.6	478	427	439	17	87	14	0	0
14216	7642	4376	5	910	708	27940	614	293	998	154	82	260	102	184	274.91	244	438.57	472	381	435	22	89	16	6	4
4180	13526	9802	913	346	194	28058	1262	7	603	1	10	54	3	68	273.8	91	427.03	288	291	299	19	40	0	0	2
12600	8008	4888	5	1416	1140	28234	643	181	850	62	168	177	95	221	280.48	183	445.32	479	333	420	39	84	10	12	5
5728	12410	8888	550	676	412	28146	1057	28	721	2	32	74	16	105	282.72	109	439.26	395	393	339	15	42	1	0	4
3924	13674	9912	962	326	206	28062	1295	5	562	1	20	61	3	65	271.06	90	423.8	283	267	269	18	41	0	0	2
3998	13620	9902	976	346	184	28054	1311	9	569	2	4	47	5	58	273.33	91	425.8	281	269	281	14	34	0	0	2
14136	7616	4342	5	1022	782	27970	631	292	943	149	72	255	118	157	272.77	248	436.98	513	365	414	25	91	15	0	1
10954	7880	5594	0	2136	1676	28720	520	71	548	7	412	159	32	300	323.43	88	466.27	466	195	349	121	87	8	62	28
14452	7692	4358	6	746	560	27856	645	338	1083	158	42	258	134	155	269.72	267	431.58	470	453	447	18	82	24	6	1
13450	7722	4648	4	1196	928	28102	600	237	914	96	140	224	119	212	278.25	201	445.75	474	330	447	37	110	10	18	1
13944	7550	4442	4	1116	850	28028	607	288	907	108	116	228	127	217	278.57	188	445.16	437	347	461	34	101	22	10	3
11202	7842	5480	3	2124	1598	28636	509	79	576	12	342	162	46	261	313.31	108	461.21	521	174	394	84	97	3	44	30
4624	13178	9520	772	484	284	28106	1195	5	643	0	16	58	2	89	275.02	104	431.65	327	333	315	11	41	0	0	2
13300	7690	4596	1	1344	1070	28172	600	211	802	83	158	195	96	192	283.97	174	445.3	486	305	448	56	80	10	14	6
14184	7800	4460	6	802	594	27880	677	304	1074	150	36	243	116	174	269.9	250	431.61	444	469	440	11	81	20	4	2
11412	7814	5428	2	2056	1508	28574	544	105	590	21	332	165	53	274	312.89	114	459.71	477	193	375	103	88	10	24	17
4200	13504	9772	893	362	216	28068	1269	6	613	1	12	61	4	74	271.35	94	424.37	293	313	286	12	39	0	2	2
14546	7582	4320	3	778	572	27858	615	365	1041	167	56	252	149	163	271.6	251	435.64	470	414	469	19	70	25	4	2
4792	13048	9424	731	524	308	28116	1181	6	648	0	20	68	3	88	277.39	96	432.65	342	340	293	16	50	0	0	1
10870	7950	5490	2	2212	1762	28720	535	64	525	13	390	153	29	283	316.25	111	463.8	484	174	369	108	78	2	40	29
3938	13636	9918	974	358	202	28062	1316	8	559	1	8	60	5	59	271.19	86	424.34	279	273	258	19	42	0	2	2
4366	13230	9650	838	552	302	28110	1251	9	586	1	10	52	6	79	274.05	87	427.68	314	305	289	9	30	0	0	2
4228	13528	9794	900	320	172	28048	1264	3	622	1	6	55	2	63	272.8	96	426.72	303	306	293	10	41	0	0	4
11032	7868	5564	0	2160	1644	28706	514	77	550	14	388	166	43	289	324.01	71	465.84	438	190	390	129	90	1	44	32
5712	12424	8874	590	678	432	28158	1097	18	715	5	36	70	9	129	283.81	104	440.88	338	380	332	19	42	0	2	2
12200	7964	5250	5	1602	1094	28292	626	139	791	41	166	190	62	238	292.74	154	449.6	478	296	377	48	110	13	16	12
5176	12752	9218	652	604	352	28134	1142	9	693	1	32	68	5	89	282.08	91	438.9	366	371	309	19	54	0	0	0
3996	13576	9868	924	390	230	28074	1292	4	560	0	12	64	3	67	270.88	75	422.71	296	288	261	12	49	0	0	3
5338	12586	9070	617	702	436	28172	1064	10	638	1	36	62	8	102	282.85	100	438.73	390	317	355	23	32	0	4	0
4308	13388	9696	854	428	252	28086	1226	6	601	1	14	63	5	70	272.12	93	425.6	319	302	299	19	39	0	0	1

5410	12648	9092	605	618	352	28136	1092	10	709	0	16	58	7	110	281.36	96	437.52	356	363	353	18	40	0	0	2
12566	7812	5000	3	1542	1152	28302	599	161	795	51	212	155	77	213	290.22	164	450.23	502	291	441	60	81	9	16	5
5244	12714	9172	630	624	362	28140	1107	5	713	1	22	57	3	90	279.49	102	436.35	367	365	350	24	36	0	2	0
6788	11718	8272	416	828	536	28196	968	28	785	4	50	73	17	132	288.12	139	443.7	417	398	379	21	48	0	4	1
7038	11590	8154	398	802	528	28174	939	42	800	9	58	86	24	120	286.77	142	444.63	447	397	368	19	55	1	4	5
3960	13578	9902	935	410	218	28072	1285	4	546	1	4	67	3	72	274.25	76	428.71	286	275	273	12	44	0	0	4
11342	7766	5462	0	2184	1528	28594	546	96	576	21	274	167	47	288	311.06	103	461.11	463	182	387	94	88	8	34	21
3898	13760	9954	976	258	158	28038	1295	9	566	1	8	60	6	69	270.58	84	422.31	278	267	278	11	44	0	2	2
13630	7862	4704	7	980	708	27970	599	260	1038	124	76	248	106	172	276.01	242	440.1	493	382	445	34	103	17	10	0
4708	13110	9518	784	478	258	28092	1215	9	649	0	20	60	5	73	277.86	97	432.31	326	336	298	17	41	0	0	3
13420	7772	4654	2	1238	904	28086	612	248	899	101	92	221	112	193	279.17	222	446.38	495	349	431	36	90	14	6	1
13882	7842	4566	6	912	670	27928	654	282	1024	135	56	240	114	164	273.83	252	437.38	469	401	439	23	89	9	0	1
5094	12672	9164	656	748	474	28198	1139	2	632	0	44	62	1	100	280.9	79	439.15	361	341	305	24	44	0	0	3
4522	13356	9632	840	350	192	28058	1243	6	663	0	6	60	3	74	275.61	107	430.13	310	327	293	11	40	0	0	1
4120	13508	9828	922	398	210	28068	1269	5	597	0	4	63	4	65	271.9	112	424.16	311	283	275	12	42	0	0	1
4908	12906	9374	696	600	326	28130	1142	6	642	0	14	63	4	80	278.91	99	435.7	367	327	322	18	40	0	2	3
4510	13332	9604	794	390	234	28084	1206	2	640	0	12	59	2	70	278.67	96	434.24	322	333	314	21	40	0	2	1
7108	11378	8056	377	986	632	28220	906	42	754	11	50	91	24	156	287.34	140	445.84	422	347	378	28	58	1	10	3
13642	7780	4572	1	1068	852	28046	574	247	937	82	122	233	127	170	283.46	223	445.48	494	335	458	50	117	15	10	6
3854	13674	9978	973	356	186	28054	1312	7	551	1	6	58	3	64	272.77	71	426.14	271	273	266	17	40	0	0	2
4422	13378	9660	858	368	218	28060	1245	7	637	0	14	55	3	72	276.03	95	428.6	302	318	303	15	36	0	0	1
11890	7788	5196	2	1860	1418	28472	519	130	649	31	284	186	75	269	302.23	140	456.3	469	206	418	92	96	5	36	14
4518	13202	9558	804	484	314	28122	1179	7	611	0	46	58	4	80	278.5	102	432.9	351	283	308	12	41	0	0	5
4324	13438	9718	888	346	214	28066	1270	5	626	0	22	66	3	69	273.85	82	427.68	291	318	284	14	50	0	4	3
4478	13312	9616	813	422	244	28082	1213	9	639	3	10	57	5	79	273.05	94	427.98	316	327	314	13	36	0	0	3
4134	13462	9792	894	430	248	28082	1276	5	578	1	16	63	3	72	271.41	86	425.47	295	305	273	14	39	0	0	3
4394	13332	9644	840	450	260	28086	1238	8	613	1	6	61	5	66	274.72	97	429.34	324	306	281	17	42	0	0	3
4070	13586	9872	926	330	180	28050	1271	12	603	1	12	66	7	68	273.11	90	426.89	297	297	278	12	45	0	0	1
3742	13792	10038	1011	302	164	28042	1319	7	544	1	4	62	5	69	270.68	79	422.76	259	262	271	12	40	0	0	4
11220	7902	5472	0	2086	1580	28642	541	84	556	16	330	167	43	277	315.23	88	464.74	466	205	364	118	89	5	50	19
13054	7816	4796	4	1368	1002	28172	597	209	845	70	126	200	99	211	282.19	184	447.27	486	313	440	47	101	19	8	3
4006	13636	9872	968	332	204	28064	1301	9	583	1	12	51	6	69	271.02	91	423.51	276	277	284	12	39	0	2	3
11122	8072	5418	4	2004	1630	28646	523	82	566	9	358	168	44	283	315.89	109	461.02	482	183	380	116	80	4	40	14
4086	13618	9870	939	304	162	28044	1271	5	599	1	4	63	4	64	271.77	89	426.01	287	279	293	18	45	0	0	1
13556	7914	4690	5	1008	742	27986	653	258	1003	124	74	244	102	181	275.9	212	441.31	463	423	418	27	105	13	2	4
7170	11434	8060	386	894	574	28190	942	38	772	12	56	91	21	123	289.14	135	446.56	431	384	367	30	54	1	2	3

SUPPLEMENTARY INFORMATION:Monte Carlo Atomistic Simulation and Machine Learning Analysis of Na-K Eutectic Alloy in Condensed Phases, D. Reitz and E. Blaisten-Barojas, George Mason University, Fairfax, VA 22030

14014	7750	4534	3	908	660	27942	650	300	1010	120	74	220	140	135	276.2	244	441.23	484	405	462	37	79	22	2	1
4024	13534	9850	928	420	240	28080	1277	7	561	1	12	52	3	59	273.55	83	426.13	291	275	288	20	35	0	0	6
10632	7910	5742	0	2344	1720	28782	491	67	486	12	378	177	33	309	330	79	470.94	450	164	382	118	76	3	48	37
11162	8006	5482	1	2066	1558	28620	551	82	605	11	308	157	45	280	311.74	114	457.56	484	203	378	97	89	8	34	15
13796	7710	4570	7	1040	770	27984	604	295	964	139	88	232	113	182	273.29	244	438.68	494	367	449	26	77	18	10	0
4396	13330	9658	843	432	254	28090	1252	7	612	1	18	63	6	80	274.51	80	430.27	295	318	286	14	45	0	2	5
14010	7792	4472	5	884	702	27946	620	300	1007	145	82	243	121	169	275.4	248	439.27	501	411	437	20	82	14	4	0
13610	7868	4664	7	1014	756	28006	634	247	977	101	90	218	113	177	275.83	228	442.89	477	373	445	34	90	11	4	0
12168	7956	5092	4	1594	1280	28376	564	142	757	36	260	178	84	233	297.43	166	452.68	479	257	437	78	101	6	26	9
14012	7684	4448	4	978	754	27968	621	277	986	129	86	243	108	174	275.74	233	442.13	472	386	443	33	92	22	4	3
13640	7664	4590	2	1160	868	28050	625	263	915	109	120	226	109	186	276.5	233	444.28	490	340	419	36	96	16	8	1
4584	13254	9580	775	420	230	28074	1192	7	648	0	4	55	5	78	275.87	90	430.19	322	355	325	16	39	0	2	3
14314	7634	4356	1	868	662	27904	662	314	1039	154	66	261	124	162	270.84	244	434.88	463	453	424	21	90	21	4	3
14280	7710	4384	6	808	640	27902	644	314	1052	157	80	250	120	145	271.83	262	435.33	500	436	434	20	82	21	0	1
4700	13194	9522	784	418	232	28076	1186	10	656	0	8	44	6	73	276.07	95	430.26	333	326	337	15	34	0	2	2
6262	11944	8538	457	866	552	28222	1030	16	782	2	54	72	9	108	286.39	137	441.54	417	391	330	25	48	0	4	5
13904	7758	4492	10	956	768	27986	620	281	993	126	90	216	115	169	276.58	240	440.85	496	390	456	27	75	23	16	1
4174	13400	9748	896	484	282	28104	1224	10	560	1	16	52	8	75	274.16	104	426.63	323	246	305	16	36	0	0	1
5702	12344	8886	524	766	450	28176	1000	12	717	5	26	81	5	104	284.06	120	439.17	431	340	356	22	49	0	2	1
12654	7916	4984	3	1438	1068	28242	635	165	810	48	158	186	73	213	294.09	161	450.57	479	330	409	52	97	18	24	6
13736	7916	4664	7	920	656	27946	649	270	1061	120	50	235	117	170	276.13	256	439.46	462	420	443	29	101	16	4	1
4848	13046	9430	759	476	272	28096	1190	8	685	0	24	55	4	81	276.07	109	430.68	331	344	321	12	41	0	0	4
13500	7874	4670	11	1038	822	28032	622	272	964	133	116	232	107	179	276.96	239	441.93	505	382	421	26	81	17	12	3
4026	13624	9880	969	328	186	28052	1300	6	588	1	8	51	5	69	270.66	92	422.13	275	279	285	12	35	0	0	2
4436	13348	9668	838	382	214	28064	1213	5	641	1	16	50	3	69	273.34	100	427.59	322	305	322	14	40	0	0	2
11400	7836	5470	1	2078	1478	28582	542	88	597	17	272	180	44	282	311.58	116	458.79	468	178	370	101	88	6	40	16
14222	7748	4450	5	810	600	27886	644	309	1063	159	56	257	111	137	269.09	278	432.53	505	421	427	23	89	17	0	0
13898	7770	4560	5	944	704	27964	670	287	1024	114	78	228	130	165	275.96	226	442.04	457	432	438	28	97	22	10	3
5114	12798	9270	645	596	330	28132	1105	8	709	1	24	51	3	97	277.1	116	434.25	369	358	356	12	35	0	0	3
14098	7732	4426	8	910	706	27948	612	314	1008	139	70	234	132	182	275.15	253	438.62	492	389	458	14	80	20	6	0
3962	13526	9860	949	454	272	28094	1294	12	548	1	16	46	6	65	270.92	86	422.99	297	263	275	16	30	0	4	1
4218	13422	9746	886	436	250	28084	1234	6	578	1	12	67	3	74	272.34	88	425.44	312	277	293	15	47	0	0	3
12078	8142	5212	4	1514	1164	28344	580	138	783	39	222	170	74	246	293.3	134	449.29	479	301	438	56	91	7	12	11
6762	11700	8288	386	860	542	28204	956	19	799	2	52	79	11	126	286.69	130	444.49	445	403	359	18	47	0	0	3
14320	7664	4408	6	840	600	27880	636	337	1048	161	48	259	127	136	273.35	292	436.62	503	415	431	23	82	26	0	3
5436	12646	9050	608	598	380	28148	1083	6	736	0	36	65	4	89	281.5	128	437.76	397	362	342	16	43	0	2	3

7200	11358	8028	386	892	620	28208	933	52	753	12	106	100	29	144	287.65	124	444.21	425	370	357	23	59	1	4	3
13782	7624	4474	1	1132	900	28054	593	259	891	98	128	218	119	193	278.76	205	445.05	489	326	455	32	99	18	14	5
4052	13532	9852	898	404	224	28076	1235	7	561	1	12	67	5	82	274.16	74	428.11	302	281	293	12	38	0	0	4
14280	7562	4394	2	944	672	27920	622	326	990	155	64	252	132	146	273.56	248	438.8	504	402	439	25	88	16	4	4
14304	7586	4330	4	900	708	27920	622	330	1013	164	88	261	122	162	273.49	246	436.66	488	408	428	27	79	23	4	2
4478	13284	9648	802	436	226	28080	1209	5	638	0	8	64	4	82	276.44	81	431.28	307	328	315	12	41	0	0	7
11124	8026	5572	0	2026	1500	28596	547	81	599	16	302	158	46	256	310.29	111	460.92	489	210	401	102	79	2	46	20
12054	7890	5102	1	1728	1356	28414	571	139	698	26	260	152	80	231	298.46	133	454.39	478	264	436	84	84	5	20	13
11146	7880	5460	2	2112	1648	28674	519	89	545	15	386	169	47	284	325.08	94	467.77	460	199	375	115	83	5	42	31
14222	7750	4486	3	754	574	27866	628	326	1092	161	78	277	125	172	271.93	262	437.22	463	432	430	21	108	20	2	1
5582	12398	8982	555	752	428	28178	1075	14	692	2	30	68	8	100	284.98	109	441.41	400	382	329	20	38	0	6	1
12418	7650	4862	1	1788	1416	28420	568	161	666	45	262	189	89	267	293.13	137	451.15	465	244	403	76	92	7	18	9
4920	12808	9306	667	684	410	28166	1092	5	645	0	36	46	2	101	280.07	111	434.97	381	314	354	12	28	0	2	5
5824	12360	8856	553	678	398	28142	1061	18	717	4	26	75	11	116	283.17	114	439.79	376	360	346	17	41	0	0	1
11748	7748	5276	0	2006	1436	28496	546	121	651	26	248	180	58	266	302.84	120	455.67	466	226	404	92	96	13	30	16
5282	12646	9108	677	652	410	28136	1104	18	625	8	38	92	4	108	281.86	110	439.79	362	297	304	20	54	0	0	1
4280	13298	9700	843	500	298	28112	1233	6	571	0	34	64	4	76	275.37	78	432.08	316	299	279	21	46	0	2	3
4746	13188	9496	780	408	228	28072	1173	6	681	0	4	59	4	83	276.12	111	430.88	331	320	334	11	40	0	2	3
11542	7868	5290	1	1958	1530	28546	536	119	607	29	322	176	65	250	306.98	129	457.55	492	215	385	106	86	3	34	20
6880	11582	8200	392	900	588	28220	930	25	759	3	66	87	14	127	286.76	124	444.38	443	374	373	27	55	0	4	4
13004	7938	4750	0	1306	1036	28182	588	190	867	60	138	185	87	200	283.61	205	445.53	505	304	452	40	94	16	10	10
4526	13168	9558	781	540	310	28118	1214	7	623	0	16	65	6	99	278.65	79	435.18	317	330	291	11	46	0	0	0
14376	7516	4320	2	936	690	27912	644	329	984	157	72	260	134	164	272.32	226	436.88	463	416	436	27	92	20	2	2
14206	7688	4416	5	878	656	27906	664	312	1042	153	58	254	111	153	269.57	254	431.81	486	438	421	17	84	25	4	1
5286	12646	9106	584	692	414	28168	1058	7	690	0	24	61	4	93	283.03	122	437.1	406	357	355	19	37	0	0	3
4414	13272	9624	812	468	296	28112	1212	6	594	1	32	52	2	74	275.25	92	430.58	339	304	297	15	35	0	6	2
11666	7878	5302	4	1926	1432	28510	535	120	648	29	280	180	57	273	311.58	122	459.09	474	217	387	94	98	10	24	16
14408	7576	4306	5	878	664	27898	621	328	1017	160	64	267	122	152	270.83	273	434.81	507	409	421	22	93	21	2	1
4572	13170	9538	779	506	304	28116	1216	4	626	0	24	61	3	81	276.48	93	432.62	332	335	290	11	37	0	2	3
4848	13032	9424	725	502	282	28108	1165	8	674	2	20	68	5	73	279.31	112	436.43	360	338	308	17	43	0	0	2
6666	11616	8364	460	914	568	28214	937	31	707	7	74	81	19	124	288.15	116	443.82	445	331	375	25	55	1	12	2
4636	13154	9518	770	492	284	28100	1169	9	630	1	16	58	4	84	276.11	105	430.67	347	312	322	14	37	0	0	0
11482	7950	5394	1	1960	1446	28540	554	79	641	15	278	163	39	244	309.03	135	457.67	498	197	399	99	97	7	28	14
13642	7818	4628	9	1018	788	28008	634	272	979	124	100	228	114	187	276.44	222	439.7	473	396	435	29	86	11	12	1
5086	12754	9246	628	670	376	28150	1097	5	678	0	18	65	3	96	279.74	107	436.66	382	336	331	16	42	0	0	4
12872	7914	4908	6	1364	982	28172	623	194	840	69	126	172	93	187	284.74	174	447.87	501	335	440	49	81	8	6	3

SUPPLEMENTARY INFORMATION:Monte Carlo Atomistic Simulation and Machine Learning Analysis of Na-K Eutectic Alloy in Condensed Phases, D. Reitz and E. Blaisten-Barojas, George Mason University, Fairfax, VA 22030

11870	8006	5300	1	1708	1256	28398	581	117	745	33	244	179	54	247	297.75	158	453.96	472	256	402	74	97	6	14	14
14506	7590	4362	8	784	552	27844	641	350	1077	173	48	265	134	139	268.18	293	429.95	496	435	436	14	82	21	2	3
7392	11114	7914	328	1038	680	28246	935	34	752	9	102	96	16	145	287.71	122	445.54	427	391	345	29	57	2	6	7
8258	10656	7428	267	1100	716	28238	870	54	816	15	78	109	32	138	290.37	154	445.65	450	396	378	42	53	1	2	3
4162	13462	9780	888	410	246	28082	1276	7	594	1	18	68	3	66	273.06	85	425.86	298	304	267	17	47	0	2	4
11768	7926	5258	1	1856	1378	28456	521	118	665	30	250	181	65	272	300.1	126	454.65	485	216	415	78	92	5	18	14
5682	12286	8892	537	810	478	28192	1090	17	666	1	42	66	14	109	285.73	91	441.12	380	377	323	23	41	0	2	1
4708	13106	9494	797	488	280	28100	1212	5	663	0	24	60	3	79	275.41	100	429.76	324	333	297	20	40	0	0	0
5734	12380	8872	537	716	434	28168	1038	19	715	1	32	53	13	115	280.06	108	435.86	382	368	383	20	31	0	0	2
5490	12462	8998	560	742	448	28182	1067	13	693	0	38	73	7	116	285.24	105	440.22	381	369	332	21	41	0	4	2
11518	7798	5428	2	2004	1456	28556	511	106	596	28	318	187	43	270	311.24	115	461.86	487	195	370	96	90	4	34	29
12322	7980	5004	3	1554	1234	28322	594	145	779	33	206	160	79	223	290.65	156	450.04	474	275	456	62	89	12	22	10
14436	7634	4370	9	780	574	27852	628	343	1058	175	56	279	130	156	272.06	278	436.17	482	415	435	15	90	22	2	3
13422	7908	4672	9	1084	854	28052	631	238	961	110	96	227	104	167	275.88	231	443.16	489	390	427	42	97	11	12	5
7922	10970	7682	301	938	608	28204	836	52	821	12	80	94	31	131	289.55	178	445.54	474	363	422	30	61	3	4	4
4666	13106	9492	765	532	302	28110	1209	7	654	0	12	66	3	84	277.49	101	431.98	332	335	286	14	53	0	0	2
4932	12954	9358	677	540	314	28122	1135	8	692	1	22	69	6	84	276.73	130	433.22	362	353	323	21	39	0	2	0
4738	13142	9482	767	450	260	28088	1188	9	676	0	16	67	7	86	276.98	107	434.04	320	330	312	16	44	0	0	4
14534	7550	4310	3	830	590	27860	624	348	1042	169	44	274	140	138	269.38	284	431.63	520	421	420	15	94	17	2	2
5134	12796	9218	656	598	356	28122	1116	11	683	1	20	71	6	106	277.51	99	435.92	349	362	334	16	40	0	0	2
12968	7752	4770	2	1414	1112	28224	567	190	809	67	196	198	80	223	291.61	161	449.93	505	297	417	55	100	14	10	4
4208	13502	9784	892	356	200	28060	1264	6	604	1	10	65	5	61	273.84	103	427.81	308	307	282	16	41	0	0	1
10646	7816	5634	1	2458	1812	28788	513	68	492	14	380	144	33	268	322.54	90	466.38	516	164	362	129	70	6	36	29
4384	13348	9672	842	418	242	28082	1208	8	595	0	18	58	4	83	273.71	80	429.09	305	292	319	19	41	0	0	2
11166	8104	5512	2	1948	1486	28554	551	79	638	11	314	179	39	299	308.46	127	459.19	457	215	371	86	97	5	24	16
14452	7682	4346	3	768	570	27860	635	314	1089	154	40	259	124	147	270.78	279	434.67	486	427	446	21	94	19	2	0
14278	7622	4374	4	904	678	27930	646	312	983	158	66	253	118	154	273.09	241	439.49	477	405	436	28	76	25	8	3
5456	12696	9100	594	534	306	28112	1083	9	753	2	20	51	4	90	280.54	114	437.19	377	383	375	15	37	0	0	2
14338	7772	4424	4	716	546	27850	630	317	1126	166	54	249	112	143	269.52	279	431.23	495	454	457	17	75	15	0	0
12152	7930	5148	3	1658	1228	28344	583	154	710	34	214	166	82	231	300.49	146	451.31	456	255	427	80	81	13	10	16
4228	13562	9780	917	294	174	28046	1276	12	619	1	8	48	5	63	272.92	105	426.1	292	303	298	14	34	0	0	1
14302	7672	4386	4	840	628	27886	654	320	1048	159	56	242	124	150	267.96	271	429.36	486	433	437	19	70	19	2	0
10558	7634	5668	0	2544	1944	28900	504	56	430	13	482	163	28	294	335.16	86	471.82	502	128	311	140	77	1	62	32
4452	13322	9626	817	420	248	28082	1196	10	636	0	14	63	6	81	275.85	113	431.14	327	301	308	16	41	0	0	1
3924	13684	9928	976	320	186	28050	1318	6	573	1	6	58	3	70	270.43	84	422.24	264	282	270	13	42	0	2	2
12940	7718	4774	2	1492	1136	28254	618	204	748	72	178	193	105	216	287.31	160	447.51	500	302	411	45	86	11	16	5

SUPPLEMENTARY INFORMATION:Monte Carlo Atomistic Simulation and Machine Learning Analysis of Na-K Eutectic Alloy in Condensed Phases, D. Reitz and E. Blaisten-Barojas, George Mason University, Fairfax, VA 22030

4072	13606	9856	906	326	186	28054	1272	5	606	1	8	53	2	62	272.76	94	424.58	289	299	301	16	35	0	0	3
5066	12972	9302	685	464	278	28102	1139	5	694	0	20	71	5	88	279.53	105	436.51	339	364	339	21	45	0	0	2
4276	13430	9736	882	398	222	28070	1241	5	609	1	8	68	3	75	274.05	98	427.31	305	296	290	15	34	0	0	2
11288	7874	5484	4	2010	1544	28612	544	88	589	15	364	186	38	302	314.12	98	461.48	430	184	365	99	108	14	42	27
6310	11950	8518	480	812	532	28186	996	29	722	10	62	100	14	132	286.27	116	442.43	401	371	340	21	59	3	2	3
6212	12040	8628	429	784	470	28180	982	26	756	9	44	80	12	119	286.43	122	442.76	422	402	365	20	42	1	2	3
14082	7462	4358	3	1134	866	28028	623	287	885	109	116	235	142	191	277.57	197	445.11	462	341	440	38	107	17	10	2
5016	12958	9342	688	502	274	28102	1162	7	709	2	10	57	4	92	277.9	103	434.72	334	374	338	11	42	0	0	3
4350	13452	9720	892	340	186	28052	1252	8	618	2	4	71	6	72	273.09	91	426.6	296	305	289	16	47	0	0	1
4228	13452	9766	893	398	218	28070	1247	8	607	1	8	57	6	74	274.53	100	427.49	307	290	294	12	36	0	0	2
14036	7706	4462	10	930	718	27942	669	314	1035	155	86	251	118	139	272.09	268	435.14	493	423	409	32	88	22	4	1
5980	12274	8750	532	680	426	28148	1026	16	742	2	36	69	8	121	283.33	115	440.59	394	378	359	16	43	1	2	1
14336	7644	4392	3	832	612	27876	639	315	1083	157	56	261	125	154	271.37	277	434.42	489	440	431	16	91	16	4	2
4160	13550	9838	931	326	166	28044	1267	5	610	1	4	70	2	70	271.89	93	425.3	295	285	281	10	52	0	0	3
10344	7884	5750	1	2442	1914	28876	481	60	466	6	484	145	40	304	334.26	89	470.78	471	138	382	130	78	4	56	38
4966	12970	9350	707	502	294	28104	1152	7	699	0	20	69	3	103	279.98	113	436.78	326	343	323	18	44	0	2	1
4326	13368	9682	850	434	260	28088	1242	7	596	1	18	66	3	79	275.35	94	428.85	307	310	287	13	39	1	0	3
3762	13828	10026	1011	266	152	28036	1312	7	547	1	2	53	3	64	270.48	85	424.34	271	255	284	9	35	0	0	3
5734	12334	8880	529	744	446	28180	1028	7	727	2	38	61	3	114	284.67	117	439.77	412	359	360	14	39	0	4	2
4138	13446	9794	897	456	246	28084	1266	6	564	1	4	63	4	70	272.36	86	425.82	300	286	276	19	38	0	0	1
4644	13228	9552	789	422	226	28074	1201	11	655	0	2	55	5	81	277.03	92	431.51	318	337	322	15	41	0	0	1
3870	13788	9994	1008	230	132	28026	1315	8	601	2	12	65	5	56	270.83	106	422.35	274	270	268	15	47	0	0	2
12118	7884	5066	1	1734	1344	28408	584	151	699	33	240	159	85	248	292.97	143	451.81	503	260	414	55	78	6	20	11
4900	12942	9334	707	572	356	28136	1101	5	622	0	32	58	3	93	278.04	110	433.22	368	298	352	20	36	0	0	2
4330	13332	9682	854	474	266	28092	1235	7	607	2	6	64	3	90	272.88	89	429.33	293	299	295	15	36	0	2	3
4336	13398	9692	859	404	236	28078	1233	5	605	1	12	54	2	68	276.25	95	430.01	313	301	302	18	33	0	0	3
14264	7682	4428	5	834	614	27884	638	330	1059	165	58	264	128	152	269.38	260	430.52	486	436	430	19	87	16	4	4
5644	12354	8890	574	776	476	28178	1074	14	649	3	38	62	8	106	285.58	85	441.02	393	350	338	17	46	0	0	2
4562	13176	9552	817	510	292	28108	1222	12	636	0	16	50	8	95	276.45	94	433.1	283	315	317	24	35	0	0	1
14490	7720	4320	7	704	562	27852	631	338	1090	167	56	252	139	142	269.38	302	432.11	508	433	449	10	78	17	0	1
11872	7838	5192	2	1848	1420	28484	544	126	646	18	280	172	66	252	311.57	119	458.42	492	206	389	88	98	15	28	18
4492	13140	9588	809	562	316	28124	1201	9	576	0	24	59	7	93	277.35	85	432.07	320	292	307	15	50	0	2	2
4038	13556	9868	952	390	208	28064	1293	8	576	1	4	64	5	66	270.63	94	423.78	289	267	268	13	47	0	0	3
4160	13464	9796	905	422	226	28072	1270	9	582	1	4	58	5	75	271.34	85	424.37	287	291	288	14	36	0	0	3
3816	13744	9996	992	308	172	28044	1321	11	552	1	8	58	5	70	270.83	78	423.71	268	276	257	11	40	0	0	3
10730	7904	5568	1	2272	1808	28760	533	76	530	16	426	154	38	288	316.49	93	463.58	500	183	351	120	80	8	42	19

SUPPLEMENTARY INFORMATION:Monte Carlo Atomistic Simulation and Machine Learning Analysis of Na-K Eutectic Alloy in Condensed Phases, D. Reitz and E. Blaisten-Barojas, George Mason University, Fairfax, VA 22030

12508	7784	4978	1	1596	1204	28304	582	172	753	67	224	212	86	227	294.02	158	452.14	469	263	409	72	100	8	8	10
13796	7736	4530	9	1030	804	28010	624	264	966	121	104	233	113	181	277.13	231	443.03	480	365	445	27	92	15	10	4
5510	12538	9036	580	650	376	28140	1060	9	721	1	30	74	5	92	282.93	136	440.47	393	362	345	25	49	0	0	2
5636	12436	8912	516	732	440	28180	1054	7	745	0	24	69	7	115	283.73	108	438.97	380	401	360	20	45	0	0	2
4506	13172	9602	825	518	284	28104	1216	5	609	0	22	58	2	82	275.51	84	431.86	322	308	295	15	41	0	0	3
5100	12816	9288	671	576	316	28120	1127	6	692	0	24	69	5	80	279.21	99	436.68	360	364	333	25	51	0	0	2
11966	8030	5236	2	1712	1238	28388	614	118	734	31	180	163	69	232	300.36	142	454.49	483	276	403	68	91	7	24	10
13546	7902	4666	2	1002	786	28014	622	261	992	117	102	233	114	161	275.97	241	442.96	493	392	435	40	101	9	10	4
4694	12980	9454	740	632	362	28150	1170	5	615	0	28	62	2	82	276.67	89	431.95	348	311	306	24	40	0	0	3
10910	7938	5594	7	2196	1650	28686	552	78	548	13	346	166	44	306	317.35	119	462.38	444	194	369	100	83	7	48	25
4202	13494	9784	885	364	206	28062	1265	6	609	1	12	77	3	78	271.91	86	425.11	290	321	271	14	60	0	0	2
10928	7812	5548	0	2272	1732	28746	515	73	518	18	416	167	37	303	323	105	467.66	487	166	353	104	81	2	36	30
12926	8082	4892	4	1158	926	28144	634	200	900	67	150	190	103	195	281.97	189	446.68	457	356	448	53	94	11	10	5
11062	7796	5520	0	2176	1700	28728	484	84	487	21	400	159	45	303	325.25	95	468.36	481	139	393	105	71	3	70	31
6190	11910	8602	490	912	546	28220	1033	16	687	3	60	81	9	118	288.2	102	443.64	412	368	319	21	48	1	0	6
12098	7742	5096	2	1842	1378	28440	553	138	656	30	270	178	76	252	303.22	128	457.33	466	216	407	90	105	10	14	17
4306	13384	9722	883	426	232	28080	1269	6	593	0	10	52	5	83	273.92	82	427.62	285	303	293	11	37	0	0	2
4794	12988	9410	710	568	338	28128	1138	3	662	0	30	60	0	93	279.26	103	435.58	350	338	327	18	38	0	0	4
3982	13602	9914	960	366	192	28066	1293	4	578	1	10	61	3	69	271.73	91	425.64	285	273	272	15	44	0	0	1
4790	13140	9458	783	420	254	28086	1212	7	682	0	24	53	3	85	276.64	101	431.07	309	351	316	15	39	0	0	2
5968	12094	8672	536	826	568	28218	1015	15	661	2	82	80	11	131	285.1	107	443.29	394	336	345	23	56	0	6	5
4036	13548	9854	936	398	228	28078	1281	5	578	1	12	55	2	75	271.9	98	425.09	293	279	280	11	35	0	2	1
7724	11022	7774	301	1006	618	28200	884	48	812	6	48	95	33	162	289.29	127	444.95	406	393	395	35	63	1	8	3
14150	7704	4450	6	872	658	27908	641	302	1034	148	70	258	112	183	274.62	236	439.11	449	400	429	24	98	17	4	2
4172	13512	9824	909	350	184	28052	1266	8	612	1	8	61	5	67	272.89	91	425.71	290	306	288	17	47	0	2	3
14384	7634	4344	7	834	626	27878	599	338	1036	166	52	266	135	168	269.77	256	432.2	484	396	458	17	90	15	4	3
4350	13368	9688	838	416	242	28082	1216	4	603	0	18	61	1	73	275.09	102	430.75	322	300	305	15	41	0	0	3
14078	7760	4474	7	882	662	27920	649	316	1014	148	62	237	133	165	271.64	245	435.57	459	407	450	27	79	16	2	2
13400	7664	4634	1	1292	986	28138	595	247	806	87	154	209	117	212	282.14	180	445.77	494	313	433	38	95	21	6	2
4586	13250	9552	768	434	256	28090	1163	3	636	0	12	62	3	80	275.33	112	428.93	346	310	326	14	39	0	0	3
13180	7804	4722	7	1272	998	28142	601	210	863	88	152	225	87	202	280.67	190	446.19	481	325	421	54	111	19	14	2
13804	7842	4598	6	928	704	27966	691	273	1041	134	86	242	108	170	276.01	247	440.69	470	454	387	21	93	11	4	3
14458	7656	4382	4	740	554	27860	644	325	1099	148	66	249	132	140	270.6	267	434.82	486	447	449	23	91	21	4	0
11176	7992	5448	0	2036	1600	28646	524	77	573	7	346	150	43	278	321.47	116	463.26	472	169	397	97	84	9	48	32
11672	7918	5232	1	1872	1488	28522	566	112	650	22	298	170	53	265	313.08	118	459.96	464	223	397	88	99	12	36	17
12518	7964	4992	2	1448	1132	28268	594	169	813	46	192	179	94	226	290.4	168	449.7	493	309	420	52	105	8	22	7

4432	13374	9670	843	364	208	28064	1238	2	658	1	16	71	1	69	273.31	99	425.65	307	327	292	18	48	0	0	2
3998	13628	9904	961	334	180	28050	1310	8	586	1	6	63	5	58	270.14	95	422.64	281	287	264	18	46	0	0	1
4356	13400	9692	841	388	224	28072	1234	7	621	0	12	61	4	68	271.68	89	425.85	320	320	296	15	38	0	0	1
6520	11866	8444	427	810	496	28176	984	19	775	6	34	86	11	122	286.08	118	442.01	400	402	371	24	51	0	6	6
10698	7998	5732	3	2262	1644	28722	505	65	521	9	334	160	32	275	322	94	468.73	482	165	365	124	84	0	52	35
13594	7774	4574	3	1138	862	28036	616	264	900	116	92	219	108	189	275.63	195	443.74	462	346	464	39	86	18	2	2
13600	7614	4580	4	1204	924	28084	592	268	851	114	158	232	114	204	279.4	184	445.3	480	308	441	40	94	20	4	2
12288	7962	5040	4	1570	1242	28352	574	142	753	23	226	180	77	226	295.4	146	452.56	474	258	419	83	119	15	24	13
14266	7644	4412	3	868	642	27902	671	309	1042	154	62	255	119	159	270.51	256	434.49	473	441	411	21	92	16	8	0
13198	7948	4800	7	1182	854	28068	635	223	926	96	80	209	94	179	277.67	210	444.26	472	370	446	43	96	16	6	3
12348	7954	5108	1	1528	1146	28314	553	149	784	34	216	178	72	232	297.9	176	454.58	494	267	424	73	97	10	14	6
13500	7862	4634	7	1082	848	28036	626	257	941	114	102	238	108	197	278.43	205	443.81	469	381	425	29	104	16	6	5
7262	11160	7968	371	1070	690	28246	929	45	700	20	90	106	15	141	288.83	104	444.32	435	362	338	33	57	2	6	5
11026	8010	5440	1	2036	1700	28676	517	77	537	10	422	186	47	289	320.16	101	462.87	455	174	366	122	91	4	42	19
5882	12460	8856	595	558	332	28104	1095	22	743	5	16	73	12	88	280.85	126	439.56	386	385	333	14	43	0	0	2
4136	13426	9716	880	490	326	28120	1239	5	563	1	22	53	4	69	274.93	76	427.67	315	273	298	19	34	0	4	5
4056	13502	9820	926	440	256	28086	1262	7	565	2	12	53	3	68	274.06	88	427.38	305	267	288	15	34	0	0	3
3930	13672	9932	974	328	182	28050	1301	10	560	1	6	66	6	78	272.56	82	425.7	267	272	267	9	40	0	0	5
5326	12626	9108	614	668	396	28154	1099	10	690	3	28	60	6	92	281.06	114	435.73	392	352	327	16	36	0	2	3
13984	7696	4462	5	994	754	27976	649	292	968	122	82	212	128	173	275.38	245	442.54	467	391	455	28	76	18	4	1
4682	13106	9472	742	536	310	28116	1165	3	634	0	10	76	3	94	279.14	97	434.3	344	318	305	9	50	0	0	5
5138	12902	9244	686	500	308	28116	1127	4	708	0	22	56	4	84	279.85	114	436.08	373	347	345	13	36	0	2	0
5688	12386	8894	567	732	442	28176	1056	12	699	2	32	56	9	108	281.91	117	438.37	392	338	364	17	38	0	2	2
5896	12412	8852	581	580	344	28110	1057	21	720	6	26	76	12	103	282.23	117	441.23	380	367	358	14	48	0	0	3
4356	13438	9710	887	348	196	28056	1268	10	629	1	8	57	5	74	271.86	96	426.18	283	319	295	15	35	0	0	2
4206	13492	9774	902	378	212	28068	1264	6	602	0	6	53	2	70	273.36	87	428.63	302	292	292	10	38	0	0	3
13880	7710	4578	8	1020	710	27968	643	267	982	114	64	240	118	175	276.28	226	442.1	478	388	418	26	111	12	6	3
4788	13120	9452	748	456	266	28098	1163	5	660	1	14	67	1	91	277.12	119	432.9	341	338	319	13	40	0	2	1
3822	13684	9982	996	364	202	28068	1333	5	545	1	14	51	2	71	271.3	84	423.56	259	266	268	14	31	0	0	1
14298	7764	4424	6	760	568	27858	655	313	1106	159	42	260	122	148	268.13	275	429.28	476	463	434	19	87	16	2	1
7114	11502	8154	398	810	514	28170	950	36	795	12	74	90	19	114	289	129	442.72	427	413	367	27	55	0	2	8
5022	12820	9276	672	628	372	28148	1142	8	678	2	28	60	4	100	280.84	88	437.18	345	362	324	20	35	0	2	2
13714	7852	4670	5	950	690	27962	653	288	1009	131	84	233	125	175	275.18	220	439.15	452	428	443	30	94	13	2	2
13450	7574	4536	3	1340	1074	28166	589	248	783	64	172	190	128	197	282.56	195	446.76	515	299	440	42	90	22	20	5
4438	13198	9566	779	550	348	28132	1169	5	583	0	32	57	3	84	277.86	80	432.02	346	294	317	17	43	0	0	4
12054	7988	5208	1	1622	1236	28378	583	133	764	20	256	150	83	225	300.34	165	452.93	507	272	425	74	102	8	12	5

5820	12400	8916	577	608	340	28116	1085	23	728	12	32	93	11	90	280.97	94	439.1	367	401	329	31	56	0	0	1
4684	13106	9482	747	518	306	28120	1162	2	638	1	24	55	1	69	278.94	108	434.25	353	320	334	21	34	0	0	4
4146	13536	9792	920	368	218	28068	1286	9	596	1	8	54	6	80	271.74	86	424.09	278	302	285	10	37	0	0	2
12900	7880	4880	2	1354	1012	28190	619	188	869	64	152	193	97	203	282.92	174	449.1	503	334	420	39	103	7	10	6
13264	7798	4768	1	1230	920	28138	641	234	899	76	144	191	111	200	281.96	195	447.33	464	345	437	47	96	22	14	2
13746	7840	4586	4	972	746	27978	626	270	988	121	82	230	113	175	275.55	231	439.14	462	390	459	35	88	15	4	3
4722	12992	9444	729	580	354	28134	1174	5	650	0	34	59	4	89	278.61	98	435.89	326	339	320	25	40	0	8	2
4440	13284	9624	788	450	276	28106	1183	4	608	0	30	58	4	79	276.28	81	432.79	338	310	317	15	40	0	2	4
4136	13592	9826	912	306	180	28050	1245	6	600	1	8	57	3	64	272.99	88	428.18	304	287	312	14	41	0	2	2
13764	7624	4486	1	1158	900	28064	616	267	890	103	118	215	118	185	277.46	181	445.56	473	354	455	40	86	21	14	5
4282	13450	9732	870	378	216	28066	1266	7	632	1	8	69	5	66	273.65	97	427.39	301	324	276	14	47	0	0	3
4044	13648	9874	932	288	176	28046	1280	6	606	1	16	56	3	68	271.74	88	424.01	284	305	288	13	40	0	0	3
11774	7812	5162	3	1922	1520	28540	563	118	608	20	318	159	73	263	304.75	127	457.74	483	213	393	93	83	8	28	15
4712	13178	9494	784	442	254	28090	1210	5	666	0	10	62	4	78	279.03	114	434.19	320	331	304	20	46	0	0	0
12412	7914	4986	0	1576	1218	28326	604	169	754	42	202	166	100	230	294.37	145	451.8	454	292	431	73	89	9	16	12
4174	13470	9778	913	406	236	28080	1288	10	590	1	16	49	4	83	271.74	85	425.48	282	303	280	11	36	0	0	0
5378	12600	9070	603	682	404	28158	1114	12	684	1	24	62	9	101	280.48	91	437.75	370	375	326	16	34	0	0	3
12444	7724	4988	0	1730	1262	28366	547	153	697	38	184	181	87	235	298.49	152	452.15	503	254	424	72	97	4	28	11
5610	12614	8982	579	544	346	28128	1099	8	756	1	28	77	4	100	280.11	113	437.58	370	398	329	17	46	1	4	2
4654	13204	9508	777	448	266	28090	1187	10	637	4	10	63	2	74	276.65	102	432.97	340	322	307	18	39	0	0	2
11992	7728	5172	0	1850	1398	28464	544	144	630	37	292	194	70	261	310.98	140	456.4	472	218	377	91	93	8	32	18
11110	7880	5416	2	2132	1704	28684	545	75	546	11	406	159	38	287	317.27	97	465.79	479	186	354	108	89	5	34	22
13804	7684	4502	4	1086	838	28028	632	278	937	129	106	239	112	196	276.45	207	443.65	465	378	421	34	96	21	8	1
4082	13618	9868	946	304	166	28044	1293	6	611	3	6	65	3	64	270.32	91	423.48	272	296	282	19	47	0	0	2
3936	13698	9944	974	300	160	28040	1299	4	586	1	2	66	3	57	270.48	95	423.14	291	273	268	13	46	0	0	2
5390	12658	9114	597	598	344	28128	1087	8	762	0	20	69	7	85	280.57	125	438.41	394	378	339	19	51	0	4	1
3846	13690	9974	972	338	192	28056	1312	7	564	2	16	53	3	75	270.93	76	424.67	258	279	278	15	36	0	0	2
13746	7714	4612	6	1048	760	27980	601	292	953	129	98	231	122	173	275.39	234	438.94	492	356	457	31	88	24	2	2
6572	11702	8386	403	932	572	28226	995	20	756	3	58	73	13	123	287.02	111	443.13	403	411	362	34	43	2	4	2
11712	7806	5276	0	1918	1464	28526	517	131	594	28	316	167	66	285	312.7	111	458.49	497	200	387	81	74	4	30	19
13578	7714	4546	1	1180	930	28084	608	246	909	98	130	217	104	194	277.66	213	443.92	500	343	437	31	97	24	4	2
13772	7854	4612	12	940	700	27952	650	266	1020	124	66	235	108	171	274.52	239	440.33	460	396	432	30	93	10	8	3
13026	7922	4756	1	1276	1028	28174	581	215	870	76	152	200	103	221	283.77	189	448.46	473	302	456	49	96	19	14	4
5074	12774	9248	668	636	382	28152	1139	3	679	0	34	71	0	100	279.89	93	438.23	355	358	308	19	46	0	4	1
4524	13280	9612	805	428	232	28084	1220	6	654	0	8	51	3	79	277.3	91	432.81	309	342	320	17	35	1	0	0
7284	11300	7960	356	974	642	28236	947	33	776	7	72	88	19	126	287.84	141	444.96	433	376	358	30	54	0	4	6

SUPPLEMENTARY INFORMATION:Monte Carlo Atomistic Simulation and Machine Learning Analysis of Na-K Eutectic Alloy in Condensed Phases, D. Reitz and E. Blaisten-Barojas, George Mason University, Fairfax, VA 22030

13638	7808	4602	4	1070	804	28012	636	257	976	121	86	227	101	178	276.78	237	443.23	490	385	418	26	86	13	4	5
5532	12528	9014	581	658	384	28142	1071	12	736	2	24	74	7	104	281.41	113	437.46	394	377	335	15	50	0	2	0
12294	7758	4966	1	1746	1364	28400	582	157	682	46	244	174	86	260	295.69	147	451.42	493	259	400	60	91	3	28	7
4482	13234	9602	793	500	278	28106	1185	3	599	0	10	61	2	81	276.51	84	432.86	329	296	320	18	41	0	0	3
4824	12958	9404	703	574	338	28134	1180	4	658	0	36	59	4	99	280.29	91	437.33	318	364	320	19	40	0	0	2
4222	13446	9768	863	398	224	28074	1222	5	587	0	16	66	4	99	274.69	88	428.5	289	284	306	12	42	0	0	1
14060	7638	4456	2	992	730	27960	631	299	966	138	82	245	116	193	275.97	226	441.44	460	384	430	24	93	24	2	2
11010	7804	5592	1	2262	1628	28688	516	90	516	12	360	153	50	276	318.91	95	465.87	489	169	384	114	71	1	32	27
4892	12980	9396	715	530	294	28110	1160	5	671	0	18	60	4	83	277.59	95	433.27	350	359	326	16	37	0	0	1
5184	12530	9122	616	838	498	28210	1122	12	624	0	30	53	9	109	282.42	72	438.2	364	362	319	23	37	0	8	2
4150	13506	9790	899	396	226	28074	1261	8	574	2	6	57	4	66	271.98	92	425.24	315	278	279	12	36	0	0	2
4510	13114	9536	777	582	362	28146	1194	7	597	1	40	60	4	89	276.28	86	432.36	338	309	296	18	37	0	2	1
6762	11678	8326	423	864	520	28202	964	26	744	6	46	74	17	116	288.01	116	442.44	417	365	385	27	41	0	6	8
10658	7840	5614	0	2320	1856	28824	497	54	486	10	476	147	18	306	323.65	97	463.53	475	138	368	130	79	4	58	25
12062	7862	5164	0	1796	1290	28396	597	139	709	36	204	161	76	239	297.27	153	454.29	473	258	427	65	86	8	16	13
4354	13354	9682	845	438	244	28078	1222	11	601	1	6	60	7	84	273.1	85	426.99	303	302	308	15	42	0	0	2
13138	7720	4800	2	1376	988	28178	591	206	847	65	142	189	108	209	285.45	184	449.19	486	310	440	54	92	12	14	4
4236	13414	9748	890	438	238	28078	1255	7	593	1	4	63	5	76	273.03	88	426.21	305	293	283	14	40	0	0	0
14336	7716	4452	5	756	538	27846	641	320	1109	161	46	262	114	145	267.39	276	429.29	496	455	433	11	97	15	2	3
3972	13648	9916	980	324	180	28050	1294	7	578	2	10	56	4	67	271.29	97	423.09	274	257	288	14	43	0	0	3
5268	12622	9106	579	726	430	28174	1086	8	686	0	22	69	6	121	279.98	102	436.16	375	370	325	13	45	0	0	2
4750	13176	9482	760	422	244	28086	1192	9	681	0	12	67	8	74	279.3	112	435.02	337	341	307	15	45	0	0	3
4554	13258	9568	801	432	260	28090	1223	5	632	0	16	68	3	80	278.32	85	433.95	310	337	290	21	37	0	2	1
14322	7680	4430	8	802	578	27868	613	319	1067	154	54	258	132	161	271.69	265	435.69	472	424	466	21	93	16	2	3
11142	7750	5478	1	2228	1674	28682	511	98	502	22	342	189	42	278	320.54	106	463.47	500	160	340	102	80	7	64	33
4606	13268	9568	822	392	226	28074	1228	8	657	0	14	56	4	67	276.63	106	431.13	325	340	302	12	39	0	0	1
14348	7650	4430	8	806	572	27864	635	328	1048	157	56	259	132	147	271.18	262	434.38	486	428	450	19	92	21	2	1
11126	7954	5594	2	2054	1514	28614	520	80	623	20	320	164	39	279	313.96	123	460.88	486	190	376	102	100	2	46	25
4770	13138	9484	744	448	242	28090	1192	6	676	1	8	67	3	99	277.51	93	432.01	307	360	316	13	38	0	0	2
13848	7770	4534	5	964	762	27988	621	273	1005	135	94	238	109	191	274.37	230	438.52	473	403	445	25	85	13	14	0
13576	7610	4518	2	1270	990	28114	597	264	838	106	140	220	123	217	279.55	188	444.99	457	299	453	38	87	18	8	8
13000	7862	4736	7	1338	1078	28192	576	205	821	82	166	203	91	231	282.2	195	446.06	496	303	426	42	87	9	12	3
3956	13688	9922	965	306	170	28044	1274	7	583	1	2	58	3	67	272.24	96	425.09	282	263	298	14	39	0	0	3
11326	7858	5390	1	2058	1602	28638	491	96	580	25	360	190	42	285	326.19	105	469.81	445	167	383	125	104	7	42	28
14242	7700	4420	3	834	630	27892	643	316	1053	164	60	266	116	155	269.22	255	432.02	493	441	421	17	85	18	6	1
4622	12986	9488	732	658	370	28148	1181	8	600	2	22	53	4	86	278.41	83	434.4	355	325	296	16	39	0	2	2

SUPPLEMENTARY INFORMATION:Monte Carlo Atomistic Simulation and Machine Learning Analysis of Na-K Eutectic Alloy in Condensed Phases, D. Reitz and E. Blaisten-Barojas, George Mason University, Fairfax, VA 22030

4362	13328	9660	851	464	270	28098	1248	9	598	2	14	58	6	74	274.18	85	427.62	304	310	291	19	41	0	0	2
13506	7842	4638	5	1084	850	28040	636	258	940	112	112	213	106	189	277.66	211	443.01	468	383	442	33	78	19	8	3
4488	13250	9620	814	460	256	28092	1228	4	615	2	18	63	1	74	275.37	90	430.68	314	313	295	18	48	0	0	3
14470	7668	4324	3	774	582	27862	672	305	1093	168	42	262	104	146	268.43	273	430.95	476	448	420	16	86	15	2	2
14364	7678	4368	4	806	604	27870	627	329	1054	165	44	253	121	158	269.51	252	432.2	479	428	454	18	83	19	6	2
3994	13652	9896	962	324	182	28052	1293	4	580	0	4	64	3	79	271.74	80	425.29	274	271	273	11	46	0	0	1
4854	12996	9388	719	548	318	28122	1150	3	668	0	18	55	1	86	278.34	107	435.44	336	339	343	24	41	0	0	2
6526	11842	8468	440	794	486	28176	945	35	760	10	54	78	18	121	287.55	128	442.63	437	359	382	22	47	1	6	1
11566	7890	5318	1	1978	1478	28542	529	98	598	11	282	143	55	259	309.89	137	456.95	518	187	395	92	81	9	26	14
13394	7696	4656	5	1272	950	28104	637	234	879	98	124	222	100	201	277.46	179	444.46	451	353	424	48	100	18	12	4
10248	7972	5790	0	2488	1894	28866	492	51	484	8	410	165	23	304	332.78	101	472.55	499	141	322	133	86	3	54	31
5406	12582	9064	635	662	400	28150	1103	14	666	1	36	66	11	107	280.1	83	436.94	355	343	343	14	45	0	0	6
4326	13422	9714	867	378	216	28068	1225	5	626	1	12	70	2	72	274.46	97	427	316	292	294	15	49	0	0	3
6350	11852	8486	454	926	566	28226	989	31	721	8	44	68	16	133	287.44	120	444.04	415	368	355	25	40	2	2	1
12084	7878	5212	2	1714	1252	28400	546	132	708	24	230	163	77	250	302.08	148	453.77	480	238	439	74	95	10	30	12
4542	13200	9566	809	476	290	28108	1226	5	596	0	34	61	4	76	277.02	72	432.4	322	323	289	17	43	0	0	3
4892	13026	9380	691	500	296	28114	1140	5	670	0	20	65	4	83	278.19	104	433.24	357	350	331	16	38	0	0	4
5080	12864	9226	664	588	364	28140	1140	10	689	2	18	58	3	94	279.54	110	436.56	369	356	319	12	42	0	0	1
4740	13080	9458	766	514	298	28108	1190	10	650	0	18	58	6	83	278.77	104	435.57	340	326	298	17	42	0	0	1
4618	13084	9500	775	574	334	28132	1208	4	603	0	20	56	2	78	277.24	82	433.26	337	312	294	21	37	0	2	0
6344	11970	8590	498	784	442	28162	1047	28	731	8	30	88	17	113	287.37	115	442.72	393	382	326	24	51	0	2	0
4038	13516	9826	912	422	262	28090	1266	5	572	0	24	65	1	81	274.59	87	428.61	282	282	278	16	40	0	2	6
7614	11090	7786	360	994	658	28222	940	52	775	20	76	103	26	122	287.01	145	444.72	446	392	342	30	57	1	2	6
14382	7640	4406	11	800	574	27860	655	352	1030	163	58	249	138	137	270.32	252	436.26	474	437	448	27	76	27	0	1
5220	12844	9236	650	500	284	28106	1112	8	727	1	22	60	6	94	278.53	109	435.47	362	361	350	13	42	0	0	2
14674	7606	4280	3	706	520	27828	647	350	1106	169	38	248	143	135	267.96	286	429.3	483	463	459	19	71	18	4	0
10972	7794	5422	0	2312	1808	28754	538	60	482	10	410	171	31	303	323.44	83	465.93	467	154	331	107	88	5	36	31
4378	13408	9690	851	376	210	28068	1244	6	626	2	6	48	3	65	275.95	86	430.3	295	313	319	22	31	0	0	2
7196	11380	8006	354	936	632	28232	971	33	794	10	78	88	20	134	289.09	135	442.72	423	420	348	25	52	1	4	0
6104	12086	8640	494	800	508	28192	1002	22	702	5	54	92	13	113	284.77	127	439.69	434	352	328	23	59	1	0	0
4862	13104	9438	718	422	242	28088	1167	6	694	0	18	65	4	96	278.51	100	432.89	328	360	322	12	44	0	2	2
11628	8068	5332	0	1820	1358	28460	556	111	655	24	220	152	65	269	304.86	119	454.88	462	224	432	72	79	3	30	21
10600	7726	5664	1	2410	1902	28874	472	57	463	11	500	191	31	349	333.99	80	473.03	426	131	356	124	92	1	64	38
11380	7926	5332	2	2002	1588	28606	581	94	587	13	338	160	50	278	311.52	110	459.31	484	206	355	85	87	6	32	21
13792	7696	4512	3	1086	836	28038	580	280	893	115	110	225	125	206	276.46	226	443.84	472	306	474	31	92	20	6	3
4024	13632	9878	946	310	190	28054	1292	3	596	0	20	62	1	71	271.68	84	424.27	275	296	278	14	40	0	0	3

4390	13306	9634	825	492	282	28110	1242	2	612	0	6	53	1	79	275.75	90	432.36	315	319	289	14	39	0	0	2
4668	13148	9518	746	486	270	28104	1182	1	658	0	14	53	0	82	277.4	90	432.86	323	345	333	17	33	0	0	5
4692	13136	9506	755	490	270	28106	1161	7	650	1	12	63	4	72	277.02	113	432.46	358	319	324	20	41	0	0	0
4000	13624	9900	957	340	184	28054	1296	3	586	0	6	50	3	69	271.35	82	425.41	269	288	296	16	29	0	0	1
11246	7984	5396	1	2008	1588	28604	550	89	610	21	354	170	40	305	315.48	104	461.81	443	200	367	94	88	6	26	23
7680	11048	7808	295	996	620	28228	879	34	837	8	68	105	17	138	287.22	147	447.26	448	412	372	39	67	1	8	4
5142	12802	9258	641	594	316	28118	1115	6	697	0	6	71	5	94	276.19	101	433.28	360	365	335	17	46	0	0	3
14236	7686	4408	5	874	648	27910	658	300	1048	153	52	260	118	141	272.98	267	436.83	508	430	400	21	98	8	6	2
4836	13076	9426	698	476	272	28100	1139	4	680	1	14	70	1	90	278.11	107	432.67	343	345	329	21	45	0	0	0
3832	13754	9982	1013	296	174	28048	1329	4	548	1	8	59	3	68	271.89	88	424.12	263	262	259	13	41	0	2	2
12394	8090	5040	0	1458	1114	28266	594	153	848	35	152	164	95	223	287	191	448.15	495	323	453	46	89	6	18	5
14196	7588	4350	1	974	752	27946	660	311	975	156	80	264	118	165	271.62	229	436.87	485	413	399	22	89	21	6	1
4788	13082	9456	754	486	270	28094	1153	8	652	0	12	62	5	75	275.73	104	433.12	353	311	335	17	44	0	0	2
4092	13476	9816	911	438	250	28090	1259	3	558	0	18	60	1	75	275.53	89	430.21	298	262	287	14	39	0	0	4
11692	7974	5292	2	1860	1382	28468	588	110	672	26	248	159	56	256	299.72	130	452.32	486	246	389	77	83	10	14	10
6288	11928	8552	428	878	518	28198	968	21	734	2	34	69	17	113	284.9	130	443.64	429	358	377	27	47	0	0	4
14380	7736	4408	7	746	540	27844	652	321	1085	166	32	258	119	152	267.15	269	428.65	466	436	449	18	81	15	2	0
11606	8012	5284	1	1826	1464	28532	570	94	637	15	288	164	52	250	314.18	116	458.81	481	217	389	93	95	5	44	16
4238	13480	9782	888	354	192	28058	1266	7	601	1	12	57	4	73	272.43	88	426.32	285	307	299	12	34	0	0	4
11524	7952	5364	1	1924	1444	28518	537	114	640	33	274	165	55	274	310.51	116	457.05	468	205	398	93	78	6	32	19
11462	8070	5378	2	1862	1450	28556	512	105	632	16	298	156	59	262	314.56	125	461.33	516	201	406	88	88	12	34	21
13732	7598	4448	3	1198	956	28074	595	274	849	122	128	239	116	209	277.05	201	446.07	466	315	439	38	92	17	14	5
4592	13098	9558	765	552	294	28112	1181	4	633	0	16	74	2	95	277.02	94	433.78	327	320	298	17	53	0	2	2
4316	13348	9700	868	466	254	28090	1246	3	606	0	6	59	2	74	273.66	92	429.6	311	290	288	16	41	0	0	1
4756	13078	9460	751	496	288	28100	1195	10	656	1	22	63	6	80	275.42	94	429.56	324	346	307	19	42	0	0	4
13848	7768	4612	6	968	684	27956	609	273	1011	129	74	242	104	167	275.87	241	440.68	488	382	451	27	100	18	0	3
4852	13114	9430	716	424	248	28084	1171	6	689	1	16	73	4	84	277.86	95	432.55	334	365	313	17	43	0	0	2
10788	7846	5664	0	2258	1714	28748	498	60	512	12	440	176	30	307	329.11	84	468.18	450	163	356	128	87	5	32	34
12534	7942	4934	4	1476	1186	28300	597	154	815	45	202	183	73	240	290.97	165	449.2	468	291	422	58	102	14	24	8
14506	7556	4286	7	834	632	27884	639	322	1053	160	70	261	126	151	271.73	259	435.5	492	427	428	20	87	15	0	1
4416	13364	9676	872	384	212	28062	1258	12	618	1	10	58	7	81	272.97	81	427.78	283	321	295	15	44	0	0	2
13564	7930	4712	5	978	722	27992	641	252	1002	121	82	243	99	179	275.01	224	438.75	475	394	424	27	98	17	4	3
3982	13554	9878	939	420	234	28078	1301	8	581	2	10	52	4	63	271.42	94	423.88	293	283	269	13	31	0	0	3
11420	8166	5388	2	1784	1418	28494	527	86	724	13	290	184	47	247	306.5	152	458.43	499	228	389	93	114	2	26	20
3964	13592	9902	941	384	214	28068	1289	5	571	1	10	60	3	70	270.35	87	424.16	280	276	280	15	36	0	2	3
6434	11878	8484	483	814	512	28182	1012	26	702	9	56	85	14	131	286.04	101	441.59	390	363	353	21	51	0	4	2

3952	13650	9894	961	346	212	28068	1290	7	577	2	12	55	4	71	269.8	96	422.56	289	272	279	10	39	0	2	2
13914	7774	4504	1	960	734	27966	641	302	1008	129	76	227	129	170	274.97	230	439.54	478	422	437	28	83	23	4	2
4106	13554	9844	900	358	192	28060	1249	4	582	1	6	61	2	71	270.83	85	425.32	301	296	297	13	39	0	0	3
5308	12762	9170	621	542	310	28110	1110	7	723	2	18	71	4	95	281	117	436.97	357	380	341	16	46	0	0	3
3964	13628	9886	948	364	216	28068	1277	6	559	1	10	59	3	65	272.56	85	425.4	293	270	281	14	37	0	0	5
3668	13822	10060	1022	316	180	28052	1335	10	523	1	6	57	6	63	271.41	91	423.21	268	249	256	14	31	0	0	1
12538	7850	5050	0	1530	1106	28280	584	168	774	39	194	183	95	222	293.41	177	450.92	481	278	418	63	105	5	10	13
4212	13418	9766	853	440	240	28088	1228	1	594	0	12	70	0	81	274.85	107	429.69	317	285	283	10	42	0	0	5
14144	7644	4416	2	946	704	27924	628	307	1010	168	68	254	109	167	270.22	257	433.19	493	408	434	15	76	11	2	3
5572	12418	8982	573	722	428	28168	1047	12	660	4	40	71	6	96	283.91	103	437.45	410	327	347	20	46	2	6	2
6772	11654	8294	404	892	544	28204	969	21	773	4	44	81	11	132	288.1	126	443.73	418	397	357	26	52	0	4	2
12300	7856	5062	3	1650	1238	28342	554	140	775	46	220	195	66	269	293.08	156	450.02	458	260	421	65	108	7	16	11
4202	13512	9800	917	350	184	28050	1257	8	597	1	2	62	4	70	272.11	94	425.49	290	283	297	13	45	0	0	5
13176	7736	4744	2	1332	1008	28166	617	201	865	84	154	198	87	197	282.16	168	445.33	479	361	434	52	84	12	16	4
3950	13642	9918	940	352	190	28054	1282	7	572	1	2	60	5	66	272.34	84	424.74	293	270	280	12	44	0	0	2
3898	13712	9972	980	290	156	28038	1304	6	578	1	10	69	4	72	271.14	83	423.48	263	279	271	16	47	0	0	1
6660	11648	8304	432	946	598	28214	984	29	727	5	58	81	18	130	286.86	115	444.32	401	368	363	29	53	0	0	4
5186	12764	9178	645	626	372	28142	1135	8	680	2	16	73	4	90	279.88	122	436.45	363	338	314	20	47	0	0	2
4150	13454	9776	899	434	256	28088	1256	8	595	1	18	56	4	68	272.24	92	425.31	311	287	288	15	41	0	0	2
4946	12978	9370	730	492	286	28098	1159	8	690	1	26	72	6	85	278.72	111	434.01	346	335	312	17	46	0	0	2
13608	7602	4470	0	1242	1022	28120	586	245	848	93	158	216	113	223	283.17	183	447.65	476	333	451	30	98	18	16	2
4024	13600	9876	931	348	200	28062	1279	5	578	2	14	57	3	74	271.96	90	425.25	294	285	280	8	38	0	0	2
6418	11920	8490	470	782	504	28174	1000	28	744	8	52	72	15	135	285.32	113	440.81	380	371	377	23	47	1	8	1
11076	8018	5510	2	2062	1586	28624	555	84	580	17	330	174	38	279	311.15	82	461.25	449	202	376	114	98	7	36	16
14526	7598	4324	3	778	576	27862	604	339	1060	159	58	257	139	143	271.16	274	432.99	519	413	456	15	90	16	0	0
13700	7706	4528	2	1118	872	28046	614	251	937	98	114	218	118	192	277.78	215	443.02	473	350	453	32	103	16	6	3
4462	13268	9606	809	476	276	28098	1225	7	613	0	6	59	3	69	275.78	107	430.55	331	325	286	18	36	0	2	1
13130	7800	4806	2	1328	952	28148	606	200	865	79	126	191	89	203	283.68	185	446.66	493	323	448	38	93	12	6	3
14326	7670	4438	9	794	574	27864	612	344	1064	171	56	265	136	153	272.14	259	438.71	479	421	459	25	82	20	6	2
3924	13688	9944	984	304	172	28044	1303	10	569	1	12	54	6	71	271.17	80	422.6	260	269	288	16	39	0	0	2
4650	13142	9516	759	476	286	28102	1168	5	619	0	32	62	3	80	279.49	85	433.82	337	325	319	18	37	0	0	6
4764	13162	9484	757	416	236	28074	1187	7	698	0	12	74	4	89	275.98	102	434.16	314	356	309	18	54	0	0	2
4648	13184	9548	771	448	244	28086	1140	5	624	2	14	60	2	83	276.92	101	431.16	347	293	353	15	46	0	0	1
12120	7872	5048	1	1754	1354	28402	564	141	705	31	238	188	74	245	292.73	159	452.08	504	241	394	65	109	16	16	15
11852	8086	5200	1	1720	1326	28424	596	119	718	35	210	169	62	238	298.79	148	451.41	512	252	391	52	97	7	30	15
4676	13132	9496	788	496	288	28106	1200	4	642	0	18	57	3	83	276.99	85	434.26	321	332	317	17	43	0	0	2

12960	7880	4836	3	1322	1014	28180	595	195	864	76	154	197	97	201	283	187	446.3	505	319	447	42	91	10	12	4
7236	11408	7978	358	900	622	28222	924	37	782	13	76	97	15	142	287.32	138	444.54	428	383	366	30	61	3	2	3
11278	8072	5550	1	1932	1398	28532	529	90	632	19	266	170	46	276	312.2	125	456.98	520	221	369	85	95	3	36	10
4556	13214	9586	791	468	258	28098	1179	3	632	0	14	70	2	78	275.75	114	431.93	345	298	306	16	46	0	2	2
4214	13502	9784	911	354	196	28058	1278	10	617	2	8	61	5	65	273.46	91	427.37	291	311	282	16	42	0	0	2
14362	7642	4370	7	812	622	27882	635	342	1042	162	68	274	139	152	271.85	248	435.62	493	433	412	21	100	19	4	3
13562	7660	4530	1	1254	966	28098	633	222	886	93	112	216	100	215	276.79	180	445.13	439	342	440	42	102	11	14	3
4504	13316	9590	829	404	252	28084	1238	4	650	0	18	58	2	81	274.79	103	431.1	301	321	300	16	38	0	0	1
6784	11492	8248	352	1036	628	28250	914	23	747	3	58	82	17	142	286.35	124	444.61	452	386	380	20	49	1	4	4
5338	12582	9092	601	710	420	28178	1106	6	708	1	36	77	4	100	282.26	106	437.23	392	380	299	16	51	0	0	3
11732	8104	5190	2	1748	1398	28434	531	119	698	29	230	171	66	259	298.47	138	454.24	488	228	428	70	94	6	30	19
12790	7982	4858	4	1348	1060	28200	606	197	828	65	148	182	93	220	283.58	167	445.75	475	317	450	43	92	14	14	6
14614	7624	4314	6	704	518	27818	638	346	1109	174	40	279	129	150	270.37	257	433.1	478	456	431	17	92	17	4	0
14346	7662	4384	7	802	612	27878	642	320	1074	163	68	253	117	154	269.26	268	430.94	483	431	444	17	85	19	4	1
4930	13118	9436	730	372	198	28066	1176	8	714	2	12	66	5	85	276.24	110	431.13	326	370	327	15	48	0	0	0
5514	12472	8978	596	732	442	28168	1102	13	701	2	28	63	7	92	282.71	109	436.51	400	362	315	15	42	0	0	3
4304	13418	9726	872	398	218	28070	1280	6	623	0	6	62	3	69	272.4	91	425.16	295	327	272	12	40	0	0	4
13844	7718	4554	7	1016	754	27980	635	266	969	119	84	231	115	181	275.58	217	440.31	461	383	446	32	100	15	10	2
4126	13514	9834	891	386	204	28074	1239	5	594	0	10	54	4	65	277.67	93	431.85	323	288	303	11	38	0	0	2
11552	7930	5288	1	1924	1514	28568	525	107	608	26	334	186	59	269	312.1	123	460.38	499	194	375	96	98	5	26	19
11744	7988	5240	1	1814	1402	28470	563	110	680	27	234	165	65	249	299.73	138	453.19	492	230	423	79	93	7	44	10
4998	12892	9300	680	582	344	28140	1128	5	665	0	24	53	5	86	277.7	120	433.8	369	335	341	18	30	0	0	1
12454	7894	5060	1	1556	1128	28284	594	167	760	49	176	192	83	245	292.53	164	449.52	462	280	420	56	92	12	14	8
7470	11230	7890	343	936	618	28226	904	43	802	8	78	80	28	145	287.58	148	444.71	448	382	387	24	52	2	4	0
11596	7828	5278	0	2036	1508	28554	551	99	594	19	286	168	48	264	310.85	126	457.39	511	193	370	88	92	9	20	15
5120	12742	9224	636	648	380	28144	1105	8	660	2	30	67	3	99	278.45	101	435.53	371	346	328	19	39	0	0	2
4358	13276	9690	820	488	262	28092	1203	10	603	2	18	64	5	74	276.04	92	430.5	334	299	298	16	45	0	0	3
5380	12726	9144	592	548	304	28118	1081	15	740	0	16	66	9	95	281.27	113	438.38	377	392	355	17	46	0	0	2
13818	7762	4566	8	978	748	27976	605	290	990	128	96	246	130	177	275.28	229	441.38	485	380	450	28	97	18	8	2
10870	7876	5674	2	2244	1622	28684	526	81	537	12	358	153	38	292	324	103	463.75	484	169	363	108	84	3	36	21
3904	13630	9902	952	392	238	28080	1305	3	555	1	12	63	2	70	272.07	89	425.04	273	274	266	19	38	0	2	2
4558	13290	9560	796	402	256	28088	1197	13	637	0	22	54	8	66	275.68	110	429.94	344	318	310	16	36	0	0	3
4338	13388	9696	857	414	234	28078	1241	6	626	0	6	50	4	67	272.88	100	427.28	307	310	310	18	32	0	2	2
3820	13760	10006	995	286	158	28040	1301	6	554	2	10	56	3	68	270.27	84	422.57	271	262	285	10	41	0	0	4
11778	7988	5194	1	1812	1428	28502	551	112	658	32	272	185	53	254	305.46	130	458.08	480	231	392	94	84	6	26	17
11686	7902	5244	2	1888	1468	28512	612	113	618	19	290	161	62	245	299.36	101	456.33	451	224	389	91	98	11	28	21

SUPPLEMENTARY INFORMATION:Monte Carlo Atomistic Simulation and Machine Learning Analysis of Na-K Eutectic Alloy in Condensed Phases, D. Reitz and E. Blaisten-Barojas, George Mason University, Fairfax, VA 22030

4978	12830	9294	715	614	380	28140	1152	15	637	1	40	72	11	88	281.54	95	437.33	349	328	304	27	41	0	4	1
4718	13106	9492	754	492	278	28108	1166	8	639	0	22	50	6	83	277.2	106	431.85	348	314	331	14	28	0	0	1
5092	12892	9258	665	540	320	28116	1117	3	698	0	14	62	3	84	279.34	113	434.89	377	347	336	16	38	0	0	1
4422	13332	9660	837	418	234	28078	1242	7	636	0	10	54	5	70	274.03	90	429.29	321	331	290	13	38	0	2	1
4966	12834	9318	678	632	362	28140	1149	6	652	0	28	59	1	96	277.41	97	434.15	356	341	310	15	35	0	0	3
4716	13138	9472	767	478	284	28104	1171	7	633	2	14	66	4	81	276.34	91	432.11	330	308	323	21	45	0	2	4
4970	12916	9338	717	546	320	28118	1160	9	662	3	24	54	5	87	278.98	105	434.1	341	337	328	18	34	0	4	3
4184	13444	9754	884	426	260	28090	1245	6	584	0	20	54	5	80	274.75	82	430.22	291	284	304	18	39	0	2	3
11952	7986	5224	0	1720	1270	28388	575	111	751	27	218	185	60	234	300.76	156	451.7	488	260	404	75	113	9	18	12
5180	12900	9256	655	472	270	28092	1132	12	720	1	12	67	7	89	280.59	99	436.05	344	399	341	20	42	0	2	1
3812	13770	9984	1007	294	178	28048	1310	8	553	1	10	62	6	65	270.86	98	424.18	280	253	265	10	42	0	0	3
14140	7720	4384	6	910	710	27926	682	306	995	169	58	265	100	172	270.71	240	434.38	461	432	394	17	86	20	4	3
3918	13618	9904	944	404	230	28080	1286	4	546	1	6	58	3	74	272.61	79	426.41	286	261	278	11	38	0	0	4
4286	13466	9726	870	372	214	28068	1241	8	606	2	4	64	6	81	273.29	92	426.69	297	306	301	12	38	0	0	2
5276	12708	9154	653	596	362	28132	1116	10	662	3	34	65	3	94	281.64	98	438.29	351	354	340	24	36	0	2	3
4716	13082	9490	749	524	286	28112	1155	3	631	0	12	59	1	79	278.14	103	434.11	350	308	326	20	38	0	2	3
5266	12654	9108	598	708	420	28172	1134	7	682	0	14	54	6	90	282.1	93	437.86	383	386	317	12	32	0	2	4
14424	7536	4350	5	890	632	27894	661	321	1037	172	56	266	118	144	269.8	273	433.31	490	435	412	20	85	16	6	3
4190	13434	9784	887	434	228	28074	1258	6	585	1	4	57	3	65	273.06	75	427.34	307	294	290	16	42	0	0	2
11244	7906	5530	1	2108	1498	28606	529	83	577	22	272	166	42	291	314.95	109	462.47	470	177	389	94	83	4	46	21
13956	7674	4542	6	990	710	27960	661	305	974	147	84	247	118	160	275.13	228	438.71	464	395	423	31	90	19	4	6
12658	7804	4936	3	1548	1136	28268	616	181	773	59	172	192	89	206	288.48	159	450.75	505	308	401	57	101	10	10	5
4142	13532	9784	909	374	230	28074	1273	8	589	1	10	54	3	68	271.19	88	424.12	301	301	285	11	32	0	2	3
12066	7734	5094	0	1860	1418	28482	547	127	687	28	282	156	64	238	301.97	145	455.08	508	246	410	86	79	7	28	14
4438	13296	9600	834	458	288	28102	1225	8	608	2	22	58	3	76	275.48	99	428.67	321	301	300	15	34	1	0	1
4634	13196	9534	746	452	262	28096	1188	5	667	0	18	65	3	67	274.92	109	429.12	348	353	306	19	43	0	0	2
4034	13642	9876	955	310	180	28050	1302	8	601	1	8	60	6	66	271.27	95	423.04	273	288	276	16	44	0	0	2
5192	12722	9168	635	638	394	28150	1102	11	667	1	36	53	5	93	279.6	102	435.8	390	357	332	15	35	0	0	2
3904	13636	9946	988	364	196	28056	1304	10	554	1	10	61	5	75	272.45	88	425.23	276	251	265	10	49	0	0	2
4994	12854	9312	696	612	342	28130	1156	3	688	0	16	62	2	104	278.76	107	436.02	338	346	315	14	43	0	0	2
13428	7678	4628	2	1262	960	28098	579	248	862	110	124	227	97	217	276.61	177	445.03	466	329	440	48	95	13	18	3
4446	13334	9654	847	400	222	28066	1223	8	625	0	10	55	5	71	274.19	88	429.17	303	308	320	19	37	0	0	3
14428	7730	4358	7	714	564	27852	639	328	1091	160	52	270	123	138	269.3	290	432.13	503	435	426	15	96	18	6	3
12360	8004	5006	1	1536	1202	28318	613	138	785	44	190	165	67	222	286.01	178	449.44	487	277	427	58	92	8	18	8
4414	13236	9612	845	516	310	28114	1234	3	594	0	26	50	2	72	274.55	98	429.66	315	288	303	18	34	0	0	4
4346	13462	9712	878	328	194	28054	1257	10	619	2	12	70	5	83	274.18	90	427.4	288	314	283	11	51	0	0	2

SUPPLEMENTARY INFORMATION:Monte Carlo Atomistic Simulation and Machine Learning Analysis of Na-K Eutectic Alloy in Condensed Phases, D. Reitz and E. Blaisten-Barojas, George Mason University, Fairfax, VA 22030

14364	7692	4432	6	772	548	27856	624	320	1099	165	44	256	126	149	269.57	267	431.85	481	439	460	22	75	14	4	1
5444	12704	9082	617	542	328	28124	1101	7	699	0	22	54	3	95	281.24	112	438.28	360	366	359	20	33	0	2	2
12404	7924	5018	6	1554	1178	28278	615	165	785	54	182	189	87	233	286.24	173	447.09	475	297	408	55	94	5	16	5
4498	13292	9620	850	418	236	28078	1263	8	639	2	14	60	3	79	274.48	91	429.25	289	333	287	15	36	0	0	2
11944	7956	5142	2	1726	1384	28460	549	115	703	33	274	174	54	255	307.5	145	457.81	487	227	399	89	96	6	30	9
6686	11614	8312	374	972	594	28238	964	19	784	3	60	63	10	123	287.03	122	444.31	448	422	359	23	32	0	0	3
13398	7908	4808	6	1054	750	28020	623	242	972	115	96	227	102	168	276.02	229	442.76	494	372	443	36	94	10	6	1
5896	12268	8716	496	754	514	28204	1038	9	722	1	52	74	4	114	284.64	114	440.34	398	374	339	27	50	0	4	1
11604	7932	5352	1	1936	1406	28504	548	98	636	18	234	155	58	297	309.8	121	457.59	465	220	405	83	90	6	38	7
13820	7820	4550	8	954	740	27976	671	290	1000	127	90	215	131	159	273.98	242	438.97	468	424	450	29	76	14	2	1
12362	7998	4986	1	1530	1224	28316	584	147	780	47	178	187	77	233	291.88	161	451.12	466	292	425	67	97	6	32	13
11780	7904	5304	1	1836	1354	28470	527	122	664	29	264	207	58	283	311.06	129	459.7	458	200	384	85	124	10	26	17
12598	7922	4890	2	1494	1168	28260	630	173	767	61	176	180	82	200	287.16	164	447.89	479	282	414	63	85	11	12	9
5832	12346	8836	561	684	416	28146	1069	19	700	3	28	69	11	110	285.54	107	441.9	370	369	350	20	45	0	4	3
11040	8096	5538	3	1980	1578	28644	495	79	592	22	370	192	44	309	316.53	99	459.56	463	210	393	94	102	1	38	26
7362	11312	7916	366	896	632	28212	904	39	740	12	88	88	18	138	288.58	127	444.01	424	358	388	36	51	0	6	4
4970	12954	9332	708	540	310	28118	1166	14	656	0	12	54	9	91	278.63	93	434.42	340	352	321	17	36	1	0	1
4820	13058	9436	735	498	278	28104	1164	5	675	0	14	58	3	82	277.55	117	432.07	357	325	321	10	46	0	0	2
14246	7762	4432	4	786	594	27872	637	314	1073	159	50	254	119	154	269.8	273	433.86	487	438	442	15	79	14	2	1
14384	7636	4390	12	794	594	27872	665	339	1042	170	70	265	123	152	272.78	270	437.73	464	416	422	22	85	27	4	3
12898	7946	4922	7	1322	946	28152	613	197	864	69	98	201	98	190	283.35	178	448.63	493	323	428	49	104	9	18	6
4022	13536	9854	917	412	238	28078	1274	6	570	1	16	58	5	77	271.89	81	425.08	281	290	290	17	40	0	0	1
12456	8018	5060	0	1466	1084	28256	596	160	811	40	156	176	77	232	287.56	175	449.59	499	291	425	43	102	11	14	2
4876	13144	9422	711	400	232	28082	1140	1	690	0	8	62	0	73	277.18	120	432.98	358	346	346	15	42	0	0	3
14178	7662	4378	10	922	714	27930	593	328	977	160	74	258	126	151	272.23	260	435.43	528	366	440	22	82	25	2	2
13018	7612	4682	3	1516	1198	28246	585	194	813	73	202	201	87	215	282.77	184	450.34	504	308	419	58	92	8	16	2
13376	7718	4686	4	1230	940	28116	590	231	847	72	160	198	126	198	283.79	185	446.12	476	314	467	50	96	14	6	3
13488	7650	4616	5	1274	950	28120	622	226	877	63	130	206	118	201	283.9	196	447.74	473	318	437	43	119	22	12	3
12386	7882	5050	3	1574	1192	28318	552	152	775	46	214	184	77	233	294.53	189	453.01	499	256	426	67	100	6	20	8
13818	7652	4504	4	1090	840	28026	642	274	955	130	110	245	112	191	276.84	219	441.04	460	374	416	31	100	22	12	6
4574	13162	9554	753	502	292	28112	1162	10	617	1	28	56	2	85	278.57	85	435.36	347	321	315	20	33	0	0	1
5948	12248	8754	549	716	450	28160	1054	18	736	2	42	64	13	128	286.5	108	439.54	367	377	361	15	39	0	2	2
14172	7734	4424	8	862	658	27914	622	304	1032	151	60	238	120	138	269.71	288	432.55	525	394	453	19	79	21	4	0
4116	13476	9790	882	434	258	28092	1244	4	579	1	18	63	3	73	272.12	95	425.17	311	286	286	17	42	0	0	2
4008	13574	9860	953	392	228	28072	1326	8	583	1	10	62	4	75	271.43	78	424.14	262	304	255	13	45	0	0	3
11290	7910	5430	2	2080	1550	28602	512	93	576	20	310	168	46	291	320.21	113	463.78	507	169	368	87	91	5	28	16

SUPPLEMENTARY INFORMATION:Monte Carlo Atomistic Simulation and Machine Learning Analysis of Na-K Eutectic Alloy in Condensed Phases, D. Reitz and E. Blaisten-Barojas, George Mason University, Fairfax, VA 22030

4980	12932	9322	741	540	322	28120	1186	9	656	1	22	62	4	87	280.71	99	436.3	322	334	310	25	35	0	2	1
4980	13066	9382	695	408	232	28084	1140	9	713	1	16	66	7	66	277.62	133	433.69	374	363	331	16	46	0	0	2
13354	7852	4684	2	1170	904	28090	634	236	910	90	118	196	116	185	282	204	445.66	466	358	449	45	82	13	4	5
4748	13098	9450	774	504	290	28096	1174	9	637	2	4	63	5	84	275.54	103	430.2	333	309	319	18	45	0	2	2
5456	12546	8996	609	704	436	28164	1109	14	674	3	26	66	8	97	282.77	103	438.86	392	357	313	13	45	0	0	2
12008	7758	5172	1	1868	1362	28442	555	143	656	42	252	151	62	268	299.86	135	453.6	500	222	409	68	76	11	22	9
11322	8076	5352	3	1928	1552	28586	529	82	653	15	318	164	43	261	308.18	141	458.85	522	205	385	81	88	9	34	25
12044	7838	5184	3	1740	1320	28424	534	148	694	34	262	177	77	245	303.53	157	454.89	509	237	417	69	97	11	36	16
11910	8018	5230	1	1698	1292	28408	558	126	721	36	234	189	65	236	298.6	141	452.41	509	255	402	76	107	9	24	9
11674	7838	5204	1	1924	1548	28570	522	104	567	30	348	177	44	289	310.65	117	460.83	491	195	365	95	73	8	30	17
11860	8036	5274	2	1678	1278	28404	586	121	739	29	270	157	66	231	299.4	151	455.47	493	270	414	69	88	10	8	17
4404	13332	9690	860	416	216	28066	1256	10	627	1	8	56	5	79	273.03	95	426.49	292	311	291	15	51	0	0	1
5678	12536	8980	562	584	326	28120	1055	9	768	1	16	72	5	90	280.87	127	438.04	405	387	352	17	51	0	0	1
11752	7748	5168	2	2054	1530	28552	536	114	583	24	276	169	60	277	312.49	117	458.23	498	186	386	84	83	5	24	16
4776	13134	9464	754	442	254	28082	1184	8	662	0	12	63	4	73	276.38	93	431.62	326	349	324	24	38	0	0	1
11566	7904	5380	1	1944	1414	28508	535	96	631	23	276	171	56	254	307.83	118	459.65	491	228	402	101	94	6	24	11
4620	13252	9588	803	382	206	28064	1223	9	678	0	16	61	7	65	275.9	95	428.71	321	352	302	19	51	0	0	1
13846	7840	4578	3	882	702	27958	614	273	1041	125	104	243	110	168	276.55	260	439.04	493	393	442	27	108	17	6	1
11564	7900	5288	1	1970	1506	28558	529	119	555	27	304	179	56	265	314.51	118	461.98	493	166	381	103	89	8	24	15
4182	13528	9778	904	346	212	28060	1261	7	592	1	14	57	5	72	272.31	78	425	287	297	302	12	37	0	0	4
5382	12582	9078	619	680	402	28156	1085	12	711	1	32	62	10	105	279.95	124	438.16	373	331	351	20	42	0	0	0
14124	7648	4410	2	954	726	27940	626	320	954	155	68	248	131	170	271.71	227	437.14	463	371	450	33	77	17	8	2
4216	13564	9796	890	296	168	28048	1263	3	617	1	8	55	1	72	273.6	87	427.18	277	317	308	20	38	0	0	0
12092	8196	5202	7	1502	1132	28310	566	127	800	33	168	201	63	240	303.7	172	453.27	480	267	411	68	122	5	18	9
4154	13524	9812	914	362	200	28060	1269	8	598	2	8	58	4	68	273.8	100	427.25	285	289	293	18	37	0	0	3
3896	13694	9942	960	320	188	28052	1300	6	581	1	12	53	3	68	270.54	79	423.44	263	286	292	17	38	0	0	3
4650	13222	9528	794	428	246	28082	1199	6	647	1	8	56	4	85	273.76	92	428.47	312	326	329	14	42	0	0	2
4134	13512	9844	895	372	188	28056	1266	7	595	1	4	68	2	65	272.59	96	427.25	293	295	274	22	39	0	2	2
4888	12764	9338	682	746	410	28170	1133	8	607	0	24	64	4	92	283.12	90	437.68	376	315	305	19	44	0	0	3
4252	13378	9728	857	456	260	28090	1257	11	586	1	16	55	6	80	274	82	428.78	290	312	288	19	43	0	0	2
12242	7764	4974	0	1816	1380	28416	552	148	653	48	206	194	72	256	301.17	145	457.15	499	221	376	71	86	13	34	14
4744	13136	9492	751	454	250	28086	1182	8	675	0	6	62	3	87	278.46	106	435.51	327	337	316	15	39	1	4	2
13856	7726	4524	5	1042	760	27978	624	289	954	146	68	241	114	180	275.27	236	439.29	491	378	435	22	75	11	2	0
4720	13062	9456	778	542	320	28124	1188	10	637	0	22	68	9	88	277.43	98	433.18	327	312	305	20	41	0	2	2
11932	7904	5194	1	1742	1370	28470	579	118	701	23	296	163	62	239	302.64	140	454.54	496	256	397	82	89	12	32	14
5358	12682	9100	587	606	368	28142	1046	3	705	0	28	69	3	104	280.32	104	438.15	387	355	369	18	49	0	0	3

SUPPLEMENTARY INFORMATION:Monte Carlo Atomistic Simulation and Machine Learning Analysis of Na-K Eutectic Alloy in Condensed Phases, D. Reitz and E. Blaisten-Barojas, George Mason University, Fairfax, VA 22030

10992	7960	5588	2	2100	1606	28668	463	84	585	18	382	162	47	302	313.34	104	462.43	499	177	421	89	94	6	38	27
5650	12552	9006	600	566	318	28116	1053	13	753	6	22	74	7	90	280.53	128	436.97	396	350	365	15	50	0	2	3
10710	7954	5666	1	2268	1704	28718	505	65	526	12	366	150	31	310	315.6	91	461.9	484	154	362	118	82	2	46	15
13636	7626	4578	3	1210	896	28072	585	260	893	107	108	227	116	206	280.36	200	446.59	477	342	443	41	89	12	18	3
5372	12540	9018	591	732	476	28190	1071	11	652	2	52	61	6	102	282.29	98	438.96	405	346	334	14	38	0	0	4
14276	7760	4422	6	744	592	27868	623	318	1092	164	70	265	120	149	268.41	272	430.93	504	440	437	13	88	13	4	3
11192	7742	5456	2	2222	1658	28676	529	88	505	12	362	182	41	302	315.46	97	461.3	478	157	338	108	91	9	44	18
4248	13428	9750	870	412	228	28076	1232	5	616	1	10	67	3	68	271.42	99	424.28	314	287	298	19	50	0	0	1
12652	7960	4992	1	1432	1042	28236	628	177	819	58	144	174	87	207	285.06	175	448.03	499	331	420	46	84	9	14	5
12094	7814	5118	3	1718	1360	28432	561	151	687	34	292	185	81	266	299.03	135	454.74	475	255	402	65	95	14	36	20
13800	7788	4572	11	984	744	27980	638	275	1011	125	84	223	119	165	276.12	249	441.59	472	383	458	31	83	11	8	4
11298	7944	5434	0	2014	1530	28570	536	95	592	21	314	186	45	286	313.43	91	462.47	436	196	386	104	99	7	30	23
13652	7870	4642	7	992	748	27994	656	257	1002	114	82	217	105	166	277.09	231	441.04	467	406	443	34	85	17	8	3
14272	7620	4366	5	912	680	27912	634	312	1019	151	54	270	117	162	271.91	244	435.78	489	422	411	23	95	17	8	2
3814	13710	9978	1004	338	202	28060	1304	6	539	1	16	63	3	75	269.83	88	422.41	274	236	267	11	38	0	2	2
12282	7956	5034	2	1578	1248	28342	610	138	760	37	218	177	72	248	295.48	150	453.21	449	282	410	68	80	8	24	9
14204	7666	4398	3	888	688	27930	629	316	992	153	82	248	132	145	274.44	242	438.31	500	391	439	32	86	17	4	0
11550	8020	5388	4	1858	1380	28486	564	114	659	20	266	164	60	254	307.36	131	455.15	508	234	386	74	91	7	20	13
13246	7802	4664	4	1290	996	28130	585	215	852	91	124	216	95	201	279.84	215	445.58	516	294	427	36	95	10	8	3
5054	12904	9298	634	526	310	28118	1077	4	705	0	24	78	1	85	280.95	106	437.05	400	350	334	17	52	0	2	2
11400	7718	5460	1	2178	1518	28598	514	90	598	22	284	172	38	290	317.2	120	461.18	484	198	369	98	95	4	34	25
4732	13130	9496	737	468	256	28094	1157	4	663	0	12	71	3	99	277.25	99	433.46	327	332	326	15	46	0	0	1
6702	11678	8348	422	896	530	28200	949	36	753	9	46	90	24	137	287.24	106	443.23	418	369	374	24	55	0	0	2
14216	7648	4436	2	876	652	27912	617	306	1048	153	84	252	121	165	274.48	241	438.15	474	431	454	30	84	16	0	0
12868	7892	4804	4	1350	1096	28208	607	186	862	59	180	196	90	226	284.71	176	448.5	444	315	446	60	104	18	18	5
6478	11946	8486	451	750	464	28168	994	20	759	6	40	82	10	124	285.17	112	441.75	405	399	370	17	47	0	4	2
5438	12592	9002	583	668	432	28166	1059	7	663	1	32	66	4	99	281.42	108	439.54	406	339	346	14	44	0	2	4
5878	12214	8798	515	798	466	28190	1049	11	694	1	34	86	9	114	284.23	88	440.56	387	371	331	26	57	0	2	3
4026	13598	9916	947	332	162	28040	1269	7	594	1	6	64	5	71	270.58	94	424.43	280	272	291	14	41	0	0	4
11328	7850	5376	1	2110	1592	28614	554	83	569	26	330	161	37	279	313.57	100	459.59	501	205	360	85	73	3	26	18
4136	13544	9856	934	334	166	28042	1302	8	617	1	6	62	5	67	271.28	89	423.62	272	313	277	14	42	0	0	2
3848	13670	9958	983	370	210	28068	1312	3	550	0	12	57	2	72	270.91	77	422.57	268	266	272	13	45	0	0	3
5378	12564	9096	612	680	400	28162	1111	10	696	1	42	78	7	115	284.59	110	441.42	352	369	316	19	43	0	2	2
14356	7590	4344	7	864	658	27888	628	324	1030	161	74	261	128	162	269.77	255	432.4	485	421	436	19	87	15	2	1
11250	7902	5486	1	2098	1528	28600	527	84	595	21	304	165	38	290	316.09	129	460.89	522	197	360	76	79	3	28	18
11854	7858	5154	3	1900	1442	28488	579	116	663	28	252	177	51	262	302.37	134	456.44	477	248	388	78	100	16	28	14

SUPPLEMENTARY INFORMATION:Monte Carlo Atomistic Simulation and Machine Learning Analysis of Na-K Eutectic Alloy in Condensed Phases, D. Reitz and E. Blaisten-Barojas, George Mason University, Fairfax, VA 22030

3824	13762	10006	994	290	154	28040	1312	10	563	1	4	58	7	60	270.51	90	423.53	272	270	279	12	34	0	0	4
4834	13078	9434	741	474	264	28094	1160	9	664	0	10	59	7	76	278.23	102	433.68	352	330	332	16	38	0	0	1
11414	7822	5426	1	2072	1506	28574	502	96	599	20	290	177	50	272	315.85	130	462.91	510	193	375	91	91	6	44	25
13722	7722	4558	9	1084	820	28012	642	272	924	131	104	242	102	188	277.35	199	442.88	461	364	423	31	91	22	2	4
4444	13336	9650	816	416	228	28080	1237	1	642	0	6	67	0	72	275.41	84	431.86	306	341	293	22	48	0	0	0
4262	13490	9756	931	336	198	28058	1295	11	614	2	16	59	8	73	274.32	87	427.86	274	300	280	12	39	0	0	3
13926	7728	4518	4	968	730	27958	622	299	981	146	80	255	111	156	274.53	232	437.21	497	383	426	35	93	16	8	1
4750	13074	9424	733	528	326	28126	1158	3	645	0	24	62	1	89	277.65	101	433.93	344	332	323	17	35	0	0	3
6484	11726	8376	408	942	636	28262	993	19	741	2	92	65	13	126	285.98	112	443.18	422	399	347	29	42	0	6	5
4784	13044	9450	738	530	290	28110	1154	9	642	1	12	52	6	83	278.25	97	433.28	342	330	344	15	37	0	0	5
4184	13496	9788	899	376	212	28066	1251	4	605	0	10	59	4	69	274.91	90	429.75	285	288	311	21	40	0	0	3
4406	13304	9676	843	448	238	28084	1264	8	616	0	12	47	3	83	275.83	71	429.9	290	329	292	11	34	0	0	3
11620	7976	5154	0	1894	1574	28570	537	104	602	20	304	171	57	267	311.08	126	460.14	481	186	391	98	96	8	44	21
10996	7812	5556	1	2254	1676	28716	511	75	540	10	386	159	42	297	324.54	92	466.92	470	184	360	121	87	2	34	22
4170	13528	9820	891	332	186	28054	1247	8	607	1	18	56	5	70	273.73	93	427.34	301	295	303	15	42	0	0	1
14648	7584	4300	4	728	524	27828	683	350	1079	158	40	248	149	159	270.77	235	434.36	431	485	454	15	83	23	4	2
4750	13012	9446	730	578	326	28132	1171	4	637	0	18	58	3	91	278.88	93	434.07	336	334	320	18	43	0	2	2
13036	7848	4822	2	1318	982	28152	602	212	870	77	130	202	93	196	281.96	220	447.82	478	303	447	53	93	25	16	6
4190	13534	9794	881	336	194	28060	1252	7	598	2	12	64	3	75	274.23	82	427.07	301	315	290	10	43	0	0	3
13208	7778	4702	2	1338	998	28156	625	222	821	79	128	193	104	202	283.85	191	446.82	496	325	432	34	84	19	4	4
11700	8008	5386	0	1800	1294	28456	537	119	678	20	252	163	64	261	315.92	150	458.29	490	222	391	90	80	9	14	12
4116	13550	9836	909	356	198	28068	1260	10	600	1	10	50	6	64	274.72	96	429.34	304	283	303	16	33	0	2	0
13932	7820	4560	6	918	660	27940	648	272	1027	122	36	236	114	151	276.6	244	440.53	483	425	442	32	92	17	14	0
4594	13246	9570	794	412	238	28078	1198	8	653	0	18	62	3	82	275.48	99	430.94	326	331	310	11	39	0	0	2
4676	13188	9554	797	422	220	28070	1199	9	687	1	8	67	4	78	274.51	123	428.31	333	331	305	10	46	0	2	2
11296	7950	5478	0	2054	1478	28560	533	97	600	15	280	171	60	306	311.13	113	459.44	447	188	391	91	95	3	24	18
11794	7832	5236	1	1914	1434	28520	565	124	617	23	278	155	67	269	307.37	126	455.97	484	206	400	82	81	10	30	14
7154	11362	8044	375	974	626	28232	934	35	739	7	70	98	23	132	289.68	131	444.83	438	368	355	28	58	0	2	6
4440	13304	9628	823	444	260	28092	1211	7	603	0	16	64	3	74	275.25	96	430.92	334	306	295	13	50	0	0	3
4934	13032	9370	699	480	274	28098	1138	8	685	0	8	65	4	79	278.93	112	435.41	371	341	325	14	42	0	0	1
13522	7740	4608	1	1184	894	28064	576	239	916	82	112	233	118	196	282.64	217	445.84	495	329	437	40	118	16	4	3
4142	13578	9824	922	310	184	28054	1267	5	592	2	16	65	2	71	273.61	89	427.88	291	290	286	15	43	0	0	1
4496	13322	9638	842	386	214	28070	1235	11	636	1	14	63	7	70	274.14	100	428.81	319	326	294	10	42	0	0	4
10730	7882	5722	1	2250	1686	28732	494	60	514	11	408	162	34	308	323.05	92	467.19	443	144	374	129	85	3	50	26
7926	10840	7608	287	1060	712	28254	846	50	798	14	106	92	27	158	288.73	145	448.57	462	373	394	26	48	0	2	6
14270	7604	4402	6	928	656	27916	635	308	1001	151	54	248	125	159	272.85	243	436.47	491	407	442	20	82	17	2	0

SUPPLEMENTARY INFORMATION:Monte Carlo Atomistic Simulation and Machine Learning Analysis of Na-K Eutectic Alloy in Condensed Phases, D. Reitz and E. Blaisten-Barojas, George Mason University, Fairfax, VA 22030

14434	7530	4334	5	890	644	27900	654	319	1026	157	60	266	123	162	273.05	235	437.25	474	433	417	22	101	21	8	0
13308	7684	4632	0	1348	1036	28166	601	248	802	82	142	192	123	225	283.12	175	447.35	444	317	451	53	74	18	16	8
5056	12916	9318	694	506	288	28108	1146	4	707	0	22	59	1	85	277.71	97	434.66	339	357	338	20	39	0	2	5
5238	12884	9228	667	466	266	28094	1107	7	737	0	10	52	5	96	278.34	104	437.52	348	360	378	12	41	0	2	3
12410	7790	4976	2	1664	1264	28344	522	170	715	51	230	193	86	254	291.79	153	453.93	499	221	430	61	102	16	10	12
4042	13532	9856	933	412	222	28068	1282	14	552	1	4	55	10	68	272.11	92	426.53	292	268	277	14	41	0	0	3
13798	7800	4594	4	946	724	27964	645	282	1032	120	94	232	130	179	274.54	254	438.97	467	405	441	22	99	14	8	3
14256	7610	4416	2	918	652	27914	632	311	1014	140	60	228	128	147	273.54	260	437.13	501	410	462	20	80	17	2	1
5666	12506	8956	624	594	366	28128	1125	30	711	8	38	70	16	111	282.93	98	439.94	353	386	319	13	40	0	2	0
14344	7672	4354	3	830	632	27890	639	327	1037	152	56	241	128	167	269.49	265	431.75	463	415	459	20	82	21	2	1
4310	13404	9706	867	416	238	28084	1235	8	602	0	10	65	6	77	275.96	86	431.6	311	302	288	17	41	0	0	1
3888	13668	9940	991	356	202	28062	1314	4	565	0	6	57	2	78	271.34	90	423.45	263	257	270	10	45	0	2	3
4222	13492	9784	890	356	194	28056	1258	7	610	1	8	60	5	81	270.88	85	425.71	288	315	295	9	42	0	0	3
4036	13558	9892	929	374	190	28058	1291	13	582	1	8	59	7	63	271.94	85	425.12	292	292	266	14	37	0	0	4
4996	12954	9316	698	510	314	28120	1153	7	696	0	30	64	4	79	281.05	114	437.29	362	357	316	18	40	0	0	2
12084	7860	5054	0	1756	1392	28434	523	149	665	39	256	199	80	249	303.78	132	455.02	511	215	388	87	103	10	28	8
4812	12984	9392	743	566	350	28140	1155	4	655	0	28	51	2	85	277.06	112	433.29	360	316	332	10	35	0	8	5
11384	7994	5386	2	1906	1526	28582	539	96	635	23	338	165	53	254	311.24	129	459	509	207	384	96	84	9	40	19
3740	13786	10026	1019	302	178	28044	1327	8	544	2	12	57	5	70	270.19	71	422.62	245	255	271	20	35	0	0	2
4490	13246	9576	813	470	296	28108	1218	5	611	0	30	55	2	86	274.76	80	430.98	312	315	305	14	34	0	0	4
5316	12636	9050	620	670	452	28174	1083	10	658	0	48	68	8	113	281.13	115	438.03	373	342	338	16	41	1	2	4
13192	7870	4718	1	1260	964	28130	620	221	868	78	118	190	103	188	282.01	208	446.04	480	321	464	44	86	19	8	4
4416	13274	9642	835	480	270	28096	1206	7	609	0	12	55	2	82	275.79	99	430.43	331	296	299	14	40	0	2	1
13462	7712	4610	1	1230	950	28110	582	231	869	80	136	201	100	199	282.67	194	445.87	494	313	463	34	100	19	10	9
3968	13690	9908	982	290	178	28048	1325	7	585	0	14	63	6	74	271.45	82	424.49	255	291	263	10	41	0	0	5
4262	13452	9730	869	378	232	28074	1229	5	602	0	20	66	2	87	274.86	86	429.75	297	292	300	14	45	0	0	2
5828	12404	8888	541	630	358	28132	1043	7	757	0	24	74	2	96	283.3	132	440.18	420	379	342	13	57	1	0	1
5076	12822	9228	684	604	378	28138	1132	14	641	1	28	57	12	99	282.15	111	438.56	356	324	331	14	33	0	2	3
4074	13518	9838	924	410	224	28070	1283	6	580	1	6	54	4	71	272.19	89	426.64	288	280	284	13	37	0	0	2
11548	7778	5336	7	2014	1522	28564	524	114	587	25	328	168	58	262	315.76	118	461.72	490	190	393	100	90	10	36	19
5588	12394	8892	584	764	498	28184	1070	22	655	7	44	78	9	103	281.83	99	438.83	385	324	329	27	52	2	2	3
5816	12410	8872	560	624	376	28130	1071	19	720	7	32	74	9	93	281.37	118	438.89	399	380	339	15	43	0	0	2
4194	13508	9794	917	356	198	28060	1268	9	610	1	10	65	5	73	272.31	98	425.19	295	293	277	13	41	0	0	2
5232	12674	9136	635	678	410	28158	1140	9	677	0	28	55	6	94	283.12	91	438.54	371	359	315	13	40	0	0	3
5594	12422	8912	539	742	472	28188	1072	2	717	0	46	69	1	110	285.2	105	440.67	383	394	338	16	45	1	0	7
4498	13246	9604	798	456	266	28094	1193	4	630	0	24	69	4	78	275.99	96	433.45	337	313	300	11	44	0	0	6

SUPPLEMENTARY INFORMATION:Monte Carlo Atomistic Simulation and Machine Learning Analysis of Na-K Eutectic Alloy in Condensed Phases, D. Reitz and E. Blaisten-Barojas, George Mason University, Fairfax, VA 22030

11292	7950	5402	2	2034	1562	28590	523	100	595	19	312	176	48	266	308.28	115	458.69	515	200	365	96	95	6	30	18
13834	7812	4566	5	970	710	27954	649	276	980	140	60	242	107	175	273.52	222	437.19	469	389	431	23	89	13	2	2
14414	7492	4322	3	952	672	27910	623	336	994	152	52	243	154	168	273.66	230	437.87	471	410	464	22	82	15	6	2
5750	12406	8838	569	692	446	28168	1083	15	708	1	36	61	11	106	281.23	102	439.35	378	362	347	18	45	0	0	2
13646	7978	4684	5	894	682	27964	634	252	1048	112	74	226	110	186	275.83	213	440.88	439	413	472	32	96	17	6	2
12660	7946	4944	3	1422	1080	28230	599	162	835	50	168	189	88	207	287.42	175	447.37	474	295	448	60	98	11	10	7
14176	7474	4348	2	1074	810	27992	597	315	888	133	102	251	139	196	278.36	208	445.01	443	342	455	41	98	21	8	6
11642	7804	5282	0	2000	1484	28526	516	122	596	30	288	199	55	262	309.97	112	460.08	495	198	368	96	101	8	22	23
5172	12828	9244	660	544	306	28110	1129	14	688	0	14	56	10	84	279.12	109	434.62	357	354	345	18	36	0	2	3
13886	7816	4534	4	932	714	27960	619	281	995	132	72	245	112	176	273.78	242	438.18	480	380	444	26	94	18	4	2
4268	13366	9716	852	448	268	28094	1221	8	591	0	28	60	6	81	274.28	97	428	310	286	302	17	40	0	0	3
4518	13248	9586	821	456	266	28090	1200	10	628	1	16	55	5	80	275.75	111	430.56	322	303	316	18	33	0	0	1
7320	11190	7992	333	1058	618	28228	929	41	768	10	50	90	21	151	289.08	110	447.04	403	381	382	31	64	4	0	5
4576	13186	9570	793	480	266	28094	1207	4	629	0	14	64	2	75	275.57	87	431.29	322	330	305	18	42	0	2	4
11752	8010	5312	0	1806	1324	28468	554	112	651	22	252	157	62	250	302.14	140	453.66	498	205	414	79	97	10	10	13
12196	7892	5088	2	1670	1268	28354	610	148	746	48	214	188	69	258	292.58	151	450.99	456	266	385	57	105	9	26	15
5636	12478	8964	577	666	382	28146	1099	14	727	2	16	61	9	91	281.15	96	437.89	386	408	334	17	39	0	4	3
13706	7820	4602	6	1014	768	28004	592	273	993	112	86	201	128	169	275.65	241	440.19	494	376	491	34	77	11	4	3
11960	7934	5166	1	1722	1360	28446	542	127	690	27	268	155	64	266	305.95	140	457.5	503	229	408	71	96	9	30	11
4314	13398	9700	838	412	242	28080	1221	6	593	1	14	62	3	81	275	91	429.64	320	295	301	9	39	0	0	1
11870	7982	5238	3	1774	1314	28426	619	128	704	37	230	168	66	254	294.59	140	451.71	482	265	388	62	84	6	18	7
3842	13718	9970	978	336	186	28054	1321	10	555	2	2	55	5	68	269.87	83	422.37	273	274	259	12	35	0	0	2
4060	13648	9896	969	274	146	28032	1311	7	604	1	8	69	4	66	273.52	103	425.89	263	288	263	18	46	0	0	2
12086	7896	5108	1	1742	1324	28410	556	142	666	30	230	164	84	257	305.03	130	457.33	476	244	431	72	102	9	24	9
11000	7994	5590	0	2070	1586	28648	512	77	570	25	356	173	29	275	317.56	100	462.87	480	199	368	113	76	1	52	29
4076	13592	9862	938	330	184	28054	1291	5	606	1	8	65	3	68	271	95	423.83	286	301	276	11	43	0	2	2
4000	13618	9896	953	342	192	28060	1298	6	578	1	10	51	4	65	272.13	87	425.22	281	282	284	14	37	0	2	2
7550	11176	7850	307	936	618	28214	895	37	815	8	84	99	15	154	289.31	153	444.91	419	395	385	34	50	5	0	2
13390	7742	4576	1	1290	1006	28134	627	219	864	91	120	216	102	218	281.08	187	447.14	458	321	419	39	99	10	10	5
14126	7646	4384	6	966	746	27950	653	298	981	155	80	255	108	147	269.74	256	432.63	508	401	411	21	83	20	2	3
4124	13500	9808	920	402	230	28078	1283	8	592	1	14	56	5	79	275.94	97	430.46	286	284	274	12	34	0	0	2
4220	13492	9784	897	356	196	28058	1267	7	614	1	10	56	4	65	274.33	93	426.34	296	302	288	17	41	0	0	2
3838	13714	9976	994	332	186	28054	1337	10	549	1	8	55	8	76	270.64	67	422.84	252	274	264	12	36	0	0	0
14388	7584	4342	2	852	650	27896	627	329	1015	172	70	265	122	167	273.89	258	437.97	475	389	434	21	85	20	10	3
13716	7630	4524	0	1180	886	28050	616	263	897	120	102	241	96	198	276.58	222	446.91	485	334	404	35	94	21	10	2
4060	13680	9884	954	258	146	28032	1284	10	576	1	4	60	8	65	271.97	92	425.71	281	274	291	9	41	0	0	5

5740	12362	8876	544	722	432	28164	1043	14	728	1	30	68	10	110	284.68	120	443.23	394	373	350	20	44	0	2	3
12884	7904	4830	3	1330	1064	28208	644	200	840	64	182	198	99	209	283.03	186	446.92	498	348	378	44	98	9	14	3
12956	7886	4820	2	1352	1024	28188	613	207	871	49	136	170	128	223	282.71	198	446.03	463	323	465	46	100	12	12	1
12370	8000	5040	5	1512	1162	28290	605	143	775	43	194	180	78	254	293.68	149	452.42	433	298	441	59	90	6	12	6
5096	12978	9304	694	446	254	28088	1121	9	695	0	10	59	5	97	280.14	111	434.88	351	337	358	9	44	0	0	1
14176	7708	4464	2	870	626	27898	636	306	1040	148	52	252	116	165	274.89	250	440.58	468	412	440	25	87	17	0	2
13678	7780	4522	13	1062	864	28028	611	265	902	119	114	229	104	173	276.83	226	443.94	493	344	437	39	92	16	8	3
13808	7710	4576	4	1000	768	27990	635	280	966	121	120	226	117	195	277.19	206	442.7	444	386	443	35	87	19	8	2
13720	7474	4528	1	1288	932	28082	611	249	860	113	136	231	104	185	276.69	203	445.89	491	320	420	46	94	15	4	2
4582	13240	9568	782	432	250	28090	1185	3	650	0	18	52	3	78	278.14	102	434.15	327	335	334	17	32	0	0	1
14346	7770	4436	8	716	530	27840	680	330	1132	158	34	261	136	155	267.47	273	429.03	446	467	435	16	93	15	8	2
14446	7592	4328	8	814	624	27878	607	325	1064	156	64	261	131	150	271.01	274	435.16	499	398	455	21	96	15	8	2
13690	7810	4622	6	1038	756	27994	643	270	971	111	72	202	129	157	276.99	211	441.54	466	403	475	43	72	9	4	1
11616	7862	5354	0	1950	1426	28520	573	112	608	17	288	162	66	267	303.44	117	457.73	470	216	392	86	87	13	20	17
4358	13258	9634	814	524	318	28120	1201	3	574	0	28	36	0	73	275.91	86	432.18	333	301	329	15	24	0	0	4
5492	12504	9026	624	686	404	28146	1097	24	648	8	32	73	12	111	282.61	94	439.13	361	347	330	17	43	1	2	3
13592	7590	4540	1	1242	982	28130	587	247	852	83	176	202	121	203	283.48	190	447.7	476	306	464	45	96	18	8	6
3900	13604	9940	970	402	214	28070	1305	8	545	1	10	55	6	85	271.56	68	424.99	261	264	279	11	42	0	0	1
11354	7898	5420	3	2078	1510	28572	525	108	567	15	290	164	57	276	315.84	123	459.47	505	180	380	87	77	7	22	18
11034	7898	5576	1	2158	1604	28668	555	75	581	8	356	163	41	265	314.66	112	461.99	481	198	352	115	92	6	40	22
12234	7858	5032	1	1698	1302	28372	558	141	722	39	238	177	79	239	293.41	135	452.92	489	254	430	78	90	7	10	5
4020	13550	9872	923	404	218	28070	1276	6	560	1	4	61	2	66	273.33	81	427.73	300	275	273	14	40	0	2	3
6400	11828	8416	417	936	598	28222	1006	16	768	5	42	74	9	114	285.7	122	442.33	411	403	357	29	51	0	2	4
12870	7690	4852	1	1524	1116	28244	579	176	808	51	178	194	83	227	291.81	156	450.81	473	277	430	56	114	18	14	11
14490	7528	4312	5	848	628	27878	638	346	1033	160	66	262	143	149	271.36	269	434.3	484	426	435	24	86	22	6	1
5568	12616	9024	608	544	334	28122	1083	11	739	3	34	67	8	84	281.95	137	439.06	398	359	348	13	44	0	2	3
13812	7808	4586	4	948	720	27968	617	280	1001	110	88	230	141	171	276.17	231	442.36	464	390	472	34	102	16	4	4
5400	12690	9104	620	570	338	28128	1129	9	712	1	26	82	5	88	278.88	104	437.16	366	390	304	21	49	0	0	3
13280	7894	4756	4	1198	864	28082	652	243	895	102	84	200	105	189	278.37	180	441.59	454	376	446	41	73	15	6	4
5252	12828	9162	635	528	336	28134	1090	5	709	0	28	68	4	102	278.4	129	435.82	376	356	346	15	46	0	0	0
5212	12828	9208	652	538	314	28116	1133	8	722	0	16	54	6	83	278.79	120	435.42	349	362	352	20	34	0	0	3
7244	11246	7948	320	1044	692	28258	912	33	768	9	80	91	17	157	286.59	121	445.56	417	392	380	34	55	2	4	2
11770	8024	5382	4	1744	1244	28414	561	131	731	35	230	185	69	261	302.25	158	456.69	480	251	395	70	102	10	18	16
4930	12954	9332	716	554	336	28130	1171	8	684	2	24	71	4	95	280.96	107	435.69	331	356	308	17	47	0	0	3
4152	13498	9804	907	390	218	28074	1269	6	592	1	12	67	4	69	272.63	91	425.98	305	288	272	14	44	0	0	1
13882	7884	4598	4	848	634	27912	634	289	1037	147	66	246	105	176	273.45	238	437.69	458	419	452	20	85	19	0	4

SUPPLEMENTARY INFORMATION:Monte Carlo Atomistic Simulation and Machine Learning Analysis of Na-K Eutectic Alloy in Condensed Phases, D. Reitz and E. Blaisten-Barojas, George Mason University, Fairfax, VA 22030

12630	7794	4964	2	1572	1138	28288	615	165	746	59	170	191	72	236	296.33	146	452.51	456	292	404	60	93	11	20	8
3946	13696	9942	975	288	158	28038	1309	5	604	1	8	62	3	72	272.48	94	425.37	257	292	278	14	46	0	0	3
11860	7878	5184	0	1852	1414	28480	526	114	652	34	260	180	43	259	306.22	138	457.2	522	211	378	77	89	11	30	16
5738	12432	8916	552	650	380	28146	1059	12	738	1	30	58	8	90	282.85	119	438.65	407	376	362	17	39	0	0	1
6206	11962	8592	452	864	526	28202	994	21	730	3	52	71	12	110	286.12	122	442.17	412	375	360	34	42	1	0	4
14076	7660	4394	8	984	760	27950	638	313	958	160	68	252	112	154	271.91	258	437.03	512	373	415	18	78	20	8	4
13622	7834	4602	12	1024	816	28020	620	274	966	130	116	234	102	168	276.24	249	443.69	493	362	439	31	82	20	6	6
12152	7922	5168	1	1640	1220	28352	556	126	757	33	242	182	65	238	295.67	145	451.8	499	266	433	67	97	13	8	6
4222	13496	9776	883	354	202	28062	1269	5	620	1	10	57	2	78	273.12	88	426.84	278	324	296	14	41	0	2	3
10514	7698	5660	0	2520	1950	28888	477	58	436	6	480	146	28	316	334.14	73	473.29	500	154	346	125	61	1	60	33
12306	7948	5056	2	1604	1202	28322	609	156	766	33	198	171	91	210	294.86	162	452.93	492	282	418	70	90	13	8	6
13792	7968	4592	6	846	672	27942	641	294	1015	132	70	239	117	171	274.08	230	437.76	471	419	442	25	90	19	2	1
4564	13250	9580	792	426	250	28094	1227	6	656	0	24	62	4	83	275.89	87	432.9	309	358	298	15	39	0	0	2
14248	7732	4412	7	830	618	27886	637	329	1042	149	42	251	143	163	269.52	260	433.28	475	437	450	17	92	18	4	1
13784	7738	4566	6	1052	772	28000	618	265	980	120	84	220	118	181	276.63	231	441.45	481	386	463	25	85	12	4	3
11904	7826	5150	0	1834	1450	28506	549	124	626	27	306	175	63	282	304.26	129	458.87	496	225	370	76	92	6	36	13
13670	7926	4664	5	946	694	27966	656	243	1035	106	64	224	110	159	276.1	236	440.05	478	424	442	27	97	13	2	3
3922	13624	9928	974	372	206	28064	1303	7	564	2	12	51	3	70	270.24	79	423.18	279	268	279	11	37	0	0	2
12714	7736	4804	1	1588	1248	28318	582	168	759	47	208	166	87	193	293.83	163	451.37	539	280	426	62	90	13	18	9
12006	7896	5112	0	1764	1378	28444	531	124	666	27	270	172	63	250	305.32	139	456.79	500	210	399	89	95	10	14	12
5304	12826	9166	687	466	302	28102	1123	16	687	5	38	74	10	93	282.09	100	437.13	352	346	338	16	52	0	0	2
12150	7846	5118	0	1670	1306	28396	549	133	716	36	284	177	70	266	299.39	147	452.43	481	253	405	70	100	4	22	11
4548	13252	9582	803	438	250	28082	1235	8	652	1	12	69	4	71	275.2	97	429.29	318	346	281	14	47	0	0	5
5414	12616	9030	595	656	410	28150	1075	3	687	0	24	67	1	95	282.91	108	439.2	395	343	338	19	41	0	0	2
12724	7748	4792	1	1550	1248	28302	624	171	758	62	220	175	77	222	286.68	170	450.31	477	290	407	60	83	9	18	7
4372	13290	9672	822	470	266	28092	1192	5	600	0	22	67	4	83	274.85	100	430.61	317	291	315	17	46	0	0	5
14360	7732	4402	4	732	564	27848	637	319	1129	162	50	268	122	146	268.07	300	430.63	489	444	437	17	95	16	8	1
14068	7686	4466	6	938	696	27926	630	288	1022	161	66	259	94	152	271.63	255	434.92	507	406	425	22	87	15	6	1
14404	7656	4386	7	802	566	27850	649	333	1073	166	36	268	136	154	270.27	255	432.4	467	442	435	20	92	17	0	1
12170	7996	5146	6	1568	1214	28354	589	142	780	42	244	184	73	234	293.77	154	452.21	470	276	420	74	96	13	16	10
10566	8106	5752	2	2190	1714	28794	501	62	496	10	384	160	28	303	329.41	92	470.36	479	152	362	109	83	6	74	41
10800	7854	5684	0	2320	1664	28728	529	75	494	15	380	165	39	291	324.88	91	465.55	475	160	348	120	82	7	24	25
10792	7964	5580	0	2264	1716	28710	533	81	516	15	350	155	39	282	313.58	103	463.81	478	163	375	110	76	5	40	23
13456	7884	4672	4	1106	834	28048	623	253	939	106	92	217	111	179	277.13	244	441.29	528	354	415	14	91	20	4	6
10862	7886	5676	1	2228	1646	28736	498	69	482	15	392	177	31	300	325.82	78	463.9	480	170	357	114	78	2	44	30
4028	13474	9838	918	460	268	28092	1285	7	570	0	24	53	5	69	274.8	91	429.89	292	278	277	15	36	0	0	4

SUPPLEMENTARY INFORMATION:Monte Carlo Atomistic Simulation and Machine Learning Analysis of Na-K Eutectic Alloy in Condensed Phases, D. Reitz and E. Blaisten-Barojas, George Mason University, Fairfax, VA 22030

3952	13674	9938	970	304	164	28040	1305	10	579	1	8	61	4	67	270.88	89	423.09	269	276	273	12	39	0	0	5
5298	12796	9182	631	514	300	28114	1118	6	752	0	24	61	4	78	277.78	114	435.01	376	397	345	18	42	1	0	1
12664	7766	4884	2	1564	1198	28300	569	170	787	52	210	204	91	233	292.45	180	450.58	487	272	400	70	117	6	14	7
5196	12818	9190	657	568	344	28136	1122	7	683	0	20	68	4	87	281.42	111	435.33	380	342	320	12	44	0	0	5
4590	13254	9562	821	414	244	28080	1229	11	649	0	16	61	9	77	274.65	102	430.45	307	330	300	14	40	0	0	5
3928	13714	9958	976	280	148	28032	1315	7	572	1	4	60	4	82	270.27	79	422.7	245	286	275	11	41	0	0	3
3966	13632	9918	962	346	184	28050	1297	7	573	1	4	62	6	66	270.49	78	423.09	273	275	279	16	44	0	0	3
14256	7700	4426	4	836	618	27896	671	312	1047	153	54	265	124	165	271.9	247	435.25	450	441	421	24	91	18	6	1
12110	7732	4952	0	1898	1506	28498	553	133	590	38	266	163	59	261	303.04	134	458.67	497	195	394	83	79	8	34	13
12932	7830	4850	3	1398	1032	28198	627	179	841	54	140	191	78	217	286.02	196	449.91	467	312	417	45	103	17	14	9
3816	13744	9998	994	308	172	28048	1315	6	554	0	10	56	3	74	271	85	423.63	261	264	271	14	38	0	0	1
12628	7834	4850	6	1556	1206	28264	576	180	741	66	182	185	88	229	285.77	161	449.37	496	266	436	56	82	9	6	1
4826	13132	9416	710	428	274	28102	1124	5	706	0	26	59	4	82	276.93	120	431.05	369	335	346	12	38	0	0	3
5044	12832	9266	706	620	360	28140	1172	5	671	1	18	64	3	100	277.73	107	434.89	325	332	313	20	40	0	0	2
4754	13152	9500	776	428	234	28082	1187	6	668	0	14	76	4	94	278.55	110	434.77	316	332	301	16	49	0	0	2
6044	12072	8646	525	840	546	28214	1054	15	674	1	64	74	8	120	287.52	90	444.07	388	361	327	24	44	0	2	4
13320	7736	4718	3	1298	932	28126	609	207	887	72	112	218	100	208	281.53	178	447.02	481	323	427	38	118	15	10	4
12414	7956	4942	3	1570	1240	28332	589	131	775	31	186	163	78	234	295	168	452.65	485	285	442	58	88	5	20	6
12188	7882	5074	2	1698	1302	28396	549	142	702	35	212	182	80	239	302.19	148	453.57	464	216	438	88	96	12	40	15
13816	7528	4398	2	1200	980	28090	596	297	849	116	152	228	133	187	278.23	211	446.69	500	330	432	40	86	22	16	5
14298	7722	4466	4	776	550	27860	643	324	1092	167	40	255	121	136	267.82	281	429.59	499	451	443	19	83	14	8	0
5176	12824	9224	641	548	324	28120	1096	8	721	0	24	59	6	88	279.02	136	435.76	368	356	359	19	40	0	0	3
4862	13046	9428	735	482	264	28096	1161	8	655	0	14	72	6	87	275.75	89	434.33	343	335	312	15	48	0	0	3
6830	11604	8258	385	892	562	28212	940	35	764	7	58	90	24	130	287.78	134	443.92	429	394	368	29	51	2	8	4
11018	7988	5544	0	2124	1608	28662	528	74	551	12	334	173	36	305	316.68	100	462.09	471	173	360	105	92	9	46	18
12218	7880	5084	1	1650	1274	28376	563	132	755	38	254	177	69	246	300.18	156	452.53	470	250	428	75	88	11	14	9
13776	7820	4584	6	984	732	27974	617	276	998	132	78	235	111	170	275.09	244	438.41	498	378	441	25	86	13	0	2
3856	13666	9958	962	370	208	28070	1303	2	568	0	10	58	2	64	273.28	92	426.4	279	275	272	17	46	0	2	3
4404	13232	9632	812	534	298	28114	1198	4	589	3	14	56	0	73	276.45	80	431.57	339	301	308	20	36	0	0	1
12128	7984	5134	1	1618	1252	28372	553	131	752	34	234	173	65	258	299.79	163	454.57	477	252	419	69	94	10	22	11
10962	7986	5488	1	2158	1682	28670	493	81	543	16	360	172	30	288	324.54	119	467.54	496	164	363	118	85	6	30	21
11772	7904	5232	0	1866	1426	28512	548	96	624	19	288	168	48	250	310.39	131	456.69	520	212	376	84	94	6	24	16
5366	12648	9068	591	622	406	28158	1069	10	705	0	46	66	4	89	281.16	110	437.93	392	365	340	24	45	0	2	7
14260	7746	4390	8	788	632	27890	639	312	1066	156	74	252	117	164	267.75	291	429.2	483	414	436	15	82	16	0	1
10444	7990	5770	0	2338	1798	28826	456	48	478	7	424	145	26	300	331.44	95	473.78	497	130	373	137	76	1	56	30
14060	7788	4528	3	852	620	27910	606	296	1046	124	62	237	139	179	275.38	256	440.44	451	389	490	25	100	20	0	4

4476	13340	9620	810	406	234	28082	1214	5	656	1	6	68	2	73	276.06	113	429.59	337	321	289	10	48	0	0	3
12730	8020	5006	3	1322	958	28180	637	186	852	61	138	187	100	222	285.97	182	446.13	471	321	423	35	100	8	6	5
14212	7682	4380	5	892	684	27916	644	314	995	148	66	245	123	147	273.92	237	438.89	497	423	439	22	84	19	0	1
4420	13378	9648	824	382	234	28080	1216	2	634	0	18	52	2	77	276.05	94	429.99	314	321	318	15	26	0	0	2
11442	7934	5348	0	1946	1532	28580	544	95	595	20	346	172	40	260	317.01	123	462.41	492	209	361	106	81	3	32	20
14032	7732	4506	8	906	672	27924	627	310	1026	153	74	245	126	158	270.13	268	432.81	486	397	452	22	80	14	2	4
4394	13258	9648	820	502	284	28106	1208	3	616	0	20	73	3	83	275.89	94	430.52	324	286	288	18	51	0	0	2
14020	7802	4544	5	830	630	27906	640	302	1056	146	72	248	113	156	274.28	244	438.66	464	430	444	31	87	17	8	4
3946	13618	9926	967	368	194	28058	1300	8	561	2	6	53	2	58	271.99	90	424.85	280	272	280	19	35	0	0	3
13368	7760	4700	3	1226	910	28092	609	234	895	97	112	201	107	195	277.36	221	442.94	483	327	463	35	82	15	16	2
3948	13620	9906	960	370	210	28064	1286	7	562	1	10	57	4	73	271.48	81	424.41	271	265	289	18	34	0	0	2
11494	7888	5324	0	2042	1516	28574	541	94	589	15	284	158	44	286	311.22	107	457.96	466	198	399	89	90	7	24	20
4544	13132	9538	781	558	330	28128	1198	9	607	1	26	46	5	73	275.96	87	429.21	350	318	307	17	29	0	0	1
13428	7782	4644	2	1210	912	28092	602	225	902	82	106	218	102	188	283.05	204	445.01	479	340	431	55	113	13	10	3
3772	13724	10004	1003	360	196	28060	1331	6	540	1	4	55	3	58	270.48	80	422.32	271	270	264	16	38	0	0	2
11058	8062	5522	0	2036	1586	28648	507	107	553	20	330	153	51	274	315.05	115	464.87	505	160	392	98	77	12	52	28
4072	13552	9834	908	374	222	28070	1252	6	571	0	16	57	5	82	272.84	87	426.63	294	280	293	13	40	0	0	1
4748	12966	9418	741	618	364	28142	1173	7	616	0	26	62	4	90	277.76	97	434.83	349	313	297	19	44	0	2	1
4660	13136	9504	772	496	294	28116	1204	7	662	0	24	62	5	90	278.35	104	435	326	334	299	15	43	0	2	0
11240	7878	5378	1	2070	1652	28638	503	82	577	15	378	182	43	309	312.59	97	460.3	444	164	377	109	97	2	42	27
3876	13688	9942	980	338	202	28060	1308	4	570	1	14	58	3	70	270.73	96	423.05	265	263	275	15	33	0	0	4
11154	7750	5464	0	2236	1674	28688	533	78	542	9	364	158	42	277	319.06	91	463.95	481	167	368	105	85	8	44	30
4228	13414	9752	899	430	242	28080	1287	6	618	1	14	54	3	64	272.61	89	425.68	285	312	280	23	41	0	0	1
4034	13692	9894	946	268	150	28040	1265	6	602	1	2	53	4	64	270.87	116	423.38	295	264	306	11	36	0	0	2
5016	12972	9338	680	476	276	28100	1123	7	706	0	22	58	6	80	278.5	100	434.28	359	366	356	15	41	0	0	5
10612	7786	5748	2	2434	1780	28840	484	63	464	7	428	155	28	329	332.87	66	473.15	459	136	360	120	81	2	50	31
14402	7706	4350	2	752	596	27874	646	315	1107	167	66	264	115	144	269.76	305	433.77	501	442	423	17	85	16	2	0
5378	12690	9104	617	586	352	28138	1089	6	726	0	28	68	4	101	280.83	118	436.87	377	368	343	15	45	0	0	2
4756	13074	9456	757	506	294	28108	1187	14	643	1	22	54	9	85	276.11	99	432.17	335	334	316	15	42	0	0	1
4378	13382	9692	849	382	216	28064	1240	10	639	1	14	54	7	80	275.22	89	429.17	297	317	303	14	39	0	0	2
11910	7928	5180	0	1790	1360	28442	611	123	697	31	262	159	61	232	296.33	144	450.98	486	263	391	79	94	11	12	12
3954	13628	9906	953	356	206	28064	1295	8	575	2	14	60	5	72	271.34	90	423.8	293	278	263	9	37	0	0	1
10844	7848	5586	0	2252	1748	28748	511	79	497	15	408	172	43	321	322.76	87	466.28	463	160	341	101	84	3	56	33
4426	13374	9632	844	392	242	28078	1226	8	600	1	12	61	4	78	274.79	95	430.87	313	303	302	12	36	0	0	3
13438	7816	4648	6	1148	890	28062	605	260	946	110	110	223	115	185	277.65	225	442.24	493	367	438	42	96	15	12	0
10996	7872	5502	1	2156	1712	28702	509	91	521	12	416	150	53	299	315.91	101	464.51	494	164	379	99	78	2	44	28

5844	12360	8868	581	646	374	28120	1065	25	727	7	28	78	15	97	281.54	114	439.02	380	367	351	21	56	0	0	2
11558	7776	5244	2	2058	1582	28566	536	113	592	31	314	177	57	279	306.53	119	457.65	491	202	354	91	91	3	30	21
4826	12982	9418	732	554	316	28124	1174	8	652	1	28	61	6	97	277.53	104	434.5	329	336	315	15	40	0	0	3
14402	7660	4458	7	766	514	27846	648	332	1093	151	44	257	130	148	272.8	269	437.35	480	446	434	16	95	24	2	3
14052	7754	4482	5	906	670	27922	631	293	1037	133	54	251	128	185	275.9	234	441.34	434	415	453	33	97	17	4	2
4644	13198	9532	796	438	254	28082	1222	8	657	2	16	61	5	78	273.04	102	427.76	321	339	301	11	45	0	0	2
4284	13408	9722	874	414	238	28080	1235	2	602	0	14	63	1	81	275.98	83	429	307	290	296	12	48	0	0	1
4476	13262	9588	802	468	290	28110	1219	5	630	0	24	66	4	74	276.69	106	431.51	324	324	292	18	41	0	2	3
4980	12814	9304	682	642	374	28148	1131	7	652	0	34	64	4	101	278.33	100	437.11	363	319	318	14	44	0	0	2
4548	13278	9568	807	424	256	28088	1222	6	648	0	14	61	3	73	276.24	101	430.63	326	337	291	15	36	0	0	3
14522	7612	4356	4	780	542	27852	640	358	1065	156	40	240	161	128	269.36	277	432.22	496	438	467	20	78	24	0	2
5056	12790	9274	667	638	358	28136	1115	5	675	0	20	69	3	103	279.17	113	435.91	359	328	331	17	48	0	0	1
14400	7638	4374	5	810	592	27870	615	341	1055	165	54	256	139	159	268.68	286	431.89	497	411	448	15	79	17	2	0
11572	7780	5288	0	1980	1542	28542	495	119	585	25	348	173	61	260	307.93	120	456.98	526	202	395	88	85	9	30	19
11402	7950	5454	2	1928	1464	28568	561	87	664	16	336	161	48	251	310.67	116	460.1	482	229	398	91	92	3	34	21
13794	7800	4528	4	992	778	27988	626	272	990	129	96	224	113	157	275.82	251	440.98	507	383	451	25	80	14	0	2
4482	13218	9594	799	508	292	28112	1183	4	600	0	16	55	1	84	277.98	91	434.95	323	299	325	22	36	0	2	2
13506	7492	4564	1	1402	1036	28162	586	231	808	79	148	212	115	221	281.94	163	447.94	474	283	444	41	111	23	12	6
14436	7558	4324	3	876	638	27890	627	316	1026	167	52	259	112	145	271.72	273	435.71	508	407	436	19	82	24	6	1
4858	12956	9422	693	568	298	28116	1129	4	664	0	14	61	3	93	278.3	99	434.5	361	339	334	14	38	0	0	1
10844	7850	5590	0	2288	1726	28728	553	61	547	10	382	160	38	286	322.77	92	465.15	442	185	367	122	80	4	42	30
11794	8026	5282	1	1772	1312	28436	568	119	733	28	228	165	54	242	300.99	162	454.4	513	255	390	67	91	11	22	17
11290	7854	5464	1	2072	1562	28632	560	103	564	25	340	169	51	263	315.02	104	462.68	482	196	362	105	83	9	48	25
14592	7484	4300	4	848	586	27860	638	345	1050	177	48	256	129	141	270.7	265	433.59	494	433	444	19	73	21	2	1
4144	13446	9792	913	434	248	28082	1265	7	565	1	16	64	4	85	273.15	87	426.88	292	278	272	12	39	0	2	2
5964	12318	8752	563	646	422	28146	1039	29	732	7	44	69	15	108	282.73	141	441.12	403	341	356	16	40	0	0	0
7234	11344	8024	360	928	604	28214	919	40	757	13	74	96	23	148	288.67	115	444.14	425	391	371	23	52	0	6	5
4904	13072	9394	736	452	260	28092	1180	6	694	0	10	72	5	85	276.51	114	430.16	331	347	314	16	48	1	0	1
3840	13732	9982	993	306	174	28044	1293	8	553	2	10	55	5	70	270.36	83	422.39	272	254	290	13	34	0	0	2
5384	12688	9124	613	572	328	28118	1096	11	752	1	22	68	5	77	279.39	116	436.94	392	385	333	22	47	0	0	2
13084	7904	4828	11	1240	924	28110	615	223	904	78	118	216	98	199	282.75	210	448.02	501	325	402	33	112	17	12	6
10918	7928	5630	0	2156	1628	28692	508	76	509	15	394	170	35	321	322.33	85	462.88	473	155	367	86	91	5	34	26
14486	7568	4328	6	830	598	27862	646	343	1037	169	50	258	131	155	267.78	269	430.63	480	424	434	16	80	21	2	1
13738	7736	4566	2	1052	806	28010	609	259	991	118	102	236	109	178	277.69	226	442.53	477	378	447	38	103	19	8	4
12828	8008	4912	6	1274	988	28178	605	189	876	68	162	186	89	197	286	194	448.4	485	302	452	49	97	12	6	8
12918	7752	4824	3	1464	1094	28234	613	187	798	56	164	193	96	219	286.24	168	447.49	478	303	419	49	106	10	16	8

4624	13206	9534	767	458	262	28094	1179	3	654	0	10	61	2	88	275.85	92	429.95	331	327	320	13	52	0	0	2
5908	12338	8768	526	670	434	28156	1026	18	716	2	38	60	10	101	283.21	125	438.61	418	357	365	14	36	1	0	4
12442	7634	4906	0	1764	1376	28414	558	161	700	53	260	192	73	248	297.06	125	452.93	464	252	415	85	95	11	30	15
6718	11604	8234	430	932	648	28230	950	34	690	7	84	83	20	137	287.63	114	443.53	413	332	367	33	56	1	10	4
13562	7684	4562	4	1190	932	28074	621	265	898	110	140	231	110	191	277.75	205	442.55	503	366	414	25	93	24	4	4
4844	12978	9440	741	542	286	28110	1166	4	645	0	20	71	2	91	277.28	84	433.17	340	328	309	17	50	0	0	1
6290	12070	8564	473	738	470	28176	1004	25	747	6	42	69	15	104	286.32	121	443.12	429	382	365	17	45	1	2	4
3856	13694	9986	972	332	174	28050	1300	4	561	1	6	69	3	88	270.8	82	423.7	250	270	278	13	47	0	2	1
13646	7914	4668	7	942	716	27978	638	281	990	101	88	213	144	168	275.1	222	438.72	452	404	471	38	99	19	4	5
5422	12580	9058	595	692	394	28160	1091	5	721	0	14	64	3	100	280.67	124	436.24	393	379	328	16	41	0	0	0
13438	7820	4634	6	1138	906	28074	622	243	924	92	128	208	115	188	276.99	200	446.68	474	346	458	38	95	18	10	3
14010	7758	4552	7	876	644	27924	654	307	1023	147	82	242	121	168	273.27	231	438.98	461	435	440	26	77	15	2	0
13218	7808	4722	0	1270	982	28162	597	197	886	63	148	194	106	196	284.92	208	447.67	486	337	456	50	100	8	14	6
5072	12820	9292	680	602	326	28128	1125	9	658	0	16	71	8	91	277.8	110	433.48	358	332	332	15	51	0	0	6
13696	7810	4630	6	1026	750	28000	642	268	988	116	84	228	102	178	277.25	232	443.36	483	385	424	27	98	16	4	2
14490	7692	4356	11	726	536	27842	652	340	1084	174	42	265	126	167	269.6	256	431.58	457	454	440	13	79	15	0	0
4402	13260	9656	824	500	270	28100	1244	7	595	1	12	52	4	79	276.72	78	432.09	312	325	294	13	31	0	0	1
4716	13044	9474	742	564	310	28122	1155	3	633	0	12	61	3	97	275.6	77	431.66	333	336	332	18	44	0	2	1
13932	7730	4558	5	992	684	27948	619	306	986	125	48	236	139	160	275.9	259	441.27	495	373	446	27	92	15	4	3
14220	7756	4466	6	788	584	27872	640	312	1078	150	58	254	125	158	270.71	275	433.48	487	434	440	11	94	19	0	2
11230	7940	5558	0	2032	1494	28628	508	89	575	19	332	174	51	265	327.05	114	465.67	512	185	375	98	94	1	36	27
4736	13142	9464	782	460	276	28094	1217	10	662	1	16	54	3	78	276.49	99	430.87	323	346	302	16	36	0	0	1
5670	12536	8918	624	592	380	28122	1075	25	688	3	24	62	14	105	282.52	110	439.08	356	338	362	26	40	0	2	1
13910	7772	4490	8	958	746	27960	619	289	983	131	82	230	117	182	274.49	233	438.88	464	387	463	29	82	19	2	1
10366	7778	5640	1	2612	2020	28926	502	51	403	8	444	134	25	335	331.56	65	471.68	471	130	346	125	55	3	58	37
4576	13242	9556	804	444	262	28094	1252	8	644	0	12	64	4	83	274.55	85	429.51	303	357	278	14	43	0	2	1
14190	7746	4472	10	818	600	27882	626	333	1029	147	52	248	148	165	271.75	252	436.29	473	418	459	17	88	19	4	3
11312	7898	5514	2	2088	1456	28564	544	95	595	22	268	175	43	285	307.11	138	457.51	504	191	362	73	95	4	28	19
4164	13542	9790	897	346	210	28064	1249	5	601	1	8	56	2	70	271.43	90	425.03	294	295	308	14	32	0	4	5
4804	13026	9444	744	530	290	28110	1194	7	660	1	16	60	6	88	276.6	79	431.38	316	352	319	21	39	0	0	0
13350	7764	4718	2	1186	920	28108	604	223	907	74	150	206	106	195	283.99	205	446.49	486	359	440	43	106	15	20	5
4736	13078	9484	747	516	280	28108	1159	6	650	1	14	62	5	87	277.53	96	433.2	346	328	323	15	37	0	0	2
4540	13216	9574	793	470	274	28094	1189	8	618	0	20	68	5	87	275.5	99	431.85	318	307	314	16	51	0	0	4
4730	13026	9420	715	574	358	28142	1139	5	618	0	32	59	2	93	279.02	85	433.88	358	318	327	17	44	0	2	2
13448	7658	4588	1	1316	990	28134	595	226	833	85	124	221	88	179	281.6	211	447.3	535	281	402	46	106	23	10	2
13578	7852	4654	2	1062	784	28014	618	271	982	115	76	221	121	170	275.86	257	439.38	504	373	440	30	92	15	8	2

SUPPLEMENTARY INFORMATION:Monte Carlo Atomistic Simulation and Machine Learning Analysis of Na-K Eutectic Alloy in Condensed Phases, D. Reitz and E. Blaisten-Barojas, George Mason University, Fairfax, VA 22030

5124	12754	9220	635	650	380	28154	1103	0	691	0	24	64	0	97	279.13	103	434.98	381	353	332	17	43	0	2	1
14386	7606	4378	3	836	610	27882	632	338	1042	159	62	258	136	158	273.17	251	438.05	479	414	440	20	86	21	4	2
11188	7886	5308	3	2170	1744	28696	538	90	537	21	338	163	48	284	314.74	100	462.78	497	191	370	101	80	8	58	19
4042	13578	9872	941	356	198	28058	1287	8	591	1	12	56	5	72	270.72	94	423.84	269	295	288	18	34	0	0	3
7490	11162	7890	290	996	624	28232	913	39	818	7	64	91	22	139	287.74	141	444.9	455	415	363	27	55	2	6	0
12430	7774	4994	2	1656	1242	28332	588	162	753	49	220	192	85	237	294.14	161	452.06	470	256	411	70	106	8	16	8
4346	13396	9702	876	392	222	28070	1242	9	612	1	12	53	4	59	271.63	101	426.74	311	291	303	22	35	0	0	2
11956	8068	5152	2	1648	1314	28404	582	125	726	28	244	160	64	242	300.6	154	453.66	490	259	418	71	78	8	22	10
4574	13176	9554	793	480	288	28104	1182	8	631	0	30	59	5	86	275.72	102	431.61	333	309	320	14	40	0	2	2
11416	7914	5386	1	2016	1510	28578	548	104	577	24	306	157	52	283	311.15	102	459.3	483	197	383	87	76	6	30	17
11262	7918	5310	2	2128	1660	28626	552	91	549	32	312	158	43	257	303.75	112	458.84	505	181	383	104	75	5	34	16
5740	12296	8846	519	794	480	28194	1055	13	690	1	36	67	11	111	284.24	96	440.27	396	368	342	21	38	0	2	2
14334	7698	4408	7	806	580	27868	636	336	1076	156	42	246	143	149	269.47	272	431.94	492	435	455	13	83	14	0	2
13256	7838	4744	1	1198	932	28130	623	222	891	79	146	187	109	190	280.88	207	447.87	497	340	443	37	83	6	14	4
10646	7806	5572	5	2376	1906	28824	465	65	450	11	438	156	24	299	321.71	96	466.45	532	146	332	115	68	5	66	34
14396	7720	4366	5	746	576	27858	641	324	1090	170	52	264	121	156	267.72	288	429.41	484	442	438	12	77	20	2	1
14056	7504	4346	2	1130	880	28040	574	280	892	111	114	240	131	202	282.8	211	447.2	475	330	454	39	109	15	10	4
14320	7710	4434	9	782	564	27858	668	335	1068	160	46	253	135	137	269.31	271	432.44	482	464	430	18	80	19	2	3
4174	13512	9822	894	354	184	28054	1238	11	575	1	8	67	7	74	272.33	94	426.14	312	277	291	11	46	0	0	1
12078	7888	5152	1	1724	1290	28388	553	132	721	34	240	187	62	252	300.5	155	452.77	476	236	404	78	101	13	16	15
12450	7850	5028	0	1582	1180	28312	619	159	760	49	202	184	83	255	294.57	140	451.53	434	289	412	61	100	12	20	7
12910	7698	4792	3	1492	1142	28242	596	202	793	72	196	187	98	213	284.83	153	447.3	482	299	444	54	89	12	12	8
4860	13002	9416	744	522	286	28100	1185	10	652	3	14	74	6	88	277.93	85	432.62	325	349	305	19	40	0	0	1
12082	7932	5152	3	1694	1286	28410	550	125	723	26	242	164	64	269	302.86	140	456.31	493	237	411	63	94	4	22	12
5014	12994	9350	690	468	258	28094	1144	7	720	0	10	55	4	90	277.73	103	433.67	351	372	341	12	37	0	0	0
4108	13522	9830	895	388	212	28068	1253	5	586	2	8	72	3	76	272.55	82	427.71	294	282	282	16	48	0	0	3
5362	12600	9122	644	680	366	28142	1098	9	700	2	12	69	5	94	281.04	112	437.96	378	340	331	19	56	0	0	3
3996	13586	9886	952	382	210	28068	1306	5	577	1	8	54	3	72	270.12	86	422.75	281	280	270	12	38	0	0	0
14428	7706	4356	2	740	570	27854	635	321	1110	165	52	264	125	140	268.77	289	429.56	492	444	444	18	89	13	2	3
4358	13380	9666	855	402	252	28080	1252	7	620	0	22	62	4	81	275.45	86	429.64	292	324	290	16	49	0	0	2
4048	13548	9840	938	388	232	28074	1296	9	577	3	18	61	5	73	270.99	88	424.9	280	284	265	16	39	0	0	2
14334	7668	4334	7	832	662	27906	610	324	1020	155	72	254	128	172	269.77	246	433.29	487	403	456	17	88	25	4	0
11446	8044	5456	3	1886	1382	28502	538	110	650	27	252	188	58	271	306.77	122	456.35	461	209	392	98	97	3	30	18
12096	8118	5110	3	1550	1242	28352	569	128	810	20	216	156	85	219	293.07	178	450.82	497	269	454	69	99	8	20	10
5544	12388	8994	548	782	438	28178	1029	13	679	2	32	69	6	121	284.25	107	440.56	399	345	353	16	39	0	0	3
12382	7938	5100	2	1542	1130	28296	565	163	790	41	182	191	86	244	293.15	151	454.45	476	277	431	64	107	10	22	5

10410	7732	5788	1	2562	1892	28896	435	47	424	7	444	154	24	306	331.4	78	472.29	504	109	368	132	87	2	60	44
4822	12962	9396	730	586	342	28134	1179	11	642	1	24	47	6	76	277.66	105	433.6	351	342	313	21	31	0	2	2
5632	12376	8924	585	766	450	28180	1091	10	689	0	32	69	6	94	283.48	115	439.85	395	353	315	24	48	0	0	2
13474	7956	4694	11	1016	784	28018	663	241	973	109	84	221	95	187	277.47	221	444.02	449	397	435	32	89	18	10	1
4206	13530	9788	905	342	188	28056	1264	7	611	1	2	60	1	86	274.4	83	428.62	277	289	295	9	44	0	0	2
4550	13322	9606	823	366	208	28062	1227	6	677	0	10	67	4	84	275.84	99	432.17	301	335	301	10	43	0	0	3
11792	7964	5232	5	1766	1392	28454	569	118	715	28	262	196	60	255	303.7	155	455.07	485	252	370	79	102	9	44	18
10772	7858	5626	0	2330	1736	28750	494	73	509	15	382	169	34	297	321.36	90	467.42	474	152	369	117	79	3	46	30
4244	13460	9756	888	388	216	28072	1243	5	592	0	8	65	2	75	273.73	92	429.09	313	287	287	13	42	0	0	0
12588	7902	5038	2	1486	1066	28258	627	153	841	57	158	158	70	204	288.34	181	448.25	479	337	455	55	71	8	20	5
4028	13548	9874	942	406	210	28066	1272	8	561	1	0	50	5	66	271.7	95	424.23	292	259	295	14	37	0	0	4
13464	7810	4662	2	1168	860	28058	652	236	941	107	82	224	96	183	276.45	225	443.27	491	364	414	25	94	14	12	3
4546	13272	9586	821	428	242	28082	1207	6	639	1	8	75	4	79	273.78	115	429.69	331	305	291	13	46	0	0	1
4332	13408	9686	881	396	240	28076	1241	7	608	0	14	65	5	72	274.21	103	428.07	322	277	283	10	44	0	0	2
12890	7954	4870	4	1308	1004	28186	600	191	872	58	148	184	96	193	284.9	179	448.48	488	311	457	53	106	12	10	7
5780	12330	8874	554	720	420	28158	1087	17	712	3	34	75	9	97	284.07	106	438.33	378	382	329	22	46	0	0	5
13436	7612	4580	2	1284	1034	28146	589	234	846	92	186	228	107	215	280.89	175	448.43	469	300	428	48	112	13	14	8
7576	11030	7762	305	1076	728	28268	911	39	788	7	80	91	22	151	288.79	120	447.34	431	413	360	35	54	2	16	5
4196	13456	9770	905	402	234	28074	1271	8	609	1	12	52	4	65	271.82	87	424.8	299	299	292	14	36	0	4	3
12498	8024	5030	4	1412	1096	28268	574	140	846	35	194	163	86	229	286.94	176	450.19	491	320	466	51	98	2	10	3
13374	7808	4750	4	1180	842	28062	639	246	937	109	104	199	104	182	276.43	206	442.49	476	368	452	34	76	15	4	4
11326	7852	5450	1	2088	1538	28606	527	90	585	24	310	179	41	283	315.97	113	460.17	492	196	371	98	84	6	36	15
10874	7730	5464	0	2382	1866	28798	508	68	489	12	430	152	29	298	322.97	73	468.46	475	145	366	122	84	5	50	33
5622	12510	8980	571	622	370	28138	1076	14	745	2	32	73	8	101	279.98	127	436.09	372	381	344	26	55	0	2	0
11684	7790	5304	0	1966	1458	28540	550	110	619	23	314	177	54	263	307.55	126	460.68	472	219	390	100	84	10	24	18
4046	13568	9866	953	366	206	28066	1291	9	569	2	12	63	6	74	272.78	80	425.61	275	281	278	15	40	0	2	2
4852	12996	9426	735	526	288	28108	1174	12	637	1	18	70	9	87	278.38	99	434.42	341	327	307	16	43	0	2	1
4924	12938	9324	710	584	348	28134	1154	9	651	1	16	65	4	83	280.92	109	436.53	373	337	299	17	36	0	0	0
11386	7942	5364	0	1944	1552	28580	514	112	600	28	352	165	59	271	313.94	121	459.78	502	199	389	99	83	6	38	17
4076	13546	9844	935	376	212	28066	1278	5	567	1	12	67	3	77	271.3	83	424.42	287	282	273	11	46	0	0	3
5896	12300	8816	566	686	406	28134	1063	27	702	4	30	75	18	95	282.49	125	440.88	396	359	342	20	46	1	0	1
4514	13252	9614	765	454	248	28098	1189	4	630	0	16	71	3	74	277.14	99	431.08	338	337	305	19	46	0	0	2
3838	13700	9976	982	344	190	28056	1313	4	565	1	8	67	2	67	270.39	91	423.4	276	266	258	10	49	1	0	5
4550	13152	9536	798	554	316	28116	1197	11	596	2	8	54	6	88	275.4	99	430.44	331	295	309	15	35	0	0	0
11566	7758	5296	0	2078	1550	28586	528	103	593	27	290	188	44	307	316.62	107	464.17	445	207	370	104	97	5	44	19
7692	11146	7842	330	882	544	28168	884	40	846	8	60	108	22	155	287.55	127	444.26	417	403	388	27	79	1	2	3

SUPPLEMENTARY INFORMATION:Monte Carlo Atomistic Simulation and Machine Learning Analysis of Na-K Eutectic Alloy in Condensed Phases, D. Reitz and E. Blaisten-Barojas, George Mason University, Fairfax, VA 22030

13756	7542	4414	2	1246	996	28106	609	257	827	108	132	231	105	211	282.5	182	445.14	450	297	426	51	99	18	18	5
4462	13204	9626	824	526	280	28112	1235	4	612	0	14	50	2	77	277.71	89	431.96	320	319	298	15	37	0	0	1
4446	13272	9606	829	478	288	28110	1247	4	617	0	16	62	4	71	276.71	85	430.52	308	328	284	23	38	0	4	2
4060	13562	9848	933	372	212	28064	1266	3	582	1	10	59	2	59	273.21	90	427.16	293	279	294	23	44	0	0	2
4122	13522	9828	920	376	206	28064	1259	7	596	1	10	61	4	67	272.06	97	424.44	303	278	287	14	41	0	0	3
5244	12670	9164	638	664	384	28156	1124	8	693	1	30	69	6	84	280.82	118	435.98	388	355	311	19	47	0	0	1
12512	7692	4940	3	1654	1278	28348	591	164	749	62	250	179	64	244	290.39	169	449.58	501	280	391	57	82	10	18	5
12530	7894	4942	2	1550	1182	28290	583	169	776	51	166	171	84	210	290.56	183	448.53	520	287	428	55	91	14	26	8
14038	7694	4456	10	954	724	27948	630	308	988	148	78	251	130	158	273.26	258	438.05	499	392	432	25	94	12	4	1
4518	13150	9510	766	564	368	28148	1178	5	571	0	36	55	2	94	277.92	82	433.92	335	290	305	22	31	0	2	1
4000	13570	9886	935	392	212	28068	1290	7	581	1	8	54	2	62	270.95	102	422.52	297	283	277	13	35	0	0	2
11272	7986	5416	1	2034	1542	28584	538	90	617	12	308	148	54	243	311.79	134	458.65	534	206	393	88	77	5	24	19
10786	7786	5714	0	2350	1686	28760	521	71	482	11	396	153	32	302	332.84	85	472.25	464	166	349	128	65	5	38	27
5006	12824	9262	673	630	392	28152	1116	15	636	1	38	59	9	98	279.8	99	434.47	372	328	328	15	34	0	0	3
13608	7642	4564	1	1198	922	28080	581	247	906	114	134	231	101	204	277.15	198	446.69	489	327	437	42	101	16	12	1
5006	12864	9304	672	590	340	28126	1152	6	668	0	22	61	5	83	280.15	101	436.09	352	363	326	20	41	0	0	2
7588	11088	7808	301	1024	656	28236	878	36	781	6	68	93	21	160	287.42	111	444.38	430	385	389	27	59	1	4	7
14330	7640	4354	2	868	648	27896	641	306	1067	170	54	270	103	148	268.05	284	430.1	510	429	407	17	94	13	2	1
4966	12930	9322	698	546	332	28126	1135	8	666	1	30	51	7	91	277.04	92	432.81	354	336	351	15	31	0	0	1
6066	12034	8688	481	854	508	28204	1028	19	708	3	50	66	10	118	285.31	96	441.96	399	375	354	21	40	1	4	5
4450	13256	9624	840	472	272	28094	1214	9	602	1	18	50	3	71	274.15	107	427.24	324	290	311	18	31	0	2	3
6804	11574	8232	374	934	610	28234	942	23	776	5	76	78	15	148	288.87	115	444.6	419	394	373	27	54	0	4	1
14142	7750	4450	8	848	648	27904	643	315	1036	162	62	259	118	147	269.31	265	431.96	497	418	421	24	85	15	2	1
11390	7870	5372	2	2000	1562	28588	567	107	591	24	362	174	58	275	311.6	107	458.77	461	218	366	94	86	9	32	26
3970	13584	9900	945	386	214	28066	1285	5	582	1	10	68	2	65	272.05	83	424.72	284	278	271	21	45	1	2	2
13574	7838	4598	7	1090	840	28038	634	260	916	121	96	230	114	171	277.53	224	441.85	486	370	437	33	80	11	0	5
12004	8058	5134	2	1688	1290	28386	567	124	725	30	186	181	68	240	301.44	147	452.66	499	263	410	71	100	13	22	7
12876	7890	4832	4	1332	1076	28218	604	200	822	55	200	162	106	202	283.2	187	449.54	490	321	457	53	80	14	12	6
13496	7920	4716	1	1054	760	28020	649	252	968	102	68	216	120	173	277.21	213	444.61	483	395	437	31	97	15	6	0
12516	7920	5014	0	1502	1126	28280	595	170	773	40	186	177	83	222	292.54	189	450.03	498	282	417	53	96	15	16	8
10518	7808	5624	0	2456	1944	28878	500	65	455	12	454	163	35	336	326.81	87	470.58	469	141	329	121	76	2	62	33
4064	13600	9872	938	332	178	28050	1289	9	585	1	2	64	4	65	272.03	88	425.06	293	293	271	11	44	0	2	2
11940	7956	5126	2	1796	1364	28418	561	107	680	29	222	171	56	258	295.12	133	453.48	494	223	409	69	92	6	12	9
4400	13246	9622	808	530	308	28124	1206	6	597	0	16	53	5	86	276.81	89	433.15	330	296	306	14	34	0	2	1
4676	13176	9512	781	460	260	28094	1191	4	653	0	10	76	2	87	278.78	104	433.53	327	315	299	15	55	0	0	2
12060	7942	5148	1	1706	1292	28392	562	129	731	30	210	173	63	234	299.2	164	454.04	507	238	415	67	99	12	30	13

SUPPLEMENTARY INFORMATION:Monte Carlo Atomistic Simulation and Machine Learning Analysis of Na-K Eutectic Alloy in Condensed Phases, D. Reitz and E. Blaisten-Barojas, George Mason University, Fairfax, VA 22030

11624	7886	5316	1	1906	1452	28514	532	114	637	24	288	178	60	256	306.66	110	459.11	460	213	398	110	97	3	38	24
13080	7894	4812	4	1274	944	28128	587	217	878	75	112	203	108	220	281.01	177	445.89	456	335	468	46	99	15	10	4
11420	7806	5340	0	2070	1600	28622	543	117	558	18	334	174	63	280	318.38	105	460.04	483	185	353	95	97	7	46	25
6008	12108	8690	539	796	516	28186	1065	28	689	7	64	81	16	114	285.98	100	441.75	377	363	324	24	57	1	4	6
14398	7578	4316	4	876	658	27888	623	343	1005	181	58	275	114	147	271.02	274	436.06	520	392	410	17	82	19	4	1
3814	13696	9982	986	358	200	28060	1303	4	556	2	10	62	2	60	270.42	90	422.57	281	262	271	17	42	0	0	3
14128	7608	4388	5	966	758	27952	618	326	957	160	102	255	123	163	272.39	262	435.75	512	379	423	19	76	21	0	3
14336	7672	4412	6	796	588	27866	631	323	1077	154	58	267	134	147	273.72	273	438.78	484	433	438	23	102	16	4	3
12622	7902	4958	1	1438	1122	28266	594	171	822	38	206	194	98	214	291.32	166	452.14	469	312	419	70	113	10	18	12
4806	13032	9396	750	534	328	28120	1161	12	631	2	24	59	5	96	279.27	100	436.01	329	308	329	15	34	1	0	5
4476	13334	9648	840	378	212	28062	1231	11	633	1	12	61	8	78	274.58	95	430.3	301	327	305	15	40	0	2	2
13954	7686	4494	2	1008	744	27966	640	282	975	145	74	238	101	156	275.04	235	438.47	488	384	435	31	80	15	6	3
14458	7568	4264	5	844	674	27888	628	327	1043	173	76	272	113	161	269.7	254	432	494	418	423	14	91	21	4	3
4648	13164	9532	769	472	264	28096	1209	4	650	0	16	70	2	79	277.18	97	433.51	323	344	290	21	43	1	0	1
4010	13528	9870	943	426	232	28076	1289	6	563	1	10	61	5	80	271.87	82	425.52	277	270	277	12	45	0	0	2
6094	12110	8660	547	760	492	28180	1049	20	696	4	62	71	11	117	286.72	110	443.17	379	349	346	26	43	0	2	2
4704	13212	9542	762	390	210	28072	1201	4	689	0	14	61	1	76	276.19	95	432.78	325	365	315	14	41	1	0	2
4122	13548	9804	900	368	222	28078	1274	2	608	0	14	55	2	78	275.14	91	428.32	291	306	289	8	38	0	0	3
5928	12312	8826	555	648	382	28134	1081	21	760	10	36	85	9	102	283.27	120	438.35	382	403	324	20	54	0	2	0
4984	12902	9326	670	564	324	28122	1139	8	689	0	22	63	7	92	278.88	92	435.51	343	371	338	20	42	0	0	2
14486	7634	4356	5	750	560	27848	642	326	1106	160	58	257	128	148	267.86	289	429.41	483	451	442	16	88	20	4	2
13800	7828	4582	8	946	722	27966	652	278	1021	130	80	231	110	165	276.1	250	439.17	470	422	436	29	82	13	8	4
11644	7752	5278	0	2004	1532	28576	552	105	604	23	312	172	57	252	315.77	122	461.57	487	201	382	92	90	7	50	28
11132	7818	5588	0	2158	1552	28628	493	84	554	13	336	186	39	276	322.32	86	466.86	467	167	369	120	95	8	42	34
6142	12122	8646	470	748	476	28190	996	7	767	1	54	80	4	115	285.1	124	440.76	418	391	358	22	48	0	2	4
4976	12916	9314	677	574	334	28130	1124	5	675	0	16	58	2	93	278.8	114	435.55	351	336	342	15	37	0	0	7
4046	13692	9894	942	254	144	28036	1285	4	604	0	6	67	3	79	272.69	83	424.98	270	296	286	10	42	0	0	1
4864	13038	9424	686	500	270	28106	1160	6	701	0	8	58	2	80	278.58	103	433.51	344	388	332	19	35	0	2	2
4546	13226	9558	779	494	280	28108	1185	5	616	0	4	56	3	76	277.65	103	432.16	347	307	315	13	38	0	0	3
10794	7862	5656	0	2374	1682	28740	528	59	505	8	338	150	37	287	323.42	92	466.4	505	171	355	105	80	2	34	26
11378	8146	5394	2	1878	1434	28510	568	94	678	20	256	178	43	283	311.13	140	459.99	452	217	371	85	112	5	18	18
5382	12636	9094	624	634	368	28134	1112	17	682	2	20	72	10	96	281.96	88	439.32	375	370	315	17	50	0	0	2
4702	12928	9454	723	666	372	28152	1164	11	583	0	30	53	5	101	279.37	79	436.6	345	313	309	15	28	0	0	2
12144	7872	5124	3	1706	1284	28388	548	159	697	44	236	189	77	250	301.53	138	454.55	482	256	405	81	97	8	22	11
11862	7844	5144	1	1856	1462	28488	578	136	647	46	284	191	52	259	302.71	148	456.68	487	228	362	77	99	13	32	15
4406	13358	9660	838	394	236	28074	1244	5	635	0	20	62	2	80	274.95	87	430.46	293	338	294	14	42	0	0	4

SUPPLEMENTARY INFORMATION:Monte Carlo Atomistic Simulation and Machine Learning Analysis of Na-K Eutectic Alloy in Condensed Phases, D. Reitz and E. Blaisten-Barojas, George Mason University, Fairfax, VA 22030

11448	8014	5320	1	1936	1506	28544	546	103	636	21	290	162	58	250	305.8	134	455.06	493	213	414	84	89	8	30	24
5574	12478	8968	609	688	414	28152	1134	16	721	2	26	73	8	109	283.81	106	437.87	353	393	306	16	51	1	4	2
4698	13104	9504	799	494	276	28094	1193	9	635	1	14	65	6	77	275.51	96	429.29	334	313	303	17	45	0	4	3
11312	7856	5496	3	2060	1510	28598	527	90	603	19	320	178	48	264	315.44	126	464.4	494	205	373	106	90	4	42	17
4254	13506	9772	898	328	182	28050	1264	10	625	1	8	53	6	73	272.54	94	425.25	281	306	309	14	41	0	0	2
5192	12766	9194	627	600	360	28142	1102	1	697	0	30	67	1	96	280.81	107	435.92	373	350	341	18	43	0	0	1
13976	7810	4514	2	892	678	27938	634	274	1054	131	58	247	106	172	275.21	248	438.72	478	410	436	23	100	14	10	1
4992	12842	9300	691	624	360	28142	1117	9	653	0	24	53	5	93	277.95	111	436.18	372	315	341	16	32	0	0	1
6182	11930	8556	487	914	584	28230	1024	21	692	1	60	68	14	119	284.62	97	441.21	397	354	347	31	45	0	4	4
4042	13660	9894	940	280	156	28040	1267	9	613	1	6	61	4	66	272.35	106	425.92	293	283	292	13	42	0	2	2
11506	8004	5316	1	1862	1490	28532	531	100	655	20	322	164	56	270	304.36	121	455.35	459	220	419	101	100	6	26	15
5682	12470	8946	602	660	370	28140	1109	19	729	6	12	65	9	90	280.32	115	438.04	375	376	333	22	39	0	0	0
4334	13440	9714	864	368	206	28068	1269	10	628	0	6	44	4	71	275.68	89	430.53	294	321	303	11	34	0	0	2
5040	12908	9278	662	556	328	28122	1125	5	678	0	12	65	2	88	278.68	111	434.77	363	359	337	17	37	0	0	2
13952	7810	4556	9	888	654	27926	647	324	984	142	60	241	134	169	275.05	227	438.54	459	415	438	29	82	20	6	2
4474	13278	9614	788	438	264	28096	1172	4	623	0	28	61	3	83	275.3	99	431.94	340	306	322	13	39	0	0	2
3982	13658	9920	956	308	170	28050	1296	7	577	1	12	67	5	62	271.98	96	425.27	285	289	268	17	44	0	0	1
12256	7744	5044	1	1764	1326	28402	555	140	692	41	248	197	69	244	299.98	158	455.98	477	231	394	82	99	7	18	15
4694	13136	9488	772	488	282	28102	1192	5	647	0	14	60	4	85	276.42	121	429.91	347	320	302	7	36	0	0	1
12786	7924	4924	5	1378	1032	28214	644	184	810	55	158	203	99	203	286.15	160	448.07	494	329	390	42	110	11	12	8
11136	7952	5478	1	2098	1596	28628	522	95	577	16	322	163	48	255	315.16	122	463.43	513	193	369	103	89	6	46	26
5488	12532	9010	592	682	416	28164	1056	11	690	0	34	59	7	102	280.14	107	438.04	396	348	357	18	35	0	2	3
4028	13590	9872	942	354	202	28058	1296	11	567	2	12	63	8	80	270.75	81	425.32	265	291	271	14	39	0	0	3
5330	12792	9170	629	488	296	28108	1113	8	756	1	26	69	5	92	279.65	114	438.11	362	389	344	15	48	0	6	2
4496	13306	9614	823	412	240	28084	1241	9	640	1	16	51	6	81	274.46	90	427.43	290	339	314	16	33	1	0	3
11472	7768	5414	0	2002	1522	28584	496	103	626	25	360	178	46	280	316.21	107	463.9	470	209	402	96	99	8	40	30
4716	13060	9502	768	528	280	28102	1205	8	645	0	16	55	2	71	277.41	90	433.8	330	345	309	16	42	0	0	5
5672	12364	8860	559	762	490	28198	1078	14	696	1	50	54	10	103	285.37	103	439.44	400	370	330	18	31	0	0	4
14324	7680	4362	5	800	642	27892	640	316	1049	152	78	258	121	148	272.88	256	437.85	478	432	437	29	89	23	6	2
14042	7744	4528	5	884	652	27932	644	288	1031	130	76	239	120	166	276.38	245	440.68	471	420	444	23	96	19	6	3
12284	7872	5120	2	1658	1190	28332	585	133	724	35	188	182	59	232	300.29	148	449.47	487	260	415	64	98	14	20	10
4658	13236	9510	765	410	254	28082	1197	6	658	0	12	55	2	92	274.16	92	429.83	306	360	325	16	31	0	2	1
14446	7730	4392	4	704	524	27844	662	318	1116	152	46	265	127	136	272.99	286	436.99	478	468	428	23	98	18	2	1
14386	7728	4408	10	712	548	27848	639	333	1111	165	64	273	130	147	270.92	276	434.73	493	463	427	15	92	22	2	1
11052	7886	5548	2	2142	1632	28682	573	76	569	7	372	149	39	287	316.98	103	465.76	460	197	358	106	85	5	42	25
3948	13738	9932	961	264	152	28036	1307	8	591	1	2	62	5	64	270.81	98	423.1	272	295	271	16	43	0	0	1

SUPPLEMENTARY INFORMATION:Monte Carlo Atomistic Simulation and Machine Learning Analysis of Na-K Eutectic Alloy in Condensed Phases, D. Reitz and E. Blaisten-Barojas, George Mason University, Fairfax, VA 22030

4736	13098	9474	726	494	284	28106	1155	9	652	0	18	55	8	80	278.2	92	434.85	338	338	344	22	35	0	2	2
4116	13640	9870	938	258	138	28028	1269	9	617	1	6	60	5	72	270.82	95	424.54	283	288	298	11	45	1	0	1
12476	7828	5000	1	1574	1212	28348	601	171	735	44	240	177	93	228	295.79	144	455.19	474	285	409	79	87	14	14	6
4786	13040	9472	704	520	276	28112	1152	2	683	0	16	64	2	77	277.92	106	433.37	362	356	325	17	42	0	2	1
12290	7776	5142	0	1682	1208	28350	583	176	730	51	240	201	88	217	293.81	166	451.37	502	261	388	70	104	17	12	16
4910	13012	9356	681	514	308	28116	1148	9	691	1	16	63	4	86	278.58	103	435.07	361	364	319	15	40	0	0	1
5026	12920	9320	682	524	298	28106	1117	5	690	1	18	68	2	80	278.96	116	435.66	371	336	334	18	43	0	0	4
5170	12908	9250	687	470	282	28102	1147	8	724	0	20	60	5	79	278.64	122	435.44	357	367	328	17	39	0	2	3
4842	12994	9406	742	540	308	28108	1184	8	657	1	16	58	3	81	276.45	87	431.25	328	345	321	22	41	0	2	1
4868	13032	9400	716	490	294	28114	1163	4	692	0	30	70	4	98	277.16	103	433.04	328	360	318	16	50	0	0	3
11276	7892	5378	0	2072	1622	28634	520	93	568	21	360	180	48	296	316.77	109	461.75	457	176	382	107	97	7	30	24
6136	12090	8614	527	764	512	28184	1022	23	696	4	68	74	15	127	285.75	115	443.05	398	345	356	14	47	1	0	3
3828	13706	9984	993	332	188	28052	1320	7	554	1	14	65	3	69	270.56	81	423.12	264	273	264	14	44	0	0	2
4740	13110	9472	773	478	278	28098	1202	5	655	0	20	58	4	95	276.11	83	431.14	312	327	316	11	39	0	0	2
12776	7870	4892	4	1440	1072	28212	604	207	812	75	152	191	103	186	281.43	172	448.57	488	326	441	66	82	8	8	7
13142	7952	4804	6	1204	900	28118	653	204	927	63	104	182	104	196	280.49	206	444.78	474	357	441	34	90	16	10	1
12840	7748	4842	2	1470	1132	28250	584	174	808	71	204	214	66	236	292.77	162	452.27	454	291	407	70	98	15	14	8
13362	7696	4580	4	1284	1040	28136	592	224	829	102	164	224	77	215	281.7	186	446.46	489	299	416	41	94	20	10	4
4222	13504	9750	902	358	220	28066	1269	9	606	2	12	57	4	81	271.77	92	424.29	279	297	295	13	35	0	0	2
10922	7966	5626	5	2226	1588	28656	530	77	567	16	278	153	38	310	315.88	97	462.83	468	189	363	101	85	6	44	22
5158	12798	9252	629	576	314	28112	1100	8	698	0	14	70	3	89	281.05	108	437.64	373	360	340	20	50	0	0	1
10960	8148	5528	0	2034	1602	28640	495	75	584	8	322	176	37	302	318.04	109	461.65	477	165	367	109	105	7	40	24
4630	13024	9478	780	624	362	28142	1198	6	602	0	24	56	3	83	277.74	80	434.76	338	318	301	17	41	0	0	3
4792	13054	9428	729	500	306	28112	1153	4	665	0	30	65	2	92	279.74	84	435.44	340	333	329	16	46	1	2	2
13536	7694	4550	3	1226	952	28088	593	253	852	98	124	220	114	208	276.16	182	445.58	468	310	454	38	104	21	6	5
4264	13430	9764	879	386	208	28066	1233	7	604	1	12	66	4	76	273.67	101	426.98	311	284	293	14	50	0	2	1
14164	7672	4468	2	904	644	27914	604	324	1004	147	58	262	133	184	274.17	235	439.04	479	384	439	21	98	17	4	0
4254	13424	9738	898	414	236	28078	1289	11	595	1	12	60	6	66	273.86	84	426.88	295	311	265	15	36	0	0	3
4682	13214	9532	776	410	228	28076	1193	8	672	1	10	66	5	75	274.22	111	427.56	341	339	303	12	42	0	0	2
4190	13520	9798	875	348	194	28060	1242	4	614	0	10	69	3	88	275.19	87	428.57	284	304	295	13	51	0	0	3
4368	13320	9686	849	456	246	28086	1222	8	591	1	8	62	3	84	275.48	92	430.67	322	284	291	9	43	0	2	2
13446	7834	4632	6	1114	902	28072	620	247	949	111	130	211	108	182	277.54	226	442.47	486	373	448	36	80	11	14	5
4490	13164	9588	787	552	306	28116	1206	7	594	0	16	65	5	80	276.54	90	433.68	337	311	285	16	42	0	0	3
4330	13370	9702	877	424	240	28080	1238	8	592	1	12	72	5	77	273.6	90	427.51	314	289	281	14	51	0	2	1
4372	13328	9652	852	452	272	28096	1228	13	600	1	20	52	8	75	271.41	91	426.02	319	303	304	11	37	0	0	5
4594	13162	9554	775	496	278	28104	1204	4	640	0	20	50	2	78	278.43	91	431.58	330	325	318	16	32	0	0	0

SUPPLEMENTARY INFORMATION:Monte Carlo Atomistic Simulation and Machine Learning Analysis of Na-K Eutectic Alloy in Condensed Phases, D. Reitz and E. Blaisten-Barojas, George Mason University, Fairfax, VA 22030

11068	8082	5468	0	2096	1598	28640	549	77	573	13	284	147	42	279	312.72	118	459.94	484	192	384	100	80	4	42	17
4328	13284	9672	836	514	292	28106	1249	6	584	1	14	58	4	94	276.62	75	431.33	286	324	294	13	38	0	2	3
14374	7752	4378	8	718	574	27862	663	325	1079	158	64	253	127	158	270.33	282	434.18	475	438	432	11	87	20	2	0
7846	10872	7650	279	1076	714	28260	900	52	795	14	94	88	29	140	287.7	133	445.88	460	390	362	29	46	2	8	5
13668	7640	4604	4	1182	838	28034	633	275	931	130	98	231	120	175	275.96	222	441.72	491	384	426	32	85	15	4	3
3996	13636	9906	953	322	178	28048	1278	8	596	1	10	53	4	74	270.67	99	423.61	276	277	297	12	38	0	0	3
4356	13384	9684	859	400	236	28076	1251	8	629	2	16	66	4	70	272.91	89	427.81	310	317	281	14	47	0	0	3
5198	12700	9240	648	638	334	28130	1128	13	674	2	18	73	4	93	281.16	104	436.88	364	356	307	23	55	0	2	0
3956	13526	9880	940	462	254	28086	1264	5	538	1	8	60	4	67	272.76	90	425.13	305	244	279	19	38	0	0	1
12048	8012	5188	4	1640	1234	28354	603	122	759	38	210	169	59	217	293.26	154	451.42	498	281	403	77	83	10	22	5
3982	13622	9912	965	346	184	28052	1298	5	595	1	6	61	3	70	270.39	110	422.21	281	270	272	12	44	0	0	1
13202	7866	4756	4	1206	942	28132	592	206	884	76	150	219	93	211	284.84	211	447.63	492	310	424	36	104	16	8	6
14330	7692	4388	8	824	598	27870	648	337	1059	167	38	274	135	158	269.52	267	432.54	487	447	412	15	91	15	0	0
3748	13788	10026	1006	302	174	28048	1329	7	556	1	10	63	4	67	270.76	86	423.27	262	271	261	12	43	0	0	3
10926	7748	5508	0	2292	1820	28812	484	57	501	10	446	152	29	304	331.43	92	469.76	492	163	371	121	79	4	70	25
4856	13126	9408	776	422	258	28082	1202	11	672	1	12	58	7	83	275.96	94	430.36	317	347	321	12	36	0	0	3
3960	13680	9928	964	306	166	28044	1300	4	567	0	4	69	2	70	271.55	72	425.2	269	283	269	15	48	0	0	3
5996	12076	8714	524	850	512	28200	1049	7	712	1	48	75	5	115	284.27	104	444.53	382	350	342	26	53	0	2	5
3938	13564	9878	938	430	260	28092	1275	4	546	0	22	61	2	75	275.3	95	427.49	300	259	267	15	37	0	0	1
12884	7956	4846	6	1344	1018	28184	637	175	870	54	122	185	79	186	286.05	193	445.98	479	323	429	55	96	22	14	8
4452	13266	9616	827	468	272	28090	1207	8	628	1	16	50	4	79	273.53	109	426.45	325	298	317	13	33	0	0	2
11092	7938	5616	1	2072	1526	28640	502	89	560	17	360	174	43	297	321.98	116	466.99	470	173	367	112	95	6	34	25
4608	13126	9512	790	532	314	28112	1216	4	648	0	18	66	2	82	276.36	97	431.26	313	329	293	19	44	0	2	4
4004	13600	9888	935	368	198	28060	1272	7	576	1	0	61	2	77	275.97	90	428.89	285	272	283	13	41	0	2	2
5092	12732	9238	670	692	386	28156	1115	6	639	0	14	63	6	86	281.88	98	438.42	396	324	320	10	45	0	2	5
4674	13154	9502	772	474	276	28096	1181	9	649	1	16	68	5	67	277.03	111	431.22	346	322	312	20	49	0	0	2
13036	7942	4886	9	1234	896	28116	625	209	917	81	108	205	97	191	282.72	193	445.31	490	345	427	38	98	11	10	6
5846	12210	8804	494	810	482	28196	1050	15	729	1	42	62	8	112	285.17	91	441.44	395	406	346	23	40	2	2	2
14318	7748	4410	8	736	580	27862	624	312	1091	156	68	253	115	163	271.04	270	433.32	490	423	451	10	88	19	2	0
4486	13288	9624	826	432	238	28076	1217	6	626	1	8	69	1	79	274.83	90	428.78	311	318	300	17	52	0	0	1
3838	13690	9964	971	352	206	28066	1296	2	562	0	16	55	0	82	271.43	84	424.29	268	264	281	12	40	0	0	1
10678	7840	5576	0	2320	1880	28830	508	58	465	10	460	166	34	274	330.5	95	470.49	477	144	349	132	72	2	74	47
13258	7880	4680	3	1218	954	28114	635	208	901	88	110	213	93	201	278.5	194	446.08	476	350	428	35	97	13	14	3
13040	7870	4800	2	1278	1002	28170	569	183	853	60	172	201	91	184	285.2	220	448.38	513	285	457	57	109	14	8	5
4908	12940	9342	707	564	346	28132	1154	7	634	0	30	64	5	97	281.64	94	436.84	339	334	319	18	35	0	2	3
7158	11256	7998	316	1082	700	28278	895	21	723	4	82	83	14	133	286.08	114	445.06	463	373	377	34	46	1	0	5

SUPPLEMENTARY INFORMATION:Monte Carlo Atomistic Simulation and Machine Learning Analysis of Na-K Eutectic Alloy in Condensed Phases, D. Reitz and E. Blaisten-Barojas, George Mason University, Fairfax, VA 22030

5204	12740	9194	663	616	360	28142	1148	8	681	1	28	59	4	82	279.81	103	438.92	368	361	320	16	36	1	0	3
3974	13678	9900	953	308	182	28048	1284	9	594	1	6	66	4	67	270.98	111	423.37	296	281	265	10	40	1	0	3
4864	13040	9396	732	496	292	28106	1178	8	668	0	18	77	6	75	275.84	103	431.35	354	354	294	17	58	0	0	2
4278	13366	9700	854	478	272	28102	1249	5	590	0	8	65	4	77	276.55	96	431.6	311	316	278	13	41	1	0	4
4394	13240	9634	846	518	300	28108	1237	9	599	0	22	57	5	68	274.19	93	428.42	320	298	288	24	45	0	0	0
14344	7738	4402	5	748	572	27858	633	338	1087	166	46	248	134	140	268.29	279	429.65	504	443	449	15	76	12	8	1
13648	7880	4630	2	974	754	27980	595	272	1010	118	86	228	123	187	275.01	242	439.07	494	377	462	22	97	15	8	0
4320	13370	9708	854	426	242	28084	1238	6	603	1	18	60	1	64	274.83	101	428.44	322	293	290	18	36	1	0	2
14418	7704	4392	2	714	552	27850	642	335	1111	166	66	262	135	150	268.44	302	430.06	496	430	430	9	90	14	4	2
11452	7806	5316	1	2046	1596	28610	534	93	556	20	356	189	53	260	315.29	95	459.69	469	174	379	119	100	5	38	20
4034	13636	9868	965	324	186	28050	1322	6	588	1	2	63	3	72	271.49	81	424.39	269	300	259	9	38	0	0	2
4036	13666	9906	960	268	148	28036	1306	9	601	2	12	70	5	61	272.4	94	424.48	277	296	263	17	52	0	0	1
10808	7710	5536	2	2448	1848	28814	519	77	472	9	418	141	42	322	331.82	83	467.74	458	162	375	109	73	6	44	30
11728	7942	5296	3	1826	1394	28500	565	116	666	26	276	161	59	262	309.4	141	453.54	504	221	386	80	91	15	32	9
4182	13514	9804	888	356	194	28058	1245	6	591	0	8	57	3	81	275.02	85	428.45	297	297	297	11	38	0	0	2
14012	7836	4550	3	834	622	27926	630	277	1046	119	72	223	115	166	275.25	222	441.03	449	408	485	34	94	20	0	1
10946	7842	5528	2	2242	1736	28750	551	80	513	20	402	159	32	300	324.49	83	466.95	451	172	350	111	79	6	52	31
5534	12516	9032	579	662	376	28152	1098	13	742	2	32	69	7	92	279.46	132	435.28	388	386	325	20	44	0	0	1
10566	7816	5680	0	2422	1848	28828	501	69	431	7	442	161	40	324	336.25	76	472.86	464	133	351	97	67	3	52	44
6168	11992	8634	514	844	498	28178	1005	28	672	8	40	79	19	112	283.63	105	440.76	392	337	373	28	45	0	2	6
13500	7922	4744	5	1056	732	28016	641	259	986	107	58	224	127	182	276.76	220	444.44	482	390	437	25	103	12	4	1
5122	12816	9228	696	592	354	28136	1149	12	665	1	24	54	8	89	281.8	105	437.68	347	332	336	17	41	0	0	4
4486	13278	9598	847	436	268	28090	1223	11	625	3	22	64	5	82	274.53	112	430.62	320	291	288	14	43	0	2	1
4970	13052	9384	692	424	238	28082	1114	9	717	0	14	50	4	89	276.88	116	433.63	351	339	372	15	39	0	0	1
4858	13014	9366	698	518	334	28124	1141	9	650	1	28	65	5	96	281.14	95	437.91	343	336	327	17	42	0	6	4
6020	12130	8698	476	800	494	28190	1017	14	726	3	46	80	7	117	285.19	113	440.16	407	376	342	27	44	0	2	0
5160	12766	9236	672	598	342	28132	1131	5	662	0	30	54	5	84	281.09	93	437.52	358	354	344	23	36	0	0	1
4114	13526	9824	916	376	214	28068	1291	4	604	1	14	56	2	78	272.52	79	426.34	279	312	279	9	36	0	0	3
10578	7798	5510	1	2460	1988	28862	498	70	448	10	482	146	39	310	322.89	92	466.25	513	152	351	115	61	5	46	30
12160	7926	5088	2	1652	1286	28368	595	131	779	32	230	178	75	237	295.03	163	450.31	490	295	403	61	103	6	22	12
5278	12830	9202	643	496	278	28094	1117	10	713	0	10	72	6	89	277.98	110	434.66	360	369	338	16	50	0	0	2
11662	7754	5256	1	2028	1524	28560	508	100	609	21	296	174	56	281	311.49	114	458.27	456	208	409	107	91	8	34	20
5402	12552	9052	596	702	426	28172	1082	11	676	2	34	53	7	102	280.12	101	438.3	375	364	360	18	29	0	2	4
14444	7570	4342	3	856	612	27876	641	335	1038	171	48	269	111	155	269.88	250	431.76	490	437	420	17	85	26	4	1
13584	7780	4618	7	1118	830	28024	599	262	939	125	90	239	104	194	276.58	219	442.67	488	364	430	30	92	13	4	4
3860	13732	9976	984	300	168	28044	1311	8	570	0	8	49	5	68	270.39	87	422.23	266	272	283	12	34	0	0	3

13924	7632	4526	5	1052	752	27976	607	297	946	138	90	247	120	162	274.54	236	437.54	508	373	434	32	93	17	0	1
14432	7654	4400	9	776	546	27852	656	332	1083	160	40	269	129	140	271.14	273	434.25	489	449	422	18	102	19	2	1
13684	7734	4574	2	1086	840	28044	597	260	926	100	112	225	120	175	278.52	204	444.81	496	359	447	39	103	17	14	6
5350	12732	9136	655	556	326	28122	1123	14	706	3	22	69	9	81	281.51	116	437.57	374	353	325	21	49	0	0	1
14212	7700	4422	5	874	640	27898	656	304	1043	150	48	250	122	166	267.75	244	429.24	466	431	443	12	92	16	2	4
13584	7664	4554	1	1244	930	28092	556	245	871	80	110	231	124	221	281.6	187	445.16	476	302	454	44	123	12	6	2
11760	7842	5306	2	1890	1380	28474	563	123	633	26	268	186	69	267	307.82	127	460.09	468	231	374	84	91	6	26	16
14548	7490	4254	3	880	650	27884	632	316	1038	171	60	272	111	165	270.45	261	434.53	483	419	422	18	87	18	2	1
4480	13350	9620	825	382	232	28080	1234	5	650	0	14	60	3	77	275.81	106	430.33	311	332	301	10	33	0	2	4
12946	7778	4838	1	1440	1054	28218	585	203	805	77	144	207	94	223	294.26	181	450.28	487	294	421	53	86	16	16	4
3738	13788	10050	1018	304	158	28042	1343	6	553	1	4	55	4	61	270.44	81	423.52	263	266	261	11	35	0	0	2
14170	7728	4430	4	828	664	27916	606	310	1016	161	94	267	120	164	272.61	259	437.12	498	389	438	19	93	13	2	3
4202	13492	9740	884	382	246	28080	1265	6	609	1	16	57	4	76	272.29	88	425.42	289	308	294	15	40	0	2	2
13456	7780	4686	4	1150	858	28054	592	231	934	117	120	236	87	196	277.33	226	444.31	495	342	434	35	102	10	4	1
5046	12954	9322	682	464	286	28112	1147	7	669	0	36	66	6	97	282.22	88	439.02	332	376	332	19	40	0	4	2
5570	12504	9022	569	658	362	28136	1054	9	718	1	18	73	2	75	280.24	114	437.73	419	365	343	25	52	1	2	2
4292	13524	9752	887	296	174	28048	1262	6	645	1	8	68	5	68	273.15	95	426.3	298	326	285	13	50	0	2	2
4788	13056	9444	738	518	292	28114	1180	6	654	0	14	57	3	93	277.67	96	433.46	327	345	321	16	35	0	2	1
11548	7746	5344	2	2092	1512	28562	564	114	591	25	282	165	55	274	307.77	120	458.74	475	209	372	88	83	9	36	18
13444	7860	4756	4	1106	788	28056	587	251	909	86	94	222	129	182	282.83	210	447.52	489	339	449	46	106	11	6	5
14336	7654	4416	5	820	592	27878	621	328	1053	142	56	253	143	160	274.26	251	438.11	475	408	456	25	92	16	4	0
11104	8060	5510	2	2038	1542	28594	510	90	560	10	310	156	56	286	316.01	113	460.06	488	189	408	88	75	9	28	22
14244	7696	4412	3	836	642	27908	630	294	1048	152	76	245	100	158	273.25	252	437.33	490	414	445	21	83	19	2	1
5846	12252	8786	493	776	484	28190	1046	14	726	0	44	60	12	86	282.57	122	437.46	433	396	342	22	34	0	2	2
13334	7748	4706	4	1252	922	28088	608	220	914	88	118	226	101	197	277.66	208	443.98	464	327	445	49	112	12	8	2
14198	7678	4430	7	870	660	27920	638	311	1022	136	84	231	130	159	274.72	228	438.28	464	425	471	28	80	18	0	2
3746	13760	10022	1016	334	184	28050	1316	6	531	1	4	58	4	67	270.77	83	423.59	264	247	274	14	37	0	0	3
5526	12618	9036	609	568	346	28126	1084	7	735	2	28	75	4	97	279.88	112	436.35	378	370	342	15	53	0	4	4
4584	13334	9616	827	328	176	28046	1210	10	675	1	8	73	5	71	274.45	118	428.75	318	316	306	14	53	0	0	3
14230	7680	4420	6	846	642	27892	636	312	1043	149	72	262	123	144	272.08	259	435.53	482	416	442	27	98	24	2	4
4798	13076	9438	747	492	282	28100	1173	10	651	1	14	53	7	88	278.82	97	433.75	338	340	325	11	35	0	0	3
5432	12524	9010	583	708	452	28174	1057	11	684	0	46	73	9	104	280.26	121	437.96	395	344	338	21	35	0	0	4
5650	12370	8936	584	744	426	28154	1078	17	672	6	28	62	8	127	282.27	80	438.39	360	350	355	11	35	0	0	1
12402	7848	5012	1	1632	1216	28320	618	158	766	40	190	182	88	223	292.64	173	448.96	489	289	396	61	94	14	18	6
4886	13010	9424	686	494	264	28094	1145	9	716	0	16	60	6	85	278.72	117	435.79	349	372	334	16	39	0	0	2
4450	13286	9624	803	454	264	28096	1214	5	620	0	18	76	3	85	274.39	93	431.03	322	318	284	14	52	0	0	2

SUPPLEMENTARY INFORMATION:Monte Carlo Atomistic Simulation and Machine Learning Analysis of Na-K Eutectic Alloy in Condensed Phases, D. Reitz and E. Blaisten-Barojas, George Mason University, Fairfax, VA 22030

4862	13010	9400	732	516	302	28116	1165	7	669	1	26	62	4	84	276.5	102	433.37	340	343	325	19	42	0	0	1
4650	13118	9496	756	512	306	28106	1181	9	639	0	24	56	3	87	275.9	102	431.53	319	330	324	20	39	0	0	4
4904	12934	9336	720	584	350	28128	1149	12	619	0	18	58	6	96	279.59	92	436.15	355	319	319	13	41	0	2	1
4164	13506	9818	912	362	196	28058	1267	7	612	1	10	59	5	80	271.71	94	424.09	287	285	289	11	43	0	2	1
11984	7872	5030	0	1820	1470	28480	561	118	653	35	278	158	58	248	303.43	144	453.41	514	217	401	79	84	3	26	10
13076	7828	4784	1	1350	988	28152	606	199	859	76	122	220	87	195	281.89	180	446.58	484	310	429	54	117	17	2	3
11818	7948	5262	0	1822	1340	28448	544	116	691	29	234	175	63	264	303.47	141	456.01	494	220	400	77	102	7	24	10
11938	7920	5276	2	1748	1268	28414	572	123	703	28	246	186	62	233	303.9	144	454.86	490	250	386	83	106	8	18	17
6002	12000	8654	521	934	580	28228	1005	27	647	8	58	72	15	133	285.06	96	441.96	400	330	353	26	38	0	0	2
14176	7768	4466	5	822	610	27900	663	316	1054	158	58	258	120	157	270.36	266	434.89	489	447	414	14	86	19	0	0
4020	13592	9888	952	356	192	28056	1295	8	578	1	8	67	4	69	272.03	98	425.83	280	272	268	16	50	0	0	1
12914	7984	4868	4	1252	982	28170	610	189	901	65	166	178	96	193	281.9	196	447.31	507	342	456	42	95	13	4	1
10868	7848	5570	0	2286	1724	28714	508	74	521	15	364	156	33	294	320.74	108	465.56	483	159	361	120	77	8	50	25
12112	7876	5070	3	1706	1360	28422	570	143	699	44	272	172	73	255	298.5	154	453.21	476	243	420	72	88	9	22	12
4104	13562	9850	916	348	184	28052	1257	9	583	1	4	68	3	73	273.75	90	427.4	293	282	282	14	41	0	0	4
5546	12528	9016	553	640	378	28144	1056	9	723	1	36	77	7	99	285.03	107	440.3	387	389	347	24	39	0	0	3
4016	13562	9868	942	392	220	28068	1283	8	554	1	10	62	4	66	272.63	76	426.03	294	272	271	17	38	0	0	1
14182	7756	4460	8	802	616	27888	647	318	1055	152	68	254	126	160	271.44	251	435.42	473	432	438	15	89	20	4	5
4180	13512	9816	907	346	186	28052	1243	6	592	3	12	69	3	82	273.63	85	427.85	284	277	301	13	46	0	0	4
12410	7848	5014	2	1652	1208	28330	590	158	733	46	184	186	77	237	297.23	152	452.58	483	262	396	65	92	15	14	10
12938	7786	4792	2	1424	1092	28212	594	185	818	64	166	212	88	221	287.37	177	449.07	491	305	409	51	115	12	14	4
4066	13594	9848	911	342	200	28060	1285	6	591	1	10	57	3	76	273.28	78	426.21	276	301	286	12	40	0	0	3
3790	13698	10022	1006	354	180	28054	1314	8	549	1	10	58	3	72	269.73	88	422.79	269	257	266	12	40	0	0	2
5002	12980	9328	683	500	290	28114	1127	3	705	0	14	56	3	79	276.76	111	434.17	366	341	350	15	40	0	0	4
5552	12414	9026	598	730	400	28158	1084	22	665	3	34	66	14	81	286.51	101	440.78	405	348	333	20	41	1	2	3
12022	8074	5120	3	1580	1304	28382	517	107	767	31	252	193	43	235	301.22	155	456.84	497	252	415	91	110	8	30	11
10632	7740	5620	1	2420	1906	28868	464	53	471	8	486	148	25	328	336.26	93	474.16	453	147	385	135	81	4	58	37
12932	7884	4858	4	1356	1006	28182	630	180	888	62	130	195	88	200	283.59	199	448.09	501	350	416	39	107	9	14	4
14400	7588	4398	6	824	590	27868	617	338	1060	174	62	273	132	136	270	281	433.53	517	424	426	23	92	16	6	1
12340	7864	5062	2	1612	1212	28322	591	164	754	48	208	172	74	262	293.7	157	451.37	451	265	429	55	93	17	24	11
3784	13672	9988	993	388	222	28072	1326	6	538	1	16	56	4	59	270.91	88	423.58	269	270	263	20	39	0	2	3
11260	8026	5410	0	2030	1540	28584	567	98	579	16	284	151	56	280	311.66	117	458.85	463	201	394	87	85	5	34	22
11754	7964	5218	4	1848	1420	28488	522	122	644	24	252	171	77	262	302.72	131	457.89	467	204	437	93	81	9	30	17
5092	12880	9278	684	540	308	28114	1114	9	672	2	16	70	5	83	278.75	123	436.83	372	324	333	21	45	0	0	1
3864	13688	9962	962	338	190	28052	1286	5	555	1	10	58	1	88	270.92	78	422.68	246	261	294	16	45	0	0	4
5356	12770	9146	614	516	302	28108	1067	9	734	0	16	81	8	94	279.41	117	436.38	393	364	344	15	56	0	2	1

SUPPLEMENTARY INFORMATION:Monte Carlo Atomistic Simulation and Machine Learning Analysis of Na-K Eutectic Alloy in Condensed Phases, D. Reitz and E. Blaisten-Barojas, George Mason University, Fairfax, VA 22030

4200	13558	9812	896	306	162	28040	1262	10	610	1	2	64	6	63	272.99	85	426.98	300	315	291	12	41	0	0	3
14340	7748	4388	7	748	584	27868	646	326	1087	166	60	256	131	143	267.89	297	429.45	498	440	432	16	82	15	0	1
4042	13530	9846	939	416	234	28078	1286	6	570	1	10	54	4	77	271.63	93	424.32	286	267	281	9	38	0	0	3
12374	8028	5076	3	1514	1120	28284	624	149	808	47	144	190	78	220	290.11	157	449.86	479	319	396	53	95	8	28	13
14476	7662	4324	4	746	588	27862	642	360	1042	162	58	247	152	163	268.33	267	429.29	468	434	456	15	74	20	6	0
5058	12956	9330	664	474	264	28100	1143	4	739	0	18	63	3	74	276.74	110	431.72	372	389	327	15	44	0	0	1
5594	12454	8954	544	688	432	28176	1063	7	721	2	52	59	2	105	282.58	108	438.64	402	379	346	16	42	0	2	0
8000	10774	7520	296	1112	754	28250	865	59	744	27	82	108	25	141	286.35	131	445.34	469	360	367	35	51	1	6	2
5002	12882	9320	673	568	326	28124	1137	6	675	0	26	64	6	81	278.42	105	435.76	378	358	322	9	50	0	0	5
12230	7962	5062	4	1618	1246	28344	608	151	752	50	204	168	64	234	292.93	161	449.72	489	279	403	54	73	14	22	13
13860	7678	4544	4	1026	772	27994	622	294	940	126	104	222	124	193	276.63	220	443.27	459	361	457	30	81	20	10	2
4104	13520	9804	926	396	236	28072	1282	4	583	1	12	60	2	79	273.82	88	427.87	274	282	284	11	41	0	0	6
3906	13714	9932	976	310	184	28052	1310	5	561	2	6	57	3	72	271.51	84	425.12	269	273	278	11	37	0	0	1
12416	7974	4966	1	1556	1218	28342	609	155	734	46	190	176	82	218	292.36	172	451.15	500	262	407	61	88	7	22	9
4492	13150	9526	762	574	368	28146	1189	2	622	0	34	62	2	84	277.78	95	433.15	339	331	294	16	35	0	2	7
14506	7534	4310	5	858	612	27872	623	351	1014	162	52	270	142	150	272.97	267	437.05	499	399	433	19	91	27	0	1
13086	7660	4702	4	1458	1124	28226	532	213	767	74	186	202	108	229	286.04	187	448.33	526	248	446	41	95	10	8	6
4240	13496	9780	913	340	188	28056	1276	10	610	1	12	61	7	71	273.55	98	427.22	293	294	280	11	35	0	0	2
11718	7936	5160	0	1848	1516	28526	500	119	625	26	310	200	65	255	310.54	132	459.71	498	184	386	102	102	9	36	22
5322	12736	9174	618	544	310	28114	1080	10	743	0	28	52	7	84	279.7	123	434.87	383	360	372	18	36	0	0	3
4542	13264	9586	786	440	246	28084	1189	4	636	0	6	57	2	87	273.77	94	428.67	319	313	326	15	38	0	0	1
11596	7846	5338	0	1968	1466	28540	532	105	616	25	280	186	54	261	310.85	125	459.01	491	208	371	93	94	5	42	23
4760	13088	9470	729	490	278	28108	1146	4	652	1	22	64	3	87	279.22	99	435.39	352	330	333	14	42	0	0	2
4744	13072	9460	730	518	296	28106	1187	5	669	0	14	61	3	85	279.13	90	434.17	328	358	310	19	38	0	2	2
4584	13208	9562	799	460	264	28098	1213	7	659	0	20	62	3	86	277.1	102	432.44	314	330	300	15	41	0	0	2
5586	12560	9036	618	580	322	28106	1088	30	673	8	22	86	18	90	282.37	105	438.51	380	350	329	19	48	3	0	2
13114	7758	4760	3	1344	1022	28174	592	211	810	68	172	185	103	227	282.12	169	447.29	477	301	453	42	91	14	4	2
13890	7746	4520	6	980	746	27972	651	252	1026	128	82	243	97	178	275.75	236	440.29	472	419	421	26	96	9	8	0
14498	7618	4320	3	790	588	27870	639	322	1067	160	56	260	127	147	272.27	279	437.27	494	429	437	17	87	18	0	1
4126	13508	9828	924	382	208	28064	1296	3	594	0	12	71	2	69	274.39	82	427.9	274	296	265	20	49	0	0	2
10626	7936	5716	0	2314	1730	28756	505	64	513	10	380	172	29	289	323.99	101	467.36	517	153	333	100	88	6	42	34
3732	13766	10028	1001	338	186	28054	1319	8	543	1	4	64	5	68	270.5	83	422.56	267	260	260	14	43	0	0	3
4014	13614	9894	958	338	184	28052	1294	7	601	1	8	57	3	67	270.89	97	423.82	275	277	284	16	41	0	0	2
14394	7698	4374	9	764	574	27854	649	318	1096	171	50	268	115	138	267.91	291	429.36	496	445	421	17	86	17	0	3
12668	7796	4898	1	1576	1160	28270	592	163	788	50	148	200	88	227	291.98	168	449.31	465	271	428	63	110	9	24	7
3910	13602	9922	980	404	224	28072	1310	8	544	0	8	68	6	71	272.17	88	425.65	281	255	250	10	51	0	2	5

SUPPLEMENTARY INFORMATION:Monte Carlo Atomistic Simulation and Machine Learning Analysis of Na-K Eutectic Alloy in Condensed Phases, D. Reitz and E. Blaisten-Barojas, George Mason University, Fairfax, VA 22030

13022	7814	4804	6	1358	1010	28152	605	208	857	85	130	195	86	188	281.75	185	445.89	511	320	433	44	85	19	14	2
4974	12890	9308	651	592	350	28138	1113	6	683	0	24	61	4	96	280.54	101	437.92	363	358	345	17	45	0	0	2
13432	7934	4760	4	1034	756	28006	648	253	1012	106	84	215	110	160	276.03	244	438.59	495	412	434	31	89	13	6	2
12770	7746	4864	3	1564	1146	28266	606	170	782	65	156	204	82	230	286.37	176	449.48	482	291	408	49	98	10	20	8
7312	11208	7962	367	1032	648	28234	935	48	735	13	68	97	25	127	287.23	136	445.04	470	364	334	18	59	2	4	6
10716	7804	5656	1	2364	1782	28800	533	49	491	7	432	147	29	289	325.43	94	469.72	474	163	370	119	86	5	38	34
4968	12776	9298	685	676	396	28152	1142	8	630	0	38	62	8	87	279.53	94	436.92	349	329	325	27	37	0	0	3
4880	13032	9402	723	490	278	28096	1147	10	675	1	14	61	6	97	278.05	94	434.94	335	341	339	13	44	0	0	2
3994	13668	9918	974	296	158	28038	1294	6	583	1	4	49	5	62	270.29	83	422.16	276	274	297	13	31	0	0	3
13668	7884	4654	4	958	722	27976	640	275	1011	121	86	228	126	182	275.54	222	441.4	444	396	461	31	96	16	4	5
12132	7874	5060	2	1722	1348	28414	530	149	710	36	260	167	82	251	298.65	153	455.04	499	226	433	75	89	9	18	10
4092	13526	9836	927	386	214	28064	1301	7	587	2	10	61	4	59	271.29	84	425.11	282	307	271	19	42	0	0	3
5980	12216	8728	494	746	464	28174	1000	16	736	6	38	75	8	126	281.99	117	440.12	409	359	368	15	45	0	2	1
10834	7868	5610	0	2248	1716	28732	488	60	559	13	420	174	26	297	327.36	121	468.88	506	162	338	115	88	4	30	27
10750	7928	5650	1	2250	1724	28762	555	53	524	13	408	149	28	279	323.39	87	465.16	455	192	372	113	67	1	52	39
13682	7784	4614	7	1064	774	27998	659	272	967	128	72	236	120	184	276.16	214	441.34	458	420	422	27	91	12	8	4
4376	13292	9664	804	470	270	28094	1180	8	596	2	22	67	3	90	275.29	87	428.74	335	302	307	9	36	0	0	3
10864	7902	5576	1	2212	1716	28716	535	84	530	21	386	167	40	287	315.63	95	460.89	500	190	346	97	86	6	60	29
4178	13470	9788	883	402	224	28074	1257	6	585	1	12	68	5	63	274.75	93	428.2	310	295	274	15	46	0	0	6
4288	13348	9688	827	468	284	28100	1212	6	582	0	24	71	5	86	274.74	75	429.26	311	297	297	15	44	0	0	5
4650	13142	9526	761	502	272	28100	1177	8	635	0	8	67	5	85	277.5	88	433.86	342	326	309	14	52	0	0	1
3982	13686	9922	965	280	158	28038	1290	9	591	1	10	57	4	72	271.14	93	423.53	273	284	286	11	40	0	0	3
5166	12780	9200	632	614	362	28140	1120	9	684	3	16	53	4	89	280.66	97	438.01	362	369	352	17	32	0	2	4
11424	7858	5384	2	1988	1532	28562	516	92	642	25	328	176	31	259	313.22	127	459.67	509	217	389	94	97	9	48	21
12144	7906	5062	3	1694	1322	28384	535	134	702	32	228	172	77	247	298.19	157	451.73	488	238	439	78	90	7	28	10
13592	7890	4708	7	992	714	27972	615	263	1016	126	72	230	108	162	276.21	261	439.91	496	380	461	27	91	15	2	3
11338	7894	5450	0	1976	1526	28586	534	105	610	19	370	186	56	284	319.18	107	462.7	453	217	362	109	108	3	28	23
3876	13688	9954	984	332	192	28056	1309	8	564	1	14	58	6	73	272.16	82	424.99	273	274	270	10	37	0	0	2
13834	7748	4554	5	1008	746	27972	628	284	980	142	76	247	104	168	274.14	238	438.15	489	385	429	30	86	14	4	1
4834	13104	9440	759	438	252	28084	1189	7	671	1	16	66	5	75	276.09	109	431.63	335	339	316	15	50	0	0	2
12770	7994	4914	0	1324	1032	28212	610	174	836	51	164	176	96	200	287.29	177	450.55	482	317	446	54	91	6	14	9
4098	13612	9840	914	310	184	28054	1268	10	590	1	10	59	8	65	272.27	89	425.14	285	296	299	19	38	0	0	2
12214	7820	5050	3	1696	1322	28378	547	144	714	37	244	178	75	232	298.89	143	453.57	475	232	441	82	93	9	32	15
4464	13278	9578	806	468	296	28104	1227	9	622	2	20	63	5	97	273.27	80	427.55	305	328	290	9	47	0	0	3
4316	13372	9718	849	416	236	28080	1253	5	606	1	22	59	3	59	274.59	87	428.65	314	326	290	16	40	0	0	5
4206	13404	9754	876	448	258	28090	1253	5	573	0	20	56	2	75	274.14	80	428.66	293	291	294	21	37	0	0	2

SUPPLEMENTARY INFORMATION:Monte Carlo Atomistic Simulation and Machine Learning Analysis of Na-K Eutectic Alloy in Condensed Phases, D. Reitz and E. Blaisten-Barojas, George Mason University, Fairfax, VA 22030

3888	13658	9928	957	360	216	28066	1288	9	552	1	16	59	6	78	271.57	86	425.01	271	273	275	14	37	0	0	5
4408	13290	9650	840	472	262	28092	1225	10	611	1	8	58	9	68	273.6	107	427.74	325	296	298	17	38	0	2	3
10964	8022	5448	4	2184	1690	28656	511	65	568	10	308	185	34	287	312.6	109	456.26	506	167	353	102	93	6	30	22
4900	12990	9376	694	522	302	28108	1135	9	654	0	18	62	9	87	279.56	104	435.09	352	344	336	21	47	0	0	0
4910	12994	9376	700	524	294	28108	1131	7	679	1	10	58	3	78	278.15	110	434.62	367	339	341	15	42	0	0	4
11228	7910	5516	0	2070	1530	28614	545	72	588	12	304	166	35	251	319.89	113	463.82	520	197	351	106	94	3	52	16
12702	7928	4840	2	1436	1158	28264	602	177	797	53	182	181	97	221	285.43	181	449.88	488	304	426	52	85	11	16	6
14012	7768	4458	5	896	722	27950	662	295	1025	145	86	240	119	176	272.29	227	438.55	459	430	429	24	82	16	6	1
4970	12902	9320	686	572	338	28128	1104	5	670	1	26	73	2	95	278.37	108	434.37	361	324	336	23	47	0	0	2
4626	13206	9558	766	434	246	28092	1180	5	649	0	20	55	5	74	277.5	103	432.88	342	332	328	15	37	0	2	2
11536	8076	5454	4	1766	1328	28474	554	98	655	21	280	168	48	248	309.38	121	457.11	487	225	404	78	95	7	34	19
14282	7620	4440	7	860	620	27900	646	310	1024	148	72	248	131	164	272.81	235	436.68	465	420	448	23	89	18	6	0
3878	13660	9932	964	368	218	28070	1295	4	530	0	14	63	3	64	272.62	79	425.27	284	269	272	18	39	0	0	2
14376	7638	4348	6	822	630	27880	629	340	1038	159	60	260	135	168	268.01	266	430.01	479	421	442	12	86	21	4	3
6348	11854	8448	418	912	606	28240	972	12	731	0	66	60	8	129	287.25	119	444.36	425	378	370	24	35	0	6	4
11756	7892	5316	1	1866	1364	28484	562	105	646	31	258	190	54	256	306.49	136	457.83	486	216	377	85	94	9	30	12
6434	11870	8442	426	846	554	28210	960	23	743	4	58	65	15	136	285.18	125	441.78	390	366	405	29	40	1	6	6
4146	13548	9818	896	340	194	28058	1252	10	590	2	12	56	5	64	272.37	93	426.63	306	293	297	17	33	0	0	1
4622	13122	9526	750	520	298	28112	1165	4	641	0	24	57	4	83	278.12	92	434.57	339	320	324	19	33	0	0	4
13086	7878	4870	7	1264	894	28112	616	222	878	78	116	205	107	195	282.88	207	447.91	474	321	440	47	107	17	4	4
5040	12936	9316	672	502	292	28108	1126	5	687	0	20	69	3	93	279.22	92	436.35	354	359	324	21	45	0	2	1
12666	7860	4886	0	1504	1158	28266	607	175	776	57	168	200	91	220	291.27	165	451.56	484	292	399	60	104	5	24	5
3956	13556	9880	932	428	248	28084	1294	7	563	1	16	60	4	67	271.83	78	426.17	289	287	265	18	40	0	0	2
13858	7716	4520	3	1002	780	27986	611	288	971	135	94	240	121	184	273.8	207	437.55	462	376	459	32	87	11	16	5
4296	13474	9736	892	342	200	28060	1278	9	617	1	10	66	6	75	272.91	97	428.5	287	313	276	12	45	0	2	2
5244	12766	9162	661	572	352	28122	1123	13	708	4	26	80	6	96	279.05	112	438.42	357	346	319	19	56	0	0	2
11320	7804	5568	1	2056	1474	28604	537	84	584	9	330	161	46	287	318.75	109	463.8	456	197	386	98	83	8	46	29
5216	12860	9238	679	492	280	28106	1123	9	720	0	20	57	6	73	280.22	123	435	370	360	351	21	37	0	0	1
11536	8006	5426	3	1850	1364	28480	540	95	663	15	266	193	48	290	310.23	122	458.05	446	226	380	92	112	6	30	12
4960	12978	9326	696	530	314	28120	1145	7	683	1	12	69	5	84	279.14	110	434.75	365	340	313	16	52	0	0	1
4786	13118	9428	753	470	284	28098	1152	4	662	0	12	67	2	75	276.04	115	431.53	355	319	322	18	46	0	0	4
7332	11194	7934	319	1044	672	28258	926	25	780	3	74	94	17	145	288.28	113	445.24	433	413	358	30	55	0	8	5
5252	12808	9200	616	538	306	28120	1091	5	721	0	16	54	3	96	279.96	117	438.31	367	363	359	18	39	0	0	3
3908	13680	9962	999	320	168	28046	1324	10	563	1	8	56	7	62	271.34	99	425.26	266	267	268	17	40	0	0	1
13334	7884	4684	5	1142	906	28084	631	231	925	105	124	226	91	189	278.68	189	445.81	498	385	409	33	96	14	10	2
11876	7804	5184	0	1886	1420	28466	591	132	653	32	270	161	57	256	302.1	123	456.1	467	247	385	80	79	13	26	17

5346	12752	9170	624	530	292	28106	1112	8	742	0	16	62	4	97	279.76	102	438.02	358	385	341	17	45	0	0	1
14222	7758	4472	4	786	582	27882	636	311	1037	154	60	248	115	150	273.46	251	437.4	473	405	454	27	83	14	2	2
3686	13822	10064	1017	296	166	28042	1298	8	544	1	8	57	5	69	270.18	84	422.67	266	241	289	15	39	0	0	2
4396	13312	9650	830	452	264	28092	1221	8	620	0	18	58	5	73	275.07	95	429.23	331	308	298	11	41	0	0	3
4754	13026	9448	784	568	314	28122	1194	8	623	1	12	65	6	73	277.91	108	434.04	346	310	299	19	42	0	0	2
12290	8028	5126	0	1528	1138	28318	623	137	789	36	198	177	71	213	292.49	170	451.17	494	295	396	57	100	7	8	13
5562	12510	9012	644	646	372	28130	1091	19	672	8	28	74	6	105	283.09	111	440.23	373	328	332	11	52	0	0	4
5658	12414	8910	570	732	432	28166	1090	10	728	1	18	64	6	111	283.17	104	438.28	380	371	332	12	52	0	2	3
4348	13390	9706	854	398	218	28068	1239	8	601	1	8	68	6	64	274.08	84	427.24	312	312	293	18	41	0	0	3
10798	7834	5536	2	2306	1820	28772	512	78	489	9	416	160	39	298	323.25	101	464.7	480	155	354	133	82	3	56	21
12098	7972	5154	0	1630	1264	28388	543	140	729	35	246	176	64	232	300.79	156	457.15	511	241	414	78	89	11	22	11
6560	11792	8340	445	884	580	28202	955	45	697	12	42	87	25	129	285.63	121	443.96	441	345	359	17	51	3	4	3
14286	7714	4402	4	800	620	27892	643	324	1078	156	60	241	126	157	269.46	286	431.82	477	435	456	16	76	20	10	3
6430	11906	8550	496	788	444	28154	1017	27	761	6	34	89	17	98	285.03	127	442.41	426	371	340	21	67	1	2	2
11176	7720	5468	0	2226	1678	28706	504	82	520	6	398	155	49	301	320.78	104	465.39	470	168	397	98	83	6	36	31
11848	7752	5232	2	1928	1432	28506	565	124	654	30	268	182	67	266	307.51	129	460.48	490	228	363	81	99	9	40	14
3858	13640	9942	973	386	228	28074	1308	3	557	0	20	66	2	74	272.13	88	424.47	277	263	252	16	46	0	0	1
13602	7716	4642	5	1154	818	28024	603	271	902	115	90	236	121	187	277.96	199	443.57	489	353	440	33	98	13	2	2
5052	12828	9234	684	630	382	28144	1153	6	656	1	18	65	4	99	278.64	91	434.3	342	342	318	19	40	0	0	1
13164	7884	4766	6	1236	946	28140	608	224	866	77	134	196	117	218	281.22	181	447.16	469	323	464	33	94	11	10	3
14398	7660	4352	2	786	608	27874	624	323	1074	161	70	265	128	162	269.26	281	431.47	483	435	442	15	92	20	0	3
6670	11582	8316	415	964	620	28248	968	23	686	8	90	94	10	147	289.27	97	447.28	421	363	338	21	57	0	6	3
4474	13284	9618	791	450	256	28096	1196	5	613	0	14	61	4	73	277.03	106	431.49	320	311	322	24	38	0	0	2
13562	7668	4594	1	1214	910	28078	612	241	862	96	124	218	98	205	278.76	188	444.75	470	331	444	38	94	24	6	1
4264	13408	9728	862	422	246	28086	1246	4	602	1	18	46	2	71	274.13	85	429.27	300	305	308	16	30	0	0	5
4524	13208	9596	787	484	268	28096	1198	4	635	0	12	68	2	85	276.18	102	432.02	325	327	300	16	48	0	4	2
13884	7730	4494	4	1000	776	27978	577	277	995	126	82	253	112	179	276.16	246	441.33	502	367	446	32	112	16	12	1
4036	13618	9886	952	328	176	28048	1288	9	589	2	4	67	3	72	272.37	96	425.16	287	280	268	11	39	0	0	1
4568	13262	9602	795	404	222	28074	1218	5	661	0	14	64	4	62	276.22	103	432.98	320	348	307	23	46	0	2	2
13760	7876	4622	6	940	694	27964	638	279	997	122	70	228	128	170	275.08	248	439.04	488	394	443	20	84	13	2	1
4206	13522	9774	898	340	206	28064	1251	7	609	2	16	76	3	69	272.84	101	426.21	309	297	275	15	46	0	0	2
4030	13596	9846	939	368	222	28072	1273	3	564	0	10	58	2	80	273.21	86	426.47	276	263	288	18	43	0	0	1
13052	7788	4664	2	1410	1124	28200	604	211	795	79	144	193	92	210	284.56	173	448.8	483	300	428	53	88	13	18	6
14120	7738	4496	7	844	624	27892	644	308	1062	162	70	268	115	172	270.44	238	433.27	471	448	426	15	95	16	0	1
14286	7724	4452	3	778	570	27870	628	314	1094	147	58	246	125	149	270.42	267	434.98	501	434	456	12	91	19	2	1
12484	7812	4926	3	1606	1256	28324	560	164	764	44	226	188	81	234	293.24	148	453.08	474	265	436	70	96	11	10	12

SUPPLEMENTARY INFORMATION:Monte Carlo Atomistic Simulation and Machine Learning Analysis of Na-K Eutectic Alloy in Condensed Phases, D. Reitz and E. Blaisten-Barojas, George Mason University, Fairfax, VA 22030

4158	13422	9758	884	470	276	28100	1230	5	572	0	16	53	4	66	272.69	95	425.58	328	280	302	15	30	0	0	3
10946	7854	5498	1	2248	1740	28722	514	77	512	14	398	155	40	321	318.6	76	462.78	452	163	389	110	84	5	34	24
11984	7908	5100	0	1800	1402	28464	555	114	689	22	222	183	66	276	304.17	133	456.87	445	243	409	82	101	6	46	18
5610	12548	9000	608	604	344	28124	1095	14	712	4	16	65	7	88	281.82	111	438.17	375	382	348	23	38	0	2	0
4376	13296	9646	851	472	284	28098	1214	5	605	0	24	68	2	84	275.36	89	430.23	312	282	299	18	47	0	0	2
5892	12088	8780	519	854	514	28196	1048	21	675	4	64	78	13	110	284.89	100	442.85	394	359	333	25	54	0	4	5
11584	7884	5382	1	1968	1406	28506	513	108	619	32	254	183	48	273	308.08	128	459.82	502	192	388	86	109	5	24	15
11542	7852	5330	0	1996	1500	28548	548	106	628	27	284	165	59	294	302.69	106	454.22	461	221	397	82	81	6	40	18
12280	7944	5046	1	1616	1238	28340	589	165	718	43	184	161	89	230	291.23	132	451.91	474	270	443	66	78	7	26	12
7122	11428	8066	365	932	608	28224	922	34	776	9	58	85	21	144	288.96	120	446.13	411	383	392	34	53	1	10	3
11150	7860	5396	2	2148	1700	28682	525	89	552	15	390	149	50	303	314.39	96	463.04	476	189	380	99	69	2	38	21
4736	13104	9484	754	484	272	28098	1164	7	646	0	18	61	5	80	278.13	83	435.43	332	325	336	23	40	0	0	1
4352	13366	9656	882	428	268	28090	1227	12	591	1	20	54	10	68	274.26	107	429.57	325	272	308	13	36	0	0	3
3892	13666	9928	977	356	212	28066	1313	7	542	1	10	63	3	72	273.16	84	426.56	282	276	254	9	38	0	0	2
5068	12780	9236	687	648	386	28142	1146	16	637	2	22	68	12	107	280.57	77	437.26	336	348	311	19	45	0	2	3
14398	7702	4408	8	742	546	27850	635	345	1073	155	52	254	149	161	270.11	261	433.12	459	428	465	20	97	22	2	0
12634	7974	4988	1	1406	1062	28248	585	161	825	38	164	185	89	227	289.86	172	450.47	492	299	430	52	117	14	18	5
10996	7796	5482	2	2210	1760	28726	511	91	509	15	430	161	42	288	319.95	93	466.05	470	152	362	124	92	5	38	31
12200	8160	5212	4	1464	1062	28268	612	161	834	41	164	168	91	234	286	165	449.48	474	313	434	51	97	9	6	4
4100	13456	9798	904	458	266	28096	1270	8	563	0	18	63	6	84	273.87	74	427.83	289	282	272	15	47	0	0	2
14450	7598	4374	5	812	574	27862	646	324	1078	156	54	263	134	130	270.41	273	434.58	493	455	435	26	96	20	0	2
4248	13474	9752	880	376	212	28068	1270	4	604	0	6	65	2	66	273.35	90	427.68	294	316	277	21	44	0	0	1
13046	7660	4756	2	1476	1092	28206	572	208	802	71	170	219	94	223	286.01	168	449.18	505	286	409	51	115	13	6	2
7034	11404	8108	364	1014	626	28242	938	24	784	4	54	86	14	132	288.48	136	444.34	441	383	363	31	60	0	2	1
4912	12912	9354	697	576	342	28128	1176	13	633	2	28	65	8	90	278.02	78	433.54	325	361	312	22	41	0	2	3
11822	7610	5126	0	2038	1590	28562	521	115	589	40	328	182	53	291	311.44	110	460.62	467	177	390	94	98	6	42	20
13374	7700	4624	2	1300	994	28138	618	254	836	93	134	218	115	203	278.96	190	446.33	481	324	415	45	96	21	12	4
4476	13266	9598	802	474	274	28098	1206	4	627	1	10	66	3	89	276.57	90	431.55	317	312	299	15	41	0	0	2
4022	13662	9882	953	300	174	28044	1294	8	595	3	4	49	4	78	270.12	78	422.54	257	288	299	14	30	0	0	2
13760	7876	4644	2	914	672	27940	631	294	1017	133	72	250	122	175	275.87	231	441.38	454	394	439	37	99	22	2	1
4292	13482	9730	892	346	202	28058	1254	12	612	2	6	72	6	76	273.6	97	425.78	306	293	276	9	50	0	0	2
14000	7828	4522	6	846	648	27914	625	282	1068	147	68	256	108	163	273.2	252	438.42	479	405	447	24	99	15	2	2
14148	7750	4452	6	838	646	27906	665	315	1050	151	64	247	125	173	271.04	249	433.34	448	433	433	19	81	19	8	4
5938	12348	8784	528	642	398	28138	1019	13	757	3	26	79	6	125	282.21	118	439.95	378	378	372	19	53	0	2	1
4526	13234	9584	780	464	270	28098	1217	5	632	0	20	66	3	88	277.4	83	432.69	300	349	303	19	39	1	0	3
7140	11324	8028	343	994	664	28244	923	35	766	7	88	88	24	135	287.77	136	445.23	443	370	375	27	57	1	6	4

12626	7858	5024	4	1462	1078	28262	571	163	805	44	196	190	80	222	292.02	178	450.74	498	275	420	58	112	20	18	9
5590	12446	8938	563	712	446	28178	1073	13	711	1	46	57	4	109	284.23	121	438.5	396	373	334	14	39	0	0	3
4114	13572	9868	938	320	162	28044	1284	7	615	1	8	65	4	65	273.13	91	427.91	282	301	282	16	49	0	0	2
13270	7852	4744	3	1200	906	28104	563	217	935	80	120	222	108	205	283.41	212	445.96	487	327	464	45	122	14	12	2
4224	13494	9768	888	358	208	28064	1245	4	617	0	10	63	2	67	273.01	107	426.7	312	297	290	15	45	0	2	2
5590	12474	9006	624	662	372	28130	1103	24	680	10	22	86	10	81	283.21	113	438.54	400	357	307	17	48	2	4	2
5324	12706	9156	609	594	330	28124	1081	9	720	0	12	65	6	95	278.9	117	434.71	376	365	356	15	47	0	0	5
13586	7930	4672	10	988	744	28004	661	274	998	101	78	211	135	184	279.22	214	442.9	447	416	452	31	95	18	6	2
3926	13668	9916	986	332	202	28060	1319	4	551	1	16	61	3	70	271.09	78	424.23	261	261	266	16	45	0	0	3
14498	7522	4250	2	874	676	27894	642	331	1022	166	70	274	125	147	271.97	263	435.49	505	421	407	21	96	24	4	0
14220	7616	4344	5	928	736	27932	619	313	1002	168	76	258	110	166	272.02	260	437.44	494	391	436	22	76	19	12	1
4514	13230	9606	803	484	256	28094	1201	6	620	0	2	59	5	74	277.72	94	431.31	332	299	310	19	41	0	2	0
11570	8020	5218	1	1886	1516	28530	570	96	637	22	296	172	51	244	303.42	129	453.89	502	237	374	90	89	5	20	19
4902	13058	9416	711	454	248	28090	1162	5	703	0	12	68	2	90	276.85	107	433.7	339	359	320	15	51	0	0	0
5314	12692	9148	643	590	352	28132	1081	11	686	1	32	67	7	104	279.13	118	435.99	368	332	349	21	45	0	4	1
4274	13378	9712	875	438	264	28092	1259	7	586	1	26	49	4	78	273.18	79	426.85	290	301	301	19	33	0	0	0
11278	7944	5358	0	2034	1612	28610	519	103	575	28	356	171	49	266	305.55	119	458.45	489	192	395	107	86	3	28	19
4208	13476	9756	883	390	234	28080	1263	6	588	1	16	52	3	69	272.25	87	425.65	307	294	291	13	31	0	0	1
5356	12636	9076	625	656	402	28154	1079	12	654	2	26	67	9	107	281.23	97	438.63	368	332	353	18	43	0	2	3
3968	13610	9894	973	374	210	28062	1296	10	554	2	6	62	6	65	271.02	91	425.11	273	265	277	20	38	0	0	3
5358	12694	9150	625	560	326	28124	1088	8	717	0	32	59	6	99	280.95	108	439.17	358	366	372	17	37	0	4	4
12230	7952	5102	3	1600	1214	28326	561	153	759	39	208	151	78	219	292.28	168	450.27	507	260	457	61	82	14	20	12
5218	12674	9148	640	682	410	28162	1121	11	654	1	28	72	9	103	281.97	86	437.11	364	351	320	17	46	0	2	2
7092	11324	8062	379	1020	654	28230	915	45	729	14	76	88	19	134	287.33	130	444.39	442	359	370	33	49	1	2	2
7328	11086	7900	344	1130	726	28256	895	46	702	10	82	95	27	156	291.43	107	446.62	412	344	382	38	58	2	4	8
5874	12202	8788	510	798	480	28186	1056	16	718	2	40	79	7	113	283.22	106	439.18	385	383	329	26	47	0	4	3
13672	7726	4642	6	1110	774	28004	618	282	928	115	72	222	117	186	275.57	200	442.89	468	361	448	35	94	19	8	3
6800	11686	8326	436	800	494	28170	967	36	770	8	60	83	20	105	287.1	139	445.48	417	364	385	31	44	1	2	3
12646	7858	4806	1	1546	1236	28290	572	174	762	55	178	176	87	225	285.98	171	448.35	500	256	445	58	92	12	16	5
14170	7680	4452	5	896	646	27898	632	315	1033	162	48	262	113	153	269.58	263	432.66	508	414	421	17	89	18	4	0
5834	12220	8796	523	798	494	28194	1049	11	731	3	46	68	4	109	285.57	124	440.25	404	374	338	19	40	0	6	3
14300	7620	4402	4	876	632	27892	632	345	1001	170	62	259	133	146	270.31	262	435.15	517	417	424	14	76	24	0	1
14346	7666	4344	4	846	636	27886	655	316	1058	158	48	252	124	154	270.51	256	433.12	479	440	440	16	86	21	0	1
4400	13344	9678	851	414	226	28072	1244	6	628	1	10	61	3	72	273.98	93	428.04	312	315	289	12	41	0	0	3
11148	7884	5486	1	2132	1616	28652	514	81	576	12	332	175	46	284	317.64	117	464.54	465	176	369	123	91	5	44	24
12496	7908	5010	1	1526	1150	28298	582	148	813	50	184	204	64	216	299.12	185	454.18	491	283	399	65	121	11	24	13

SUPPLEMENTARY INFORMATION:Monte Carlo Atomistic Simulation and Machine Learning Analysis of Na-K Eutectic Alloy in Condensed Phases, D. Reitz and E. Blaisten-Barojas, George Mason University, Fairfax, VA 22030

4198	13554	9824	897	302	156	28040	1264	4	619	1	6	67	2	75	276.07	90	429.55	290	309	286	10	46	0	0	2
4668	13080	9488	761	548	316	28122	1151	6	618	0	22	62	5	90	276	96	433.02	342	295	326	17	44	0	0	5
11522	8066	5386	2	1820	1396	28504	504	93	696	22	284	181	51	276	311.18	151	460.36	514	235	389	81	98	4	26	14
11976	7928	5118	1	1734	1392	28454	579	130	703	28	266	164	69	245	303.27	154	456.35	466	247	408	91	85	6	38	14
4474	13312	9632	824	418	234	28080	1206	6	618	0	8	66	5	75	276.13	88	431.2	330	302	301	11	41	0	2	5
5270	12748	9158	630	584	350	28136	1106	9	675	1	26	70	4	94	280.42	108	436.98	364	348	334	21	41	0	0	4
3882	13704	9948	980	324	188	28054	1324	7	570	1	6	71	5	61	271.54	94	424.32	268	282	252	18	48	0	2	2
5016	12972	9338	684	494	274	28104	1148	6	708	1	10	60	2	80	278.94	115	434.36	366	369	328	9	31	0	0	4
4222	13572	9812	917	280	146	28034	1267	8	625	1	2	62	4	74	273.19	103	426.95	285	293	291	11	43	0	0	2
4206	13512	9780	889	354	200	28058	1270	9	604	1	6	57	4	68	273.6	77	427.68	292	312	284	18	34	0	0	1
4010	13676	9900	962	278	164	28040	1288	8	588	1	12	68	6	70	272.02	76	425.14	269	279	283	16	48	0	0	3
3840	13676	9964	995	368	208	28068	1335	8	547	0	12	60	7	76	270.37	86	422.87	256	264	254	14	41	0	0	1
14128	7652	4414	4	954	718	27940	638	313	975	152	66	246	121	173	272.16	235	436.09	475	389	440	19	85	19	8	3
5068	12772	9266	707	648	364	28140	1166	10	614	1	22	59	6	82	282.49	97	438.8	351	327	311	21	39	0	0	3
4396	13228	9638	825	534	302	28120	1215	5	601	1	22	61	3	86	278.53	90	434.72	316	290	295	17	38	0	0	4
11832	8044	5398	7	1660	1182	28362	543	119	793	29	224	176	63	258	299.69	148	454.51	467	281	443	69	100	8	22	12
4358	13388	9692	873	398	224	28068	1250	8	631	1	8	62	5	68	273.11	105	427.43	306	299	290	14	44	0	0	4
14300	7594	4378	5	908	660	27904	647	324	1011	155	64	255	126	169	270.65	241	432.78	467	408	426	22	89	26	0	2
4898	12942	9394	712	556	306	28120	1161	6	662	0	24	61	5	90	276.72	101	433.87	340	342	328	15	46	0	0	3
4196	13512	9806	910	348	184	28052	1280	7	613	0	6	62	4	74	273.59	81	426.21	274	309	287	16	41	0	0	2
12812	8012	4932	3	1288	972	28162	636	184	906	52	134	174	94	197	285.19	197	445.6	484	339	447	40	95	22	12	4
13704	7772	4610	4	1052	774	28006	624	284	953	118	88	221	134	178	276.81	224	442.27	466	382	467	33	82	14	6	4
5058	12858	9302	684	552	316	28114	1124	12	695	1	26	70	7	89	279.67	106	436.72	366	339	325	18	49	0	2	1
4724	13186	9502	792	422	238	28082	1213	11	663	2	10	50	6	80	274.04	100	429.81	319	343	319	12	38	0	0	1
5168	12888	9242	654	494	294	28106	1131	8	725	1	20	60	4	69	279.56	106	436.65	376	383	332	21	47	0	0	2
13508	7732	4668	0	1142	870	28070	593	263	899	104	138	225	109	201	282.48	193	447.76	479	339	434	44	97	24	12	2
7524	11026	7730	334	1106	786	28286	904	40	716	3	106	86	26	167	288.69	108	444.96	410	348	379	39	54	2	8	3
10724	7910	5660	0	2288	1734	28758	500	76	489	15	374	169	35	301	325.73	103	465.87	486	127	352	123	78	2	62	23
4216	13386	9754	881	460	260	28096	1244	6	576	1	16	59	2	70	275.07	82	428.95	316	279	285	19	44	0	4	1
5474	12644	9042	598	592	360	28134	1079	8	735	2	20	66	3	102	281.11	117	437.5	375	371	353	18	41	0	2	1
13432	7720	4708	4	1234	876	28086	603	235	874	85	106	217	113	190	282.69	185	447.68	475	321	446	46	100	16	10	5
13376	7974	4776	8	1018	768	28018	629	257	991	114	104	224	100	178	277.36	232	441.44	489	400	425	32	91	20	2	3
4734	13184	9464	776	426	266	28090	1207	2	695	0	14	56	2	72	277.43	112	432.43	324	343	320	16	39	0	2	3
5188	12862	9240	651	480	296	28102	1107	10	720	1	32	61	7	89	278.87	118	435.57	356	363	359	21	40	0	4	1
4608	13072	9548	763	578	298	28114	1192	7	621	0	10	51	4	77	277.95	81	432.43	328	317	322	22	41	0	0	2
5114	12856	9246	694	562	324	28110	1140	8	675	5	8	71	3	100	279.55	93	436.26	336	349	327	18	42	0	0	2

6646	11708	8266	426	894	628	28222	958	31	712	7	72	77	19	130	286.8	119	444.27	420	359	373	28	36	1	8	4
4352	13366	9712	860	418	218	28072	1230	17	581	2	6	56	9	63	273.9	89	428.89	323	292	299	19	37	0	0	1
4234	13448	9754	875	386	230	28074	1229	9	597	2	20	61	5	78	274.01	83	427.16	311	289	302	10	41	1	2	4
4602	13152	9530	777	518	296	28114	1178	1	646	0	16	63	1	84	278.27	102	434.01	343	317	314	15	46	0	0	1
4822	12980	9380	695	598	348	28140	1148	6	619	0	8	52	4	99	279.05	91	434.24	339	335	336	19	35	0	4	1
3970	13600	9884	932	384	224	28074	1257	3	560	1	10	74	1	71	272.11	98	425.05	305	258	275	15	48	0	2	2
4522	13206	9586	813	500	274	28096	1237	3	607	0	8	65	2	79	275.59	84	428.97	311	320	284	13	40	0	0	5
13748	7842	4568	5	974	766	27996	635	261	986	119	86	224	107	186	274.68	232	438.92	466	407	450	23	87	14	12	4
5456	12526	9006	606	708	438	28170	1120	9	702	0	34	57	5	102	281.68	112	438.05	364	376	324	20	39	0	2	3
4328	13478	9714	881	328	200	28062	1252	8	639	3	14	60	2	82	271.97	98	425.82	295	309	297	8	38	0	0	2
7840	10966	7622	295	1022	708	28246	878	39	812	10	78	106	22	152	288.36	152	447.09	425	382	383	43	66	1	10	2
4350	13406	9710	882	382	210	28066	1270	11	632	1	6	61	8	70	272.1	89	427.17	283	329	290	21	40	0	2	1
4116	13524	9798	871	394	238	28086	1241	3	578	0	16	48	2	66	274.89	84	428.38	313	290	307	14	28	0	0	5
12308	8008	5084	4	1580	1150	28294	604	159	776	40	146	171	86	234	286.9	171	450.67	478	273	439	49	95	15	18	7
11558	7694	5304	0	2108	1576	28608	505	95	578	15	324	175	54	308	311.84	91	461.2	459	183	399	98	101	4	38	18
14218	7724	4438	3	822	624	27896	609	317	1051	147	68	235	134	167	272.65	263	437.55	480	418	479	19	72	11	0	1
11722	7958	5178	0	1838	1492	28522	559	101	637	18	292	187	56	278	304.69	131	455.33	492	222	365	71	111	11	36	21
12772	7954	4872	1	1352	1088	28236	602	162	862	54	168	197	85	223	285.67	164	448.68	451	326	439	63	108	8	28	5
14406	7640	4370	5	794	594	27870	630	332	1092	162	64	257	131	151	270.56	271	433.46	497	436	442	16	88	21	2	0
4098	13480	9794	929	432	264	28090	1287	8	569	1	20	64	5	76	271.44	91	425.44	295	282	262	10	50	0	2	3
5240	12726	9164	635	610	368	28138	1085	6	688	1	28	60	4	91	280.84	107	436.45	370	339	356	27	38	0	2	2
3842	13758	9960	992	302	184	28052	1328	6	563	1	6	56	3	62	271.12	83	423.4	267	278	262	15	33	0	0	3
11498	8014	5432	1	1878	1380	28504	573	90	685	19	284	173	48	258	303.98	125	455.94	465	239	392	93	100	7	16	16
12414	7862	5090	3	1594	1154	28334	520	166	722	44	200	204	79	261	308.52	158	456.95	482	234	414	76	111	9	18	8
6318	12146	8594	434	654	406	28152	1005	15	843	5	32	73	8	103	282.03	123	438.45	409	450	380	22	41	0	2	3
5056	12826	9260	679	606	364	28142	1182	7	687	0	28	58	6	92	279.88	86	435.64	331	378	307	21	41	0	2	2
4292	13434	9728	870	372	220	28064	1242	12	613	1	18	63	7	68	272.95	96	426.5	317	305	284	12	41	0	0	4
13718	7592	4534	0	1168	900	28064	586	252	929	95	140	215	120	197	276.96	223	443.8	481	345	462	38	94	18	10	2
14294	7692	4390	7	796	632	27890	651	319	1075	165	84	254	117	168	270.02	259	431.77	468	451	437	15	81	17	2	1
4622	13166	9516	773	498	292	28110	1194	5	652	1	16	55	3	83	274.85	113	428.34	328	321	318	16	35	0	0	2
14132	7656	4432	7	946	702	27944	635	311	983	146	72	244	121	157	274.32	258	437.71	493	388	435	28	79	20	4	0
5762	12374	8888	568	676	406	28146	1069	14	719	1	40	79	10	107	285.67	103	439.94	375	372	336	21	48	0	0	4
10828	7812	5582	0	2286	1778	28780	505	81	479	14	442	165	43	314	322.62	81	465.76	482	156	351	119	87	2	52	24
14134	7680	4420	4	908	706	27936	605	325	1005	148	80	240	131	142	270.78	266	435.09	533	382	451	23	86	23	8	0
12514	8026	4978	3	1390	1124	28248	593	177	826	49	196	191	93	254	286.58	180	448.26	476	297	408	40	102	4	20	6
13812	7784	4624	5	960	692	27958	639	277	1017	126	82	233	115	167	275.44	264	438.85	480	378	439	27	98	17	4	2

4972	12914	9340	714	556	314	28114	1176	9	671	0	16	67	6	83	278.36	95	434.24	334	359	308	23	47	0	2	2
12028	7998	5154	1	1606	1300	28386	589	120	779	31	270	189	60	229	294.24	172	451.81	515	274	375	59	107	9	30	18
11940	7984	5236	1	1726	1274	28406	564	135	719	33	232	169	77	251	300.57	157	452.51	502	245	412	64	88	7	12	11
7032	11598	8124	359	856	550	28196	912	24	826	3	34	87	12	138	288.58	150	444.83	452	397	377	19	61	2	2	1
4126	13454	9784	885	448	262	28090	1250	6	582	1	16	57	4	78	272.15	88	426.03	300	295	293	12	34	0	0	5
5344	12786	9172	641	494	282	28098	1111	8	735	0	20	63	5	87	280.66	115	436.63	366	379	350	16	35	0	0	1
3934	13712	9964	980	278	140	28030	1313	7	588	1	2	72	4	69	269.69	93	421.96	260	284	265	15	49	0	0	2
5022	12930	9316	672	518	304	28112	1135	9	684	2	18	68	4	88	280.8	96	436.75	356	358	328	14	47	0	4	5
13058	7796	4720	3	1376	1078	28200	606	210	805	64	162	196	103	187	281.98	173	447.39	513	320	416	58	93	16	8	3
5962	12370	8792	530	594	372	28124	1070	17	775	3	34	70	12	115	281.57	122	437.71	364	411	358	15	49	0	0	1
13238	7770	4682	2	1310	1000	28142	604	225	850	69	134	198	115	193	282.85	200	446.39	480	312	450	52	100	20	8	4
4708	13180	9496	768	438	256	28092	1199	7	675	2	12	70	5	87	276.63	113	431.75	323	343	301	16	45	0	2	0
4084	13512	9784	922	416	272	28092	1279	2	571	0	20	61	2	80	271.86	79	426.34	286	287	276	14	34	0	4	2
4072	13546	9868	940	374	194	28062	1312	5	586	0	8	58	4	71	270.81	75	423.65	268	304	272	15	40	0	0	1
10740	7960	5626	0	2234	1726	28732	487	69	511	14	404	176	40	298	322.86	90	467.81	482	171	374	94	77	4	40	49
13922	7710	4502	4	988	756	27978	610	278	1007	134	94	238	113	176	275.94	256	439.3	496	365	442	21	88	15	6	4
6928	11458	8160	377	990	630	28236	921	33	776	6	62	77	20	132	288.31	142	445.5	455	364	377	27	49	1	8	0
12448	8022	5034	4	1474	1114	28274	563	160	817	39	166	181	82	220	293.57	197	449.04	524	266	428	45	104	10	14	12
7756	11050	7770	301	986	596	28204	888	61	800	7	40	85	44	136	289.45	139	445.68	437	398	405	27	50	2	6	8
4526	13116	9560	767	572	326	28126	1164	6	595	1	26	56	3	80	279.75	93	435.57	360	294	314	15	37	0	0	3
13132	7862	4832	7	1268	908	28124	634	217	887	79	116	194	105	194	281.88	202	446.61	483	351	444	34	86	15	4	5
4666	13130	9506	760	510	286	28110	1189	6	626	0	10	52	3	77	277.7	95	432.7	344	325	313	15	35	0	2	2
4412	13366	9650	842	404	236	28076	1236	5	636	2	8	68	3	80	275.96	101	429.45	304	313	289	13	46	0	0	4
3904	13690	9924	980	322	202	28060	1305	7	571	1	18	53	4	69	271.2	96	423.06	278	270	272	14	37	0	0	1
14134	7716	4478	6	864	638	27898	611	327	1034	157	64	259	129	157	270.24	257	434.48	498	401	448	21	89	20	4	1
13930	7582	4368	2	1128	912	28050	582	269	877	113	120	226	107	191	276.6	216	445.79	487	315	449	42	90	22	6	4
10528	7854	5730	2	2316	1850	28852	500	53	506	5	512	151	28	291	334.48	85	474.69	497	165	347	118	82	5	50	43
12004	7884	5234	1	1718	1284	28406	582	137	711	26	246	168	80	242	306.82	138	459.63	473	263	402	78	91	5	32	14
13024	7936	4818	3	1230	974	28152	612	194	910	77	158	198	87	209	282.83	205	446.41	470	333	448	40	91	10	12	7
4604	13162	9566	788	490	258	28090	1195	10	629	0	10	59	7	73	274.41	95	429.83	327	331	317	22	40	0	0	1
4812	13078	9440	732	488	272	28100	1151	3	669	0	10	72	2	84	276.23	112	432	344	331	323	21	47	0	0	1
4362	13452	9724	870	330	176	28048	1233	6	642	2	4	70	3	73	274.21	108	428.32	308	310	295	13	48	0	0	2
5256	12792	9158	637	552	344	28128	1132	8	704	1	26	64	4	91	281.23	102	437.79	365	376	318	15	46	0	0	4
4010	13624	9914	971	328	166	28046	1298	5	600	1	4	57	4	61	271.56	102	424.85	280	269	283	15	42	0	0	2
10660	7976	5654	0	2264	1744	28744	534	64	502	8	410	161	27	280	321.01	86	463.54	490	166	339	138	85	6	30	12
4332	13396	9716	850	400	216	28066	1238	8	605	0	6	56	5	81	275.18	81	429.7	293	314	305	16	41	0	0	2

SUPPLEMENTARY INFORMATION:Monte Carlo Atomistic Simulation and Machine Learning Analysis of Na-K Eutectic Alloy in Condensed Phases, D. Reitz and E. Blaisten-Barojas, George Mason University, Fairfax, VA 22030

4382	13376	9670	859	392	234	28070	1254	11	625	1	16	65	6	71	271.86	88	424.52	309	325	282	10	43	0	0	4
4610	13256	9596	792	380	204	28062	1199	10	663	0	14	69	8	67	276.21	101	431.56	322	341	316	20	47	0	2	3
4014	13642	9886	928	318	184	28054	1271	9	587	1	10	60	6	65	272.34	88	424.91	295	292	289	13	35	0	0	3
11264	7814	5458	0	2152	1586	28648	519	79	577	20	338	165	37	290	324.15	103	464.94	454	187	380	120	87	3	34	22
4660	13154	9544	774	474	248	28088	1179	10	647	1	6	59	7	86	278.39	96	435.44	331	326	326	11	39	0	2	3
14382	7620	4268	6	876	700	27916	671	304	1025	165	68	260	104	163	272.54	259	437.39	470	424	405	22	84	19	2	1
4314	13444	9736	865	368	194	28056	1230	12	620	2	0	58	6	69	273.86	91	428.94	312	290	309	15	45	0	0	1
14274	7684	4396	3	838	638	27900	628	325	1035	151	68	233	140	160	269.45	269	432.24	488	395	470	14	73	21	2	1
14170	7760	4456	10	812	624	27890	670	310	1060	161	62	259	110	143	269.93	267	432.54	487	428	413	21	89	22	6	1
4202	13410	9762	889	442	252	28088	1236	8	571	1	20	66	5	80	273.24	89	427.77	300	266	292	17	42	0	0	4
4612	13096	9530	785	538	306	28108	1228	4	629	1	26	66	2	74	275.77	91	431.76	340	336	263	15	47	0	0	2
5052	12966	9342	706	468	252	28092	1152	6	718	0	12	70	5	77	278.11	131	435.09	349	353	323	21	52	0	0	2
14176	7566	4320	2	984	804	27970	638	316	962	156	116	249	121	183	274.09	221	438.46	456	396	434	31	82	16	4	2
6236	12050	8580	481	762	496	28184	1037	19	753	4	58	80	12	114	287.94	114	444.7	386	386	344	27	52	0	2	3
4318	13452	9754	895	346	176	28050	1251	6	606	0	4	73	5	84	274.12	92	427.34	283	291	290	13	55	0	0	2
13104	7886	4718	3	1264	1030	28178	614	201	887	65	162	188	108	225	281.12	204	446.55	469	332	449	38	98	13	14	3
7240	11266	8010	379	1008	628	28218	912	43	717	14	62	100	24	127	286.54	136	445.12	451	336	361	31	54	1	4	5
13190	7822	4718	1	1284	990	28152	608	204	863	66	134	185	97	184	284.55	196	447.16	488	312	451	58	95	14	12	3
13072	7746	4672	4	1382	1132	28208	594	208	801	79	182	204	97	213	285.19	193	449.45	503	303	410	43	88	9	22	9
13586	7850	4662	7	1028	784	28020	601	246	990	101	98	231	105	178	277.24	238	443.55	493	375	440	36	107	18	10	3
13772	7736	4586	9	1046	762	27992	642	256	988	123	86	250	98	166	278.14	238	443.89	484	394	415	32	109	20	4	4
4624	13216	9538	790	440	256	28088	1214	9	639	0	14	58	7	72	275.13	97	429.16	332	329	302	16	40	0	0	0
3996	13584	9872	953	376	224	28070	1284	12	568	1	18	63	8	75	273.06	88	427.01	283	269	268	14	41	0	0	4
5248	12710	9156	615	636	374	28146	1096	6	689	1	20	64	3	87	281.48	105	438.37	383	357	337	22	39	0	2	3
12536	8044	5036	3	1430	1046	28240	575	160	832	43	128	192	79	214	294.93	177	451.43	489	294	442	56	101	18	20	9
3866	13654	9942	962	374	218	28068	1280	8	546	2	12	54	3	78	270.8	84	423.57	276	255	291	14	35	0	2	2
12504	7954	4958	1	1498	1166	28280	599	168	815	56	182	185	86	217	286.14	169	450.58	506	315	423	49	96	10	16	6
4136	13558	9832	930	332	184	28052	1266	12	588	1	10	47	7	64	271.98	94	425.3	289	276	308	18	36	0	0	1
10700	7964	5694	2	2220	1704	28740	508	61	518	10	396	170	33	294	325.34	96	469.9	481	158	359	113	83	4	60	23
4398	13254	9642	804	506	290	28112	1205	7	612	0	20	50	3	85	276.02	93	431.97	316	311	316	19	37	0	2	2
11536	7882	5426	1	1974	1396	28500	520	109	616	24	258	188	50	269	312.51	126	460.01	487	193	374	94	97	9	26	19
4218	13540	9782	906	310	188	28056	1249	5	609	0	18	58	2	67	273.68	98	426.56	306	285	299	11	34	0	0	4
10700	7692	5678	0	2464	1802	28806	479	62	491	6	416	181	32	276	329.86	83	469.48	498	153	345	134	99	4	50	38
12276	7864	5000	3	1696	1298	28360	556	145	711	50	206	190	75	235	294.46	149	451.99	493	241	418	70	92	10	18	11
14116	7682	4418	6	926	710	27928	647	313	990	155	72	250	117	175	270.46	259	433.25	467	387	436	18	79	25	4	2
4844	12930	9388	735	612	340	28128	1130	5	624	1	14	67	3	102	280.65	102	437.86	355	290	325	14	43	0	0	1

SUPPLEMENTARY INFORMATION:Monte Carlo Atomistic Simulation and Machine Learning Analysis of Na-K Eutectic Alloy in Condensed Phases, D. Reitz and E. Blaisten-Barojas, George Mason University, Fairfax, VA 22030

11250	7894	5518	0	2082	1520	28626	518	83	622	14	326	173	44	275	316.79	127	462.89	480	183	387	108	106	4	32	23
4418	13238	9634	814	514	288	28110	1225	5	595	1	18	52	2	74	276.36	83	432.72	326	315	297	18	35	0	0	1
6242	11856	8578	431	938	550	28218	992	20	726	6	52	69	7	116	287.67	110	443.76	419	383	362	25	37	2	2	5
13510	7692	4636	2	1210	908	28098	616	235	881	85	132	220	107	207	281.81	189	447.38	474	340	426	38	107	18	8	3
4168	13468	9788	890	406	232	28078	1260	3	572	1	14	56	0	67	272.3	82	426.29	303	294	289	16	44	0	2	3
4998	12836	9274	641	644	380	28152	1120	4	669	0	18	55	3	84	278.39	107	434.37	375	358	338	20	32	0	2	3
11144	8016	5482	2	2050	1554	28586	525	81	599	14	310	185	44	269	316.6	123	460.95	489	199	366	104	95	6	20	24
5102	12772	9230	650	628	380	28148	1115	6	695	0	34	62	4	112	279.39	115	437.92	350	340	338	14	40	0	2	3
4426	13220	9606	803	540	314	28126	1207	6	576	0	20	52	4	85	278.55	82	432.55	331	297	308	10	29	0	0	4
4414	13294	9660	823	460	250	28090	1229	5	615	1	12	59	4	81	275.88	78	432.29	303	324	303	19	34	0	0	1
12684	7932	4906	3	1414	1110	28244	626	177	803	45	182	159	91	226	289.78	164	448.19	451	309	444	59	80	17	16	5
11400	8030	5420	0	1900	1456	28540	492	102	644	17	298	185	48	286	317.57	118	462.49	467	195	396	108	99	11	32	17
4170	13586	9808	898	300	176	28046	1263	4	613	1	4	66	2	75	271.03	87	423.41	283	313	293	14	45	0	2	2
6144	11920	8610	478	922	558	28212	1030	23	705	4	58	69	12	115	285.81	113	443.19	416	378	332	23	39	0	0	2
12112	7850	5132	0	1760	1308	28420	567	135	694	43	238	178	68	259	303.49	140	454.47	481	247	395	72	94	4	18	12
13464	7716	4628	2	1244	924	28098	610	254	868	99	118	223	115	204	282.04	210	448.23	489	314	422	33	104	19	4	3
5146	12762	9218	670	616	362	28130	1109	17	647	3	26	67	8	106	283.25	93	437.46	359	333	334	17	41	2	0	0
3798	13832	10026	990	230	132	28026	1302	6	581	2	8	66	4	53	270.04	91	422.53	279	277	275	17	40	0	0	3
4876	12954	9376	721	576	326	28122	1171	8	652	0	12	62	5	78	276.26	92	433.66	348	336	310	23	51	0	2	1
13600	7880	4680	6	962	746	27984	622	271	1015	130	112	218	109	180	274.27	250	438.4	484	389	460	23	76	12	4	1
13950	7654	4542	9	1022	718	27966	619	291	996	148	74	246	107	166	274.2	246	438.55	504	393	435	21	87	19	6	3
4468	13344	9654	854	372	208	28060	1239	8	628	1	14	66	4	89	274.74	87	428.86	284	313	298	11	45	0	0	5
12092	7856	5046	1	1776	1378	28410	561	152	679	33	244	153	84	239	296.35	148	452.66	514	214	423	70	85	12	18	6
14504	7632	4346	4	768	552	27844	637	333	1075	173	42	270	118	143	268.13	281	430.06	496	434	430	15	93	22	0	2
4924	13024	9362	699	486	294	28110	1150	4	685	0	16	56	3	74	277.32	108	434.95	342	357	345	27	41	0	4	2
4530	13240	9558	803	470	288	28106	1211	3	635	0	20	54	0	74	276.89	98	431.15	335	315	306	15	36	0	0	1
13740	7596	4490	4	1224	912	28072	586	270	848	98	98	222	127	203	281.51	201	448.09	486	309	447	34	92	17	12	5
12390	7870	4990	1	1602	1258	28370	577	162	712	42	240	179	86	253	300.55	165	453.87	488	262	406	53	90	11	18	13
14254	7738	4414	7	806	612	27878	632	318	1071	158	50	255	120	140	267.69	286	429.69	515	425	439	9	82	18	4	5
11866	7966	5244	2	1738	1328	28430	559	127	676	31	266	153	59	269	305.44	142	457.36	478	257	407	78	75	7	20	8
4146	13520	9802	906	382	216	28074	1261	8	578	2	8	66	5	66	272.87	93	425.94	315	280	275	13	43	0	0	1
13926	7716	4528	5	992	722	27960	627	295	978	137	72	229	122	186	273.96	218	438.18	452	392	471	26	80	10	4	2
12846	7890	4890	6	1346	1040	28208	589	185	843	63	190	202	90	233	288.23	182	447.44	479	297	427	45	98	12	4	5
5640	12572	9008	607	548	312	28102	1100	11	765	2	22	80	5	108	283.34	115	438.81	355	391	329	14	62	0	0	3
11812	7926	5258	3	1816	1358	28452	571	97	691	18	262	183	51	253	302.46	134	456.62	466	235	391	94	99	7	18	11
12302	7790	5082	0	1662	1252	28356	526	143	759	44	244	196	71	250	298.73	149	455.31	481	241	433	74	109	11	24	13

SUPPLEMENTARY INFORMATION:Monte Carlo Atomistic Simulation and Machine Learning Analysis of Na-K Eutectic Alloy in Condensed Phases, D. Reitz and E. Blaisten-Barojas, George Mason University, Fairfax, VA 22030

6530	11836	8500	422	792	458	28170	961	28	772	6	50	80	21	117	285.27	125	443.15	429	391	380	21	50	0	4	3
11548	8066	5370	1	1838	1368	28458	572	98	659	24	250	177	47	236	305.14	144	455.15	483	224	373	93	89	7	18	21
12650	7944	4988	1	1418	1054	28230	582	184	840	54	160	178	95	251	286.45	173	449.2	460	304	459	43	91	15	14	5
13500	7644	4578	5	1268	974	28112	563	250	824	86	134	225	118	207	282.29	191	445.25	483	273	448	50	101	19	14	5
4570	13156	9568	764	520	282	28112	1153	4	635	0	16	54	4	82	276.1	106	432.61	354	304	337	14	34	0	0	1
3864	13670	9966	968	350	194	28058	1298	7	573	1	14	64	3	62	270.69	110	423.3	291	270	261	15	41	0	0	2
4566	13304	9596	819	372	214	28066	1224	7	682	1	14	63	5	82	273.68	99	427.06	297	331	317	13	46	1	0	3
14224	7724	4416	5	820	636	27890	609	328	1057	156	66	259	134	160	270.11	259	434.58	492	407	451	19	85	17	4	3
10852	8054	5600	1	2120	1640	28676	530	60	582	5	380	152	33	254	321.35	108	464.11	513	201	374	108	86	4	28	24
14384	7588	4346	4	876	638	27888	619	331	1037	165	54	262	131	153	269.24	268	432.59	514	418	432	14	86	16	2	0
4676	13142	9508	765	490	274	28102	1194	15	638	1	12	56	11	83	276.84	98	431.48	337	337	311	11	39	1	0	2
10876	8018	5620	0	2148	1618	28670	494	86	525	13	350	166	45	271	325.68	100	468.03	509	173	374	110	81	4	38	25
5274	12722	9198	635	596	318	28122	1140	9	730	1	14	71	4	89	279.29	97	435.19	369	394	307	16	51	0	0	0
3884	13628	9958	939	386	202	28068	1268	8	539	2	10	58	3	71	271.98	84	426.32	287	265	295	13	41	0	0	5
5032	12708	9242	689	718	432	28178	1181	8	638	0	44	61	7	86	280.31	83	436.33	356	352	287	22	44	0	2	2
13798	7768	4608	5	984	722	27974	611	287	977	131	86	256	112	172	278.04	236	442.26	484	378	430	36	109	21	8	2
12474	7908	5044	3	1576	1124	28304	586	146	768	42	176	176	72	241	293.63	161	453.56	490	258	426	51	85	13	2	7
11492	7898	5308	2	1998	1542	28586	519	111	593	19	310	189	63	280	311.47	109	461.6	486	180	379	89	103	12	36	25
4432	13280	9646	870	454	254	28082	1234	14	597	0	16	49	7	90	273.53	81	428.96	290	287	311	14	34	0	0	1
10686	7822	5632	0	2350	1830	28830	519	60	470	6	452	132	38	287	332.94	70	471.64	461	155	387	124	65	5	50	40
11922	7740	5134	3	1952	1462	28500	545	127	625	37	250	184	62	252	307.69	130	454.18	519	221	355	90	100	5	36	12
13848	7812	4612	4	930	668	27942	618	284	1016	138	70	252	113	165	273.85	235	439.73	494	407	436	26	100	10	2	1
11964	7920	5204	2	1744	1314	28416	555	126	694	34	240	169	67	255	299.64	137	453.31	480	242	425	75	86	8	30	7
4448	13204	9626	813	528	288	28112	1213	3	606	0	18	48	0	82	276.76	81	432.01	324	310	311	15	34	0	0	1
4612	13152	9520	770	502	298	28106	1197	10	638	2	22	53	6	81	275.97	98	431.81	324	336	320	19	38	1	0	1
14212	7662	4398	5	892	678	27914	646	313	997	164	66	264	112	154	272.06	239	436.45	499	424	409	18	90	17	6	3
3932	13710	9942	974	280	164	28040	1282	9	578	2	8	66	5	63	271.65	97	424.89	289	263	279	12	40	0	4	4
11204	7808	5502	1	2190	1586	28656	530	78	524	15	324	173	36	284	320.12	89	462.83	494	187	359	102	83	5	40	22
10908	7734	5510	3	2290	1816	28768	492	80	480	13	448	164	46	313	332.57	76	468.88	451	154	367	125	88	0	58	36
4492	13376	9632	825	344	204	28060	1208	7	664	1	12	61	3	74	275.75	111	429.41	318	318	315	16	41	0	0	1
4700	13164	9532	764	442	234	28084	1186	8	679	1	12	59	4	88	277.11	110	433.13	318	336	326	16	39	0	0	1
5784	12296	8858	524	754	442	28170	1047	12	705	1	36	71	8	114	283.51	113	438.49	386	363	352	20	48	1	0	3
12540	7740	4930	2	1636	1244	28326	565	179	716	53	212	197	78	245	294.19	161	452.99	496	262	398	58	89	13	24	12
11864	8058	5302	3	1738	1230	28386	549	120	721	31	168	175	54	252	302.89	147	453.4	512	246	415	53	102	6	20	16
4788	13072	9470	776	482	262	28090	1220	7	676	0	16	66	5	96	278.7	102	434.41	298	352	291	16	45	0	0	2
4584	13358	9612	842	310	174	28048	1227	5	683	0	10	65	3	69	276.8	113	430.14	317	319	298	12	52	0	0	3

SUPPLEMENTARY INFORMATION:Monte Carlo Atomistic Simulation and Machine Learning Analysis of Na-K Eutectic Alloy in Condensed Phases, D. Reitz and E. Blaisten-Barojas, George Mason University, Fairfax, VA 22030

4266	13440	9764	877	390	200	28062	1273	8	624	1	2	50	2	54	273.08	99	425.93	303	320	292	20	35	0	0	1
5818	12142	8782	467	896	540	28230	1013	9	698	1	50	75	5	116	285.72	96	441.41	436	382	327	23	48	0	2	0
4760	13002	9426	736	590	338	28134	1150	10	627	0	18	62	6	80	279.96	114	434.9	380	307	308	12	46	0	0	2
11524	7976	5358	3	1896	1450	28530	563	85	641	13	288	163	49	250	311.4	117	460.52	478	230	392	100	82	6	34	17
4742	12892	9398	707	680	410	28166	1152	6	601	1	44	65	1	94	277.85	91	434.57	358	330	303	17	38	0	0	5
14246	7706	4438	3	836	610	27892	667	321	1051	156	52	262	118	156	273.21	238	436.34	463	448	419	23	90	21	4	3
4450	13338	9610	832	422	260	28092	1230	2	620	0	10	53	1	70	273.4	96	428.07	325	311	301	15	38	0	0	1
6456	11752	8398	394	940	624	28258	982	16	743	3	80	79	10	122	287.36	115	444.1	434	409	340	31	44	1	8	2
4058	13614	9874	951	320	176	28048	1294	9	602	1	4	58	6	71	272.37	91	425.8	277	292	280	12	40	0	2	2
13484	7644	4584	5	1294	980	28128	592	236	836	75	128	191	119	206	282.98	179	446.63	507	321	452	32	94	21	14	1
4216	13516	9782	912	340	192	28054	1249	12	591	2	8	65	8	72	271.83	99	425.1	306	278	291	8	46	0	0	4
12956	7820	4788	5	1386	1074	28200	621	191	835	78	160	188	83	201	283.45	200	447.91	485	324	431	50	80	11	16	5
14344	7614	4346	6	892	652	27896	621	333	1004	165	44	262	121	146	271.36	275	435.97	506	387	429	23	87	20	4	3
5272	12760	9160	602	578	342	28132	1099	8	726	1	20	73	4	96	282.55	107	440.09	385	379	329	14	53	0	0	0
12206	7962	5084	6	1656	1242	28358	594	133	753	27	182	164	64	215	300.86	157	451.36	516	268	404	65	98	13	24	11
5048	12992	9338	693	452	248	28086	1150	9	709	0	8	68	8	85	279.41	102	436.44	348	377	330	14	46	0	0	1
13850	7740	4478	9	1020	804	27986	645	301	948	146	84	244	119	169	270.1	250	435.31	504	377	413	20	83	20	10	0
14418	7712	4380	4	748	548	27842	632	328	1086	168	34	266	117	143	268.03	275	429.34	497	436	437	15	88	18	2	2
13452	7660	4554	2	1298	1022	28142	611	243	820	87	142	202	110	193	282.1	191	447.88	478	320	443	42	92	13	12	11
7696	10892	7714	300	1134	730	28254	883	50	759	10	84	92	28	151	286.97	132	445.46	443	380	372	39	53	2	4	0
4472	13354	9656	839	370	202	28064	1241	7	662	1	10	58	3	68	274.36	103	427.33	306	334	298	18	37	0	0	1
11402	7882	5382	2	2050	1530	28586	539	77	585	16	314	165	38	286	313.99	114	457.45	487	192	383	90	92	5	26	12
13234	7818	4718	6	1254	962	28136	630	220	890	68	148	199	120	212	281.85	172	447.28	453	344	451	42	102	17	2	3
11182	7858	5492	0	2148	1586	28636	498	87	597	14	336	181	48	302	312.46	111	460	488	184	378	97	104	7	32	21
13000	7946	4864	3	1254	942	28154	601	193	921	71	144	198	83	209	281.08	201	446.3	504	329	440	33	105	13	4	2
14456	7734	4410	8	676	498	27816	643	329	1127	170	34	277	119	147	267.84	297	430.07	474	442	432	18	93	20	6	1
4046	13592	9874	938	344	190	28056	1279	3	582	2	10	68	1	67	270.76	78	424.11	290	286	273	15	44	0	0	3
11634	7984	5366	1	1856	1350	28460	586	111	697	23	254	162	56	249	300.11	144	454.21	488	249	394	70	84	6	12	16
5200	12896	9256	691	452	260	28084	1179	12	740	2	20	72	7	90	280.92	94	437.06	314	394	314	19	54	0	0	4
4888	13018	9384	740	514	294	28110	1198	4	684	0	12	51	3	80	276.98	110	431.68	328	357	319	18	37	0	0	1
4480	13286	9640	792	424	232	28078	1203	5	644	0	16	65	1	75	275.14	100	428.98	328	347	304	17	43	0	0	1
4402	13258	9614	801	518	308	28118	1203	3	598	0	18	64	3	78	276	94	432.03	330	305	301	21	36	0	0	1
11834	7806	5278	4	1816	1396	28486	526	114	665	27	314	177	55	292	306.78	125	457.54	467	221	380	82	96	5	36	19
3820	13738	9992	972	312	176	28048	1305	7	565	1	10	67	2	71	270.05	87	423.11	272	283	262	13	41	0	0	3
5738	12414	8880	559	652	418	28152	1055	12	716	4	50	70	7	105	283.79	114	439.9	385	378	351	18	44	0	0	6
13360	7740	4590	2	1256	1024	28140	612	241	818	98	154	211	107	206	277.49	190	445.33	489	306	435	35	90	16	16	4

8136	10704	7506	257	1092	708	28234	837	54	804	23	84	121	26	160	289.59	136	444.87	454	373	382	34	73	0	4	3
12074	7990	5208	2	1654	1206	28346	588	141	761	40	190	193	69	254	299.84	148	451.7	433	276	410	82	106	10	22	11
4376	13346	9692	842	420	228	28074	1235	7	631	0	12	61	2	74	274.28	104	427.52	317	314	292	11	47	0	0	3
14364	7604	4374	8	854	622	27878	639	338	1035	173	56	257	124	144	270.6	273	433.2	506	424	432	15	74	23	4	1
4416	13236	9598	823	532	320	28122	1219	4	577	0	20	51	3	84	276.81	87	431.24	322	305	301	14	34	0	0	3
4756	13192	9500	778	382	222	28072	1211	13	664	0	20	58	7	74	275.93	99	432.65	318	352	312	17	37	0	0	2
12420	8044	5122	4	1426	1048	28244	581	149	824	40	170	180	72	217	292.18	168	450.19	477	290	438	67	101	8	14	8
4466	13236	9580	803	506	306	28112	1188	5	599	0	16	59	2	79	275.93	109	430.96	341	285	307	17	34	0	2	2
5270	12754	9178	644	572	330	28126	1121	8	709	3	22	61	4	83	279.31	113	435.27	383	367	334	11	42	0	0	3
4328	13348	9678	858	466	266	28094	1237	5	592	0	8	61	3	69	274.71	85	429.14	322	296	289	15	42	0	0	3
10746	8084	5638	5	2150	1658	28680	533	72	565	9	366	153	41	291	314.72	103	460.46	452	182	394	111	82	5	38	30
5070	12822	9238	671	602	376	28142	1139	14	667	1	30	64	10	102	282.63	115	438.88	356	354	315	15	32	0	4	1
3944	13676	9934	969	312	172	28046	1314	9	575	2	8	63	6	62	270.99	88	423.89	277	287	263	15	40	0	0	1
4826	13068	9440	726	480	270	28102	1155	1	667	0	18	58	0	90	278.76	96	435.59	340	329	336	15	39	0	0	1
4548	13212	9554	793	474	290	28104	1204	5	642	0	26	67	3	82	276.48	93	432.4	317	316	304	22	48	0	0	1
4586	13226	9570	779	446	248	28088	1172	4	648	0	12	45	1	67	276.37	93	430.26	350	328	338	17	34	0	0	2
5180	12816	9196	680	576	344	28126	1142	7	675	0	14	67	4	87	282.87	114	438.49	362	343	318	19	41	0	0	0
6212	12004	8610	502	826	486	28170	1001	25	699	7	32	82	14	110	286.5	116	441.24	401	342	363	32	51	0	0	2
4536	13330	9610	849	364	212	28064	1248	4	650	0	8	62	3	76	275.61	94	430.52	288	333	299	19	43	0	4	2
5994	12260	8760	534	672	418	28152	1027	19	735	2	48	81	13	117	285.87	130	441.03	392	360	348	20	51	1	0	2
5054	13008	9338	697	436	242	28088	1160	8	742	0	10	53	5	97	278.19	103	433.56	327	387	342	11	39	0	0	2
4502	13184	9548	804	528	328	28116	1213	9	615	1	24	54	4	74	276.63	99	429.83	328	313	300	20	39	0	2	3
12280	7968	5060	1	1594	1216	28336	603	156	761	38	202	169	88	248	293.28	146	451.37	477	292	418	53	93	8	14	7
13400	7820	4712	5	1150	856	28056	620	260	939	99	112	212	126	189	276.43	221	439.2	492	370	451	26	91	14	6	1
11332	7924	5420	2	2036	1530	28586	543	110	598	21	314	158	56	291	315.03	113	460.08	480	197	382	90	78	8	26	15
4376	13364	9678	858	414	234	28076	1224	5	619	1	10	65	3	70	272.95	94	426.62	315	292	304	18	38	0	0	2
11158	7900	5498	0	2068	1598	28648	490	87	572	12	398	182	52	276	319.26	126	461.21	490	168	374	118	97	2	28	21
11258	7924	5356	0	2058	1642	28630	554	97	570	20	340	169	50	309	310.47	99	456.75	450	209	363	96	80	7	50	21
13814	7776	4548	8	988	760	27986	622	276	985	110	98	221	127	162	275.45	247	439.28	500	389	454	27	89	21	2	3
11418	7850	5422	2	2004	1516	28588	507	107	600	20	330	164	62	275	315.25	122	460.24	484	191	405	96	92	6	46	23
4978	12878	9276	669	612	380	28152	1140	3	666	1	26	55	1	93	279.73	82	435.34	347	360	335	21	44	0	2	4
5012	12828	9286	694	614	366	28140	1158	9	660	1	34	63	4	102	280.43	100	435.66	344	346	311	16	44	0	0	0
5258	12724	9160	636	608	364	28144	1086	10	692	2	28	62	6	104	281.47	111	437.56	368	341	353	15	42	0	2	4
5408	12540	9054	605	708	420	28164	1101	12	679	1	34	66	9	99	280.27	117	438.03	383	343	331	13	46	0	0	4
14524	7644	4306	3	738	576	27850	633	333	1095	166	60	268	131	151	267.35	287	429.03	489	433	437	14	87	19	2	2
13216	7684	4714	2	1394	1018	28170	563	218	805	80	130	224	100	211	289.83	182	449.73	492	268	422	57	108	15	12	6

14386	7712	4364	3	754	586	27860	638	327	1067	164	56	257	126	136	269.39	278	432.21	511	433	434	15	78	19	2	1
11178	7908	5476	1	2094	1594	28638	578	87	566	14	350	156	54	298	312.67	107	460.7	459	210	359	91	70	3	34	20
3972	13704	9936	973	270	146	28034	1302	6	597	1	6	60	4	67	271.29	102	423.9	278	281	274	11	48	0	0	1
11628	7984	5270	2	1874	1452	28510	505	97	634	23	260	169	52	269	304.57	123	457.53	499	204	416	91	79	6	38	16
11500	7822	5306	0	2012	1580	28610	537	101	582	18	342	177	53	273	315.29	126	459.9	495	180	363	86	90	6	38	26
13050	7766	4796	2	1352	1034	28180	611	210	850	77	158	209	101	216	281.96	166	447.68	464	326	427	50	99	11	20	7
4312	13570	9782	892	232	120	28018	1251	8	628	1	2	68	3	76	273.85	82	427.27	275	309	309	13	45	0	0	3
14054	7646	4448	7	988	734	27950	625	308	973	148	76	238	124	181	273.42	223	438.06	450	392	463	32	72	13	4	2
11008	7856	5554	0	2230	1638	28670	508	70	533	16	352	182	35	287	317.05	98	462.36	490	170	359	115	95	5	32	19
4344	13280	9694	839	492	268	28100	1206	5	597	0	22	60	3	82	275.13	82	430.02	325	290	303	13	48	0	0	4
5824	12288	8810	535	748	464	28178	1041	10	716	1	44	72	7	100	286.51	118	442.4	400	355	350	26	43	0	0	4
4690	13024	9486	749	584	322	28128	1157	6	620	1	22	62	2	92	281.05	98	435.91	349	305	320	13	41	0	0	3
12738	7860	4878	1	1440	1128	28254	593	179	831	52	192	191	92	210	285.27	192	449.08	499	300	436	55	101	9	18	4
4048	13548	9882	919	376	196	28062	1277	9	601	1	12	62	5	73	271.79	86	424.05	292	302	278	11	39	0	0	2
4686	13124	9508	772	500	270	28094	1187	8	633	0	6	63	7	80	273.9	87	428.16	330	334	317	17	45	0	0	2
13700	7786	4604	5	1078	770	28002	646	253	968	123	52	223	101	182	277.82	224	442.53	465	384	445	27	82	17	12	3
14292	7772	4424	6	752	572	27864	649	317	1089	157	52	264	122	147	271.83	286	435.78	491	440	421	19	96	20	0	0
4980	12988	9350	720	498	278	28102	1160	1	697	0	8	64	0	94	277.01	104	433.75	341	347	323	13	45	0	0	0
14100	7660	4442	8	950	708	27934	624	309	1004	166	66	260	111	159	269.09	254	432.05	493	410	430	28	82	17	6	1
13720	7866	4670	3	956	680	27962	649	268	1006	119	66	229	117	170	275.59	217	439.36	467	411	447	22	93	12	4	6
11568	8040	5364	3	1842	1392	28508	555	91	641	13	280	172	51	254	311.7	135	459.61	485	200	395	85	109	6	18	20
11018	7954	5466	0	2162	1694	28698	528	61	571	9	346	183	31	297	323.25	100	465.01	469	172	338	112	101	4	54	22
6566	11896	8428	382	780	472	28170	932	19	798	1	26	75	14	123	283.2	130	442.88	441	412	397	21	49	1	2	0
13648	7768	4562	5	1092	844	28016	610	262	958	115	94	239	113	198	277.02	217	440.85	473	367	436	29	96	14	8	3
4786	13156	9450	749	418	262	28098	1184	3	673	0	24	61	2	88	277.76	82	434.13	322	353	324	17	39	0	2	1
12956	7730	4826	6	1422	1074	28204	579	220	800	95	180	218	98	204	281.96	174	449.01	512	301	412	56	94	9	16	3
4452	13314	9630	839	424	248	28084	1224	7	625	1	16	55	6	79	274.45	102	430.16	307	304	315	15	43	0	0	2
4168	13484	9746	894	400	264	28088	1252	10	570	2	26	62	4	88	274.86	86	429.62	285	283	290	16	39	1	0	2
5100	12824	9224	644	598	370	28146	1089	4	662	0	30	70	4	90	281.25	113	438.36	382	336	338	20	41	0	0	5
14346	7686	4384	8	794	602	27872	646	337	1054	160	54	251	140	153	268.07	273	429.73	480	435	447	15	82	17	6	2
12204	8014	5124	2	1550	1202	28340	549	149	743	38	230	199	78	230	301.44	161	455.13	500	258	402	79	113	9	16	9
14072	7802	4490	6	856	640	27918	654	277	1063	137	58	237	108	166	274.11	250	437.81	459	413	450	22	87	14	0	3
5278	12760	9158	672	560	340	28122	1125	16	663	2	24	76	11	82	282.2	109	439.44	363	340	321	22	45	0	2	5
11814	8160	5240	1	1646	1288	28394	570	125	731	27	228	160	69	234	301.52	170	454.13	493	241	428	78	82	7	16	8
12756	7904	4886	1	1416	1084	28224	613	169	846	59	170	184	87	204	286.72	172	450.67	491	325	441	48	94	14	8	7
4606	13244	9564	776	418	236	28080	1223	7	667	0	12	68	4	67	276.31	98	429.91	325	359	290	18	46	0	0	3

SUPPLEMENTARY INFORMATION:Monte Carlo Atomistic Simulation and Machine Learning Analysis of Na-K Eutectic Alloy in Condensed Phases, D. Reitz and E. Blaisten-Barojas, George Mason University, Fairfax, VA 22030

4978	13014	9354	676	456	272	28096	1120	6	682	0	20	60	3	82	279.75	95	437.14	357	361	354	18	43	0	2	2
13614	7852	4708	4	992	722	27986	617	283	977	115	90	214	134	174	275.72	235	438.62	484	396	465	26	79	13	8	3
12936	7706	4832	0	1502	1080	28220	573	196	769	60	154	207	99	209	293.11	160	452.75	470	287	441	72	97	13	10	8
5218	12716	9180	639	626	372	28146	1089	8	682	0	34	64	6	92	281.1	120	437.95	391	335	337	16	43	0	0	3
14266	7740	4418	7	816	598	27876	642	326	1063	162	34	263	129	168	268.78	268	431.6	464	426	440	14	88	16	4	4
10976	7924	5548	1	2182	1656	28692	502	75	534	15	362	176	40	293	323.32	111	465.66	486	160	371	102	91	2	42	31
4484	13208	9620	833	494	272	28100	1231	7	639	0	20	59	6	82	273.57	87	428.57	294	319	308	22	42	0	2	2
4742	13100	9446	730	504	304	28116	1156	5	644	0	18	51	3	90	278.35	92	434.28	340	330	338	15	35	0	2	3
14026	7728	4516	6	914	670	27926	647	278	1063	136	62	240	101	156	273.31	284	435.55	504	427	426	15	91	18	10	1
4966	12950	9342	710	530	308	28118	1147	7	669	0	22	63	3	86	279.1	116	434.43	367	331	319	13	48	1	0	0
11386	7732	5370	2	2088	1620	28624	509	107	562	24	390	169	63	281	312.02	125	459.02	503	174	385	96	76	7	34	23
12640	7988	4964	3	1398	1068	28236	608	159	849	47	160	185	86	210	285.43	197	449.88	507	327	414	46	100	5	18	7
11240	7808	5422	1	2212	1608	28628	539	93	550	16	318	150	46	267	318.78	97	463.62	514	205	367	96	70	7	20	20
3806	13690	9982	1005	360	208	28062	1313	6	541	1	14	58	4	70	271.14	91	423.32	274	259	264	13	37	0	2	2
5298	12574	9096	611	738	438	28176	1101	7	637	0	30	60	4	92	280.3	94	438.17	376	339	339	25	37	0	2	3
11220	7752	5390	0	2212	1698	28686	524	82	527	19	366	162	40	274	320.49	103	465.41	492	192	362	115	75	3	42	21
5342	12740	9160	640	550	308	28118	1126	7	699	0	18	66	4	100	282.54	101	437.57	356	377	330	15	44	0	0	0
13172	7790	4738	1	1302	992	28150	575	221	864	81	146	216	106	220	284.91	195	447.75	489	315	428	45	105	7	10	3
13882	7592	4430	5	1076	890	28034	584	275	927	107	156	242	125	196	277.06	224	447.5	484	336	438	38	105	17	8	3
5326	12656	9152	619	622	352	28140	1102	8	714	1	32	61	2	99	280.74	100	436.54	362	372	346	23	44	0	0	0
4420	13340	9658	806	416	232	28074	1213	7	617	1	8	61	6	76	274.49	80	428.65	324	321	304	15	39	0	0	0
14030	7796	4514	9	866	646	27914	645	291	1062	143	58	252	116	163	275.19	254	437.95	467	438	445	21	92	14	2	4
5630	12300	8924	554	802	478	28188	1099	10	713	2	52	68	7	121	283.72	83	439.87	358	404	328	18	38	0	2	2
4512	13244	9580	800	478	274	28096	1199	7	626	1	8	60	5	77	275.72	94	430.58	337	312	308	15	40	0	0	0
4184	13492	9798	893	370	206	28064	1246	6	598	1	14	57	3	82	274.39	99	428.33	296	289	300	9	35	0	0	3
4366	13312	9662	852	454	272	28092	1245	11	600	2	26	61	5	87	274.77	74	429.58	297	309	285	16	43	0	0	1
5076	12800	9294	651	610	330	28132	1116	6	668	2	22	83	3	83	280.93	90	436.77	383	359	307	20	54	0	0	3
13244	7858	4740	4	1210	914	28092	604	211	901	80	122	212	102	196	277.75	205	443.97	476	333	459	40	99	13	4	4
4242	13416	9740	885	430	244	28084	1263	8	602	1	12	57	6	70	273.42	102	426.42	305	300	282	15	43	0	0	2
11470	8032	5370	1	1974	1426	28512	514	82	640	12	206	165	46	273	308.55	123	457.02	530	203	406	68	103	8	34	11
11028	7868	5560	2	2174	1642	28696	503	76	542	13	376	173	41	283	323.26	100	464.52	495	189	360	117	88	6	44	23
5820	12428	8868	557	628	372	28138	1061	17	757	0	22	67	13	103	281.22	121	437.92	396	387	349	14	46	0	0	1
5218	12752	9180	645	618	356	28138	1115	2	702	0	14	68	1	94	280.25	116	437.08	376	355	325	11	47	0	0	5
4326	13382	9712	852	414	228	28072	1258	10	620	1	10	56	4	83	272.82	80	426.56	296	327	288	10	44	0	0	1
11304	7892	5312	3	2118	1642	28622	535	92	554	19	326	169	47	257	319.58	106	461.47	487	175	377	109	88	4	28	28
13608	7598	4530	3	1256	964	28096	622	255	859	106	122	228	104	179	277.28	196	445.4	490	322	404	47	102	20	14	7

SUPPLEMENTARY INFORMATION:Monte Carlo Atomistic Simulation and Machine Learning Analysis of Na-K Eutectic Alloy in Condensed Phases, D. Reitz and E. Blaisten-Barojas, George Mason University, Fairfax, VA 22030

11930	7908	5156	3	1776	1396	28478	566	127	652	31	284	182	67	251	304.9	121	458.17	489	227	383	79	105	12	26	18
4510	13280	9600	822	426	250	28082	1208	6	639	1	14	56	4	72	274.62	90	431.15	324	312	317	14	39	0	2	4
4722	13122	9510	777	468	254	28094	1209	8	629	0	18	63	6	76	277.2	89	433.63	323	340	302	19	43	0	0	2
11570	7776	5184	1	2102	1614	28570	526	108	584	29	294	173	53	269	300.72	111	456.94	511	203	373	90	83	5	30	20
4724	13114	9476	727	502	282	28108	1133	1	646	0	10	64	1	85	278.1	98	433.44	360	306	338	15	46	0	0	2
5108	12740	9226	659	666	382	28142	1073	7	650	0	20	67	6	95	279.62	115	435.37	396	298	343	15	44	0	0	3
7572	11022	7802	332	1076	688	28240	900	53	740	16	76	87	28	150	289.08	126	446.35	439	357	383	27	51	2	4	3
4524	13238	9588	809	444	268	28092	1214	7	621	0	30	53	5	81	275.23	84	430.49	316	321	310	16	38	0	0	2
3898	13632	9938	972	368	212	28068	1296	8	540	0	18	61	7	82	272.66	75	426.77	271	259	276	11	41	0	2	1
13090	7740	4728	1	1396	1066	28190	615	190	849	68	162	204	85	205	285.58	189	447.69	477	319	414	59	107	15	8	3
3794	13768	10016	1020	290	164	28044	1322	9	542	1	8	61	5	71	271.94	81	423.57	257	252	268	13	41	0	4	3
4672	13172	9508	783	468	264	28090	1213	6	656	0	6	68	3	80	276.75	96	432.27	326	341	293	12	49	0	0	3
13882	7810	4582	6	942	670	27942	631	274	1035	125	54	244	104	164	275.34	250	440.34	487	386	433	25	106	18	2	3
4728	13120	9474	762	474	286	28110	1215	7	664	1	24	63	4	83	277.51	98	434.45	328	357	295	13	48	0	4	1
3920	13676	9940	979	328	180	28050	1300	5	571	1	6	59	3	69	271.3	91	424.48	276	267	281	10	39	0	0	3
10936	7942	5560	0	2130	1678	28698	489	82	559	14	400	173	49	306	321.38	116	467.51	477	162	370	106	90	5	48	29
5944	12260	8730	534	736	468	28172	1054	19	721	6	30	80	12	97	282.84	109	439.08	407	375	332	22	44	0	4	3
4282	13396	9734	846	416	234	28080	1238	6	610	1	18	60	2	80	275.81	88	429.1	303	313	292	16	40	0	0	1
14204	7620	4390	5	920	706	27928	626	295	1008	137	82	247	124	153	271.75	243	437.38	481	404	445	34	91	12	6	3
4808	12992	9356	749	572	380	28152	1171	5	632	0	44	63	5	90	278.83	101	435.88	345	315	306	20	43	0	0	2
4066	13578	9854	937	356	198	28058	1285	6	590	0	6	55	5	75	271.53	85	425.46	276	291	289	13	42	0	0	2
5722	12386	8880	576	700	434	28162	1051	14	699	4	38	62	6	119	282.59	105	440.75	380	353	364	15	40	0	0	2
3842	13594	9956	1002	424	240	28080	1334	9	533	2	22	63	6	72	271.28	76	424.5	265	258	245	14	48	0	2	3
14380	7772	4430	11	690	514	27832	643	321	1131	169	46	265	117	142	269.76	301	432.31	497	441	437	11	88	18	0	0
4912	12936	9350	707	564	336	28126	1161	6	657	1	28	61	3	104	278.17	90	434.45	324	343	325	17	44	0	0	2
5032	12858	9310	696	570	322	28116	1138	11	678	0	24	60	6	88	279.19	96	434.51	360	342	332	14	42	1	0	2
4468	13386	9622	815	352	226	28072	1198	4	653	1	16	60	3	74	272.75	112	428.78	320	317	324	13	38	0	2	6
4018	13588	9882	955	362	200	28060	1300	3	576	0	10	69	3	68	272.16	88	424.79	283	281	257	14	50	0	0	3
5138	12860	9242	669	532	324	28126	1142	6	704	0	28	77	5	84	279.35	107	434.54	350	363	317	27	53	0	2	1
4530	13266	9608	820	420	238	28082	1194	8	629	1	20	55	4	77	275.17	95	430.6	325	303	328	15	40	0	0	2
4252	13468	9734	882	384	228	28076	1235	8	593	1	10	69	6	79	274.64	98	429.29	307	287	288	16	41	0	0	1
14614	7540	4274	7	796	576	27846	645	361	1034	168	44	270	138	143	271.52	264	435.65	484	413	431	19	94	28	2	2
5182	12798	9218	638	552	338	28128	1092	7	668	0	40	74	6	102	282.26	108	436.39	372	344	339	11	52	0	0	4
4090	13574	9850	928	338	190	28052	1293	9	592	1	8	65	5	75	271.59	70	424.32	263	310	279	17	44	0	2	3
14472	7586	4276	5	846	646	27882	620	338	1006	160	52	261	127	146	271.49	253	434.55	497	396	443	25	85	28	4	1
7044	11340	8032	382	1050	708	28260	954	36	712	8	76	73	23	137	286.95	106	445.56	424	372	365	30	47	0	10	5

SUPPLEMENTARY INFORMATION:Monte Carlo Atomistic Simulation and Machine Learning Analysis of Na-K Eutectic Alloy in Condensed Phases, D. Reitz and E. Blaisten-Barojas, George Mason University, Fairfax, VA 22030

13016	7728	4760	4	1446	1088	28210	619	200	808	76	162	196	85	235	284.39	171	448.64	465	325	415	42	93	8	10	6
4590	13100	9574	764	544	282	28110	1193	3	644	0	20	40	2	73	277.94	94	432.6	329	338	337	18	29	0	0	4
5208	12688	9124	656	654	434	28158	1101	13	642	3	46	65	7	99	281.77	111	439.36	375	311	331	21	46	0	4	1
4012	13622	9886	944	324	192	28054	1282	5	572	1	18	60	4	65	271.33	86	424.57	283	271	284	17	40	0	0	3
5356	12608	9062	619	668	428	28170	1078	6	656	0	48	60	5	96	281.32	109	437.91	392	330	343	19	36	1	0	4
3896	13630	9922	959	382	226	28074	1291	6	547	0	18	59	5	63	271.05	87	423.47	304	269	263	12	39	0	0	2
14254	7720	4412	12	816	626	27896	670	310	1080	154	64	248	124	149	271.74	270	434.51	475	456	429	19	83	19	4	3
4724	13080	9444	740	528	324	28126	1170	5	630	0	22	66	3	83	277.2	96	432.64	356	332	303	14	45	0	4	3
11086	7886	5472	1	2158	1666	28688	516	83	525	15	390	160	41	293	320.05	76	467.31	432	165	394	121	90	5	30	33
4484	13302	9626	823	422	238	28086	1210	6	646	0	14	54	3	83	275.73	89	430.66	311	313	320	16	36	0	0	1
11890	7936	5160	1	1786	1390	28444	571	130	683	30	254	168	72	256	302.64	135	453.52	474	223	395	76	93	7	26	15
4070	13546	9836	931	374	224	28070	1282	7	564	3	20	62	4	70	273.43	78	426.36	281	291	282	16	36	0	0	4
12132	8086	5226	4	1582	1114	28308	611	123	791	24	160	165	70	217	294.34	173	451.11	490	285	423	59	106	8	8	10
4578	13290	9574	793	384	230	28072	1211	6	667	1	16	60	1	77	275.99	102	428.77	326	356	306	13	43	0	0	0
14438	7660	4354	5	790	578	27862	653	333	1079	173	36	264	133	135	268.72	277	429.75	495	444	431	18	79	16	6	2
7328	11236	7924	338	1012	672	28258	912	37	766	12	78	78	21	143	288.04	148	445.63	460	373	375	22	49	1	8	1
5726	12410	8880	556	674	426	28158	1052	18	715	3	36	64	11	103	282.96	113	439.09	400	357	354	18	44	0	6	1
11284	7934	5322	0	2072	1652	28646	514	91	561	23	336	157	40	273	317.61	117	461.49	508	180	388	100	79	7	44	22
3714	13818	10036	1005	298	176	28050	1316	4	544	0	8	55	2	59	271.67	86	424.3	277	249	270	15	38	0	0	2
10968	7892	5550	1	2212	1670	28704	524	68	564	9	374	162	31	286	324.98	108	466.69	465	162	377	112	90	3	34	27
13002	7722	4734	3	1496	1114	28216	590	206	771	75	130	197	97	216	285.7	168	447.59	478	254	431	58	91	13	16	6
4730	13066	9464	731	518	308	28118	1150	3	644	0	32	72	2	95	279.09	102	435	350	328	312	15	49	0	0	1
12260	7822	5134	1	1708	1216	28362	538	136	704	33	200	201	68	246	297.79	159	454.26	505	220	398	78	118	11	20	5
11644	7930	5214	0	1926	1510	28538	541	99	624	24	270	183	47	279	310.19	121	460.66	478	215	377	83	97	9	40	17
13842	7770	4564	4	978	724	27958	611	281	993	119	76	220	126	168	276.46	232	443.09	469	383	483	36	84	16	4	1
4172	13486	9790	894	390	222	28072	1271	5	606	1	8	62	3	72	272.18	79	426.72	278	316	291	18	39	0	4	5
4220	13528	9796	939	318	174	28044	1306	9	630	1	8	62	3	73	271.85	105	424.95	271	300	267	10	42	0	0	3
5410	12754	9156	630	484	262	28078	1072	20	726	3	12	67	11	91	278.77	109	435.28	363	348	377	20	41	0	0	2
5094	12864	9290	685	536	306	28118	1155	7	696	2	28	71	5	90	281.21	96	436.14	349	367	310	18	48	0	0	0
10614	7714	5598	1	2474	1928	28854	506	64	465	11	464	160	30	301	333.28	87	471.87	474	136	330	136	80	5	56	34
12858	7974	4886	4	1280	1000	28166	615	201	878	66	154	200	101	204	280.64	192	446.71	492	325	418	45	103	14	14	4
12806	8004	4946	10	1298	966	28164	651	192	872	58	134	183	94	193	283.67	179	447.55	472	341	430	47	92	19	10	5
14324	7654	4392	5	826	618	27878	620	326	1065	166	56	270	120	164	269.77	265	431.71	475	420	441	22	94	19	8	2
4822	13024	9436	716	520	288	28108	1135	4	653	0	16	64	2	92	279.93	89	436.3	341	329	343	18	43	0	2	3
13688	7696	4604	3	1120	814	28028	627	257	944	104	102	208	117	184	278.02	214	444.59	467	372	464	35	84	18	2	1
13880	7766	4514	6	960	752	27970	623	285	988	144	90	244	118	186	273.77	225	439.86	470	415	448	24	84	8	8	1

SUPPLEMENTARY INFORMATION:Monte Carlo Atomistic Simulation and Machine Learning Analysis of Na-K Eutectic Alloy in Condensed Phases, D. Reitz and E. Blaisten-Barojas, George Mason University, Fairfax, VA 22030

4254	13526	9764	882	312	186	28056	1258	7	612	1	14	63	4	78	273.43	92	427.16	291	310	293	12	41	0	0	1
4066	13590	9876	949	338	176	28050	1282	9	591	1	4	51	5	67	271.04	97	424.5	287	281	289	13	36	0	0	2
10384	7812	5734	0	2460	1942	28914	494	53	437	6	528	152	24	313	330.73	73	470.84	475	147	346	139	65	2	54	34
13644	7646	4530	1	1180	934	28090	570	258	870	101	142	221	120	204	283.08	204	446.42	478	306	459	47	93	12	14	4
4304	13472	9754	886	330	178	28046	1273	13	621	1	8	60	5	73	273.13	86	427.3	285	329	288	12	46	0	0	2
14352	7526	4332	6	912	696	27912	635	322	1009	160	90	253	124	175	272.37	248	435.09	472	399	438	18	83	16	4	1
13868	7788	4556	6	966	718	27978	655	278	1005	123	78	229	117	160	276.7	226	441.71	480	428	432	30	89	19	4	3
11168	7886	5482	3	2152	1592	28638	544	92	557	14	324	153	47	278	317.98	98	461.82	483	190	377	105	81	6	28	19
11924	7892	5176	1	1776	1382	28456	554	120	643	24	276	178	63	247	301.24	124	457.15	472	224	402	85	89	11	26	22
6932	11488	8164	385	956	612	28220	947	37	759	7	64	88	22	137	287.33	119	444.83	413	375	370	30	56	1	4	5
13402	7868	4730	6	1104	828	28052	644	246	943	109	118	213	99	195	276.63	210	443.2	476	392	426	27	82	16	2	2
4566	13222	9576	778	456	254	28086	1193	2	657	0	12	59	0	76	274.36	87	428.64	324	338	322	18	39	0	0	3
13636	7928	4704	5	928	680	27952	612	264	1027	115	72	231	122	178	276.04	240	439.87	462	402	479	28	97	15	4	3
4408	13208	9620	811	546	314	28118	1207	3	596	0	22	54	3	92	279.79	91	436.05	312	299	309	16	41	0	0	3
13368	7934	4682	4	1108	866	28060	602	243	938	110	84	224	100	180	277.09	218	442.36	506	351	436	38	94	13	18	1
3910	13706	9958	992	302	160	28038	1314	8	590	1	2	55	3	59	270.79	103	423.78	276	269	275	11	43	0	0	4
5554	12604	8958	593	598	398	28144	1081	14	693	1	30	63	9	94	282.14	107	437.24	391	363	344	16	37	0	2	3
4074	13562	9828	903	378	220	28068	1268	6	580	1	6	65	4	68	271.82	99	425.14	307	299	269	12	37	0	0	3
4790	13050	9444	737	500	294	28108	1141	8	644	0	30	51	4	92	276.2	100	430.43	348	319	345	12	35	0	0	3
12708	7872	4904	1	1456	1126	28278	568	187	761	46	200	192	101	244	293.84	139	453.41	465	285	434	59	103	8	10	12
4072	13546	9820	942	400	232	28074	1289	5	564	1	4	55	3	70	271.31	85	424.33	285	280	282	14	41	0	0	2
6896	11526	8178	355	946	614	28232	949	24	760	11	68	92	10	133	288.46	104	445.14	429	415	362	28	49	0	4	3
3964	13630	9922	962	344	184	28052	1306	8	569	2	8	59	5	64	271.85	84	424.48	277	278	271	13	34	0	0	4
12144	8042	5172	3	1584	1194	28368	582	126	804	24	210	157	69	236	299.11	154	451.88	504	293	429	56	95	11	22	11
14204	7584	4450	4	946	660	27912	614	322	1016	152	66	258	124	170	273.68	243	437.89	483	400	436	23	93	17	2	3
14338	7680	4412	11	814	584	27876	634	335	1038	157	44	245	142	153	270.21	257	432.49	489	425	458	13	75	17	4	2
4328	13488	9714	859	328	196	28062	1243	7	650	0	8	61	4	62	273.99	109	429.62	315	327	296	16	43	0	0	2
4306	13314	9692	852	488	282	28106	1239	2	610	0	24	59	1	80	276.53	90	431.21	313	305	289	12	39	0	0	4
10810	7960	5622	0	2156	1700	28724	508	69	554	10	436	168	36	266	323.51	107	466.15	492	169	356	126	91	7	34	30
4076	13584	9864	910	338	186	28060	1261	6	568	0	12	52	4	75	275.32	76	429.51	293	287	299	11	35	0	0	2
4312	13388	9718	882	410	230	28072	1252	10	624	1	14	62	5	78	272.1	101	427.63	297	292	289	14	45	0	0	2
4452	13330	9652	848	396	224	28070	1236	5	638	1	16	76	2	89	275.89	87	430.02	289	306	290	14	53	0	0	3
12752	8016	4940	6	1324	1006	28192	654	171	900	67	138	185	79	202	281.49	178	445.5	486	367	419	41	96	10	14	0
10432	7928	5750	1	2332	1852	28836	465	67	463	11	480	163	32	316	329.75	91	473.62	482	130	346	147	77	3	52	24
13878	7758	4470	4	1014	786	27982	634	280	957	137	66	238	103	169	274.84	223	437.95	479	395	432	35	86	18	10	1
3920	13710	9946	985	292	164	28038	1302	6	572	1	6	64	4	69	271.34	92	422.97	271	263	273	12	42	0	0	3

SUPPLEMENTARY INFORMATION:Monte Carlo Atomistic Simulation and Machine Learning Analysis of Na-K Eutectic Alloy in Condensed Phases, D. Reitz and E. Blaisten-Barojas, George Mason University, Fairfax, VA 22030

3914	13608	9898	950	406	240	28078	1279	8	527	2	12	70	6	75	272.66	78	425.03	292	251	262	15	46	0	0	2
11076	7908	5402	2	2200	1722	28702	514	85	505	16	336	166	44	283	314.21	101	462.88	509	155	347	108	83	6	58	25
13798	7740	4554	3	1040	776	28002	598	281	959	124	88	233	125	174	277.36	250	441.4	501	352	452	32	89	17	6	1
5756	12394	8884	566	660	410	28150	1063	9	730	2	44	72	6	99	283.16	125	439.5	397	373	345	16	42	0	2	3
3798	13724	10016	1011	326	172	28046	1333	7	556	1	10	70	4	67	270.44	80	422.27	266	266	246	12	54	0	0	2
11756	7854	5138	1	1882	1540	28526	558	134	613	21	304	156	77	290	302.82	111	455.15	452	215	406	86	91	7	46	14
14488	7590	4314	4	812	612	27882	672	320	1066	169	58	260	108	155	269.29	265	432.08	471	445	416	18	86	21	8	1
13628	7728	4598	10	1142	836	28026	610	280	901	130	92	218	124	167	272.97	205	438.54	505	362	461	30	75	8	2	3
14412	7676	4396	8	762	554	27850	649	323	1090	163	46	273	120	139	271.19	279	434.38	493	444	421	19	100	20	4	1
14226	7620	4392	7	934	686	27922	630	310	994	150	60	244	127	161	272.84	250	436.38	500	402	439	14	82	16	2	3
4936	12908	9346	715	572	336	28130	1176	5	670	0	32	66	3	112	278.36	88	434.85	315	346	310	15	43	0	0	1
13756	7810	4552	2	994	788	28010	634	254	1002	129	102	239	94	178	275.8	258	438.45	488	389	428	25	93	23	6	3
14404	7546	4216	5	942	756	27940	661	311	996	169	68	253	112	165	269.53	251	431.83	476	397	421	21	73	17	6	1
12880	7964	4840	5	1328	1018	28166	588	204	866	77	124	196	86	211	282.21	201	445.49	517	304	425	38	100	14	10	1
11550	7878	5390	1	1958	1434	28530	527	114	623	23	286	168	61	272	307.43	121	457.75	503	200	396	82	81	5	34	15
4730	13088	9470	768	502	292	28104	1174	4	637	1	22	71	2	77	276.62	100	430.5	344	321	304	19	50	0	0	4
13576	7810	4580	2	1096	872	28054	608	248	924	120	106	235	103	195	277.91	211	443.5	456	369	444	49	90	11	12	2
11504	7824	5270	0	1970	1616	28622	506	101	566	27	392	187	49	277	322.45	112	464.24	467	181	391	107	90	7	46	25
11070	7988	5546	2	2088	1574	28644	519	69	596	6	340	171	38	265	321.93	123	468.22	499	191	358	111	99	5	36	24
4390	13308	9684	871	456	238	28084	1254	7	600	0	8	56	3	73	274.99	80	430.1	302	291	291	14	44	0	0	4
4318	13434	9754	898	354	184	28056	1283	6	642	0	12	60	3	51	274.25	109	427.07	304	322	277	18	44	0	0	2
11212	7906	5442	2	2096	1598	28628	553	82	604	17	334	160	42	279	311.32	120	460.07	483	202	368	100	91	7	34	16
4032	13622	9868	922	330	194	28056	1286	10	591	1	10	55	5	69	271.52	81	425.23	275	309	282	19	31	0	0	3
12866	8058	4884	4	1236	968	28162	626	209	856	69	148	177	98	188	281.45	186	446.08	491	334	452	44	89	19	2	5
4486	13298	9636	803	418	230	28082	1182	4	623	0	12	70	3	80	275.98	100	431.5	337	304	310	14	48	0	2	2
4412	13314	9652	847	444	254	28092	1247	3	604	0	14	63	3	60	276.17	95	431.24	330	310	277	18	47	0	2	1
13558	7924	4688	7	998	754	28016	629	256	976	115	84	223	108	164	277.92	240	442.15	489	372	441	38	93	12	10	3
12094	8000	5188	1	1632	1224	28382	594	115	759	31	224	169	59	245	301.35	154	454.64	485	285	408	58	96	6	18	14
14278	7624	4326	2	904	714	27930	613	328	993	151	84	240	139	157	275.1	277	438.27	522	379	441	15	75	10	0	1
11266	7990	5462	0	1996	1516	28584	533	91	601	17	324	185	42	277	312.09	115	461.1	496	191	360	85	99	6	26	22
11724	7796	5286	2	1912	1450	28514	557	115	619	23	306	169	60	256	306.77	116	457.1	458	209	402	91	88	13	38	24
7160	11370	8034	370	946	638	28242	915	38	742	11	86	93	18	137	286.36	125	446.16	437	366	364	37	49	2	8	3
5376	12754	9154	621	502	290	28102	1084	10	753	0	26	65	7	86	279.38	113	436.37	372	380	363	19	45	0	0	4
13154	7772	4802	1	1358	940	28134	653	196	875	67	104	191	103	210	281.43	153	445.64	423	363	456	48	100	7	4	6
11788	7966	5246	0	1814	1370	28458	581	121	704	25	246	155	72	266	298.39	154	455.88	481	268	415	70	92	4	28	4
4454	13364	9644	850	384	218	28070	1226	10	628	0	6	63	7	80	275.2	94	430.22	309	302	295	15	41	1	0	2

13234	8030	4790	10	1040	832	28052	630	231	969	105	110	216	90	195	276.76	223	442.24	478	387	438	26	87	16	14	4
11578	7896	5418	2	1930	1386	28512	531	111	649	21	272	174	55	249	305.83	130	456.47	506	195	388	95	97	6	30	18
14506	7626	4388	4	736	522	27832	651	346	1095	177	50	265	127	135	270.82	290	435.04	489	447	428	19	73	19	4	2
4380	13316	9658	840	450	266	28092	1243	9	602	0	22	56	6	71	273.98	83	428.86	306	309	297	20	36	0	0	2
4520	13246	9590	811	450	262	28086	1219	10	629	1	18	72	6	81	274.23	87	429.34	316	330	289	16	53	0	0	2
11824	7760	5258	2	1932	1406	28476	591	119	668	27	266	172	58	258	304.6	129	456.69	478	239	378	68	100	8	28	17
5580	12502	8946	564	688	422	28162	1076	6	716	1	22	86	2	115	279.66	114	437.94	389	373	312	12	62	0	2	3
5406	12648	9066	639	626	378	28146	1091	10	680	0	20	68	9	93	281.28	112	437.92	383	330	341	21	46	1	2	0
7378	11064	7840	308	1130	770	28298	913	34	747	4	110	78	19	145	288.68	128	446.46	455	389	354	32	48	2	6	4
4034	13568	9854	929	380	222	28072	1291	7	588	1	14	63	5	72	270.84	94	423.13	295	289	259	12	40	0	0	1
4682	13154	9494	785	472	278	28096	1178	4	634	0	16	71	2	78	275.68	104	430.3	351	306	303	14	44	0	0	1
13278	7754	4684	3	1290	990	28150	619	203	888	72	136	194	100	213	283.03	186	446.78	478	337	432	40	101	9	18	3
12890	7876	4862	1	1364	1036	28202	608	196	847	68	164	188	99	203	284.11	179	448.84	492	324	438	51	94	12	10	4
12354	7832	5044	2	1666	1222	28332	592	156	727	44	202	176	68	255	300.77	145	450.24	462	264	395	70	92	10	10	5
14216	7610	4440	6	912	660	27914	645	316	998	156	64	252	120	175	275.29	225	437.59	447	401	444	28	88	15	12	0
13104	7718	4726	3	1410	1076	28214	583	196	826	64	168	213	94	213	289.48	179	450.55	496	286	406	56	107	13	10	8
5270	12690	9160	602	622	370	28150	1121	9	689	2	36	68	7	99	280.02	79	437.1	350	395	332	23	42	0	2	2
13278	7964	4788	10	1106	820	28058	615	247	932	121	98	241	102	169	276.63	225	442.45	501	353	422	43	101	13	4	3
4126	13476	9814	909	408	230	28072	1267	10	593	1	18	58	6	75	271.87	80	423.8	291	288	284	13	42	0	0	4
13576	7900	4700	6	986	736	27996	646	246	1026	113	96	247	98	160	276.12	256	442.13	482	407	416	36	103	15	2	5
4358	13362	9686	872	414	240	28078	1251	5	628	1	18	64	3	73	272.69	97	428.14	302	311	286	17	41	0	0	2
5484	12468	9010	609	708	438	28162	1073	16	670	4	52	78	10	107	283.12	114	439.69	377	332	335	23	51	0	2	2
11736	7868	5208	1	1904	1474	28502	564	102	638	21	270	183	48	260	304.34	126	456.87	491	209	364	78	93	11	40	21
11022	8060	5492	2	2072	1620	28642	524	89	563	15	336	164	48	270	316.15	115	464.8	487	184	382	100	79	6	38	32
4150	13490	9808	894	390	216	28066	1264	9	588	1	12	56	5	73	272.81	96	426.53	296	296	285	13	36	0	0	3
11392	7762	5388	5	2140	1562	28584	528	103	576	25	308	185	45	287	313.35	96	458.37	455	184	385	104	100	6	30	18
4382	13344	9650	846	434	264	28094	1220	6	603	0	20	52	5	72	275.75	107	432.03	333	290	303	13	37	0	0	1
13200	7790	4868	1	1288	860	28114	616	210	897	74	96	205	104	195	282.79	202	446.64	484	330	441	38	114	15	12	6
4480	13306	9616	828	416	246	28080	1197	7	621	1	14	66	4	81	276.38	99	431.03	328	290	307	13	46	0	2	2
7002	11390	8116	383	1000	654	28258	969	21	779	5	92	78	11	115	288.08	123	445.31	452	404	341	29	48	0	4	4
4322	13416	9710	852	390	224	28074	1233	7	616	2	12	55	3	65	273.72	98	427.62	325	312	301	12	42	0	0	3
4528	13176	9534	773	504	334	28122	1172	5	623	0	44	60	4	92	279.88	96	435.94	328	320	319	17	38	0	2	4
5844	12354	8836	494	680	408	28152	1020	13	781	2	30	79	9	97	283.92	128	440.63	411	386	360	24	56	1	0	3
7620	11004	7736	320	1086	728	28262	901	51	757	19	76	107	26	150	288.59	139	446.11	438	364	355	33	51	2	10	6
12798	7974	4938	2	1330	986	28166	605	186	893	71	118	205	84	189	283.44	196	446.44	494	329	434	48	104	12	22	10
4708	13052	9500	775	542	294	28120	1198	6	641	0	24	60	4	87	278.52	88	434.33	318	334	311	23	47	0	0	0

SUPPLEMENTARY INFORMATION:Monte Carlo Atomistic Simulation and Machine Learning Analysis of Na-K Eutectic Alloy in Condensed Phases, D. Reitz and E. Blaisten-Barojas, George Mason University, Fairfax, VA 22030

11382	7848	5386	0	2052	1564	28608	560	82	610	13	334	146	43	276	313.34	109	461.04	474	205	386	99	79	11	42	19
4478	13232	9604	825	496	282	28110	1209	5	612	0	18	63	4	81	276.93	89	433.73	331	297	293	14	46	0	0	3
6812	11540	8186	424	974	648	28232	956	31	710	10	64	91	14	143	288.5	98	446.04	401	364	359	29	59	0	6	9
4024	13698	9898	927	262	150	28036	1264	4	589	1	4	68	3	59	272.75	97	426.42	304	278	287	14	41	0	0	1
11900	7946	5148	2	1764	1396	28450	572	124	718	41	268	171	54	236	301.99	145	454.38	517	266	387	73	71	9	28	14
14300	7690	4378	4	824	636	27896	636	309	1080	151	64	237	122	132	269.61	283	433.37	515	440	455	19	72	16	4	1
5356	12732	9128	623	544	328	28114	1112	6	721	1	26	66	2	93	278.95	99	437.3	361	370	339	14	43	0	0	6
4740	13024	9420	756	542	358	28136	1164	3	638	0	44	68	3	84	284.9	108	437.9	361	307	301	16	52	0	8	2
4514	13262	9584	817	462	264	28092	1227	9	613	0	6	68	6	79	274.86	92	430.5	312	313	288	16	47	0	0	3
5462	12546	9062	575	662	382	28148	1098	9	741	5	32	62	3	98	281.08	117	437.75	360	402	351	20	33	0	2	6
13946	7742	4522	3	980	702	27950	619	285	1004	139	58	250	121	166	274.02	240	437.6	497	411	434	25	90	9	0	1
11544	7868	5368	2	1950	1470	28550	560	93	627	18	312	158	52	277	315.16	131	462.45	465	221	376	90	72	6	36	24
10884	7894	5682	1	2222	1608	28694	554	73	560	12	362	141	42	272	321.15	105	463.5	491	192	358	108	71	4	42	26
11718	7814	5284	0	1936	1444	28522	536	102	645	24	296	168	55	255	304.21	134	457.46	493	208	408	92	94	8	30	18
4868	13030	9416	697	504	276	28106	1129	8	661	0	12	50	5	92	278.09	109	433.25	351	335	354	15	32	0	0	1
4316	13400	9694	869	416	244	28080	1243	8	595	2	10	56	3	68	273.51	97	426.47	315	295	298	13	37	0	0	3
4410	13246	9642	815	502	282	28100	1207	5	593	0	16	62	2	76	273.88	79	428.39	320	304	305	21	40	0	2	3
5404	12536	9014	649	718	458	28172	1116	10	656	2	38	66	6	107	281.61	99	437.56	360	333	324	23	34	0	4	0
11702	8054	5264	4	1756	1398	28492	585	104	665	21	286	154	60	241	301.06	141	454.23	494	233	404	82	90	6	32	14
14446	7686	4386	4	742	540	27846	673	339	1096	162	42	267	135	147	269.28	258	432.14	464	468	425	20	98	23	4	0
13486	7884	4668	0	1052	832	28050	622	220	997	76	120	226	109	186	281.83	202	445.76	457	385	439	49	126	12	6	4
3834	13726	10006	990	306	160	28042	1310	6	570	1	10	63	4	60	269.43	94	422.08	281	266	266	12	42	0	0	3
11406	7926	5314	0	2000	1580	28590	524	107	630	19	320	156	56	281	316.01	121	461.36	511	201	378	86	88	10	42	17
3956	13658	9916	971	324	188	28056	1305	8	574	1	14	56	3	67	272.34	93	425.34	278	274	273	13	33	0	0	2
13342	7984	4752	10	1046	806	28036	659	237	976	100	100	221	107	187	278.51	227	443.85	467	398	429	27	93	13	6	1
12464	7766	4896	0	1640	1316	28352	553	147	733	42	246	198	77	265	299.59	154	451.34	452	249	414	75	96	8	24	8
12986	7790	4748	6	1396	1100	28206	560	213	776	71	172	193	111	197	283.62	179	447.31	496	273	468	62	96	15	14	7
5200	12762	9188	629	606	360	28140	1080	8	688	1	22	61	4	102	276.83	114	433.01	380	344	356	15	39	0	2	1
14440	7640	4334	4	794	600	27864	632	340	1059	168	54	263	133	159	268.1	266	430.23	484	426	439	16	82	15	2	0
13986	7708	4492	6	988	720	27962	636	290	988	124	62	225	137	166	276.07	221	440.91	456	405	463	38	84	11	6	3
5284	12576	9080	589	746	462	28190	1089	7	694	1	42	66	4	114	281.02	107	436.85	376	362	328	20	44	0	0	1
5950	12330	8824	571	644	360	28124	1081	25	748	5	14	69	15	97	283.35	119	438.83	383	381	338	20	41	0	2	0
13376	7966	4780	6	1040	770	28032	669	232	968	111	94	225	98	186	278.55	205	443.53	462	397	415	31	91	10	6	1
4406	13284	9630	826	474	288	28110	1226	4	599	0	28	48	3	82	276.07	86	431.39	312	316	309	16	30	0	0	2
4694	13100	9490	741	514	292	28108	1193	3	659	0	18	65	2	78	278.52	87	433.55	330	356	312	17	47	0	0	4
13874	7722	4420	5	1022	852	28010	607	244	945	98	114	246	108	186	277.54	215	443.43	476	343	429	35	116	16	4	6

SUPPLEMENTARY INFORMATION:Monte Carlo Atomistic Simulation and Machine Learning Analysis of Na-K Eutectic Alloy in Condensed Phases, D. Reitz and E. Blaisten-Barojas, George Mason University, Fairfax, VA 22030

14418	7696	4344	8	764	586	27856	635	354	1066	170	44	258	146	166	267.78	281	429.16	464	434	454	13	79	15	4	3
3986	13698	9930	985	264	146	28034	1326	10	592	1	10	62	5	71	272.86	89	425.53	259	296	259	13	40	0	0	1
4584	13200	9562	777	466	268	28100	1180	2	649	0	16	74	2	85	277.95	102	432.68	338	319	305	13	50	0	2	3
5144	12838	9244	660	554	322	28124	1146	8	685	2	22	66	3	86	278.77	102	435.22	364	366	316	17	49	1	0	0
13740	7580	4508	2	1188	906	28062	595	264	896	99	134	211	123	182	277.35	203	443.68	511	335	450	32	92	16	6	3
4440	13248	9596	806	522	302	28116	1211	6	585	0	8	49	4	79	276.64	96	431.8	334	293	308	11	25	0	0	3
4484	13244	9602	789	486	274	28102	1191	3	636	0	12	57	3	84	275.68	102	432.33	333	309	314	13	37	0	0	2
6064	12078	8706	478	824	478	28192	1006	21	722	0	40	68	16	111	285.71	121	441.35	425	380	355	14	43	0	2	6
4360	13342	9660	821	448	270	28100	1249	5	606	0	18	53	2	75	276.23	87	431.9	308	330	291	14	34	0	2	4
13716	7834	4630	7	994	730	27986	631	281	991	124	74	218	119	180	276.31	238	440.74	473	393	450	26	75	16	8	4
4068	13584	9830	930	360	216	28066	1266	9	561	1	8	61	6	67	272.42	97	425.72	299	270	287	16	35	0	0	1
4590	13248	9600	819	410	212	28068	1191	10	644	0	8	57	7	75	274.87	98	429.65	330	297	327	9	44	0	0	5
4566	13280	9580	795	396	236	28076	1191	5	643	0	16	59	5	70	276.09	98	430.26	327	320	325	19	34	0	2	2
13616	7704	4592	2	1140	872	28056	586	255	909	97	124	207	117	203	276.03	189	446.12	466	339	475	38	92	17	6	5
4084	13582	9850	926	326	194	28056	1287	8	589	0	20	66	4	67	272.26	91	425.77	292	301	262	16	35	0	0	1
7320	11256	7958	324	982	638	28236	891	26	803	4	78	85	17	136	286.02	131	447.01	438	386	406	32	55	1	4	4
3980	13754	9934	966	218	130	28026	1308	10	603	2	10	52	5	63	270.82	76	422.45	267	300	288	13	36	0	0	2
4516	13204	9544	802	514	318	28118	1198	5	612	0	22	58	4	75	276.75	108	432.15	344	294	303	16	41	0	0	2
13788	7820	4586	3	940	730	27964	606	290	997	128	90	224	118	170	275.11	241	439.62	481	393	464	31	80	21	10	5
4134	13494	9816	929	392	218	28068	1296	8	591	1	14	71	6	61	273.57	95	426.28	288	296	256	21	47	0	0	2
5948	12152	8764	549	772	474	28172	1038	19	677	4	60	75	10	113	286.42	107	440.75	392	336	347	25	43	0	2	1
14062	7786	4474	3	862	668	27926	636	282	1049	143	70	251	102	166	273.78	245	439.25	473	416	438	27	88	14	4	1
13094	7930	4818	5	1244	924	28130	627	207	916	58	102	173	105	187	283.87	207	445.19	491	348	461	38	93	17	18	4
10988	7976	5474	0	2180	1684	28682	518	72	546	10	336	175	31	287	315.67	109	465.36	480	177	350	112	84	4	40	28
4904	12888	9376	697	580	336	28126	1113	5	632	0	42	79	5	94	280.19	91	437.6	363	331	322	20	46	0	0	2
3802	13692	9962	986	372	228	28074	1302	8	533	1	16	51	4	79	270.79	81	424.28	268	257	279	13	31	0	2	2
14464	7644	4350	7	780	570	27854	622	346	1049	166	44	267	139	160	270.64	259	433.5	486	430	444	14	87	20	2	1
5170	12872	9230	635	524	306	28114	1095	6	717	0	12	60	4	99	278.22	123	434.32	369	364	357	14	40	0	0	0
4718	13126	9486	770	468	278	28104	1195	8	650	0	28	66	5	80	276.83	89	432.59	332	344	302	19	49	0	0	1
13130	7832	4812	0	1258	948	28144	593	186	918	73	152	217	80	211	285.08	202	449.8	465	309	441	47	112	13	12	9
4896	12868	9324	709	624	392	28150	1151	10	613	1	42	59	6	93	283.43	87	438.81	338	328	322	25	38	0	4	4
12148	7946	5078	2	1676	1290	28370	563	134	732	34	202	174	70	235	301.13	154	453.14	481	257	417	78	92	7	26	13
12590	7890	4968	0	1488	1132	28274	575	175	759	54	186	185	80	234	293.72	149	452.74	483	280	417	63	94	10	18	8
5400	12664	9080	578	624	364	28146	1109	4	718	0	14	45	4	80	280.16	101	437.92	383	392	353	19	32	0	0	2
4190	13536	9820	907	312	170	28042	1265	7	623	0	14	61	3	71	272.62	92	424.96	284	305	295	14	44	0	0	3
14512	7684	4344	7	712	534	27830	637	341	1121	172	42	270	122	156	267.8	278	429.59	474	458	441	14	87	20	2	1

SUPPLEMENTARY INFORMATION:Monte Carlo Atomistic Simulation and Machine Learning Analysis of Na-K Eutectic Alloy in Condensed Phases, D. Reitz and E. Blaisten-Barojas, George Mason University, Fairfax, VA 22030

13492	7676	4520	2	1270	1018	28130	607	238	850	110	146	226	98	209	281.17	201	445.61	494	317	411	39	90	11	8	1
4878	12834	9380	719	670	360	28144	1138	9	609	0	18	68	8	92	283.02	93	439.01	348	297	320	26	47	0	4	2
4334	13474	9728	883	324	182	28048	1254	8	642	3	6	50	5	68	271.72	97	424.98	296	315	316	11	35	0	0	2
4984	12964	9356	703	510	278	28102	1149	4	696	1	10	66	2	81	277.76	103	433.41	353	362	325	18	42	0	0	2
14478	7442	4312	4	926	664	27902	654	334	1017	164	72	263	122	161	271.72	244	438.87	471	399	420	20	93	30	8	4
14314	7688	4386	5	806	624	27890	637	319	1066	152	64	273	132	158	270.76	279	433.06	488	418	415	20	105	14	8	2
11276	7910	5380	0	2072	1602	28606	558	96	564	22	330	172	51	265	313.46	125	458.78	491	189	351	94	87	6	34	26
13852	7724	4484	3	1018	810	28000	631	280	969	123	104	243	117	165	274.93	230	440.45	470	382	431	46	103	18	8	3
4246	13512	9802	911	308	162	28044	1262	9	617	2	14	67	4	60	272.19	100	425.86	306	289	282	16	48	0	0	1
5032	12878	9292	692	566	334	28128	1143	6	669	0	26	63	3	85	276.88	98	434.21	359	346	322	16	43	1	0	5
4662	13122	9486	733	524	310	28124	1162	4	647	0	20	53	2	89	277.51	107	435.2	342	328	327	15	35	0	0	4
10810	8032	5662	1	2150	1632	28704	531	57	557	5	376	137	34	277	318.17	107	464	494	192	389	100	67	4	40	31
7822	10952	7670	325	1006	678	28222	925	41	794	7	92	90	25	143	287.43	129	445.72	415	395	380	30	59	3	2	5
4262	13468	9750	868	364	210	28068	1233	9	596	4	14	59	3	76	274.16	86	428.94	304	293	308	14	39	0	0	2
13454	7642	4526	4	1300	1046	28136	602	236	837	96	160	219	96	228	277.87	207	446.65	470	304	430	28	92	21	8	9
4510	13198	9574	822	514	296	28106	1225	4	616	0	10	63	2	83	276.35	99	432.35	323	318	290	10	45	0	4	3
4210	13472	9764	879	382	226	28070	1266	5	608	1	14	59	1	62	272.58	90	425.66	299	311	286	19	36	0	2	3
4158	13560	9820	907	320	182	28052	1274	7	607	1	12	65	4	76	271.37	81	425.7	266	309	294	21	45	0	0	1
4776	13144	9446	751	438	270	28094	1186	10	680	1	20	53	4	74	278.07	102	433.22	345	358	314	13	37	0	0	4
4244	13458	9750	885	382	220	28064	1254	8	609	1	8	65	5	78	273.37	87	427.94	289	301	291	17	49	0	2	1
5192	12806	9230	644	564	314	28120	1138	1	730	0	12	67	1	90	279.58	101	435.19	364	387	321	14	43	0	2	1
4078	13508	9808	894	418	254	28084	1260	5	585	0	16	56	3	88	273.22	93	428.25	279	287	296	12	37	0	2	3
4006	13660	9912	969	290	162	28042	1309	8	598	1	10	60	4	62	271.88	99	423.07	284	290	264	10	43	0	2	3
4452	13306	9606	838	440	272	28096	1236	6	615	0	20	66	3	86	275.39	99	429.7	310	304	278	16	40	0	0	0
4598	13194	9558	791	460	262	28092	1202	3	654	0	20	67	1	100	275.49	101	430.1	299	321	309	15	47	0	0	1
14338	7706	4396	3	768	592	27866	642	338	1045	168	60	273	130	147	270.06	282	433.51	500	428	414	15	94	19	6	2
11226	7926	5498	0	2094	1534	28618	562	97	584	23	294	166	50	281	316.3	97	464.18	460	202	354	103	85	2	42	24
11072	7760	5540	0	2218	1676	28714	546	85	524	17	388	181	43	297	324.21	80	467.41	437	170	352	112	106	6	54	32
12018	8074	5154	2	1662	1258	28378	587	130	731	32	198	164	72	236	296.74	166	453.16	514	254	397	58	82	8	14	10
14370	7674	4402	7	810	572	27868	643	326	1049	151	40	251	129	147	271.91	256	436.99	482	431	452	19	83	23	0	1
5330	12646	9110	645	638	386	28144	1117	14	661	3	32	75	9	105	281.17	94	439.63	375	351	310	11	49	1	2	1
4110	13562	9832	932	354	196	28058	1288	9	596	1	4	51	5	61	272.65	97	426.27	296	297	280	11	33	0	0	4
11610	7924	5258	1	1890	1506	28546	588	100	643	17	324	151	58	253	307.74	127	456.81	467	234	397	94	84	6	30	17
4130	13594	9836	902	312	174	28052	1266	8	607	1	6	58	5	68	274.81	94	428.03	290	309	294	18	41	0	0	0
4844	13014	9420	721	530	290	28110	1160	5	650	0	12	65	3	86	278.08	99	433.78	345	334	317	19	43	0	0	1
13510	7844	4592	1	1124	888	28070	642	247	950	112	102	210	106	197	277.34	223	442.3	469	370	444	25	81	16	10	5

SUPPLEMENTARY INFORMATION:Monte Carlo Atomistic Simulation and Machine Learning Analysis of Na-K Eutectic Alloy in Condensed Phases, D. Reitz and E. Blaisten-Barojas, George Mason University, Fairfax, VA 22030

12650	7764	4918	3	1582	1176	28296	583	172	780	45	188	183	93	234	291.47	138	449.05	469	291	430	67	100	15	16	7
14260	7634	4418	5	880	642	27904	646	300	1059	149	68	253	121	157	271.3	266	436.76	479	421	434	24	95	18	2	0
4314	13422	9738	875	376	204	28066	1255	12	604	2	10	62	7	80	273.89	87	426.78	301	316	287	9	38	0	2	1
4100	13562	9864	939	342	174	28046	1288	11	593	1	4	63	7	60	271.36	102	425.13	290	288	274	15	45	0	0	3
11876	7874	5194	1	1840	1392	28462	523	121	670	29	260	197	63	272	302.33	134	455.72	474	225	403	83	114	8	24	15
11042	7878	5520	1	2178	1664	28702	534	80	548	14	370	159	45	266	321.28	108	466.5	488	185	367	114	80	4	44	29
10628	7812	5806	2	2320	1716	28784	430	73	491	12	440	170	42	299	331.8	95	469.78	485	130	386	141	82	2	60	33
12132	7736	5090	4	1846	1364	28430	550	144	680	35	232	183	76	248	297.19	133	456.65	508	234	392	72	111	7	26	12
3990	13612	9896	961	356	196	28056	1292	5	577	1	4	61	4	71	270.24	91	422.51	283	275	276	11	42	0	2	2
5006	12890	9322	679	566	320	28126	1122	6	675	1	22	64	4	83	277.72	104	435.1	370	332	331	22	45	0	0	1
5290	12726	9170	648	578	334	28124	1113	9	710	1	24	76	4	94	280.2	109	438.32	361	359	322	23	55	1	2	2
13404	7756	4704	0	1188	888	28082	623	240	903	99	138	208	107	172	278.39	227	443.07	518	343	428	35	91	12	4	1
11574	7926	5386	0	1896	1404	28504	526	122	638	23	294	171	71	242	305.9	132	455.54	492	195	414	86	96	9	22	27
4156	13514	9824	919	364	192	28058	1273	2	619	0	8	64	1	69	273.83	109	427.03	299	291	274	12	48	0	0	2
13596	7806	4648	3	1094	792	28018	652	252	978	116	74	221	98	174	276.98	219	444.25	475	402	427	33	88	15	6	3
5006	12786	9256	653	686	404	28160	1144	8	658	0	22	57	6	91	278.35	85	434.87	355	363	323	24	36	0	0	1
4514	13296	9626	840	402	220	28070	1239	7	652	0	12	63	4	96	275.18	88	429.42	286	326	299	9	49	0	0	2
11828	7856	5290	2	1850	1350	28462	561	138	651	30	256	154	74	256	303.18	135	459.25	490	230	402	75	83	13	32	16
4104	13556	9826	918	360	208	28064	1264	7	569	1	10	59	3	70	272.23	79	426.37	292	279	292	15	37	0	0	3
11508	7758	5302	1	2048	1596	28612	580	102	596	18	352	156	54	268	314.56	107	460.74	457	224	368	103	85	7	44	27
10362	7800	5684	0	2552	1984	28920	495	62	438	6	490	153	30	297	329.62	72	473.08	511	139	331	121	70	6	42	47
4890	12906	9374	717	602	336	28130	1157	6	640	0	22	73	3	81	278.27	93	434.53	361	330	294	19	49	0	0	5
5324	12750	9168	611	538	310	28116	1109	9	741	0	26	68	7	92	279	108	435.83	354	401	345	20	48	0	0	5
7824	10918	7588	308	1082	754	28248	890	62	733	14	68	104	37	166	288.44	128	445.41	413	359	375	32	59	2	12	6
6502	11836	8394	474	836	564	28202	1017	30	731	5	64	76	18	134	288.57	103	445.23	384	384	342	24	46	1	6	6
14458	7522	4338	8	880	622	27876	659	327	1041	171	50	269	118	153	269.97	252	432.4	485	443	411	16	88	17	4	1
5094	12720	9246	659	684	382	28150	1120	10	634	3	24	70	6	102	280.6	83	436.67	347	336	330	20	44	0	0	6
10982	7682	5436	0	2326	1858	28796	478	79	459	10	442	174	41	304	331.26	80	471.81	459	120	370	132	91	4	62	38
5018	12914	9324	695	526	304	28112	1153	14	660	0	26	66	11	87	281.34	109	436.38	352	343	308	19	40	0	0	2
10808	7932	5624	1	2246	1698	28738	522	71	530	11	390	147	37	248	323.63	115	466.27	534	183	367	101	67	1	36	32
13720	7856	4652	5	930	704	27964	637	270	1029	114	100	227	127	162	276.8	234	438.89	488	419	442	30	89	14	2	0
4568	13280	9570	831	400	242	28076	1208	9	633	1	14	50	4	84	275.06	106	428.95	316	299	318	8	36	0	2	5
13762	7864	4606	4	924	720	27976	646	260	1022	111	90	226	114	171	275.77	239	440.53	490	409	429	20	99	14	10	3
4488	13300	9642	820	414	218	28068	1205	8	629	0	6	60	3	88	275.19	92	431.89	309	317	309	12	37	0	0	4
3980	13648	9906	949	330	182	28050	1291	9	578	1	4	59	6	61	272.8	101	426.81	293	285	269	15	39	0	0	2
12104	7848	5136	1	1738	1310	28404	553	124	700	32	236	189	66	248	301.77	152	453.54	487	254	403	75	103	4	32	14

13854	7562	4478	3	1152	872	28052	596	288	888	122	128	230	125	167	277.79	221	444.24	513	340	431	44	86	18	6	2
4820	13152	9464	752	404	228	28082	1174	8	682	0	14	59	7	76	277.05	110	432.36	338	343	332	14	43	0	0	3
4646	13190	9530	783	442	258	28084	1184	9	627	0	16	72	6	88	274.04	106	430.11	325	308	307	13	50	0	2	4
4790	13010	9424	719	566	320	28126	1175	9	651	0	16	60	8	84	278.07	100	435.14	339	340	321	17	41	0	0	3
4582	13140	9532	775	532	312	28122	1170	5	624	0	22	61	4	90	278.72	93	434.61	328	307	328	21	34	0	2	1
5706	12346	8884	543	756	450	28176	1031	16	705	0	34	69	11	109	283.69	116	439.22	415	342	345	20	45	0	0	0
3990	13592	9862	946	382	232	28072	1260	8	550	2	14	56	3	67	272.49	94	427.38	296	247	294	18	29	0	0	3
12558	7942	5056	4	1454	1050	28238	596	161	841	52	168	191	82	219	286.81	176	448.71	502	307	418	44	115	11	10	7
5852	12274	8808	543	746	456	28178	1070	19	723	4	38	67	13	123	285.52	101	440.94	377	390	340	14	42	1	2	2
5156	12766	9264	638	600	320	28128	1103	8	693	0	22	60	5	93	280.53	100	437.97	366	364	349	16	30	0	0	4
12170	8068	5236	2	1496	1100	28288	568	139	820	38	208	176	67	225	287.45	176	452.33	513	292	422	59	103	10	10	5
7366	11200	7918	318	1012	660	28238	898	36	748	9	76	100	16	128	286.89	122	446.21	461	388	355	37	63	3	6	3
3732	13768	10032	999	328	186	28058	1321	5	533	1	10	59	3	74	270.09	86	421.6	257	260	270	14	36	0	2	2
6020	12108	8670	478	828	530	28216	980	18	699	5	58	67	7	108	286.61	127	444.56	443	345	368	21	37	0	2	5
11944	8078	5230	2	1642	1244	28372	573	138	710	36	210	178	61	230	300.64	158	455.13	505	259	384	77	96	5	22	10
4938	12900	9334	700	586	348	28138	1152	8	658	0	32	63	4	94	279.53	101	436.64	360	343	309	13	44	0	0	2
13508	7540	4518	0	1348	1060	28152	579	221	820	82	158	214	109	217	281.3	195	445.86	486	293	446	42	111	18	20	4
5348	12756	9120	646	526	334	28116	1111	13	721	0	32	75	9	86	279.4	130	436.76	382	363	323	15	53	0	0	2
11726	7882	5278	2	1886	1412	28480	540	122	640	29	260	168	63	251	302.73	124	455.49	480	224	427	91	87	8	34	15
11568	8080	5350	2	1806	1392	28500	548	99	653	17	274	169	56	250	305.54	139	458.41	499	220	401	88	99	5	30	14
5132	12740	9192	641	670	398	28152	1136	6	692	2	20	76	2	94	280.84	104	435.37	359	375	305	21	43	0	0	4
12974	7744	4752	3	1462	1120	28234	568	210	787	59	170	192	114	198	286.33	186	449.4	515	273	451	54	103	17	12	9
13906	7688	4436	6	998	838	28006	597	280	977	119	126	225	130	183	276.57	226	441.96	479	369	467	33	87	12	14	4
13040	7880	4824	7	1314	960	28142	614	204	882	71	118	199	98	198	280.58	182	446.24	497	345	426	35	99	13	6	9
4538	13226	9592	813	466	256	28090	1212	8	645	1	12	66	5	84	274.08	112	429.57	325	309	295	12	53	0	0	1
4344	13464	9718	875	334	190	28058	1250	7	623	0	8	61	5	69	276.11	114	429.92	308	304	289	13	40	0	0	2
12124	8006	5068	4	1648	1292	28368	596	126	744	35	208	165	69	259	290.88	148	450.94	466	264	418	55	95	7	16	11
13688	7612	4530	1	1188	910	28066	600	258	896	110	124	215	116	204	278.21	202	446.88	462	334	472	35	90	18	14	3
4766	13006	9430	721	552	336	28130	1164	10	656	0	38	56	3	94	280.51	95	434.67	326	345	322	25	42	0	2	2
14316	7482	4266	4	994	788	27950	580	316	974	169	96	259	102	157	271.93	259	436.07	533	367	438	24	80	29	6	2
4848	13052	9396	722	492	300	28112	1155	4	662	0	24	64	2	96	276.66	108	433.5	340	340	326	14	45	0	0	1
12406	7726	5046	1	1698	1240	28364	568	155	700	39	228	186	81	264	303.73	127	453.2	442	264	415	79	106	5	16	12
11442	7888	5360	1	2010	1532	28584	532	119	585	22	316	169	70	260	316.59	115	461.39	491	195	386	100	92	10	32	22
11806	8078	5204	2	1710	1346	28406	566	122	731	32	244	172	65	262	297.08	142	452.37	504	267	399	61	92	7	18	7
12214	7846	5038	1	1674	1326	28382	574	134	737	42	258	177	70	239	293.93	132	455.13	483	266	426	69	98	7	26	11
6878	11652	8266	407	830	506	28180	977	28	808	5	46	72	15	139	285.51	121	443.44	400	419	383	17	48	2	2	2

SUPPLEMENTARY INFORMATION:Monte Carlo Atomistic Simulation and Machine Learning Analysis of Na-K Eutectic Alloy in Condensed Phases, D. Reitz and E. Blaisten-Barojas, George Mason University, Fairfax, VA 22030

4046	13634	9902	976	290	154	28036	1309	9	612	1	8	62	5	53	271.02	108	423.34	288	287	266	14	40	0	2	2
13830	7800	4596	5	946	696	27950	646	272	1042	139	82	247	102	179	276.09	244	437.77	474	417	427	19	93	17	0	1
4174	13550	9804	905	334	188	28056	1256	5	601	0	6	61	3	61	272.67	104	426.92	307	287	295	15	41	0	0	2
14252	7614	4386	5	930	676	27918	642	313	1002	154	58	255	112	138	271.65	255	434.19	505	404	426	28	87	20	2	1
4234	13494	9764	892	352	206	28064	1249	5	616	1	14	52	2	71	274.6	105	429.7	302	296	305	13	39	0	0	2
10946	7832	5538	0	2262	1716	28716	500	89	513	9	360	159	54	305	320.37	94	465.6	472	147	378	112	89	2	60	26
5622	12374	8894	559	766	488	28194	1056	19	664	4	42	69	11	102	285.25	94	441.82	397	365	333	30	38	0	8	1
3942	13646	9910	956	336	208	28066	1287	8	565	2	22	53	4	80	271.84	82	425.34	261	277	296	17	36	0	2	2
13778	7708	4562	4	1046	804	28028	632	245	943	89	122	217	108	167	277.49	215	445.05	484	365	448	38	103	22	8	3
10940	7862	5498	2	2262	1738	28720	498	74	514	17	374	181	41	266	318.98	106	464.03	520	152	342	118	92	2	44	31
4214	13456	9758	886	392	236	28078	1243	9	593	1	20	64	4	55	272.89	96	426.21	328	289	282	20	45	0	2	2
12094	7962	5210	0	1720	1194	28358	557	137	665	28	160	191	65	248	301.81	141	455.92	490	235	397	71	97	10	16	9
6410	11698	8400	434	986	670	28276	996	16	698	5	102	61	8	140	288.13	117	445.23	402	361	355	28	36	0	10	5
4502	13226	9606	800	480	266	28096	1205	5	625	1	16	51	2	71	276.77	91	431.7	324	328	322	19	29	0	0	3
12060	7834	5150	4	1790	1322	28410	545	135	675	28	228	187	68	261	303.16	132	457.83	497	233	379	74	99	8	24	14
4154	13490	9798	904	392	226	28076	1263	6	591	2	12	60	4	79	274.21	94	427.49	292	284	286	13	32	0	4	2
10870	7984	5496	1	2152	1768	28746	502	71	545	8	418	154	38	295	320.99	116	463.19	520	167	358	111	85	1	54	18
13606	7752	4610	7	1116	840	28034	604	281	905	116	100	232	115	171	277.26	210	443.9	505	357	433	38	96	24	10	3
4980	12920	9306	684	542	342	28128	1135	9	665	0	38	77	4	80	279.27	110	437.95	377	342	296	22	44	0	0	2
4744	13076	9458	776	516	298	28110	1205	10	644	0	16	57	7	79	276.55	101	431.37	334	333	301	15	38	0	2	1
14354	7548	4398	3	906	624	27888	599	336	996	158	58	268	138	174	271.44	251	435.36	488	387	440	19	91	15	0	1
14246	7736	4430	6	798	608	27880	651	312	1071	169	54	265	104	144	269.65	271	431.18	489	425	417	23	86	22	8	2
3992	13638	9892	951	342	190	28056	1290	5	585	1	2	54	2	63	271.51	92	423.56	293	274	280	13	38	0	0	1
4662	13150	9504	773	486	284	28104	1199	8	636	1	18	69	4	87	275.47	103	430.39	323	325	299	14	40	0	0	3
5366	12722	9100	638	572	350	28132	1129	14	699	1	22	66	8	86	280.78	115	438.41	371	369	316	20	43	0	0	2
12734	7846	4898	2	1442	1122	28260	578	159	829	52	200	204	81	220	291.13	189	450.82	490	302	413	60	103	8	18	10
6724	11634	8314	382	902	570	28222	970	24	789	3	74	78	16	118	287.03	138	441.93	459	405	350	18	58	0	4	0
4784	13106	9472	749	470	254	28096	1159	4	666	1	10	53	3	86	276.99	103	433.74	335	321	346	15	38	0	0	2
4508	13288	9624	831	410	230	28078	1209	8	628	1	18	71	6	80	276.38	101	431.46	317	306	302	17	49	0	0	1
5200	12750	9208	640	608	344	28130	1098	8	716	0	20	66	4	100	278.34	123	435.53	383	346	338	8	49	0	0	2
5198	12802	9188	688	560	348	28124	1149	17	696	3	26	68	9	103	281.75	105	437.75	342	349	312	16	47	0	2	1
4632	13220	9526	774	430	262	28088	1202	5	665	1	14	57	1	94	274.39	97	429	304	350	321	14	37	0	4	2
4288	13478	9742	922	344	196	28056	1280	9	623	1	8	47	4	67	270.35	100	423.43	290	296	297	11	32	0	0	2
13000	7812	4784	4	1376	1046	28176	609	204	833	74	144	204	97	230	284.02	172	447.87	465	305	426	43	102	15	14	4
4476	13244	9612	824	470	270	28092	1213	9	615	2	20	58	4	68	274.14	95	428.77	332	310	302	17	37	0	0	3
5064	12780	9252	660	650	380	28154	1132	8	677	0	26	55	6	88	280.01	100	436.34	368	362	329	21	32	0	2	1

SUPPLEMENTARY INFORMATION:Monte Carlo Atomistic Simulation and Machine Learning Analysis of Na-K Eutectic Alloy in Condensed Phases, D. Reitz and E. Blaisten-Barojas, George Mason University, Fairfax, VA 22030

4720	13076	9478	758	526	294	28110	1182	7	639	0	16	64	5	74	278.05	102	434.42	346	330	304	23	47	0	0	0
11678	7788	5282	2	1966	1484	28544	523	108	593	23	310	196	54	265	312.18	126	461.7	502	177	367	98	111	12	36	13
5200	12730	9180	666	610	382	28150	1123	5	689	0	48	73	4	103	283.1	101	438.66	362	341	315	17	49	0	0	2
5132	12762	9248	641	610	350	28136	1115	7	695	0	34	58	5	91	279.13	95	437.06	368	369	339	18	42	0	0	3
13700	7782	4588	3	1048	790	28004	603	270	971	131	90	252	102	188	276.83	235	442.65	495	365	416	27	100	19	6	2
14322	7634	4372	4	866	640	27892	632	319	1035	156	54	249	127	153	270.39	247	432.8	491	438	446	20	79	19	4	1
13084	7826	4744	4	1318	1036	28176	624	212	843	74	146	214	103	193	284.97	182	448	487	324	407	55	104	13	18	5
13198	7818	4720	4	1266	988	28148	610	224	866	79	138	200	107	205	281.66	196	443.94	485	294	446	40	104	11	20	3
6386	11938	8510	453	788	502	28186	996	23	765	3	62	68	15	121	287.68	119	444.46	399	390	367	28	45	0	0	3
4340	13420	9694	840	380	228	28078	1224	3	630	0	16	66	2	81	275.06	99	429.73	311	321	303	13	44	0	0	1
4848	13032	9402	730	512	298	28108	1158	7	665	1	14	54	3	93	276.04	93	431.53	323	340	345	20	44	0	2	2
5496	12598	9040	624	614	360	28128	1111	9	720	0	18	70	5	94	280.75	111	439	366	353	333	14	54	0	2	6
4664	13118	9532	804	508	268	28102	1215	8	646	1	12	52	7	75	277.48	93	432.6	328	323	307	15	39	0	0	2
4110	13592	9832	922	322	188	28052	1276	5	600	1	8	58	4	74	271.98	92	425.53	280	290	292	13	37	0	0	2
13642	7608	4498	2	1282	954	28090	602	264	863	128	100	233	103	195	277.56	203	445.38	476	315	432	47	85	17	6	4
13212	7960	4694	7	1160	950	28118	612	243	876	100	138	207	106	187	278.29	199	443.86	503	344	438	35	87	16	2	6
4316	13304	9666	850	506	298	28108	1238	8	570	1	18	57	5	71	274.5	81	430.35	335	300	280	9	33	0	0	4
12746	7930	4954	1	1410	1026	28222	625	181	814	54	146	174	101	209	285.42	188	448.81	484	307	446	43	84	11	10	6
14376	7570	4352	6	888	648	27898	603	335	1011	163	58	272	137	142	271.2	277	437.5	531	393	425	21	94	18	4	1
4660	13112	9482	748	540	316	28126	1181	6	640	0	16	52	5	84	277.43	113	431.81	340	328	324	16	33	0	0	1
4316	13470	9718	888	338	206	28064	1255	6	616	0	16	70	3	77	274.79	90	429.74	296	302	280	12	48	0	0	4
4320	13460	9714	877	354	206	28060	1217	14	617	1	6	53	9	77	274.21	95	428.85	314	280	321	11	35	0	0	1
13366	7746	4694	4	1234	918	28092	646	235	864	77	128	198	120	181	277.01	184	445.04	472	345	443	39	98	18	6	5
4386	13280	9654	847	502	272	28098	1240	8	600	0	4	50	2	70	275.05	102	429.53	316	306	296	18	35	0	0	2
4452	13344	9650	842	388	220	28066	1219	11	629	2	12	61	7	74	273.49	108	427.57	315	308	309	13	43	0	0	2
13856	7818	4566	4	952	700	27956	627	275	1021	120	56	238	123	173	275.2	240	439.53	472	407	448	29	101	13	8	2
11228	7978	5360	2	2046	1640	28642	545	87	580	16	348	179	54	270	312.53	113	461.93	466	190	375	115	97	4	42	22
6402	11924	8494	438	800	506	28180	947	26	745	5	54	72	13	127	284.19	125	441.8	419	354	397	19	41	0	0	7
4006	13606	9874	926	356	210	28066	1266	4	587	1	14	65	2	67	270.86	95	424.26	298	278	280	16	44	0	0	3
6678	11764	8366	406	830	508	28196	949	25	789	4	46	76	16	116	286.45	152	443.71	458	385	363	19	43	1	4	2
13524	7654	4566	7	1246	968	28106	581	245	838	101	134	219	109	229	278.83	195	446.11	459	298	465	32	93	15	10	6
4206	13504	9802	901	348	186	28056	1250	9	617	2	10	63	4	59	272.54	102	427.14	310	295	291	20	43	0	0	1
4944	13024	9360	691	462	284	28100	1132	3	703	1	24	61	1	93	277.41	102	433.57	337	351	348	20	41	0	2	2
13596	7976	4730	5	914	670	27960	639	231	1061	107	70	220	100	167	275.79	236	439.53	464	408	467	32	95	15	4	1
12702	7798	4910	5	1490	1142	28260	586	186	781	59	200	194	92	233	286.36	174	447.13	488	291	407	50	98	12	16	10
4686	13026	9446	770	588	358	28136	1205	8	608	0	32	60	5	81	276.11	96	432.7	329	316	296	20	44	0	0	4

SUPPLEMENTARY INFORMATION:Monte Carlo Atomistic Simulation and Machine Learning Analysis of Na-K Eutectic Alloy in Condensed Phases, D. Reitz and E. Blaisten-Barojas, George Mason University, Fairfax, VA 22030

13114	7618	4672	2	1452	1152	28230	595	227	738	77	206	195	114	233	281.09	148	447.24	479	295	434	49	87	17	14	1
4428	13290	9646	820	462	254	28090	1211	7	604	1	10	66	3	77	276.13	93	430.65	328	303	292	16	41	0	0	2
10592	7800	5672	0	2352	1868	28842	482	66	493	12	490	163	35	290	333.42	94	471.49	475	155	370	124	84	0	64	45
4800	12994	9438	726	558	308	28118	1161	3	648	0	18	59	2	87	280.12	102	434.72	351	329	319	13	35	0	2	3
4790	13120	9450	755	452	266	28096	1153	5	681	0	18	75	3	82	277.36	116	434.9	360	319	304	15	57	0	0	2
13010	7918	4858	3	1304	944	28156	609	196	874	74	112	190	86	209	284.68	189	447.92	488	311	447	38	90	14	10	3
3854	13678	9974	1005	348	190	28056	1330	9	563	1	12	59	5	66	270.96	86	423.49	278	268	249	10	45	0	0	1
5406	12534	9030	582	720	450	28186	1097	10	683	0	42	54	6	111	284.17	104	439.24	369	364	343	16	38	0	4	4
4628	13188	9504	745	470	296	28112	1154	5	633	0	24	50	4	89	277.93	100	432.5	329	329	353	21	30	0	2	1
5432	12634	9048	568	634	384	28154	1058	7	722	0	20	54	6	91	280.78	129	438.12	399	363	368	22	35	0	2	1
13752	7890	4562	6	934	750	27976	633	266	1011	122	72	227	117	164	276.82	241	440.88	476	400	449	37	89	14	16	1
4336	13390	9718	882	384	220	28072	1255	8	622	1	24	61	5	71	272.85	98	426.86	296	314	295	16	38	0	0	3
3930	13624	9894	948	380	232	28076	1298	8	545	1	14	56	4	72	271.53	79	424.59	270	272	279	19	30	0	2	2
14270	7688	4408	6	838	620	27878	644	306	1054	158	54	254	113	145	270.24	276	431.8	497	419	440	13	85	18	0	3
4138	13572	9830	936	324	182	28056	1278	6	601	1	10	61	4	62	273.55	101	427.24	301	279	281	13	47	0	0	1
4554	13178	9596	786	488	262	28100	1187	5	615	0	22	58	3	78	276.52	90	433.51	340	312	319	13	46	1	0	1
5900	12188	8780	540	810	470	28178	1078	20	720	2	30	69	12	89	282.7	116	439.39	401	388	316	28	45	0	0	4
4304	13368	9728	879	434	230	28072	1235	6	597	0	8	69	6	66	274.66	95	430.16	313	282	288	21	43	0	0	2
6584	11778	8408	467	856	516	28188	996	31	742	8	46	81	17	111	287.21	131	442.76	421	374	349	28	44	1	0	2
3990	13622	9902	946	340	190	28056	1276	11	559	1	12	52	8	73	272.94	85	426.24	294	263	290	8	35	0	0	1
5018	12912	9308	685	538	320	28124	1149	8	681	0	28	66	2	76	277.28	115	433.22	377	356	306	15	43	0	0	3
12576	7944	4978	1	1446	1112	28260	603	154	823	44	192	171	88	213	284.93	163	450.92	495	298	444	56	94	10	10	2
14498	7576	4326	4	820	598	27874	642	335	1028	160	46	267	123	136	271.99	269	435.06	498	412	428	22	91	31	8	3
12170	7840	5072	0	1768	1318	28404	570	148	667	32	208	175	77	249	298.42	142	454.47	481	230	407	68	94	10	28	15
11624	7708	5180	2	2072	1632	28600	465	113	560	28	346	194	55	279	313.54	137	462.1	522	152	374	101	94	6	36	19
5854	12380	8836	548	640	394	28138	1048	25	734	8	34	77	15	98	281.89	110	437.86	386	372	359	24	44	0	0	3
13736	7744	4538	4	1076	818	28006	667	258	932	121	92	236	95	185	275.4	194	440.37	449	383	424	32	99	19	2	0
4570	13202	9560	799	476	274	28100	1216	8	633	3	18	60	3	88	277.19	94	432.04	321	329	297	10	44	0	0	2
13634	7836	4588	3	1062	810	28016	641	261	966	113	80	221	117	167	276.26	230	440.72	501	396	432	27	91	12	6	1
11722	7872	5186	0	1896	1508	28524	554	132	608	35	302	154	71	265	305.95	112	458.43	477	220	417	80	74	9	34	22
11506	7892	5324	0	1990	1496	28522	544	105	631	22	292	167	60	264	301.19	133	454.53	495	222	393	79	89	6	20	19
13684	7670	4554	5	1138	872	28048	565	245	903	101	120	227	111	182	276.08	222	444.47	510	303	462	35	108	12	10	5
5634	12606	8994	566	532	322	28116	1084	10	749	3	28	73	3	98	281.32	101	437.65	363	401	352	22	53	0	0	2
3946	13636	9902	943	356	210	28062	1274	8	547	1	12	64	5	79	273.13	82	425.99	273	261	287	18	43	0	0	1
12542	8082	5068	0	1356	1006	28218	592	162	860	41	154	186	84	210	286.9	173	449.11	497	324	425	58	113	10	8	3
14348	7648	4396	9	822	600	27870	639	335	1047	161	52	279	134	171	272.11	252	436.78	456	428	427	20	99	24	2	3

SUPPLEMENTARY INFORMATION:Monte Carlo Atomistic Simulation and Machine Learning Analysis of Na-K Eutectic Alloy in Condensed Phases, D. Reitz and E. Blaisten-Barojas, George Mason University, Fairfax, VA 22030

14412	7750	4384	6	696	544	27840	629	354	1091	159	46	259	148	145	269.68	288	431.91	488	436	449	20	85	25	8	0
6264	12050	8556	464	780	496	28188	1005	21	735	5	42	70	13	117	286.4	127	444.9	409	380	365	22	40	0	0	3
12904	7862	4956	2	1350	946	28166	606	171	871	55	140	192	84	210	288.36	189	452.03	472	313	436	51	107	8	8	6
5262	12704	9154	620	640	366	28140	1113	9	687	1	14	72	6	102	280.19	107	437.16	376	362	323	8	48	0	0	3
13404	7858	4700	5	1128	852	28054	618	239	928	104	104	229	102	176	277.03	228	443.63	503	361	427	34	100	12	8	3
4952	12954	9320	696	542	332	28122	1171	6	678	0	20	63	6	92	278.83	85	432.93	330	374	319	19	37	0	2	2
4088	13472	9804	920	442	262	28088	1272	11	571	1	20	60	6	77	274.15	94	426.16	299	269	273	11	41	0	0	3
14514	7540	4372	8	824	556	27856	637	338	1073	159	48	274	136	159	271.44	254	433.67	479	429	428	15	102	25	2	2
4236	13444	9760	893	406	222	28076	1253	10	568	2	8	60	5	83	274.21	87	427.91	296	282	289	11	37	0	0	2
14240	7624	4360	7	940	698	27918	619	314	1006	161	54	252	117	152	270.06	257	432.33	518	406	437	16	82	19	2	1
10762	7760	5656	1	2432	1748	28776	486	70	465	12	370	159	33	317	331.69	91	471.06	459	143	359	135	73	7	46	24
12586	7892	4926	3	1518	1166	28288	570	175	766	65	182	183	82	245	290.46	174	449.68	485	283	433	54	87	8	18	6
5046	12840	9268	704	606	356	28138	1152	10	656	0	22	66	8	104	279.14	107	436.39	343	315	310	15	48	0	0	2
12912	7948	4948	2	1270	922	28142	597	201	919	78	124	209	93	198	281.43	213	444.97	509	349	430	37	105	11	16	5
5306	12652	9166	619	628	350	28130	1091	6	701	0	24	68	3	75	282.31	119	437.33	406	353	322	22	51	0	4	2
14390	7556	4386	3	872	612	27878	651	353	1009	166	60	264	141	160	271.29	244	433.72	470	406	430	18	90	25	2	2
11368	7998	5312	1	1944	1592	28598	544	91	605	13	334	139	53	255	309.93	129	459.55	510	210	413	81	68	5	44	24
11206	7894	5486	1	2072	1564	28596	531	87	590	19	328	161	46	267	313.88	95	463.28	457	193	416	102	83	6	42	29
3992	13550	9890	941	412	220	28074	1300	6	546	1	10	58	5	75	273.77	73	427.21	270	290	278	14	44	0	0	3
4822	12908	9418	713	628	338	28136	1127	5	618	0	22	53	3	90	278.73	96	434.09	352	311	349	22	31	1	0	2
4298	13388	9710	860	418	248	28084	1238	6	623	0	22	60	4	86	273.82	101	428.86	295	312	298	15	37	0	0	2
4864	12828	9366	703	692	380	28152	1146	6	622	1	22	66	3	100	278.41	83	434.17	353	320	313	16	49	0	0	2
5090	12732	9234	641	682	388	28146	1117	11	645	3	20	78	7	99	278.68	93	435.39	360	344	321	21	54	0	0	2
14412	7648	4360	8	798	592	27864	623	321	1080	169	52	265	118	144	269.29	265	432.17	502	430	437	19	84	17	2	2
4762	13094	9490	752	476	254	28090	1169	9	663	1	14	59	5	82	277.05	95	432.01	326	338	335	21	39	0	0	2
3832	13764	9994	1007	280	160	28040	1313	8	564	1	10	67	6	67	270.52	85	423.93	262	264	267	16	47	0	0	3
14262	7712	4436	5	822	596	27878	655	310	1075	161	50	265	117	160	269.13	276	432.01	475	453	426	16	89	17	0	0
4234	13488	9768	898	368	202	28064	1263	5	616	0	4	59	1	69	274.22	103	427.63	291	300	293	18	37	0	0	2
4360	13276	9672	811	504	276	28102	1191	3	606	0	14	59	2	83	276.06	98	431.45	320	302	316	17	40	0	0	6
13290	7726	4674	5	1338	980	28124	613	233	829	90	108	201	98	198	278.24	193	445.83	483	297	447	39	88	21	8	5
13774	7578	4504	3	1196	882	28046	568	268	890	111	104	233	116	183	277.08	200	444.1	505	317	460	39	106	19	8	3
13954	7772	4504	2	938	712	27958	649	276	1024	148	74	265	96	178	275.05	234	439.83	481	434	400	22	101	12	4	0
11198	7906	5464	1	2086	1578	28604	553	97	583	20	342	159	45	292	310.06	119	458.42	473	210	373	95	78	5	30	16
4698	13116	9488	747	506	290	28116	1190	3	657	0	16	53	2	91	278.28	89	434.54	333	354	320	11	36	0	2	0
13252	7672	4626	2	1382	1084	28190	586	217	812	83	150	208	101	218	284.87	194	447.27	485	291	438	46	87	13	22	6
4342	13424	9708	831	380	210	28068	1209	4	617	0	4	72	3	89	274.24	87	428.07	302	308	309	14	45	0	0	2

SUPPLEMENTARY INFORMATION:Monte Carlo Atomistic Simulation and Machine Learning Analysis of Na-K Eutectic Alloy in Condensed Phases, D. Reitz and E. Blaisten-Barojas, George Mason University, Fairfax, VA 22030

3904	13704	9952	968	310	172	28048	1303	7	567	1	4	55	4	61	269.91	85	422.51	279	281	279	14	34	0	2	3
13940	7650	4426	1	1046	832	28012	625	255	964	105	112	246	115	175	277.04	233	445.04	483	370	422	33	123	16	6	5
4052	13574	9840	930	366	222	28070	1266	5	596	0	16	54	4	87	273.16	89	425.38	280	277	297	8	37	0	0	3
13818	7590	4420	2	1138	944	28076	625	258	894	113	146	231	97	223	277.86	183	446.5	452	346	423	31	102	21	20	1
5692	12436	8904	517	700	416	28172	1023	13	733	0	24	57	9	101	280.35	134	437.61	415	369	372	23	27	0	0	0
14202	7734	4438	3	848	626	27902	644	314	1041	153	54	249	124	146	269.57	266	432.25	494	429	438	22	89	16	0	1
11950	8078	5218	2	1682	1244	28384	598	125	728	21	200	142	66	238	297.53	156	453.54	476	245	420	70	77	7	12	12
4284	13414	9726	841	408	232	28076	1215	7	603	0	12	50	5	73	275.95	86	430.66	312	308	321	20	30	0	0	1
12364	7868	5032	5	1640	1214	28326	573	170	740	53	194	180	80	245	295.1	168	453.01	490	267	420	58	93	11	14	8
6168	12126	8634	500	730	462	28164	1067	18	770	3	42	68	9	101	284.26	119	440.57	394	423	337	21	46	0	0	2
4526	13164	9562	783	536	310	28118	1234	2	617	0	16	58	2	77	278.06	87	435.8	322	328	285	17	46	0	4	1
3932	13672	9950	956	316	168	28048	1286	7	563	1	10	59	4	69	271.1	79	423.58	280	274	284	14	37	0	0	2
12002	7776	5168	1	1874	1372	28468	544	131	636	43	250	195	69	283	305.45	129	456.49	467	220	403	72	106	3	24	15
13672	7656	4538	3	1172	898	28066	633	271	893	118	120	231	113	198	276.56	211	443.26	464	330	421	38	93	24	10	3
6430	11956	8496	466	748	486	28182	979	27	747	5	66	78	16	100	286.52	133	442.15	442	377	363	23	49	0	0	5
14418	7578	4366	5	826	612	27880	625	339	1039	153	80	256	136	139	273.22	270	436.31	520	402	436	17	90	23	0	0
6016	12162	8726	499	776	456	28166	1003	18	743	7	30	74	5	103	281.72	104	439.59	402	379	378	27	47	1	0	6
4384	13354	9686	856	400	228	28072	1234	7	635	0	20	57	3	61	273.92	100	427.32	315	307	306	18	38	0	0	4
11420	7914	5396	3	1982	1490	28538	521	106	612	22	310	171	52	264	316.71	123	462.63	462	206	409	106	81	8	24	21
4442	13204	9586	814	542	328	28130	1192	4	593	0	28	61	1	90	276	108	432.89	333	268	296	14	41	0	0	5
4010	13586	9890	925	364	198	28058	1274	8	567	1	10	57	4	72	271.79	80	424.17	289	287	286	13	38	0	0	2
11358	7980	5384	0	2010	1524	28582	521	98	599	14	292	161	55	284	314.15	124	460.92	486	190	396	95	87	8	32	16
4644	13144	9496	764	498	306	28114	1177	7	633	1	24	54	2	92	275.68	84	430.77	326	325	328	17	39	0	0	1
13854	7776	4640	10	948	654	27946	617	286	1024	126	74	244	125	182	276.26	230	439.72	469	413	451	25	108	14	0	2
13216	7770	4696	2	1314	1008	28158	634	200	837	78	144	202	90	180	282.85	192	447.52	492	314	413	55	95	15	10	5
12672	8118	4972	5	1250	996	28178	621	167	911	61	158	186	79	183	281.71	190	445.6	506	356	442	43	97	11	10	6
4856	12934	9336	730	600	382	28144	1150	11	619	1	34	69	7	96	279.07	84	437.51	347	314	311	18	46	0	2	4
5162	12774	9198	674	598	368	28132	1141	14	665	2	32	60	8	89	279.75	101	436.49	341	355	333	28	34	0	0	1
4352	13320	9658	825	468	282	28102	1223	7	610	0	20	70	4	87	276.02	89	431.86	311	311	287	16	51	0	2	2
5174	12744	9204	657	628	366	28142	1115	8	657	1	26	66	4	102	281.6	91	437.56	356	349	332	21	41	0	0	1
14324	7692	4408	8	790	588	27856	627	322	1090	162	48	281	123	146	270.52	284	434.61	487	430	427	25	104	17	6	2
5822	12216	8792	530	804	506	28200	1071	13	711	6	60	74	6	109	286	123	439.86	389	376	328	25	52	0	0	1
4202	13428	9706	875	456	294	28106	1262	5	578	1	20	60	2	81	273.86	81	427.86	298	296	276	15	34	1	0	3
4136	13548	9808	939	350	206	28058	1306	7	594	1	10	61	6	62	273.5	91	426.6	289	293	263	11	46	0	0	3
4864	13014	9380	736	536	312	28118	1143	4	650	0	12	56	3	67	277.85	115	433.46	375	312	335	19	38	0	0	2
4280	13416	9704	854	404	256	28086	1242	5	607	0	26	60	5	90	274.39	76	429.04	290	312	296	14	35	0	0	2

11654	7946	5298	0	1896	1424	28518	560	117	623	25	274	163	61	277	307.84	136	457.53	467	196	401	83	91	10	26	16
4726	13108	9478	760	484	286	28108	1186	4	674	0	22	65	3	75	277.22	98	432.83	346	341	301	17	47	0	4	2
13798	7668	4548	5	1056	812	28012	608	273	954	133	116	244	101	201	277.33	235	444.46	471	355	425	33	90	16	14	1
13006	8086	4816	6	1144	934	28134	612	187	943	65	148	171	93	185	281.79	218	444.63	522	358	454	32	86	9	0	1
14512	7580	4294	3	838	606	27868	652	341	1044	157	34	263	143	143	270.67	276	433.09	492	427	430	14	89	22	4	2
5400	12688	9112	613	572	332	28126	1108	11	726	2	20	54	5	94	281.64	105	437.76	367	390	348	18	32	1	0	1
12108	7946	5250	7	1648	1174	28358	578	139	739	24	214	150	94	239	296.94	131	452.69	456	264	471	72	95	6	18	11
4224	13510	9778	889	338	194	28056	1252	8	606	1	12	61	5	73	271.06	94	424.48	293	296	298	13	43	0	0	4
4594	13118	9562	776	546	280	28106	1174	5	622	0	6	60	3	84	278.6	92	435.4	346	302	317	12	39	0	0	2
4832	13060	9416	725	486	290	28108	1149	6	667	0	24	71	3	89	278.51	110	433.24	358	329	313	11	51	0	0	3
7814	10796	7524	309	1188	860	28302	867	45	722	16	110	101	24	164	288.24	142	446.7	446	332	370	37	56	1	6	5
13558	7772	4604	6	1118	868	28042	632	263	907	120	114	224	103	211	276.43	196	443.66	438	370	447	31	86	19	8	4
4270	13328	9710	863	490	284	28106	1254	5	611	1	20	58	3	92	275.58	93	429.79	289	305	283	14	41	0	4	3
4468	13278	9626	817	440	256	28092	1205	10	623	2	24	66	6	58	276.17	106	430.63	342	299	297	23	45	0	0	3
14138	7716	4448	12	862	662	27904	645	321	1034	166	76	259	115	169	271.59	237	435.69	460	417	437	24	81	22	2	1
5848	12134	8784	503	882	522	28220	1013	17	684	4	50	68	11	110	286.29	115	440.08	439	343	343	19	40	0	0	1
13542	7812	4660	3	1094	820	28038	638	244	953	100	102	221	99	189	276.55	217	444.32	465	378	429	33	105	20	8	6
4176	13442	9784	892	426	238	28082	1249	6	584	0	16	59	5	73	273.56	94	427.44	302	276	296	15	41	0	0	3
3860	13710	9968	975	320	184	28056	1311	3	571	1	14	63	2	65	270.09	81	424.03	264	280	269	20	47	0	0	3
4746	13136	9458	779	448	282	28100	1212	8	653	0	30	69	5	82	276.29	99	431.56	330	339	283	13	50	0	0	3
13684	7800	4574	9	1070	800	28004	663	268	940	118	66	219	120	176	275.05	217	440.55	463	386	437	28	86	13	10	2
4630	13160	9538	778	478	272	28100	1210	12	645	1	22	58	6	73	275.37	101	430.61	333	340	299	16	41	0	0	3
13860	7746	4514	5	1002	770	27984	616	278	964	121	80	235	123	185	276.79	225	441.89	451	377	464	36	92	18	12	4
13668	7876	4604	10	982	768	27994	621	274	989	120	90	242	118	173	277.2	234	443.24	476	370	440	40	104	18	6	0
3932	13662	9956	975	324	164	28044	1301	6	562	1	6	70	4	75	272.31	75	425.14	269	275	266	10	47	0	0	4
5904	12206	8772	486	770	478	28184	1024	10	778	0	50	69	6	102	283.93	134	441.57	399	395	365	31	47	0	4	3
6826	11486	8158	354	1020	692	28276	959	17	729	1	86	72	10	136	287.2	123	444.23	433	388	353	29	29	1	6	5
13536	7682	4624	4	1208	886	28052	593	239	904	120	114	233	88	188	276.88	220	442.94	516	332	413	32	88	14	2	2
12732	7750	4790	0	1548	1240	28300	590	165	751	43	236	184	84	221	292.39	156	449.38	505	286	413	60	108	9	4	4
3766	13732	10006	1002	350	196	28058	1322	8	547	0	8	56	3	70	270.55	83	423.22	265	262	264	14	39	0	0	2
11166	7992	5442	0	2040	1600	28622	541	69	618	18	340	172	37	296	316.18	98	461.44	441	206	395	104	90	1	38	17
13104	7790	4712	4	1354	1050	28168	558	220	820	74	150	201	107	224	281.55	179	446.39	468	293	470	58	87	9	8	2
10802	7698	5658	1	2390	1768	28788	513	67	493	10	394	154	27	277	329.39	95	470.93	488	172	341	123	77	2	68	37
4302	13360	9710	858	460	250	28088	1210	7	600	1	6	57	5	63	272.6	110	427.93	342	274	305	18	38	0	0	0
13920	7848	4570	5	860	652	27926	648	285	1049	140	72	240	106	169	275.48	250	438.55	473	426	439	19	85	17	4	2
5516	12564	9054	589	616	354	28138	1089	10	716	0	32	62	6	101	282.28	105	437.74	362	379	357	19	45	0	2	4

SUPPLEMENTARY INFORMATION:Monte Carlo Atomistic Simulation and Machine Learning Analysis of Na-K Eutectic Alloy in Condensed Phases, D. Reitz and E. Blaisten-Barojas, George Mason University, Fairfax, VA 22030

10870	7890	5572	2	2240	1726	28748	501	74	538	10	400	154	43	247	323.69	96	464.46	480	171	395	141	81	5	48	31
14298	7656	4430	8	848	596	27876	650	312	1060	159	46	265	118	148	270.61	254	434.57	479	428	424	25	96	15	2	1
5240	12586	9158	622	730	420	28174	1126	6	691	1	34	65	4	106	281.99	105	440.2	362	364	314	15	49	0	6	4
14006	7708	4476	2	940	724	27948	644	312	969	149	92	245	123	147	273.57	253	438.49	511	388	407	30	82	18	2	0
13874	7696	4502	3	1042	782	27986	627	285	987	147	88	244	113	159	274.63	234	437.97	521	414	418	22	85	9	2	2
13514	7918	4728	8	1008	744	28006	636	258	966	118	88	224	112	183	278.27	216	442.16	460	375	450	33	85	12	6	3
10704	7830	5714	1	2356	1718	28762	497	68	500	4	390	149	34	315	332.56	82	469.75	473	152	363	118	79	7	50	27
13670	7764	4628	8	1034	780	27988	613	267	979	128	104	239	115	180	274.89	245	438.48	484	395	436	28	92	10	8	5
14300	7644	4428	6	862	598	27876	637	325	1065	169	44	261	116	158	267.28	262	429.54	499	448	423	11	82	23	0	1
10538	7808	5730	1	2468	1824	28840	505	60	449	10	418	175	23	314	333.74	74	471.1	492	151	309	118	78	6	50	33
11048	7618	5568	1	2284	1728	28760	473	73	488	15	468	179	35	306	329.39	97	470.87	470	147	346	125	102	2	40	37
3700	13768	10034	1029	334	202	28058	1317	4	522	2	20	73	2	75	270.8	79	422.92	261	238	253	17	40	0	0	1
4182	13494	9786	903	372	218	28070	1261	6	595	2	18	51	1	63	272.71	92	424.93	299	282	298	18	32	0	0	3
6170	11952	8616	468	890	534	28216	1022	16	719	3	48	79	10	107	286.01	114	442.28	424	382	334	24	43	0	6	4
6350	11982	8536	459	784	480	28170	1000	21	761	7	36	84	9	118	285.34	107	443.78	396	385	361	26	55	0	2	5
4856	13038	9426	722	494	270	28096	1151	3	694	0	12	74	2	76	277.75	106	434.16	356	343	316	21	51	0	0	1
11988	7844	5158	1	1826	1346	28412	555	126	693	19	234	173	71	248	300.99	152	454.6	479	242	409	79	90	15	16	16
13698	7646	4506	4	1168	910	28060	628	274	847	107	124	225	133	179	279.2	206	444.48	482	327	431	42	85	19	8	2
4350	13452	9722	893	326	188	28052	1267	6	638	1	12	59	3	73	271.94	99	425.36	288	312	292	14	38	0	0	1
11010	8044	5478	0	2086	1646	28652	536	76	581	13	350	164	37	276	314.19	113	460.8	509	198	363	97	86	4	36	18
4824	13028	9420	711	534	298	28116	1149	7	656	0	12	51	5	80	276.74	104	433.69	359	330	338	13	33	0	0	4
4904	13062	9360	739	464	294	28104	1166	9	672	2	18	72	6	85	275.43	106	429.81	339	328	313	20	51	0	2	1
14216	7544	4412	4	984	702	27938	653	302	997	149	78	245	108	157	270.49	216	436.27	484	414	424	24	85	23	2	3
13172	7616	4702	2	1452	1080	28202	571	226	759	90	176	214	98	225	284.87	161	447.81	505	274	420	45	86	16	4	3
4062	13584	9886	926	338	172	28048	1276	4	598	0	6	51	2	69	272.36	98	424.56	284	293	295	13	37	0	0	3
5332	12706	9134	631	606	346	28140	1125	9	729	2	16	58	3	99	279.8	120	438.46	372	381	325	10	38	0	0	2
4192	13498	9784	908	370	210	28064	1236	8	596	1	10	56	4	73	272.37	92	425.31	308	270	303	15	39	0	0	1
13986	7836	4532	6	842	652	27924	637	288	1058	156	64	262	102	172	274.41	253	438.04	472	427	423	25	90	11	12	1
4866	12990	9366	688	552	330	28122	1144	5	642	0	18	60	2	85	278.68	84	435.43	342	362	332	23	40	0	0	5
5626	12384	8948	552	726	438	28176	1049	14	682	4	52	65	5	110	284.19	102	440.74	377	351	365	27	38	1	2	2
12974	7956	4864	2	1266	946	28140	649	192	892	78	128	200	76	196	282.44	193	446.8	501	357	399	32	90	18	6	2
5080	12884	9248	664	562	338	28126	1113	6	671	0	14	56	3	90	278.3	98	436.02	366	347	349	15	39	0	0	3
12496	8018	4998	1	1422	1126	28278	638	143	822	46	206	179	79	221	293.31	169	449.39	480	327	401	52	102	10	12	7
11610	7958	5350	1	1888	1404	28510	540	115	628	20	270	152	57	268	304.12	126	458.99	498	198	410	82	85	6	26	14
4970	13044	9384	719	432	242	28086	1141	5	705	1	10	78	4	81	276.85	115	432.27	361	345	322	13	57	0	4	2
4300	13502	9752	886	310	174	28046	1274	7	630	2	8	63	4	68	272.05	79	425.9	290	326	285	14	43	0	0	1

SUPPLEMENTARY INFORMATION:Monte Carlo Atomistic Simulation and Machine Learning Analysis of Na-K Eutectic Alloy in Condensed Phases, D. Reitz and E. Blaisten-Barojas, George Mason University, Fairfax, VA 22030

14010	7664	4488	4	988	720	27946	629	279	999	152	70	262	94	177	274.62	222	438.75	480	419	421	24	94	10	6	1
4724	13166	9490	788	426	256	28084	1210	9	673	0	20	60	6	76	274.65	104	429.35	326	338	305	11	41	0	2	5
4206	13470	9762	890	388	230	28072	1243	9	593	2	16	58	5	61	275.79	103	429.2	320	290	293	12	32	0	0	6
14090	7646	4446	7	954	716	27940	651	321	983	153	88	242	126	149	271.29	238	434.25	486	411	437	28	76	21	0	2
6238	11940	8586	416	886	520	28210	983	16	759	2	36	56	10	123	285.06	119	442.66	428	399	382	18	34	0	4	1
4552	13272	9590	807	424	232	28072	1219	7	643	1	2	56	4	71	275.07	81	430.8	319	326	309	15	42	0	0	3
11030	7858	5578	1	2190	1612	28666	517	85	539	12	364	153	56	284	311.91	95	460.66	503	175	395	103	84	5	32	16
14256	7704	4412	9	828	626	27890	630	322	1041	155	62	267	130	162	272.86	263	437.35	480	406	432	22	93	18	2	1
4224	13432	9732	878	428	256	28086	1238	7	577	2	14	58	1	68	274.47	94	429.78	318	284	290	17	35	0	0	3
5442	12676	9064	638	552	354	28128	1117	14	702	2	40	57	7	98	280.75	108	435.61	366	362	338	15	35	0	0	0
4714	13198	9526	746	404	222	28078	1187	5	687	0	14	61	3	75	277.19	98	430.26	330	364	325	15	35	0	0	3
10918	7944	5538	0	2198	1706	28736	541	69	544	12	384	148	37	285	319.38	101	461.28	506	208	354	104	62	6	46	23
4946	12838	9304	701	646	386	28152	1169	8	655	0	30	55	6	84	279.28	87	435.38	346	353	317	23	37	0	2	2
3814	13730	10002	993	322	172	28046	1305	7	577	1	6	59	4	64	270.26	96	421.69	278	259	271	14	38	0	0	2
4580	13062	9510	755	608	356	28146	1205	4	617	0	30	70	3	84	278.85	98	433.82	332	336	280	22	43	0	0	3
13810	7832	4514	6	976	770	27986	619	274	970	140	80	233	106	179	274.15	209	438.16	485	400	452	25	76	11	4	1
14458	7674	4334	2	742	584	27860	659	324	1088	153	68	265	128	149	272.67	267	436.59	472	453	427	22	95	20	0	0
5590	12536	9028	617	600	334	28112	1081	20	713	4	24	70	11	120	282.45	115	438.97	358	349	353	9	50	0	0	1
3936	13712	9952	985	278	150	28034	1314	9	580	1	6	67	5	62	270.99	91	423.33	269	283	264	15	46	0	0	3
4422	13326	9648	816	412	250	28086	1217	7	617	1	28	51	5	76	276.7	86	432.17	314	320	310	19	37	0	0	2
5086	12808	9200	668	636	400	28152	1121	8	655	1	16	59	7	98	280.05	98	438.26	361	335	331	19	38	0	6	3
4468	13348	9608	818	400	248	28086	1204	8	637	1	14	62	4	79	275.47	117	430.32	329	309	308	13	37	0	0	1
5544	12586	9048	588	586	336	28130	1104	4	755	0	28	77	4	99	280.04	96	436.88	365	403	329	20	49	0	2	0
11578	7894	5348	0	1930	1448	28526	519	98	615	17	296	172	53	286	309.9	125	460.33	479	199	403	87	98	4	32	17
6274	11980	8598	477	802	476	28178	1018	19	761	6	48	88	9	103	286.29	117	443.16	415	399	344	23	59	0	0	5
12304	8108	5046	4	1500	1158	28292	630	147	797	55	158	183	63	195	287.36	181	448.82	527	301	387	45	96	13	18	11
12750	8012	4966	8	1294	988	28178	649	195	897	57	154	160	103	171	281.03	197	447.8	501	360	454	39	80	14	12	10
14258	7678	4412	0	836	634	27892	633	327	1047	158	74	264	137	157	269.83	262	432.54	495	434	431	17	97	19	0	0
7380	11158	7868	377	1040	708	28252	926	38	757	11	94	90	23	123	287.85	132	446.45	437	357	364	37	57	1	4	8
12560	7838	4948	6	1568	1176	28290	605	173	793	57	184	185	89	223	291.04	152	451.4	473	308	419	64	100	4	16	9
4252	13420	9734	863	422	242	28082	1210	5	584	0	12	65	3	95	274.35	82	430.78	307	279	303	12	47	1	0	1
4290	13480	9744	880	330	196	28058	1248	6	631	1	18	64	3	71	273.02	90	426.56	300	317	296	9	45	0	0	7
4072	13644	9854	936	290	178	28050	1293	5	611	1	12	62	4	75	272.18	91	425.73	281	302	278	9	45	0	0	0
14212	7698	4496	9	848	576	27870	645	304	1090	159	38	263	115	150	271.7	259	433.9	479	446	436	20	94	13	0	2
12294	8052	5090	2	1524	1152	28306	604	147	783	41	178	159	62	221	292.92	162	451.63	489	282	428	56	83	14	14	12
10586	7766	5714	0	2434	1832	28832	506	53	476	9	430	173	23	314	333.56	91	471.95	475	140	338	122	83	5	64	30

SUPPLEMENTARY INFORMATION:Monte Carlo Atomistic Simulation and Machine Learning Analysis of Na-K Eutectic Alloy in Condensed Phases, D. Reitz and E. Blaisten-Barojas, George Mason University, Fairfax, VA 22030

4572	13126	9546	791	550	308	28120	1229	4	594	0	16	57	3	87	276.47	82	431.89	313	313	293	16	37	0	2	1
12596	7884	4950	4	1502	1146	28286	597	172	768	49	192	202	87	232	293.25	148	452.36	475	282	402	54	106	12	16	14
5430	12646	9084	629	580	352	28128	1108	18	689	5	34	65	7	85	284.62	108	441.14	382	371	330	20	43	0	2	1
4506	13168	9580	804	542	304	28118	1218	4	603	0	18	54	2	80	275.24	91	430.45	322	304	303	17	42	1	0	2
4302	13474	9736	865	342	198	28064	1250	4	621	0	10	52	3	74	273.25	96	427.34	293	317	311	14	39	0	2	2
13632	7826	4584	6	1050	820	28008	624	272	952	135	80	232	105	184	274.18	226	438.23	480	377	433	32	79	9	14	1
12498	8004	5062	7	1416	1058	28220	578	164	830	42	166	196	96	229	286.83	185	450.36	461	283	456	56	106	7	16	8
5170	12798	9234	696	556	328	28118	1142	11	696	0	30	65	3	86	277.77	111	436.46	368	345	312	11	49	2	2	5
11984	7870	5104	2	1772	1410	28448	547	135	674	35	276	184	72	247	299.87	153	454.31	502	231	396	82	91	8	30	14
4098	13546	9844	926	368	198	28060	1267	7	596	1	6	65	6	68	273.5	92	424.75	289	283	288	18	42	0	0	2
4602	13112	9550	792	538	292	28114	1194	5	619	0	20	64	3	82	277.64	94	432.37	334	312	298	20	41	0	0	1
14464	7720	4386	7	700	518	27830	627	349	1096	166	36	266	138	155	268.01	279	429.91	487	447	443	10	88	20	6	2
10530	7740	5714	0	2498	1878	28866	476	76	451	10	428	167	47	324	329.42	91	470.42	488	119	347	107	87	2	74	37
7078	11324	8064	361	1034	670	28258	930	24	757	1	82	75	16	117	287.46	119	444.24	467	382	368	27	48	2	6	6
12764	7770	4912	4	1480	1116	28260	600	173	810	48	198	197	82	210	290.04	175	452.34	497	293	396	56	108	16	20	14
13768	7648	4474	2	1126	906	28072	572	254	891	79	142	200	133	201	281.92	199	446.24	479	345	486	38	89	13	8	5
14484	7642	4292	5	770	610	27862	648	330	1082	170	64	254	125	147	268	276	428.85	480	445	445	20	72	15	0	0
13716	7680	4502	2	1124	900	28062	567	247	912	101	128	230	109	192	278.71	223	445.02	495	325	465	40	102	19	8	2
4260	13388	9732	889	448	250	28090	1277	10	604	0	10	52	7	75	274.75	85	429.02	293	296	282	16	39	0	2	0
4740	13128	9496	725	476	252	28098	1173	6	688	0	6	68	4	90	276.82	94	432.9	329	362	315	18	44	0	0	1
6812	11548	8216	386	962	626	28240	953	20	750	3	68	87	10	136	289.63	129	444.74	432	384	349	28	53	1	8	3
5474	12482	9028	579	720	424	28168	1053	8	690	2	38	73	2	99	285.15	122	441.7	405	343	342	18	46	1	2	3
12188	7826	5082	2	1766	1300	28392	576	136	696	39	204	172	65	248	293.38	140	453.57	487	247	414	63	86	15	26	12
13842	7774	4598	5	948	706	27970	616	280	987	118	98	226	125	171	276.29	241	441.91	488	380	451	29	89	18	4	2
14220	7786	4420	4	786	614	27882	641	311	1058	152	52	248	122	161	270.62	263	432.76	471	423	456	16	84	20	4	2
12326	7664	4962	1	1838	1374	28418	564	150	653	46	232	188	79	253	297.46	130	454.14	460	229	413	79	94	9	18	18
13438	7618	4592	5	1288	1008	28118	584	236	848	88	166	223	109	200	284.36	181	447.39	467	308	448	55	103	16	6	7
14460	7708	4352	5	712	556	27846	635	328	1116	161	54	267	126	142	268.05	284	429.79	489	450	438	21	100	19	4	1
4828	13096	9440	717	460	260	28098	1154	6	687	0	14	65	3	84	276.29	100	432.86	353	345	323	12	46	0	0	3
3826	13740	9984	1003	312	180	28052	1317	7	554	1	8	58	4	68	270.95	85	423.27	271	267	267	12	35	0	2	2
4246	13446	9750	895	398	224	28074	1287	8	606	2	10	54	3	57	272.84	100	424.84	295	312	283	19	42	0	0	2
4106	13622	9870	936	290	148	28036	1290	5	625	1	0	67	2	64	270.85	100	422.93	283	309	278	14	45	0	0	1
12444	7836	5010	1	1606	1206	28330	601	153	750	34	208	153	85	230	294.18	152	452.34	488	296	435	58	76	11	20	8
3876	13664	9966	998	354	186	28052	1328	11	557	1	6	63	6	71	270.72	94	422.23	272	272	245	12	42	0	0	0
5808	12256	8792	550	782	498	28186	1061	23	697	4	46	76	15	116	285.96	109	442.32	386	357	329	22	43	1	4	2
4028	13560	9864	954	394	218	28072	1310	7	583	0	8	67	4	68	271	92	423.37	282	291	254	13	45	0	0	3

SUPPLEMENTARY INFORMATION:Monte Carlo Atomistic Simulation and Machine Learning Analysis of Na-K Eutectic Alloy in Condensed Phases, D. Reitz and E. Blaisten-Barojas, George Mason University, Fairfax, VA 22030

4620	13322	9594	843	318	178	28044	1206	9	650	1	12	57	6	86	274.25	104	429.5	300	314	331	8	36	0	0	3
6112	11994	8644	458	892	524	28206	983	18	723	4	40	71	9	125	284.84	112	440.85	430	358	362	15	44	0	0	4
14386	7684	4398	7	774	564	27856	638	328	1063	156	50	246	129	140	272.37	272	437	489	432	454	20	77	22	0	2
14382	7668	4404	2	760	576	27868	629	338	1080	160	76	262	134	150	270.62	257	432.87	490	438	444	19	90	22	2	1
4410	13214	9628	805	528	310	28124	1198	1	599	1	34	63	0	102	277.96	84	432.11	311	292	308	15	42	0	0	1
4872	12944	9414	713	560	304	28120	1131	6	663	0	26	73	5	88	279.92	116	436.22	369	320	314	15	55	0	0	3
5320	12636	9098	580	648	410	28160	1036	9	686	1	46	56	6	92	279.5	106	434.87	406	337	373	23	39	0	2	4
11814	7912	5198	1	1854	1414	28480	574	106	665	24	268	167	52	241	302.1	123	458	487	230	398	87	96	8	18	15
4026	13536	9868	946	408	224	28074	1292	6	574	1	10	64	2	82	271.47	91	423.84	278	274	266	11	42	0	2	2
14242	7690	4432	5	830	624	27890	616	326	1040	161	68	269	128	155	270.87	271	433.54	511	404	428	14	98	16	4	2
4108	13490	9828	914	400	228	28078	1244	5	584	1	24	50	3	68	274.39	93	428.21	314	273	304	13	33	0	0	2
4880	13082	9410	720	452	256	28088	1154	7	702	1	8	67	4	78	278.58	104	432.71	339	347	338	18	48	0	0	3
14460	7538	4266	2	890	678	27900	644	336	1015	158	68	259	128	150	271.38	253	436	478	426	436	27	92	27	0	2
5116	12666	9204	615	734	426	28180	1105	5	658	0	34	56	4	107	280.44	94	438.03	373	351	333	17	33	0	0	2
5282	12576	9084	585	754	460	28194	1084	8	664	1	38	59	6	98	284.05	107	440.3	401	355	330	18	35	0	0	3
4432	13290	9638	833	462	258	28090	1233	6	619	1	10	54	3	80	272.04	90	425.82	311	311	304	13	28	0	0	1
4274	13458	9744	882	364	212	28068	1239	3	627	0	14	64	0	77	275.64	99	430.58	305	286	297	9	46	0	2	5
4150	13588	9848	927	290	156	28042	1260	4	594	1	8	61	2	67	272.69	99	426.4	284	279	301	19	42	0	0	2
3728	13782	10032	1018	314	182	28050	1330	4	554	1	12	62	3	61	271.2	92	424.38	265	259	261	15	38	0	0	3
4284	13358	9714	858	458	258	28086	1245	10	600	1	14	53	6	76	272.33	85	427.22	304	299	296	15	35	0	0	3
14384	7518	4264	7	944	736	27930	644	329	980	164	76	264	122	155	272.12	258	435.55	505	403	400	22	84	19	8	1
5706	12396	8896	542	702	428	28168	1075	10	742	1	40	54	6	95	281.24	112	438.18	394	397	355	20	37	0	0	0
4588	13090	9556	783	550	304	28118	1181	8	606	1	28	64	4	75	280.84	95	435.26	353	305	301	18	48	0	2	2
4390	13458	9700	861	316	180	28050	1249	5	649	1	4	65	2	77	274.55	88	428.67	302	320	289	9	48	0	2	2
6836	11620	8244	380	868	564	28204	953	30	800	5	68	84	18	128	285.36	128	442.12	435	406	353	22	53	0	4	5
14406	7690	4358	4	764	588	27864	654	328	1087	160	52	256	129	156	267.39	270	429.71	478	455	432	14	87	18	6	1
14410	7668	4316	8	806	624	27878	637	323	1062	156	54	250	116	153	271.89	258	435.42	475	421	448	26	82	19	0	0
11142	7724	5364	0	2260	1794	28752	500	83	509	19	412	175	48	299	322.61	100	468.02	496	155	355	113	84	3	56	23
4172	13538	9822	912	328	180	28054	1272	7	607	1	14	60	5	66	274.7	79	426.47	293	297	286	15	38	0	0	2
14262	7670	4438	7	818	620	27890	612	313	1079	154	66	261	122	164	273.2	253	436.61	467	409	457	28	99	15	16	2
4472	13390	9632	842	346	210	28060	1221	6	636	1	10	59	3	71	275.21	103	428.86	311	307	314	15	43	0	0	3
12164	7706	5028	1	1810	1420	28446	564	131	645	26	286	187	78	253	305.78	123	456.47	440	218	393	108	108	10	30	14
4602	13274	9584	801	384	210	28062	1231	7	686	0	8	55	3	68	276.05	109	431.59	324	357	302	11	38	0	0	0
4500	13198	9594	805	522	282	28102	1228	8	612	4	6	66	4	78	275.93	89	431.02	315	318	290	17	43	0	0	3
14208	7766	4444	4	790	610	27884	651	316	1073	161	62	267	127	144	269.58	251	431.75	479	462	426	26	93	14	4	3
5094	12856	9302	706	520	300	28110	1132	6	692	1	36	71	3	94	283.01	115	438.74	354	337	327	16	49	0	2	1

SUPPLEMENTARY INFORMATION:Monte Carlo Atomistic Simulation and Machine Learning Analysis of Na-K Eutectic Alloy in Condensed Phases, D. Reitz and E. Blaisten-Barojas, George Mason University, Fairfax, VA 22030

6446	11852	8448	455	848	546	28206	994	19	762	2	66	74	12	132	285.21	113	444	404	381	361	23	49	0	0	2
4678	13078	9478	724	554	320	28128	1178	1	634	0	20	63	1	79	277.41	94	433.66	348	332	310	16	38	0	0	4
5734	12420	8904	565	656	394	28142	1048	17	711	5	34	73	11	97	281.94	112	438.22	401	363	354	19	49	0	0	2
3944	13656	9928	964	332	184	28054	1307	6	585	2	10	66	3	67	270.35	87	422.82	276	287	267	11	46	0	0	4
11724	7812	5280	1	1962	1440	28526	518	91	621	26	288	187	40	268	307.07	137	459.39	506	203	384	86	103	7	16	19
14174	7672	4452	8	864	656	27906	623	332	1018	152	82	253	130	170	273.13	244	438.04	481	403	447	17	89	24	6	2
5252	12818	9186	649	532	314	28118	1107	5	721	0	14	61	3	77	278.8	121	436.74	376	343	350	20	45	0	2	4
4876	13000	9370	711	524	320	28116	1160	9	670	1	26	69	6	85	280.03	118	435	350	336	308	21	43	0	0	0
3920	13668	9952	957	332	174	28052	1295	7	575	1	4	66	6	62	271.22	87	424.41	289	283	271	12	42	0	2	3
5128	12902	9268	660	496	292	28106	1126	7	693	1	18	64	4	99	277.9	94	433.25	357	368	334	11	42	0	0	1
7636	10956	7736	335	1098	728	28248	909	56	727	11	88	76	28	144	289.3	125	445.75	435	344	378	35	47	3	6	3
11206	8022	5400	1	2044	1594	28618	563	93	572	16	314	146	54	268	313.36	113	457.65	468	212	400	95	70	4	36	26
7810	10798	7648	308	1124	762	28270	893	40	755	9	116	89	20	149	289.69	118	448.25	426	367	376	42	54	1	12	7
5716	12392	8892	548	694	426	28160	1060	12	746	1	36	67	7	105	282.78	115	439.01	390	383	348	21	43	0	4	1
11122	7936	5514	2	2130	1576	28634	537	73	550	16	322	175	35	271	316.84	100	463.84	487	190	367	105	92	6	32	23
4450	13412	9676	839	326	180	28052	1250	7	646	1	8	53	2	72	274.57	93	428.71	306	330	299	10	30	0	0	1
14354	7658	4368	5	836	618	27886	594	330	1023	150	48	243	132	137	271.25	285	434.8	528	387	465	20	82	23	4	2
14038	7778	4498	3	866	668	27932	637	295	1035	137	80	252	114	177	276.13	246	440.27	445	406	442	32	100	27	4	4
4368	13318	9662	835	466	268	28096	1239	3	623	0	12	59	1	89	275.92	91	430.75	292	312	296	18	39	0	2	1
4464	13326	9640	840	402	226	28068	1237	10	620	2	10	58	4	78	276.38	89	429.76	305	323	300	9	36	0	0	5
5670	12400	8912	554	728	430	28168	1064	14	701	1	28	66	10	105	284.2	120	440.74	401	363	338	13	42	0	0	4
4522	13248	9602	803	434	256	28092	1199	8	631	1	30	71	5	88	275.22	101	428.7	320	314	300	15	45	0	0	2
13590	7764	4676	7	1100	782	28004	611	275	968	140	88	238	99	172	275.49	232	443.45	513	380	419	27	81	16	4	3
10752	7776	5624	1	2348	1802	28804	491	71	467	9	454	163	34	305	332.67	87	471.25	492	128	345	125	89	3	42	26
4984	12838	9278	681	650	382	28150	1148	5	638	1	18	47	3	89	277.08	98	434.94	359	337	337	16	24	0	0	0
4304	13338	9696	877	464	272	28098	1266	12	584	0	24	56	7	73	274.26	78	428.23	308	301	272	16	38	0	0	1
12894	7904	4860	2	1344	1020	28180	606	199	853	74	144	197	101	193	281.55	200	447.27	489	318	448	51	91	10	14	5
4590	13126	9526	756	544	316	28124	1168	6	609	0	20	67	4	83	276.28	97	433.29	341	300	311	22	42	0	2	3
4362	13368	9678	836	402	248	28088	1214	8	623	1	30	60	2	63	274.99	116	428.61	344	296	298	13	46	0	0	3
5586	12572	9028	602	566	330	28114	1098	12	735	0	30	70	11	100	279.19	115	436.58	366	381	346	12	49	0	2	1
4860	13042	9370	712	502	318	28118	1134	6	644	0	26	65	2	79	279.29	115	437.17	371	308	319	18	46	1	0	4
11470	7976	5380	2	1922	1462	28542	557	99	640	20	306	152	45	268	307.15	116	457.88	492	224	398	85	78	10	22	13
13998	7732	4456	5	970	734	27960	626	295	952	130	68	236	119	177	275.69	228	438.24	478	360	451	21	88	26	2	3
11430	7848	5404	0	2012	1526	28594	504	86	645	19	320	169	38	283	313.63	132	465.68	514	201	374	82	104	4	52	25
14382	7528	4326	6	898	684	27906	646	320	1007	163	76	258	118	167	272	228	436.29	464	433	436	21	82	22	12	2
4934	13010	9366	713	496	286	28106	1139	9	703	0	14	57	5	73	278.31	118	434.04	368	338	333	18	43	0	0	3

SUPPLEMENTARY INFORMATION:Monte Carlo Atomistic Simulation and Machine Learning Analysis of Na-K Eutectic Alloy in Condensed Phases, D. Reitz and E. Blaisten-Barojas, George Mason University, Fairfax, VA 22030

4400	13230	9618	824	534	314	28118	1221	2	606	0	22	65	0	84	276.57	101	430.54	309	292	293	20	47	0	0	4
4278	13472	9762	879	352	186	28054	1260	6	611	1	4	75	4	75	274.42	85	429.57	283	316	278	18	54	0	0	4
4340	13418	9704	831	380	218	28070	1216	5	628	1	8	55	1	79	273.26	85	426.24	308	318	321	13	38	0	2	3
4802	12942	9406	728	608	352	28140	1153	7	642	0	28	51	2	82	277.59	99	432.62	356	321	324	20	32	0	2	4
4790	13002	9410	726	580	334	28132	1194	7	642	0	16	53	5	94	276.83	92	434.44	332	360	298	16	34	0	0	1
14328	7698	4382	8	804	610	27880	649	312	1071	158	56	251	116	155	271.05	266	435.46	474	434	445	19	82	21	2	1
12428	7776	5004	0	1676	1230	28332	568	153	733	46	206	191	81	228	293.17	154	453	495	261	425	68	103	11	10	5
3952	13650	9898	938	352	208	28070	1261	7	549	1	10	62	4	65	272.79	96	426.25	309	257	285	12	44	0	0	2
6472	11896	8472	421	808	496	28190	983	18	788	1	42	61	12	113	286.75	125	444.44	438	398	377	17	38	0	4	1
4598	13094	9516	743	560	332	28132	1161	2	600	0	30	71	1	95	279.03	81	435.55	330	311	318	22	45	0	2	1
12736	8056	4954	7	1318	982	28172	589	189	861	65	108	178	100	187	282.61	193	445.25	497	311	479	52	86	7	16	5
4010	13606	9870	916	350	212	28066	1253	7	569	1	18	71	4	85	271.47	82	425.91	293	281	283	10	38	0	0	2
13734	7808	4624	7	996	732	27982	637	282	977	121	82	226	121	183	276.59	209	441.36	453	390	457	34	92	14	6	0
13726	7774	4568	5	1024	802	28014	632	273	959	126	116	235	112	174	277.17	221	442.58	487	391	423	33	91	16	4	3
11746	7964	5262	2	1824	1378	28456	563	128	671	26	268	167	71	257	299.99	139	456.42	498	244	378	75	76	5	12	16
5736	12248	8872	566	812	478	28194	1077	18	694	4	42	69	6	117	280.99	113	439.5	388	371	320	15	39	0	6	4
4352	13382	9718	853	384	210	28062	1221	10	630	1	14	56	5	69	273.26	102	426.29	311	307	315	16	37	0	2	4
13462	7960	4672	14	1032	806	28024	603	244	966	108	84	212	106	186	275.43	230	439.9	492	361	466	28	93	13	8	1
4358	13322	9678	840	452	262	28096	1223	7	609	0	24	67	5	82	274.5	95	429.98	312	298	290	18	47	0	0	2
12076	8040	5220	1	1578	1192	28352	540	133	789	23	226	173	71	255	300.83	164	452.07	499	250	421	64	111	6	18	8
13896	7612	4444	3	1112	852	28028	589	255	935	100	108	226	121	195	278.08	221	446	482	328	455	36	101	13	4	1
4258	13474	9742	883	376	214	28068	1237	8	611	1	4	56	7	65	272.73	100	425.81	318	287	304	16	36	0	0	0
4412	13280	9656	814	480	260	28100	1204	4	619	0	12	53	2	83	276.68	102	431.27	322	308	314	15	40	0	0	2
13920	7800	4510	7	936	718	27958	635	284	1011	113	66	222	128	173	276.62	237	442.21	466	396	460	26	96	17	6	4
4184	13438	9766	883	434	250	28088	1248	2	588	0	16	60	2	78	275.29	76	429.88	295	295	293	19	44	0	0	2
11660	7574	5294	0	2094	1566	28590	493	116	545	26	354	184	52	283	318.1	99	462.56	466	162	386	102	99	11	48	34
4338	13428	9700	871	378	220	28074	1274	9	619	1	10	60	7	62	273.56	90	428.24	300	324	281	20	46	0	0	0
4072	13586	9836	929	354	210	28068	1286	6	577	0	10	60	4	77	272.14	87	425.46	283	294	273	11	41	0	0	3
5992	12174	8710	541	756	488	28178	1050	24	669	8	54	69	13	113	284.55	96	441.19	382	344	353	21	36	0	4	5
13560	7666	4556	1	1230	952	28110	616	232	875	73	140	187	114	198	284.29	195	447.77	470	334	463	41	88	22	6	5
13768	7520	4444	4	1248	966	28090	609	262	862	106	128	219	113	208	278.03	209	446.43	473	325	441	34	89	23	14	4
13776	7852	4592	4	976	716	27976	680	257	1020	102	60	228	116	153	276.13	247	439.34	484	408	415	26	110	23	4	3
11984	7914	5202	0	1710	1310	28408	542	136	691	30	258	193	65	264	300.33	128	455.38	431	224	423	98	111	15	28	16
5220	12660	9140	649	688	420	28164	1122	7	670	0	36	70	6	101	283.31	107	438.36	363	335	318	19	39	0	0	3
4974	12876	9364	675	592	304	28120	1140	11	669	2	8	60	7	97	279.33	100	435.08	350	341	331	12	38	0	2	3
14366	7656	4338	3	844	634	27886	636	334	1030	164	44	253	135	169	269.69	266	432.48	469	416	450	14	75	13	4	3

SUPPLEMENTARY INFORMATION:Monte Carlo Atomistic Simulation and Machine Learning Analysis of Na-K Eutectic Alloy in Condensed Phases, D. Reitz and E. Blaisten-Barojas, George Mason University, Fairfax, VA 22030

4156	13462	9784	899	420	244	28084	1264	4	592	0	18	63	2	78	275.33	93	428.64	289	288	283	16	44	0	0	3
14344	7534	4338	10	916	692	27910	602	328	1001	169	78	274	125	149	271.8	247	436.08	523	396	418	24	97	17	8	2
4050	13478	9830	936	444	262	28090	1259	6	535	1	26	49	4	75	274.7	81	428.56	295	249	294	19	31	0	0	1
4092	13584	9878	907	326	162	28046	1272	8	615	0	4	62	5	82	272.46	95	424.61	282	311	284	8	45	0	0	2
6244	11926	8520	396	904	582	28238	981	13	735	2	60	65	5	126	288.79	115	445.49	416	400	370	27	35	0	2	5
13686	7564	4506	4	1214	954	28088	587	276	837	121	146	237	109	198	278.09	192	445.05	491	323	427	45	95	22	18	2
12382	7862	4976	1	1580	1276	28356	605	179	709	47	258	162	96	228	294.46	126	454.23	461	295	429	78	79	8	22	9
13584	7544	4570	1	1314	962	28118	595	250	829	98	136	220	117	204	284.27	171	445.84	484	320	427	41	98	13	8	7
4492	13300	9610	835	426	242	28076	1209	8	632	2	6	54	5	80	275.66	109	429.41	307	295	328	15	40	0	0	3
4664	13186	9512	777	448	266	28094	1184	3	655	0	16	56	1	78	276.35	114	432.66	334	316	327	13	39	0	2	4
4658	13226	9548	803	404	222	28066	1210	9	683	1	8	65	3	72	273.89	120	430.08	328	334	304	13	49	0	0	2
4652	13174	9538	776	470	250	28088	1179	10	644	0	4	62	7	86	276	89	430.66	325	323	324	15	46	0	0	3
13082	7818	4804	6	1324	980	28154	618	216	839	92	132	220	93	201	282.81	211	444.41	491	309	411	40	98	15	14	7
13746	7864	4626	5	948	702	27960	645	266	1026	130	66	247	105	156	274.76	238	438.79	484	423	427	30	107	13	8	4
11444	7822	5424	2	1994	1500	28562	480	105	588	18	336	175	53	295	314.38	104	464.9	473	170	402	95	98	7	42	23
11318	7950	5426	3	2020	1524	28578	566	96	623	15	300	156	48	273	309.92	113	459.52	462	206	387	104	87	7	38	13
4600	13226	9576	823	432	232	28074	1219	10	658	2	8	48	6	60	274.67	105	429.07	326	321	314	17	32	0	0	5
11698	7934	5208	1	1918	1454	28480	621	112	676	31	246	143	57	221	294.69	142	453.15	525	259	384	70	75	5	22	12
4166	13472	9800	881	412	218	28072	1251	12	584	1	4	56	5	85	272.9	78	426.63	290	289	296	11	38	0	0	3
4580	13212	9540	784	466	282	28100	1178	11	604	4	20	63	5	92	276.18	112	433.67	322	294	322	15	36	0	0	3
4746	13078	9492	739	492	270	28102	1169	6	661	0	24	65	4	86	277.26	107	435.57	333	339	323	17	44	0	0	3
11414	7984	5356	3	1948	1520	28570	549	101	624	31	308	169	46	283	310.27	128	457.47	486	211	382	76	91	7	38	23
10718	7844	5676	1	2298	1730	28728	512	67	514	16	428	166	31	301	325.05	92	468.88	467	157	358	110	76	4	30	35
3952	13620	9910	957	368	204	28062	1307	12	579	2	8	63	7	66	272.06	91	424.01	285	280	261	12	46	0	0	2
13652	7740	4596	6	1104	828	28030	641	244	972	119	106	232	93	190	277.03	220	442.45	470	394	425	29	97	14	4	3
14176	7674	4436	4	906	660	27910	645	315	1008	153	52	256	121	134	271.72	268	434.35	516	401	417	24	93	22	6	1
13634	7834	4644	6	1020	768	28002	622	269	976	108	100	216	125	166	277.02	230	441.12	486	369	462	36	91	17	2	1
13278	7660	4650	6	1382	1042	28168	588	220	819	101	146	206	82	220	283.47	191	448.56	490	299	435	39	73	18	10	5
14250	7630	4382	4	906	678	27916	644	319	1008	152	68	244	130	159	273.05	249	435.96	486	402	442	17	81	16	2	1
13862	7780	4586	5	962	694	27960	673	275	1025	118	74	227	120	165	275.14	237	439.59	451	421	438	35	92	20	2	0
4838	13062	9426	722	476	278	28104	1151	8	684	0	24	58	3	79	278.49	106	433.24	358	345	325	17	39	0	0	2
7446	11188	7878	343	1002	644	28222	901	51	725	22	60	101	22	153	285.78	113	441.72	441	364	375	21	45	2	4	2
4288	13476	9754	867	338	190	28060	1236	8	623	1	14	53	7	75	272.02	97	425.08	304	310	311	12	41	0	0	2
13150	7746	4826	3	1340	942	28144	641	230	842	90	132	200	106	199	283.4	174	448.29	462	336	431	47	79	13	8	5
4108	13596	9870	967	306	154	28036	1319	9	621	1	2	57	4	59	271	100	423.66	283	305	258	12	44	0	0	1
13242	7722	4732	0	1328	980	28156	630	207	861	75	138	198	102	214	282.76	180	447.06	456	340	442	40	101	14	14	6

4518	13224	9594	804	478	270	28102	1168	4	639	0	16	56	3	89	277.14	116	432.26	329	289	333	12	41	0	2	5
5010	12816	9294	663	624	366	28146	1136	4	668	1	36	64	3	84	278.39	107	436.24	365	354	315	25	43	0	0	2
6582	11710	8402	451	884	552	28206	1007	25	740	6	72	82	13	128	290.12	121	444.73	407	362	339	25	52	1	2	1
5778	12380	8870	573	680	406	28142	1063	16	712	2	28	74	9	116	284.19	113	441.99	365	357	346	21	48	1	0	3
12356	7776	4942	1	1670	1354	28396	564	159	700	41	266	174	80	240	300.66	151	456.28	471	248	416	84	92	16	28	16
14326	7690	4360	7	812	632	27882	629	336	1050	157	56	259	132	152	272.14	278	436.73	493	406	433	22	91	19	6	1
4166	13512	9818	927	360	190	28052	1287	10	609	2	6	55	3	62	272.55	96	425.24	291	305	279	15	31	0	0	2
6038	11982	8680	509	912	544	28212	1008	17	694	5	50	79	9	112	285.98	118	441.25	418	335	344	28	56	0	6	3
12594	7794	4870	1	1606	1234	28308	567	174	735	56	200	185	81	242	291.48	152	450.05	490	266	423	61	86	13	10	6
12942	7912	4884	2	1290	970	28158	629	198	893	56	150	173	110	185	282.95	207	445.9	499	331	450	37	80	18	10	8
4008	13566	9872	932	388	220	28066	1272	11	579	1	12	61	7	69	272.51	99	425.05	291	277	281	15	39	0	0	4
14302	7656	4358	6	836	662	27896	596	319	1052	157	78	264	120	166	272.44	248	438.72	496	420	455	20	95	17	4	0
11296	7834	5574	0	2078	1454	28582	516	91	588	17	314	167	48	247	322.05	124	462.28	513	193	382	101	91	3	28	24
14448	7588	4314	4	850	628	27882	621	330	1039	166	52	267	128	166	270.05	265	432.66	487	417	433	18	91	17	2	0
5670	12424	8872	544	686	468	28184	1073	7	728	1	60	67	5	103	281.86	105	439.28	394	387	340	20	40	0	4	1
13510	7756	4638	7	1160	872	28054	636	241	942	111	112	235	92	189	276.83	226	442.97	486	369	410	29	106	16	6	4
4590	13268	9546	806	408	256	28088	1195	4	662	0	18	45	2	78	276.02	94	430.42	329	322	329	11	29	0	2	4
4460	13228	9588	762	516	306	28116	1162	4	601	0	16	50	3	68	273.06	89	429.27	348	303	339	26	35	0	2	2
12896	7754	4816	2	1424	1122	28238	577	187	804	63	208	205	91	220	291.76	177	451.78	495	289	414	51	103	9	18	13
3824	13774	10000	1008	270	158	28040	1333	7	562	1	14	61	3	64	271.1	90	423.28	256	279	264	17	39	0	0	1
4750	13038	9454	711	552	312	28126	1149	5	615	0	18	63	5	100	279.17	92	434.71	341	320	327	13	37	0	2	3
12372	7968	5044	3	1510	1180	28308	593	153	779	40	216	190	74	215	294.33	160	454.21	484	290	405	71	102	10	14	11
4824	12946	9408	719	588	336	28132	1152	2	647	0	30	58	1	91	279.27	91	435.65	341	329	330	22	41	0	0	1
4826	13066	9460	748	474	248	28082	1185	2	698	0	8	71	1	85	275.08	104	430.18	335	360	303	13	57	0	0	1
12144	8158	5202	9	1482	1114	28298	579	134	834	37	186	172	71	209	288.21	193	450.33	531	301	439	49	104	11	12	7
3994	13600	9902	933	360	196	28064	1260	3	564	1	10	57	1	56	271.22	83	423.97	309	269	294	17	35	0	2	3
6922	11418	8136	372	1020	674	28266	963	29	749	6	92	85	18	132	288.95	113	445.54	438	396	335	34	54	0	4	1
5118	12852	9252	641	550	324	28118	1107	9	679	1	22	61	3	93	279.96	107	436.13	367	352	341	17	45	0	0	3
10464	7938	5716	0	2382	1846	28840	527	60	465	6	434	146	28	307	333.58	91	469.7	475	153	340	114	65	3	56	32
4560	13184	9570	796	494	276	28100	1209	7	628	0	14	52	5	72	276.45	106	430.56	346	325	298	11	32	0	2	1
5874	12278	8792	546	718	452	28160	1046	20	729	5	42	74	12	111	281.53	118	438.95	397	367	344	17	52	0	4	1
6736	11634	8320	420	892	550	28198	965	30	775	8	64	74	12	132	287.93	134	443.86	400	375	378	32	51	0	2	3
14316	7600	4386	5	900	640	27896	651	319	1011	153	54	257	124	142	272.05	253	435.4	494	420	425	24	90	19	0	1
6362	11988	8602	486	736	418	28148	1001	32	754	7	42	86	17	120	287.16	129	442.79	397	379	362	21	53	0	0	3
13876	7606	4444	3	1116	868	28034	621	272	901	113	120	230	119	193	276.4	214	444.43	468	351	440	35	91	20	4	2
5094	12872	9266	677	554	316	28112	1125	13	678	1	10	62	9	83	275.76	93	430.49	373	348	334	12	42	0	0	3

SUPPLEMENTARY INFORMATION:Monte Carlo Atomistic Simulation and Machine Learning Analysis of Na-K Eutectic Alloy in Condensed Phases, D. Reitz and E. Blaisten-Barojas, George Mason University, Fairfax, VA 22030

4198	13564	9796	903	304	178	28050	1265	3	637	0	10	64	2	72	271.99	103	424.62	289	310	289	15	46	0	0	1
10678	7812	5568	0	2382	1882	28830	513	63	486	9	448	138	27	297	320.35	89	465.97	498	155	356	120	78	4	58	25
6324	11908	8504	472	858	550	28204	992	24	716	4	56	81	13	118	288.08	120	443.81	424	355	347	22	59	0	4	5
13230	7638	4620	0	1424	1112	28210	587	220	769	72	176	196	110	212	284.82	185	447.94	495	276	436	50	95	16	10	6
11280	7848	5368	3	2150	1636	28648	553	97	548	18	324	159	52	306	312.96	94	458.66	471	185	366	92	83	7	42	17
5798	12514	8874	551	568	354	28128	1098	16	785	1	20	68	12	97	281.19	142	437.97	398	414	321	8	40	0	0	0
13784	7834	4608	8	962	698	27952	633	277	1005	143	66	245	100	177	275.02	230	437.44	470	398	437	26	82	14	0	2
4502	13244	9598	802	462	268	28094	1205	4	644	0	20	66	3	75	275.73	102	430.76	325	322	304	17	45	0	0	4
12682	7838	4914	3	1504	1138	28278	549	179	779	57	188	212	87	236	294.57	168	452.35	502	270	412	55	107	18	12	11
5988	12134	8744	490	796	472	28180	1030	21	737	2	42	73	15	103	285.87	123	441.68	412	388	347	23	49	0	4	2
7240	11328	7970	370	972	646	28228	942	34	748	5	70	80	20	143	288.17	127	444.41	418	360	379	21	49	1	2	5
4668	13198	9522	782	438	250	28088	1201	9	669	1	12	62	6	77	276.61	105	431.31	333	336	307	13	45	0	0	2
13362	7944	4756	6	1058	810	28048	632	245	956	113	110	212	107	175	277.78	230	443.25	474	370	459	36	81	14	6	5
11514	7880	5336	1	1992	1496	28536	523	119	598	21	274	168	54	270	308.48	127	459.16	497	193	383	89	93	9	42	22
14136	7712	4448	5	886	668	27924	665	290	1047	142	72	257	118	177	275.81	234	439.43	439	417	422	30	98	12	2	1
12866	7784	4844	2	1450	1094	28220	587	201	827	60	164	194	112	206	282.81	182	447.97	490	305	446	51	103	8	14	10
4154	13520	9798	907	366	216	28070	1260	9	577	2	16	56	5	63	273.36	98	427.84	304	283	293	16	31	0	0	3
4310	13406	9738	871	394	208	28064	1243	9	597	2	8	77	7	73	274.11	93	427.97	307	301	280	14	52	0	0	3
11792	8000	5290	5	1814	1310	28436	550	112	693	26	202	181	55	285	301.89	132	454.5	464	247	396	73	102	7	28	13
10930	7866	5608	1	2224	1656	28710	534	67	533	13	390	169	38	295	321.34	98	466.26	461	173	365	111	77	3	34	26
4040	13554	9878	948	384	200	28062	1291	7	573	1	6	60	6	56	272.5	80	424.5	296	280	277	15	40	0	0	3
13490	7690	4666	3	1200	896	28090	583	244	874	113	134	240	100	214	277.95	209	445.35	498	315	418	27	107	13	14	6
4552	13224	9544	797	478	290	28104	1224	7	624	1	16	67	4	70	277.31	100	432.81	321	326	284	19	41	0	0	7
11330	7862	5446	1	2068	1542	28620	540	94	597	12	336	152	47	267	314.75	121	462.94	488	188	386	105	87	11	36	20
14304	7622	4358	8	872	668	27898	625	319	1018	168	70	255	103	156	272.49	258	436.11	480	395	451	23	77	25	4	4
5436	12574	9052	583	664	396	28154	1063	13	695	1	32	69	11	106	279.69	101	438.57	385	373	355	17	47	0	0	2
14396	7648	4400	9	790	566	27850	634	335	1068	167	46	275	122	148	270.56	286	433.68	500	433	418	15	93	23	4	1
4804	12966	9406	702	598	342	28138	1157	4	644	0	22	67	2	89	278.48	102	433.71	360	350	305	14	43	0	0	3
4816	12962	9408	719	590	334	28132	1173	6	650	0	22	75	3	81	276.84	92	433.53	346	343	295	23	47	0	0	3
14334	7708	4398	5	786	594	27882	626	339	1033	154	60	255	137	158	272.3	278	435.36	488	396	451	14	88	23	2	3
4576	13218	9562	776	470	262	28096	1223	6	640	1	8	66	2	78	276.65	95	433.65	325	342	284	16	42	0	0	1
14350	7694	4352	5	786	624	27876	633	326	1070	158	70	267	133	154	270.92	267	433.4	489	417	435	19	94	17	0	0
4254	13430	9752	864	396	226	28078	1240	5	612	0	20	66	2	63	275.02	105	430.19	322	302	284	19	46	0	0	1
5582	12376	8926	545	770	478	28180	1043	16	670	5	46	79	10	101	281.68	89	439.89	395	367	348	26	51	0	2	5
5332	12720	9120	648	576	354	28128	1142	16	699	0	20	60	12	93	280.11	99	436.29	344	371	330	20	37	0	6	3
14480	7544	4294	3	858	640	27878	653	318	1062	167	56	283	115	170	272.36	259	437.79	465	431	399	19	105	17	6	2

SUPPLEMENTARY INFORMATION:Monte Carlo Atomistic Simulation and Machine Learning Analysis of Na-K Eutectic Alloy in Condensed Phases, D. Reitz and E. Blaisten-Barojas, George Mason University, Fairfax, VA 22030

14290	7794	4400	3	732	592	27874	651	313	1096	158	64	254	119	149	270.74	281	433.81	485	446	439	15	86	17	2	2
14004	7744	4500	6	962	686	27944	643	295	1014	148	44	245	106	161	273.33	251	438.53	491	397	427	20	82	17	4	4
14264	7716	4442	7	802	594	27880	640	327	1065	163	58	253	125	143	269.96	288	433.54	507	416	436	13	82	20	4	2
11518	7888	5364	0	1922	1480	28536	514	105	620	17	330	175	59	270	310.71	114	459.5	502	199	391	81	100	8	32	26
11560	7910	5342	1	1924	1464	28540	529	105	650	29	308	185	58	270	309.46	132	459.07	498	214	379	88	101	4	32	19
4546	13070	9556	768	616	326	28124	1185	4	586	0	10	55	2	81	276.18	86	432.62	336	302	313	22	34	0	0	0
6214	12126	8634	450	706	442	28170	1022	21	787	1	42	81	15	104	287.93	113	444.32	410	439	344	27	49	0	6	2
3802	13686	9994	998	366	200	28060	1315	5	540	1	12	57	3	65	271.64	87	424.12	276	258	266	15	36	0	0	1
12864	7868	4804	2	1408	1100	28216	605	192	828	68	152	205	92	208	284.92	196	449.24	475	301	408	64	107	12	20	8
4558	13286	9568	812	412	246	28080	1209	8	636	1	10	57	6	78	275.13	91	429.52	318	327	315	14	37	0	0	3
11178	7956	5442	0	2102	1598	28630	519	89	572	9	310	163	46	295	316.47	111	462.66	496	175	381	86	99	5	42	21
11222	7710	5360	0	2286	1732	28714	534	85	527	9	362	152	45	305	324.65	104	467.76	479	172	360	104	80	8	40	23
13044	7870	4768	1	1290	1024	28170	640	199	851	52	164	179	107	193	280.96	175	446.23	495	353	432	37	107	14	6	6
6124	12164	8674	495	730	442	28168	1021	17	759	3	34	71	9	119	286.32	123	440.81	387	375	361	26	50	1	0	2
4202	13468	9756	902	394	240	28080	1267	6	594	1	20	52	3	81	272.82	95	425.97	289	298	293	11	36	0	0	2
3840	13716	9978	980	328	182	28050	1287	3	554	1	4	51	2	73	270.63	91	423.49	274	250	293	14	34	0	2	1
5484	12638	9076	609	572	324	28112	1092	12	731	0	18	75	7	95	280.41	100	437.45	357	380	347	19	48	0	0	6
13358	7820	4744	3	1176	860	28082	599	236	916	75	122	211	116	185	283.78	213	447.21	497	333	449	37	115	20	2	6
5642	12494	8956	558	652	378	28140	1064	9	731	0	18	77	6	92	280.63	133	437.69	415	370	330	14	49	0	0	1
4336	13466	9710	893	334	202	28062	1239	5	622	1	14	69	2	71	273.55	106	426.37	305	286	296	17	49	0	0	1
4300	13418	9726	870	394	222	28072	1234	6	604	0	12	67	4	86	274.48	91	427.88	289	295	303	15	46	0	0	3
3890	13674	9946	976	348	194	28060	1304	8	571	1	8	44	5	61	271.52	97	423.25	293	267	277	11	27	0	0	1
4438	13212	9600	796	536	316	28126	1214	2	611	0	22	44	1	79	277.51	86	432.64	321	319	319	17	27	0	2	3
5342	12688	9120	678	584	354	28116	1157	18	676	7	26	68	6	107	281.96	89	438.33	317	357	322	17	44	0	0	4
7088	11530	8118	419	838	552	28196	965	32	789	14	68	85	15	113	290.11	145	445.19	443	387	367	23	46	1	2	1
12864	7780	4784	2	1452	1150	28240	590	188	819	69	200	183	81	213	283.7	170	446.65	495	318	435	55	95	14	10	6
4300	13512	9764	888	300	160	28040	1259	6	633	1	4	63	4	70	272.39	95	425.72	293	325	296	12	36	0	0	2
12198	7808	4968	2	1706	1430	28450	525	142	680	40	302	179	68	257	303.03	137	455.59	496	227	413	90	88	12	38	9
7392	11326	7940	339	892	584	28198	904	35	806	9	60	102	18	136	288.93	140	446.24	433	390	369	38	58	1	4	1
4242	13490	9754	892	358	208	28060	1254	6	624	0	8	58	4	72	274.03	104	428	293	295	299	16	42	0	0	2
4664	13258	9536	789	378	222	28070	1202	2	682	0	12	64	0	69	275.12	113	430.85	343	337	300	14	45	0	0	0
14246	7770	4424	6	776	602	27876	624	297	1099	160	50	258	111	156	268.93	288	431.89	486	422	457	18	89	14	8	0
4072	13470	9804	881	460	272	28096	1240	5	567	1	18	66	4	75	274.01	80	429.13	311	286	281	18	43	0	0	3
4434	13362	9656	814	380	222	28070	1216	7	635	0	16	66	3	85	275.78	91	430.86	299	329	306	16	42	0	0	4
11364	7808	5290	0	2056	1680	28646	491	90	576	18	416	166	48	290	312.13	119	460.29	484	180	397	110	89	7	32	22
14066	7674	4472	6	970	694	27936	663	296	1008	143	58	244	115	159	272.62	246	437.28	484	415	422	19	84	17	2	2

4056	13614	9882	954	318	170	28046	1276	9	582	2	6	63	7	73	270.85	81	424.14	282	270	288	13	47	0	0	0
10648	7672	5614	2	2496	1908	28856	489	61	442	7	456	157	32	306	332.79	79	471.05	491	134	342	116	86	4	56	45
5024	12912	9334	680	516	296	28114	1133	8	675	1	32	58	4	105	280.44	87	438.85	342	363	337	14	44	0	0	1
10258	7898	5808	0	2466	1908	28872	479	64	456	8	476	154	31	297	332.26	97	469.87	483	140	352	140	66	2	52	39
14092	7660	4486	10	918	680	27924	602	315	1011	145	78	249	126	129	271.28	284	437.2	532	380	438	32	92	21	10	4
13964	7710	4492	8	988	734	27964	657	284	981	148	66	245	97	184	274.49	212	439.83	459	409	422	21	85	16	10	2
4278	13448	9728	876	386	224	28074	1259	7	611	0	10	61	3	78	276.81	99	431.1	306	317	277	9	42	0	0	2
3876	13694	9954	973	334	190	28058	1310	6	549	1	10	58	3	79	271.49	73	424.71	274	269	265	6	34	0	0	2
4152	13464	9796	902	418	234	28078	1255	5	590	1	14	61	2	84	272.15	82	425.68	290	278	290	13	42	0	0	2
3962	13692	9920	958	296	170	28048	1269	8	576	3	8	56	4	57	271.11	104	424.54	299	265	296	14	37	0	0	4
13986	7690	4454	2	980	764	27974	644	284	969	141	94	239	111	173	276.63	210	439.89	466	420	432	28	79	16	6	5
12160	7882	5098	0	1708	1292	28386	567	135	720	36	218	175	68	237	297.65	153	453.08	497	242	410	74	99	7	26	10
11474	7860	5338	0	1982	1540	28574	517	110	600	22	352	168	54	257	308.88	131	460.14	510	189	384	101	84	8	28	22
13158	7718	4676	2	1416	1072	28188	605	200	807	73	134	182	96	220	281.77	178	447.63	511	288	427	30	77	14	14	2
4714	13076	9470	760	542	304	28118	1160	4	619	0	12	62	4	98	279.23	97	433.88	340	301	326	10	37	0	0	2
4434	13278	9648	834	466	252	28088	1228	7	618	1	10	58	3	80	275.46	91	431.95	312	312	299	12	40	0	0	4
4158	13542	9834	913	326	172	28040	1243	13	612	2	8	61	7	69	271.22	102	424.59	305	285	294	15	37	0	0	1
13886	7682	4458	2	1052	820	28002	598	266	931	102	102	234	124	203	277.85	204	443.47	453	355	455	34	104	19	2	5
4552	13230	9588	793	450	254	28094	1214	9	620	1	20	70	6	87	279.67	70	435.69	314	341	294	16	48	0	0	1
13760	7772	4508	4	1066	826	28020	604	248	984	125	78	235	91	184	276.42	261	440.43	488	346	438	33	91	15	8	4
4010	13640	9906	936	314	170	28048	1300	8	593	2	6	55	3	63	271.82	88	424.84	271	306	285	16	37	0	2	4
14454	7592	4328	2	840	612	27878	631	327	1060	164	50	265	123	150	269.2	276	432.06	493	431	436	21	88	21	2	0
11176	7934	5396	0	2054	1670	28674	525	78	570	10	408	163	44	294	318.93	96	463.47	489	189	353	105	96	5	36	25
14408	7666	4376	4	778	582	27870	643	325	1099	146	54	251	136	156	271.47	262	435.66	475	442	447	17	96	25	4	2
13360	7886	4710	2	1140	858	28058	637	244	936	107	98	224	100	184	276.03	209	444.35	476	374	428	35	91	17	6	5
10814	7890	5604	1	2294	1716	28734	518	66	526	8	372	134	37	242	321.88	100	465.63	517	164	392	119	69	7	44	33
14402	7658	4384	5	790	572	27854	627	336	1060	166	48	260	139	168	267.48	272	430.12	475	422	451	12	81	17	0	0
14174	7662	4410	6	906	690	27920	595	319	1017	160	76	262	118	156	272.38	270	435.38	520	389	436	20	83	16	2	3
12196	7916	5150	2	1622	1218	28354	567	133	750	37	228	176	66	258	299.4	151	453.72	467	251	432	60	103	10	24	14
4316	13396	9714	868	408	228	28072	1231	6	615	1	10	70	3	72	275.92	108	430.37	318	285	286	14	46	0	0	3
4086	13468	9834	935	440	238	28080	1298	6	569	0	14	65	6	70	272.97	90	425.62	281	279	265	16	45	0	0	3
4596	13184	9520	772	484	296	28100	1173	7	614	1	20	66	4	92	277.83	94	432.92	320	305	317	21	44	0	0	1
4432	13334	9648	866	412	236	28074	1238	10	634	2	12	64	5	80	271.66	112	425.33	308	302	292	10	46	0	0	3
6466	11886	8454	421	844	516	28204	992	14	778	3	36	79	9	123	286.03	111	443.39	421	409	357	18	52	0	2	4
13510	7690	4606	0	1198	932	28090	622	235	918	94	142	211	103	203	277.26	194	446.69	476	361	440	31	100	15	12	4
4482	13308	9620	794	414	244	28088	1217	4	627	0	20	59	2	87	275.81	75	430.54	301	347	315	17	41	0	0	1

4260	13528	9784	919	298	164	28044	1287	10	616	3	8	66	5	80	272.62	92	425.67	273	309	278	8	44	0	2	3
4882	13030	9388	720	490	294	28108	1196	4	692	1	22	55	1	80	279.84	100	435.92	342	371	303	14	40	0	2	1
11968	8010	5136	1	1692	1336	28398	593	129	727	42	216	180	61	244	293.07	150	453.44	490	260	395	54	91	12	38	19
5360	12586	9052	593	708	436	28174	1070	10	667	2	32	51	6	114	280.84	107	438.73	379	339	360	18	35	0	0	0
4674	13212	9576	799	390	196	28060	1188	9	669	0	12	64	4	61	275.52	120	428.93	343	327	314	20	41	0	0	1
5172	12860	9214	662	528	324	28120	1160	12	702	1	22	60	4	87	279.78	110	437.02	356	389	306	16	36	1	0	2
3998	13608	9878	945	360	212	28070	1296	9	567	1	12	50	5	59	273.59	97	426.74	284	283	284	20	32	0	2	2
11406	7816	5358	0	2058	1586	28610	496	101	565	17	338	169	55	291	312.87	91	464.57	474	171	398	107	94	4	46	22
4508	13284	9626	854	412	226	28068	1282	11	645	1	10	56	3	73	275.21	84	428.94	288	343	275	13	39	0	2	3
5306	12604	9084	628	712	432	28166	1093	6	665	1	26	62	5	90	282.07	103	438.85	387	331	340	21	45	0	2	3
12560	7840	4916	3	1552	1212	28310	618	158	762	54	214	174	78	229	290.92	166	450.83	491	287	402	55	87	3	16	6
5936	12366	8770	542	638	406	28144	1074	14	748	3	28	72	9	107	282.54	109	437.31	380	396	342	15	47	0	0	3
3868	13682	9948	1000	340	202	28054	1318	9	555	2	12	62	4	76	270.14	79	422.67	252	253	269	14	40	0	2	4
10804	7826	5668	2	2234	1720	28754	504	70	513	7	446	159	37	294	328.12	94	471.05	450	146	385	126	88	5	50	36
4442	13410	9630	853	354	226	28074	1243	5	631	1	10	56	2	75	275.3	98	429.06	304	313	303	13	38	0	2	2
12534	7810	4992	2	1552	1178	28306	593	176	763	53	224	186	90	232	292.73	152	452	480	292	420	56	96	13	12	9
4278	13528	9760	917	300	174	28050	1286	7	634	1	10	61	6	63	273.31	102	427.26	297	323	278	11	45	0	0	1
11276	7948	5412	1	2076	1558	28604	534	79	581	15	310	164	43	266	316.29	119	462.97	497	190	370	92	79	6	18	33
4068	13572	9838	934	356	216	28068	1303	5	581	1	18	62	3	71	273.39	87	426.43	280	301	262	14	42	0	0	2
11788	7944	5272	0	1788	1374	28482	533	102	684	21	284	181	56	274	306.32	137	457.69	488	246	389	83	93	2	30	12
4088	13696	9870	942	228	140	28032	1286	8	620	1	10	54	4	62	270.93	100	423.89	283	307	295	10	39	0	0	4
4352	13356	9678	825	434	252	28086	1205	8	615	2	12	60	4	71	274.35	98	428.15	329	300	310	19	38	0	2	1
11724	8012	5394	1	1808	1250	28404	575	124	709	22	196	179	71	254	304.03	125	452.71	477	265	397	67	105	7	18	17
4378	13252	9640	801	514	306	28120	1180	7	595	0	28	42	4	91	278	90	433.15	326	281	334	15	29	0	2	3
4674	13138	9536	781	472	254	28092	1187	5	652	1	18	67	4	80	275.73	99	430.28	324	324	315	23	49	0	0	0
5386	12734	9070	615	564	358	28128	1114	12	715	3	14	60	5	83	279.69	112	436.26	369	388	345	22	33	0	0	2
12982	7832	4852	5	1360	990	28158	585	213	836	65	130	176	112	215	281.99	171	444.69	475	326	485	39	85	14	12	7
4422	13402	9672	839	354	204	28066	1234	5	641	0	12	59	2	77	274.89	100	429.59	312	324	297	12	40	0	0	1
4180	13522	9774	881	364	220	28070	1256	9	605	3	8	63	5	70	273.83	98	427.24	298	307	292	13	36	0	0	5
4232	13462	9760	890	394	218	28072	1232	7	594	1	6	56	2	77	274.05	94	427	312	275	305	13	34	0	0	0
4420	13348	9670	829	404	220	28070	1207	4	631	0	8	60	2	78	275.24	100	430.95	310	328	319	19	41	0	0	1
11134	7828	5532	1	2170	1616	28696	522	62	600	9	376	169	32	286	324.96	107	467.05	503	194	357	103	100	1	36	23
10440	7834	5708	1	2462	1908	28874	532	68	448	11	450	163	33	327	330.91	81	470.01	466	160	315	122	77	2	70	28
5392	12690	9082	633	578	362	28136	1121	5	704	1	32	66	4	94	280.49	107	440.24	362	359	332	20	45	0	0	1
14464	7648	4320	6	806	592	27866	655	321	1050	163	34	257	124	147	269.34	275	431.85	486	430	428	17	81	16	2	1
13100	7962	4844	8	1182	894	28122	655	187	964	58	132	196	95	200	283.15	191	446.61	459	406	433	42	113	13	8	1

11266	7798	5364	0	2108	1680	28654	501	92	542	14	388	189	48	280	319.32	101	462.12	471	159	377	117	100	10	50	28
12342	7838	5070	1	1642	1210	28328	583	136	759	36	208	182	64	244	294.56	142	452.99	468	269	415	63	100	11	18	13
5360	12508	9026	583	778	486	28202	1093	6	665	0	42	53	4	122	284.76	106	441.66	376	348	326	13	27	0	0	3
11816	7992	5130	0	1826	1446	28486	556	116	659	23	242	168	65	274	303.14	123	455.86	454	219	413	87	95	11	32	17
4750	13256	9518	785	334	188	28054	1205	8	710	1	8	70	5	80	275.42	108	429.3	319	353	309	9	49	0	0	3
12210	7904	5084	4	1658	1268	28378	598	132	729	36	238	179	63	242	299.68	145	453.53	469	266	404	65	98	13	16	12
4622	13312	9578	799	346	192	28054	1199	6	685	2	4	59	1	73	273.06	109	427.79	319	343	326	14	39	0	0	3
11648	7916	5338	1	1856	1416	28512	542	100	641	20	304	176	53	256	312.63	128	458.53	490	205	373	91	102	6	28	25
12260	7886	5062	4	1634	1258	28356	603	137	747	36	244	160	69	219	295.05	147	451	502	275	425	62	91	15	12	9
11590	8048	5324	0	1846	1402	28494	583	99	670	26	256	181	52	261	304.94	124	456.92	460	242	378	83	95	6	26	23
12080	7756	5184	0	1822	1322	28442	530	121	641	41	244	188	43	259	308.23	122	458.48	485	189	402	75	99	9	32	15
12938	7924	4804	3	1272	1038	28162	579	207	863	85	172	206	90	203	283.14	191	445.77	499	299	440	49	88	8	12	5
6280	12024	8626	476	754	438	28166	1034	24	769	7	40	61	9	103	285.94	104	444.03	399	411	364	23	35	1	4	4
6470	11838	8440	439	860	542	28204	972	26	737	3	48	77	15	130	287.4	117	441.49	425	374	357	20	49	0	4	4
4154	13548	9828	932	330	180	28050	1283	5	604	0	10	66	4	63	272.95	106	426.58	295	289	274	13	46	0	0	2
13930	7774	4522	4	928	706	27940	627	319	992	159	76	249	120	164	270.71	250	433.04	507	401	427	18	79	19	4	0
3758	13746	10014	998	314	196	28058	1304	7	545	1	30	53	5	74	270.91	81	423.14	261	264	282	18	41	0	0	1
12286	7956	5062	1	1584	1228	28360	560	141	781	34	222	175	79	222	301.03	163	454.79	487	288	423	82	94	3	22	15
3988	13604	9876	952	372	216	28064	1299	6	559	0	8	52	5	69	272.29	88	425.35	275	270	280	15	35	0	0	3
12152	7810	5042	3	1754	1378	28432	560	125	698	33	272	182	62	257	300.34	143	454.68	501	237	387	68	98	8	24	12
7620	10966	7762	313	1110	712	28252	895	56	755	19	76	89	29	143	290.08	113	448.39	441	386	384	34	53	5	6	4
11440	7922	5450	0	1970	1450	28572	535	88	639	19	300	161	44	271	311.65	128	458.97	489	209	405	95	81	5	40	13
4182	13492	9802	896	376	202	28062	1252	10	612	1	8	60	5	74	273.28	98	426.22	302	286	291	12	45	0	0	2
12642	7878	4906	3	1490	1154	28276	582	163	784	59	192	193	78	241	294.46	155	449.02	461	287	429	59	109	11	14	11
14426	7610	4366	6	818	592	27866	624	326	1043	153	46	261	128	138	274.39	275	437.67	488	405	449	31	87	23	8	1
4658	13054	9504	761	566	314	28116	1154	10	600	0	18	60	8	75	279.5	95	436.09	361	307	319	17	40	0	2	5
4078	13646	9880	938	270	148	28028	1272	11	611	2	6	52	5	62	270.31	94	422.49	281	290	308	15	35	0	0	3
11174	7894	5484	1	2122	1592	28642	523	67	592	19	334	158	37	259	313.39	113	460.54	517	186	376	109	86	1	40	20
4750	13010	9436	696	582	334	28132	1143	2	637	0	18	53	1	98	280.91	87	436.23	338	344	341	14	34	0	2	6
13570	7712	4550	3	1200	934	28090	643	243	887	96	102	214	99	207	278.59	192	444.77	458	352	416	37	98	27	20	4
3850	13696	9982	1017	336	178	28048	1338	8	556	1	6	55	5	69	270.02	68	423.12	252	275	261	15	37	0	0	2
5734	12374	8880	531	706	432	28168	1034	10	750	2	42	68	4	111	281.79	136	439.16	406	366	350	14	45	0	0	5
4568	13314	9584	816	380	220	28074	1207	7	628	3	8	63	2	63	276.21	101	431.8	331	314	309	21	46	0	0	1
4066	13562	9842	919	368	214	28064	1282	6	583	1	12	64	2	65	272.93	86	424.98	294	295	271	14	37	0	0	4
5134	12868	9274	672	514	296	28114	1119	8	686	0	28	57	7	89	279.61	90	437.16	345	338	359	25	35	0	0	1
6194	12108	8610	492	748	468	28162	1016	27	737	5	32	69	19	103	286.25	116	441	395	383	378	29	40	0	2	3

SUPPLEMENTARY INFORMATION:Monte Carlo Atomistic Simulation and Machine Learning Analysis of Na-K Eutectic Alloy in Condensed Phases, D. Reitz and E. Blaisten-Barojas, George Mason University, Fairfax, VA 22030

14024	7730	4494	4	916	694	27944	658	296	1027	133	80	233	125	156	274.08	248	437.94	483	422	431	26	86	12	6	1
4368	13322	9644	834	460	284	28100	1225	6	595	0	18	61	3	90	275.87	82	431.51	302	304	300	13	34	0	4	4
10866	7888	5632	0	2192	1682	28722	510	76	539	9	404	172	44	282	324.07	108	465.57	489	160	354	109	92	4	56	36
13936	7730	4578	3	942	672	27940	645	299	1029	124	80	247	136	185	275.59	232	441.12	452	419	435	26	104	16	2	0
12674	7798	4868	6	1506	1198	28284	598	168	810	52	222	190	84	224	291.93	170	450.04	496	314	400	58	100	9	18	6
13908	7822	4578	5	896	662	27940	626	282	1032	125	68	242	124	170	275.82	245	440.9	482	403	442	23	99	16	6	3
4186	13476	9756	890	404	242	28074	1258	9	578	1	10	61	4	74	271.72	76	425.29	297	288	285	15	38	0	0	3
11206	8044	5476	2	1960	1540	28616	538	87	581	21	352	168	45	288	317.56	118	463.43	455	180	384	104	85	7	38	21
14022	7704	4474	3	962	714	27948	617	297	1001	129	66	245	127	177	276.36	246	439.88	468	387	451	30	87	23	6	2
4784	13160	9462	739	428	244	28084	1173	9	668	2	6	64	4	82	276.86	103	432.57	341	350	315	15	47	0	0	1
5418	12576	9084	590	670	376	28146	1086	8	740	0	22	67	2	105	278.85	118	436.81	377	383	341	13	51	0	0	4
4288	13388	9694	866	444	268	28100	1261	4	601	0	18	54	2	74	274.77	87	430.06	291	299	296	17	40	0	0	5
6166	12008	8556	495	856	566	28210	1047	21	692	8	54	79	10	116	287.7	104	442.03	395	354	327	26	47	1	4	3
4584	13196	9554	796	482	270	28096	1195	9	630	0	10	68	8	85	273.44	93	427.71	321	315	311	14	42	0	0	4
7576	11116	7826	304	986	642	28228	893	39	813	11	78	94	22	137	286.73	128	445.56	442	411	385	32	54	1	4	6
4400	13376	9666	837	388	228	28072	1218	6	633	1	14	56	3	83	273.43	90	427.58	301	310	319	14	40	0	0	2
3990	13624	9894	933	352	194	28058	1274	6	577	1	4	55	3	78	272.72	86	426.63	273	281	297	14	37	0	0	3
4016	13608	9878	944	358	198	28062	1284	8	582	1	4	48	5	72	271.28	89	424.29	281	278	295	10	32	0	0	4
13444	7634	4640	1	1302	962	28130	601	237	859	92	138	229	103	215	282.17	193	447.3	465	305	420	46	117	16	8	7
12954	7880	4788	1	1322	1056	28182	585	200	883	67	170	189	104	206	282.08	183	447.81	485	335	469	48	91	14	12	5
4144	13510	9808	911	378	216	28070	1258	4	578	0	12	61	2	73	271.66	86	423.21	300	280	289	13	36	0	2	2
5034	12886	9302	657	560	322	28126	1108	5	699	0	22	67	2	88	279.05	111	434.89	375	364	339	16	40	0	0	3
4158	13592	9826	944	292	166	28042	1297	12	611	1	8	61	8	67	272.91	101	428.1	281	303	272	11	44	0	0	3
11568	7918	5378	0	1932	1420	28526	511	108	623	26	280	201	55	274	316.1	117	461.71	450	187	387	103	109	10	28	26
13734	7924	4648	11	898	674	27950	649	270	1044	121	62	228	108	173	276.35	257	441.23	454	430	452	26	95	17	10	4
4356	13340	9692	864	440	240	28078	1233	7	624	1	10	61	2	68	273.98	120	427.46	329	293	289	14	46	0	0	0
14266	7668	4390	4	846	648	27888	633	332	1028	159	66	255	132	149	271.83	253	435.55	488	422	439	23	84	19	4	3
11388	7950	5438	0	1986	1472	28566	565	82	600	15	304	177	38	296	317.28	86	462.01	451	219	365	87	103	8	26	22
4848	13026	9434	744	496	274	28098	1169	6	663	0	18	56	5	86	277.72	90	433.23	332	336	334	17	37	0	2	1
4272	13426	9744	867	392	224	28076	1241	5	607	0	14	62	2	73	274.34	93	428.91	302	297	296	15	45	0	4	4
3880	13776	9970	1020	248	148	28030	1326	8	569	1	8	60	4	79	272.16	81	425.13	240	267	276	13	39	0	0	1
5284	12838	9166	646	484	308	28106	1084	9	721	1	22	74	4	95	280.6	132	436.46	384	335	340	15	50	0	4	0
3866	13686	9962	967	344	190	28056	1302	7	553	1	8	59	5	67	271.97	88	424.48	270	271	278	20	36	0	0	0
14566	7644	4310	5	716	540	27820	628	358	1078	177	42	292	140	159	271.57	259	438.03	460	424	432	23	101	22	2	1
5332	12658	9168	642	610	330	28122	1108	10	721	1	24	58	8	85	278.44	102	435.79	359	354	355	25	39	0	0	3
12388	7874	5032	5	1606	1218	28356	591	155	721	39	214	175	79	197	300.68	156	453.37	520	265	398	75	91	13	24	13

SUPPLEMENTARY INFORMATION:Monte Carlo Atomistic Simulation and Machine Learning Analysis of Na-K Eutectic Alloy in Condensed Phases, D. Reitz and E. Blaisten-Barojas, George Mason University, Fairfax, VA 22030

13592	7650	4568	2	1240	922	28092	628	215	910	96	106	212	95	178	278.02	210	445.49	501	352	430	37	92	14	14	3
4264	13512	9784	875	312	164	28042	1243	9	629	1	6	63	5	70	272.2	97	426.31	304	315	299	12	44	0	0	2
13736	7824	4612	9	1004	736	27990	674	264	1007	111	70	209	123	169	275.61	234	439.12	464	415	450	24	87	17	8	2
6574	11796	8420	445	848	508	28190	991	19	770	2	42	78	14	117	287.33	127	443.96	412	377	359	27	59	0	2	4
14250	7720	4406	5	820	634	27898	700	310	1052	155	62	249	118	153	270.12	244	433.55	457	466	418	20	81	22	4	0
10998	7870	5492	0	2166	1726	28722	523	80	500	13	418	168	52	286	316.53	95	462.9	496	165	375	106	87	7	50	22
4490	13288	9590	816	446	264	28086	1235	9	638	0	8	59	5	81	276.69	96	432.18	302	328	291	20	39	0	0	1
14328	7692	4414	6	790	588	27876	634	334	1066	154	62	252	148	163	269.68	265	433.71	470	438	459	17	90	14	2	1
14144	7676	4494	4	880	630	27894	619	324	1035	154	68	276	126	162	272.08	254	436.7	486	413	418	25	107	21	2	3
13900	7810	4554	10	918	688	27944	635	290	1024	125	68	235	120	165	276.39	242	439.77	468	399	453	30	89	19	6	3
11716	7936	5212	1	1888	1454	28492	562	113	668	23	244	165	62	286	301.68	127	454.2	476	234	406	71	81	6	42	9
4034	13588	9848	930	370	220	28070	1278	5	586	1	10	62	3	78	272.18	92	424.86	283	279	280	13	43	0	0	2
14316	7794	4410	8	704	570	27864	630	328	1083	170	68	253	122	149	267.77	291	430.18	500	437	446	14	73	15	2	0
13498	7712	4568	1	1216	960	28098	624	234	888	86	134	206	121	202	278.14	185	446.19	475	356	438	33	101	15	10	4
13776	7682	4498	4	1084	862	28032	598	268	932	130	116	247	109	199	274.75	214	441.41	471	338	436	39	97	13	14	1
12242	7920	5064	0	1640	1252	28352	559	152	735	42	216	191	75	237	297.26	158	452.85	499	250	394	72	104	15	18	11
4388	13246	9642	803	522	294	28108	1183	5	580	1	16	61	4	87	274.34	91	429.02	328	285	315	18	31	0	0	2
11614	7926	5328	0	1900	1424	28500	544	118	617	32	282	174	53	288	310.74	105	458.26	447	218	389	83	92	9	26	24
4326	13464	9730	857	340	192	28062	1239	7	621	1	8	59	3	70	273.54	92	429.22	307	315	299	17	43	0	2	0
5024	12848	9248	678	618	386	28152	1120	6	674	0	24	58	6	92	278.14	113	435.71	378	335	330	17	40	0	4	0
11502	7668	5340	0	2106	1592	28602	491	112	570	28	336	179	52	299	313.74	103	463.11	477	188	383	97	100	7	52	20
5188	12712	9196	663	654	374	28148	1125	9	680	3	24	74	3	91	279.84	90	435.37	375	350	310	16	54	0	0	4
5406	12626	9110	624	622	344	28126	1117	13	694	1	18	67	11	87	282.4	107	437.08	375	376	331	15	36	0	0	3
4284	13458	9738	899	360	210	28064	1268	8	624	1	12	57	5	73	273.51	102	425.21	298	305	284	13	36	0	2	0
4586	13160	9542	763	512	294	28114	1186	5	640	0	20	65	5	77	277.74	91	434.74	341	325	307	18	43	0	0	3
4658	13160	9504	762	490	284	28110	1158	3	615	0	14	64	3	83	276.5	101	432.41	334	296	334	22	41	0	0	3
11634	7812	5300	1	2004	1468	28522	589	108	629	22	280	173	57	253	303.8	122	454.9	475	224	376	91	99	12	22	16
7422	11170	7836	359	1012	710	28254	936	45	740	8	100	96	27	151	288.07	140	445.46	423	373	352	25	58	5	4	7
4232	13394	9754	871	448	244	28084	1248	8	597	0	12	64	3	81	275.39	91	430.84	297	296	281	19	46	0	0	1
4158	13482	9808	915	394	214	28066	1282	8	600	2	8	57	4	82	273.1	83	425.27	282	304	282	9	40	0	2	1
13930	7632	4482	4	1052	788	27982	625	284	973	130	88	243	113	201	276.68	206	443.43	438	388	445	35	96	21	10	1
7164	11366	8038	412	932	626	28216	973	36	756	15	84	97	14	137	287.18	117	444.89	396	392	347	29	59	1	6	8
14198	7660	4412	9	906	672	27914	621	316	1021	159	66	257	117	160	270.61	270	434.46	513	397	431	11	84	23	0	2
4304	13366	9696	877	456	260	28092	1263	9	608	2	10	65	6	84	274.98	96	427.83	288	297	281	15	49	0	0	2
4740	13124	9482	759	458	266	28092	1188	8	674	1	22	67	6	73	276.74	112	431.4	352	354	302	14	44	0	0	0
14328	7646	4380	3	848	622	27876	655	331	1037	163	50	271	131	155	269.9	256	432.53	479	437	414	19	93	19	2	1

SUPPLEMENTARY INFORMATION:Monte Carlo Atomistic Simulation and Machine Learning Analysis of Na-K Eutectic Alloy in Condensed Phases, D. Reitz and E. Blaisten-Barojas, George Mason University, Fairfax, VA 22030

7366	11222	7954	330	1000	620	28224	900	45	797	7	54	84	28	134	287.66	135	444.65	447	399	388	31	55	3	6	3
14358	7644	4366	4	828	620	27876	614	329	1035	159	58	248	128	156	267.69	274	429.85	494	404	460	15	79	15	2	3
13054	7814	4784	0	1320	1024	28176	584	206	838	68	166	204	102	208	282.48	192	447.7	495	317	433	45	98	11	14	8
11504	7830	5332	1	2062	1518	28564	542	107	552	28	296	175	49	258	316.82	107	462.62	477	193	376	106	85	1	20	25
14066	7668	4408	6	982	758	27966	679	278	988	151	78	253	92	169	274.08	234	437.3	481	430	388	22	83	18	6	0
12146	8042	5070	4	1632	1262	28360	583	131	759	38	186	186	67	230	293.38	174	451.24	520	267	387	54	105	11	20	11
11886	7898	5242	2	1792	1338	28440	605	116	680	27	260	174	65	279	306.53	134	454.93	455	247	383	60	88	5	24	14
13412	7706	4558	0	1298	1026	28138	581	213	857	79	112	217	107	204	281.05	192	445.86	462	305	453	56	103	15	22	10
4380	13308	9650	827	468	272	28092	1221	6	599	1	14	67	3	66	275.2	89	428.85	322	300	292	23	43	0	0	2
13750	7820	4602	9	962	740	27976	636	274	1003	133	94	255	108	164	276.2	233	440.22	478	395	419	37	105	13	8	3
4842	12966	9404	728	574	320	28124	1146	8	634	1	18	56	5	69	280.47	100	434.66	360	317	338	27	37	0	0	1
14340	7518	4300	2	968	730	27934	637	307	986	150	72	249	118	173	273.06	224	437.45	477	410	435	22	84	19	6	0
3850	13736	9958	987	306	188	28050	1291	6	558	1	12	63	4	65	271.41	87	423.33	277	257	282	16	40	0	0	3
13754	7756	4616	3	1038	738	27978	644	268	967	119	66	237	109	173	276.5	208	440.46	462	410	442	33	102	18	10	2
14266	7694	4432	8	816	604	27874	648	326	1074	167	60	273	122	153	270.51	280	433	494	433	417	9	89	21	2	4
13376	7708	4618	3	1282	996	28134	591	236	838	79	146	199	114	197	283.81	212	448.18	504	314	440	40	92	19	6	4
4536	13328	9612	832	376	208	28064	1255	9	666	2	4	64	3	75	274.26	103	428.79	304	342	282	11	41	0	0	2
4324	13288	9678	829	508	292	28112	1206	3	581	0	18	66	3	82	277.33	106	431.82	326	275	301	14	38	0	4	4
7540	11178	7812	327	958	656	28226	917	47	790	8	72	104	30	142	288.77	133	444.27	437	394	357	25	65	1	10	7
13590	7628	4556	0	1264	936	28090	598	257	853	96	102	208	118	187	276.73	202	444.53	508	327	448	34	94	21	14	3
6244	11922	8528	408	916	570	28226	966	14	747	0	44	62	10	101	286.32	119	443.43	450	381	374	31	37	0	2	4
4398	13240	9652	825	510	284	28104	1211	4	607	0	16	61	2	78	275.73	99	430.93	318	305	306	17	39	0	4	5
13430	7596	4576	2	1318	1042	28148	602	226	849	69	168	209	102	211	283.2	173	446.68	473	342	428	48	110	16	18	5
13130	7734	4798	6	1316	992	28150	630	212	884	81	154	209	96	211	281.2	180	445.19	475	355	412	41	103	13	26	4
13962	7834	4568	9	860	632	27920	628	294	1057	146	58	243	116	149	274.23	250	439.06	502	437	446	21	80	9	6	2
7286	11326	7974	387	956	614	28214	964	32	737	8	54	101	19	126	287.63	116	446.25	420	381	339	33	54	1	4	5
4024	13616	9878	939	338	192	28056	1266	9	571	1	6	59	4	66	272.32	91	425.58	301	261	287	14	41	0	2	1
4106	13610	9860	938	288	162	28038	1307	5	624	1	12	57	2	73	272.57	82	424.55	264	314	279	10	45	0	0	3
5790	12458	8912	556	592	342	28118	1083	9	779	1	24	73	5	98	282.84	112	438.85	386	412	338	11	43	0	0	3
14006	7696	4530	2	928	686	27940	615	293	1019	147	86	257	106	162	274.43	242	440.16	494	420	429	30	86	13	8	1
4522	13264	9590	796	444	258	28094	1179	4	626	0	16	57	4	81	278.3	103	434.88	341	310	326	12	33	0	0	1
14524	7596	4308	6	770	594	27866	675	353	1057	177	70	250	129	169	270.76	258	434.67	447	459	437	13	62	21	4	1
14288	7642	4410	4	862	630	27898	661	316	1064	163	62	247	126	136	270.79	264	434.59	503	460	427	18	78	13	4	2
4346	13338	9704	836	434	238	28076	1221	6	605	1	14	75	3	72	273.73	97	426.12	326	310	286	15	48	0	2	2
13786	7648	4502	8	1106	860	28030	624	292	941	117	120	219	129	178	275.93	235	442.64	469	358	450	42	83	22	8	3
4486	13290	9614	802	442	248	28088	1207	4	644	1	8	65	3	79	277.73	105	433.7	318	310	307	19	41	0	0	1

SUPPLEMENTARY INFORMATION:Monte Carlo Atomistic Simulation and Machine Learning Analysis of Na-K Eutectic Alloy in Condensed Phases, D. Reitz and E. Blaisten-Barojas, George Mason University, Fairfax, VA 22030

13146	7660	4694	2	1412	1100	28214	556	215	822	79	184	199	104	213	282.3	179	445.63	525	306	442	45	100	14	18	3
5656	12494	8928	583	636	400	28150	1107	19	732	2	32	72	11	112	283.5	103	440.03	364	397	319	16	49	0	4	1
4508	13264	9586	811	456	268	28096	1223	7	624	3	14	54	2	75	275.86	104	430.52	331	316	294	12	30	0	0	2
4326	13420	9720	854	374	212	28066	1251	6	621	1	14	55	4	72	274.62	89	429.4	301	330	297	12	38	0	0	3
11482	7944	5418	4	1946	1430	28532	530	110	610	15	280	180	62	265	310.49	125	459.82	505	192	387	77	102	9	26	25
11824	7796	5114	1	1926	1520	28510	519	119	639	34	304	181	55	270	304.95	134	458.37	492	202	396	92	98	9	26	15
5836	12382	8846	573	642	396	28138	1091	19	742	4	34	76	12	110	283.62	114	440.75	361	378	334	19	46	0	2	2
13962	7806	4514	4	892	686	27936	640	300	1007	152	72	254	118	157	272.93	267	437.17	496	412	418	25	87	13	4	1
14256	7678	4428	3	838	622	27892	649	313	1072	155	70	266	123	145	271	275	434.21	498	430	412	22	102	19	0	1
4492	13208	9576	800	522	302	28114	1197	2	602	0	14	68	2	82	276.76	94	431.67	340	295	293	13	45	0	0	3
6076	12134	8708	532	750	452	28168	1033	24	691	6	46	67	14	105	285.85	113	440.43	404	346	354	21	44	0	2	4
14040	7788	4522	9	846	640	27910	653	305	1061	144	66	248	123	156	272.27	271	435.28	482	435	428	21	89	20	6	2
14384	7736	4422	7	704	536	27848	657	321	1102	158	66	259	127	129	269.23	280	432.37	495	464	430	21	95	20	0	1
14612	7576	4338	11	734	518	27830	659	334	1097	172	48	274	128	139	270.19	272	433.01	478	457	422	19	90	18	4	2
14242	7614	4394	7	894	682	27916	630	348	985	172	88	262	123	157	270.03	249	434.33	512	397	408	18	80	26	2	1
13464	7758	4562	4	1208	972	28104	635	254	878	110	124	224	110	191	278.85	227	442.77	508	359	406	27	87	17	16	2
11174	7764	5446	3	2250	1658	28668	527	90	532	19	340	171	46	306	319.62	90	460.23	470	176	366	96	94	10	34	26
5116	12820	9288	680	558	306	28114	1133	8	705	1	26	68	6	96	277.91	111	433.89	344	337	325	20	49	0	0	3
14374	7766	4384	6	708	562	27852	657	331	1096	161	54	257	128	136	268.16	291	429.22	478	452	437	24	81	18	4	2
7728	10908	7704	265	1102	728	28272	857	39	796	6	88	94	25	156	288.15	136	444.04	459	400	380	35	62	2	14	3
6614	11814	8378	398	836	516	28198	985	19	824	1	38	72	14	116	285.08	134	442.84	430	435	365	20	47	0	2	3
14112	7746	4500	7	842	624	27892	619	318	1049	151	64	261	131	156	270.45	273	434.47	488	394	444	21	100	20	2	4
4070	13708	9854	936	248	158	28040	1294	7	608	1	2	61	5	65	272.58	88	425.62	278	300	283	11	43	0	0	4
5066	12780	9260	671	646	370	28146	1139	7	659	1	22	68	5	84	282.53	116	437.52	367	335	314	22	49	0	2	2
4884	13014	9392	715	508	292	28108	1183	12	683	3	18	55	5	83	280.08	119	435.52	348	369	312	11	40	0	0	1
4076	13510	9828	912	416	236	28078	1274	9	583	2	12	54	3	70	272.31	86	425.59	291	293	286	17	30	0	0	2
11212	7856	5488	1	2094	1586	28638	514	92	556	23	356	173	42	294	315.57	101	465.21	480	181	362	105	85	5	44	24
13672	7542	4542	2	1272	928	28084	631	247	843	109	118	228	93	198	277.35	184	445.43	484	336	412	31	91	20	8	4
5372	12672	9112	643	602	348	28124	1110	8	699	0	16	70	5	83	279.21	120	437.43	385	338	321	20	50	1	2	1
4396	13306	9660	847	452	256	28086	1248	12	597	2	16	62	8	83	274.59	88	428.33	299	313	286	15	40	0	0	1
10856	7838	5600	0	2320	1714	28738	525	61	513	8	358	156	33	302	320.65	87	467.46	488	174	338	118	80	3	48	22
4076	13508	9828	905	424	236	28080	1259	4	564	2	8	59	1	78	272.74	75	427.13	286	289	297	17	36	0	0	2
4922	12894	9320	703	610	370	28146	1152	11	647	2	28	61	6	85	280.44	104	436.65	359	325	311	21	40	0	2	3
12186	7980	5186	4	1588	1162	28318	585	159	766	40	194	193	90	247	290.41	157	450.85	478	281	413	52	107	10	20	10
5252	12840	9208	643	496	290	28106	1137	3	767	0	18	58	3	85	278.43	128	436.32	362	389	339	12	35	0	2	2
6818	11656	8240	389	848	570	28210	957	22	781	7	74	83	13	142	285.15	116	443.96	396	406	378	32	45	0	4	2

SUPPLEMENTARY INFORMATION:Monte Carlo Atomistic Simulation and Machine Learning Analysis of Na-K Eutectic Alloy in Condensed Phases, D. Reitz and E. Blaisten-Barojas, George Mason University, Fairfax, VA 22030

4418	13400	9684	866	348	194	28054	1244	7	642	1	10	66	4	75	272.72	103	425.12	305	297	290	11	48	0	0	1
6912	11420	8172	365	1020	636	28232	934	35	730	6	72	90	19	148	287.1	119	445.47	427	376	355	26	52	1	0	4
11026	7792	5630	1	2204	1606	28686	478	87	519	17	386	179	39	305	322.89	88	466.09	481	168	357	114	91	2	40	27
4146	13486	9820	897	398	210	28068	1272	3	585	1	6	61	2	72	272.55	83	426.79	291	299	277	18	40	0	2	1
11300	7890	5358	4	2100	1610	28610	547	101	570	22	314	153	56	270	309.57	111	456.7	489	206	391	96	74	5	34	19
12382	7884	4998	1	1616	1232	28326	589	160	744	39	188	177	83	243	295.4	152	452.97	474	277	411	62	88	8	22	11
10886	7856	5522	0	2284	1752	28728	493	75	509	9	382	145	37	292	322.43	95	465.31	510	152	375	107	77	5	46	24
4914	12962	9338	719	554	336	28126	1154	7	674	0	22	58	4	81	277.94	112	433.31	355	330	326	18	43	0	0	2
4522	13226	9590	814	468	270	28098	1214	7	627	0	22	55	3	75	274.19	106	427.91	332	315	303	12	39	0	0	3
11898	7918	5246	3	1768	1318	28426	539	128	708	27	258	178	69	247	298.08	153	456.98	495	238	419	70	103	9	20	21
4058	13544	9832	911	406	234	28082	1267	9	565	1	6	60	6	65	273.2	103	426	312	276	274	13	28	0	2	2
11140	7880	5486	0	2124	1610	28630	499	75	570	10	356	189	32	303	320.18	103	465.07	426	168	376	126	98	4	30	28
12264	7884	5050	1	1602	1282	28374	526	165	728	47	270	176	79	248	298.49	149	453.62	505	252	433	68	90	12	20	13
3870	13674	9980	981	342	176	28050	1321	9	570	1	8	53	5	64	269.92	86	422.15	271	277	266	14	41	0	0	2
5114	12752	9226	677	642	380	28144	1154	11	646	6	28	67	3	108	280.8	88	439.42	334	353	307	15	41	0	0	5
14194	7688	4396	7	872	686	27918	678	318	1030	166	74	259	117	154	269.12	275	430.98	493	430	390	15	83	16	8	2
3964	13698	9928	985	276	160	28038	1321	8	586	1	12	66	5	61	271.18	85	424.23	269	274	259	15	43	0	0	3
11806	8068	5328	2	1728	1250	28408	592	120	738	24	204	163	70	232	298.04	142	454.1	486	250	418	74	95	10	20	11
11626	8080	5338	1	1810	1340	28442	593	107	698	25	232	153	41	248	305.64	140	456.91	462	245	400	85	86	8	16	13
7056	11200	8086	345	1132	702	28274	933	28	749	8	92	81	16	138	288.96	117	444.71	445	382	360	27	51	1	6	6
10964	7788	5596	0	2236	1680	28718	533	84	526	14	406	160	48	292	316.25	105	465.24	474	176	366	113	75	4	44	22
4624	13098	9512	723	562	314	28124	1141	4	628	0	14	55	4	89	276.05	86	432.76	360	331	335	14	35	0	0	1
7788	10940	7692	343	1014	682	28216	906	59	755	17	96	113	29	155	288.59	133	446.96	414	358	355	36	66	3	4	3
13330	7642	4698	1	1314	978	28130	602	246	847	97	158	223	109	222	279.47	179	446.38	465	317	433	42	98	19	10	2
13716	7904	4594	7	932	738	27974	639	255	1020	123	84	233	105	182	277.09	245	441.66	467	398	449	21	92	18	6	2
4376	13414	9690	855	368	208	28062	1249	7	641	0	6	75	6	70	274.75	111	429.16	305	326	283	15	43	0	0	2
4954	12932	9338	679	558	322	28122	1131	6	667	1	16	68	4	89	276.81	107	433.87	361	344	325	18	42	0	2	2
5424	12538	9060	598	712	408	28168	1081	8	691	1	24	74	5	93	280.04	112	436.78	405	363	321	16	55	0	0	3
5122	12896	9288	687	490	280	28100	1134	5	700	0	24	78	5	91	279.41	115	435.39	343	354	326	20	43	0	0	3
14298	7728	4420	4	756	594	27878	655	333	1055	168	82	267	122	160	269.6	257	431.93	481	431	416	15	88	23	0	0
11742	7980	5260	0	1830	1384	28470	540	108	670	17	240	162	64	254	302.82	144	453.85	488	213	419	86	83	9	32	13
4578	13246	9550	795	434	264	28090	1206	7	644	1	18	66	5	70	276.14	91	430.13	339	334	300	12	38	0	0	4
4650	13186	9532	788	460	254	28090	1210	7	649	0	8	62	6	89	276.32	91	432.65	319	337	302	11	44	0	0	1
4920	12926	9360	713	564	328	28128	1142	5	634	0	30	52	3	90	280.31	91	437.08	352	321	335	18	35	0	0	2
4584	13142	9552	766	522	290	28108	1193	8	656	0	18	60	6	95	276.56	90	432.96	308	329	314	20	43	0	0	2
13934	7780	4546	7	912	684	27938	618	275	1040	122	80	240	117	179	273.9	236	438.53	460	396	470	27	102	15	2	1

SUPPLEMENTARY INFORMATION:Monte Carlo Atomistic Simulation and Machine Learning Analysis of Na-K Eutectic Alloy in Condensed Phases, D. Reitz and E. Blaisten-Barojas, George Mason University, Fairfax, VA 22030

4578	13190	9578	791	480	258	28098	1205	8	629	0	14	72	4	77	275.56	109	431.13	334	310	288	19	50	0	0	0
4108	13520	9824	927	382	218	28066	1269	7	578	1	14	64	6	81	273.58	90	426.48	288	270	285	9	41	0	0	2
13182	7910	4740	8	1228	932	28102	679	231	885	111	100	212	93	182	278.06	199	443.11	483	376	406	32	79	11	10	1
4172	13470	9780	902	402	234	28074	1269	9	588	1	16	68	5	75	272.59	98	425.05	303	294	263	12	49	0	0	2
11700	7778	5288	0	1944	1464	28524	530	125	610	25	328	154	72	260	304.4	119	457.18	494	205	415	82	78	6	20	23
12812	8098	4822	4	1244	1044	28188	621	181	877	67	160	194	87	222	283.01	174	445.24	474	342	438	39	90	12	8	1
4180	13508	9806	890	356	196	28058	1248	8	583	2	12	66	5	65	271.85	84	424.73	295	300	301	20	44	0	0	3
10944	7954	5586	2	2196	1612	28654	526	72	549	11	330	168	39	279	317.9	107	459.46	509	175	356	95	94	2	32	24
11210	7798	5468	1	2216	1604	28642	507	81	530	17	302	171	42	292	317.66	99	462.52	488	180	374	101	87	3	38	21
14304	7756	4404	5	756	592	27876	645	328	1071	159	60	251	126	135	270.37	298	434.42	503	428	438	20	83	21	4	1
6730	11504	8230	389	1020	676	28258	923	28	677	5	96	75	17	148	288.07	105	445.43	412	356	387	33	34	0	2	8
3990	13634	9914	941	328	176	28050	1257	8	580	2	6	56	3	65	270.3	93	422.92	303	270	297	13	37	0	2	2
6130	12016	8602	468	878	550	28228	1006	14	709	2	50	72	8	124	287.12	125	443.02	414	364	349	24	46	0	2	3
14304	7658	4398	6	828	628	27892	654	334	1050	152	72	240	145	147	271.59	252	434.28	475	451	454	21	78	18	4	3
12008	7958	5178	3	1726	1288	28402	531	126	694	39	230	189	60	252	298.78	143	454.32	500	216	409	76	105	7	14	9
14156	7686	4446	9	880	664	27910	596	325	1021	154	74	236	130	165	272.24	265	435.64	496	385	478	19	72	20	4	1
4400	13418	9702	877	340	184	28052	1221	8	624	2	8	53	5	73	272.64	101	425.26	317	290	318	10	37	0	0	1
4652	13030	9462	698	616	364	28150	1118	2	621	0	26	58	2	93	277.26	81	433.86	365	322	343	20	37	0	0	1
13202	7634	4716	1	1390	1056	28198	582	203	814	71	190	218	92	222	286.57	185	450.14	474	293	431	52	112	22	8	8
4132	13558	9832	929	322	190	28056	1266	8	613	0	22	55	6	66	271.83	107	424.54	290	284	295	17	38	0	0	3
13540	7916	4696	4	978	766	28018	621	248	1006	108	114	231	99	160	276.46	245	442.73	515	378	426	32	101	23	8	3
4996	12804	9270	666	670	394	28158	1172	8	631	1	24	60	5	95	278.51	85	435.78	336	365	307	25	41	0	0	0
14348	7746	4394	8	722	576	27856	648	348	1083	157	68	262	142	158	270.89	261	434.85	446	453	456	25	85	28	2	2
11732	8016	5342	3	1770	1316	28454	584	125	686	33	242	179	59	242	301.95	133	456.88	459	246	397	100	91	12	34	12
4520	13378	9670	835	312	156	28044	1218	7	670	0	8	77	6	65	275.92	104	431.74	325	330	298	13	53	0	0	2
12040	7848	5166	1	1804	1318	28430	588	124	675	26	240	186	69	226	306.38	130	455.9	490	229	383	82	101	12	14	19
10472	7994	5760	2	2352	1770	28786	488	55	474	5	372	152	28	290	323.4	93	464.54	537	138	341	112	72	2	58	28
11268	7732	5490	2	2218	1572	28634	542	90	535	19	318	163	42	265	323.32	96	464.99	492	174	363	112	79	8	34	18
4778	13038	9424	733	558	316	28122	1190	2	647	0	8	72	2	87	277.56	99	433.12	349	346	284	10	45	0	0	3
14188	7656	4408	7	900	686	27918	628	326	995	157	78	255	128	161	272.18	255	435.47	482	392	439	28	89	15	2	0
11834	7786	5148	3	1928	1484	28492	536	136	655	30	284	175	78	270	298.46	132	455.03	487	227	402	72	100	7	26	22
4834	12994	9402	727	556	318	28122	1165	6	655	0	18	58	4	92	280.05	86	436.66	326	330	335	19	44	0	0	3
14258	7672	4390	9	844	656	27900	648	328	1036	154	78	247	133	150	271.55	244	435.72	470	432	454	25	80	21	2	3
14094	7548	4438	6	1044	746	27950	616	311	989	157	76	272	122	180	272.54	256	437.78	492	379	415	22	100	17	4	0
13604	7950	4690	4	938	710	27980	618	261	1021	119	88	229	113	180	275.96	252	439.11	487	390	452	23	89	15	0	1
4582	13294	9564	810	388	234	28074	1223	9	649	1	12	54	3	68	275.1	97	428.78	335	332	302	10	41	0	0	1

14258	7722	4410	10	826	618	27886	660	332	1020	166	50	256	126	142	269.96	258	433.36	484	421	427	25	78	19	2	0
4100	13578	9834	924	344	196	28058	1282	6	594	2	6	58	2	57	271.68	109	424.9	302	293	275	15	34	0	0	3
6480	11898	8458	454	788	500	28176	994	21	744	5	48	85	13	121	284.89	116	441.84	402	393	360	26	53	0	4	3
13958	7504	4386	1	1172	902	28052	581	289	882	104	126	219	142	183	277.98	225	445.24	497	318	463	40	96	20	4	3
6598	11780	8382	470	834	530	28182	1008	34	722	6	56	72	20	114	285.7	116	441.3	404	378	364	25	44	1	2	2
11104	7992	5564	0	2078	1524	28608	482	78	558	13	318	144	42	267	319.1	118	463.32	519	173	413	93	68	5	22	29
10736	7758	5612	0	2404	1820	28810	511	59	498	11	424	152	26	302	331.12	87	467.34	471	160	374	125	72	5	56	33
5570	12434	8926	559	756	468	28188	1023	16	671	2	32	80	8	108	283.59	117	440.99	431	338	327	18	45	0	2	2
10968	7866	5508	0	2236	1724	28732	524	70	505	16	378	170	33	280	324.07	93	464.62	485	159	350	106	86	4	44	39
7136	11388	8098	363	936	578	28198	913	38	784	13	56	88	18	135	289	121	445.09	416	370	394	36	54	1	6	4
4660	13152	9520	788	480	272	28102	1209	4	646	0	18	57	3	83	277.58	95	430.79	325	332	306	14	37	0	0	1
5670	12568	8966	575	552	338	28124	1068	13	755	5	26	83	7	103	280.41	128	438.13	384	388	343	12	52	0	4	4
4744	13050	9452	752	532	308	28106	1179	7	650	1	20	59	4	92	278.91	89	434.17	320	333	319	16	37	0	0	4
4056	13632	9876	940	296	172	28046	1289	7	596	1	14	65	5	67	272.34	81	425.03	278	297	278	15	42	0	0	3
4456	13304	9658	831	420	224	28074	1239	8	623	0	12	62	4	79	275.76	85	430.56	306	320	289	14	44	0	0	2
4148	13474	9804	890	412	226	28074	1248	5	582	1	8	55	1	71	272.22	93	425.79	309	287	296	13	44	0	2	2
10822	7796	5616	1	2380	1718	28726	535	67	539	7	354	157	40	292	324.88	93	466.02	447	171	383	129	88	3	40	22
13894	7764	4510	4	976	746	27974	623	283	989	138	76	243	109	190	275.41	228	438.87	465	397	446	22	80	14	8	4
6204	12102	8648	478	732	436	28160	966	19	788	3	38	71	11	126	284.99	135	440.26	410	359	396	18	52	0	0	3
13428	7692	4672	3	1256	914	28088	605	239	880	102	122	230	114	198	277.14	201	445.72	494	340	427	35	99	12	2	2
4040	13556	9872	917	376	206	28062	1266	5	581	0	10	63	2	87	271.29	89	425.58	269	288	290	16	39	0	2	2
11316	7976	5418	1	2004	1526	28588	525	90	631	11	318	154	51	292	311.98	128	462.65	476	191	406	92	104	12	30	17
14210	7554	4410	5	1004	698	27934	630	333	953	154	54	233	137	154	273.2	241	437.65	494	397	453	22	68	18	4	4
3978	13602	9892	962	364	212	28064	1308	8	582	1	14	55	4	68	271.15	91	422.82	278	300	270	12	37	0	2	3
4248	13362	9724	893	474	270	28094	1248	8	574	1	16	67	4	84	274.5	91	429.67	303	276	276	13	46	1	0	3
14380	7706	4356	8	756	602	27868	653	335	1077	165	62	256	128	147	268.57	283	430.34	488	453	435	14	84	17	6	2
4554	13338	9608	856	352	198	28056	1264	7	658	1	6	62	1	79	275.61	94	431.2	282	340	284	14	40	1	0	4
5006	12966	9342	661	494	280	28106	1128	3	708	2	18	58	1	84	278.9	114	435.2	355	365	352	19	28	0	0	1
14266	7684	4392	7	820	650	27900	646	311	1039	161	84	255	113	143	270.37	274	433.27	495	420	434	24	82	21	4	0
4538	13252	9586	785	438	256	28090	1176	4	632	0	20	67	3	96	277.71	90	433.8	310	303	329	17	46	0	0	1
14336	7618	4332	4	860	680	27912	610	332	1011	163	78	262	129	175	272.47	238	438.68	459	393	455	32	83	16	8	1
12092	7826	5032	1	1834	1400	28432	563	147	651	32	222	175	83	247	299.71	143	456.74	486	222	399	92	93	8	26	6
4258	13410	9718	876	430	256	28088	1261	7	595	1	16	61	4	90	272.31	77	425.1	294	302	278	8	41	0	0	2
4856	12970	9360	680	568	354	28142	1149	4	648	0	32	58	3	76	278.13	93	434.39	372	350	323	17	34	0	2	3
12232	7936	5038	4	1648	1268	28344	562	159	727	46	202	174	75	240	290.9	161	450.31	490	263	422	67	87	11	20	9
4598	13148	9516	788	526	312	28120	1218	5	610	0	20	55	3	74	277.3	89	433.32	330	311	297	20	39	0	0	1

SUPPLEMENTARY INFORMATION:Monte Carlo Atomistic Simulation and Machine Learning Analysis of Na-K Eutectic Alloy in Condensed Phases, D. Reitz and E. Blaisten-Barojas, George Mason University, Fairfax, VA 22030

12464	7912	4894	5	1554	1274	28342	562	148	754	52	212	187	75	220	294.17	181	453.12	529	260	412	56	89	6	32	11
10908	7816	5646	0	2256	1660	28722	462	70	531	10	388	183	37	308	329.11	92	471.34	483	151	383	97	103	5	44	34
12396	7754	5012	0	1720	1258	28362	590	162	709	46	196	190	67	246	299.12	138	455.93	481	271	368	73	101	22	22	9
13718	7760	4610	6	1042	772	28006	611	268	982	118	100	239	117	206	276.06	224	438.98	483	375	429	19	105	13	4	0
4602	13064	9496	730	598	352	28138	1139	9	603	2	26	60	6	88	279.65	89	435.18	356	296	329	22	41	0	0	1
7766	11002	7686	323	984	688	28232	888	35	797	6	100	100	20	158	286.96	154	447.75	434	362	371	31	64	1	6	2
12766	7994	4942	3	1322	1000	28178	637	181	886	65	134	198	80	195	281.94	189	447.61	507	360	406	32	105	17	20	9
11198	8084	5390	1	1966	1600	28624	558	69	584	17	348	169	32	277	313.9	116	458.34	484	199	364	97	79	4	36	18
13372	7692	4622	2	1280	1006	28144	602	228	835	79	164	210	108	196	284.62	187	447.47	494	325	427	45	94	17	6	7
12692	8150	5012	4	1232	932	28156	611	183	909	51	134	181	99	212	284.17	184	447.98	478	359	454	39	100	8	4	4
4784	13004	9416	752	556	336	28128	1163	4	641	0	28	58	3	89	279.88	83	435.24	347	317	319	16	38	0	4	2
4578	13308	9606	785	368	198	28066	1199	10	658	0	8	70	6	79	275.82	106	431.77	330	343	299	12	43	0	0	2
3948	13620	9918	957	364	200	28060	1303	4	561	0	10	53	2	68	271.04	80	423.47	270	272	278	18	36	0	0	2
12130	7994	5126	3	1668	1240	28364	554	130	748	34	184	182	73	236	291.97	159	450.6	512	243	418	67	105	5	22	8
6682	11730	8328	418	868	542	28198	934	20	772	1	48	99	17	127	285.21	143	444.17	444	356	367	22	75	0	0	3
11802	8006	5238	1	1764	1340	28410	548	112	737	26	246	199	61	241	294.71	156	453.45	500	238	389	86	111	7	14	9
3918	13666	9960	986	320	170	28048	1307	9	567	1	14	56	6	79	270.17	87	422.85	265	279	274	9	40	0	0	2
12716	7732	4944	2	1562	1124	28276	601	170	738	56	182	184	84	237	292.86	144	452.9	453	277	424	60	92	11	14	10
4174	13512	9804	891	362	200	28062	1217	6	575	1	10	65	2	81	273.51	95	429.05	306	252	312	12	43	0	0	3
3732	13834	10030	1016	272	168	28048	1310	4	545	1	12	58	3	63	269.5	94	422.34	283	245	272	8	38	0	0	3
6542	11770	8394	429	874	562	28212	973	15	766	5	68	70	7	132	285.8	121	442.86	406	379	376	29	44	0	2	1
5132	12776	9226	648	620	362	28140	1103	11	650	2	22	54	8	96	279.54	98	437.66	375	351	347	15	30	0	2	2
13854	7746	4578	6	982	720	27968	599	281	1003	124	78	240	119	187	275.26	244	441.7	487	372	442	22	93	16	8	5
13478	7644	4648	3	1254	926	28090	616	251	875	102	126	219	114	203	275.36	191	445.79	471	324	438	38	93	20	10	3
3934	13648	9932	966	344	190	28060	1310	8	576	1	12	64	4	80	271.54	78	424.84	268	272	261	12	46	0	0	0
14238	7728	4424	7	816	620	27888	649	315	1051	167	58	260	115	147	269.72	256	432.46	491	439	425	22	80	17	2	0
13756	7828	4554	5	988	770	27986	646	258	997	116	84	231	115	170	275.49	216	440.59	468	414	441	32	97	14	6	3
14348	7642	4376	6	844	620	27886	629	329	1056	162	52	252	131	158	269.52	269	431.88	487	433	449	15	79	14	4	3
13480	7692	4616	2	1250	932	28094	647	229	891	90	116	212	100	200	280.99	179	446.95	439	364	434	45	94	12	6	7
5546	12494	9006	591	688	390	28144	1085	17	716	0	20	65	11	94	282.34	117	439.04	397	352	337	12	46	0	0	3
11430	7912	5264	1	2018	1598	28574	566	93	590	17	322	172	54	270	310.16	124	458.4	494	213	356	90	89	7	30	16
6156	12006	8644	483	854	498	28198	1023	24	710	5	36	76	16	101	286.44	121	440.8	436	369	335	20	53	0	4	2
4266	13462	9770	874	364	190	28058	1242	8	603	1	6	60	5	73	273.05	85	427.01	302	301	301	15	40	0	0	1
12116	7868	5078	2	1734	1350	28426	543	142	676	33	254	184	88	240	302.93	153	456.55	515	239	394	73	91	6	24	14
14472	7772	4430	7	632	462	27810	653	327	1154	162	40	268	124	136	269.41	299	432.12	472	467	449	16	98	23	2	3
10982	7834	5532	0	2228	1684	28678	494	82	541	11	386	174	31	272	318.51	108	464.28	509	174	356	117	89	6	30	28

SUPPLEMENTARY INFORMATION:Monte Carlo Atomistic Simulation and Machine Learning Analysis of Na-K Eutectic Alloy in Condensed Phases, D. Reitz and E. Blaisten-Barojas, George Mason University, Fairfax, VA 22030

14292	7664	4386	7	832	644	27894	645	328	1015	157	64	253	119	152	272.94	239	437.32	476	426	434	28	83	23	12	1
14162	7612	4420	5	976	698	27928	649	296	1002	154	60	262	105	163	272.55	248	436	487	391	413	17	95	24	0	4
4390	13226	9632	809	530	312	28120	1197	8	587	0	30	53	5	87	275.25	92	430.78	332	291	309	14	38	0	0	2
5030	12890	9306	670	564	318	28124	1102	5	682	0	16	57	3	88	279.42	111	436.99	380	330	354	13	36	1	0	1
6810	11574	8244	459	924	584	28198	959	46	704	10	60	84	24	129	288.59	120	444.81	402	340	373	35	49	3	2	4
4446	13154	9636	790	570	296	28120	1188	5	571	0	18	73	4	72	278.48	89	433.26	356	289	281	22	50	0	0	1
6610	11828	8414	435	804	480	28172	990	26	763	4	34	78	13	111	285.17	129	440.96	434	391	351	21	50	0	2	1
5406	12596	9084	597	646	388	28162	1114	3	752	0	36	61	3	90	281.64	120	435.43	373	400	335	21	43	0	4	3
14436	7588	4348	7	846	608	27880	629	337	1012	164	52	255	138	150	270.29	271	432.21	506	408	443	13	77	17	2	0
3862	13660	9954	988	368	206	28060	1302	10	532	2	10	56	5	71	270.8	79	423.08	274	256	271	14	37	0	0	3
13038	7962	4880	9	1230	898	28134	662	208	890	72	116	187	103	182	281.41	199	447.05	484	372	430	39	88	21	10	3
5998	12136	8678	463	824	526	28214	1030	11	721	0	48	61	7	106	285.86	100	442.46	411	402	354	28	41	0	4	2
10988	7730	5528	2	2330	1748	28772	542	77	478	12	394	158	48	301	324.25	81	468.06	482	163	344	91	79	2	46	42
12540	7894	4944	0	1492	1192	28306	556	154	795	44	220	209	72	240	301.47	177	455.93	473	259	418	68	108	15	24	10
8508	10474	7268	252	1152	764	28256	850	60	809	16	86	110	34	145	289.19	160	447.37	477	373	375	32	62	1	4	3
14266	7734	4400	5	792	620	27878	649	310	1089	157	64	268	120	144	270.78	285	433.46	493	447	419	21	98	16	2	1
4256	13436	9732	867	394	240	28080	1232	5	601	0	20	56	1	73	274.71	98	429.34	305	301	306	16	35	1	2	5
5188	12880	9206	656	510	318	28124	1142	8	704	1	22	53	4	86	277.78	103	433.37	368	371	332	12	34	0	0	1
12192	7996	5108	4	1604	1222	28352	600	147	715	42	220	184	76	242	294.15	139	451.2	475	269	415	62	99	11	10	8
13490	7678	4636	3	1248	912	28080	620	239	911	114	102	228	92	180	278.45	218	442.96	495	358	417	44	89	14	12	2
4704	13030	9448	701	588	344	28136	1151	2	649	0	22	60	0	94	277.11	91	434.43	339	333	328	20	45	0	0	3
14284	7718	4438	8	790	582	27870	638	312	1080	160	58	254	116	147	267.73	276	429.05	499	430	434	15	87	15	0	2
3898	13714	9950	977	292	174	28042	1296	8	556	1	14	56	4	65	270.78	95	423.68	278	266	278	14	35	0	0	3
12196	7958	5164	1	1620	1176	28320	561	149	758	32	188	206	86	239	300.65	159	454.74	469	261	405	78	121	10	16	12
14416	7674	4336	4	806	600	27872	656	332	1048	161	38	251	136	150	268.03	254	430.35	479	441	443	17	79	12	2	0
5064	12876	9270	679	552	334	28122	1099	10	658	0	24	61	6	100	280.69	97	435.4	357	329	358	19	40	0	2	1
12684	7896	4878	2	1448	1148	28260	556	187	760	38	188	182	117	233	292.86	168	451.44	467	255	459	59	106	12	12	13
13754	7800	4586	7	1000	754	27984	644	265	1002	122	82	223	118	192	276.46	214	442.97	444	404	453	29	83	6	8	2
13788	7774	4564	9	1020	762	27998	636	281	968	116	88	212	129	173	275.78	225	441.34	472	385	462	30	79	18	2	2
6326	12072	8560	471	714	450	28162	1000	17	772	7	38	85	8	123	285.94	126	444.86	411	386	357	15	54	0	2	2
12124	7710	5034	1	1848	1430	28452	530	146	634	38	284	178	74	270	304.05	125	456.03	483	196	407	72	92	12	22	19
14158	7634	4386	4	948	734	27950	625	336	953	154	86	246	140	160	273.4	251	436.31	499	380	435	26	75	17	4	1
14148	7764	4468	5	860	616	27892	626	285	1071	160	36	263	96	147	271.15	268	433.41	508	416	432	20	92	15	0	0
4510	13192	9608	810	498	268	28092	1191	8	617	1	16	69	3	77	274.24	91	428.8	338	305	300	14	49	0	0	3
4360	13352	9668	842	432	258	28088	1221	18	584	0	16	50	14	90	273.61	87	429.06	292	294	324	17	31	0	2	1
4022	13638	9888	937	316	178	28050	1294	4	596	1	8	59	3	80	271.62	84	425.2	262	301	281	13	42	0	0	3

SUPPLEMENTARY INFORMATION:Monte Carlo Atomistic Simulation and Machine Learning Analysis of Na-K Eutectic Alloy in Condensed Phases, D. Reitz and E. Blaisten-Barojas, George Mason University, Fairfax, VA 22030

14364	7674	4412	6	788	574	27872	664	332	1072	164	58	264	130	157	271.23	260	433.59	475	465	420	14	92	21	2	1
3932	13700	9950	973	298	158	28042	1312	8	587	1	4	54	6	73	272.33	85	425.93	261	290	282	12	40	0	0	1
4914	12906	9346	699	596	348	28136	1136	8	649	1	24	55	5	81	278.94	101	435.08	362	327	337	22	35	0	2	2
7648	11230	7836	345	824	552	28166	910	57	818	14	76	100	35	134	288.94	142	445.54	418	390	385	32	62	2	0	5
12168	8056	5176	4	1564	1154	28320	628	123	794	30	190	160	71	213	293.51	157	453.67	465	305	428	72	95	5	10	8
10614	7732	5586	2	2424	1950	28874	461	64	465	13	500	172	33	330	332.88	86	470.01	464	123	351	124	77	1	60	44
14040	7704	4504	6	936	678	27932	629	302	1018	155	64	248	109	148	273.74	260	438.36	511	396	434	23	78	17	6	1
14350	7698	4390	5	796	584	27860	643	321	1081	161	40	271	123	159	267.51	270	429.35	485	441	418	15	99	17	2	0
3890	13668	9946	1003	352	194	28056	1297	10	537	1	6	59	6	73	270.53	101	423.86	278	226	274	10	36	0	0	3
11586	7868	5254	1	1934	1544	28566	535	101	633	31	352	182	47	292	305.84	121	457.74	474	225	366	94	104	3	24	16
5726	12400	8872	505	710	438	28182	1043	9	748	0	34	54	6	101	283.04	102	438.22	406	392	369	22	30	0	2	0
11538	8054	5364	5	1850	1392	28478	578	107	716	19	248	168	62	252	303.89	146	454.79	475	245	380	88	93	5	32	17
4620	13176	9518	758	482	290	28108	1175	4	640	0	22	67	3	83	276.24	81	433.01	334	336	314	22	39	0	0	1
4562	13340	9606	824	342	196	28056	1216	7	657	1	10	68	4	79	275.84	105	429.67	308	321	308	15	47	0	0	1
4424	13310	9646	805	460	252	28098	1216	6	629	0	6	57	5	76	278.65	97	434.11	316	314	308	21	39	0	0	1
14160	7638	4422	3	922	698	27928	645	301	1015	143	88	244	125	144	271.65	243	436.65	470	416	448	40	86	18	0	2
4802	13170	9472	746	396	224	28074	1199	7	702	1	8	65	1	61	276.15	107	432.99	348	370	300	18	43	0	2	1
4022	13580	9862	946	366	220	28070	1295	6	570	1	20	61	3	64	270.26	91	422.61	298	275	265	9	42	0	0	4
14442	7616	4340	6	814	602	27870	629	343	1055	170	54	252	133	142	267.68	273	429.3	495	428	451	22	72	21	2	1
14298	7702	4394	5	810	614	27876	625	321	1087	164	56	252	122	149	268.16	286	429.86	502	425	447	16	84	12	2	1
5684	12304	8908	567	762	464	28186	1080	14	691	2	62	72	8	119	284.6	105	438.29	377	368	325	19	41	0	2	1
4924	12946	9334	689	564	340	28132	1146	6	665	0	24	54	3	107	277.93	102	434.39	343	354	330	11	37	0	0	1
13232	7900	4756	9	1172	892	28074	646	223	922	95	118	219	95	185	276.58	200	442.6	458	362	427	51	102	15	4	0
4154	13570	9810	924	318	190	28054	1270	11	598	2	10	65	6	72	271.86	87	424.87	289	293	282	13	39	0	2	3
4062	13572	9856	931	362	200	28058	1281	12	587	2	6	54	8	63	273.51	84	426.54	287	288	289	19	36	0	0	1
13462	7664	4576	4	1256	1000	28130	589	220	862	88	160	211	95	194	283.41	192	448.52	487	312	446	51	93	14	10	4
14344	7626	4352	4	860	652	27902	638	317	1038	161	62	268	116	154	271.96	272	435.69	499	424	413	19	91	20	4	2
4600	13150	9528	768	522	298	28112	1179	4	620	0	14	73	3	95	278.26	87	436.08	330	312	301	14	47	1	0	3
13664	7856	4628	5	994	756	27990	638	265	986	115	82	220	117	179	277.4	224	438.93	464	401	458	28	85	13	10	3
14144	7664	4460	3	904	670	27926	626	314	997	145	82	237	125	165	273.85	235	438.33	466	404	467	29	80	20	2	2
13686	7554	4516	4	1254	940	28086	632	260	849	101	124	215	124	188	277.66	194	445.22	474	327	433	41	99	17	12	4
13612	7468	4566	1	1384	972	28130	571	277	761	110	108	217	132	199	283.25	194	447.54	503	273	444	49	82	11	20	2
5418	12560	9088	578	676	380	28150	1072	9	712	0	26	74	7	91	280.39	114	437.26	399	373	338	16	44	0	2	3
3978	13634	9898	954	338	198	28060	1285	6	584	1	12	60	2	70	270.53	84	423.36	284	276	275	17	41	0	2	1
6110	12174	8644	507	732	474	28176	1027	20	727	3	38	72	10	110	285.44	125	443.2	405	369	348	24	45	0	4	2
11922	7810	5170	2	1868	1394	28438	530	139	678	46	248	208	70	277	300.73	138	450.42	477	227	381	75	109	4	24	20

SUPPLEMENTARY INFORMATION:Monte Carlo Atomistic Simulation and Machine Learning Analysis of Na-K Eutectic Alloy in Condensed Phases, D. Reitz and E. Blaisten-Barojas, George Mason University, Fairfax, VA 22030

13926	7718	4528	5	982	730	27974	625	266	995	129	80	243	112	163	275.98	222	440.65	490	402	438	33	92	11	10	1
3786	13812	10040	1020	258	132	28030	1327	6	565	1	2	55	4	62	271.29	88	423.48	262	265	274	10	34	0	0	4
13494	7838	4700	5	1110	802	28038	627	244	949	111	92	220	107	182	277.25	214	444	464	375	455	40	90	13	2	3
13528	7730	4660	1	1208	852	28074	614	237	900	91	92	210	114	184	283.51	202	446.48	481	352	456	35	97	10	4	8
12900	7908	4850	2	1372	1016	28178	620	198	860	70	120	190	96	198	280.6	198	445.25	492	328	426	39	101	9	12	11
11750	7818	5160	1	1954	1528	28538	509	104	604	19	288	166	53	268	307.46	125	456.45	497	183	399	103	90	10	36	12
13156	7798	4694	2	1336	1034	28168	619	229	816	90	142	197	102	201	283.68	178	446.97	485	307	430	47	73	14	6	4
6750	11672	8342	387	874	516	28206	955	16	788	3	48	84	9	121	288.33	120	443.42	422	401	372	31	48	1	4	5
11828	7916	5252	0	1812	1356	28446	577	125	669	22	258	170	62	256	303.62	130	455.28	468	244	396	77	93	9	24	17
14382	7560	4358	4	900	642	27902	628	338	1009	153	56	266	142	170	273.57	235	438.52	478	414	431	19	96	16	4	2
4606	13172	9502	762	510	312	28120	1173	3	643	1	18	65	0	75	276.93	106	433.5	357	311	305	17	37	0	0	3
14540	7618	4318	2	768	562	27852	701	337	1067	162	44	243	134	151	270.6	257	432.17	445	481	432	17	70	18	2	1
13846	7706	4494	4	1046	818	28020	613	270	972	119	92	230	114	174	277.45	240	441.76	500	376	437	31	93	16	14	3
13886	7858	4602	9	888	634	27920	630	285	1056	132	52	239	114	179	277.21	231	439.67	456	430	455	27	89	11	0	0
12296	7914	4980	0	1616	1292	28342	596	168	729	56	218	171	74	219	294.96	151	453.6	477	266	409	82	79	15	24	8
13554	7906	4716	5	984	732	27988	615	255	1022	121	90	242	105	159	275.2	252	442.72	489	380	442	42	107	14	6	2
6038	12276	8714	550	660	418	28136	1062	30	728	7	30	69	15	114	281.79	134	439.18	376	358	350	18	40	4	0	0
11990	7758	5058	1	1856	1496	28502	570	117	632	27	300	176	62	262	300.17	118	456.83	459	242	397	93	89	9	38	13
14470	7642	4384	7	752	544	27848	639	333	1087	165	50	259	129	159	267.66	261	429.5	477	454	445	12	82	15	6	1
5616	12350	8882	533	790	510	28208	1024	8	685	1	60	76	1	108	284.38	119	440.01	432	338	330	21	49	0	0	1
14294	7644	4350	3	874	674	27906	653	299	1027	151	68	255	119	163	271.08	261	435.21	478	420	422	19	88	15	2	1
4568	13230	9562	786	458	268	28104	1177	7	611	1	16	66	4	91	276.82	102	432.83	339	307	314	9	40	0	2	2
14442	7762	4376	3	674	526	27828	655	328	1136	176	46	262	111	149	268.12	285	430.25	473	461	442	10	78	18	2	3
14212	7786	4448	5	778	598	27880	631	308	1100	161	52	255	114	145	269.18	279	431.55	499	430	442	20	86	15	4	1
3842	13690	9950	962	370	212	28068	1286	6	524	0	4	54	4	70	273.93	73	427.05	287	255	281	16	35	0	0	0
11578	7916	5358	0	1944	1416	28500	546	106	640	24	266	167	43	260	305.85	128	457.81	511	222	388	71	92	12	22	17
13426	7586	4576	3	1326	1044	28142	594	240	836	98	168	217	97	198	283.9	196	446.89	490	324	426	50	95	21	16	6
4432	13322	9680	833	410	214	28070	1204	10	623	1	12	55	2	67	274.1	97	430.32	323	298	321	20	38	0	0	2
4048	13446	9830	916	492	268	28094	1274	8	545	4	10	52	2	76	273.83	84	427.84	292	264	286	12	35	0	0	4
4520	13310	9604	815	382	236	28078	1186	7	610	1	24	62	2	83	274.07	88	428.22	314	305	329	18	37	0	2	3
7088	11342	8084	384	994	640	28236	922	38	728	9	82	89	26	157	290.86	109	445.59	423	366	370	25	50	0	6	2
11280	7900	5424	1	2090	1580	28638	542	84	555	23	318	184	31	297	320.6	105	464.17	446	182	354	103	97	4	42	27
14228	7644	4398	5	890	672	27906	671	318	1017	159	72	267	126	152	269.11	262	432.31	467	425	411	27	92	20	2	2
5068	12920	9304	677	498	290	28104	1133	8	688	1	24	61	6	83	276.45	96	432.15	368	362	336	13	40	0	0	1
14368	7632	4330	9	848	652	27898	660	315	1045	154	68	248	126	146	272.6	251	436.49	473	446	438	24	85	15	0	4
5110	12870	9260	675	524	318	28112	1126	10	690	2	30	64	7	87	278.74	89	434.14	355	356	341	21	49	0	0	1

14272	7668	4438	4	812	612	27888	641	328	1014	158	86	250	130	165	274.18	233	437.9	454	418	452	27	77	14	0	0
4114	13590	9854	923	310	168	28044	1256	9	600	2	8	52	5	71	270.81	83	422.58	285	282	316	14	39	0	0	2
6160	11974	8612	496	868	536	28208	1047	12	741	4	50	66	5	124	284.18	109	440.69	392	394	335	23	39	0	8	1
3958	13702	9920	946	288	170	28048	1292	5	589	2	10	55	2	55	270.12	87	423.45	289	295	287	14	32	0	0	4
13724	7572	4480	6	1224	936	28064	611	267	856	115	116	210	104	197	276.69	194	443.24	484	333	457	24	77	25	12	5
4138	13488	9796	902	416	234	28080	1261	6	582	2	8	73	3	71	272.65	85	427.25	308	283	267	16	48	0	0	1
5510	12590	9022	609	610	370	28128	1103	15	723	2	24	71	9	102	278.96	114	435.55	352	379	339	21	51	0	2	4
14416	7634	4312	4	828	634	27880	652	321	1030	158	54	262	126	146	271.97	249	436.52	490	436	419	19	89	15	2	3
4838	12822	9338	673	728	426	28184	1138	6	607	1	32	49	3	80	278.78	87	435.48	380	330	321	23	30	0	0	3
7718	10916	7736	332	1114	688	28240	907	50	748	14	64	107	27	151	289.53	136	444.81	427	352	354	35	60	1	4	5
14362	7530	4312	5	916	710	27928	627	328	991	156	94	250	136	145	273.05	262	435.87	506	393	433	28	82	17	4	2
10378	8026	5902	0	2362	1704	28802	524	52	482	6	366	149	31	346	328.8	72	470.41	455	161	353	98	81	2	58	29
11922	7886	5090	0	1810	1446	28454	541	117	689	28	272	182	54	269	304.69	141	456.03	467	231	419	70	99	9	26	20
4708	13206	9512	760	408	232	28076	1183	6	671	1	10	71	4	83	276.44	89	432.96	324	349	312	19	47	0	0	1
4160	13540	9800	889	344	204	28062	1256	8	613	1	14	57	3	72	270.98	97	424.41	298	303	296	12	41	0	0	3
3908	13598	9922	963	400	228	28074	1291	8	551	1	18	56	4	72	269.69	90	422.72	276	263	279	17	35	0	0	3
4110	13508	9828	910	392	220	28074	1259	4	579	0	14	59	3	72	274.85	84	428.05	302	286	287	16	42	0	2	0
4162	13486	9790	884	388	230	28078	1230	7	594	1	22	63	5	71	272.79	95	426.17	320	285	299	13	51	0	0	2
11868	7910	5202	5	1804	1376	28446	552	124	674	30	264	173	63	273	300.76	116	455.62	483	245	401	76	92	8	20	9
11784	7928	5344	0	1780	1318	28462	521	105	712	24	278	179	54	249	307.9	157	457.51	527	239	391	73	101	4	30	16
3650	13832	10066	1022	316	182	28052	1346	8	524	1	6	62	6	64	271.87	84	422.86	257	257	247	16	37	0	0	2
14244	7734	4412	2	824	624	27894	643	326	1048	161	52	245	128	148	270.61	264	433.28	492	423	446	20	77	18	4	0
4570	13284	9588	801	404	226	28082	1176	3	643	0	10	75	2	79	276.55	111	430.18	344	307	311	12	47	0	0	2
14258	7668	4376	2	866	672	27914	595	330	1012	152	62	259	128	157	271.75	271	436.34	520	388	444	15	88	24	12	4
4828	13120	9438	747	422	258	28094	1170	10	667	1	28	62	7	78	277.67	113	434.14	354	338	316	13	41	0	0	1
10882	7948	5500	1	2156	1762	28722	514	79	535	14	416	151	46	292	318.89	99	463.92	486	172	376	111	82	2	52	26
11866	7834	5154	1	1862	1472	28528	533	128	631	24	294	163	68	268	304.72	123	458.73	503	219	396	80	91	11	44	21
4048	13606	9868	932	326	190	28052	1273	10	590	2	14	53	7	78	271.37	87	425.53	271	282	301	15	36	0	0	2
14498	7640	4360	2	760	544	27844	637	352	1090	164	36	258	135	139	270.03	277	432.02	493	454	446	17	87	21	6	2
4390	13348	9676	862	414	234	28076	1261	13	612	1	12	52	9	60	273.32	95	427.38	313	322	286	16	39	0	2	3
13496	7816	4650	4	1128	850	28046	644	241	956	109	100	227	102	188	275.23	211	440.49	477	363	423	31	100	12	6	1
4656	13070	9508	767	562	306	28116	1195	6	631	0	14	54	3	82	276.3	96	431.41	341	325	303	15	40	0	0	1
3924	13634	9944	955	358	188	28056	1304	2	566	0	6	62	2	62	272.02	75	424.75	282	293	265	19	48	0	2	0
10988	7668	5512	0	2298	1790	28762	484	85	493	13	442	153	47	306	321.41	82	463.58	467	160	393	110	77	5	56	40
14418	7662	4366	6	800	578	27866	652	325	1085	160	42	255	124	137	270.99	291	432.91	508	427	427	14	88	18	0	0
10712	7916	5672	1	2278	1712	28724	481	70	515	18	388	179	36	310	323.63	97	464.47	489	163	350	109	92	3	46	32

SUPPLEMENTARY INFORMATION:Monte Carlo Atomistic Simulation and Machine Learning Analysis of Na-K Eutectic Alloy in Condensed Phases, D. Reitz and E. Blaisten-Barojas, George Mason University, Fairfax, VA 22030

4072	13624	9882	948	294	158	28038	1286	9	597	1	8	69	6	75	270.7	95	424.75	267	283	280	18	46	0	0	0
11786	7826	5206	1	1926	1458	28508	543	127	619	43	272	194	53	292	309.06	115	457.07	480	214	367	75	98	6	34	14
4928	13066	9398	704	420	248	28082	1122	6	703	1	20	72	4	77	279.3	117	434.55	363	347	340	19	48	0	0	2
5530	12508	9006	612	682	396	28146	1106	15	671	3	24	68	10	102	280.61	97	437.21	352	348	339	25	41	0	0	2
4326	13392	9686	866	406	252	28086	1241	8	585	2	24	59	4	71	273.94	86	428.31	307	297	299	17	39	0	0	3
10490	7820	5834	1	2482	1760	28832	506	48	457	7	390	170	23	311	336.7	86	473.01	479	141	323	129	73	4	48	23
13846	7654	4458	2	1098	862	28036	607	265	928	104	104	215	117	178	277.8	203	445.34	478	352	469	40	97	21	14	3
14138	7738	4478	4	860	632	27910	611	315	1041	147	62	252	126	177	274.88	260	437.5	472	395	460	22	89	19	2	0
14474	7614	4344	5	820	572	27852	649	340	1052	173	26	264	131	156	268.01	278	430	472	427	434	14	83	14	2	3
4802	12894	9400	678	662	372	28156	1147	2	646	0	22	56	2	93	279.13	90	435.32	358	358	322	14	35	0	4	5
11158	7952	5542	1	2060	1524	28596	537	98	583	26	328	168	45	287	315.55	126	458.75	503	185	355	83	79	4	30	21
7594	11138	7742	291	980	702	28256	891	35	798	8	96	83	20	139	289.85	144	446.42	460	389	383	29	53	3	4	4
7790	10960	7592	262	1042	766	28264	865	40	791	11	112	92	20	139	289.57	129	447.14	467	408	380	38	52	1	2	4
11396	7800	5306	0	2128	1628	28622	528	103	572	20	332	159	61	287	304.82	106	458.55	481	185	396	94	79	5	30	19
4124	13500	9822	939	384	220	28072	1283	7	587	1	22	60	3	57	275.86	99	429.94	307	274	270	16	44	0	0	2
4574	13246	9574	818	432	242	28076	1200	10	640	1	8	60	4	82	274.68	106	429.84	324	307	315	11	41	1	0	2
5356	12732	9132	613	554	326	28122	1098	5	750	0	22	68	2	70	278.13	132	434.47	393	379	340	24	43	0	0	2
11850	7834	5180	6	1902	1428	28472	537	123	660	22	242	168	70	254	302.26	147	452.58	523	226	406	69	92	11	36	12
4612	13262	9582	806	388	212	28068	1211	6	652	2	12	64	3	73	273.09	107	427.6	320	325	311	15	43	0	0	2
4512	13260	9626	808	442	234	28086	1194	4	641	0	12	64	3	87	277.09	95	432.57	330	313	306	11	46	0	0	1
14296	7736	4440	7	760	566	27852	650	329	1077	158	48	259	126	154	266.96	254	429.02	474	449	437	16	90	22	6	1
14188	7740	4394	9	866	666	27908	662	315	1030	149	54	253	135	149	271.04	271	434.1	499	431	419	15	91	13	0	0
4352	13462	9728	891	312	176	28042	1254	10	614	1	12	67	6	80	274.1	91	429.47	280	299	292	15	45	0	0	3
13578	7936	4642	9	948	778	28002	600	257	1029	105	112	225	122	173	276.86	257	439.36	485	379	462	39	95	10	8	2
13832	7828	4608	7	912	676	27936	638	267	1057	137	76	234	96	177	275	246	438.92	449	409	457	31	86	10	4	1
4562	13104	9532	768	570	336	28134	1181	5	609	0	26	57	4	83	278.01	84	433.57	347	315	304	17	31	0	4	3
4154	13476	9804	895	400	222	28070	1235	9	565	1	14	57	5	71	275.61	82	427.66	297	266	308	18	40	0	0	6
12102	7786	5104	1	1870	1344	28430	523	142	673	38	202	172	72	264	302.83	152	457.63	521	220	410	66	82	6	20	7
4030	13530	9856	920	418	232	28076	1262	5	567	2	10	61	2	68	275.16	94	427.76	296	275	291	17	39	0	0	3
4174	13448	9766	922	426	250	28078	1278	10	572	2	12	54	6	73	272.32	83	425.35	276	278	291	22	34	0	2	1
4314	13354	9720	850	450	236	28080	1237	7	596	1	6	68	5	77	273.64	80	428.39	308	316	287	16	43	0	0	2
12288	7870	5060	0	1660	1254	28370	578	136	714	39	206	186	67	243	298.45	142	454.35	475	240	400	68	103	12	30	16
4808	13080	9446	742	472	272	28100	1174	4	664	0	22	60	3	80	276.27	104	433.61	336	334	326	18	40	0	0	2
14346	7644	4352	3	844	638	27884	622	355	1000	156	60	254	150	160	270.32	270	432.89	487	389	451	19	88	26	0	0
4944	12966	9380	707	514	282	28104	1140	7	678	0	18	69	3	78	279.18	107	436.66	369	333	320	16	48	0	0	2
5964	12080	8712	493	846	536	28210	1014	15	701	3	66	83	8	119	286.79	98	441.7	424	363	330	19	60	1	6	3

SUPPLEMENTARY INFORMATION:Monte Carlo Atomistic Simulation and Machine Learning Analysis of Na-K Eutectic Alloy in Condensed Phases, D. Reitz and E. Blaisten-Barojas, George Mason University, Fairfax, VA 22030

4118	13570	9848	949	320	178	28048	1266	7	613	1	14	62	3	65	272.88	110	426.05	299	269	284	13	45	0	0	3
4762	13084	9434	737	520	306	28120	1160	9	645	1	14	54	7	87	277.63	102	433.11	348	316	331	15	32	0	0	1
7016	11406	8106	379	990	642	28238	945	33	737	6	72	91	21	142	288.97	123	444.21	413	374	362	32	50	2	4	5
5406	12594	9096	600	644	372	28146	1126	6	723	0	32	71	3	91	280.18	110	436.64	373	394	314	18	50	0	2	2
3838	13674	9948	971	360	226	28072	1308	8	533	1	26	56	6	76	270.93	79	424.66	268	266	268	15	42	0	0	2
11648	7706	5304	1	2108	1478	28520	528	115	581	25	246	172	63	276	309.18	100	460	468	199	408	88	88	8	30	22
5760	12496	8904	538	564	364	28134	1039	13	743	0	42	66	10	99	280.81	124	438.04	415	395	356	14	40	0	4	0
11264	7934	5412	1	2036	1582	28606	542	100	618	23	336	144	48	280	308.08	120	457.5	514	217	384	81	74	4	42	13
11876	7896	5096	4	1806	1484	28496	516	123	635	42	300	190	49	258	302.83	157	458.59	528	196	374	91	90	10	38	9
4176	13562	9802	895	322	186	28056	1277	5	606	1	8	65	4	66	273.66	100	427.35	295	310	276	14	45	0	0	2
4522	13354	9636	836	342	188	28050	1222	11	635	1	8	66	7	72	272.37	95	425.57	305	319	311	17	50	0	0	2
12478	7872	4918	1	1596	1246	28328	580	158	736	48	200	181	73	233	291.92	164	451.89	507	259	409	64	95	14	16	2
11026	7976	5512	0	2094	1652	28686	501	77	530	17	372	181	39	327	323.13	94	465.68	464	161	359	102	97	5	48	25
4924	12952	9358	725	546	318	28122	1174	2	664	0	24	53	2	94	279.16	87	435.29	331	341	330	12	44	0	0	3
3838	13726	9970	975	320	190	28058	1314	8	553	2	14	67	5	61	270.72	90	423.54	276	271	258	16	41	0	0	4
4464	13352	9640	841	382	218	28064	1212	9	633	1	8	58	3	75	274.69	101	429.41	312	309	314	14	37	0	0	5
4148	13562	9796	886	350	206	28066	1264	9	606	2	4	56	5	69	270.63	76	423.88	296	309	293	16	40	0	0	1
11314	7898	5450	1	2060	1534	28618	547	88	585	9	334	155	44	293	320.98	120	460.39	456	201	376	88	82	12	24	31
13476	7798	4678	4	1142	848	28058	601	250	914	98	114	216	120	181	277.1	209	445.68	491	337	455	40	96	14	2	3
4844	12910	9368	727	624	370	28148	1178	13	635	1	30	64	11	92	277.1	93	434.23	341	340	301	16	38	0	2	4
4718	13012	9460	752	582	332	28130	1170	4	647	0	26	78	3	87	277.6	102	433.57	354	321	288	18	55	0	0	1
5308	12756	9192	608	544	298	28118	1088	12	715	3	18	72	6	100	281.21	116	437.88	364	365	349	19	48	1	2	2
5186	12870	9234	648	508	294	28106	1105	7	737	1	14	69	2	84	282.74	134	439.89	372	361	334	21	50	0	0	3
4702	13092	9486	762	518	292	28106	1172	6	636	0	16	60	4	73	276.66	103	430.91	358	316	317	13	42	0	0	3
4050	13638	9860	940	314	184	28050	1298	4	594	1	4	60	3	61	271.64	92	424.17	279	295	282	15	36	0	0	3
4716	13166	9500	736	444	252	28092	1176	0	659	0	14	66	0	74	277.88	94	433.26	340	334	320	18	45	0	0	3
11802	7918	5296	0	1832	1332	28450	555	115	687	28	254	174	64	263	299.49	126	454	487	226	411	70	111	4	14	11
5218	12712	9164	647	646	384	28150	1121	6	689	3	26	71	3	89	280.17	100	437.47	371	344	320	21	46	0	0	3
14376	7642	4336	8	844	640	27896	634	317	1070	163	52	249	114	148	267.98	280	429.75	506	442	442	15	78	17	4	1
5526	12564	9024	576	622	376	28150	1071	5	726	1	34	63	4	100	280.97	113	438.16	381	375	355	21	38	0	4	2
14534	7550	4326	3	808	580	27856	617	345	1053	165	56	260	134	150	271.4	265	434.57	493	404	452	20	84	22	2	0
4056	13606	9856	958	324	196	28054	1308	10	581	1	16	60	4	63	273.66	87	425.78	280	287	266	16	41	0	0	1
4390	13364	9640	868	414	260	28086	1261	7	622	1	18	65	3	84	275.42	109	428.58	295	316	277	13	41	0	0	1
13538	7606	4552	0	1268	986	28112	613	251	875	78	154	212	125	235	281.3	172	444.19	429	340	443	45	110	17	8	2
14308	7704	4404	5	806	598	27870	601	333	1077	176	46	269	118	162	267.7	267	429.24	500	422	445	14	83	18	4	2
4004	13640	9900	970	320	176	28046	1305	14	569	3	6	60	9	71	271.62	87	423.99	270	275	269	14	40	0	0	2

SUPPLEMENTARY INFORMATION:Monte Carlo Atomistic Simulation and Machine Learning Analysis of Na-K Eutectic Alloy in Condensed Phases, D. Reitz and E. Blaisten-Barojas, George Mason University, Fairfax, VA 22030

11462	7952	5356	2	1936	1494	28542	545	114	622	26	308	159	62	275	305.73	134	456.27	499	217	378	84	80	2	32	15
3856	13734	9976	992	302	170	28046	1314	5	555	1	8	55	4	56	271.08	97	424.27	266	266	285	19	31	0	0	3
7020	11470	8136	363	932	596	28226	920	29	758	6	68	70	20	137	287.66	114	444.23	423	365	411	29	44	1	4	2
4462	13348	9622	838	398	240	28084	1202	12	632	0	14	56	8	77	275.01	107	431.38	335	302	312	11	27	0	0	1
6664	11678	8328	409	922	574	28222	963	21	761	3	50	96	16	119	286.64	122	444.39	435	401	342	33	63	0	4	3
11418	7912	5396	1	1928	1514	28556	503	87	618	13	340	173	45	255	307.46	135	455.89	541	181	386	92	98	7	48	16
4868	13004	9426	733	516	272	28096	1134	9	652	0	10	59	8	74	277.71	106	433.14	356	311	350	20	44	0	0	3
7278	11238	7922	336	1026	700	28258	882	30	753	9	86	94	18	147	288.67	131	446.29	457	360	374	30	56	0	8	4
4476	13294	9624	776	442	248	28096	1205	4	642	0	10	67	4	77	277.23	101	432.02	346	353	292	9	44	0	2	1
14140	7690	4478	3	876	648	27912	638	314	1035	144	76	259	136	153	274.13	244	439.43	496	420	417	26	104	13	4	0
10914	7840	5562	0	2228	1730	28738	483	70	530	15	402	166	35	305	322.18	98	465.65	501	173	357	111	75	4	52	27
5878	12334	8824	535	660	406	28144	1052	21	721	1	42	71	13	92	283.31	112	440.02	409	380	340	20	46	0	0	3
4232	13550	9780	877	304	176	28050	1237	3	616	0	8	63	2	59	273.5	109	427.16	322	295	295	16	45	0	0	1
14402	7614	4390	3	810	582	27856	621	338	1062	170	56	289	128	143	269.63	277	432.63	507	417	413	19	109	18	2	3
11102	7848	5442	2	2184	1678	28646	521	90	517	14	340	176	49	287	312.94	95	457.56	467	174	368	108	82	4	52	26
4198	13516	9790	909	344	198	28060	1282	10	596	1	14	55	6	67	272.78	84	426.79	286	308	285	16	36	0	0	2
4448	13368	9646	838	378	220	28070	1233	9	634	0	8	53	4	84	275.58	89	429.32	305	319	305	9	34	0	2	2
10238	7842	5800	0	2492	1956	28904	474	50	418	6	518	149	27	310	330.79	74	472.65	476	123	351	145	73	1	54	38
14388	7748	4406	4	710	544	27856	655	326	1106	160	58	266	129	139	268.77	292	429.26	499	458	422	15	94	19	2	0
4092	13572	9866	934	336	176	28050	1298	6	600	0	8	70	2	77	271.66	100	425.46	275	304	264	11	44	0	0	1
13598	7874	4678	6	984	752	27996	647	276	996	111	104	214	137	171	275.04	226	439.74	463	411	458	34	85	16	6	2
6846	11528	8204	341	984	618	28240	943	19	786	5	56	71	9	143	289.2	128	445.24	432	417	373	24	50	0	4	1
13132	8030	4826	6	1116	860	28082	642	228	950	93	106	204	106	197	278.47	217	443.47	465	368	448	33	92	13	12	2
4180	13496	9774	900	372	228	28070	1273	6	615	1	20	60	3	81	273.28	83	426.85	276	317	284	18	34	0	0	1
4032	13676	9888	970	272	162	28040	1303	8	604	3	8	58	4	61	270.45	103	423.63	277	282	278	15	41	0	2	2
11616	8020	5160	0	1882	1532	28522	582	104	636	20	288	160	63	252	305.08	137	457.54	474	204	401	86	97	3	22	18
13704	7848	4646	13	948	720	27968	651	259	1006	121	98	238	106	158	275.7	236	441.33	469	399	428	39	92	14	4	3
4184	13506	9804	918	352	196	28056	1306	8	602	1	14	71	3	64	272.74	90	427.05	292	311	247	13	46	0	0	1
10464	7890	5726	1	2382	1880	28882	491	60	467	7	454	164	31	307	333.27	82	473.49	461	148	351	143	70	4	78	39
14388	7606	4364	5	834	618	27878	614	330	1024	165	68	266	124	156	271.4	258	433.78	508	420	431	15	89	22	0	1
4388	13294	9640	836	484	286	28112	1226	8	615	0	18	57	7	78	275.32	99	430.44	316	310	301	19	39	0	2	1
11784	7860	5166	4	1922	1476	28506	545	140	597	30	272	184	77	272	308.37	113	455.4	444	200	408	93	88	13	26	23
12000	8124	5184	8	1604	1228	28354	589	127	774	29	198	177	65	245	293.43	160	451.37	482	282	404	69	97	6	12	5
4912	13030	9378	721	472	284	28100	1175	7	690	1	24	62	6	78	275.37	111	431.95	337	368	321	21	37	0	0	1
12098	7940	5200	3	1670	1226	28372	579	143	738	55	210	188	68	237	293.53	144	451.58	472	278	414	78	99	3	26	9
13826	7790	4606	3	938	698	27954	652	272	1022	143	94	245	93	157	275.18	237	439.23	492	428	415	26	82	14	2	2

SUPPLEMENTARY INFORMATION:Monte Carlo Atomistic Simulation and Machine Learning Analysis of Na-K Eutectic Alloy in Condensed Phases, D. Reitz and E. Blaisten-Barojas, George Mason University, Fairfax, VA 22030

7160	11410	8040	339	932	612	28224	917	30	829	12	66	79	17	139	288.1	125	444.91	421	405	402	34	49	0	4	1
11712	7704	5294	2	2038	1464	28522	544	137	595	26	278	164	69	267	310.67	115	456.9	455	206	400	103	83	9	30	22
14450	7670	4346	3	748	576	27850	642	336	1071	170	58	265	126	164	268.57	280	430.49	472	423	435	10	80	15	2	3
7846	10772	7564	310	1170	812	28284	865	50	763	15	120	105	21	164	290.67	144	445.38	451	345	358	35	59	5	0	5
4714	13138	9496	740	474	264	28098	1172	7	686	1	12	59	2	94	276	104	432.33	336	350	318	11	35	0	0	1
5622	12560	9030	592	558	312	28112	1088	14	744	0	30	65	9	89	281.08	117	439.21	373	379	354	16	51	0	0	6
3900	13606	9924	974	410	226	28074	1326	4	555	0	8	47	3	68	270.51	72	423.64	261	277	274	16	36	0	0	3
4090	13564	9848	922	356	194	28058	1269	5	588	0	6	59	4	67	273.23	97	427	294	277	291	12	39	0	0	4
11754	7820	5238	0	1966	1442	28494	546	144	586	31	234	163	80	279	304.06	123	454.86	454	192	416	97	82	7	38	10
4038	13604	9870	957	334	194	28052	1282	8	578	2	10	62	4	67	270.62	95	423.34	283	270	286	13	37	0	2	4
11064	7810	5514	1	2238	1670	28702	523	71	568	16	364	175	31	269	321.43	128	465.14	502	163	347	106	95	1	34	33
14464	7538	4296	4	880	650	27888	619	336	1017	161	54	252	132	157	271.39	265	436.5	497	401	445	17	83	19	6	2
4580	13206	9546	809	486	278	28106	1223	4	641	1	10	61	3	79	277.31	101	433.51	318	311	299	15	42	0	0	3
4690	13032	9438	709	608	358	28142	1161	3	620	0	16	62	2	91	278.04	96	434.4	356	326	306	13	40	0	0	4
3858	13738	9966	1008	292	178	28048	1307	5	549	1	16	53	4	75	269.74	83	422.35	259	249	288	11	33	0	0	3
13260	7892	4766	8	1186	880	28104	625	212	911	64	114	191	107	194	282.22	199	446.87	485	366	449	35	101	15	6	3
12764	7846	4762	0	1462	1210	28262	635	169	784	57	202	171	81	202	284.71	161	448.18	482	294	428	54	94	17	12	10
12514	7852	5012	2	1552	1152	28298	611	153	774	47	208	184	75	224	294.64	157	451.71	470	285	407	65	99	11	6	10
14126	7706	4460	6	880	668	27924	665	291	1044	151	82	253	103	166	272.31	239	438.2	454	430	430	27	92	16	2	0
14422	7590	4346	3	832	616	27870	633	338	1036	160	62	272	129	149	271.18	258	434.45	481	413	427	27	102	23	2	2
5242	12612	9146	602	704	420	28168	1105	6	678	1	42	55	2	89	281.82	118	438.02	374	366	343	25	30	0	2	3
3912	13612	9944	971	388	200	28062	1292	6	579	1	6	66	3	83	271.66	88	424.8	278	260	266	9	52	0	0	1
14346	7640	4386	8	852	610	27882	674	329	1031	162	48	259	133	167	267.18	244	429.76	458	440	421	13	89	14	0	2
11956	7708	5120	1	1956	1460	28496	574	139	624	20	270	157	83	249	299.21	112	454.55	462	230	413	92	84	13	24	20
14370	7638	4368	9	822	616	27878	660	341	1040	170	60	269	127	156	270.78	247	432.16	471	440	416	21	92	19	4	1
11168	7820	5522	0	2146	1592	28644	527	86	561	10	350	151	53	292	313.03	93	461.31	439	180	420	106	84	6	44	25
12574	7644	4806	1	1730	1358	28364	590	170	688	58	228	188	80	229	291.78	141	450.41	517	255	386	61	87	18	22	7
4712	13042	9416	720	600	364	28152	1177	2	623	0	18	56	2	87	278.08	88	433.7	345	339	308	20	31	0	0	2
4188	13514	9810	916	356	184	28054	1276	10	594	1	2	50	8	68	272.4	99	425.97	281	286	301	16	34	0	0	2
4806	13052	9426	741	514	296	28110	1152	8	660	0	16	59	4	72	275.85	122	433.04	372	320	324	13	46	0	0	3
4096	13458	9806	894	458	258	28088	1259	6	564	1	12	67	3	67	272.74	84	426.26	311	280	271	17	44	0	0	3
5308	12780	9198	618	516	284	28106	1109	11	751	1	20	69	6	79	278.18	118	436.57	391	386	323	14	49	0	0	3
10922	7980	5634	0	2166	1590	28670	519	87	552	17	344	166	48	292	314.13	100	467	485	170	379	92	95	6	30	25
4034	13570	9888	946	368	190	28056	1303	6	600	1	6	65	4	60	270.28	101	422.75	281	291	267	16	43	0	0	4
13476	8004	4800	5	930	684	27990	630	245	1036	120	90	253	89	198	276.7	230	442.86	464	415	416	25	111	19	6	3
11418	7840	5414	1	2036	1510	28568	531	111	600	24	312	168	58	260	313.26	127	458.43	524	205	380	78	81	2	36	21

5284	12668	9126	636	644	392	28148	1107	13	666	3	34	75	6	114	280.89	103	435.93	354	337	321	15	47	0	0	5
14312	7760	4390	6	758	594	27872	652	335	1045	158	58	247	128	144	270.85	261	434.77	484	444	446	19	75	22	0	1
14172	7680	4462	7	870	646	27910	645	319	1048	152	76	258	129	144	271.08	258	434.32	515	438	414	17	97	15	4	1
4958	12946	9318	706	536	336	28126	1132	8	666	0	30	61	6	83	280.91	98	435.57	363	342	336	17	38	0	2	4
11252	8002	5516	0	2020	1472	28584	558	91	600	15	288	161	48	275	311.38	100	461.23	474	213	379	91	93	8	32	21
11052	7820	5560	1	2188	1660	28734	537	74	532	17	398	170	42	280	323	115	466.02	493	173	351	113	82	3	56	29
4824	13176	9452	748	382	226	28072	1187	9	688	1	12	66	6	84	275.08	93	431.26	327	355	319	12	46	0	0	1
14132	7732	4410	8	866	696	27920	615	309	1009	144	78	245	124	152	272.52	252	436.63	497	396	452	25	87	15	6	4
11122	7742	5434	1	2206	1748	28732	502	81	523	17	422	158	43	299	323.71	91	467.74	470	180	387	109	79	7	48	29
13766	7660	4516	2	1126	860	28050	657	243	907	100	114	219	112	197	277.1	183	445.21	453	372	434	29	108	18	8	3
11938	7964	5176	1	1710	1336	28404	565	147	721	36	254	164	78	245	300.26	162	452.84	516	246	402	64	95	9	24	9
4016	13722	9910	952	238	138	28030	1284	5	593	0	6	59	4	57	271.44	90	424.57	281	297	296	15	37	0	0	4
4014	13518	9858	925	430	244	28080	1281	7	550	0	16	53	4	77	274.1	95	427.58	281	273	276	17	36	0	0	3
6516	11952	8462	442	724	460	28162	992	33	762	9	48	80	17	110	284.54	118	442.18	421	409	363	22	46	1	0	2
14266	7784	4452	8	730	564	27860	639	320	1075	169	60	270	117	144	269.89	283	431.99	501	427	426	15	90	17	4	1
4244	13470	9768	879	362	204	28062	1232	5	622	1	12	59	3	72	272.14	90	425.83	309	293	312	11	40	0	2	3
10782	7690	5566	0	2414	1868	28838	495	68	441	9	456	158	47	308	330.71	77	471.25	467	140	367	124	67	2	56	38
13998	7770	4524	11	912	668	27936	642	279	1046	120	54	246	131	180	277.37	225	440.64	453	426	444	25	103	13	10	2
14128	7754	4434	9	856	672	27918	641	311	1024	146	66	232	128	158	269.72	267	433.08	480	420	457	22	74	17	8	1
11104	8098	5484	2	2016	1558	28610	544	74	618	15	322	170	36	284	310.89	114	460.63	474	183	388	86	99	10	26	25
4218	13464	9778	899	384	210	28064	1270	11	604	3	10	54	5	76	274.26	92	428.03	290	305	293	10	31	0	0	2
13252	7732	4724	2	1300	978	28142	598	247	844	91	134	205	113	215	280.98	200	446.13	485	316	435	43	85	11	22	0
12054	7934	5082	0	1706	1358	28418	553	149	700	39	270	157	77	238	292.13	144	453.7	513	243	427	73	83	13	10	8
4730	13140	9474	737	470	272	28098	1156	4	672	0	12	66	1	95	277.6	116	433.98	345	328	321	10	43	0	0	1
14518	7632	4296	3	736	596	27854	614	331	1084	177	68	278	111	147	267.54	274	429.32	499	427	436	22	88	21	6	0
4046	13644	9874	930	296	174	28044	1283	7	608	1	10	55	3	79	271.48	96	424.26	273	307	293	9	41	0	0	2
14612	7610	4342	6	716	500	27820	654	338	1129	163	40	262	133	146	269.16	289	431.47	477	463	440	13	87	21	0	0
3806	13670	9968	980	394	224	28072	1315	5	548	1	10	56	3	77	270.34	81	423.49	264	266	272	12	35	0	0	2
13772	7866	4616	5	932	694	27954	633	264	1035	125	70	246	104	167	274.43	260	438.79	479	405	429	31	99	18	2	2
4752	13174	9514	751	410	214	28070	1156	9	672	0	6	65	7	95	277.07	106	433.76	330	331	338	10	46	0	0	1
4108	13558	9822	926	358	210	28068	1286	4	594	1	12	64	2	72	271.49	93	424.25	282	294	275	18	46	0	0	0
4150	13430	9772	893	460	264	28088	1263	10	574	2	10	55	5	73	272.62	91	424.47	294	288	290	17	34	0	2	3
5080	12834	9236	672	608	366	28144	1153	13	655	3	18	65	7	93	281.36	97	437.97	356	354	311	17	40	0	2	1
12110	7848	5130	0	1746	1314	28422	570	133	680	25	258	175	76	269	300.66	150	454.53	496	230	385	60	93	6	14	8
6180	11982	8590	451	894	534	28214	971	13	740	2	34	73	9	128	287.29	114	442.34	420	361	375	23	45	0	0	3
11498	7966	5326	1	1962	1478	28530	544	115	613	25	280	174	62	299	310.31	108	459.41	469	212	394	72	80	10	18	17

SUPPLEMENTARY INFORMATION:Monte Carlo Atomistic Simulation and Machine Learning Analysis of Na-K Eutectic Alloy in Condensed Phases, D. Reitz and E. Blaisten-Barojas, George Mason University, Fairfax, VA 22030

11132	7950	5438	3	2134	1628	28642	516	82	570	18	324	167	38	279	315.68	111	463.04	501	196	366	102	84	5	34	22
13964	7748	4486	4	946	730	27962	637	291	1012	138	84	241	116	171	275.97	230	439.02	478	410	440	26	86	15	4	1
14056	7854	4512	13	764	620	27894	671	295	1086	145	88	248	128	145	271.18	268	434.44	478	456	433	17	98	11	0	4
12210	7922	5040	1	1626	1310	28392	558	146	716	42	258	179	71	243	301.85	144	454.71	477	268	412	81	80	9	26	12
12970	7794	4812	0	1404	1062	28226	617	171	844	58	176	182	83	203	284.2	173	450.6	477	325	435	61	93	18	8	5
13114	7586	4652	0	1500	1170	28228	589	209	786	81	192	212	100	235	283.61	167	448.28	490	295	424	41	95	12	12	3
11460	7886	5388	1	1954	1496	28548	519	117	591	22	324	172	64	261	304.09	110	458.52	499	196	391	102	94	8	38	15
14190	7666	4426	8	900	666	27918	614	306	1025	143	70	250	117	168	272.67	241	436.53	487	401	452	20	95	18	0	2
7296	11278	7982	352	980	622	28226	937	35	794	11	58	96	15	136	288.82	126	445.27	440	391	350	29	61	1	10	1
4270	13474	9750	882	356	202	28062	1270	6	620	1	10	61	4	82	275.15	86	428.82	281	326	289	12	38	0	0	1
13054	7846	4922	3	1262	894	28132	584	205	873	66	144	214	102	187	285.22	184	447.52	493	325	437	59	113	11	10	5
14156	7784	4440	4	822	644	27916	620	295	1051	146	64	252	105	164	274.4	266	438.93	489	402	445	22	94	15	6	1
14414	7664	4338	3	782	610	27876	649	323	1068	166	66	258	121	160	270.61	251	433.29	472	452	443	15	84	13	2	1
4962	12926	9322	712	576	330	28126	1176	6	669	0	10	66	3	100	279.86	98	436.25	331	354	306	15	43	0	0	1
4800	13014	9410	730	548	324	28118	1164	5	664	1	22	57	1	79	279.14	106	434.54	353	336	317	18	41	1	0	3
4608	13076	9520	763	570	326	28128	1206	4	612	0	28	55	3	84	277.63	80	433.35	325	323	306	17	37	0	0	3
14014	7646	4402	2	1028	804	27994	638	283	953	144	98	244	115	175	274.62	220	437.87	478	392	430	28	82	11	2	3
5628	12490	8988	593	624	364	28130	1054	17	710	5	36	71	10	105	282.09	117	439.66	389	346	362	13	43	0	0	2
10730	7738	5788	0	2424	1670	28764	469	70	478	14	354	179	34	282	331.98	82	469.41	489	141	376	123	91	5	58	34
4770	12938	9378	715	634	396	28156	1136	6	599	0	40	57	2	100	279.7	92	435.13	352	294	327	18	36	0	0	3
12232	7974	5068	1	1604	1240	28350	610	125	769	39	212	193	60	223	294.96	172	452.64	509	284	373	60	106	9	20	9
6426	11944	8494	464	772	490	28182	1012	15	760	2	56	76	8	111	285.84	116	443.59	419	383	344	24	57	1	0	1
4794	13134	9462	725	434	250	28090	1153	6	654	0	14	53	5	73	279.51	89	434.93	336	340	355	23	35	0	2	3
4374	13354	9664	845	418	254	28086	1216	3	606	0	20	66	2	92	274.95	93	431.26	297	295	303	15	44	0	2	4
3788	13832	10014	1012	244	148	28034	1323	10	564	2	8	62	5	62	269.65	94	421.86	266	264	266	14	41	0	0	2
4800	13030	9408	703	536	324	28124	1134	7	658	1	26	64	4	93	278.39	120	433.69	358	333	323	17	40	0	0	2
12126	7810	5118	0	1808	1318	28414	553	163	657	37	202	165	86	255	300.23	135	455.42	502	245	408	63	86	8	26	13
4998	12836	9308	667	636	350	28142	1130	6	679	0	14	77	4	115	279.36	106	437.35	346	342	316	14	44	0	0	0
11542	7970	5288	0	1942	1494	28548	548	93	636	16	280	165	54	254	311.52	127	458.36	492	219	389	93	89	4	30	23
5302	12678	9124	614	636	384	28154	1127	4	707	0	24	57	4	106	278.04	89	435.19	353	382	329	17	37	0	6	2
11426	7852	5392	0	2030	1536	28602	497	100	544	28	314	181	53	280	315.73	129	462.32	466	158	401	122	91	7	48	16
4506	13288	9626	839	422	224	28070	1219	9	622	1	4	67	4	62	274.45	95	429.83	327	318	296	18	50	0	0	4
6894	11660	8190	411	822	570	28214	970	23	772	6	72	66	13	125	287.04	123	443.92	402	381	393	33	45	1	6	3
11350	7990	5358	1	1992	1556	28602	524	94	607	18	328	181	50	296	310.5	120	463.6	528	213	349	64	107	5	26	16
7510	11060	7802	304	1086	718	28262	918	34	798	5	76	99	23	138	288.48	136	445.07	447	408	353	32	63	3	10	5
14274	7588	4326	4	914	730	27932	637	315	997	162	98	262	117	152	270.19	253	434.03	508	401	408	23	93	18	2	2

SUPPLEMENTARY INFORMATION:Monte Carlo Atomistic Simulation and Machine Learning Analysis of Na-K Eutectic Alloy in Condensed Phases, D. Reitz and E. Blaisten-Barojas, George Mason University, Fairfax, VA 22030

14464	7600	4338	4	802	602	27876	647	333	1051	167	66	270	127	145	269.55	269	431.85	497	434	418	17	90	19	4	2
13056	7862	4836	3	1320	946	28138	612	203	887	69	110	199	93	189	280.64	198	446.33	492	342	439	49	90	20	6	3
13824	7754	4564	5	1000	744	27978	608	269	996	117	92	229	121	168	276.28	240	440.14	493	380	459	29	99	13	0	3
4256	13464	9774	912	362	190	28052	1281	9	610	1	6	62	7	79	273.55	93	427.19	280	304	282	9	48	0	0	2
12128	7958	5072	3	1676	1298	28368	597	149	727	34	220	166	83	227	286.23	173	448.68	505	252	417	64	81	12	14	6
11288	7878	5416	1	2040	1596	28622	525	87	607	25	350	157	38	286	312.04	114	460.27	495	210	385	97	79	4	48	18
11302	7994	5440	0	1998	1506	28570	514	102	596	14	290	187	58	235	312.97	126	461.39	508	190	370	127	108	6	38	14
4112	13504	9820	913	400	224	28072	1287	9	575	1	12	56	6	74	272.17	76	425.86	270	295	285	19	39	0	0	3
11904	7994	5268	1	1726	1272	28422	569	117	692	27	244	175	58	235	307.61	134	458.3	476	247	399	87	102	11	14	19
12332	7720	4984	0	1788	1324	28382	529	160	658	47	222	177	78	240	300.01	153	455.3	507	213	430	75	91	9	8	11
14222	7580	4412	10	930	688	27920	655	316	1012	164	84	265	111	164	271.11	233	435.92	482	430	405	22	90	21	4	0
4474	13376	9618	855	360	226	28068	1240	8	636	0	14	61	4	78	275.43	95	430.14	298	315	295	17	44	0	0	2
13918	7714	4528	5	998	730	27970	652	312	983	143	80	226	134	158	273.03	246	436.25	502	404	434	18	73	16	2	2
4538	13194	9546	805	502	308	28114	1230	5	610	0	26	68	4	77	276.11	91	432.95	316	316	282	18	48	0	0	5
4298	13394	9714	874	416	240	28076	1236	8	599	2	12	61	4	78	273.22	100	427.88	303	281	289	19	43	0	2	2
11190	7918	5456	0	2128	1594	28640	505	94	554	19	308	163	54	294	312.55	121	462.31	498	166	384	95	78	5	44	23
4082	13494	9808	913	418	260	28090	1267	6	574	1	24	61	4	73	271.5	89	424.2	306	285	271	15	41	0	4	1
5048	12768	9268	636	658	378	28148	1110	7	660	1	24	61	5	94	279.84	93	435.87	374	356	341	17	38	0	4	1
11388	7806	5406	1	2116	1540	28592	543	93	588	17	304	155	49	261	310.09	120	456.49	477	199	397	100	87	13	32	27
4058	13566	9846	950	370	214	28064	1325	10	589	1	8	62	7	70	272.13	89	425.49	263	296	257	15	42	0	2	3
13534	7848	4600	6	1070	862	28034	629	266	919	128	110	227	109	177	276.07	210	440.77	497	387	431	25	80	13	10	5
4968	12962	9334	691	524	308	28114	1169	8	684	0	18	63	4	82	276.86	97	432.18	354	373	310	14	46	0	0	2
4888	12930	9376	704	584	328	28126	1113	9	648	0	20	49	8	82	279.22	104	436.18	372	313	353	20	33	0	0	2
13782	7626	4506	1	1114	876	28052	594	274	896	114	136	232	121	194	276.5	216	446.18	489	338	431	37	99	17	12	4
4218	13458	9790	905	378	204	28064	1257	6	603	1	16	58	4	75	274.02	93	428.44	305	298	290	8	41	0	0	2
4866	12954	9366	708	578	342	28128	1181	6	658	1	20	70	3	95	276.91	105	434.34	329	347	301	18	45	0	2	2
14146	7752	4468	6	840	632	27902	645	306	1053	150	54	249	121	156	271.83	271	435.13	491	415	439	16	88	19	10	0
10946	7714	5592	0	2326	1730	28780	486	82	487	11	424	167	43	291	332.72	92	470.26	490	146	357	127	89	3	46	31
5178	12794	9158	671	608	386	28146	1150	9	682	0	22	60	5	99	280.39	103	436.87	342	359	320	21	39	0	0	1
6434	12004	8532	467	708	434	28156	1013	26	805	6	42	74	15	111	286.86	114	441.11	403	427	369	22	53	0	0	1
4978	12780	9286	659	702	396	28160	1149	7	638	0	18	58	4	109	280.12	78	437.06	347	339	313	16	36	0	0	0
5676	12380	8894	563	734	452	28178	1076	12	707	0	40	60	8	93	281.37	111	437.82	388	373	349	28	42	0	2	1
4138	13514	9816	911	376	210	28066	1270	10	582	2	12	67	7	71	272.22	90	425.25	291	292	277	16	41	0	0	3
12736	7816	4928	0	1468	1098	28248	587	177	819	42	188	199	95	223	291.94	176	450.06	485	298	416	54	113	13	14	9
12690	7892	4856	0	1454	1174	28292	601	177	790	46	208	171	96	226	293.21	167	449.12	487	312	429	56	81	11	18	6
4302	13464	9736	886	354	196	28056	1262	9	633	2	4	69	5	69	272.68	94	426.77	289	319	285	17	51	0	0	4

5734	12516	8936	562	550	340	28114	1038	20	742	5	38	77	8	108	282.33	132	438.91	383	375	364	17	52	1	0	3
13958	7754	4490	6	944	726	27952	637	272	1025	122	70	238	116	160	275.68	263	441.44	480	389	448	30	95	18	8	1
4436	13126	9578	794	612	358	28136	1194	5	563	0	24	65	2	90	274.46	87	430.57	340	287	281	16	40	0	2	3
4434	13224	9646	803	508	272	28100	1225	6	630	0	14	60	4	69	275.78	98	430.55	330	326	291	15	37	0	2	3
4840	12984	9362	718	548	356	28132	1152	6	660	0	42	64	6	81	277.63	110	434.93	354	326	319	22	46	0	0	3
11928	7888	5216	0	1810	1342	28452	596	116	681	26	232	170	59	250	304.35	137	456.73	488	256	360	83	97	7	32	9
13740	7736	4588	9	1052	788	28014	622	273	974	133	102	244	109	175	276.5	237	442.07	499	387	421	32	96	11	8	0
13794	7632	4468	0	1112	904	28066	616	267	907	118	138	223	113	195	277.76	193	446.33	451	364	451	49	84	18	18	3
11338	7940	5380	4	2018	1554	28574	497	97	593	20	292	176	43	264	309.87	142	458.91	506	163	387	99	95	7	50	27
5350	12694	9086	606	598	382	28144	1100	12	716	0	32	68	10	107	278.96	104	436.31	363	375	337	18	46	0	2	1
4828	12948	9420	737	596	320	28128	1181	7	647	0	16	62	2	84	278.63	109	435.45	352	331	298	13	42	0	0	3
13762	7860	4596	7	936	722	27966	635	269	995	128	86	238	109	187	275.97	228	442.92	463	396	439	23	88	20	4	3
13536	7864	4698	2	1060	774	28024	609	233	983	92	82	212	104	186	277.09	220	445.71	464	363	474	35	102	16	10	6
13130	7856	4736	2	1232	1010	28156	591	208	862	79	178	207	96	210	283	197	447.27	475	310	454	46	97	17	12	6
10498	7924	5744	0	2394	1784	28800	490	53	477	8	424	141	22	305	333.17	79	471.6	480	148	359	134	61	3	32	27
13412	7708	4594	1	1250	996	28124	626	227	866	86	154	194	99	200	281.22	190	444.79	486	325	433	36	90	21	10	4
14196	7698	4446	6	836	636	27892	625	326	1036	161	80	264	132	153	270.27	257	432.52	494	413	431	24	93	12	0	1
4074	13554	9880	940	354	178	28046	1282	11	577	2	4	65	6	72	272.3	80	426.44	278	285	280	15	40	0	2	2
4558	13288	9590	819	402	228	28076	1237	7	633	2	10	60	4	67	274.28	88	429.08	313	335	301	17	40	0	0	2
4792	13088	9460	763	454	266	28088	1174	8	677	2	28	73	5	80	276.3	110	430.89	343	331	308	14	49	0	0	3
5100	12748	9252	690	644	364	28134	1133	7	676	0	26	61	5	100	279.28	117	435.74	360	333	322	14	45	0	0	1
4316	13446	9728	865	354	202	28058	1244	8	646	1	12	63	4	73	274.16	97	428.7	305	323	291	14	44	0	0	2
4484	13222	9604	782	500	280	28106	1199	1	617	0	16	63	1	78	277.09	83	433.56	332	326	303	18	41	0	0	2
13660	7708	4588	2	1114	850	28054	601	236	960	98	130	225	102	193	276.07	214	444.89	470	345	455	44	105	15	4	1
4130	13476	9808	920	424	230	28074	1260	8	565	2	6	75	4	73	273.37	105	427.52	307	261	258	16	39	0	0	2
11326	7950	5400	0	2026	1540	28588	528	92	593	14	318	148	49	259	313.36	117	457.58	515	196	399	93	76	6	28	19
4384	13384	9676	847	390	226	28072	1231	7	627	0	12	58	6	70	273.57	97	428.18	310	313	308	18	39	0	0	1
4458	13348	9624	829	404	238	28080	1202	10	616	1	8	67	7	76	274.8	103	429.81	337	294	300	12	46	0	0	1
3962	13640	9898	956	342	204	28060	1299	7	570	1	14	65	5	74	270.59	84	422.8	266	274	274	17	40	0	0	2
6694	11710	8298	411	896	564	28204	964	33	781	6	40	72	21	121	286.84	131	444.36	417	395	382	29	38	0	2	3
11580	7942	5346	3	1880	1432	28512	537	111	654	21	308	162	62	276	305.02	145	455.13	501	213	403	74	87	9	24	12
4656	13204	9528	765	442	250	28090	1208	3	658	0	10	59	2	83	276.26	98	431.48	312	348	312	20	41	0	0	0
4758	13152	9480	752	422	250	28084	1156	6	669	4	20	67	2	81	276.56	114	431.29	348	333	331	9	42	0	2	4
14300	7776	4430	6	732	564	27860	645	311	1085	159	54	257	116	161	269.49	266	431.34	465	430	447	16	92	14	4	2
4206	13520	9794	883	334	188	28054	1250	5	611	0	12	65	3	68	272.01	99	425.66	306	304	291	12	37	0	0	3
4738	13132	9470	754	466	276	28102	1190	8	658	1	20	66	5	91	276.12	102	431.75	325	343	307	15	43	0	0	1

SUPPLEMENTARY INFORMATION:Monte Carlo Atomistic Simulation and Machine Learning Analysis of Na-K Eutectic Alloy in Condensed Phases, D. Reitz and E. Blaisten-Barojas, George Mason University, Fairfax, VA 22030

6018	12272	8792	512	638	372	28132	1048	23	779	2	40	66	16	92	283.04	116	439.76	410	427	354	17	48	1	0	2
13416	7582	4588	1	1356	1046	28170	597	232	797	88	172	209	103	216	282.58	183	449.18	469	302	440	52	93	20	10	2
4710	13042	9432	720	568	356	28144	1154	2	633	0	34	58	2	99	276.45	94	432.34	348	328	321	11	41	0	2	3
12420	7910	4950	2	1604	1242	28338	554	153	719	40	196	187	84	246	301.43	161	454.27	485	257	416	69	88	8	14	10
4940	13120	9424	729	372	200	28064	1167	5	730	0	8	52	3	62	276.62	126	430.1	342	364	348	17	37	0	0	5
4168	13528	9784	900	358	214	28062	1243	8	589	2	10	55	4	64	272.81	101	427.82	310	279	301	17	37	0	0	2
4920	12978	9380	734	506	294	28106	1175	9	668	2	28	71	4	87	279.29	104	435.51	332	338	313	19	46	0	0	1
3816	13802	10002	1005	258	150	28034	1309	5	571	1	6	59	2	61	270.17	93	423.46	274	260	274	12	36	0	0	4
11334	8030	5408	1	1964	1504	28572	556	85	639	20	300	178	48	288	315.37	129	460.66	447	215	379	94	86	2	30	21
13816	7736	4622	6	1006	706	27970	652	268	998	130	80	234	102	161	274.71	232	439.25	492	399	425	25	97	13	4	1
6364	11924	8498	493	824	528	28196	1031	19	741	5	56	73	9	120	287.58	113	443.15	400	377	342	21	46	0	0	2
4508	13216	9602	806	480	268	28092	1190	6	614	1	18	61	4	82	275.51	84	429.73	319	299	325	15	44	0	0	5
5684	12272	8832	569	814	534	28202	1069	17	652	6	62	72	8	121	284.81	95	441.29	387	341	327	17	42	0	4	2
10708	7884	5628	2	2294	1782	28776	521	64	460	11	428	164	30	321	331.59	94	470.45	436	143	348	117	72	4	48	37
6904	11364	8152	357	1080	686	28276	914	22	728	9	84	77	10	141	290	126	446.48	461	355	362	25	43	0	6	5
4218	13446	9768	870	410	224	28074	1258	7	593	1	8	53	4	79	273.7	89	427.33	291	309	293	17	29	0	0	0
4510	13254	9582	822	458	276	28102	1212	10	619	0	22	59	8	91	277.51	106	432.38	314	305	306	14	39	0	0	0
14426	7686	4398	4	766	540	27854	620	353	1064	170	38	248	143	127	270.55	298	432.34	522	408	458	18	70	24	0	0
14374	7720	4388	5	748	574	27866	649	317	1089	164	62	254	117	147	270.14	277	432.31	474	453	445	24	76	16	0	0
4206	13520	9770	902	344	210	28066	1243	12	593	1	16	53	8	87	273.8	100	426.75	292	278	307	10	32	0	0	1
14078	7740	4472	6	892	670	27916	646	310	1032	154	56	252	130	158	269.33	256	432.01	491	423	431	16	84	13	4	2
4398	13316	9648	877	446	260	28082	1267	8	619	1	14	50	3	69	273.26	95	427.31	296	311	293	17	36	0	0	2
13786	7920	4572	6	898	710	27962	624	263	1031	126	66	230	109	141	274.44	268	439.34	496	392	458	39	94	13	10	3
4670	13170	9530	749	452	250	28086	1162	8	653	0	14	70	5	80	277.84	106	434.2	354	335	312	13	47	0	0	2
5962	12152	8694	513	810	528	28208	1007	16	675	1	62	58	10	94	285.67	115	441.02	451	343	362	16	39	0	0	5
5350	12584	9124	621	684	380	28148	1097	12	678	0	24	74	9	108	280.73	106	439.79	369	355	326	17	52	0	2	2
3984	13554	9878	967	410	234	28076	1302	10	558	1	16	51	5	80	269.83	75	422.11	260	262	285	16	39	0	0	2
4744	13182	9468	747	422	260	28094	1159	8	643	0	16	65	3	95	276.79	84	433.15	332	331	326	15	43	0	2	1
5944	12116	8702	525	854	538	28204	1021	21	680	5	42	71	11	133	286.19	95	441.99	379	340	363	25	46	1	8	2
4534	13182	9566	784	510	296	28110	1193	8	599	0	22	55	5	96	278.21	78	433.87	311	315	322	17	34	0	0	1
14496	7654	4354	5	766	544	27846	615	337	1086	163	28	260	130	154	270.87	278	431.32	495	425	453	13	91	21	2	1
4668	13036	9470	730	590	342	28128	1157	3	627	0	22	62	3	85	277.9	97	434.02	354	317	315	17	41	0	0	2
12610	7674	4926	2	1668	1230	28342	573	174	714	61	218	182	80	234	298.2	155	453.01	501	246	406	68	90	8	16	7
3986	13652	9880	961	322	204	28062	1281	5	587	0	18	54	2	70	270.36	107	423.41	287	264	288	14	33	0	0	1
10822	7864	5478	3	2290	1842	28776	516	68	503	15	428	172	31	289	323.29	94	467.58	509	161	325	107	94	4	50	35
6340	11934	8510	443	832	526	28192	978	23	739	6	46	78	12	116	287.6	113	446.49	413	383	370	34	43	0	2	1

SUPPLEMENTARY INFORMATION:Monte Carlo Atomistic Simulation and Machine Learning Analysis of Na-K Eutectic Alloy in Condensed Phases, D. Reitz and E. Blaisten-Barojas, George Mason University, Fairfax, VA 22030

4170	13560	9832	911	312	162	28040	1258	11	616	1	4	57	6	66	272.81	100	425.52	304	294	292	12	41	0	0	1
5490	12532	9042	592	668	386	28150	1108	8	727	0	32	67	6	113	280.99	97	438.65	358	383	332	14	50	0	0	2
12968	7682	4832	3	1472	1074	28212	594	196	835	78	172	212	85	227	286.21	175	450.43	461	314	409	61	101	10	10	7
10996	7856	5500	1	2244	1688	28670	528	75	570	16	346	161	35	262	310.95	113	460.59	512	190	357	111	85	2	36	25
11296	7938	5510	2	2004	1476	28574	508	95	606	21	312	174	50	292	315.04	107	461.94	467	188	391	101	81	4	36	17
4628	13136	9498	787	524	314	28118	1191	8	615	0	14	56	6	95	276.48	88	432.62	327	308	313	11	42	0	2	2
5494	12592	9034	616	632	366	28132	1083	12	675	1	14	78	7	102	281.88	105	436.89	377	338	330	20	54	0	0	1
4496	13266	9602	806	452	260	28090	1216	9	621	2	14	60	6	70	274.57	97	429.57	330	315	298	16	39	0	0	3
13738	7860	4614	4	998	714	27978	625	266	988	112	52	235	116	176	277.93	231	443.16	489	390	437	24	100	21	2	2
4062	13510	9848	909	410	230	28078	1270	6	580	0	16	67	4	63	272.09	92	425.64	301	292	273	18	41	0	2	4
4886	13042	9404	734	468	276	28102	1135	7	668	1	24	69	3	86	279.48	100	435.89	356	328	331	13	44	0	2	5
5662	12450	8950	564	662	386	28138	1081	11	747	2	26	63	6	92	281.28	117	436.81	388	394	344	21	40	0	2	1
14322	7712	4406	3	780	590	27868	634	325	1079	152	50	268	132	141	269.38	266	432.93	505	442	429	18	103	25	8	1
4656	13174	9526	762	460	262	28094	1195	2	655	0	14	73	2	67	278.89	92	434.76	338	344	299	24	50	0	2	1
13636	7750	4660	2	1094	780	28016	642	243	997	106	88	228	107	180	277.03	225	445.12	482	386	423	28	105	13	6	3
14280	7666	4382	2	864	648	27900	641	323	1040	168	56	272	124	151	270.17	270	435.08	508	407	407	15	95	16	4	2
10550	7968	5608	1	2352	1860	28800	472	57	498	8	400	169	27	298	327.83	95	472.45	479	139	362	130	72	3	54	36
11980	7892	5162	1	1750	1362	28442	585	124	706	24	252	178	65	252	301.39	128	454.03	473	260	372	87	114	8	40	12
4092	13610	9866	954	300	164	28040	1294	12	590	1	8	58	7	73	272	91	424.92	272	297	281	13	37	0	0	1
5634	12462	8940	591	678	408	28152	1104	11	720	1	30	56	5	90	284.32	109	439.57	390	384	330	19	37	0	0	0
13640	7796	4600	11	1070	810	28014	656	281	951	136	94	224	111	171	275.92	238	439.39	492	386	427	22	74	15	4	2
12046	7826	5202	2	1772	1294	28412	562	132	695	27	256	158	75	239	304.34	149	456.41	498	224	421	70	90	7	16	16
4796	13024	9444	732	528	296	28110	1175	4	668	0	22	61	1	89	278.06	113	433.24	332	339	315	19	38	0	0	1
11106	7860	5406	0	2194	1704	28668	524	100	525	17	362	166	42	290	318.11	96	463.5	454	174	374	111	79	6	32	27
10644	7944	5624	0	2348	1782	28754	515	66	546	14	354	170	34	286	322.65	111	464.71	496	170	343	126	91	5	50	24
4112	13540	9834	924	368	200	28060	1290	6	602	1	6	56	4	66	271.73	95	425.77	287	289	281	11	37	0	0	4
5032	12818	9268	656	610	376	28146	1109	7	646	0	40	75	6	99	279.74	92	436.9	350	336	331	28	54	0	2	3
11228	7788	5510	1	2098	1600	28674	552	89	551	18	404	167	46	272	321.33	113	464.51	501	188	340	99	101	4	44	24
13188	7802	4720	3	1286	990	28136	613	198	875	89	140	221	77	204	279.81	176	444.8	471	319	417	46	105	17	8	8
6506	11834	8450	476	824	512	28184	1034	38	708	10	56	84	20	116	287.36	110	442.81	400	397	328	27	45	0	2	0
4628	13252	9532	791	420	248	28088	1184	5	642	0	8	66	3	89	276.2	110	430.54	321	312	322	15	44	0	0	1
4090	13548	9840	925	366	208	28066	1288	7	607	1	14	68	4	71	272.87	96	426.96	295	300	254	13	45	0	0	2
13730	7882	4602	6	954	728	27974	645	251	1008	115	70	217	100	165	276.33	235	443.41	465	401	454	37	85	15	8	1
14134	7798	4512	7	770	594	27888	591	301	1102	149	70	254	126	186	274.04	244	438.55	446	408	489	27	94	11	10	1
10464	7792	5806	1	2486	1800	28818	483	64	461	14	424	167	27	319	334.49	69	470.01	454	150	337	138	86	3	42	34
11058	7898	5556	3	2138	1610	28674	532	76	550	8	380	151	41	283	316.97	99	462.69	456	180	381	122	67	7	34	25

SUPPLEMENTARY INFORMATION:Monte Carlo Atomistic Simulation and Machine Learning Analysis of Na-K Eutectic Alloy in Condensed Phases, D. Reitz and E. Blaisten-Barojas, George Mason University, Fairfax, VA 22030

14164	7768	4440	7	818	640	27900	642	315	1046	157	64	258	131	159	271.4	276	434.7	483	423	434	18	88	13	6	2
11342	7914	5390	2	2072	1566	28630	512	94	561	23	290	173	46	275	324.15	103	467.39	478	186	381	102	86	7	56	33
8458	10708	7444	228	932	572	28178	810	57	922	12	62	106	32	163	288.39	162	447.43	432	407	431	34	72	3	2	3
12816	8116	4952	3	1184	920	28128	623	196	915	70	126	196	91	192	283.19	206	447.62	486	352	434	43	105	10	12	3
11566	7858	5400	2	1984	1424	28542	525	114	608	21	278	162	59	259	312.57	141	460.19	518	190	393	84	78	5	30	20
6588	11896	8386	403	782	500	28190	975	18	789	6	36	77	10	126	286.34	135	443.38	427	402	371	18	52	0	2	0
14328	7740	4384	7	764	598	27874	610	310	1087	162	54	263	107	142	270.54	289	433.06	532	411	435	11	93	21	6	1
14250	7666	4392	2	878	658	27904	654	321	1023	163	52	257	119	138	269.79	263	431.65	513	426	411	18	79	19	6	2
5658	12394	8930	611	716	420	28148	1107	16	706	3	28	70	6	98	282.96	98	439.62	370	360	316	20	47	0	2	5
4052	13606	9876	948	324	180	28048	1283	10	584	1	10	62	5	68	271.98	88	424.82	285	284	279	13	42	0	0	3
11606	7978	5410	0	1852	1342	28476	584	99	664	24	266	196	46	272	305.36	131	456.67	455	240	358	84	103	6	20	15
13874	7722	4542	1	998	746	27976	597	310	946	142	84	256	120	167	275.96	229	442.67	485	331	431	43	96	19	10	5
12862	7810	4866	3	1428	1082	28238	603	181	815	60	166	188	76	203	287.22	179	450.07	501	308	412	55	98	20	16	9
4392	13324	9670	836	440	244	28082	1243	5	649	0	12	58	0	84	276.98	102	431.11	296	333	292	12	38	0	0	5
4464	13338	9656	846	390	210	28066	1213	13	610	1	8	57	7	71	275.99	102	429.6	317	299	316	17	34	0	0	2
12592	8104	5022	4	1312	1004	28198	646	178	874	47	156	175	100	195	282.57	197	445.25	485	340	440	45	100	15	8	3
14282	7670	4368	6	842	662	27902	583	340	1003	155	74	254	138	160	270.19	271	435.33	517	382	463	14	87	21	2	3
14544	7662	4340	5	708	530	27834	638	337	1100	169	44	267	129	137	270.7	299	432.3	497	454	439	16	83	23	6	1
4556	13216	9570	808	474	266	28092	1216	11	608	1	10	58	4	76	275.77	114	432.01	330	315	297	13	40	1	0	2
6378	11930	8470	444	810	544	28198	965	26	743	7	60	85	16	126	286.25	142	444.66	422	361	366	27	45	0	4	1
4296	13374	9730	887	422	236	28078	1256	7	601	1	18	65	5	92	274.79	90	429.02	275	293	291	16	44	0	2	1
14168	7628	4408	2	932	704	27920	643	309	1028	164	76	269	114	175	270.68	248	433.78	481	425	407	17	90	16	4	1
14476	7638	4378	6	752	544	27842	629	331	1101	169	50	284	126	143	271.68	269	435.38	488	448	424	26	99	13	4	0
7838	11002	7736	298	970	598	28208	898	44	816	9	58	96	20	142	287.84	136	446.71	431	389	377	32	56	2	6	6
11640	7868	5276	3	1930	1482	28524	502	111	630	26	282	190	59	282	310.18	125	457.46	473	191	409	90	111	3	42	21
4438	13276	9628	807	466	268	28092	1211	7	630	0	16	62	5	75	273.83	96	428.77	324	322	300	18	44	0	0	3
4634	13154	9554	761	462	260	28094	1181	9	633	1	30	56	5	86	275.93	93	430.45	321	335	326	18	34	0	0	2
4510	13316	9640	842	374	204	28058	1244	12	637	3	14	63	6	71	274.81	101	428.94	303	333	297	10	39	0	0	6
5122	12726	9208	644	684	398	28162	1103	2	651	1	24	66	1	103	280.49	100	435.43	377	333	332	12	40	0	0	4
11502	7966	5352	3	1958	1460	28532	548	100	631	23	256	192	49	260	316.63	134	458.81	479	198	365	97	100	9	36	22
3948	13690	9936	984	302	162	28040	1309	12	592	1	2	45	5	62	270.13	92	423.58	270	266	287	15	33	0	0	2
12292	7998	5036	0	1548	1232	28344	574	161	739	41	208	184	86	241	293.04	165	450.92	496	268	404	61	95	11	24	11
14248	7694	4444	5	840	606	27888	631	318	1093	156	54	253	126	149	270.38	292	433.89	501	438	445	16	87	18	2	1
14418	7744	4380	5	696	548	27848	660	326	1129	162	60	260	125	152	268.07	282	430.12	479	464	427	13	88	17	2	0
4122	13508	9842	905	388	200	28068	1264	4	574	0	6	64	4	66	274.72	73	428.97	293	285	290	17	49	0	2	4
11754	7932	5304	0	1840	1348	28448	562	122	654	30	244	170	58	257	304.95	138	454.99	472	222	399	95	80	9	26	8

SUPPLEMENTARY INFORMATION:Monte Carlo Atomistic Simulation and Machine Learning Analysis of Na-K Eutectic Alloy in Condensed Phases, D. Reitz and E. Blaisten-Barojas, George Mason University, Fairfax, VA 22030

8142	10630	7428	272	1174	802	28282	829	56	754	11	90	99	36	161	286.97	137	447.86	466	342	395	30	59	2	14	7
11554	7802	5262	1	2040	1564	28570	535	112	583	21	320	174	61	278	309.79	124	458.38	486	191	372	95	95	7	24	21
5468	12420	8996	574	782	474	28188	1087	14	668	6	44	68	7	99	282.84	106	440.04	400	354	316	22	41	0	4	0
14432	7720	4396	5	712	528	27836	636	344	1113	163	48	254	135	147	268.3	280	430.6	495	452	448	7	78	25	0	3
4114	13548	9842	923	352	190	28054	1265	11	598	1	8	69	7	69	272.46	102	425.05	302	282	275	11	39	1	0	3
10908	7872	5550	1	2236	1722	28720	508	89	489	11	370	141	41	283	322.77	82	465.48	464	163	383	136	67	6	52	20
4492	13228	9590	789	500	284	28106	1171	3	613	0	12	61	2	86	276.8	88	433.34	325	302	331	21	42	0	0	2
4522	13138	9542	782	584	336	28136	1179	4	623	0	12	59	3	103	277.45	98	430.71	324	298	316	15	42	0	2	1
12054	8086	5214	1	1604	1184	28346	577	148	749	36	182	173	74	225	300.96	161	451.8	513	257	405	66	102	11	22	8
14314	7790	4460	6	696	534	27862	673	328	1104	146	64	246	141	146	274.53	260	438.52	469	476	438	18	89	16	4	1
5080	12884	9290	673	532	304	28110	1131	8	706	2	20	64	1	89	277.14	120	434.97	366	357	323	14	39	0	0	2
14288	7554	4378	5	930	686	27920	640	317	1011	160	80	265	126	164	272.55	241	435.99	484	425	414	23	97	14	4	2
3708	13794	10058	1033	308	168	28046	1334	5	544	1	10	54	4	76	270.38	76	422.3	246	255	270	12	41	0	0	2
3984	13674	9906	964	306	174	28050	1299	10	567	2	6	56	6	60	270.9	90	423.81	281	278	281	15	37	0	0	3
14102	7752	4474	6	874	652	27920	652	310	1031	144	66	246	134	159	271.66	250	435.83	475	421	437	24	93	17	0	1
5490	12584	9032	622	630	380	28146	1098	11	687	1	26	67	7	98	281.7	109	439.52	381	348	334	15	47	2	4	2
14440	7726	4392	6	722	522	27834	660	317	1130	175	30	270	105	145	268.75	293	430.36	490	471	416	10	84	17	0	1
5388	12668	9134	614	596	324	28126	1114	9	723	0	16	58	6	78	281.69	119	436.74	382	382	342	15	39	0	0	5
4428	13262	9662	809	474	250	28090	1209	5	600	0	14	57	3	85	278.47	80	433.8	311	314	311	18	35	0	0	1
6122	12104	8660	486	774	476	28182	1025	14	758	1	42	69	8	117	284.63	116	442.3	400	390	356	19	46	0	4	4
13898	7504	4386	3	1198	938	28060	579	278	846	113	122	244	129	199	277.23	206	445.66	479	309	446	39	105	16	14	6
10840	7918	5598	2	2246	1724	28768	529	65	523	10	376	161	32	286	330.03	98	471.62	486	169	349	121	78	2	56	26
3974	13658	9900	934	320	190	28054	1289	8	573	0	12	62	6	60	271.42	84	424.68	291	288	277	12	44	0	0	5
13684	7586	4414	1	1278	1010	28102	596	252	836	97	126	236	101	216	280.81	181	446.77	487	316	410	34	110	21	4	4
13326	7772	4682	2	1250	950	28118	575	218	891	75	126	210	107	205	281.67	205	447.47	484	326	460	44	110	14	10	5
4150	13460	9784	919	424	246	28080	1290	7	578	1	16	62	5	68	273.82	88	426.66	291	298	267	15	43	0	0	3
4190	13504	9792	921	364	202	28060	1287	5	601	1	8	64	2	69	273.9	93	427.29	294	296	267	11	47	0	0	2
11652	7970	5390	0	1784	1362	28510	531	122	657	23	324	175	62	266	317.99	158	460.1	504	197	386	80	99	7	26	19
13760	7930	4638	1	874	666	27946	636	273	1064	120	74	236	123	166	275.62	248	440.35	477	416	448	27	101	13	4	1
14448	7626	4382	6	780	564	27860	643	328	1088	165	56	262	125	147	270.06	263	432.2	489	446	441	16	87	20	4	0
4504	13292	9602	835	410	250	28082	1255	9	644	1	24	56	4	75	276.36	93	431.2	294	353	288	19	32	0	0	3
12992	7800	4722	3	1436	1106	28208	623	198	786	64	140	180	86	200	282.95	183	447.68	505	288	413	50	82	18	10	2
4018	13586	9866	947	374	212	28062	1292	10	571	1	6	58	7	68	272.31	91	425.43	275	282	282	16	37	0	0	4
10654	7830	5684	1	2334	1814	28846	493	56	482	6	464	154	29	290	333.98	85	474.05	489	142	341	130	88	3	64	41
12998	8008	4874	4	1218	912	28134	609	202	901	64	112	178	105	199	281.37	219	445.63	495	324	457	39	90	17	10	1
10978	7958	5598	1	2160	1600	28676	536	73	571	11	332	153	41	280	315.04	111	463.16	473	204	383	101	79	7	50	31

SUPPLEMENTARY INFORMATION:Monte Carlo Atomistic Simulation and Machine Learning Analysis of Na-K Eutectic Alloy in Condensed Phases, D. Reitz and E. Blaisten-Barojas, George Mason University, Fairfax, VA 22030

4328	13408	9702	857	386	230	28072	1239	4	606	0	18	55	4	79	273.47	86	427.37	298	311	313	11	38	0	0	3
14056	7808	4524	4	830	624	27912	621	303	1048	144	64	250	116	158	274.25	271	438.32	494	408	438	25	95	15	6	1
14380	7714	4444	9	718	522	27834	627	327	1103	166	52	272	125	148	269.64	280	432.77	501	451	430	12	99	16	4	1
4068	13576	9874	941	344	180	28048	1275	8	584	1	6	65	4	77	272.18	93	426.76	284	275	278	10	44	0	0	3
14268	7782	4426	6	736	592	27880	640	337	1069	155	68	238	147	140	270.43	294	433.25	496	428	461	17	69	14	8	2
12804	7808	4840	3	1468	1134	28250	589	178	793	63	174	197	82	220	285.59	175	450.58	498	290	424	51	95	11	20	6
5806	12324	8842	538	730	434	28166	1078	10	730	1	28	74	5	99	283.74	109	440.63	392	390	327	19	53	0	2	4
5480	12648	9034	627	556	366	28128	1094	16	702	4	42	63	9	103	281.38	105	438.72	361	364	351	18	36	0	2	2
6352	11894	8580	469	830	474	28180	1025	37	743	8	46	77	24	124	286.46	101	440.79	388	408	354	20	47	0	4	4
14016	7608	4530	2	1016	708	27968	630	274	987	106	86	239	120	166	276.41	217	445.38	472	380	440	38	111	23	4	2
11676	7730	5150	1	2018	1628	28598	505	96	628	23	348	164	48	258	311.76	134	458.4	516	191	402	104	89	6	46	20
3960	13668	9924	980	312	174	28046	1322	10	594	2	6	62	6	61	270.55	93	423.02	272	295	258	17	44	0	2	1
10422	7730	5696	1	2470	1996	28936	470	61	416	9	546	169	30	302	329.53	86	472.11	485	130	329	144	67	2	64	45
11094	7704	5492	0	2274	1720	28740	487	90	500	13	410	169	46	292	334.57	104	471.8	491	144	349	121	90	4	40	29
4516	13338	9614	817	368	218	28068	1222	7	645	1	12	56	4	69	276.27	95	430.73	319	335	311	16	38	0	2	1
14180	7736	4416	4	848	658	27906	661	304	1052	160	66	257	111	156	271.46	267	434.3	487	428	418	17	89	16	2	0
4600	13294	9546	792	378	242	28080	1194	0	664	0	20	61	0	75	275.56	102	430.53	332	335	317	12	42	0	0	4
3888	13722	9952	983	296	176	28046	1308	5	575	1	12	54	2	64	270.94	82	423.64	266	273	280	16	39	0	0	4
7470	11094	7886	316	1050	656	28234	920	39	805	12	74	108	21	145	288.77	128	445.2	437	413	346	30	62	0	4	3
4350	13338	9672	833	468	262	28096	1236	3	592	0	6	56	2	70	276.79	78	431.68	326	317	291	16	35	0	0	0
14302	7800	4474	8	690	518	27840	621	314	1133	150	54	259	131	147	271.15	281	434.2	480	452	462	22	97	10	2	1
4426	13288	9634	823	460	268	28094	1228	7	632	0	16	58	5	69	273.44	110	428.44	338	318	287	10	42	0	2	3
13226	7802	4638	2	1310	1034	28150	610	236	817	86	126	209	108	223	285.08	181	444.7	469	313	436	39	91	19	14	1
14116	7776	4526	6	850	588	27896	637	310	1065	141	38	238	131	145	274.19	253	440.27	487	428	459	26	81	18	2	0
5084	12908	9272	651	548	308	28122	1129	8	696	0	2	55	6	86	277.43	105	435	369	365	335	15	33	0	0	2
14204	7606	4430	4	934	672	27918	615	310	986	148	70	260	120	173	273.12	223	438.64	484	403	436	21	98	18	2	2
5012	12888	9286	672	580	346	28132	1106	3	659	0	18	59	3	87	280.43	105	436.37	374	329	349	20	42	0	2	1
4102	13590	9852	951	320	174	28042	1305	9	603	2	4	63	6	68	272.19	95	425.44	269	291	270	18	41	0	0	0
6762	11646	8284	434	888	562	28204	1014	27	767	10	58	93	16	123	284.81	113	442.48	404	416	331	27	55	0	4	2
14232	7560	4418	4	950	682	27922	623	333	1014	147	76	246	132	182	275.42	249	440.11	471	388	439	18	91	22	4	4
11468	8102	5426	1	1840	1376	28502	562	87	670	16	264	165	48	249	306.98	121	457.55	496	218	386	92	100	3	24	12
12470	7858	4998	1	1594	1200	28348	563	144	771	32	212	189	67	248	303.97	154	455.73	482	256	410	69	115	17	16	8
14182	7704	4386	4	874	696	27922	637	316	1015	163	74	253	118	161	272	271	434.99	496	424	425	20	72	13	4	0
5110	12910	9262	686	510	302	28108	1141	9	685	3	12	68	3	89	280.01	94	436.49	351	351	323	17	45	0	2	3
5452	12500	8992	580	730	466	28192	1061	11	668	1	52	64	4	110	286.29	115	439.7	417	331	324	11	43	0	0	2
5214	12788	9176	665	574	352	28128	1143	11	696	3	24	65	2	101	281.01	91	438.39	340	372	323	17	39	1	0	3

SUPPLEMENTARY INFORMATION:Monte Carlo Atomistic Simulation and Machine Learning Analysis of Na-K Eutectic Alloy in Condensed Phases, D. Reitz and E. Blaisten-Barojas, George Mason University, Fairfax, VA 22030

4068	13554	9828	923	384	228	28074	1273	9	580	1	12	58	6	62	271.35	96	425.56	298	283	287	19	33	0	0	1
4626	13232	9540	790	426	248	28084	1181	6	646	0	12	62	4	77	275.77	99	430.94	334	323	326	13	43	0	0	4
4216	13418	9742	862	426	262	28092	1237	4	576	0	24	56	2	80	275.71	80	430.32	304	296	296	15	34	0	4	4
4362	13334	9678	846	446	252	28086	1239	6	602	0	14	50	4	59	276.37	88	431.37	321	301	298	17	38	0	0	6
5036	12886	9266	652	572	354	28142	1118	6	663	0	26	64	2	94	279.34	113	434.03	365	350	328	22	44	0	2	1
5848	12084	8754	539	938	560	28232	1056	16	648	2	46	76	12	135	286.55	97	441.35	368	320	332	27	42	0	2	1
4556	13210	9560	799	482	278	28100	1186	3	638	0	14	60	1	92	274.78	92	431.3	322	303	321	12	39	0	0	3
14150	7648	4398	3	922	726	27940	611	323	977	158	86	253	132	165	272.77	248	437.04	508	395	435	20	80	20	10	1
4390	13346	9638	838	440	270	28100	1250	5	613	0	12	58	4	74	276.86	87	431.23	312	325	288	14	33	0	4	2
11790	7838	5238	3	1866	1426	28484	544	120	640	30	300	174	69	258	303.11	118	455.71	464	218	418	95	98	5	26	17
14376	7614	4344	5	876	634	27888	665	320	1038	170	42	271	112	155	268.94	248	431.18	485	445	398	15	87	16	2	3
13714	7594	4456	5	1206	962	28078	627	290	835	116	132	215	129	204	278.35	177	443.32	437	331	454	45	84	24	14	4
11582	8048	5312	0	1882	1402	28474	558	117	650	27	216	168	64	247	303.89	135	455.13	486	222	414	84	93	4	30	13
13186	7856	4840	7	1220	876	28112	633	207	906	70	128	200	100	187	283.11	195	446.19	480	366	440	44	97	17	6	2
3830	13764	10002	1020	286	152	28036	1341	6	564	1	2	56	3	68	270.46	80	422.47	256	266	260	11	40	0	0	1
4324	13428	9746	896	360	188	28056	1289	8	610	1	10	56	4	60	273.92	89	427.62	291	332	279	15	33	0	0	3
4518	13280	9592	803	438	252	28090	1196	4	632	0	10	58	4	60	274.59	111	427.86	351	318	318	15	37	0	0	2
4166	13536	9808	911	344	194	28058	1284	4	609	1	10	63	2	72	272.48	85	424.66	277	315	285	14	43	0	0	3
4660	13202	9512	807	424	264	28088	1207	5	646	0	26	52	4	82	275.58	99	428.9	312	316	322	19	40	0	0	0
4760	13178	9464	773	410	254	28086	1217	3	693	0	20	59	2	67	276.38	96	430.85	330	360	308	14	37	0	0	3
5890	12126	8764	496	878	512	28208	1026	12	706	0	38	69	8	125	287.67	106	443.81	405	373	335	22	43	0	0	2
12946	7770	4890	2	1402	1002	28168	615	219	840	76	144	198	100	199	283.26	182	446.78	476	309	430	51	97	20	14	9
14350	7688	4362	8	810	614	27876	635	329	1050	171	50	271	119	154	269.46	271	432.77	496	428	422	10	88	15	2	6
4530	13196	9570	764	502	292	28112	1171	3	629	0	22	72	1	73	276.29	105	431.8	359	316	301	16	44	0	0	4
5166	12724	9208	659	644	374	28144	1104	13	634	1	24	60	9	96	279.91	84	436.85	365	325	344	23	40	0	4	1
4262	13532	9790	889	296	154	28038	1246	5	631	2	4	66	2	76	272.25	107	425.86	292	301	301	13	44	0	0	1
3896	13660	9954	985	342	188	28052	1318	8	581	0	10	55	4	72	270.9	95	423.61	264	280	270	11	39	0	2	3
5676	12390	8954	531	706	398	28160	1052	9	734	0	36	80	5	114	283.3	113	440.65	383	382	342	16	56	0	0	6
4366	13386	9692	840	384	226	28076	1201	7	608	1	22	57	4	79	273.46	91	429.53	322	291	323	11	42	0	0	3
14270	7714	4378	5	812	648	27900	668	310	1050	158	76	253	119	145	270.71	262	433.44	498	436	412	16	82	12	2	1
13956	7728	4482	6	982	740	27966	622	284	980	114	76	218	127	175	276.32	232	441.87	467	382	470	32	86	17	2	1
13586	7822	4694	7	1004	758	27984	631	269	1014	124	116	250	110	189	275.85	237	440.63	464	390	424	26	106	18	4	4
5502	12746	9118	642	446	248	28078	1105	15	778	4	18	81	9	98	279.68	118	435.5	354	381	340	14	55	0	0	1
7656	11112	7738	281	970	668	28224	866	36	809	10	74	112	19	142	289.29	137	447.8	447	397	378	34	71	1	6	8
14376	7570	4322	3	896	674	27910	635	325	1023	159	70	259	125	155	273.35	263	438.04	487	402	432	23	84	17	2	3
5712	12398	8888	507	698	434	28174	1028	8	733	1	40	83	5	91	285.13	132	441.57	441	376	330	19	55	0	4	2

SUPPLEMENTARY INFORMATION:Monte Carlo Atomistic Simulation and Machine Learning Analysis of Na-K Eutectic Alloy in Condensed Phases, D. Reitz and E. Blaisten-Barojas, George Mason University, Fairfax, VA 22030

5190	12766	9196	604	622	356	28144	1088	9	686	0	14	70	6	100	281.13	99	438.11	386	369	334	14	46	0	0	2
4854	13080	9400	726	478	282	28104	1177	5	687	0	8	52	4	80	276.96	99	431.88	338	359	323	17	34	0	2	3
3994	13578	9888	939	382	212	28066	1299	6	573	1	12	64	3	70	272.01	77	424.26	277	295	268	15	45	0	0	3
5850	12208	8806	560	788	480	28188	1088	20	674	5	54	68	11	89	285.16	91	441.5	404	365	316	26	44	0	2	1
4418	13294	9648	851	458	256	28086	1236	7	617	1	12	58	5	72	273.85	95	428.4	315	312	297	13	39	0	0	4
14358	7618	4340	1	854	658	27904	603	323	1015	153	70	266	129	151	271.18	262	435.9	506	410	438	28	96	23	6	1
5388	12492	9026	586	790	470	28196	1084	11	647	1	30	51	6	91	284.23	105	441.01	397	336	344	21	28	1	0	4
5078	12916	9268	699	510	318	28116	1129	9	682	0	24	67	7	86	280.28	111	436.92	357	338	335	21	44	0	0	0
6938	11576	8164	381	896	576	28198	938	31	799	5	46	97	19	127	286.69	139	443.1	429	396	359	33	66	1	2	2
6984	11370	8134	397	998	662	28260	926	28	728	12	98	81	10	133	288.13	129	444.16	436	332	376	34	57	1	12	3
14338	7700	4440	4	750	560	27862	666	339	1097	164	74	261	140	154	269.74	262	432.62	455	476	434	23	79	13	0	1
4556	13166	9552	804	506	300	28106	1198	11	614	1	24	62	7	77	275.39	94	430.25	331	305	306	18	42	0	2	3
10730	7774	5586	0	2372	1848	28810	473	66	456	11	434	147	30	278	320.23	102	467.38	533	128	351	130	60	4	60	32
5194	12748	9200	633	632	354	28140	1099	5	692	0	12	69	3	100	279.19	104	435.21	368	339	335	21	49	0	0	1
4670	13078	9482	754	546	320	28120	1176	4	624	0	24	64	2	86	277.92	95	434.39	339	321	309	16	40	0	0	4
4316	13446	9732	893	360	198	28058	1261	8	627	1	4	46	4	61	270.98	95	423.22	299	301	306	17	34	0	2	2
4318	13450	9698	870	366	226	28070	1252	3	656	0	10	60	1	73	274.55	105	427.28	305	314	292	13	42	0	2	1
11160	7958	5538	4	2096	1528	28622	523	92	552	14	306	148	49	318	314.44	114	464.69	475	167	390	88	78	6	32	16
14180	7652	4368	8	940	722	27932	650	301	1004	154	68	247	119	147	270.35	266	432.5	513	417	420	18	79	15	2	0
3998	13630	9906	964	330	180	28054	1289	6	584	1	10	63	3	74	271.41	95	424.35	283	279	271	11	46	0	0	2
4938	12884	9344	680	600	344	28136	1126	4	650	0	22	70	1	98	277.95	97	433.47	362	335	323	15	50	0	2	2
4562	13266	9574	826	410	248	28082	1216	6	626	1	22	62	4	74	276.22	87	432.14	316	320	304	17	43	0	0	4
11490	8008	5380	2	1880	1446	28534	536	91	639	20	288	189	47	228	318	128	462.52	496	214	376	105	107	5	38	29
14220	7698	4428	8	832	636	27888	631	311	1075	157	72	267	120	148	269.9	303	433.77	491	404	430	25	99	15	0	2
5718	12282	8844	525	824	498	28204	1060	11	684	2	38	65	7	114	285.35	91	442.01	386	369	346	22	41	0	0	3
5548	12494	8950	568	698	448	28182	1062	15	704	2	38	63	10	96	285	118	442.27	403	362	342	24	35	0	6	2
10426	7780	5656	0	2514	1992	28922	497	55	413	10	474	141	24	296	331.23	83	469.43	499	138	324	127	62	0	68	42
3834	13736	9998	993	304	162	28040	1321	12	557	1	6	60	9	62	270.91	89	423.33	264	275	261	18	35	0	0	3
12922	7770	4798	3	1418	1108	28220	622	213	798	71	188	199	102	226	284.3	168	448.61	469	304	407	49	101	11	16	6
11922	7972	5210	3	1762	1304	28406	574	140	673	36	218	172	80	228	298.62	155	453.49	504	234	414	79	96	8	16	8
4196	13520	9802	900	346	186	28056	1261	8	599	1	6	63	6	68	272.86	98	426.14	299	298	289	15	43	0	0	1
4886	12968	9386	713	560	310	28124	1173	5	695	0	14	52	4	89	276.09	97	432.71	342	365	321	14	35	0	0	2
3912	13710	9966	956	284	156	28042	1288	4	585	1	14	61	1	80	270.19	81	423.2	272	292	284	9	39	0	0	1
14110	7572	4382	4	1006	782	27956	606	302	966	150	98	255	116	161	272.82	243	437.77	506	381	434	31	91	17	6	0
4670	13184	9504	769	446	272	28100	1196	3	654	0	24	63	1	91	277.4	93	433.61	321	339	308	11	45	0	0	4
4472	13332	9650	843	382	214	28066	1229	9	647	1	16	61	6	76	275.39	109	430.2	294	318	313	18	43	0	0	4

SUPPLEMENTARY INFORMATION:Monte Carlo Atomistic Simulation and Machine Learning Analysis of Na-K Eutectic Alloy in Condensed Phases, D. Reitz and E. Blaisten-Barojas, George Mason University, Fairfax, VA 22030

11746	7704	5308	1	2014	1442	28528	558	115	588	27	276	175	63	263	310.9	121	458.49	483	202	383	96	86	7	30	11
4576	13108	9520	760	574	334	28134	1191	0	630	0	22	61	0	84	276.61	104	432.5	326	331	309	24	37	0	0	2
12196	7968	5044	1	1630	1292	28380	573	136	718	33	222	174	70	235	299.93	163	453.66	503	248	408	60	88	10	26	17
14424	7664	4334	4	792	604	27872	647	333	1060	170	50	260	123	152	267.5	277	430.4	502	434	422	10	84	14	4	0
14264	7446	4366	3	1030	738	27932	612	326	959	170	84	274	116	170	273.17	230	437.42	488	372	419	28	97	18	4	2
5408	12482	9012	605	770	474	28186	1105	13	653	1	36	73	8	120	284.06	97	440.73	357	354	311	20	44	0	4	4
5880	12160	8704	539	816	564	28202	1034	27	650	8	72	71	15	108	286.4	102	442.73	399	352	351	26	42	1	4	5
4256	13480	9772	880	336	192	28056	1235	9	608	1	20	58	5	58	272.9	98	426.77	308	300	309	21	33	0	0	4
4464	13382	9648	848	336	208	28062	1213	5	654	2	24	65	1	67	274.05	113	428.43	321	307	311	16	39	0	0	3
4084	13664	9886	949	248	136	28026	1278	7	612	1	8	57	4	79	271.02	100	423.53	274	287	294	7	40	0	0	2
10416	7696	5638	0	2548	2034	28918	470	56	411	9	514	158	30	316	323.63	86	469.16	482	115	354	134	64	3	70	37
14430	7762	4366	1	680	548	27846	648	333	1131	160	54	261	124	153	270.04	284	432.63	472	459	439	16	91	21	6	2
4246	13452	9758	884	394	214	28068	1262	6	601	1	2	53	3	75	273.21	88	426.87	290	300	293	18	39	0	2	0
12910	7720	4856	0	1454	1084	28222	571	176	804	54	178	192	90	217	286.06	159	450.66	491	297	448	57	100	12	16	4
4992	12988	9324	687	488	300	28116	1124	6	706	0	20	55	4	77	276.92	122	433.6	374	348	346	17	33	0	4	2
7282	11362	8058	395	888	536	28184	960	50	776	16	56	95	24	137	288.55	128	444.61	410	395	358	26	51	2	2	2
6512	11760	8436	429	908	538	28198	981	31	735	5	42	77	21	121	286.49	116	441.47	421	373	366	25	49	0	2	1
3756	13772	10020	1009	318	178	28050	1317	9	541	1	6	52	6	59	270.02	96	422.99	270	251	275	18	34	0	0	2
13550	7828	4682	4	1100	786	28028	646	232	983	101	76	219	92	174	277.07	230	443.29	491	383	428	28	106	18	4	2
5190	12784	9258	647	570	298	28114	1114	3	713	0	12	84	2	91	281.32	110	437.53	363	358	323	20	59	0	2	1
5002	12862	9290	665	606	360	28148	1114	5	668	1	28	66	3	87	277.92	102	433.52	372	342	332	22	47	0	0	3
14498	7622	4344	4	768	566	27854	638	326	1073	164	52	267	134	165	270.9	266	433.31	464	431	441	16	93	16	4	2
4322	13316	9690	853	478	272	28098	1235	5	601	0	20	65	4	74	273.83	99	427.72	320	301	283	14	40	0	0	4
11554	7780	5378	2	1994	1478	28540	524	116	615	24	322	182	63	271	311.31	131	459.14	471	188	383	109	92	7	30	17
4582	13100	9478	752	580	370	28144	1178	6	596	0	34	60	1	98	277.37	90	433.33	336	311	298	20	39	0	0	0
4496	13282	9622	810	424	240	28082	1186	8	621	1	18	55	2	87	275.77	93	431.17	321	297	329	13	36	0	0	2
4582	13040	9516	745	636	354	28148	1173	1	613	0	20	53	1	96	276.3	93	432.03	341	305	317	15	37	0	0	0
14550	7572	4306	6	790	580	27854	638	353	1041	172	56	277	135	155	271.03	257	435.7	478	423	425	20	89	23	0	1
4494	13350	9614	811	366	228	28072	1226	9	647	0	20	59	5	80	275.75	103	431.2	311	336	299	10	36	0	0	5
13848	7758	4606	7	968	692	27954	634	269	1022	116	78	224	121	162	275.27	234	439.17	474	420	461	30	88	14	4	3
13642	7840	4664	8	1006	744	27992	613	275	958	122	92	228	112	166	275.41	248	441.11	498	359	446	34	92	21	4	1
4274	13448	9766	916	366	190	28048	1275	11	599	1	2	67	8	77	273.17	88	426.49	272	295	286	14	45	0	2	4
5288	12616	9098	592	712	430	28174	1114	5	678	1	28	76	3	103	284.74	116	440.33	387	368	298	15	41	0	2	1
5218	12766	9200	631	586	336	28126	1080	9	680	1	20	67	5	91	281.53	111	437.82	378	337	357	17	37	0	0	4
4102	13550	9854	928	348	186	28050	1273	9	594	2	10	56	5	75	270.61	79	423.28	276	299	298	13	36	0	0	2
3966	13660	9906	953	320	190	28056	1294	10	577	2	14	55	4	57	271.01	95	423.08	291	284	275	19	33	0	0	1

SUPPLEMENTARY INFORMATION:Monte Carlo Atomistic Simulation and Machine Learning Analysis of Na-K Eutectic Alloy in Condensed Phases, D. Reitz and E. Blaisten-Barojas, George Mason University, Fairfax, VA 22030

4826	13090	9426	728	470	276	28104	1173	7	681	0	14	51	5	80	275.52	104	431.88	343	354	331	12	36	0	0	4
13416	7716	4674	4	1230	914	28082	586	250	878	107	120	221	102	195	278.2	198	442.16	498	317	440	36	89	15	12	6
10710	7770	5540	1	2432	1892	28824	490	80	473	14	412	169	43	312	323.47	80	467.96	455	139	365	131	80	4	62	34
4230	13500	9778	902	344	194	28058	1256	7	620	2	12	59	3	63	272.81	115	425.76	315	290	286	13	39	0	0	1
4202	13440	9762	912	422	240	28078	1277	6	586	2	12	60	3	75	272.82	80	426.05	289	287	284	10	37	0	0	4
4444	13240	9636	805	488	272	28102	1204	4	620	0	22	55	4	89	277.04	71	431.27	306	315	318	17	40	0	0	3
4842	13050	9426	748	492	274	28096	1188	9	677	1	12	67	4	91	276.57	100	432.31	329	347	305	14	49	1	0	0
13180	7910	4770	1	1208	926	28130	609	202	907	75	118	195	100	201	284.5	200	447.88	483	346	452	40	91	11	18	4
6040	12036	8610	486	890	594	28240	1029	14	676	1	58	62	10	126	286.34	94	441.2	396	360	350	26	36	1	12	3
14312	7762	4412	3	738	578	27866	619	329	1089	159	60	246	129	143	267.71	302	430.29	505	420	457	18	76	13	4	1
12880	7926	4858	2	1338	1036	28212	612	172	865	55	156	199	85	220	291.14	163	449.77	446	325	428	59	109	13	18	10
4190	13476	9786	924	392	214	28064	1288	9	586	1	6	56	6	67	272.35	88	426.42	278	286	287	17	37	0	0	2
5988	12394	8826	545	558	314	28092	1026	21	782	6	12	80	10	102	281.23	126	439.59	400	379	366	13	51	0	0	2
12228	7866	5094	3	1656	1262	28368	562	134	719	31	240	182	66	254	300.13	146	454.01	476	249	405	77	92	11	22	7
4324	13422	9706	870	374	226	28072	1257	6	621	0	20	51	5	73	273.36	90	427.45	294	318	302	14	36	0	0	3
11792	7900	5238	0	1876	1398	28488	551	115	661	30	266	188	57	263	305.36	130	453.97	483	227	390	75	99	11	18	20
5758	12438	8878	580	636	398	28144	1082	23	710	8	36	68	12	107	281.67	124	439.84	380	361	339	14	41	0	0	2
14140	7624	4376	5	984	752	27952	630	310	965	154	66	244	122	164	272.83	223	435.98	483	392	448	26	80	16	8	2
11684	7896	5252	1	1894	1472	28540	558	99	654	26	318	186	49	251	308.05	142	458.89	526	230	345	83	96	7	22	15
11984	7882	5208	0	1758	1312	28424	581	128	699	38	264	182	73	254	302.11	140	453	480	267	399	65	94	5	14	13
5028	12952	9290	667	516	314	28116	1116	2	700	0	14	70	2	84	279.96	116	436.76	376	350	334	14	47	0	2	2
4184	13452	9804	894	400	212	28066	1240	12	577	2	14	58	6	73	273.94	86	428.68	309	279	294	12	40	0	0	4
14410	7592	4322	3	832	656	27902	637	334	1020	168	80	266	122	174	273.15	253	437.82	472	411	423	19	85	21	10	1
4042	13576	9870	943	364	198	28056	1279	9	594	1	6	58	5	77	272.54	91	426.68	275	269	289	14	43	0	0	2
5730	12470	8908	595	610	378	28130	1087	20	726	8	32	81	11	91	282.51	112	438.35	378	366	332	25	49	0	2	1
13404	7810	4700	12	1150	874	28072	637	263	903	100	126	212	120	202	278.06	186	444.88	444	343	452	42	91	24	8	1
4318	13270	9670	835	530	304	28112	1221	3	603	1	20	64	1	77	274.04	101	428.85	332	299	284	15	41	0	0	2
5740	12320	8918	560	722	412	28156	1037	16	685	3	42	66	5	119	287.51	93	442.42	377	347	363	24	44	0	2	1
4780	13172	9480	757	402	230	28078	1187	6	672	1	14	52	3	81	277.19	103	433.04	323	343	335	14	37	0	0	2
13934	7826	4530	6	914	680	27940	620	275	1042	124	48	243	119	168	276.09	258	440.76	485	397	446	22	98	15	8	5
4346	13330	9646	829	468	292	28104	1205	7	596	0	20	56	4	75	273.71	91	428.92	335	298	303	14	29	0	2	4
11064	7836	5408	1	2218	1748	28698	522	80	534	20	376	165	37	272	323.35	93	462.89	467	182	372	119	75	3	40	32
13968	7614	4494	2	1060	762	27988	604	275	938	111	88	227	125	194	277.35	207	445.73	457	357	469	31	89	17	2	4
4428	13280	9632	833	464	268	28088	1231	9	615	1	16	56	4	82	275.59	107	430.46	306	310	297	15	31	0	0	3
5666	12522	8966	569	596	350	28126	1078	10	748	2	26	69	8	87	283.53	120	439.36	389	391	345	19	47	0	0	3
4754	13186	9466	738	432	250	28090	1155	4	685	1	2	63	2	70	277.11	129	432.45	360	331	329	19	33	0	0	0

SUPPLEMENTARY INFORMATION:Monte Carlo Atomistic Simulation and Machine Learning Analysis of Na-K Eutectic Alloy in Condensed Phases, D. Reitz and E. Blaisten-Barojas, George Mason University, Fairfax, VA 22030

6224	11946	8592	500	838	518	28176	1025	35	699	11	50	86	19	112	281.15	113	440.28	393	365	346	32	48	1	8	0
5908	12296	8784	525	702	434	28162	1022	15	744	3	38	68	7	111	283.98	122	441.17	406	368	367	18	44	0	0	1
5772	12414	8896	574	650	380	28138	1070	17	735	1	24	71	11	113	280.91	115	438.53	364	376	348	21	45	0	2	2
4630	13190	9538	793	458	264	28098	1209	4	653	0	16	61	4	83	278.01	104	432.76	314	320	309	19	38	0	0	1
5178	12866	9222	671	520	310	28112	1151	9	696	3	16	60	4	88	281.91	106	437.36	349	365	328	17	35	0	0	1
3976	13640	9912	962	338	182	28052	1317	6	596	1	4	61	3	75	271.33	75	424.45	251	295	270	19	48	0	0	1
13030	7804	4744	2	1354	1072	28186	618	197	855	87	168	201	74	205	282.24	191	446.06	498	338	413	42	93	14	12	4
4678	13192	9536	790	422	232	28072	1188	13	631	1	12	64	9	81	276.38	91	429.68	319	321	321	19	40	0	0	1
4272	13448	9726	869	382	230	28074	1245	8	612	1	16	61	5	68	274.57	101	427.89	310	304	293	14	41	0	0	4
13130	7754	4742	0	1372	1028	28182	571	208	835	65	142	191	102	199	283.71	183	449.19	517	296	448	39	98	11	14	10
4064	13594	9878	967	316	176	28044	1298	7	593	1	16	68	4	67	271.62	107	423.73	273	277	266	17	44	0	0	3
12386	7892	5052	4	1610	1182	28326	560	162	734	40	188	180	86	240	299.41	139	451.58	463	258	443	67	91	11	14	16
6666	11622	8368	386	960	552	28216	945	23	761	7	46	83	13	114	287.13	124	444.95	451	374	370	25	55	1	2	5
14354	7634	4352	4	860	638	27892	632	319	1032	153	50	251	129	133	272.03	266	434.95	505	404	443	29	91	20	4	0
4870	13072	9414	724	472	262	28096	1135	9	675	0	6	60	6	84	276.43	103	432.64	347	330	347	18	46	0	0	3
4740	13018	9448	710	572	324	28120	1163	7	645	1	18	52	5	86	280.24	84	435.83	331	350	335	25	26	0	0	1
5302	12658	9108	606	650	400	28152	1067	16	669	5	34	66	8	98	280.12	98	438.17	379	354	351	26	39	1	0	3
4856	13038	9426	715	510	270	28104	1153	5	685	0	4	75	5	73	276.91	110	432.65	372	344	310	14	54	0	0	2
4068	13618	9870	946	310	174	28050	1286	12	575	1	10	52	9	63	270.27	89	423.04	284	280	290	17	33	0	0	1
13872	7738	4520	5	992	758	27974	622	289	970	131	90	235	114	203	274.97	213	440.91	462	382	436	20	91	16	2	3
4724	13160	9492	783	432	256	28086	1212	12	671	2	22	67	10	91	276.11	101	431.81	306	347	303	14	41	0	0	2
4024	13538	9838	930	416	248	28078	1295	4	569	0	14	63	3	82	270.83	71	424.57	263	296	273	18	42	0	0	2
5172	12816	9202	661	556	350	28130	1122	6	693	0	32	60	3	90	278.64	112	434.26	363	343	335	15	34	0	2	5
5936	12160	8726	487	814	516	28210	1041	9	744	1	54	71	5	112	286.24	114	442.39	405	411	336	24	45	0	2	3
4358	13432	9690	844	362	216	28070	1234	9	631	2	12	64	3	83	273.76	97	426.43	304	320	298	13	40	0	0	0
4164	13440	9796	900	434	232	28074	1263	6	585	1	6	73	3	73	276.08	88	429.8	286	285	279	22	54	0	2	2
5176	12764	9224	644	598	338	28120	1126	10	699	4	20	66	3	89	279.55	109	436.45	377	352	310	16	40	0	0	1
12292	8002	5004	2	1568	1254	28352	548	148	732	33	208	164	74	236	299.27	170	451.54	503	240	426	72	83	7	18	10
4682	13058	9490	767	564	310	28118	1179	5	648	0	14	72	3	86	278.25	102	434.98	339	311	300	17	47	0	0	3
4886	13014	9384	720	502	298	28106	1156	5	671	0	20	76	1	89	279.1	87	435.31	351	340	305	15	51	0	2	2
14252	7658	4422	8	850	640	27904	655	341	1025	161	80	252	135	147	271.53	262	435.8	490	418	424	23	84	22	2	1
13796	7866	4584	7	932	706	27958	616	291	1011	135	72	239	125	173	274.73	250	438.26	481	396	454	29	92	12	2	0
13652	7818	4572	8	1066	824	28030	676	247	953	115	92	215	98	170	276.08	219	438.1	474	397	426	28	73	16	6	3
11614	7912	5250	1	1856	1536	28582	513	112	615	29	378	194	58	282	314.49	130	458.34	495	212	365	102	102	8	34	16
11650	7922	5330	5	1898	1404	28496	574	109	677	28	254	171	62	269	302.95	136	453.87	487	244	380	80	94	3	36	9
14364	7614	4344	3	844	650	27892	658	336	1043	162	70	264	134	160	270.04	239	431.35	460	444	422	26	88	19	6	2

14086	7672	4468	11	920	690	27918	633	299	1000	152	74	258	107	168	271.29	261	436.22	484	403	426	21	87	22	8	1
11888	7878	5160	6	1812	1414	28456	569	131	675	22	276	167	79	263	304.53	120	456.62	474	230	413	76	94	5	26	10
5180	12832	9226	678	546	316	28116	1108	5	707	0	14	64	5	85	278.67	113	435.34	377	338	343	17	43	0	2	1
12500	7848	4980	3	1598	1180	28304	567	159	736	38	192	187	80	236	293.4	153	452.35	509	271	410	57	101	12	6	5
13598	7956	4664	4	938	734	27980	632	254	1045	126	80	231	101	187	273.81	232	439.76	459	420	448	30	89	8	10	1
4148	13600	9836	924	292	160	28040	1288	8	619	1	4	75	5	69	270.85	90	423.34	277	311	271	13	50	0	0	4
5970	12264	8730	507	716	456	28176	1046	18	728	2	38	81	12	119	285.16	106	441.13	388	388	338	20	51	0	2	2
14290	7538	4392	1	954	676	27922	621	317	1015	155	68	260	124	159	274.46	239	437.72	503	411	430	21	94	14	4	1
4812	12998	9420	729	544	316	28118	1138	4	665	0	28	68	2	100	276.86	89	433.81	332	328	340	19	43	0	0	2
5838	12354	8864	557	674	386	28140	1051	13	742	4	22	78	7	111	280.46	126	439.29	372	351	359	20	48	0	2	4
4092	13480	9818	908	436	244	28082	1276	10	563	2	12	48	5	59	271.3	75	424.61	306	293	282	17	29	0	0	3
4858	12960	9374	704	576	342	28138	1120	3	643	1	28	49	2	92	278.62	89	432.55	364	325	352	16	29	0	0	2
11772	7994	5268	5	1792	1350	28446	585	127	680	25	244	170	66	274	302.54	131	455.35	465	261	399	59	90	16	26	18
4686	13070	9488	774	558	306	28118	1206	13	624	0	8	54	10	77	276.58	78	433.16	331	329	305	20	42	0	2	1
3848	13728	9974	974	306	180	28050	1293	4	562	1	12	64	2	73	270.68	65	424.7	260	274	283	18	51	0	2	4
11284	7944	5494	1	2028	1494	28586	508	89	584	9	304	160	50	291	317.83	101	464.41	458	185	410	102	87	5	34	22
6060	11946	8586	501	922	632	28246	1018	27	655	7	94	78	15	138	284.63	108	441.57	400	332	329	23	40	2	6	4
13906	7796	4536	4	928	704	27948	627	299	1011	140	74	247	128	177	274.71	237	440.46	469	415	447	23	95	13	2	4
12798	7878	4854	4	1386	1104	28226	602	181	835	64	186	182	81	224	287.46	179	448.64	481	316	435	45	82	14	20	8
12646	7804	5034	2	1516	1070	28266	594	178	784	46	182	190	89	230	293.78	140	453.14	455	297	431	61	99	13	12	13
11412	8090	5320	0	1870	1508	28532	521	97	645	22	294	181	40	284	313.56	121	457.5	468	214	380	100	103	9	34	16
5652	12524	8982	586	608	344	28130	1083	11	742	0	20	76	9	95	284.63	119	440.01	378	373	336	22	51	0	0	1
4592	13208	9558	783	452	264	28098	1212	1	661	0	24	61	1	92	275.11	84	433.57	301	341	313	15	45	0	0	3
7386	11394	7944	341	836	562	28182	892	42	802	14	56	98	19	129	287.43	155	447.28	463	393	375	27	57	2	4	0
6234	12084	8612	497	736	462	28180	1021	25	722	4	48	75	18	110	284.78	134	442.67	409	358	354	20	43	0	4	4
11476	8062	5324	1	1854	1484	28542	546	95	663	17	306	170	57	263	304.95	139	457.11	501	232	377	87	100	4	34	19
14152	7726	4414	7	880	678	27916	631	305	1025	154	64	247	122	161	270.53	266	430.52	494	408	440	17	84	12	2	2
4898	12990	9384	716	518	300	28114	1133	5	676	0	24	58	4	78	278.76	109	435.15	364	325	344	17	38	0	0	3
4936	12904	9312	679	618	362	28146	1136	3	653	1	14	50	2	84	279.27	93	435.43	358	341	345	22	32	0	0	2
4014	13642	9914	960	298	160	28040	1289	8	591	1	12	54	6	69	270.79	92	423.44	275	279	295	13	42	0	0	1
11756	7964	5230	2	1798	1430	28516	577	96	651	15	308	156	56	278	310.66	120	458.43	467	234	382	85	97	6	28	12
11394	8002	5402	0	1956	1478	28550	509	86	639	19	280	182	47	264	310.23	124	458.54	487	198	412	100	102	5	36	17
4574	13240	9558	779	436	262	28090	1164	9	625	0	20	53	7	85	278.47	98	434.25	332	306	344	15	33	0	0	2
5338	12738	9182	647	534	288	28096	1127	19	724	3	16	64	12	91	279.92	100	435.48	352	385	342	15	45	0	0	2
5212	12748	9192	660	610	354	28140	1127	3	689	1	24	72	2	99	280.98	108	437.76	370	359	311	15	47	0	0	0
13846	7860	4574	7	902	694	27958	685	280	1027	127	72	233	116	172	275.5	244	440.88	451	427	419	27	96	25	10	1

SUPPLEMENTARY INFORMATION:Monte Carlo Atomistic Simulation and Machine Learning Analysis of Na-K Eutectic Alloy in Condensed Phases, D. Reitz and E. Blaisten-Barojas, George Mason University, Fairfax, VA 22030

11682	7818	5290	1	1946	1456	28522	554	117	625	18	308	165	59	295	306.59	119	455.66	484	203	380	69	97	11	20	13
4278	13550	9774	907	268	154	28034	1272	11	644	2	10	63	5	60	271.29	101	424.82	297	319	285	15	42	0	0	1
4322	13402	9720	882	400	218	28070	1249	7	609	1	8	60	3	69	273.32	98	427.97	309	290	292	14	39	0	0	2
12462	7894	5074	6	1564	1104	28270	566	169	777	55	158	198	80	228	291.59	167	453.05	491	274	419	64	95	8	14	7
5666	12436	8934	548	690	404	28158	1054	8	730	1	28	69	7	94	284.14	116	441.03	408	380	345	19	41	0	0	3
4756	13076	9452	727	516	296	28112	1150	5	646	0	16	61	3	81	278.81	110	434.38	366	334	323	12	41	0	0	2
13880	7702	4548	9	1022	738	27970	632	284	985	135	72	245	117	173	274.16	235	439.4	482	387	430	27	101	11	8	2
5030	12894	9284	738	558	334	28120	1206	6	658	0	18	53	2	93	280.42	98	437.81	316	350	303	17	39	0	2	2
4346	13396	9680	864	404	242	28082	1227	7	615	1	14	50	3	60	273.06	101	425.95	331	294	306	18	37	0	0	1
7226	11398	7994	356	928	610	28212	953	36	811	9	52	96	26	147	288.72	135	443.4	406	409	361	26	59	0	4	2
5844	12328	8810	555	706	440	28166	1081	16	731	0	36	63	9	101	281.32	119	437.34	398	383	331	17	47	0	2	0
12492	7836	5008	2	1620	1162	28302	577	165	767	51	178	184	87	249	293.08	159	451.82	471	274	428	63	100	9	6	4
10418	7830	5722	0	2504	1900	28874	514	61	412	8	444	162	39	298	332.87	65	470.16	463	139	343	140	69	2	54	34
4730	13076	9486	740	512	284	28108	1171	9	652	1	18	63	5	82	277.97	108	433.94	342	328	316	17	44	0	2	3
11216	7864	5564	0	2102	1512	28628	534	79	578	10	334	162	41	285	320.24	97	468.69	429	189	391	115	88	1	34	28
5924	12456	8866	563	510	304	28088	1063	32	786	8	26	75	17	96	281.79	140	438.17	377	402	363	15	49	1	2	3
4170	13486	9770	912	396	238	28074	1275	8	569	0	14	69	3	71	274.28	87	428.51	294	289	260	18	50	0	0	3
13172	7754	4788	7	1350	956	28150	610	206	847	70	124	192	106	196	284.04	185	447.71	495	322	440	40	96	8	6	6
3912	13742	9960	985	268	148	28034	1308	7	590	0	4	63	5	55	270.28	99	422.68	285	283	267	14	40	0	0	2
4280	13294	9720	877	514	274	28096	1231	7	578	1	14	64	5	78	275.14	78	431.13	313	281	290	16	45	0	0	3
14114	7816	4478	9	818	616	27890	642	309	1053	156	44	264	116	153	267.67	280	430.3	493	419	427	16	94	22	4	2
13430	7810	4622	2	1186	922	28094	622	233	900	97	120	207	99	184	279.33	203	444.72	482	333	452	44	85	17	2	1
14586	7602	4308	6	756	546	27838	626	348	1077	177	36	262	127	141	267.3	277	430.08	501	426	444	17	76	19	4	0
4338	13264	9654	819	532	310	28120	1232	7	586	0	22	62	6	80	277.26	84	432.83	324	308	281	15	36	1	0	2
5304	12624	9162	637	646	366	28140	1095	11	670	1	34	69	5	98	281.18	105	437.56	372	342	334	21	47	0	4	2
4446	13386	9664	828	360	198	28058	1184	10	635	2	4	56	6	74	273.16	95	428.07	323	296	339	16	32	0	0	2
11742	7924	5282	3	1854	1392	28490	545	107	663	24	264	189	59	273	310.61	126	458.9	460	232	397	82	108	6	28	22
4574	13232	9588	807	432	238	28080	1193	9	627	0	16	62	7	75	275.14	103	431.28	324	310	317	20	45	0	0	2
3894	13664	9938	942	362	200	28062	1263	4	565	0	4	49	3	68	271.41	97	424.21	296	261	300	12	36	0	0	4
4928	12956	9374	717	538	298	28110	1166	8	678	1	16	66	5	89	278.58	95	435.02	339	349	315	16	45	0	0	3
4926	12918	9336	712	596	346	28142	1168	5	654	0	20	53	5	77	278.28	105	434.96	355	344	321	16	32	0	0	6
4960	12992	9386	706	478	258	28088	1126	10	697	1	14	68	7	86	280.99	114	437.34	358	341	338	15	48	0	0	2
10848	7922	5562	0	2262	1728	28740	552	74	543	11	366	152	41	272	317.34	90	462.13	456	177	368	118	89	6	46	38
7794	10906	7712	309	1050	682	28246	892	48	777	18	92	98	22	140	288.95	152	445.44	456	374	372	33	56	4	10	1
5722	12548	8936	585	554	332	28110	1084	15	753	4	18	70	8	94	278.87	128	437.18	376	388	353	17	50	1	0	1
11496	8012	5284	2	1882	1524	28554	524	91	655	17	316	185	51	263	304.44	137	457.09	502	212	380	90	96	7	38	22

SUPPLEMENTARY INFORMATION:Monte Carlo Atomistic Simulation and Machine Learning Analysis of Na-K Eutectic Alloy in Condensed Phases, D. Reitz and E. Blaisten-Barojas, George Mason University, Fairfax, VA 22030

12366	7972	5118	2	1524	1108	28282	569	154	787	39	180	165	73	241	292.89	169	451.61	499	281	442	47	85	12	14	6
11842	7786	5240	0	1932	1412	28510	552	124	631	25	272	152	64	273	310.31	117	458.39	470	223	417	83	85	8	22	16
11600	7798	5258	0	2044	1548	28586	514	109	593	26	300	168	59	290	317.65	110	461.82	451	190	422	106	85	9	36	16
4372	13416	9694	881	360	208	28062	1277	9	624	1	12	61	7	81	274.88	83	430.13	273	332	290	11	41	0	0	4
3848	13668	9958	1002	366	210	28064	1311	5	543	1	14	58	3	66	270.56	85	422.04	271	249	269	19	39	0	0	1
6530	11840	8458	488	782	486	28154	983	37	730	11	52	94	22	128	285.86	122	441.13	377	344	369	27	55	0	6	9
14430	7700	4392	5	730	538	27838	619	316	1121	164	46	261	119	121	268	298	429.71	519	449	453	21	89	11	2	1
11216	7798	5568	3	2162	1518	28622	521	84	583	17	336	181	41	288	321.21	105	462.85	486	192	357	105	90	2	24	19
12726	7844	4794	1	1466	1218	28288	563	180	786	56	218	186	86	235	293.08	167	452.35	512	294	414	47	93	10	22	12
12006	8002	5174	2	1696	1276	28390	584	120	737	28	222	157	69	241	299.61	137	450.26	479	279	437	67	79	5	14	11
5010	12966	9366	737	470	256	28088	1178	6	693	1	20	64	3	86	275.94	106	430.19	328	356	323	16	42	0	0	1
14494	7562	4306	6	830	620	27878	630	339	1031	157	62	244	141	171	272.35	260	436.18	466	417	464	15	76	18	4	1
4724	13088	9472	762	514	296	28114	1203	6	651	1	18	52	3	85	277.71	104	434.63	319	334	312	19	29	0	2	2
11622	7748	5330	1	1998	1488	28546	533	111	598	19	334	167	56	273	316.18	103	463.22	453	201	407	102	89	4	24	25
5562	12584	8998	595	592	366	28134	1075	12	738	2	32	71	7	102	278.43	126	436.48	382	371	347	13	42	0	0	4
7216	11294	8020	363	992	628	28224	913	42	747	14	74	95	22	135	290.52	119	446.55	424	361	383	33	48	1	0	8
4120	13568	9822	913	346	198	28060	1255	9	589	0	6	55	5	70	272.48	96	424.78	298	277	296	13	39	0	0	4
14112	7766	4446	7	864	666	27922	659	322	1009	150	66	262	122	167	274.94	242	438.59	468	418	407	27	94	19	2	0
3930	13654	9914	950	344	206	28064	1287	6	565	1	16	51	4	76	271.27	81	423.13	277	282	286	13	37	0	0	2
10774	7900	5602	2	2238	1760	28752	520	61	498	8	416	156	27	309	323.83	81	470.51	461	172	354	112	72	3	60	30
13752	7806	4598	2	1000	746	27994	650	259	991	119	90	230	112	157	276.64	232	440.19	500	406	428	28	91	14	2	1
8442	10618	7300	235	1062	730	28236	819	64	800	17	82	99	36	165	288.07	141	446.84	445	362	415	39	63	3	2	2
4638	13196	9538	798	450	256	28094	1205	10	647	1	16	58	6	67	278.9	97	434.26	342	333	302	12	38	0	0	6
4124	13474	9816	914	420	230	28076	1256	8	578	1	10	56	6	81	272.1	87	425.19	290	276	298	13	37	0	2	2
6270	12080	8566	515	720	476	28166	1034	35	717	12	52	82	19	118	285.34	108	441.49	389	362	344	21	47	0	2	2
11816	7700	5244	2	2006	1450	28518	534	118	607	19	264	162	71	256	305.85	118	456.92	506	209	410	81	97	7	34	16
4414	13336	9652	845	418	246	28084	1248	12	626	2	16	58	5	71	275.86	94	429.19	310	318	289	17	43	0	2	1
11720	8038	5230	0	1818	1404	28480	601	99	708	17	238	153	52	245	303.83	141	456.73	468	253	400	84	87	7	30	18
4442	13340	9638	842	404	238	28076	1233	7	631	2	14	69	4	67	274.29	93	428.7	319	323	284	18	45	0	0	3
5046	12952	9310	681	494	284	28098	1116	10	681	0	12	73	8	86	277.39	104	433.7	364	342	338	18	50	0	0	1
12156	7840	5058	0	1708	1352	28410	567	140	706	39	272	188	72	251	299.11	156	452.94	481	253	393	74	91	10	20	15
3964	13724	9932	976	256	148	28032	1288	7	598	1	8	63	4	61	271.17	106	424.24	289	269	281	9	46	0	0	4
12542	7910	4950	2	1538	1156	28272	592	152	798	47	162	197	80	220	288.12	167	449.2	492	276	420	51	112	10	12	11
5498	12486	9014	609	708	418	28160	1099	10	693	3	36	67	6	101	284.6	97	440.4	370	359	331	24	47	0	0	1
14518	7674	4382	8	680	506	27820	612	354	1136	168	60	271	137	146	267.83	296	429.42	494	451	452	16	96	16	0	1
10990	7972	5560	1	2136	1616	28660	493	73	560	12	344	166	36	291	317.93	98	465	480	174	385	110	81	3	34	28

SUPPLEMENTARY INFORMATION:Monte Carlo Atomistic Simulation and Machine Learning Analysis of Na-K Eutectic Alloy in Condensed Phases, D. Reitz and E. Blaisten-Barojas, George Mason University, Fairfax, VA 22030

14392	7534	4372	4	870	634	27882	625	334	1025	166	72	275	124	175	272.78	228	436.94	447	414	439	26	99	21	8	5
6176	12022	8656	470	820	472	28188	1027	29	718	9	42	61	16	111	286.49	107	442.76	402	393	361	21	30	1	0	5
4158	13526	9788	916	352	222	28070	1273	6	595	1	24	54	4	78	273.47	89	427.4	289	297	289	11	34	0	0	1
3906	13674	9948	979	334	184	28056	1308	7	576	2	10	69	4	63	269.95	101	421.46	289	270	256	11	43	0	0	2
12986	7722	4770	2	1440	1108	28220	606	201	814	67	176	187	101	209	283.92	185	450.33	503	298	425	46	100	12	14	5
5648	12394	8920	579	720	438	28158	1058	23	685	9	34	77	8	105	282.04	112	439.05	394	353	340	20	39	2	2	1
13208	7814	4754	4	1268	944	28122	567	238	865	83	124	215	108	223	281.33	202	446.43	489	311	445	34	106	20	8	6
12034	7886	5100	1	1758	1376	28442	549	134	656	32	262	163	68	242	303.66	137	455.19	495	246	416	80	87	10	24	19
5412	12546	9062	593	690	410	28156	1081	8	699	0	32	77	4	93	284.86	121	441.23	391	356	324	22	54	0	4	4
14126	7540	4404	4	1018	768	27964	634	325	947	153	104	235	130	164	273.79	231	437.78	468	387	453	37	71	20	4	1
11776	7916	5254	0	1840	1388	28460	541	126	658	25	250	174	71	250	302.23	130	457.59	459	214	433	91	94	9	36	17
4420	13328	9640	807	438	254	28092	1211	5	615	1	12	54	2	78	273.7	94	427.98	313	317	320	17	35	0	0	3
13050	7790	4738	4	1394	1062	28188	617	187	853	80	144	203	82	211	283.76	170	446.8	484	323	423	46	102	11	10	3
5206	12824	9222	658	522	308	28108	1156	9	710	1	26	68	4	91	280.46	97	438.07	348	390	314	14	50	1	0	2
11388	7976	5468	2	1980	1434	28554	583	86	640	10	278	157	38	267	313.5	128	457.66	474	214	366	89	95	10	30	20
12280	7812	5004	1	1684	1322	28376	536	187	670	51	252	188	90	261	302.6	143	453.98	499	241	396	65	100	9	22	13
4948	12956	9324	692	540	328	28116	1138	5	690	2	20	79	3	93	278.54	103	434.92	354	346	313	17	50	0	0	1
12204	7852	5254	1	1616	1162	28370	601	133	752	34	264	196	63	241	305.92	153	456.09	472	267	369	69	119	9	18	16
5046	12938	9308	692	506	294	28108	1150	7	702	0	10	62	4	62	279.36	129	437.49	374	369	319	22	39	0	6	3
12970	7922	4778	4	1326	1034	28178	650	186	897	62	142	186	87	197	282.84	215	447.01	493	343	405	39	97	14	6	6
4388	13428	9682	850	350	206	28064	1237	6	631	1	10	63	3	60	274.55	106	429.47	319	324	298	15	47	0	0	4
6000	12268	8762	544	670	404	28136	1050	27	730	10	30	75	13	107	281.92	114	438.01	391	373	352	18	49	0	2	0
14292	7620	4382	4	882	658	27904	633	327	1023	172	64	277	111	151	272.02	252	436.46	496	412	404	29	91	19	4	1
4806	13140	9446	758	440	250	28086	1166	9	680	1	4	69	8	71	276.96	113	431.31	351	328	316	19	55	0	0	2
4418	13384	9660	863	374	220	28068	1251	9	642	1	12	54	3	72	274.1	104	429.4	306	321	293	13	41	0	0	2
3916	13662	9938	976	338	190	28056	1304	7	564	1	12	59	3	63	271.38	91	423.66	280	270	270	16	40	0	0	2
13882	7754	4490	8	982	778	27988	631	271	977	119	92	224	105	194	277.82	225	444.41	453	375	457	26	89	19	10	3
3780	13742	10002	978	328	192	28060	1296	4	556	1	16	62	3	61	270.71	91	422.66	290	268	270	15	39	0	0	1
4536	13192	9582	828	496	276	28098	1222	11	609	2	16	66	6	77	274.46	91	429.84	323	306	287	18	45	0	0	1
3794	13726	9986	997	342	200	28060	1324	9	539	1	12	53	4	82	271.46	73	424	255	266	260	12	34	0	0	2
11580	8074	5278	3	1864	1432	28498	581	101	668	21	244	152	61	228	299.07	140	453.1	510	232	408	80	84	8	24	16
11952	8066	5166	2	1646	1312	28418	562	128	717	28	254	173	72	257	301.36	148	454.36	478	234	416	71	91	5	22	12
4352	13422	9662	837	394	246	28086	1211	6	610	1	10	56	4	73	274.66	102	429	328	295	311	14	37	0	0	2
3998	13702	9928	985	260	138	28030	1317	6	595	1	4	60	4	67	270.54	89	422.88	261	291	278	13	40	0	0	1
11790	8066	5284	4	1716	1312	28442	544	128	700	33	248	179	75	262	303.46	132	454.68	478	251	415	79	92	3	22	14
3910	13674	9936	965	346	188	28054	1291	8	565	1	0	56	6	70	269.96	76	423.49	280	263	282	14	40	0	0	1

4886	12940	9376	729	576	326	28124	1180	7	651	0	20	64	4	88	276.77	98	433.96	342	326	305	17	42	0	0	0
11168	8038	5466	1	2008	1572	28632	517	74	591	19	334	199	29	289	314.94	113	463.16	491	193	353	94	109	6	44	27
5466	12426	8998	589	796	468	28188	1115	10	669	1	32	79	8	117	285.25	78	441.55	354	369	302	23	52	0	2	3
14340	7676	4402	7	792	592	27864	652	338	1051	178	58	275	118	170	269.45	259	431.87	460	442	417	16	88	16	4	2
4716	13164	9500	772	440	252	28088	1173	4	657	0	16	67	3	88	276.25	103	432.79	326	320	321	17	45	0	0	2
13640	7968	4746	4	880	634	27944	642	287	1046	116	74	238	135	175	275.71	250	438.47	465	434	438	26	104	14	2	2
3986	13606	9870	937	372	224	28070	1288	4	578	0	10	59	3	71	271.28	94	423	281	272	273	18	39	0	2	2
12300	7910	5048	3	1584	1240	28332	554	158	756	41	220	197	80	238	292.42	178	450.4	498	257	402	69	117	10	28	11
4544	13250	9574	814	456	260	28092	1207	8	613	2	8	59	3	86	275.51	102	431.91	323	306	309	11	38	0	0	1
14526	7584	4280	4	824	612	27870	628	340	1025	173	34	273	122	144	269.32	266	431	504	412	422	21	90	22	10	1
4726	12990	9438	742	600	354	28138	1165	7	620	0	26	63	6	91	277.7	93	434.69	352	315	305	18	42	0	4	0
4072	13450	9806	918	474	276	28096	1281	8	565	2	16	54	4	74	270.97	86	423.6	292	281	273	18	33	0	2	1
11224	7836	5314	0	2172	1730	28680	533	84	552	20	348	169	39	291	315.85	104	461.48	507	184	342	96	85	6	50	25
13988	7792	4530	2	880	666	27938	603	288	1030	126	80	220	132	151	275.79	266	440.93	494	387	481	34	79	15	2	2
12960	7822	4844	4	1398	1016	28182	624	196	854	65	128	210	94	195	284.82	175	450.23	487	329	416	54	118	21	14	3
4066	13534	9798	915	416	262	28092	1274	3	554	1	16	66	1	83	275.44	79	428.86	294	272	266	10	36	0	0	3
4256	13412	9726	881	428	248	28082	1256	6	590	1	12	60	3	82	272.74	90	425.34	294	284	282	14	38	0	0	3
11372	7914	5436	1	2024	1492	28568	485	94	605	17	296	169	51	268	317.04	127	459.73	492	178	412	102	96	2	32	27
4096	13604	9856	935	314	174	28052	1292	7	596	0	8	51	5	81	271.81	82	425.68	266	297	294	9	38	0	0	2
11800	7934	5268	1	1866	1344	28448	557	145	665	34	208	171	80	267	304.37	139	453.89	479	236	405	78	90	3	26	9
4622	13056	9488	759	594	350	28136	1176	7	620	1	24	51	4	90	277.97	83	433.9	332	311	325	18	35	0	2	3
10988	7994	5526	1	2160	1640	28678	475	72	537	8	312	159	34	263	320.83	118	461.02	515	147	403	109	80	6	56	31
4520	13284	9586	839	420	254	28080	1231	6	632	1	14	55	4	79	275.2	88	429.41	302	308	307	17	42	0	2	2
11218	7974	5482	0	1992	1560	28634	526	82	602	12	360	181	45	263	313.61	110	463.14	499	188	366	97	98	7	42	31
5040	12828	9274	689	610	358	28136	1174	10	672	1	26	68	8	87	281.86	102	435.45	353	366	297	14	43	0	0	3
4732	13050	9452	738	552	318	28122	1175	8	635	0	16	62	7	86	276.22	87	434.22	342	337	310	16	37	0	2	2
3808	13728	9994	983	326	186	28056	1318	7	562	2	14	69	3	69	270.26	84	422.55	261	278	261	18	40	0	0	2
5512	12424	8982	576	760	458	28180	1041	5	679	0	42	83	3	110	284.87	123	440.92	404	330	327	21	55	0	2	3
11270	7872	5548	3	2076	1494	28620	532	89	592	10	312	174	52	272	325.48	96	467.58	465	216	379	111	93	5	44	23
5814	12328	8800	514	702	474	28184	1036	16	723	2	64	61	11	102	283.97	116	439.98	405	379	358	27	41	0	2	1
13106	7866	4700	6	1332	1030	28162	630	199	830	81	122	201	87	215	282.9	190	447.17	473	305	426	40	90	12	4	2
3760	13804	10018	1008	290	166	28040	1298	6	556	1	2	68	5	69	269.4	92	423.18	273	247	274	12	41	0	0	3
4726	13202	9480	748	408	252	28084	1171	5	697	0	14	59	4	86	276.25	99	431.38	331	348	335	10	36	0	2	4
4942	12930	9348	701	556	324	28126	1159	3	681	0	24	59	2	80	278.06	102	436.23	358	349	318	17	43	0	2	3
4494	13230	9586	809	474	288	28100	1213	5	619	0	28	54	3	78	274.04	89	429.28	321	314	308	16	41	0	0	3
12060	7770	5170	0	1840	1332	28430	554	124	679	30	220	183	59	265	314.47	125	459.13	467	234	391	75	109	11	36	18

SUPPLEMENTARY INFORMATION:Monte Carlo Atomistic Simulation and Machine Learning Analysis of Na-K Eutectic Alloy in Condensed Phases, D. Reitz and E. Blaisten-Barojas, George Mason University, Fairfax, VA 22030

4242	13432	9748	894	398	236	28078	1262	10	586	1	18	64	4	75	274.26	86	428.29	298	298	279	16	38	0	4	1
4336	13490	9742	888	304	164	28040	1259	9	654	3	4	70	6	75	273.55	106	426.96	295	323	285	9	47	0	0	2
4328	13422	9708	871	384	218	28066	1265	9	631	1	4	59	6	64	273.3	105	427.51	299	322	292	14	36	0	2	4
11222	7752	5450	0	2200	1646	28664	522	84	547	18	328	175	40	266	318.09	91	465.47	496	188	350	114	95	5	60	30
4034	13612	9868	957	334	196	28056	1298	8	580	1	12	49	4	61	271.22	93	424.04	278	286	289	15	31	0	0	4
5466	12486	9034	600	726	416	28156	1092	14	681	3	28	66	6	103	283.34	115	439.95	382	358	324	19	41	0	0	2
5674	12550	8964	583	570	336	28114	1079	18	745	3	20	72	9	90	279.44	115	436.78	378	379	351	19	45	1	0	4
4214	13454	9752	864	416	238	28082	1240	6	568	1	8	67	2	71	273.51	98	429.1	316	287	283	16	39	0	0	2
11500	7864	5292	0	2036	1544	28566	543	86	617	21	312	149	42	264	310.1	110	457.15	490	213	420	85	80	8	16	16
5262	12798	9166	642	536	332	28120	1115	11	699	0	24	73	6	90	280.33	101	434.73	362	350	326	22	51	0	2	3
12296	7890	5064	1	1630	1234	28350	537	149	730	43	214	184	80	235	293.69	147	451.99	505	259	435	69	95	9	20	9
10886	7904	5488	2	2232	1768	28710	518	83	545	16	370	163	42	282	316.92	110	461.64	491	172	357	119	78	4	54	21
14430	7590	4330	8	822	628	27874	669	337	1022	164	70	265	133	152	271.53	249	437.62	470	441	420	17	84	22	4	3
5066	12898	9284	661	542	314	28120	1116	3	669	0	14	74	1	91	278.83	109	435.1	362	337	333	18	52	0	2	3
3816	13726	9982	995	336	190	28056	1315	6	555	0	4	54	4	56	270.71	94	423.52	272	270	277	19	31	0	2	3
4824	13082	9456	717	464	250	28086	1153	6	684	1	8	57	2	78	278.47	100	434.33	338	346	344	21	39	0	2	2
4004	13672	9878	976	302	186	28050	1339	8	600	2	8	68	6	68	270.73	84	423.48	250	306	253	14	45	0	0	5
3900	13700	9948	993	316	178	28048	1315	11	569	2	4	59	6	72	270.97	85	423.85	265	275	271	9	44	0	2	4
4338	13332	9666	827	460	280	28100	1204	6	579	1	22	56	3	86	273.51	72	428.94	331	303	305	9	41	0	2	2
4114	13454	9804	905	448	250	28082	1263	7	577	1	12	65	5	80	272.38	84	425.83	297	284	277	13	44	0	0	0
4230	13456	9734	873	416	242	28082	1246	7	606	1	4	51	4	67	273.34	93	427.53	314	296	302	14	37	0	0	2
11838	7870	5200	1	1860	1414	28480	581	125	632	29	274	168	66	256	304.68	126	454.28	471	218	393	91	89	10	24	11
4364	13472	9740	875	296	156	28036	1257	3	682	0	8	66	2	65	271.96	104	424.99	295	325	295	15	44	0	0	2
4464	13368	9654	827	362	206	28068	1220	6	645	1	14	58	1	60	273.12	113	428.18	332	324	303	17	35	0	0	2
14294	7690	4408	6	800	616	27884	640	321	1033	159	70	264	122	156	274.65	241	438.38	478	431	428	23	94	12	6	2
11778	8066	5234	3	1692	1370	28454	610	113	722	23	282	168	62	247	298.7	153	451.76	476	262	394	77	101	8	28	9
14014	7738	4498	6	932	692	27948	674	295	1012	142	68	231	113	165	275.07	212	439.65	455	448	430	27	75	12	6	3
4282	13466	9742	865	354	206	28064	1225	3	618	0	12	61	1	78	274.56	92	428.61	308	302	306	11	41	0	2	4
4234	13426	9736	886	418	252	28088	1266	5	592	1	22	60	2	79	274.8	92	428.32	287	291	282	17	37	0	0	3
4824	12958	9374	705	592	360	28138	1180	7	656	0	30	62	4	84	279.98	90	436.8	334	357	302	27	41	0	0	2
3822	13714	9982	1013	340	190	28056	1329	6	548	1	8	57	3	66	270.15	79	422.38	267	258	260	11	34	0	0	4
4800	13050	9412	707	526	314	28122	1140	4	652	0	20	61	2	93	277.26	95	432.23	358	330	328	10	37	0	0	4
13624	7892	4624	5	992	774	28002	624	268	980	125	88	229	114	161	276.07	244	442.12	501	390	433	33	82	8	6	3
14474	7668	4346	7	746	560	27844	643	326	1091	177	50	277	108	151	269.54	263	431.39	483	456	415	19	85	16	0	1
4174	13568	9824	928	300	166	28042	1281	10	610	1	10	59	6	65	271.56	87	425.19	287	301	285	15	46	0	0	1
12746	7824	4898	4	1486	1112	28254	580	179	767	53	172	205	85	263	288.6	147	450.3	447	262	416	50	106	15	16	11

12958	7980	4828	6	1286	976	28150	618	198	874	72	110	207	101	215	284.73	197	445.64	461	336	438	48	99	11	12	0
14388	7698	4358	6	758	594	27858	623	325	1072	162	58	261	127	149	267.65	266	429.56	492	425	450	18	91	15	4	1
4598	13188	9548	774	472	272	28096	1193	5	626	0	16	67	4	80	276.48	99	430.12	314	317	315	24	45	0	2	3
3968	13666	9894	943	328	196	28060	1276	4	578	0	8	63	3	66	271.56	96	424.99	289	275	286	15	42	0	0	3
13386	7856	4758	7	1120	822	28060	624	245	918	98	108	206	116	192	276.71	194	443.69	462	363	461	41	91	11	8	1
3842	13716	9960	1001	334	196	28056	1316	8	533	1	8	62	6	71	271.35	76	423.11	269	261	265	12	42	0	0	2
13020	7916	4934	8	1230	878	28120	604	211	949	63	134	172	110	199	282.79	214	444.05	494	350	476	31	94	15	8	4
11596	7910	5400	0	1930	1382	28506	545	110	658	28	252	177	58	273	305.47	133	456.49	456	218	402	87	99	5	36	24
13808	7550	4454	2	1188	922	28064	602	260	915	99	132	217	118	185	279.4	209	447.07	482	339	449	46	106	21	10	2
4270	13440	9740	857	390	220	28070	1234	10	602	2	10	58	6	78	274.09	94	427.71	303	302	308	12	39	0	0	3
6238	12110	8618	463	718	440	28162	1002	14	811	3	38	81	6	105	281.69	128	438.57	432	399	356	16	56	0	0	2
3982	13630	9904	965	336	190	28054	1300	8	582	1	12	56	5	69	270.39	95	423.02	280	279	279	9	37	0	0	3
5170	12890	9264	643	480	274	28098	1123	8	712	1	20	65	4	87	279.84	111	435.88	373	382	335	10	46	0	0	1
4284	13482	9750	875	340	192	28058	1243	5	628	1	10	63	2	66	274.26	91	428.82	300	321	304	19	44	0	0	2
12392	7976	5072	2	1520	1132	28286	590	150	825	34	170	155	83	227	290.49	158	449.19	472	296	464	58	93	12	24	8
14318	7762	4436	5	728	548	27842	658	319	1113	169	44	266	109	153	267.77	281	430.41	474	450	422	15	91	17	6	1
5278	12830	9150	606	522	330	28132	1107	6	728	0	20	73	3	96	281.71	119	438.56	375	385	322	18	45	0	2	1
4702	13174	9480	742	452	280	28110	1174	5	644	0	16	55	4	71	278.32	105	433.03	354	350	321	20	36	0	6	0
4104	13590	9848	925	322	178	28048	1260	9	580	1	6	56	5	72	271.91	95	425.29	293	280	297	12	37	0	0	2
14138	7732	4506	8	848	610	27900	612	320	1029	155	66	254	122	162	275.85	244	438.22	487	406	445	27	85	15	0	0
4590	13148	9544	802	518	292	28110	1189	6	624	0	18	54	6	86	278.46	102	433.04	331	292	314	18	41	0	0	0
4392	13294	9640	818	482	278	28098	1204	4	584	0	12	65	2	79	275.93	90	431.61	323	296	306	18	40	0	0	2
11480	8044	5314	2	1886	1482	28528	544	105	630	25	300	154	51	252	306.24	134	456.96	504	214	393	89	79	7	20	19
4906	12946	9348	709	554	342	28132	1140	8	643	0	34	58	5	98	278.75	91	434.77	350	330	329	13	45	0	2	4
14500	7522	4304	4	880	624	27878	665	328	1041	159	44	263	132	164	270.02	245	432.34	466	451	422	13	91	19	4	2
4268	13394	9726	868	440	250	28092	1259	5	598	0	12	64	3	71	273.88	93	427.23	314	309	274	12	43	0	2	3
4176	13468	9804	904	402	210	28066	1265	7	597	0	6	64	4	69	273.77	98	427.14	300	290	279	14	44	0	0	3
5484	12532	9014	617	666	416	28158	1108	12	676	3	44	81	5	123	284.65	104	440.66	345	348	309	21	51	0	2	2
4072	13618	9878	973	300	164	28042	1327	10	602	2	10	48	4	58	271.03	88	423.56	259	310	277	21	36	0	0	1
14222	7720	4430	3	846	622	27890	632	312	1061	150	48	247	125	144	270.13	262	433.24	502	428	446	20	84	13	2	1
3934	13690	9952	960	300	158	28040	1272	4	588	1	6	67	3	60	271.68	91	424.13	289	261	286	18	49	0	0	3
4508	13308	9614	832	406	230	28076	1221	8	644	1	10	58	4	71	273.67	98	428.1	314	323	311	18	43	0	0	2
4630	13212	9524	778	434	268	28092	1214	5	658	0	24	63	1	75	276.3	102	430.55	324	346	294	18	45	0	0	3
4402	13398	9676	863	356	216	28070	1236	8	636	1	20	66	5	92	273.89	94	428.47	283	315	305	11	35	0	2	4
11910	7928	5206	1	1804	1348	28462	559	118	650	28	236	181	58	267	307.59	137	457.89	489	222	388	77	86	12	30	10
13220	7742	4722	0	1304	990	28138	615	207	875	88	150	223	92	206	281.9	201	446.31	479	344	405	48	100	7	10	4

SUPPLEMENTARY INFORMATION:Monte Carlo Atomistic Simulation and Machine Learning Analysis of Na-K Eutectic Alloy in Condensed Phases, D. Reitz and E. Blaisten-Barojas, George Mason University, Fairfax, VA 22030

11094	7948	5538	0	2126	1564	28624	504	74	565	13	326	162	33	288	316.85	109	462.19	482	168	385	104	81	12	28	23
7346	11172	7876	345	1068	714	28258	942	40	762	8	74	77	27	146	286.74	133	443.01	421	384	367	29	42	0	8	5
13800	7816	4588	11	950	722	27968	640	284	1003	131	84	242	114	184	276.87	238	441.48	452	386	443	31	92	19	8	1
5026	12864	9298	700	558	340	28128	1124	11	652	1	42	54	8	90	279.3	101	435.6	360	324	343	21	30	0	0	0
5348	12518	9060	573	766	456	28182	1073	6	657	0	32	73	4	109	284.49	86	441.58	380	355	336	23	39	0	2	1
14384	7562	4372	5	860	636	27900	645	352	1013	159	82	248	150	166	271.56	256	434.44	469	426	442	19	78	21	4	2
4614	13140	9538	773	510	288	28112	1164	6	641	0	22	51	5	81	276.39	115	431.37	340	308	344	17	28	0	0	2
14436	7706	4352	7	752	564	27850	610	328	1104	161	40	273	132	158	267.46	294	429.71	507	425	440	6	102	20	0	2
5078	12818	9232	649	610	376	28144	1102	9	666	2	30	56	5	96	279.35	105	436.23	375	327	346	15	35	0	0	3
5494	12492	8998	625	696	438	28172	1105	6	673	0	54	72	4	98	284.08	102	440.22	380	330	315	21	53	0	0	3
10998	7838	5554	0	2184	1676	28702	503	74	518	16	408	174	38	270	319.07	109	467.36	518	167	355	109	78	4	42	33
4264	13458	9756	884	360	204	28056	1250	10	616	2	14	66	6	73	271.07	96	426.06	295	294	290	17	41	0	0	2
6936	11542	8182	386	918	574	28202	945	44	747	10	46	88	26	143	288.31	119	445.07	402	379	383	28	54	0	4	1
8026	10642	7522	256	1198	786	28284	855	58	772	13	102	115	39	159	290.15	128	447.26	450	369	365	42	64	1	6	4
4990	13060	9354	696	412	250	28084	1125	7	713	1	18	61	4	82	278.26	106	434.58	349	353	358	21	40	0	0	0
4654	13190	9532	753	450	256	28100	1191	4	674	0	18	53	2	84	277.63	111	432.86	332	342	318	15	31	0	0	0
6370	11910	8504	489	840	520	28192	1011	24	730	4	46	77	15	127	288.83	118	442.15	385	353	359	28	46	0	2	3
12362	7636	4924	0	1822	1404	28430	591	146	665	36	252	182	79	257	297.65	127	451.72	467	248	391	76	96	11	24	11
13788	7574	4380	2	1192	998	28102	633	262	868	95	162	217	121	191	278.16	187	446.43	472	351	424	47	105	24	6	2
4540	13160	9556	763	528	310	28120	1199	6	613	0	24	53	4	71	278.07	93	432.51	338	321	309	23	35	0	2	1
13846	7810	4582	6	940	698	27956	637	290	1010	138	76	247	114	157	274.8	249	439.53	492	403	432	29	84	18	4	1
13376	7760	4700	6	1226	900	28082	644	235	919	92	110	208	99	205	280.99	200	446.05	459	358	415	37	91	16	10	3
4708	13142	9494	782	474	266	28094	1198	9	649	1	10	57	4	85	273.93	105	429.45	325	315	313	11	40	0	0	4
12628	7972	5004	6	1428	1044	28234	616	174	838	54	136	185	93	203	286.03	173	447.07	502	311	424	49	100	12	20	4
13688	7746	4626	3	1062	782	28010	629	260	960	114	94	233	101	186	277.52	219	442.79	468	375	435	34	100	19	12	2
5758	12320	8820	522	748	488	28190	1035	22	702	5	54	81	13	126	283.93	118	442.45	401	365	331	17	47	0	2	1
4118	13520	9834	891	376	206	28068	1254	4	595	0	14	60	2	69	274.57	89	428.44	300	298	292	16	38	0	0	3
7972	10792	7606	256	1112	692	28246	839	43	803	7	68	101	29	137	288.8	137	448.44	471	362	396	41	70	0	4	3
4676	13228	9532	790	400	224	28066	1194	10	668	2	6	58	3	82	275.02	103	428.71	316	317	328	16	38	0	0	0
14384	7738	4358	8	728	588	27862	609	343	1067	166	62	262	138	143	270.63	277	432.73	510	426	449	20	86	15	4	0
4302	13472	9732	879	348	198	28058	1247	8	606	3	6	62	2	72	272.95	103	427.59	297	296	296	17	37	0	0	2
4524	13220	9594	783	474	268	28100	1188	7	620	0	18	60	5	96	277.78	79	431.95	311	320	322	17	43	0	2	1
4364	13388	9718	857	384	200	28062	1235	7	636	1	8	62	5	75	273.83	101	427.94	299	308	305	18	43	0	0	1
5414	12640	9092	617	606	354	28132	1109	10	730	1	24	62	7	95	278.92	121	435.48	378	371	327	16	44	0	2	1
4058	13636	9874	944	294	170	28042	1289	7	608	2	8	59	3	67	270.8	91	423.02	265	293	293	19	39	0	2	3
12626	7892	4900	1	1494	1168	28288	596	167	779	57	188	189	75	212	291.86	202	451.72	506	277	409	59	91	13	20	8

SUPPLEMENTARY INFORMATION:Monte Carlo Atomistic Simulation and Machine Learning Analysis of Na-K Eutectic Alloy in Condensed Phases, D. Reitz and E. Blaisten-Barojas, George Mason University, Fairfax, VA 22030

5632	12508	8964	547	638	378	28148	1039	9	751	3	28	66	6	102	280.17	116	438.69	393	378	369	23	47	0	0	1
5160	12784	9258	661	578	316	28118	1158	7	725	0	20	68	5	86	278.13	104	435.89	339	386	319	23	47	0	2	2
10598	7864	5664	0	2412	1800	28770	508	63	486	9	386	166	38	303	323.65	90	468	471	157	360	108	76	1	42	31
4420	13202	9610	810	542	320	28122	1216	7	588	0	26	60	3	82	275.3	89	430.88	325	304	293	15	41	0	2	4
14298	7670	4392	4	834	632	27896	639	325	1050	174	66	253	119	162	270.94	263	434.48	483	437	441	16	67	14	4	1
4080	13510	9812	882	416	252	28092	1227	6	574	0	22	58	4	88	275.06	95	429.22	314	277	297	8	37	0	0	1
13162	7740	4714	0	1344	1048	28194	574	197	854	67	170	201	100	214	286.55	200	448.82	486	307	443	55	103	9	14	6
11942	7912	5150	0	1756	1382	28444	604	124	680	28	274	164	65	273	297.94	113	455.07	442	252	404	70	88	13	28	11
4712	13230	9510	800	364	230	28074	1175	5	684	0	26	56	5	66	276.08	122	431.5	340	319	340	17	42	0	2	3
14244	7678	4412	8	858	640	27896	673	306	1050	168	62	251	108	151	268.12	260	429.42	486	465	415	14	74	11	2	2
14300	7628	4354	4	860	676	27902	613	333	1021	160	76	264	137	166	270.81	255	434.52	475	398	447	28	89	18	8	1
4802	12988	9388	736	582	348	28128	1181	8	642	0	20	55	3	83	279.79	91	434.77	326	335	317	26	39	0	0	3
12312	7872	5130	1	1590	1192	28366	541	136	759	38	252	170	69	244	304.35	160	457.34	491	259	437	70	93	5	18	14
14368	7672	4360	5	794	618	27884	662	323	1060	160	68	257	132	154	271.42	253	434.6	474	451	433	17	82	15	4	1
4952	12862	9330	721	606	354	28138	1158	5	644	0	34	64	5	84	277.53	99	434.66	353	330	314	20	42	0	0	3
5380	12686	9136	634	574	322	28120	1097	13	699	1	22	76	10	94	280.6	114	436.73	377	351	335	14	46	0	0	3
4062	13650	9896	955	278	144	28034	1274	10	595	2	4	50	5	74	271.41	90	423.36	279	279	307	8	34	0	0	2
5914	12442	8840	544	560	340	28122	1059	13	797	0	24	74	13	98	283.28	118	439.96	386	407	364	17	54	0	2	1
14350	7580	4314	5	894	696	27920	639	315	1026	145	86	247	136	147	270.03	267	432.11	505	419	436	18	92	10	0	3
4444	13278	9610	811	466	282	28102	1209	5	597	1	22	61	2	71	275.03	94	430.34	332	311	304	17	43	0	0	3
5416	12630	9078	610	630	366	28140	1085	7	699	1	20	55	5	116	279.34	97	438.08	357	369	365	13	35	0	0	2
5350	12556	9028	624	740	470	28182	1123	18	628	2	38	65	14	112	282.79	88	440.4	351	341	319	22	38	0	0	2
4556	13230	9590	792	450	250	28094	1226	4	647	0	18	62	2	75	277.91	84	432.95	315	351	298	16	44	0	0	4
10702	7760	5630	0	2418	1812	28788	509	59	488	7	420	156	29	297	321.37	82	470.24	466	155	381	114	87	8	44	39
5532	12534	9044	575	644	360	28140	1080	11	733	0	24	73	8	86	280.55	112	437.54	390	367	343	24	56	1	0	1
14454	7566	4324	6	850	622	27874	621	323	1070	167	56	268	115	144	270.01	292	431.59	514	408	423	18	88	18	2	2
4364	13326	9662	846	462	266	28092	1221	8	600	1	12	60	4	77	273.7	106	429.83	320	284	296	14	47	0	0	4
5258	12674	9126	631	654	404	28150	1095	12	665	1	34	55	7	93	281.5	106	437.55	382	338	337	24	39	0	0	0
6176	12134	8658	449	730	438	28174	1003	8	792	2	38	64	4	107	283.61	130	442.27	425	395	375	17	44	0	0	2
4032	13598	9868	930	350	202	28062	1260	7	558	1	12	60	5	67	273.65	86	427.65	293	269	296	17	42	0	0	3
7140	11462	8110	360	878	548	28200	952	34	815	13	60	85	21	120	286.06	127	443.89	439	422	375	22	43	0	2	3
4866	13012	9384	743	524	306	28106	1177	10	670	0	14	61	6	78	278.12	111	434.59	331	337	318	24	42	0	0	3
4818	13102	9434	758	446	268	28090	1188	14	675	0	22	50	7	83	275.98	109	430.59	330	340	325	13	31	0	0	1
3848	13686	9960	991	362	202	28064	1323	7	556	1	6	60	6	64	269.97	85	422.47	269	265	259	19	34	0	0	1
10918	7830	5656	2	2342	1618	28724	512	77	510	9	338	170	46	304	324.82	102	467.97	474	156	369	113	92	4	22	19
5104	12784	9246	667	632	352	28128	1143	9	694	2	10	55	5	98	279.83	110	436.7	341	360	340	15	36	0	0	3

5258	12766	9186	643	568	326	28124	1109	5	691	0	20	64	5	100	281.01	92	437.08	366	359	344	10	39	0	0	3
3854	13722	9962	976	326	184	28050	1277	9	547	1	2	65	6	64	270.79	93	424.95	285	242	286	18	44	0	0	2
14362	7702	4362	4	778	608	27876	641	326	1064	147	60	249	135	149	269.79	255	433.03	474	453	452	22	87	20	4	4
13850	7560	4416	3	1166	930	28070	592	275	870	121	140	220	116	190	279.48	211	445.97	490	327	452	40	80	15	6	3
4472	13282	9634	851	426	242	28076	1236	9	613	1	20	64	4	78	272.85	94	426.41	306	304	289	18	41	0	0	1
4514	13210	9578	824	498	284	28096	1217	13	612	0	12	61	8	74	274.15	90	428.66	319	309	298	19	44	0	0	4
4502	13272	9638	825	424	226	28078	1209	7	634	1	16	59	4	73	274.45	91	428.62	322	313	317	14	41	0	0	3
4034	13640	9870	924	320	186	28056	1266	6	592	1	6	53	4	59	271.6	94	425.59	302	291	296	16	34	0	0	2
13098	7896	4750	2	1228	998	28146	625	197	883	69	166	192	95	209	282.59	191	447.72	454	340	447	47	89	10	8	6
5120	12620	9182	658	746	458	28180	1109	12	611	4	52	63	6	107	282.47	90	439.02	374	310	318	18	38	0	0	1
14516	7524	4262	4	852	658	27892	629	342	1011	164	78	253	140	155	271.94	222	437.13	468	431	462	28	73	20	2	2
14054	7694	4476	7	946	696	27940	622	312	969	144	74	239	123	157	274.25	231	437.46	492	400	455	24	73	16	0	4
4362	13390	9654	836	408	256	28084	1224	7	608	0	12	51	5	72	274.32	91	427.9	309	314	319	20	33	0	2	1
4556	13220	9540	779	478	294	28108	1188	1	642	0	20	63	1	80	276.38	105	431.05	336	312	313	15	43	0	0	3
3862	13652	9954	994	370	212	28068	1317	6	531	2	18	58	4	62	270.46	84	423.73	276	264	264	16	32	0	0	3
14446	7650	4394	5	760	546	27850	606	329	1073	159	50	260	129	148	272.07	279	435.7	495	411	459	20	91	21	4	2
13252	7720	4646	4	1314	1058	28182	588	218	810	75	174	200	107	213	284.45	185	448.31	470	283	454	49	88	18	16	9
3896	13736	9982	992	272	140	28030	1328	8	587	1	4	71	5	67	271.35	85	422.06	249	291	261	18	51	0	0	2
12650	7946	4956	1	1418	1092	28262	620	164	817	55	184	192	76	221	287.59	179	447.96	471	300	412	52	93	11	12	11
5250	12768	9142	652	586	368	28140	1139	7	708	2	26	64	4	97	280.39	119	436.47	357	363	324	14	42	0	0	2
13644	7838	4634	2	1026	764	27994	639	247	998	113	86	238	105	173	274.56	232	439.15	475	411	436	29	98	17	2	4
11748	7942	5194	0	1870	1456	28508	547	129	649	34	264	166	66	271	309.99	124	456.11	469	233	401	96	75	10	30	14
4210	13530	9818	894	318	160	28040	1253	12	608	1	4	53	7	71	271.13	89	424.14	294	310	300	15	35	0	0	1
4080	13588	9844	932	340	196	28056	1278	7	578	1	8	50	6	74	272.83	83	426.04	275	283	302	14	36	0	0	2
7344	11280	7972	351	950	600	28214	923	46	792	10	66	95	28	138	287.55	142	446.02	437	388	363	24	59	0	2	6
4166	13494	9782	886	396	226	28070	1267	5	583	1	6	67	1	74	274.31	90	428.31	285	294	274	22	41	0	0	2
4996	12954	9328	670	516	300	28112	1153	4	696	0	18	72	2	89	279.52	94	436.29	335	374	322	22	50	0	0	3
5518	12538	9036	598	638	374	28140	1065	14	678	4	34	89	7	108	282.91	105	439.06	370	338	334	21	60	1	2	5
4608	13192	9542	788	460	270	28092	1200	7	649	0	18	64	4	89	275.21	92	431.26	320	318	304	10	44	0	2	5
11892	8028	5242	1	1676	1294	28410	553	132	716	37	252	168	64	228	292.44	160	453.31	520	229	412	71	92	8	26	13
13898	7756	4508	4	960	750	27974	611	280	995	140	102	236	106	173	275.95	251	442.65	479	376	453	33	80	17	0	3
4796	13114	9452	752	454	262	28094	1195	8	681	0	16	57	6	76	275.33	93	429.95	342	362	310	13	39	0	0	1
5400	12724	9104	618	548	328	28124	1070	12	720	2	16	62	10	101	280.66	123	438	374	352	371	17	44	0	4	1
13184	7884	4726	1	1198	970	28128	600	214	931	75	150	210	95	200	281.56	210	446.65	494	335	443	38	112	19	16	4
11572	7908	5260	3	1938	1524	28548	534	129	617	32	314	179	68	260	312.28	132	457.32	496	193	393	94	89	11	30	16
13396	7670	4668	3	1276	948	28104	627	253	863	98	138	217	111	199	282.88	187	446.52	448	342	424	54	88	14	8	7

SUPPLEMENTARY INFORMATION:Monte Carlo Atomistic Simulation and Machine Learning Analysis of Na-K Eutectic Alloy in Condensed Phases, D. Reitz and E. Blaisten-Barojas, George Mason University, Fairfax, VA 22030

5308	12748	9168	608	556	320	28124	1090	7	726	0	24	67	4	92	279.92	104	436.35	386	378	346	14	44	0	0	1
4586	13244	9558	783	436	254	28092	1200	10	639	1	14	69	4	96	277.47	104	433.56	328	320	295	7	45	0	0	0
12648	7998	4974	5	1384	1052	28228	590	169	840	59	150	198	80	204	288.49	193	449.59	513	320	415	48	102	4	20	10
4740	12970	9448	717	614	342	28138	1167	6	638	0	24	54	3	85	278.1	90	434.72	346	328	317	21	33	0	0	2
5426	12592	9046	562	656	404	28160	1063	7	727	0	34	72	5	106	280.14	113	436.77	385	369	348	21	42	1	2	0
11834	7954	5296	2	1794	1304	28442	552	124	690	28	236	176	62	245	305.11	142	456.99	492	230	402	85	102	7	24	15
13530	7894	4700	6	1014	766	28010	617	265	1007	126	104	229	108	176	276.97	250	439.35	506	373	440	22	88	16	2	2
11250	8102	5542	1	1862	1430	28546	548	81	646	16	318	185	40	261	321.67	146	463.18	470	206	373	101	91	1	42	25
4110	13462	9826	909	434	232	28076	1250	7	568	3	12	53	4	82	273.05	83	426.6	288	279	306	13	31	0	0	3
4592	13160	9550	772	508	280	28102	1186	5	645	0	12	56	2	78	276.46	102	433.17	340	319	319	11	33	0	0	5
5612	12360	8920	585	778	472	28186	1050	15	660	3	42	63	8	111	285.82	97	442.82	395	319	343	22	44	0	2	3
14228	7728	4410	7	830	638	27898	649	317	1045	152	62	249	129	150	270.32	277	434.5	494	442	436	15	80	19	0	2
4348	13312	9682	834	470	264	28092	1222	4	607	1	14	66	2	78	275.42	92	428.82	312	300	299	16	44	0	2	4
14086	7782	4504	6	846	624	27900	606	299	1063	144	54	264	115	169	273.76	263	438.89	485	406	440	21	105	8	4	4
4126	13600	9844	934	300	166	28042	1270	7	603	1	6	63	5	67	271.93	95	424.82	285	278	295	15	45	0	0	2
4248	13450	9748	853	392	224	28074	1226	8	599	2	12	56	3	81	273.47	80	427.52	305	305	309	15	35	0	0	1
11720	7792	5222	1	1972	1494	28516	521	119	632	32	278	192	50	273	309.18	133	456.86	513	189	341	85	103	10	34	19
4542	13050	9534	726	616	366	28154	1163	1	604	0	46	58	1	81	277.58	88	433.97	369	325	303	20	39	0	0	0
3816	13778	10014	987	278	146	28034	1306	4	586	1	2	69	2	68	270.09	98	422.88	270	270	265	15	44	0	0	1
11452	7908	5336	2	2014	1532	28570	531	94	609	14	280	161	51	286	310.96	121	459.4	484	199	397	89	93	5	46	17
5160	12782	9204	666	612	358	28130	1123	13	669	3	12	64	7	102	279.54	89	436.79	358	342	331	12	47	0	2	3
12414	7882	4948	2	1614	1250	28318	597	156	774	56	186	182	74	215	285.58	159	447.83	497	300	415	60	86	9	22	12
13944	7720	4488	4	950	754	27966	639	288	1036	146	98	243	110	160	273.31	260	438.78	497	416	424	26	79	15	12	2
4690	13218	9532	791	396	222	28070	1209	7	636	0	12	54	2	75	276.86	100	431.47	335	342	306	7	31	0	0	3
5018	12922	9342	704	528	284	28106	1167	7	698	1	10	66	4	91	278.69	110	435.39	347	359	308	10	49	0	2	3
12148	7912	5042	2	1704	1332	28390	574	151	701	33	234	168	85	245	298.21	147	451	477	256	417	79	85	9	16	9
11800	7842	5270	0	1884	1388	28474	513	133	628	24	254	165	80	258	306.27	126	459.43	481	213	435	90	78	7	32	19
6122	12104	8612	459	804	516	28202	1035	18	749	2	42	73	12	116	283.26	117	439.45	412	410	334	18	46	0	2	3
5310	12732	9146	626	586	336	28122	1115	10	712	0	12	73	6	102	278.14	109	434.27	366	378	323	14	52	0	0	0
4620	13190	9516	775	484	284	28104	1163	9	638	1	10	58	5	84	277.95	103	434.86	339	308	328	15	42	0	0	5
12484	7958	4990	2	1512	1152	28286	571	176	802	44	166	174	107	232	288.18	171	449.49	496	279	455	45	109	11	20	11
14134	7694	4456	9	894	662	27908	623	304	1033	142	66	247	123	162	272.44	261	436.14	489	424	446	20	94	17	2	2
5070	12878	9254	690	562	344	28132	1122	8	641	0	24	67	5	88	279.43	116	434.44	380	315	324	14	43	0	0	1
10482	7896	5686	0	2404	1862	28826	492	66	474	10	458	164	31	314	326.45	94	470.34	491	143	341	116	75	4	34	34
14276	7656	4462	3	836	588	27880	652	308	1072	145	58	258	119	144	272.66	253	436.94	481	436	432	24	102	26	4	2
6746	11682	8320	412	872	526	28192	989	22	797	4	46	84	15	122	287.79	121	446	415	415	360	24	55	1	0	1

SUPPLEMENTARY INFORMATION:Monte Carlo Atomistic Simulation and Machine Learning Analysis of Na-K Eutectic Alloy in Condensed Phases, D. Reitz and E. Blaisten-Barojas, George Mason University, Fairfax, VA 22030

5686	12554	8912	615	588	366	28116	1065	13	687	2	10	70	9	112	282	105	438.97	359	339	365	20	48	0	0	1
4818	13214	9476	750	342	198	28058	1209	6	715	1	8	62	4	74	274.07	109	429.88	324	379	307	14	41	0	2	1
4244	13532	9766	872	318	186	28054	1231	7	629	1	8	51	4	73	272.7	97	426.71	308	298	320	11	35	0	0	1
13938	7742	4538	4	950	702	27954	651	286	1006	141	82	246	115	163	273.92	232	437.9	479	404	424	27	92	11	2	2
13544	7672	4570	3	1224	954	28116	638	245	876	95	136	217	111	213	282.58	188	446.46	463	358	415	34	99	18	14	5
11626	7890	5270	4	1936	1494	28556	552	104	602	24	312	191	51	275	309.29	116	458.13	481	218	373	83	102	11	24	24
12108	7882	5082	2	1716	1334	28386	572	137	744	35	234	181	68	257	293.18	163	451.02	481	253	408	61	104	8	30	12
12186	7904	5148	2	1682	1226	28374	570	130	749	35	206	168	63	238	296.26	175	454.31	502	253	410	66	90	8	22	11
4370	13380	9688	865	394	224	28068	1265	10	627	1	12	65	6	74	273.36	82	427.31	292	333	278	12	45	0	0	6
4646	13116	9508	753	532	298	28112	1192	3	647	0	12	69	2	86	277.74	109	433.18	337	339	293	16	43	0	0	1
14134	7666	4422	6	942	706	27946	680	286	1027	139	74	230	115	160	274.16	224	437.12	452	450	440	29	81	12	2	2
13502	7788	4614	4	1154	892	28072	631	252	907	118	114	209	107	194	277.92	196	441.79	469	383	450	35	72	18	8	2
14174	7602	4352	6	994	752	27942	635	335	930	159	64	242	138	162	272.91	240	436.09	486	391	440	27	67	15	4	0
4428	13306	9648	815	432	248	28080	1208	3	621	0	18	79	3	88	273.91	90	429.67	306	313	297	14	55	0	0	5
3820	13688	9990	981	356	192	28056	1297	8	539	1	10	61	4	71	271.76	77	424.82	274	253	271	15	44	0	0	3
4190	13530	9800	919	340	186	28050	1269	10	601	1	4	52	8	70	272.26	85	426.04	274	295	308	18	40	0	0	3
5198	12638	9164	625	712	420	28168	1095	7	676	1	34	59	3	97	279	100	436.51	383	337	341	16	42	0	2	3
11096	7794	5458	2	2234	1712	28728	553	93	489	20	392	154	44	279	323.15	91	466.38	472	179	343	122	70	4	38	24
5340	12738	9126	664	546	336	28112	1133	21	663	6	24	68	12	91	282.16	84	439.12	331	347	337	31	42	0	2	1
5604	12650	9022	578	510	300	28104	1094	15	777	3	16	61	7	90	279.91	112	437.63	374	412	347	18	43	0	2	2
14258	7592	4346	4	930	720	27934	659	306	997	157	84	253	108	159	271.88	245	434.37	485	412	414	20	81	19	4	3
13244	7694	4712	1	1372	1006	28178	642	212	845	70	136	199	104	211	281.88	183	448.91	481	337	405	41	100	18	14	3
5266	12718	9148	629	600	368	28132	1099	13	680	1	30	69	7	97	278.85	110	434.63	367	345	342	18	46	0	2	3
4914	12994	9398	715	510	272	28096	1151	9	651	2	8	68	5	83	278.25	96	435.6	349	357	327	16	39	0	0	2
4312	13484	9736	868	332	188	28058	1255	5	621	1	4	55	1	77	273.77	97	427.28	300	318	296	9	37	0	0	1
4682	13202	9516	759	416	246	28080	1147	6	673	0	18	56	1	87	275.94	116	431.79	338	322	340	16	35	0	0	3
4078	13554	9826	928	382	224	28074	1286	8	579	1	10	59	6	80	272.65	88	425.96	277	288	278	12	33	0	0	3
10904	7854	5622	1	2208	1674	28722	523	73	539	16	418	193	35	290	325.59	105	466.99	485	169	328	107	98	5	42	35
4456	13214	9614	800	538	286	28110	1200	4	583	0	2	60	1	89	273.88	97	428.72	322	298	310	15	31	0	0	1
5624	12506	9022	598	608	328	28110	1068	14	709	0	22	80	12	108	281.45	99	438.79	370	359	347	15	59	0	0	2
4132	13488	9786	901	428	244	28082	1279	7	588	1	4	57	4	76	272.81	95	427.16	302	297	270	9	35	0	0	1
12856	7980	4928	9	1294	960	28158	626	200	892	73	124	194	93	213	282.08	202	447.72	496	344	428	26	96	15	14	4
14174	7678	4430	5	876	672	27914	645	300	1047	162	78	275	100	156	269.54	251	432.78	494	420	404	21	98	21	6	2
7718	10958	7746	306	1022	682	28242	904	40	790	15	114	97	19	159	290.09	114	445.74	422	409	375	30	55	1	2	3
12230	8038	5156	1	1504	1148	28308	606	128	805	39	218	192	70	228	291.33	150	450.88	473	303	402	65	109	5	10	7
3894	13710	9970	977	300	162	28046	1304	3	583	0	10	70	3	79	276.01	79	428.62	257	281	269	16	46	0	0	1

SUPPLEMENTARY INFORMATION:Monte Carlo Atomistic Simulation and Machine Learning Analysis of Na-K Eutectic Alloy in Condensed Phases, D. Reitz and E. Blaisten-Barojas, George Mason University, Fairfax, VA 22030

5052	12914	9280	650	540	324	28132	1116	4	704	0	22	49	2	83	280.82	126	436.17	384	361	345	10	26	0	0	5
14258	7674	4382	6	828	668	27906	625	301	1046	150	90	254	114	167	272.63	250	435.19	475	422	451	21	90	18	6	2
4102	13542	9816	952	370	220	28062	1308	9	573	1	12	62	3	86	271.91	81	425.77	256	281	265	12	46	0	0	3
7716	10994	7698	261	1084	700	28254	838	37	793	14	58	101	21	145	288.96	147	447.42	489	378	396	22	61	1	4	5
5326	12728	9092	626	600	378	28146	1123	6	730	0	22	69	5	97	282.91	126	436.79	375	370	310	16	47	0	0	1
6210	12022	8574	521	788	522	28184	1006	21	689	8	68	84	12	111	285.04	103	442.13	400	334	360	31	54	0	0	3
3860	13732	9982	993	302	162	28042	1297	10	557	1	4	48	4	72	271.14	85	423.48	270	256	294	9	31	0	0	3
13234	7714	4674	4	1364	1028	28160	617	232	819	86	136	218	105	198	282.21	171	446.32	476	314	417	56	99	18	10	4
14278	7696	4416	5	816	614	27888	649	337	1041	164	68	247	130	138	269.58	266	433.31	509	448	427	19	79	17	0	0
10942	7848	5626	0	2218	1662	28746	469	79	528	8	392	155	49	298	332.52	109	473.78	483	139	387	127	92	3	54	26
5576	12504	8964	585	658	412	28154	1092	16	702	3	38	73	6	92	283.51	105	439.98	390	366	316	22	43	1	2	3
4692	13136	9504	753	488	272	28106	1192	3	654	0	14	55	2	85	277.21	87	434.82	332	342	318	12	35	0	0	2
14176	7688	4356	6	902	730	27936	659	300	1008	154	76	256	115	161	268.81	259	431.68	486	419	414	21	84	18	8	0
11396	7960	5414	1	1972	1492	28578	520	104	587	25	308	161	50	293	314.38	110	460.13	459	197	412	95	71	6	32	21
10972	7948	5564	0	2106	1656	28704	530	76	541	13	414	162	38	272	320.51	97	466.3	464	181	359	133	82	6	42	26
4650	13164	9534	783	470	262	28096	1220	8	668	0	14	54	5	75	274.6	103	429.25	329	355	300	14	33	0	2	1
4012	13616	9900	965	334	180	28050	1309	8	585	2	6	65	3	70	271.27	81	424.13	280	290	257	10	41	0	2	2
11940	8016	5196	6	1672	1308	28404	562	132	725	29	232	188	71	268	300.81	154	455.56	476	256	403	65	111	6	40	12
4288	13366	9702	874	452	266	28092	1258	6	624	1	16	58	3	79	274.66	101	429.2	295	304	286	12	47	0	2	6
14200	7722	4400	5	828	672	27910	631	301	1074	154	78	248	115	161	269.77	266	433.68	486	441	448	17	83	13	8	2
4984	12856	9334	680	598	332	28128	1140	14	666	3	22	70	7	91	279.95	106	435.05	367	338	309	15	47	0	2	1
12040	8064	5118	1	1608	1302	28400	573	120	759	28	238	159	60	248	298.78	164	451.8	500	265	415	58	90	12	22	13
4604	13152	9526	750	502	304	28120	1181	0	663	0	30	71	0	95	277.02	102	432.42	332	344	300	16	51	0	2	1
14490	7716	4324	5	712	556	27844	646	323	1110	162	44	270	123	149	267.39	288	429.75	482	455	430	16	98	17	2	1
11552	7842	5396	0	1950	1460	28570	524	106	600	26	338	185	60	280	315.17	126	461.68	469	185	387	98	98	4	30	20
4442	13370	9672	820	374	200	28064	1196	5	641	0	6	62	1	81	276.2	105	431.64	325	306	308	12	40	0	0	3
5080	12866	9276	648	540	328	28126	1099	8	680	2	32	63	5	100	278.8	99	435.87	358	357	359	17	36	0	4	2
12540	7966	4950	2	1472	1150	28276	597	185	800	54	176	189	92	234	292.27	186	449.36	468	302	423	55	94	18	22	10
14244	7662	4396	6	894	654	27902	634	320	1015	155	52	264	128	156	272.99	256	436.49	496	407	421	17	93	16	0	4
3930	13740	9974	977	250	128	28028	1288	9	589	1	6	59	4	59	269.73	99	422.95	291	266	283	11	44	0	0	2
7780	10782	7644	285	1186	784	28288	869	45	729	11	102	102	28	157	288.81	120	444.75	462	380	359	33	52	1	10	2
14252	7668	4410	9	828	648	27896	637	311	1048	157	82	260	114	152	270.75	257	436.09	497	412	427	17	92	20	8	3
13394	7732	4648	3	1262	950	28118	611	219	880	85	118	220	111	196	282.14	208	445.23	509	340	418	32	107	7	14	3
13124	7928	4828	5	1232	896	28118	636	196	936	72	96	193	93	183	282.69	208	448.22	490	349	432	42	108	12	12	4
12284	7708	5080	2	1686	1306	28386	577	152	715	39	290	183	78	254	300.98	146	452.01	463	264	403	73	100	9	32	12
7684	10858	7690	305	1208	770	28288	885	43	748	10	68	85	28	147	289.58	142	445.74	464	351	378	24	53	1	10	7

SUPPLEMENTARY INFORMATION:Monte Carlo Atomistic Simulation and Machine Learning Analysis of Na-K Eutectic Alloy in Condensed Phases, D. Reitz and E. Blaisten-Barojas, George Mason University, Fairfax, VA 22030

13874	7652	4464	10	1066	840	28018	639	286	923	138	110	239	113	193	276.16	193	443.68	462	375	422	32	91	17	12	2
5240	12670	9114	637	682	428	28168	1115	12	656	2	32	54	5	101	280.95	93	437.49	369	342	335	16	37	0	2	2
13588	7868	4658	4	1024	770	28000	612	261	975	112	86	230	121	200	278.19	230	443.1	465	363	446	26	95	11	6	4
4390	13302	9660	844	464	262	28094	1225	3	594	1	16	68	2	78	277.34	94	432.34	317	294	291	15	44	0	0	3
5352	12700	9130	616	576	344	28136	1124	14	730	3	34	62	8	81	280.88	111	437.68	387	392	322	14	47	0	0	3
4076	13496	9846	926	424	224	28074	1257	8	562	1	8	57	6	73	271.49	74	425.06	294	272	297	16	42	0	0	1
7736	10870	7676	259	1148	764	28304	837	34	800	4	100	86	20	159	288.84	150	448.04	461	380	401	35	57	0	8	8
3970	13658	9900	965	326	192	28056	1307	11	562	1	10	60	8	75	272.27	84	425.06	267	284	270	13	42	0	0	2
12044	8068	5130	2	1628	1276	28384	589	147	729	33	216	170	73	238	300.26	169	453.32	505	260	390	59	92	11	20	14
13704	7708	4618	1	1110	788	28024	591	257	933	109	94	223	114	185	277.46	225	444.61	493	333	458	36	99	14	2	0
12536	7972	4954	1	1484	1140	28266	654	144	830	45	160	161	79	207	284.82	165	450.18	480	330	413	49	87	5	20	9
5214	12776	9206	646	582	334	28134	1150	7	709	1	20	56	6	81	278.97	101	434.73	352	395	331	22	34	0	2	3
11058	7886	5498	1	2164	1654	28668	512	79	556	18	374	185	37	289	315.1	101	462.73	489	185	362	106	96	11	32	25
10966	8042	5464	2	2076	1692	28672	558	75	556	11	404	161	33	286	315.85	100	461.11	474	183	349	107	88	5	26	23
13478	7956	4694	5	1026	776	28014	644	247	998	111	84	212	104	172	275.37	235	439.55	475	385	450	34	88	12	0	1
4850	13154	9446	760	384	222	28068	1185	9	691	0	12	56	6	72	275.39	97	430.2	337	351	326	14	40	0	0	2
14222	7678	4374	6	858	692	27916	666	316	1020	162	88	249	114	164	269.94	249	432.42	456	425	435	26	74	23	4	0
3756	13774	10006	1012	322	190	28056	1313	6	550	1	8	44	2	62	270.48	92	422.7	278	249	279	12	24	0	0	3
12934	7780	4816	3	1430	1074	28208	576	203	807	79	164	213	96	238	285.49	168	448.59	466	309	435	48	95	8	10	7
13238	7844	4798	6	1186	880	28086	612	208	953	82	134	232	100	197	280	213	446.19	469	346	430	44	122	11	4	5
5638	12452	8920	598	694	428	28166	1083	14	694	2	34	57	9	99	283.78	128	440.43	382	353	351	19	32	0	0	3
13330	8068	4814	7	1018	726	28012	669	235	998	117	50	244	89	184	277.44	221	441.21	462	421	402	28	93	14	6	3
12184	7984	5136	1	1636	1202	28344	585	129	741	39	186	179	60	250	300.5	156	451.76	470	260	403	68	93	8	10	10
14314	7782	4396	6	760	576	27864	659	327	1069	153	34	250	135	155	269.61	267	432.03	467	443	440	19	82	18	2	0
12254	8030	5130	4	1554	1162	28340	585	146	798	44	184	188	72	243	298.13	167	452.53	498	283	392	62	104	11	26	5
13308	7702	4672	3	1312	996	28148	603	222	858	85	148	217	101	206	283.63	198	446.94	493	307	417	43	108	13	8	4
3942	13690	9942	973	302	160	28038	1292	6	581	1	2	61	3	64	270.84	96	423.65	272	269	287	14	41	0	0	5
5624	12562	8996	595	582	338	28126	1087	11	763	2	24	73	7	98	283.2	116	437.13	385	388	336	14	53	0	0	1
11990	7834	5128	0	1804	1398	28460	558	117	712	30	282	183	62	244	304.26	154	457.68	494	245	388	75	96	3	24	23
13414	7946	4726	5	1062	796	28038	619	244	959	108	90	210	106	190	276.28	232	441.86	486	355	457	25	86	17	4	2
13786	7532	4410	1	1224	982	28088	591	268	850	116	134	222	116	216	277.42	160	445.18	443	318	463	49	88	14	20	3
11058	7838	5424	0	2236	1754	28742	508	88	504	17	378	153	45	291	321.74	93	465.54	473	163	378	117	74	9	42	33
4766	13046	9448	732	528	306	28120	1158	9	638	0	26	61	5	85	278.09	90	435.42	350	336	322	17	32	0	0	2
11098	7998	5500	2	2070	1594	28638	518	77	568	17	328	159	37	300	317.7	101	460.56	478	183	382	93	80	7	46	24
4868	13006	9396	708	512	304	28116	1174	10	663	1	30	58	4	89	278.18	97	434.53	330	366	319	21	30	0	0	1
11796	7956	5182	1	1762	1432	28460	555	125	675	27	302	188	66	271	298.89	137	454.82	477	232	374	83	101	14	30	15

SUPPLEMENTARY INFORMATION:Monte Carlo Atomistic Simulation and Machine Learning Analysis of Na-K Eutectic Alloy in Condensed Phases, D. Reitz and E. Blaisten-Barojas, George Mason University, Fairfax, VA 22030

3894	13690	9952	1015	320	182	28050	1337	8	553	1	12	58	5	75	271.9	78	424.64	243	264	267	12	46	0	0	4
4974	12844	9308	702	632	360	28136	1136	5	640	0	16	68	3	90	279.88	95	435.46	361	325	315	23	50	0	2	0
14108	7630	4404	3	958	750	27950	633	328	982	161	94	269	128	169	272.56	249	436.74	484	394	408	31	89	17	4	0
5370	12690	9134	630	578	324	28112	1130	13	731	1	16	71	7	85	281.37	112	437.41	366	388	320	19	51	0	0	0
4218	13388	9730	897	472	274	28096	1272	5	561	1	14	60	3	83	275.92	77	429.96	278	283	280	20	43	0	0	2
4252	13500	9742	890	352	212	28068	1241	3	603	0	10	56	3	74	275.4	94	428.03	301	282	308	13	32	0	0	3
4092	13668	9860	940	250	156	28040	1273	3	601	1	12	60	2	66	272.79	95	426	288	288	299	10	38	0	2	3
4238	13474	9752	912	376	220	28072	1259	10	582	2	10	64	6	79	274.41	90	427.83	303	277	281	8	41	0	2	2
12892	7730	4796	3	1514	1136	28254	612	211	769	56	180	169	119	216	285.8	168	448.45	497	312	431	45	79	17	6	7
5230	12754	9176	641	606	352	28134	1121	5	700	0	16	66	5	87	280.06	107	436.43	378	366	325	18	49	0	0	0
14108	7734	4492	2	870	636	27904	617	304	1031	152	62	271	116	158	274.48	264	438.84	499	388	422	23	103	14	2	2
4298	13274	9634	809	562	348	28140	1216	2	571	0	24	54	2	69	278.37	94	432.7	344	294	291	19	37	0	0	3
11416	7796	5318	0	2084	1616	28614	532	111	574	25	338	165	55	284	303.75	104	458.41	483	205	389	92	90	5	44	21
13028	7800	4686	1	1380	1120	28200	586	211	793	65	178	187	98	228	281.18	175	447.02	478	291	440	40	95	18	8	12
4550	13144	9546	782	540	316	28120	1174	7	610	0	22	55	5	95	277.85	87	434.02	321	305	323	23	38	0	2	0
5436	12488	9036	602	724	440	28176	1111	15	673	1	52	56	9	105	280.81	90	438.16	364	371	332	18	37	0	0	5
14440	7602	4316	5	826	628	27876	638	332	1050	164	62	270	129	158	268.96	263	431.25	491	433	421	15	91	18	2	1
13564	7706	4624	9	1162	872	28054	650	248	895	95	114	203	104	180	276.53	199	442.75	463	354	439	44	91	25	10	1
11622	7954	5276	3	1922	1448	28508	550	102	660	22	266	167	58	279	299.6	134	457.15	477	223	403	81	92	7	16	13
4350	13354	9660	841	440	274	28102	1229	6	604	0	22	58	5	83	277.1	87	433.35	296	303	306	22	39	0	0	2
4040	13644	9892	941	294	162	28040	1301	8	599	2	8	60	4	70	271.12	81	423.49	270	313	277	12	37	0	0	3
4028	13526	9868	942	414	226	28076	1293	5	566	0	14	71	3	64	272.06	80	425.69	294	282	256	15	45	0	0	4
13536	7918	4706	6	992	746	27990	618	281	975	122	84	229	125	167	276.38	233	439.18	488	387	454	33	85	17	6	2
3938	13724	9914	966	288	178	28048	1323	11	583	1	6	48	5	72	269.71	81	421.85	258	293	279	10	33	0	0	3
4816	12968	9398	702	580	342	28132	1121	4	649	0	26	66	4	95	280.03	110	436.62	353	315	340	18	47	0	2	4
4662	13126	9522	768	500	278	28108	1203	5	662	0	20	53	3	80	277.9	94	433.48	328	342	315	15	38	0	0	2
4538	13310	9588	829	396	234	28076	1208	6	636	1	10	58	3	81	273.52	108	427.27	322	307	311	12	38	0	0	1
12056	7826	5024	1	1766	1452	28448	545	141	681	34	284	173	71	256	299.85	144	453.12	487	226	416	76	90	13	36	16
12698	7868	4896	2	1476	1126	28252	579	157	808	46	174	191	84	196	290.28	161	450.94	508	281	433	59	111	11	12	12
4270	13444	9772	899	372	190	28054	1279	10	623	1	6	62	7	58	273.52	84	428.59	300	314	275	18	38	0	0	1
4286	13400	9736	872	422	224	28072	1240	8	613	1	4	53	5	78	272.96	94	426.98	305	297	305	11	37	0	0	2
5436	12502	9054	560	728	416	28168	1097	7	694	0	32	59	6	95	282.44	93	439.5	384	385	337	21	38	0	0	1
6766	11558	8254	404	968	614	28234	967	31	706	9	74	85	18	137	285.8	104	444.15	405	375	359	33	51	0	0	4
14430	7610	4330	4	826	624	27886	627	335	1021	156	62	246	139	153	271.23	265	434.69	489	408	460	20	82	23	4	0
14560	7632	4342	5	726	524	27828	658	338	1117	174	42	281	116	142	271	280	432.12	472	453	418	22	99	26	2	1
5214	12788	9208	641	562	330	28130	1128	6	713	0	26	66	3	87	282.03	114	437.39	389	377	309	12	38	0	2	0

SUPPLEMENTARY INFORMATION:Monte Carlo Atomistic Simulation and Machine Learning Analysis of Na-K Eutectic Alloy in Condensed Phases, D. Reitz and E. Blaisten-Barojas, George Mason University, Fairfax, VA 22030

4216	13450	9778	885	386	218	28068	1229	6	614	1	20	63	4	70	273.82	97	426.9	316	286	299	13	49	0	0	4
3974	13612	9890	946	374	212	28070	1288	6	548	1	8	53	3	61	271.2	83	424.46	290	267	283	18	34	0	0	2
7718	11008	7716	298	1012	688	28236	857	48	782	12	82	110	30	156	288.66	140	445.09	441	375	386	34	65	1	10	5
14476	7612	4356	5	772	578	27866	658	332	1081	166	66	263	130	154	270.06	273	433.47	463	445	434	22	88	16	6	1
5338	12654	9116	631	642	372	28144	1120	12	671	0	22	67	9	114	281.07	85	437.88	350	370	326	16	42	0	0	0
13680	7724	4530	2	1124	880	28060	608	250	944	104	110	210	115	175	277.22	211	443.71	484	369	461	42	89	11	12	6
14154	7666	4470	0	880	654	27908	605	321	1031	163	76	251	128	134	271.68	272	434.75	535	410	440	22	77	14	8	3
5564	12560	9006	579	622	362	28134	1078	12	736	1	20	63	7	89	281.15	126	437.4	380	380	354	25	40	0	0	2
14156	7752	4448	3	836	646	27914	634	315	1034	155	74	250	120	157	273.85	277	438.64	484	421	439	26	80	18	2	1
13096	7752	4738	0	1378	1046	28168	595	231	811	80	144	197	120	203	282.96	174	444.75	480	316	456	48	86	7	12	5
11508	8194	5362	2	1742	1370	28464	573	108	715	19	266	168	59	250	303.01	148	457.01	475	259	398	87	99	7	20	15
4848	12904	9382	698	628	356	28144	1124	6	645	0	26	51	5	88	278.23	112	435.69	378	310	344	10	33	0	0	3
10938	8030	5626	1	2140	1560	28640	523	72	568	20	310	154	34	262	315.31	120	464.63	486	170	379	125	88	2	34	16
11778	8080	5284	2	1732	1302	28424	568	112	736	26	226	179	63	265	301.99	145	452.35	476	252	404	68	102	11	20	13
6356	11838	8498	456	902	560	28218	982	25	703	4	62	64	13	127	283.98	92	443.04	401	358	377	36	37	1	2	0
3860	13700	9958	988	334	194	28058	1321	7	563	2	12	53	4	69	270.76	82	423.22	273	276	266	11	36	0	0	1
5458	12600	9058	629	618	370	28136	1092	15	677	1	30	64	8	96	283.97	103	438.52	370	339	342	23	52	1	2	2
4614	13102	9530	772	546	304	28116	1198	6	633	0	20	59	5	74	274.71	111	429.01	344	317	301	18	38	0	0	1
4394	13404	9690	838	368	202	28064	1201	7	615	0	6	62	6	81	273.55	95	428.56	320	302	320	10	36	0	0	2
12168	7858	5102	1	1702	1298	28398	556	125	711	41	246	181	62	247	301.08	154	455.66	486	263	428	73	88	9	24	10
13786	7810	4528	7	960	788	27994	619	265	968	118	120	241	105	175	276.49	230	441.24	476	372	437	37	107	21	2	3
12882	8022	4954	2	1232	908	28136	647	171	926	49	132	183	92	190	282.77	186	446.58	482	373	440	40	101	11	6	2
11360	7930	5380	2	2080	1524	28564	531	86	602	20	258	171	44	255	310.19	122	455.42	513	198	389	89	95	5	32	21
12684	7792	4920	0	1514	1154	28290	565	179	768	47	206	167	98	223	287.26	166	450.1	509	276	455	47	92	16	20	11
13496	7640	4564	4	1266	998	28134	606	242	816	89	160	202	110	209	285.7	176	448.52	466	319	445	48	86	19	8	2
13518	7826	4696	3	1106	792	28022	610	248	952	113	72	237	101	170	275.84	222	442.16	498	372	432	38	99	14	12	3
11910	7974	5224	1	1758	1308	28424	567	143	700	33	228	175	78	257	301.05	150	451.96	496	258	395	64	92	5	20	13
4460	13262	9628	846	474	258	28090	1243	5	602	1	8	58	2	74	274.45	88	430.98	304	311	295	19	37	0	0	2
4360	13356	9692	862	426	234	28078	1258	7	616	0	10	61	4	71	274.79	84	429.18	302	322	283	19	46	0	0	0
5326	12732	9122	670	572	350	28124	1132	15	691	6	20	71	7	113	280.04	97	437.9	346	353	317	10	48	0	0	2
12194	7954	5088	4	1638	1248	28350	597	139	752	34	210	169	77	219	293.39	148	454.75	471	272	433	84	96	9	16	5
14198	7626	4428	5	920	676	27924	649	296	1018	149	66	271	104	149	272.56	251	436.42	514	409	389	20	102	23	10	1
4222	13376	9710	864	480	298	28114	1235	3	560	0	28	57	3	76	276.19	81	431.63	311	274	298	17	39	0	0	2
14380	7680	4376	4	788	590	27870	630	343	1045	166	54	270	141	173	269.83	260	431.34	464	425	443	16	92	13	2	1
4762	13138	9486	758	444	246	28090	1196	4	667	0	14	54	4	84	278.86	102	435.5	330	353	323	8	38	0	0	2
5078	12876	9282	717	524	314	28104	1156	14	681	3	30	77	8	80	282.68	93	439.04	352	362	303	23	48	0	0	1

10794	7860	5616	1	2294	1754	28788	469	73	474	10	422	187	36	292	330.28	80	471.39	484	160	357	139	88	3	42	27
13720	7676	4490	4	1116	906	28048	627	257	913	96	120	215	115	176	276.83	203	443.58	465	350	445	48	100	17	18	5
11938	7854	5138	2	1834	1400	28442	604	118	698	25	254	177	69	235	297.46	140	453.01	490	260	388	67	92	9	24	20
11816	7874	5282	2	1804	1372	28480	559	117	687	25	308	175	66	262	308.55	123	458.44	442	226	415	93	103	7	20	20
4024	13578	9884	953	368	196	28056	1294	8	590	1	6	49	3	57	272.42	87	425.89	294	287	281	15	37	0	0	2
14446	7604	4358	10	806	588	27862	656	344	1050	153	60	258	145	151	271.76	268	435.67	470	432	439	17	94	26	0	3
3990	13612	9868	943	366	220	28066	1305	10	570	2	10	55	7	67	270.28	82	422.12	276	298	277	16	34	0	0	1
4504	13312	9610	833	402	234	28074	1204	9	627	2	12	59	4	75	273.46	107	427.73	326	296	315	11	42	0	0	4
11692	8136	5280	1	1684	1352	28440	576	111	706	19	264	168	62	266	302.84	131	454.46	470	247	408	68	100	13	32	14
4448	13368	9658	843	370	210	28066	1217	4	648	0	12	61	1	73	274.55	103	430.41	312	314	314	13	45	0	0	5
7180	11178	8022	353	1090	692	28264	925	40	720	13	96	91	16	144	288.48	107	445.44	437	377	353	35	54	2	6	2
4934	13010	9340	715	502	310	28116	1148	7	677	2	18	65	3	86	275.95	111	430.59	356	342	323	12	37	0	2	5
13680	7920	4638	11	936	708	27950	643	274	1015	135	60	252	104	179	274.12	256	439.12	478	396	419	21	90	17	8	0
14114	7634	4400	8	966	744	27946	632	308	951	151	84	256	114	166	272.26	234	435.6	488	368	421	26	90	28	4	2
14320	7648	4428	8	844	590	27880	674	333	1038	160	48	248	133	139	272.05	265	435.35	488	454	423	16	78	22	2	3
12762	7934	4968	3	1348	1008	28198	645	193	878	52	160	175	104	199	284.17	186	448.08	480	355	426	48	97	11	18	5
14206	7686	4430	2	874	646	27906	682	310	1046	145	62	242	121	166	272.48	240	436.68	448	427	434	19	88	18	2	2
5326	12796	9118	581	522	340	28132	1065	7	722	1	30	56	3	84	283.23	105	437.71	403	388	363	16	34	0	0	4
4046	13640	9876	934	314	172	28048	1270	9	567	1	0	56	5	67	273.36	99	426.84	296	274	289	12	36	0	0	2
5226	12694	9152	604	662	396	28158	1094	10	652	1	28	66	6	101	282.85	91	438.62	384	357	327	17	40	0	0	2
14024	7734	4536	6	882	652	27912	621	295	1027	154	82	258	118	149	271.28	247	435.92	501	420	439	25	86	14	2	2
12168	7948	5112	3	1628	1250	28346	594	139	730	45	206	171	73	233	290.37	149	450.06	484	269	418	68	86	6	34	5
6308	11952	8554	472	822	500	28180	1002	28	736	9	40	81	15	111	286.72	113	443.83	419	383	350	27	50	1	2	0
14520	7648	4320	7	732	566	27848	664	343	1064	175	60	262	126	135	270.29	278	433.5	493	449	428	14	72	24	2	1
5426	12644	9062	572	632	370	28148	1101	11	703	0	14	65	4	106	280.51	115	437.89	371	392	328	16	47	0	0	2
5654	12498	8974	564	632	354	28128	1065	13	748	1	16	72	9	92	281.44	135	437.67	388	375	352	23	49	0	0	1
3780	13670	10000	981	392	212	28066	1290	8	519	1	12	61	4	69	271.84	87	424.85	278	245	278	17	34	0	0	3
4258	13496	9752	895	336	202	28060	1264	10	620	1	16	59	8	76	271.04	89	424.47	282	304	295	15	40	0	0	3
13746	7850	4606	8	978	722	27972	643	272	1008	131	64	226	107	151	275.38	259	438.28	492	387	444	32	80	17	6	2
11902	7888	5142	0	1810	1434	28486	549	110	650	32	258	180	51	260	302.59	130	457	482	210	385	82	92	8	46	22
12630	7776	4954	0	1590	1156	28304	557	158	769	43	178	177	78	238	295.22	150	455.46	487	263	436	55	81	9	20	10
11460	7832	5382	1	2058	1510	28564	542	107	601	25	270	174	51	261	310.42	119	458.46	483	199	376	105	95	10	52	18
14206	7602	4414	5	946	686	27926	626	316	1005	158	70	260	116	148	272.46	248	438.23	510	410	425	23	91	22	2	3
4488	13204	9584	796	522	302	28120	1222	3	614	2	20	65	1	76	276.18	91	433.05	334	323	284	14	40	0	0	3
4458	13362	9652	836	380	210	28068	1230	10	638	1	6	49	5	68	274.83	93	429.01	303	316	319	22	31	0	0	0
4440	13410	9640	837	346	216	28062	1217	7	646	2	8	61	3	70	274.59	96	428.56	324	313	305	11	42	0	2	4

SUPPLEMENTARY INFORMATION:Monte Carlo Atomistic Simulation and Machine Learning Analysis of Na-K Eutectic Alloy in Condensed Phases, D. Reitz and E. Blaisten-Barojas, George Mason University, Fairfax, VA 22030

5808	12416	8846	546	652	402	28150	1064	11	754	1	24	67	7	102	281.11	120	437.57	400	381	344	13	47	1	2	1
14176	7692	4412	6	886	674	27906	604	318	1022	152	62	244	135	158	267.98	271	429.45	510	397	455	16	85	15	4	2
11070	7940	5420	0	2148	1702	28688	476	82	534	14	360	173	45	305	324.05	96	464.66	470	167	389	112	93	5	44	26
13580	7794	4622	8	1094	828	28022	611	254	964	112	98	230	104	193	275.08	232	441.69	486	365	443	24	90	16	6	3
13640	7910	4696	8	936	694	27962	637	273	1026	116	80	230	127	175	275.8	241	439.1	461	411	457	29	98	16	6	2
12092	8172	5274	4	1452	1088	28298	573	125	854	37	208	177	67	216	294.24	181	451.89	491	297	441	69	94	7	12	9
11000	7718	5470	1	2328	1782	28752	527	85	468	22	408	159	33	293	320.53	81	465.38	479	160	346	115	65	9	42	33
13962	7626	4438	7	1056	816	28008	593	296	923	135	100	244	132	181	276	226	441.5	489	351	450	35	88	12	10	2
12624	7986	4936	1	1400	1102	28248	565	171	818	49	198	175	90	219	286.15	193	448.36	525	295	440	43	95	12	2	7
11110	7830	5592	0	2152	1582	28690	465	104	546	12	384	160	59	311	324.61	101	468.12	490	168	397	110	76	6	40	20
5410	12682	9126	612	572	312	28114	1090	15	737	2	10	72	9	87	279.77	132	435.88	389	367	333	17	44	0	2	2
3934	13730	9938	977	262	160	28038	1301	7	595	1	14	61	4	64	270.15	93	423.13	276	282	276	14	44	0	0	2
4220	13486	9780	886	362	202	28062	1262	11	601	1	12	55	5	60	273.7	87	425.98	312	309	284	13	39	0	0	4
13026	7786	4726	1	1400	1096	28210	627	224	782	71	162	196	114	203	285.35	182	446.72	491	299	409	42	88	7	14	11
5076	12892	9272	643	542	322	28126	1137	5	675	1	22	64	2	84	279.18	103	434.65	374	377	322	14	38	0	0	1
6406	11930	8512	437	798	488	28182	963	23	757	3	48	74	15	127	287.53	125	442.62	424	387	378	20	38	0	0	2
7252	11194	7930	337	1072	726	28278	912	36	734	8	96	97	24	154	287.01	126	446.31	435	374	350	31	55	0	6	7
14316	7618	4392	7	880	634	27898	600	313	1023	154	52	249	123	167	273.57	256	436.91	489	386	465	19	83	18	6	2
10576	7710	5498	0	2522	2046	28912	483	55	429	7	500	142	24	302	333.28	84	471.61	496	142	351	127	67	4	52	46
12984	7792	4698	2	1428	1142	28226	637	188	775	70	168	187	86	214	283.43	176	449.11	475	301	420	41	85	16	14	8
4110	13588	9846	926	316	178	28048	1267	6	601	1	10	66	5	72	271.31	95	423.86	290	287	285	14	45	0	0	1
6486	11796	8454	441	882	530	28196	991	32	737	8	42	77	20	131	288.03	108	444.32	410	384	365	19	47	0	6	1
4206	13408	9746	883	450	264	28094	1276	9	582	1	20	52	6	79	274.55	83	429.32	289	293	279	14	34	0	0	3
3814	13726	9984	994	328	190	28054	1311	7	538	1	12	60	4	59	271.35	87	423.02	282	263	263	14	38	0	0	4
5228	12736	9196	654	608	344	28134	1117	7	663	0	22	78	2	100	279.44	80	436.58	360	367	308	25	59	1	0	0
11942	8030	5090	5	1720	1384	28428	585	116	716	33	236	177	65	258	297.37	168	451.52	501	240	392	58	95	8	22	10
14288	7520	4308	1	996	756	27954	619	318	969	154	82	240	126	168	273.5	220	438.06	484	390	453	28	78	15	4	1
4348	13404	9704	848	392	216	28070	1230	7	611	1	6	57	2	68	274.17	89	429.14	311	316	305	18	33	0	0	3
13942	7790	4530	3	906	694	27944	643	288	1008	134	70	240	107	174	273.78	218	437.05	463	419	440	29	94	22	10	0
5168	12794	9214	661	586	346	28132	1151	7	679	0	24	63	3	77	280.41	107	436.67	355	371	317	26	35	0	0	3
11732	7906	5238	1	1868	1448	28518	541	115	642	20	296	163	55	269	316.49	133	461.25	462	224	400	90	89	11	28	28
14112	7672	4446	4	924	698	27936	647	318	989	158	82	252	114	162	274.98	232	438.6	486	402	422	22	81	18	0	2
7388	11012	7804	340	1148	818	28312	915	38	685	11	130	83	16	155	290.66	132	446.53	440	357	349	34	38	2	8	7
4500	13166	9566	789	544	320	28120	1194	4	591	0	24	53	2	76	277.29	88	430.91	343	313	305	17	37	0	0	3
4120	13540	9818	895	372	208	28062	1226	9	580	2	4	49	4	71	271.47	93	425.81	296	261	327	19	36	0	0	4
4814	12950	9422	722	604	322	28122	1146	9	631	2	10	54	5	90	280.44	87	435.19	346	322	335	19	36	0	0	2

SUPPLEMENTARY INFORMATION:Monte Carlo Atomistic Simulation and Machine Learning Analysis of Na-K Eutectic Alloy in Condensed Phases, D. Reitz and E. Blaisten-Barojas, George Mason University, Fairfax, VA 22030

5856	12308	8816	531	704	432	28156	1051	8	742	2	40	71	5	113	283.42	111	440.95	378	382	358	21	44	0	0	2
6722	11718	8336	407	858	516	28190	962	33	755	5	36	75	21	127	287.35	117	444.22	424	396	376	21	47	1	4	3
5046	12752	9238	651	672	416	28170	1125	5	664	0	40	66	2	95	280.6	98	437.23	367	342	316	22	45	0	6	2
13596	7644	4590	0	1214	906	28084	599	239	888	92	122	194	118	195	278.25	199	443.97	472	330	490	37	85	13	12	4
5180	12656	9180	620	704	408	28158	1092	5	655	0	30	71	2	108	277.38	101	437.94	381	335	316	17	44	0	0	2
5928	12320	8792	564	658	408	28144	1056	14	765	1	36	71	9	105	284.79	124	440.45	368	367	362	27	52	0	2	2
14298	7648	4424	7	852	608	27888	605	315	1031	153	56	253	125	162	272	248	436.89	490	391	458	19	87	18	2	3
6360	12036	8574	508	692	432	28146	1022	28	769	7	44	74	17	117	285.16	114	442.05	372	389	372	28	48	0	8	3
4880	13086	9394	724	450	272	28098	1152	2	686	0	14	70	2	91	275.76	104	429.97	341	344	321	15	55	0	2	3
4498	13306	9628	835	412	226	28080	1229	7	646	0	10	72	5	87	275.98	100	432.74	315	311	283	10	46	0	0	0
3982	13718	9932	966	246	140	28028	1292	10	606	2	10	55	3	75	270.83	91	423.74	263	289	297	8	39	0	0	4
4356	13384	9690	848	390	234	28078	1224	5	612	1	24	66	1	70	275.91	100	428.6	325	303	294	17	41	0	0	0
7586	10978	7800	294	1104	704	28266	902	47	766	13	90	92	26	146	288.01	114	445.38	448	406	368	26	61	2	2	8
5368	12560	9086	609	712	412	28170	1078	4	693	0	32	64	2	82	281.01	122	437.72	402	349	340	23	48	0	0	4
11568	8024	5368	2	1838	1402	28510	542	111	627	21	264	171	54	274	313.41	127	458.04	463	199	384	96	94	8	40	21
4996	12900	9350	689	548	300	28118	1169	3	680	0	24	60	2	89	276.37	106	432.03	336	360	318	14	42	0	0	6
4970	12926	9344	679	554	308	28116	1145	1	692	0	14	73	0	73	279.32	115	435.07	384	364	305	14	50	0	0	2
4642	13058	9492	762	588	330	28124	1223	4	629	0	14	55	2	76	276.27	81	431.58	328	339	293	18	33	0	0	1
4604	13164	9522	777	498	302	28116	1207	3	658	0	22	56	2	81	276.82	92	431.67	321	341	308	20	40	0	4	2
4392	13448	9710	871	304	176	28048	1249	7	641	1	18	62	4	68	272.91	107	426.93	302	317	300	14	46	0	0	1
4266	13486	9740	894	354	208	28062	1245	6	620	1	8	67	4	69	271	107	424.1	310	284	294	11	51	0	0	3
4034	13710	9894	954	246	144	28032	1294	8	594	0	4	57	4	64	271.57	97	424.16	273	286	289	16	37	0	0	1
14428	7704	4416	10	730	518	27836	642	332	1116	166	36	265	126	145	267.93	276	429.25	482	460	440	14	95	16	4	4
14454	7680	4406	5	734	520	27840	635	349	1090	155	46	256	145	144	271.77	263	436.07	476	445	458	22	88	23	0	1
4060	13596	9870	919	320	188	28056	1253	4	585	1	22	65	3	71	272.07	98	424.97	299	284	291	13	44	0	0	3
4648	13174	9568	764	440	232	28082	1187	6	656	0	20	63	4	80	276.58	90	433.82	325	349	319	19	45	0	0	1
13866	7828	4586	2	936	668	27936	654	278	1024	141	50	246	107	175	273.87	236	439.28	460	433	430	21	86	10	2	4
4314	13414	9700	889	404	236	28076	1269	8	605	1	6	67	5	80	273.71	89	427.92	297	309	271	11	47	0	2	1
3982	13662	9912	964	308	174	28048	1292	6	594	1	10	70	5	59	270.49	102	422.52	288	278	269	16	51	0	0	3
4242	13586	9794	925	262	144	28028	1263	10	643	1	0	60	8	70	271.49	99	425.54	274	287	312	17	44	0	0	1
13392	7814	4696	7	1188	876	28072	626	221	941	94	96	218	101	195	277.37	198	443.5	481	375	437	28	100	11	8	5
7352	11188	7982	351	1004	616	28222	906	35	762	10	80	94	19	150	289.96	133	445.7	425	356	373	31	52	0	0	3
10806	7768	5558	2	2382	1812	28784	475	80	484	17	402	170	35	289	329.78	94	470.86	457	151	370	138	76	3	52	34
4280	13410	9716	878	402	246	28078	1264	8	611	1	24	67	6	78	272.63	87	425.7	293	307	279	13	42	0	0	3
5480	12608	9000	591	636	404	28156	1110	13	679	1	28	67	5	103	282.87	107	439.32	366	378	321	22	35	0	0	1
4588	13218	9560	785	444	264	28100	1169	5	617	0	22	61	4	94	276.62	91	432.55	325	299	332	15	44	0	4	0

4630	13142	9512	781	494	300	28108	1186	12	617	2	30	54	6	83	279.71	89	437.56	337	321	312	17	37	0	0	1
7516	11100	7778	332	1036	726	28262	908	42	736	9	104	86	27	145	287.95	117	444.34	427	366	388	36	49	0	2	3
4182	13526	9796	885	348	198	28060	1267	5	609	1	10	65	2	71	271.99	88	424.81	291	308	285	14	45	0	0	3
14408	7678	4332	6	802	606	27868	659	324	1076	154	38	255	127	147	268.45	263	430	476	462	435	20	86	18	4	1
3840	13668	9980	972	372	196	28062	1295	8	533	1	4	53	6	61	272.6	76	425.22	289	259	278	11	33	0	0	6
4520	13330	9632	838	362	202	28060	1223	8	638	1	14	66	5	62	275.02	94	429.24	329	320	298	15	46	0	0	2
13782	7874	4588	11	932	708	27956	663	263	1022	126	66	243	106	167	273.81	242	438.12	455	429	428	32	93	10	6	3
5554	12414	8948	621	756	458	28164	1092	15	650	4	34	79	8	109	281.42	104	438.16	378	328	319	19	50	0	0	0
5134	12698	9212	651	686	396	28156	1122	11	635	1	30	68	7	89	282.68	96	437.14	379	347	316	18	41	0	0	3
14332	7718	4458	7	772	530	27842	628	320	1107	164	30	259	124	157	267.88	274	429.91	482	433	457	12	88	17	2	0
13194	7902	4764	1	1180	918	28104	631	206	934	71	142	216	96	207	282.32	203	446.54	460	363	428	43	116	18	4	1
13692	7822	4582	11	1056	778	27992	635	259	960	122	52	229	99	168	275.27	236	442.61	481	380	427	38	92	18	10	2
10882	7966	5588	2	2142	1670	28690	524	86	548	16	402	170	38	304	316	105	462.12	459	175	363	113	89	9	38	22
11582	7846	5332	2	1984	1472	28538	524	99	634	19	288	170	58	285	310.4	128	458.09	487	209	398	90	90	3	34	12
12558	7762	4962	3	1658	1186	28312	619	155	740	40	176	157	82	235	291.01	141	449.64	468	294	434	51	84	10	10	9
11398	7854	5324	2	2074	1592	28602	573	115	563	24	336	156	58	292	312.38	105	458.78	463	196	369	92	82	8	20	15
5002	12922	9310	721	552	318	28116	1168	4	677	1	12	75	2	84	278.61	106	436.23	355	348	293	14	52	0	0	4
3774	13708	10008	1003	364	194	28052	1329	5	545	1	4	58	2	68	270.63	69	421.53	262	264	261	15	42	0	0	1
12206	7888	5094	3	1642	1272	28368	567	135	758	38	236	185	70	263	299.54	136	452.5	438	270	413	78	97	6	24	14
5214	12804	9172	664	552	356	28134	1116	7	679	0	34	73	3	86	279.82	100	436.04	381	350	319	18	50	0	2	2
11240	7906	5438	1	2046	1598	28626	537	87	616	15	342	157	35	264	319.12	109	463.91	449	206	371	125	90	8	44	33
5012	12954	9320	652	510	300	28116	1119	2	691	0	18	60	1	95	277.59	95	434.21	357	374	344	17	43	0	2	1
13954	7788	4564	2	902	656	27936	640	275	1063	124	68	235	116	160	274.91	246	439.94	480	428	446	27	96	15	4	1
5680	12334	8892	551	770	468	28192	1058	6	726	0	46	72	5	116	283.33	111	440.97	392	377	337	15	49	0	2	5
14430	7596	4302	5	860	646	27888	623	336	1018	172	50	251	125	158	267.95	264	429.38	490	420	450	15	72	17	4	4
13892	7770	4562	4	974	690	27944	641	276	1025	150	56	266	102	185	274.92	233	439.03	467	408	411	21	105	7	0	2
4486	13294	9642	805	404	226	28074	1208	7	651	1	22	69	4	73	274.97	98	430.69	323	334	303	15	40	0	0	4
4268	13524	9778	902	300	164	28042	1280	8	651	1	8	64	6	56	272.8	102	425.76	299	328	279	15	48	0	0	2
4414	13376	9680	862	374	208	28064	1254	6	655	1	12	58	1	65	273.58	113	426.51	305	325	290	18	40	0	0	2
13308	7820	4732	2	1200	906	28106	614	231	929	75	130	205	120	203	280.87	202	445.23	470	353	459	34	104	13	10	5
5540	12528	9018	605	660	376	28144	1085	16	702	1	22	72	10	103	281.42	114	440.49	384	351	335	14	49	1	0	2
4452	13316	9628	835	422	250	28086	1215	6	616	1	18	54	5	72	274.51	95	429.52	324	302	314	16	36	0	0	1
5028	12860	9282	673	606	346	28134	1148	12	654	3	12	68	4	96	280.92	95	436.57	350	356	308	21	40	1	0	0
3852	13774	9996	1000	262	142	28030	1307	8	583	2	4	61	4	76	270.71	86	422.32	252	264	280	12	42	0	0	4
14334	7544	4358	4	926	680	27918	621	333	971	149	68	246	144	177	272.56	238	436.4	467	391	456	23	84	18	4	1
13178	7720	4744	1	1382	992	28140	601	203	853	75	112	205	94	231	281.87	174	445.94	462	346	443	37	90	15	12	3

SUPPLEMENTARY INFORMATION:Monte Carlo Atomistic Simulation and Machine Learning Analysis of Na-K Eutectic Alloy in Condensed Phases, D. Reitz and E. Blaisten-Barojas, George Mason University, Fairfax, VA 22030

11814	7976	5268	1	1750	1336	28436	553	124	703	18	268	168	75	252	298.67	127	456.24	479	235	424	74	100	9	24	18
7394	11144	7842	326	1084	724	28270	912	37	750	5	80	79	25	142	289.04	108	446.38	419	377	390	47	48	0	2	1
12452	7822	5022	1	1580	1202	28326	571	147	754	35	222	196	79	241	297.2	168	452.07	449	252	417	83	110	11	26	13
12496	7902	5018	2	1524	1138	28282	576	160	815	47	192	183	77	243	293.5	173	450.65	488	286	421	56	100	8	10	4
11108	7820	5530	2	2210	1620	28678	496	74	539	11	354	158	44	287	323.39	99	463.77	497	172	396	92	86	2	34	34
14292	7730	4422	5	798	586	27876	663	325	1064	159	48	245	128	151	269.55	264	432.55	474	447	441	17	79	17	0	1
14336	7722	4396	6	762	588	27868	634	329	1061	162	62	259	128	135	271.49	288	435.56	504	416	438	21	86	20	2	2
13078	7622	4660	1	1490	1186	28256	576	234	709	83	196	209	101	218	293.59	154	452.45	495	272	401	61	87	18	22	12
5572	12612	9064	599	544	292	28100	1119	15	767	2	16	72	12	92	280.2	106	435.61	355	417	341	17	48	0	0	1
5538	12662	9082	621	510	284	28098	1086	8	743	0	22	80	7	93	283.82	128	438.47	380	361	342	14	54	0	0	1
3890	13640	9932	968	374	216	28066	1309	8	548	1	14	59	5	70	271.42	81	424.96	270	274	269	14	43	0	0	4
11618	7954	5226	1	1892	1508	28526	548	101	631	21	284	181	53	268	307.34	130	455.15	505	203	360	87	102	7	42	13
12000	7836	5144	1	1788	1376	28444	544	124	690	34	272	202	68	253	307.22	148	458.75	471	226	393	89	107	6	28	15
13606	7570	4554	3	1286	948	28096	617	272	868	104	126	207	115	195	279.42	197	444.55	477	340	454	34	83	26	4	5
4966	12992	9372	691	484	270	28102	1151	7	680	0	18	66	5	77	278.82	103	436.64	358	356	325	17	43	0	0	2
5606	12620	9042	549	532	294	28114	1087	5	807	0	20	66	2	87	280.94	113	438.74	383	436	354	13	48	0	0	5
4210	13472	9778	865	386	212	28066	1234	5	599	1	8	73	2	89	274.68	76	428.29	298	301	290	10	46	0	0	2
11156	7810	5392	2	2174	1706	28656	508	80	538	15	372	191	42	282	315.39	116	464.37	482	166	348	115	96	4	44	27
13114	7780	4728	2	1344	1044	28180	608	197	848	72	152	208	86	198	283.59	192	447.65	492	306	415	52	114	19	18	7
3922	13718	9920	965	294	186	28052	1279	5	549	1	10	55	3	66	272.87	80	426.25	282	265	298	13	38	0	2	4
5100	12856	9236	647	576	344	28126	1122	7	690	1	14	66	4	86	279.64	115	435.72	368	358	332	18	40	0	0	3
4802	13088	9444	729	470	274	28098	1175	10	650	1	18	67	6	77	275.49	90	431.26	351	355	303	15	43	0	2	3
11372	7910	5490	1	2030	1442	28548	540	85	625	16	266	175	50	288	312.43	110	460.17	467	205	374	89	100	3	36	21
14240	7612	4378	9	894	698	27918	647	337	1001	162	92	253	133	172	271.9	244	435.17	461	416	432	26	83	22	4	0
12662	7948	4928	1	1430	1102	28260	622	158	814	54	178	178	77	214	287.94	174	451.68	480	302	418	58	94	10	10	6
5076	12932	9282	652	502	298	28106	1071	7	676	1	16	72	5	97	278.93	108	435.19	368	317	365	20	53	0	0	2
11510	8058	5406	3	1828	1394	28514	573	101	658	23	282	160	52	248	303.92	142	457.63	481	217	387	96	93	6	32	14
10954	7850	5582	0	2220	1692	28756	517	77	508	9	404	169	45	302	331.68	98	469.84	472	158	354	104	84	5	50	37
4550	13158	9560	766	514	304	28120	1176	3	613	0	34	60	3	83	277.18	88	433.08	339	318	319	17	36	0	0	3
5164	12808	9260	660	536	310	28116	1122	11	689	4	36	59	5	95	280	93	436.63	348	363	344	18	34	0	2	4
4730	13070	9476	716	522	296	28118	1148	3	645	0	24	56	3	86	277.78	100	436.21	354	331	334	15	40	0	0	2
14522	7606	4342	3	788	560	27866	657	317	1086	151	46	244	126	150	269.9	285	432.81	482	438	444	13	82	24	2	1
4470	13356	9670	839	362	192	28062	1214	8	642	0	12	72	5	87	273.73	91	428.01	304	323	302	12	53	0	0	3
4292	13454	9754	893	358	190	28054	1283	4	620	1	6	56	3	76	272.32	76	425.32	271	331	295	10	44	0	0	5
12170	7842	5068	1	1742	1332	28422	558	135	682	29	244	158	74	238	302.88	156	454.88	499	239	420	72	85	11	24	17
6590	11922	8416	461	700	468	28158	996	23	791	7	60	85	14	113	287.28	147	444.18	400	394	368	25	42	0	2	5

12704	7862	4874	1	1518	1134	28254	598	168	786	53	150	189	85	224	284.1	168	449.58	485	283	429	53	102	8	12	5
7000	11548	8188	377	866	536	28196	941	29	807	5	56	98	18	145	288.64	130	445.59	422	396	358	15	63	1	2	8
14426	7686	4360	7	744	576	27854	670	323	1069	167	56	269	114	145	272.14	259	436.08	462	445	420	25	87	23	6	2
4320	13396	9706	870	400	238	28082	1265	6	607	1	20	55	3	80	273.44	81	427.28	287	321	296	13	35	0	2	2
4510	13278	9586	818	426	264	28086	1221	6	621	1	20	68	3	81	273.29	89	429.03	318	326	289	12	43	0	0	4
11718	7858	5204	0	1942	1502	28544	557	109	647	23	272	173	53	279	308.51	138	458.96	481	220	363	90	85	11	46	16
11330	7902	5432	0	2084	1520	28588	522	99	598	11	296	153	56	271	310	107	460.37	503	204	395	94	90	8	20	20
11112	7794	5560	1	2174	1610	28668	553	73	573	11	370	164	29	256	324.48	108	467.48	491	197	339	104	89	7	44	33
14398	7622	4360	4	852	608	27884	648	319	1047	149	42	232	125	144	270.36	264	433.4	490	439	462	15	71	23	2	2
4286	13490	9758	896	330	180	28050	1259	10	630	2	6	61	7	67	273.51	108	427.18	307	304	288	9	37	0	0	3
5786	12314	8842	555	754	448	28174	1069	13	719	1	30	73	6	103	285.66	124	441.14	412	351	319	12	51	2	0	3
13496	7840	4674	8	1102	828	28048	628	273	925	118	98	219	129	174	277.5	215	442.42	482	385	448	35	81	10	10	6
4906	12990	9410	754	508	270	28096	1181	11	644	2	8	63	8	79	278.95	96	434.06	346	326	310	12	40	0	4	3
4838	12970	9350	722	586	372	28150	1148	5	626	0	34	46	1	81	280.16	112	435.26	369	321	325	18	34	1	0	3
4552	13338	9640	819	336	172	28044	1238	10	692	1	6	73	3	62	274.52	114	428.8	322	362	281	14	53	0	0	2
14312	7650	4452	4	828	574	27866	627	334	1077	160	42	264	137	156	273.3	259	438.41	477	426	449	19	92	12	6	3
13344	7758	4650	5	1256	974	28128	602	221	863	89	128	216	91	199	282.12	193	447.41	492	336	424	41	104	14	18	7
14304	7680	4374	6	828	638	27892	647	307	1047	156	66	251	114	137	269.75	261	432.75	513	444	427	17	83	15	2	1
4798	13082	9462	725	476	262	28096	1153	4	685	0	16	71	4	76	276.89	113	432.87	364	336	316	14	46	0	0	2
13838	7550	4476	3	1180	886	28058	612	270	898	109	116	221	129	198	277.24	191	444.94	471	352	451	34	92	17	12	2
11842	7908	5202	0	1808	1392	28448	537	124	668	42	276	192	57	254	301.47	139	452.71	495	209	398	86	101	7	18	12
14350	7628	4346	5	850	652	27896	643	324	1048	156	62	259	131	141	269.58	265	432.23	507	427	424	20	88	19	8	1
3890	13660	9970	971	350	176	28050	1325	7	560	1	4	53	4	70	273.18	78	424.73	248	281	279	18	33	0	0	2
5504	12642	9062	616	568	328	28126	1102	8	749	2	22	63	4	93	278.91	117	435.59	370	376	345	18	48	0	0	2
4388	13336	9658	836	444	252	28086	1227	10	607	1	8	52	5	84	272.42	99	427	310	306	307	12	28	0	0	1
12444	7866	4948	4	1576	1244	28316	547	167	759	59	218	203	81	219	289.61	180	451.23	505	242	420	71	99	13	20	11
5216	12794	9194	686	554	334	28118	1159	10	706	2	26	64	4	88	278.57	123	436.72	347	355	314	19	46	0	0	1
4796	12970	9420	712	586	336	28136	1175	9	642	1	26	65	7	96	277.4	91	434.37	335	351	311	15	39	0	2	2
13108	7736	4826	3	1350	982	28178	602	199	848	64	172	186	102	199	284.31	160	450.27	485	321	450	54	100	11	4	4
14542	7588	4332	3	756	562	27848	667	335	1106	179	68	279	123	150	267.91	274	429.01	470	471	412	18	90	15	0	1
4588	13170	9550	805	490	282	28100	1198	7	618	0	16	51	5	84	276.4	81	431.73	315	310	326	17	38	0	4	3
11058	7850	5500	0	2222	1666	28698	532	73	535	16	372	169	40	282	323.85	109	467.8	476	170	360	115	85	1	30	25
4306	13440	9724	888	372	212	28062	1282	3	636	1	6	59	1	70	273.7	99	426.4	283	327	280	18	46	0	2	1
13744	7860	4614	7	948	716	27964	621	266	1020	139	74	250	100	158	275.29	245	439.46	503	393	433	27	99	14	8	3
4146	13516	9816	910	366	206	28064	1265	4	602	1	12	74	2	70	272.71	97	426.15	302	289	273	14	49	0	2	1
4036	13626	9868	936	326	190	28054	1274	6	577	1	8	56	3	70	270.66	101	423.17	295	274	290	10	37	0	0	1

SUPPLEMENTARY INFORMATION:Monte Carlo Atomistic Simulation and Machine Learning Analysis of Na-K Eutectic Alloy in Condensed Phases, D. Reitz and E. Blaisten-Barojas, George Mason University, Fairfax, VA 22030

4400	13288	9640	829	480	280	28108	1210	6	628	0	20	51	6	75	276.73	103	433.05	335	302	311	15	38	0	0	0
4716	13148	9504	758	454	254	28092	1181	6	680	0	16	62	3	84	278.91	94	435.54	341	343	312	12	44	0	0	1
14320	7652	4354	6	842	656	27896	645	310	1039	164	62	271	111	141	272.03	261	436.92	502	425	409	23	97	15	10	3
5054	12974	9314	688	462	276	28104	1157	9	717	1	24	58	4	77	277.03	115	434.19	352	377	333	17	39	0	0	2
4478	13352	9676	851	356	182	28054	1258	10	660	2	10	61	4	70	272.37	100	426.24	305	337	285	12	43	0	0	1
4386	13440	9714	861	320	174	28042	1228	10	647	2	8	66	5	62	273.65	110	427.42	319	307	301	15	45	0	0	2
4192	13482	9770	895	386	230	28078	1246	8	589	1	18	51	5	77	273.71	85	427.82	298	277	304	15	39	0	0	2
4506	13200	9588	806	514	284	28102	1213	9	622	2	10	63	5	78	275.38	95	429.58	326	308	292	18	41	0	0	2
4454	13218	9628	811	498	280	28104	1216	5	614	0	26	64	4	86	276.08	98	430.19	314	310	298	16	42	0	0	2
4100	13486	9816	914	430	238	28078	1269	5	568	0	8	63	3	70	273.46	94	427.45	301	278	275	15	37	0	0	2
12562	7912	5010	3	1512	1094	28262	609	163	796	58	164	183	78	217	292.94	153	450.65	469	293	428	57	95	11	8	11
4582	13226	9564	823	444	254	28084	1228	8	648	1	14	65	5	84	273.52	90	427.6	312	329	294	12	48	0	0	1
4084	13586	9860	918	328	184	28056	1256	7	585	1	14	55	5	85	273.48	92	427.44	278	278	309	13	34	0	0	0
4208	13582	9808	905	280	156	28040	1263	5	627	1	6	64	3	76	273.97	94	426.85	286	312	292	12	41	0	0	1
4174	13482	9770	916	408	238	28082	1283	5	586	1	10	53	4	74	274.74	93	428.25	285	292	286	14	32	0	0	2
8152	10804	7540	298	978	644	28208	861	69	785	19	84	118	38	162	289.04	141	445.71	434	368	374	25	71	2	6	8
11348	7996	5450	2	1962	1472	28560	532	99	635	15	300	165	57	269	306.69	123	456.25	523	218	371	82	88	3	26	15
13312	7756	4666	3	1250	984	28134	624	240	853	83	154	200	117	190	280.68	200	446.79	494	298	432	44	94	18	10	0
4078	13558	9834	931	370	216	28068	1280	6	579	1	12	64	4	63	271.86	91	425.34	299	280	275	16	42	0	0	1
13310	7724	4578	1	1306	1076	28174	603	214	824	72	154	188	109	199	283.02	157	446.31	492	335	444	50	83	11	24	4
12404	7868	5020	4	1610	1212	28338	535	149	713	34	202	164	85	227	293.67	156	453.17	498	224	477	71	89	12	22	8
5752	12430	8916	563	638	370	28134	1072	11	745	4	28	77	5	106	282.21	123	439.31	387	378	336	13	55	0	0	2
4488	13328	9638	844	388	216	28070	1226	4	648	0	12	63	4	68	275.65	106	431.19	325	313	299	13	43	0	0	2
3776	13806	10026	1011	264	152	28034	1316	8	565	2	10	54	5	71	269.85	81	422.59	257	262	279	14	35	0	0	1
14302	7618	4370	4	880	660	27898	602	320	1035	157	62	264	127	180	273.24	248	438.78	470	390	451	21	100	20	6	3
4028	13560	9838	932	374	248	28082	1269	7	571	1	28	58	4	71	272.96	93	427.5	299	267	278	16	42	0	6	2
13554	7894	4642	8	1028	796	28006	617	260	1000	132	80	226	101	171	275.05	252	439.82	509	400	448	19	81	11	10	4
3992	13546	9904	943	408	210	28070	1312	3	562	1	8	64	2	72	272.95	70	426.14	270	297	259	14	44	0	2	4
4146	13484	9818	892	394	212	28066	1238	10	586	2	12	60	6	81	274.2	81	426.4	297	287	301	15	38	0	0	1
7762	11042	7696	312	964	664	28216	884	46	792	13	82	106	26	150	288.22	131	447.74	431	384	370	34	53	1	6	5
5220	12724	9148	637	626	396	28152	1088	10	679	1	36	66	6	92	281.94	123	439.43	398	324	329	15	49	0	2	2
4016	13592	9896	937	360	188	28058	1265	7	571	1	6	52	3	74	271.73	87	424.92	281	275	307	16	40	0	0	1
5062	12948	9290	688	484	300	28112	1135	5	722	0	26	57	3	84	277.44	118	435.14	349	347	347	21	38	0	2	2
4898	12916	9350	702	604	346	28130	1133	10	616	0	16	57	6	97	277.76	96	433.53	364	316	328	11	38	0	0	2
12582	7798	4916	1	1566	1214	28308	558	168	735	40	208	183	93	233	297.64	151	451.28	476	255	431	74	93	8	22	9
4048	13588	9876	957	346	186	28050	1284	9	596	1	6	64	7	63	271.99	100	424.7	283	270	279	18	48	0	0	3

4058	13542	9836	933	384	230	28068	1295	7	588	1	18	71	4	74	272.76	76	425.82	281	297	260	14	56	0	0	2
4338	13358	9698	850	440	240	28080	1250	7	612	1	4	64	5	88	273.4	82	427.34	284	320	292	14	41	0	2	2
13948	7696	4520	6	980	720	27946	634	301	973	137	78	229	122	177	272.53	223	437.33	461	405	461	24	77	15	4	3
3822	13696	9980	982	356	198	28062	1276	7	527	1	10	60	3	57	271.45	99	424.44	311	232	274	14	45	0	0	2
4514	13290	9606	844	420	240	28082	1226	7	639	0	10	50	4	70	275.35	108	430.81	324	303	305	14	33	0	2	2
13190	7792	4698	2	1274	1032	28180	586	243	814	66	174	180	129	213	285.12	171	448.79	456	306	477	62	88	19	20	4
4324	13480	9732	874	316	184	28048	1238	11	628	2	12	76	4	78	271.82	98	425.94	303	304	282	13	53	1	0	2
4276	13350	9734	876	478	246	28086	1238	12	582	1	2	59	8	76	273.04	92	427.19	321	281	293	7	41	0	0	3
4272	13502	9760	875	318	186	28054	1227	6	634	1	16	63	2	82	273.98	92	427.53	299	299	312	11	48	0	0	2
14378	7678	4400	5	792	570	27866	649	343	1073	170	48	259	134	147	269.83	292	433.68	488	443	426	18	76	16	0	2
11536	7756	5316	0	2052	1552	28584	535	110	596	22	344	172	59	285	314.88	101	459.79	478	201	373	95	97	4	28	19
3926	13622	9928	980	380	202	28062	1320	6	548	1	4	58	4	64	270.26	88	423.87	278	268	260	13	41	0	0	2
12404	7800	5052	6	1626	1210	28338	572	153	759	29	226	164	77	239	299.2	164	452.05	503	274	427	54	88	17	20	9
4110	13546	9848	909	350	190	28056	1278	7	599	1	10	58	5	62	273.72	78	427.01	285	307	293	19	42	0	2	2
14276	7690	4380	8	840	644	27892	642	319	1049	152	58	241	141	134	269.63	271	430.9	511	430	447	16	78	11	4	3
5420	12620	9098	608	620	352	28136	1072	12	703	1	26	66	7	101	281.62	112	439.59	384	347	352	17	48	0	0	1
11344	7872	5322	1	2008	1652	28644	510	99	597	9	406	159	55	303	316.14	108	460.02	473	195	394	93	91	7	38	25
13564	7498	4506	2	1376	1042	28144	593	237	846	86	148	218	112	227	282.25	181	446.39	485	325	421	35	104	18	10	3
4586	13136	9566	759	520	278	28102	1172	9	622	3	16	63	4	105	276.13	89	432.7	327	314	314	8	39	0	0	2
4278	13382	9736	883	434	236	28078	1272	2	589	1	12	66	1	86	274.27	83	429.54	274	300	280	16	40	0	0	2
12040	8060	5164	5	1608	1246	28358	565	139	778	36	222	188	74	237	301.84	177	453.22	499	274	402	63	92	9	18	14
3972	13626	9916	950	332	188	28052	1275	8	566	2	18	58	5	74	272.87	93	424.86	290	269	282	9	40	0	0	3
11440	7946	5374	1	1990	1500	28582	557	100	599	19	304	162	50	265	316.21	123	460.95	496	197	368	91	77	10	24	18
14288	7734	4446	6	776	570	27868	626	328	1074	155	48	258	133	159	271.7	281	433.82	480	424	451	18	91	17	6	1
5600	12532	8982	592	618	370	28130	1081	22	689	7	28	76	13	102	280.12	111	438.88	370	359	344	21	45	1	0	1
4154	13602	9844	921	270	152	28036	1288	4	632	1	14	69	2	63	272.14	91	425.6	291	318	268	10	47	0	0	4
5600	12298	8918	546	834	504	28208	1065	17	656	2	48	60	14	120	285.5	87	441.96	383	353	342	17	35	0	6	5
11138	7866	5512	1	2178	1608	28684	513	76	527	14	342	164	34	299	326.7	98	465.51	456	172	371	128	79	2	36	17
4126	13618	9854	935	276	154	28038	1277	10	605	2	10	62	6	68	271.57	104	424.82	278	300	292	17	41	0	0	1
5224	12894	9218	645	458	282	28098	1121	8	738	2	18	79	5	93	279.19	116	436.13	358	382	327	14	53	0	4	3
11996	7928	5164	1	1744	1326	28422	576	135	685	27	238	165	80	259	303.08	132	453.89	488	253	402	67	89	9	22	8
13606	7728	4584	2	1162	870	28056	614	249	903	110	98	220	104	179	279.6	221	444.59	489	347	443	42	86	12	6	1
5338	12648	9126	677	628	362	28126	1103	19	689	1	24	54	13	91	280.01	126	436.22	372	315	348	16	38	0	0	4
11276	7802	5512	1	2144	1536	28626	530	82	560	18	312	167	43	261	315.76	106	463.37	505	185	368	94	93	4	38	32
14334	7706	4398	5	794	588	27868	662	332	1071	160	44	261	126	138	268.4	273	429.43	499	454	409	16	90	17	4	3
5340	12706	9126	629	582	346	28124	1096	11	707	0	24	63	6	106	279.02	115	436.98	351	362	353	20	39	0	0	2

SUPPLEMENTARY INFORMATION:Monte Carlo Atomistic Simulation and Machine Learning Analysis of Na-K Eutectic Alloy in Condensed Phases, D. Reitz and E. Blaisten-Barojas, George Mason University, Fairfax, VA 22030

13056	7802	4846	5	1300	974	28154	631	211	867	79	158	202	98	191	282.91	187	446.68	476	341	421	48	101	13	18	10
14204	7688	4418	4	868	658	27908	659	310	1051	149	70	254	129	144	271.63	283	435.52	491	422	424	23	90	17	2	1
11814	7910	5218	0	1818	1404	28472	529	122	647	31	280	176	56	277	304.21	124	457.88	498	216	401	77	100	10	28	10
5260	12704	9168	611	626	358	28138	1109	9	698	1	22	68	6	88	282.44	111	437.34	388	375	327	14	45	0	0	2
4264	13386	9728	885	444	252	28090	1289	10	609	1	16	56	4	64	274.67	90	428.08	302	318	258	16	40	0	0	4
13048	7794	4700	4	1374	1108	28216	616	209	833	64	178	174	104	193	280.96	193	446.67	507	310	436	49	84	21	14	3
14158	7674	4424	3	880	694	27932	672	307	1020	150	98	252	115	163	272.32	245	437.04	473	423	409	22	89	19	4	1
14210	7666	4448	7	864	636	27898	655	316	1058	164	72	268	114	138	270.71	256	433.8	509	447	401	23	94	16	2	0
14136	7738	4514	6	830	600	27884	614	318	1058	145	64	244	128	169	274.08	262	438.64	468	407	474	20	84	19	2	2
7046	11382	8080	437	980	646	28214	951	48	689	14	76	98	25	136	290.6	102	446.91	391	330	364	38	56	2	4	7
14106	7790	4414	4	844	694	27930	620	312	1018	147	74	232	128	144	269.05	275	431.61	514	408	459	23	71	15	8	1
4586	13224	9538	793	450	276	28094	1207	7	629	0	20	58	4	84	275.69	87	430.52	310	322	316	16	41	0	0	4
4694	13114	9518	790	480	262	28086	1198	10	659	2	18	63	5	84	275.46	100	429.64	324	331	308	15	45	0	0	1
14342	7704	4432	5	794	550	27852	636	329	1088	156	28	272	134	144	268.9	292	432.01	501	429	420	18	104	17	2	0
11500	7804	5402	3	1956	1510	28590	522	98	612	22	382	191	51	299	317.59	125	463.36	483	203	349	88	105	3	30	23
4092	13564	9866	925	346	178	28052	1254	10	590	2	6	64	6	68	271.14	111	424.5	303	261	294	12	42	0	0	2
4044	13572	9854	919	382	214	28072	1299	7	579	1	6	61	4	71	271.51	80	425.65	274	297	271	19	39	0	0	1
8026	10964	7630	279	922	584	28184	890	53	872	14	58	118	30	154	290.02	144	447.46	413	432	383	30	67	2	0	4
5432	12668	9038	620	580	384	28142	1104	5	721	2	40	67	2	92	280.86	115	438.19	373	364	332	21	45	0	0	3
4500	13316	9632	838	388	216	28064	1198	13	639	3	12	68	7	73	274.06	96	429.4	328	301	311	16	49	0	0	1
5244	12654	9140	631	688	404	28154	1118	9	669	1	24	62	7	100	280.01	106	436.33	364	360	328	21	34	0	0	0
11322	7844	5382	1	2122	1588	28600	552	85	597	14	300	177	45	271	313.34	112	460.38	486	195	357	94	98	4	40	21
14486	7640	4368	8	774	542	27846	682	340	1069	169	34	258	137	149	269.15	267	432.23	457	447	431	18	78	17	2	0
3910	13732	9980	959	268	134	28026	1276	6	585	1	2	69	3	69	270.12	84	423.05	275	271	287	16	47	0	0	2
4702	13184	9528	789	430	228	28078	1190	8	648	2	6	72	5	72	276.29	109	432.02	324	325	317	22	48	0	0	2
4904	12924	9348	691	596	344	28134	1129	7	640	1	18	64	4	95	278.77	95	434.61	378	323	318	10	45	0	0	0
5412	12672	9124	633	576	316	28116	1124	11	727	1	16	69	8	96	281.73	124	437.98	359	369	325	16	48	0	0	2
12228	7820	5108	2	1730	1260	28382	594	139	679	30	216	167	75	230	300.26	137	454.01	481	265	409	80	87	11	18	7
13670	7838	4612	3	998	776	28002	661	274	990	123	100	232	119	179	275.83	212	438.95	456	416	432	33	95	17	8	1
13294	7668	4738	2	1314	966	28150	628	228	862	85	156	219	91	196	285.02	191	448.65	479	343	398	51	104	26	14	6
14458	7636	4396	7	760	540	27842	666	324	1103	163	46	270	125	134	269.58	255	431.94	480	490	423	23	94	21	6	0
14356	7506	4286	3	964	732	27922	624	328	958	157	74	269	117	178	273.3	227	438.14	467	382	426	25	89	29	4	3
4498	13224	9558	823	514	308	28114	1213	7	582	1	12	63	4	87	276.36	91	430.44	320	283	294	17	40	0	0	1
4466	13334	9650	810	390	216	28068	1224	10	637	2	12	69	6	73	273.03	89	427.87	306	327	300	23	47	0	0	0
4834	13134	9474	750	400	214	28068	1188	7	714	0	12	66	4	67	276.65	114	431.25	338	363	314	17	49	0	0	3
12550	8002	5012	6	1426	1080	28250	592	174	825	46	158	192	94	238	287.43	183	447.54	484	292	423	46	105	8	16	4

SUPPLEMENTARY INFORMATION:Monte Carlo Atomistic Simulation and Machine Learning Analysis of Na-K Eutectic Alloy in Condensed Phases, D. Reitz and E. Blaisten-Barojas, George Mason University, Fairfax, VA 22030

5876	12472	8868	579	542	322	28098	1078	25	774	7	18	78	14	101	282.82	132	441.59	376	393	346	13	52	0	0	1
12138	7900	5170	0	1710	1240	28390	561	131	717	41	210	193	51	250	302.06	151	455.59	490	254	383	70	106	10	20	16
14362	7646	4312	4	840	668	27902	636	307	1042	166	72	267	101	133	269.29	274	432.29	528	429	411	19	90	23	2	2
13794	7820	4602	7	988	706	27972	624	272	1002	112	58	219	130	177	275.84	229	440.97	471	387	474	25	95	18	4	2
4510	13288	9614	813	420	234	28076	1209	8	645	1	10	62	3	74	272.95	109	427.73	328	313	306	12	39	0	0	3
14336	7640	4358	7	838	644	27888	626	345	1013	151	70	250	148	160	271.02	266	434.78	491	407	448	17	84	24	2	0
4996	12894	9324	744	540	322	28110	1156	12	652	4	32	75	7	80	278.82	115	435.26	356	310	301	20	57	0	2	3
4920	12992	9360	698	498	310	28116	1154	5	693	1	36	62	1	85	278.63	118	435.1	353	358	317	17	39	0	0	3
3852	13748	9988	985	286	160	28044	1294	5	568	1	8	56	3	60	270.21	94	422.72	279	257	287	17	43	0	2	2
4160	13448	9780	905	420	250	28082	1273	7	588	1	22	61	4	68	272.48	93	426	291	290	281	18	39	0	2	4
5194	12762	9174	661	616	376	28148	1133	5	653	2	24	64	3	93	281.05	94	436.12	354	355	325	21	46	0	2	4
7488	11286	7942	358	850	534	28160	907	42	813	14	56	102	21	122	288.54	122	445.42	430	393	387	34	67	2	4	3
4706	13092	9476	788	518	300	28112	1202	6	632	0	20	63	4	79	277.26	100	432.82	330	314	301	20	46	1	0	1
4978	13014	9336	718	478	284	28102	1151	13	667	0	12	67	6	76	278.1	115	434.09	355	331	318	21	41	0	0	3
13658	7806	4682	6	1044	724	27984	594	256	983	119	60	237	100	196	276.03	226	443.34	451	358	470	38	97	16	10	2
6708	11408	8212	360	1168	730	28302	931	26	709	4	74	73	17	149	288.21	107	445.18	409	372	372	44	41	0	2	7
4022	13508	9856	928	442	244	28084	1278	8	553	0	12	50	5	79	274.03	81	427.44	283	273	285	16	37	0	0	1
11102	7988	5552	0	2090	1546	28632	534	80	587	6	312	153	48	285	320.35	105	463.41	464	192	392	111	84	10	38	19
3810	13778	10010	1011	282	152	28034	1332	7	570	1	2	66	5	55	270.39	95	421.95	267	274	258	13	42	0	0	5
4716	13146	9484	753	468	272	28100	1186	8	662	0	12	50	5	89	275.79	94	429.42	334	350	327	6	34	1	0	3
4442	13374	9650	852	378	216	28066	1210	11	615	1	6	57	9	71	273.19	88	426.42	323	295	320	13	42	0	0	2
14378	7684	4386	7	800	578	27866	673	322	1075	155	38	251	129	148	270.17	263	432.14	466	462	430	21	85	15	0	1
5780	12284	8848	539	764	456	28170	1043	16	685	3	34	70	11	97	284.72	102	440.88	397	359	351	27	45	0	4	4
11096	7856	5550	3	2200	1592	28664	555	83	532	19	340	165	41	293	324.32	96	465.48	442	193	369	106	68	5	28	26
12700	7876	4920	1	1480	1102	28256	591	193	786	49	170	183	112	217	286.44	173	448.53	504	289	435	48	97	12	6	5
14586	7658	4308	4	680	536	27832	629	351	1103	175	62	277	131	147	269.68	291	432.01	500	445	424	13	95	20	2	1
5792	12274	8802	576	774	492	28180	1092	17	688	3	40	81	12	109	282.79	108	438.15	363	363	322	22	52	0	6	6
11220	7800	5492	3	2110	1606	28656	521	88	532	14	374	163	44	290	320.94	105	461.39	465	180	373	115	79	5	52	21
14250	7586	4406	4	928	674	27922	629	314	1018	151	78	249	125	152	273.18	231	438.79	487	428	442	30	83	15	0	2
3892	13622	9918	960	406	230	28074	1285	6	546	1	6	47	3	66	271.86	82	425.28	279	266	292	19	27	0	0	4
4104	13516	9830	935	386	218	28070	1277	4	594	1	16	65	2	81	271.99	83	425.19	280	280	276	15	47	0	0	1
3994	13572	9884	956	394	216	28068	1301	7	577	2	8	64	3	76	270.21	80	423.53	283	286	261	10	42	0	0	1
4570	13148	9520	760	544	326	28130	1203	3	614	0	22	66	2	92	278.8	80	432.83	325	336	293	17	40	0	0	1
10554	7782	5638	1	2498	1898	28846	477	70	431	9	418	171	35	317	327.3	73	471.54	466	142	347	132	74	5	44	35
6474	11838	8460	478	844	520	28190	1018	22	757	4	52	73	14	130	287.86	121	444.29	387	379	359	20	51	1	2	4
4730	13012	9462	745	568	322	28120	1195	5	645	0	26	60	3	83	276.62	87	431.62	331	344	301	19	40	0	0	3

SUPPLEMENTARY INFORMATION:Monte Carlo Atomistic Simulation and Machine Learning Analysis of Na-K Eutectic Alloy in Condensed Phases, D. Reitz and E. Blaisten-Barojas, George Mason University, Fairfax, VA 22030

14224	7594	4378	2	952	708	27928	659	312	972	158	64	246	115	149	270.89	240	433.22	503	417	420	18	74	20	8	0
4354	13312	9662	851	480	278	28102	1235	4	586	0	16	57	1	81	276.89	88	432.11	302	295	296	18	41	0	0	4
11094	7896	5516	0	2136	1636	28698	533	81	563	13	368	175	43	278	324.02	112	466.54	468	183	337	121	87	3	46	32
6398	11792	8482	449	902	564	28212	1031	19	743	3	70	69	11	117	284.89	103	443.6	404	402	337	28	47	0	4	1
13830	7842	4586	7	890	702	27954	632	267	1054	130	94	244	99	145	275.7	261	441.33	510	400	418	35	97	21	10	1
12510	7758	4962	3	1616	1244	28350	551	159	712	48	232	205	86	256	299.98	161	453.35	478	223	409	73	107	9	26	7
4894	12894	9352	686	620	358	28144	1143	6	661	0	26	57	4	81	278.41	95	435.49	353	348	336	27	36	0	0	1
14394	7698	4376	5	778	572	27858	669	322	1081	168	36	256	118	140	267.99	279	428.73	482	448	429	17	85	14	4	1
4250	13320	9710	854	522	296	28116	1209	5	558	0	18	61	5	75	277.36	93	432.72	338	265	295	15	44	0	0	3
5302	12714	9120	627	612	370	28136	1155	7	708	0	16	59	4	93	279.18	111	435.71	345	395	320	18	34	0	0	3
3966	13624	9906	951	358	200	28064	1280	5	553	1	10	78	3	72	270.42	84	422.59	288	264	261	14	53	0	0	4
5406	12694	9110	646	564	326	28118	1143	11	747	1	16	65	5	86	279.18	123	436.5	345	388	323	27	35	0	0	1
13528	7678	4526	4	1256	986	28110	594	249	849	89	126	214	114	192	281.79	204	446.48	493	311	434	49	100	13	10	3
11596	7946	5316	1	1846	1466	28544	532	110	634	28	342	179	56	269	312.29	125	460.62	499	200	379	86	97	5	32	20
3964	13652	9918	963	324	180	28046	1300	10	594	1	8	60	4	73	269.89	97	423.15	271	292	273	14	35	0	0	1
4596	13226	9564	807	436	244	28076	1202	9	635	0	10	68	7	76	276.66	103	431.39	327	314	302	16	46	0	0	2
3794	13708	10018	992	346	176	28048	1313	8	551	2	6	55	4	59	271.01	83	423.41	278	264	271	14	38	0	0	3
3906	13752	9964	986	256	146	28032	1324	7	575	0	8	54	4	57	270.83	84	424.38	267	288	271	15	37	0	0	3
4310	13416	9684	849	406	254	28086	1232	5	603	0	14	59	4	68	273.9	79	428	329	303	294	11	37	0	2	3
4178	13514	9806	907	348	194	28052	1252	6	601	1	12	67	3	74	272.12	101	426.51	285	279	296	20	47	0	0	1
11808	7828	5190	2	1874	1464	28504	525	130	620	35	314	171	69	254	306.8	120	457.51	482	211	411	98	89	8	26	19
4808	12920	9386	699	642	370	28150	1148	5	644	0	24	55	4	96	277.66	87	434.32	352	343	329	14	37	0	0	3
6528	11762	8378	392	900	592	28236	951	26	750	4	68	72	16	119	285.37	128	441.96	448	372	368	28	48	1	8	3
5768	12420	8864	572	660	406	28144	1105	22	750	2	24	65	13	104	281.87	121	438.55	374	407	328	13	40	1	2	2
14150	7696	4418	7	876	692	27922	647	316	1028	159	84	257	120	154	269.67	254	432.18	505	426	412	16	88	19	6	2
4576	13322	9584	786	350	218	28074	1206	6	657	1	24	56	3	68	276.42	99	431.64	333	339	312	15	39	0	0	2
4234	13438	9782	896	392	206	28064	1266	5	601	1	12	58	2	85	273.65	78	428.37	274	301	295	14	43	0	0	2
5104	13026	9320	692	386	226	28080	1164	5	758	0	18	61	4	72	277.33	125	433.06	348	390	332	14	43	0	0	4
4462	13150	9554	781	576	362	28144	1196	1	618	0	40	58	0	94	277.9	91	432.62	320	307	303	19	43	0	0	4
5220	12768	9204	658	580	328	28116	1111	9	714	0	16	61	6	98	278.14	111	434.65	351	349	348	21	42	1	0	1
7156	11300	8046	393	990	638	28214	894	63	697	15	80	98	36	143	289.28	106	442.26	448	328	373	29	59	0	4	1
11976	7820	5026	1	1822	1510	28508	556	137	676	51	326	183	65	254	304.82	138	457.14	495	243	387	87	93	6	28	16
13124	7962	4844	5	1170	880	28112	630	202	953	51	116	174	105	186	283.52	205	444.4	476	358	469	45	102	15	14	1
5086	12898	9274	646	532	312	28120	1110	4	707	0	16	66	3	99	277.37	106	435.29	363	364	345	11	47	0	2	3
5276	12704	9168	678	604	348	28126	1139	20	645	3	26	66	11	87	281.63	89	436.93	361	351	318	19	44	0	0	2
14506	7472	4256	7	918	682	27904	609	344	992	162	64	259	144	130	271.16	287	435.5	538	374	436	23	84	21	6	1

4714	13174	9506	776	444	242	28084	1185	5	658	0	4	69	3	67	276.75	118	431.27	359	325	302	13	52	0	0	1
11852	7918	5226	2	1774	1378	28458	554	128	692	24	272	185	69	258	302.53	131	456.65	467	235	399	93	111	7	32	13
14410	7782	4350	4	676	568	27854	641	310	1129	155	66	252	117	143	267.39	303	429.57	496	450	444	13	86	16	2	2
4192	13512	9768	898	364	220	28068	1263	8	585	1	12	58	3	72	275.18	96	429.18	299	299	289	11	35	0	0	3
4256	13474	9754	870	358	206	28060	1263	12	608	2	12	63	5	60	273.66	89	427.11	311	322	279	13	42	0	0	4
11470	7846	5352	0	2044	1526	28576	526	103	607	17	308	184	57	268	314.3	129	458.16	483	188	382	99	98	11	28	21
7550	11174	7894	353	944	576	28198	887	58	754	10	58	90	39	125	287.05	139	446.74	454	366	399	29	50	0	2	5
14364	7628	4376	8	834	616	27882	633	329	1051	156	62	255	143	148	270.43	267	432.46	492	425	449	18	90	14	2	2
14174	7460	4350	5	1078	812	27984	615	301	942	125	98	254	124	194	277.27	195	445.46	436	372	437	45	106	25	12	4
7574	11220	7902	341	874	540	28170	903	50	806	16	58	103	26	141	289.2	134	446.74	408	394	396	29	60	0	0	8
4544	13324	9594	820	366	222	28066	1222	8	656	1	16	69	4	76	273.64	115	427.76	310	326	303	14	41	0	0	3
13956	7764	4494	3	936	722	27956	636	283	1022	136	78	245	107	165	274.63	258	438.22	501	410	417	21	95	11	6	2
4446	13344	9654	809	408	218	28070	1231	6	638	2	0	66	3	70	274.58	89	429.47	307	338	299	19	39	0	0	3
12496	7970	4996	2	1482	1130	28262	576	180	800	49	172	173	105	233	287.04	169	450.02	457	293	471	62	96	8	12	8
4832	13018	9408	712	528	312	28124	1175	7	674	0	22	61	5	73	279.26	103	435.05	358	353	308	15	39	0	4	4
14450	7590	4310	3	846	632	27886	631	318	1048	166	54	263	118	158	269.14	272	432.18	491	416	436	15	83	19	4	2
12778	7832	4918	5	1418	1068	28208	578	183	839	70	182	202	83	232	285.77	160	447.23	469	301	442	53	105	12	12	5
14422	7576	4346	5	848	628	27892	634	319	1039	167	66	261	121	155	272.26	265	436.65	493	424	434	19	82	16	6	0
13444	7766	4582	1	1192	968	28104	608	222	903	91	140	217	91	196	278.35	198	444.13	497	352	423	37	98	17	8	4
14494	7712	4362	3	726	518	27834	664	331	1104	161	20	261	127	142	269.31	264	432.27	481	470	428	14	81	15	2	1
10958	7914	5520	3	2224	1696	28714	529	65	511	12	348	155	35	287	316.7	99	463.39	499	155	351	113	79	4	50	20
3760	13772	10038	1017	312	160	28044	1332	5	546	0	2	62	3	66	271.45	73	423.06	265	264	258	11	49	0	0	2
4092	13606	9878	954	298	154	28034	1290	11	604	1	6	63	4	64	270.79	107	423.12	292	283	270	10	42	0	0	2
4848	12942	9396	754	578	332	28124	1200	14	661	1	26	47	8	85	277.66	101	433.12	317	348	318	20	35	0	2	3
14202	7706	4434	4	860	636	27892	637	308	1049	158	46	259	110	155	268.85	282	432.04	501	431	426	14	87	22	6	1
7290	11174	7944	335	1066	698	28270	913	28	758	7	90	96	18	158	288.36	118	445.41	442	378	355	22	54	0	6	5
13532	7742	4562	3	1148	942	28090	618	261	884	112	158	209	111	199	277.15	221	443.91	482	347	437	36	73	13	6	2
4710	13108	9458	754	514	308	28116	1192	8	649	0	18	53	7	85	277.38	93	433.59	329	348	323	13	37	0	0	2
12316	7798	5066	3	1686	1246	28354	571	157	737	47	228	189	73	240	300.61	152	452.31	475	258	401	76	98	11	12	14
14472	7586	4322	5	830	608	27872	653	328	1065	166	52	263	122	153	269.66	277	431.35	474	434	432	16	88	19	2	3
4802	12990	9414	745	564	326	28118	1197	7	647	1	22	61	6	80	278.32	96	433.82	329	337	302	21	43	0	0	3
4222	13494	9794	919	346	186	28052	1271	9	610	2	8	72	5	79	272.74	101	426.62	291	293	272	6	45	0	2	4
13970	7792	4502	6	908	696	27942	644	301	999	139	68	243	122	167	274.1	238	439.19	468	399	443	28	87	18	6	1
13226	7764	4686	0	1328	1006	28146	581	222	830	76	122	211	114	194	280.64	201	447.67	505	286	445	45	103	14	12	6
12254	7874	5074	1	1640	1260	28358	603	137	751	46	226	180	62	222	293.81	144	451.65	492	276	405	64	95	14	30	13
4248	13492	9770	907	342	194	28060	1270	8	626	3	14	59	4	65	273.66	97	427.47	296	302	286	16	38	0	0	2

SUPPLEMENTARY INFORMATION:Monte Carlo Atomistic Simulation and Machine Learning Analysis of Na-K Eutectic Alloy in Condensed Phases, D. Reitz and E. Blaisten-Barojas, George Mason University, Fairfax, VA 22030

4832	13142	9464	717	408	224	28082	1168	4	702	0	12	62	2	71	278.49	106	434.63	351	375	324	17	41	0	0	1
11858	7956	5280	4	1720	1306	28414	555	121	713	31	268	187	55	261	302.12	158	454.19	496	243	377	74	110	8	24	10
14272	7696	4372	4	840	650	27890	653	321	1063	166	54	265	113	156	268.76	260	429.78	489	448	407	17	90	16	6	2
13996	7698	4494	6	962	716	27950	629	293	999	138	82	248	113	177	273.5	223	438.19	473	408	439	22	89	17	2	4
4374	13326	9692	834	438	236	28080	1218	11	621	1	14	57	7	72	274.98	96	427.7	322	306	308	14	39	0	0	3
13186	7864	4728	4	1254	968	28138	622	212	894	71	122	194	104	180	282.38	196	445.99	490	335	444	54	97	17	16	1
11232	8034	5556	0	1970	1442	28568	554	93	613	21	304	169	44	260	311.59	125	460.57	497	205	364	98	92	4	28	16
5306	12642	9086	593	694	420	28170	1052	6	654	0	22	61	3	104	279.35	96	436.54	402	332	352	18	35	0	0	2
5764	12286	8822	538	778	494	28196	1112	11	711	1	44	69	9	111	285.07	81	439.29	361	416	318	26	41	0	8	0
5618	12530	8972	589	610	374	28140	1086	9	738	1	34	73	6	100	281.36	107	436.32	376	381	337	22	54	0	2	0
4520	13256	9580	823	430	270	28086	1228	7	621	2	30	60	5	61	275.48	76	430.32	317	334	304	22	32	0	0	3
4810	13086	9430	762	484	278	28098	1180	7	665	0	10	67	5	83	278.12	108	433.29	335	328	308	19	47	0	0	1
11132	7784	5532	2	2168	1624	28672	497	76	552	13	398	172	39	277	321.31	103	466.61	477	168	364	126	93	5	32	27
4342	13352	9700	847	434	242	28086	1246	9	604	1	14	55	5	78	275.32	74	429.14	313	319	280	14	40	0	2	1
4878	12964	9414	752	544	290	28106	1155	7	651	0	16	71	6	92	280.33	106	437.35	335	308	319	22	55	0	0	0
13474	7638	4610	1	1282	968	28122	605	229	841	80	136	194	112	199	283.63	199	445.55	481	309	456	45	94	17	12	2
11528	7856	5276	0	2030	1556	28592	542	100	572	22	320	160	48	272	317.72	131	461.62	504	195	367	80	76	5	26	26
14374	7556	4362	5	858	654	27904	634	327	1022	164	98	269	127	165	272.77	239	436.29	482	421	417	21	94	18	2	3
14152	7684	4484	5	868	632	27894	623	320	1043	152	74	252	124	145	273.44	244	439.22	484	426	447	34	86	17	0	2
12586	7928	4936	3	1476	1138	28254	593	171	806	60	174	201	90	222	286.32	167	448.6	485	296	419	55	106	8	16	8
4290	13498	9748	890	326	184	28052	1270	7	618	0	6	62	5	70	272.25	89	425	295	314	286	13	44	0	0	0
4896	12972	9384	700	536	308	28120	1134	10	684	1	20	62	7	87	276.1	125	432.82	366	338	329	13	36	0	4	3
4136	13482	9808	896	404	228	28074	1267	1	594	0	16	60	1	62	271.07	89	423.9	293	294	292	21	49	0	0	3
14474	7612	4342	2	794	590	27878	647	314	1060	162	64	264	110	153	268.98	279	431.39	485	428	424	20	89	22	2	0
14400	7656	4384	7	794	574	27854	631	335	1079	166	42	267	129	150	268.26	286	431.54	497	428	433	14	88	21	4	1
14106	7766	4520	9	834	604	27890	647	299	1084	150	56	252	117	159	270.99	267	433.76	473	436	447	17	87	14	4	2
3856	13652	9970	979	386	198	28064	1317	5	543	0	2	62	4	70	273.11	74	426.84	252	270	270	22	46	0	0	3
6518	11768	8374	403	922	596	28240	957	16	753	3	56	76	9	133	287.31	128	441.89	424	392	370	25	41	1	4	5
11308	7996	5426	0	1998	1506	28560	539	91	655	22	298	154	52	287	301.58	130	455.26	508	216	406	70	86	5	28	12
5510	12642	9076	595	544	310	28104	1081	12	751	2	18	77	5	112	279.62	107	437.59	358	374	351	15	52	1	4	0
4998	12938	9312	678	532	318	28120	1132	4	683	0	22	61	3	91	279.05	101	434.83	354	342	338	17	46	0	0	2
3930	13768	9978	999	224	114	28016	1311	11	593	1	2	59	5	74	271.67	82	423.93	244	272	290	14	43	0	0	2
11574	7906	5392	6	1878	1408	28490	521	121	645	24	296	164	62	278	305.36	129	457.74	497	211	410	72	90	5	32	17
5884	12318	8788	521	712	438	28170	1054	11	755	2	28	68	5	98	281.83	131	439.6	417	385	344	11	44	1	2	4
13408	7574	4586	0	1360	1052	28156	587	236	832	82	154	201	114	206	281.75	174	446.4	467	316	460	52	92	17	20	8
4426	13362	9666	859	386	216	28066	1223	11	616	1	10	55	7	77	272.31	102	426.55	305	305	320	14	35	0	0	2

SUPPLEMENTARY INFORMATION:Monte Carlo Atomistic Simulation and Machine Learning Analysis of Na-K Eutectic Alloy in Condensed Phases, D. Reitz and E. Blaisten-Barojas, George Mason University, Fairfax, VA 22030

11350	7982	5394	2	1986	1528	28586	552	82	648	16	314	159	39	286	310.73	119	460.5	462	212	386	95	97	6	22	21
13056	7776	4796	1	1368	1026	28194	591	204	797	59	158	179	106	215	287.64	177	450.14	491	282	448	49	94	16	14	1
14102	7676	4454	7	910	694	27924	628	337	980	165	82	255	133	170	270.33	251	433.84	488	400	432	19	76	19	6	1
11186	7912	5478	1	2076	1590	28642	522	91	556	10	368	159	56	291	317.19	111	463.3	449	171	394	115	92	8	28	24
3952	13636	9904	962	364	204	28064	1289	5	549	1	4	67	3	72	271.47	81	425.71	282	262	269	15	46	0	0	2
11144	7864	5474	0	2172	1618	28640	523	80	541	15	332	152	33	270	315.95	102	464.41	502	192	374	99	71	9	34	28
12296	7758	5016	1	1780	1310	28394	568	148	648	38	224	179	78	229	300.71	138	451.72	518	230	395	71	88	7	10	10
4326	13442	9720	872	346	208	28064	1254	5	613	1	22	68	3	73	274.33	91	428.71	297	319	286	17	49	0	0	1
12360	7854	5004	1	1614	1266	28358	589	123	762	31	230	186	60	230	294.72	158	453.54	485	253	397	68	114	16	28	16
5074	12772	9252	648	644	376	28148	1107	8	673	1	28	56	4	92	279.2	108	436.84	370	341	347	19	39	0	2	1
5624	12494	8948	531	640	404	28156	1019	5	725	1	46	69	2	101	283.44	110	440.68	416	385	361	21	45	0	0	2
7136	11396	8120	387	944	554	28196	917	36	788	13	44	100	15	131	287.81	130	444.82	449	377	365	24	54	0	2	3
3962	13564	9856	926	426	264	28092	1280	8	558	1	20	53	3	68	271.13	97	424.33	306	269	272	14	31	0	0	1
5106	12888	9262	656	518	316	28120	1113	13	698	0	30	66	9	90	278.04	106	434.49	369	359	340	17	43	2	0	1
12384	7978	5118	4	1528	1100	28300	568	141	801	37	184	181	71	243	297.24	173	451.23	498	295	436	46	104	14	8	9
4228	13428	9780	905	402	216	28070	1273	6	600	1	14	46	3	67	272.53	80	426.96	288	307	300	15	32	0	2	4
10982	7882	5538	0	2198	1664	28672	475	90	520	15	376	189	47	309	317.29	99	465.91	468	146	374	111	96	5	28	31
4370	13362	9700	829	418	220	28076	1216	9	624	0	6	64	6	71	274.79	99	429.25	328	312	296	14	41	0	0	3
5284	12628	9082	620	708	442	28176	1098	7	653	1	30	72	6	111	281.41	112	437.51	367	338	321	23	40	0	2	1
6646	11796	8380	439	800	502	28178	980	27	757	11	50	99	16	119	284.29	114	440.6	421	390	350	22	57	0	4	4
7256	11238	7970	346	1042	670	28256	964	25	766	9	78	92	11	137	289.34	123	445.4	427	412	329	31	57	1	0	5
13468	7886	4714	6	1064	794	28030	627	258	959	130	98	236	97	173	278.14	231	443.33	517	379	400	28	89	15	6	2
14344	7678	4370	7	788	622	27880	624	317	1082	153	72	264	122	156	270.98	280	435.68	488	428	433	22	103	16	6	2
11382	7724	5460	1	2138	1546	28634	526	89	535	15	352	161	52	296	321.54	79	467.41	460	168	371	107	91	1	30	22
5092	12780	9214	697	654	390	28148	1131	9	634	4	18	70	3	102	278.88	98	435.6	357	309	317	13	45	0	0	5
12750	7822	4934	2	1452	1080	28230	612	196	803	56	170	176	100	237	285.39	169	449.67	446	299	444	53	88	12	22	4
13688	7704	4490	1	1150	906	28052	605	282	927	122	106	231	125	181	277.31	203	443.04	472	355	452	47	91	18	8	4
13680	7488	4498	4	1334	970	28092	576	262	820	122	114	235	98	210	276.98	188	444.76	488	294	436	37	88	21	8	5
13026	8000	4890	3	1190	876	28100	617	217	924	65	110	198	105	192	283.46	212	444.58	483	332	442	39	106	18	8	4
10688	7624	5640	1	2498	1874	28840	525	74	430	22	462	169	27	294	331.14	72	473.5	464	138	322	133	78	1	48	40
4112	13452	9790	880	456	268	28096	1242	2	577	0	16	48	0	79	272.84	101	427.68	307	285	304	14	30	0	0	1
13116	7870	4854	7	1216	904	28110	571	212	929	70	130	210	101	202	280.06	212	444.92	498	331	455	40	108	13	20	5
4448	13358	9650	803	386	222	28078	1213	4	626	0	14	59	3	90	277.38	79	433.62	304	338	312	15	34	0	0	0
4622	13192	9546	791	456	258	28090	1190	7	649	0	16	58	4	70	276.93	103	432.16	336	332	312	23	39	0	0	1
14442	7578	4354	5	832	602	27870	608	342	1035	174	62	269	120	157	268.98	269	432.5	496	401	443	18	84	23	0	2
5464	12600	9070	611	620	358	28140	1086	12	699	2	26	67	10	102	279.43	116	436.11	368	356	347	21	47	0	2	2

SUPPLEMENTARY INFORMATION:Monte Carlo Atomistic Simulation and Machine Learning Analysis of Na-K Eutectic Alloy in Condensed Phases, D. Reitz and E. Blaisten-Barojas, George Mason University, Fairfax, VA 22030

5130	12858	9262	633	544	310	28124	1121	5	706	0	20	66	3	99	278.89	107	435.64	359	375	334	16	45	0	0	0
13876	7706	4486	7	1036	792	27986	607	293	949	123	86	220	124	178	274.51	241	442.26	475	355	463	36	75	19	0	2
14148	7738	4440	3	834	660	27904	646	312	1049	159	78	267	115	144	270.7	280	432.81	508	428	404	15	97	16	6	7
13018	7874	4760	2	1330	1042	28180	589	217	862	69	136	195	116	201	286.01	197	447.94	496	310	452	43	90	11	18	6
3858	13712	9952	973	326	198	28060	1298	6	555	1	12	58	3	72	270.84	71	424.61	268	274	282	14	41	0	2	4
5050	12806	9264	689	624	368	28140	1135	9	634	2	26	63	4	98	279.78	101	435.11	355	326	323	15	41	0	2	4
12312	7766	5108	0	1682	1238	28376	574	148	689	43	250	177	68	244	297.33	154	453.97	484	243	412	62	81	14	20	16
14136	7772	4408	5	850	688	27934	641	293	1017	146	74	240	110	157	271.38	242	435.96	497	418	435	19	82	22	6	2
4314	13440	9728	894	366	204	28060	1252	8	622	1	8	69	5	80	273.81	96	429.79	292	299	288	13	48	0	0	2
14334	7614	4394	8	862	620	27882	625	333	1026	166	54	264	128	161	269.33	252	431.89	495	417	433	13	90	21	4	1
5036	12928	9310	689	518	302	28116	1147	8	689	1	22	64	4	78	280.43	106	435.89	356	357	323	24	43	0	0	1
4384	13364	9704	847	404	208	28070	1206	3	630	0	6	58	2	84	275.91	97	430.92	311	291	320	14	45	0	0	1
13734	7652	4610	8	1116	792	28008	622	268	943	125	98	234	100	181	278.27	219	444.82	477	363	433	35	94	19	6	3
4212	13480	9752	904	386	234	28080	1288	4	603	0	16	62	3	65	271.49	92	425.45	290	308	273	17	42	0	0	3
11406	7854	5354	0	2076	1578	28626	516	105	534	18	304	185	52	285	315.49	106	463.53	489	182	360	105	88	5	52	20
5672	12508	8910	542	634	398	28148	1067	15	712	3	26	73	10	99	281.66	104	437.96	391	379	349	16	47	0	0	3
6570	11650	8372	436	956	608	28242	972	22	704	3	76	80	14	137	289.39	106	444.63	399	347	362	38	48	1	10	2
11476	7888	5328	1	1986	1536	28574	527	96	561	24	328	185	42	283	310.88	112	458.49	467	179	367	104	92	7	30	22
12066	7868	5200	4	1736	1266	28392	534	142	705	29	240	206	71	254	306.03	150	458.35	504	221	378	73	124	7	16	14
5426	12640	9060	602	592	376	28134	1073	10	686	1	38	79	4	123	281.16	101	439.38	369	354	336	11	50	0	2	1
14106	7652	4488	2	948	668	27930	637	318	1002	154	66	264	125	177	272.91	237	437.37	478	401	419	20	97	20	2	0
5298	12612	9082	599	680	446	28178	1069	9	654	1	60	71	6	90	279.74	102	438.17	411	350	330	21	45	0	0	3
5328	12666	9114	579	624	384	28158	1079	6	730	1	42	60	2	89	280.28	127	438.47	383	366	350	26	40	0	0	4
14254	7764	4476	10	748	554	27856	661	310	1079	157	60	253	116	137	271.18	269	434.81	480	450	435	22	89	18	0	2
4608	13174	9546	761	484	272	28100	1171	9	633	0	16	58	8	88	274.63	94	431.36	336	317	324	14	40	0	0	2
3790	13724	10010	1010	336	182	28052	1317	8	537	2	10	59	4	71	270.65	76	422.84	265	251	270	11	39	0	0	3
11370	7846	5460	0	2066	1500	28586	541	102	600	16	312	155	54	259	315.98	97	460.78	460	204	402	110	91	9	28	28
4364	13374	9690	864	394	230	28072	1225	9	635	1	20	67	4	79	274.45	110	427.85	315	285	294	11	45	0	0	3
11674	8006	5278	3	1768	1406	28454	554	120	693	33	286	181	63	239	297.83	136	457.38	464	240	402	100	92	5	36	21
10944	7892	5558	0	2152	1702	28726	503	80	534	9	422	167	52	291	325.96	87	468.46	479	173	376	103	97	3	54	38
10996	7834	5496	1	2226	1732	28728	469	83	526	12	384	164	44	290	323.79	106	465.6	501	138	373	111	78	2	50	34
4234	13478	9780	895	368	194	28058	1258	7	615	1	4	64	4	66	273.66	95	427.13	299	291	290	19	44	0	0	0
3904	13628	9920	949	384	224	28076	1279	6	530	1	16	61	5	67	270.52	70	423.8	297	261	278	13	42	0	0	3
3848	13624	9918	957	430	258	28092	1295	0	534	0	14	70	0	73	273.15	82	426.29	284	258	258	13	49	0	0	6
11236	7822	5416	2	2142	1634	28646	524	75	555	11	360	160	38	277	315.01	100	460.98	485	190	375	104	75	7	36	28
4948	13030	9348	685	460	288	28098	1122	4	698	0	24	63	1	92	278.01	104	433.62	351	351	342	18	43	0	0	2

SUPPLEMENTARY INFORMATION:Monte Carlo Atomistic Simulation and Machine Learning Analysis of Na-K Eutectic Alloy in Condensed Phases, D. Reitz and E. Blaisten-Barojas, George Mason University, Fairfax, VA 22030

4140	13604	9834	907	296	164	28040	1269	7	621	1	2	65	4	65	272.48	95	425.18	288	306	288	18	46	0	0	1
4718	13006	9434	741	586	356	28134	1175	6	643	0	34	64	5	67	279.4	90	433.75	352	333	305	25	37	0	0	6
11342	7908	5470	1	2014	1486	28570	538	108	624	23	328	167	57	287	310.16	132	458.27	499	212	380	83	88	4	18	11
13888	7724	4452	6	1034	810	28000	626	252	969	127	88	243	98	176	275.68	255	441.56	500	370	417	24	98	9	4	3
4516	13116	9558	787	590	330	28126	1211	4	577	0	16	60	3	82	278.88	73	435.39	323	318	303	15	37	0	0	5
13690	7922	4716	8	908	642	27946	639	262	1052	112	64	233	114	161	275.55	243	438.62	473	411	442	29	107	13	4	6
14404	7634	4372	4	796	596	27870	621	329	1073	173	64	273	121	145	269.38	279	432.47	513	438	424	17	92	19	4	1
5072	12792	9228	625	646	392	28158	1079	8	633	0	26	63	6	92	282.7	101	440.05	395	326	345	19	41	0	2	2
4370	13330	9652	812	446	274	28096	1213	3	597	0	22	52	0	80	275.52	84	430.06	315	321	311	17	26	0	2	2
4336	13422	9710	885	368	214	28064	1255	6	631	1	12	60	2	68	273.22	114	428.55	306	293	283	17	42	0	2	1
4482	13170	9588	776	552	312	28124	1181	2	594	0	18	64	2	90	277.61	83	432.71	341	316	308	11	44	0	2	3
13218	7776	4790	4	1250	930	28126	630	214	902	87	150	213	95	179	281.85	208	445.99	501	357	416	44	105	13	10	4
13824	7650	4532	4	1122	808	28030	637	286	889	116	92	221	123	186	278.81	199	444.84	467	353	448	33	85	24	2	1
4262	13422	9754	896	398	222	28076	1267	6	600	0	16	57	5	81	274.58	82	428.6	287	301	287	14	41	0	2	1
4118	13458	9780	884	452	270	28096	1251	3	589	0	18	69	1	77	274.34	89	428.59	301	283	275	19	50	0	0	3
10794	7852	5656	0	2278	1710	28742	497	76	510	12	398	185	32	286	325.01	91	465.96	482	168	338	138	85	9	54	23
12300	7832	5016	2	1698	1286	28362	595	145	725	34	200	188	76	238	299.16	136	451.92	451	280	403	75	100	15	28	19
11196	7950	5478	0	2046	1568	28624	546	72	574	9	348	145	38	278	319.72	95	463.7	492	212	385	88	83	3	38	25
4440	13276	9624	814	456	278	28104	1205	7	593	2	24	68	3	77	275.85	88	430.76	336	308	289	19	41	0	6	0
4390	13400	9676	838	362	220	28068	1225	7	628	1	20	54	4	73	272.96	96	427.43	307	317	315	17	39	0	0	2
5622	12544	8978	607	602	358	28128	1102	15	715	4	22	65	4	97	280.67	103	439.56	370	370	337	15	47	1	2	4
14280	7584	4372	3	896	692	27920	663	307	1045	155	86	250	113	167	272.24	232	436.64	457	449	427	25	86	19	8	2
4350	13284	9656	834	506	296	28114	1244	2	590	0	22	62	1	77	276.47	80	432.24	312	321	283	19	32	0	0	1
4506	13320	9604	819	392	238	28078	1205	7	625	0	18	61	3	85	277.25	99	431.54	310	313	318	11	43	0	0	5
4304	13416	9726	882	396	220	28072	1263	10	591	2	10	53	3	84	274.6	77	429.34	277	314	298	14	29	0	0	2
4460	13204	9620	811	514	288	28108	1196	5	605	0	18	61	2	72	275.83	98	430.65	343	295	303	19	41	0	2	1
4364	13386	9706	853	388	210	28062	1225	5	636	1	6	68	3	79	273.29	90	427.81	301	309	302	17	48	0	2	2
11810	7726	5194	0	1966	1500	28530	547	112	621	23	278	173	56	266	308.81	129	456.31	484	196	382	86	94	9	48	21
12210	7928	5090	2	1608	1262	28368	584	125	753	26	242	175	68	236	300.87	147	450.43	478	261	412	77	105	10	28	8
12274	7946	5046	2	1604	1242	28348	594	139	745	26	220	170	84	215	292.34	165	453.35	479	281	427	79	100	12	16	10
13974	7766	4498	5	918	708	27948	608	312	978	135	76	229	140	169	274.91	237	440.04	482	389	480	26	75	16	8	1
5712	12384	8916	546	710	412	28168	1052	11	717	1	32	70	7	103	284.51	120	439.05	408	380	334	20	47	0	2	1
4874	13056	9380	690	482	296	28108	1123	3	674	0	20	62	2	83	278.74	90	435.68	359	347	343	21	43	0	0	2
14410	7620	4320	4	844	636	27886	655	332	1046	168	54	259	125	160	268.01	250	429.35	480	443	424	16	83	17	2	0
5484	12620	9040	623	596	364	28134	1111	21	713	1	30	57	15	105	282.79	106	440.15	366	370	340	9	38	0	0	3
6070	12070	8660	485	842	516	28206	1037	14	723	2	48	74	7	105	285.85	111	442.44	413	383	330	26	50	0	0	4

14364	7722	4432	4	764	532	27844	671	321	1100	154	30	248	128	150	269.58	280	432.35	466	457	439	13	82	16	0	2
4070	13654	9894	942	272	142	28038	1291	7	621	1	6	60	4	70	272.59	105	424.84	279	291	283	11	46	0	0	1
5256	12792	9180	646	536	330	28128	1110	6	704	1	32	69	2	105	278.73	102	435.79	369	380	333	8	48	0	2	2
4072	13544	9844	925	374	216	28066	1280	9	586	1	14	54	3	69	272.29	93	426.09	290	284	281	14	40	1	2	3
4312	13488	9746	895	320	176	28048	1251	10	616	1	6	65	5	72	272.89	110	425.89	306	286	280	11	51	0	0	3
14294	7674	4428	11	822	596	27868	597	326	1048	172	46	277	115	165	272.35	254	438.68	467	400	462	27	95	19	8	3
13776	7872	4634	10	914	670	27936	626	285	1014	126	70	234	124	164	274.1	224	438.58	468	411	469	30	90	15	0	2
4402	13404	9682	854	358	204	28060	1214	8	607	1	10	69	5	82	273.23	96	426.14	305	299	310	13	41	0	0	3
4636	13198	9500	757	474	286	28104	1182	6	644	1	10	52	4	89	275.5	96	432.04	332	344	327	12	32	0	0	1
4236	13466	9732	885	384	242	28080	1259	6	595	2	18	54	3	62	271.85	91	425.43	316	313	288	13	33	0	2	3
13814	7780	4512	5	984	788	27986	634	295	983	128	106	241	123	188	276.05	234	441.82	457	381	436	29	97	23	2	3
11108	7790	5518	0	2202	1634	28656	496	77	560	20	370	175	32	277	323.85	105	465.32	485	168	374	120	90	6	32	25
4712	13140	9484	759	474	270	28088	1191	6	663	0	8	58	3	78	275.74	97	430	346	342	303	13	43	0	0	0
4190	13402	9732	856	486	280	28094	1233	6	572	1	4	66	3	78	273.21	84	427.8	308	277	289	18	39	0	0	3
3996	13662	9912	972	300	166	28044	1306	3	585	1	6	67	1	68	270.85	91	424.8	265	291	271	16	40	0	2	3
10642	7824	5710	1	2338	1770	28780	487	76	472	13	456	165	33	320	330.74	96	471.54	478	133	347	105	78	7	38	37
4708	13250	9498	767	370	230	28068	1176	8	684	1	12	63	4	83	275.88	112	428.97	329	340	325	16	42	1	0	0
5324	12686	9124	629	610	366	28140	1114	10	699	1	30	62	6	99	280.95	96	436.32	357	364	342	16	42	0	0	5
12686	7840	4862	3	1536	1166	28266	582	179	782	63	156	202	84	218	284.88	159	450.11	500	289	411	61	92	15	20	6
4502	13282	9602	818	426	254	28086	1208	5	637	1	18	62	2	86	275.49	93	430.34	316	317	307	12	39	0	2	3
4542	13190	9578	776	502	280	28110	1200	8	644	0	18	54	8	85	276.2	89	433.59	331	327	311	13	35	0	0	1
13752	7798	4562	5	998	778	27986	633	273	980	124	84	247	121	185	276.42	229	442.23	461	388	427	34	107	14	12	2
14154	7646	4344	6	918	776	27958	631	305	996	148	110	253	124	167	271.46	271	435.85	494	384	424	22	89	22	8	3
14474	7654	4340	3	774	566	27846	636	320	1101	169	36	272	116	155	267.89	288	430.08	482	433	433	15	92	14	2	1
4334	13374	9704	849	422	234	28078	1224	9	610	2	8	58	4	77	273.18	91	427.34	326	305	300	10	34	0	2	0
13678	7830	4696	8	988	700	27982	646	282	984	108	88	232	138	172	276.56	211	442.8	454	412	446	36	109	17	2	4
13338	7902	4718	6	1124	860	28052	639	243	938	121	100	233	89	183	275.43	223	444.08	480	376	416	38	82	16	10	1
5020	13022	9346	692	436	248	28082	1130	3	709	0	10	59	1	82	277.53	106	434.01	344	353	357	20	41	0	0	2
11652	7856	5422	2	1882	1358	28498	525	108	632	24	300	165	60	248	313.86	132	459.32	506	204	396	91	83	3	24	17
13516	7662	4572	4	1224	966	28100	624	246	853	107	146	225	107	213	279.13	188	444.82	444	324	431	43	99	18	14	6
12246	7926	5102	4	1600	1224	28350	563	145	752	33	234	184	79	246	297.26	149	451.58	471	263	419	72	107	8	18	10
6060	12134	8668	524	782	490	28176	1032	19	708	3	42	80	13	126	286.28	111	440.37	395	368	340	16	46	0	0	2
10656	7762	5546	0	2462	1914	28828	554	63	443	10	436	141	30	296	322.85	88	466.75	460	158	346	121	66	1	52	31
10506	7824	5634	1	2454	1926	28858	490	54	471	9	450	160	27	304	330.56	97	471.05	458	134	365	154	76	1	60	25
4574	13162	9558	785	512	286	28110	1228	3	653	0	18	61	2	77	277.65	91	431.83	323	347	289	16	41	0	0	2
4076	13570	9834	935	358	214	28066	1279	4	591	1	14	60	3	64	270.8	110	422.28	291	283	284	16	43	0	0	3

SUPPLEMENTARY INFORMATION:Monte Carlo Atomistic Simulation and Machine Learning Analysis of Na-K Eutectic Alloy in Condensed Phases, D. Reitz and E. Blaisten-Barojas, George Mason University, Fairfax, VA 22030

12564	7848	4936	0	1532	1192	28300	600	160	778	48	204	200	77	224	293.55	155	451.97	486	292	388	64	108	11	24	11
12882	7912	4898	0	1312	1010	28206	635	191	859	74	182	198	85	203	285.12	189	446.74	480	328	412	54	105	12	8	3
4194	13534	9796	914	332	190	28058	1275	5	616	0	12	49	2	68	272.66	93	426.41	283	300	299	19	27	0	0	0
10866	7894	5516	1	2270	1764	28744	512	77	504	13	390	177	39	296	323.61	90	464.37	464	170	344	124	77	4	38	30
6042	12144	8766	484	770	412	28154	1042	13	768	2	20	68	8	102	284.17	105	439.56	417	415	345	12	49	0	0	3
3902	13656	9932	979	358	204	28062	1299	5	564	1	10	60	3	74	269.85	91	422.42	278	263	271	10	38	0	0	3
4838	13024	9404	727	520	306	28114	1158	4	670	0	22	64	1	89	277.52	95	434.72	332	341	328	20	38	0	0	4
14318	7712	4396	3	782	604	27882	634	320	1056	154	70	250	133	170	269.34	266	432.04	463	428	457	19	83	17	0	0
11810	7884	5194	2	1836	1440	28486	555	122	664	35	292	159	57	279	308.45	135	454.31	471	244	408	72	87	10	22	15
8180	10752	7486	257	1030	690	28230	820	57	823	15	84	103	35	154	288.3	139	445.82	471	379	408	33	62	2	8	0
13856	7738	4544	2	1024	744	27982	636	285	964	128	76	226	125	167	275.98	239	438.92	500	392	437	21	77	16	0	2
5272	12608	9132	632	696	414	28162	1084	6	678	2	38	64	2	91	281.84	121	439.51	384	337	339	26	39	0	2	3
13812	7794	4580	0	1008	718	27970	643	265	997	126	56	247	106	178	276.17	236	441.88	465	403	423	32	102	16	2	1
11020	7874	5544	3	2142	1666	28698	527	76	567	11	396	162	40	299	322.77	109	466.27	471	180	366	101	89	5	52	29
5474	12598	9052	646	608	368	28134	1117	15	692	1	28	52	10	89	281.22	101	438.37	361	365	347	25	33	0	6	0
4506	13286	9594	850	420	258	28086	1240	10	626	2	18	58	7	77	272.66	95	427.21	309	307	298	14	37	1	4	1
14200	7678	4402	6	896	676	27918	659	317	1026	155	62	258	124	155	269.97	267	432.41	492	407	415	16	93	18	4	2
4230	13442	9758	878	406	228	28076	1258	10	588	1	12	63	6	71	273.17	89	428.37	298	293	281	17	43	0	0	4
6912	11372	8144	377	1060	682	28260	958	29	737	9	84	84	16	147	289.35	108	444.18	399	386	358	38	53	0	6	3
4892	13010	9388	729	518	294	28116	1187	9	680	1	10	53	4	79	277.98	100	435.54	351	349	310	13	40	0	4	1
11736	7942	5182	0	1868	1464	28488	555	128	657	25	266	167	68	252	301.01	123	456.4	491	214	396	88	90	12	28	15
12846	7782	4824	3	1464	1130	28250	597	185	808	74	192	194	86	223	288.3	179	451.64	483	297	418	52	92	10	12	10
3972	13704	9930	954	268	152	28036	1308	10	609	2	10	66	5	74	270.61	86	423.71	262	314	272	14	39	0	0	0
3918	13666	9948	964	332	178	28050	1275	7	560	0	8	58	3	68	272.83	100	424.97	286	260	290	16	40	0	0	1
5200	12724	9176	631	642	382	28152	1116	9	691	1	28	51	8	95	279.69	95	438.11	361	362	348	23	37	0	0	0
6264	11978	8520	471	830	550	28204	1008	27	716	3	62	71	18	126	287.51	109	442.41	399	371	359	24	49	0	0	3
11282	7922	5470	0	2060	1528	28612	520	95	568	12	306	169	55	293	320.54	104	466.36	472	183	375	89	82	6	42	31
13596	7714	4546	5	1172	918	28074	609	241	905	97	114	207	114	189	278.14	204	444.13	477	340	461	43	90	12	14	1
13664	7590	4470	2	1252	978	28090	612	245	867	99	124	226	107	193	275.86	197	443.55	485	344	434	38	102	16	10	4
5058	12930	9300	683	508	294	28106	1143	8	682	1	16	64	5	91	277.79	108	435.97	345	359	329	19	42	0	0	1
7286	11222	7954	361	1010	670	28238	922	36	748	6	94	99	22	141	288.92	111	448.5	421	361	361	37	61	3	2	6
13558	7602	4500	1	1294	1028	28144	607	249	829	94	142	220	112	202	281.48	173	447.74	470	316	424	54	97	15	18	5
4668	13190	9500	747	442	278	28106	1198	3	673	0	24	64	3	84	277.26	83	433.89	332	360	304	13	43	0	4	2
14320	7722	4360	4	802	630	27894	666	311	1074	161	52	257	121	150	269.8	272	432.4	483	444	423	15	83	16	8	3
4686	13140	9520	771	466	262	28096	1182	4	650	0	20	69	4	81	278.5	103	432.95	333	326	305	20	54	0	2	2
5810	12372	8842	627	654	412	28126	1124	32	694	7	34	81	15	112	282.02	100	439.16	337	351	310	23	58	2	0	0

SUPPLEMENTARY INFORMATION:Monte Carlo Atomistic Simulation and Machine Learning Analysis of Na-K Eutectic Alloy in Condensed Phases, D. Reitz and E. Blaisten-Barojas, George Mason University, Fairfax, VA 22030

5684	12310	8896	514	770	470	28188	1052	13	732	1	58	84	7	115	283.42	116	440.86	399	381	318	21	59	0	0	1
14454	7544	4324	5	858	634	27884	662	333	1028	164	64	263	124	158	270.7	249	436.33	464	442	422	23	84	26	6	1
3920	13712	9942	975	302	166	28042	1287	7	578	1	0	56	3	74	269.51	101	422.45	280	257	287	9	42	0	0	1
7684	11092	7712	328	954	686	28232	878	31	792	5	100	101	22	139	288.55	140	445.12	456	367	381	27	67	0	4	8
4106	13486	9822	911	428	234	28088	1268	6	586	1	12	56	3	72	275.34	92	428.66	295	286	287	19	39	0	0	0
5306	12578	9116	600	730	416	28174	1078	8	674	0	28	64	5	95	282.28	102	437.24	395	344	338	22	43	0	0	2
5184	12802	9188	641	564	358	28130	1099	10	697	1	32	73	3	95	279.57	100	438.17	374	367	324	21	45	0	2	1
5244	12670	9138	601	666	406	28160	1111	5	698	2	36	68	1	90	281.35	103	436.8	368	375	322	25	44	0	0	5
14258	7600	4364	3	922	698	27916	646	318	990	155	64	256	126	168	273.36	228	438.03	460	403	433	27	87	17	10	2
7562	11166	7826	285	968	634	28228	863	32	829	6	68	101	19	152	289.76	133	449.09	437	404	397	36	59	0	4	3
14372	7738	4374	5	742	578	27858	639	334	1092	163	50	248	132	145	268.12	274	429.9	486	454	461	17	73	18	4	0
5204	12736	9210	622	612	352	28146	1109	6	687	0	30	73	6	90	279.2	112	436.72	390	347	314	17	51	0	0	1
4486	13240	9588	806	488	284	28098	1185	11	604	0	10	56	9	77	275.04	95	431.23	327	302	326	19	40	0	2	4
7972	10978	7596	293	910	652	28206	854	43	844	13	88	128	23	148	287.14	158	446.91	430	381	378	41	81	1	6	8
4436	13258	9640	826	486	266	28100	1218	7	621	2	14	67	3	76	276.93	110	432.76	327	300	288	11	49	0	0	7
4036	13650	9870	962	288	184	28048	1299	11	588	1	20	51	5	66	271.37	98	423.24	280	286	282	12	31	0	0	2
13968	7660	4478	4	994	770	27984	628	290	977	123	102	241	120	191	276.87	236	443.23	447	381	444	38	92	21	12	0
14142	7752	4462	10	842	638	27902	635	315	1031	156	60	255	125	162	270.88	268	433.43	482	408	443	15	82	22	6	4
6746	11562	8236	408	986	636	28232	946	29	705	7	58	78	13	134	286.49	105	444.17	416	363	366	36	44	0	8	5
4372	13418	9720	859	356	184	28054	1234	7	634	1	4	68	5	77	273.25	104	427.63	306	303	298	12	49	0	0	1
4038	13490	9830	918	464	262	28094	1294	3	569	0	10	57	1	89	272.33	80	425.04	268	287	273	13	40	0	0	2
12590	8024	5014	6	1394	1042	28234	591	165	869	44	162	163	89	224	287.81	166	449.63	477	334	467	50	97	11	6	4
13908	7736	4530	12	982	728	27966	613	294	988	125	78	234	136	179	275.97	252	439.35	485	386	451	25	89	14	2	1
4480	13288	9596	828	442	270	28096	1228	6	622	1	20	62	3	75	275.08	96	430.48	322	322	291	14	43	0	0	3
11538	7984	5328	0	1916	1456	28532	526	94	651	20	284	176	48	270	315.31	135	458.13	495	210	375	86	88	8	26	26
4730	13094	9488	733	506	276	28108	1144	6	644	0	14	63	6	81	279.95	102	435.17	353	325	339	15	47	0	0	4
5864	12338	8810	542	682	428	28162	1070	19	736	1	34	65	16	106	284.16	113	441.35	388	400	339	20	40	0	6	1
14294	7714	4380	4	812	630	27894	628	321	1046	151	60	260	127	170	269.24	268	432.38	485	416	435	14	97	19	4	1
3768	13802	10024	1007	272	160	28038	1305	5	558	0	12	54	3	60	270.12	93	422.44	279	262	274	13	35	0	0	4
11582	7952	5194	1	1936	1562	28570	537	106	566	27	314	168	52	292	311.94	123	462.88	487	182	370	90	79	6	28	15
11780	8044	5310	2	1740	1286	28410	551	113	728	25	234	186	67	253	299.08	156	453.79	510	256	402	63	106	5	14	10
5928	12222	8776	480	762	454	28178	1033	9	768	4	30	75	2	114	285.84	100	441.61	394	414	352	21	53	0	4	3
14150	7642	4438	4	914	690	27924	639	309	1033	159	88	264	122	170	269.5	241	431.8	478	418	416	25	96	12	2	0
3808	13720	9978	996	340	202	28062	1313	9	539	1	12	56	5	69	271.85	85	424.1	270	258	269	14	33	0	2	3
3934	13602	9898	961	388	234	28078	1326	8	573	1	22	61	4	68	272.17	92	424	283	289	241	13	47	0	0	1
4400	13332	9638	847	440	266	28092	1230	7	618	1	14	53	5	71	272.8	95	426.47	314	305	310	15	37	0	2	4

4516	13224	9586	802	484	272	28092	1218	8	617	1	10	45	3	79	275.5	88	431.1	311	318	318	15	29	0	0	4
4444	13214	9598	776	540	308	28116	1177	5	591	0	12	64	4	84	278.56	93	433.24	347	293	304	14	38	0	0	3
13804	7784	4536	3	1010	770	27990	649	254	999	122	82	230	105	188	276.78	213	442.26	453	412	438	24	88	12	4	6
12924	7736	4806	2	1440	1116	28236	579	183	815	66	198	185	82	215	285.51	173	449.48	496	289	444	53	98	13	16	7
14392	7548	4394	4	888	606	27880	589	337	1038	161	46	264	141	164	272.38	259	436.21	492	413	469	17	94	15	6	3
5442	12700	9062	610	556	346	28130	1079	16	735	2	24	69	8	94	281.28	124	439.45	396	364	337	16	44	0	0	0
13876	7712	4520	5	1016	764	27980	592	287	973	127	84	243	120	174	275.92	218	442.37	499	383	445	32	101	19	8	2
4678	13162	9512	766	462	264	28094	1210	8	667	1	16	61	3	73	277.47	104	433.54	336	355	290	19	43	0	0	0
11294	7972	5494	5	1996	1478	28572	601	87	617	12	300	177	46	248	305.56	118	456.74	465	226	350	106	101	6	34	19
12266	7956	4992	0	1618	1290	28364	572	139	736	42	226	170	67	217	293.7	164	451.09	510	260	426	65	91	10	14	16
10912	7892	5552	2	2194	1702	28698	529	86	524	22	412	166	41	270	313.32	90	461.4	494	176	362	107	81	4	32	31
4712	13076	9504	768	520	276	28102	1190	10	657	0	14	68	8	84	276.38	98	433.05	342	317	299	13	50	0	0	0
5550	12474	8976	535	704	432	28182	1042	5	732	0	44	71	4	99	284.94	134	438.76	414	373	346	19	45	0	2	4
13882	7530	4434	3	1162	904	28056	574	266	897	111	134	240	114	198	278.85	207	447.18	488	330	441	38	110	16	8	5
4140	13536	9836	933	352	184	28054	1282	7	590	1	6	60	3	70	272.42	84	425.29	286	298	281	13	40	0	0	2
11538	8070	5324	0	1850	1432	28518	536	103	650	17	280	155	53	274	311.36	133	458.49	480	215	417	83	84	6	24	17
4268	13408	9744	875	418	226	28072	1224	11	570	1	8	57	7	78	273.5	88	425.19	311	274	309	13	44	0	0	2
13904	7780	4540	1	964	702	27952	620	293	991	128	60	234	134	158	276.12	235	439.33	487	378	463	30	89	17	2	2
6430	11798	8472	404	898	554	28218	993	17	768	2	60	74	11	111	289.01	106	444.3	424	429	352	28	45	0	6	6
5614	12378	8950	543	752	440	28174	1034	10	708	0	38	69	8	107	284.35	114	440.43	400	361	354	27	48	0	2	0
5540	12648	9050	618	536	310	28100	1078	20	714	8	14	80	8	93	281.32	109	438.57	386	370	343	14	47	0	2	1
14418	7626	4370	5	810	590	27872	655	328	1081	173	56	270	111	139	270.81	281	433.21	510	447	409	14	90	20	2	0
5012	12968	9362	724	480	256	28090	1175	9	697	1	12	60	6	88	277.5	96	433.66	329	358	329	14	46	0	0	1
13664	7818	4608	4	1010	788	28002	607	249	964	115	108	234	97	180	276.01	233	442	491	373	431	37	90	16	6	0
12886	7662	4844	0	1518	1134	28262	585	172	764	62	198	209	80	217	292.1	152	450.52	480	283	411	66	94	14	18	10
14514	7672	4376	7	706	512	27828	636	334	1124	169	48	264	130	152	267.56	278	429.01	476	465	451	15	83	20	0	0
4544	13166	9550	768	534	308	28118	1202	7	593	0	14	56	6	81	278.5	74	434.51	326	335	307	21	37	0	2	1
4852	12982	9430	721	546	286	28108	1141	8	647	1	12	61	5	93	277.67	101	434.82	352	316	332	11	44	0	0	3
12972	7818	4748	0	1394	1106	28228	617	189	814	60	178	197	92	193	287.63	170	449.27	493	306	412	57	107	12	12	11
4956	12894	9346	685	576	326	28122	1135	6	679	1	22	68	2	78	278.47	98	433.8	363	359	322	26	44	0	2	1
12204	7880	5062	2	1640	1314	28400	552	132	702	45	276	201	64	252	295.09	151	451.48	492	254	390	73	99	6	20	12
14208	7700	4418	9	866	650	27904	636	319	1025	146	60	253	134	173	272.48	241	437.08	455	422	448	27	92	17	0	0
11766	7854	5206	4	1890	1464	28508	568	113	630	28	304	154	56	261	304.73	128	454.71	478	228	401	83	76	6	24	18
4468	13226	9598	794	506	290	28102	1202	6	601	1	14	62	4	80	274.61	81	428.64	326	314	307	17	47	0	0	2
10858	7784	5558	2	2332	1770	28756	539	82	482	11	418	153	44	292	321.91	79	465.97	464	148	358	116	75	7	28	32
5128	12782	9254	651	590	344	28136	1130	5	686	0	36	71	4	96	281.59	110	437.58	364	369	315	17	47	0	2	1

4776	13164	9474	773	402	242	28082	1183	5	664	1	24	61	2	77	277.19	96	432.75	336	335	322	13	45	0	0	3
4454	13380	9664	838	348	198	28058	1218	7	643	1	14	60	4	79	274.97	97	428.92	301	316	321	16	46	0	0	1
4272	13386	9696	861	448	276	28100	1244	2	610	0	22	56	2	79	275.55	91	430.31	304	311	298	14	35	0	0	3
4076	13602	9848	939	330	190	28052	1271	7	580	1	6	60	3	71	271.85	97	424.32	277	284	294	19	40	0	0	2
12968	7796	4760	3	1418	1100	28218	630	213	809	71	158	181	114	206	281.78	180	445.31	498	308	428	37	85	14	18	6
11928	8018	5292	1	1658	1236	28398	553	108	747	26	238	178	55	235	299.92	168	452.35	511	256	407	74	102	5	26	10
5262	12774	9204	619	562	304	28114	1083	5	730	1	4	67	4	94	279.67	109	436.86	372	368	361	15	50	0	4	4
7468	11188	7904	320	972	614	28218	892	34	784	3	70	93	23	130	286.97	139	444.82	457	369	379	25	62	0	2	8
5038	12804	9252	660	654	384	28152	1141	2	690	1	20	57	1	93	277.39	115	434.31	370	362	321	12	35	0	0	2
14288	7650	4366	7	886	662	27912	669	328	1010	145	60	242	140	172	271.87	236	434.14	444	431	444	21	88	22	0	2
13054	7840	4864	2	1308	944	28160	623	208	869	66	144	197	105	208	282.85	167	449.91	483	341	426	37	99	21	6	7
11956	7978	5170	2	1700	1338	28428	551	134	700	38	248	206	62	283	306.91	150	457.35	448	235	380	79	110	12	38	12
4548	13294	9578	795	414	242	28084	1202	6	658	1	8	59	3	82	274.28	101	429.46	329	323	313	10	39	0	0	1
14508	7652	4338	6	742	554	27844	625	343	1094	174	46	266	127	154	268.01	283	429.47	491	446	442	13	85	18	4	1
13800	7506	4494	3	1242	904	28070	621	277	848	96	114	231	126	195	280.27	176	446.26	473	323	424	40	110	27	10	3
14304	7768	4442	6	748	552	27860	665	331	1067	156	42	252	133	156	269.73	273	432.16	471	447	435	12	85	17	4	1
10670	7768	5576	2	2422	1894	28834	526	69	455	12	438	163	41	311	321.49	85	468.52	469	142	336	120	84	4	58	32
14402	7654	4418	5	772	550	27854	649	333	1097	172	58	264	121	167	269.72	263	431.71	459	465	432	17	82	13	0	1
5610	12530	9002	581	606	352	28130	1061	15	750	1	26	72	11	105	282.19	129	439.15	384	368	352	14	48	0	4	4
3974	13504	9830	898	482	296	28106	1252	10	532	2	20	53	7	90	271.61	69	424.87	284	268	300	15	31	0	0	3
11382	7982	5304	2	1982	1584	28594	520	92	614	18	320	175	45	278	310.36	131	460.56	494	190	375	92	91	9	40	24
4014	13514	9842	932	446	260	28092	1300	6	579	0	16	64	3	60	272.08	102	424.2	313	284	239	14	41	0	0	2
4396	13348	9654	844	424	248	28082	1243	5	631	0	12	58	3	76	274.71	99	430.19	296	318	304	17	40	0	0	3
4384	13258	9618	812	530	314	28118	1247	5	597	2	10	63	2	76	274.55	80	428.73	313	333	274	19	38	0	4	3
11294	8024	5596	2	1966	1356	28516	531	85	632	20	254	166	35	297	317.93	116	462.66	442	202	403	92	79	3	24	18
11326	7930	5444	2	1976	1522	28580	565	88	615	14	342	167	47	284	312.35	115	461.27	485	219	365	87	86	5	40	13
4276	13418	9744	862	400	220	28070	1223	5	596	1	12	68	3	77	273.04	95	426.64	317	304	296	11	46	0	0	2
11370	7978	5400	1	1980	1514	28584	524	99	603	22	300	177	55	248	313.28	120	458.42	518	194	386	95	101	5	36	24
10764	7912	5658	0	2270	1710	28756	513	70	506	6	388	146	42	302	326.98	93	467.43	493	155	366	100	79	3	52	33
12272	7732	4986	0	1786	1360	28392	535	136	709	48	230	201	69	272	294.05	137	452.64	476	238	420	69	102	8	26	10
4150	13458	9770	898	432	260	28088	1250	7	564	0	16	54	2	79	273.77	76	429.82	293	285	300	16	34	0	2	3
4632	13116	9512	754	514	312	28124	1179	4	639	0	36	59	2	72	278.41	102	433.83	348	323	314	20	41	0	2	4
4610	13110	9510	776	558	320	28124	1191	6	612	0	16	55	5	88	276.75	94	432.33	324	302	319	18	34	0	0	2
4580	13232	9546	789	450	274	28104	1200	6	647	0	22	54	4	85	277.15	109	431.73	333	335	313	10	40	0	0	1
4704	13124	9486	745	498	282	28106	1175	5	659	0	12	62	3	86	276.29	102	432.45	343	338	316	13	45	0	0	1
3998	13630	9870	934	352	210	28068	1285	4	564	0	8	51	4	61	270.71	95	423.59	297	279	285	13	33	0	0	3

3986	13584	9890	952	386	212	28066	1289	5	569	0	8	58	3	69	271.43	102	423.08	281	272	279	19	34	0	0	0
11792	7946	5176	1	1860	1446	28510	537	107	653	25	250	178	61	255	304.94	111	454.99	515	214	399	86	107	4	38	11
4518	13314	9600	823	404	236	28082	1226	4	643	1	8	61	2	76	273.42	100	428.42	312	329	305	14	38	0	2	3
4108	13496	9808	906	412	240	28080	1242	5	558	1	16	61	3	70	273.59	98	427.04	316	265	291	15	40	0	0	1
6474	11886	8482	438	816	488	28188	981	14	798	5	40	92	5	123	287.55	137	444.13	407	384	361	29	50	0	2	2
13512	7796	4644	4	1126	854	28046	639	261	915	111	106	227	115	177	278.04	205	443.99	475	365	425	40	93	20	8	4
12740	7984	4926	3	1360	1032	28200	625	192	854	76	146	179	87	208	283.86	183	448.13	491	351	432	42	66	8	12	4
4196	13446	9754	890	426	250	28086	1266	8	588	1	14	55	7	73	273.75	84	427.28	299	299	290	13	36	0	0	2
3968	13632	9892	959	354	208	28064	1295	9	566	1	8	53	5	65	271.11	94	423.49	287	277	278	15	37	0	2	1
14154	7718	4492	5	866	614	27898	622	320	1038	147	48	246	135	158	273.55	238	437.32	483	427	457	25	84	15	6	0
11918	7976	5200	1	1718	1344	28454	552	121	698	32	266	178	64	267	305.13	147	456.39	479	222	392	84	99	4	28	10
14472	7574	4312	7	830	620	27870	621	355	1038	176	60	267	137	158	269.78	274	432.1	484	420	447	20	85	21	2	0
4032	13510	9860	931	438	234	28082	1273	7	557	2	8	60	4	66	272.59	92	426.37	299	268	280	16	36	0	0	3
5130	12720	9198	643	674	408	28166	1147	4	679	0	34	66	3	84	282.02	113	437.38	379	359	303	16	52	0	0	3
10848	7988	5632	2	2172	1648	28698	519	85	524	11	352	167	45	288	325.3	94	466.5	454	153	359	119	93	5	54	38
4536	13170	9544	764	522	318	28118	1194	3	625	0	28	71	3	83	277.72	81	433.49	331	336	292	19	47	0	0	4
13736	7838	4576	7	988	764	27994	641	289	955	123	86	221	129	188	276.14	220	442.29	455	401	451	28	86	16	4	3
4180	13558	9802	893	328	184	28056	1263	6	603	0	4	49	3	74	272.38	89	425.55	288	308	305	12	34	0	0	2
6076	12128	8716	541	746	450	28170	1057	20	738	9	50	83	10	114	287.83	115	442.3	376	378	333	26	53	0	4	3
14322	7758	4436	8	726	548	27846	642	326	1107	167	56	264	121	150	268.17	291	429.35	481	441	440	17	90	19	0	0
4358	13424	9678	844	376	228	28074	1242	7	625	0	8	56	5	72	273.19	89	428.51	292	311	308	20	39	0	2	4
5332	12558	9064	591	728	456	28186	1078	10	661	1	44	75	5	99	283.99	102	440.74	412	344	308	16	44	1	4	2
14394	7674	4396	7	786	566	27862	645	324	1096	158	40	255	131	145	271.14	265	432.51	490	447	442	16	91	17	6	1
14282	7666	4384	3	866	644	27900	660	323	1040	162	58	261	110	144	270.54	253	433.11	501	439	400	19	87	18	0	3
3944	13674	9932	962	314	176	28050	1298	5	586	1	8	62	3	77	270.7	89	422.22	273	274	275	10	47	0	2	1
4080	13518	9828	914	408	232	28078	1285	7	576	1	12	50	4	71	271.44	80	424.75	284	287	291	12	35	0	0	4
4492	13238	9606	795	470	270	28098	1187	5	642	0	22	63	3	87	275.62	106	431.09	332	310	308	16	43	0	0	0
14026	7782	4484	8	842	686	27920	652	297	1035	151	90	247	112	162	273.81	247	437.82	467	447	434	26	81	12	10	1
11158	7976	5514	1	2044	1546	28612	516	93	585	15	344	154	51	284	312.83	105	460.55	501	198	391	89	79	6	28	18
13270	7888	4758	3	1180	874	28082	621	224	915	76	110	215	110	190	282	206	445.33	458	346	446	47	108	15	0	5
4080	13580	9830	945	346	212	28062	1301	9	584	1	12	53	4	61	271.23	83	424.74	284	287	274	16	33	0	2	3
5508	12540	9030	575	666	382	28150	1071	10	734	1	24	64	5	110	279.44	125	436.26	385	376	349	13	41	0	0	1
5204	12766	9190	619	606	354	28140	1089	4	698	0	20	70	4	95	281.83	117	439.41	382	344	338	15	45	0	0	5
4408	13330	9670	845	418	234	28076	1226	6	641	2	16	54	4	70	272.69	99	426.16	308	308	322	15	40	0	0	4
3996	13716	9916	974	250	148	28036	1301	3	614	0	8	69	3	71	270.26	98	422.52	276	290	269	10	43	0	2	1
4488	13224	9626	813	486	258	28096	1222	7	628	0	14	59	2	74	277.36	91	432.57	309	326	300	24	40	0	0	3

SUPPLEMENTARY INFORMATION:Monte Carlo Atomistic Simulation and Machine Learning Analysis of Na-K Eutectic Alloy in Condensed Phases, D. Reitz and E. Blaisten-Barojas, George Mason University, Fairfax, VA 22030

4120	13466	9776	911	440	270	28092	1287	11	565	1	18	55	7	70	274.69	95	427.33	301	282	266	14	37	0	2	1
5380	12656	9040	626	646	412	28158	1135	10	689	2	22	59	7	105	282.81	96	439.82	360	364	319	14	44	0	2	1
3844	13690	9978	1004	350	188	28056	1330	8	551	2	4	61	4	66	269.83	81	422	265	261	255	14	46	0	2	3
4162	13440	9792	895	444	238	28084	1260	10	567	1	8	64	6	73	277.87	84	432.42	309	278	273	12	44	0	0	3
4236	13426	9750	869	424	236	28082	1266	6	606	0	10	50	3	77	274.84	73	428.36	283	320	295	19	32	0	0	2
12652	7932	4886	1	1460	1140	28252	597	178	824	63	166	183	91	220	282.6	179	447.6	489	301	442	47	91	11	12	8
11264	7874	5472	0	2090	1544	28600	518	89	582	16	320	160	47	291	312.9	94	458.59	462	184	416	86	96	8	34	29
4592	13256	9592	809	396	216	28066	1223	6	651	0	14	53	3	62	275.72	92	431.28	316	337	312	22	40	0	0	2
10910	7834	5580	1	2340	1692	28736	472	76	477	11	336	166	42	315	321.71	89	467.1	517	136	356	103	86	2	42	22
5352	12716	9126	633	564	338	28122	1099	11	728	0	24	66	8	101	279.13	109	435.49	356	360	348	19	47	0	2	3
7454	11038	7848	331	1114	724	28278	896	32	738	3	92	102	22	157	289.51	116	446.57	425	363	362	37	61	2	8	7
11876	7744	5202	0	1908	1438	28496	466	138	630	35	298	186	72	255	313.13	126	457.91	531	188	409	90	103	7	30	17
4328	13442	9714	875	350	212	28066	1246	7	610	1	18	54	3	61	273.58	93	426.97	314	300	299	18	38	0	2	1
4042	13576	9862	944	366	208	28066	1299	6	585	0	12	51	3	71	271.76	88	425.49	280	285	279	13	38	0	0	1
4068	13692	9888	937	240	136	28030	1262	9	612	2	6	62	5	61	270.22	106	422.69	299	286	295	14	44	0	0	1
4086	13566	9844	943	352	198	28054	1289	7	596	1	8	58	5	73	272.34	84	424.59	277	282	282	13	38	0	0	2
4490	13350	9642	854	364	204	28060	1244	9	640	2	8	55	6	74	274.15	95	428.9	296	324	308	16	39	0	2	1
10570	7884	5762	1	2378	1734	28766	499	63	520	11	398	172	29	278	331.65	89	469.18	469	171	343	142	89	0	38	35
5640	12454	8922	567	684	422	28154	1061	13	696	4	30	77	8	126	282.22	89	439.42	350	358	356	22	52	0	2	3
11340	7864	5412	0	2044	1556	28586	541	92	617	22	326	180	46	260	311.4	132	461.45	490	200	367	100	102	4	40	23
13734	7844	4620	7	964	724	27974	642	265	1009	136	82	246	97	172	273.55	240	439.15	479	411	420	31	97	14	6	0
11300	8154	5496	0	1842	1408	28526	553	84	665	14	306	173	45	262	309.63	130	458.77	459	228	397	97	97	4	18	19
14430	7698	4384	4	734	550	27852	626	338	1093	166	54	264	135	141	270.6	300	433.41	506	434	439	16	93	10	2	1
13008	7842	4748	7	1326	1072	28186	620	212	829	78	178	205	91	215	281.16	159	445.75	469	320	422	46	96	18	10	5
4352	13336	9676	855	450	258	28086	1218	6	591	0	14	55	4	74	274.57	107	430.58	330	287	304	12	35	0	0	1
4642	13272	9556	811	358	216	28066	1186	9	660	1	22	60	5	74	274.6	122	428.54	329	310	326	16	38	0	0	2
11902	7904	5284	5	1772	1288	28416	586	129	703	40	238	185	61	260	303.11	133	455.89	472	262	378	72	96	5	26	12
5234	12802	9218	662	526	304	28112	1135	7	715	0	28	68	6	87	282.35	123	439.43	367	357	327	9	46	0	0	4
14656	7430	4208	5	878	642	27870	636	339	1033	168	50	276	134	151	273.5	254	436.76	476	424	425	25	89	19	4	4
4122	13630	9842	935	272	164	28042	1285	8	605	1	10	56	4	64	271.9	96	425.53	284	303	286	16	36	0	2	1
5550	12424	8932	601	762	474	28178	1086	17	653	2	34	62	12	107	281.92	116	438.19	387	331	334	18	32	0	2	0
4304	13386	9726	860	404	234	28080	1227	11	590	1	24	49	8	79	273.85	85	428.71	295	292	326	17	35	0	2	3
13002	7918	4858	4	1276	952	28150	602	210	882	79	134	194	98	214	279.88	193	446.24	485	337	440	39	87	11	10	4
5676	12422	8928	546	688	410	28160	1060	10	729	3	34	75	5	107	284.76	117	440.4	391	382	339	18	48	0	2	3
14276	7728	4402	10	794	620	27888	686	311	1068	157	60	258	112	146	269.63	259	433.48	475	447	411	20	92	26	6	0
4526	13358	9630	808	344	192	28058	1219	8	673	2	6	65	2	68	275.78	102	430.78	327	344	298	13	44	0	2	2

SUPPLEMENTARY INFORMATION:Monte Carlo Atomistic Simulation and Machine Learning Analysis of Na-K Eutectic Alloy in Condensed Phases, D. Reitz and E. Blaisten-Barojas, George Mason University, Fairfax, VA 22030

12408	7870	5102	0	1568	1132	28294	549	164	762	46	202	195	77	212	297.17	138	454.29	493	287	424	83	108	8	12	10
3992	13666	9924	975	282	160	28042	1302	7	595	2	16	63	5	66	270.82	94	422.69	273	285	277	15	42	0	2	1
14080	7688	4476	8	932	682	27928	655	291	1031	131	64	244	121	161	276.1	237	440.77	467	423	429	28	98	26	6	3
12736	7866	4884	2	1458	1120	28260	581	172	818	53	182	199	90	242	293.58	159	451	454	290	434	60	112	11	14	6
14216	7678	4386	8	868	684	27912	586	336	974	161	68	262	128	175	274.4	270	436.95	493	357	447	24	83	19	12	1
5148	12868	9236	684	518	314	28106	1126	14	697	3	22	69	9	98	279.55	114	435.49	345	343	340	17	39	0	0	1
4422	13318	9656	837	424	242	28080	1199	9	609	1	18	50	6	87	273.1	101	429.12	314	280	328	12	33	0	0	2
6748	11684	8292	428	852	552	28192	980	37	729	10	56	85	16	123	288.49	115	443.6	416	374	356	24	48	2	8	5
4682	12986	9432	728	642	386	28158	1161	8	600	1	28	63	5	97	277.18	98	433.99	355	311	299	17	42	0	2	1
12756	7806	4844	4	1480	1164	28266	601	180	777	58	194	175	85	202	283.78	171	446.86	516	301	423	57	88	14	20	4
7266	11318	8032	371	944	580	28202	938	34	800	10	60	88	19	128	285.65	117	445.82	423	400	375	34	54	0	2	2
3916	13586	9888	965	424	254	28082	1304	8	548	2	12	60	4	70	270.2	95	422.16	286	260	256	15	42	0	2	2
13430	7486	4576	0	1418	1080	28184	567	242	765	84	178	231	114	213	285.09	165	448.51	493	278	430	51	113	25	16	7
14422	7614	4350	3	814	614	27886	680	325	1050	161	66	266	122	152	272.22	251	435.53	475	461	402	17	92	21	6	1
4410	13408	9678	847	356	202	28062	1227	3	643	0	8	56	2	56	273.21	95	427.41	331	322	309	15	44	0	0	2
12224	7892	5114	2	1622	1246	28368	562	163	740	51	242	189	70	268	300.26	145	452.94	467	258	396	65	99	13	28	15
13232	7804	4712	1	1272	974	28140	631	204	888	74	132	205	91	186	282.26	198	446.3	487	345	427	47	101	16	14	4
4312	13482	9750	878	324	176	28052	1240	4	641	0	8	68	2	76	273.17	103	429.21	286	306	303	20	47	0	0	2
11986	7926	5158	0	1736	1336	28420	540	143	685	29	262	166	73	264	303.52	137	457.33	480	226	424	81	84	18	14	9
4802	12924	9384	708	652	370	28146	1143	5	595	1	14	66	3	94	280.66	93	436.6	359	304	316	14	32	0	0	3
14218	7530	4392	8	1012	718	27938	625	324	973	173	62	275	107	153	272	229	436.39	509	402	403	29	86	21	4	1
12730	7866	4914	2	1440	1096	28242	603	181	835	64	182	204	90	225	284.87	180	449.11	493	304	409	48	105	13	14	4
4868	13058	9414	726	468	272	28100	1173	5	678	0	16	70	4	80	277.35	100	434.15	345	366	311	15	44	0	4	3
10952	7840	5514	0	2240	1740	28734	529	79	522	19	388	172	34	266	323.14	112	464.33	513	167	328	106	70	7	56	39
13128	7816	4696	1	1302	1054	28180	586	205	835	68	172	203	102	226	284.83	183	449	476	313	443	42	98	12	12	7
13640	7882	4628	11	984	762	27992	608	272	995	116	90	225	120	193	275.72	223	442.08	467	389	471	27	93	18	6	1
11776	7964	5272	2	1826	1356	28466	537	105	671	28	254	161	54	239	298.66	142	455.92	518	228	425	81	84	6	18	11
4304	13448	9712	857	366	226	28076	1243	5	623	0	20	45	3	67	273.79	96	427.44	314	317	306	12	29	0	0	4
10876	7982	5594	0	2206	1644	28678	527	72	535	9	340	158	40	290	318.03	103	463.58	492	168	372	95	95	5	34	23
4072	13538	9850	919	380	214	28070	1272	3	601	0	14	65	2	68	271.16	89	424.37	293	290	281	18	48	0	2	1
11506	7832	5370	0	2038	1488	28552	497	101	612	23	286	178	53	303	308.14	132	457.81	475	166	403	82	92	6	30	19
11676	7856	5316	2	1900	1430	28506	545	112	624	27	288	188	48	264	306.14	126	455.34	487	199	376	87	97	13	38	18
11634	7930	5256	1	1864	1482	28514	575	104	679	25	316	167	52	267	303.53	134	456.08	479	237	388	85	97	6	32	9
11576	7968	5380	1	1888	1390	28492	491	118	623	31	256	180	67	263	310.7	132	458.02	495	184	421	95	88	4	30	16
4264	13476	9750	865	358	206	28066	1237	7	627	1	12	64	5	65	274.33	90	429.01	307	308	303	22	44	0	0	0
3926	13778	9970	986	220	122	28022	1322	3	629	0	6	63	1	66	270.27	99	422.64	267	294	264	10	47	0	0	1

SUPPLEMENTARY INFORMATION:Monte Carlo Atomistic Simulation and Machine Learning Analysis of Na-K Eutectic Alloy in Condensed Phases, D. Reitz and E. Blaisten-Barojas, George Mason University, Fairfax, VA 22030

13328	7826	4736	2	1212	880	28090	621	237	903	82	98	202	115	196	280.1	184	446.26	473	352	448	36	96	15	8	6
4790	13146	9466	743	430	244	28088	1170	4	687	0	12	53	3	84	274.39	107	429.52	341	348	334	11	37	0	0	1
3990	13572	9876	940	400	226	28074	1277	6	560	0	10	52	3	74	272.2	93	425.77	285	263	288	14	36	0	0	3
10906	7914	5642	0	2232	1610	28680	528	74	526	12	340	149	38	286	324.29	91	464.77	469	173	373	112	81	3	36	27
5502	12610	9032	583	610	364	28142	1072	14	726	0	24	61	11	94	280.86	117	438.94	395	372	354	18	42	0	0	0
11860	8004	5286	2	1740	1288	28442	539	126	691	36	236	180	69	254	302.73	144	456.61	469	232	428	94	89	3	28	8
11482	8084	5336	1	1846	1456	28522	559	107	656	21	278	175	56	255	305.41	118	458.24	477	240	391	90	95	9	40	19
3832	13628	9938	973	420	248	28082	1306	11	513	2	16	59	6	79	271.35	76	425.53	259	258	271	21	38	0	0	1
13890	7696	4550	7	1026	730	27968	624	289	969	125	74	239	117	175	276.98	235	440.69	478	373	436	32	94	21	2	1
14270	7654	4394	7	870	640	27882	628	335	1015	171	54	272	128	150	269.45	260	432.82	495	415	426	23	90	18	0	1
4090	13542	9826	903	378	224	28078	1237	4	583	0	18	64	2	67	274.56	99	430.11	319	265	291	16	45	0	0	2
14334	7704	4408	6	770	582	27860	613	341	1068	162	60	251	137	146	268.04	289	429.35	500	413	460	20	77	20	2	0
12046	8010	5216	3	1662	1212	28364	607	116	742	36	202	170	60	228	291.2	175	450.21	514	276	393	54	101	6	16	10
5390	12668	9092	619	600	360	28136	1118	10	700	1	24	66	7	95	282.42	106	438.78	366	376	335	13	43	0	2	3
3928	13698	9938	972	300	172	28046	1282	6	575	1	10	53	4	61	270.39	103	423.66	293	263	289	13	31	0	0	2
4492	13338	9638	810	378	210	28066	1204	9	634	1	10	73	8	85	275.7	84	431.82	314	332	308	14	47	0	0	0
4746	12992	9430	719	610	344	28136	1153	2	628	1	14	65	0	92	278.05	79	433.84	351	324	318	14	45	0	0	4
10456	7816	5696	1	2508	1896	28850	504	59	440	7	408	149	33	287	328.98	91	472.36	479	134	352	136	68	3	68	36
14480	7662	4362	5	746	544	27838	659	333	1091	165	42	265	125	150	268.01	282	429.37	472	441	433	13	89	23	2	2
10984	7878	5476	1	2242	1720	28710	509	80	534	14	376	149	33	283	317.21	106	460.16	522	185	365	111	79	6	34	20
3974	13614	9906	943	356	198	28060	1276	7	576	1	12	63	4	76	273.9	85	428.04	287	276	279	8	40	0	0	5
14348	7648	4374	7	822	624	27888	655	314	1045	145	70	246	128	155	273	252	439.09	451	426	456	30	83	16	2	1
4820	13022	9426	711	528	300	28118	1150	4	674	1	22	68	3	84	277.5	102	432.64	365	338	314	13	45	0	0	2
5014	12906	9320	685	544	310	28114	1134	7	667	1	20	61	4	78	278.46	112	434.84	364	337	329	21	43	0	0	3
4606	13182	9526	783	484	290	28110	1192	3	625	1	22	58	2	82	279.69	98	433.61	335	312	311	15	38	0	0	2
4160	13556	9820	893	328	180	28050	1249	6	617	1	6	73	5	70	271.98	108	425.44	317	302	273	10	47	0	0	1
4148	13520	9804	906	372	212	28066	1275	6	592	1	10	60	4	71	272.93	85	425.75	292	304	279	12	42	0	0	4
4040	13532	9854	893	416	228	28078	1270	7	565	1	8	60	4	67	273.59	80	425.77	300	291	281	15	42	0	0	3
14346	7612	4388	3	866	622	27890	629	327	1033	162	48	256	124	146	269.86	274	431.78	509	412	437	12	79	22	6	5
14364	7646	4394	5	816	594	27872	655	317	1077	172	56	262	109	163	267.46	271	429.48	476	444	424	12	78	16	2	1
10798	7888	5648	0	2268	1696	28728	526	92	530	15	378	150	51	288	315.99	107	464.89	473	162	374	118	85	5	50	26
14224	7680	4412	7	868	652	27902	642	323	1018	147	62	246	139	166	271.43	255	435.18	471	408	442	21	86	20	4	1
14060	7572	4482	9	1026	730	27960	619	298	953	140	82	258	116	180	276.25	230	441.86	474	363	425	31	102	18	8	2
10930	7780	5582	0	2308	1718	28762	526	74	501	14	398	179	28	291	331.66	93	470.42	492	163	317	116	77	8	42	26
5082	12786	9214	660	638	400	28156	1113	5	661	0	36	61	3	98	278.49	115	437.45	371	343	330	18	42	0	0	2
4760	13070	9434	768	516	310	28110	1180	9	638	0	18	63	7	91	279.41	93	435.14	329	311	313	16	43	0	2	2

SUPPLEMENTARY INFORMATION:Monte Carlo Atomistic Simulation and Machine Learning Analysis of Na-K Eutectic Alloy in Condensed Phases, D. Reitz and E. Blaisten-Barojas, George Mason University, Fairfax, VA 22030

4970	12784	9274	661	696	410	28160	1125	4	659	0	26	75	3	111	279.83	98	437.26	345	335	315	23	48	0	0	1
4472	13272	9602	811	460	272	28094	1209	8	620	0	16	54	7	85	275.63	90	431.36	304	319	324	16	36	0	0	3
3772	13762	10032	1029	306	162	28042	1309	8	532	2	8	64	4	64	270.94	97	422.96	274	227	266	16	44	0	0	1
4242	13418	9718	862	420	266	28092	1234	9	593	1	28	56	4	79	274.62	98	428.41	305	289	297	16	34	0	0	3
11216	7874	5460	2	2042	1606	28638	558	94	578	24	408	161	48	265	306.18	116	462.73	504	210	358	105	78	7	32	14
4280	13466	9722	884	370	222	28070	1245	5	614	1	10	62	3	60	272.81	110	425.88	335	296	282	8	46	0	0	3
5578	12546	8956	575	638	408	28160	1060	15	697	2	26	61	8	104	281.46	112	438.13	391	355	358	20	35	1	8	0
13350	7690	4634	5	1286	1006	28134	613	234	831	94	154	234	99	219	277.13	156	444.3	479	335	400	38	115	13	14	2
12328	7856	5008	0	1654	1274	28368	524	151	725	38	232	188	77	247	299.63	179	455.21	509	218	414	72	104	4	16	8
4390	13458	9688	848	314	192	28054	1242	8	653	1	10	53	3	63	273.14	97	428.2	310	344	305	16	36	0	0	2
5392	12654	9102	605	588	358	28134	1092	10	715	1	38	74	6	95	279.69	113	436.61	377	369	334	21	44	0	2	1
4112	13538	9834	901	364	204	28066	1262	7	580	2	14	68	3	68	273.12	89	429.28	295	293	282	18	45	0	0	3
6804	11778	8260	422	766	518	28182	961	32	787	6	56	82	22	135	290.05	132	446.24	398	375	388	22	55	0	0	6
12578	7716	4890	3	1646	1270	28344	552	159	683	51	212	201	74	260	300.76	142	452.49	498	223	376	58	101	7	30	12
5142	12786	9228	668	594	352	28132	1144	8	676	0	30	57	7	104	279.17	80	434.24	325	362	345	21	38	0	0	1
12824	7896	4858	3	1410	1064	28206	594	182	867	49	140	188	101	194	287.65	207	447.64	496	307	452	56	110	11	14	6
4220	13554	9798	905	292	168	28046	1269	6	629	1	14	73	4	83	272.94	100	427.28	277	307	285	11	47	0	0	1
4318	13312	9680	834	478	288	28108	1206	8	589	0	32	61	4	97	276.37	85	432.17	297	290	309	16	46	0	0	6
4150	13446	9796	911	432	242	28084	1252	11	574	2	18	54	7	69	272.89	95	427.66	307	263	296	16	37	1	0	2
4200	13370	9770	880	484	256	28092	1224	10	562	0	12	57	8	76	275.58	90	430.42	323	267	299	12	40	0	0	3
5016	12838	9292	694	616	354	28138	1177	8	661	0	20	48	5	77	279.36	89	434.49	349	351	317	22	36	0	2	2
6068	12156	8760	542	718	402	28142	1051	22	748	4	38	88	14	96	286.11	116	441.38	397	376	329	26	59	1	0	3
11950	7826	5328	0	1834	1242	28418	542	121	682	27	216	183	64	249	305.97	131	457.76	496	228	399	73	100	7	22	20
4136	13606	9852	948	280	152	28034	1290	10	618	1	8	63	7	61	270.98	106	423.53	289	301	276	9	48	0	0	5
4704	13172	9510	754	434	250	28090	1170	4	665	0	20	67	3	78	276.58	106	433.04	345	335	322	13	48	0	0	3
10586	7750	5692	0	2468	1854	28854	505	52	468	5	452	146	22	310	330.16	100	470.24	509	138	321	113	80	3	48	30
7824	11014	7726	335	958	610	28204	881	55	801	17	68	93	29	132	289.85	172	445.11	475	373	381	22	51	1	4	2
13704	7824	4596	4	1016	768	27996	657	257	987	126	80	228	106	186	275.74	230	443.04	453	397	437	29	84	10	8	1
7226	11274	8040	340	980	618	28226	935	26	803	7	84	90	17	143	289.08	111	444.79	418	423	366	33	54	0	4	1
5372	12620	9106	616	650	372	28144	1095	16	653	3	22	61	8	88	282.19	97	438.97	383	339	337	23	39	0	2	2
4724	13014	9436	731	594	346	28134	1145	5	624	0	20	59	3	86	277.43	87	432.35	351	315	332	19	38	0	0	4
14088	7730	4478	7	876	666	27918	622	305	1026	158	74	249	107	156	272.95	266	437.17	502	414	437	21	78	16	6	1
14386	7716	4414	7	742	542	27846	630	322	1092	163	40	257	120	136	269.82	279	433.71	496	437	448	22	84	21	6	1
5408	12668	9062	637	590	370	28124	1101	23	665	10	26	82	9	104	280.77	96	437.68	361	342	320	19	49	0	0	3
12596	8114	5034	7	1304	980	28176	611	190	888	66	144	192	89	211	280.1	181	447.16	503	319	420	34	100	13	4	6
4512	13232	9568	808	474	292	28102	1224	5	615	0	22	75	4	81	276.41	91	430.86	312	318	283	18	43	0	2	4

SUPPLEMENTARY INFORMATION:Monte Carlo Atomistic Simulation and Machine Learning Analysis of Na-K Eutectic Alloy in Condensed Phases, D. Reitz and E. Blaisten-Barojas, George Mason University, Fairfax, VA 22030

5386	12562	9062	580	708	418	28164	1089	6	690	0	28	69	3	100	280.82	88	437.24	379	381	333	21	47	0	0	3
13662	7870	4650	5	980	734	27988	658	257	1012	113	92	230	112	168	276.52	228	440.29	470	432	432	31	98	15	0	2
4946	12988	9380	699	492	274	28098	1141	6	669	0	18	68	4	88	279.75	90	434.45	341	353	334	19	50	0	0	3
4136	13552	9824	920	344	192	28056	1248	7	601	1	8	51	3	66	272.25	94	424.63	302	263	310	16	38	1	0	1
5224	12784	9192	630	574	338	28136	1085	5	707	0	24	68	4	109	281.54	107	438.02	380	355	347	9	51	0	0	0
11484	8000	5434	3	1930	1380	28498	550	117	643	15	248	146	72	270	310.75	121	459.05	458	199	435	91	89	8	22	14
4340	13352	9710	853	414	236	28078	1244	8	617	1	24	64	4	71	274.46	96	430	303	318	287	20	45	0	2	3
4354	13348	9692	850	442	240	28084	1237	9	619	1	8	54	4	64	274.16	98	428.64	330	302	291	14	35	0	0	2
13462	7580	4534	0	1336	1060	28146	607	240	814	90	150	222	110	217	283.12	174	445.66	464	321	418	51	108	15	22	3
10980	7910	5576	2	2160	1634	28676	522	81	520	16	382	167	45	291	321.51	87	464.97	458	172	384	116	85	10	26	22
4620	13162	9538	770	478	282	28112	1173	9	650	0	32	64	7	84	278.24	109	434.42	337	319	317	20	43	0	0	1
5382	12776	9128	649	496	298	28098	1133	14	715	4	18	69	7	83	279.59	112	435.17	351	390	335	19	46	0	0	4
4492	13274	9570	802	444	292	28104	1194	8	622	0	32	62	5	92	275	83	430.49	315	323	313	12	39	0	0	6
12460	7718	4984	1	1676	1262	28356	580	169	717	52	230	186	85	238	293.48	138	450.87	494	275	392	66	96	12	26	11
4412	13256	9656	841	496	264	28094	1228	10	584	0	10	62	7	80	276.51	83	432.68	311	298	296	15	38	0	0	2
14088	7762	4452	4	872	682	27936	630	311	998	143	74	240	131	162	275.11	251	439.74	483	407	451	27	77	16	6	0
4106	13636	9874	941	268	142	28032	1285	7	619	2	6	63	4	60	271.39	99	423.37	300	299	273	9	41	0	0	2
14360	7614	4372	3	856	620	27876	662	330	1046	168	54	269	119	154	271.02	260	437.09	468	440	417	23	84	22	0	1
14062	7796	4500	6	852	634	27900	645	319	1034	149	56	262	137	157	270.08	243	433.44	475	413	430	24	101	17	0	2
5582	12448	8968	589	686	420	28150	1057	15	692	5	46	68	6	103	280.65	106	439.87	382	344	356	25	46	0	0	1
12180	7950	5056	2	1678	1290	28386	562	141	721	41	212	196	78	236	300.63	174	452.18	492	225	410	73	104	6	20	14
12440	7988	5034	4	1476	1142	28298	598	158	814	48	198	197	90	246	290.81	166	451.12	449	292	414	64	110	5	18	10
10762	7764	5682	0	2304	1748	28770	525	80	498	13	464	169	35	298	324.7	89	466.88	465	158	345	121	86	6	46	33
10872	7712	5540	0	2354	1822	28796	480	77	492	16	430	170	35	269	332.83	91	471.96	464	139	369	156	76	4	60	29
4222	13482	9784	887	370	196	28058	1253	8	617	1	4	54	5	61	271.11	100	424.12	307	307	304	15	35	0	0	2
11510	8010	5366	1	1932	1420	28512	547	96	648	22	252	172	52	264	310	110	458.48	477	232	408	89	94	5	22	14
3904	13714	9940	951	306	178	28048	1281	9	570	1	6	56	5	63	271.79	85	424.9	283	279	290	17	32	0	0	3
13132	7742	4726	3	1358	1052	28190	629	204	847	71	160	187	98	204	283.46	188	447.11	483	317	436	45	97	16	18	2
14488	7660	4316	5	742	584	27854	642	348	1064	170	62	250	142	157	270.28	287	432.82	480	436	449	9	71	18	2	3
14206	7758	4460	4	802	594	27870	659	326	1072	159	46	258	131	153	267.23	265	428.58	472	440	430	18	88	20	2	2
4282	13394	9734	856	430	232	28080	1218	5	592	1	8	57	2	83	273.92	96	428.81	308	284	313	16	39	0	0	0
4632	13202	9552	801	438	246	28088	1219	10	650	0	18	60	7	75	275.59	90	431.7	323	343	294	17	42	0	0	2
12362	7790	5078	1	1702	1206	28344	583	161	697	49	186	184	71	243	298.26	151	452.49	491	243	395	67	86	17	18	6
12508	7818	4934	1	1584	1238	28326	576	170	761	43	228	175	91	242	291.65	170	449.94	477	276	430	67	87	14	14	5
3914	13638	9958	966	358	180	28054	1292	6	559	2	6	71	2	63	270.89	89	423.61	284	264	267	18	52	0	0	2
5522	12500	9004	583	682	414	28166	1091	12	703	1	42	61	6	91	285.12	108	439.44	384	374	333	28	37	0	2	1

SUPPLEMENTARY INFORMATION:Monte Carlo Atomistic Simulation and Machine Learning Analysis of Na-K Eutectic Alloy in Condensed Phases, D. Reitz and E. Blaisten-Barojas, George Mason University, Fairfax, VA 22030

13038	7886	4816	7	1292	970	28144	615	219	853	77	138	201	111	230	281.97	184	445.42	467	314	433	31	92	7	4	3
4738	13056	9456	731	534	312	28122	1148	8	621	1	26	73	6	83	277.72	101	433.28	363	313	311	17	43	0	0	2
5098	12792	9200	654	632	402	28160	1087	5	652	0	36	68	3	92	281.42	110	437.54	388	311	340	22	44	0	0	1
4002	13652	9882	961	328	190	28058	1301	6	574	2	4	59	3	67	271.62	98	424.91	287	281	272	8	34	0	0	3
4990	12940	9284	683	544	346	28130	1162	5	689	0	26	65	5	100	281.2	89	435.84	316	365	329	25	38	0	0	1
4042	13602	9864	946	340	198	28058	1300	5	594	1	12	58	3	69	270.49	86	423.78	272	296	281	14	42	0	0	3
5378	12534	9026	593	734	466	28184	1098	9	670	1	44	66	2	106	285.2	85	440.02	374	369	323	22	48	0	2	3
3932	13664	9918	985	336	196	28056	1336	11	568	2	10	52	6	64	270.09	83	423.04	263	289	262	12	30	0	0	4
14554	7552	4290	3	810	600	27866	617	330	1067	168	56	259	126	152	268.64	289	430.77	515	430	440	6	83	11	4	3
14264	7756	4440	6	784	580	27874	632	321	1071	152	50	251	133	157	270.29	266	433.62	490	429	449	13	87	16	0	1
4510	13330	9640	827	370	200	28060	1215	8	649	0	10	55	4	71	277.27	111	432.61	319	314	316	14	35	0	0	2
5574	12654	8998	576	532	342	28132	1064	13	746	1	32	70	7	103	279.77	124	437.64	378	389	358	20	47	0	0	1
4412	13386	9662	860	380	220	28068	1264	10	646	2	8	61	3	79	272.59	94	426.06	303	326	279	6	43	0	0	2
4822	13126	9462	765	426	228	28066	1186	9	675	3	2	65	4	94	276.01	95	431.94	307	348	320	14	44	0	0	3
4376	13364	9680	850	412	232	28074	1234	7	619	0	10	61	2	87	273.7	93	428.33	300	303	295	12	48	0	0	2
6984	11448	8142	385	954	626	28248	951	23	775	6	90	90	12	130	287.62	134	443.67	435	382	355	33	63	0	4	1
5692	12464	8918	559	650	394	28148	1063	15	698	4	30	74	7	107	282.71	110	439.66	390	368	340	16	46	1	0	2
5464	12488	9018	590	724	434	28166	1094	15	700	1	36	72	10	98	284.91	111	438.8	386	371	316	22	49	0	2	2
4000	13640	9900	950	330	178	28050	1300	7	586	1	2	57	4	67	270.53	95	422.39	276	290	275	15	43	0	0	2
4066	13586	9854	952	346	196	28056	1297	11	587	2	8	55	7	66	270.99	95	423.54	287	279	276	10	35	0	0	3
4500	13236	9584	814	480	286	28108	1223	4	631	0	20	62	2	73	277.33	100	432.33	327	313	291	18	44	0	2	2
4416	13242	9646	826	500	276	28098	1221	5	618	0	18	61	4	82	278.15	86	433.38	314	311	299	16	44	0	0	2
11878	8030	5262	7	1712	1280	28412	612	112	765	27	220	151	64	264	293.39	144	453.1	468	290	418	57	88	8	30	8
10562	7978	5610	0	2352	1854	28826	486	68	445	13	420	179	32	322	331.94	87	470.71	485	129	332	115	86	4	48	35
14362	7678	4382	5	782	598	27870	653	321	1080	168	66	259	113	160	268.08	270	430.04	472	451	431	17	84	16	2	0
4632	13192	9528	788	444	270	28092	1186	10	637	0	24	61	5	82	277.03	113	431.93	341	314	303	12	44	0	2	3
14456	7556	4356	3	876	594	27874	668	343	1036	159	34	253	139	161	272.13	253	436.13	463	423	426	17	80	23	2	1
11064	7998	5482	1	2106	1638	28684	538	81	580	13	360	141	45	290	320.56	116	462.7	490	192	390	102	79	4	28	15
12518	7766	4978	1	1592	1216	28324	576	167	758	50	232	216	84	237	300.58	150	450.74	482	265	390	65	118	14	22	12
4006	13628	9902	949	328	178	28050	1270	7	563	2	8	59	4	60	271.34	86	423.89	301	263	282	17	40	0	0	2
11544	7804	5282	2	2040	1548	28552	515	113	583	36	294	200	52	286	308.73	112	457.35	472	165	372	100	101	5	36	19
4862	13018	9420	735	502	280	28102	1150	2	672	0	20	66	1	88	277.98	109	434.02	348	336	328	14	37	1	0	2
4574	13216	9542	789	468	284	28104	1211	6	633	2	18	62	3	66	275.35	97	429.37	329	338	306	17	40	0	2	7
6110	12212	8730	515	676	386	28140	1036	17	769	1	26	79	14	109	285.44	110	441.35	395	403	355	16	57	0	0	2
6048	12226	8718	523	686	436	28166	1047	16	724	4	50	79	9	98	287.03	118	441.15	384	365	346	32	53	0	2	4
4632	13220	9522	753	428	270	28100	1174	1	643	0	26	64	1	89	277.41	100	432.3	328	326	321	18	40	0	2	1

SUPPLEMENTARY INFORMATION:Monte Carlo Atomistic Simulation and Machine Learning Analysis of Na-K Eutectic Alloy in Condensed Phases, D. Reitz and E. Blaisten-Barojas, George Mason University, Fairfax, VA 22030

13864	7830	4568	7	946	694	27970	633	281	980	124	68	230	119	171	276.06	245	439.93	497	389	442	19	87	19	0	0
14204	7678	4468	6	864	618	27892	631	319	1034	166	52	269	118	147	271.14	266	434.7	505	418	414	21	88	17	6	3
4216	13418	9764	898	422	238	28076	1262	8	592	1	18	57	5	69	274.92	88	429.24	297	292	288	16	41	0	0	4
5406	12504	9068	609	734	422	28170	1113	10	677	2	34	65	4	97	286	108	441.58	378	364	317	19	40	0	2	3
4162	13586	9828	900	294	164	28042	1271	9	616	1	8	61	7	62	273.23	82	426.7	287	321	296	15	38	0	0	4
13196	7808	4706	1	1312	1000	28164	601	219	846	80	132	192	98	208	283.61	194	447.92	485	304	450	46	93	17	8	2
11918	7712	5124	2	1928	1478	28486	575	128	632	42	306	167	58	267	297.54	138	454.84	494	241	384	78	80	6	20	7
11322	7742	5342	1	2146	1674	28646	517	89	540	19	378	172	47	285	319.6	107	461.59	474	172	375	94	82	7	42	36
14450	7638	4344	5	786	590	27868	654	329	1060	161	58	262	128	155	269.4	271	432.03	480	438	423	16	91	16	2	1
6112	12120	8668	468	782	464	28174	1009	11	781	1	26	83	8	103	286.58	125	442.41	422	387	345	24	61	0	2	3
3896	13686	9938	955	330	198	28066	1292	5	575	0	18	59	3	64	274.35	94	428.69	288	271	271	16	41	0	0	3
4636	13234	9534	769	420	248	28086	1196	5	689	0	14	69	5	80	277.21	105	433.15	326	354	308	16	43	0	0	2
13110	7696	4662	0	1424	1128	28208	587	212	790	64	170	181	113	214	282.09	162	447.89	473	285	468	58	87	18	18	2
5302	12808	9196	653	502	276	28094	1136	12	723	0	10	74	10	92	280.28	103	436.84	334	382	338	21	50	1	0	2
4710	13138	9496	783	466	264	28088	1212	11	674	1	14	59	9	83	274.1	107	429.98	311	345	311	15	38	0	0	2
4158	13486	9760	903	408	254	28082	1277	8	586	2	16	53	4	75	272.7	99	425.38	289	300	282	16	32	0	0	1
4068	13612	9882	940	308	164	28042	1289	9	596	1	8	65	5	57	270.68	101	423.76	291	294	278	14	41	0	0	3
13592	7872	4648	5	1044	774	28010	637	272	957	108	74	225	119	172	277.2	204	442.78	474	374	446	32	99	21	6	5
4032	13586	9860	945	350	214	28064	1294	9	579	1	22	65	7	71	269.88	85	422.68	282	292	268	13	42	0	0	3
3926	13612	9922	955	376	214	28068	1295	8	557	1	18	52	4	66	270.21	87	423.58	286	285	277	16	36	0	0	0
6910	11352	8156	385	1080	674	28250	931	25	714	7	72	95	14	130	286.87	105	445.64	439	367	359	34	56	0	4	5
13400	7902	4748	13	1078	800	28036	624	261	941	121	106	229	106	193	276.35	207	442.06	477	377	433	28	95	18	2	4
4626	13208	9562	801	424	240	28082	1215	6	643	0	20	63	5	78	274.51	96	430.89	323	337	303	12	47	0	2	2
13416	7696	4616	2	1262	986	28138	596	242	830	85	142	202	105	208	282.69	176	447.04	477	313	442	48	90	22	20	4
11540	7830	5304	1	1988	1540	28572	523	117	609	28	336	174	61	276	314.08	138	460.83	477	181	393	98	97	1	28	19
4728	13080	9468	735	538	294	28112	1209	6	645	0	4	62	5	80	278.13	100	432.94	337	358	288	13	38	0	0	3
4700	13144	9490	743	464	278	28100	1162	6	652	0	22	67	3	87	276.97	102	433.15	343	337	318	14	47	0	2	3
4472	13204	9622	798	520	276	28106	1196	6	601	2	12	57	0	84	276.61	90	431.21	338	304	308	11	39	0	0	1
14368	7694	4372	5	780	596	27868	648	340	1069	158	56	259	147	143	267.48	271	429.67	499	443	428	14	88	17	0	2
4238	13478	9750	890	366	220	28068	1263	8	613	2	16	55	2	70	272.37	98	424.64	290	302	294	20	37	0	0	1
11312	7944	5346	2	2050	1590	28582	546	97	566	23	298	159	53	269	304.9	112	457.47	495	182	392	91	91	4	40	19
11604	7934	5316	3	1904	1442	28516	572	106	645	24	292	174	55	274	308.14	132	458.64	481	228	372	80	95	8	22	16
11170	7872	5490	0	2036	1624	28660	484	82	585	13	420	168	36	274	322.18	123	466.98	507	183	371	114	83	3	46	30
5142	12708	9210	635	676	390	28154	1098	6	667	1	28	69	3	95	281.67	97	436.62	384	345	332	17	49	0	0	2
11152	8058	5484	2	2058	1528	28584	557	88	602	22	268	151	42	269	311.91	126	456.57	493	216	388	94	64	5	34	14
14330	7666	4412	9	836	586	27868	642	329	1041	163	38	253	129	160	269.78	248	431.86	473	433	444	16	80	15	0	2

5398	12690	9114	622	562	330	28118	1096	5	743	0	20	77	3	95	279.64	118	436.4	367	376	341	19	51	0	2	2
14328	7730	4418	5	756	570	27860	650	319	1087	156	56	259	125	156	269.22	268	433.1	476	454	438	14	91	16	2	1
13564	7776	4636	3	1128	834	28038	608	250	924	97	92	220	105	190	278.04	210	443.97	485	352	441	36	99	25	6	1
4248	13464	9756	889	382	210	28064	1282	8	616	1	4	53	6	71	272.28	88	424.17	287	321	288	12	36	0	0	2
11174	7868	5484	0	2146	1598	28644	534	88	549	14	336	172	49	270	322	114	462.64	475	176	367	108	84	6	34	30
4744	13040	9492	725	538	282	28114	1172	4	658	0	18	56	1	79	276.75	96	432.44	346	346	322	14	39	0	0	5
4596	13272	9564	807	400	232	28074	1239	5	681	0	10	55	3	77	275.78	106	430.13	300	346	304	16	37	0	0	2
12752	7950	4922	3	1362	1054	28226	591	177	854	56	162	193	84	219	284.71	170	451.66	479	312	438	52	117	16	24	8
3930	13596	9906	956	404	230	28078	1300	6	554	2	8	60	4	69	270.68	88	423.34	284	269	267	16	37	0	4	1
12296	7772	5026	1	1736	1298	28370	585	148	705	42	220	176	69	257	301.55	129	454.41	446	260	411	73	88	20	22	14
11216	8040	5422	1	1964	1568	28588	519	104	631	20	336	158	57	271	312.31	120	461.14	496	214	402	92	87	5	40	20
14270	7588	4376	1	918	692	27932	638	308	1020	155	84	261	114	171	274.26	233	440.4	473	432	417	27	91	9	4	2
5888	12194	8784	584	776	478	28172	1082	31	650	3	44	72	15	103	288.54	92	442.59	385	342	316	22	45	2	6	5
4194	13434	9766	869	438	244	28086	1229	9	561	1	10	61	4	74	275.43	88	430.2	328	273	288	11	38	0	0	2
4736	13130	9480	759	474	266	28096	1217	5	662	2	10	68	2	95	276.12	81	431.26	302	358	295	15	50	0	0	2
4238	13534	9748	892	322	206	28062	1270	5	614	1	12	67	4	72	271.82	99	424.77	290	312	280	14	42	0	2	3
4136	13554	9830	921	328	188	28052	1254	6	600	1	16	65	4	65	272.54	100	426.77	305	286	290	12	46	0	0	4
11414	7880	5330	2	2026	1568	28576	522	111	591	27	322	170	58	283	305.8	111	456.84	514	190	383	82	87	8	36	13
11196	8044	5508	2	2002	1496	28576	530	84	612	10	294	165	46	276	311.89	144	463.53	491	189	382	96	95	7	34	19
11624	7826	5238	0	1996	1534	28554	526	109	598	23	304	172	67	282	315.54	111	460.95	462	192	408	85	88	5	30	26
4280	13472	9740	875	350	206	28062	1244	9	622	1	14	62	8	70	273.54	96	427.47	300	307	304	16	43	0	0	2
4078	13462	9820	891	462	256	28090	1236	5	548	1	12	55	3	68	273.33	80	425.79	322	275	298	16	32	0	0	1
13542	7744	4596	2	1142	900	28068	636	240	939	105	140	207	105	176	277.84	232	445.63	481	366	444	40	81	11	4	3
11778	7960	5270	0	1798	1368	28472	572	114	678	22	270	154	66	252	305.87	141	456.44	499	235	405	77	90	6	28	11
14010	7774	4532	5	880	656	27930	648	292	1055	139	74	234	122	159	274.71	251	438.77	481	436	448	22	76	19	2	1
14426	7616	4276	7	820	672	27894	626	334	1039	166	80	266	128	168	271.62	258	436.19	479	401	434	21	84	21	2	0
7944	10856	7626	295	1028	674	28220	868	52	798	17	88	118	29	165	288.09	122	444.72	410	386	384	36	70	0	2	6
4108	13610	9858	934	292	164	28044	1286	7	604	1	12	60	4	81	271.87	84	425.24	257	294	297	13	41	0	0	3
14324	7726	4418	4	780	568	27858	619	317	1079	161	42	268	120	145	271.01	277	434.25	505	408	440	18	93	16	0	0
14068	7796	4476	7	864	650	27906	630	319	1020	153	50	245	135	156	270.18	266	432.18	487	415	458	20	81	15	2	1
4016	13574	9844	907	388	240	28080	1251	3	578	0	18	63	1	71	271.55	92	425.75	317	284	280	9	39	0	0	4
13440	7910	4616	4	1070	890	28062	592	222	939	112	136	232	76	194	277.22	218	444.09	487	327	446	36	101	15	0	4
4198	13504	9796	924	350	196	28058	1293	8	604	1	14	60	5	70	270.72	91	422.76	286	306	273	12	36	0	0	1
12184	7832	5074	2	1746	1302	28378	554	157	685	40	228	188	85	239	299.79	145	453.52	497	233	408	71	104	9	12	14
6864	11396	8162	375	1084	684	28266	917	22	708	8	72	100	9	156	293.46	121	446.52	451	334	333	22	55	1	4	5
12718	7858	4920	5	1426	1096	28222	607	201	801	71	192	183	87	187	285.67	190	448.97	521	306	420	54	85	13	12	5

13892	7700	4532	9	992	748	27964	624	310	971	145	94	252	122	165	275.4	236	441.03	485	395	427	31	85	16	4	5
12396	7820	4974	0	1662	1272	28362	565	165	697	42	218	191	90	240	301.87	153	454.73	492	234	404	66	98	13	18	15
4750	13078	9416	724	510	338	28134	1142	5	628	0	40	66	2	101	277.83	95	434.41	347	312	322	14	43	1	2	3
4670	13136	9510	771	502	276	28102	1169	8	615	2	8	62	6	83	277.68	81	433.7	337	311	326	15	41	0	0	4
4744	12998	9460	730	574	322	28126	1169	5	652	0	28	54	3	97	277.09	94	433.49	319	328	332	21	34	0	0	2
4572	13160	9566	769	528	280	28114	1210	6	620	0	8	59	4	68	278.34	85	433.57	341	339	297	19	39	0	0	2
14254	7702	4438	8	808	610	27886	667	327	1054	165	70	255	129	148	271.32	266	434.31	480	455	425	18	78	16	4	2
5358	12540	9082	603	738	424	28168	1082	17	656	6	22	83	8	113	283.75	94	439.39	383	351	313	17	49	0	4	2
4094	13484	9810	892	428	250	28084	1262	7	582	2	18	53	4	69	273.7	88	426.64	300	294	289	18	33	0	0	2
3960	13644	9930	957	320	176	28044	1297	8	590	1	14	57	4	68	270.28	86	423.4	276	283	277	14	40	0	0	2
4318	13280	9662	806	526	310	28118	1208	0	576	0	20	62	0	93	277.72	68	432.52	309	301	307	17	39	0	2	1
3970	13646	9876	947	348	218	28072	1300	6	566	0	14	65	2	72	272.66	87	427.05	283	274	258	13	42	0	0	3
14102	7756	4506	8	842	626	27906	668	282	1075	142	72	239	108	161	273.74	247	436.17	459	456	443	18	83	12	2	4
4390	13394	9680	864	366	218	28066	1229	7	617	1	16	58	3	65	274.89	93	428.23	313	301	307	17	41	0	2	4
5226	12708	9180	617	640	366	28142	1090	9	676	1	22	65	5	100	284.37	102	438.05	375	344	343	19	37	0	0	1
11830	7842	5260	4	1882	1356	28428	558	129	656	31	238	167	71	266	298.28	146	453.32	488	216	398	71	79	8	20	12
4082	13526	9836	911	400	222	28076	1266	5	566	1	10	58	4	78	273.35	72	427.27	284	289	295	15	43	0	0	2
13586	7512	4544	2	1356	994	28138	607	267	773	96	140	210	117	201	283.15	185	449.45	481	303	431	46	89	20	4	6
14506	7650	4312	8	752	580	27856	640	331	1072	169	50	272	121	153	272.43	267	435.67	477	445	432	20	93	19	6	1
4960	12944	9340	711	528	314	28112	1146	9	672	2	24	72	5	96	280.59	107	435.87	344	334	318	16	42	0	2	2
5038	12826	9254	688	618	380	28150	1143	6	638	0	34	49	5	104	281.54	85	438.03	336	331	344	20	30	0	0	0
4848	13054	9428	719	484	272	28102	1134	8	654	1	12	67	4	93	276.25	102	433.91	347	339	338	15	39	0	4	2
11582	8066	5268	2	1796	1466	28516	566	106	648	16	296	167	64	264	302.4	120	455.96	459	226	400	94	94	4	40	18
13510	7802	4602	2	1158	886	28062	604	243	935	117	98	228	95	191	276.11	229	443.46	500	357	431	32	88	10	6	2
5312	12514	9082	643	766	458	28174	1116	12	619	2	42	72	8	104	282.66	78	439.26	360	328	315	25	45	0	0	2
6428	11798	8466	406	902	558	28218	958	17	748	1	66	72	10	120	288.28	116	443.8	441	399	363	21	44	1	0	8
12952	8078	4934	4	1182	864	28122	617	178	956	54	106	190	97	184	282.97	205	446.78	504	365	446	40	107	9	6	2
4466	13148	9580	787	574	334	28128	1193	6	593	0	24	68	5	95	278.67	92	434.39	327	301	291	17	43	0	2	2
4956	13020	9338	703	484	292	28102	1148	3	683	2	12	74	0	93	280.92	96	437.72	334	346	323	18	51	0	0	3
14292	7690	4400	9	818	616	27876	653	329	1052	170	58	272	119	157	270.98	263	435.53	478	445	413	17	89	21	2	3
4044	13566	9842	916	374	230	28078	1265	6	585	2	22	56	4	65	274.73	92	428.5	312	293	281	13	28	0	0	2
12244	7992	4982	0	1598	1304	28366	594	147	715	42	218	165	76	225	292.72	155	449.35	495	278	419	68	79	9	26	10
4560	13266	9562	815	418	256	28078	1191	10	619	1	14	71	7	89	273.78	96	429.86	309	297	313	20	51	0	2	1
4678	13100	9484	789	518	308	28114	1206	8	641	0	26	60	5	93	279.47	91	435.18	312	331	303	17	36	0	0	1
13146	7894	4666	8	1260	1036	28154	614	200	850	86	134	195	87	208	281.62	195	446.5	483	308	442	40	85	12	14	3
10892	7928	5700	1	2194	1578	28690	548	67	549	11	366	157	35	296	319.1	86	467.51	440	195	365	111	86	5	30	32

SUPPLEMENTARY INFORMATION:Monte Carlo Atomistic Simulation and Machine Learning Analysis of Na-K Eutectic Alloy in Condensed Phases, D. Reitz and E. Blaisten-Barojas, George Mason University, Fairfax, VA 22030

11340	8122	5396	0	1868	1480	28538	518	93	655	21	294	184	47	276	315.79	122	460.71	481	218	395	86	102	6	36	23
4048	13598	9884	956	330	178	28048	1302	10	589	1	10	60	4	62	270.21	90	422.36	291	281	267	10	49	0	0	2
5220	12808	9206	646	542	320	28120	1124	8	718	0	22	56	7	111	280.62	96	438.45	332	374	353	15	33	0	2	2
10436	7866	5664	0	2436	1938	28878	493	64	441	9	476	155	32	298	331.57	86	472.78	478	135	339	147	75	6	58	32
13158	7950	4782	2	1166	922	28126	624	202	941	67	124	191	106	186	284.11	205	445.53	489	364	451	39	97	12	22	6
4576	13164	9530	795	520	308	28118	1197	1	626	0	20	59	0	75	274.73	94	429.69	327	314	315	26	40	0	0	0
4678	13248	9558	788	370	196	28056	1211	12	678	0	6	54	7	92	275.26	99	429.86	297	349	324	10	36	0	0	2
4894	12890	9340	693	624	370	28148	1165	3	663	0	30	70	2	78	280.64	94	436.64	361	357	302	16	46	0	0	7
14318	7676	4420	5	822	588	27872	654	335	1044	158	44	260	134	150	269.97	264	432.15	490	434	425	15	91	21	4	0
11970	7694	5140	3	1912	1444	28482	545	135	624	17	300	160	74	267	312.01	125	459.31	464	210	409	86	83	16	18	21
4906	12964	9328	675	550	354	28138	1113	3	679	0	34	59	3	90	277.36	115	435.11	375	329	343	15	36	0	2	2
13192	7914	4800	5	1184	888	28110	646	241	900	83	124	200	111	192	284.23	210	446	496	373	418	29	87	20	8	3
4806	13082	9406	747	498	304	28112	1172	6	672	1	16	64	4	73	278.75	113	434.27	357	340	308	17	41	0	0	3
10950	7864	5620	1	2160	1654	28714	503	71	538	14	414	191	27	293	327.66	101	469.1	469	168	327	131	87	5	44	26
14230	7764	4484	5	778	562	27868	627	323	1070	156	44	247	126	151	271.55	274	435.64	482	423	462	21	77	21	6	1
14470	7706	4346	4	742	552	27852	629	323	1106	162	32	260	122	156	267.68	291	428.95	484	431	452	13	87	20	4	1
14136	7718	4420	5	890	686	27920	635	313	1009	155	66	242	117	164	271.56	242	434.43	473	414	454	22	76	22	4	3
13932	7752	4520	8	956	710	27940	644	297	995	138	64	239	120	174	273.96	222	438.56	453	402	448	27	86	17	6	3
6114	12024	8652	467	842	516	28210	1024	11	721	1	62	64	8	105	287.19	116	441.08	423	380	350	25	37	0	0	0
3956	13638	9920	964	344	188	28054	1308	8	568	0	8	56	5	72	270.78	88	423.71	273	282	268	12	41	0	0	2
6738	11750	8324	425	824	508	28186	990	22	790	4	40	73	15	133	287.26	124	442.84	390	400	376	23	52	0	2	4
5638	12528	9018	576	594	318	28112	1081	20	753	4	14	62	12	89	280.93	104	439.54	378	403	356	22	39	0	2	1
4620	13172	9554	792	464	260	28092	1183	9	623	0	22	48	4	89	276.96	88	432.24	321	304	334	14	34	0	0	2
5060	12896	9264	673	550	334	28124	1126	8	667	2	20	72	3	101	279.39	114	436.92	346	341	329	20	46	0	0	1
4708	13160	9508	750	452	252	28094	1190	5	675	0	14	57	5	76	276.79	102	433.16	340	368	318	14	34	0	0	1
11148	7770	5504	2	2218	1630	28670	521	73	532	17	374	166	38	284	321.56	100	462.79	502	171	367	100	81	2	24	18
6952	11576	8188	366	878	554	28204	938	26	817	5	56	80	17	118	290.22	133	443.99	444	411	378	23	54	0	0	6
4670	13122	9476	762	498	316	28118	1185	5	644	0	36	58	2	85	275.89	107	431.27	333	321	310	19	40	0	0	2
10508	7856	5800	1	2350	1794	28832	465	68	493	12	444	158	31	311	326.58	85	466.71	505	149	367	115	88	4	68	31
4198	13552	9820	902	306	160	28042	1247	8	618	1	6	69	6	65	274.07	99	427.53	308	293	289	15	50	0	0	1
11568	8008	5440	0	1874	1322	28470	528	97	685	23	236	177	52	253	307.36	147	458.76	509	231	399	81	104	3	20	16
11376	8048	5426	1	1974	1432	28524	555	95	638	22	254	165	49	285	309.1	109	458.66	484	227	387	74	84	4	14	15
13386	7822	4716	3	1176	858	28064	601	241	932	103	102	219	106	178	277.64	228	444.24	511	344	447	31	95	14	4	4
4256	13580	9778	908	258	156	28038	1267	6	623	1	8	65	4	75	272.15	90	425.19	274	308	296	17	45	0	2	1
12302	7892	5142	1	1596	1152	28298	551	171	751	45	196	180	87	230	292.17	171	451.71	495	258	430	61	99	7	18	15
11852	7974	5184	1	1756	1380	28436	587	122	680	34	268	172	64	234	296.59	139	454.17	482	252	384	89	84	4	20	14

SUPPLEMENTARY INFORMATION:Monte Carlo Atomistic Simulation and Machine Learning Analysis of Na-K Eutectic Alloy in Condensed Phases, D. Reitz and E. Blaisten-Barojas, George Mason University, Fairfax, VA 22030

4878	12828	9336	701	678	400	28154	1136	8	638	0	32	68	4	110	279.77	96	434.11	350	330	315	14	45	0	0	2
14388	7534	4354	9	896	650	27894	600	337	1001	174	66	257	121	170	272.72	257	437.05	489	387	455	19	74	15	6	1
4522	13166	9584	796	526	292	28110	1218	9	626	0	20	65	5	81	276.23	86	431.29	301	316	302	25	49	1	0	4
3904	13668	9922	978	348	210	28066	1302	4	568	1	12	56	2	74	269.94	88	423	273	263	275	12	39	0	2	3
4044	13506	9860	904	424	232	28084	1251	4	565	0	18	58	2	74	274.37	95	428.8	302	274	296	16	36	0	0	1
12828	7944	4898	2	1310	1018	28180	596	189	867	64	178	202	100	210	284.97	184	447.91	487	309	432	50	103	8	4	4
12704	7818	4866	2	1490	1180	28290	585	182	776	55	212	170	88	209	292.15	177	448.24	501	292	440	66	84	14	18	6
13828	7656	4428	3	1104	904	28056	616	267	905	110	118	220	113	191	277.5	212	445.79	477	348	448	31	91	21	16	5
14022	7656	4456	4	970	752	27962	646	297	996	158	98	247	104	169	273.5	236	438.6	486	407	418	23	76	18	8	2
12644	8050	4916	1	1364	1078	28218	607	174	845	56	152	177	85	204	283.06	198	447.92	495	301	446	49	97	16	14	5
4256	13494	9760	891	340	196	28058	1244	5	607	1	12	64	3	72	275.27	90	429.78	294	297	301	15	47	0	0	5
14086	7738	4418	4	890	720	27940	665	314	1002	154	72	237	123	159	272.45	242	435.97	476	416	432	23	75	21	16	1
14450	7600	4334	2	824	612	27882	666	334	1058	161	56	256	134	145	269.35	260	432.23	504	474	404	12	88	14	6	1
4864	12934	9392	712	588	328	28128	1139	6	612	0	22	66	3	81	280.01	91	435.27	368	323	319	17	43	0	0	4
11018	8020	5448	0	2096	1690	28686	544	70	566	15	356	173	36	274	315.06	104	462.33	465	192	365	116	86	6	56	25
4502	13212	9562	780	518	310	28122	1186	7	588	0	16	58	5	82	276.6	85	433.45	339	314	307	21	31	0	2	0
4288	13368	9706	856	460	260	28092	1227	3	572	0	10	58	2	68	274.84	89	429.37	317	288	299	21	36	0	0	3
13160	7900	4830	3	1224	876	28096	621	220	941	72	96	204	109	178	282.22	203	446	475	352	458	48	104	14	8	5
5784	12318	8842	501	746	450	28174	1023	11	703	2	30	71	5	104	284.08	93	440.61	419	385	352	20	41	0	4	2
10990	7820	5552	1	2252	1686	28726	526	77	506	14	380	156	41	264	322.78	85	466.13	483	164	369	132	91	7	44	28
11280	8058	5480	1	1926	1472	28566	517	83	637	10	322	184	47	277	310.25	130	459.92	500	207	376	76	106	4	26	28
6948	11506	8188	342	940	576	28212	894	13	838	1	50	81	8	126	285.35	143	445.1	454	392	393	33	49	0	4	3
4014	13582	9868	928	378	216	28068	1302	8	568	1	10	48	3	69	273.59	93	426.15	282	298	273	16	32	0	0	0
11308	7852	5414	0	2116	1570	28606	516	85	597	17	310	160	47	287	315.43	119	461.69	488	197	381	95	84	2	34	23
4236	13460	9752	900	390	222	28068	1280	12	597	2	8	70	7	91	274.4	82	429.4	267	306	272	13	43	0	0	2
6182	12076	8648	493	740	464	28174	1023	17	742	3	62	69	10	117	284.1	114	442.24	390	388	364	26	46	0	2	1
11398	7852	5366	0	2098	1548	28582	506	95	582	14	288	159	50	264	311.04	114	461.06	505	181	399	93	87	11	28	27
11622	7984	5224	3	1890	1494	28528	587	97	636	16	286	156	65	219	303.81	146	455.95	559	244	362	79	89	5	24	12
4136	13506	9814	915	386	216	28070	1266	7	571	1	10	55	5	66	274.03	89	428.08	289	288	298	19	43	0	2	3
12040	7902	5132	2	1746	1332	28416	559	139	701	37	240	184	75	249	300.96	158	452.87	521	241	383	70	98	7	22	5
5100	12786	9224	663	638	378	28148	1125	8	634	2	22	61	5	99	281.99	79	439.03	353	340	333	22	34	0	0	1
13862	7704	4546	4	1020	750	27972	631	286	979	132	86	242	108	184	275.05	219	440.96	467	385	439	24	98	21	4	4
4752	13032	9454	752	556	308	28116	1191	7	665	0	14	71	5	84	276.76	108	432.22	334	337	294	19	47	0	0	2
7632	11056	7770	308	1006	678	28234	908	43	798	10	80	106	21	148	288.95	122	446.76	413	407	360	36	72	3	8	11
6566	11830	8442	436	782	492	28182	990	27	783	6	68	94	15	118	286.74	119	442.86	412	413	348	25	59	0	0	5
13016	7876	4802	1	1296	1014	28182	617	169	892	45	168	181	88	199	286.42	197	450.23	474	325	458	49	99	17	10	7

SUPPLEMENTARY INFORMATION:Monte Carlo Atomistic Simulation and Machine Learning Analysis of Na-K Eutectic Alloy in Condensed Phases, D. Reitz and E. Blaisten-Barojas, George Mason University, Fairfax, VA 22030

13236	7758	4680	5	1332	1002	28138	604	229	812	70	120	206	116	221	283.01	165	447.57	448	300	447	48	108	20	10	5
4990	13006	9372	738	452	250	28086	1200	4	709	0	16	62	2	86	275.81	107	432.34	315	372	318	13	44	0	0	3
10928	8014	5602	0	2092	1632	28710	495	80	535	17	396	178	44	300	325.7	102	466.87	476	150	377	108	86	2	42	24
14224	7716	4392	5	850	656	27898	628	325	1017	163	54	265	117	139	270.45	269	433.27	518	381	424	20	92	25	6	2
11608	7854	5310	0	1940	1480	28538	516	121	626	32	314	185	59	276	310.83	132	460.41	470	190	385	100	104	4	28	19
4156	13586	9832	922	304	164	28046	1262	7	613	1	4	57	5	73	272.22	91	424.35	290	288	302	10	40	0	0	2
13596	7628	4614	0	1212	890	28076	602	271	845	124	126	222	109	192	278.68	212	445.86	477	314	443	42	76	12	10	6
11366	7782	5338	1	2136	1644	28656	543	97	541	17	334	161	49	271	313.58	108	462.68	483	182	374	103	79	7	54	24
5010	12996	9346	708	466	260	28086	1147	8	688	1	8	70	6	91	277.68	101	434.5	327	344	342	19	48	0	0	2
6314	12020	8562	450	750	480	28186	1014	16	784	5	52	79	6	114	284.16	125	441.86	421	427	348	18	54	1	8	1
5106	12870	9296	675	532	286	28102	1161	11	707	1	12	58	7	89	279.31	97	436.16	343	383	326	13	40	0	0	2
11088	8054	5600	0	2008	1488	28592	538	92	594	26	324	171	47	275	310.35	108	458.72	483	209	382	90	89	1	24	26
12182	7794	4982	0	1796	1404	28428	578	144	649	43	250	165	66	240	301.58	129	454.3	455	240	419	100	76	10	18	7
5084	12830	9266	648	578	342	28132	1119	7	692	1	32	66	4	96	277.95	118	433.11	367	358	327	18	42	0	0	0
13750	7720	4568	6	1052	808	28022	628	257	928	101	114	230	106	194	278.03	190	444.25	459	369	433	37	106	21	10	2
11580	8206	5412	1	1680	1284	28440	532	93	730	14	262	191	57	256	303.73	153	455.93	505	222	396	81	114	6	12	11
4492	13320	9622	831	408	228	28076	1226	8	631	1	6	65	5	74	274.75	97	429.25	316	318	298	16	41	0	0	1
4484	13310	9612	849	410	246	28080	1225	8	626	1	18	55	6	84	275.08	110	429.76	304	296	308	14	38	0	0	2
14472	7660	4362	5	748	552	27846	633	327	1096	172	52	273	117	161	269.88	264	432.03	472	448	435	15	93	17	0	3
4610	13204	9582	788	442	232	28084	1193	6	651	0	14	54	6	78	276.72	106	432.92	328	323	323	16	34	0	0	1
14192	7694	4418	10	878	660	27906	669	312	1028	159	62	262	118	155	270.5	249	432.97	488	444	397	17	91	17	2	2
4196	13508	9768	896	376	220	28076	1278	2	611	1	8	56	1	71	273.54	90	428.21	291	301	284	13	38	0	0	3
5132	12706	9218	628	670	394	28160	1090	3	654	0	40	54	2	101	282.05	92	437.69	372	337	354	20	35	0	0	1
7482	11124	7860	343	992	670	28234	903	39	764	11	100	100	18	154	288.85	113	444.82	409	376	380	41	62	1	4	2
4078	13560	9846	931	362	204	28060	1259	4	579	1	10	57	3	82	271.6	100	424.25	287	262	299	7	41	0	0	4
4058	13496	9830	919	442	248	28084	1273	4	556	1	10	57	0	87	273.03	71	428.36	277	278	284	15	36	0	0	1
13748	7890	4664	6	930	662	27958	610	272	999	120	60	226	128	167	276.27	250	441.07	487	370	472	28	87	12	4	1
14248	7606	4340	7	924	724	27930	657	327	986	164	88	253	119	157	271.29	244	433.97	488	430	415	22	76	20	0	1
5042	12896	9306	678	544	310	28118	1136	9	694	1	20	67	6	82	280.75	97	435.25	361	373	327	18	47	0	0	4
3928	13636	9928	969	362	198	28060	1302	6	567	2	8	70	3	82	270.88	78	423.67	264	271	268	9	49	0	0	4
4474	13326	9612	800	420	250	28096	1208	4	642	1	14	53	2	83	279.37	95	436.02	325	326	312	11	36	0	0	2
14038	7772	4488	4	864	682	27936	661	296	1058	145	80	245	121	160	275.7	251	439.98	467	448	429	28	86	12	12	2
7706	11064	7786	288	976	614	28214	865	44	834	15	60	104	24	150	289.43	131	446.3	444	407	394	30	54	1	8	3
13794	7942	4640	7	856	636	27930	640	266	1083	117	60	237	112	159	275.59	258	439.03	480	418	442	28	101	15	2	1
11992	7836	5134	0	1786	1398	28466	567	111	707	27	294	183	65	250	306.42	143	458.02	476	236	405	77	106	9	24	19
14448	7690	4392	4	740	532	27846	638	336	1086	151	44	256	146	155	271.12	265	435.84	460	430	464	21	97	21	0	1

SUPPLEMENTARY INFORMATION:Monte Carlo Atomistic Simulation and Machine Learning Analysis of Na-K Eutectic Alloy in Condensed Phases, D. Reitz and E. Blaisten-Barojas, George Mason University, Fairfax, VA 22030

4176	13542	9786	912	344	204	28060	1273	7	597	2	8	60	4	73	272.75	94	427.06	287	294	281	13	39	1	0	4
5448	12566	9002	590	692	432	28168	1094	5	676	0	28	61	4	101	282.89	102	438.95	384	353	334	17	43	0	0	2
13102	7832	4820	4	1268	954	28136	617	215	876	84	146	212	96	213	282.03	192	446.29	476	348	422	39	90	10	12	4
13882	7744	4510	8	988	752	27960	633	284	988	140	82	239	103	177	274.43	227	439.3	467	397	444	29	80	14	2	1
4114	13540	9826	890	370	208	28068	1245	5	586	0	10	56	3	71	273.97	92	428.56	304	295	303	12	33	0	0	4
12442	7866	5008	1	1590	1188	28306	603	162	765	47	204	166	75	228	290.44	172	452.14	482	274	426	63	82	17	8	5
13842	7730	4556	6	1028	748	27986	650	272	990	124	76	232	114	180	276.2	236	440.87	455	395	439	32	91	17	6	2
12308	7774	5006	2	1748	1304	28374	608	152	703	45	220	176	77	218	292.57	137	449.26	499	259	404	67	87	12	12	10
12622	7768	4892	3	1608	1214	28314	645	189	723	51	190	165	100	219	287.74	146	449.35	478	297	418	50	82	22	20	8
14434	7594	4300	5	828	654	27890	630	334	1045	161	74	252	132	151	268.1	293	430.23	505	416	438	15	78	22	4	1
12022	7860	5132	0	1800	1362	28456	604	127	656	28	268	170	64	252	300.88	119	454.37	479	252	381	68	93	10	12	16
12296	7928	4984	1	1644	1280	28348	571	161	713	48	192	168	77	219	293.91	165	450.65	499	247	425	74	86	15	20	10
4568	13184	9558	783	500	284	28112	1186	6	628	0	18	63	2	85	276.45	104	432.67	342	300	305	11	42	0	0	2
5254	12766	9196	678	554	320	28114	1160	13	683	5	20	69	5	86	280.07	100	438.34	346	361	314	17	45	0	4	3
4424	13250	9644	821	488	272	28098	1233	6	633	1	20	56	4	87	277.17	78	432.45	289	325	308	18	34	0	0	4
5014	12980	9358	711	468	254	28086	1158	11	706	0	12	61	9	74	276.36	113	431.52	353	358	332	14	43	0	0	2
4674	13080	9464	726	566	332	28132	1192	7	612	0	16	63	4	87	280.29	86	434.92	335	334	296	20	42	0	0	1
13882	7830	4580	7	916	670	27944	646	273	1010	125	62	221	113	150	275.63	232	438.58	486	423	460	24	79	16	4	4
14392	7582	4386	8	852	606	27880	646	328	1044	153	56	257	132	165	270.8	249	433.01	462	426	442	19	93	21	6	2
12442	7816	4984	2	1644	1234	28342	617	172	741	53	200	171	77	220	293.58	155	451.25	487	289	401	69	91	13	20	8
4110	13512	9814	898	402	228	28076	1265	6	579	1	10	58	4	72	271.9	77	426.71	293	292	293	14	36	0	0	3
4056	13606	9840	925	350	206	28060	1281	6	571	1	2	62	3	72	272.91	92	425.81	285	285	276	15	42	0	0	2
13886	7732	4532	5	986	738	27958	633	291	983	128	74	235	122	166	275.88	237	440.46	467	401	451	34	88	21	10	3
4260	13396	9728	876	438	250	28086	1262	4	608	0	14	63	4	73	274.62	105	428.04	300	298	286	12	44	0	0	4
11700	7722	5176	3	2010	1580	28558	519	112	564	28	330	179	48	259	313.1	105	459.69	480	180	373	100	92	4	38	29
13852	7762	4578	8	990	712	27970	656	286	997	135	74	233	117	169	275.58	218	441.34	471	413	432	27	90	17	2	2
11244	7890	5482	1	2050	1556	28614	525	95	578	19	350	157	52	268	321.38	133	462.38	492	176	383	110	80	4	42	20
12526	8102	5034	4	1352	1038	28222	633	169	861	62	154	178	85	178	282.83	199	444.68	515	334	428	53	88	9	16	3
11418	8056	5396	0	1868	1452	28526	579	94	662	20	312	167	39	280	311.88	113	458.42	457	235	367	86	85	10	22	20
4766	13166	9456	765	428	258	28086	1202	10	676	2	12	66	4	82	274.72	112	429.1	336	349	292	8	42	0	0	4
4648	13148	9504	784	504	288	28100	1167	7	637	0	8	61	2	82	278	112	436.08	340	298	323	18	40	0	0	2
4750	13172	9490	775	434	236	28084	1184	7	663	0	2	67	5	77	276.96	116	433.41	336	314	314	18	47	0	0	0
12348	7844	4990	6	1672	1272	28350	525	166	716	43	202	189	97	226	299.5	173	453.74	506	234	432	73	99	8	20	18
11892	7930	5300	2	1726	1280	28418	538	119	729	24	266	173	67	251	301	133	455.45	482	253	419	87	108	4	22	11
4098	13576	9860	943	310	180	28048	1291	11	598	1	24	56	7	59	271.08	88	424.52	279	298	286	18	43	0	0	4
13106	7840	4780	2	1308	980	28156	607	209	873	57	128	203	105	199	287.17	196	451.56	477	305	432	48	112	19	14	10

SUPPLEMENTARY INFORMATION:Monte Carlo Atomistic Simulation and Machine Learning Analysis of Na-K Eutectic Alloy in Condensed Phases, D. Reitz and E. Blaisten-Barojas, George Mason University, Fairfax, VA 22030

4144	13510	9808	920	382	214	28068	1297	6	622	1	10	59	5	76	272.18	90	425.7	273	311	276	11	38	0	0	4
4152	13470	9776	859	432	248	28086	1242	7	577	1	8	58	3	82	272.56	85	426.97	306	299	294	11	43	0	0	2
14236	7666	4422	7	874	640	27898	647	315	1037	166	56	260	114	148	267.8	266	429.49	505	420	417	15	86	12	2	2
12120	7958	5098	1	1684	1286	28378	573	137	727	47	208	181	62	237	303.12	149	453.53	495	264	393	71	84	10	22	15
11552	7828	5260	1	2016	1564	28572	554	105	621	29	308	180	50	260	306.78	114	457.54	481	221	370	96	103	6	44	24
4728	12998	9446	716	596	344	28140	1153	3	626	0	28	71	3	98	280.17	92	435.86	340	332	312	18	42	0	0	4
7026	11338	8070	359	1068	682	28254	917	35	697	8	66	102	19	155	288.03	110	445.35	411	342	361	41	65	1	4	2
4480	13284	9638	827	436	232	28078	1224	4	638	0	8	64	3	72	276.29	92	428.92	317	325	302	16	45	0	0	2
6144	12006	8658	474	840	496	28198	992	17	740	5	54	79	12	111	284.87	134	443.28	429	371	354	25	54	0	0	2
4310	13396	9706	871	406	242	28080	1245	7	619	0	18	64	6	78	274.06	91	427.75	296	307	292	17	50	0	2	3
4168	13468	9786	886	396	232	28070	1243	8	580	1	20	54	4	71	272.35	90	426.24	308	292	295	16	32	0	0	2
14118	7730	4454	8	842	668	27900	613	311	1054	157	78	267	116	173	270.82	267	433.13	479	404	437	22	97	21	8	1
12662	7780	4918	0	1534	1164	28282	561	184	762	56	216	190	89	231	291.59	160	448.73	502	262	430	59	94	7	8	5
12102	7964	5146	2	1652	1264	28382	590	129	745	35	230	171	62	213	298.31	155	456.28	480	266	395	93	88	7	22	16
5800	12382	8838	547	664	426	28154	1052	18	730	3	42	65	15	118	282.45	119	441.13	381	365	364	14	42	0	2	1
4830	13066	9416	719	484	290	28110	1135	4	675	0	24	59	3	87	277.75	113	434.14	352	330	343	16	35	0	0	3
11704	7984	5178	2	1882	1476	28506	580	121	618	36	244	177	55	261	305.24	121	460.36	464	220	375	91	78	7	34	17
11344	7892	5438	2	2018	1532	28600	542	101	596	17	332	169	43	290	316.81	94	463.43	454	215	370	111	91	9	42	16
4340	13332	9678	831	466	264	28090	1205	7	595	1	8	71	4	92	273.77	82	428.83	311	295	298	15	49	0	2	2
4958	13062	9370	735	430	252	28084	1169	5	728	0	12	63	4	79	274.83	117	430.75	343	365	328	14	48	0	0	1
12614	7864	4936	3	1492	1162	28294	569	184	762	56	200	178	91	205	291.26	164	451.07	518	267	440	56	96	17	24	10
12444	7860	4990	0	1610	1212	28330	585	152	739	41	196	176	76	234	294.92	143	451.32	480	278	422	66	86	8	14	9
6818	11554	8272	377	958	566	28218	970	24	787	7	48	73	15	118	286.09	121	444.7	434	409	364	22	51	0	2	7
4452	13284	9652	842	440	236	28076	1243	9	620	1	12	64	6	74	275.33	80	428.97	305	318	291	15	44	0	0	3
13702	7736	4652	1	1098	748	28008	635	278	950	120	64	218	121	176	276.24	222	442.84	468	382	445	37	85	16	8	2
12278	7836	5084	0	1630	1268	28392	562	149	722	34	270	165	80	243	298.33	154	452.06	503	244	421	63	97	10	24	14
4828	13074	9418	720	472	288	28108	1201	4	690	0	28	59	3	74	278.12	105	434.15	335	375	304	19	37	0	0	2
14376	7688	4378	6	788	588	27870	641	316	1086	150	48	251	121	155	271.75	259	434.49	473	449	454	19	93	24	4	1
12646	7838	4940	0	1526	1140	28294	605	164	760	46	194	170	85	231	292.09	165	451.57	487	286	416	56	83	13	8	6
11146	7856	5404	2	2138	1692	28650	517	90	571	14	352	179	46	305	313.99	110	461.29	471	182	358	89	92	6	60	26
4552	13208	9592	806	472	258	28100	1193	2	641	0	16	55	2	76	277.56	113	432.55	325	318	323	20	39	0	2	3
4436	13284	9634	816	468	262	28094	1193	9	604	0	8	62	5	90	275.91	113	430.37	324	295	312	7	43	0	2	6
14440	7596	4332	7	836	616	27878	646	343	1038	178	58	277	123	147	269.45	280	431.9	505	419	400	18	86	21	0	0
10948	7822	5594	1	2300	1672	28738	542	80	492	13	358	154	39	268	322.7	104	464.78	487	152	360	124	70	8	40	25
4406	13364	9650	840	418	240	28082	1232	13	621	2	4	62	9	75	274.94	105	429.79	322	326	291	12	36	0	0	1
7400	11242	7904	377	956	636	28218	943	39	744	12	80	107	21	150	289.31	104	446.06	398	391	356	31	65	1	0	6

SUPPLEMENTARY INFORMATION:Monte Carlo Atomistic Simulation and Machine Learning Analysis of Na-K Eutectic Alloy in Condensed Phases, D. Reitz and E. Blaisten-Barojas, George Mason University, Fairfax, VA 22030

12106	8156	5126	5	1514	1200	28312	651	119	836	42	186	176	59	221	288.13	169	450.31	464	309	409	57	96	6	24	9
12184	7912	5086	1	1624	1290	28382	577	162	674	40	258	180	81	242	301.86	141	457.37	472	232	401	78	95	15	26	14
6592	11658	8348	456	934	608	28226	987	28	688	6	80	92	19	114	288.14	109	444.06	419	347	338	31	57	0	6	7
4206	13480	9782	908	372	210	28064	1257	8	593	2	14	65	4	72	270.61	96	423.37	304	278	284	13	43	0	0	1
14098	7674	4492	3	884	672	27922	612	322	1007	157	94	264	129	172	273.34	242	436.81	484	391	434	24	86	17	6	2
5958	12192	8742	483	802	470	28188	995	9	716	0	22	63	8	103	282.48	120	442.7	432	361	378	20	40	0	2	3
6710	11660	8324	378	902	560	28220	982	18	802	2	58	70	12	135	287.98	130	445.17	406	423	366	23	49	0	4	5
5590	12616	9034	604	536	306	28102	1084	17	719	3	20	73	11	95	282.82	111	438.89	377	380	349	16	47	1	0	0
13820	7782	4482	3	1028	806	28004	667	257	976	134	84	243	95	193	273.86	209	440.67	456	415	412	21	91	15	2	3
3968	13680	9900	931	314	186	28054	1278	6	582	1	6	59	3	63	270.76	99	424.36	293	291	281	14	32	0	0	4
4182	13546	9798	890	326	192	28058	1262	7	622	1	14	65	4	73	272.43	87	425.22	301	308	285	8	43	0	0	3
12924	8002	4848	4	1276	974	28154	608	186	883	69	124	211	85	201	281.23	192	446.88	507	324	416	39	108	16	6	3
4556	13192	9568	771	490	278	28102	1194	5	626	1	18	61	3	87	277.62	74	434.62	318	332	319	14	39	0	0	2
14302	7710	4342	3	812	660	27900	633	312	1061	154	68	265	121	155	272.12	263	435.6	502	440	422	16	95	18	6	2
4812	13164	9446	744	402	242	28080	1170	5	692	0	14	51	2	79	276.33	110	430.15	338	358	340	13	33	0	0	2
5594	12474	8964	587	670	408	28148	1099	18	720	4	38	68	6	99	282.35	116	439.13	386	385	319	18	42	0	0	0
4766	13052	9454	757	514	296	28106	1196	6	644	0	24	77	5	75	277.89	101	434.55	337	338	290	22	57	1	0	1
14454	7618	4332	6	802	606	27878	654	329	1054	156	64	251	129	148	268.38	267	429.73	491	437	432	15	89	20	2	1
3996	13592	9888	957	364	208	28064	1287	6	570	2	16	56	3	64	272.12	81	424.73	288	275	285	14	43	0	0	3
5402	12570	9098	590	672	378	28150	1096	4	689	0	30	58	1	97	285.59	103	442.17	385	385	335	16	34	0	0	2
5376	12518	9042	594	738	458	28180	1096	12	649	3	48	66	6	109	281.33	87	437.12	380	365	326	17	42	0	0	1
4608	13020	9484	749	640	370	28144	1183	7	591	0	20	56	6	86	276.95	84	432.21	343	307	308	13	35	0	2	5
4236	13382	9728	876	452	268	28088	1231	6	593	1	22	61	2	95	273.02	96	426.2	294	266	292	12	45	0	0	4
13852	7850	4554	4	896	710	27956	626	274	1034	121	90	230	111	176	275.5	240	440.62	477	406	454	23	96	18	2	2
4664	13210	9484	780	442	282	28098	1223	6	645	0	14	59	4	85	274.89	90	429.85	314	358	298	12	36	0	0	3
5306	12652	9158	625	652	354	28138	1109	8	703	0	16	65	6	114	280.28	100	437.07	344	357	345	16	50	0	0	2
4494	13196	9584	796	516	304	28122	1216	3	622	0	26	58	3	80	278.13	95	432.35	328	331	300	15	36	0	2	2
11464	7912	5494	0	1986	1382	28530	532	102	619	16	266	167	58	277	311.06	112	456.89	494	205	394	79	94	7	24	21
14468	7686	4376	3	738	530	27834	639	354	1083	168	36	255	145	152	267.38	276	428.7	475	456	455	13	78	17	0	2
11282	7832	5272	3	2146	1726	28662	520	100	518	21	356	159	52	266	318.35	106	459.9	502	174	375	108	77	7	46	28
4658	13216	9550	784	408	228	28076	1182	7	659	0	14	69	4	69	274.16	117	429.53	342	321	315	15	46	0	2	4
11434	7904	5414	1	1978	1490	28578	525	98	631	17	324	160	55	272	310.9	129	461.6	496	200	399	87	89	7	30	21
13676	7552	4490	2	1270	978	28118	631	260	831	107	138	215	120	195	280.65	175	443.88	484	346	425	38	83	16	12	3
13326	7844	4614	7	1216	982	28120	611	222	865	76	132	205	96	216	282.79	192	444.58	464	318	443	38	97	24	4	5
4994	12996	9344	709	470	276	28100	1168	9	703	0	20	54	7	81	277.83	109	433.79	339	362	327	20	41	0	0	1
11988	7808	5094	1	1848	1428	28458	565	111	679	32	264	171	53	255	300.56	150	452.68	493	239	389	71	91	6	28	17

SUPPLEMENTARY INFORMATION:Monte Carlo Atomistic Simulation and Machine Learning Analysis of Na-K Eutectic Alloy in Condensed Phases, D. Reitz and E. Blaisten-Barojas, George Mason University, Fairfax, VA 22030

10546	7938	5836	2	2294	1682	28756	466	61	502	11	414	187	29	302	328.88	95	473.55	469	146	356	134	93	3	42	35
4560	13274	9584	784	394	242	28084	1201	4	644	1	30	64	1	86	275.52	99	430.79	320	333	307	12	39	0	0	2
13964	7812	4544	3	906	654	27930	638	270	1074	136	44	246	99	167	275.98	257	439.61	491	422	425	18	94	13	4	2
10584	7804	5608	0	2426	1894	28816	478	62	460	12	446	159	27	332	329.39	82	470.78	462	133	350	127	72	6	52	31
5660	12496	8966	531	618	368	28146	1058	12	767	2	36	75	8	80	283.63	146	438.26	436	404	318	20	50	0	2	0
4108	13482	9832	933	406	224	28070	1284	5	571	1	16	64	3	70	272.1	85	425.02	285	283	274	17	49	0	2	2
4588	13254	9562	799	398	252	28088	1197	8	635	0	32	54	6	72	273.64	95	429.29	325	321	326	17	36	0	2	3
11312	7904	5388	1	2032	1598	28634	537	91	560	13	352	160	51	293	323.4	106	464.81	449	188	398	96	87	7	46	26
11106	7916	5478	1	2120	1640	28670	546	79	580	12	370	152	47	296	317.79	116	462.35	471	191	395	95	84	6	38	19
11156	7870	5460	1	2138	1628	28646	496	84	545	10	360	189	41	268	317.26	104	460.78	516	164	375	91	97	8	34	33
5464	12670	9080	579	552	328	28122	1055	11	748	1	28	70	9	95	280.48	123	437.54	387	374	365	19	40	0	0	3
4620	13250	9532	788	414	254	28086	1224	6	631	1	16	61	4	77	274.2	88	427.18	318	335	297	16	44	0	0	1
14282	7620	4418	5	896	628	27894	660	329	1036	152	46	246	140	164	268.73	247	432.8	474	449	436	11	83	15	2	2
14352	7692	4370	6	774	616	27884	645	323	1058	158	74	255	127	153	272.21	253	437.4	487	438	437	17	81	21	6	1
6532	11928	8388	451	768	520	28184	967	36	766	7	44	73	26	122	285.42	131	444.06	406	370	394	29	49	0	4	2
14414	7580	4408	2	816	578	27868	637	361	1027	160	68	270	152	173	273.2	226	438.26	440	428	442	25	92	24	4	4
12326	8054	5148	2	1464	1086	28278	576	146	780	34	184	182	80	240	293.54	160	450.65	470	285	440	59	106	12	16	8
4932	12892	9330	695	612	352	28136	1139	9	658	0	18	71	6	88	277.31	103	436.36	355	343	315	23	48	0	0	3
14544	7606	4336	9	754	550	27844	646	352	1086	174	50	261	133	146	267.72	270	430.28	483	442	443	15	80	22	4	1
3940	13688	9922	961	310	182	28052	1281	7	561	1	10	59	4	71	271.31	101	423.94	280	256	286	16	36	0	0	1
12272	7624	5010	0	1820	1394	28430	536	172	660	47	276	184	87	246	298.48	129	455.85	487	230	418	89	92	12	34	13
11640	7886	5304	4	1928	1450	28524	573	101	665	23	282	160	48	248	302.78	116	459.23	473	244	376	101	93	8	30	17
14212	7710	4416	5	868	646	27908	628	313	1031	152	54	248	123	169	272.25	251	437.67	471	421	456	19	80	21	2	3
4066	13528	9870	943	388	200	28060	1290	6	580	1	6	58	2	61	271.29	95	425.61	293	277	273	17	42	0	2	1
6730	11670	8272	389	884	586	28214	966	29	761	5	68	74	15	127	286.78	137	444.3	423	389	363	26	43	1	4	5
3878	13704	9930	965	334	208	28068	1296	7	555	1	14	55	4	60	272.43	95	424.62	299	266	266	15	32	0	0	1
7238	11412	8056	349	900	550	28206	924	49	795	11	50	74	30	129	287.54	150	444.05	441	391	394	26	41	1	0	0
4024	13578	9862	931	366	220	28070	1256	4	583	0	18	59	2	80	273.22	90	426.76	283	269	298	18	40	0	2	1
4684	13162	9520	794	458	250	28082	1226	11	650	1	8	52	6	71	275.66	91	431.47	311	338	310	21	35	0	0	1
14150	7630	4416	3	948	712	27938	627	319	973	165	76	255	109	176	272.74	230	437.02	470	398	432	25	70	16	6	4
5766	12220	8834	519	812	504	28200	1025	15	709	2	60	59	7	118	284.4	107	439.61	399	361	365	23	42	0	2	2
4124	13574	9840	902	320	182	28054	1260	5	612	2	12	68	2	60	273.76	107	427.09	313	298	275	15	41	0	2	2
11418	7820	5368	2	2068	1558	28598	546	94	583	12	338	150	57	298	313.68	105	462.29	461	198	385	94	83	9	28	19
5012	12890	9328	697	564	310	28120	1158	6	679	0	14	69	3	88	278.02	101	433.29	346	347	314	15	45	0	2	5
11138	7870	5410	1	2110	1706	28690	542	88	524	12	404	155	42	329	319.76	84	463.92	466	185	340	94	82	8	50	15
6776	11590	8174	392	950	672	28256	960	25	712	3	92	79	11	134	286.35	105	445.71	426	371	353	34	45	1	2	2

11970	7888	5260	3	1734	1284	28424	578	118	729	24	258	172	71	252	298.79	141	457.3	465	243	407	78	105	8	28	17
4114	13578	9848	903	326	176	28048	1245	4	582	0	6	61	2	68	271.33	98	424.92	303	278	298	15	38	0	0	2
4642	13140	9520	764	496	288	28110	1179	6	630	0	22	52	4	85	277.83	91	433.03	334	319	329	16	37	0	2	1
13174	7652	4714	5	1388	1064	28184	575	229	772	88	172	203	100	205	282.1	172	446.07	516	281	437	42	84	15	18	6
5686	12490	8932	574	626	376	28136	1074	12	721	2	26	70	4	106	282.23	107	437.29	375	388	350	18	42	0	0	1
4914	12930	9356	704	558	332	28122	1163	6	685	0	32	54	2	91	279.61	105	436.34	326	357	332	25	39	0	0	1
12778	7792	4796	1	1494	1188	28254	574	193	774	57	180	201	86	241	289.9	172	449.95	493	276	398	51	106	11	24	8
11848	7866	5108	1	1878	1488	28498	548	126	652	27	282	158	71	266	305.59	130	455.08	485	220	420	83	97	5	26	12
14230	7760	4414	2	780	628	27892	612	323	1059	146	78	254	136	156	269.1	284	430.77	508	411	449	13	100	19	2	2
11550	8110	5328	1	1834	1386	28460	544	95	691	23	228	172	45	261	302.6	141	456.42	498	231	393	79	97	9	22	12
4808	12928	9390	724	636	362	28142	1174	7	644	0	16	54	4	81	276.77	88	433.19	353	334	315	14	39	0	2	5
4486	13332	9626	798	390	230	28082	1208	8	641	0	16	57	4	73	276.12	91	431.88	330	344	309	14	35	0	2	2
12466	7922	4968	2	1528	1202	28318	581	140	772	36	220	174	68	239	293.95	163	451.25	487	281	418	63	96	9	8	6
4612	13196	9564	798	462	246	28088	1215	6	649	0	8	55	3	69	275.16	95	430.36	324	326	310	19	37	0	0	2
10856	7816	5526	1	2348	1788	28766	491	77	508	12	378	151	41	302	321.86	101	465.88	492	135	379	118	78	4	48	22
5096	12832	9266	659	584	330	28124	1135	8	694	0	14	68	6	100	278.76	105	435.72	331	359	335	24	42	0	2	2
4038	13618	9864	950	316	198	28056	1283	9	574	2	22	58	5	68	270.55	92	423.66	286	278	287	13	34	0	0	2
5264	12618	9160	621	698	390	28158	1088	7	660	1	28	66	5	106	282.01	97	437.63	387	338	330	14	40	0	0	1
13026	7912	4796	2	1306	984	28152	606	203	892	71	120	199	99	199	282.56	220	445.64	516	338	428	32	97	15	8	4
11900	7784	5218	1	1904	1386	28472	548	121	625	27	252	150	65	244	309.62	139	457.25	508	219	414	77	70	9	26	18
6206	11968	8560	522	854	548	28194	1025	29	661	6	58	85	16	116	285.97	108	443.44	401	328	336	25	54	1	0	4
7494	11090	7854	347	1018	678	28240	919	49	734	17	102	102	23	173	288.95	123	447.89	403	357	359	24	59	3	2	5
13688	7870	4646	6	982	714	27970	628	284	988	126	70	233	122	174	276.06	233	442.01	488	380	444	23	90	17	0	1
3724	13760	10042	1013	338	182	28054	1329	8	524	1	8	52	5	66	270.97	72	423.08	260	257	268	17	27	0	0	1
5302	12764	9142	641	554	338	28122	1118	15	697	2	20	70	8	81	280.45	130	438.79	369	359	327	25	38	0	2	2
13174	7846	4704	3	1258	1002	28144	637	218	889	83	152	203	100	187	283.13	204	446.5	484	358	422	48	87	14	6	4
10848	7888	5594	1	2216	1726	28738	484	66	530	12	404	181	30	302	330.78	102	470.51	466	160	346	121	83	5	58	38
4792	13062	9460	764	496	272	28098	1204	9	653	0	16	74	6	84	275.54	97	430.33	339	343	284	9	51	0	0	2
12466	7894	5032	2	1522	1156	28292	558	152	796	43	194	200	76	247	300.83	171	451.06	473	265	412	61	121	12	26	7
11228	7754	5368	0	2184	1724	28706	506	88	521	21	394	170	40	279	322.07	106	466.68	494	172	359	113	90	4	52	29
7324	11296	7976	344	944	606	28218	879	36	782	9	66	85	18	142	287.84	144	445.81	441	350	404	31	52	2	6	4
4306	13460	9706	839	370	222	28070	1226	6	615	0	4	60	1	71	274.25	91	429.51	321	319	297	13	39	0	2	3
5074	12976	9300	682	448	274	28094	1129	10	684	0	18	69	8	80	280.17	114	436.44	352	349	342	20	46	0	4	4
12218	8036	5158	2	1572	1140	28308	638	139	826	27	170	163	83	219	293.78	166	452.57	443	312	423	69	101	12	14	14
4034	13640	9888	946	302	172	28046	1284	6	585	1	8	59	2	61	272.02	82	423.99	291	292	284	14	39	0	0	2
5008	12870	9292	702	592	348	28132	1155	7	655	0	22	58	5	84	277.28	106	432.2	365	346	313	16	41	0	0	1

SUPPLEMENTARY INFORMATION:Monte Carlo Atomistic Simulation and Machine Learning Analysis of Na-K Eutectic Alloy in Condensed Phases, D. Reitz and E. Blaisten-Barojas, George Mason University, Fairfax, VA 22030

4398	13362	9682	848	396	220	28070	1236	7	620	2	10	75	3	70	273.4	107	427.29	320	311	280	15	47	0	2	1
13248	7784	4716	4	1292	960	28128	599	218	884	72	116	200	109	190	281.84	202	447.55	501	331	451	40	96	13	12	5
10952	8108	5558	3	2094	1584	28642	537	81	571	13	316	150	44	282	316.49	122	464.78	480	164	379	92	85	6	26	29
11152	7892	5468	2	2172	1616	28660	524	75	557	10	318	165	38	284	320.43	113	461.74	500	172	363	92	84	9	40	28
12472	7810	4972	1	1648	1218	28324	595	155	774	37	190	173	86	231	292.8	154	450.98	482	281	430	52	106	12	14	15
10976	7994	5616	1	2102	1580	28672	517	71	549	16	370	179	28	288	325.1	101	465.56	488	188	362	98	83	9	30	26
4502	13288	9584	812	440	268	28098	1215	2	637	0	14	61	1	80	275.27	94	430.7	316	320	306	17	40	0	2	2
13646	7616	4528	1	1232	936	28090	628	250	875	96	120	221	106	202	280.62	188	446.05	444	359	422	57	99	19	10	2
6532	11766	8406	455	912	548	28202	983	17	740	6	38	81	9	121	285.37	123	442.17	410	362	360	31	56	0	0	3
5408	12694	9084	609	562	350	28128	1072	13	718	4	26	64	6	83	280.14	134	438.88	400	354	355	17	42	0	4	3
6262	11954	8552	443	860	536	28220	1000	15	739	6	54	74	7	125	288	105	443.95	417	385	348	24	47	0	2	2
7160	11308	7982	394	980	696	28240	916	43	711	17	110	92	16	143	288.84	132	445.37	445	336	359	29	50	1	4	2
14324	7638	4422	3	832	598	27876	648	329	1063	176	56	265	111	149	269.68	266	431.88	489	445	425	17	76	19	6	2
4630	13286	9558	782	378	218	28080	1180	5	662	1	8	57	1	77	276.54	106	431.96	328	330	332	18	38	0	2	2
4446	13378	9654	850	368	210	28064	1229	8	629	0	8	57	3	62	274.56	114	428.43	317	304	308	21	40	0	0	0
10546	8070	5726	2	2264	1724	28756	534	67	534	9	382	144	43	286	321.19	111	464.58	494	157	369	113	78	4	36	26
14462	7712	4378	10	714	524	27828	652	346	1092	174	38	256	127	146	269	265	431.43	468	454	443	19	77	15	0	2
5234	12812	9198	661	534	312	28106	1098	12	712	2	16	77	5	107	280.78	119	436.4	344	344	341	19	52	0	0	4
3974	13600	9896	950	382	208	28064	1283	8	545	3	4	69	4	73	272.56	85	426.11	288	264	266	15	37	0	0	1
4072	13582	9870	927	332	182	28050	1283	5	595	1	10	68	4	53	271.64	85	425.04	297	301	273	20	50	0	2	2
5136	12802	9240	674	582	338	28122	1145	7	671	0	24	72	1	94	281.23	103	438.03	361	346	302	14	51	1	0	2
5118	12902	9318	705	486	252	28088	1147	10	722	1	12	65	5	90	279.09	111	435.05	349	350	332	12	44	1	0	0
4080	13578	9854	931	344	192	28058	1267	8	583	1	10	55	5	63	272.46	97	425.68	297	268	297	14	37	0	0	3
10638	7858	5684	1	2312	1794	28794	479	62	509	12	450	157	28	294	333.57	91	472.07	481	158	379	118	78	6	56	38
4754	13090	9432	767	506	310	28112	1189	10	638	0	18	51	7	79	274.49	115	430.74	327	323	322	21	32	0	2	3
5292	12716	9136	633	606	362	28134	1125	7	704	1	22	59	3	95	281.62	108	437.95	354	384	340	18	31	1	0	3
3832	13654	9960	994	392	220	28068	1305	7	549	1	10	62	3	79	270.85	78	423.55	266	254	270	13	43	0	0	2
6010	12112	8686	517	836	518	28210	1029	18	696	3	44	68	11	123	287.52	100	442.92	393	370	354	22	42	1	4	4
6428	11956	8480	458	768	498	28186	997	18	754	1	54	68	10	111	283.87	130	443.72	411	377	374	23	39	2	2	6
3978	13606	9924	935	358	182	28054	1277	7	564	0	4	72	4	66	271.54	85	423.83	300	284	264	12	48	0	2	3
4256	13438	9726	885	408	240	28078	1281	9	596	1	10	62	6	85	276.55	89	431.54	277	311	274	13	36	0	0	2
4510	13284	9634	817	422	220	28078	1216	7	636	0	8	64	6	70	276.55	95	432.65	316	316	311	17	41	0	0	4
5734	12300	8810	501	816	510	28202	1013	22	653	1	28	71	16	109	284.85	111	442.12	423	352	345	24	39	0	4	3
12238	7854	5080	4	1656	1280	28384	566	149	734	41	248	189	77	248	297.66	143	451.17	479	249	403	71	100	9	26	14
4418	13442	9722	853	296	150	28036	1217	6	653	1	8	79	4	71	274.28	98	428.24	304	317	304	20	61	0	0	1
13644	7736	4570	9	1124	852	28030	646	269	907	123	96	227	106	172	276.72	224	443	488	353	413	32	85	18	8	5

SUPPLEMENTARY INFORMATION:Monte Carlo Atomistic Simulation and Machine Learning Analysis of Na-K Eutectic Alloy in Condensed Phases, D. Reitz and E. Blaisten-Barojas, George Mason University, Fairfax, VA 22030

4790	13120	9452	765	442	266	28094	1190	6	671	1	22	48	3	74	277.5	96	433.2	326	333	337	18	31	0	2	3
14382	7734	4430	7	722	524	27836	644	329	1119	165	40	258	130	144	267.92	296	429.06	479	452	450	16	84	16	4	2
13948	7702	4504	3	986	740	27972	626	270	1015	121	86	225	107	172	277.24	222	442.39	443	387	474	47	91	21	6	1
11556	7928	5238	2	1932	1546	28542	558	109	615	31	294	178	52	269	304.75	129	455.56	481	225	374	88	86	8	42	19
5700	12472	8936	645	606	370	28120	1108	28	686	8	32	73	13	101	282.27	108	438.34	353	351	330	22	51	2	4	2
4130	13560	9844	938	324	178	28050	1271	9	600	1	14	63	5	65	272.76	98	426.06	288	275	288	19	44	0	0	1
14424	7598	4328	4	832	632	27884	633	328	1033	172	66	264	122	147	270.5	265	432.39	505	428	426	19	80	20	4	0
4672	13000	9466	720	640	352	28140	1140	7	586	0	10	58	6	91	279.88	84	436.26	359	314	331	17	36	0	0	1
13692	7586	4518	4	1260	916	28074	588	259	865	117	92	240	95	175	278.01	216	445.41	543	326	408	32	95	20	8	1
4178	13484	9784	906	390	222	28068	1255	8	585	2	8	60	4	73	271.41	87	424.6	303	282	287	13	38	0	2	2
11394	8002	5530	0	1968	1358	28520	527	83	623	19	246	180	44	267	311.81	123	461.33	505	202	389	76	91	5	20	25
12116	7744	5090	1	1814	1374	28428	587	128	698	35	266	180	63	255	305.62	122	454.91	457	266	375	86	97	4	24	18
13788	7552	4512	1	1186	880	28046	601	277	902	120	120	239	120	185	278.16	206	445.03	490	348	431	42	100	19	6	1
3958	13718	9944	982	258	144	28032	1304	4	577	1	10	70	1	70	269.24	94	422.96	272	266	263	12	42	0	0	2
14340	7700	4420	7	804	568	27870	647	329	1066	162	34	262	133	150	269.96	281	431.93	487	429	432	14	91	16	4	3
14358	7728	4360	7	772	602	27872	654	316	1085	169	46	255	110	149	268.02	271	429.39	488	454	428	16	77	14	4	1
7160	11384	8030	403	940	620	28202	948	43	743	11	64	96	23	137	290.16	128	445.31	404	356	355	36	51	2	4	5
4644	13194	9536	810	438	256	28088	1210	10	635	1	14	65	8	84	274.54	82	429.43	319	325	305	11	49	0	6	3
12652	7820	4802	0	1538	1270	28324	553	170	734	54	202	191	86	262	293.07	160	452.35	472	259	430	60	87	10	38	8
12122	7806	5110	1	1766	1334	28410	547	138	698	35	240	165	72	234	298.82	153	455.24	484	248	430	85	86	9	30	14
13288	7608	4652	1	1376	1060	28170	625	207	828	75	162	203	101	200	283.23	168	446.66	465	321	437	48	100	14	24	9
5000	12950	9364	676	496	268	28096	1106	4	706	0	18	71	2	87	278.32	113	437.63	360	344	351	17	50	0	0	4
13650	7698	4596	0	1128	856	28064	585	232	925	83	126	210	113	213	283.81	198	448.51	450	329	475	43	101	16	10	4
4566	13238	9560	768	470	264	28102	1205	5	640	0	4	52	3	102	277.07	83	431.48	303	340	320	11	33	0	0	1
3974	13712	9932	955	260	146	28032	1262	6	585	1	8	58	4	68	270.33	88	422.4	289	263	300	13	38	0	0	2
4340	13476	9710	872	320	198	28060	1234	6	638	0	14	57	3	86	272.65	105	426.25	296	309	310	10	39	0	2	1
12414	7838	4930	2	1620	1302	28368	532	154	717	45	232	187	76	247	298.81	151	453.29	486	237	430	75	104	12	32	13
5814	12340	8842	518	700	428	28164	1017	9	754	2	38	76	4	110	283.49	125	439.41	396	371	367	27	46	0	2	1
5472	12582	9044	581	624	382	28144	1074	12	723	0	38	69	8	97	279.95	101	437.74	388	370	341	20	48	0	2	2
4462	13296	9596	804	450	276	28096	1185	6	622	0	16	61	3	86	275.27	115	431.09	328	295	318	17	39	0	0	0
4378	13290	9668	821	480	264	28092	1216	2	608	0	10	63	2	78	275.96	99	431.81	323	305	293	13	43	0	2	4
11550	7836	5294	2	2000	1534	28568	504	116	555	27	322	196	58	293	314.68	108	462.14	466	169	391	96	90	4	32	24
13006	7898	4880	3	1244	948	28152	614	195	910	58	164	191	103	206	285.02	196	450.66	492	354	424	40	102	7	10	5
11730	7926	5252	0	1852	1420	28490	557	113	681	22	288	193	58	261	303.09	151	456.91	486	220	386	88	118	18	22	9
14060	7750	4462	10	898	684	27922	612	307	1030	148	66	235	131	150	269.96	256	432.86	507	401	464	23	80	11	2	1
4274	13394	9704	855	442	264	28094	1223	5	596	0	16	62	2	85	274.86	85	429.61	316	291	291	12	44	0	0	3

SUPPLEMENTARY INFORMATION:Monte Carlo Atomistic Simulation and Machine Learning Analysis of Na-K Eutectic Alloy in Condensed Phases, D. Reitz and E. Blaisten-Barojas, George Mason University, Fairfax, VA 22030

6440	11830	8422	415	888	578	28222	990	14	753	4	62	88	6	129	286.86	104	441.95	400	414	350	32	56	1	2	4
4586	13332	9608	828	330	184	28050	1222	5	677	1	6	70	3	62	275.04	107	430.67	318	330	302	19	50	0	4	2
13990	7826	4550	8	836	634	27910	638	300	1059	146	66	238	123	167	271.5	255	435.41	476	434	461	15	79	11	8	1
4964	12846	9320	699	624	360	28144	1142	6	642	0	30	63	2	79	278.63	88	436.59	369	341	310	25	46	0	0	2
5408	12638	9104	618	600	350	28132	1121	11	737	1	30	71	7	93	281.19	115	438.23	358	395	328	21	47	0	2	3
7338	11168	7914	342	1070	692	28268	918	39	737	4	86	82	29	147	289.25	129	443.77	431	366	380	32	52	1	0	3
14088	7670	4362	2	982	782	27970	605	302	975	156	82	238	113	166	273.91	263	438.18	513	368	448	22	68	16	4	1
4530	13244	9590	807	448	262	28098	1207	2	636	0	24	57	1	82	277.31	98	431.84	322	323	314	11	35	0	0	4
4250	13432	9734	880	394	242	28076	1257	9	601	2	22	57	7	82	272.62	86	427.76	285	307	294	16	42	0	2	2
13578	7840	4642	4	1050	804	28024	622	251	960	123	106	241	92	189	276.47	219	441.91	484	365	422	31	94	17	4	1
5460	12616	9056	605	600	364	28128	1097	12	730	1	32	63	7	95	280.45	115	437.74	380	370	335	15	40	0	0	3
4452	13202	9582	821	550	320	28118	1225	14	592	1	10	51	9	73	275.51	91	430.27	329	305	294	17	32	0	2	3
11816	7874	5282	2	1830	1366	28476	585	115	687	26	276	167	61	260	305.25	133	458.31	467	229	389	82	98	9	32	16
5018	12968	9340	680	492	270	28096	1153	9	713	0	8	55	7	87	277.39	101	433.22	341	374	339	13	37	0	0	4
11202	7946	5508	1	2054	1546	28628	506	90	587	15	318	173	50	266	319.65	102	463.45	480	185	389	115	92	3	50	28
4440	13274	9596	816	488	296	28110	1235	7	588	1	16	60	3	75	276.82	83	431.22	326	318	283	12	34	0	0	4
5888	12306	8824	541	682	410	28150	1040	24	739	2	36	68	19	109	285.17	116	440.65	380	372	368	21	40	0	4	5
14210	7682	4430	6	874	642	27898	621	323	1025	166	58	259	126	149	267.52	286	430.17	520	401	433	13	81	13	2	1
3968	13560	9884	939	430	234	28080	1298	7	564	1	4	55	4	78	272.89	89	424.95	283	283	274	9	40	0	0	1
4580	13254	9596	805	404	220	28068	1198	11	648	1	14	61	7	79	275.21	105	429.55	325	317	312	15	45	0	0	1
12098	7960	5058	1	1688	1346	28418	554	140	697	29	240	184	78	243	299.23	136	454.68	469	250	416	87	113	11	28	16
4960	13032	9392	697	454	242	28088	1131	5	692	0	8	61	3	86	278.69	99	437.37	355	343	346	13	44	0	0	2
12358	7740	4952	1	1778	1344	28416	555	146	684	34	218	171	86	247	300.29	134	454.48	489	230	430	73	94	10	24	11
13570	7734	4624	3	1140	856	28042	623	263	931	132	108	251	102	191	277.55	201	442.38	475	380	404	42	106	13	10	1
12556	7956	4872	3	1474	1214	28296	591	169	807	49	206	177	94	216	287.88	179	450.14	498	286	430	59	97	10	16	8
12656	7956	4912	3	1424	1108	28238	612	167	843	49	166	195	89	194	285.12	179	448.86	486	329	422	62	107	10	16	11
4344	13388	9716	879	392	212	28062	1235	9	617	0	10	57	4	75	274.45	111	430.41	307	292	300	14	39	0	0	2
14430	7662	4344	3	782	590	27860	641	329	1067	169	50	266	119	150	269.46	254	431.89	477	448	434	23	85	17	2	1
4872	13056	9390	702	494	288	28110	1145	2	679	0	8	56	2	91	279.19	113	436.27	349	346	342	13	35	0	2	1
13392	7848	4684	3	1168	886	28096	597	220	906	80	104	201	99	175	281.62	247	448.58	551	321	427	28	91	15	14	1
7640	11040	7826	311	1016	624	28214	887	44	803	15	56	104	18	134	288.01	150	443.46	450	394	373	33	55	2	12	5
4986	12840	9316	691	624	350	28138	1138	10	654	4	22	72	5	94	280.89	101	438.02	363	327	304	18	46	0	0	2
4160	13510	9816	903	370	198	28062	1251	6	591	1	8	67	4	59	271.63	106	424.73	315	281	280	20	46	0	0	1
5458	12682	9042	614	564	360	28134	1110	15	708	5	26	74	7	92	279.84	124	435.91	374	360	326	18	47	0	2	2
11984	8044	5170	0	1670	1290	28404	573	127	734	35	214	165	71	224	302.54	160	450.86	497	253	441	72	91	11	32	14
5674	12456	8958	569	650	372	28136	1048	14	723	2	22	73	7	104	284.14	126	442.1	390	367	349	19	51	0	4	4

SUPPLEMENTARY INFORMATION:Monte Carlo Atomistic Simulation and Machine Learning Analysis of Na-K Eutectic Alloy in Condensed Phases, D. Reitz and E. Blaisten-Barojas, George Mason University, Fairfax, VA 22030

10698	7836	5656	0	2348	1788	28804	496	71	473	15	416	170	35	287	334.35	82	468.75	461	146	371	133	84	4	56	36
4552	13260	9592	787	426	244	28094	1198	5	659	0	18	50	3	73	276.53	96	432.77	322	334	328	20	36	0	2	3
4128	13606	9816	938	304	188	28052	1287	6	593	2	10	65	2	71	271.98	83	425.82	280	294	274	15	42	1	0	2
13822	7596	4510	5	1144	850	28038	606	280	902	119	100	235	121	213	278.57	217	444.85	469	328	430	26	95	19	16	3
13628	7630	4552	5	1194	924	28078	614	262	858	118	140	230	103	217	278.41	188	446.27	452	332	432	37	89	14	10	4
4166	13570	9824	925	310	168	28042	1270	12	587	2	4	55	5	69	271.65	82	425.14	292	291	292	11	40	0	0	2
4610	13096	9520	746	550	320	28126	1172	9	620	0	30	58	7	97	279.78	86	434.63	333	322	311	19	37	0	0	0
5106	12824	9214	644	606	374	28148	1100	4	677	0	22	55	4	108	280.24	104	437	361	340	355	13	36	0	2	2
11492	7986	5400	2	1910	1428	28530	547	107	634	26	280	177	57	273	306.55	133	459.12	487	205	382	84	97	2	30	18
4748	13144	9472	779	452	260	28086	1177	8	628	2	10	72	4	83	275.23	93	429.72	333	315	308	19	46	0	0	1
4250	13454	9754	866	382	216	28068	1238	5	614	0	12	64	3	65	272.41	100	425.09	320	301	292	11	44	0	0	5
12946	7760	4790	4	1442	1092	28204	604	193	798	75	164	214	77	222	283.65	169	449.87	479	303	398	56	106	17	8	3
3944	13692	9922	947	306	178	28050	1295	9	580	2	8	57	6	74	270.48	93	422.08	271	283	280	11	36	0	0	4
4662	13150	9530	760	474	264	28100	1191	7	662	0	20	67	6	87	277	110	432.13	332	331	306	13	47	0	0	1
6428	11772	8454	441	944	572	28222	983	22	729	2	48	72	14	129	286.84	119	442.37	428	383	350	20	42	0	4	1
3970	13616	9908	937	360	196	28056	1285	8	565	1	4	66	5	68	270.88	81	424.04	277	281	278	16	45	0	2	5
7190	11510	8112	406	778	502	28162	960	38	791	13	68	102	16	121	288.42	125	443.29	429	400	349	22	63	3	2	3
4206	13464	9768	893	398	224	28068	1252	8	603	1	8	57	6	71	271.67	93	424.71	298	295	296	17	35	0	0	2
4826	12964	9386	715	572	350	28134	1171	5	649	1	32	60	1	71	278.66	101	436.12	359	340	302	23	38	0	4	4
13790	7760	4518	8	1010	806	27998	637	275	990	143	108	243	100	177	273.06	240	438.29	485	385	418	28	92	14	6	1
4690	13178	9516	776	430	248	28080	1193	9	661	1	18	71	6	84	275.73	106	430.64	335	331	296	12	46	0	0	1
5402	12682	9096	599	588	346	28136	1075	8	722	1	22	65	5	103	281.54	120	437.6	376	363	353	19	48	0	0	1
4490	13378	9644	826	350	192	28056	1213	10	654	1	2	65	7	77	273.54	109	427.78	316	323	315	11	45	0	0	1
5602	12410	8964	560	712	428	28166	1063	12	693	2	48	66	8	104	282.54	95	439.09	378	370	354	25	40	0	2	3
11066	7820	5456	0	2176	1730	28716	514	89	548	19	416	155	46	304	321.92	106	466.52	467	184	362	118	87	3	48	23
14416	7674	4376	5	772	570	27860	674	329	1076	156	50	255	135	143	269.85	268	432.51	487	456	421	13	92	17	2	0
14010	7866	4584	3	790	584	27902	602	306	1083	126	64	229	135	168	276.23	267	439.85	467	413	489	29	91	16	4	1
6556	11940	8448	408	740	450	28164	962	17	824	3	28	91	13	117	285.55	139	442.91	422	402	375	25	61	0	2	2
4408	13414	9690	874	338	190	28050	1251	6	629	1	10	67	3	63	274.13	91	427.21	312	328	287	12	45	0	0	3
13752	7962	4648	6	856	658	27954	633	268	1058	117	68	210	121	169	276.75	246	441.33	466	415	480	26	70	20	10	2
12146	7900	5136	1	1690	1264	28384	545	133	722	28	228	156	81	255	297.89	154	453.74	493	242	451	58	88	7	16	14
4514	13290	9610	817	408	234	28070	1197	6	631	1	14	54	2	86	273.63	90	429.88	311	315	324	15	42	0	0	2
13776	7744	4628	4	1026	724	27984	618	269	989	126	80	237	113	183	274.72	239	442.71	478	371	445	27	92	16	6	2
4842	13056	9396	742	488	302	28110	1185	11	669	1	26	54	7	79	275.96	89	431.22	343	358	316	16	43	0	0	1
3932	13664	9932	956	332	184	28052	1303	10	575	2	8	54	4	63	270.33	79	422.81	284	283	275	10	41	0	0	4
4440	13262	9648	816	456	256	28086	1220	12	628	0	24	69	11	77	275.84	89	431.79	320	316	291	16	55	0	0	2

SUPPLEMENTARY INFORMATION:Monte Carlo Atomistic Simulation and Machine Learning Analysis of Na-K Eutectic Alloy in Condensed Phases, D. Reitz and E. Blaisten-Barojas, George Mason University, Fairfax, VA 22030

4160	13472	9814	915	396	212	28068	1290	8	584	1	14	60	5	61	272.85	80	425.99	291	302	274	19	43	0	0	1
13734	7840	4566	4	1010	768	27994	642	281	965	145	68	228	100	147	273.6	239	437.48	517	402	423	31	69	16	8	1
14300	7618	4388	4	870	646	27892	627	336	1025	172	66	262	131	182	267.37	246	429.73	459	423	452	13	76	19	4	3
3902	13678	9954	996	328	178	28048	1321	10	577	1	8	60	5	76	271.23	90	423.5	253	275	270	12	49	0	0	3
14338	7712	4346	3	786	634	27886	623	334	1055	168	64	256	131	157	268.49	279	430.06	506	414	439	10	76	16	6	2
10974	7982	5408	1	2182	1758	28710	517	86	545	13	344	145	42	288	313.94	111	458.84	485	183	397	100	67	13	54	30
4542	13236	9560	816	458	280	28098	1215	9	627	1	22	55	4	69	273.88	102	429.37	338	314	301	13	39	0	0	3
12392	7942	5166	0	1508	1064	28266	617	160	810	33	174	172	98	239	293.12	145	451.97	426	313	432	72	102	7	20	7
12228	7758	5100	2	1742	1306	28420	585	139	727	37	256	174	60	273	301.31	149	453.77	490	263	383	59	100	12	28	6
3926	13642	9916	963	352	210	28064	1299	6	570	1	18	70	4	58	270.18	95	422.26	280	273	267	22	43	0	0	3
4936	12964	9370	723	500	300	28108	1155	7	677	0	38	66	6	89	278.23	105	434.67	342	347	330	15	48	0	0	2
12428	7874	5034	3	1614	1162	28284	604	161	743	45	152	187	91	206	286.7	169	447.57	506	272	410	59	99	8	16	6
13614	7766	4638	2	1114	810	28042	627	253	945	106	94	219	115	189	276.68	210	442.8	472	364	453	29	91	14	6	3
4530	13330	9652	825	350	176	28044	1229	11	653	1	6	78	7	68	272.73	98	427.59	309	334	291	18	54	0	0	2
3908	13658	9930	957	354	202	28062	1293	7	560	1	10	63	4	71	272.48	89	425.01	280	269	265	18	44	0	0	1
13856	7766	4564	4	968	720	27958	626	281	1009	140	76	254	112	162	273.4	229	439.59	479	401	438	35	103	12	8	2
12436	7776	5002	1	1612	1244	28338	555	166	720	60	246	226	71	242	299.52	149	452.31	477	275	377	83	116	11	20	9
4960	12958	9320	706	526	328	28120	1164	9	665	0	26	74	3	91	280.05	104	434.72	355	339	288	15	52	0	2	2
14324	7726	4352	3	788	628	27878	640	323	1066	161	56	265	117	150	269.35	276	432.68	497	433	423	17	94	25	4	1
11580	7848	5386	2	1958	1424	28512	525	99	616	19	284	176	53	268	312.04	128	458.1	441	206	413	116	85	3	30	19
13280	7708	4696	2	1322	992	28152	587	224	849	81	138	195	107	206	282.76	183	445.98	486	314	466	39	92	14	14	7
5338	12660	9110	625	610	384	28150	1089	8	696	1	44	63	4	95	279.27	132	435.17	398	357	331	13	44	0	4	2
13268	7676	4618	5	1354	1070	28158	620	225	809	83	156	204	99	205	282.65	177	444.76	452	303	438	55	83	15	16	8
4430	13368	9650	865	382	226	28068	1260	9	650	1	10	62	4	68	274.64	114	429.33	294	329	292	15	41	0	2	4
4336	13366	9706	856	422	234	28078	1255	5	599	1	14	63	2	78	276.08	94	430.38	305	319	278	12	40	0	0	2
6256	12094	8600	483	724	450	28164	1010	19	767	2	38	68	15	125	283.59	113	439.18	376	384	391	20	48	0	2	5
5346	12728	9114	633	570	350	28134	1118	8	692	2	24	64	6	91	283.24	98	439.27	369	372	340	14	40	0	2	3
12002	7842	5286	2	1780	1244	28412	583	129	684	26	248	158	72	233	307.41	138	453.46	501	262	399	71	79	5	10	15
4768	13068	9452	743	506	294	28112	1170	6	663	0	24	60	3	82	275.84	95	432.42	356	333	311	11	41	0	0	3
12898	7732	4838	1	1484	1106	28252	592	182	780	57	176	189	87	227	292.42	165	450.51	469	300	430	60	93	13	16	7
4878	12900	9368	725	598	352	28132	1170	7	661	0	36	69	5	97	278.49	102	434.79	333	343	303	17	43	0	0	4
5864	12198	8768	469	818	506	28204	997	14	718	3	50	78	9	138	281.99	115	438.84	412	362	347	16	45	0	0	1
4390	13396	9666	850	370	228	28068	1240	11	633	1	18	63	5	63	274.33	99	428.73	303	330	299	22	44	0	0	4
4964	12876	9324	675	590	346	28128	1134	3	674	0	24	67	3	97	279.63	90	437.05	345	353	327	21	48	0	4	2
4586	13248	9576	807	406	236	28072	1210	12	659	1	20	68	8	76	274.06	112	428.38	327	323	298	14	52	0	0	1
11834	7866	5290	1	1868	1342	28472	553	122	667	30	246	177	66	267	305.74	131	456.36	482	229	403	76	93	9	24	14

SUPPLEMENTARY INFORMATION:Monte Carlo Atomistic Simulation and Machine Learning Analysis of Na-K Eutectic Alloy in Condensed Phases, D. Reitz and E. Blaisten-Barojas, George Mason University, Fairfax, VA 22030

13970	7674	4526	4	996	712	27958	637	307	961	144	78	245	125	169	275.89	228	439.23	475	391	438	29	82	18	2	1
5000	12948	9342	683	500	288	28102	1143	7	707	0	24	59	4	92	279.33	107	435.19	348	371	332	14	43	0	0	2
13464	7844	4702	5	1084	826	28042	600	258	967	122	104	238	98	192	275.21	227	439.33	481	377	431	42	98	18	18	1
14386	7626	4332	6	858	634	27882	648	327	1041	162	44	254	129	138	268.54	268	430.22	503	425	433	16	85	17	2	3
4438	13432	9674	855	318	180	28046	1239	10	627	1	4	56	3	54	274.96	104	429.25	322	313	297	18	36	0	0	2
4986	12842	9308	696	620	358	28140	1154	6	633	0	22	63	3	102	279.13	86	433.53	340	347	318	17	38	0	4	1
12868	7976	4842	0	1306	1028	28182	633	196	851	72	156	198	91	211	281.43	177	446.13	477	336	420	40	103	15	6	6
4266	13482	9744	892	352	206	28060	1239	8	603	1	10	53	4	78	271.95	92	426.74	298	290	311	12	33	0	0	2
11744	8074	5210	1	1758	1388	28448	564	108	686	25	254	173	59	256	306.47	114	458.05	463	256	413	79	100	5	18	19
14038	7706	4456	6	972	718	27954	646	283	1006	128	60	248	119	165	275.66	258	441.33	469	398	434	31	98	20	4	2
11498	8160	5326	3	1830	1412	28492	563	101	700	30	244	173	54	258	301.44	146	453.41	479	239	414	87	96	5	20	10
4068	13602	9860	930	330	186	28054	1266	8	601	1	8	53	5	70	270.38	105	423.48	297	286	292	10	35	0	0	3
7664	11106	7800	286	956	620	28228	900	28	847	6	78	81	14	143	288.85	134	445.27	443	422	383	29	54	0	4	3
12162	7870	5128	2	1736	1258	28368	550	128	719	37	192	191	61	248	302.01	156	456.19	471	226	410	76	104	5	18	15
7370	11062	7840	334	1118	772	28284	874	45	725	12	118	88	23	143	291.85	139	447.21	465	329	353	45	54	1	4	4
11194	8028	5454	1	1968	1566	28608	516	71	635	13	364	180	35	272	315.22	121	460.83	499	193	375	106	107	3	32	16
5612	12396	8904	576	756	472	28180	1114	18	677	2	40	73	13	112	283.75	94	439.97	349	372	316	25	47	1	0	4
4852	13116	9444	784	414	234	28074	1215	12	679	1	14	62	7	87	275.33	111	430.11	311	345	307	10	36	0	0	2
4314	13474	9732	890	328	194	28058	1270	11	633	3	16	67	5	66	273.46	99	426.29	294	308	279	16	46	0	0	3
14054	7700	4510	3	920	672	27938	606	287	1030	134	76	258	122	171	275.5	245	438.49	489	393	440	24	109	16	6	4
5340	12676	9098	656	588	388	28146	1102	15	696	2	56	57	11	93	282.62	119	438.81	373	339	348	17	38	0	0	4
4232	13370	9738	839	468	272	28106	1215	3	585	0	22	59	2	81	277.26	86	431.18	311	286	309	19	42	0	4	3
5188	12750	9206	645	614	356	28140	1102	5	680	1	24	69	4	90	279.29	106	438.97	377	349	328	24	48	0	2	1
4418	13282	9634	826	476	274	28100	1221	3	611	0	16	53	2	81	276.61	86	432.41	321	309	305	13	33	0	0	1
4918	12990	9390	696	502	284	28106	1133	8	686	0	20	69	5	91	280.29	107	436.57	347	351	329	23	44	1	0	0
13796	7730	4506	3	1026	832	28020	590	280	911	125	114	258	105	188	275.62	210	443.54	489	341	420	38	107	22	14	3
5898	12170	8760	543	804	498	28184	1045	28	664	4	54	76	18	123	285.03	101	440.96	383	345	334	24	43	0	0	2
4098	13602	9856	943	320	170	28046	1278	10	583	2	0	57	4	77	271.27	89	425.59	277	284	286	14	36	0	0	1
3918	13650	9918	971	352	210	28064	1321	9	555	0	16	57	5	70	273.35	87	426.39	267	281	264	14	34	0	0	2
14304	7646	4404	5	864	618	27884	636	319	1038	164	42	267	120	154	270.53	259	433.36	483	404	427	21	97	18	6	4
4266	13358	9716	889	470	268	28094	1275	7	572	1	16	59	4	67	275.46	87	428.76	304	293	269	18	44	0	0	2
4862	13074	9434	737	460	250	28090	1181	7	674	0	10	65	6	76	277.82	90	434.52	339	361	319	18	39	0	0	0
14274	7776	4408	6	754	598	27874	639	332	1062	153	62	250	137	150	269.44	278	431.87	477	428	452	23	80	22	2	1
12496	7866	5000	3	1570	1158	28282	609	165	763	44	178	163	91	235	286.63	166	448.53	475	296	438	48	87	12	12	8
13770	7804	4596	5	990	732	27974	620	276	999	129	78	234	113	173	275.72	235	441.35	479	388	452	29	88	14	4	3
4006	13588	9900	945	366	192	28060	1288	7	574	2	8	64	4	71	272.78	90	426.06	287	279	271	11	39	0	0	3

SUPPLEMENTARY INFORMATION:Monte Carlo Atomistic Simulation and Machine Learning Analysis of Na-K Eutectic Alloy in Condensed Phases, D. Reitz and E. Blaisten-Barojas, George Mason University, Fairfax, VA 22030

12664	7692	4866	1	1626	1234	28308	533	181	740	51	206	180	101	234	293.81	152	450.95	493	234	463	64	96	13	20	9
11220	7888	5436	2	2094	1608	28630	499	101	590	13	336	157	51	302	315.92	116	464.32	486	182	394	91	94	9	44	21
4240	13434	9744	884	404	238	28078	1242	5	602	2	16	63	3	72	273.21	91	427.39	302	293	296	18	45	0	2	3
11186	7960	5582	0	2088	1484	28636	543	85	567	16	298	160	42	296	316.56	122	458.93	489	166	379	86	91	9	34	15
14442	7688	4330	4	754	590	27862	640	330	1095	164	56	251	126	143	268.26	287	429.79	495	439	446	15	78	19	2	2
4032	13490	9848	918	452	252	28088	1270	4	578	1	12	59	2	78	272.27	94	425.29	299	275	273	11	34	0	0	3
4784	13002	9440	718	550	314	28118	1128	6	645	1	28	65	3	98	280.36	94	437.02	346	318	340	11	44	0	0	7
11100	7830	5630	1	2218	1528	28652	542	69	546	13	312	174	33	298	321.44	80	468.73	416	187	382	122	89	5	34	24
4348	13474	9736	880	316	168	28046	1259	8	647	1	4	59	2	71	272.48	112	427	305	311	290	7	42	0	0	2
14204	7690	4360	6	902	702	27920	627	315	988	151	58	254	129	178	270.34	252	435.25	470	390	447	19	93	17	4	1
5174	12782	9186	668	604	366	28132	1143	9	659	3	20	72	6	94	282.85	84	438.82	345	351	315	24	46	0	0	1
14198	7650	4404	6	884	694	27926	646	316	994	155	88	251	121	166	272.6	238	435.36	476	404	428	21	86	22	6	3
12242	7802	5070	0	1694	1298	28384	529	147	717	40	252	188	78	255	299.64	157	455.57	500	240	424	71	96	8	26	9
4142	13522	9818	912	366	204	28064	1268	6	606	1	12	56	4	74	272.35	91	424.66	284	298	295	18	36	0	0	0
5644	12380	8932	571	734	438	28172	1089	15	708	3	44	76	7	108	283.76	101	440.72	391	383	309	16	44	0	0	2
6934	11572	8218	396	870	538	28186	969	36	754	12	50	98	15	122	289.68	125	442.9	431	389	340	27	53	2	2	1
7002	11390	8114	357	1014	648	28242	942	29	753	5	64	94	16	150	288.91	115	445.19	407	383	355	34	58	2	8	2
11668	7862	5282	2	1952	1448	28508	537	114	632	26	266	187	58	297	312.24	111	461.63	459	220	370	83	89	3	28	18
13602	7886	4710	2	988	712	27982	634	260	1031	112	82	234	113	173	275.78	237	439.66	472	405	448	32	96	19	2	1
10840	7942	5728	0	2172	1598	28710	493	73	528	8	376	176	41	300	320.37	96	466.11	483	164	359	112	86	4	50	28
4380	13322	9646	828	464	272	28096	1231	6	616	1	12	57	3	71	275.14	87	429.79	331	326	291	13	39	0	0	1
5320	12558	9106	603	712	424	28164	1103	10	686	0	40	74	5	110	281.29	88	439.72	369	363	316	16	48	0	4	4
4640	13204	9536	799	448	252	28090	1204	6	654	2	10	60	4	78	275.58	104	430.32	320	317	317	19	36	0	0	0
4388	13388	9706	854	376	196	28060	1223	5	632	0	6	71	3	78	276.91	91	431.22	314	310	294	12	55	0	0	2
4732	13114	9492	756	466	266	28094	1180	2	664	0	24	59	0	81	278.2	117	434.55	335	334	323	14	38	0	0	3
14234	7716	4386	4	832	660	27900	645	314	1040	157	66	257	121	163	267.57	256	429.21	485	440	425	16	83	15	2	1
11888	7994	5146	4	1730	1394	28442	588	128	703	25	240	164	69	258	293.37	156	452.2	497	242	386	67	102	10	48	9
14344	7684	4378	6	816	600	27864	639	327	1071	164	42	271	124	137	267.55	282	429.55	517	438	414	15	89	15	0	1
4104	13528	9822	896	384	220	28070	1276	5	576	0	12	68	2	84	272	86	425.46	282	307	269	10	42	0	0	4
4328	13352	9708	853	432	244	28086	1226	9	586	1	22	49	3	86	273.57	91	427.63	316	289	308	5	32	0	0	3
14426	7722	4420	11	710	514	27840	638	331	1113	159	46	254	127	141	269.82	300	432.1	495	457	448	14	83	20	2	1
11344	7844	5458	0	2090	1504	28568	507	88	609	22	302	180	40	269	316.58	117	462.8	505	180	384	84	97	5	26	27
4232	13456	9770	892	384	212	28066	1268	11	605	1	12	62	6	79	272.3	93	425.62	277	308	290	16	44	0	0	3
14042	7600	4412	1	1056	784	27972	618	303	940	154	72	254	118	188	272.63	220	436.2	475	384	435	22	81	18	6	2
3852	13748	9962	976	300	180	28050	1310	6	564	1	8	63	4	73	270.78	80	423.53	267	287	262	12	42	0	0	4
12182	7936	5072	2	1664	1288	28392	583	140	725	35	226	172	78	227	297.71	145	451.53	498	275	416	67	98	9	22	15

10826	8006	5644	0	2194	1640	28708	559	53	550	9	356	152	31	282	318.52	92	463.12	495	196	358	90	82	6	40	29
13634	7856	4656	9	986	754	27992	614	278	979	136	96	229	113	177	274.62	232	438.43	482	381	464	29	80	12	10	1
13266	7798	4668	3	1250	992	28130	636	219	900	76	140	196	109	193	278.68	207	445.48	458	359	448	51	97	12	16	4
4578	13296	9606	802	376	198	28058	1213	5	677	1	4	66	2	70	274.61	109	428.85	319	348	306	18	43	0	0	1
13146	7742	4846	4	1344	934	28152	630	210	856	60	124	201	108	217	285.89	175	449.27	464	322	426	38	113	20	16	6
4154	13524	9806	909	370	204	28062	1272	7	590	1	4	65	4	64	271.95	99	425.1	305	286	275	14	42	0	0	1
11834	8038	5348	2	1720	1224	28394	585	103	754	29	208	167	52	234	298.46	160	453.79	494	260	417	74	90	6	22	6
3936	13602	9950	978	374	186	28058	1307	9	561	1	10	61	6	71	272.45	83	424.55	272	269	265	16	42	0	0	1
4186	13476	9792	888	388	214	28068	1242	4	596	1	12	70	2	78	272.31	102	426.13	304	280	281	15	50	0	0	2
4814	12976	9418	729	580	322	28128	1153	5	617	0	18	67	3	85	281.15	99	437.29	349	305	314	21	46	0	0	4
14296	7818	4440	6	712	544	27856	654	317	1100	157	44	245	133	147	270.57	271	434.09	483	474	452	13	77	12	0	0
5552	12486	8980	580	702	418	28170	1086	14	685	0	32	57	8	101	285.4	106	440.41	385	365	341	18	36	0	0	3
3998	13634	9882	942	334	200	28062	1287	6	589	1	14	51	3	73	273.09	93	425.36	291	283	283	7	31	0	0	2
13044	7964	4858	11	1188	912	28116	624	212	941	75	142	213	90	192	282.14	219	445.94	468	352	428	49	106	19	8	7
14366	7728	4420	7	740	542	27842	670	311	1127	166	46	267	110	150	269.17	278	432.44	465	474	420	17	87	14	0	2
6622	11696	8414	443	890	512	28182	997	30	721	12	48	93	14	109	286.42	109	440.8	440	376	330	21	55	1	0	1
14270	7532	4398	5	962	684	27920	639	312	989	145	68	256	128	177	273.51	229	437.44	457	401	441	23	96	23	4	2
4642	13130	9520	742	516	288	28110	1163	2	636	0	12	55	1	92	276.83	90	432.99	341	332	328	12	37	0	2	2
12416	7950	5038	2	1534	1156	28296	555	140	801	41	182	183	75	225	291.87	176	449.43	508	277	440	56	106	9	18	10
4608	13242	9554	798	426	244	28084	1229	4	658	0	8	63	2	80	275.94	99	430.54	317	345	288	14	50	0	2	1
14588	7606	4342	4	730	518	27832	620	349	1097	171	48	269	135	135	268.36	298	430.04	520	448	435	13	89	16	0	0
11978	8010	5280	3	1704	1194	28362	589	138	747	28	174	172	69	258	303.81	152	454.66	468	258	407	60	109	12	20	9
4242	13598	9778	894	260	158	28042	1263	4	632	0	6	57	2	76	274.57	93	428.42	284	302	298	13	35	0	0	1
4822	13058	9392	758	508	316	28118	1185	2	648	0	20	50	1	82	278.93	88	433.44	335	342	322	15	34	0	2	3
5412	12620	9116	639	604	344	28132	1121	10	712	1	36	72	6	94	283.64	135	438.24	375	360	312	16	47	0	0	1
13360	7826	4710	7	1174	884	28080	610	228	915	96	120	225	108	190	278.14	227	443.45	510	344	423	27	108	11	6	4
12560	7728	4948	2	1634	1212	28302	530	160	733	50	202	205	69	252	298.26	151	451.94	495	222	413	57	115	9	18	13
14372	7720	4386	4	776	568	27858	649	316	1077	162	36	257	120	130	269.79	291	432.43	509	434	432	15	82	16	0	2
5516	12632	8982	592	590	398	28158	1092	8	744	0	36	72	7	102	279.97	134	438.72	392	374	323	12	53	0	4	2
11670	8094	5386	0	1744	1284	28440	536	104	697	17	242	173	56	261	308.01	138	457.71	484	221	406	81	110	5	14	14
14426	7632	4320	3	818	624	27876	630	333	1047	168	48	252	123	153	269.92	267	431.43	487	415	453	18	75	22	8	1
4478	13326	9638	825	388	222	28068	1216	10	636	1	16	62	5	69	276.09	106	430.07	323	311	304	13	42	0	0	5
14210	7688	4398	3	868	674	27918	641	301	1017	150	80	254	113	161	273.61	239	437.67	475	410	441	21	88	18	0	4
5172	12788	9164	657	596	392	28150	1132	10	663	0	34	58	7	92	278.22	104	434.12	345	347	338	23	28	0	4	6
14308	7704	4460	4	794	556	27872	638	318	1078	152	48	255	132	157	273.28	262	438.13	479	444	450	17	88	10	2	0
12524	7952	4980	2	1464	1138	28262	609	161	821	52	178	169	75	208	285.25	166	447.84	483	302	449	59	91	22	26	5

SUPPLEMENTARY INFORMATION:Monte Carlo Atomistic Simulation and Machine Learning Analysis of Na-K Eutectic Alloy in Condensed Phases, D. Reitz and E. Blaisten-Barojas, George Mason University, Fairfax, VA 22030

7260	11268	7974	351	1014	648	28230	948	39	748	6	60	82	25	139	289.54	119	444.33	411	387	367	34	53	2	6	4
13456	7882	4684	7	1082	824	28028	642	257	963	129	86	231	94	173	277.64	217	444.89	492	394	416	32	89	15	14	2
4140	13578	9814	925	324	192	28058	1262	7	593	1	10	52	5	79	271.61	82	425.43	280	279	307	14	36	0	0	1
14330	7596	4334	3	886	684	27910	635	332	998	163	76	246	121	156	270.24	269	436.29	497	410	430	22	72	22	4	0
4302	13446	9712	878	380	222	28068	1240	3	620	0	6	60	2	74	272.75	97	427.43	299	293	304	18	44	0	0	1
3866	13678	9976	985	338	180	28048	1311	8	567	1	10	58	5	67	269.99	91	423.77	275	265	268	12	44	0	0	2
14346	7710	4384	5	788	594	27874	652	328	1057	164	48	261	124	124	269.52	289	431.84	524	434	410	19	82	21	4	1
12098	7976	5132	2	1690	1270	28394	592	112	745	23	204	154	70	222	294.35	157	453.84	523	255	420	66	95	5	22	4
14444	7682	4342	3	764	578	27858	669	336	1082	177	46	270	121	154	269.55	269	431.93	473	456	411	15	88	15	2	1
5234	12636	9110	612	718	448	28184	1086	8	656	1	34	63	6	102	282.41	97	438.59	391	334	333	17	33	0	4	3
14204	7696	4400	6	866	674	27918	616	333	998	146	74	242	146	158	272.2	266	436.46	497	398	458	20	78	20	4	2
3720	13722	10050	1007	372	188	28058	1321	5	539	1	6	49	4	65	271.14	79	423.95	273	259	271	11	29	0	0	3
4562	13252	9562	789	438	264	28098	1197	1	646	1	20	59	0	95	275.19	103	430.74	311	326	324	10	41	0	0	3
13182	7844	4692	3	1226	1020	28150	615	210	869	79	160	207	100	214	281.2	175	447.81	454	330	440	47	95	12	24	6
11250	8022	5442	2	1988	1532	28582	514	73	609	19	302	189	42	279	311.98	118	461.11	502	188	362	103	98	1	46	12
4104	13546	9820	923	372	216	28068	1283	7	588	1	10	53	5	72	272.1	95	425.29	285	291	285	15	35	0	0	1
5542	12436	8984	568	734	436	28174	1102	9	699	2	42	68	6	95	285.06	97	440.81	386	393	310	24	41	0	0	3
7514	11114	7820	304	1030	676	28230	902	31	780	9	76	106	14	147	287.8	121	446.89	425	383	365	34	69	2	0	7
13676	7842	4612	7	1014	762	27992	640	255	980	128	86	233	110	171	275.45	230	438.72	484	393	436	27	84	8	0	2
10518	7784	5706	1	2434	1890	28876	522	48	460	6	474	157	24	331	329.2	89	472.09	485	146	302	115	70	0	62	30
13716	7878	4624	4	964	718	27974	609	268	1023	119	68	232	116	155	275.08	273	438.35	509	383	456	31	94	16	6	1
14346	7512	4326	4	928	714	27928	671	319	1029	157	94	251	128	183	273.27	247	436.54	446	437	415	19	81	19	8	3
11130	8100	5568	2	1966	1476	28586	546	73	621	19	310	168	43	293	317	108	463.66	458	213	389	93	89	2	36	19
11476	8018	5346	2	1884	1478	28542	571	103	623	26	302	171	54	258	313.92	126	459.81	468	226	377	98	84	5	38	18
5590	12454	8964	588	684	420	28156	1100	19	714	3	38	70	9	104	284.19	99	441.19	365	380	324	23	46	1	6	3
4494	13250	9594	813	480	270	28094	1196	10	602	1	6	72	7	85	273.76	94	428.94	333	296	295	11	52	1	0	3
5940	12042	8676	499	908	600	28252	1029	13	685	0	74	59	11	131	286.84	100	442.16	388	362	352	29	40	0	12	3
5048	12930	9338	707	494	266	28090	1126	17	671	5	14	71	11	80	277.27	109	435.41	353	345	338	22	41	0	0	3
4470	13246	9616	819	488	266	28092	1203	5	600	0	6	57	4	86	274.78	95	431.34	319	297	314	13	39	0	0	2
13952	7702	4468	2	988	766	27970	642	294	963	148	86	259	115	173	274.15	207	438.28	473	415	409	34	86	15	6	1
4238	13442	9760	872	400	222	28074	1236	3	587	0	12	57	2	73	275.97	94	429.44	314	278	297	16	42	0	0	0
4378	13334	9668	824	434	256	28094	1214	4	633	0	24	64	2	88	277.59	87	431.76	312	314	298	15	42	0	0	2
13228	7836	4764	3	1236	926	28136	613	237	859	68	140	183	121	190	283.82	183	447.49	494	337	450	45	85	15	6	3
14062	7770	4506	5	866	644	27916	619	295	1063	129	64	223	126	164	273.08	257	439.35	491	407	472	17	83	14	4	0
12154	7788	5160	2	1802	1266	28390	592	147	691	45	194	174	65	239	298.24	154	451.12	504	243	395	63	86	12	22	7
7094	11338	7996	367	1038	712	28266	954	34	738	8	80	87	22	142	288.93	115	446.16	421	372	349	34	48	0	8	3

SUPPLEMENTARY INFORMATION:Monte Carlo Atomistic Simulation and Machine Learning Analysis of Na-K Eutectic Alloy in Condensed Phases, D. Reitz and E. Blaisten-Barojas, George Mason University, Fairfax, VA 22030

4136	13482	9800	894	408	236	28080	1272	3	602	0	18	72	3	74	274.43	98	427.91	284	307	267	23	44	0	0	2
5366	12650	9112	585	622	364	28144	1081	9	714	0	30	55	4	101	279.19	96	435.15	373	378	361	20	35	0	0	1
4110	13516	9806	939	408	230	28072	1296	13	545	2	2	65	8	80	274.28	84	427.38	279	269	259	13	47	0	0	1
4504	13278	9598	829	442	254	28086	1230	8	630	0	10	53	6	66	274.86	98	429.49	325	314	301	15	41	0	0	3
4622	13214	9546	782	436	252	28086	1192	11	650	2	14	56	5	78	274.63	100	430.04	339	329	316	13	36	1	2	0
7218	11350	8010	345	968	618	28226	948	33	805	8	62	91	20	129	289.29	126	443.14	429	417	361	31	59	0	0	2
11332	7984	5384	1	1936	1558	28590	532	87	657	24	352	193	39	279	311.05	121	461.69	453	211	366	115	104	6	42	21
4922	12882	9334	716	600	364	28142	1152	7	625	0	38	73	7	81	280.45	96	437.83	353	329	310	27	49	0	2	2
3926	13596	9896	950	406	242	28084	1305	5	550	1	16	58	2	82	272.94	86	426.08	272	273	267	12	36	0	2	1
7308	11042	7896	359	1144	770	28286	887	45	683	9	112	88	24	155	289.63	120	445.53	430	337	375	45	52	0	14	2
4610	13258	9578	802	394	216	28066	1175	9	657	0	10	54	5	72	274.62	100	429.44	343	303	335	13	41	0	0	1
5168	12788	9212	641	594	350	28136	1114	8	706	1	24	64	6	87	278.67	113	433.91	391	364	328	9	34	0	0	3
13026	7806	4700	2	1356	1114	28202	578	213	798	77	188	196	103	205	282.38	179	447.14	497	296	451	48	85	17	12	10
3960	13672	9932	994	308	164	28040	1324	9	594	1	4	61	6	70	271.48	100	423.81	262	270	264	9	48	0	0	4
13766	7780	4552	5	1030	780	27996	611	289	969	135	84	234	121	176	275.64	236	438.03	499	376	442	26	81	12	4	2
3756	13712	10018	989	370	198	28062	1311	8	526	1	8	56	6	67	270.52	71	423.28	269	259	272	15	40	0	0	4
4344	13360	9702	844	434	230	28072	1238	7	627	0	2	73	5	70	274.01	95	428.08	312	318	278	15	47	0	0	6
4846	13004	9386	702	542	324	28124	1160	3	683	0	18	57	1	96	279.16	103	434.91	345	344	324	11	35	0	4	2
4600	13260	9576	782	414	222	28074	1211	10	670	2	2	67	5	77	273.75	106	429.69	330	346	295	12	41	0	0	1
4896	12904	9344	694	600	364	28144	1138	6	632	0	32	51	2	98	279.81	99	435.08	353	339	335	18	29	0	4	0
10964	7900	5578	0	2234	1630	28674	508	65	521	15	328	173	32	276	313.43	105	464.53	499	157	385	104	83	3	40	25
11246	8006	5468	1	1976	1526	28584	549	83	637	17	302	159	44	275	310.47	115	459.05	474	223	388	92	84	3	60	23
6622	11690	8326	381	966	592	28238	979	21	743	8	42	68	11	125	288.99	125	445.07	429	406	362	22	28	0	0	4
5482	12462	8996	595	744	452	28178	1067	11	692	4	40	80	5	125	285.01	102	440.85	379	344	324	17	47	0	2	1
12610	7748	4872	1	1600	1246	28308	594	151	786	51	208	187	71	209	293.42	185	451.62	540	284	384	48	95	13	20	9
14470	7642	4318	4	784	596	27864	628	349	1042	156	52	256	143	168	270.99	253	435.58	463	421	459	18	88	24	2	1
12786	7852	4748	1	1486	1198	28258	592	176	782	61	176	181	84	215	284.67	191	447.61	529	282	424	38	90	12	10	5
13006	8020	4766	6	1230	988	28144	619	202	896	61	130	196	109	187	280.44	237	444.54	508	330	437	37	95	17	4	4
11988	7936	5216	1	1742	1284	28422	548	135	721	35	238	166	78	275	301.48	140	453.31	482	241	443	60	86	8	18	8
10968	7958	5592	3	2184	1604	28680	513	84	566	14	344	155	48	267	320.28	121	462.73	507	178	381	95	84	4	28	36
13602	7580	4524	2	1286	972	28098	606	243	857	100	122	215	105	213	278.75	180	444.95	468	315	452	35	91	22	12	2
11360	7950	5286	5	1976	1628	28594	598	97	594	20	348	147	56	253	304.69	115	458.33	471	224	374	95	69	5	40	24
4346	13354	9688	854	430	248	28084	1226	7	607	1	18	50	4	61	272.47	105	425.39	331	290	308	15	32	0	0	3
4078	13618	9866	911	306	172	28050	1229	6	585	0	10	62	3	68	273.13	104	427.74	321	271	301	9	43	0	0	3
4244	13420	9728	859	428	252	28086	1244	4	591	0	14	51	3	76	271.91	77	424.73	306	304	304	14	32	0	0	1
4168	13450	9788	928	426	234	28076	1287	12	585	0	10	56	8	79	273.94	87	428.2	276	283	277	12	41	0	0	5

SUPPLEMENTARY INFORMATION:Monte Carlo Atomistic Simulation and Machine Learning Analysis of Na-K Eutectic Alloy in Condensed Phases, D. Reitz and E. Blaisten-Barojas, George Mason University, Fairfax, VA 22030

4858	12982	9374	728	544	334	28122	1157	4	645	0	28	60	3	95	277.75	94	435.21	336	338	325	18	42	0	0	1
5438	12624	9058	621	624	374	28144	1104	15	706	2	26	66	8	102	283.41	104	439.79	375	370	324	17	48	0	0	1
13234	7862	4694	3	1214	972	28132	596	214	888	87	146	201	93	208	282.09	197	444.41	480	324	450	42	91	17	8	5
3854	13736	9992	980	298	156	28040	1291	5	573	1	4	57	3	50	271.28	102	422.79	290	261	289	18	41	0	0	2
4318	13412	9710	840	390	228	28074	1208	3	618	0	16	67	2	71	274.21	100	429.58	317	303	311	17	42	0	0	4
4576	13272	9566	837	396	240	28066	1222	15	643	2	16	54	10	63	274.81	107	429.17	314	303	313	19	37	0	0	5
14286	7640	4378	6	874	656	27900	651	320	1018	170	62	262	112	158	271.6	258	436.41	470	423	425	24	81	16	4	3
7504	11116	7818	300	1026	698	28262	833	30	776	3	92	90	20	145	287.11	145	447.15	499	358	393	23	52	0	8	4
4600	13254	9534	784	420	264	28092	1191	14	632	2	20	60	10	82	279.43	98	434.1	332	330	318	12	29	0	0	2
11568	7798	5326	1	2016	1506	28554	535	97	599	16	298	169	58	275	310.93	103	462.23	490	194	380	90	97	5	42	18
12662	7862	4874	0	1480	1174	28270	561	154	801	53	202	209	63	249	291.82	183	450.22	491	277	406	49	107	11	16	8
5282	12746	9186	610	572	316	28116	1098	10	705	2	12	63	5	92	280.8	89	438.13	366	378	351	20	38	0	2	2
5744	12312	8866	539	760	456	28178	1064	15	715	3	36	74	7	96	283.63	114	441.28	411	381	318	23	39	0	4	2
11606	7816	5304	3	1994	1490	28538	545	114	610	23	296	182	62	267	310.83	115	460.91	476	200	382	100	100	3	30	16
11338	7944	5412	1	2034	1518	28566	540	83	610	24	282	174	39	262	314.91	112	460.68	495	201	372	100	85	4	34	19
4166	13444	9784	885	436	242	28084	1254	7	568	0	12	54	4	82	272.47	77	425.41	290	297	297	15	34	0	0	2
4150	13462	9794	906	416	238	28078	1266	2	605	0	18	72	1	72	273.41	100	426.13	299	285	268	17	48	0	0	2
12064	7748	5022	1	1908	1450	28456	553	143	637	39	242	175	72	257	297.86	135	454.35	515	217	392	59	88	13	22	18
13848	7776	4544	3	984	738	27970	645	278	970	139	74	262	97	173	275.18	231	440.17	483	390	402	23	103	19	6	5
14434	7628	4330	7	802	610	27864	658	329	1090	169	58	269	114	147	268.06	274	429.34	489	458	413	15	88	18	2	2
4784	13128	9436	724	468	276	28100	1185	7	655	0	8	61	5	92	279.27	85	434.44	320	361	323	14	39	0	0	2
13264	7760	4670	1	1282	1006	28144	612	214	866	61	146	194	107	196	282.95	206	444.72	506	313	431	39	105	18	16	2
13980	7790	4544	4	914	654	27938	651	283	1037	124	52	237	126	169	276.22	217	438.59	467	422	441	22	101	14	4	3
12718	7848	4860	1	1460	1166	28274	576	199	770	57	192	196	108	230	287.76	149	449.08	487	303	433	56	97	17	28	6
5536	12650	9078	632	516	288	28088	1091	26	719	5	20	74	17	91	281.75	107	436.83	362	360	351	18	49	1	0	4
12524	7756	4998	0	1638	1186	28314	575	166	735	50	196	172	78	230	299.92	140	454.01	489	276	427	62	78	13	16	12
14392	7686	4400	8	782	552	27844	615	339	1077	165	30	267	136	154	269.64	271	432.41	491	433	451	16	89	23	2	0
13040	7720	4752	1	1414	1092	28212	608	205	824	63	176	191	107	218	286.31	185	448.23	492	327	424	37	96	16	18	9
4370	13382	9690	858	398	222	28070	1222	8	608	1	6	59	3	67	274.42	101	428.61	313	302	309	20	42	0	2	3
3880	13720	9956	990	292	180	28048	1301	8	575	1	20	58	4	61	270.36	104	423.65	291	264	266	12	37	0	0	2
4714	12986	9452	705	624	348	28142	1159	5	624	0	16	58	3	100	279.24	84	434.13	339	340	321	17	38	0	2	1
14376	7646	4354	8	814	620	27874	629	315	1056	172	62	259	112	155	268.53	268	429.16	501	429	427	15	76	12	2	1
5892	12320	8818	518	706	406	28162	1042	9	777	0	20	71	7	108	281.95	125	440.08	419	407	344	9	54	0	0	1
5042	12954	9312	687	486	288	28104	1166	5	728	0	22	57	4	62	277.55	117	435.32	357	375	329	22	39	0	0	3
12074	7750	5148	0	1820	1358	28446	590	133	647	29	266	148	65	256	299.95	138	453.98	464	234	414	76	79	13	28	16
13236	7668	4638	0	1408	1076	28184	600	229	786	82	144	202	105	203	283.31	193	446.12	499	296	421	48	80	19	12	4

SUPPLEMENTARY INFORMATION:Monte Carlo Atomistic Simulation and Machine Learning Analysis of Na-K Eutectic Alloy in Condensed Phases, D. Reitz and E. Blaisten-Barojas, George Mason University, Fairfax, VA 22030

3948	13674	9928	977	312	176	28048	1305	11	582	1	10	64	9	67	272.22	97	424.8	271	272	273	14	44	0	0	3
4832	13024	9444	729	502	274	28096	1156	12	655	0	20	67	9	99	278	95	434.34	319	338	331	18	38	0	0	3
4148	13568	9840	911	318	166	28044	1261	6	597	1	4	66	3	77	272	102	425.61	293	284	285	9	42	0	0	2
4598	13174	9534	782	500	288	28108	1190	7	626	0	12	48	4	68	276.13	105	431.35	340	311	322	22	29	0	2	2
4588	13212	9578	791	444	250	28096	1190	6	630	0	22	67	5	79	278.45	86	433.31	329	325	308	22	45	0	2	0
4394	13336	9668	833	426	244	28086	1239	5	622	0	18	64	2	76	276.7	93	431.35	314	321	285	13	37	0	0	3
4240	13436	9744	886	402	238	28080	1241	6	596	0	20	68	5	72	272.65	101	426.36	307	281	289	16	42	0	0	4
14158	7656	4442	8	910	676	27920	657	318	998	159	74	253	115	148	271.64	253	435.86	499	418	414	22	77	20	4	0
4156	13586	9854	925	292	144	28034	1291	12	615	2	2	65	7	70	273.26	89	426.46	285	314	273	10	49	0	0	0
4968	12926	9290	701	572	352	28124	1151	6	671	1	16	65	3	92	277.52	105	435.21	348	342	316	17	35	0	0	3
14264	7740	4434	3	814	588	27880	627	325	1060	137	38	232	145	162	271.06	251	434.92	471	422	478	15	83	24	2	4
4234	13392	9722	874	458	270	28090	1264	5	574	0	12	60	2	77	276.51	73	431.29	287	296	281	18	38	0	2	5
7338	11250	7914	371	970	664	28228	953	42	745	11	90	94	24	151	287.97	121	445.52	389	373	366	32	52	2	0	6
14260	7748	4462	7	768	568	27864	648	330	1078	159	54	254	134	149	268.69	277	433.16	486	450	434	17	82	17	4	1
11330	7982	5378	1	1990	1560	28606	562	86	607	19	324	158	44	275	311.48	129	460.07	504	194	366	80	80	3	40	22
4174	13408	9748	867	480	278	28100	1235	5	554	1	12	48	2	83	274.56	87	429.41	307	275	300	15	31	0	0	2
14376	7708	4436	10	746	530	27842	643	351	1071	171	46	272	138	153	267.63	276	429.67	481	435	425	17	92	19	0	0
12398	7786	4992	1	1638	1268	28346	552	153	728	46	244	191	74	232	300.85	165	452.9	487	229	415	75	89	11	20	16
4158	13562	9820	912	322	180	28050	1265	8	614	1	8	63	6	77	272.12	97	426.16	288	295	288	12	44	0	0	1
14426	7660	4340	4	788	598	27868	648	323	1059	167	56	265	126	150	268.72	270	431.14	483	423	431	18	91	12	0	1
4212	13502	9778	917	354	202	28058	1280	6	605	1	10	74	3	63	273.35	92	427.98	298	302	263	15	50	0	0	3
4114	13580	9852	932	312	174	28046	1271	6	610	0	14	73	6	75	270.83	91	424.31	284	287	276	14	52	0	0	2
14270	7772	4416	2	798	588	27878	648	317	1070	161	34	253	128	172	269.27	261	431.91	465	446	447	10	81	17	0	1
12890	7928	4922	7	1300	962	28156	625	203	893	61	144	178	108	178	280	195	445.75	506	340	443	46	92	12	8	3
4194	13422	9766	886	428	250	28084	1258	6	602	1	24	64	2	79	273.74	102	428.95	297	299	278	13	45	0	0	3
4592	13270	9574	805	390	230	28076	1212	9	642	0	20	49	7	78	274.71	88	429.19	306	339	330	14	34	0	0	5
4616	13198	9520	779	472	282	28102	1206	5	650	0	12	65	3	86	275.85	95	430.76	325	343	301	11	47	0	2	3
6708	11748	8312	408	844	542	28210	993	20	774	6	54	77	8	125	286.79	122	444.59	404	398	356	29	45	0	2	4
6974	11440	8116	392	976	642	28224	956	36	725	10	72	101	23	140	287.64	120	446.46	406	373	349	33	50	0	4	5
6816	11722	8320	434	790	470	28154	996	30	803	10	32	89	14	113	285.34	126	443.74	403	397	353	30	58	0	4	1
13726	7790	4582	5	1026	778	28000	625	268	960	115	96	228	122	170	277.11	222	439.9	463	385	462	44	88	17	2	1
4522	13238	9570	817	474	280	28098	1211	8	623	0	14	54	6	86	274.6	89	430.5	314	316	310	14	38	0	0	3
11506	7922	5368	0	1932	1468	28532	553	108	623	20	298	162	62	276	306.83	130	454.46	485	210	395	73	81	7	36	21
4582	13082	9520	784	594	336	28132	1198	5	594	1	18	55	4	90	278.3	84	433.13	319	307	315	20	35	0	0	1
3872	13690	9960	1006	334	188	28054	1309	7	550	1	8	51	4	59	270.81	86	422.24	287	253	276	10	34	0	2	3
12170	7826	5120	1	1746	1278	28380	596	156	696	40	222	161	84	231	302.1	139	451.35	481	268	415	71	79	10	18	14

SUPPLEMENTARY INFORMATION:Monte Carlo Atomistic Simulation and Machine Learning Analysis of Na-K Eutectic Alloy in Condensed Phases, D. Reitz and E. Blaisten-Barojas, George Mason University, Fairfax, VA 22030

11428	7778	5262	1	2118	1664	28640	530	92	555	22	350	166	49	266	312.27	121	459.1	513	185	371	99	82	2	34	24
11352	7860	5478	0	2050	1498	28594	520	101	581	19	320	173	52	280	318.67	108	460.48	473	188	367	115	95	5	32	19
3792	13726	9996	1013	338	192	28054	1323	5	535	1	8	65	4	59	270.43	78	422.35	270	259	263	14	42	0	2	5
4534	13270	9606	783	422	238	28088	1188	6	641	1	16	50	4	76	275.91	107	431.73	340	313	325	14	34	0	2	0
4818	13096	9440	751	458	268	28102	1162	6	658	0	22	58	5	81	276.24	103	433.28	339	319	334	17	41	0	0	4
13068	7862	4722	3	1320	1040	28160	563	200	874	87	132	217	90	206	282.68	207	445.31	504	299	445	52	107	12	16	1
11872	7804	5124	0	1938	1482	28508	545	116	623	33	244	178	48	254	308.6	145	457.13	509	211	368	82	92	11	36	24
14088	7760	4530	4	868	604	27896	637	324	1028	160	46	264	121	166	273.98	237	438.62	476	416	424	21	92	18	0	2
14080	7670	4436	4	950	724	27946	621	299	999	160	84	255	104	167	274.06	239	439.06	490	398	432	20	82	15	2	7
13890	7762	4548	3	954	726	27980	623	285	992	129	90	231	118	174	276.47	213	442.66	464	390	461	32	90	19	10	6
3982	13646	9896	944	332	192	28056	1285	9	579	1	6	59	6	67	270.71	87	424.78	281	284	280	17	34	0	2	3
5248	12610	9138	643	708	420	28162	1093	7	654	0	38	67	3	102	281.45	121	438.04	378	316	329	23	51	0	0	0
11752	7850	5254	0	1922	1428	28508	528	103	673	22	286	167	43	253	316.64	148	458.59	500	217	397	96	87	13	14	15
4550	13190	9566	785	498	284	28106	1195	5	641	0	18	56	5	80	276.54	106	432.93	331	320	313	18	37	0	0	1
5462	12458	8986	579	748	478	28192	1089	11	677	2	60	69	7	96	282.25	102	438.7	408	354	313	15	47	0	0	4
14384	7618	4372	6	838	610	27880	634	320	1051	159	54	253	120	140	270.43	286	433.02	506	414	440	18	80	24	4	2
8046	10862	7594	288	958	644	28206	886	63	810	15	98	111	34	139	287.92	136	446.83	441	404	369	32	72	2	4	7
11244	7872	5456	4	2116	1578	28630	511	99	568	23	324	184	44	276	318.48	113	463.67	485	171	378	103	98	12	34	26
14084	7612	4440	2	968	748	27970	625	316	992	132	114	253	141	183	276.85	231	440.91	481	388	426	24	106	23	4	1
4430	13432	9666	861	320	198	28062	1246	6	664	0	12	62	5	67	274.06	101	429.06	306	324	299	14	48	0	4	3
11892	8076	5206	5	1698	1294	28390	584	122	724	27	190	166	67	228	294.11	152	450.8	489	248	427	72	100	9	30	13
13674	7908	4648	6	940	718	27976	629	267	1017	124	80	234	105	162	275.91	251	440.16	499	390	427	30	101	17	8	1
11106	8016	5506	1	2030	1588	28648	511	79	571	13	358	179	44	290	318.83	89	462.72	457	164	374	116	95	4	40	26
3950	13572	9904	966	410	224	28070	1293	7	574	1	10	57	5	83	270.5	90	423.24	266	259	281	12	41	0	0	3
7654	11036	7728	356	1046	692	28226	905	50	709	11	70	89	32	136	289.19	134	446.22	448	333	375	29	56	1	0	3
6360	11834	8476	455	928	572	28220	986	16	734	4	50	77	8	127	284.82	121	443.65	414	371	362	25	47	0	0	3
4098	13580	9828	923	340	202	28058	1282	8	599	2	10	61	4	73	270.4	90	423	279	305	283	14	34	0	0	3
4612	13192	9558	769	462	254	28092	1181	7	644	0	14	64	5	81	277.72	111	433.65	347	306	306	11	50	0	0	2
4950	12930	9314	678	564	342	28120	1117	6	666	0	20	65	5	103	278.85	94	435.09	334	339	348	25	47	0	0	1
4456	13336	9622	814	406	246	28082	1206	8	618	0	16	54	6	72	274.1	91	430.07	323	306	323	14	36	0	0	4
12406	7844	5028	0	1630	1212	28346	567	150	762	53	210	190	69	247	295.01	170	450.68	519	270	395	50	99	7	16	8
4702	13014	9438	752	596	360	28140	1162	8	625	1	28	66	4	91	280.09	97	435.48	346	309	308	21	42	0	2	1
4556	13194	9562	761	492	284	28108	1187	3	643	0	20	56	3	85	276.18	90	431.78	337	341	313	13	32	0	0	2
4346	13380	9688	830	420	240	28084	1212	2	618	0	8	62	1	85	274.92	88	428.51	314	305	306	12	42	0	2	3
5006	13062	9392	742	384	208	28066	1170	11	730	0	12	55	7	88	275.35	121	430.62	330	369	337	10	44	0	2	1
5660	12252	8888	555	866	500	28200	1061	19	629	3	32	78	12	117	284.75	78	441.59	376	347	329	25	49	1	2	6

SUPPLEMENTARY INFORMATION:Monte Carlo Atomistic Simulation and Machine Learning Analysis of Na-K Eutectic Alloy in Condensed Phases, D. Reitz and E. Blaisten-Barojas, George Mason University, Fairfax, VA 22030

5174	12706	9152	622	666	424	28164	1109	6	674	1	42	64	5	85	280.5	104	438	399	368	317	15	45	0	0	3
4750	13174	9496	796	400	234	28076	1202	8	673	1	22	67	4	65	276.05	110	431.8	338	328	303	14	51	0	0	4
4392	13388	9698	841	380	200	28062	1238	6	638	2	4	58	3	67	272.92	106	426.57	318	334	298	11	38	0	0	3
4504	13172	9588	794	538	294	28108	1187	4	621	0	12	44	1	85	275.38	93	431.02	322	300	331	19	28	0	0	2
4452	13348	9674	843	376	200	28062	1226	4	667	0	12	58	2	69	276.75	117	431.66	323	313	302	12	40	0	0	2
4046	13644	9880	955	306	168	28046	1293	7	577	1	2	56	4	63	273.15	89	425.54	276	278	288	17	36	0	0	2
11264	7872	5370	0	2108	1642	28642	525	97	591	21	342	169	51	270	317.01	114	459.95	490	189	377	118	99	9	38	16
14384	7614	4370	2	846	612	27882	629	329	1045	157	54	259	138	147	270.57	284	433.05	507	421	432	17	89	17	2	1
14008	7678	4464	2	990	740	27960	618	299	978	127	74	246	130	178	275.74	215	440.53	465	392	448	30	99	22	6	5
10530	7898	5684	0	2396	1844	28840	484	55	455	7	434	149	28	282	334.09	90	472.17	517	141	363	128	70	5	50	35
10488	7902	5676	0	2380	1888	28864	499	60	439	10	470	163	31	304	333.62	77	471.08	482	137	336	139	71	6	56	29
11624	7912	5370	3	1906	1388	28492	525	116	635	21	256	177	65	267	308.65	139	459.53	498	191	383	94	97	6	34	13
4384	13458	9728	848	308	160	28044	1220	3	658	0	6	73	2	74	271.98	108	425.56	316	319	299	12	54	0	0	1
13494	7898	4654	3	1034	828	28034	600	251	961	126	126	229	98	175	276.64	217	443.15	489	374	459	38	76	9	0	4
14374	7648	4434	6	790	552	27852	628	324	1082	159	54	262	131	140	270.66	281	433.59	506	437	443	16	90	16	0	0
4830	13094	9422	706	466	276	28106	1149	7	696	0	18	61	5	89	277.54	116	432.72	359	354	319	12	44	0	0	1
5050	12906	9276	687	546	326	28120	1150	5	660	1	14	67	2	96	280.73	96	437.31	332	351	328	21	43	0	2	2
5410	12660	9080	595	616	360	28142	1092	9	733	1	14	59	4	101	279.74	113	435.81	373	374	345	20	41	1	2	0
4256	13378	9756	878	442	234	28080	1239	8	590	2	14	63	4	72	272.34	87	426.76	320	292	287	12	47	0	0	2
4628	13142	9538	775	498	278	28104	1177	6	627	0	20	62	4	91	279.88	97	435.05	322	300	320	19	47	0	0	2
13886	7796	4534	5	934	718	27950	640	294	998	146	76	247	113	174	274.63	250	438.76	476	400	423	25	83	15	2	2
7364	11260	7960	371	940	604	28202	946	46	765	11	66	104	23	139	288.88	106	445.78	418	393	347	32	63	2	8	1
4540	13224	9576	808	472	268	28092	1206	10	626	2	12	60	7	74	274.7	93	429.35	330	309	307	16	41	0	0	2
3934	13590	9904	983	402	228	28070	1307	10	558	1	12	58	7	69	270.19	90	423.65	281	272	265	13	34	0	0	2
4656	13228	9492	780	410	276	28090	1206	10	645	0	26	61	7	80	278.16	98	436.24	315	342	307	23	42	0	2	0
4306	13362	9700	853	436	262	28094	1237	7	585	1	28	57	5	74	274.62	81	428.65	317	309	292	15	39	0	0	2
4568	13062	9500	750	620	372	28152	1172	5	601	0	28	57	4	84	278.67	94	435.86	359	308	309	14	38	0	0	3
12538	7978	4942	3	1434	1160	28280	553	157	775	56	216	196	65	240	291.84	170	451.2	478	261	435	60	86	11	12	12
12348	7824	4978	4	1636	1298	28354	581	170	719	46	248	179	83	240	296.79	155	454.53	478	248	410	66	88	11	22	11
14248	7594	4438	10	890	648	27906	623	293	1039	158	86	268	103	160	272.44	264	435.92	494	415	427	22	93	15	2	1
4192	13528	9788	896	342	198	28058	1246	7	597	2	10	63	5	73	273.08	101	427.52	307	287	289	12	41	0	0	2
13104	7746	4772	5	1380	1024	28190	629	204	825	64	152	180	101	192	284.51	171	449.04	496	313	436	42	95	16	12	8
7122	11290	8062	393	996	664	28252	927	38	709	12	112	85	23	125	290.99	131	444.23	450	354	358	34	47	1	6	6
14454	7638	4376	7	764	562	27858	639	343	1076	166	64	256	141	155	270.79	267	433.99	470	439	457	17	81	18	0	2
11176	8114	5536	1	1930	1464	28558	518	79	644	17	308	162	39	271	314.59	145	457.77	485	191	391	96	93	2	26	23
14240	7744	4420	8	788	616	27878	642	308	1077	155	70	260	119	143	270.15	266	432.48	503	443	428	17	92	16	0	1

12482	7736	4958	2	1696	1264	28366	538	150	694	56	198	220	68	252	299.84	147	454.1	471	235	401	74	107	7	30	17
4078	13568	9848	926	352	202	28062	1307	8	606	1	14	68	6	68	271.59	93	424.83	277	307	260	14	46	0	0	3
11760	7798	5232	1	1982	1466	28540	566	105	605	17	270	149	62	246	310.99	118	459.92	479	215	406	92	82	8	28	22
4856	12996	9372	728	548	332	28128	1168	8	650	1	24	62	2	74	279.51	112	435.35	356	335	307	21	40	0	0	4
4914	12966	9370	698	542	308	28118	1139	9	664	2	18	62	6	88	277.43	93	431.78	353	336	335	16	46	0	0	3
4680	13118	9488	759	508	296	28108	1189	9	652	0	18	55	6	94	277.5	95	433.15	310	335	327	18	37	0	0	2
4106	13502	9808	899	398	244	28086	1240	5	581	0	28	58	4	70	274.81	88	429.23	308	277	299	19	39	0	0	2
14340	7600	4332	5	880	680	27912	652	323	984	159	76	262	120	171	272.72	231	436.97	471	412	420	18	90	24	4	1
3942	13594	9894	950	404	230	28072	1280	10	548	1	8	57	6	80	272	84	425.24	275	260	283	17	38	0	0	1
4836	13012	9408	722	538	306	28116	1157	6	658	0	16	70	3	95	279.84	94	435.24	333	328	320	20	50	0	0	0
14302	7584	4348	2	916	688	27910	634	316	1004	155	70	268	119	152	271.82	243	434.88	485	398	421	33	102	18	2	0
10732	7798	5630	3	2432	1780	28798	508	67	511	7	382	151	30	333	333.66	78	472.49	465	174	340	116	84	1	40	30
13904	7750	4456	3	1014	794	28006	622	233	1000	98	82	215	104	173	276.84	226	444.25	485	393	463	30	96	10	4	2
5242	12566	9118	619	752	458	28186	1110	7	660	1	50	65	4	95	285.53	98	441.2	388	346	308	22	35	0	0	1
13726	7730	4572	2	1036	816	28016	609	291	943	137	126	249	127	166	278.4	232	442.92	520	391	415	30	95	12	10	3
13062	7838	4702	1	1352	1074	28186	597	177	853	61	140	197	89	227	284.86	185	446.51	474	306	442	38	102	11	16	8
3678	13814	10070	1031	306	168	28044	1333	6	545	1	8	59	4	62	269.91	81	422.63	263	254	254	17	39	0	0	2
7024	11438	8138	347	946	604	28230	906	26	767	4	76	90	14	148	288.69	130	444.06	451	374	372	20	56	1	2	2
4758	13280	9534	806	294	164	28042	1181	5	714	0	12	67	4	62	273.32	112	427.43	329	328	338	17	54	0	0	4
4392	13378	9684	860	378	218	28068	1245	7	639	1	18	61	5	77	272.97	92	427.61	297	318	297	14	42	0	0	3
3994	13648	9926	961	312	158	28042	1280	11	576	1	4	56	6	70	272.59	93	425.76	281	263	293	12	38	0	0	2
4180	13544	9798	887	330	194	28060	1264	7	621	0	14	54	4	71	273.47	88	426.3	291	322	299	14	39	0	0	2
14108	7772	4416	4	874	690	27926	654	304	1016	157	62	246	114	158	269.56	251	433.83	490	434	426	17	72	15	4	2
4448	13412	9652	821	336	204	28066	1205	4	646	0	14	58	3	64	274.8	118	429.23	339	314	313	14	36	0	0	1
4362	13334	9672	844	448	258	28090	1254	5	593	1	16	59	3	78	276.74	75	431.06	295	312	290	14	31	0	0	5
3988	13696	9916	958	272	156	28036	1289	13	592	1	8	64	7	60	271.07	93	424.11	287	278	276	14	42	0	0	3
3950	13680	9946	991	296	158	28040	1324	13	590	2	10	58	7	67	270.7	89	422.4	262	289	268	14	41	0	0	1
14294	7686	4408	8	804	612	27872	635	323	1058	162	62	268	126	161	271.35	269	434.65	486	418	427	16	93	16	6	0
14558	7708	4352	2	670	496	27820	661	353	1111	169	28	261	140	135	267.74	282	430.05	478	462	440	17	86	21	8	1
4164	13544	9812	929	340	188	28054	1283	7	616	1	6	68	5	73	272.5	92	426.12	275	302	278	17	46	0	0	2
12670	7872	4950	8	1448	1096	28232	628	172	839	60	178	185	79	204	285.93	187	449.55	483	305	417	56	102	12	18	6
11336	7926	5436	1	2022	1510	28574	531	87	621	16	320	180	52	296	308.15	127	457.52	486	200	385	83	103	7	20	16
11700	7884	5344	2	1884	1376	28484	536	103	647	22	262	174	54	261	311.26	117	460.34	478	218	392	82	93	3	28	27
6068	11940	8660	487	936	560	28228	1009	17	690	2	60	78	11	119	285.6	95	441.21	411	360	346	26	49	0	4	3
10930	7860	5562	3	2228	1692	28692	527	86	550	13	364	165	47	310	320.38	95	462.76	448	170	377	109	85	7	50	24
11476	7842	5316	1	2008	1568	28590	542	121	563	23	338	153	60	279	313.18	95	459.61	453	187	420	97	74	10	40	21

SUPPLEMENTARY INFORMATION:Monte Carlo Atomistic Simulation and Machine Learning Analysis of Na-K Eutectic Alloy in Condensed Phases, D. Reitz and E. Blaisten-Barojas, George Mason University, Fairfax, VA 22030

4696	13016	9452	722	592	352	28140	1162	5	621	0	32	54	3	92	278.93	98	434.85	343	327	320	19	35	0	0	2
11548	7890	5310	1	1962	1502	28546	563	117	613	29	292	181	62	279	304.38	116	455.55	467	206	377	88	101	8	40	17
11282	8074	5404	3	1902	1536	28576	537	97	645	24	340	174	40	269	309.62	134	460.8	486	204	370	98	87	4	36	22
11100	7928	5432	0	2122	1676	28672	500	89	534	11	374	175	46	294	319.38	99	461.15	463	167	379	113	84	5	38	30
12668	7806	4916	5	1562	1144	28270	573	180	792	61	154	179	93	203	287.58	160	447.78	508	284	454	66	94	6	18	2
4470	13290	9600	807	434	276	28102	1203	3	635	0	32	60	1	78	274.82	98	429.29	334	312	304	12	40	0	0	3
14166	7646	4406	5	930	698	27914	619	316	1007	157	62	243	123	163	268.74	256	429.81	493	402	451	20	72	17	6	1
4932	12958	9378	675	524	296	28114	1142	5	714	0	26	61	4	74	275.72	106	432.08	363	373	332	20	43	0	0	3
12586	7834	4906	6	1580	1190	28280	593	179	759	58	164	183	90	203	285.98	169	450.9	524	283	418	56	98	12	20	4
14292	7728	4456	7	774	556	27854	627	328	1090	166	46	272	119	146	270.86	287	432.69	504	424	435	13	94	20	2	1
4536	13228	9564	767	452	288	28106	1187	2	620	0	38	58	1	98	275.35	93	429.72	311	330	327	13	38	0	0	3
14022	7718	4496	6	924	704	27962	639	276	1012	126	92	224	115	176	275.25	231	440.61	457	411	459	33	80	21	6	0
14516	7564	4364	5	808	552	27850	634	330	1069	155	46	265	139	152	272.02	265	435.47	471	431	447	23	100	14	0	1
4110	13594	9826	928	330	192	28056	1271	11	585	2	4	56	8	62	273.61	89	427.72	294	282	296	17	39	0	0	1
11024	7842	5464	1	2254	1714	28696	513	80	521	13	356	160	33	280	317.24	111	463.16	495	163	365	107	85	11	40	30
4508	13192	9610	795	502	272	28104	1209	4	622	1	18	70	2	79	276.73	82	431.06	324	321	298	20	45	1	2	0
11194	7818	5484	0	2168	1608	28656	556	93	543	19	342	148	47	261	316.61	105	463.71	454	197	390	130	71	6	34	16
4008	13640	9886	953	320	188	28054	1295	6	566	1	12	60	2	70	272.88	84	425.68	281	277	272	14	47	0	0	1
13020	7692	4738	2	1450	1126	28230	583	214	777	76	190	191	104	218	283.05	172	447.66	499	292	440	46	88	18	12	7
14094	7836	4520	3	798	590	27890	634	321	1060	149	50	241	128	154	273.44	271	439.05	487	423	457	18	81	20	2	1
11714	8080	5254	2	1752	1376	28454	559	107	665	29	238	163	52	269	306.07	136	455.16	495	244	394	63	89	9	38	16
11866	7956	5236	2	1774	1338	28442	566	112	688	31	242	196	52	261	303.36	131	457.42	464	229	379	89	110	11	26	13
5748	12152	8762	554	924	592	28236	1065	17	615	6	50	77	6	104	285.86	101	442.76	404	325	307	31	43	0	8	4
12262	7820	5066	2	1708	1270	28366	563	147	720	35	224	170	80	258	297.91	158	452	457	251	445	65	94	14	16	10
12826	7872	4852	0	1440	1076	28224	558	184	780	41	156	190	94	221	292.2	175	447.57	497	263	451	53	106	21	2	7
10532	7824	5532	1	2434	2010	28890	499	65	446	16	490	155	27	309	330.94	81	469.05	471	145	349	119	71	3	60	44
13714	7412	4456	2	1358	1026	28124	630	278	796	120	132	215	111	195	278.61	173	445.26	482	336	418	42	75	15	22	4
11758	7774	5236	0	1942	1492	28562	543	113	634	23	316	168	64	271	310.43	118	457.42	482	206	389	98	96	10	44	16
14232	7660	4406	7	906	652	27902	618	314	1016	164	44	257	120	161	269.28	260	432.54	494	395	445	20	82	11	2	0
6936	11586	8176	364	856	586	28226	943	22	807	1	72	83	17	123	287.47	144	443.76	448	410	360	28	52	1	10	3
11198	7754	5280	0	2252	1810	28734	520	79	495	8	384	157	52	292	318.75	82	462.26	456	167	393	117	82	4	56	33
4046	13604	9872	952	340	186	28052	1285	4	586	0	4	60	4	67	271.68	94	424.53	286	281	283	13	45	0	0	2
13248	7670	4638	0	1380	1068	28170	587	216	808	69	150	189	103	213	282.71	173	446.31	455	285	466	52	91	16	16	12
13868	7840	4576	8	912	676	27936	604	289	1014	134	58	240	117	158	275.05	265	440.23	500	370	459	27	86	22	4	2
5100	12740	9208	657	680	404	28156	1127	6	663	1	24	72	2	92	281.04	104	437.39	385	339	302	13	49	0	0	3
14668	7474	4240	4	804	600	27858	630	374	1039	172	72	264	159	153	270.2	264	432.27	487	433	442	18	81	20	0	0

SUPPLEMENTARY INFORMATION:Monte Carlo Atomistic Simulation and Machine Learning Analysis of Na-K Eutectic Alloy in Condensed Phases, D. Reitz and E. Blaisten-Barojas, George Mason University, Fairfax, VA 22030

12074	7898	5124	1	1718	1324	28412	583	130	719	40	248	170	60	241	300.88	163	454.99	489	260	392	78	82	2	24	12
4658	13082	9514	749	540	298	28112	1165	7	637	0	20	57	4	72	278.8	90	433.83	353	326	323	19	43	0	0	4
10816	7972	5670	2	2224	1622	28678	507	59	585	11	328	173	34	289	314.88	121	464.48	508	167	352	96	89	1	42	27
14206	7658	4412	7	908	668	27912	628	320	997	155	54	263	125	146	270.41	266	435.65	511	402	422	21	96	19	6	3
4156	13532	9810	917	340	202	28060	1266	11	590	1	18	48	5	72	272.87	90	425.26	295	284	297	10	35	0	2	3
6510	11768	8400	415	912	578	28234	989	25	742	6	66	85	17	125	285.58	117	443.22	424	389	342	26	46	0	0	3
4686	13084	9460	770	536	332	28130	1207	4	634	0	30	60	3	80	277.9	94	432.41	338	333	291	18	30	0	2	1
4528	13278	9626	806	400	220	28072	1214	10	653	2	20	49	4	77	275.84	96	430.38	306	343	331	16	33	0	0	2
10834	7964	5680	1	2238	1614	28704	498	69	532	7	326	158	44	276	329.91	107	469.77	489	161	371	127	87	3	46	28
10694	7798	5586	3	2410	1842	28794	540	67	450	12	410	156	30	312	324.7	75	468.56	472	163	340	111	57	5	54	28
5206	12668	9186	653	678	384	28148	1125	5	677	2	26	69	2	93	280.59	110	434.77	357	347	327	18	47	0	0	7
3980	13636	9908	950	332	188	28058	1285	8	582	1	14	52	4	70	272.13	83	425.64	277	285	292	17	39	0	0	1
3848	13754	9980	994	288	164	28040	1300	10	565	1	6	59	6	62	270.44	101	423.18	277	256	277	17	40	0	0	1
4762	13018	9422	734	570	336	28130	1188	4	626	0	22	56	3	80	278.48	89	435.56	342	337	312	15	36	0	0	4
4458	13280	9634	838	454	248	28082	1218	7	636	1	8	65	6	74	275.7	116	429.77	310	302	310	20	45	0	0	2
13140	7814	4782	7	1294	970	28156	601	228	842	70	148	179	120	202	282.49	190	448.27	490	326	464	43	80	14	8	3
4260	13364	9718	868	478	266	28094	1249	6	601	0	8	57	5	79	272.94	101	428.16	312	299	281	13	43	0	0	1
4730	12996	9438	738	582	352	28138	1150	6	614	0	40	65	4	92	279.3	101	434.96	362	311	308	13	40	0	0	3
12058	7810	5122	0	1782	1368	28440	553	131	670	35	278	175	68	253	298.84	137	454.27	493	237	413	78	93	10	20	11
13292	7722	4664	2	1296	998	28128	632	222	834	82	150	207	107	206	283	192	446.25	480	334	407	38	92	14	6	3
11560	7742	5296	1	2078	1544	28556	513	126	558	27	306	164	66	266	312.43	107	459.07	477	185	413	100	88	5	28	25
7118	11250	8002	371	1062	726	28272	914	33	728	4	106	93	21	145	286.55	119	445.03	437	330	355	33	55	1	6	8
7440	11212	7870	340	940	664	28238	852	31	771	6	104	92	21	139	287.29	147	445.55	455	345	401	41	57	0	8	4
4170	13434	9766	883	440	258	28086	1254	10	571	1	18	49	5	74	272.08	86	425.48	303	288	298	8	32	0	0	7
6188	12026	8608	481	818	504	28190	999	21	738	5	40	81	12	124	286.33	110	444.3	399	382	363	26	53	1	6	2
12384	7822	5080	2	1622	1190	28334	541	159	722	51	218	193	77	247	300.22	154	452.19	486	236	417	75	103	6	16	8
11180	7760	5444	1	2224	1656	28646	494	101	572	17	340	145	56	275	312.54	110	460.85	491	183	416	98	72	7	36	31
14334	7730	4432	5	760	552	27854	657	326	1077	168	44	271	119	158	267.51	271	429.88	470	444	420	16	91	20	2	1
12014	7826	5172	2	1802	1348	28442	597	121	677	26	244	169	70	239	303.25	136	455.09	486	251	385	71	95	3	34	17
4748	13150	9474	770	444	256	28082	1185	12	661	1	10	54	6	84	275.83	95	430.77	334	333	318	10	39	0	0	3
12602	7918	4940	1	1498	1130	28266	620	172	795	49	160	175	91	220	287.29	158	446.72	454	314	437	61	88	14	18	10
4758	13072	9486	757	498	268	28100	1170	5	655	1	18	58	2	79	278.61	98	433.84	348	316	321	15	43	1	0	2
13444	7772	4632	3	1194	920	28094	608	238	894	95	122	203	103	204	278.32	207	443.13	470	332	456	38	89	11	10	4
5454	12470	9000	588	748	462	28180	1076	13	657	4	46	67	6	112	281.85	102	437.7	384	339	337	18	38	0	0	1
4002	13616	9878	947	350	204	28060	1304	4	565	2	10	54	2	63	270.04	80	423.31	277	296	281	17	27	0	0	1
5288	12808	9208	650	496	276	28094	1117	9	717	1	18	64	5	103	282.17	106	437.67	356	368	335	12	40	0	0	0

SUPPLEMENTARY INFORMATION:Monte Carlo Atomistic Simulation and Machine Learning Analysis of Na-K Eutectic Alloy in Condensed Phases, D. Reitz and E. Blaisten-Barojas, George Mason University, Fairfax, VA 22030

4098	13548	9850	933	366	192	28058	1286	9	594	1	4	53	5	68	272.15	85	425.17	281	293	290	15	35	0	0	2
3910	13722	9964	984	278	156	28044	1286	6	595	1	14	56	3	63	270.66	115	423.66	290	260	285	13	39	0	0	1
3882	13666	9938	958	356	210	28068	1281	10	552	2	16	53	6	57	272.27	96	426.85	295	256	287	21	36	0	0	1
14482	7648	4344	3	752	568	27854	654	326	1091	158	58	257	123	140	267.6	294	429.52	484	446	435	18	88	21	2	3
4002	13702	9934	997	248	130	28022	1321	9	613	1	6	62	5	64	270.05	89	421.7	264	284	270	10	48	0	0	3
12354	8032	5134	1	1486	1086	28280	617	142	832	39	170	171	76	228	292.3	187	452.82	470	304	421	62	96	9	18	4
13658	7850	4638	4	1000	756	28000	608	270	988	120	94	245	119	165	277.19	253	443.83	512	385	426	31	103	12	2	3
5624	12388	8900	593	760	468	28172	1099	16	670	1	28	60	10	109	281.35	96	437.93	375	350	335	17	40	0	4	0
13462	7780	4654	4	1190	888	28086	606	230	920	79	96	197	107	190	282.55	199	446.48	487	355	462	37	94	14	12	4
4108	13476	9808	899	432	248	28090	1241	5	572	0	18	60	4	64	274.97	106	430.33	320	264	290	19	35	0	0	2
11604	7878	5242	1	1932	1526	28534	555	102	638	27	324	168	50	255	308.51	123	459.01	497	233	375	93	82	5	26	16
12480	7822	4996	3	1560	1208	28324	589	170	759	48	242	169	89	234	292.03	167	450.72	482	276	440	51	81	15	12	14
11194	7800	5330	0	2150	1754	28684	520	103	540	19	402	162	53	281	312.99	104	459.53	490	189	354	122	78	8	54	22
4388	13376	9658	846	398	242	28078	1239	6	611	2	16	75	3	80	273.74	93	428.08	312	321	276	12	41	0	0	2
5190	12810	9172	624	578	364	28136	1069	6	684	1	18	70	3	111	279	105	436.67	365	338	358	19	39	0	4	2
4742	13126	9474	760	470	268	28092	1177	7	670	0	12	50	1	79	276.74	107	430.8	339	337	325	16	35	0	0	2
13706	7704	4592	4	1104	808	28016	631	265	937	125	96	236	96	183	276.83	199	442.07	473	392	425	35	88	20	6	3
5778	12330	8866	556	730	426	28156	1069	17	728	6	20	64	9	115	283.47	107	440.32	372	379	358	18	43	0	6	0
4410	13250	9644	801	512	280	28108	1182	9	581	0	10	59	6	84	276.92	88	431.63	337	278	313	17	35	0	2	1
11992	7894	5178	1	1766	1332	28440	557	127	668	40	258	179	60	263	299.59	119	455.84	478	242	399	78	91	3	18	13
14358	7612	4344	1	878	650	27900	633	299	1072	163	56	269	100	149	274.65	274	437.75	498	428	419	25	93	17	2	1
6498	11914	8466	433	762	484	28184	1002	26	779	0	60	74	17	129	286.94	120	442.25	399	419	359	23	52	2	0	1
4380	13336	9642	863	442	274	28096	1237	9	601	2	22	56	3	91	274.86	86	429.3	298	282	296	15	41	0	0	0
11830	7888	5234	3	1846	1392	28488	562	121	674	25	268	164	69	255	307.16	129	456.14	483	227	399	87	84	4	28	15
3924	13732	9964	997	258	142	28030	1315	9	578	1	10	63	6	55	270.63	84	422.19	272	280	269	18	43	0	0	2
10686	7680	5550	0	2526	1928	28858	532	59	429	10	430	161	31	298	330.05	77	471.02	458	142	346	126	69	3	48	37
13612	7776	4662	7	1080	780	28008	613	278	926	121	98	241	103	196	276.69	216	443.63	480	337	422	30	104	24	0	1
4460	13312	9642	793	426	236	28088	1199	7	624	0	10	61	6	80	275.76	96	431.91	336	332	306	10	36	0	2	2
14454	7570	4356	3	834	600	27880	679	308	1067	152	66	248	118	154	272.9	239	436.48	453	455	437	22	82	20	0	1
7448	11222	7886	362	938	636	28220	892	35	796	6	82	87	16	129	288.2	144	442.02	443	357	399	37	65	1	6	3
4150	13578	9822	908	310	180	28052	1274	5	603	1	12	63	3	80	272.27	73	424.61	277	309	284	13	40	0	0	2
14128	7760	4488	8	840	620	27896	667	308	1050	165	56	258	105	151	269.46	271	433.45	480	448	417	19	86	20	4	2
3902	13700	9956	1000	312	170	28046	1333	7	552	1	6	58	5	66	270.81	72	423.96	258	286	265	15	42	0	0	1
4688	13152	9478	761	484	292	28112	1176	8	656	0	18	51	6	90	278.59	98	434.37	339	320	327	11	37	0	0	1
4452	13300	9624	848	432	256	28082	1243	10	618	1	18	57	6	82	274.25	88	428.66	295	306	301	15	41	0	0	2
13850	7786	4524	10	952	758	27972	628	309	955	139	90	235	128	164	273.11	245	437.59	497	383	446	22	83	18	8	3

SUPPLEMENTARY INFORMATION:Monte Carlo Atomistic Simulation and Machine Learning Analysis of Na-K Eutectic Alloy in Condensed Phases, D. Reitz and E. Blaisten-Barojas, George Mason University, Fairfax, VA 22030

4230	13546	9782	894	302	178	28050	1250	7	635	1	10	58	2	65	273.09	100	426.32	299	308	303	17	39	0	2	2
4022	13598	9866	922	356	210	28066	1273	7	576	1	14	59	3	74	272.1	79	425.86	291	283	283	9	37	0	0	5
11636	7892	5288	2	1906	1460	28508	555	112	641	23	298	167	63	284	305.8	118	457.78	475	214	388	75	99	5	22	18
12768	7706	4878	0	1580	1150	28274	605	194	749	55	184	181	101	208	293.78	167	449.46	507	272	409	55	99	10	6	9
4736	13100	9476	724	488	284	28110	1147	6	662	0	26	61	3	90	278.02	103	434.19	348	332	331	15	48	0	0	2
3922	13638	9936	970	360	194	28058	1303	7	571	1	8	57	3	70	269.93	89	422.71	275	283	274	11	42	0	0	4
4826	13004	9356	717	562	368	28154	1151	1	626	0	34	69	1	105	280.54	99	436.81	342	311	311	16	44	0	4	3
4852	13052	9394	757	480	302	28110	1187	9	654	1	26	68	6	93	277.05	99	433.23	327	330	305	14	47	0	2	2
5056	12972	9262	663	504	316	28122	1116	3	701	0	12	59	3	89	278.37	123	434.32	377	350	346	7	38	0	0	4
12662	7800	4882	2	1560	1178	28276	600	188	727	52	182	172	100	233	287.8	155	450.27	499	280	423	44	82	16	12	5
6070	12130	8630	490	794	520	28190	1041	21	717	6	42	82	12	109	284.87	114	442.18	404	384	334	25	47	1	4	1
5396	12738	9122	654	528	308	28110	1123	12	734	4	18	64	5	92	279.22	101	436.98	368	373	336	11	44	0	0	1
4018	13686	9892	950	274	164	28044	1289	12	586	2	10	59	8	68	273.16	94	425.72	278	288	283	13	45	0	0	2
3988	13530	9864	957	442	250	28086	1313	7	532	0	12	61	4	84	275.54	65	429.87	263	274	258	14	40	0	0	2
10548	7712	5622	0	2538	1946	28882	508	55	422	7	464	158	24	323	333.79	84	471.38	468	113	330	137	77	2	44	25
13712	7812	4644	14	1008	724	27978	651	263	997	106	70	224	123	174	276.24	233	439.85	464	406	446	27	97	16	8	3
3822	13694	9986	968	348	194	28058	1248	9	530	1	14	64	5	66	272.19	90	425.09	308	230	293	15	40	0	0	2
4806	12994	9452	724	556	290	28110	1142	5	642	0	12	69	4	81	277.43	96	433.08	360	318	324	18	51	0	0	2
4784	13028	9422	745	538	320	28116	1145	9	642	1	22	64	8	89	276.29	90	431.67	346	315	330	18	38	0	2	3
4282	13436	9718	895	382	236	28076	1258	4	624	1	22	64	2	75	271.17	104	424.92	300	294	286	14	45	0	0	1
13846	7868	4654	5	882	614	27918	598	281	1053	122	48	241	123	173	275.39	242	439.71	479	410	467	28	102	16	6	0
13848	7814	4536	5	972	730	27964	649	278	1003	126	56	226	114	168	275.58	227	440.45	464	399	450	29	85	18	6	3
5184	12616	9152	634	750	446	28186	1119	8	657	1	38	51	4	101	281.67	109	436.69	377	338	328	15	36	0	0	3
14236	7582	4404	3	908	686	27910	597	330	1024	160	88	275	125	164	272.51	247	437.58	486	405	433	29	100	21	6	6
5446	12588	9070	633	630	368	28132	1101	11	684	2	30	67	7	103	281.65	103	439.32	356	352	344	19	40	0	0	3
14092	7748	4428	8	882	704	27940	628	285	1041	144	80	262	102	150	274.61	269	438.66	502	400	422	25	102	19	6	6
14466	7566	4352	6	824	596	27870	646	347	1038	163	64	257	140	150	268.86	261	432.12	489	444	433	16	80	18	2	1
3866	13712	9958	983	328	186	28056	1311	6	561	0	6	53	4	76	270.68	79	422.63	262	266	280	9	33	0	0	4
7216	11388	8066	349	922	560	28204	910	31	789	8	50	78	19	149	289.02	127	445.09	413	378	415	27	44	0	2	1
10526	7758	5712	0	2410	1882	28850	517	63	449	6	508	170	36	281	330.64	85	469.03	466	143	336	147	76	5	46	33
14164	7686	4394	9	918	700	27928	625	321	976	147	64	241	134	160	272.48	262	435.29	497	383	448	19	73	21	2	2
14380	7660	4354	8	806	618	27884	658	328	1045	165	62	251	126	142	270.44	277	433.26	500	425	429	14	77	23	4	1
6726	11538	8256	356	1030	642	28258	970	20	746	2	64	60	12	127	287.56	132	442.49	446	406	362	17	34	0	2	5
5138	12732	9208	652	652	388	28150	1110	9	665	1	30	54	6	96	280.69	101	434.77	358	347	352	25	36	0	2	0
10378	7896	5818	5	2432	1814	28824	477	55	462	12	436	169	29	303	333.54	88	469.8	495	131	359	126	78	4	48	28
4852	13010	9378	729	536	326	28126	1152	6	665	0	22	63	4	79	279.36	120	436.16	369	319	313	16	46	0	2	2

SUPPLEMENTARY INFORMATION:Monte Carlo Atomistic Simulation and Machine Learning Analysis of Na-K Eutectic Alloy in Condensed Phases, D. Reitz and E. Blaisten-Barojas, George Mason University, Fairfax, VA 22030

4300	13470	9742	906	342	192	28054	1270	7	618	2	6	74	3	69	271.79	104	425.82	296	307	269	15	51	0	2	2
6190	12132	8632	505	722	452	28172	1045	21	753	2	44	60	14	109	282.43	116	437.79	400	390	362	18	38	2	0	0
5286	12758	9172	622	544	328	28122	1086	11	714	3	32	59	7	94	279.85	106	438.25	375	354	365	15	40	0	2	1
11816	7910	5254	1	1844	1364	28454	558	125	681	35	232	177	70	249	300.57	134	456.03	494	245	396	77	88	5	28	14
4200	13478	9802	912	376	196	28060	1274	10	609	1	8	62	5	72	272.84	99	425.83	290	289	277	17	48	0	0	0
5270	12630	9140	621	684	400	28156	1095	13	661	2	30	75	7	106	280.06	88	437.77	360	354	328	26	52	0	2	2
12138	7956	5096	3	1640	1294	28398	569	144	717	44	250	195	64	255	298.88	140	455.15	489	250	387	61	96	12	24	15
11148	7872	5406	0	2104	1688	28658	541	90	573	17	408	164	48	279	314.88	118	459.17	454	188	379	113	75	6	26	24
7930	10866	7612	302	1048	688	28224	907	61	777	16	70	96	34	133	288.58	121	446.6	434	393	382	38	50	3	10	1
14370	7638	4374	3	832	606	27872	637	341	1040	166	52	258	138	155	270.9	257	433.92	482	430	440	20	75	15	0	0
6754	11606	8330	414	898	544	28202	950	29	748	2	64	87	20	123	287.48	124	444.47	437	378	362	25	62	1	4	3
4616	13132	9520	799	528	300	28110	1221	7	617	0	14	53	3	86	275.96	82	431.27	309	318	301	20	37	0	0	1
5692	12456	8928	575	634	388	28136	1078	15	726	4	36	74	7	91	285.18	113	440.14	388	376	335	24	52	0	2	1
5390	12444	9016	557	826	494	28206	1063	9	679	0	30	61	4	100	282.01	108	437.69	417	352	327	20	38	0	6	2
7006	11366	8092	349	1034	674	28258	893	29	722	3	80	97	18	149	287.06	121	445.82	454	353	366	28	59	3	6	3
11994	7960	5142	2	1708	1352	28452	527	120	661	26	264	173	65	235	304.89	136	462.22	514	213	413	79	91	5	30	22
12884	7852	4826	1	1426	1076	28226	605	181	836	61	148	166	94	209	284.06	180	446.75	480	310	470	51	88	14	14	5
12752	7800	4850	1	1516	1156	28264	622	183	791	59	168	173	91	230	288.88	156	450.24	469	320	422	51	95	8	20	6
4252	13518	9758	886	318	194	28056	1255	10	630	2	16	62	6	69	272.36	99	425.3	302	309	291	12	46	0	0	2
4398	13288	9670	836	474	254	28096	1224	4	592	0	10	53	3	72	278.31	91	432.03	316	307	311	15	37	0	2	5
11316	7856	5412	1	2084	1574	28602	465	94	574	26	308	179	44	295	313.16	113	461.09	481	147	420	94	101	5	48	25
6002	12148	8698	508	808	494	28186	1024	16	721	1	36	78	10	110	284.18	120	440.69	409	365	346	24	46	0	0	2
14310	7462	4270	1	1020	794	27960	625	324	961	166	100	254	120	166	272.34	245	436.41	496	393	426	24	75	25	4	3
4482	13276	9610	805	452	258	28090	1213	2	628	0	12	60	1	72	275.36	106	430.04	330	328	300	15	34	0	0	3
4200	13494	9756	907	370	234	28076	1276	14	584	2	22	56	8	65	272.72	93	425.8	299	298	280	15	36	0	0	3
12614	7610	4898	3	1698	1268	28334	585	177	678	57	224	180	85	227	293.32	124	450.52	489	246	417	67	90	11	20	9
12028	7934	5190	2	1722	1278	28400	565	135	683	26	226	154	72	249	301.72	139	455.78	475	224	429	75	86	10	20	14
3976	13636	9890	958	338	206	28064	1303	5	578	1	18	58	4	62	270.3	82	422.34	282	283	274	15	42	0	0	3
4512	13314	9610	841	386	230	28070	1219	7	637	1	18	58	3	76	273.2	99	427.62	304	315	316	20	41	0	0	1
4342	13392	9706	872	400	222	28072	1239	5	611	0	10	65	3	66	275.53	91	430.16	309	296	293	22	47	0	0	1
7008	11450	8114	343	964	632	28250	924	23	812	8	78	79	13	143	286.87	149	445.22	433	395	383	31	50	0	4	0
4674	13164	9508	764	464	270	28098	1166	6	636	0	18	60	4	89	277.57	99	432.8	340	317	327	10	39	0	0	3
5276	12682	9138	646	630	386	28150	1099	6	675	0	34	68	2	90	281.92	109	437.68	375	337	332	25	36	2	4	2
4372	13304	9678	856	462	254	28084	1236	6	609	0	14	48	4	74	276.13	93	431.13	303	302	312	18	31	0	0	2
4200	13458	9760	899	406	238	28076	1275	9	592	1	14	62	5	78	275.09	88	429.64	286	287	275	14	47	0	0	3
5780	12324	8850	513	734	442	28166	1008	11	727	3	36	71	5	113	284.95	114	442.52	398	362	372	26	46	0	0	2

4630	13160	9498	748	498	308	28118	1158	9	615	1	20	61	4	93	280.34	94	435.5	344	319	323	16	39	0	4	0
11284	7998	5314	1	2030	1626	28612	570	81	581	18	326	159	45	260	311.94	124	457.8	503	208	373	90	78	6	28	20
3924	13682	9910	969	324	204	28060	1295	6	556	1	16	64	4	76	270.3	92	423.57	273	266	276	11	37	0	0	3
12708	7906	4802	3	1482	1186	28272	608	164	769	43	176	168	95	221	291.78	163	449.28	472	282	443	57	85	11	12	9
4278	13440	9738	869	384	220	28074	1220	5	603	0	12	59	3	74	275.41	93	430.54	314	291	309	16	46	0	2	1
5824	12482	8894	576	554	332	28112	1106	21	765	2	24	66	13	104	282.68	123	437.26	345	395	347	20	42	0	2	2
5232	12918	9246	675	420	244	28078	1153	10	741	3	18	73	5	84	279.63	110	436.27	351	387	322	12	44	0	0	1
14260	7618	4404	9	894	652	27892	638	310	1039	165	62	279	114	156	271.24	255	434.51	493	413	410	20	102	19	2	1
12190	7882	5108	2	1714	1246	28348	580	134	724	22	196	179	76	232	298.06	150	452.19	508	253	395	56	119	15	10	14
4578	13256	9578	786	418	236	28078	1211	3	650	0	12	69	2	91	275.28	90	430.34	312	344	299	10	43	0	0	1
4416	13400	9692	856	348	188	28052	1239	10	648	1	8	61	7	69	272.65	102	426.38	313	314	299	11	41	0	0	2
4098	13488	9838	908	422	220	28072	1277	10	561	1	6	58	7	72	273.26	69	426.21	290	298	280	15	35	0	0	2
4758	13058	9464	750	510	292	28108	1171	7	673	1	26	62	4	89	275.24	93	430.84	338	336	317	17	44	0	0	0
11530	7810	5278	0	2016	1574	28586	517	103	588	19	346	172	50	270	318.57	107	460.02	504	197	386	88	90	8	28	25
11758	7952	5322	1	1762	1354	28480	591	106	703	17	294	166	51	229	305.52	152	458.16	513	261	355	83	99	9	38	13
4004	13684	9916	957	278	150	28036	1298	5	606	1	4	54	3	75	270.86	94	422.06	265	285	287	12	39	0	0	1
5028	12858	9274	687	596	354	28130	1132	5	656	1	18	69	4	97	282.4	95	439.76	349	336	328	20	40	0	2	0
14388	7612	4356	5	830	626	27884	601	328	1057	157	68	266	132	166	271.89	270	436.47	499	405	440	16	96	19	4	2
12524	7770	5000	1	1602	1186	28310	601	162	758	52	206	198	82	223	292.74	175	451.07	513	280	386	55	97	11	20	6
4738	12988	9442	735	612	342	28140	1178	7	633	1	18	60	6	95	276.72	91	431.54	332	329	308	20	41	0	0	1
4212	13452	9778	896	406	218	28074	1242	7	588	1	8	60	5	76	274.02	96	428.97	303	271	302	12	43	0	0	3
12680	7980	4954	3	1392	1054	28232	605	181	847	53	160	177	95	197	291.49	179	449.33	486	315	450	62	93	12	10	6
12836	7958	4894	3	1338	1012	28190	567	165	860	51	136	184	94	220	286.43	193	448.64	474	303	478	53	96	8	14	5
14034	7758	4468	3	878	702	27934	649	304	1024	153	84	248	109	156	271.49	268	436.38	491	403	424	21	88	22	10	3
4660	13174	9538	773	444	250	28086	1188	5	663	1	18	69	1	85	276.05	92	430.13	320	337	313	18	50	0	2	2
14166	7748	4506	2	816	590	27888	643	300	1084	138	54	241	120	157	273.32	250	438.68	464	429	455	28	94	16	8	0
13648	7712	4548	5	1132	886	28052	592	274	925	129	108	241	108	190	277.72	219	442.82	499	336	435	33	95	16	18	3
14286	7560	4340	3	960	716	27938	656	329	976	165	74	249	125	179	273.89	225	436.86	459	407	428	20	73	18	2	2
5364	12742	9148	619	532	310	28124	1103	8	733	1	26	66	5	87	280.74	114	438.96	375	371	343	19	41	0	2	2
4442	13324	9640	838	420	242	28082	1254	5	642	1	14	70	3	78	275.01	102	430.09	307	332	270	13	43	0	0	2
4818	12940	9388	697	608	358	28142	1148	6	632	1	30	58	4	85	278.13	91	434.97	363	343	318	19	37	0	0	2
5416	12554	9022	611	702	440	28172	1100	7	683	0	36	71	5	100	281.17	106	439.67	377	350	323	21	49	1	2	3
10742	7910	5720	0	2266	1672	28740	482	58	493	9	362	181	32	306	329.99	101	469.93	492	138	353	103	89	1	64	37
3900	13700	9942	968	314	186	28056	1297	5	560	1	14	63	3	68	270.55	84	423.41	283	277	274	9	46	0	0	4
11236	7830	5438	1	2122	1622	28652	536	89	549	20	364	166	37	276	324.24	101	466.08	455	197	376	107	80	8	32	31
13518	7574	4608	3	1314	958	28110	602	268	831	105	120	206	122	210	278.91	183	444.67	454	304	466	43	79	27	18	6

SUPPLEMENTARY INFORMATION:Monte Carlo Atomistic Simulation and Machine Learning Analysis of Na-K Eutectic Alloy in Condensed Phases, D. Reitz and E. Blaisten-Barojas, George Mason University, Fairfax, VA 22030

4188	13484	9784	894	382	218	28070	1266	7	614	0	14	52	5	66	272.88	100	424.89	304	314	294	12	34	0	0	2
13680	7746	4566	3	1086	836	28024	604	253	964	115	96	241	104	183	277.16	222	441.77	489	356	435	36	105	19	14	2
4982	12820	9302	658	648	370	28146	1126	5	646	0	24	59	5	95	277.43	86	435.38	359	352	335	18	44	0	0	3
14406	7626	4368	1	832	596	27876	637	327	1063	168	46	261	124	143	268.97	285	431.59	502	427	433	18	81	20	2	1
12660	7972	5006	2	1378	1024	28216	605	186	829	59	164	178	87	214	287.76	185	449.03	495	303	426	47	86	20	10	6
4202	13600	9824	904	258	140	28030	1246	7	634	0	6	65	6	71	271.95	93	425.15	294	297	308	11	50	0	0	2
14396	7650	4376	7	800	590	27870	642	319	1079	161	56	272	124	144	268.4	283	430.14	505	433	422	10	98	21	2	4
11786	7880	5160	2	1858	1488	28508	570	106	653	24	298	164	58	246	301.5	135	455.62	486	231	399	87	85	8	36	17
13896	7814	4562	5	922	684	27948	645	299	1005	141	62	223	126	160	276.23	234	441.2	467	400	453	36	74	11	6	0
14006	7828	4508	10	826	664	27924	633	296	1036	151	88	242	105	150	274.55	264	440.03	500	407	442	19	79	20	4	5
4458	13344	9644	833	398	220	28068	1216	8	631	1	4	50	3	91	274.49	89	430.25	295	304	325	13	25	0	0	1
11810	7962	5184	2	1764	1428	28480	525	123	652	28	298	166	63	275	305.99	127	456.67	485	236	423	79	86	8	34	13
12806	7952	4898	2	1368	1020	28184	595	196	846	75	128	206	82	200	283.56	186	449.21	496	312	421	53	96	14	10	7
14356	7664	4382	4	798	610	27884	622	330	1024	160	70	255	133	139	270.65	271	433.43	516	411	438	19	82	17	4	2
7640	11148	7780	310	936	632	28212	879	42	811	10	70	102	22	157	287.29	142	447.23	426	383	392	31	66	1	4	2
4236	13450	9750	875	388	232	28078	1265	7	593	1	22	55	2	68	273.98	92	428.13	302	315	288	15	35	0	0	2
4268	13424	9728	890	394	240	28078	1268	4	617	0	22	62	3	79	273.25	83	426.68	293	320	280	13	44	0	2	1
3980	13658	9888	951	332	196	28060	1302	7	585	1	6	61	5	76	272.13	81	425.02	280	290	265	7	40	0	0	3
4338	13336	9682	851	454	264	28094	1247	4	612	1	20	57	3	72	274.55	108	428.86	319	306	282	15	39	0	0	1
4924	12918	9332	669	592	348	28134	1126	5	650	0	20	58	4	94	276.98	85	434.16	357	349	333	20	30	0	0	2
4436	13258	9604	816	484	300	28112	1219	3	595	0	30	58	3	71	277.88	96	433.61	332	300	297	13	39	0	0	6
4366	13284	9658	816	484	286	28106	1227	2	605	0	28	56	1	90	276.17	79	431.54	301	316	303	17	37	0	0	1
12580	7842	4962	2	1528	1164	28296	588	170	780	39	192	166	101	242	293.81	163	449.85	468	287	429	63	93	7	28	7
5400	12644	9084	632	616	368	28140	1104	10	712	0	28	63	5	88	281.04	117	436.7	380	355	332	20	46	0	0	3
5026	12842	9292	672	598	348	28134	1125	10	663	2	28	66	4	86	278.83	95	435.02	370	345	327	19	42	0	0	1
4920	13064	9372	726	458	272	28096	1141	8	668	0	8	68	6	83	279.42	112	434.7	354	320	329	17	48	0	2	2
4974	12956	9330	663	520	312	28116	1143	4	695	0	22	69	3	98	279.93	100	436.51	340	381	325	14	47	0	2	5
14164	7778	4450	10	802	624	27882	642	321	1043	161	58	252	121	158	271.21	271	433.76	482	428	442	17	82	22	6	0
13616	7966	4700	5	904	694	27974	648	258	1038	114	88	225	99	148	275.94	248	440.75	495	426	435	34	91	23	6	2
5874	12296	8810	521	728	428	28160	1034	14	736	3	22	79	9	112	282.18	123	438.96	404	375	341	19	52	0	2	1
5148	12858	9232	642	536	324	28122	1108	10	696	0	24	61	6	86	280.1	113	436.73	383	356	341	12	37	0	0	3
4152	13532	9812	922	354	198	28056	1291	14	592	1	8	54	9	77	272.31	83	425.74	263	309	288	17	35	0	0	2
12432	7898	5030	2	1570	1168	28304	576	145	806	43	192	188	71	213	291.36	197	450.41	527	279	404	60	105	13	14	5
13008	7746	4692	4	1438	1150	28230	605	214	783	76	184	190	100	213	284.91	189	448.52	498	295	429	45	83	14	12	6
11906	8072	5170	1	1674	1322	28396	616	127	762	36	224	176	60	226	298.16	160	450.79	471	299	375	83	92	8	22	14
11550	8000	5338	1	1858	1446	28528	544	109	628	26	302	172	57	273	305.18	132	458.5	487	204	388	78	93	6	34	22

SUPPLEMENTARY INFORMATION:Monte Carlo Atomistic Simulation and Machine Learning Analysis of Na-K Eutectic Alloy in Condensed Phases, D. Reitz and E. Blaisten-Barojas, George Mason University, Fairfax, VA 22030

11146	7774	5494	1	2264	1644	28696	527	71	514	10	320	158	36	322	331.62	68	470.21	421	183	391	103	76	8	54	31
13788	7806	4552	6	1008	754	27978	630	271	989	141	64	225	107	178	274.92	216	439.16	460	398	469	29	70	9	6	3
4126	13606	9828	919	304	178	28048	1256	7	606	1	6	59	4	65	272.05	102	425.86	293	283	304	17	40	0	0	2
4114	13472	9808	914	416	246	28082	1266	5	569	2	26	66	3	77	272.29	88	425.93	296	272	273	16	44	0	0	1
4582	13110	9526	777	572	322	28126	1212	7	622	1	14	64	4	79	275.75	76	430.41	329	334	293	18	47	0	0	3
13722	7788	4594	3	1028	772	28000	609	249	995	115	90	229	93	189	278.29	234	442.14	477	369	458	27	92	18	6	2
5114	12832	9232	643	594	348	28134	1136	8	691	0	14	67	4	92	280.64	98	436.16	359	380	318	20	44	0	0	1
4158	13516	9782	928	370	228	28072	1292	10	598	1	18	57	6	71	270.46	88	422.63	283	301	277	14	40	0	0	2
4384	13312	9656	859	462	264	28090	1240	8	606	0	10	64	6	76	275.29	89	428.75	310	304	290	13	44	0	2	4
11724	7940	5246	1	1828	1420	28462	514	118	694	29	268	182	65	265	300	128	460.2	502	218	406	85	104	5	32	9
11464	8022	5344	1	1848	1498	28548	559	86	676	20	324	184	39	260	309.07	125	459.2	472	238	365	103	108	5	42	20
5272	12844	9204	636	478	280	28098	1122	10	734	1	18	66	4	87	280.11	122	435.31	376	385	325	12	45	0	2	1
4936	13064	9358	686	438	278	28098	1133	2	693	0	24	62	2	78	278.57	118	433.82	362	354	339	18	37	0	0	2
4062	13532	9836	943	398	232	28076	1296	8	562	1	16	54	5	69	273.23	83	425.48	287	277	272	15	34	0	0	1
5702	12324	8882	571	784	456	28172	1081	21	667	5	22	63	12	107	281.48	109	440.45	371	350	344	23	34	0	2	3
4260	13354	9686	842	488	300	28110	1241	6	581	2	22	54	2	74	275.19	107	429.43	323	302	284	15	30	0	0	2
6290	12058	8604	517	720	438	28154	1058	33	770	6	40	66	19	124	282.21	115	438.89	371	390	356	14	44	0	4	2
3766	13702	9994	1009	374	218	28070	1299	8	530	1	14	53	5	72	271.42	85	423.27	275	237	281	14	35	0	2	2
13878	7888	4570	3	856	670	27946	628	265	1066	124	80	235	118	171	275.96	253	439.03	478	419	458	23	100	15	4	1
11306	7866	5426	1	2062	1570	28616	534	83	591	19	354	182	35	272	319.11	111	461.98	484	186	354	110	95	10	30	21
4546	13254	9596	797	426	244	28088	1199	3	650	0	22	63	2	87	277.71	104	431.62	332	328	302	9	38	0	0	1
4566	13282	9570	817	412	242	28082	1193	5	642	0	10	58	3	75	275.43	104	430.87	332	310	319	15	31	0	0	2
11208	7994	5546	3	1960	1486	28576	533	87	639	21	350	146	41	270	312.91	138	458.01	510	216	387	88	72	9	30	17
4624	13200	9538	761	452	266	28102	1178	4	657	0	20	56	3	92	278.27	92	432.77	328	342	330	11	40	0	2	2
14112	7710	4428	6	910	700	27936	612	301	995	142	72	258	124	164	273.38	257	437.37	505	383	436	18	102	17	2	2
14442	7670	4388	8	770	540	27844	630	339	1082	152	34	256	148	145	267.87	272	429.5	494	437	459	12	91	24	0	1
4292	13320	9718	849	480	264	28094	1225	6	592	0	20	64	4	87	275.88	92	430.92	312	296	293	12	45	0	0	2
4684	13228	9550	775	384	208	28066	1184	6	682	0	12	56	4	79	276.06	97	432.03	316	328	340	14	39	0	0	5
3748	13742	10010	1014	348	204	28068	1336	3	545	0	16	49	1	71	271.9	89	424.11	272	261	259	6	36	0	0	2
10836	7802	5610	1	2254	1766	28788	488	68	502	9	470	154	27	319	334.09	98	472.11	430	138	379	142	77	6	40	30
13856	7602	4416	5	1142	906	28048	620	273	893	119	116	227	108	191	276.91	206	443.25	467	336	439	40	83	22	8	3
12878	7990	4878	5	1304	980	28160	664	193	897	60	120	167	93	186	282.94	184	444.28	474	362	445	42	93	17	10	5
12684	7834	4840	5	1506	1202	28290	608	181	744	43	208	163	110	210	290.33	156	450.86	490	275	445	59	84	15	16	7
7784	10816	7630	259	1208	768	28274	825	41	771	9	64	97	30	166	288.14	137	445.27	456	369	405	34	59	0	4	5
4562	13308	9600	819	376	210	28064	1197	11	655	0	8	60	7	66	276	105	429.89	330	312	318	18	45	0	0	3
13770	7636	4514	4	1134	876	28072	611	237	922	76	134	206	115	197	282.01	229	447.25	467	334	453	40	99	16	8	5

SUPPLEMENTARY INFORMATION:Monte Carlo Atomistic Simulation and Machine Learning Analysis of Na-K Eutectic Alloy in Condensed Phases, D. Reitz and E. Blaisten-Barojas, George Mason University, Fairfax, VA 22030

11142	8030	5494	1	2010	1558	28608	538	91	590	19	330	163	45	279	316.09	114	460.06	453	207	389	109	83	8	42	20
6006	12108	8650	528	808	558	28212	1025	18	683	6	74	68	10	138	287.98	95	441.98	373	344	355	27	44	1	8	1
4394	13524	9734	878	240	130	28028	1254	8	655	0	6	68	5	65	274.52	107	428.55	302	327	295	9	50	0	0	3
11822	7914	5256	2	1852	1354	28462	523	134	642	36	248	173	73	296	307.14	127	456.24	452	204	443	76	84	4	16	13
5028	12800	9272	655	632	378	28146	1136	6	657	0	34	54	4	94	277.96	87	434.41	348	359	338	22	38	0	2	2
12114	7920	5136	1	1668	1284	28390	561	130	687	30	236	174	79	254	298.19	137	454.95	468	232	430	78	94	7	30	8
13146	7740	4690	3	1376	1066	28186	600	209	812	68	156	175	109	221	281.54	192	445.63	485	295	460	37	86	12	12	4
4306	13428	9710	860	390	228	28072	1250	6	607	0	10	66	3	68	273.83	96	427.91	321	311	276	11	47	0	0	2
4060	13560	9850	918	378	210	28064	1282	5	569	1	6	72	3	60	271.78	88	425.94	301	291	260	17	46	0	0	3
14006	7712	4456	4	946	742	27956	646	288	996	128	84	248	120	180	276.75	225	442.33	448	415	429	32	100	21	10	4
13384	7682	4634	2	1310	984	28132	599	218	858	69	120	217	111	204	284.96	184	450.39	481	324	430	49	107	17	18	3
14000	7690	4460	2	994	744	27966	621	286	984	128	76	245	116	172	276.05	243	441.66	480	380	433	33	93	20	2	2
4752	13066	9478	724	506	278	28098	1181	7	669	0	18	67	3	88	276.87	88	433.72	326	361	302	21	42	0	0	2
3952	13656	9914	974	330	190	28052	1307	7	570	1	10	64	5	62	271.07	93	423.15	287	268	256	14	49	0	0	2
11390	7752	5380	0	2130	1602	28650	528	97	554	14	362	180	49	292	323.7	109	465.03	467	195	362	108	83	6	34	20
13714	7802	4570	5	1036	792	28010	634	247	988	110	94	226	114	176	274.35	242	438.15	488	393	440	26	98	10	2	1
13886	7772	4572	5	944	698	27958	639	282	1022	114	78	241	127	173	276.23	213	442.06	444	404	450	35	112	22	8	7
4736	13078	9434	729	518	322	28114	1157	7	635	0	26	63	4	73	279.21	108	434.75	372	324	312	15	37	0	0	2
11220	7870	5376	0	2174	1666	28682	503	76	538	12	336	160	39	287	317.92	107	461.47	510	170	383	94	90	3	36	29
3938	13664	9896	960	340	214	28068	1292	7	565	1	16	50	4	70	271.82	95	424.85	284	266	285	12	27	0	0	2
4052	13462	9852	897	458	248	28092	1233	4	552	0	18	61	1	78	274.27	99	429.5	314	255	290	13	39	0	2	4
12988	7862	4772	3	1354	1048	28176	630	206	864	71	134	195	97	226	281.61	179	446.37	453	333	427	46	97	15	18	3
14246	7706	4390	5	840	650	27898	630	319	1049	154	62	253	131	157	272.32	274	437.01	491	412	442	18	88	19	4	2
10970	7840	5468	1	2282	1754	28724	523	75	526	17	350	167	36	301	317	98	463.8	461	174	370	105	84	7	54	31
4570	13132	9528	783	552	318	28114	1192	12	599	0	14	55	8	78	276.07	77	431.46	342	310	307	14	34	0	0	4
4004	13536	9862	932	416	244	28084	1266	6	552	1	22	66	3	62	270.37	99	423.9	317	260	269	15	45	0	0	2
14304	7620	4362	7	896	666	27910	620	306	1010	153	56	245	119	141	272.46	279	436.77	506	387	457	26	82	17	6	1
5812	12362	8884	583	650	378	28118	1079	32	680	11	32	82	14	96	281.35	88	439.12	365	366	338	26	55	0	0	4
14164	7640	4358	2	954	744	27938	604	303	982	154	76	241	119	155	270.19	258	434.66	525	385	451	18	77	15	2	0
5144	12774	9232	664	628	348	28138	1120	6	676	1	12	67	4	79	277.48	122	434.27	387	327	320	19	49	0	0	2
4248	13372	9750	858	460	246	28088	1232	3	595	0	10	74	3	82	273.51	87	429.15	305	296	287	19	46	0	2	0
13930	7742	4470	2	974	766	27976	663	282	984	125	88	246	120	159	274.8	227	441.8	479	417	403	34	113	13	6	3
4844	13006	9374	738	556	332	28128	1175	5	653	0	16	62	4	78	278.72	117	433.6	359	331	303	17	42	0	0	1
4292	13430	9714	883	392	232	28072	1240	9	605	3	12	73	6	76	273.77	96	427.13	308	289	282	16	47	0	0	1
4082	13596	9852	932	332	186	28054	1289	9	580	2	6	55	5	72	272.4	92	426.79	283	280	281	13	35	0	0	0
4710	13166	9508	763	446	248	28090	1182	9	649	2	12	64	7	73	277.23	89	432.41	334	342	324	16	43	0	0	5

SUPPLEMENTARY INFORMATION:Monte Carlo Atomistic Simulation and Machine Learning Analysis of Na-K Eutectic Alloy in Condensed Phases, D. Reitz and E. Blaisten-Barojas, George Mason University, Fairfax, VA 22030

10318	7880	5762	0	2434	1936	28896	477	47	442	14	490	165	19	305	331.99	77	466.84	472	127	356	130	82	2	68	45
5136	12894	9260	649	502	294	28104	1114	14	704	0	18	60	9	92	279.89	102	440.31	352	365	350	19	42	0	0	3
6318	11832	8492	415	964	584	28234	966	22	737	1	42	75	14	123	286.55	147	444.99	449	353	351	19	45	1	2	5
12170	7902	5060	1	1692	1316	28400	606	140	731	34	236	170	71	232	300.48	146	455.29	487	256	396	70	95	11	22	12
3794	13810	10040	1017	250	128	28026	1297	11	558	3	4	49	4	74	270.06	86	422.52	251	241	304	15	34	0	0	2
4464	13316	9642	841	408	234	28084	1207	4	639	1	18	58	3	80	277.83	96	432.62	319	309	318	12	39	0	2	3
5334	12768	9170	591	524	298	28116	1105	5	776	0	22	67	3	89	279.17	114	433.64	374	412	344	14	45	0	0	3
6004	12140	8692	484	798	506	28194	1029	15	752	3	54	72	7	120	285.22	120	442.05	386	391	357	26	44	1	0	3
10946	8002	5490	0	2114	1702	28684	532	78	573	15	378	156	43	334	315.82	99	462.74	452	177	378	87	85	4	46	23
4814	12870	9360	721	666	406	28158	1148	10	604	0	40	62	6	96	279.21	91	433.97	360	310	303	21	38	0	2	0
14198	7592	4366	6	950	740	27944	652	315	969	159	94	259	111	159	271.73	242	435.55	489	385	410	24	81	28	4	2
14304	7718	4434	11	792	568	27858	661	321	1070	174	42	269	110	142	270.04	262	433.74	495	456	410	16	86	15	0	0
7484	11230	7832	317	972	650	28230	896	33	803	4	60	96	22	142	289.91	162	446.16	461	387	358	26	64	0	2	3
13436	7744	4640	1	1220	924	28096	614	231	885	80	128	210	109	192	276.82	194	443.87	467	333	459	44	104	24	4	5
3766	13716	10012	988	358	196	28058	1298	6	511	0	10	63	5	59	271.22	80	424.1	290	255	265	17	42	0	0	2
5088	12784	9228	656	646	386	28160	1121	6	664	1	28	51	3	86	278.88	91	436.06	372	346	339	25	35	0	0	0
5860	12362	8842	587	642	388	28126	1032	32	701	12	30	70	11	109	282.46	124	439.36	387	328	370	20	43	4	2	1
13622	7688	4580	2	1170	882	28066	643	257	924	95	114	222	120	194	276.87	196	445.66	466	362	421	39	108	20	10	1
5054	12848	9258	682	592	360	28140	1150	7	645	3	26	64	4	96	283.5	90	439.42	343	346	323	19	41	0	2	2
5218	12692	9168	639	650	388	28150	1114	8	690	2	34	68	4	96	279.28	110	436.58	373	368	323	17	38	0	0	3
4558	13300	9610	807	388	206	28066	1190	6	670	0	4	67	5	73	274.64	114	429.4	328	323	320	14	45	0	0	4
13126	7804	4824	2	1298	934	28118	611	223	883	86	118	206	98	204	280.93	206	447.37	472	315	437	45	96	15	14	4
13508	7758	4570	0	1188	938	28092	628	262	869	111	116	222	113	219	276.84	182	445.04	450	321	432	33	90	19	14	3
11694	7952	5244	0	1866	1442	28504	512	107	634	26	278	177	54	255	313.21	119	458.15	493	196	405	104	100	6	26	15
4982	12956	9328	698	498	310	28108	1165	8	671	0	32	67	5	97	282.25	97	437.7	333	370	310	17	46	0	2	1
12406	7816	5066	1	1626	1184	28320	578	139	770	35	202	186	76	236	298.34	160	453.11	472	252	424	64	106	10	20	11
4466	13254	9610	804	474	276	28100	1230	5	634	1	20	60	4	75	275.64	92	431.08	314	341	296	20	39	0	0	1
11762	7832	5220	2	1912	1470	28530	506	112	603	27	304	186	55	259	316.47	121	460.61	516	174	375	94	96	12	28	19
3906	13680	9946	961	328	182	28050	1287	7	570	0	8	64	5	72	270.57	98	423.5	272	271	281	17	43	0	0	2
14446	7728	4428	3	688	494	27832	636	325	1139	148	48	261	130	148	269.33	285	430.74	487	448	446	14	109	23	0	0
13652	7676	4536	6	1150	908	28066	625	274	880	102	134	215	136	178	277.19	199	446.21	470	341	454	39	95	19	10	7
13314	7766	4704	3	1246	948	28132	566	201	898	77	142	198	94	195	281.36	200	448.62	501	312	469	44	98	13	12	8
4314	13308	9688	859	486	282	28102	1228	3	589	0	22	54	1	85	277.29	78	433.82	306	292	302	15	36	0	2	3
4086	13506	9826	907	412	234	28076	1261	5	579	1	10	62	1	80	272.92	80	428.14	282	289	291	19	38	0	0	1
14580	7520	4282	8	796	604	27860	622	362	1024	169	74	266	145	171	270.47	249	435.49	469	414	445	17	83	25	4	1
11714	7802	5346	1	1974	1382	28494	548	118	624	34	252	183	48	294	309.53	130	457.58	470	197	367	75	91	12	22	20

SUPPLEMENTARY INFORMATION:Monte Carlo Atomistic Simulation and Machine Learning Analysis of Na-K Eutectic Alloy in Condensed Phases, D. Reitz and E. Blaisten-Barojas, George Mason University, Fairfax, VA 22030

4736	13160	9478	761	448	262	28098	1201	8	659	0	12	56	5	69	279.26	95	434.35	349	357	304	14	29	0	2	1
13592	7656	4538	1	1198	948	28084	613	234	909	90	142	223	115	188	275.52	208	444.91	482	355	435	37	109	12	10	8
12966	7762	4776	0	1418	1104	28224	555	207	775	79	184	212	90	225	291.5	176	450.89	491	269	425	59	93	14	12	7
13444	7666	4706	2	1234	896	28096	629	239	878	85	140	206	116	207	282.94	177	447.35	433	346	449	46	101	14	10	9
14384	7680	4384	5	800	580	27874	640	330	1053	162	46	264	136	169	268.85	261	430.65	476	427	434	11	91	17	0	1
13538	7806	4624	11	1098	860	28056	608	249	907	110	122	237	99	179	278.97	212	446.55	496	355	422	35	97	23	8	9
6344	11890	8468	471	864	580	28222	983	22	703	2	72	80	16	138	285.33	111	442.34	406	342	359	26	52	1	4	1
14244	7598	4356	6	928	716	27930	648	308	1018	159	88	263	108	157	272.89	257	436.91	496	418	407	21	89	21	0	2
13730	7664	4604	5	1142	802	28034	638	275	914	102	76	208	129	165	279.11	229	445.31	486	348	447	38	86	17	14	3
3990	13502	9846	929	474	272	28096	1284	6	557	2	12	48	3	73	272.93	73	426.02	278	274	292	19	29	0	0	3
5182	12732	9220	640	612	354	28136	1096	11	684	0	36	54	4	102	278.61	103	435.5	367	346	356	15	32	0	0	2
14222	7570	4362	5	988	728	27938	620	334	954	148	62	242	138	159	272.04	249	435.56	498	364	452	23	72	25	6	1
13376	7724	4592	1	1260	1020	28142	565	223	867	69	152	212	118	194	283.94	221	448.8	498	289	450	55	120	13	16	4
13384	7626	4648	1	1318	992	28134	553	243	843	92	160	229	105	214	283.07	189	448.28	501	295	434	44	109	14	6	6
3914	13570	9916	971	424	236	28074	1308	4	565	1	12	68	3	74	270.46	87	422.67	274	271	250	17	45	0	2	2
4764	13046	9432	724	554	318	28128	1148	4	643	0	14	58	3	87	278.32	113	435.75	357	311	327	17	41	0	0	1
13568	7564	4512	1	1276	1020	28122	602	225	878	91	170	227	102	221	283.44	193	448.89	461	333	429	41	105	17	12	3
3830	13748	9986	992	304	170	28042	1307	8	558	2	4	64	6	55	270.02	98	423.18	286	263	266	13	37	0	0	4
13136	7784	4772	2	1322	992	28168	612	203	854	64	152	187	96	199	282.94	185	447.38	494	314	443	40	94	22	10	7
4658	13160	9518	759	480	270	28098	1191	6	671	0	12	58	4	70	277.22	96	432.92	343	338	309	20	45	0	0	1
14462	7646	4382	6	770	542	27842	661	334	1084	164	38	270	129	151	269.25	259	432.18	462	468	430	20	95	22	2	1
4670	13156	9502	767	470	280	28100	1196	6	646	1	22	66	2	70	274.94	98	431.07	348	334	298	18	47	0	0	0
5454	12582	9058	589	640	376	28138	1071	8	728	0	26	65	5	110	279.94	113	439.03	372	380	356	16	42	0	2	2
4510	13084	9554	791	622	348	28138	1216	4	587	0	20	54	3	87	275.77	94	431.67	339	300	285	9	38	0	0	3
14072	7614	4448	3	996	742	27968	620	296	961	115	90	232	128	173	277.49	233	442.6	469	373	462	34	101	24	6	1
4490	13318	9618	841	398	236	28078	1234	8	650	1	18	58	5	71	274.96	96	429.95	310	330	301	18	42	0	0	2
13486	7962	4716	5	1010	748	27996	640	243	1020	116	70	222	88	191	275.16	225	439.25	461	413	450	25	87	21	4	1
11594	7880	5360	0	1904	1440	28520	560	89	669	20	300	179	48	257	303.4	127	455.16	496	237	370	95	107	2	38	11
4078	13602	9834	940	328	204	28060	1297	4	606	1	12	61	1	60	271.6	89	423.92	291	293	270	14	34	0	2	3
11180	8004	5440	2	2022	1592	28624	559	77	578	14	346	145	43	287	319.51	119	463.34	468	198	380	97	69	6	38	19
4526	13230	9580	795	482	272	28100	1186	4	629	0	10	59	2	94	279.27	91	434.36	309	304	325	19	38	0	0	2
12290	7940	5090	3	1562	1194	28310	575	171	745	38	216	178	90	245	292.11	155	451.89	472	264	431	62	105	18	14	9
5514	12330	8936	599	842	526	28206	1062	15	609	4	52	68	8	110	284.94	84	440.6	391	312	334	26	41	0	6	2
13510	7604	4608	1	1294	946	28092	589	249	851	96	122	213	113	192	277.34	185	446.66	492	317	458	39	92	17	6	4
10784	7908	5640	0	2268	1704	28734	500	64	546	10	386	166	33	281	325.94	111	466.29	493	166	358	115	80	2	40	38
11758	7936	5234	0	1822	1422	28490	587	105	677	20	284	160	55	254	305.34	131	455.63	474	256	388	84	98	5	34	17

SUPPLEMENTARY INFORMATION:Monte Carlo Atomistic Simulation and Machine Learning Analysis of Na-K Eutectic Alloy in Condensed Phases, D. Reitz and E. Blaisten-Barojas, George Mason University, Fairfax, VA 22030

4544	13290	9566	805	416	256	28086	1254	9	650	1	14	54	5	72	276.74	81	430.04	291	362	302	19	33	0	0	4
4052	13568	9874	940	358	194	28058	1287	6	588	1	12	61	4	66	272.67	93	425.78	291	276	272	14	44	0	0	2
11764	7860	5242	1	1932	1416	28484	533	118	653	26	246	179	58	246	306.78	133	458.98	491	206	411	93	95	11	24	18
14176	7650	4396	7	954	698	27928	649	323	999	166	54	256	126	159	268.96	262	432.18	505	409	416	13	76	14	0	0
4338	13422	9730	848	362	196	28060	1228	7	634	1	12	61	3	75	272.91	93	427.27	311	316	305	12	41	0	0	2
5376	12592	9106	622	670	380	28152	1112	13	679	0	26	69	8	95	283.86	93	438.46	371	364	318	23	50	0	2	2
3924	13678	9908	960	348	204	28064	1299	5	562	1	2	57	4	57	270.51	89	421.78	293	280	274	14	33	0	0	3
4782	12986	9424	750	578	332	28126	1183	9	644	0	24	51	4	93	276.72	104	434.02	335	317	315	14	35	0	0	1
13818	7804	4626	4	924	688	27964	607	290	1007	128	102	227	125	173	275.57	243	438.99	478	383	476	29	79	15	2	2
4428	13254	9642	811	496	272	28108	1226	5	613	0	16	64	2	81	277.71	86	430.7	316	315	291	18	46	0	0	1
6830	11648	8238	383	890	558	28208	936	27	766	9	42	67	14	117	287.99	131	446.26	444	378	399	22	36	1	2	5
14240	7694	4368	1	840	686	27922	632	298	1029	145	88	258	117	174	273.11	252	436.64	472	427	436	23	92	15	6	0
13148	7866	4830	1	1228	906	28122	580	228	892	73	134	194	123	209	281.22	200	447.28	487	314	480	31	84	17	10	7
5642	12372	8872	584	766	488	28182	1075	21	666	6	42	63	9	108	282.15	104	439.37	398	353	333	16	32	0	0	0
5260	12760	9142	625	574	368	28140	1081	8	687	0	34	68	7	91	280.85	97	437.95	372	351	360	23	45	0	2	4
13730	7850	4600	5	978	736	27972	641	270	993	133	74	234	105	168	276.13	237	438.35	487	406	440	21	86	14	4	3
4808	13106	9432	755	460	278	28106	1191	7	689	0	20	63	6	89	276.91	105	430.83	341	349	303	8	52	0	2	1
14192	7748	4430	7	838	630	27890	648	317	1024	168	52	252	115	146	269.63	252	433.16	492	434	432	22	71	16	0	0
4500	13276	9586	783	456	268	28096	1206	8	624	0	10	56	6	79	276.91	99	431.12	320	332	313	19	39	0	0	1
5452	12538	8982	592	696	464	28186	1084	9	697	1	50	59	6	113	283.01	111	438.31	378	351	343	15	41	0	4	2
4308	13458	9752	880	350	182	28054	1247	5	612	0	4	63	2	71	274.91	100	428.72	295	300	299	16	42	0	0	3
13690	7692	4588	7	1092	832	28024	618	277	938	138	120	258	105	191	277.42	206	442.47	484	386	403	30	107	17	10	5
11358	7902	5490	6	2016	1456	28550	518	95	643	18	298	162	53	275	310.65	122	456.84	512	216	395	84	91	3	28	16
6538	11684	8416	427	966	576	28240	972	18	714	2	48	83	10	130	289.67	113	446.28	423	349	345	30	54	0	12	3
13000	7940	4784	4	1264	1016	28170	647	190	862	72	150	202	85	197	283.98	185	449.18	485	341	392	44	96	10	12	8
10564	8030	5680	3	2262	1768	28760	501	63	542	7	406	146	31	266	323.41	104	463.82	538	172	351	121	92	1	46	25
11778	7926	5212	1	1850	1426	28492	551	104	655	27	270	161	51	246	300.83	140	455.72	510	232	406	86	87	6	30	11
4820	13076	9450	723	470	262	28096	1184	5	677	0	18	63	3	79	278.43	102	434.4	339	370	308	17	40	0	0	2
3950	13624	9904	964	366	210	28064	1304	9	568	1	8	55	3	69	271.55	97	424.58	284	267	269	12	40	0	2	1
11048	7838	5574	1	2246	1588	28634	519	74	547	20	310	180	39	298	320.18	102	465.19	468	172	368	102	95	1	26	20
14160	7680	4408	4	890	700	27930	671	297	1015	153	90	251	107	162	274.37	242	438.9	474	433	413	20	80	15	2	2
4494	13252	9600	810	462	270	28098	1211	7	617	1	18	64	4	81	273.95	91	428.94	318	321	304	17	40	0	2	2
14278	7670	4466	5	826	578	27876	654	322	1060	158	56	262	122	155	271.42	268	433.95	484	451	419	16	95	19	2	1
14046	7740	4474	9	924	688	27938	653	296	1013	144	64	224	120	159	273.63	240	436.48	469	419	463	23	72	14	2	3
3838	13720	10004	998	316	160	28042	1320	6	571	1	2	64	4	68	270.17	89	422.08	271	269	260	10	43	0	2	2
4932	12934	9306	674	576	364	28142	1144	7	650	1	30	71	5	96	278.41	102	434.37	356	340	309	16	44	0	0	3

SUPPLEMENTARY INFORMATION:Monte Carlo Atomistic Simulation and Machine Learning Analysis of Na-K Eutectic Alloy in Condensed Phases, D. Reitz and E. Blaisten-Barojas, George Mason University, Fairfax, VA 22030

12534	7934	5012	3	1480	1114	28274	576	168	770	58	182	183	73	220	292.73	163	449.27	508	275	421	58	95	15	16	6
11384	8036	5362	2	1888	1534	28596	540	84	600	15	356	182	41	263	321.42	118	465.25	476	203	358	116	90	8	34	19
4892	12902	9362	709	608	344	28128	1177	10	648	0	20	59	8	87	279.36	84	435.28	332	348	311	20	39	0	0	4
5130	12806	9196	634	604	382	28148	1115	2	695	0	26	68	2	95	278.87	114	434.82	364	358	332	21	39	0	4	2
12386	7876	5032	0	1600	1206	28326	580	149	782	43	212	178	72	235	293.78	149	450.41	502	298	423	54	102	9	12	7
13856	7788	4564	5	948	722	27978	628	267	998	104	98	214	127	173	275.64	241	442.02	469	383	474	31	98	19	2	1
11098	8080	5444	2	1998	1604	28606	522	78	615	15	348	149	33	277	309.89	116	461.73	470	185	392	113	77	5	32	22
13438	7790	4620	4	1188	928	28092	635	233	891	98	110	220	98	185	277.75	204	445.55	496	349	414	36	104	16	18	2
14404	7592	4314	4	834	666	27904	624	328	1035	162	88	259	122	155	271.18	264	434.52	495	421	440	23	91	25	4	0
5580	12518	8988	586	638	382	28138	1097	9	734	1	32	81	4	98	280.37	105	437.51	364	387	328	25	50	0	0	2
11134	7976	5468	0	2080	1592	28614	490	90	569	15	326	165	46	300	310.28	121	460.11	490	175	381	103	86	4	38	18
3954	13584	9888	935	396	236	28078	1276	8	553	1	18	50	4	73	271.5	91	425.49	290	266	288	14	32	0	2	1
11688	7760	5252	0	1972	1514	28548	551	106	633	20	328	166	58	247	312.63	122	458.94	492	223	376	106	91	7	30	17
11160	7990	5492	1	2080	1550	28612	526	94	610	13	308	181	52	286	311.5	125	459.8	502	192	353	93	101	5	30	17
4452	13232	9600	808	492	306	28120	1211	5	620	0	36	50	3	93	275.46	87	431.55	308	318	312	17	36	0	2	2
10922	8010	5556	0	2114	1674	28720	503	81	534	14	394	170	43	300	320.72	115	464.98	493	165	355	109	87	5	46	24
4492	13324	9620	818	406	226	28070	1206	9	630	1	2	60	5	66	273.75	109	426.66	334	316	308	16	42	0	0	2
5582	12512	8978	584	636	400	28158	1109	16	722	1	48	72	8	104	283.52	126	439.19	383	393	308	14	47	1	2	1
11294	7930	5474	1	2040	1510	28590	536	106	556	22	298	165	56	261	320.34	107	464.29	460	184	401	111	85	9	40	25
5076	12854	9264	681	578	334	28120	1129	15	650	3	14	67	8	98	280.53	91	436.59	350	332	331	18	40	0	0	1
3914	13616	9918	968	384	222	28070	1299	6	560	1	16	59	3	73	273.89	83	425.99	279	275	274	13	41	0	0	1
13740	7784	4592	4	1020	768	28004	634	264	992	102	94	210	124	194	276.4	220	440.67	447	393	471	31	88	21	4	1
4748	13022	9438	713	564	334	28134	1142	6	641	0	26	46	4	95	278.4	81	435.02	338	330	358	15	32	0	0	3
14508	7472	4266	3	906	670	27894	615	347	989	165	72	260	135	170	271.82	246	433.93	480	389	449	19	86	25	0	1
5196	12778	9224	630	580	328	28130	1130	7	700	0	24	71	6	96	277.8	102	434.8	361	398	324	15	47	1	0	1
3928	13668	9904	960	338	212	28066	1287	4	567	1	14	56	3	72	271.32	90	424.79	283	269	282	12	37	0	2	3
3854	13700	9978	969	332	180	28052	1305	6	550	0	6	58	3	64	271.29	73	425.85	266	290	280	19	37	0	2	2
4222	13518	9782	879	332	190	28056	1241	6	608	1	12	62	3	67	273.09	97	428.65	306	300	300	14	39	0	0	4
11260	7892	5404	2	2088	1608	28630	508	100	535	18	330	162	63	295	319.48	111	463.59	471	168	398	93	84	2	48	28
12808	7954	4968	3	1350	962	28178	608	188	875	59	128	196	98	231	282.07	172	447.94	457	323	457	40	103	16	8	3
4606	13144	9556	768	500	278	28110	1154	4	635	0	26	59	4	100	278.19	88	433.3	335	300	335	12	48	0	0	1
11176	7800	5386	0	2156	1732	28710	509	76	518	12	394	164	37	322	324.25	84	465.4	447	152	357	118	84	4	64	22
5132	12808	9246	668	570	336	28122	1118	8	689	2	28	74	5	103	281.06	116	436.38	346	342	333	18	45	0	2	3
4074	13580	9842	938	348	206	28064	1284	8	582	1	14	58	6	59	272.12	82	425.14	294	285	281	19	46	0	0	1
4056	13582	9860	917	344	204	28068	1270	4	580	0	22	65	2	76	272.81	84	426.5	289	293	283	14	44	0	0	1
12032	7910	5076	2	1758	1386	28442	545	123	670	42	258	187	64	258	303.83	136	452.58	503	223	400	68	88	1	20	15

SUPPLEMENTARY INFORMATION:Monte Carlo Atomistic Simulation and Machine Learning Analysis of Na-K Eutectic Alloy in Condensed Phases, D. Reitz and E. Blaisten-Barojas, George Mason University, Fairfax, VA 22030

4456	13278	9602	816	454	282	28098	1208	9	628	1	26	62	4	74	274.37	111	428.15	340	306	287	18	40	0	0	0
3984	13594	9876	962	372	224	28068	1296	8	571	1	18	58	5	77	272.67	92	425.44	279	265	268	12	35	0	0	2
5870	12288	8816	553	724	430	28162	1057	16	729	3	34	71	11	102	282.71	104	440.41	390	376	351	25	47	0	0	0
3966	13600	9902	940	376	210	28066	1276	8	558	1	12	59	5	72	271.94	80	424.14	285	279	287	14	36	0	0	3
14334	7796	4420	6	706	542	27842	629	316	1123	161	38	259	122	138	267.26	310	429.44	508	451	444	12	84	11	6	2
4186	13472	9784	906	392	226	28078	1283	4	600	2	14	68	2	66	273.63	84	425.89	297	314	265	13	47	0	4	5
11920	7838	5202	0	1820	1388	28486	529	127	646	29	290	168	75	251	304.42	119	455.62	492	217	431	87	90	8	26	12
4962	12864	9332	713	592	346	28132	1120	11	633	3	36	61	6	90	281.41	97	436.23	354	304	345	20	37	0	0	5
12332	7748	4972	1	1776	1338	28408	544	153	671	27	216	170	81	257	303.77	142	455.33	482	208	417	79	105	16	22	12
11232	7946	5416	1	2082	1596	28640	542	88	570	16	338	159	49	289	319.87	120	463.18	475	179	369	103	88	8	26	21
7136	11310	8082	342	1036	616	28236	924	35	754	9	56	78	18	136	289.79	125	444.44	448	385	374	24	43	1	0	2
5664	12402	8904	552	730	442	28174	1099	9	747	1	28	63	4	97	281.08	114	437.82	378	423	327	24	40	0	4	3
12026	7778	5146	2	1842	1364	28428	555	148	675	39	248	178	79	258	300.47	149	451.89	495	226	409	59	88	16	24	17
4114	13496	9816	918	408	228	28072	1279	7	589	2	8	67	3	77	271.11	82	423.64	289	297	270	10	45	0	2	4
13302	7684	4654	3	1322	1028	28172	585	199	849	64	174	214	100	216	286.6	169	448.6	479	307	437	46	125	15	8	7
4904	12960	9358	737	556	324	28120	1162	15	623	2	18	57	6	82	279.24	88	436.79	344	313	320	23	37	0	0	2
4786	13036	9422	733	540	316	28118	1170	12	647	3	16	57	7	85	277.54	93	432.55	334	340	325	20	39	0	2	3
4010	13536	9862	949	422	236	28076	1266	8	551	1	10	56	7	78	271.7	81	424.94	296	255	287	10	38	0	0	2
5290	12660	9070	653	656	440	28162	1132	11	649	5	44	62	4	95	282.34	92	440.53	373	343	309	14	37	0	2	5
12204	7954	5102	3	1640	1240	28370	581	159	734	46	204	172	83	237	298.91	150	454.6	493	284	410	68	82	6	24	11
7462	11254	7888	304	934	616	28228	895	29	835	11	70	97	14	152	286.95	147	447.24	435	394	384	25	53	1	4	4
10660	7758	5720	0	2446	1764	28792	487	68	462	12	394	197	30	290	331.98	84	473.75	463	143	328	132	96	5	42	45
14070	7698	4436	1	936	728	27958	646	292	1008	142	82	243	113	172	273.89	230	437.83	476	401	431	24	86	16	6	1
13448	7958	4752	6	988	752	28004	627	258	986	117	102	235	103	178	277.21	244	441.74	482	384	424	37	100	12	2	0
5588	12446	8940	590	722	438	28164	1083	13	704	3	30	74	6	116	284.2	115	439.16	378	351	326	15	48	1	0	1
4090	13608	9858	947	312	174	28050	1298	7	591	1	8	60	5	65	271.97	92	424.7	289	287	273	8	34	0	0	3
13246	7638	4660	2	1412	1068	28198	582	212	785	66	164	191	113	225	286.41	173	450.26	484	278	446	47	99	14	10	3
12842	7726	4846	2	1486	1134	28252	589	201	754	69	204	191	97	215	294.86	171	453.11	489	278	413	63	85	13	14	8
11724	7998	5236	2	1752	1426	28474	543	120	685	28	294	167	60	254	302.47	147	453.41	497	241	398	85	97	5	42	13
13402	7754	4560	0	1256	1016	28138	596	240	842	87	140	212	112	207	283.19	176	447.37	467	306	449	48	99	20	6	7
5160	12714	9186	638	676	396	28154	1121	5	663	0	22	61	4	113	280.66	88	438.69	339	354	340	18	36	0	0	3
4582	13264	9564	782	422	244	28086	1217	8	657	0	10	65	6	82	276.42	93	430.97	320	357	294	13	44	0	0	2
11610	8104	5388	3	1780	1312	28448	577	96	725	25	220	186	53	258	303.15	148	452.81	490	247	360	78	101	1	34	13
7010	11436	8086	353	1000	652	28250	899	33	750	8	60	74	16	135	289.7	145	446.75	464	365	386	26	37	1	6	3
5138	12802	9230	615	604	346	28136	1069	8	695	0	16	69	6	91	280.31	111	437.57	403	352	347	13	46	0	0	3
5238	12680	9172	590	656	378	28154	1084	5	690	0	30	58	5	93	279.61	112	436.84	397	374	347	15	37	0	0	1

SUPPLEMENTARY INFORMATION:Monte Carlo Atomistic Simulation and Machine Learning Analysis of Na-K Eutectic Alloy in Condensed Phases, D. Reitz and E. Blaisten-Barojas, George Mason University, Fairfax, VA 22030

13126	7800	4744	5	1314	1010	28150	634	209	847	76	138	202	97	205	282.01	186	446.54	483	319	403	43	98	17	18	3
11238	8016	5454	1	2008	1540	28612	556	82	598	14	314	164	42	258	316.56	109	458.38	497	204	360	97	90	6	38	23
4040	13610	9886	963	324	178	28048	1310	7	600	1	8	58	3	74	271.01	80	423.18	258	295	280	14	45	0	2	2
14518	7578	4298	4	804	612	27878	599	339	1053	164	58	260	141	151	272.05	287	436.31	509	408	455	20	80	11	10	0
4458	13270	9608	813	482	276	28104	1211	4	600	0	10	60	3	81	277.91	93	432.27	328	309	298	13	41	0	0	3
14190	7734	4474	4	794	614	27894	648	318	1085	162	80	264	125	159	270.97	268	433.56	477	452	425	21	94	15	6	1
12946	7796	4828	1	1408	1060	28214	596	192	827	58	156	181	102	204	288.62	175	449.91	480	315	458	58	84	13	18	6
4220	13422	9736	877	438	258	28088	1229	7	566	0	14	53	4	70	274.11	87	428.74	309	268	306	19	34	0	0	4
4628	13206	9554	780	440	244	28086	1181	6	642	1	14	66	4	74	277.2	99	432.15	341	332	313	15	47	0	0	4
4996	12824	9306	690	616	360	28138	1153	16	632	2	36	68	9	83	281.38	92	438.09	353	334	304	27	36	0	0	2
4206	13416	9796	935	422	218	28072	1292	7	607	1	12	50	4	71	272.89	90	427.68	273	284	288	17	39	0	2	3
7326	11362	7942	351	904	614	28220	926	37	775	6	68	99	24	152	288.69	132	446.35	413	388	365	29	59	1	4	4
13788	7662	4524	1	1136	836	28044	622	251	910	101	86	229	113	177	277.74	214	445.9	472	333	441	42	110	20	12	6
4080	13552	9846	927	366	208	28066	1268	5	591	1	12	71	3	64	271.83	106	425.15	303	279	274	15	47	0	2	3
4032	13644	9882	952	308	176	28050	1300	6	589	1	8	61	2	60	272.31	91	424.04	286	295	270	14	45	0	0	3
13214	7742	4602	3	1370	1094	28180	612	235	803	93	146	205	104	193	281.35	184	447.94	518	310	405	45	87	16	10	2
5258	12776	9206	651	542	308	28116	1100	10	728	3	26	62	6	104	280.11	114	436.07	351	361	359	14	40	0	0	4
4502	13202	9594	763	514	286	28114	1154	5	615	0	14	64	4	86	277.24	95	432.91	360	310	319	11	45	0	2	2
11108	7820	5398	0	2204	1740	28702	513	88	524	22	386	173	45	294	314.74	83	463.35	456	176	375	118	89	3	38	26
11276	7830	5320	0	2120	1692	28644	540	88	576	24	356	165	43	265	313.02	126	462.53	501	170	373	93	91	6	42	29
12066	7820	5088	1	1810	1386	28452	574	141	676	33	258	168	66	242	297.45	145	454.17	516	230	384	60	89	13	22	22
4518	13358	9636	834	346	192	28060	1239	6	644	0	10	58	3	84	275.06	85	429.46	296	330	307	10	41	0	0	1
4294	13382	9712	851	434	248	28084	1232	4	607	1	14	59	2	73	273.13	79	426.75	317	311	298	12	41	0	0	4
10832	7634	5656	0	2414	1780	28812	482	87	474	18	430	169	42	305	331.31	76	472.62	472	148	347	126	77	3	56	41
5152	12710	9224	656	660	372	28146	1123	9	685	0	28	71	8	85	279.72	112	437.58	376	345	315	21	46	0	0	3
14348	7716	4388	8	774	584	27860	622	336	1072	163	48	260	139	146	270.87	287	434.42	506	423	444	13	85	15	2	2
11684	7984	5254	0	1864	1426	28498	563	120	663	31	254	167	64	264	305.61	134	455.27	486	220	403	75	94	6	30	12
4082	13564	9830	918	376	214	28070	1290	9	592	0	4	54	5	57	271.69	93	425	309	292	265	16	36	0	0	0
13674	7864	4592	1	1002	778	27998	610	256	977	117	76	228	105	176	275.63	236	440.44	481	378	462	32	91	17	10	3
13566	7700	4610	3	1166	896	28090	640	240	887	89	148	202	112	172	281.02	211	446.39	477	345	442	46	86	17	4	6
11540	7956	5380	0	1916	1428	28532	524	95	675	21	268	166	44	278	315.48	132	463.07	492	224	395	82	92	6	44	20
14318	7720	4440	5	766	556	27850	635	334	1064	169	50	271	113	141	270.49	283	433.14	501	432	424	16	90	23	0	3
13638	7780	4616	4	1050	810	28012	600	258	969	122	104	241	102	200	276.07	226	442.83	466	362	447	32	92	19	14	3
11568	8024	5334	1	1804	1440	28530	525	112	640	22	328	171	64	261	311.6	150	461.78	482	205	400	96	82	6	32	23
13584	7734	4590	2	1156	878	28056	609	244	917	105	102	241	98	192	278.96	210	445.53	491	331	412	33	119	18	10	6
4702	13174	9520	752	436	242	28090	1180	3	685	0	16	59	1	74	277.43	113	433.42	343	346	320	17	42	0	0	0

SUPPLEMENTARY INFORMATION:Monte Carlo Atomistic Simulation and Machine Learning Analysis of Na-K Eutectic Alloy in Condensed Phases, D. Reitz and E. Blaisten-Barojas, George Mason University, Fairfax, VA 22030

4794	13274	9508	770	298	166	28044	1174	11	689	0	2	65	10	82	275.22	111	432.18	315	353	341	14	46	0	2	3
5186	12652	9184	612	712	406	28170	1106	6	682	0	30	65	4	87	279.04	111	434.02	410	358	313	12	47	0	0	2
11282	7980	5504	0	2004	1482	28600	522	76	643	11	312	183	40	242	322.63	135	462.53	515	203	363	101	115	6	36	27
14522	7622	4340	5	750	554	27844	649	328	1097	174	54	272	113	139	271.1	270	433.17	492	453	421	20	88	20	2	0
13276	7716	4704	0	1310	980	28136	606	236	867	79	136	209	116	204	282.01	201	447.38	469	326	438	48	106	16	14	6
10710	7868	5680	1	2284	1754	28796	493	74	505	6	454	154	36	323	333.3	113	471.25	495	151	359	91	80	11	44	36
3938	13652	9924	947	342	192	28058	1294	6	568	1	10	50	4	71	270.58	77	422.89	277	291	287	9	36	0	0	5
12878	7612	4788	1	1594	1198	28292	573	194	711	69	212	194	81	225	290.63	157	450.25	517	240	403	58	87	15	10	5
14376	7756	4386	10	710	566	27860	614	326	1103	155	62	241	135	153	270.96	292	434.57	487	420	478	18	78	16	4	0
6948	11414	8152	379	1002	638	28234	926	36	719	6	78	91	22	138	289.55	110	443.89	424	344	368	30	61	2	0	9
4940	12914	9304	685	588	368	28146	1158	3	659	0	32	61	2	86	279.73	89	434.83	348	349	318	22	42	0	0	3
11488	7904	5384	1	1940	1486	28560	514	111	607	25	306	172	49	267	317.84	132	459.07	504	200	368	105	88	3	48	16
10330	7786	5720	0	2538	1994	28926	474	59	407	7	468	157	24	293	332.86	93	471.53	494	120	336	149	70	2	84	44
3786	13754	10020	994	298	170	28046	1313	8	555	1	16	59	5	66	270.97	83	424.62	268	275	271	15	42	0	2	2
12638	7906	4998	6	1434	1068	28236	577	169	822	60	172	198	83	222	285.7	186	449.64	492	296	435	53	84	10	20	5
11568	7888	5342	2	1956	1452	28520	510	116	637	26	292	186	61	263	310.15	114	455.85	471	206	406	108	103	7	20	17
4138	13516	9824	875	384	202	28068	1231	3	568	1	4	64	2	68	275.12	98	430.11	318	283	294	15	38	0	0	3
14462	7500	4272	3	948	678	27904	596	343	968	159	40	251	137	167	272.52	241	437.55	495	378	459	22	80	18	4	0
11260	7814	5434	0	2192	1594	28640	537	88	551	23	322	161	45	280	317.98	101	460.85	512	178	366	90	79	7	22	17
4362	13482	9756	875	282	140	28028	1224	5	636	1	6	63	1	63	275.19	103	431.53	306	299	316	18	46	0	0	2
5600	12556	8972	611	594	376	28136	1105	21	738	3	36	70	13	110	281.61	119	438.74	355	375	333	18	49	0	2	1
3900	13700	9944	981	320	180	28048	1310	6	579	1	4	54	4	61	270.13	90	422.6	280	274	270	13	32	0	0	3
4400	13320	9648	795	448	260	28090	1195	5	598	0	14	62	3	76	273.99	86	429.38	329	311	315	16	41	0	0	3
14394	7520	4322	9	912	678	27900	617	342	996	147	66	239	145	158	272.71	221	438.42	468	403	475	32	79	16	4	1
14366	7648	4358	5	824	628	27890	601	335	1025	158	56	263	141	162	273.96	249	438.62	496	406	455	19	89	20	10	1
4242	13436	9734	879	416	242	28080	1247	9	582	1	10	47	4	68	272.68	93	426.73	318	292	297	12	35	0	0	2
13648	7838	4656	5	1020	748	27998	650	245	995	114	80	233	108	171	275.71	219	440.56	460	406	441	34	99	14	8	5
4424	13320	9658	825	418	240	28080	1230	10	619	3	18	70	4	80	275.87	82	430.09	309	325	290	15	45	0	2	1
4924	12834	9326	657	652	386	28162	1109	8	648	0	38	72	5	86	280.28	98	439.39	377	336	318	28	44	0	2	3
13710	7798	4636	7	994	740	27982	645	280	982	140	104	248	112	187	273.89	225	438.23	450	397	429	31	95	10	0	3
4170	13538	9796	921	340	202	28060	1282	9	616	2	14	63	4	80	273.86	96	427.09	281	296	274	11	41	0	0	2
4198	13528	9814	898	326	172	28046	1274	10	624	1	8	59	4	76	272.29	86	426.02	285	326	286	10	42	0	0	2
4392	13332	9658	831	434	256	28092	1223	3	637	0	18	70	1	81	276.13	103	430.63	321	315	281	15	48	0	2	2
14282	7646	4384	5	850	656	27900	641	328	1012	163	78	250	125	159	273.76	246	436.61	470	422	445	20	75	15	4	6
4262	13474	9760	875	356	198	28060	1240	5	605	2	10	68	3	78	273.12	97	426.18	305	297	291	11	40	0	0	2
12766	7834	4824	1	1504	1158	28268	597	183	798	54	162	175	102	222	286.17	164	450.47	493	301	450	43	95	14	20	9

SUPPLEMENTARY INFORMATION:Monte Carlo Atomistic Simulation and Machine Learning Analysis of Na-K Eutectic Alloy in Condensed Phases, D. Reitz and E. Blaisten-Barojas, George Mason University, Fairfax, VA 22030

13940	7804	4576	5	886	648	27926	620	283	1047	134	66	251	108	181	273.83	255	438.63	479	398	442	15	107	20	6	3
4766	13144	9468	762	440	256	28088	1188	5	676	1	12	61	1	94	276.79	90	432.96	307	339	325	18	41	0	2	1
11196	7804	5508	0	2152	1602	28678	535	85	547	11	382	163	46	304	324.68	100	469.28	443	189	363	121	91	6	34	20
5488	12712	9068	643	488	312	28102	1108	19	696	2	30	63	15	105	281.52	107	437.97	355	357	348	10	37	0	4	2
4582	13124	9524	774	552	322	28126	1191	11	589	0	22	65	7	70	277.39	100	434.96	373	316	277	16	38	0	0	0
4530	13198	9590	794	500	272	28102	1205	3	626	0	10	51	3	83	278.09	91	432.66	323	313	317	16	37	0	2	0
4578	13278	9556	814	410	250	28086	1225	8	646	0	12	50	4	66	274.27	110	427.6	334	326	299	15	34	0	2	1
14386	7718	4428	6	740	528	27840	628	317	1097	158	34	276	119	157	269.49	276	431.15	483	438	433	15	105	19	6	1
11618	7832	5356	0	1932	1440	28520	540	98	634	26	310	196	48	247	302.51	141	456.89	527	193	359	85	101	7	30	18
3908	13602	9916	962	406	230	28074	1299	6	544	0	12	58	3	64	272.83	78	425.3	285	273	270	14	38	1	0	5
4220	13496	9784	889	352	196	28060	1260	9	593	2	12	73	6	79	273.45	96	428.17	295	288	277	13	48	1	0	0
10802	7700	5614	1	2400	1796	28792	504	74	478	6	422	167	49	300	325.29	86	470.57	474	156	346	130	79	4	58	30
4876	12844	9344	698	668	392	28160	1152	8	633	1	36	53	7	89	280.79	106	434.24	364	324	319	14	37	0	0	4
4554	13256	9584	792	436	246	28088	1219	7	667	0	12	61	5	88	275.04	92	429.5	307	353	309	14	43	0	0	0
12802	7782	4860	0	1502	1120	28252	621	177	802	43	178	159	106	197	286.39	176	447.71	497	321	443	48	79	6	8	11
7102	11534	8076	379	844	582	28212	918	30	795	4	66	71	18	137	288.36	124	445.73	426	380	404	28	51	2	8	2
7532	11048	7804	342	1070	702	28240	877	44	726	7	76	97	26	129	290.94	133	444.5	483	343	371	25	66	1	8	5
5222	12710	9160	626	640	392	28162	1126	9	691	1	36	65	7	96	280.51	120	436.1	379	367	312	13	41	0	2	3
4034	13602	9880	941	338	188	28052	1271	6	577	1	10	58	4	61	273.14	94	426.63	295	270	287	18	41	0	0	2
4366	13408	9696	854	372	214	28070	1227	4	636	0	14	59	3	70	272.28	117	424.6	321	300	308	11	35	0	0	2
10554	7882	5770	1	2332	1766	28804	538	62	475	8	446	155	31	297	332.18	85	470.05	471	150	313	124	78	5	48	35
14124	7730	4458	6	854	664	27918	614	320	1026	158	86	248	125	163	271.08	270	433.86	504	407	446	14	78	14	2	3
7874	10862	7694	309	1048	656	28222	918	52	799	15	82	96	27	159	287.38	126	444.61	397	402	376	35	53	3	6	4
12620	7952	4990	8	1388	1076	28234	644	165	868	59	188	186	79	190	282.45	191	448.1	487	335	408	60	92	9	16	6
4644	13214	9534	798	436	248	28086	1212	8	672	0	10	64	6	71	276.15	102	430.13	323	337	305	18	39	0	0	3
4678	13076	9492	727	554	308	28124	1162	6	642	0	16	65	6	95	278.33	88	434.79	338	342	321	15	45	0	0	2
13828	7760	4560	7	986	750	27982	596	287	995	118	84	212	128	173	276.2	228	441.9	481	380	488	35	86	18	12	1
3986	13650	9890	937	326	196	28062	1276	8	582	1	14	52	7	78	271.94	85	424.75	266	280	307	19	38	0	0	0
5964	12182	8732	493	764	492	28202	1042	6	765	2	64	66	3	124	283.36	112	441.12	396	396	351	15	44	0	4	2
4292	13382	9734	856	410	234	28078	1235	5	603	1	26	62	3	83	274.05	85	427.45	294	304	302	17	46	0	0	2
4496	13232	9608	821	472	264	28088	1220	8	620	2	16	62	4	83	275.56	84	428.86	321	320	297	11	42	0	0	1
4340	13300	9654	840	498	298	28110	1223	4	573	0	16	52	1	84	277.1	82	430.56	324	285	297	12	41	0	4	1
10672	7942	5654	1	2318	1750	28764	522	57	507	7	374	155	34	281	322.65	89	465.24	487	153	344	117	85	2	52	36
4114	13558	9826	925	356	202	28064	1289	9	595	1	8	48	4	64	272.05	91	425.78	283	305	293	15	32	0	0	3
12096	7828	5124	3	1750	1328	28402	582	145	726	43	250	177	65	238	292.73	158	450.32	511	249	393	63	91	16	26	12
11810	7914	5154	0	1808	1466	28486	553	128	663	30	298	151	73	248	301.47	161	454.44	518	216	414	73	70	9	32	10

SUPPLEMENTARY INFORMATION:Monte Carlo Atomistic Simulation and Machine Learning Analysis of Na-K Eutectic Alloy in Condensed Phases, D. Reitz and E. Blaisten-Barojas, George Mason University, Fairfax, VA 22030

3966	13646	9906	942	330	194	28056	1268	4	570	1	12	54	1	68	270.48	84	424.86	290	275	297	14	38	0	2	3
5832	12482	8878	581	544	340	28108	1095	23	748	6	32	78	13	103	281.67	124	438.86	368	389	331	15	54	0	0	1
13544	7606	4578	3	1264	962	28108	579	239	873	78	142	222	107	203	282.41	194	447.23	504	318	423	40	118	18	10	5
4768	12922	9386	704	656	396	28164	1157	5	624	1	36	56	3	77	276.95	106	433.81	381	325	306	17	32	0	0	2
14442	7622	4344	8	780	604	27870	628	331	1080	156	72	263	131	155	271.66	269	434.32	482	430	449	18	93	23	6	2
12942	7982	4942	7	1228	888	28104	629	196	952	80	108	217	86	210	281.76	197	443.94	482	367	414	29	107	10	10	3
4268	13398	9728	868	434	244	28082	1253	2	620	0	8	59	1	69	274.04	98	427.44	307	308	293	14	41	0	2	3
5082	12832	9250	673	596	354	28138	1130	7	670	0	24	56	5	94	278.72	114	434.23	364	337	334	14	36	0	0	2
4210	13450	9798	895	404	202	28068	1261	4	599	0	4	60	1	81	275.6	90	428.11	291	287	289	13	43	0	0	0
13516	7844	4636	4	1096	842	28042	622	266	937	114	106	219	113	181	277.69	237	443.51	474	355	443	38	89	22	2	6
4676	13176	9520	757	442	258	28096	1175	4	656	0	24	59	2	82	277.69	94	433.2	331	343	330	16	42	1	0	2
14316	7668	4392	3	828	616	27876	607	325	1033	155	50	265	132	158	271.43	261	434.37	479	400	458	27	99	17	4	1
4898	13054	9370	686	462	298	28114	1116	3	687	0	26	58	2	89	277.85	106	431.71	359	342	355	16	34	0	6	3
11690	7918	5342	2	1828	1386	28506	578	100	677	19	322	167	57	244	307.8	126	455.56	493	241	389	80	102	5	20	17
5348	12570	9134	658	672	376	28134	1143	16	659	2	30	69	12	109	281.69	85	439.38	320	350	329	22	54	0	4	3
12490	7940	4930	2	1504	1210	28304	589	164	794	55	214	197	91	247	285.13	165	450.24	480	306	413	48	106	6	16	9
14580	7612	4282	7	724	578	27850	648	330	1104	174	68	270	120	143	268.53	282	430.14	502	451	423	10	84	19	6	1
13552	7724	4618	3	1176	884	28080	616	240	874	81	114	214	109	210	282.68	186	447.31	461	344	445	34	100	28	12	2
4580	13138	9556	787	534	290	28112	1204	6	635	0	14	69	0	86	279.13	96	435.24	340	318	280	10	43	0	0	3
13970	7734	4516	7	938	702	27944	645	292	1009	135	82	242	113	155	273.81	240	439.27	487	404	439	23	94	21	2	4
4618	13230	9574	784	408	230	28084	1203	7	653	1	24	65	2	83	277.55	92	432.63	313	339	310	17	39	0	0	3
14288	7684	4368	4	848	656	27912	636	325	1031	169	62	266	123	143	271.9	277	434.3	520	419	409	21	89	16	4	0
4082	13546	9844	947	366	210	28064	1299	10	581	2	14	60	6	72	270.45	89	423.72	272	291	271	16	40	0	2	3
5204	12900	9264	699	454	246	28076	1168	16	720	5	8	87	8	79	279.51	116	435.19	352	369	290	17	60	0	0	0
4066	13592	9848	921	346	200	28060	1279	6	606	1	8	67	4	66	271.45	97	424.2	298	301	270	12	38	0	0	2
11268	7904	5508	0	2108	1494	28590	536	87	626	21	266	174	38	285	315.58	124	461.71	491	195	365	94	98	6	42	15
13122	7816	4796	1	1266	974	28154	621	215	886	73	170	182	110	196	285.45	189	446.99	497	337	446	38	82	12	10	3
4360	13426	9720	879	350	190	28056	1273	9	635	1	10	57	4	70	273.4	94	425.39	291	338	284	14	42	0	0	2
5768	12356	8846	555	716	446	28170	1065	10	733	4	32	73	3	109	286.74	111	441.34	397	370	329	15	50	0	6	3
5524	12496	9002	626	672	408	28140	1079	20	685	6	34	81	12	114	281.35	112	437.68	363	324	335	17	56	0	4	4
4134	13464	9784	899	436	254	28086	1274	4	584	1	14	63	3	76	273.02	85	427.34	285	289	277	18	37	0	0	3
4578	13214	9552	818	460	272	28094	1221	7	620	0	16	62	3	84	276.18	97	432.26	324	318	287	12	41	0	2	1
13782	7768	4550	4	1004	780	27990	608	292	963	131	100	231	123	178	275.42	222	439.56	481	375	459	28	88	18	6	5
4590	13248	9580	792	402	234	28078	1196	8	646	1	22	56	3	69	272.95	94	428.4	323	328	322	22	40	0	2	3
12264	7808	5020	2	1700	1328	28400	557	152	697	36	254	173	79	280	303.34	140	453.29	468	248	413	62	91	10	24	12
5754	12270	8858	554	778	466	28170	1056	20	686	3	42	74	12	122	284.25	99	440.32	375	370	334	24	44	1	2	1

SUPPLEMENTARY INFORMATION:Monte Carlo Atomistic Simulation and Machine Learning Analysis of Na-K Eutectic Alloy in Condensed Phases, D. Reitz and E. Blaisten-Barojas, George Mason University, Fairfax, VA 22030

14408	7576	4332	3	858	644	27888	622	334	1026	164	66	252	127	158	272.82	255	437.26	477	413	457	24	77	25	2	2
11120	8040	5470	2	2088	1572	28614	528	97	576	18	288	161	50	258	314.16	109	460.71	502	194	394	100	77	9	32	21
11108	7876	5432	1	2202	1672	28666	540	79	519	17	344	171	37	291	316.03	92	461.13	465	183	364	109	86	6	30	24
12346	7800	4940	5	1712	1328	28372	592	159	707	47	240	160	79	247	289.61	138	452.93	479	269	425	68	80	11	6	4
4462	13346	9646	839	384	220	28072	1239	6	648	0	12	53	4	78	274.86	100	429.09	297	337	315	10	36	0	2	5
4956	13072	9408	712	408	218	28070	1184	7	729	1	6	69	2	76	276.58	102	434.45	325	398	315	22	44	0	2	1
3924	13656	9944	973	326	184	28052	1310	8	558	1	16	65	4	62	271	96	423.98	282	274	258	15	46	0	2	2
10804	7798	5656	0	2250	1758	28800	497	66	498	15	464	181	28	301	333.02	86	469.08	469	166	338	138	84	4	68	32
3918	13668	9932	966	344	190	28056	1286	10	574	1	4	61	6	69	270.63	108	423.52	288	256	277	12	46	0	0	2
5532	12554	8994	606	642	398	28156	1095	16	701	1	36	60	10	108	282.4	112	439.05	378	366	337	13	40	0	0	1
5188	12718	9194	645	638	374	28142	1125	12	670	2	30	60	9	90	280.66	95	437.95	359	353	333	20	37	1	0	5
12024	7972	5158	1	1710	1288	28392	548	119	716	28	226	171	59	217	302.33	166	458.49	514	225	423	75	97	9	14	16
12414	7804	5044	0	1626	1208	28336	577	162	763	43	220	191	92	234	296.78	167	452.28	484	262	408	62	107	8	20	15
13922	7834	4504	9	892	712	27950	632	295	1013	150	84	248	115	186	274.45	242	439.45	456	405	444	25	85	14	2	2
13324	7716	4688	1	1278	970	28136	562	228	866	81	148	204	115	216	279.98	184	447.65	486	296	461	42	103	6	12	7
12546	7724	4840	1	1678	1324	28356	592	171	706	54	216	194	83	242	293.11	136	450.85	460	260	409	71	100	12	24	11
13182	7904	4738	4	1214	948	28128	609	225	883	83	138	210	109	197	281	206	445.62	496	337	425	41	100	10	4	3
4420	13406	9686	842	342	190	28054	1225	11	638	1	10	56	4	83	275.13	100	429.19	299	306	313	12	37	0	0	2
11396	7856	5294	1	2022	1640	28618	588	105	595	18	358	153	59	261	311.49	122	457.71	487	218	361	88	87	7	42	23
3720	13758	10034	991	340	192	28056	1296	6	538	2	12	59	3	62	271.64	86	424.89	288	255	272	16	35	0	0	1
6066	12128	8688	479	790	470	28172	1011	23	727	5	28	79	14	105	281.51	117	440.17	422	386	352	17	44	0	2	6
11080	7970	5538	2	2122	1564	28620	520	88	576	17	314	182	47	289	318.23	105	465.1	490	178	359	96	97	10	30	24
11136	8128	5462	4	1972	1550	28590	543	71	607	19	296	176	31	278	314.87	116	459.27	476	205	360	104	82	2	40	15
4134	13606	9824	929	304	178	28052	1265	9	592	1	6	58	6	71	271.3	92	424.41	294	277	297	10	30	0	0	2
11204	7848	5554	0	2090	1528	28604	518	109	613	28	334	184	42	292	318.54	110	462.88	446	208	368	115	101	10	46	22
4442	13254	9618	783	492	282	28104	1192	0	628	0	16	75	0	81	277.79	90	431.02	329	326	296	20	51	0	0	3
5252	12604	9104	609	732	454	28188	1100	6	667	1	36	66	3	85	281.05	122	436.65	405	344	315	21	38	0	6	3
13194	7742	4746	2	1322	988	28152	569	218	827	81	152	215	94	214	281.98	192	450.23	498	283	442	38	103	22	8	8
12498	7936	4958	4	1524	1174	28292	561	162	776	48	194	175	86	216	291.78	185	450.15	514	269	445	65	91	6	8	3
4494	13202	9616	770	504	268	28096	1172	6	632	0	10	68	4	90	277.21	83	432.88	322	313	317	21	47	0	2	2
13612	7658	4548	1	1208	934	28104	624	233	896	82	132	198	118	169	279.13	218	445.59	513	358	440	38	88	12	10	3
4426	13272	9626	837	472	284	28108	1220	4	611	0	26	73	3	79	276.33	104	432.32	333	288	278	13	52	0	2	2
5298	12712	9154	636	596	342	28120	1108	15	685	2	18	74	7	99	279.26	100	434.32	373	357	324	11	48	0	0	3
4166	13502	9810	902	372	202	28060	1273	9	586	1	4	63	5	71	271.23	78	425.39	286	311	287	14	38	0	4	3
3908	13730	9938	981	292	172	28044	1284	7	543	1	4	68	5	73	273.14	92	425.62	280	253	276	11	42	0	0	3
4624	13152	9524	752	508	288	28110	1192	5	629	0	14	56	2	73	276.75	90	431.25	336	339	317	19	37	0	0	3

SUPPLEMENTARY INFORMATION:Monte Carlo Atomistic Simulation and Machine Learning Analysis of Na-K Eutectic Alloy in Condensed Phases, D. Reitz and E. Blaisten-Barojas, George Mason University, Fairfax, VA 22030

11308	7910	5420	1	2034	1554	28596	523	87	586	14	336	163	49	286	314.86	112	461.49	492	185	383	91	81	3	32	17
12578	7836	4870	0	1556	1246	28324	599	163	737	58	214	203	68	245	290.46	140	451.66	475	266	372	68	107	12	24	6
14456	7518	4284	3	890	676	27902	614	342	991	166	72	267	129	171	273.85	236	439.61	468	397	440	31	88	19	6	0
3936	13742	9960	976	252	136	28032	1305	7	582	1	6	51	4	68	269.65	86	422.75	260	291	297	13	30	0	0	2
3766	13720	9992	983	370	212	28068	1299	9	502	1	8	55	5	59	271.9	72	424.08	288	248	274	19	31	0	0	1
4014	13592	9870	937	362	214	28068	1286	8	573	2	14	54	4	67	272.48	97	424.17	285	286	282	16	35	0	2	3
14294	7746	4426	4	772	578	27868	650	317	1080	163	48	256	109	148	271.84	263	435.71	489	444	435	15	83	23	4	1
4346	13278	9656	827	504	304	28120	1229	3	572	0	30	58	3	73	277.95	79	434.4	312	298	297	21	38	0	2	6
13728	7870	4622	6	954	710	27956	630	304	989	129	68	237	130	193	273.98	224	439.42	443	397	455	27	93	19	4	3
11994	8016	5186	1	1660	1282	28404	579	126	739	31	248	165	54	230	300.49	160	452.75	510	246	391	73	98	11	16	10
12076	7912	5106	3	1752	1326	28418	575	127	713	27	224	172	68	265	304.72	144	454.52	459	247	414	74	97	14	22	12
13510	7836	4662	2	1082	834	28040	625	249	937	120	98	227	102	181	276.21	227	442.33	486	358	434	31	92	12	16	5
5182	12788	9208	638	576	348	28134	1104	9	719	0	30	64	7	107	276.83	124	433.84	363	361	340	13	44	0	2	2
3938	13748	9948	983	248	142	28030	1309	7	591	1	6	53	4	62	272.19	91	423.9	264	281	287	16	36	0	0	2
4164	13546	9808	909	336	192	28056	1251	7	595	1	10	71	4	81	272.78	86	426.75	284	286	291	17	50	0	0	1
4760	13068	9442	743	512	306	28112	1156	5	647	2	22	57	1	82	276.2	106	431.91	348	319	331	17	33	0	2	3
13112	7642	4662	3	1494	1138	28216	614	198	790	83	148	206	92	213	282.72	149	449.93	480	317	411	51	92	7	20	7
4158	13514	9802	905	372	210	28066	1273	8	588	1	10	61	5	77	273.19	94	426.71	288	292	282	13	41	0	0	1
4444	13270	9618	838	472	276	28098	1232	8	634	0	18	54	3	84	274.73	99	429.27	315	312	297	10	40	0	0	2
14372	7514	4358	7	946	658	27906	630	319	991	165	58	268	118	161	272.19	236	436.4	493	398	423	20	93	18	0	1
11020	8018	5612	1	2112	1536	28644	508	76	566	14	302	160	41	271	317.74	105	463.78	501	180	388	96	87	6	40	28
4340	13438	9716	873	364	200	28062	1234	9	626	2	4	48	7	72	274.37	113	427.42	309	295	320	8	36	0	0	4
4902	13048	9400	723	464	262	28088	1180	9	672	1	12	63	6	72	273.72	103	429.92	350	355	311	16	43	0	0	1
4088	13608	9856	932	306	178	28050	1298	6	618	1	14	65	5	76	272.18	89	425.07	268	313	270	13	46	0	0	3
4136	13450	9790	895	442	254	28090	1274	8	580	0	18	48	5	88	273.78	79	427.87	282	296	286	11	33	0	0	2
4482	13302	9626	802	424	238	28084	1216	9	635	1	12	58	5	70	278.8	89	433	328	340	298	19	42	1	0	1
3844	13644	9940	981	394	236	28076	1308	8	531	2	16	55	5	70	270.26	85	422.02	263	252	274	21	39	0	2	3
4018	13660	9900	940	300	162	28042	1289	3	597	1	2	58	2	67	270.95	95	424.5	281	297	286	12	38	0	0	2
14016	7738	4504	6	920	682	27932	621	302	979	152	66	244	112	152	273.15	243	437.31	501	391	452	25	80	18	6	1
12660	7766	4866	4	1544	1214	28288	567	192	736	64	224	193	89	224	291.84	154	449.79	474	262	435	71	94	10	14	12
14374	7724	4406	4	740	556	27856	654	311	1113	164	54	273	104	162	270.89	254	436.07	466	449	416	18	98	20	2	1
5408	12660	9102	616	592	340	28122	1099	12	709	1	20	77	11	94	281.45	98	437.23	383	359	330	12	59	0	0	2
11780	7934	5194	0	1854	1440	28498	549	95	661	19	260	156	52	280	306.12	136	456.05	463	208	426	79	92	7	32	15
13600	7798	4620	4	1074	824	28030	634	261	952	129	104	232	95	174	276.45	224	443.05	479	383	424	39	86	15	10	3
4160	13534	9824	935	346	186	28060	1287	7	609	1	10	58	6	73	272.48	93	426.8	287	290	281	11	38	0	0	0
6648	11752	8402	437	842	488	28168	997	29	784	7	34	80	14	126	286.47	113	444.64	414	405	342	19	54	0	2	2

11158	7866	5462	1	2150	1636	28674	505	92	565	16	366	142	49	275	316.1	119	466.29	493	175	409	106	76	6	36	25
4036	13684	9882	958	276	160	28040	1271	10	584	1	2	60	7	68	271.15	106	423.11	286	257	293	15	39	0	0	1
4700	13138	9504	732	470	268	28100	1159	5	652	0	18	56	3	84	278.42	90	434.76	344	343	336	13	31	0	2	3
4982	12936	9336	704	530	310	28120	1137	3	677	0	24	62	3	85	276.34	106	432.11	355	332	338	21	47	0	2	0
3920	13706	9952	983	298	162	28044	1310	7	570	1	6	63	4	81	272.04	89	424.81	257	272	263	13	45	0	0	2
3914	13692	9928	972	314	192	28056	1316	5	571	1	16	64	3	67	270.69	95	423.7	273	284	263	14	45	0	0	1
12096	7860	5092	1	1748	1350	28416	556	132	702	39	230	182	58	252	299.96	146	452.53	477	232	413	83	95	9	38	9
4986	12876	9306	664	596	346	28130	1153	9	669	0	20	60	7	104	278.15	86	437.21	332	374	322	19	44	0	0	1
6126	11972	8606	447	912	562	28230	1017	15	733	0	46	68	12	125	287.75	108	444.23	422	383	334	18	43	0	4	2
12092	7900	5104	0	1762	1316	28410	607	115	713	36	222	177	55	224	293.35	159	453.94	525	256	365	69	102	10	14	7
11236	7936	5484	1	2030	1548	28620	552	75	599	18	348	181	35	289	315.84	116	460.68	477	198	341	98	99	2	32	21
4098	13506	9830	896	402	224	28074	1270	2	601	1	14	57	1	83	273.87	90	427.48	282	302	290	14	39	0	0	0
14102	7730	4474	10	844	660	27902	647	327	1027	162	90	274	126	157	270.76	263	434.38	497	412	404	13	102	24	2	4
4494	13208	9598	784	498	284	28104	1199	4	641	0	22	63	4	86	275.02	105	430.79	324	331	310	14	39	0	0	2
13342	7710	4650	2	1300	986	28132	621	221	830	73	132	200	109	202	280.61	164	446.1	470	324	442	41	95	16	12	7
12150	7956	5150	2	1622	1240	28378	532	134	734	30	236	179	70	254	304.32	166	456.98	511	222	412	67	100	11	20	6
11368	7862	5382	0	2068	1568	28612	532	97	577	24	328	179	48	301	314.9	127	458.41	479	185	357	90	86	6	32	22
6262	12158	8646	468	646	390	28140	999	30	788	5	36	86	20	111	282.89	124	439.3	419	414	361	15	54	1	2	2
13552	7876	4682	5	1032	768	28000	645	266	971	116	82	216	117	175	276.12	230	440.56	467	393	455	27	80	11	8	5
4548	13220	9558	779	470	284	28102	1193	9	618	0	20	42	3	83	274.35	86	429.39	318	320	333	19	29	1	2	2
5582	12362	8916	562	778	498	28200	1050	14	679	2	62	65	10	109	286.28	112	443.19	385	347	354	25	36	0	2	6
4078	13514	9844	914	406	220	28072	1250	8	571	1	10	52	4	78	272.07	90	425.87	293	269	305	14	42	0	0	2
5110	12870	9248	667	542	328	28122	1099	7	697	0	24	62	2	88	279.24	116	435.19	377	338	343	18	44	0	0	3
4480	13298	9594	836	444	264	28088	1229	9	610	1	8	75	6	98	273.9	103	428.79	296	304	281	14	47	0	0	1
4120	13576	9844	942	312	180	28050	1278	11	584	1	18	56	8	71	273.13	90	426.42	286	284	291	12	39	0	0	1
14260	7684	4428	7	832	616	27884	648	322	1074	168	60	267	123	167	267.66	260	430.04	462	448	425	21	92	11	4	1
4744	13124	9462	738	466	282	28102	1153	8	643	0	24	61	5	110	279.32	97	432.37	315	325	343	12	40	1	0	2
4778	13014	9402	736	568	346	28134	1183	3	647	0	26	68	2	94	278.02	93	435.03	336	340	291	19	47	0	0	1
14200	7572	4404	2	976	710	27942	590	319	964	149	78	239	129	184	273.03	230	437.94	476	360	473	26	79	18	2	1
7000	11596	8136	336	862	568	28216	941	24	835	3	50	58	16	105	288.18	150	443.93	460	420	396	25	36	0	2	4
14592	7602	4292	7	746	558	27840	652	345	1100	177	46	271	127	134	269.74	293	432.32	488	444	426	22	84	22	4	1
4220	13526	9758	885	332	210	28062	1251	8	601	0	14	60	5	81	275.54	88	428.98	292	297	292	13	40	0	2	2
13428	7762	4610	1	1232	956	28124	582	230	854	77	126	191	99	188	282.24	198	444.69	528	318	446	37	92	14	8	3
10844	7908	5506	1	2236	1790	28752	495	87	514	9	416	158	44	284	320.79	109	462.61	509	144	366	124	84	10	50	19
4280	13462	9750	864	366	198	28060	1231	11	612	1	4	54	7	74	274.04	93	428.73	297	297	317	18	37	0	0	2
7520	11130	7818	359	974	682	28234	924	45	754	17	102	105	21	149	287.11	117	443.51	409	368	352	42	53	1	8	3

SUPPLEMENTARY INFORMATION:Monte Carlo Atomistic Simulation and Machine Learning Analysis of Na-K Eutectic Alloy in Condensed Phases, D. Reitz and E. Blaisten-Barojas, George Mason University, Fairfax, VA 22030

4134	13468	9782	921	436	254	28086	1278	11	572	1	12	64	8	83	273.6	100	427.46	284	276	270	14	39	0	0	2
11992	8008	5276	1	1654	1196	28358	523	138	742	36	220	188	74	250	302.61	170	451.94	499	244	427	68	105	7	10	10
10566	8002	5702	1	2288	1762	28770	502	63	494	13	388	169	28	316	324.32	83	469.41	455	158	361	112	85	1	58	38
10664	7930	5624	3	2338	1792	28798	506	59	499	7	408	171	28	332	331.99	84	470.86	452	161	355	111	84	7	36	30
11618	8184	5352	3	1694	1322	28454	553	110	731	29	258	196	59	259	305.44	162	456.8	486	241	389	79	115	5	26	13
11046	7830	5484	0	2164	1718	28714	500	82	552	11	422	177	44	287	325.69	107	466.33	477	165	364	122	94	7	40	27
5850	12362	8876	556	646	364	28122	1035	14	766	1	24	72	9	108	280	109	437.73	361	370	383	29	53	1	0	3
4746	13076	9450	743	506	306	28112	1176	7	638	1	26	57	4	92	279.19	83	435.38	329	340	322	18	34	1	2	0
6452	11906	8540	437	770	446	28164	975	24	771	3	50	72	14	109	288.75	126	442.09	412	394	386	28	50	0	0	5
3934	13610	9892	945	396	234	28078	1270	2	545	0	12	58	1	66	270.16	89	422.92	294	260	288	19	41	0	0	2
4278	13376	9664	843	472	302	28112	1236	7	582	0	20	50	4	98	275.18	89	428.29	292	292	302	12	28	0	0	3
4574	13340	9600	851	336	194	28054	1235	11	646	1	10	66	5	67	274.11	105	429.58	309	324	300	14	36	0	0	4
13908	7868	4542	5	870	668	27918	627	293	1024	141	60	237	112	171	273.41	240	440.09	472	412	451	20	89	17	2	4
10828	7662	5586	0	2384	1826	28802	498	75	488	14	456	166	33	320	324.07	90	465.92	464	141	364	113	91	8	58	32
4680	13126	9492	781	492	290	28100	1195	7	656	1	18	61	4	81	274.73	104	429.48	328	328	308	12	45	0	2	7
4442	13240	9620	819	480	290	28108	1197	9	626	2	36	60	6	88	276.83	104	432.75	331	292	305	14	43	0	0	0
13844	7718	4458	3	1020	840	28008	606	305	912	140	124	241	118	181	275.09	240	441.68	497	354	425	31	77	22	4	3
4818	13130	9416	729	448	274	28098	1145	5	665	0	12	59	2	73	278.32	114	433.35	366	323	330	17	46	0	0	2
13844	7754	4560	6	1032	730	27978	662	287	992	139	52	244	119	160	275.3	245	437.94	493	416	406	26	87	9	6	0
14166	7738	4422	5	846	662	27908	663	328	1020	160	72	265	123	141	269.55	262	432.42	510	424	397	18	90	21	2	1
4980	12786	9280	670	690	402	28166	1153	4	651	0	28	68	4	102	278.69	91	435.73	359	339	298	15	44	0	0	1
5752	12486	8912	599	586	354	28116	1100	20	730	10	26	76	7	108	283.21	118	439.66	353	385	331	21	46	1	0	0
11940	7948	5210	6	1732	1316	28424	566	129	686	36	258	181	70	251	301.31	152	453.55	507	239	398	64	90	7	20	11
14378	7636	4404	5	810	578	27860	647	322	1078	157	52	259	125	142	269.4	268	432.43	480	453	440	24	89	18	2	1
11500	8176	5470	1	1756	1296	28468	581	92	712	19	240	169	43	266	310.66	162	458.82	487	242	367	77	84	11	28	11
3992	13624	9906	962	340	184	28054	1300	3	584	1	8	62	2	69	272.16	86	425.13	274	281	273	14	44	0	0	3
5370	12668	9112	646	604	350	28124	1120	14	682	1	20	78	8	100	282.26	101	437.23	363	358	309	17	50	0	0	2
13938	7784	4502	10	946	714	27952	636	286	989	125	66	229	121	166	274.95	229	439.15	477	403	456	24	87	13	2	3
5396	12660	9074	570	612	376	28148	1094	8	733	1	30	66	7	94	279.63	100	436.07	380	412	347	17	41	0	0	2
7322	11272	7990	344	940	602	28208	919	42	790	8	78	91	25	137	287.91	154	446.1	445	375	369	24	62	0	4	2
3872	13720	9966	983	302	176	28050	1313	7	571	1	12	60	5	71	272.08	76	424.36	268	281	269	12	38	0	2	2
12418	7872	4948	1	1586	1266	28340	548	156	780	45	228	201	74	237	297.89	167	452.26	491	284	404	71	111	8	18	14
14390	7652	4342	8	820	620	27880	636	330	1045	167	54	240	135	149	271.8	293	434.93	493	414	465	13	62	18	2	1
11328	8308	5576	0	1752	1256	28460	578	98	720	16	224	163	63	270	312.37	130	460.68	464	259	399	83	95	2	16	7
11512	7944	5326	1	1940	1502	28566	523	100	621	23	298	179	51	290	311.36	117	458.93	489	212	372	86	97	10	42	19
4930	12918	9344	690	572	340	28136	1136	4	669	0	30	58	3	85	278.97	104	435.64	371	342	332	15	40	0	2	1

6834	11670	8266	380	858	524	28192	918	26	803	9	40	100	14	138	287.54	135	442.43	434	389	379	22	60	0	0	2
4026	13624	9868	931	336	196	28056	1268	5	585	1	4	56	2	65	271.02	91	423.83	295	280	289	13	38	0	2	5
11692	8040	5304	0	1778	1372	28488	545	108	674	23	272	162	50	238	310.16	168	458.12	510	210	402	84	70	9	28	20
4114	13508	9820	922	400	218	28064	1264	8	583	1	4	59	5	77	270.34	87	423.47	286	277	290	13	40	0	0	4
11050	7832	5584	0	2164	1614	28672	511	78	567	12	388	169	46	293	324.82	91	467.58	447	174	389	114	87	7	40	30
3986	13628	9880	954	330	212	28062	1283	8	564	1	26	60	5	64	271.29	93	423.83	284	263	285	19	45	0	0	2
12402	7802	5004	1	1636	1258	28364	563	163	708	42	240	187	83	255	295.86	139	451.88	492	249	393	65	105	10	20	8
14094	7660	4480	5	930	682	27928	630	302	990	148	76	256	121	169	271.73	242	436.82	494	386	423	18	97	14	6	1
12980	7842	4824	1	1352	1016	28174	611	203	861	83	156	206	92	205	282.33	196	448.49	493	333	415	42	94	9	4	7
4198	13520	9798	903	338	188	28052	1267	9	605	1	10	73	5	60	273.8	107	427.51	319	306	261	11	50	0	0	2
6366	11966	8522	463	790	490	28178	1000	23	777	4	42	75	16	117	286.56	126	441.46	411	391	367	20	48	0	2	3
14214	7644	4320	8	910	754	27942	642	310	983	155	94	250	121	158	272.1	245	437.44	487	392	426	26	81	21	6	3
5558	12498	9004	585	660	388	28142	1083	14	724	4	32	75	7	87	282.47	97	439.24	398	390	318	26	50	0	2	0
13526	7616	4534	0	1334	1002	28140	609	240	823	80	118	204	126	194	281.8	189	445.37	470	304	457	47	93	17	10	9
4316	13306	9674	873	492	292	28104	1263	5	588	2	24	66	1	85	274.12	86	427.48	300	307	266	13	48	0	0	2
4316	13440	9708	886	362	222	28066	1228	9	604	2	18	57	4	79	273.93	98	429.37	301	287	308	15	37	0	0	3
3908	13698	9948	978	302	178	28052	1301	4	560	1	18	56	3	75	271.06	87	423.58	272	269	278	10	33	0	0	2
12782	7888	4904	5	1418	1068	28236	591	181	845	48	154	188	94	214	295	165	454.29	466	318	437	67	106	13	20	8
11516	7848	5448	0	1996	1416	28542	555	105	585	13	292	133	59	259	311.33	127	460.66	510	190	377	85	73	4	24	18
3834	13724	10004	1010	312	162	28044	1321	10	553	1	8	62	5	67	270.25	91	423.18	272	259	256	14	41	0	0	0
11808	8088	5336	1	1686	1246	28414	576	121	698	34	222	176	60	250	299.58	161	454.92	488	237	397	75	88	10	24	8
4216	13496	9806	909	346	180	28054	1256	4	605	1	8	65	1	72	276.53	105	431.48	294	288	290	13	48	0	2	4
4062	13492	9846	935	432	234	28078	1288	5	571	1	12	51	4	74	271.51	82	423.89	289	282	282	11	35	0	0	1
14272	7660	4392	5	870	644	27896	641	321	1041	168	56	259	115	169	269.21	250	430.86	484	424	418	15	85	13	2	1
6014	12094	8690	502	826	520	28204	1040	18	722	4	52	63	9	118	282.12	112	438.89	379	372	361	25	39	0	8	5
4438	13292	9626	816	444	266	28088	1181	6	610	1	22	56	3	88	277.4	89	429.86	316	294	335	18	39	0	0	1
13454	7630	4562	0	1324	1014	28124	590	233	868	98	124	234	100	224	281.59	202	447.41	483	328	409	35	105	16	16	5
13746	7850	4566	6	974	760	27986	656	255	1001	110	86	230	107	175	276.17	237	438.33	474	402	428	26	99	17	4	1
12186	7880	5086	4	1668	1288	28378	588	154	696	47	256	187	76	241	297.83	135	452.51	463	246	400	78	100	9	14	15
4116	13636	9860	953	268	148	28034	1310	9	613	2	6	56	4	78	272.11	86	425.84	259	308	281	10	35	0	0	0
4770	13140	9502	721	428	226	28078	1135	5	686	0	12	71	3	94	279.76	98	434.38	334	340	340	17	52	0	0	2
7220	11356	8044	389	914	588	28196	928	34	782	6	64	104	21	136	288.87	134	444.81	400	377	370	38	68	1	10	9
5254	12758	9190	654	572	322	28110	1114	9	693	3	14	67	3	90	279.93	104	436.8	362	363	337	18	40	0	0	3
13376	7850	4744	4	1128	840	28062	611	247	954	103	118	223	103	194	282.64	195	444.34	450	384	441	51	91	11	4	7
14326	7672	4398	5	814	606	27878	639	324	1040	161	60	253	128	148	271.25	278	435.68	495	422	433	17	84	16	2	3
11556	7890	5354	5	1950	1454	28524	547	102	645	23	286	161	59	265	308.68	118	456.92	463	230	413	96	87	6	32	15

SUPPLEMENTARY INFORMATION:Monte Carlo Atomistic Simulation and Machine Learning Analysis of Na-K Eutectic Alloy in Condensed Phases, D. Reitz and E. Blaisten-Barojas, George Mason University, Fairfax, VA 22030

13808	7746	4594	4	1010	732	27978	613	278	974	125	82	250	118	193	275.48	228	441.58	487	388	420	23	109	16	6	0
14280	7540	4390	3	940	680	27910	630	323	1008	153	80	250	129	165	273.2	222	436.05	464	412	444	30	88	15	0	3
4220	13414	9770	883	430	230	28074	1253	7	586	1	10	63	5	66	274.5	89	428.92	306	294	285	19	41	0	0	2
11002	7778	5526	1	2262	1722	28740	477	71	506	14	394	179	35	301	332.59	93	470.58	474	136	359	133	92	2	54	29
4804	13054	9442	702	500	284	28104	1164	5	669	0	20	64	2	70	279.42	97	436.92	356	365	317	21	42	1	0	2
4552	13212	9598	790	460	248	28086	1194	8	627	1	16	72	7	81	278.14	93	433.64	320	314	307	21	39	0	0	1
12522	8000	5010	2	1420	1098	28250	583	185	805	54	184	196	103	209	284.31	162	450.92	506	315	416	63	109	7	16	3
13020	7828	4786	4	1320	1036	28170	612	199	860	70	162	196	99	210	284.48	159	449.47	438	347	451	62	97	11	16	8
12772	7840	4890	2	1432	1096	28226	621	173	830	54	184	172	91	219	287.33	160	450.77	451	312	450	60	82	11	8	5
11864	8068	5336	2	1680	1208	28386	559	130	746	19	210	156	73	235	306.24	150	457.53	482	260	426	84	92	7	20	14
14316	7664	4402	1	844	612	27892	624	333	1048	155	50	270	134	156	270.48	274	434.77	504	417	422	16	99	20	4	2
10964	7782	5534	1	2292	1716	28706	515	72	494	10	374	157	36	279	315.75	95	464.97	508	156	372	107	73	8	36	22
4900	12958	9352	700	558	336	28130	1160	2	678	0	24	69	2	87	278.16	117	435.78	362	342	298	16	43	0	2	1
4222	13478	9760	897	376	220	28068	1261	8	600	0	10	56	5	81	273.28	87	426.39	279	286	298	14	38	0	2	4
11226	7994	5542	1	2032	1464	28580	547	78	625	16	298	167	45	253	311.57	120	462.49	498	217	376	93	88	5	22	23
5456	12682	9102	576	560	308	28120	1074	5	763	0	12	66	3	96	280.37	125	436.87	379	394	357	18	43	0	0	2
4888	13020	9414	702	492	268	28096	1133	3	685	0	14	60	2	83	278.28	101	433.85	345	342	351	21	37	0	0	1
4892	12988	9376	708	536	310	28120	1156	7	664	0	16	59	5	77	280.34	107	435.69	360	341	323	18	35	0	2	2
11778	7888	5236	0	1830	1426	28494	542	128	654	28	310	180	71	260	308.28	115	456.88	471	232	397	90	105	8	26	21
4816	13032	9430	709	530	294	28116	1149	5	670	1	14	61	4	87	276.9	102	432.09	355	341	331	14	36	0	0	1
4124	13482	9792	910	428	244	28076	1267	10	585	2	6	55	4	69	272.64	101	425.69	295	275	284	19	33	0	0	2
4040	13596	9870	931	348	196	28060	1270	7	578	1	10	56	3	62	271.85	88	425.79	298	277	292	18	38	0	0	0
4654	13142	9532	746	500	266	28102	1184	9	648	0	8	68	7	95	275.28	101	429.77	325	337	310	11	46	0	0	3
4720	13052	9428	756	572	344	28134	1165	3	642	0	18	66	2	83	278.24	113	434.71	361	318	304	14	44	0	0	2
13872	7790	4636	9	916	642	27928	610	281	1048	132	62	253	113	182	277.26	241	440.4	461	398	451	28	96	17	10	3
13788	7822	4602	7	964	714	27972	636	297	979	120	76	217	134	179	275.96	227	440.64	460	399	456	30	80	19	6	3
13550	7852	4646	4	1072	812	28034	633	233	986	103	100	211	96	156	278.01	242	443.08	500	371	448	39	89	12	2	2
14174	7600	4440	4	980	680	27930	622	314	970	151	54	254	121	167	274.2	231	439.37	480	387	437	29	80	13	2	1
4252	13384	9694	862	468	288	28104	1237	7	578	0	18	58	7	88	276.27	87	430.2	304	286	293	12	42	0	0	3
14296	7662	4414	4	828	616	27888	655	312	1053	156	72	251	124	146	274.02	276	437.79	489	429	433	20	84	18	0	0
13236	7676	4570	3	1392	1128	28186	593	200	826	78	184	209	89	208	283.42	195	446.61	470	298	444	59	91	18	0	4
4246	13402	9744	858	432	246	28090	1246	5	604	0	20	55	5	78	274.6	83	427.86	314	311	292	10	39	0	0	1
13836	7744	4596	4	984	714	27966	630	284	987	134	90	233	108	164	274.98	238	441.46	473	386	451	37	77	23	2	1
5166	12784	9228	666	588	334	28118	1099	9	678	2	18	80	6	95	280.36	96	437.76	365	336	333	20	53	0	0	3
4514	13290	9606	800	410	242	28082	1188	6	623	1	18	70	3	86	276.75	95	431.15	318	316	312	18	47	0	2	2
3926	13670	9926	983	316	194	28054	1318	8	585	1	22	58	5	53	270.41	96	422.48	285	275	263	15	43	0	0	3

SUPPLEMENTARY INFORMATION:Monte Carlo Atomistic Simulation and Machine Learning Analysis of Na-K Eutectic Alloy in Condensed Phases, D. Reitz and E. Blaisten-Barojas, George Mason University, Fairfax, VA 22030

5202	12740	9160	654	630	390	28152	1123	6	667	0	28	65	5	94	281.63	100	436.96	378	345	314	16	44	0	2	2
4352	13416	9728	875	358	190	28054	1251	8	633	2	10	58	6	63	271.87	101	426.34	302	305	299	18	42	0	0	2
4872	13020	9392	727	512	296	28108	1160	6	675	1	16	62	3	72	276.92	102	432.1	349	331	326	23	43	0	0	3
4232	13396	9742	878	448	252	28082	1244	7	585	2	12	53	3	68	271.57	81	425.84	306	295	297	21	35	0	0	2
4906	13060	9372	686	470	284	28108	1153	4	702	1	16	67	1	93	278.78	99	434.98	350	374	319	12	46	0	0	1
4108	13548	9826	931	368	208	28066	1275	8	588	1	8	61	6	71	271.82	92	424.91	292	265	282	12	42	0	0	3
3960	13652	9908	954	342	190	28054	1303	7	573	1	2	63	3	79	271.35	73	423.42	264	281	273	13	46	0	0	1
11566	7854	5324	1	1948	1500	28552	489	93	630	25	316	199	47	288	313.39	118	461.59	470	208	395	105	102	4	42	17
12566	8020	5022	4	1394	1048	28220	638	166	854	54	150	171	89	204	285.95	187	446.76	467	332	450	49	94	10	20	8
3986	13636	9910	982	322	180	28046	1321	10	569	1	12	64	7	69	272.08	87	426.6	261	275	263	15	41	0	0	2
5746	12426	8888	587	646	394	28128	1078	23	683	10	26	77	9	92	279.69	98	440.11	379	364	339	26	50	0	2	1
4696	13200	9508	781	414	242	28072	1204	13	652	0	12	48	5	76	274	95	429.76	316	348	324	19	35	0	0	1
5552	12510	9012	569	664	380	28144	1070	7	712	2	26	75	4	105	281.49	104	438.56	385	382	342	16	48	0	0	2
11540	7902	5254	1	1964	1550	28546	527	118	635	33	282	164	63	255	300.6	132	456.43	512	199	408	95	86	8	48	10
6800	11712	8276	389	808	532	28196	978	17	819	6	64	82	8	125	287.48	119	443.86	406	416	371	27	57	0	4	4
4194	13512	9826	875	338	168	28044	1224	5	605	0	6	67	1	74	275.47	114	430.04	310	295	299	15	46	0	0	1
13124	7644	4690	3	1462	1118	28230	610	206	775	72	180	207	94	221	292.3	165	450.23	483	316	393	54	104	10	12	7
14366	7614	4334	2	844	660	27900	633	329	1032	169	76	255	117	147	270.22	276	432.15	504	423	433	17	73	21	6	4
13084	7810	4822	4	1352	962	28156	629	209	855	64	116	195	120	207	284.6	173	447.15	464	336	442	45	98	13	10	3
4106	13538	9828	903	372	214	28074	1270	4	594	1	16	58	2	71	272.53	88	426.47	309	297	282	5	40	0	0	3
13212	7746	4644	1	1350	1060	28174	584	230	811	89	156	211	106	203	281.37	187	445.6	498	298	439	52	90	19	6	2
5128	12760	9216	657	638	380	28150	1140	5	700	0	24	59	3	95	280.64	125	436.64	358	360	320	16	33	1	2	4
4138	13498	9808	925	388	222	28070	1272	7	584	0	16	57	4	74	272.67	78	426.41	280	283	290	19	43	0	0	2
13136	7714	4694	1	1414	1076	28190	585	208	804	80	132	212	92	205	282.9	160	448.25	494	298	437	50	105	15	22	7
11402	7722	5378	1	2134	1598	28618	512	97	557	24	338	199	43	302	314.73	96	462.35	463	174	344	107	112	5	42	26
13726	7636	4546	7	1126	866	28038	608	288	920	121	126	228	124	179	276.06	217	442.22	495	345	440	34	92	22	10	4
5284	12708	9134	623	610	374	28142	1090	6	699	2	32	68	3	99	278.94	104	436.23	367	360	347	21	47	0	0	3
4328	13356	9682	826	454	264	28098	1220	4	588	0	12	54	2	85	275.72	83	430.47	313	320	305	15	32	0	2	1
4144	13526	9812	921	374	204	28062	1289	8	609	2	0	58	3	76	270.33	91	422.55	277	308	278	12	42	0	2	3
4248	13346	9728	864	486	274	28102	1257	6	584	1	20	62	2	78	275.91	94	429.4	312	295	272	11	39	0	0	3
4490	13222	9588	799	500	290	28108	1219	11	592	1	18	60	5	80	275.83	94	430.38	333	302	288	14	40	0	0	0
3932	13722	9950	956	280	152	28040	1277	4	584	1	4	61	3	64	271.76	92	425.04	292	274	284	13	41	0	0	2
11678	7830	5216	0	1974	1514	28534	550	94	635	20	288	162	53	276	306.46	114	456.35	475	227	401	82	94	10	34	17
4310	13414	9712	878	390	232	28078	1268	5	624	0	20	55	2	75	276.94	90	431.41	304	316	277	10	36	0	0	2
5064	12868	9270	674	566	338	28132	1137	8	679	0	26	74	6	84	277.67	109	433.92	370	351	310	20	48	0	0	1
5304	12756	9180	643	534	314	28122	1149	6	705	1	30	62	4	88	280.25	85	437.15	351	396	326	16	49	0	4	2

SUPPLEMENTARY INFORMATION:Monte Carlo Atomistic Simulation and Machine Learning Analysis of Na-K Eutectic Alloy in Condensed Phases, D. Reitz and E. Blaisten-Barojas, George Mason University, Fairfax, VA 22030

7704	10838	7656	287	1162	810	28306	875	43	755	12	124	89	20	151	288.5	131	446.97	455	372	373	37	52	3	12	7
14188	7652	4368	5	952	714	27928	629	310	1001	162	48	246	114	164	269.31	252	431.31	495	407	434	20	71	12	6	1
13382	7558	4632	1	1354	1030	28142	584	230	842	85	176	199	99	215	281.89	194	445.78	476	308	447	44	83	14	10	7
4912	12952	9328	697	560	352	28136	1146	7	651	0	32	62	4	74	278.4	98	434.78	373	345	316	19	37	0	0	4
10800	7906	5620	0	2220	1722	28732	506	75	508	8	410	153	40	263	322.18	92	466.42	487	163	384	123	80	3	50	31
6298	11972	8518	451	816	536	28202	965	19	721	9	60	66	7	124	287.32	120	442.94	411	356	392	29	33	0	2	3
13994	7830	4512	5	884	654	27920	640	297	1020	151	42	241	104	135	274.71	258	438.89	497	412	437	37	73	15	4	0
3990	13594	9884	928	370	216	28072	1267	7	567	1	18	69	4	67	272.52	94	426.01	308	285	265	14	38	0	0	3
13796	7764	4574	1	1020	750	27990	584	272	952	125	84	244	109	174	275.53	238	440.15	502	370	446	35	89	15	2	2
11390	7714	5382	0	2136	1608	28630	526	90	570	21	364	180	48	279	311.45	107	462.6	505	194	357	97	96	8	30	18
11502	8052	5234	3	1898	1526	28526	565	101	609	25	292	159	57	244	300.14	129	454.43	494	209	404	88	84	5	22	18
4442	13372	9674	876	362	196	28054	1262	9	656	2	8	68	6	72	274.91	114	429.18	295	317	279	11	39	0	0	4
4050	13458	9828	922	468	268	28092	1286	7	549	1	18	55	2	72	271.37	79	424.8	291	281	267	19	32	0	2	0
11900	7756	5262	1	1930	1360	28484	526	125	628	35	254	180	58	277	307.56	121	457.15	500	209	380	82	98	8	22	12
11326	7938	5426	0	1976	1542	28598	528	102	627	16	342	164	45	265	321.56	124	460.52	489	207	374	99	102	8	48	26
14462	7684	4368	4	722	550	27848	649	327	1119	159	60	257	131	145	267.72	273	430.4	486	470	438	13	86	14	2	3
13274	7736	4644	1	1296	1032	28158	605	230	830	76	166	207	115	209	282.39	184	446.67	479	303	427	50	100	17	10	3
4362	13404	9714	866	354	204	28062	1231	8	619	0	24	57	4	80	275.23	86	428.95	299	308	306	15	42	0	0	2
4104	13532	9842	915	374	202	28064	1272	4	593	0	10	55	1	57	271.35	93	424.45	307	285	283	18	33	0	0	1
7112	11238	8002	360	1088	730	28272	922	30	712	10	92	97	11	140	288.68	121	447.04	445	363	344	31	44	2	6	8
14244	7700	4504	4	804	560	27874	665	297	1092	142	58	261	121	160	274.07	246	436.45	452	450	432	22	106	17	4	2
4164	13550	9814	923	326	186	28052	1273	6	612	1	12	65	4	57	271.1	87	423.48	300	298	280	17	51	0	0	3
12568	7816	4976	2	1578	1150	28278	595	165	784	45	178	184	86	232	286.93	152	449.85	505	280	411	42	106	12	12	7
7304	11278	7910	352	994	680	28250	908	28	752	10	82	94	15	135	286.27	139	445.22	439	367	376	38	52	0	2	3
11650	8024	5248	1	1838	1432	28480	573	91	701	18	260	171	47	236	302.92	149	458.99	487	242	396	94	99	6	28	16
13710	7814	4636	5	988	734	27972	616	270	1017	132	76	243	109	182	273.63	231	441.11	464	392	451	32	100	15	12	3
4392	13372	9656	853	404	240	28074	1241	11	610	1	10	54	4	77	274.18	96	429.5	300	311	301	16	36	0	0	2
4660	13190	9516	787	444	260	28084	1205	5	659	1	14	64	3	79	275.12	100	430.37	317	343	312	15	41	0	0	4
12074	7788	5160	2	1800	1328	28424	526	144	684	31	244	169	70	242	300.48	155	455.6	511	213	427	74	91	14	30	16
4172	13506	9826	901	354	186	28058	1243	7	590	0	14	67	3	71	273.91	107	428.29	314	279	282	12	47	0	0	1
4124	13508	9824	907	388	212	28066	1283	7	592	2	10	63	5	82	272.14	67	424.3	276	301	280	11	43	0	0	2
10764	7814	5570	0	2308	1838	28814	500	69	482	8	456	149	34	308	324.81	76	468.99	482	152	360	115	82	2	58	30
14242	7594	4356	2	940	714	27922	647	313	1006	152	72	266	124	163	271.95	245	435.5	472	399	419	28	101	21	4	1
13940	7770	4488	1	924	742	27968	642	274	1028	120	94	223	118	175	275.64	240	440.49	451	416	469	30	86	18	10	2
4562	13232	9574	805	436	258	28086	1221	5	640	1	24	59	3	79	273.23	93	427.45	323	333	299	12	38	0	0	1
13848	7530	4366	0	1220	984	28098	565	257	884	101	138	224	118	197	278.42	221	445.28	520	303	447	32	104	14	12	4

SUPPLEMENTARY INFORMATION:Monte Carlo Atomistic Simulation and Machine Learning Analysis of Na-K Eutectic Alloy in Condensed Phases, D. Reitz and E. Blaisten-Barojas, George Mason University, Fairfax, VA 22030

11960	7906	5154	1	1790	1362	28434	556	136	672	30	224	174	74	257	296.89	126	451.68	474	252	417	77	81	7	36	17
4402	13346	9682	864	404	222	28070	1244	5	617	1	14	67	3	77	273.15	92	427.63	292	305	297	16	44	0	0	4
7158	11290	7972	405	1014	714	28258	931	53	684	17	100	89	27	152	288.63	113	445.6	412	337	364	33	41	3	10	6
13130	7844	4814	5	1302	930	28142	601	212	861	69	120	197	113	205	281.92	181	448.82	466	319	465	48	97	14	2	5
14308	7708	4436	7	778	576	27870	629	329	1067	161	62	263	129	158	271.85	251	435.83	487	447	436	16	93	15	2	2
11054	7944	5620	2	2166	1518	28626	528	79	568	9	290	167	43	299	319.97	92	463.16	447	183	389	103	77	4	34	26
5034	12740	9252	648	718	404	28164	1139	6	665	0	16	52	3	96	278.31	93	434.98	355	357	325	23	37	0	0	1
11366	8032	5492	0	1946	1402	28528	561	111	626	23	262	161	65	288	308.14	127	456.64	479	216	378	76	92	2	26	13
12586	8048	4934	3	1414	1096	28228	620	169	840	58	124	176	78	220	281.92	197	447.12	478	310	435	41	87	16	24	8
14148	7694	4466	6	880	654	27920	628	303	1027	145	72	244	114	144	274.72	257	436.78	517	405	438	19	90	14	6	2
14288	7690	4372	5	834	644	27892	659	326	1031	165	62	250	122	149	270.98	257	433.3	480	436	434	22	69	19	2	0
4428	13338	9664	842	400	228	28078	1233	8	636	1	20	63	6	74	273.05	93	426.09	309	318	299	13	41	0	0	5
4700	13084	9456	758	542	324	28126	1180	4	641	0	18	57	3	84	277.97	112	433.17	342	318	312	16	39	0	2	3
4034	13594	9868	953	358	200	28060	1309	6	580	1	6	61	3	66	270.51	71	422.84	279	304	267	13	39	0	0	2
4970	12990	9348	727	476	288	28098	1172	9	662	0	24	72	7	81	278.96	107	435.7	340	339	305	18	46	0	2	4
5178	12906	9268	668	458	260	28088	1118	11	721	0	18	53	5	87	277.66	109	433.25	348	354	362	17	33	0	0	3
3972	13672	9906	978	320	180	28052	1300	6	571	1	2	54	3	66	270.97	82	423.59	277	269	281	14	38	0	0	2
14470	7576	4326	7	824	614	27878	623	337	1038	162	64	259	132	158	270.31	263	433.22	490	408	442	17	88	21	4	2
14344	7580	4360	5	858	664	27904	614	337	1014	153	96	252	134	154	271.93	255	435.27	503	404	448	22	86	27	2	0
12382	7864	5076	4	1628	1168	28316	566	171	741	49	184	166	86	247	296.26	153	450.69	476	275	448	59	80	11	14	9
4734	13016	9442	748	582	336	28132	1184	4	639	0	22	57	1	81	278.61	94	434.6	349	332	303	17	37	0	0	2
4844	13034	9392	711	530	310	28122	1165	5	665	0	10	61	4	88	279.28	105	435.45	344	350	323	15	40	0	0	2
14438	7626	4330	3	798	616	27882	654	322	1073	156	74	264	127	155	270.52	267	434.39	478	449	421	20	94	21	0	1
4060	13594	9858	923	342	194	28056	1281	8	584	1	8	62	7	75	271.95	82	426.21	281	298	281	14	48	0	0	1
4464	13284	9636	820	442	246	28088	1229	3	647	0	16	69	2	78	276.18	89	431.61	301	345	293	22	45	0	0	3
14132	7744	4448	6	856	662	27920	632	304	1033	149	76	247	117	175	273.62	250	437.7	464	412	456	20	80	20	2	2
3974	13676	9906	965	308	180	28052	1316	5	591	1	6	61	1	81	271.59	90	424.42	259	291	261	11	35	0	2	2
10918	7858	5506	0	2224	1766	28752	512	62	524	6	440	143	39	293	320.33	96	466.36	484	183	365	128	75	0	36	18
4614	13238	9576	805	396	226	28072	1206	10	650	2	18	75	5	75	275.49	99	429.9	319	337	297	20	50	0	4	2
12254	7816	5104	0	1662	1260	28372	555	144	697	39	248	190	70	240	299.02	143	453.92	474	244	411	77	98	8	28	17
4208	13398	9742	874	448	272	28096	1226	7	570	1	24	57	4	75	274.3	85	428.28	318	272	302	16	38	0	4	2
4676	13130	9502	787	492	284	28104	1174	7	629	1	20	49	4	85	275.48	100	430.44	330	303	338	15	37	0	0	2
14350	7598	4366	5	884	634	27882	672	314	1040	168	50	260	107	161	269.2	254	431.88	464	434	419	18	75	20	0	0
13248	7842	4680	5	1200	982	28120	611	214	922	81	152	209	102	217	283.42	191	445.47	457	357	441	44	103	15	14	3
5784	12316	8826	542	732	470	28182	1040	15	713	2	48	77	10	118	284.02	105	440.64	397	365	339	18	50	0	6	3
4108	13620	9856	933	290	162	28044	1270	9	611	1	8	50	5	68	271.78	91	426.83	279	290	310	18	31	0	0	0

SUPPLEMENTARY INFORMATION:Monte Carlo Atomistic Simulation and Machine Learning Analysis of Na-K Eutectic Alloy in Condensed Phases, D. Reitz and E. Blaisten-Barojas, George Mason University, Fairfax, VA 22030

12790	7772	4994	1	1492	1022	28236	589	179	801	48	148	194	93	229	293.1	168	453.07	470	287	428	52	110	12	16	11
14318	7654	4424	7	822	592	27870	629	353	1026	167	60	263	142	169	270.34	248	433.16	462	424	445	23	82	20	0	0
11824	7914	5228	1	1830	1386	28474	581	114	682	29	270	172	56	254	304.02	140	454.62	473	256	393	92	94	7	20	9
12322	7936	5082	3	1604	1180	28322	569	138	741	41	176	199	74	258	293.37	141	452.17	479	263	397	59	114	8	18	8
3982	13604	9884	950	366	216	28068	1297	4	569	1	16	65	3	77	272.02	95	425.44	276	281	265	12	40	0	0	3
14402	7602	4386	3	816	596	27874	633	340	1031	152	70	262	142	151	272	264	434.07	487	418	436	22	94	23	2	1
12036	7864	5116	1	1796	1364	28438	547	135	660	39	226	187	64	252	300.44	140	455.08	508	219	375	82	100	8	32	9
14476	7540	4348	3	856	596	27866	643	327	1046	157	46	266	123	160	271.83	262	437.78	475	445	425	19	89	24	4	1
4244	13394	9738	883	442	252	28086	1254	14	578	2	14	50	9	72	275.37	92	431.74	300	289	297	19	36	0	2	1
11432	7912	5328	0	2002	1552	28576	534	115	602	22	314	173	68	284	308.24	117	458.67	454	204	399	101	87	7	32	20
4660	13076	9500	737	568	310	28124	1164	8	613	0	8	58	3	98	278.07	78	435.27	333	320	324	15	38	0	2	2
14224	7586	4362	3	932	738	27954	618	310	997	152	106	244	117	165	273.39	247	437.37	498	407	444	24	75	24	6	1
5020	12894	9304	685	546	326	28120	1142	7	658	0	30	65	4	104	277.91	95	434.71	332	341	328	16	45	0	0	3
12504	8078	5056	4	1386	1036	28226	585	161	855	46	160	187	85	223	291.44	187	448.93	470	300	438	65	95	9	6	4
12174	7828	5108	1	1712	1290	28374	564	144	724	44	240	190	63	257	299.58	141	453.41	471	252	403	67	96	11	20	16
4274	13434	9726	876	390	236	28082	1240	6	615	1	22	64	3	72	273.14	103	427.52	314	286	292	14	43	0	0	2
4596	13152	9566	760	508	268	28102	1183	3	648	0	12	63	3	72	277.94	95	432.98	346	345	312	18	42	0	0	2
13428	7740	4680	4	1206	894	28078	594	247	890	100	126	222	115	198	279.05	204	445.59	478	329	456	41	101	14	4	2
4472	13162	9600	803	564	304	28112	1224	5	611	0	10	69	2	94	275.71	73	430.35	317	325	273	11	49	0	0	3
12314	8160	5086	5	1428	1100	28258	638	129	862	44	152	174	65	197	287.16	190	449.33	501	328	414	46	95	8	18	11
13808	7564	4484	0	1156	894	28050	560	269	889	115	132	219	109	214	277.88	205	445.32	479	317	465	35	82	20	10	4
11570	7946	5416	1	1890	1380	28512	535	105	649	21	280	191	53	266	316.53	123	459.27	491	220	365	86	100	4	28	20
11840	7900	5200	2	1848	1400	28462	560	106	672	23	232	176	56	254	305.42	135	457.09	489	245	398	78	91	9	40	15
12618	7842	4942	3	1516	1150	28272	597	179	791	53	176	179	89	224	286.31	168	449.88	472	282	427	67	89	9	28	6
4456	13262	9626	819	468	266	28096	1223	6	631	0	16	54	5	86	276.06	81	432.34	301	331	309	18	33	0	2	2
14098	7742	4428	6	886	694	27922	631	297	1022	157	72	249	109	157	270.02	266	432.17	506	411	434	16	77	19	2	1
5048	12980	9348	697	440	244	28078	1144	13	718	0	18	64	8	75	278.84	102	434.88	350	370	337	21	49	0	0	1
4776	13092	9472	766	478	262	28092	1200	6	678	0	10	52	1	75	274.99	106	430.2	334	346	313	16	39	0	0	1
11696	7850	5262	0	1914	1464	28518	549	105	692	27	300	168	50	269	309.02	125	458.41	479	232	392	90	93	4	30	16
4014	13652	9902	950	296	166	28040	1286	8	591	2	10	68	5	75	271.71	88	425.69	268	284	282	16	43	0	0	1
4054	13608	9872	950	332	182	28054	1276	11	566	0	6	60	10	56	272.39	94	426	304	270	280	16	42	0	0	2
4098	13512	9810	915	390	242	28078	1291	6	597	1	24	63	2	75	271.97	94	427.05	276	298	265	21	47	0	2	1
14624	7612	4332	4	704	502	27818	662	338	1111	167	42	274	135	156	267.83	269	429.82	462	472	426	13	97	15	2	0
11622	7814	5256	0	2022	1528	28570	531	111	587	14	294	162	53	282	312.23	123	459.14	479	180	382	91	91	8	30	23
4470	13280	9630	825	444	246	28082	1204	9	607	0	12	71	4	88	275.15	105	430.78	317	302	291	16	44	0	0	2
4992	12852	9296	655	614	362	28144	1115	3	649	0	28	71	2	92	279.91	92	437.35	362	342	331	24	52	0	0	2

SUPPLEMENTARY INFORMATION:Monte Carlo Atomistic Simulation and Machine Learning Analysis of Na-K Eutectic Alloy in Condensed Phases, D. Reitz and E. Blaisten-Barojas, George Mason University, Fairfax, VA 22030

4796	13090	9422	756	478	292	28096	1180	12	648	2	16	63	6	85	275.55	94	431.92	326	328	321	18	40	0	2	2
12960	8020	4918	8	1202	890	28114	660	186	965	71	108	208	87	202	281.23	216	444.3	477	381	411	31	112	14	16	0
14284	7634	4342	6	896	690	27916	666	304	1035	163	68	261	108	155	270.37	258	432.46	491	435	402	19	87	14	2	0
5712	12438	8908	567	650	398	28142	1067	14	707	1	36	68	8	101	284.07	112	441.36	377	358	356	20	45	0	0	4
4188	13534	9812	916	332	178	28050	1272	5	623	1	4	61	4	68	271.53	92	424.96	290	304	294	13	50	0	2	1
5732	12376	8876	593	694	434	28156	1085	23	709	3	40	67	14	104	282.42	117	438.34	383	355	328	20	42	0	4	1
5650	12648	9006	620	496	284	28092	1099	18	758	2	6	85	12	92	280	127	438.2	378	391	326	12	57	1	2	2
4760	13066	9436	738	510	314	28118	1156	8	632	0	32	52	4	85	279.78	102	435.9	355	324	325	16	34	0	0	1
4200	13450	9786	897	406	218	28070	1260	7	584	1	10	51	5	56	272.11	89	425.22	308	286	299	18	41	0	0	3
13638	7606	4598	1	1214	886	28072	621	265	859	102	114	233	126	201	276.97	186	445.34	466	319	424	42	105	17	14	1
11554	8030	5332	3	1834	1432	28504	550	95	663	24	292	167	49	273	305.94	141	457.75	478	222	397	82	87	7	24	16
14244	7712	4440	6	810	610	27888	646	317	1046	169	72	262	109	154	271.65	259	435.4	485	432	428	18	84	19	0	2
14384	7476	4296	11	992	714	27922	599	319	955	158	54	261	117	164	272.37	258	436.14	510	354	433	23	87	24	6	0
11312	7926	5376	1	2016	1580	28588	563	110	594	17	348	161	64	269	313.47	102	458.96	472	229	373	105	81	6	28	17
13830	7782	4536	7	976	758	27978	607	282	975	138	88	250	111	170	274.62	247	438.68	496	366	442	28	95	12	8	3
4566	13168	9548	769	506	296	28106	1178	12	610	1	22	62	8	79	276.51	96	432.36	343	315	312	14	39	0	0	5
11598	7888	5376	2	1906	1422	28532	547	115	631	25	316	179	65	290	311.14	108	458.54	447	216	394	89	95	3	26	21
14482	7626	4298	6	806	614	27882	630	324	1071	164	54	253	123	151	268.83	289	431.72	505	428	443	13	85	18	2	0
13880	7592	4480	2	1134	842	28042	631	269	913	110	110	214	119	174	279.03	209	443.27	477	355	444	44	92	23	4	1
14600	7524	4252	1	804	612	27860	656	341	1043	161	68	276	136	163	271.62	241	436.23	457	437	418	23	98	24	0	0
5654	12502	8958	598	620	368	28128	1096	11	743	1	24	76	6	103	283.86	117	439.35	369	383	324	21	50	1	2	0
13900	7732	4514	4	996	746	27970	621	273	997	120	76	235	118	161	275.73	250	437.74	499	393	441	28	98	16	6	3
13520	7848	4674	5	1098	800	28024	638	252	949	119	82	233	105	171	275.19	228	441.07	499	391	419	28	99	14	2	3
4086	13544	9830	941	380	218	28068	1294	11	570	1	10	70	10	72	270.52	85	423.87	280	283	265	13	54	0	0	4
13320	7912	4780	11	1130	810	28040	663	235	941	95	84	221	94	175	276.9	222	443.82	487	361	405	33	98	21	4	0
13806	7788	4576	1	962	744	27988	624	275	1028	129	108	230	115	175	275.4	227	441.62	482	422	451	29	89	18	4	1
4314	13398	9716	869	400	230	28076	1252	8	604	1	18	63	6	74	274.66	91	427.9	305	315	288	14	43	0	0	1
5092	12788	9264	672	600	354	28138	1110	12	668	0	38	58	6	97	281.22	112	437.52	361	329	340	20	41	0	2	3
4472	13300	9602	822	434	266	28094	1219	5	634	1	20	65	4	79	273.53	103	426.2	322	311	296	15	46	0	0	1
14162	7698	4402	3	906	692	27924	642	300	1008	139	56	244	125	163	274.82	223	438.36	463	414	453	29	83	15	8	1
4458	13308	9652	820	422	226	28074	1205	13	630	2	6	55	8	73	274.26	107	427.62	330	303	317	13	41	0	2	1
11440	7814	5308	1	2062	1606	28610	525	98	586	17	330	169	53	295	313.87	124	463.13	481	185	379	91	85	9	48	24
14218	7722	4450	5	824	610	27884	618	310	1075	166	56	268	111	163	270.79	270	433.49	494	418	437	15	86	19	4	1
11334	7912	5426	2	2008	1542	28606	526	96	610	13	356	179	52	258	313.17	130	460.16	526	182	361	93	100	6	24	20
11744	8018	5296	2	1792	1334	28448	572	106	716	14	246	170	63	255	304.41	128	455.12	486	262	405	74	104	11	18	13
10690	7828	5724	0	2340	1744	28802	519	74	494	11	402	142	36	302	333.27	87	472.75	460	164	368	125	70	3	70	31

SUPPLEMENTARY INFORMATION:Monte Carlo Atomistic Simulation and Machine Learning Analysis of Na-K Eutectic Alloy in Condensed Phases, D. Reitz and E. Blaisten-Barojas, George Mason University, Fairfax, VA 22030

4850	12970	9412	740	554	304	28106	1174	14	663	1	16	62	8	84	278.81	108	434.12	339	324	314	15	45	1	0	4
4380	13348	9680	861	424	236	28080	1231	8	604	1	12	63	4	81	273.69	90	429.15	308	292	297	12	48	0	0	3
14196	7606	4398	5	950	704	27930	614	294	968	146	74	251	114	153	272.23	238	436.08	509	370	439	24	92	16	2	2
13018	7760	4714	4	1406	1126	28224	582	201	802	66	184	199	101	228	292.93	178	448.83	470	288	441	56	98	10	14	6
11650	7910	5256	1	1928	1468	28516	560	121	640	21	276	142	52	254	310.11	125	461.19	490	230	398	90	75	13	26	14
4862	12964	9378	744	564	336	28134	1166	8	670	1	28	52	4	94	277.46	108	432.79	345	325	327	11	38	0	2	2
5148	12838	9216	668	552	344	28128	1132	11	685	2	30	72	6	89	279.25	111	434.41	362	355	320	19	42	0	0	2
4460	13346	9644	845	402	218	28070	1233	9	633	1	0	54	7	59	272.78	109	427.52	325	319	306	16	37	0	0	2
4124	13496	9826	919	402	214	28070	1261	8	583	1	8	54	3	80	271.88	85	425.25	280	275	300	19	38	0	0	0
5336	12646	9142	638	610	356	28128	1124	17	694	5	36	64	8	83	280.99	108	438.2	376	365	323	18	41	0	2	3
7594	11084	7858	296	1016	612	28226	888	37	807	7	56	109	22	129	290.12	146	445.31	458	395	373	28	67	2	6	9
4176	13526	9790	890	356	204	28058	1227	6	607	1	6	53	3	73	273.01	97	426.13	300	278	319	19	37	0	0	1
14504	7638	4404	6	738	502	27826	663	336	1102	165	40	272	125	139	267.65	278	429.05	483	464	420	13	98	24	0	3
6832	11642	8288	385	868	520	28198	958	32	784	8	44	82	18	121	288.49	124	442.38	441	410	361	24	48	1	4	1
6764	11798	8382	409	736	432	28156	939	31	789	9	44	78	16	116	286.88	124	442.37	437	397	389	24	43	0	0	2
14116	7684	4414	10	914	720	27940	657	326	998	157	86	249	128	179	272	240	436.9	457	408	429	22	81	28	6	1
4914	12906	9320	709	608	370	28146	1150	10	636	0	28	58	7	100	277.76	89	434.28	352	334	316	14	37	0	0	2
3934	13700	9936	966	302	168	28044	1320	6	592	1	4	64	5	77	271.3	82	423.05	249	295	268	16	47	0	0	1
6410	11822	8444	455	906	578	28220	970	17	709	4	54	74	6	128	285.68	111	441.96	417	347	363	29	47	1	6	4
13570	7806	4654	5	1100	800	28014	590	259	967	125	74	236	99	184	277.62	229	444.72	493	352	449	33	95	14	10	3
4004	13650	9888	935	320	186	28056	1280	4	583	1	6	62	3	57	272.5	104	425.16	310	277	272	13	45	0	2	1
5018	12792	9306	674	644	356	28142	1147	9	640	0	24	49	5	83	283.14	81	436.86	371	352	319	17	31	0	2	2
4476	13262	9632	802	456	248	28088	1187	6	612	0	14	55	2	79	276.14	94	430.89	326	314	323	18	35	0	0	3
4786	13168	9476	759	404	230	28076	1196	9	690	2	12	53	4	72	276.39	100	431.5	334	359	322	10	35	0	0	4
14560	7650	4300	2	726	556	27842	652	335	1092	158	48	255	137	147	267.59	289	429.24	483	449	440	14	93	17	2	0
14446	7518	4316	7	882	654	27894	637	340	1015	158	76	251	140	152	270.53	255	434.83	484	409	442	23	83	20	2	2
4650	13240	9562	773	392	210	28060	1196	11	669	1	6	58	4	86	274.61	99	431.36	305	343	321	18	41	1	0	2
8376	10674	7414	220	1024	656	28216	780	67	845	21	68	117	31	150	289.91	149	445.74	486	379	415	31	70	5	4	6
14388	7436	4284	4	1004	740	27934	634	331	953	168	74	265	117	179	273.31	227	436.54	463	391	416	28	83	23	8	3
4518	13290	9590	800	430	252	28092	1181	1	649	0	12	56	1	77	273.9	115	428.88	341	304	326	13	38	0	0	2
4712	13196	9486	778	414	258	28088	1201	9	663	2	22	60	4	77	275.6	108	431.31	331	345	309	14	40	0	0	2
12292	7778	5052	1	1708	1284	28368	539	165	722	37	224	195	89	246	305.25	152	457.17	472	243	403	87	100	7	28	11
14260	7664	4426	8	858	628	27904	632	303	1062	153	66	250	123	166	273.23	266	436.34	489	426	438	16	80	6	2	0
5778	12204	8786	514	838	544	28218	1062	8	725	2	60	68	3	107	283.97	114	440.72	422	387	310	18	43	0	8	2
11184	8014	5462	2	2004	1574	28624	569	75	617	15	336	174	39	293	315.66	94	460.91	452	211	353	92	98	4	46	26
12946	8026	4886	6	1186	930	28132	646	206	941	82	152	202	89	212	282.13	190	444.94	454	374	438	33	99	17	6	6

SUPPLEMENTARY INFORMATION:Monte Carlo Atomistic Simulation and Machine Learning Analysis of Na-K Eutectic Alloy in Condensed Phases, D. Reitz and E. Blaisten-Barojas, George Mason University, Fairfax, VA 22030

6970	11484	8118	316	968	636	28246	923	26	785	6	64	90	9	141	286.42	126	443.83	458	412	346	23	51	0	6	3
5758	12376	8898	581	660	398	28134	1083	26	709	8	44	84	16	98	281.64	113	438.07	384	380	327	19	52	0	0	1
8460	10574	7336	245	1046	710	28230	853	60	843	16	96	104	32	148	288.67	162	444.98	439	399	403	38	53	4	8	3
4582	13202	9562	787	466	264	28092	1193	8	635	2	16	54	3	78	276.48	100	432.95	335	326	316	14	34	0	0	2
3956	13680	9936	962	300	164	28046	1312	7	591	1	10	57	4	61	271.09	88	423.55	279	302	267	13	37	0	0	3
4466	13204	9600	792	536	296	28110	1196	6	603	0	6	61	4	82	275.76	87	431.94	337	299	300	15	38	0	2	2
5176	12682	9152	605	706	430	28178	1092	3	677	0	32	55	2	99	279.76	107	437.24	397	345	333	14	38	0	0	2
11374	7914	5496	2	1994	1444	28552	545	92	616	23	286	194	45	276	316.49	111	461.09	479	204	361	103	113	9	44	12
3864	13586	9908	965	450	268	28092	1316	6	539	1	14	52	2	73	270.99	79	423.32	272	265	259	19	32	0	2	1
14254	7496	4328	3	1000	772	27960	608	320	934	154	104	249	121	170	273.14	230	437.34	506	374	431	24	80	13	6	1
3952	13530	9870	930	446	270	28096	1277	4	553	0	28	49	1	85	274.06	85	427.02	282	271	282	13	34	0	0	3
5342	12666	9148	617	612	338	28126	1109	8	726	0	20	79	8	92	277.91	106	435.26	366	394	324	20	56	0	0	4
12128	7796	5112	0	1794	1338	28442	529	137	653	31	250	172	73	276	305.41	120	455.67	469	207	423	83	98	12	24	10
5578	12534	8948	590	640	414	28152	1066	8	714	2	38	66	6	97	284.4	120	440.11	395	351	348	20	47	0	0	1
3972	13658	9928	959	310	166	28042	1301	9	584	2	8	57	3	64	271.39	80	425.2	266	283	285	18	35	0	0	3
11748	7816	5200	2	1916	1494	28504	527	113	629	25	278	176	51	268	308.64	110	457.14	477	220	390	89	91	7	50	25
4272	13436	9758	884	390	202	28060	1240	6	607	0	2	64	3	67	274.52	110	428.63	310	279	290	18	38	0	0	2
13244	7768	4634	2	1292	1048	28160	602	226	820	85	158	204	105	193	283.34	202	445.68	515	308	424	42	82	18	16	4
14340	7558	4360	6	902	662	27892	615	329	1016	156	66	263	122	159	274.79	226	437.32	477	403	447	29	98	19	4	2
5142	12754	9228	658	636	362	28146	1122	11	646	4	24	64	4	84	283.41	101	437.39	390	339	319	14	44	0	0	2
13142	7854	4734	3	1270	990	28136	607	204	902	76	128	184	94	220	276.37	197	442.91	475	337	460	26	88	10	18	8
7448	11166	7850	340	1024	678	28242	913	31	770	7	70	88	21	163	288.64	126	446.45	410	363	383	28	55	0	6	4
4194	13522	9798	900	344	190	28056	1262	8	605	0	8	50	5	66	272.29	93	425.12	289	304	304	20	34	0	0	1
4340	13376	9674	845	424	258	28090	1246	6	601	1	16	68	4	89	274.29	86	429.02	291	308	288	13	44	0	2	3
4734	13046	9448	733	548	322	28122	1139	4	631	1	24	64	2	80	277.77	106	434.81	348	300	338	23	43	0	0	5
14220	7674	4450	10	850	628	27892	620	333	1022	160	58	251	129	153	270.31	265	434.93	502	408	446	17	83	22	10	2
5314	12598	9136	637	680	386	28146	1120	12	689	1	32	76	7	94	281.32	110	437.44	370	346	307	22	53	0	0	2
4868	12952	9370	712	578	338	28126	1159	4	668	1	16	59	2	96	276.34	94	432.89	344	341	322	14	36	0	4	1
10832	7920	5604	0	2156	1724	28738	530	70	556	7	450	162	32	283	324.58	111	465.79	485	176	346	111	94	7	52	34
4320	13454	9712	860	356	212	28066	1232	9	621	0	12	51	6	71	274.01	101	428.23	311	300	311	14	33	0	0	2
5050	12870	9248	687	576	368	28148	1136	7	661	0	34	54	7	96	277.17	99	435.61	357	354	333	15	36	0	2	2
3902	13702	9926	982	318	196	28056	1308	7	553	1	12	55	3	59	271.25	83	423.09	281	270	270	15	36	0	0	4
14116	7742	4496	7	862	624	27898	648	317	1053	151	56	241	131	142	270.28	267	433.9	510	433	442	11	86	18	2	2
4740	13130	9500	766	450	246	28080	1198	10	670	1	14	65	3	69	274.61	111	429.57	350	336	290	15	44	0	0	1
4638	13196	9522	786	450	272	28102	1216	3	647	1	24	56	0	93	277	77	431.75	308	340	308	12	44	0	0	2
10562	7920	5706	1	2354	1806	28830	518	56	469	11	418	166	26	309	330.57	77	469.49	457	139	343	136	85	5	56	28

SUPPLEMENTARY INFORMATION:Monte Carlo Atomistic Simulation and Machine Learning Analysis of Na-K Eutectic Alloy in Condensed Phases, D. Reitz and E. Blaisten-Barojas, George Mason University, Fairfax, VA 22030

13180	7782	4752	4	1306	982	28156	623	196	878	69	138	200	98	214	283.3	163	447.07	447	334	441	51	111	11	16	6
14154	7688	4476	7	884	636	27902	629	316	1021	160	62	260	117	156	273.39	233	437.18	487	426	435	25	85	14	2	1
5084	12730	9198	650	698	432	28180	1100	9	642	1	36	54	6	95	282.09	116	438.23	384	319	342	18	38	0	2	2
4654	13128	9530	802	498	272	28098	1198	5	633	0	16	67	2	80	276.1	101	431.26	338	309	296	13	47	0	0	1
14354	7652	4416	4	792	582	27866	633	323	1085	164	70	266	127	154	270.24	286	433.36	483	439	438	17	93	16	0	3
11954	7782	5096	1	1882	1468	28496	520	115	657	31	278	186	59	256	309.66	118	458.57	479	234	410	98	97	6	32	19
5052	12998	9290	680	452	290	28106	1134	7	717	1	22	56	4	86	276.48	108	431.4	364	365	342	12	39	0	2	1
11632	7842	5292	3	2014	1460	28518	518	111	619	17	252	187	56	302	315.87	128	461.44	464	180	369	95	104	9	26	14
12770	7732	4958	4	1548	1074	28256	591	173	778	64	162	212	69	214	292.93	163	449.88	491	288	402	64	113	16	12	7
4528	13206	9574	803	488	284	28100	1204	8	611	2	20	60	2	83	275.26	91	430.7	336	308	298	10	41	0	0	2
11806	7824	5186	2	1908	1464	28500	584	131	613	24	278	161	76	228	303.91	132	454.54	527	224	386	72	83	10	30	17
4248	13394	9720	852	434	270	28098	1228	5	590	0	32	52	3	84	275.31	94	429.18	307	296	305	15	38	0	0	2
4098	13590	9864	935	308	172	28048	1282	7	588	2	14	72	3	77	272.27	92	426.5	276	285	270	17	47	0	2	0
13322	7784	4680	3	1250	954	28132	636	218	877	72	132	198	113	193	283.51	188	446.65	461	344	448	51	99	16	10	1
14394	7658	4364	8	802	602	27882	667	338	1047	165	60	246	131	148	271.59	273	435.63	470	432	438	21	71	24	2	2
6912	11482	8146	416	978	642	28230	943	41	687	9	62	98	21	138	288.24	115	443.61	421	339	350	28	53	2	6	9
6578	11680	8364	413	942	594	28226	961	25	740	6	58	69	11	113	288.44	138	443.84	462	362	359	16	41	1	10	6
4376	13240	9642	809	528	308	28122	1221	3	589	1	28	56	2	89	277.63	77	432.56	319	311	295	13	32	0	0	2
6094	12070	8700	511	794	468	28178	1009	19	718	4	52	82	11	107	286.51	123	441.63	424	341	352	20	60	1	0	2
3970	13556	9864	942	426	256	28092	1304	9	554	1	20	64	6	85	272.97	69	426.64	272	280	259	13	50	0	0	1
14068	7850	4520	6	806	598	27888	640	310	1052	156	42	254	124	148	268.99	274	432.22	498	431	434	18	87	16	4	1
14198	7744	4492	7	798	584	27884	628	299	1070	138	68	252	133	150	271.95	262	434.48	488	409	451	24	104	16	0	0
3856	13704	9984	1001	314	174	28048	1319	7	558	1	16	59	4	71	270.41	84	423.4	265	268	263	14	41	0	0	1
3978	13650	9926	948	314	168	28046	1278	9	582	1	10	62	3	68	270.65	102	423.43	293	272	278	9	42	0	0	4
12364	7822	5114	0	1632	1180	28352	574	129	775	37	220	177	65	262	300.53	152	453.75	461	256	424	60	107	7	20	13
5562	12574	9018	568	600	356	28142	1060	6	745	0	30	69	4	102	281.07	122	438.1	375	368	363	24	50	0	2	2
13440	7798	4620	1	1204	926	28108	614	198	915	67	106	190	98	179	283.89	207	449.52	499	345	455	37	96	14	14	7
4818	13008	9446	720	546	284	28110	1154	5	651	0	8	59	3	74	277.79	89	434.13	355	335	330	22	40	0	0	1
4142	13518	9808	922	374	212	28064	1282	5	613	1	10	60	4	79	271.34	88	424.53	272	284	286	16	45	0	0	1
12136	7988	5128	2	1628	1250	28372	607	134	746	34	214	169	77	234	297.21	154	452.55	485	296	403	65	91	4	26	10
14224	7700	4354	5	866	696	27916	643	318	996	168	74	259	113	166	269.44	250	432.21	479	394	427	19	78	18	2	2
4436	13386	9670	833	344	206	28066	1216	4	665	0	22	67	3	79	274.91	109	428.4	304	319	310	19	50	0	2	1
11782	7852	5194	5	1880	1472	28520	522	118	601	25	316	178	64	276	301.86	120	456.69	488	183	403	84	105	13	22	18
13210	7734	4704	4	1328	1018	28160	623	214	846	77	154	202	99	205	284.51	184	445.72	475	334	416	50	99	17	12	5
13540	7754	4556	7	1178	932	28088	637	245	895	92	114	210	115	185	277.76	195	446.11	490	357	436	35	100	19	14	0
12810	8070	4932	9	1218	952	28132	615	199	904	68	140	210	99	204	281.35	199	447.24	487	344	434	36	106	18	8	4

SUPPLEMENTARY INFORMATION:Monte Carlo Atomistic Simulation and Machine Learning Analysis of Na-K Eutectic Alloy in Condensed Phases, D. Reitz and E. Blaisten-Barojas, George Mason University, Fairfax, VA 22030

14466	7610	4334	4	812	594	27868	629	340	1048	166	52	258	134	150	272.02	260	436.36	481	432	448	22	78	18	0	3
5074	12812	9246	655	606	368	28138	1109	3	681	0	32	63	3	93	276.94	93	433.71	341	354	357	26	38	0	0	7
7388	11260	7976	312	938	580	28212	890	23	838	4	68	99	15	124	289.74	142	444	454	408	385	35	71	1	2	4
4480	13382	9648	830	344	196	28060	1234	6	651	2	10	69	1	78	274.51	94	428.79	308	337	294	12	45	0	0	1
5106	12640	9210	646	756	432	28176	1129	5	655	0	30	62	5	85	279.21	99	437.69	386	343	303	24	40	0	2	0
13814	7490	4452	2	1262	932	28072	603	276	842	117	116	229	114	189	278.51	199	445.64	508	325	429	32	88	21	4	1
5000	12904	9322	687	560	316	28118	1159	9	677	0	16	62	6	78	277.38	94	432.87	361	364	314	16	45	0	0	3
6586	11824	8422	388	814	490	28178	951	11	835	3	40	88	4	111	285.83	157	444.9	444	405	371	24	60	0	2	3
4258	13420	9726	872	410	248	28084	1276	5	612	0	22	57	3	75	275.25	88	429.1	285	323	282	19	36	0	0	1
5554	12496	8982	609	688	404	28144	1083	18	684	3	18	76	11	113	282.17	107	438.67	373	338	329	15	43	1	2	2
4194	13422	9752	869	458	260	28094	1246	6	582	0	6	57	5	67	272.37	86	426.51	308	295	298	18	34	0	0	4
14184	7734	4486	7	838	596	27886	648	326	1038	147	38	261	141	154	271.54	255	436.5	473	427	431	25	99	21	10	1
14448	7564	4300	8	852	654	27896	640	335	1023	162	76	258	134	164	271.37	236	434.11	476	433	437	20	83	18	2	0
4606	13202	9534	787	466	276	28102	1200	5	638	0	18	52	3	84	277.56	89	433.98	317	325	326	16	36	1	0	2
14240	7708	4446	10	822	604	27880	638	325	1052	163	58	263	120	147	270.29	282	433.89	507	407	425	15	93	20	2	0
4174	13528	9802	894	346	196	28056	1251	9	587	1	10	52	4	67	270.46	87	424.18	294	294	309	18	31	0	0	2
5124	12762	9242	685	610	362	28142	1148	10	668	2	42	63	5	87	279.55	109	437.34	356	349	318	20	43	0	0	3
11838	8004	5286	1	1752	1290	28418	531	119	722	29	218	184	63	252	298.52	154	455.53	503	240	413	67	96	8	28	16
4066	13598	9860	910	334	188	28054	1272	6	593	1	8	65	3	69	270.35	93	424	288	307	282	14	47	0	0	4
10706	7868	5752	0	2286	1684	28758	495	65	476	9	414	179	42	301	331.26	73	471.79	451	142	359	131	92	2	44	34
7608	11082	7752	305	992	704	28250	858	39	782	17	104	100	13	161	288.3	119	444.02	441	375	388	33	57	2	8	5
14486	7582	4340	4	810	588	27864	691	324	1076	158	54	269	124	138	270.41	254	433.37	469	477	402	24	98	21	4	0
4672	13040	9466	746	584	344	28132	1136	4	603	0	26	67	3	89	278.07	92	434.55	351	299	331	21	42	0	0	3
11448	8008	5392	1	1960	1440	28528	516	105	609	19	248	162	55	275	310.62	135	459.55	473	173	414	96	78	8	32	18
4378	13446	9720	864	322	172	28044	1258	10	649	1	6	62	4	73	273.28	95	426.2	294	334	294	12	43	0	0	1
14522	7524	4282	6	856	636	27888	626	346	997	166	66	265	134	154	273.56	244	438.61	481	393	438	28	87	20	2	1
7646	11118	7824	318	950	600	28206	926	42	816	12	62	99	24	137	289.58	144	445.1	416	419	372	36	59	1	4	3
13832	7830	4620	2	926	664	27940	665	279	1034	144	66	234	106	179	274.44	221	439.72	445	434	440	25	78	14	2	1
4152	13548	9832	922	324	180	28050	1288	5	612	1	12	66	3	68	272.4	88	424.33	282	308	279	11	40	0	0	4
14236	7756	4426	5	790	606	27872	633	318	1056	170	58	264	109	154	269.7	266	432.48	487	407	435	19	85	19	0	1
14120	7804	4478	7	824	618	27892	662	303	1061	157	42	260	116	165	269.21	263	432.88	471	449	417	16	94	14	2	1
13624	7798	4656	5	1064	772	28006	623	279	962	125	88	217	116	160	275.64	221	439.21	494	371	459	33	77	19	4	4
14202	7686	4426	6	860	654	27904	620	316	1055	167	72	267	116	174	270.08	250	432.26	478	422	437	19	86	18	4	1
14342	7626	4392	7	840	612	27872	628	322	1050	164	58	272	112	163	270.84	261	434.23	485	408	428	15	94	24	2	1
4050	13616	9862	949	328	190	28054	1314	9	592	2	8	67	6	65	271.11	93	423.86	276	308	258	12	45	0	0	4
5792	12362	8838	558	702	438	28170	1045	10	728	0	34	67	5	113	282.84	128	440.93	399	353	343	19	41	0	4	1

3820	13694	9976	1000	358	202	28060	1308	5	560	1	10	62	2	63	270.77	95	423.7	277	258	267	16	45	0	0	3
7200	11368	8024	389	918	620	28222	915	31	773	6	88	90	19	138	288.62	134	444.37	431	364	385	28	60	1	2	5
3848	13828	10004	1013	208	124	28022	1326	9	584	1	10	72	7	68	270.12	91	422.2	259	278	259	12	42	0	0	1
14212	7650	4440	4	884	650	27908	622	316	1027	147	58	251	133	146	274.73	239	438.59	491	417	448	30	87	10	14	3
4156	13500	9816	934	378	202	28060	1307	8	601	1	8	56	6	68	270.89	74	423.43	273	311	276	13	36	0	0	3
13360	7640	4602	1	1362	1032	28138	604	233	835	93	130	213	103	213	282.91	188	446.82	469	319	429	49	90	13	10	3
11978	7958	5134	1	1730	1344	28404	554	139	716	43	232	193	68	248	299.31	135	453.04	461	249	417	84	102	7	28	16
7314	11286	7906	349	970	682	28256	924	32	745	6	92	75	17	157	286.89	130	445.09	439	362	365	21	44	2	6	2
5910	12190	8778	522	784	470	28178	1070	12	703	2	46	78	6	123	283.79	93	440.23	376	380	325	19	54	0	0	1
13636	7692	4528	1	1156	924	28082	618	263	872	104	130	216	116	187	279.1	195	444.83	473	328	446	43	88	19	16	4
5766	12312	8854	545	716	458	28172	1059	20	715	6	66	65	9	110	285.68	101	441.37	371	375	356	27	44	0	0	2
10842	7924	5648	2	2200	1668	28722	521	79	499	11	378	163	35	271	325.6	92	465.08	494	162	355	120	72	7	62	27
13736	7764	4600	3	1054	764	28002	624	257	1006	122	78	245	103	161	278.16	243	442.08	509	380	410	35	109	12	6	2
11042	7902	5574	1	2160	1600	28668	505	72	548	17	346	185	34	284	324.34	94	466.93	441	174	383	123	89	5	38	34
4818	12960	9376	708	602	360	28140	1127	2	641	0	24	60	1	95	279.01	96	435.08	354	319	339	18	40	1	0	2
4874	12994	9386	700	534	308	28114	1164	2	686	1	18	69	1	98	276.59	110	433.95	340	362	312	12	45	0	0	1
13404	7570	4506	4	1426	1110	28168	581	242	783	97	138	225	109	234	283.06	164	447.02	481	296	410	41	94	10	12	6
11624	7946	5284	3	1934	1440	28500	548	121	638	36	248	194	63	265	309.75	119	457.09	476	210	383	85	104	11	18	22
5186	12836	9246	646	534	296	28114	1115	6	731	0	16	54	4	86	279	116	435.66	365	368	358	17	40	0	0	1
3774	13742	10006	1018	332	192	28060	1326	5	545	1	12	47	3	62	271.35	81	423.46	268	260	275	15	30	0	2	2
4062	13620	9874	949	310	172	28046	1289	7	597	1	8	57	5	64	271.47	97	425.42	287	281	284	12	42	0	0	2
4874	13008	9356	702	526	334	28128	1145	5	662	0	28	72	4	92	277.44	99	433.07	362	339	310	13	42	0	2	1
13050	7830	4776	6	1332	1016	28156	620	191	852	74	138	206	81	197	282.41	179	443.59	469	324	440	50	105	21	14	6
14364	7606	4366	5	870	626	27882	647	328	1061	164	46	262	124	156	269.64	276	432.68	492	432	423	13	89	20	4	1
4886	12924	9346	699	620	354	28140	1182	6	669	0	10	59	5	80	277.19	98	432.8	346	345	305	20	38	0	0	3
14254	7780	4402	10	782	612	27886	629	289	1069	149	56	262	112	156	269.84	289	432.4	510	422	431	8	92	16	0	1
5796	12102	8806	582	912	540	28210	1084	25	637	2	46	63	16	112	285.45	102	441.73	380	343	323	26	42	0	8	2
4306	13434	9724	903	374	216	28068	1258	10	626	1	14	54	5	77	274.75	113	428.54	293	282	293	15	42	0	0	1
11318	8032	5516	2	1914	1430	28556	524	93	638	16	312	181	46	291	314.96	112	461.47	477	209	364	95	98	4	34	20
12986	7692	4692	1	1532	1176	28252	581	180	776	67	156	200	78	213	286.96	161	451.41	505	292	426	54	100	10	18	7
14300	7692	4382	7	820	626	27880	696	303	1074	152	58	256	112	150	270.32	244	433.05	451	466	415	23	92	15	2	2
14278	7700	4406	4	832	618	27888	635	327	1040	150	50	256	142	166	270.21	260	435.07	487	431	433	14	87	18	4	0
13926	7718	4552	3	956	708	27958	612	271	1028	124	96	247	114	160	274.69	235	440.43	496	380	441	29	102	16	2	5
14436	7588	4350	4	848	604	27874	609	339	1036	164	44	271	135	163	273.28	260	437.24	486	402	442	21	96	15	4	1
6658	11620	8266	377	984	650	28254	942	28	721	10	72	87	14	134	289.72	110	444.81	434	374	362	34	58	0	4	1
7292	11232	7936	391	978	682	28232	968	38	765	12	104	95	21	125	287.65	129	446.12	413	382	343	38	55	1	8	6

SUPPLEMENTARY INFORMATION:Monte Carlo Atomistic Simulation and Machine Learning Analysis of Na-K Eutectic Alloy in Condensed Phases, D. Reitz and E. Blaisten-Barojas, George Mason University, Fairfax, VA 22030

3826	13730	9994	1001	310	174	28046	1304	5	551	0	10	61	3	75	271.29	89	424.25	258	250	277	14	41	0	2	4
12342	7832	4976	1	1700	1300	28384	586	154	688	51	210	185	68	262	299.8	132	455.88	465	243	398	62	88	13	22	15
4218	13462	9774	908	392	214	28068	1280	8	598	2	8	54	5	70	274.54	93	427.55	283	302	289	12	32	0	0	6
13636	7790	4654	10	1062	764	27990	586	271	974	124	78	238	112	186	275.6	249	440.99	494	361	460	28	101	16	6	0
4352	13218	9630	805	568	336	28130	1226	4	589	0	26	70	4	77	276.23	95	431.11	333	307	268	19	40	0	0	1
12422	7912	4976	1	1580	1212	28310	586	141	787	37	196	181	75	233	289.65	169	450.44	498	281	424	53	111	14	10	7
4138	13566	9822	924	332	190	28058	1292	12	594	2	10	56	7	63	272.8	94	426.92	279	304	283	18	41	0	0	3
4482	13284	9596	812	450	268	28094	1196	8	611	1	14	59	7	90	275.96	85	429.3	314	300	322	15	41	0	0	1
5246	12788	9204	677	540	308	28104	1119	6	693	2	16	77	3	97	279.59	103	437.79	334	341	341	22	57	0	2	5
14422	7604	4328	1	846	626	27880	646	324	1036	154	52	253	129	153	272.2	273	435.19	484	410	438	15	81	22	2	2
4734	12960	9418	734	630	374	28146	1175	6	614	0	28	60	2	85	280.64	86	436.82	359	323	292	17	48	1	2	2
10984	7928	5490	3	2184	1684	28664	533	84	599	16	356	168	40	286	315.01	118	459.33	481	195	358	105	84	5	34	25
11824	8106	5300	4	1692	1244	28388	629	113	778	25	204	166	65	229	297.78	155	453.7	486	327	389	67	92	7	16	10
5868	12138	8734	487	870	554	28228	1038	11	725	3	64	69	6	118	285.09	100	439.93	410	402	332	24	42	0	0	1
6428	11784	8412	389	972	610	28258	954	12	735	1	48	70	7	137	289.35	126	444.51	436	377	363	24	34	0	2	4
3996	13640	9886	954	334	194	28058	1296	6	573	1	8	58	4	73	272.69	85	425.02	278	282	275	12	37	0	0	2
13626	7920	4694	6	956	700	27972	634	261	1006	123	70	230	108	160	275.2	243	438.42	487	384	444	33	90	15	6	1
11418	7740	5324	2	2126	1632	28634	549	97	552	20	350	165	43	291	316.1	99	460.51	461	190	361	108	92	13	40	17
5610	12514	8956	601	636	394	28144	1108	16	745	3	34	59	10	104	282.23	102	437.73	361	401	344	16	41	0	0	2
6758	11778	8306	414	806	504	28190	973	24	791	6	38	76	15	111	284.5	149	441.57	447	393	365	16	50	1	0	4
4910	13032	9354	686	492	306	28116	1157	3	683	0	22	70	2	95	278.4	100	434.14	339	368	314	17	47	0	0	2
14206	7674	4432	11	868	650	27904	626	304	1011	153	70	255	114	154	272.82	244	437	487	399	445	26	87	19	4	1
3918	13662	9910	956	356	214	28070	1291	3	555	1	8	60	2	82	271.5	95	426.13	278	269	273	8	43	0	0	3
12522	7910	4912	1	1546	1206	28300	624	153	771	52	194	185	78	240	286.79	146	448.9	478	295	401	46	98	8	10	8
4370	13368	9660	831	422	256	28092	1231	4	583	1	12	48	1	77	276.97	84	430.96	309	319	311	18	33	0	4	0
13846	7792	4518	3	966	762	27982	658	265	998	112	92	223	111	170	275.75	226	441.93	471	417	435	29	93	11	6	1
11254	7882	5484	1	2062	1550	28618	537	83	601	21	354	174	42	288	317.65	111	464.22	465	205	368	109	84	4	26	20
10946	7966	5586	0	2098	1648	28700	521	74	562	13	412	156	37	303	325.37	113	465.99	482	171	354	103	91	2	36	28
5288	12770	9166	625	526	330	28122	1090	7	723	2	42	75	3	96	280.58	110	436.02	366	369	344	23	52	0	0	1
13002	7920	4844	4	1268	960	28140	661	202	906	77	136	197	98	190	282.85	196	446.82	471	387	413	48	97	10	10	2
3886	13648	9934	980	374	214	28066	1302	4	546	1	8	55	0	70	271.15	77	422.83	265	253	280	19	38	0	2	3
11244	8022	5456	3	1988	1516	28568	575	83	635	14	312	169	46	253	306.97	118	460.26	482	219	368	99	101	6	28	20
11244	7764	5520	1	2056	1602	28672	471	83	586	18	430	179	39	271	325.99	115	467.94	490	173	379	126	109	6	50	32
13238	7986	4824	4	1076	822	28080	629	210	980	66	126	196	108	199	281.76	211	445.75	439	365	471	45	103	15	8	5
4712	13214	9520	804	396	220	28070	1199	8	684	0	8	63	4	71	277.06	120	432.16	338	336	312	12	43	0	0	1
12212	7852	5106	2	1650	1270	28370	532	165	680	39	258	187	83	250	299.73	148	451.16	481	207	428	79	94	13	20	9

5554	12480	8960	598	692	436	28166	1106	15	677	3	42	76	8	98	283.96	119	440.45	396	348	298	17	49	1	2	0
14374	7586	4334	3	856	664	27900	658	324	1030	162	80	264	121	146	273.02	256	436.94	479	438	416	26	83	21	6	4
3870	13718	9962	981	316	178	28050	1307	7	569	1	6	66	5	61	270.41	104	424	290	258	256	12	37	0	0	2
4362	13348	9674	855	428	254	28088	1222	8	615	0	22	60	6	67	275.72	98	430.25	329	299	297	15	37	0	0	4
14250	7788	4464	8	742	560	27862	647	317	1109	158	58	258	125	152	271.34	275	433.6	485	462	436	14	92	20	0	1
12506	7716	4970	4	1682	1236	28332	507	176	705	58	194	190	76	239	297.86	149	455.07	493	220	445	80	86	12	28	11
13798	7706	4520	1	1084	818	28026	632	269	912	119	94	240	108	177	278.22	211	444.98	493	365	412	29	96	21	4	5
13188	7842	4750	1	1232	966	28144	587	217	884	78	150	203	108	188	282.97	212	445.3	483	320	459	57	94	15	10	6
13762	7606	4544	3	1158	856	28050	593	268	875	101	116	219	118	180	277.11	209	444.21	505	322	453	38	98	27	4	0
4914	12932	9374	725	578	314	28124	1167	6	661	0	10	66	5	81	280.15	102	436.86	354	341	308	19	46	0	2	1
11070	8018	5552	0	2110	1540	28612	520	97	569	20	282	186	54	292	316.47	115	460.71	474	185	366	93	106	5	40	31
14414	7690	4364	9	744	578	27856	650	325	1075	170	66	274	117	146	270.73	272	435.16	490	428	419	16	93	20	0	0
11640	7794	5180	1	2006	1602	28598	525	108	567	25	330	166	57	259	317.7	104	460.91	505	200	383	103	80	7	44	22
12526	7870	4980	2	1528	1178	28312	583	145	757	45	200	203	69	228	297.84	167	451.76	473	256	397	74	115	13	28	12
12996	7862	4754	1	1364	1064	28196	584	180	821	71	138	210	71	202	283.33	180	448.59	522	304	412	45	105	13	16	6
10474	7694	5704	3	2542	1940	28898	524	57	436	6	484	144	27	316	332.08	66	473.18	457	153	342	121	64	4	50	45
13330	7720	4672	3	1268	974	28118	614	239	902	103	138	207	107	218	277.11	206	444.7	466	328	445	27	87	11	12	8
4576	13218	9554	813	460	270	28094	1218	13	618	3	14	58	4	86	275.47	85	431.31	311	320	302	15	36	1	2	2
7996	10922	7576	301	996	658	28210	883	53	844	11	60	94	32	143	289.02	151	447.04	447	399	395	23	64	3	2	4
4174	13574	9838	911	290	152	28036	1270	9	629	0	8	66	4	80	271.67	103	425.51	275	313	291	12	46	0	0	1
14298	7746	4408	5	768	592	27870	601	331	1065	170	52	260	126	162	268.36	272	431.93	503	427	456	11	77	16	6	1
5084	12886	9276	694	540	312	28114	1151	8	685	0	16	66	7	90	277.61	98	435	342	357	325	20	45	0	0	1
7244	11252	7980	337	1022	666	28252	954	32	789	9	84	83	18	120	290.05	122	446.06	424	408	372	36	50	1	4	5
4274	13484	9734	869	360	210	28068	1243	6	617	1	6	64	3	69	274.11	97	427.18	313	301	285	11	50	0	0	6
4226	13518	9772	869	344	194	28058	1222	8	607	0	4	59	3	81	271.91	102	426.68	305	286	306	15	39	0	0	1
6142	12130	8648	440	756	470	28190	998	16	782	0	40	61	12	115	283.13	125	440.96	420	413	372	18	38	0	4	4
14150	7696	4422	4	870	692	27926	650	297	1019	141	88	250	113	165	272.76	246	437.45	482	426	429	18	90	27	8	1
4064	13536	9840	887	398	226	28076	1227	4	548	0	12	57	2	70	273.91	87	428.71	309	270	308	19	38	0	0	2
11986	8032	5200	2	1630	1280	28412	584	128	709	28	260	157	65	240	302.19	145	456.04	474	247	418	74	79	10	20	15
5026	12924	9322	680	518	294	28100	1148	8	707	1	14	66	4	82	277.12	103	432.6	353	360	329	16	51	0	2	2
4948	12968	9330	708	518	326	28124	1133	11	661	1	34	55	5	84	278.84	109	434.29	368	339	334	15	30	1	0	2
4326	13420	9724	858	382	208	28068	1249	2	620	1	8	66	0	65	273.72	90	429.04	307	320	291	20	44	0	0	0
13706	7826	4622	8	996	744	27982	647	270	1011	124	78	226	110	190	275.54	227	442.71	464	420	434	18	90	12	10	5
4028	13522	9850	937	432	242	28084	1270	6	560	3	8	62	3	70	271.73	97	425.63	305	258	275	13	42	0	2	2
6166	12020	8614	470	842	514	28202	1007	11	725	1	44	76	7	118	286.85	113	443.61	414	377	346	28	47	0	2	0
13906	7746	4522	10	952	736	27962	642	278	994	124	94	242	116	184	276.23	222	440.04	446	402	442	32	98	17	6	2

SUPPLEMENTARY INFORMATION:Monte Carlo Atomistic Simulation and Machine Learning Analysis of Na-K Eutectic Alloy in Condensed Phases, D. Reitz and E. Blaisten-Barojas, George Mason University, Fairfax, VA 22030

13162	7716	4726	2	1344	1040	28176	603	225	806	95	174	204	89	213	281.92	175	449.41	489	320	425	42	84	17	14	5
5192	12784	9194	639	584	352	28132	1091	10	676	3	26	68	4	98	278.57	101	435.98	371	339	345	20	39	0	0	1
13722	7918	4674	10	884	662	27944	654	296	1025	135	80	222	127	157	277.09	247	440.66	481	416	447	24	76	15	4	2
13496	7800	4622	4	1128	894	28078	635	254	893	99	122	218	114	178	279.94	224	444.42	488	348	424	42	94	25	14	2
14414	7666	4330	5	808	612	27876	633	324	1062	159	38	258	123	154	268.69	282	431.38	492	405	439	16	86	19	6	1
11404	7868	5396	1	2050	1530	28598	536	100	575	18	318	158	49	304	319.1	120	460.07	466	189	374	92	76	6	28	16
13734	7658	4452	2	1164	930	28064	590	255	908	105	116	235	111	213	277.06	206	445.37	458	352	449	37	101	20	8	6
4070	13556	9846	911	370	212	28068	1258	6	576	0	14	62	3	59	273.84	99	426.34	306	276	286	20	43	0	0	3
4026	13648	9900	963	298	162	28040	1294	6	601	1	4	60	3	69	270.28	81	423.82	260	275	292	18	44	0	2	3
3894	13552	9918	951	466	252	28092	1291	5	541	1	10	58	3	68	271.91	78	425.4	303	259	261	11	46	0	0	3
4824	12982	9356	697	576	374	28152	1156	8	641	1	38	57	4	94	279.75	102	435.9	360	351	312	12	33	0	2	3
4714	13062	9466	726	552	314	28126	1154	5	621	0	18	65	3	90	276.94	86	432.95	351	318	314	16	48	0	0	3
4094	13570	9834	937	348	202	28058	1283	5	588	1	10	65	3	72	272.07	91	425.85	283	278	277	14	43	0	0	2
11934	8006	5270	3	1714	1236	28382	583	136	730	30	200	148	75	259	295.89	146	453.85	483	262	433	55	76	5	20	9
5390	12636	9066	583	638	392	28152	1065	11	686	2	30	65	6	100	281.64	116	437.91	409	359	344	9	38	0	0	2
4078	13568	9838	906	348	212	28064	1271	3	595	1	18	64	0	83	273.33	74	426.15	278	309	286	10	45	0	2	4
13976	7786	4450	3	920	736	27946	608	298	979	157	70	246	107	163	274.57	259	437.77	500	372	443	28	69	13	8	1
14154	7744	4444	6	844	650	27908	630	301	1039	154	68	256	110	161	274.33	246	436.3	481	412	438	25	89	16	4	1
4564	13278	9588	785	412	228	28076	1207	2	661	0	6	61	1	75	275.43	105	430.35	318	329	316	18	39	0	0	1
12182	7954	5110	2	1662	1222	28322	582	141	768	44	172	180	75	228	288.01	146	450.1	471	264	434	76	103	6	18	7
14610	7642	4340	4	694	496	27822	648	337	1118	177	36	275	131	130	267.94	290	429.48	489	462	434	18	89	13	4	3
4014	13534	9872	920	412	228	28074	1249	9	549	2	12	61	5	75	273.05	88	427.74	294	246	294	20	38	0	2	1
13580	7606	4594	3	1244	922	28086	625	243	883	95	132	237	114	190	283.2	190	446.47	476	350	407	51	115	14	6	0
4022	13640	9872	933	322	192	28058	1292	4	580	1	10	60	3	64	272.31	87	426.06	280	294	280	18	43	0	0	2
4140	13450	9782	898	432	260	28090	1254	4	589	0	26	64	4	71	273.21	100	427.49	309	271	279	18	43	0	0	1
6556	11798	8420	433	850	518	28190	950	23	728	4	46	87	14	124	288.1	113	443.78	430	357	369	27	57	2	0	2
14324	7556	4354	4	924	684	27920	631	318	1020	154	74	256	128	150	270.23	262	434.68	504	407	429	22	88	22	4	1
4590	13228	9558	783	440	260	28098	1202	3	652	0	20	56	1	81	276.66	105	432.22	322	338	318	12	39	0	2	5
4168	13436	9782	911	438	246	28086	1275	11	578	0	16	50	7	73	273.78	78	427.07	292	294	290	14	38	0	0	2
10492	7836	5660	2	2450	1914	28864	473	52	462	9	448	156	30	310	331.8	73	472.59	479	149	368	133	71	0	50	32
12102	7932	5160	2	1662	1260	28374	603	143	737	33	236	171	70	224	298.87	149	453.65	478	257	391	81	96	14	20	14
10750	8126	5686	0	2090	1612	28682	509	75	546	13	386	158	44	287	318.45	109	463.12	496	163	381	99	74	3	32	29
13184	7608	4756	2	1384	1046	28192	586	211	828	68	192	213	102	232	286.96	176	450.13	475	319	414	49	114	7	22	5
5892	12430	8870	570	566	328	28110	1073	16	784	4	22	69	6	113	281.08	120	438.08	373	400	352	8	43	0	2	2
12908	7742	4896	2	1454	1042	28224	585	183	814	55	174	189	97	244	292.78	182	451.08	469	292	442	42	102	12	8	6
5174	12838	9224	651	546	322	28124	1147	12	711	1	18	71	6	91	281.05	105	437.74	350	373	309	21	44	0	2	2

SUPPLEMENTARY INFORMATION:Monte Carlo Atomistic Simulation and Machine Learning Analysis of Na-K Eutectic Alloy in Condensed Phases, D. Reitz and E. Blaisten-Barojas, George Mason University, Fairfax, VA 22030

4364	13342	9688	846	436	240	28082	1222	7	610	0	12	58	5	71	273.75	114	427.07	323	297	306	13	34	0	0	3
4236	13424	9752	852	418	238	28086	1256	4	616	0	18	57	1	83	273.42	86	428.31	292	324	293	13	38	1	0	2
4406	13388	9696	850	370	192	28054	1244	10	632	1	2	68	6	69	272.12	95	426.04	314	316	280	12	47	0	0	3
14452	7588	4288	8	854	650	27892	620	312	1034	153	56	251	118	138	271.93	279	435.64	514	400	445	23	84	22	4	1
6400	11860	8482	448	876	540	28210	1008	23	719	7	48	77	13	119	287.7	117	443.99	420	390	340	26	46	0	4	0
4020	13590	9870	947	364	210	28066	1291	8	579	2	10	56	4	62	270.38	88	422.41	291	285	279	14	34	0	2	3
6286	11972	8582	469	822	480	28176	1022	18	764	4	34	85	7	110	281.9	116	438.85	418	408	334	19	54	1	0	3
5142	12928	9280	683	464	262	28088	1140	10	715	0	12	61	5	80	279.19	119	436.74	337	363	347	23	38	0	0	4
6764	11716	8238	390	852	580	28212	933	35	771	11	60	75	19	116	283.84	138	442.58	457	388	384	19	41	0	0	7
4380	13332	9646	843	442	274	28100	1223	9	598	1	26	62	4	85	275.2	99	429.71	316	301	293	11	40	0	0	4
12074	7930	5144	1	1700	1296	28402	583	147	690	31	224	163	77	238	302.47	153	454.15	479	245	409	81	70	10	34	12
13834	7816	4588	4	958	692	27948	601	271	1006	126	54	242	116	180	274.98	222	438.87	458	391	473	34	101	10	4	3
6564	11596	8364	433	1012	630	28248	961	24	709	4	78	81	13	119	289.26	116	443.74	450	344	344	31	54	0	2	1
13648	7696	4592	2	1146	850	28046	643	258	935	107	106	244	114	208	277.68	194	444.85	446	369	414	30	114	22	8	6
11250	7816	5428	1	2170	1624	28666	549	87	551	17	328	157	47	287	320.21	94	464.31	458	174	363	105	83	6	48	29
12336	7824	4990	1	1632	1306	28368	568	161	745	32	242	163	97	239	292.9	160	451.84	472	258	444	71	95	7	34	14
4442	13308	9664	828	424	226	28076	1220	6	630	1	12	71	1	77	274.49	92	430.14	327	321	289	10	51	0	0	2
5018	12986	9322	690	478	284	28106	1152	7	711	1	18	63	5	100	277.76	115	435.31	340	358	321	12	46	0	0	2
11546	7898	5306	1	1978	1490	28536	537	102	606	24	304	167	57	287	304.7	118	453.75	483	199	398	81	90	9	14	14
4764	13126	9494	748	446	242	28088	1181	5	684	0	16	50	1	73	275.2	107	429.99	337	349	333	17	39	0	0	2
5130	12728	9236	671	646	368	28138	1124	12	652	1	30	81	7	98	281.58	87	437.95	362	326	310	20	55	1	0	1
5810	12386	8852	529	646	410	28152	1031	12	733	0	46	62	8	106	283.11	116	438.66	406	373	362	20	45	0	2	0
13180	7916	4782	4	1182	910	28110	605	219	919	83	128	206	102	189	281.89	206	445.74	515	345	432	30	101	9	12	6
4940	12950	9340	695	546	326	28128	1128	6	680	0	26	50	4	110	276.83	111	432.76	345	336	349	11	31	0	0	0
3788	13762	9980	984	306	200	28056	1316	6	558	1	18	57	2	60	270.88	89	423.58	273	280	268	16	33	0	2	4
5436	12602	9042	587	636	398	28152	1063	16	693	1	36	63	10	101	282.18	118	437.77	384	355	353	21	38	0	2	4
6646	11664	8312	443	918	604	28222	989	36	691	9	70	82	19	130	289.82	124	443.78	411	361	347	26	49	2	8	3
12810	7916	4920	2	1384	1020	28206	584	188	843	59	142	202	104	211	287.36	167	447.7	480	305	440	56	120	10	14	9
4940	12896	9352	678	598	328	28128	1158	10	683	0	12	65	7	88	279.39	90	436.58	358	379	309	13	40	0	2	3
13272	7700	4614	0	1366	1070	28186	590	218	843	64	148	186	113	210	284.42	208	448.82	481	303	457	51	91	15	16	4
4646	13204	9534	774	434	252	28088	1187	1	675	0	18	58	0	71	276.61	113	433.61	342	329	320	13	41	0	0	4
7086	11778	8180	363	644	422	28142	937	28	911	1	28	75	15	106	286.73	180	442.27	447	436	398	17	59	2	4	6
11312	7838	5392	1	2122	1602	28622	527	98	574	16	296	179	52	280	314.59	113	463.25	460	188	368	115	91	9	52	27
11760	7826	5258	1	1928	1428	28500	564	113	644	17	272	157	69	283	304.56	115	456.15	483	237	403	73	87	3	24	7
4548	13218	9576	786	472	268	28098	1184	5	631	0	16	56	3	87	277.87	105	432.01	323	303	323	16	36	0	0	3
5254	12718	9164	603	628	362	28146	1133	9	715	0	20	58	5	104	279.88	94	438.44	347	403	327	20	39	0	0	1

SUPPLEMENTARY INFORMATION:Monte Carlo Atomistic Simulation and Machine Learning Analysis of Na-K Eutectic Alloy in Condensed Phases, D. Reitz and E. Blaisten-Barojas, George Mason University, Fairfax, VA 22030

4524	13196	9564	816	516	298	28112	1215	10	619	1	12	54	7	66	275.45	96	431.73	345	316	296	15	37	0	2	3
14378	7704	4440	4	740	532	27848	650	319	1131	157	48	263	125	150	268.35	294	430.23	472	458	438	19	94	18	6	1
6128	12058	8644	517	812	496	28180	1032	25	702	4	34	73	19	124	285.81	98	443.54	379	365	356	25	46	0	8	2
5700	12430	8912	599	664	404	28146	1094	19	705	6	36	61	9	92	283.97	105	438.7	379	358	346	19	39	1	0	3
14500	7594	4352	5	790	566	27856	654	342	1067	171	50	268	132	155	270.8	273	433.25	479	441	422	14	85	19	4	1
4094	13518	9834	925	406	216	28070	1252	7	555	0	2	60	7	74	270.96	94	424.5	303	254	290	13	41	0	0	2
5438	12644	9104	606	598	324	28118	1094	7	721	0	10	73	4	83	278.92	118	435.4	385	363	338	22	51	0	0	0
4394	13370	9670	849	396	230	28074	1220	9	630	1	14	65	5	79	275.75	101	429.51	311	304	303	15	46	0	0	2
4500	13206	9580	788	518	298	28120	1175	9	607	0	18	54	7	78	275.56	107	430.59	349	280	322	15	32	0	0	3
4164	13524	9826	921	344	184	28054	1283	6	613	1	12	75	3	69	272.25	88	425.95	295	302	261	8	58	0	0	5
4670	13062	9456	734	568	350	28138	1174	5	635	0	32	53	3	95	277.85	103	434.46	341	327	309	14	34	0	0	2
3924	13672	9928	979	320	192	28054	1317	5	579	0	18	61	4	67	271.3	91	423	263	282	271	13	47	0	0	5
5510	12582	9042	605	622	356	28132	1051	16	696	6	20	70	7	87	279.75	122	437.64	406	325	359	20	44	0	0	2
3922	13646	9940	974	344	190	28056	1293	7	580	1	14	61	3	76	273.05	99	426.41	277	263	274	11	46	0	0	2
4388	13334	9656	842	432	258	28088	1236	7	624	0	18	62	5	88	274.6	95	428.95	290	318	298	18	41	0	2	2
4120	13542	9826	916	356	204	28062	1270	8	585	1	14	59	4	82	270.31	92	422.91	283	295	288	9	36	0	0	3
6182	11994	8562	479	868	556	28212	1002	18	704	2	44	73	9	119	286.36	108	440.36	411	362	357	29	50	0	6	1
4452	13296	9634	802	434	250	28084	1214	10	629	1	18	62	2	83	275.01	94	430.28	308	332	299	20	36	0	0	1
13312	7952	4746	4	1090	838	28050	623	243	976	104	108	222	106	192	277.28	223	445.07	491	377	430	28	98	13	4	1
13554	7840	4698	6	1038	776	28020	623	251	982	103	102	217	109	157	278.24	234	443.07	478	365	460	47	99	16	12	4
4634	13208	9566	777	432	230	28082	1185	2	645	0	12	55	2	85	276.52	92	432.39	321	330	336	14	37	0	0	1
4718	13172	9528	735	434	224	28082	1145	4	667	0	6	66	2	77	276.09	98	432.31	355	330	333	16	44	0	0	2
4860	13100	9390	741	444	284	28102	1206	10	682	0	24	63	7	80	276.21	101	430.55	340	373	290	10	48	0	0	3
13906	7866	4576	7	874	642	27916	657	296	1036	145	46	253	118	158	270.49	283	433.73	492	411	419	16	88	16	6	0
11470	8066	5452	0	1860	1366	28504	540	106	653	16	260	170	58	255	304.52	142	458.01	503	218	389	87	93	11	28	17
3846	13692	9968	989	352	194	28058	1306	8	536	0	6	65	5	66	271.35	85	424.42	288	255	254	12	43	0	0	2
4898	12956	9366	729	556	324	28122	1163	7	651	1	20	65	4	90	280.72	88	436.64	349	331	312	13	44	0	2	3
7692	11020	7764	279	1034	654	28236	907	36	837	7	68	83	18	137	289.19	157	446.27	453	407	376	26	54	3	4	4
4086	13624	9878	938	284	154	28036	1289	12	601	1	10	67	7	63	272.29	95	424.99	283	301	277	11	48	0	0	5
4934	12948	9332	701	536	338	28126	1139	5	667	0	38	76	5	77	279.11	109	435.21	365	332	311	24	62	0	0	2
5448	12590	9052	620	640	386	28150	1130	14	704	2	32	65	9	95	282.36	117	437.55	374	382	311	17	38	1	2	0
5720	12390	8884	561	696	426	28150	1040	20	716	5	32	77	12	97	281.91	124	437.47	412	362	346	18	56	0	2	3
6482	11796	8414	426	918	568	28224	993	18	738	2	42	70	12	114	286.01	117	442.86	435	379	355	21	41	0	4	3
13702	7642	4532	2	1182	896	28082	606	247	884	95	112	210	114	208	280.15	208	445.84	470	323	450	38	94	12	16	1
4692	13068	9480	755	536	314	28118	1173	8	644	0	26	55	5	78	279.06	95	435.43	349	321	315	18	42	0	2	3
12792	7778	4884	3	1496	1118	28264	555	192	775	50	178	178	96	237	291.39	171	450.77	490	272	450	50	86	18	12	11

SUPPLEMENTARY INFORMATION:Monte Carlo Atomistic Simulation and Machine Learning Analysis of Na-K Eutectic Alloy in Condensed Phases, D. Reitz and E. Blaisten-Barojas, George Mason University, Fairfax, VA 22030

14476	7596	4302	9	834	620	27880	649	349	1009	178	50	268	122	148	269.93	251	431.82	504	429	412	13	81	26	2	1
4310	13424	9718	900	382	220	28066	1269	11	605	2	12	54	5	58	272.52	92	426.44	303	295	287	18	39	0	0	3
14216	7628	4386	3	884	708	27932	625	327	989	156	102	255	128	163	273.72	240	437.87	480	411	439	28	86	16	8	3
5180	12766	9208	620	604	352	28134	1098	8	677	0	24	64	6	96	278.69	98	435.68	367	361	347	19	43	0	0	3
4616	13274	9566	789	386	218	28068	1198	8	672	1	8	66	5	79	274.1	112	428.64	320	328	314	14	49	0	0	3
12018	7776	5106	1	1840	1422	28474	566	133	625	33	280	168	68	279	307.28	115	455.83	465	219	392	74	86	7	32	15
4362	13314	9644	850	464	290	28102	1245	10	606	1	28	52	8	86	273.78	94	427.77	296	312	301	13	35	0	0	3
12066	7868	5078	1	1790	1374	28438	569	132	644	34	234	165	68	231	298.88	128	453.27	488	222	413	93	83	10	26	11
4090	13548	9852	942	356	198	28060	1290	8	583	1	16	66	5	71	272.87	103	426.38	283	282	272	12	45	0	0	3
13440	7874	4682	2	1082	846	28050	601	247	941	103	118	214	101	206	277.7	203	443.37	477	372	455	30	90	16	8	1
14254	7744	4382	4	808	642	27898	630	303	1079	151	68	247	115	152	269.71	273	432.44	502	436	450	12	89	22	0	3
14294	7788	4414	6	722	582	27868	648	326	1094	156	58	258	135	148	270.15	273	431.73	487	463	435	18	93	18	10	0
6812	11644	8252	430	894	558	28206	965	24	745	5	42	77	11	125	287.9	121	446.03	423	377	370	26	45	2	4	2
11184	7902	5462	1	2084	1594	28614	524	103	585	11	358	150	66	267	313.58	111	462.69	474	197	417	102	91	9	30	27
6376	11804	8520	485	924	534	28202	994	27	701	6	38	88	16	129	285.35	106	442.13	412	343	342	19	59	1	6	6
4418	13340	9674	841	408	222	28074	1191	7	599	0	12	58	4	92	277.38	99	432.89	310	285	326	14	39	0	0	1
11422	7790	5382	1	2088	1560	28604	538	109	550	27	310	162	49	287	313.1	106	460.69	476	177	381	93	91	6	50	19
4004	13610	9886	974	350	198	28058	1288	7	550	1	10	57	3	67	271.66	79	423.79	284	260	278	16	35	0	0	2
3956	13682	9928	975	304	168	28044	1307	10	576	2	6	59	6	56	271.79	101	424.73	284	268	268	13	36	0	0	5
4710	12998	9452	729	606	348	28140	1166	2	633	0	26	71	2	77	276.94	101	434.45	360	323	300	22	48	0	0	2
5700	12470	8900	591	618	406	28142	1086	23	719	5	44	80	13	81	283.53	119	440.24	402	371	320	24	58	1	4	1
4646	13076	9496	732	568	322	28126	1133	4	599	0	18	59	3	81	278.59	95	432.99	371	300	329	19	38	0	0	1
14172	7724	4434	5	838	656	27908	624	305	1049	148	82	250	118	165	272.31	274	435.11	494	408	444	15	85	21	2	1
3876	13718	9970	969	294	172	28048	1283	7	563	1	16	58	5	76	270.22	77	423.31	261	267	299	15	36	0	2	3
14436	7618	4338	3	800	614	27878	621	344	1042	166	62	257	133	135	271.01	279	434.28	508	421	444	26	82	25	10	1
5004	12840	9306	699	590	352	28132	1114	13	607	2	38	65	8	103	282.27	91	439.97	363	301	330	14	42	0	2	2
4128	13542	9812	925	360	214	28072	1299	10	595	1	14	52	7	69	272.03	91	424.63	289	308	272	12	37	0	2	1
13602	7766	4610	2	1112	840	28042	610	268	917	125	106	227	118	195	277.94	199	443.94	477	369	441	31	79	11	6	5
4384	13312	9634	840	466	284	28098	1208	8	610	1	16	57	5	91	273.5	101	427.8	312	288	314	11	32	0	2	3
12892	7840	4820	1	1408	1072	28196	595	190	839	62	154	193	102	215	282.22	192	446.22	500	312	443	37	108	11	8	5
4898	12962	9372	696	562	318	28128	1155	5	679	0	14	52	3	91	279.71	97	434.99	350	360	330	16	37	0	2	0
4208	13482	9786	897	374	202	28058	1249	9	593	1	6	63	5	75	273.59	89	427.84	301	290	291	11	42	0	0	3
5720	12408	8886	562	680	424	28160	1082	10	733	1	42	65	6	107	282.74	117	439.37	370	387	350	20	44	0	0	2
13072	7646	4686	2	1472	1144	28216	622	201	797	67	184	205	92	212	284.79	155	448.04	480	315	403	53	105	13	12	5
4546	13160	9548	778	520	314	28120	1219	7	618	1	32	64	4	100	276.74	75	432.73	293	335	302	17	41	0	0	3
4336	13440	9732	868	354	190	28060	1243	6	627	1	8	64	3	61	273.99	104	428.1	317	301	290	18	44	0	0	1

SUPPLEMENTARY INFORMATION:Monte Carlo Atomistic Simulation and Machine Learning Analysis of Na-K Eutectic Alloy in Condensed Phases, D. Reitz and E. Blaisten-Barojas, George Mason University, Fairfax, VA 22030

4282	13484	9738	883	344	202	28060	1256	7	622	2	10	65	3	68	272	92	426.27	296	309	291	19	46	0	0	1
14290	7664	4392	7	852	634	27892	623	323	1045	156	56	266	124	156	268.87	265	430.91	496	416	438	16	98	24	4	3
11242	7944	5428	2	2062	1574	28610	521	91	579	19	326	153	48	266	312.07	131	459.19	511	184	393	97	77	2	30	19
3792	13706	9964	990	372	226	28072	1322	8	541	1	10	59	4	71	269.98	85	422.77	272	264	256	13	36	0	2	2
7116	11488	8144	367	854	522	28180	926	38	826	3	52	77	26	128	288.64	137	444.41	426	402	396	29	53	2	4	1
11388	8114	5516	2	1866	1346	28514	548	83	650	16	262	171	43	262	317.32	116	458.85	460	219	389	94	92	6	20	24
4270	13458	9760	873	372	198	28064	1245	8	626	0	6	50	3	74	275.36	92	428.95	303	302	308	10	37	0	0	3
4382	13356	9686	833	416	228	28078	1228	6	628	0	8	60	6	76	275.1	94	429.35	316	321	302	11	39	0	2	3
4328	13404	9662	856	400	268	28090	1237	8	612	1	28	51	3	88	272.95	82	427.84	285	309	312	16	33	0	0	4
14478	7606	4326	8	802	600	27872	597	338	1067	163	52	261	141	166	273.43	275	438.16	483	408	469	19	83	14	8	1
4738	13048	9450	736	542	316	28116	1165	11	630	1	22	72	8	105	280.16	98	436.78	328	334	306	12	49	0	0	4
13650	7662	4572	0	1190	876	28060	590	262	896	110	98	223	113	196	278.39	198	447.91	486	326	450	37	95	16	12	3
13810	7798	4516	5	990	778	27984	608	260	996	124	90	242	103	173	276.4	245	440.64	489	369	437	36	101	12	2	1
4558	13206	9580	786	464	262	28090	1203	10	621	0	20	69	5	78	276.21	89	431.56	327	334	292	17	45	0	0	4
4090	13582	9854	943	336	182	28048	1298	6	576	1	4	58	2	61	271.05	85	424.02	278	291	277	15	43	0	0	5
6668	11558	8286	418	960	658	28254	951	29	719	9	118	85	16	130	286.84	107	444.81	417	369	357	38	51	0	6	7
3944	13646	9934	961	338	182	28054	1306	6	581	1	10	65	4	69	270.98	89	423.17	276	286	262	14	44	0	0	2
4342	13406	9688	866	388	234	28074	1260	8	605	1	16	64	4	79	274.65	79	430.32	287	311	287	14	42	0	0	3
5708	12360	8866	542	740	464	28184	1012	8	693	1	46	75	5	110	284.64	116	441.2	413	338	356	23	48	0	0	3
12166	7844	5090	3	1746	1300	28384	604	137	745	34	214	167	79	226	290.6	153	450.14	514	272	402	53	97	8	24	14
4540	13180	9596	807	494	268	28098	1235	8	631	2	18	55	3	74	276.38	92	430.8	308	338	295	21	38	0	2	3
5518	12470	8976	614	704	444	28160	1107	21	657	4	46	76	13	119	282.63	89	441.14	350	345	320	19	49	0	2	2
4658	13184	9518	779	454	262	28088	1190	9	615	1	10	67	5	89	279.17	101	433.24	320	324	309	17	36	0	2	1
4904	13004	9380	743	500	296	28108	1154	10	674	2	22	66	5	88	277.47	115	433.6	345	323	322	17	46	0	0	2
10930	7970	5606	0	2152	1612	28660	510	87	553	18	348	168	39	294	318.45	114	463.03	480	169	382	92	82	8	38	32
12210	7950	5168	4	1594	1172	28320	598	138	765	34	210	170	71	237	294.46	156	451.97	488	289	409	57	96	12	16	8
13296	7778	4702	1	1222	956	28124	621	237	849	86	164	223	112	218	281.46	192	446.81	453	330	424	39	102	16	6	8
14326	7578	4320	10	910	706	27922	629	325	992	165	72	258	125	148	271.18	253	435.67	505	415	427	26	79	18	10	1
11448	8018	5308	0	1900	1538	28584	523	96	594	26	338	187	49	265	312.76	117	460.19	500	205	365	92	94	4	34	31
10490	7944	5718	2	2312	1820	28802	479	57	477	7	480	171	34	315	331.56	76	472.08	455	136	370	128	78	0	38	33
14332	7638	4376	6	860	628	27886	633	339	1034	163	48	257	134	140	269.7	269	432.81	511	419	433	20	84	22	4	0
4652	13094	9510	779	540	300	28112	1181	4	629	0	16	58	2	78	274.87	87	429.55	346	317	311	18	40	0	0	1
3914	13662	9934	961	344	194	28058	1295	8	555	1	10	53	4	64	271.27	78	423.39	273	276	290	19	35	0	0	2
14016	7834	4508	5	836	652	27920	660	303	1053	148	72	228	121	154	275.19	281	438.93	473	431	446	24	71	12	0	2
5020	12944	9284	668	518	326	28116	1125	11	693	2	24	71	7	104	278.76	101	436.45	345	362	333	15	44	0	0	2
5356	12748	9126	673	524	324	28106	1120	16	722	2	28	73	12	87	280.45	127	436.51	362	354	331	18	48	0	0	3

13268	7796	4682	3	1268	974	28126	604	229	858	77	130	206	114	217	281.74	192	444.26	462	317	452	38	95	17	6	5
4722	13172	9520	797	432	226	28076	1224	6	663	0	4	74	5	73	275.95	105	432	321	343	280	19	53	0	0	1
13062	7806	4740	1	1376	1046	28180	606	206	824	70	146	194	99	223	283.23	170	448.47	477	316	444	36	95	15	4	6
11828	7848	5202	2	1884	1426	28488	545	120	642	30	276	186	51	254	312.77	123	458.43	478	213	395	86	100	12	22	24
3994	13652	9888	916	318	194	28062	1264	4	577	0	14	69	1	68	272.06	92	424.65	293	275	280	18	43	0	2	3
4494	13288	9610	794	430	250	28088	1192	4	653	1	14	54	2	74	273.65	127	429.03	341	307	316	13	35	0	2	2
11222	7902	5468	2	2114	1566	28626	537	93	558	18	324	168	55	272	312.54	113	459.57	492	169	380	98	80	6	28	21
4526	13358	9596	807	362	220	28070	1194	8	660	1	8	60	5	82	275.01	104	429.46	329	320	320	8	32	0	0	2
5634	12394	8908	562	738	458	28174	1048	8	701	0	40	80	6	122	283.61	110	439.26	381	351	334	20	53	0	2	4
5818	12286	8798	553	756	474	28172	1046	18	693	5	40	76	10	122	281.2	105	438.67	384	349	347	15	50	0	0	4
14260	7708	4406	2	838	628	27898	648	325	1011	155	56	258	135	154	272.21	271	436.39	497	408	421	16	91	15	2	0
14314	7524	4340	9	968	706	27922	622	331	970	161	66	257	127	154	272.32	255	435.46	500	378	433	26	85	17	4	1
11336	7938	5440	2	2034	1496	28568	511	92	608	26	302	181	40	258	311.37	117	458.81	488	187	385	109	91	8	22	25
7044	11312	8052	343	1078	700	28270	919	33	754	9	82	97	17	149	287.21	132	445.52	426	376	358	36	58	2	2	4
11748	8088	5232	4	1768	1362	28448	580	112	690	21	224	163	61	222	299.18	150	454.59	522	235	400	69	100	6	24	17
13920	7410	4448	1	1244	902	28056	578	296	831	109	118	231	138	180	281.47	193	447.32	475	307	457	54	86	25	14	7
3886	13698	9954	994	334	180	28052	1318	7	553	1	0	64	4	81	270.94	89	423.16	263	257	260	6	41	0	0	3
13186	7750	4656	0	1360	1064	28174	586	198	852	70	144	202	92	221	283.23	200	447.51	480	294	449	45	101	19	14	2
3888	13702	9956	973	322	178	28052	1310	10	557	2	6	61	7	70	270.74	89	422.58	276	278	267	11	40	0	0	1
3866	13760	9994	1001	260	142	28032	1302	7	567	1	10	61	4	68	270.72	90	423.57	271	269	280	10	40	0	0	3
12546	7928	4994	3	1464	1126	28276	558	172	792	49	206	204	92	217	292.99	175	450.26	514	267	412	63	117	9	12	7
5246	12792	9206	644	542	306	28108	1116	7	715	2	14	75	4	90	278.57	109	436.28	370	375	328	13	54	0	2	3
4418	13406	9696	845	342	180	28046	1237	11	652	1	4	65	5	66	276.26	109	429.99	314	332	290	15	43	0	0	2
4198	13476	9758	902	386	240	28082	1248	5	569	0	24	63	5	73	275.97	75	431.4	305	287	293	15	46	0	0	2
5564	12464	8990	568	686	416	28172	1056	4	711	1	50	73	3	118	283.12	98	441.06	385	371	345	15	49	0	2	3
13854	7820	4604	6	922	674	27952	598	267	1029	119	72	239	112	178	276.41	230	443.14	457	399	473	39	103	18	6	3
11786	7896	5218	0	1854	1418	28474	574	121	649	28	284	180	52	258	304.55	120	456.49	456	218	392	93	95	12	18	16
5138	12880	9250	670	512	308	28112	1123	8	691	0	22	53	3	89	279.51	101	436.81	352	351	351	19	29	0	2	3
10702	7640	5508	0	2530	1968	28868	491	61	446	12	468	164	33	297	333.78	93	472.65	485	137	340	131	77	1	52	38
13366	7866	4710	1	1114	888	28102	604	223	924	76	138	191	101	184	283.28	218	446.27	487	338	462	44	92	12	20	5
11606	8010	5346	2	1834	1390	28484	548	116	635	19	266	172	67	276	303.95	111	455.71	503	219	379	72	94	5	26	13
6810	11668	8216	362	846	596	28224	923	27	760	4	82	89	16	144	285.58	132	442.53	429	388	378	22	46	1	4	5
14456	7546	4366	7	838	598	27876	647	356	1016	167	72	256	151	148	269.07	262	433.68	486	420	439	16	76	20	0	3
11958	7946	5130	0	1778	1366	28436	592	110	719	25	238	167	60	269	305.05	131	454.46	475	266	383	67	102	6	18	12
12944	7740	4830	3	1456	1058	28182	586	186	843	65	140	205	96	201	281.08	202	446.82	506	295	435	49	109	11	14	4
4128	13474	9804	905	426	236	28076	1293	2	598	0	6	60	1	74	272.39	75	427.92	282	314	271	15	42	0	2	1

13916	7734	4574	1	974	690	27964	638	289	1007	122	68	211	124	157	276.61	245	440.63	485	394	466	28	80	19	8	2
4006	13646	9900	954	306	176	28048	1272	8	563	1	14	57	6	72	270.7	82	423.23	276	263	297	16	35	0	0	3
4658	13212	9540	783	414	236	28076	1196	11	653	0	16	61	8	80	276.45	89	431.57	318	340	323	17	44	0	0	1
4282	13408	9738	881	408	224	28070	1247	7	598	1	8	63	3	70	273.93	91	427.73	317	299	281	12	43	0	2	2
4790	13010	9430	716	560	314	28120	1161	5	662	0	16	60	5	94	276.15	103	433.35	346	330	313	14	44	0	0	2
5376	12746	9150	646	508	294	28098	1087	10	711	2	22	78	6	90	281.15	111	437.78	367	357	341	23	50	0	2	3
4386	13400	9680	828	376	218	28072	1199	3	634	0	12	57	2	71	272.72	109	427.7	328	310	324	16	37	0	0	1
4854	13046	9416	712	496	282	28112	1152	3	683	0	18	63	1	81	277.28	120	434.83	362	340	319	17	40	0	0	0
4480	13200	9554	790	544	332	28130	1205	5	600	0	20	59	2	74	277.88	93	433.93	340	305	294	23	39	0	0	0
4046	13550	9838	925	402	232	28076	1266	7	561	1	8	60	4	63	272.33	99	425.69	310	273	277	16	39	0	0	2
12026	7770	5170	2	1794	1372	28454	497	125	679	27	282	193	68	260	304.38	130	455.91	498	217	401	88	111	6	36	18
4132	13554	9822	930	346	194	28054	1291	7	615	1	6	63	5	66	271.02	90	424.43	278	297	277	18	46	0	0	2
14360	7686	4382	5	788	596	27872	657	334	1053	167	56	260	122	149	267.31	276	429.26	489	440	418	15	80	21	4	2
4330	13406	9714	868	396	218	28072	1238	6	607	1	8	70	4	81	274.41	93	428.62	297	294	293	17	47	0	0	1
7808	10964	7698	294	1012	660	28226	882	47	824	12	80	109	25	142	291.34	148	447.93	455	403	371	25	58	2	4	7
6934	11528	8200	357	906	578	28226	957	25	803	7	78	79	12	120	288.13	142	443.73	452	415	355	24	54	1	2	2
12700	7724	4836	2	1572	1238	28322	592	186	731	58	242	197	91	250	292.45	173	450.01	502	278	384	48	87	11	10	5
11034	7748	5584	0	2232	1664	28718	504	88	523	11	414	170	46	263	331.26	102	472.01	472	173	355	132	77	4	34	42
12776	7852	4856	2	1446	1118	28238	586	180	811	57	176	189	94	224	282.29	182	448.09	494	290	442	45	99	12	12	4
6094	12260	8734	505	640	378	28134	1031	9	772	3	28	78	1	92	283.06	130	440.91	406	396	358	26	49	0	0	1
4412	13372	9678	856	378	214	28068	1211	7	621	2	12	61	4	74	273.32	101	428.68	320	300	314	16	37	0	0	0
13744	7642	4596	4	1118	810	28030	571	285	897	88	112	223	147	180	282.55	217	446.07	504	329	463	37	111	21	8	5
14474	7618	4358	3	786	568	27858	639	322	1078	161	54	269	127	140	270.83	259	434.13	480	438	441	26	96	17	0	2
14372	7642	4346	7	814	636	27886	627	330	1058	168	70	266	126	163	268.32	258	429.88	495	428	427	14	86	13	6	1
4022	13634	9896	943	318	172	28048	1271	9	573	1	6	52	4	66	271.73	89	425.53	282	268	305	18	28	0	0	1
6362	11886	8524	481	844	520	28196	1041	24	751	8	54	77	12	114	288.29	113	444.24	387	387	342	31	50	0	6	0
7052	11544	8164	353	862	524	28190	948	24	860	2	42	72	11	122	286.35	147	441.8	447	430	371	19	46	2	2	3
12882	7998	4896	4	1272	966	28158	625	213	868	76	132	181	104	219	282.32	174	445.96	459	332	452	41	86	17	12	3
12256	8000	5124	2	1562	1158	28294	566	153	792	40	166	197	76	227	295.05	172	448.25	490	264	428	62	111	13	28	10
6536	11852	8422	472	816	508	28178	1008	33	764	5	44	68	18	131	286.47	132	442.75	389	372	356	25	44	0	0	1
11350	7926	5492	2	1966	1464	28562	504	97	582	21	334	168	48	274	311.94	104	463.97	499	180	400	88	97	4	28	25
12778	7942	4898	5	1402	1044	28214	615	185	857	70	146	198	100	215	284.12	175	447.03	475	327	431	51	92	5	4	4
10630	7852	5652	0	2310	1840	28818	525	70	492	9	462	160	34	337	330.67	75	472.91	420	153	340	118	82	5	68	35
4706	13066	9486	726	548	300	28120	1154	2	630	0	12	61	1	89	278.56	94	434.01	343	325	327	15	40	0	2	4
4588	13246	9546	775	434	262	28092	1184	8	623	0	16	62	4	90	277.92	87	435.54	325	327	314	11	43	0	0	5
3976	13642	9914	946	336	180	28052	1285	8	590	1	4	68	6	58	270.81	101	423.43	302	287	269	12	48	0	0	3

4796	12980	9388	736	608	352	28134	1146	10	627	1	10	62	6	87	280.56	96	436.98	349	309	323	24	43	1	0	2
6148	11996	8632	474	862	520	28212	1022	24	749	3	52	75	13	127	286.33	132	441.32	412	385	330	18	55	0	2	3
4798	13046	9434	722	520	294	28106	1142	2	662	0	14	80	1	88	280.51	99	438.43	339	328	319	22	51	0	0	5
5290	12740	9182	614	560	322	28122	1123	13	743	3	24	77	8	89	279.33	133	436.05	372	388	311	19	52	0	4	1
13916	7840	4576	8	888	646	27924	613	303	1014	138	56	243	121	162	273.74	236	437.93	493	391	460	21	92	17	2	2
4356	13294	9626	851	490	316	28114	1225	8	576	0	30	62	6	89	275.11	79	431.5	304	280	299	15	45	0	2	4
4422	13324	9626	855	442	266	28094	1233	8	607	0	12	52	5	77	275.39	94	429.1	309	302	303	17	30	0	2	2
11860	7840	5192	0	1826	1432	28482	545	126	670	31	286	172	65	259	305.54	141	456.46	506	218	399	71	87	11	42	17
7438	11280	7934	316	940	574	28210	890	29	825	6	44	96	14	144	288.71	154	445.99	449	389	388	23	56	2	0	2
4450	13328	9646	865	402	232	28076	1233	8	626	1	18	62	5	93	274.66	93	429.41	293	299	299	11	45	0	0	2
4394	13340	9672	864	430	236	28080	1256	7	628	1	8	66	5	71	272.47	104	426.93	305	316	280	15	49	0	0	3
5126	12734	9218	683	642	388	28150	1127	6	661	0	42	58	3	98	279.01	92	436.14	359	330	331	17	39	1	0	3
4332	13494	9728	858	308	180	28052	1231	9	634	1	10	70	6	84	273.55	88	426.47	292	319	305	13	49	0	0	2
13474	7658	4566	1	1246	998	28110	577	250	832	100	150	228	126	220	278.37	175	447.25	468	298	458	38	101	13	18	5
11626	7890	5318	0	1954	1440	28522	496	109	623	20	256	162	54	254	308.85	143	459.12	523	185	402	102	90	7	36	12
4168	13486	9808	899	382	210	28070	1256	9	588	0	16	59	7	75	275.17	79	429.58	290	295	294	19	44	0	0	1
12802	7652	4782	0	1576	1236	28286	585	209	721	73	216	199	98	232	292.96	141	450.74	472	258	420	65	102	15	22	8
3692	13742	10026	991	380	220	28072	1301	4	516	1	12	54	1	72	270.89	87	423.37	278	251	273	15	33	0	0	1
13890	7858	4590	8	868	650	27928	652	300	1036	146	64	233	117	175	274.5	243	438.51	456	429	450	21	75	14	8	2
11818	7942	5066	2	1856	1510	28490	543	124	636	31	276	178	62	259	301.39	138	456.34	497	219	384	84	86	7	16	16
13352	7640	4568	3	1350	1070	28152	609	239	796	99	168	218	94	204	281.07	165	445.83	475	319	412	52	94	16	4	8
4424	13264	9628	809	486	282	28104	1199	5	610	0	20	60	4	77	274.66	93	430.37	333	308	307	16	44	0	0	3
4636	13120	9518	779	520	296	28110	1186	5	635	0	20	55	3	89	276.41	98	431.61	327	307	322	11	33	0	0	5
4006	13642	9896	963	314	180	28050	1290	5	581	1	12	57	3	74	270.51	87	423.53	278	272	281	11	41	0	0	2
13124	7782	4780	3	1316	1000	28178	627	213	802	82	162	205	97	229	281.88	155	448.52	451	323	418	49	90	14	12	2
4630	13218	9538	811	436	252	28086	1216	9	642	0	10	61	8	77	274.97	95	430.13	323	324	304	14	39	0	2	0
14090	7650	4448	3	980	716	27958	650	292	987	147	68	247	113	172	273.93	231	439.46	461	412	432	31	88	15	6	1
4442	13234	9608	800	510	300	28118	1185	5	600	0	24	62	3	87	276.55	95	433.11	328	292	316	18	43	0	0	1
13072	7794	4740	0	1358	1062	28204	580	196	835	73	154	184	81	188	283.8	198	447.29	527	294	452	45	89	17	24	5
11798	7974	5214	0	1816	1392	28470	543	119	654	32	242	164	63	259	304.84	136	459.28	491	229	405	81	89	6	34	17
14360	7670	4376	5	808	604	27872	634	332	1046	160	48	258	132	155	271.7	265	435.47	466	419	457	22	84	20	4	4
14426	7732	4394	6	714	528	27836	646	345	1087	172	42	261	134	157	267.6	277	430.04	464	444	450	16	80	19	0	0
4000	13726	9900	954	250	154	28036	1287	6	603	1	6	62	3	74	270.4	94	422.95	270	286	289	13	41	0	0	1
4828	13116	9430	715	446	262	28096	1147	1	679	0	14	72	1	77	278.72	102	436.15	353	346	324	17	49	0	0	4
5380	12628	9100	610	640	370	28142	1075	9	681	2	22	76	5	96	281.94	118	439.16	382	336	343	22	47	0	2	1
11048	8008	5524	2	2082	1596	28638	531	93	565	12	344	162	50	282	316.13	108	465.05	463	186	374	119	81	2	30	17

SUPPLEMENTARY INFORMATION:Monte Carlo Atomistic Simulation and Machine Learning Analysis of Na-K Eutectic Alloy in Condensed Phases, D. Reitz and E. Blaisten-Barojas, George Mason University, Fairfax, VA 22030

13682	7844	4616	6	994	758	27986	650	273	998	124	84	220	113	169	275.32	243	440.47	470	388	437	34	76	15	6	2
3770	13794	10032	1025	286	152	28036	1333	5	567	1	2	58	3	66	271.46	95	423.75	258	266	260	15	40	0	0	1
11678	8010	5304	0	1862	1374	28490	533	114	633	25	240	178	62	264	306.33	133	456.02	492	213	399	88	96	4	22	12
4130	13534	9830	913	348	200	28062	1255	6	585	0	18	59	4	80	274.37	83	429.71	278	285	306	12	46	0	2	6
14032	7614	4358	2	1058	836	27996	651	286	944	147	96	232	102	176	274.46	216	438.86	472	391	433	26	66	14	2	3
12520	7786	4910	0	1648	1262	28352	594	142	725	46	204	179	72	246	293.97	150	452.65	475	279	416	58	93	3	20	11
4352	13402	9702	849	382	218	28070	1234	7	596	0	14	61	2	73	275.83	94	430.59	299	304	300	22	38	0	0	2
4560	13142	9526	739	550	330	28132	1153	4	625	0	20	60	2	87	277.84	87	434.2	357	320	320	16	39	0	4	2
11518	7820	5382	1	2012	1490	28568	533	108	571	28	302	177	59	260	310.23	107	461.53	480	186	383	103	80	6	40	24
7184	11334	8036	330	988	618	28220	892	42	748	4	58	91	26	158	288.03	127	444.33	426	366	381	27	47	0	2	4
13884	7716	4534	4	996	748	27976	630	284	1005	143	94	232	106	176	273.86	236	438.1	481	411	446	24	78	15	4	2
11576	8038	5392	3	1838	1354	28480	555	110	642	19	264	158	69	264	302.18	146	455.75	495	215	412	81	83	3	16	6
10524	7958	5838	1	2410	1670	28770	508	50	496	6	322	182	28	279	334.21	82	471.67	459	158	362	134	85	4	38	33
3986	13660	9890	944	306	192	28052	1282	7	582	1	18	54	2	65	272.01	95	425.39	285	290	286	17	33	0	0	2
4236	13466	9760	901	372	216	28066	1266	9	606	2	16	58	5	75	273.33	100	426.14	303	294	281	7	42	0	0	3
4474	13386	9666	847	342	180	28050	1245	8	653	2	2	68	3	52	273.3	106	427.56	324	336	288	18	45	0	0	1
4564	13242	9564	809	454	258	28088	1201	6	626	1	6	65	4	80	276.3	99	431.17	320	305	309	18	46	0	0	2
14560	7570	4352	8	766	534	27830	633	349	1075	174	44	285	134	138	269.12	277	432.54	506	431	415	16	102	23	4	1
4912	12944	9358	720	558	326	28122	1152	10	654	0	24	56	6	87	277.88	98	434.28	343	333	335	21	42	0	0	1
3940	13698	9938	985	300	164	28044	1313	8	576	2	4	65	5	76	270.55	87	423.29	262	280	269	10	43	0	0	2
13606	7824	4668	4	1054	768	28010	613	284	957	119	80	236	129	169	276.06	231	441.72	494	378	437	40	96	20	8	0
5054	12982	9292	681	468	288	28100	1122	7	706	0	14	63	4	82	278.56	116	435.75	353	346	350	19	42	0	2	5
3974	13640	9896	947	344	198	28060	1303	8	569	1	8	63	6	66	271.98	79	424.27	272	299	271	19	38	0	0	2
4106	13402	9758	889	528	310	28116	1262	6	532	1	12	47	3	74	273.72	73	429.18	298	277	291	22	26	0	0	1
4374	13278	9692	819	482	252	28094	1200	6	602	0	16	51	5	68	275.63	94	431.92	326	297	325	19	35	0	0	5
6676	11684	8332	368	940	558	28226	988	19	785	2	34	61	13	127	288	117	442.66	434	437	358	18	37	0	2	1
4062	13610	9876	927	326	172	28048	1278	7	582	2	2	59	5	70	272.34	91	427.03	287	292	290	7	36	0	0	5
3936	13676	9948	968	308	164	28040	1320	8	587	2	8	60	3	64	270.7	79	423.77	258	300	270	18	40	0	0	3
5080	12814	9236	671	596	376	28144	1145	8	682	1	42	59	7	92	279.23	100	435.77	353	359	329	14	39	0	0	4
4814	13026	9420	713	526	308	28120	1123	6	660	0	26	59	4	91	277.68	105	433.04	364	323	344	12	38	0	0	3
7368	11140	7850	320	1086	738	28276	904	35	741	10	88	85	18	139	289.29	120	444.4	450	361	369	34	53	0	4	6
10790	7984	5650	1	2244	1654	28710	541	75	533	14	358	174	38	256	321.72	108	466.06	493	170	355	118	95	5	28	26
4036	13576	9876	939	358	200	28060	1274	5	590	1	12	64	2	72	270.15	96	422.52	291	271	279	13	50	0	2	2
4800	12988	9424	734	574	322	28128	1196	5	646	0	20	57	2	92	278.51	97	433.07	332	336	302	13	39	0	0	2
14298	7758	4434	6	762	564	27862	637	324	1073	162	44	255	119	149	271.24	274	435.49	491	441	442	16	82	20	0	1
5774	12452	8892	600	594	376	28132	1095	22	726	6	40	66	11	85	281.16	102	439.27	367	384	352	26	43	1	4	4

SUPPLEMENTARY INFORMATION:Monte Carlo Atomistic Simulation and Machine Learning Analysis of Na-K Eutectic Alloy in Condensed Phases, D. Reitz and E. Blaisten-Barojas, George Mason University, Fairfax, VA 22030

4004	13618	9902	954	334	182	28050	1279	7	581	1	10	55	4	68	269.97	85	422.24	286	276	285	15	39	0	0	2
11554	7774	5350	0	2000	1512	28564	584	111	622	19	334	177	52	283	309.91	112	459.57	459	229	336	90	99	13	38	22
5816	12252	8824	524	776	466	28176	1069	12	741	1	40	73	7	104	285.14	112	440.01	394	410	320	22	51	0	2	4
12742	7912	4886	2	1414	1092	28226	591	157	838	49	164	176	86	214	285.48	190	448.45	500	289	459	46	93	9	16	2
5050	12896	9324	715	532	288	28104	1156	8	676	0	12	70	4	95	278.6	91	434.9	342	350	315	12	46	0	2	3
4768	13102	9452	744	490	282	28108	1159	3	644	0	14	72	2	79	278.16	90	434.54	355	331	313	15	51	0	0	4
4116	13502	9814	913	396	228	28072	1273	4	568	1	16	58	1	80	273.03	73	426.17	272	289	290	20	39	0	0	1
13118	7862	4720	6	1248	1020	28146	604	210	881	73	164	199	90	209	283.01	222	447.45	498	315	429	38	94	21	14	2
4406	13276	9644	852	482	272	28094	1225	7	605	0	14	57	5	69	275.62	101	429.13	324	298	303	18	40	0	0	1
14282	7598	4404	4	890	656	27912	660	338	1001	160	78	261	141	162	271.05	243	435.63	481	431	408	18	90	18	4	2
13588	7802	4624	9	1066	832	28038	658	256	940	110	112	220	101	167	276.92	219	443.45	501	399	409	29	88	21	14	2
11328	7930	5382	0	2064	1560	28596	541	95	616	23	292	158	49	265	312.82	141	459.6	516	215	365	96	74	5	36	13
5320	12684	9134	656	616	356	28130	1122	8	704	1	20	75	6	85	279.28	114	436.44	362	351	326	25	54	0	0	2
14356	7572	4334	2	896	674	27906	625	307	1032	156	72	266	117	170	272.21	245	435.02	472	430	435	25	88	18	2	1
11798	8020	5226	1	1720	1378	28464	522	109	675	28	302	185	53	276	307.76	126	457.31	483	212	402	80	99	4	20	14
6116	12016	8590	489	886	564	28222	1012	18	680	2	48	66	11	114	285.28	107	443.72	413	342	350	26	37	0	2	5
13628	7956	4666	10	920	718	27980	631	266	1016	124	84	232	108	185	275.37	242	440.76	464	399	446	27	88	20	6	3
6114	12124	8636	463	782	498	28200	993	13	758	1	42	62	7	115	283.15	132	442.3	424	391	378	14	39	0	2	7
14226	7722	4378	9	846	670	27914	665	322	1033	162	70	252	127	143	270.19	268	433.6	489	431	423	25	80	23	2	0
4118	13522	9842	914	374	196	28060	1270	9	606	0	8	64	5	69	271.72	89	425.22	291	296	276	20	43	0	0	1
13176	7748	4644	1	1374	1074	28164	599	221	803	87	132	208	99	210	284.82	173	446.89	490	299	429	44	87	13	16	4
5366	12754	9184	660	502	268	28092	1138	10	733	0	18	54	8	84	277.58	109	432.86	347	368	351	18	39	0	0	1
13468	7652	4560	1	1320	1002	28132	635	229	819	87	118	216	104	218	281.09	188	447.8	460	309	410	38	98	22	10	5
11706	7746	5294	1	1960	1480	28550	560	106	592	26	322	170	52	260	308.75	105	457.16	484	204	380	92	88	10	38	20
11900	7904	5178	2	1824	1378	28450	555	112	666	33	236	186	48	241	309.39	139	457.75	473	227	390	107	92	6	26	10
11962	7840	5116	1	1842	1422	28470	605	122	629	33	252	167	58	252	300.77	134	454.37	476	226	378	79	77	10	30	13
3916	13616	9932	973	382	210	28070	1289	9	558	1	14	56	3	73	271.19	92	423.95	283	250	277	16	38	0	0	0
12188	8058	5204	5	1552	1116	28310	578	130	804	34	186	174	76	216	292.48	166	450.93	503	288	428	70	95	3	4	5
14224	7682	4486	9	832	594	27890	624	321	1037	147	70	246	134	138	273.95	273	438.55	516	409	448	18	88	19	2	2
4498	13352	9638	859	364	202	28062	1247	9	657	1	8	57	7	69	273.95	108	428.43	314	308	295	11	41	0	0	1
4094	13490	9820	906	418	240	28080	1256	4	590	1	18	55	2	71	273.48	91	427.32	294	286	296	20	36	0	0	2
12124	7834	5078	1	1764	1348	28412	549	141	707	35	236	183	71	247	300.9	161	451.77	502	233	406	80	101	16	24	7
14290	7652	4416	4	860	618	27894	603	327	1036	152	58	248	136	174	274.79	254	438.46	466	404	478	21	86	19	0	3
4212	13432	9766	890	422	234	28078	1264	8	606	1	12	63	4	64	274.5	97	427.55	310	294	274	17	38	0	0	2
6806	11572	8258	380	940	580	28214	944	23	774	6	52	80	13	132	287.59	115	444.09	415	392	377	31	55	1	6	6
3810	13740	9982	1009	326	188	28052	1333	8	559	1	6	62	4	62	269.68	86	422.55	270	261	252	15	42	0	0	1

SUPPLEMENTARY INFORMATION:Monte Carlo Atomistic Simulation and Machine Learning Analysis of Na-K Eutectic Alloy in Condensed Phases, D. Reitz and E. Blaisten-Barojas, George Mason University, Fairfax, VA 22030

12396	7980	5052	2	1540	1138	28290	584	149	794	45	180	188	80	233	292.54	157	451.63	486	289	429	55	102	11	2	7
6566	11760	8386	401	890	556	28212	961	27	743	6	46	74	17	122	287.69	123	444.44	446	394	370	18	41	1	8	2
6942	11498	8126	380	918	650	28242	915	28	786	7	98	81	16	138	289.15	155	445.09	452	369	372	28	47	0	10	1
5718	12294	8830	574	808	504	28194	1093	15	675	4	38	78	9	104	285.3	120	440.82	401	357	299	17	43	0	2	2
6256	12012	8570	528	782	500	28176	1045	27	720	6	54	79	18	111	286.01	109	443.58	384	367	347	26	49	1	2	3
4258	13408	9732	872	416	248	28086	1256	6	602	2	22	64	3	71	274.46	83	428.18	309	298	277	17	40	0	2	1
3920	13650	9912	963	350	214	28064	1297	7	550	1	18	71	3	65	270.19	97	423.38	289	268	255	15	39	0	0	3
5790	12078	8816	537	924	544	28216	1054	16	667	3	62	76	7	119	286.93	113	441.4	397	325	313	25	53	0	2	3
12486	7990	5000	6	1484	1128	28270	577	160	797	52	164	173	90	227	283.3	151	447.35	476	301	466	54	93	5	14	8
4106	13528	9856	926	364	190	28056	1271	11	580	2	12	57	6	71	271.64	86	423.86	286	284	292	16	44	0	0	1
13802	7892	4562	6	902	714	27952	627	276	1016	130	74	234	109	183	274.38	251	438.48	456	388	464	26	89	17	6	2
14298	7586	4338	4	930	698	27918	663	327	1004	164	66	253	124	151	270.34	255	433.01	494	423	411	15	80	19	2	5
5856	12486	8870	556	560	332	28120	1061	13	802	0	16	73	9	110	282.65	122	440.84	374	395	355	18	56	0	0	0
11374	7924	5356	0	2050	1554	28592	528	94	567	22	306	174	52	273	312.08	116	457.72	488	176	390	96	86	6	26	20
12236	7924	5046	3	1638	1278	28364	553	137	743	52	204	199	72	242	295.61	148	453.56	497	248	401	74	108	3	36	9
14218	7626	4444	7	916	638	27894	632	310	1042	168	52	265	101	157	270.28	257	431.9	486	419	430	24	82	16	0	0
4342	13414	9702	874	378	220	28070	1247	8	614	1	14	60	3	77	272.98	87	426.1	293	309	298	17	39	0	0	2
12468	7828	4978	1	1626	1216	28324	559	163	768	53	184	179	87	232	291.35	160	449.89	509	278	440	51	79	7	20	11
4838	13060	9424	728	492	274	28096	1178	9	666	0	8	56	4	69	274.95	104	429.5	360	358	311	12	43	0	0	4
14446	7610	4356	7	812	588	27866	656	340	1042	168	52	262	125	127	270.74	272	434.55	497	419	425	26	79	23	2	1
4058	13600	9874	906	330	180	28050	1265	6	608	1	8	63	4	69	271.65	91	424.55	302	298	277	13	41	0	0	1
3872	13666	9956	1003	364	196	28058	1309	9	546	1	4	61	5	67	270.68	100	423.89	273	245	270	16	38	0	0	1
10936	7818	5622	1	2228	1684	28760	499	75	531	13	420	160	37	309	330.14	96	473.14	474	155	368	109	88	4	44	27
13502	7726	4564	5	1208	954	28090	618	219	906	92	124	232	100	184	278.57	205	444.37	496	340	421	35	109	15	12	7
4338	13436	9702	860	368	214	28066	1232	6	615	1	8	60	3	64	273.61	93	427.71	323	300	300	14	42	0	0	2
12732	7720	4846	3	1594	1200	28298	596	181	732	50	190	192	101	229	293.4	135	449.6	469	284	429	64	95	9	14	6
3830	13726	9980	991	322	184	28052	1317	10	542	2	10	54	7	67	270.1	79	422.61	267	271	276	13	32	0	0	2
4678	13200	9536	777	416	234	28082	1181	6	651	1	18	66	2	82	278.13	98	433.93	312	327	331	22	42	0	0	3
11186	7744	5374	0	2246	1728	28684	515	94	514	14	348	148	50	292	319.1	110	461.73	485	176	385	101	71	10	56	28
4288	13370	9704	846	464	260	28092	1222	6	587	1	6	60	2	89	274.66	88	429.59	303	287	306	15	40	0	0	1
11070	7864	5504	0	2186	1654	28676	509	70	561	17	344	162	38	271	316.35	104	465.59	470	176	410	108	82	2	50	34
4846	12874	9384	710	630	368	28146	1163	11	625	0	44	56	8	87	278.38	84	435.63	350	340	315	18	37	0	0	4
10504	7940	5676	1	2310	1866	28840	474	56	486	6	488	153	26	303	330.42	95	472.61	489	155	361	130	78	4	56	33
10592	7888	5676	1	2390	1808	28802	504	73	496	9	376	160	36	306	332.68	91	471.75	450	149	370	122	85	5	62	37
6316	11802	8524	459	956	572	28230	1007	19	704	3	56	84	11	117	287.77	115	443.96	419	369	330	28	49	0	4	4
5730	12376	8894	534	716	418	28160	1058	14	732	0	22	65	11	103	281.57	107	436.95	403	390	349	16	43	0	4	0

SUPPLEMENTARY INFORMATION:Monte Carlo Atomistic Simulation and Machine Learning Analysis of Na-K Eutectic Alloy in Condensed Phases, D. Reitz and E. Blaisten-Barojas, George Mason University, Fairfax, VA 22030

4680	13030	9474	725	610	334	28138	1155	8	601	0	10	59	6	88	278.89	90	434.37	348	307	327	19	45	0	0	2
4884	12958	9378	673	556	324	28128	1114	5	655	0	28	62	3	79	277.23	106	434.76	378	345	340	17	35	0	0	5
5120	12844	9234	633	586	342	28140	1135	7	706	0	14	67	4	81	277.72	115	434.62	378	395	311	18	49	0	0	3
4388	13374	9682	855	390	222	28070	1220	12	617	1	14	65	6	79	274.48	108	429.63	325	294	289	11	42	1	0	1
14406	7778	4394	4	682	530	27838	655	318	1141	161	42	257	118	144	269.5	292	432.23	483	450	436	13	88	18	6	2
4184	13426	9740	900	454	276	28098	1259	6	562	1	16	62	4	74	273.31	95	427.58	302	274	282	16	44	0	2	3
4242	13378	9728	868	462	266	28092	1264	6	593	2	14	56	2	73	275.75	96	430.52	301	307	279	16	32	0	2	3
4064	13598	9860	903	342	190	28060	1258	8	585	2	6	62	2	77	272.37	82	426.97	297	288	289	10	39	0	0	2
4374	13360	9684	849	412	230	28070	1226	5	622	0	10	68	2	67	273.04	105	427.52	320	309	294	17	44	0	0	3
4672	13276	9512	795	368	232	28072	1206	10	677	2	12	55	6	72	275.3	97	430.78	313	344	327	21	38	0	0	1
4068	13516	9838	944	404	232	28076	1290	7	561	1	18	57	4	82	272.49	83	425.6	273	267	279	9	45	0	0	5
12148	8006	5136	1	1644	1220	28360	550	137	740	37	186	192	64	224	295.71	165	457.73	512	254	397	73	111	7	16	14
4826	13030	9432	753	518	284	28102	1151	9	645	1	12	69	7	86	277.85	111	435.13	347	303	320	18	50	0	0	2
13056	7794	4812	1	1362	998	28174	612	181	868	55	144	204	89	199	287.16	175	447.55	490	330	419	50	124	19	8	6
4874	13038	9400	745	486	284	28102	1182	7	673	0	20	59	2	88	279.27	99	437.55	338	354	314	12	41	0	0	1
4554	13148	9532	765	542	326	28128	1187	0	625	0	24	64	0	85	275.76	93	432.45	336	321	306	18	46	0	2	2
5096	12846	9278	672	564	316	28118	1133	11	669	1	18	63	6	86	279.32	94	436.79	360	342	335	16	42	0	0	3
12928	7978	4890	6	1282	956	28174	659	179	899	51	126	169	99	178	282.57	196	445.42	495	353	434	45	96	13	14	3
4122	13498	9812	918	402	228	28076	1294	6	579	1	14	57	3	64	272.06	72	425.04	287	299	276	16	39	0	0	3
12454	7994	5070	2	1460	1090	28262	589	161	819	37	178	186	91	236	291.97	179	449.94	469	304	425	57	110	9	16	9
11642	8148	5228	4	1808	1412	28478	583	107	699	22	198	164	56	246	305.36	139	454.97	496	267	380	87	90	6	38	7
4224	13542	9778	886	314	184	28050	1251	5	632	1	6	62	3	64	272.61	103	425.57	309	304	296	11	41	0	2	3
14286	7674	4396	10	856	624	27882	633	331	1032	172	44	260	121	160	268.58	270	432.69	490	412	433	15	78	17	2	1
4292	13382	9726	846	436	236	28080	1212	9	601	0	8	59	7	69	274.37	106	429	322	284	313	21	36	0	0	0
5498	12486	8978	599	712	450	28168	1091	9	689	2	42	62	6	106	282.03	102	439.19	371	341	345	19	45	0	0	0
14330	7698	4346	4	810	640	27888	660	322	1063	163	60	257	121	160	267.85	257	429.92	473	440	427	14	79	16	4	3
12700	8006	5022	3	1368	974	28214	676	155	869	44	134	182	77	196	287.51	184	447.02	478	335	395	48	104	14	10	5
6580	11672	8388	391	956	572	28224	971	26	763	2	52	73	14	134	286.58	119	445.88	427	404	357	22	47	2	4	2
14232	7726	4378	6	846	660	27900	611	318	1040	162	52	263	124	160	269.51	255	430.45	504	410	443	17	95	15	6	0
4662	13110	9506	758	512	298	28116	1185	8	634	0	28	66	4	90	277.02	92	433.01	328	336	302	18	52	1	0	3
10526	8004	5678	1	2360	1796	28790	525	61	489	8	370	158	27	296	324.09	89	468.24	479	155	337	126	75	3	54	24
3956	13642	9904	947	352	200	28060	1286	4	565	1	6	53	3	65	270.47	82	422.23	287	274	285	13	32	0	0	4
14270	7632	4390	7	894	658	27908	667	336	998	157	62	252	141	152	270.52	245	432.93	488	415	417	17	86	24	2	1
6774	11622	8278	399	916	566	28210	952	33	763	10	50	88	11	145	286.68	126	444	407	390	360	30	53	1	4	2
14254	7740	4458	2	784	574	27864	623	317	1091	158	54	266	120	156	269.37	261	432.59	490	430	440	16	97	18	0	2
6662	11674	8314	373	908	594	28230	957	26	786	7	70	74	15	122	286.46	120	444.93	436	408	374	26	48	0	8	4

SUPPLEMENTARY INFORMATION:Monte Carlo Atomistic Simulation and Machine Learning Analysis of Na-K Eutectic Alloy in Condensed Phases, D. Reitz and E. Blaisten-Barojas, George Mason University, Fairfax, VA 22030

4736	13058	9448	745	546	316	28120	1170	7	623	1	14	70	4	90	278.05	90	434.31	338	320	306	20	45	0	2	1
12498	7828	4938	4	1604	1232	28326	550	145	748	33	212	189	79	239	293.83	168	451.28	494	241	428	64	115	11	14	10
5106	12786	9228	654	634	374	28152	1113	3	687	1	22	67	1	92	279.96	120	437.76	377	342	328	18	43	0	2	2
12676	7844	4842	0	1520	1198	28288	573	181	763	49	190	187	97	219	290.05	175	450.31	484	255	433	68	96	11	18	9
13858	7750	4510	6	1002	770	27980	619	283	966	121	84	228	119	166	275.46	242	442	476	379	458	36	89	18	6	3
5078	12874	9296	658	556	302	28116	1117	7	687	0	10	73	6	76	279.29	115	436.67	379	336	325	22	51	0	0	2
13164	7902	4762	0	1210	930	28100	594	218	904	95	128	221	96	197	277.13	201	442.31	496	353	443	31	108	6	4	8
4910	13012	9380	709	492	290	28108	1150	4	673	0	24	58	1	89	278.03	104	434.38	346	352	334	16	39	0	0	1
11752	7934	5248	0	1846	1410	28490	540	105	647	25	274	182	53	259	304.38	127	456.45	503	228	382	89	103	2	22	11
4374	13304	9678	845	458	256	28090	1213	4	614	0	20	61	4	84	274.9	92	430.59	318	288	303	13	42	0	0	2
5272	12724	9162	605	600	356	28144	1088	5	717	0	26	63	1	107	282.07	111	438.27	361	369	348	19	46	0	4	4
14264	7710	4418	2	806	616	27884	657	322	1069	161	68	253	120	150	269.57	265	431.33	471	453	437	23	78	21	2	2
11342	7916	5374	2	2040	1560	28588	528	91	607	21	338	164	48	262	310.97	116	459.23	505	202	399	88	85	9	18	21
5348	12526	9114	589	720	410	28158	1047	11	689	3	34	74	5	106	284.29	106	440.65	391	350	347	20	49	0	6	3
7808	11010	7662	322	976	670	28208	856	64	756	20	80	116	36	167	288.32	139	447.63	427	346	381	31	60	1	2	5
14544	7618	4306	4	730	576	27848	628	350	1084	171	68	270	132	148	268.24	282	429.25	487	429	438	17	89	26	6	4
14386	7656	4414	6	778	564	27860	649	343	1058	156	58	260	143	149	272.94	268	436.25	477	441	437	20	88	27	4	1
6742	11566	8266	402	982	612	28232	937	32	715	5	64	89	18	133	287.54	119	445.06	439	353	353	32	52	0	0	2
11422	7924	5292	0	2024	1590	28606	545	79	610	24	314	164	31	280	315.02	119	463.25	463	213	384	96	85	3	36	27
4440	13286	9630	822	462	262	28092	1245	6	625	0	12	52	3	71	275.49	99	429.25	319	328	285	15	38	0	0	2
4770	13034	9472	720	514	288	28110	1147	5	657	0	32	58	3	86	277.96	82	433.81	347	335	329	19	38	0	0	3
11738	7848	5328	3	1924	1380	28504	551	110	650	21	250	162	57	248	316.44	137	462.75	491	207	398	94	90	9	34	16
14236	7674	4418	5	870	640	27898	661	325	1020	166	58	250	121	165	271.79	243	434.19	463	439	433	18	75	23	2	2
4436	13274	9624	810	484	272	28098	1217	4	618	1	8	63	1	82	273.92	86	428.4	320	317	298	14	39	0	0	2
14360	7724	4410	7	742	560	27854	640	328	1097	161	56	252	127	144	269.13	285	432.8	498	454	448	10	82	20	2	2
13374	7712	4622	5	1292	992	28138	616	229	836	79	136	204	109	194	282.37	190	445.16	494	332	429	41	87	20	10	4
4268	13484	9766	876	352	186	28058	1240	6	613	1	0	62	3	76	272.55	94	426.29	298	304	307	15	41	0	2	0
12718	8050	4896	2	1298	1070	28236	621	185	849	47	190	187	103	211	288.5	201	450.17	486	308	418	47	106	15	14	9
14078	7714	4428	8	912	722	27942	624	315	993	140	78	240	132	180	273.02	226	436.83	451	402	460	32	82	22	8	2
12416	7972	4958	0	1538	1222	28324	576	148	765	40	194	177	75	219	293.34	162	451.61	484	258	435	70	95	8	24	13
12058	7984	5026	6	1712	1378	28412	594	136	679	41	234	177	64	250	295.45	144	453.59	506	257	375	66	90	10	16	4
14352	7708	4372	10	788	598	27868	656	324	1067	159	48	255	123	153	268.71	274	430.08	491	447	422	12	86	23	2	0
3974	13676	9928	958	298	160	28042	1303	9	583	2	6	60	5	62	270.39	95	423.07	276	288	277	14	37	0	0	3
4934	13016	9384	700	494	268	28102	1127	7	675	1	6	83	5	85	278.48	105	434.46	367	340	314	16	54	0	0	3
6960	11478	8124	364	964	640	28242	962	33	774	6	64	82	20	129	288.61	113	444.36	431	406	357	27	45	0	12	3
11058	7838	5500	0	2206	1672	28684	524	84	536	21	372	179	39	290	318.72	101	463.44	455	178	369	112	83	3	28	32

SUPPLEMENTARY INFORMATION:Monte Carlo Atomistic Simulation and Machine Learning Analysis of Na-K Eutectic Alloy in Condensed Phases, D. Reitz and E. Blaisten-Barojas, George Mason University, Fairfax, VA 22030

8114	10820	7498	310	1014	698	28234	880	66	781	15	80	103	40	147	289.35	147	445.68	458	370	370	26	58	2	10	4
4482	13232	9606	786	486	278	28104	1194	6	606	0	18	67	4	91	275.86	77	432.52	302	324	311	26	43	0	2	2
14428	7738	4390	4	692	532	27836	653	330	1130	167	56	270	120	146	268.03	285	429.21	483	464	427	14	91	20	0	1
12566	7730	4792	1	1648	1358	28364	555	178	723	53	240	187	88	247	290.59	163	452.06	515	242	410	52	103	14	24	10
11186	7860	5518	2	2148	1568	28648	489	92	529	18	326	170	49	279	321.34	107	463.37	485	157	393	112	88	4	42	27
12958	7762	4896	3	1402	1008	28204	618	183	842	50	162	209	86	235	292.23	158	449.34	446	310	411	55	124	17	16	5
4934	12918	9350	744	564	326	28116	1189	15	618	4	24	60	9	98	280.51	100	435.81	317	326	310	15	38	0	0	3
4592	13164	9510	767	516	316	28120	1182	10	609	1	22	53	6	83	276.77	91	432.96	340	314	321	15	32	0	0	2
6118	12018	8654	493	846	510	28200	1011	27	686	7	52	71	16	128	284.54	115	442	405	342	352	22	43	0	2	0
6878	11660	8228	406	850	532	28188	949	34	769	4	40	79	24	120	285.28	136	442.93	426	360	385	29	55	2	0	2
4960	12934	9348	726	542	310	28116	1178	6	676	0	22	61	3	96	279.41	100	436.81	331	355	306	14	45	0	0	3
6780	11508	8230	396	1010	644	28248	925	31	675	7	66	95	19	136	289.63	113	443.77	430	335	365	31	54	0	10	9
4836	13032	9412	724	516	298	28112	1157	7	661	0	16	62	6	83	277.01	104	434.01	345	337	328	19	40	0	2	2
13836	7898	4534	2	864	724	27962	595	289	1022	138	106	237	119	157	274.58	276	439.33	511	386	457	27	84	11	0	5
14272	7630	4468	5	852	590	27874	606	335	1066	176	60	270	127	166	269.22	261	432.47	488	426	447	16	84	17	2	2
4244	13522	9790	903	312	168	28044	1246	6	621	1	8	59	1	72	272.35	98	425.05	297	294	306	11	39	0	0	3
12018	8016	5156	1	1674	1288	28400	578	121	736	29	224	172	71	236	299.94	166	454.68	510	251	405	65	100	6	24	11
4242	13436	9736	895	400	244	28080	1275	7	593	2	20	57	4	68	272.91	98	426.8	300	300	277	15	34	0	2	2
13458	7794	4712	9	1152	830	28054	626	271	913	114	104	221	120	171	277.02	223	443.75	509	357	426	33	79	16	4	2
13774	7630	4510	0	1150	870	28054	610	262	896	99	106	235	114	194	277.31	201	446.11	503	345	418	24	118	24	14	4
10642	7828	5624	0	2348	1866	28840	488	68	457	11	460	142	37	326	333.02	86	469.21	494	138	357	103	71	1	68	37
5406	12600	9078	573	660	386	28158	1091	2	733	0	24	67	1	93	282.22	109	437.36	406	389	316	14	47	0	4	2
14338	7650	4398	7	814	610	27876	629	340	1025	162	48	253	130	160	272.05	238	435.46	468	417	453	24	75	21	18	2
12028	7926	5146	0	1758	1320	28418	547	127	667	23	204	169	67	258	304.43	142	454.21	510	219	404	63	93	10	30	11
14288	7636	4370	1	868	668	27908	665	300	1045	158	72	266	110	164	269.04	258	432.09	474	429	405	19	100	15	4	1
3962	13634	9916	975	348	190	28058	1310	6	573	2	8	50	3	61	271.74	101	424.27	277	273	283	13	34	0	0	3
3846	13668	9946	968	364	224	28070	1287	7	535	1	20	69	5	66	270.66	88	424.76	286	256	268	17	38	0	2	4
4942	12958	9354	683	532	312	28124	1116	2	682	1	24	64	1	83	280.44	117	436.62	375	333	338	19	47	0	2	1
4410	13466	9704	875	292	162	28040	1248	8	660	2	6	66	5	75	273.09	93	427.66	292	324	304	12	47	0	0	1
4252	13434	9740	891	406	232	28074	1263	7	611	2	8	62	4	77	273.75	96	426.66	291	300	288	14	37	0	2	2
5026	12872	9294	677	564	338	28126	1116	9	687	1	32	57	5	86	278.65	114	436.03	368	334	340	22	42	0	0	1
5168	12728	9192	622	664	384	28154	1129	2	673	0	14	71	2	88	279.37	97	436.18	359	374	326	22	51	0	4	4
11972	7958	5214	3	1736	1272	28384	582	132	715	34	216	166	68	253	298.35	157	451.25	475	245	417	68	90	8	16	9
4248	13422	9774	868	408	208	28066	1252	9	584	1	6	66	6	80	273.06	73	426.09	285	312	291	18	48	0	0	2
4050	13548	9848	940	394	222	28070	1297	10	561	1	6	58	7	59	273.1	93	426.91	300	275	263	12	37	0	2	4
13416	7664	4554	1	1298	1048	28156	595	232	833	83	158	202	111	227	281.66	178	446.16	449	326	457	47	85	16	18	5

SUPPLEMENTARY INFORMATION:Monte Carlo Atomistic Simulation and Machine Learning Analysis of Na-K Eutectic Alloy in Condensed Phases, D. Reitz and E. Blaisten-Barojas, George Mason University, Fairfax, VA 22030

14298	7680	4376	2	836	640	27894	640	305	1054	155	58	257	118	164	270.43	266	434.89	486	411	428	15	91	14	6	1
14080	7724	4468	2	880	688	27934	624	325	1009	156	82	248	137	178	273.48	227	438.04	465	425	453	24	84	14	12	2
4426	13354	9640	843	404	242	28080	1254	8	625	1	14	54	5	73	273.81	95	427.58	307	328	293	13	36	0	0	1
11690	7942	5260	1	1820	1442	28494	552	102	696	25	310	168	54	253	301.37	136	459.42	500	254	384	94	89	3	24	7
12108	7884	5126	0	1762	1292	28396	542	146	709	35	198	163	73	257	302.76	148	455.47	509	237	413	57	72	8	26	13
10848	7930	5540	2	2236	1746	28746	536	59	540	9	408	150	34	283	324.68	105	464.08	491	178	361	120	79	0	38	22
4276	13392	9732	868	422	238	28076	1242	7	619	1	16	64	4	72	273.96	92	427.36	312	292	286	13	51	0	0	4
13936	7702	4488	5	1002	750	27956	622	294	970	153	74	253	103	171	273.48	228	438	472	393	435	34	83	12	4	3
14484	7470	4278	4	926	680	27912	641	340	989	160	68	252	126	154	272.87	245	438.4	492	409	426	21	80	21	6	3
5718	12428	8924	576	648	386	28142	1076	19	708	3	38	62	11	119	281.41	95	437.92	361	370	355	15	40	0	0	2
5012	12860	9318	681	584	328	28126	1131	8	677	1	24	58	5	79	278.18	95	434.35	374	342	333	15	38	0	0	4
4902	12992	9384	706	510	294	28102	1145	4	674	0	18	71	3	85	277.36	99	435.71	348	354	322	19	51	0	2	3
4532	13248	9610	807	442	238	28082	1200	8	632	1	12	65	6	67	274.39	100	428.63	341	320	305	18	44	0	0	0
5778	12382	8858	585	670	418	28144	1069	23	699	5	36	79	17	107	284.48	97	439.87	377	359	343	19	52	0	2	1
5416	12588	9056	595	676	396	28152	1099	8	731	1	20	66	5	100	280.4	121	437.58	379	379	333	18	47	0	0	0
11494	7942	5384	1	1964	1436	28516	557	110	631	24	282	176	61	257	306.14	102	458.07	458	227	408	94	98	9	14	20
4622	13180	9530	781	470	276	28098	1181	7	654	0	20	54	5	95	274.96	105	432.35	323	318	325	13	35	0	0	1
7044	11358	8030	351	1054	710	28282	924	28	742	8	78	76	16	122	288.31	133	444.14	464	372	365	35	48	1	8	4
11934	7832	5186	1	1834	1388	28474	561	127	669	35	270	181	57	274	299.25	106	454.46	465	238	395	81	94	12	24	13
14144	7674	4406	7	878	720	27938	621	306	1007	143	110	251	123	152	272.75	256	436.4	507	408	436	25	97	22	6	2
14398	7554	4308	4	884	680	27908	643	319	989	170	82	257	114	148	271.44	267	435.96	512	390	413	18	79	20	2	0
4320	13318	9684	848	478	278	28100	1214	2	585	0	22	63	1	86	274.79	85	429.25	325	280	295	10	45	0	0	1
4080	13524	9836	933	400	222	28070	1282	8	576	1	6	59	4	74	271.37	94	423.95	285	281	281	12	37	0	2	3
4678	13160	9522	772	458	256	28090	1194	10	668	0	16	55	6	91	277.49	91	433.36	304	336	327	19	41	0	0	2
11948	7874	5212	1	1810	1320	28412	546	140	683	39	222	162	71	239	293.27	140	451.87	518	236	425	69	85	13	26	11
3598	13886	10110	1044	280	158	28038	1341	5	532	1	6	58	1	70	270.35	75	422.39	245	250	261	13	35	0	0	4
14206	7704	4468	5	822	610	27884	641	329	1046	165	72	264	125	166	271.14	255	434.09	477	435	425	16	88	16	2	2
5506	12578	9034	590	640	366	28140	1080	14	724	0	16	56	13	87	281.73	112	437.87	390	384	364	19	37	0	0	1
3876	13652	9934	958	378	218	28068	1298	8	534	1	10	63	5	61	271.2	91	423.65	288	268	265	16	37	0	0	4
13610	7810	4614	6	1094	806	28012	615	260	950	119	72	221	119	164	276.4	224	440.49	500	373	448	34	82	8	6	4
14192	7708	4448	4	846	632	27894	681	309	1049	154	66	255	115	154	269.06	250	431.71	462	452	416	22	88	15	2	2
4946	12952	9382	709	522	284	28106	1158	9	682	1	20	68	7	83	279.16	105	435.68	341	360	322	19	46	0	0	4
10986	7822	5522	1	2266	1698	28700	531	79	509	17	362	167	44	289	322.65	95	461.96	462	167	376	107	76	6	42	31
6266	11982	8580	517	812	492	28176	1035	18	735	9	42	85	7	103	285.44	132	443.01	404	364	337	22	53	0	2	7
14586	7608	4276	3	736	578	27850	654	350	1095	179	62	268	131	138	267.87	301	429.39	490	442	423	16	84	16	4	4
14126	7788	4474	1	810	628	27900	642	291	1096	154	74	262	108	150	270.37	287	433.2	503	433	421	18	96	17	0	0

SUPPLEMENTARY INFORMATION:Monte Carlo Atomistic Simulation and Machine Learning Analysis of Na-K Eutectic Alloy in Condensed Phases, D. Reitz and E. Blaisten-Barojas, George Mason University, Fairfax, VA 22030

10644	8020	5640	1	2260	1758	28766	510	47	504	8	398	161	23	319	324.34	104	464.23	472	144	344	121	82	6	42	23
4788	13054	9474	740	496	268	28102	1164	7	644	1	22	66	4	80	276.53	100	432.42	348	328	318	17	49	0	0	2
5498	12562	8992	552	634	420	28160	1066	10	706	1	54	56	6	117	285.23	104	441.24	357	370	368	23	34	0	0	3
3882	13742	9984	996	276	146	28036	1322	7	590	2	6	63	3	64	270.57	105	422.97	269	282	262	11	41	0	0	3
11296	7904	5456	1	2060	1530	28594	537	77	593	9	314	161	41	273	316.73	132	461.98	484	184	375	94	99	6	26	28
5826	12374	8836	551	676	410	28148	1045	13	714	2	26	76	8	107	285.08	121	441.98	397	352	350	19	47	0	0	0
4088	13590	9848	941	336	188	28056	1272	9	578	3	6	69	5	74	274.81	82	426.96	291	283	276	13	47	0	0	1
11460	7984	5358	0	1936	1480	28544	551	103	618	21	298	177	54	252	309.89	122	460.43	466	204	387	107	86	7	28	22
13060	7770	4780	2	1368	1030	28176	589	221	818	84	162	221	97	208	283.81	183	447.17	471	288	421	60	104	20	6	9
4446	13302	9616	811	452	266	28094	1226	6	612	2	12	64	2	83	275.34	87	431.31	316	329	288	15	42	0	0	1
5384	12584	9098	637	640	384	28136	1121	18	704	5	46	72	8	106	282.86	90	438.94	335	377	332	23	45	0	0	3
4680	13200	9494	763	446	268	28100	1198	6	664	0	10	58	5	66	277.89	103	434.29	340	344	314	17	43	0	2	4
6292	12028	8566	475	754	484	28182	991	13	783	1	52	89	5	128	284.13	111	442.34	402	391	357	24	61	1	6	2
4152	13558	9808	922	330	196	28056	1288	6	636	1	10	58	5	59	271.39	107	425.21	292	306	282	12	36	0	2	5
4376	13350	9660	831	438	256	28092	1209	5	610	1	10	55	2	78	274.45	95	429.77	315	301	319	18	35	0	2	2
10368	7908	5854	1	2414	1794	28838	487	55	474	9	456	156	30	303	331.04	93	470.74	493	146	350	126	72	2	44	35
4750	13066	9476	768	524	278	28100	1189	6	667	1	6	72	4	86	277.85	103	433.89	335	326	297	15	47	0	0	1
4400	13262	9622	792	512	300	28112	1198	7	600	0	16	58	3	75	274.33	96	429.58	339	302	303	17	37	0	0	3
13640	7846	4626	6	1016	774	28000	614	277	978	122	96	230	121	171	276.37	231	439.77	498	375	447	30	94	14	2	1
4418	13264	9600	835	500	310	28116	1232	6	591	0	24	62	4	86	275.86	92	429.15	322	296	277	12	40	0	0	2
5230	12792	9204	680	552	314	28106	1139	14	669	4	14	70	8	83	281.19	103	437.19	365	361	320	14	41	0	0	3
3902	13672	9980	994	324	158	28042	1331	6	581	0	6	56	3	69	270.86	84	422.78	263	289	261	10	38	0	0	2
4984	12830	9264	674	652	400	28160	1137	7	633	1	30	56	4	98	279.56	88	436.41	360	335	323	16	35	1	0	3
14260	7552	4334	6	972	738	27938	618	323	963	154	76	251	134	159	271.89	237	434.28	502	394	434	27	83	16	6	0
4052	13580	9862	930	352	204	28068	1276	5	585	1	18	61	2	77	271.68	97	424.62	292	267	277	12	38	0	0	0
14292	7600	4356	4	902	690	27924	619	308	1014	150	80	260	125	163	273.18	261	438.92	486	385	439	26	99	12	4	2
5572	12468	8950	599	690	434	28154	1086	10	703	3	36	71	5	106	283.38	106	441.11	377	353	327	21	46	0	4	1
12902	7904	4908	7	1292	982	28162	610	216	902	70	152	193	109	224	281.99	171	446.78	456	340	455	41	99	20	20	5
6736	11640	8278	426	918	580	28208	957	33	749	10	54	74	16	120	287.43	129	442.61	426	376	375	35	42	0	2	0
14426	7742	4406	2	700	518	27836	653	322	1122	156	40	255	128	136	269.35	284	432.21	484	466	448	16	90	20	4	2
4268	13490	9746	879	344	202	28060	1240	7	619	1	8	58	4	77	273.52	83	428.24	297	306	306	15	41	0	2	1
12642	7860	4922	1	1486	1138	28248	547	171	811	58	186	218	83	233	290.32	163	450.59	486	287	411	63	121	7	14	11
4258	13422	9730	890	414	242	28080	1259	9	597	0	12	61	5	77	275.7	91	428.67	300	299	279	13	44	0	2	3
5200	12756	9226	642	594	326	28120	1116	7	710	1	18	69	4	103	279.26	103	435.22	355	355	331	13	50	0	0	4
3924	13670	9932	973	328	190	28058	1335	8	578	1	12	57	4	76	271.25	73	424.35	256	298	259	10	35	0	2	2
4296	13452	9740	866	370	202	28066	1212	11	601	1	6	60	7	66	272.95	113	427	341	289	305	11	41	0	0	0

SUPPLEMENTARY INFORMATION:Monte Carlo Atomistic Simulation and Machine Learning Analysis of Na-K Eutectic Alloy in Condensed Phases, D. Reitz and E. Blaisten-Barojas, George Mason University, Fairfax, VA 22030

5484	12576	9032	571	648	380	28140	1053	8	716	1	20	67	3	96	282.14	109	437.7	411	356	341	17	50	0	0	1
4270	13476	9758	870	348	194	28056	1248	9	617	1	10	56	5	63	273.58	99	426.78	310	315	301	13	37	0	0	3
5458	12520	9030	617	688	420	28160	1124	15	692	3	42	56	11	93	283.76	97	439.54	374	366	328	17	38	0	2	1
4172	13484	9794	882	398	214	28064	1250	6	594	2	2	62	1	77	272.1	95	427.53	294	291	292	18	37	0	0	0
13844	7696	4518	6	1050	794	28006	656	283	967	120	100	227	124	180	277.3	227	441.76	472	391	429	24	91	18	4	2
4536	13304	9598	842	402	226	28070	1224	12	623	2	4	60	6	74	275.11	95	429.91	315	305	302	15	44	0	0	2
5098	12870	9256	674	552	326	28120	1139	13	694	1	18	72	7	88	280.52	114	436.99	348	366	317	24	46	0	0	3
14354	7666	4396	8	796	594	27870	664	337	1067	157	60	252	138	147	269.5	261	432.87	487	451	423	15	86	17	4	1
12412	7882	5006	3	1568	1214	28320	570	160	761	42	218	184	79	224	293.17	163	451.44	490	266	420	73	104	10	18	10
7268	11108	7892	337	1128	776	28292	875	42	720	13	110	104	21	157	288.69	125	445.64	436	342	377	37	57	4	8	6
4138	13518	9804	916	372	218	28064	1278	6	604	0	14	59	3	72	272.14	98	425.2	287	289	282	13	38	0	0	3
12934	7890	4824	0	1356	1036	28202	606	179	856	57	154	177	85	225	285.82	188	450.31	473	314	452	47	85	17	6	1
4768	13084	9456	764	494	282	28100	1190	9	664	0	16	57	5	88	278.29	102	434.37	325	332	313	17	45	0	0	1
3816	13726	10000	986	324	176	28050	1313	5	560	1	4	53	2	75	270.52	78	423.39	255	273	280	15	35	0	4	2
13678	7614	4566	2	1148	890	28056	615	279	899	113	148	231	115	191	278.07	191	445.34	461	347	437	45	97	28	12	3
5360	12672	9092	610	610	378	28144	1075	12	699	0	28	61	7	98	282.07	103	437.66	396	350	344	16	45	0	4	0
12620	7882	4870	2	1510	1206	28312	604	156	755	52	206	172	67	199	292.2	171	449.21	511	292	416	67	84	14	16	8
5322	12624	9110	625	672	394	28148	1106	3	674	0	26	77	3	97	280.86	84	438.13	372	349	323	20	51	0	0	2
14376	7682	4396	5	798	570	27862	633	332	1077	155	40	264	138	160	269.49	286	432.83	485	430	437	13	99	19	0	1
4344	13424	9700	842	374	218	28072	1230	7	631	3	12	64	2	73	272.35	98	426.56	317	320	293	13	38	0	0	3
3914	13662	9930	975	336	198	28056	1301	8	545	1	16	55	4	68	270.6	84	423.02	279	271	278	12	40	0	0	2
4502	13272	9606	802	450	254	28096	1193	5	615	0	10	60	2	83	275.72	91	430.17	326	310	318	17	42	0	2	0
13798	7820	4604	5	972	702	27966	659	283	989	134	70	228	115	158	274.76	225	439.23	477	429	441	29	78	11	0	1
4056	13588	9826	910	358	226	28068	1252	6	583	1	14	63	3	61	271.86	106	425.41	316	284	282	16	40	0	0	3
3966	13634	9914	959	342	190	28056	1297	5	575	0	8	63	2	70	270.96	81	424.56	283	289	267	12	43	0	2	2
5944	12176	8786	522	772	448	28170	1045	17	732	2	44	68	9	110	283.48	105	441.41	390	365	353	24	48	0	0	1
4544	13168	9556	786	530	298	28106	1219	4	622	0	10	61	1	71	277.54	95	433.58	335	328	285	16	43	0	0	4
13792	7868	4598	4	884	706	27958	637	284	1040	124	108	242	113	169	276.69	235	441.11	477	417	430	31	100	22	2	1
6354	11886	8532	432	864	514	28196	966	27	731	4	42	81	17	131	286.54	115	444.21	418	371	371	19	49	1	4	5
4512	13224	9606	818	462	262	28090	1224	9	628	0	24	54	6	79	276.66	92	431.04	316	328	304	13	39	0	0	3
12382	7988	5032	4	1522	1172	28308	592	145	818	44	196	184	77	221	293.01	180	450.34	505	305	410	52	104	8	16	11
4218	13446	9758	889	400	236	28078	1256	6	590	1	18	72	5	69	273.66	97	427.17	314	296	270	15	51	0	2	1
5512	12672	9048	619	524	326	28112	1095	13	725	0	28	54	8	93	282.16	110	439.28	368	380	354	18	35	0	2	3
4328	13358	9708	860	424	240	28078	1226	8	594	1	20	70	3	70	274.3	85	428.7	325	289	285	16	49	0	0	2
4384	13350	9660	841	426	252	28088	1231	9	598	2	14	52	6	65	273.53	91	428.3	328	300	306	11	36	0	2	4
5314	12714	9154	621	590	336	28128	1130	6	728	0	20	67	3	84	279.6	106	436.59	384	407	312	13	49	0	0	2

SUPPLEMENTARY INFORMATION:Monte Carlo Atomistic Simulation and Machine Learning Analysis of Na-K Eutectic Alloy in Condensed Phases, D. Reitz and E. Blaisten-Barojas, George Mason University, Fairfax, VA 22030

4620	13226	9554	796	432	240	28080	1239	10	653	1	8	57	5	81	275.47	100	429.86	299	351	301	16	42	0	0	1
4126	13576	9840	941	318	176	28044	1272	6	606	1	8	58	4	68	271.52	94	424.37	284	279	298	13	37	0	0	3
14154	7770	4446	8	818	638	27896	634	305	1055	147	68	256	129	150	270.54	273	433.08	498	409	438	19	92	16	2	1
4392	13260	9628	832	504	300	28106	1188	10	558	0	22	50	5	88	275.14	91	431.89	327	261	317	16	35	0	0	2
4784	13008	9402	737	568	344	28132	1147	9	611	0	26	59	7	85	279.41	96	435.83	357	298	324	16	39	0	0	5
5266	12708	9168	639	602	356	28134	1114	12	683	1	34	69	7	98	279.91	98	436.08	368	357	329	16	50	0	0	1
4086	13488	9832	905	412	240	28086	1265	2	575	1	26	65	1	60	273.41	88	427.28	309	290	272	22	43	0	2	2
11840	7852	5240	0	1876	1390	28490	533	111	673	29	264	178	62	248	305.73	136	459.66	499	247	409	86	95	1	26	18
4112	13572	9840	905	328	188	28054	1253	6	595	1	14	68	3	77	272.61	92	424.39	304	285	280	10	43	0	0	1
12698	7922	5018	10	1390	1004	28206	611	173	869	53	166	196	90	212	288.97	176	449.3	479	321	429	48	119	9	8	8
12442	8004	4996	3	1490	1158	28292	586	160	767	52	188	188	75	226	287.1	167	448.09	506	280	411	55	102	13	14	6
11366	7970	5338	0	1988	1576	28608	502	102	607	17	328	160	62	322	313.06	105	458.64	465	202	410	90	93	6	42	14
4878	12944	9390	719	568	318	28120	1169	7	663	0	22	52	2	94	279.72	96	435.36	343	347	320	11	31	0	0	2
4406	13260	9628	799	504	294	28112	1180	5	604	0	20	73	4	87	277.9	95	431.34	351	293	292	8	44	0	0	4
12336	7800	4988	1	1734	1298	28378	571	140	717	33	196	176	74	243	299.63	149	452.98	470	243	425	71	92	13	24	16
4176	13564	9806	937	316	180	28048	1294	8	622	2	6	59	4	69	273.11	97	425.15	277	293	284	12	44	0	0	2
3908	13706	9958	976	296	164	28042	1286	6	573	1	10	62	4	62	270.22	97	422.27	287	261	283	14	42	0	0	2
13104	7812	4810	2	1350	958	28154	619	206	861	54	112	196	110	218	287.14	171	448.79	446	323	433	57	108	14	8	3
11170	7856	5392	0	2222	1680	28688	513	76	524	19	336	142	34	286	313.85	107	463.01	501	161	379	108	65	4	28	22
5566	12446	8940	538	728	462	28194	1063	6	685	0	52	75	4	100	283.56	109	439.51	403	377	325	21	47	0	0	6
4100	13528	9844	932	374	202	28058	1282	11	568	1	10	72	7	91	272.37	79	426.2	263	278	267	13	52	0	0	3
14014	7744	4514	5	908	674	27926	637	307	1016	153	60	248	121	160	271.71	257	435.31	480	410	443	21	77	21	12	5
6784	11728	8328	411	784	492	28178	947	22	812	6	54	100	9	133	288.87	145	446.16	424	390	364	20	63	2	8	4
4406	13442	9686	865	328	186	28052	1224	6	640	2	4	60	3	70	274.08	108	427.93	322	299	308	11	43	0	0	0
11662	7850	5348	1	1946	1410	28518	514	120	620	26	264	174	66	263	311.18	114	460	488	204	414	92	83	6	38	18
10884	7884	5604	0	2224	1688	28724	511	69	523	11	388	174	36	279	325.33	103	464.04	472	164	362	128	87	0	52	29
5270	12638	9150	636	684	386	28150	1119	8	657	1	22	58	6	80	280.89	104	436.88	375	354	332	25	39	0	0	3
5500	12390	8972	574	804	484	28196	1042	15	651	1	46	67	11	110	285.07	101	439.93	404	321	352	21	45	1	0	1
4296	13400	9708	872	420	246	28084	1247	8	579	0	12	60	7	85	274.99	87	429.94	290	292	297	15	42	0	2	2
13866	7812	4642	7	914	634	27934	609	290	1035	109	60	237	146	191	275.97	237	439.58	449	397	475	27	111	18	6	1
7578	11176	7818	376	926	628	28208	939	45	776	10	74	99	26	134	288.29	134	446.94	408	366	371	38	55	3	8	3
14512	7604	4342	5	772	562	27846	656	335	1089	175	52	275	119	139	270.79	275	433.45	504	449	406	11	92	23	2	2
4288	13476	9756	883	336	188	28058	1234	8	609	1	14	55	6	68	273.16	109	426.66	311	298	315	11	32	0	0	3
11226	7888	5498	2	2096	1538	28600	512	86	589	16	322	163	41	268	311.88	110	459.24	488	190	386	107	96	6	32	27
12534	7842	4870	0	1588	1266	28330	575	155	768	45	208	182	85	216	289.05	180	448.97	532	283	409	57	93	7	20	7
4354	13268	9650	820	538	300	28118	1253	8	591	0	8	58	4	84	278.16	77	433.52	302	318	273	19	43	0	0	2

SUPPLEMENTARY INFORMATION:Monte Carlo Atomistic Simulation and Machine Learning Analysis of Na-K Eutectic Alloy in Condensed Phases, D. Reitz and E. Blaisten-Barojas, George Mason University, Fairfax, VA 22030

3634	13784	10082	1038	354	194	28060	1352	2	515	0	10	58	1	56	270.45	79	423.34	269	252	246	14	36	0	2	2
4630	13166	9558	750	470	252	28092	1173	5	625	1	16	66	3	88	278.98	91	435.32	333	334	313	16	44	0	0	2
4830	12950	9376	697	606	356	28140	1139	5	644	0	22	51	4	87	278.06	81	434.81	349	336	347	21	34	0	0	3
4598	13178	9510	777	504	306	28112	1160	7	620	0	16	53	4	92	275.7	107	430.73	337	298	336	13	36	0	0	3
11686	7842	5308	3	1900	1448	28532	530	117	607	24	302	180	56	254	312.53	128	458.7	475	181	398	106	97	12	42	19
11742	7926	5148	1	1824	1518	28520	519	106	662	28	322	181	55	277	305.24	132	457.32	471	204	401	97	101	3	36	19
12912	7844	4824	2	1380	1066	28206	633	199	860	77	162	209	84	206	283.36	201	447.31	482	332	401	52	96	17	18	4
10484	7728	5618	1	2570	1962	28856	459	60	479	14	446	162	29	288	322.84	100	469.69	533	138	349	123	73	4	48	36
5520	12582	9040	602	612	352	28128	1093	13	731	0	22	60	10	99	280.95	124	439.16	373	368	352	14	41	0	0	2
11258	8006	5370	0	2022	1596	28612	542	90	590	18	320	173	51	297	312.04	111	458.11	471	195	371	89	99	5	36	22
14330	7728	4392	6	762	594	27870	634	342	1059	166	60	268	127	150	271.64	275	436.15	483	405	434	23	94	21	2	2
4036	13626	9816	951	336	238	28074	1293	7	556	1	20	56	5	81	272.43	89	423.84	277	271	279	9	33	0	2	2
13424	7688	4620	1	1278	968	28114	619	227	899	85	120	223	106	199	283.23	195	445.56	462	344	425	51	115	14	14	5
4496	13318	9638	829	398	214	28072	1221	8	631	0	8	69	6	80	278.16	90	432.32	304	309	299	21	52	0	0	0
4506	13192	9584	782	516	294	28112	1181	2	618	0	20	65	1	96	277.91	87	432.58	322	320	310	17	37	0	0	2
5210	12892	9234	648	466	278	28102	1133	2	747	1	20	65	0	82	280.31	116	437.36	362	391	329	17	49	0	2	3
12486	7878	5070	2	1562	1106	28286	551	152	793	46	172	191	76	247	291.58	169	452.22	475	274	444	57	104	10	10	10
14382	7648	4376	6	808	600	27876	638	328	1066	155	60	255	136	158	269.64	268	432.65	478	438	448	16	90	17	2	2
4048	13476	9836	929	452	256	28086	1277	11	569	2	18	46	6	74	271.07	89	422.84	288	274	291	14	31	0	0	3
11184	7940	5500	2	2072	1554	28616	533	84	559	14	326	159	43	266	314.31	119	461.59	520	180	361	87	78	5	38	21
14248	7720	4418	5	840	618	27892	638	318	1048	156	46	246	122	144	272.53	269	436.24	503	421	448	15	84	20	2	2
4792	13086	9394	712	514	324	28128	1162	2	663	0	16	68	2	82	280.73	99	437	349	350	316	18	42	0	2	4
14198	7716	4418	10	870	648	27902	654	300	1041	165	48	267	108	162	269.61	264	431.87	486	425	411	15	93	12	4	1
7460	11184	7876	284	1004	644	28234	876	32	817	8	58	85	22	138	288.07	130	444.21	444	395	406	35	50	0	8	5
5312	12676	9088	622	638	410	28162	1105	6	704	1	36	52	2	93	279.62	108	437.04	371	366	348	19	34	0	0	4
11610	8000	5228	1	1892	1486	28518	535	108	642	21	278	158	63	258	299.81	136	454.96	518	206	419	82	90	9	20	7
3950	13684	9930	994	298	168	28038	1324	12	590	2	8	65	6	57	271.97	96	425.28	274	276	252	16	44	0	0	3
14108	7690	4454	4	900	688	27926	635	314	1003	155	78	243	123	162	273.87	224	437.65	469	422	450	31	70	14	8	1
4030	13594	9866	953	362	204	28062	1296	8	572	1	6	49	4	73	271.19	92	423.92	278	280	287	10	32	0	0	2
11214	7998	5382	1	2064	1614	28632	503	84	574	17	326	191	43	301	318.77	121	462.39	486	178	356	110	92	5	32	13
11294	8018	5368	1	1964	1572	28584	540	99	587	25	322	158	51	277	313.19	118	459.7	471	198	392	91	74	3	46	21
14200	7610	4436	6	910	676	27926	598	317	1027	144	92	239	128	153	272.87	267	437.9	508	391	466	25	84	20	2	2
4816	12988	9372	733	568	360	28140	1181	5	647	1	36	51	4	93	279.04	81	435.22	328	343	320	18	33	0	0	3
14570	7674	4366	5	680	484	27810	656	332	1153	162	34	269	122	140	267.64	297	428.84	478	463	432	15	102	23	2	1
4580	13276	9578	793	408	228	28076	1209	10	641	1	6	64	7	62	273.52	96	429.81	335	334	306	17	41	0	0	3
12354	7848	5012	2	1640	1242	28318	571	163	780	41	202	181	85	242	294.12	153	451.04	479	281	412	68	103	5	20	10

4056	13590	9846	933	352	208	28062	1300	6	589	1	10	48	2	58	271.88	96	424.1	286	295	283	18	34	0	0	1
7164	11448	8074	385	890	564	28196	974	37	777	7	54	88	21	144	288.81	127	445.26	400	408	354	23	57	0	2	3
11166	7702	5432	0	2254	1714	28706	518	89	533	22	398	175	41	285	320.41	106	464.29	484	163	343	119	83	0	38	23
14418	7620	4332	4	822	624	27882	642	342	1025	164	66	269	135	143	271.21	266	434.39	503	422	414	22	93	22	0	0
4180	13438	9774	876	442	242	28082	1273	3	597	0	6	63	3	89	273.97	79	427.92	276	315	280	14	44	0	0	1
14404	7696	4388	6	754	560	27854	646	323	1100	168	50	253	114	138	272.2	285	434.28	493	447	444	19	75	19	2	0
4282	13432	9750	884	384	206	28062	1238	9	611	1	8	63	7	79	275.18	95	428.37	312	291	293	8	45	0	0	0
13906	7712	4462	3	1032	792	27990	611	278	971	139	84	230	110	179	273.98	214	439.09	483	382	458	26	83	10	2	4
5162	12840	9252	655	530	308	28122	1133	11	705	0	30	72	7	90	280.93	115	437.13	364	371	315	16	44	0	0	3
5994	12136	8746	561	778	462	28162	1077	21	678	8	44	92	12	108	286.33	108	442.76	364	359	320	29	53	0	2	3
12898	7836	4814	1	1428	1084	28226	670	196	802	63	148	179	104	213	286.59	155	446.31	462	338	411	41	85	15	18	8
4666	13138	9508	754	498	280	28102	1160	4	626	1	12	55	2	88	276.83	89	432.81	341	312	335	12	35	0	0	3
5712	12434	8898	566	652	410	28148	1079	15	723	6	40	70	8	113	280.94	95	438.81	365	382	348	20	46	0	2	0
13246	7722	4682	0	1340	1018	28164	604	216	823	71	144	189	114	190	283	179	447.13	502	326	449	42	100	16	12	8
14402	7636	4352	12	794	616	27878	653	334	1034	163	74	262	125	149	271.29	272	434.81	488	422	420	18	89	25	4	2
14336	7602	4346	4	882	664	27898	627	317	1035	157	66	257	119	170	268.87	237	430.79	470	412	448	20	93	21	2	2
14156	7710	4446	8	856	660	27908	645	312	1027	152	68	255	121	153	271.12	252	435.64	485	430	424	24	94	19	12	3
11378	8070	5482	3	1896	1390	28504	557	79	693	13	256	180	38	256	311.9	136	456.85	466	250	373	104	106	3	26	18
4418	13366	9684	813	378	208	28070	1229	5	652	0	16	66	3	69	276.33	93	430.74	317	340	294	17	47	0	0	2
4040	13632	9880	937	312	176	28048	1280	5	604	2	8	62	2	64	272.14	96	423.45	286	286	286	16	39	0	0	2
3982	13676	9914	950	292	170	28048	1282	7	578	1	14	50	5	63	270.29	80	423.88	280	282	300	16	32	0	0	3
4894	12920	9360	697	598	340	28128	1129	8	640	0	14	66	3	99	280.28	93	436.04	363	321	318	12	42	0	0	3
4496	13200	9572	771	544	304	28120	1166	6	596	0	4	68	4	92	278.81	84	435.62	340	299	313	18	39	0	0	0
5184	12842	9270	667	514	278	28110	1159	8	718	0	22	56	2	82	279.41	111	434.85	345	374	326	19	35	0	0	3
12922	7978	4886	6	1298	952	28154	643	180	892	61	106	190	86	197	284.21	194	446.01	476	347	434	37	105	8	8	8
14536	7616	4350	7	748	540	27842	660	347	1102	169	48	277	137	160	271.12	271	434.31	470	463	413	12	96	19	4	1
12132	7950	5078	2	1664	1298	28370	553	161	723	35	226	182	81	265	298.61	146	455.59	469	231	413	69	114	13	22	13
4688	13086	9450	766	540	334	28122	1185	6	642	0	22	60	4	94	280.06	99	433.52	327	311	310	17	38	0	2	0
4688	13108	9506	745	508	280	28106	1137	8	639	2	16	61	2	89	277.09	98	432.59	348	316	335	20	40	0	0	1
14390	7664	4360	8	802	598	27862	661	338	1048	171	44	264	122	155	269.28	251	431.6	466	431	427	17	86	19	2	3
13914	7848	4572	11	866	654	27926	652	289	1055	136	64	238	106	176	274.9	228	438.21	463	447	439	18	91	21	6	2
11840	7706	5164	1	1966	1508	28528	528	117	610	30	306	177	64	282	305.25	109	455.21	473	223	404	80	93	9	38	21
13014	7928	4814	2	1248	978	28142	636	203	884	71	152	198	91	206	281.44	181	446.37	460	346	429	43	95	19	8	6
5630	12452	8952	610	676	400	28138	1070	19	691	11	26	72	5	104	282.17	109	438.76	378	346	346	21	42	0	2	1
4466	13380	9656	855	354	196	28058	1249	11	646	1	6	57	9	70	274.9	89	428.32	298	334	306	12	41	0	0	4
4986	12908	9290	679	570	354	28136	1120	4	668	0	26	68	3	92	280.05	108	435.8	362	324	333	20	47	0	2	2

SUPPLEMENTARY INFORMATION:Monte Carlo Atomistic Simulation and Machine Learning Analysis of Na-K Eutectic Alloy in Condensed Phases, D. Reitz and E. Blaisten-Barojas, George Mason University, Fairfax, VA 22030

4790	13018	9454	753	534	292	28108	1201	4	663	0	20	62	3	93	279.48	93	434.74	312	338	310	16	45	0	0	1
11716	8058	5166	3	1788	1446	28460	580	115	687	23	262	160	57	242	296.47	142	452.13	470	243	422	85	80	12	22	12
4798	13046	9462	738	514	272	28104	1143	7	652	0	12	55	3	81	280.5	109	436.03	365	309	336	14	38	0	0	0
14534	7536	4334	4	838	576	27864	629	343	1049	164	46	262	135	160	271.86	255	434.99	470	418	450	19	83	20	0	3
4658	13218	9552	803	412	222	28070	1192	10	643	1	8	63	7	74	274.45	100	427.91	338	322	314	10	45	0	0	3
4004	13566	9842	936	404	252	28088	1275	4	550	0	20	52	4	68	273.04	87	427.82	297	256	288	13	34	0	0	3
3852	13738	9950	967	298	198	28060	1287	7	556	1	24	58	6	66	271.77	88	424.02	282	266	289	15	38	0	0	1
14238	7768	4442	9	790	590	27878	631	299	1081	162	48	279	104	169	270.82	271	433.31	470	428	427	20	107	16	2	1
13550	7808	4710	3	1088	766	28008	613	277	931	126	80	241	113	197	276.39	208	442.96	467	371	429	35	91	11	4	1
6260	11832	8506	448	962	610	28238	969	23	692	5	64	67	13	119	285.83	124	441.42	444	344	363	27	39	0	4	1
11146	7862	5462	1	2160	1646	28668	544	90	512	15	334	163	47	286	318.29	97	461.47	456	166	363	119	85	6	52	22
14220	7584	4438	7	936	664	27916	632	333	992	162	70	249	128	168	270.41	233	434.93	481	407	443	19	83	22	4	1
7836	10756	7600	290	1176	798	28288	905	49	725	9	112	85	28	157	287.45	122	446.95	422	381	378	36	48	2	10	5
4096	13572	9810	904	354	224	28072	1254	4	587	1	14	60	3	75	273.02	93	426.49	299	283	290	13	40	0	2	3
12832	8120	5024	5	1170	846	28112	627	197	940	63	112	193	111	198	282.28	194	444.49	472	372	464	33	103	9	8	5
11924	8062	5216	1	1694	1272	28396	603	114	755	19	206	175	63	260	294.91	155	451.71	477	277	386	61	107	10	22	10
4388	13394	9708	869	360	194	28056	1226	9	639	1	10	68	4	81	272.56	112	425.95	310	297	293	11	43	0	2	2
12460	7934	5046	2	1506	1124	28270	591	170	793	56	182	188	83	222	290.17	146	450.47	478	316	426	63	97	11	18	6
14362	7734	4386	8	742	576	27860	647	327	1073	166	60	270	122	149	269.1	264	432.48	497	454	413	15	91	14	0	0
4684	13106	9490	761	508	296	28106	1171	5	613	1	22	65	3	89	277.83	97	434.5	334	306	318	15	40	0	0	3
4704	13182	9510	798	426	244	28080	1213	10	656	2	12	63	4	65	274.89	107	429.32	330	335	297	22	47	0	2	1
14372	7690	4396	4	764	576	27860	627	327	1089	163	58	259	123	152	267.42	270	429.13	482	439	456	20	82	19	4	0
4064	13682	9900	970	246	130	28026	1305	11	611	1	4	71	6	60	270.5	102	422.21	281	294	262	12	50	0	0	3
12344	7814	4986	1	1674	1292	28358	561	151	716	38	226	161	85	228	291.86	167	452.4	515	243	434	71	87	6	22	3
4606	13204	9574	811	452	238	28082	1224	6	660	1	8	59	2	66	274.07	99	429.55	322	337	303	20	40	0	0	1
4464	13302	9628	814	428	248	28088	1210	6	643	0	18	65	3	81	276.13	99	428.48	327	326	294	13	40	0	0	2
11972	7834	5222	3	1806	1320	28424	547	144	670	31	240	179	79	246	300.61	128	456.66	488	231	414	85	102	10	30	10
10852	7854	5620	0	2304	1698	28746	550	56	522	5	378	150	36	259	323.35	109	466.06	487	156	356	121	83	6	34	36
5204	12646	9124	626	728	448	28184	1100	4	651	0	34	70	4	87	281.14	91	438.55	386	340	326	27	44	0	0	3
4348	13352	9662	849	448	266	28086	1243	6	599	1	8	70	3	83	274.35	87	428.79	291	315	287	20	42	0	2	2
10690	7852	5676	1	2334	1750	28756	498	69	493	9	406	158	36	296	333.82	89	469.74	460	150	373	117	86	0	46	37
4030	13660	9916	966	280	142	28032	1299	8	608	1	4	72	4	68	271.06	107	424.12	283	289	260	11	47	0	0	1
4570	13146	9562	786	520	290	28110	1215	3	656	1	22	58	1	68	275.11	101	430.04	340	336	291	21	38	0	0	0
4304	13434	9718	849	378	226	28080	1237	5	609	0	20	52	1	66	275.75	86	428.93	306	325	308	21	31	0	0	3
14294	7716	4404	8	788	608	27878	647	321	1066	165	66	280	126	165	271.23	269	434.34	483	436	407	12	105	15	2	2
7834	10968	7706	281	1008	634	28216	862	50	837	13	62	99	29	147	288.81	149	444.49	457	403	391	30	61	2	2	1

SUPPLEMENTARY INFORMATION:Monte Carlo Atomistic Simulation and Machine Learning Analysis of Na-K Eutectic Alloy in Condensed Phases, D. Reitz and E. Blaisten-Barojas, George Mason University, Fairfax, VA 22030

4356	13432	9686	840	366	222	28076	1242	5	642	0	14	62	2	76	274.85	93	430.45	303	328	295	13	36	0	0	4
14368	7682	4378	6	776	594	27862	646	344	1071	171	62	268	138	145	268.35	271	429.99	486	451	427	21	83	15	2	1
4704	13016	9422	735	610	370	28146	1148	6	594	0	24	48	3	93	279.27	89	434.86	354	292	332	17	23	0	0	1
4638	13240	9552	801	396	230	28074	1189	9	660	2	18	66	4	88	274.59	109	430.61	326	312	314	9	43	0	0	1
3932	13628	9930	976	360	198	28060	1314	5	561	1	10	67	4	63	270.87	91	423.69	269	277	265	17	43	0	2	4
13756	7774	4606	3	1028	740	27980	675	281	976	124	68	232	120	170	274.98	233	439.77	459	416	416	33	92	16	8	1
12922	7762	4818	2	1458	1088	28224	631	191	795	57	166	190	95	206	284.71	177	448.29	481	287	404	61	100	17	8	2
13848	7806	4560	6	986	710	27964	629	296	969	124	50	230	133	163	275.19	229	440.29	493	396	446	28	87	14	4	0
13818	7722	4618	4	1002	720	27984	639	298	987	125	100	237	121	188	276.46	215	443.88	463	398	422	28	94	26	4	4
5716	12370	8946	550	700	386	28150	1050	15	715	2	30	71	9	86	284.22	109	439.71	422	364	339	19	47	0	2	3
4656	13214	9522	789	422	250	28078	1222	8	662	1	14	54	4	72	276.38	97	431.58	318	340	303	18	37	0	0	2
5062	12858	9288	662	558	328	28126	1116	7	700	0	32	65	5	84	278.37	114	434.05	368	346	336	19	46	0	0	5
13352	7796	4720	2	1218	892	28098	601	243	915	89	110	216	111	216	282.26	217	448.47	475	345	432	38	97	18	10	1
4152	13548	9814	921	342	194	28060	1258	5	592	1	10	59	3	69	273.3	101	427.28	294	275	295	17	45	0	0	2
13868	7790	4560	9	960	710	27964	646	281	1007	130	70	240	115	174	277.18	255	443.35	485	401	414	25	98	12	6	0
12240	7650	5018	1	1880	1398	28458	560	144	628	29	230	163	77	259	301.2	132	456.19	480	222	406	83	86	10	38	11
11862	7860	5182	2	1860	1416	28474	545	127	644	36	270	175	60	270	299.93	135	453.43	487	204	392	74	88	7	22	20
4580	13158	9552	770	518	286	28106	1208	5	635	0	12	65	3	75	278	90	431.12	328	343	293	18	49	1	0	4
14050	7726	4468	5	912	700	27938	596	291	1022	149	72	254	114	208	273.4	233	438.11	456	402	458	17	88	9	10	2
11456	7672	5314	2	2140	1646	28646	485	103	541	17	374	184	59	300	317.87	102	460.26	468	161	396	102	90	11	42	30
11506	7840	5290	3	2024	1554	28558	547	104	597	17	310	148	57	271	306.64	121	454.31	481	210	411	87	77	5	32	19
11718	7968	5216	3	1792	1450	28482	571	107	699	17	306	174	61	251	299.54	135	454.62	491	242	387	78	110	11	32	17
5100	12632	9210	625	732	446	28182	1112	8	670	0	60	76	6	101	279.97	104	438.09	380	359	303	22	54	0	2	1
12084	7938	5158	1	1684	1280	28406	583	146	712	33	232	169	79	254	299.34	150	454.7	482	263	408	64	93	5	28	13
11732	8020	5240	3	1784	1386	28444	558	126	707	35	262	156	67	251	297.16	151	455.18	468	246	437	79	75	4	18	18
14410	7658	4334	6	804	616	27882	639	343	1032	166	58	248	127	159	270.53	261	433.75	488	419	443	14	74	26	2	0
8048	10770	7520	264	1086	734	28256	862	50	797	16	90	94	28	156	289.04	139	445.89	464	391	382	26	49	1	8	3
4340	13366	9668	854	426	266	28090	1249	5	623	0	24	59	2	79	276.17	89	428.94	308	317	280	15	44	0	0	1
4232	13496	9752	890	356	216	28066	1240	11	599	2	14	64	6	65	273.04	97	426.34	320	289	289	12	44	0	0	4
4174	13498	9778	864	384	230	28082	1214	5	583	0	18	59	3	77	274.54	85	429.03	312	278	313	18	38	0	0	2
14142	7528	4386	1	1022	780	27970	625	317	944	157	104	250	120	174	273.02	215	437.19	475	384	431	32	78	17	8	2
14054	7712	4478	5	912	698	27950	654	281	1013	136	96	227	94	162	274.14	233	439.27	485	410	436	22	77	21	0	1
14190	7760	4438	7	836	622	27894	650	318	1043	157	48	262	119	157	270.67	258	433.46	505	442	408	9	94	17	0	1
5520	12410	8960	601	784	470	28176	1087	14	674	1	30	62	8	98	282.34	112	438.9	390	349	329	23	36	0	2	1
10772	7818	5610	0	2306	1784	28780	521	73	477	19	440	154	31	296	321.39	86	465.87	460	162	356	123	70	2	42	37
11884	7912	5188	2	1832	1364	28436	574	109	693	23	250	163	61	244	299.85	150	454.15	489	253	415	77	87	7	4	12

SUPPLEMENTARY INFORMATION:Monte Carlo Atomistic Simulation and Machine Learning Analysis of Na-K Eutectic Alloy in Condensed Phases, D. Reitz and E. Blaisten-Barojas, George Mason University, Fairfax, VA 22030

4422	13304	9658	842	448	242	28082	1225	11	603	0	8	60	8	79	275.45	97	430.99	318	296	297	13	37	0	0	0
4658	13054	9458	733	584	358	28144	1166	3	619	0	32	62	2	95	276.29	90	433.18	345	312	309	16	39	0	0	1
5562	12528	8994	534	654	386	28150	1037	8	749	0	24	64	4	117	278.97	114	438.68	392	386	368	11	43	0	2	3
12810	7804	4844	4	1506	1120	28254	560	168	787	60	160	181	79	231	286.53	178	449.53	507	277	441	48	92	9	8	6
11262	7814	5402	0	2136	1646	28672	527	89	559	15	370	145	46	277	319.88	108	463.04	488	199	390	111	82	8	40	23
4728	13008	9432	749	592	352	28140	1159	8	619	2	28	62	4	85	277.77	96	432.97	363	306	310	16	37	0	0	0
5466	12580	9018	650	646	398	28132	1114	20	658	6	22	73	10	92	282.15	97	439.12	377	339	320	16	49	0	2	2
4944	12960	9336	722	528	324	28122	1148	10	646	1	30	60	6	104	280.2	97	436.38	336	333	331	12	38	0	0	3
4318	13412	9718	861	392	222	28076	1241	3	623	0	14	63	2	80	275.7	99	431.35	299	308	295	15	37	0	0	2
12540	7934	4990	1	1502	1122	28272	592	163	792	53	168	187	81	223	289.05	158	449.94	492	291	432	50	103	10	16	8
13474	7918	4752	13	1022	748	28012	634	260	979	120	94	230	109	178	277.29	235	444.28	482	395	422	33	91	10	4	3
3768	13738	10022	1007	330	182	28054	1304	7	542	1	14	57	5	77	270.31	79	423.95	264	256	282	11	33	0	0	2
10980	8114	5516	2	1984	1624	28650	519	73	582	8	398	166	48	295	316.08	118	460.1	492	181	377	90	98	4	32	22
4550	13182	9580	781	496	274	28100	1186	6	654	1	18	71	4	64	275.16	112	430.09	343	319	302	27	45	0	0	2
11602	8090	5324	1	1810	1384	28490	566	112	690	25	250	152	71	263	303.7	140	457.07	476	233	429	75	87	4	28	15
14380	7628	4412	8	820	572	27862	639	345	1041	160	50	274	137	159	272.45	243	436.9	473	426	419	22	96	25	0	2
5626	12502	8992	567	628	358	28134	1073	15	731	2	28	57	7	94	285.13	108	440.11	395	382	356	15	39	0	0	1
12402	7828	4916	2	1666	1306	28352	604	166	724	34	220	155	89	220	293.85	158	451.28	517	271	410	51	86	10	12	12
4926	13068	9382	717	430	260	28086	1143	8	694	0	20	59	4	83	274.63	106	431.93	344	343	344	20	42	0	0	0
4224	13472	9772	872	380	212	28072	1230	7	601	0	12	62	2	65	274.25	117	429.02	325	275	292	15	38	1	0	3
13656	7826	4572	6	1066	814	28018	611	259	964	114	76	221	115	184	276.64	227	441.57	479	355	458	33	92	15	8	2
14156	7720	4488	6	850	618	27896	619	303	1051	148	56	255	117	154	271.06	270	434.81	497	405	449	19	96	22	8	2
5720	12348	8872	503	724	460	28184	1029	5	734	0	60	88	4	113	281.87	105	439.86	408	386	325	24	54	0	0	3
4050	13638	9880	939	288	170	28042	1279	9	611	1	16	67	6	73	271.55	94	425.47	281	292	281	11	44	0	0	3
12240	7966	5096	2	1596	1202	28310	601	150	764	45	188	197	75	273	288.54	143	450.43	448	259	402	54	114	10	22	4
14448	7512	4266	6	904	700	27914	637	338	999	163	76	262	138	157	271.94	248	436.96	496	416	416	21	87	18	8	2
14304	7698	4392	7	788	624	27888	673	325	1038	160	74	266	131	160	270.36	235	433.5	449	449	419	27	97	15	8	1
10432	7956	5768	0	2378	1816	28832	489	57	483	9	420	170	24	319	333.95	76	472.82	473	149	337	115	83	4	50	38
14358	7734	4408	6	732	558	27848	676	333	1098	167	58	265	132	148	267.86	272	429.04	463	464	425	19	87	13	0	0
14298	7688	4456	7	812	566	27866	649	327	1061	153	42	254	138	141	271.35	264	433.86	476	423	448	23	88	19	4	2
11052	7882	5500	0	2142	1670	28682	524	84	559	13	388	159	51	304	316.63	115	464.23	478	178	368	101	88	8	48	24
6190	12112	8678	505	736	410	28146	1021	19	730	2	20	70	14	121	283.66	105	439.68	383	374	380	17	42	0	0	2
13922	7766	4506	9	942	738	27974	648	281	1019	135	94	236	114	166	275.18	224	438.71	479	409	434	30	91	18	6	0
13588	7772	4624	4	1102	832	28028	637	272	934	124	100	234	109	176	275.81	217	441.35	478	378	419	40	89	19	10	2
3984	13670	9902	952	300	180	28050	1273	5	586	1	14	59	3	72	271.77	93	424.66	275	277	301	16	41	0	0	2
14480	7618	4326	4	792	590	27858	629	334	1067	168	48	270	128	151	267.46	276	430.51	501	431	430	14	90	20	2	0

SUPPLEMENTARY INFORMATION:Monte Carlo Atomistic Simulation and Machine Learning Analysis of Na-K Eutectic Alloy in Condensed Phases, D. Reitz and E. Blaisten-Barojas, George Mason University, Fairfax, VA 22030

14320	7734	4434	7	764	558	27858	620	336	1097	159	46	251	132	151	269.52	282	432.36	494	440	463	15	82	20	2	0
4564	13248	9572	812	438	250	28082	1234	11	632	2	10	54	5	78	275.29	102	429.35	316	329	296	11	31	0	0	2
5004	12940	9304	697	524	324	28126	1170	7	694	0	30	56	4	85	279.92	108	433.75	340	362	321	21	38	0	0	1
13908	7794	4488	5	954	746	27970	665	279	983	124	72	233	123	152	276.89	236	441.95	485	409	422	33	94	14	8	0
12498	7878	4950	0	1514	1220	28316	548	166	790	53	226	195	84	252	294.42	168	452.21	503	289	421	43	102	7	28	14
13230	7702	4744	3	1330	988	28164	619	219	840	66	158	191	111	214	283.55	189	446.97	470	305	443	42	97	19	12	3
3950	13628	9930	951	350	186	28052	1297	9	584	1	6	56	6	65	270.32	99	421.68	274	288	282	19	39	0	2	1
11570	7968	5270	1	1938	1488	28536	556	102	631	19	270	171	54	272	307.01	149	457.38	487	211	385	84	86	9	32	16
4988	12818	9250	663	674	412	28166	1106	4	625	0	22	68	2	107	283.12	101	438.53	373	323	322	17	38	0	2	1
4150	13526	9820	898	358	196	28060	1225	9	596	0	10	69	6	67	273.14	90	426.54	317	275	298	15	47	0	0	4
13550	7634	4528	2	1258	984	28102	599	257	839	106	144	208	106	209	276.16	193	444.99	488	329	450	30	77	16	4	3
4054	13602	9860	944	332	194	28054	1269	8	587	1	12	54	4	77	270.66	87	422.86	280	272	298	13	38	0	0	2
4800	13026	9424	754	532	308	28110	1207	6	704	1	20	64	2	88	276.13	104	429.91	317	362	297	17	48	0	0	3
11616	7966	5346	1	1860	1394	28484	534	103	662	27	276	178	47	277	299.6	134	456.37	494	220	386	73	101	6	26	17
4022	13552	9854	928	402	232	28074	1258	5	558	0	12	62	3	80	271.23	95	425.92	285	263	293	14	37	0	0	4
4824	12994	9426	735	552	304	28116	1186	5	676	0	14	57	2	76	278.21	105	434.71	345	350	303	19	40	0	0	3
13388	7994	4728	6	1002	796	28020	616	249	986	105	106	215	103	194	275.09	232	439.91	470	368	466	28	90	22	6	1
4798	12886	9412	710	656	366	28150	1129	11	599	1	32	58	7	96	280.28	78	437.45	356	303	325	24	36	0	0	1
13114	7722	4728	0	1398	1062	28192	611	216	795	69	146	190	109	217	281.97	168	447.51	459	295	446	56	95	14	22	3
4500	13274	9600	815	448	258	28092	1232	6	640	0	10	55	4	76	276.78	105	430.07	312	323	306	11	36	0	2	5
10720	7864	5650	0	2314	1774	28792	507	54	493	12	388	157	31	285	324.28	94	467.62	505	143	362	107	73	3	76	33
4538	13208	9582	780	486	274	28106	1196	6	606	1	16	71	5	88	278.3	93	433.04	322	314	304	15	56	0	2	3
4134	13574	9814	884	330	196	28058	1249	8	590	2	10	65	5	61	271.06	94	424.06	311	303	291	18	42	0	0	1
13060	8010	4828	3	1168	914	28116	655	207	908	71	120	207	100	188	284.85	193	447.88	466	368	418	40	100	18	14	8
12954	7782	4834	3	1420	1052	28210	616	191	852	63	146	174	90	198	282.7	198	448.04	504	324	440	46	83	17	22	4
5942	12106	8724	478	876	524	28214	1012	8	715	0	40	74	6	115	284.79	121	440.43	419	370	348	25	42	1	2	0
4654	13166	9530	758	478	262	28100	1167	7	648	1	8	67	5	83	277.09	104	431.93	344	319	319	16	42	0	2	1
13740	7602	4510	3	1158	902	28060	595	272	893	108	136	232	132	194	276.63	219	445.14	491	336	443	35	97	16	12	2
4856	13012	9386	704	536	314	28120	1143	7	659	0	14	62	5	86	277.35	106	433.47	358	329	328	15	46	0	2	3
4090	13620	9828	921	314	196	28058	1280	9	582	2	10	58	6	70	271.72	88	424.7	286	296	284	14	36	0	0	2
4932	12886	9330	678	606	358	28142	1133	8	682	0	30	70	4	83	277.95	111	434.37	374	342	313	18	46	0	0	4
12936	7760	4806	1	1452	1096	28234	595	197	800	68	172	197	104	223	283.72	183	451.04	501	300	420	43	101	12	8	5
7822	10912	7644	290	1038	720	28246	844	49	800	12	98	104	31	163	288.24	130	443.65	435	365	404	40	60	0	12	1
4318	13414	9716	868	388	222	28072	1251	9	616	1	14	53	5	81	272.67	97	429.45	293	307	301	10	36	0	0	4
3920	13672	9932	958	330	190	28056	1300	3	570	0	12	66	3	81	271.72	74	425.85	262	288	272	12	44	0	0	3
14012	7716	4500	5	944	692	27934	604	312	981	143	66	248	127	168	274.55	244	438	495	393	452	25	84	17	4	0

SUPPLEMENTARY INFORMATION:Monte Carlo Atomistic Simulation and Machine Learning Analysis of Na-K Eutectic Alloy in Condensed Phases, D. Reitz and E. Blaisten-Barojas, George Mason University, Fairfax, VA 22030

13066	7780	4806	2	1366	1000	28168	587	198	846	75	138	196	95	193	282.68	188	446.03	496	311	453	52	96	9	12	4
4376	13204	9588	809	598	360	28142	1222	7	576	0	14	54	5	88	276.37	75	432.64	323	294	287	17	33	0	2	2
13088	7984	4902	6	1170	836	28092	661	201	962	59	106	186	110	188	281.54	212	447.78	478	386	433	31	100	16	6	3
8034	10782	7504	286	1074	752	28256	859	58	791	18	106	105	36	144	289.78	157	445.85	474	383	374	27	56	0	4	7
4692	13224	9490	782	414	258	28094	1237	8	675	0	16	56	6	77	278.01	99	432.17	310	364	296	16	38	0	0	2
3986	13696	9916	956	276	158	28040	1281	11	591	1	8	58	7	63	270.65	92	422.64	283	273	294	15	43	0	0	2
6096	12104	8682	516	776	472	28180	1047	16	728	4	50	67	9	112	287.04	115	442.54	375	363	359	30	37	0	0	2
4272	13444	9714	879	400	240	28080	1244	6	601	2	10	67	3	82	274.21	97	427.5	305	278	286	12	46	0	0	1
4012	13618	9872	949	346	202	28058	1294	9	574	1	8	62	7	71	272.41	85	424.57	279	280	275	12	34	0	0	3
7122	11452	8082	394	876	590	28210	961	28	797	4	84	81	15	147	288.9	128	443.79	383	397	370	34	48	1	4	5
11338	7698	5440	2	2192	1608	28672	474	92	538	20	344	166	47	287	324.46	104	465.02	509	164	384	107	87	6	46	26
12044	7864	5176	0	1740	1316	28422	546	138	697	29	242	162	74	254	301.74	139	454.6	501	242	415	71	84	10	40	14
5382	12716	9134	610	540	314	28112	1107	9	753	0	26	71	4	88	280.22	125	436.28	367	385	336	21	52	0	0	2
13960	7794	4588	5	856	634	27918	621	303	1029	142	80	244	127	187	275.01	229	441.26	440	413	466	26	88	10	4	3
12880	7890	4912	1	1326	1004	28198	563	205	855	62	168	187	105	226	283.91	182	448.39	470	307	480	53	87	14	18	5
6954	11454	8160	371	988	614	28232	930	28	750	3	58	88	18	142	287.86	134	446.35	445	372	359	22	57	1	4	2
11718	8060	5314	0	1774	1326	28456	572	103	709	26	244	192	48	276	304.78	156	455.48	476	234	371	66	101	10	18	15
13514	7900	4696	3	1024	778	28018	612	248	961	110	102	225	106	201	278.54	213	443.2	474	378	445	22	93	10	4	5
4314	13450	9718	881	364	210	28064	1237	8	633	1	8	52	5	73	272.85	109	426.47	306	291	313	12	41	0	0	2
14176	7692	4466	6	866	634	27902	650	317	1042	159	62	252	124	156	270.9	245	434.98	482	442	435	18	79	19	4	2
11924	8040	5152	2	1736	1336	28428	584	112	708	24	222	159	60	248	301.71	157	456.46	514	247	404	56	94	13	16	9
5258	12832	9210	634	506	284	28102	1101	7	741	1	12	65	3	101	277.58	110	435.81	359	368	352	15	42	0	0	0
4550	13164	9560	785	508	298	28108	1207	7	614	0	28	66	4	79	276.14	91	432.23	326	329	297	19	44	0	0	2
5186	12862	9226	646	520	302	28108	1117	14	722	4	12	65	7	86	279.18	117	434.74	368	366	338	13	38	0	0	4
3958	13650	9930	949	328	176	28050	1285	6	588	1	6	59	3	67	271.44	97	424.66	286	280	276	14	40	0	2	3
5312	12678	9104	624	648	392	28158	1087	11	685	1	24	69	7	90	280.13	109	437.59	402	346	325	20	43	0	0	0
4834	12972	9374	727	578	354	28144	1159	8	640	1	30	57	3	89	279.54	94	434.91	351	323	318	16	40	0	2	3
4936	12968	9344	692	528	316	28114	1118	6	657	0	22	72	5	91	278.47	118	434.31	356	322	337	19	48	0	0	3
10306	7850	5832	1	2486	1876	28870	479	68	444	13	448	176	25	283	333.04	88	473.63	506	123	309	136	81	5	72	45
13992	7650	4468	5	1012	756	27968	608	293	954	136	86	248	122	196	275.06	215	440.58	459	352	445	27	99	17	4	4
10632	7942	5662	4	2318	1770	28766	531	38	522	1	386	163	30	263	324.67	101	467.64	481	185	379	107	71	3	48	44
5340	12666	9134	613	620	352	28134	1113	8	712	1	22	68	4	89	280.13	112	436.64	377	369	326	17	45	0	0	3
3968	13696	9910	981	288	174	28046	1307	6	579	1	8	58	4	73	271.66	87	424.35	263	278	281	10	40	0	2	4
11380	7970	5398	3	1964	1508	28570	549	90	635	23	320	177	48	280	305.59	112	459.16	477	217	388	93	97	5	26	14
13690	7794	4654	2	1024	734	27982	611	291	989	119	82	227	137	179	276.03	238	440.88	486	384	460	24	91	12	4	3
3978	13660	9906	988	318	180	28048	1320	8	577	1	6	61	4	67	271.69	93	425.08	261	275	266	17	40	0	0	2

SUPPLEMENTARY INFORMATION:Monte Carlo Atomistic Simulation and Machine Learning Analysis of Na-K Eutectic Alloy in Condensed Phases, D. Reitz and E. Blaisten-Barojas, George Mason University, Fairfax, VA 22030

10584	7986	5740	2	2268	1732	28776	483	61	493	8	412	149	27	290	331.72	108	469.44	508	134	367	119	81	1	48	24
3948	13672	9924	956	316	180	28050	1288	8	580	1	10	59	5	63	270.86	93	424.26	279	284	284	19	36	0	0	2
10876	7898	5526	1	2258	1754	28746	497	64	499	8	374	175	27	278	329.57	105	467.92	514	159	343	118	89	3	56	28
14310	7650	4374	2	864	642	27896	613	314	1032	170	50	256	110	160	268.91	265	431.53	501	415	445	18	73	16	4	0
4078	13562	9850	926	356	202	28062	1270	10	592	1	14	60	5	65	269.43	95	422.07	299	280	284	16	42	0	0	1
10836	7842	5656	0	2292	1700	28774	495	71	495	10	394	161	42	299	332.43	99	468.72	467	136	370	124	72	3	48	30
11090	7946	5458	0	2090	1654	28654	504	68	575	15	380	181	33	296	314.18	108	463.1	478	164	362	111	105	4	28	22
4790	13040	9426	709	540	310	28122	1174	4	628	0	16	60	2	84	282.43	83	438.28	345	348	315	16	36	0	0	2
14298	7626	4362	4	874	670	27908	623	311	1040	164	76	256	117	159	270.1	263	434	490	412	444	20	81	15	2	3
3786	13734	10008	997	328	184	28052	1318	7	564	1	10	64	3	65	270.41	83	422.42	259	263	266	20	47	0	2	3
11948	8040	5138	2	1678	1338	28408	569	119	738	34	240	179	53	237	294.82	164	450.92	525	253	374	70	104	11	24	9
4934	12920	9362	683	564	318	28122	1127	4	665	0	24	74	3	99	279.09	110	435.2	353	327	328	15	58	0	0	2
5228	12786	9198	662	550	328	28118	1129	5	705	0	26	59	4	92	279.38	105	434.17	353	353	341	15	40	0	2	4
4080	13600	9852	927	330	186	28054	1262	3	602	0	6	52	1	67	272.66	99	426.22	296	286	303	13	34	0	0	2
6406	11794	8440	435	908	598	28228	999	25	740	3	74	82	16	127	287.6	109	444.17	402	386	346	31	57	0	8	4
5014	12862	9280	694	582	362	28138	1123	11	652	0	36	62	7	101	278.99	95	434.8	351	335	336	21	41	0	2	0
5498	12598	9040	569	618	362	28138	1092	10	725	1	20	73	2	79	281.26	110	436.94	382	391	335	28	50	0	2	4
10606	7772	5726	0	2452	1796	28820	520	66	455	8	416	157	32	316	332.18	83	472.05	467	154	333	110	68	4	46	42
11770	7822	5208	1	1894	1488	28532	510	129	626	22	302	162	74	274	310.09	125	460.01	474	205	425	88	85	6	40	25
13528	7870	4730	5	1058	740	28000	646	262	970	118	72	225	115	167	276.85	233	440.36	491	390	431	27	87	14	2	2
4118	13534	9830	922	368	202	28060	1286	7	594	1	8	54	5	69	272.18	81	424.67	282	299	288	13	36	0	0	3
6702	11550	8232	415	1020	672	28256	961	29	684	9	78	91	16	143	288.5	108	445.43	425	342	336	29	52	0	2	3
13262	7726	4664	5	1346	1014	28148	578	226	833	76	122	214	115	207	280.79	180	448.06	490	304	433	51	105	18	14	5
4808	13100	9434	737	472	276	28106	1168	3	659	0	16	57	3	83	276.44	103	432.46	355	331	318	9	41	0	0	3
6478	11830	8432	477	850	548	28204	1004	18	764	1	62	72	12	123	287.65	127	444.95	392	363	362	31	49	0	4	4
4824	12990	9368	702	576	356	28140	1125	3	632	1	26	68	1	88	276.17	103	433.39	369	327	324	21	41	0	0	1
4530	13358	9638	837	330	184	28054	1236	6	673	0	14	63	3	72	275.4	107	431.81	308	338	297	8	45	0	0	7
5096	12786	9238	659	628	368	28140	1087	7	646	1	24	66	5	68	279.29	122	437.1	414	313	338	20	47	0	0	3
4894	12872	9364	713	640	354	28146	1163	5	637	0	22	49	4	97	279.48	105	435.37	347	331	325	13	35	0	0	1
13094	7862	4804	3	1276	960	28144	629	200	877	64	138	192	97	211	283.38	191	446.13	462	328	440	39	97	16	10	7
4940	12924	9350	703	570	318	28114	1150	10	673	1	12	67	5	89	276.25	102	433.35	346	343	317	20	52	0	0	2
3788	13714	10016	1007	348	180	28052	1302	7	538	0	6	57	4	76	271.43	84	424.38	261	248	281	16	40	0	0	1
13752	7910	4650	4	906	664	27946	626	261	1076	136	54	250	98	152	274.56	270	439.21	504	415	433	27	98	8	10	2
13118	7928	4728	9	1242	980	28124	636	229	858	95	112	226	95	177	277.86	207	444.39	513	340	397	36	101	19	16	5
4674	13110	9494	745	532	294	28110	1170	5	648	0	6	59	4	86	277.48	102	434.93	343	322	318	16	38	0	0	1
4818	13004	9424	718	540	306	28114	1154	11	660	0	22	53	7	71	276.47	98	431.51	358	337	332	20	38	0	0	4

SUPPLEMENTARY INFORMATION:Monte Carlo Atomistic Simulation and Machine Learning Analysis of Na-K Eutectic Alloy in Condensed Phases, D. Reitz and E. Blaisten-Barojas, George Mason University, Fairfax, VA 22030

4606	13314	9580	792	358	200	28062	1193	6	668	0	4	63	4	71	274.12	124	429.61	334	311	312	14	48	0	0	3
4118	13648	9864	920	252	142	28034	1267	5	612	1	10	63	3	57	271.17	103	423.67	303	292	293	11	36	0	0	3
13102	7836	4862	4	1268	912	28122	605	227	900	76	134	187	108	191	284.78	196	447.58	475	331	470	47	85	22	6	5
5912	12290	8808	540	684	418	28160	1067	20	729	4	46	68	15	104	283.55	112	440.56	405	392	342	10	44	0	2	1
14478	7580	4340	3	824	592	27868	632	356	1033	171	52	258	137	162	268.79	249	432.32	482	429	448	11	83	22	2	2
5002	12914	9334	693	540	304	28114	1142	7	662	1	18	67	3	89	279.21	96	435.53	351	355	320	21	44	0	2	1
5022	12792	9322	688	630	342	28138	1151	7	647	0	28	62	5	79	279.58	106	436.46	374	342	311	16	45	0	2	2
4146	13528	9812	923	364	204	28062	1282	7	591	1	6	69	2	74	272.8	97	426.49	290	291	266	13	42	1	2	1
12966	7918	4838	2	1288	998	28174	620	189	861	74	150	193	83	208	282.12	180	447.23	479	335	431	47	89	12	16	2
4496	13344	9594	836	390	244	28080	1237	10	629	1	10	57	5	75	273.68	93	428.19	319	326	293	9	36	1	2	3
14468	7700	4302	8	754	596	27872	639	327	1059	164	50	258	128	131	271.13	285	432.89	514	428	433	20	80	12	2	0
12714	8000	5024	7	1308	966	28186	610	190	865	58	170	195	92	231	286.7	199	447.74	465	325	425	41	104	10	4	8
10640	7636	5602	0	2510	1952	28900	513	68	449	8	496	144	37	306	325.45	81	470.85	483	144	343	133	68	1	58	30
4310	13416	9720	858	398	220	28070	1233	6	607	1	6	60	4	65	273.89	89	427.31	323	303	295	15	35	0	0	2
13412	7696	4672	5	1258	932	28112	580	226	875	87	132	211	105	208	280.88	192	445.82	494	315	454	36	102	13	10	3
12702	7900	5002	4	1414	1022	28210	621	163	855	58	154	190	77	200	287.48	193	448.34	498	324	405	51	101	9	14	6
4606	13130	9520	804	506	312	28112	1235	6	649	0	38	70	2	92	275.72	95	430.09	302	341	276	17	49	0	0	1
4164	13360	9750	877	510	298	28106	1271	9	574	0	24	69	6	94	274.43	83	429.29	275	309	269	17	42	0	0	2
4078	13432	9834	929	474	252	28084	1293	8	574	1	14	57	4	67	274.29	84	426.79	298	292	263	11	43	0	0	3
12036	7900	5150	2	1762	1310	28396	581	123	718	32	212	180	57	252	293.21	142	452.03	501	257	397	53	100	13	24	14
5392	12680	9092	648	602	350	28128	1155	9	712	2	12	56	5	83	281.98	109	437.82	355	377	325	18	44	0	0	2
11756	7872	5270	3	1868	1414	28488	529	112	641	28	262	177	56	257	312.19	121	457.95	474	209	397	105	90	8	38	16
11484	7894	5346	1	1970	1514	28562	508	93	613	19	316	157	40	282	310.54	130	459.51	495	191	394	89	86	5	36	19
5538	12538	9000	626	632	392	28140	1084	14	661	5	38	84	9	105	282.1	97	438.74	372	332	334	18	61	0	2	1
14430	7658	4362	4	790	576	27858	670	341	1070	167	36	257	127	136	268.46	266	430.6	486	458	423	19	78	19	4	1
4924	12902	9348	718	590	338	28124	1167	9	655	1	22	72	4	106	279.75	90	437.01	323	329	309	15	53	0	0	3
3884	13732	9962	986	286	166	28040	1305	6	561	1	10	58	3	71	271.11	82	422.22	263	273	283	11	35	0	0	4
4700	13186	9534	776	414	226	28076	1166	4	675	0	16	64	3	93	277.22	114	433.58	326	316	332	10	50	0	0	3
4426	13248	9654	835	498	260	28094	1227	6	614	1	8	55	2	80	274	103	429.98	318	300	302	9	42	0	0	4
14060	7774	4466	5	882	678	27930	624	303	1013	144	66	248	124	159	273.66	249	437.93	489	407	441	26	89	14	4	3
14352	7712	4408	6	752	570	27854	611	331	1107	170	54	275	126	152	269.8	282	432.48	496	420	437	19	95	17	6	2
14452	7586	4290	3	858	644	27882	614	329	1051	163	50	264	120	146	271.06	282	434.1	518	409	436	17	95	23	2	0
12402	7988	5018	1	1544	1160	28292	592	137	805	39	170	180	71	241	292.98	176	449.19	495	299	416	45	102	12	6	6
13710	7846	4568	2	988	784	27996	601	275	1011	124	90	227	119	180	272.94	233	438.46	475	399	475	36	86	14	10	0
12932	8004	4920	1	1258	900	28116	669	179	937	57	92	200	98	177	281.4	191	446.97	473	377	420	42	107	8	10	6
5056	12792	9210	657	658	414	28160	1120	11	620	1	30	50	9	91	280.72	78	438.17	365	341	343	19	26	0	0	5

SUPPLEMENTARY INFORMATION:Monte Carlo Atomistic Simulation and Machine Learning Analysis of Na-K Eutectic Alloy in Condensed Phases, D. Reitz and E. Blaisten-Barojas, George Mason University, Fairfax, VA 22030

11488	7726	5344	2	2074	1578	28612	541	87	588	24	372	187	42	250	314.79	119	459.3	480	190	371	118	98	4	28	22
12468	7968	5024	1	1500	1124	28272	621	160	796	40	176	178	92	204	284.54	182	449.42	511	297	412	49	107	9	12	5
3856	13724	9974	972	312	176	28052	1301	3	561	0	10	60	2	68	271.15	81	423.35	277	277	278	13	39	0	0	1
7256	11164	7886	354	1092	766	28278	914	46	707	14	108	92	25	154	289.44	129	446.35	421	355	366	36	41	1	6	6
4008	13588	9886	960	370	202	28062	1296	9	564	1	8	56	4	64	270.22	90	422.33	285	268	274	16	39	0	0	2
4148	13536	9788	906	348	226	28072	1250	7	580	1	24	63	5	68	271.7	98	424.96	309	280	289	14	36	0	2	3
4022	13618	9866	959	346	200	28056	1289	5	572	1	4	68	2	59	270.88	98	423.5	293	268	266	18	46	0	0	2
3850	13696	9964	959	344	194	28056	1288	8	561	1	8	63	3	68	271.06	85	423.08	289	276	270	12	43	0	0	3
6154	12146	8676	474	706	430	28156	1015	11	777	1	42	84	6	114	286.74	131	442.33	402	395	352	22	51	1	2	2
13004	7870	4810	5	1310	1008	28162	614	217	861	68	144	188	105	187	282.63	206	447.41	498	323	425	50	98	16	14	8
4256	13384	9698	848	450	286	28104	1238	3	597	0	30	57	2	89	274.5	79	429.39	292	303	302	17	40	0	0	2
4864	13052	9364	726	504	316	28118	1171	1	678	0	18	62	1	100	276.76	107	431.75	328	343	317	15	46	0	0	1
4050	13620	9880	950	316	174	28048	1311	6	577	1	8	63	3	68	272.23	82	426.89	276	292	261	11	41	0	0	3
13606	7898	4638	5	1002	762	27986	578	273	996	123	74	218	116	174	273.95	254	437.8	492	364	492	32	73	12	6	3
4682	12978	9458	751	624	366	28142	1187	10	601	1	30	68	3	95	278.46	86	434.25	342	305	280	15	43	0	4	3
5786	12384	8900	551	670	376	28138	1030	17	728	2	22	76	11	110	282.02	123	440.97	394	350	363	20	52	0	0	0
11456	7862	5404	3	1980	1492	28546	552	116	648	18	300	164	55	248	313.86	124	456.04	472	211	385	115	102	13	48	20
4866	12974	9348	708	576	350	28132	1158	6	642	0	18	63	5	98	278.52	84	434.2	332	344	325	20	35	0	0	1
14314	7706	4420	5	786	584	27870	703	324	1072	165	60	255	117	157	272.86	233	436.43	435	479	420	20	80	21	0	3
11784	7960	5298	2	1794	1332	28440	524	123	691	27	242	174	68	255	299.76	155	454.36	510	229	416	76	102	9	22	12
11536	7716	5340	1	2046	1568	28618	533	129	541	8	366	149	82	282	313.01	95	460.54	486	199	395	91	83	6	42	25
14348	7664	4394	4	778	606	27876	651	334	1063	170	82	255	121	163	268.44	267	430.02	475	440	434	13	79	21	4	1
5294	12612	9144	610	696	386	28154	1106	8	689	0	22	69	4	85	281.81	108	439.31	397	367	316	18	43	0	0	2
4724	13122	9468	744	486	286	28102	1162	6	636	0	16	64	4	101	277.19	88	434.08	321	320	325	19	43	0	0	0
4556	13050	9510	782	630	372	28148	1212	5	603	0	28	62	4	101	279.11	83	435.22	319	310	289	14	38	0	2	1
14056	7724	4490	6	896	676	27926	631	320	1006	146	84	237	130	150	272.21	249	436.19	504	399	451	19	82	19	0	2
7458	11128	7842	350	1016	696	28242	923	47	755	11	92	83	25	131	287.63	127	446.23	428	373	376	36	53	2	10	8
14290	7608	4394	6	886	654	27904	646	312	1021	162	62	270	113	172	271.03	243	434.93	469	419	417	14	91	20	10	6
4196	13500	9784	890	368	208	28066	1248	6	610	1	10	64	4	61	273.92	93	426.35	310	295	293	20	47	0	0	1
4406	13354	9654	872	406	244	28084	1249	12	612	4	20	61	8	62	274.45	117	429.51	327	292	277	15	35	0	0	2
13756	7836	4638	1	938	704	27972	657	278	1028	124	94	223	119	169	273.92	240	437.38	472	424	446	22	87	17	6	3
4750	13122	9460	744	464	280	28098	1178	5	672	0	20	56	3	74	275.71	116	429.3	350	343	320	14	38	0	2	1
5026	12948	9308	675	498	306	28114	1167	9	729	2	26	59	5	93	277.72	114	433.47	335	394	322	14	39	0	0	3
14194	7740	4400	7	854	662	27912	659	313	1019	160	60	250	113	152	271.15	247	434.39	479	422	433	18	78	23	2	5
3870	13674	9956	988	350	198	28060	1302	8	548	1	10	60	7	66	272.35	88	424.64	278	253	273	13	35	0	2	4
13470	7802	4644	4	1150	882	28068	617	246	914	114	106	215	98	212	276.35	206	444.22	465	359	446	27	85	18	14	4

3896	13674	9934	972	348	200	28060	1299	9	547	1	8	59	6	67	269.91	91	422.04	284	265	270	12	36	0	0	3
14184	7754	4448	6	832	620	27890	672	312	1049	165	48	266	113	161	270.01	266	431.75	471	424	408	16	86	17	4	1
4250	13504	9746	904	346	208	28064	1247	10	606	1	10	65	6	68	272.86	114	426.29	324	274	283	7	42	0	0	2
4392	13274	9650	841	492	276	28098	1212	6	590	0	14	64	5	82	276.59	85	431.64	319	286	298	17	47	0	0	2
3844	13710	9990	980	324	172	28048	1293	9	566	2	8	54	5	66	270.44	96	423.39	278	257	285	16	39	0	0	1
13976	7766	4502	8	914	704	27946	636	295	1017	142	74	249	118	163	273.36	244	438.32	485	409	436	27	93	14	10	0
4208	13528	9792	911	326	186	28052	1261	12	595	2	12	53	3	61	271.98	94	427.07	302	295	296	15	36	1	0	3
3998	13588	9854	950	396	234	28076	1291	8	566	2	6	58	5	73	270.68	91	423.77	286	275	275	12	36	0	0	2
4564	13196	9578	781	480	264	28098	1185	7	626	0	16	60	2	73	277.34	103	433.62	335	304	315	19	46	0	0	5
4804	12954	9366	713	618	380	28150	1177	4	637	1	26	54	3	81	279.15	101	435.75	356	347	313	16	33	0	2	2
5866	12270	8810	585	728	436	28142	1063	40	655	15	30	91	21	99	281.53	120	438.31	399	338	320	18	53	0	2	1
14370	7668	4390	7	814	582	27864	612	320	1061	166	38	259	119	150	270.37	276	432.44	497	405	455	19	85	20	2	1
14222	7632	4358	5	916	712	27918	645	313	993	158	76	252	109	164	272.25	247	435.6	479	401	429	22	83	22	2	1
6228	12038	8644	475	760	450	28172	1023	15	758	3	50	77	6	114	285.75	102	443.38	387	395	356	29	49	0	2	2
7446	11222	7918	344	986	598	28214	901	25	814	3	42	87	16	141	288.08	148	446.31	449	369	389	23	51	3	2	1
6938	11490	8196	369	920	588	28218	948	34	773	9	84	83	16	137	287.09	134	446.3	422	392	365	26	52	0	2	5
12176	7950	5106	0	1626	1258	28370	586	130	762	38	228	168	63	258	298.53	157	452.09	494	269	413	49	91	11	24	9
11294	7860	5374	1	2080	1630	28636	518	95	567	15	344	176	52	310	318.99	105	458.95	444	182	376	114	96	11	52	16
3946	13696	9946	976	290	156	28042	1294	5	584	1	8	68	2	68	271.58	97	424.28	281	270	271	12	46	1	0	2
11784	8152	5290	2	1616	1288	28422	549	114	742	35	266	186	61	256	301.63	153	453.05	488	262	418	75	98	4	24	9
13702	7774	4616	2	1056	766	28000	619	242	1022	116	82	238	97	183	275.18	237	439.32	483	399	441	27	100	17	4	2
4472	13296	9592	788	452	276	28104	1174	3	626	0	16	58	2	89	276.97	108	432.52	334	295	323	15	39	0	0	1
3876	13654	9938	966	378	214	28068	1290	11	537	2	8	59	6	71	271.81	83	424.16	287	258	272	13	35	0	0	2
4546	13302	9590	835	396	230	28074	1223	7	636	1	10	64	4	74	275.63	104	430.43	313	313	307	14	43	0	0	2
7122	11332	8072	365	1010	626	28228	950	36	752	14	64	97	18	126	287.91	129	444.29	437	381	345	32	63	0	2	3
13802	7718	4548	4	1022	788	27996	624	282	959	121	114	218	119	181	277.37	226	442.6	467	378	462	33	80	12	4	2
12126	7900	5118	2	1684	1292	28384	531	137	727	36	240	179	62	238	301.33	162	454.49	505	243	415	84	96	4	22	8
4212	13550	9798	877	312	172	28050	1250	6	626	1	6	64	4	67	273.12	108	427.08	305	313	294	12	43	0	0	3
4208	13472	9784	897	376	208	28060	1242	12	584	1	12	52	5	64	271.46	104	424.92	315	272	304	11	31	0	0	4
13682	7746	4596	6	1092	804	28014	642	261	948	121	88	225	105	187	276.06	216	440.7	459	370	437	33	88	21	6	3
4422	13436	9688	876	316	178	28048	1250	11	640	2	8	59	7	60	272.45	104	425.48	312	316	297	15	40	0	0	1
12614	7912	4922	2	1472	1148	28272	589	152	819	49	182	184	81	232	294.05	181	450.83	483	301	418	54	104	3	22	10
5340	12638	9132	657	624	364	28134	1127	10	692	3	36	74	5	86	285.47	112	440.11	371	352	307	22	49	1	0	3
5514	12630	9018	602	582	360	28128	1119	11	735	1	24	72	8	93	279.54	121	435.32	370	386	319	17	46	0	0	2
5688	12522	8980	561	562	334	28128	1064	15	768	3	38	78	9	91	280.08	131	438.23	401	398	339	19	51	0	4	1
5354	12756	9170	621	522	288	28108	1085	9	742	1	18	57	5	82	282.28	116	438.74	381	376	363	18	38	0	0	3

SUPPLEMENTARY INFORMATION:Monte Carlo Atomistic Simulation and Machine Learning Analysis of Na-K Eutectic Alloy in Condensed Phases, D. Reitz and E. Blaisten-Barojas, George Mason University, Fairfax, VA 22030

11190	7818	5424	0	2198	1646	28640	501	80	582	23	316	169	35	271	311.69	116	460.52	506	173	388	97	87	1	46	29
14236	7730	4506	8	778	550	27860	653	326	1069	142	60	269	139	157	270.8	259	435.92	456	437	427	27	111	21	0	1
11572	7850	5370	3	1994	1428	28494	541	123	625	26	240	180	66	254	301.37	127	454.75	503	211	390	86	105	8	38	15
12224	8012	5070	1	1564	1240	28364	596	137	742	32	242	178	75	236	300.04	137	451.27	471	282	406	72	95	10	12	13
5102	12860	9270	664	554	318	28124	1144	4	726	0	18	57	3	100	276.62	101	433.15	343	373	336	13	42	0	2	2
12298	7970	5036	3	1566	1236	28348	584	162	755	41	216	176	83	237	291.38	151	451.04	476	271	426	72	91	13	24	7
4522	13178	9544	798	542	320	28120	1198	5	593	1	14	57	2	86	277.21	93	432.87	331	294	306	16	37	0	0	1
4988	12832	9284	710	646	378	28148	1165	7	640	0	16	65	6	86	280.09	98	436.29	348	325	307	23	46	0	4	2
14156	7734	4438	6	858	654	27906	648	303	1062	165	62	255	103	157	267.88	277	429.47	493	434	426	14	80	16	4	2
14588	7542	4316	7	802	556	27846	657	358	1072	169	40	274	136	137	271.16	272	434.37	473	436	418	29	94	28	2	2
10964	7906	5578	2	2128	1658	28694	513	82	574	14	416	160	45	320	318.34	111	465.23	459	188	368	109	76	5	42	19
14274	7736	4422	9	774	602	27880	622	306	1087	148	68	245	120	148	269.56	272	432.51	497	432	466	17	80	17	2	1
7534	11104	7830	329	1012	670	28242	922	46	788	9	84	90	28	132	287.44	141	445.89	463	388	351	24	60	2	8	4
14346	7700	4374	6	804	606	27884	644	345	1026	163	54	248	137	140	270.74	276	434.99	501	418	439	19	77	24	0	0
12498	7532	4860	3	1840	1402	28412	564	169	636	45	256	176	81	246	299.51	120	453.36	477	222	409	82	89	12	24	13
4778	13020	9404	719	552	342	28126	1154	6	642	0	30	59	3	95	277.78	87	434.1	332	323	322	23	39	0	0	3
12390	7850	5120	2	1574	1140	28304	599	154	802	44	216	197	83	237	295	166	451.92	473	278	401	56	113	9	12	12
13476	7924	4694	5	1046	790	28022	641	239	984	119	88	220	101	176	276.24	234	442.55	475	388	447	31	82	7	4	3
14136	7692	4410	5	882	706	27920	642	315	1008	156	90	245	125	172	271.45	253	435.72	461	407	450	22	73	20	4	2
3904	13678	9924	951	344	204	28064	1281	7	567	1	10	65	5	69	270.89	85	424.23	294	276	272	14	43	0	0	1
4222	13430	9728	881	434	260	28086	1251	5	585	1	12	57	3	94	274.49	80	428.46	270	294	305	17	36	0	0	2
4176	13448	9778	890	424	242	28084	1256	8	579	1	14	59	6	80	271.47	83	427.1	293	287	287	15	41	0	2	3
13160	7682	4700	4	1408	1064	28180	603	227	823	77	154	200	111	211	282.7	180	448.87	490	306	429	43	93	14	12	6
5330	12720	9146	636	572	330	28118	1109	9	707	1	20	71	6	103	280.15	110	438.61	361	361	335	14	47	0	0	1
12818	7958	4814	1	1352	1088	28200	673	194	856	71	152	180	87	219	282.37	186	446.42	447	339	421	39	85	14	18	5
7712	10992	7760	333	1028	652	28224	929	54	781	8	80	86	36	135	289.47	128	448.9	430	393	368	30	58	1	0	6
4056	13568	9864	951	368	196	28054	1296	10	583	2	2	60	4	69	271.02	81	423.55	277	286	275	14	40	0	0	3
3902	13740	9960	983	272	154	28034	1333	6	602	1	6	61	3	70	270.13	87	422.91	262	298	254	9	43	0	0	2
4790	13080	9458	736	480	270	28096	1152	10	642	1	18	67	6	86	276.63	101	433.33	344	328	327	13	42	0	0	5
14518	7582	4340	8	790	568	27852	613	338	1060	157	50	274	137	146	272.69	262	436.62	499	425	434	22	107	16	4	2
5220	12812	9176	627	560	340	28124	1074	9	711	0	16	76	8	72	279.63	132	436.5	416	342	331	21	46	0	0	2
11034	7894	5462	0	2212	1702	28698	518	76	518	15	336	154	37	303	317.77	106	465.17	474	169	368	114	66	2	52	19
4216	13410	9756	848	448	248	28090	1222	4	601	0	12	65	4	69	274.18	86	430	331	304	292	13	40	0	0	3
13402	7912	4710	7	1090	818	28024	641	264	948	113	90	228	116	208	275.88	193	444.57	432	382	438	36	93	15	2	2
11864	7908	5222	1	1848	1366	28478	544	121	663	27	254	167	65	260	306.17	137	458.18	493	228	397	86	81	7	14	13
4694	13206	9532	793	418	222	28074	1213	7	664	0	0	64	5	80	277.1	95	433.02	313	336	308	16	48	0	2	1

SUPPLEMENTARY INFORMATION:Monte Carlo Atomistic Simulation and Machine Learning Analysis of Na-K Eutectic Alloy in Condensed Phases, D. Reitz and E. Blaisten-Barojas, George Mason University, Fairfax, VA 22030

11924	7870	5084	1	1838	1460	28476	557	129	642	31	268	173	68	251	302.45	140	455.71	501	214	396	82	85	13	30	12
5040	12766	9266	654	672	384	28154	1100	4	654	0	26	67	3	94	279.1	112	435.41	392	321	326	14	43	0	0	2
14540	7730	4368	2	642	486	27810	681	335	1137	170	40	256	129	140	268.07	270	429.44	452	499	448	18	74	18	4	0
6918	11436	8154	355	1026	652	28262	946	32	753	6	72	71	23	117	287.58	134	443.63	453	383	373	35	47	0	4	0
5180	12728	9198	633	622	378	28150	1111	5	685	0	44	58	3	91	279.84	97	438.41	367	368	342	22	35	0	0	3
4616	13136	9522	771	526	298	28112	1196	10	621	0	14	66	6	97	277.26	92	432.76	321	327	298	12	46	1	0	3
13606	7816	4676	4	1058	770	28026	651	274	951	117	90	219	121	177	277.83	218	443.89	469	385	440	32	87	15	10	3
4786	12944	9426	703	612	346	28144	1145	5	636	0	26	47	3	95	277.69	97	432.35	350	318	334	20	31	0	4	0
4032	13594	9872	945	354	198	28058	1306	9	588	1	8	53	4	64	270.47	90	423.54	284	293	273	13	37	0	0	1
12518	7834	5006	1	1558	1170	28314	578	162	762	40	206	172	82	232	293.08	149	451.81	495	276	429	57	95	17	18	10
10940	7874	5592	2	2218	1644	28670	532	70	547	12	372	171	38	309	320.82	91	463.49	455	187	360	97	96	6	26	27
4536	13294	9592	824	420	236	28082	1234	4	633	0	4	62	3	78	276.65	110	431.34	317	317	294	11	40	0	0	1
11300	7948	5528	1	2040	1440	28558	514	104	607	17	270	177	59	252	312.71	126	462.17	518	203	373	102	83	10	32	16
4788	13106	9460	747	468	262	28096	1182	4	683	0	10	57	3	72	276.75	99	431.31	345	351	322	16	43	0	2	2
6986	11470	8154	356	950	602	28234	915	23	777	2	66	91	15	146	288.57	123	444.91	436	386	369	30	61	0	6	1
3796	13750	10006	985	322	174	28052	1299	6	557	1	4	60	2	64	271.03	94	423.95	294	254	266	10	38	0	0	1
11712	7882	5210	0	1910	1502	28558	546	123	623	25	302	166	71	281	312.06	137	460.68	463	200	400	90	84	8	36	18
4754	13006	9440	749	552	334	28128	1140	6	628	0	42	71	3	98	278.13	103	434.69	351	289	310	17	49	0	0	2
4448	13262	9626	840	478	268	28094	1225	7	608	0	10	58	3	75	276.18	97	430.75	314	294	299	21	40	0	2	1
4708	13136	9506	753	466	260	28092	1180	7	660	0	14	72	4	77	278.45	117	434.05	352	338	300	11	50	0	2	3
5290	12684	9096	614	644	410	28158	1102	7	651	2	34	80	5	100	279.96	100	436.91	372	353	321	18	46	0	0	5
4324	13442	9722	887	350	206	28062	1252	9	613	1	18	61	7	73	274.2	90	428.35	299	307	299	11	45	0	0	3
14322	7678	4428	8	802	582	27872	626	342	1038	150	58	258	139	156	272.2	260	436.65	484	411	446	18	95	28	2	3
14396	7736	4422	6	718	526	27846	648	350	1096	168	44	254	147	151	267.57	270	429.54	474	483	450	15	78	14	4	1
5904	12180	8770	546	784	484	28178	1073	22	718	4	56	58	11	104	283.25	107	438.63	386	382	338	26	34	0	0	1
6138	12128	8626	514	742	492	28188	1035	17	740	3	62	74	12	102	287.01	115	442.44	408	380	346	28	46	0	0	1
13852	7530	4434	1	1198	916	28062	577	261	898	103	126	230	124	194	277.18	234	444.88	507	318	430	36	107	11	6	4
13496	7780	4682	2	1138	838	28046	625	254	940	112	102	234	104	183	276.2	216	442.08	467	358	434	43	98	18	8	4
4098	13488	9814	898	428	242	28082	1260	5	581	1	12	57	4	71	272.53	85	426.72	291	288	299	19	35	0	0	2
14018	7798	4476	6	876	688	27932	599	290	1019	141	70	252	111	172	274.19	226	440.2	475	397	459	33	87	13	6	1
14276	7658	4400	6	850	640	27892	633	305	1061	161	58	273	110	144	271.59	271	438.28	504	417	416	23	99	19	10	2
3914	13572	9918	961	432	234	28080	1284	7	540	1	10	50	4	69	271.47	87	423.41	287	253	287	15	33	0	0	3
14174	7756	4488	6	832	592	27888	635	311	1071	152	44	246	122	141	271.77	284	433.73	509	425	444	18	84	16	2	0
14278	7638	4406	1	884	636	27898	579	305	1027	150	54	254	121	155	273.94	276	437.03	523	366	462	17	83	17	2	2
12972	7692	4686	0	1502	1198	28254	562	203	771	75	178	204	90	218	285.57	182	448.34	506	262	432	55	98	13	24	8
13982	7850	4550	4	848	636	27936	663	288	1066	127	68	231	122	153	276.44	269	440.09	485	434	430	24	92	16	2	1

SUPPLEMENTARY INFORMATION:Monte Carlo Atomistic Simulation and Machine Learning Analysis of Na-K Eutectic Alloy in Condensed Phases, D. Reitz and E. Blaisten-Barojas, George Mason University, Fairfax, VA 22030

3868	13660	9976	985	346	188	28056	1303	6	557	1	16	65	2	63	270.6	92	424.35	280	255	265	16	41	0	2	3
4354	13316	9664	818	486	274	28102	1196	5	589	0	6	63	4	86	276.86	93	432.45	337	287	299	9	39	0	2	3
4686	13152	9510	768	466	264	28094	1188	3	642	1	16	51	1	80	277.9	81	432.52	332	333	321	16	31	0	0	2
5202	12790	9226	677	554	316	28112	1122	16	682	0	24	68	10	98	281.52	110	439.02	359	345	331	15	44	0	0	0
5204	12754	9218	623	604	332	28128	1141	10	717	1	16	69	7	97	279.48	94	434.89	346	401	313	19	43	0	0	4
14576	7620	4306	3	752	548	27842	647	326	1088	160	40	262	119	144	272.02	268	433.99	491	446	435	13	94	18	0	1
11112	7864	5480	1	2168	1638	28642	494	65	574	9	328	174	37	294	316.81	107	465.99	471	177	371	118	95	2	46	23
12044	8062	5258	4	1600	1164	28344	599	111	790	32	200	190	51	233	300.11	151	453.84	477	296	397	66	106	8	12	13
14334	7700	4394	5	800	594	27872	639	322	1076	162	46	264	126	141	268.15	283	430.04	506	431	426	16	90	14	4	2
4994	12948	9300	687	532	330	28128	1144	4	681	0	20	50	4	88	277.14	108	432.31	357	352	345	10	26	0	4	4
11282	8002	5518	2	1984	1458	28580	559	85	618	12	300	169	48	306	315.81	106	460.5	459	223	359	78	85	4	36	22
4910	13016	9346	688	502	320	28124	1139	5	687	0	30	66	4	97	277.1	113	433.33	358	353	320	14	39	0	0	0
3886	13724	9966	1013	288	166	28042	1333	5	575	1	10	53	2	62	270.16	97	423.55	264	268	269	11	39	0	0	3
12932	8044	4932	8	1190	888	28116	635	192	952	74	120	205	88	186	281.53	226	444.46	495	349	424	38	111	11	10	3
3924	13656	9936	986	342	186	28050	1321	6	570	1	6	70	4	69	270.47	93	422.43	268	274	253	11	49	0	0	4
7108	11486	8084	350	882	580	28204	937	28	816	7	58	90	18	130	290.44	121	445.81	424	421	380	27	60	1	6	5
13076	7834	4828	3	1302	958	28148	585	214	855	70	146	191	106	214	281.85	182	448.75	459	314	474	52	86	18	4	4
7360	11020	7894	292	1210	738	28296	922	23	774	4	70	74	10	132	287.09	124	444.75	437	395	371	45	48	1	4	4
10626	7616	5630	0	2492	1950	28898	484	58	416	8	514	151	32	315	331.35	75	473.33	466	123	366	146	64	1	66	30
3930	13740	9956	989	256	142	28030	1308	9	577	1	6	59	6	61	271.33	87	424.22	275	276	279	13	39	0	0	1
13158	8010	4808	7	1098	868	28076	615	245	935	111	122	220	105	177	277.97	216	443.12	505	372	436	37	91	13	12	2
4088	13540	9824	917	388	224	28074	1277	6	587	1	10	55	3	71	271.93	79	425.24	290	298	286	11	38	0	0	5
4342	13448	9718	870	342	196	28058	1260	7	632	1	12	53	4	77	272.38	88	425.2	287	318	305	10	36	0	0	2
6222	11922	8602	484	862	528	28206	1039	25	733	6	64	82	16	132	285.47	112	441.62	374	373	340	26	48	1	4	1
4976	13062	9350	710	426	262	28094	1180	5	710	1	18	53	3	76	278.14	110	433.91	337	372	334	14	37	0	0	3
4604	13204	9572	809	446	242	28082	1240	9	660	2	14	65	6	82	275.55	88	429.67	302	356	291	14	46	0	0	2
11746	7916	5396	1	1810	1304	28474	526	120	662	19	268	175	65	276	311.97	107	458.35	464	230	411	94	103	7	32	14
4130	13516	9790	900	390	236	28076	1255	9	575	1	14	62	8	70	275.02	93	427.67	283	286	296	28	39	0	0	1
13514	7578	4634	3	1312	946	28128	639	219	866	88	124	223	95	183	284.76	174	447.33	472	335	406	54	109	17	20	6
5228	12746	9174	648	608	360	28140	1132	6	717	0	24	66	5	90	281.09	110	439.34	364	358	314	18	42	0	0	4
6786	11480	8152	427	1008	720	28262	962	29	700	4	108	82	17	143	288.57	121	443.92	411	350	348	33	48	2	6	6
13722	7806	4650	2	1016	712	27974	654	267	989	120	62	231	117	164	275.86	216	440.45	466	410	439	33	91	13	6	3
4972	12896	9302	668	590	354	28138	1123	3	672	0	24	59	2	80	280.21	106	435.36	372	336	332	24	43	0	0	2
5338	12612	9132	661	650	372	28138	1157	9	713	1	34	71	4	112	280.06	119	437.72	335	352	300	16	51	1	0	0
4542	13218	9584	808	466	268	28102	1192	8	629	0	24	61	6	77	277.18	100	432.96	325	302	314	23	41	0	0	2
3870	13724	9970	996	304	170	28046	1332	6	555	1	8	57	4	68	271.38	81	424.15	261	279	262	12	33	0	0	2

SUPPLEMENTARY INFORMATION:Monte Carlo Atomistic Simulation and Machine Learning Analysis of Na-K Eutectic Alloy in Condensed Phases, D. Reitz and E. Blaisten-Barojas, George Mason University, Fairfax, VA 22030

10482	7958	5764	1	2306	1798	28818	503	48	495	7	442	167	24	273	331.29	105	470.94	497	139	343	136	79	2	60	32
5218	12692	9158	643	662	392	28146	1110	7	677	0	24	61	3	86	282.27	113	437.2	373	335	331	24	35	0	0	4
14396	7590	4346	5	858	638	27896	645	314	1050	157	60	259	116	156	273.72	257	436.35	469	416	438	25	93	24	8	3
14200	7700	4460	6	848	618	27888	625	318	1058	150	60	247	131	161	270.53	262	433.08	474	415	463	22	85	18	2	1
5406	12608	9082	591	652	372	28136	1095	11	695	1	14	73	5	100	281.14	89	436.89	370	383	332	19	47	0	2	3
4484	13226	9610	809	504	272	28104	1194	6	604	0	8	64	6	76	277.89	100	432.94	339	289	308	13	45	0	0	4
14020	7756	4504	4	898	678	27934	636	301	1016	135	70	235	120	158	274.34	243	439.36	470	411	454	31	81	20	8	4
4226	13408	9754	863	454	246	28096	1247	3	575	0	6	59	3	66	276.52	90	429.93	317	298	282	20	33	0	2	2
10962	7886	5592	1	2216	1640	28692	507	77	522	8	338	169	37	306	317.72	100	463.26	455	170	374	113	93	7	58	25
4172	13454	9778	893	424	240	28080	1281	8	598	2	12	57	4	67	271.77	87	425.95	298	315	277	13	33	0	0	3
4072	13622	9868	955	312	168	28042	1321	9	600	1	0	66	5	68	271.7	84	424.51	265	305	260	12	48	0	0	3
4394	13346	9654	832	424	250	28082	1229	7	633	1	14	64	4	71	273.25	111	427.15	315	313	292	22	46	0	0	0
4050	13562	9846	924	390	220	28074	1279	3	572	0	6	61	2	64	271.71	92	425.76	302	284	270	17	37	0	0	1
13748	7772	4566	6	1022	786	27998	615	262	978	119	96	235	110	180	276.43	237	441.72	481	382	443	30	91	15	8	4
4062	13534	9836	914	400	230	28074	1274	7	565	1	12	63	5	74	273.08	75	426.36	281	298	284	20	43	0	0	1
14358	7690	4406	6	774	576	27864	625	325	1071	157	56	255	127	152	271.28	261	434.18	483	418	458	21	87	20	4	0
13808	7892	4632	7	862	656	27936	625	281	1053	123	80	234	121	176	274.85	249	438.31	469	413	453	24	98	14	6	3
12552	7876	4962	0	1516	1174	28308	589	166	789	47	204	182	92	208	287.96	183	449.25	522	289	417	57	98	13	22	8
14272	7592	4352	8	940	696	27914	641	311	1006	164	62	260	116	167	270.34	233	433.15	480	411	429	16	88	13	0	2
4110	13620	9852	952	292	164	28044	1281	4	607	0	4	65	2	69	271.38	103	424.53	286	286	283	10	43	0	2	3
5240	12698	9118	633	638	420	28162	1148	8	700	1	46	57	2	96	279.51	101	435.49	359	388	314	17	36	0	2	1
13014	7822	4764	1	1366	1064	28206	593	204	845	61	160	187	110	212	287.2	179	447.95	476	313	448	52	96	10	16	10
14390	7660	4340	4	798	620	27872	645	330	1060	163	60	252	121	159	269.33	249	432.46	459	442	449	25	77	17	4	1
11564	7974	5316	2	1904	1448	28514	529	100	631	26	290	182	48	279	310.04	118	459.24	467	210	402	86	96	9	16	21
11274	7758	5436	0	2166	1610	28632	544	93	573	11	354	176	55	303	315.82	105	461.08	426	191	375	99	98	7	34	33
4302	13416	9718	854	398	228	28074	1228	4	616	0	12	67	3	81	271.63	99	427.26	308	309	297	13	46	0	0	2
6196	12062	8586	547	778	506	28180	1044	34	698	13	48	76	16	122	284.07	109	442.77	386	341	341	18	47	0	4	4
4414	13312	9662	829	442	242	28082	1217	3	608	0	8	68	3	71	274.44	80	429.48	322	317	299	17	45	0	2	3
13704	7618	4502	2	1186	926	28078	635	290	851	108	126	211	121	193	278.5	190	445.09	474	337	424	41	86	28	16	1
6072	12224	8712	532	670	428	28158	1058	20	752	3	46	68	11	120	286.13	98	443.74	368	405	355	19	48	0	6	3
13570	7902	4722	6	968	714	27970	625	273	1016	119	94	229	111	158	275.65	237	440.56	482	393	450	38	100	17	0	3
4466	13204	9560	786	550	332	28128	1198	1	608	0	16	60	1	79	275.75	95	431.82	327	315	304	26	36	0	0	1
5294	12670	9112	631	634	400	28152	1089	12	687	0	42	70	6	103	280.42	117	438.06	381	336	329	15	48	0	0	4
4198	13492	9764	853	384	226	28074	1231	7	605	1	10	72	5	73	273.3	98	427.87	326	303	283	10	48	0	0	2
12176	7954	5128	1	1624	1232	28352	592	150	756	41	212	160	75	227	298.64	170	453.82	478	291	425	77	75	7	24	8
3944	13630	9900	966	364	218	28072	1291	4	549	1	14	64	2	59	272.23	86	424.64	303	257	263	17	39	0	2	0

10722	7866	5730	1	2268	1706	28768	516	65	508	11	406	151	28	324	326.23	91	467.22	472	182	361	104	75	4	62	26
5052	12916	9312	664	528	292	28112	1136	3	699	0	12	56	0	93	279.02	102	435.4	344	362	345	16	39	0	0	3
11160	7964	5562	2	2036	1520	28628	516	80	564	15	352	150	41	288	315.23	95	462.94	482	204	398	107	80	4	32	16
11960	8016	5212	1	1728	1266	28398	523	125	694	23	194	171	68	266	301.68	133	455.52	478	213	434	77	108	10	22	10
4176	13612	9828	922	266	146	28030	1272	8	600	2	2	72	4	78	272.77	84	427.91	278	301	283	9	44	0	0	3
4628	13268	9608	812	362	178	28052	1198	8	685	0	8	58	5	75	277.37	105	433.03	311	335	330	18	43	0	0	2
5222	12802	9212	637	546	312	28112	1081	10	725	0	18	62	6	89	279.6	124	436.92	377	346	359	18	43	1	0	4
12868	7830	4854	3	1424	1074	28230	615	184	824	57	168	175	91	208	286.37	171	449.03	481	300	447	47	93	18	10	9
4284	13434	9740	887	388	212	28064	1256	9	599	3	6	67	5	67	272.52	96	428.17	296	308	289	15	46	0	0	6
4274	13240	9678	828	586	334	28134	1246	4	559	0	20	62	2	80	275.32	84	430.63	325	309	268	14	42	0	2	2
11942	7784	5182	1	1876	1396	28472	557	130	630	29	260	185	66	261	313.64	124	459.08	466	215	379	86	94	7	30	21
10608	7806	5654	0	2388	1856	28832	502	69	438	12	468	177	35	308	334.33	71	472.12	469	141	347	127	89	6	48	34
4804	12972	9446	706	576	304	28118	1148	6	664	0	16	64	2	95	278.74	99	434.16	348	345	320	15	49	0	0	2
14378	7674	4378	6	786	590	27864	650	329	1065	164	56	260	125	160	268.27	265	431.26	469	435	437	14	81	16	2	3
4260	13410	9740	895	420	236	28080	1247	10	595	3	14	73	6	68	272.55	105	426.91	314	280	273	16	44	0	0	4
6082	12316	8754	568	564	352	28108	1041	28	776	12	38	78	15	103	281.96	126	439.28	380	364	372	18	52	0	2	3
3790	13708	10002	1010	356	190	28050	1315	7	555	1	4	61	4	71	269.56	96	422.24	259	249	271	15	44	0	0	3
4176	13544	9800	904	338	192	28056	1276	7	603	1	4	55	6	71	273.81	84	427.63	278	309	296	18	36	0	2	1
3952	13616	9880	959	392	232	28080	1311	2	565	0	8	56	1	69	271.71	89	423.77	278	283	268	14	39	0	0	2
4054	13566	9846	916	378	216	28068	1278	5	573	1	8	56	2	81	273.17	86	427.74	287	293	282	8	34	0	0	2
4972	13010	9394	686	448	244	28090	1105	6	724	0	22	64	2	89	279.1	130	434.12	372	346	352	10	44	0	0	2
14054	7830	4472	4	828	660	27916	658	286	1069	150	66	254	100	152	271.32	266	435.32	495	431	416	17	92	19	6	2
4130	13532	9814	907	366	212	28068	1275	9	582	1	14	61	5	68	270.71	77	424.18	292	297	282	16	38	0	0	2
14308	7672	4328	6	844	680	27908	673	330	1016	160	68	257	121	135	272.02	255	434.41	481	430	414	33	78	21	8	1
4162	13516	9806	905	358	208	28068	1271	6	606	2	14	61	4	70	271.88	93	424.79	294	296	285	14	43	0	4	2
14616	7554	4298	5	758	560	27852	643	326	1061	158	64	278	121	145	271.53	276	434.52	500	427	409	17	108	26	2	0
4436	13300	9640	831	444	254	28090	1214	9	606	0	14	67	6	80	274.75	92	430.47	313	301	302	15	50	0	2	5
4974	12966	9346	722	506	292	28102	1161	11	675	1	18	72	6	88	279.12	99	434.99	335	343	315	23	51	0	0	0
12350	7892	4964	2	1632	1276	28338	582	167	717	40	200	163	99	237	292.91	143	449.31	492	270	436	48	85	7	22	13
13798	7754	4568	8	990	768	28000	605	270	973	117	118	231	120	171	277.48	234	443.28	508	363	443	28	98	14	4	2
4576	13248	9582	793	428	236	28080	1212	8	635	3	10	67	3	80	274.51	88	429.78	318	344	297	15	41	0	0	3
4658	13164	9510	736	480	278	28106	1176	3	665	1	16	62	1	91	278.63	100	432.34	338	340	314	10	45	0	0	3
4254	13406	9736	846	432	242	28080	1234	8	600	1	10	62	4	64	275.65	98	429.78	325	316	289	15	43	0	0	4
12580	7814	4874	3	1602	1238	28318	543	148	757	39	190	193	76	240	293.37	165	450.3	503	248	423	65	109	10	20	6
5500	12418	9006	617	736	448	28166	1072	18	658	5	54	76	8	108	285.29	115	439.29	379	322	330	18	48	0	4	5
12686	8044	5024	2	1310	960	28166	616	167	917	40	124	178	97	185	287.82	204	449.85	489	347	453	45	106	11	16	10

SUPPLEMENTARY INFORMATION:Monte Carlo Atomistic Simulation and Machine Learning Analysis of Na-K Eutectic Alloy in Condensed Phases, D. Reitz and E. Blaisten-Barojas, George Mason University, Fairfax, VA 22030

3942	13678	9934	973	308	174	28048	1302	8	574	1	12	60	4	67	271.73	99	423.1	279	269	274	8	40	0	0	5
5318	12620	9066	602	690	442	28178	1090	5	680	0	40	69	4	99	282.05	100	439.55	393	360	327	15	46	0	2	4
4560	13086	9540	761	582	334	28132	1181	8	605	1	30	64	3	80	277.69	102	434.37	354	319	293	18	43	0	0	1
5910	12042	8750	547	888	546	28214	1056	20	656	3	74	76	11	122	286.36	95	441.34	372	333	329	31	49	1	4	4
11400	8026	5366	3	1898	1516	28580	556	97	622	20	342	156	45	262	315.84	124	460.72	513	209	371	81	81	8	30	21
6356	11846	8458	413	916	596	28244	941	13	731	5	72	84	6	119	288.28	145	444.89	456	358	364	27	51	0	0	4
13850	7836	4496	6	930	762	27970	614	275	988	124	90	227	113	163	275.75	255	440.22	497	362	459	30	89	21	6	0
4020	13674	9910	977	280	148	28034	1315	10	604	1	2	50	5	55	270.43	103	422.94	275	284	280	15	36	0	0	2
5250	12770	9156	641	588	354	28134	1107	5	677	1	12	65	3	102	281.84	93	437.18	364	349	342	14	37	0	4	2
14362	7700	4428	4	746	554	27856	656	324	1106	156	64	266	126	162	271.94	267	435.18	452	455	433	23	99	24	2	0
10596	7860	5714	0	2378	1798	28826	469	63	478	5	406	168	31	302	331.68	100	468.82	500	141	346	129	85	9	68	33
12082	7888	5150	1	1774	1288	28410	576	126	715	23	212	160	76	226	300.78	142	453.79	488	252	425	81	93	7	16	13
13978	7714	4502	11	974	708	27938	660	314	1010	156	54	258	126	154	270.66	253	434.32	505	433	400	16	88	17	8	2
3996	13570	9878	917	400	222	28074	1251	8	558	1	6	59	3	83	272.6	86	428.03	287	266	295	16	32	0	2	2
13180	7662	4708	0	1404	1056	28184	566	229	796	93	162	208	89	220	285.64	195	449.61	501	285	422	47	77	17	12	7
13740	7770	4582	3	1026	780	27998	623	283	967	127	82	233	120	175	275.57	225	440.62	481	403	440	35	87	13	18	1
4156	13576	9820	923	314	178	28052	1275	8	615	1	8	48	4	66	273.29	103	427.16	297	299	297	10	31	0	0	1
4548	13356	9610	814	336	196	28056	1193	9	643	2	10	69	6	73	273.67	103	427.64	339	317	314	5	45	0	0	3
11402	7860	5390	2	2058	1540	28604	567	89	578	10	318	163	45	280	314.53	94	460.63	466	221	357	103	81	7	36	18
4194	13488	9786	888	378	212	28070	1259	5	613	0	12	62	4	75	272.25	94	426.56	297	296	286	15	42	0	0	1
13880	7756	4522	3	964	752	27978	617	270	988	128	96	239	103	167	275.72	224	442.19	484	384	443	35	94	11	8	3
3998	13640	9920	953	314	164	28044	1294	8	575	1	8	59	6	66	271.38	84	424.44	273	283	285	15	42	0	0	3
14222	7806	4434	5	768	592	27870	669	302	1084	161	46	251	111	162	270.01	258	431.68	460	457	435	13	79	15	2	2
5054	12790	9302	644	622	338	28130	1140	8	696	0	22	66	4	86	277.95	99	432.62	366	378	314	18	44	0	2	2
4656	13198	9546	782	432	236	28080	1196	7	665	1	12	69	2	83	277.59	88	433.59	331	342	302	11	53	0	0	2
7038	11468	8154	354	894	572	28206	903	31	811	6	72	102	18	133	289.2	128	445.31	435	397	381	31	64	0	8	5
12282	7882	4952	0	1690	1330	28368	564	146	700	46	210	178	74	230	293.45	139	450.84	497	248	428	70	87	6	22	10
[truncated: 1,096,839 more chars]
